# Supplementary material for: An enantioselective four-component reaction via assembling two reaction intermediates
Source: Nat Commun. 2022 Nov 18;13:7088. doi: 10.1038/s41467-022-34913-2 (PMC9674633; doi:10.1038/s41467-022-34913-2)
Supplement: Supplementary file 1 — SUPPLEMENTARY INFORMATION [file 41467_2022_34913_MOESM1_ESM.pdf]

Supplementary Information

for

An enantioselective four-component reaction via assembling two reaction intermediates

Sifan Yu†, Wenju Chang†, Ruyu Hua†, Xiaoting Jie, Mengchu Zhang, Wenxuan Zhao, Jinzhou Chen, Dan Zhang, Huang Qiu*, Yong Liang*, Wenhao Hu*

Table of Contents

I. Supplementary Methods **S2**

1. General Information S2
2. Experimental Procedures S3
3. Supplementary Table 1-2 for Detailed Racemic Condition Optimizations S30
4. Supplementary Table 3-4 for Detailed Condition Optimizations S33

II. Supplementary Discussion **S37**

5. X-ray Diffraction Parameters and Data S37
6. ECD Parameters and Data of **93** S41
7. Control Experiments S42
8. DFT Calculation S59

III. Supplementary Data **S64**

9. Analytical Data for the Products S64
10. NMR Spectra for the Products S385

IV. Supplementary References **S533**

I. Supplementary Methods

1. General Information

General. Unless noted all solvents were purified and dried using standard procedures. All reactions were carried out under an inert atmosphere in oven-dried Schlenk tubes with magnetic stirring using freshly distilled solvents. Analytical thin layer chromatography (TLC) plates were purchased from EM Science (silica gel 60 F254 plates). All NMR spectra were recorded on a Bruker spectrometer at 400 or 500 MHz (^{1}H NMR), 101 or 126 MHz (^{13}C NMR), 376 or 471 MHz (^{19}F NMR), and 202 MHz (^{31}P NMR). Chemical shifts (δ value) were reported in ppm with the solvent signals as reference (in $CDCl_3$ as solvent) and coupling constants (*J*) are given in Hertz (Hz). The peak information was described as: br = broad singlet, s = singlet, d = doublet, t = triplet, q = quartet, m = multiplet, comp = composite of magnetically non-equivalent protons, down field from internal tetramethylsilane (TMS). Syringe pump was purchased from Longerpump. Needle filters were purchased from Anpel. Single-crystal X-ray diffraction data (**21** & **S28**) were collected in a Rikagu XtaLAB Synergy. The optical rotation data were collected in a Anton Paar MCP 100/150. HRMS (ESI) Mass Spectra were recorded on SHIMADZU LCMS-IT-TOF mass spectrometer. Liquid chromatography–mass spectrometry (LC-MS) was recorded on WATERS ACQUITY UPLC. HPLC analysis was performed on WATERS e2695. NMR tube experiments were handled by Demen Ultrasonic cleaning machine JP-040S.

Materials. Alcohol **1**(except cinnamyl alcohol and alkynol), arylacetic acids, indole **3A-3G**, aldehyde **4,** amine **5** and all dry solvents except toluene (99.9%, Extra Dry, with molecular sieves, Water ≤ 50 ppm (by K.F.), EnergySeal) were purchased from Energy Chemical. All catalysts including JohnphosAu(MeCN)SbF_6, [PdCl(2-butenyl)]$_2$ and $Rh_2(TFA)_4$ were purchased from Laajoo. $Cu(MeCN)_4PF_6$, CuOTf, [PdCl(allyl)]$_2$,

$Rh_2(Oct)_4$, $[PdCl(cinnyl)]_2$, $[Ir(COD)Cl]_2$, Cp*Ru(COD)Cl were purchased from Energy Chemical. $Rh_2(OAc)_4$, $Rh_2(esp)_2$ were purchased from Sigma-Aldrich. chiral BINOL phosphoric acid **S6a**, **S6b-S6e**, **S6h-S6j**, **6b**, **6c**, chiral SPINOL phosphoric acids (*S*)-**S6f-S6g, S6k, 6a** were purchased from Daicel Chiral Technologies (China) Co., LTD, Chiral SPINOL phosphoric acids **S6I** was supported from Bin Tan group. [1] Anhydrous toluene was distilled from Na under an atmosphere of argon. All other chemicals were obtained from commercial sources and used as received without further purification.

2. Experimental Procedures

2.1 Preparation of enamine 3a-3c[2]

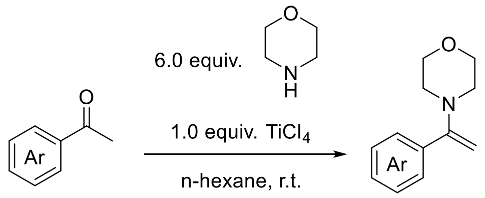

To a solution of acetophenone (10.0 g, 86.0 mmol) and morpholine (42.5 g, 516.0 mmol) in anhydrous hexane (200 mL), was added $TiCl_4$ (17.8 g, 86.0 mmol) over 30 min at 0°C. The reaction mixture was stirred at room temperature for 24 h and filtered. The filtrate was evaporated under vacuum to give colorless oil, which was distilled under reduced pressure to give enamine **3a-3c** as a yellow oil.

2.2 Preparation of [PdOAc(allyl)]2 and [PdTFA(allyl)]2 [3]

To the solution of $[PdCl(allyl)]_2$ (1.0 equiv.) in dichloromethane (0.08 M) was added AgOAc/AgTFA (2.0 equiv.) in one portion. The bright yellow solution was stirred for 30 minutes, and off-white precipitate of AgCl was filtered off using a PTFE (0.22 μm) syringe filter. Solvent was evaporated and bright yellow solid was dried under a high vacuum for 1 h and stored in the N_2-filled glove box.

2.3 Preparation of diazo compounds 2a-2l [4-6]

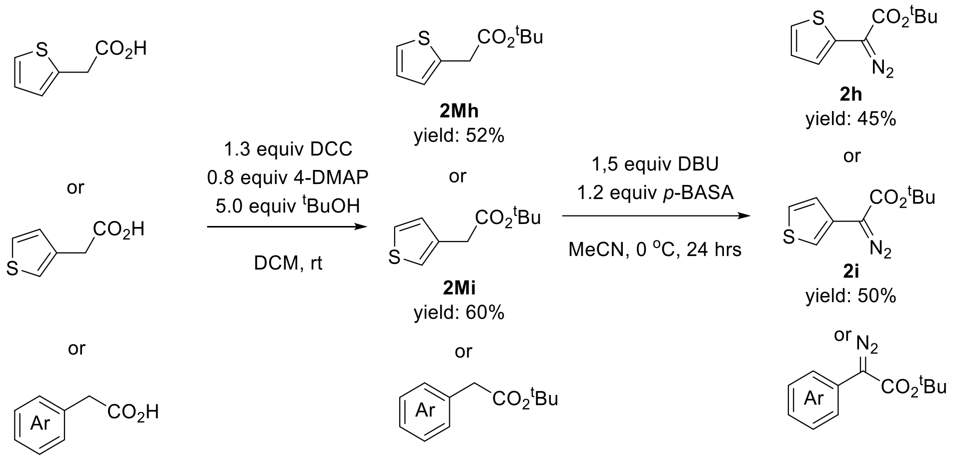

To a flame-dried 250-mL flask charged with a magnetic stirring bar, 2-(thiophen-2-yl)acetic acid or 2-(thiophen-3-yl)acetic acid (2.84 g, 20.0 mmol, 1.0 equiv.), tert-butanol (7.41 g, 100.0 mmol, 5.0 equiv.) and 4-DMAP (1.7 g, 14.0 mmol, 0.7 equiv.) in 50 mL dichloromethane at 0 °C was added DCC (5.36 g, 26.0 mmol, 1.3 equiv.) slowly. The resulting mixture was warmed up slowly to room temperature and stirred overnight. The solid was filtered off and washed with hexanes. The filtrate was concentrated in vacuo and products were purified by column chromatography (eluent: PE/EA = 50:1 ~ 30:1, v/v) to afford the pure product **2Mh** (2.06 g, 52% yield) or **2Mi** (2.38 g, 60% yield).

To a flame-dried 50-mL Schlenk flask charged with a magnetic stirring bar, *p*-ABSA (3.12 g, 13.0 mmol, 1.3 equiv.), **2Mh** or **2Mi** (1.98g, 10.0 mmol, 1.0 equiv.) in 8.0 mL MeCN were sequentially added at 0 °C. DBU (1.83 g, 12.0 mmol, 1.5 equiv.) dissolved in MeCN (2.0 mL) were added over 10.0 mins. The resulting mixture was warmed up slowly to room temperature and stirred overnight. The reaction solution was slowly added saturated NH_4Cl(aq) before extracted with diethyl ether (15 x 3 mL). The organic layers were washed with brine. After drying over $MgSO_4$ and concentration in vacuo, the crude product was purified by flash chromatography on silica gel (eluent: PE/EA =

20:1, v/v) to afford the pure product **2h** (1.01 g, 45% yield) or **2i** (1.12 g, 50% yield).

2.4 Preparation of cinnamyl alcohol [7]

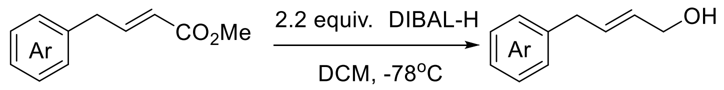

To a stirred solution of the α, β-unsaturated ester (1.0 equiv.) in anhydrous DCM (0.2 M) at −78 °C under N_2 was added DIBAL-H (1.2 M in toluene, 2.2 equiv.) dropwise. The reaction was stirred at −78 °C for 2 h, and quenched with NaOH (10% aq.) (equal volume). The resultant mixture was allowed to warm to rt and stirred for 1 h. The layers were separated and the aqueous layer extracted with DCM (2 equal volume). The combined organics were washed with brine (equal volume), dried over Na_2SO_4, filtered, and concentrated in vacuo to leave the pure cinnamyl alcohols.

2.5 Preparation of propargyl alcohols[8] and arylpropiolaldehyde [9]

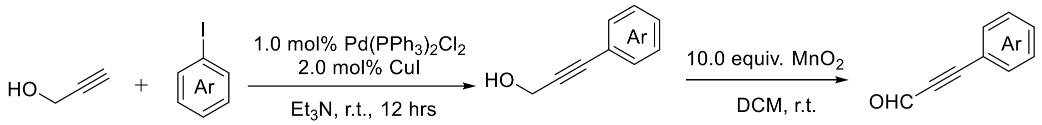

To a solution of iodoarene (1.2 equiv, 9.8 mmol) in trimethylamine (26 mL) was added $Pd(PPh_3)_2Cl_2$ (1 mol %, 0.9 mmol) and CuI (2 mol %, 0.18 mmol). The reaction was stirred for 10 min before the addition of propargyl alcohol (1.0 equiv., 8.9 mmol). The resulting mixture was stirred for 12 h. Then, the reaction was treated with a saturated $NaHCO_3$ solution. The solution was extracted with ethyl acetate (3 × 20 mL), washed with a saturated NaCl solution and dried over anhydrous $MgSO_4$. The solvent was removed under vacuum and the product was purified by column chromatography (eluent: PE/EA = 10:1, v/v).

To a solution of propargyl alcohol (10.0 mmol, 1.0 equiv.) in DCM (30 mL) was added MnO_2 (20.0 equiv.) very slowly at room temperature. The resulting mixture was stirred

for 18 h at room temperature. Then the mixture was filtered. The filtrate was evaporated under vacuum to give crude product. The solvent was removed under vacuum and the product was purified by column chromatography (eluent: PE/EA = 30:1, v/v).

2.6 Preparation of olefine aldehyde [10]

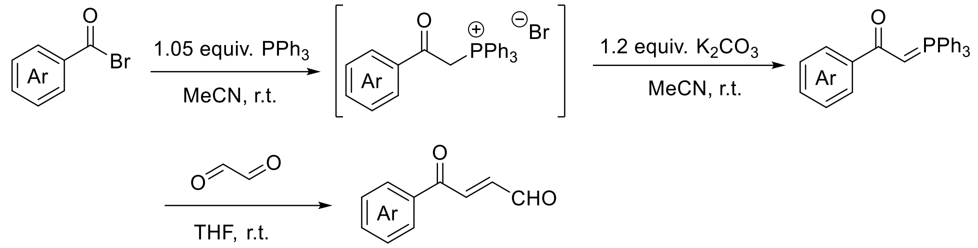

To a solution of the respective α-bromoketone (1.0 equiv.) in MeCN (0.2 M) was added PPh_3 (1.05 eq.) in one portion. The mixture was stirred at rt for 3 h. Afterwards, K_2CO_3 (1.2 equiv.) was added in one portion and the reaction mixture was stirred for additional 3 h. The solid was removed by filtration and the crude filtrate was concentrated in vacuo. The crude ylide was then dissolved in THF (0.2 M) and glyoxal was added (3.0 eq., 40% aqueous solution) and the reaction was stirred at rt overnight. The crude mixture was concentrated in vacuo, and purified by flash column. All olefine aldehyde were obtained with >20:1 E/Z.

2.7 Preparation of indole compounds 3H-3I [11-12]

1) Procedure for Synthesis of 3H

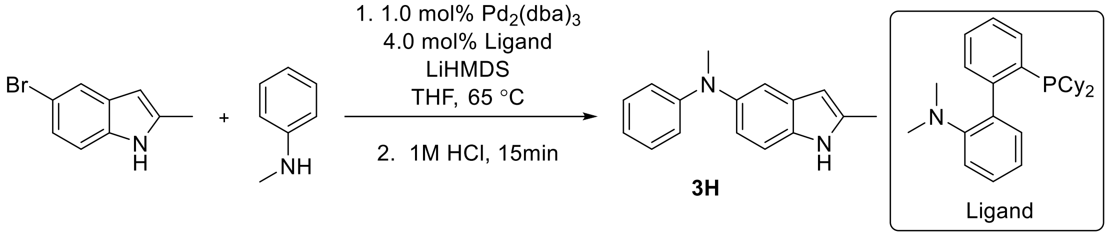

$Pd_2(dba)_3$ (45.8 mg, 0.05 mmol), 2-dicyclohexylphosphino-2′-(N,N-

dimethylamino)biphenyl (78.7 mg, 0.2 mmol) and 5-bromo-2-methyl-1*H*-indole (1.04 g, 5.0 mmol) were added to an oven dried vacuum schlenk tube under argon. The solids were dissolved in THF (5.0 mL). Lithium bis(trimethylsilyl)amide 1.0 M solution in THF (11.0 mL, 11.0 mmol) and the corresponding amine (6.0 mmol) were added to the solution. The reaction mixture was heated during 24 h at 65 °C. After reaction go to complete (monitored by TLC), the system was cooled to room temperature and 1 M HCl (10.0 mL) was added to the reaction and the mixture was stirred for 15 min. The reaction crude was poured into a separatory funnel and diluted with saturated $NaHCO_3$ (20 mL). The aqueous phase was extracted with ethyl acetate (3 x 20 mL). The combined organic extracts were washed with brine (50 mL), dried over Na_2SO_4 and the solvent was removed under vacuum. The residue was purified by column chromatography (Petroleum Ether: EtOAc = 40: 1) to give N,2-dimethyl-N-phenyl-1*H*-indol-5-amine **3H** (885.5 mg, 75% yield) as brown solid.

2) Procedure for Synthesis of 3I

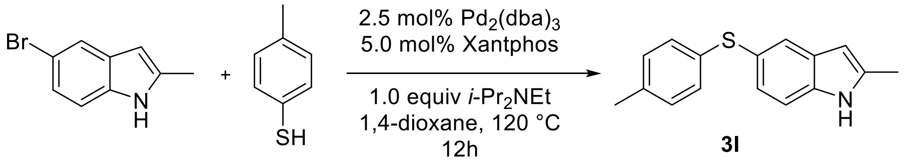

5-Bromo-2-methyl-1H-indole (1.04 g, 5.0 mmol), 4-methylbenzenethiol (682.2 mg, 5.5 mmol), $Pd_2(dba)_3$ (114.5 mg, 0.125 mmol), Xantphos (289.3 mg, 0.25 mmol), *i*-Pr2NEt (646.2 mg, 5.0 mmol) were dissolved in 1,4-dioxane (20.0 mL)，the mixture was stirred at 120 °C for 12 hrs. After reaction go to complete (monitored by TLC), the solution was cooled to room temperature, the mixture was filtered through a plug of silica/sand/celite. The residue was purified by silica-gel column chromatography (Petroleum Ether: EtOAc = 60: 1) to give 2-methyl-5-(p-tolylthio)-1H-indole **3I** (911.1 mg, 72% yield) as yellow solid.

2.8 General Procedure for Racemic Four-component Reactions

A

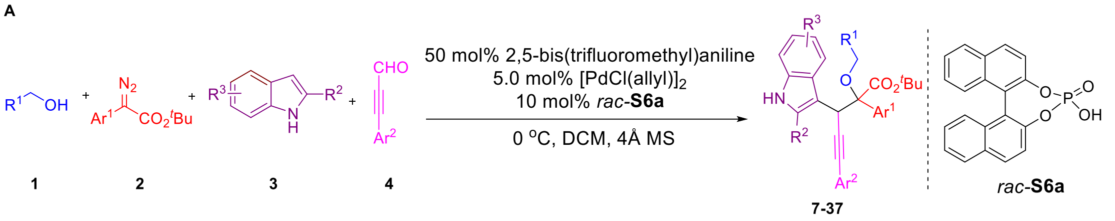

To a flame-dried 10-mL Schlenk flask charged with a magnetic stirring bar, alcohol **1** (0.15 mmol), aldehyde **4** (0.15 mmol), 2,5-bis(trifluoromethyl)aniline **5a** (50 mol%), $[PdCl(allyl)]_2$ (5 mol%), *rac*-**S6a** (10 mol%) and 4Å MS (70 mg) in DCM (1.0 mL), was added a mixture of diazoacetate **2** (0.075 mmol) and indole **3** (0.05 mmol) in DCM (1.0 mL) for 1.0 h via a syringe pump at 0 °C. The mixture was stirred for additional 6 hrs under these conditions. After the completion of the reaction, the reaction mixture was filtrated and the filtrate was evaporated in vacuo to give the crude product. And then the crude product was purified by flash chromatography on silica gel (eluent: PE: EA = 20 : 1) to afford the pure products.

B

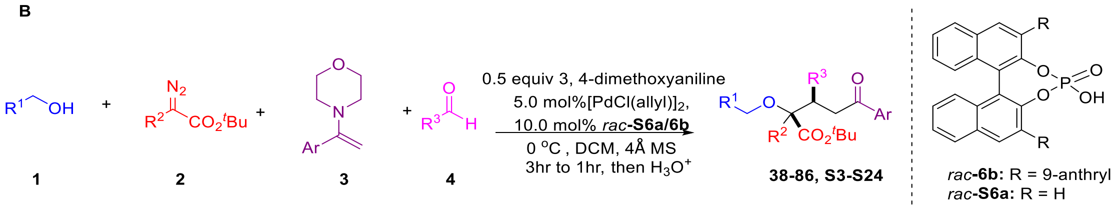

To a flame-dried 10-mL Schlenk flask charged with a magnetic stirring bar, 3,4-dimethoxyaniline **5b** (0.10 mmol), 5.0 mol% $[PdCl(allyl)]_2$, 10.0 mol% *rac*-**S6a** or *rac*-**6b**, alcohol **1**(0.24 mmol), aldehyde **4** (0.20 mmol) and 100 mg 4 Å MS in 2.0 mL DCM were sequentially added at 0 °C. Diazoacetate **2** (0.24 mmol) and enamine **3**(0.24 mmol) dissolved in DCM (1.0 mL) were added by syringe pump over 180 mins. The mixture was stirred for 1.0 hr at 0 °C. After the completion of the reaction, the reaction mixture was filtrated and the filtrate was evaporated in vacuo to give the crude product. And then the crude product was purified by flash chromatography on silica gel (eluent:

PE/EA = 50:1 ~ 30:1, v/v) to give the mixed products containing *anti*-product and *syn*-product or *syn*-product.

2.9 General Procedure and Substrate Scope of Enantioselective Four-component Reactions of Alcohol, Diazoester, Indole/Enamine and Aldehyde Derivatives

Procedure A under the condition A

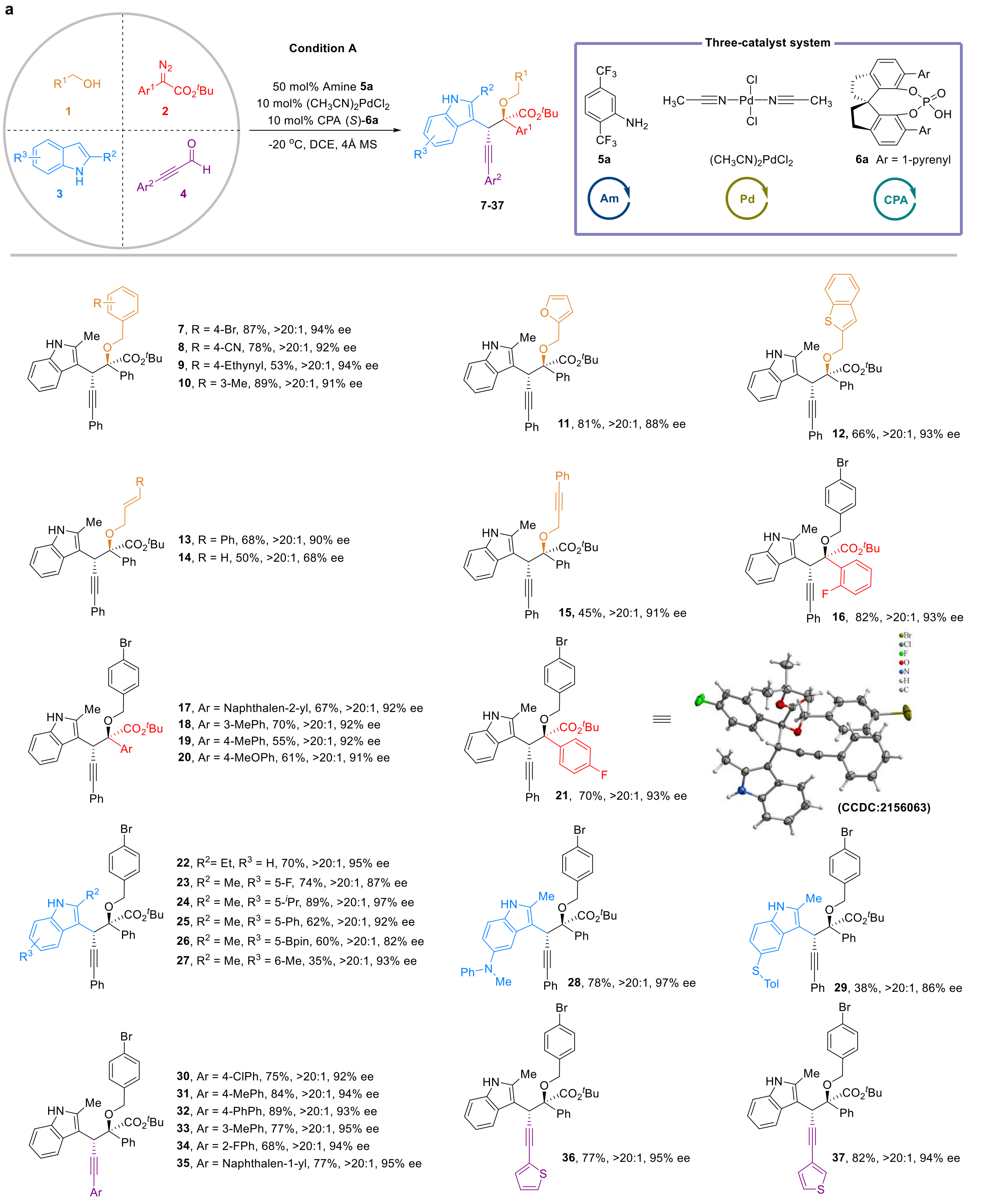

b. The following substrates can not transform into the desired four-component coupling products (The yield of product was detected by LC-MS)

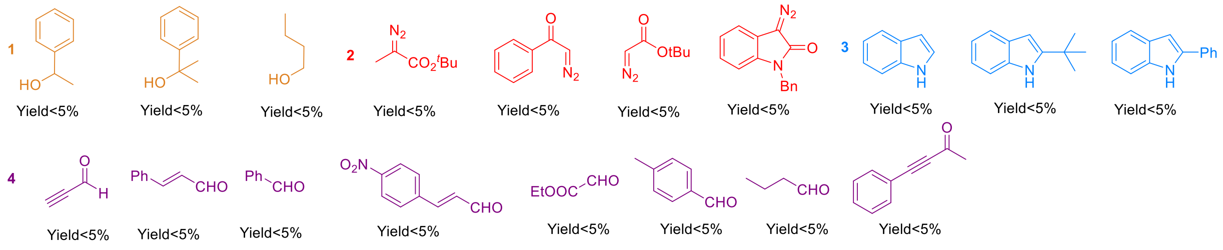

Supplementary Figure 1. Substrate scope of enantioselective four-component reactions of alcohol, diazoester, indole and aldehyde derivatives. Standard conditions: **1/2/3/4/5a/[Pd]/6a** = 0.6/0.3/0.2/0.6/0.1/0.01/0.02 mmol. **2, 3** in 4.0 mL dry DCE was added into a solution of **1**, **4**, **5a** (50 mol%), [Pd] (5 mol%), **6a** (10 mol%), and 4 Å MS (50 mg) in 4.0 mL dry DCE via a syringe pump for 60 minutes, and the resulting mixture was stirred for another 12 hours at -20 °C.

To a flame-dried 20-mL Schlenk flask charged with a magnetic stirring bar, alcohol **1** (0.6 mmol), aldehyde **4** (0.6 mmol), 2,5-bis(trifluoromethyl)aniline **5a** (50 mol%), Pd $(CH_3CN)_2Cl_2$ (10 mol%), (*S*)-**6a** (10 mol%) and 4Å MS (100 mg) in DCE (4.0 mL), was added a mixture of diazoacetate **2** (0.3 mmol) and indole **3** (0.2mmol) in DCE (4.0 mL) for 1.0 h via a syringe pump at -20 °C. The mixture was stirred for additional 12 hrs under these conditions. After the completion of the reaction, the reaction mixture was filtrated and the filtrate was evaporated in vacuo to give the crude product. And then the crude product was purified by flash chromatography on silica gel (eluent: PE: EA = 20: 1) to afford the pure products.

Procedure B under the condition B

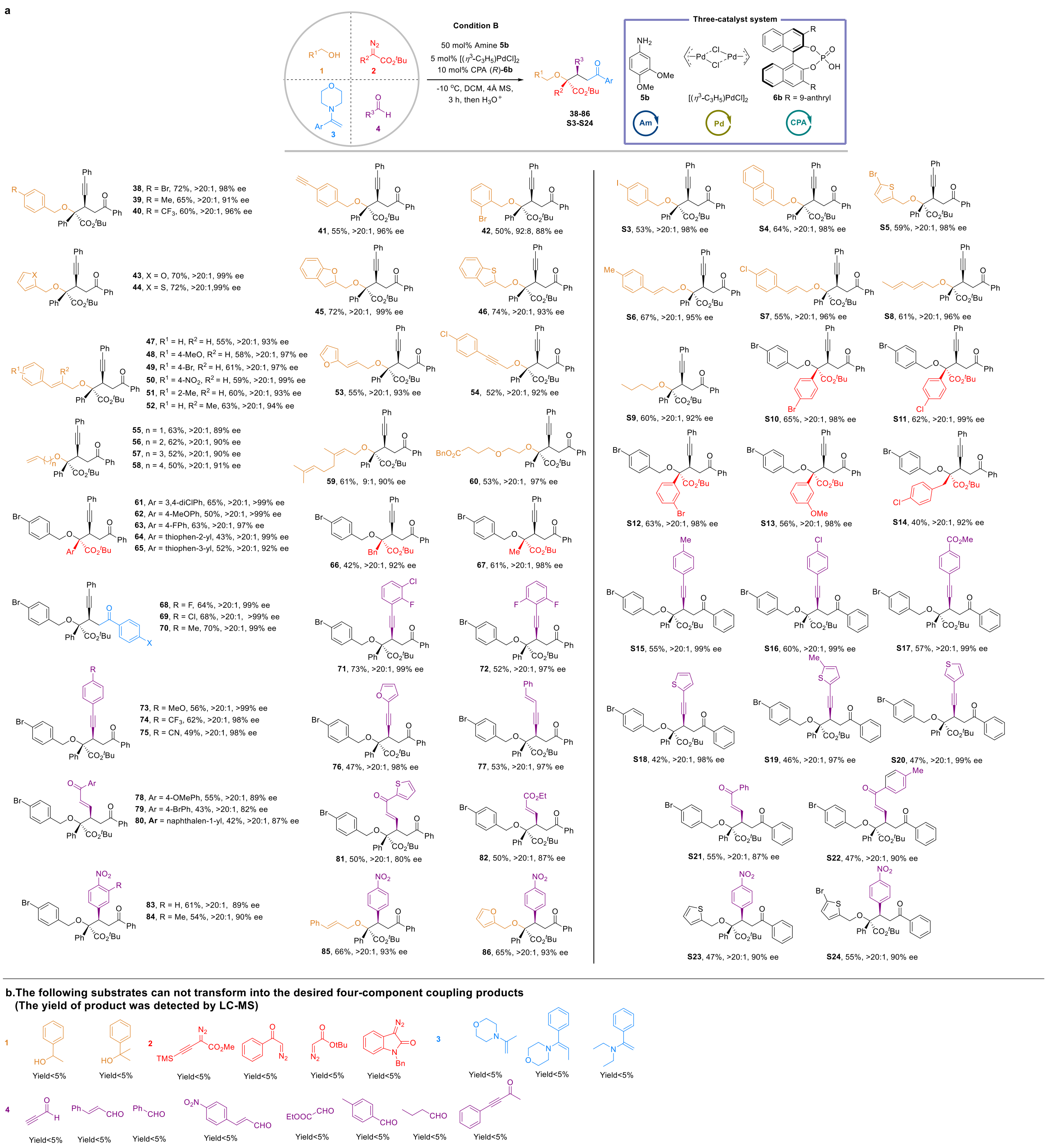

Supplementary Figure 2. Substrate scope of enantioselective four-component reactions of alcohol, diazoester, enamine and aldehyde derivatives. Standard condition: **1**/**2**/**3**/**4**/**5b**/[PdCl(allyl)]$_2$/**6b** = 0.18/0.18/0.18/0.15/0.075/0.0075/0.015 mmol, **2** and **3a** in 1.0 mL dry DCM were added into a solution of **1**, **4**, [PdCl(allyl)]$_2$, **6b**, aniline **5b** and 100 mg 4 Å MS in 1.5 mL dry DCM via a syringe pump under a nitrogen atmosphere for 3 hours, and the resulting mixture was stirred for another 1 hour at -10 °C.

To a flame-dried 10-mL Schlenk flask charged with a magnetic stirring bar, 3,4-

dimethoxyaniline **5b** (0.075 mmol), 5.0 mol% $[PdCl(allyl)]_2$, 10.0 mol% *(R)*-**6b**, alcohol **1**(0.18 mmol), aldehyde **4** (0.15 mmol) and 100 mg 4 Å MS in 2 mL DCM were sequentially added at -10 ºC. Diazoacetate **2** (0.18 mmol) and enamine **3** (0.18 mmol) dissolved in DCM (1.5 mL) were added by syringe pump over 180 mins. The mixture was stirred for 1.0 hour at -10 °C. After the completion of the reaction, the reaction mixture was filtrated and the filtrate was evaporated in vacuo to give the crude product. And then the crude product was purified by flash chromatography on silica gel (eluent: PE/EA = 50:1 ~ 30:1, v/v) to give the pure product (If the reaction was carried out on more than 0.15 mmol scale, the decreased ee value of corresponding product might be detected).

The half a gram-scale synthesis of product **38**, we could obtain product **38** in 60% yield (731.5 mg), with >20:1 d.r., and 94% ee.

The half a gram-scale synthesis of product **47**, we could obtain product **47** in 52% yield (579.0 mg), with >20:1 d.r., and 94% ee.

2.10 Procedure for Synthesis of 60. [13]

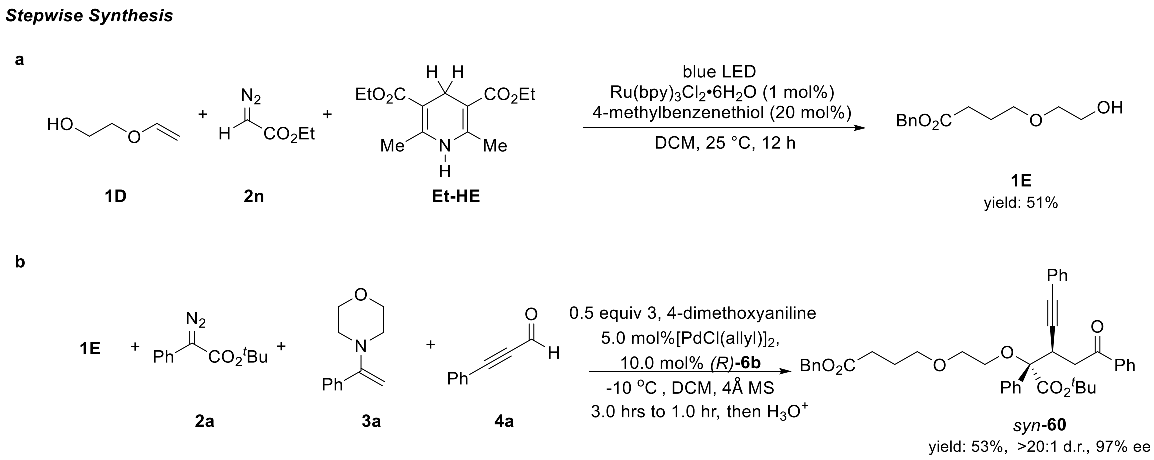

To a 10-ml glass tube equipped with a magnetic stir bar were added **Et-HE** (202.4 mg, 0.8 mmol, 2.0 equiv.), $Ru(bpy)_3Cl_2•6H_2O$ (3.0 mg, 0.004 mmol, 1.0 mol%), and 4-methylbenzenethiol (10.0 mg, 0.08 mmol, 20.0 mol%). The tube was capped. After

evacuation and backfilling with argon three times, anhydrous DCM (2.0 mL, 0.2 M) was added via a syringe, followed by the addition of **1D** (0.4 mmol, 1.0 equiv.), and **2n** (0.8 mmol, 2.0 equiv.). The resulting solution was irradiated by a 50 W blue LED with stirring at a distance of ~15 cm (with cooling by the fan) at 25°C for about 12 h until the diazo compound was consumed completely, as monitored by TLC analysis. The reaction mixture was filtered, and the filtrate were concentrated, the crude product was purified by flash chromatography on silica gel (eluent: PE/EA = 5:1 ~ 2:1, v/v) to afford the pure product **1E (**48.6 mg, 51% yield, colorless oil).

To a flame-dried 10-mL Schlenk flask charged with a magnetic stirring bar, 3,4-dimethoxyaniline **5b** (0.025 mmol), 5.0 mol% $[PdCl(allyl)]_2$, 10.0 mol% *(R)*-**6b**, alcohol **1E** (0.06 mmol), aldehyde **4** (0.05 mmol) and 50 mg 4 Å MS in 1 mL DCM were sequentially added at -10 °C. Diazoacetate **2** (0.06 mmol) and enamine **3** (0.06 mmol) dissolved in DCM (1.0 mL) were added by syringe pump over 180 mins. The mixture was stirred for 1.0 hour at -10 °C. After the completion of the reaction, the reaction mixture was filtrated and the filtrate was evaporated in vacuo to give the crude product. And then the crude product was purified by flash chromatography on silica gel (eluent: PE/EA = 50:1 ~ 30:1, v/v) to give the pure product **60** (53% yield, 17.5 mg, >20:1 d.r., 97% ee, Colorless oil).

2.11 Procedure for Synthesis of 87.

Stepwise Synthesis

a

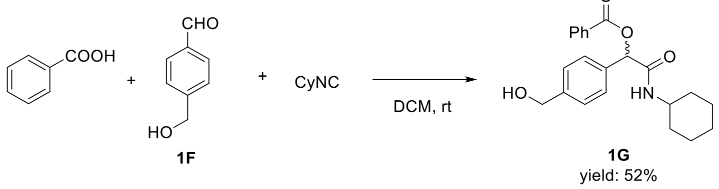

b

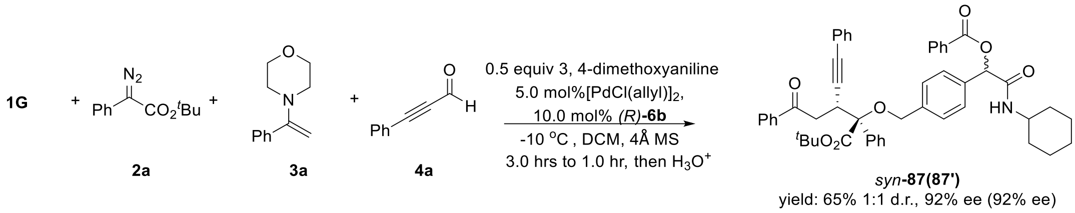

To a flame-dried 25-mL Schlenk flask charged with a magnetic stirring bar, acid (550 mg, 4.5 mmol, 1.5 equiv.), aldehyde **1F** (408.5 mg, 3.0 mmol, 1.0 equiv.), isocyanide (982.6 mg, 9.0 mmol, 3.0 equiv.) in 10 mL DCM. The mixture was stirred at room temperature for overnight. The mixture was concentrated, the crude product was purified by flash chromatography on silica gel (eluent: PE/EA = 5:1 ~ 2:1, v/v) to afford the pure product **1G (**573.2 mg, 52% yield, white solid).

To a flame-dried 10-mL Schlenk flask charged with a magnetic stirring bar, 3,4-dimethoxyaniline **5b** (0.025 mmol), 5.0 mol% $[PdCl(allyl)]_2$, 10.0 mol% *(R)*-**6b**, alcohol **1G** (0.06 mmol), aldehyde **4** (0.05 mmol) and 50 mg 4 Å MS in 1 mL DCM were sequentially added at -10 ºC. Diazoacetate **2** (0.06 mmol) and enamine **3** (0.06 mmol) dissolved in DCM (1.0 mL) were added by syringe pump over 180 mins. The mixture was stirred for 1.0 hour at -10 °C. After the completion of the reaction, the reaction mixture was filtrated and the filtrate was evaporated in vacuo to give the crude product. And then the crude product was purified by flash chromatography on silica gel (eluent: PE/EA = 50:1 ~ 30:1, v/v) to give the pure product **87** (total 65% yield, 25.6 mg, 1:1 d.r., 92% ee, 92% ee, white solid).

2.12 Procedure for Synthesis of 88. [14]

Stepwise Synthesis

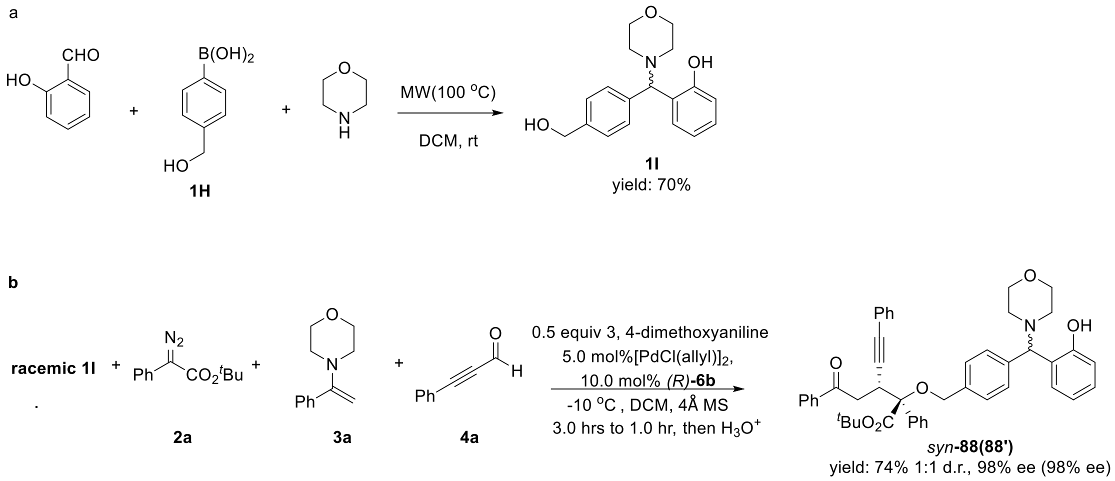

Aldehyde (122.1 mg, 1.0 mmol, 1.0 equiv.), morpholine (87.1 mg, 1 mmol, 1.0 equiv.) and boronic acid **1H** (152.0 mg, 1.0 mmol, 1.0 equiv.) were mixed in a 10 mL microwave vial and heated to 100 °C with microwave irradiation for 1 hr. The crude product was purified by flash chromatography on silica gel (eluent: PE/EA = 3:1 ~ 1:1, v/v) to afford the pure product **1I (**209.6 mg, 70% yield, colorless oil).

To a flame-dried 10-mL Schlenk flask charged with a magnetic stirring bar, 3,4-dimethoxyaniline **5b** (0.025 mmol), 5.0 mol% [PdCl(allyl)]$_2$, 10.0 mol% *(R)*-**6b**, alcohol **1H** (0.06 mmol), aldehyde **4** (0.05 mmol) and 50 mg 4 Å MS in 1 mL DCM were sequentially added at -10 °C. Diazoacetate **2** (0.06 mmol) and enamine **3** (0.06 mmol) dissolved in DCM (1.0 mL) were added by syringe pump over 180 mins. The mixture was stirred for 1.0 hour at -10 °C. After the completion of the reaction, the reaction mixture was filtrated and the filtrate was evaporated in vacuo to give the crude product. And then the crude product was purified by flash chromatography on silica gel (eluent: PE/EA = 50:1 ~ 30:1, v/v) to give the pure product **88** (total 74% yield, 26.7 mg, 1:1 d.r., 98% ee, 98% ee, white solid).

2.13 Procedure for Synthesis of 89. [15]

Stepwise Synthesis

a

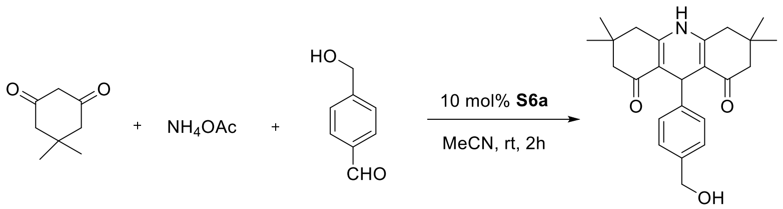

1J
yield: 90%

b

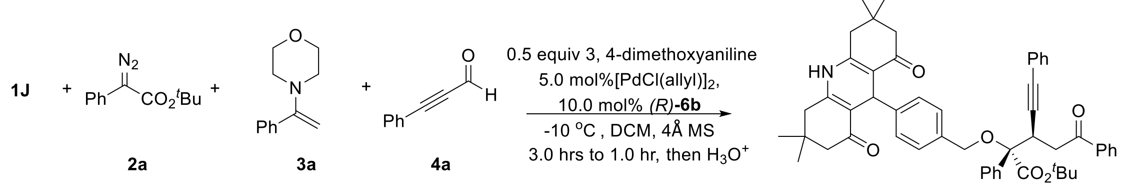

syn-**89**
yield: 64% 10:1 d.r., 96% ee

To a flame-dried 10-mL Schlenk flask charged with a magnetic stirring bar, aldehyde (27.2 mg, 0.2 mmol, 1.0 equiv.), dimedone (61.7 mg, 0.44mmol, 2.2 equiv.), NH_4OAc (15.4 mg, 0.2 mmol, 1.0 equiv.), and 10 mol% racemic **S6a** were dissolved in 3.0 mL MeCN. The mixture was stirred for 2.0 hrs at room temperature. The mixture was concentrated, the crude product was purified by flash chromatography on silica gel (eluent: PE/EA = 5:1 ~ 2:1, v/v) to afford the pure product **1J (**68.2 mg, 90% yield, colorless oil).

To a flame-dried 10-mL Schlenk flask charged with a magnetic stirring bar, 3,4-dimethoxyaniline **5b** (0.025 mmol), 5.0 mol% $[PdCl(allyl)]_2$, 10.0 mol% *(R)*-**6b**, alcohol **1J** (0.06 mmol), aldehyde **4** (0.05 mmol) and 50 mg 4 Å MS in 1 mL DCM were sequentially added at -10 °C. Diazoacetate **2** (0.06 mmol) and enamine **3** (0.06 mmol) dissolved in DCM (1.0 mL) were added by syringe pump over 180 mins. The mixture was stirred for 1.0 hour at -10 °C. After the completion of the reaction, the reaction mixture was filtrated and the filtrate was evaporated in vacuo to give the crude product. And then the crude product was purified by flash chromatography on silica gel (eluent: PE/EA = 50:1 ~ 30:1, v/v) to give the pure product **89** (64% yield, 25.6 mg, 10:1 d.r., 96% ee, white solid).

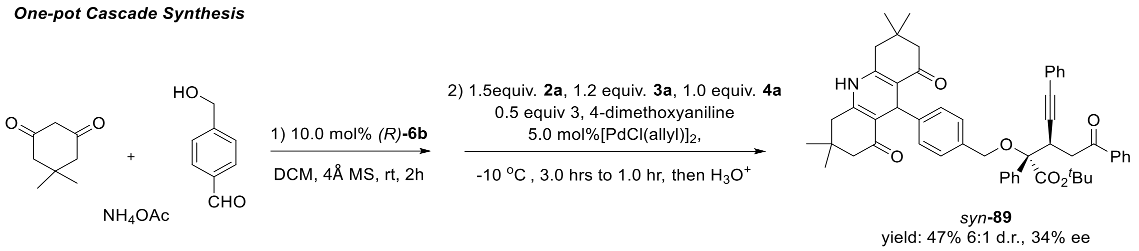

To a flame-dried 10-mL Schlenk flask charged with a magnetic stirring bar, aldehyde (0.05 mmol, 1.0 equiv.), dimedone (0.11mmol, 2.2 equiv.), NH_4OAc (0.05 mmol, 1.0 equiv.), 10 mol% **6b**, and 100 mg 4 Å MS were dissolved in 1.0 mL DCM. The mixture was stirred for 2.0 hrs at room temperature. Then 3, 4-dimethoxyaniline **5b** (0.025 mmol, 0.5 equiv.), 5.0 mol% $[PdCl(allyl)]_2$, aldehyde **4a** (0.05 mmol, 1.0 equiv.) were sequentially added at -10 ºC. Diazoacetate **2a** (0.75 mmol) and enamine **3a** (0.06 mmol, 1.2 equiv.) dissolved in DCM (0.5 mL) were added by syringe pump over 180 mins. The mixture was stirred for 1.0 hr at -10 ºC. After the completion of the reaction, the reaction mixture was filtrated and the filtrate was evaporated in vacuo to give the crude product. And then the crude product was purified by flash chromatography on silica gel (eluent: PE/EA = 10:1 ~ 5:1, v/v) to give the pure product **89**.

2.14 Procedure for Synthesis of 90.[16]

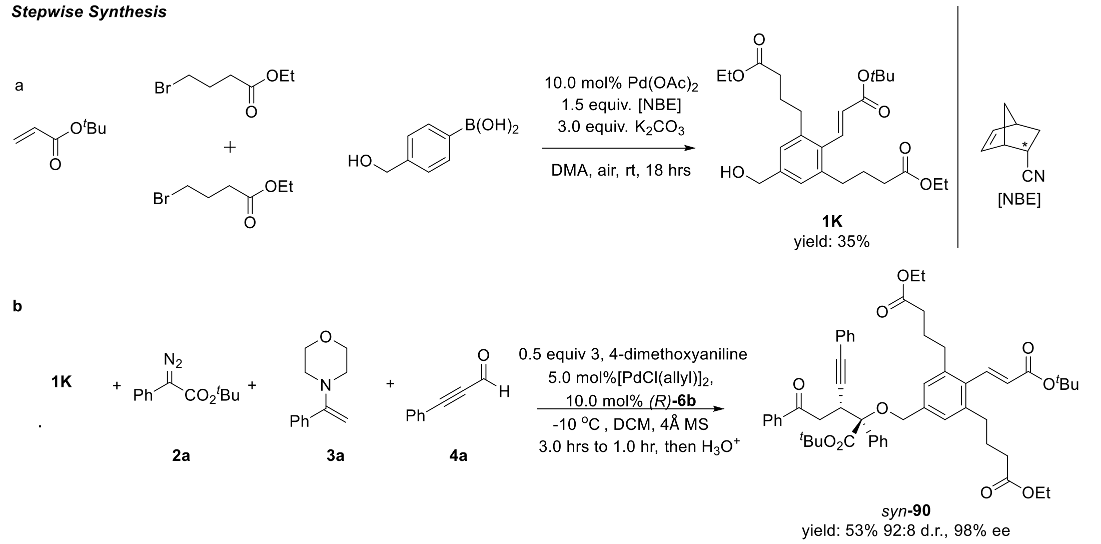

To a flame-dried 10-mL Schlenk flask charged with a magnetic stirring bar, aryl boron (1.5 mmol, 228mg, 1.5 equiv.), $Pd(OAc)_2$ (23 mg, 0.10 mmol, 0.1 equiv.), K_2CO_3 (414 mg, 3.0 mmol, 3.0 equiv.) alkyl bromide (466 mg, 1.2 mmol, 1.2 equiv.), olefin (141 mg, 1.0 mmol, 1.0 equiv.), 5-norbornene-2-carbonitrile (179 mg, 1.5 mmol, 1.5 equiv.) and DMA (1 mL) under air. The reaction was stirred at room temperature for 18 hrs. Then the mixture was filtered through a thin pad of celite eluting with ethyl acetate (30 mL), and the filtrate was sequentially washed with water, brine, dried over Na_2SO_4. After concentrated *in vacuo*, the crude product was purified by flash chromatography on silica gel (eluent: PE/EA = 10:1 ~ 5:1, v/v) to afford the pure product **1K (**161.7 mg, 35% yield).

To a flame-dried 10-mL Schlenk flask charged with a magnetic stirring bar, 3,4-dimethoxyaniline **5b** (0.025 mmol), 5.0 mol% $[PdCl(allyl)]_2$, 10.0 mol% *(R)*-**6b**, alcohol **1K** (0.06 mmol), aldehyde **4** (0.05 mmol) and 50 mg 4 Å MS in 1 mL DCM were sequentially added at -10 ºC. Diazoacetate **2** (0.06 mmol) and enamine **3** (0.06 mmol) dissolved in DCM (1.0 mL) were added by syringe pump over 180 mins. The mixture was stirred for 1.0 hour at -10 °C. After the completion of the reaction, the reaction mixture was filtrated and the filtrate was evaporated in vacuo to give the crude product. And then the crude product was purified by flash chromatography on silica gel (eluent: PE/EA = 50:1 ~ 30:1, v/v) to give the pure product **90** (53% yield, 23.5 mg, 92:8 d.r., 98% ee, colorless oil).

2.15 Procedure for Synthesis of 91. [17]

Stepwise Synthesis

a

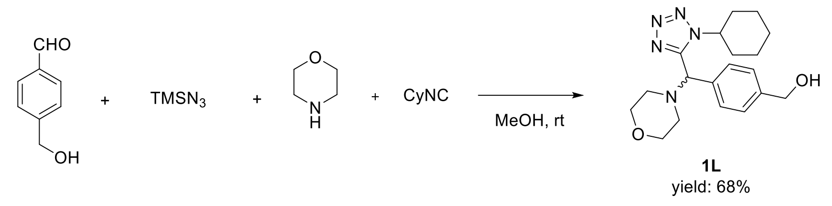

b

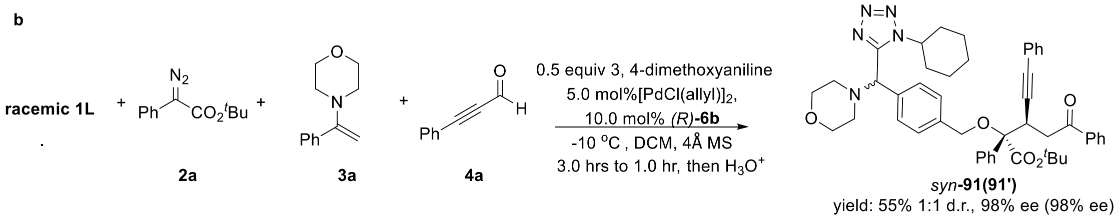

To a flame-dried 25-mL Schlenk flask charged with a magnetic stirring bar, morpholine (261.4 mg, 3.0 mmol, 1.0 equiv.), aldehyde (408.5 mg, 3.0 mmol, 1.0 equiv.) in 6 mL MeOH, the resulting solution was stirred for 15 mins at room temperature and then a solution of isocyanide (327.5 mg, 3.0 mmol, 1.0 equiv.), and $TMSN_3$ (345.6 mg, 3.0 mmol, 1.0 equiv.) in MeOH (2.0 mL) was added to the mixture in one portion. The mixture was stirred at room temperature for overnight. The mixture was concentrated, the crude product was purified by flash chromatography on silica gel (eluent: PE/EA = 5:1 ~ 2:1, v/v) to afford the pure product **1L (**243 mg, 68% yield, colorless oil).

To a flame-dried 10-mL Schlenk flask charged with a magnetic stirring bar, 3,4-dimethoxyaniline **5b** (0.025 mmol), 5.0 mol% $[PdCl(allyl)]_2$, 10.0 mol% *(R)*-**6b**, alcohol **1L** (0.06 mmol), aldehyde **4** (0.05 mmol) and 50 mg 4 Å MS in 1 mL DCM were sequentially added at -10 °C. Diazoacetate **2** (0.06 mmol) and enamine **3** (0.06 mmol) dissolved in DCM (1.0 mL) were added by syringe pump over 180 mins. The mixture was stirred for 1.0 hour at -10 °C. After the completion of the reaction, the reaction mixture was filtrated and the filtrate was evaporated in vacuo to give the crude product. And then the crude product was purified by flash chromatography on silica gel (eluent: PE/EA = 50:1 ~ 30:1, v/v) to give the pure product **91** (total 55% yield, 21.4 mg, 1:1 d.r., 98% ee, 98% ee, white solid).

2.16 Procedure for Synthesis of 92. [18]

Stepwise Synthesis

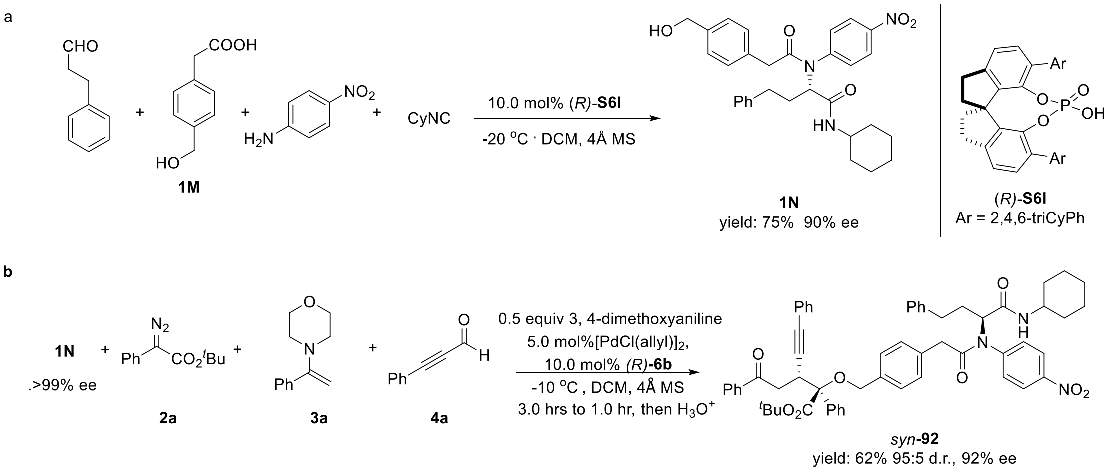

To a flame-dried 10-mL Schlenk flask charged with a magnetic stirring bar, amine (18.0 mg, 0.13 mmol, 1.3 equiv.), acid (16.6 mg, 0.1 mmol, 1.0 equiv.), 10.0 mol% (*R*)-**S6l**, and dry DCM (1.0 mL) under argon. The resulting solution was stirred for 15 mins at -20 °C and then a solution of aldehyde (17.4 mg, 0.13 mmol, 1.3 equiv.) in DCM (0.5 mL) was added to the mixture in one portion. After 15 mins, a solution of isocyanide (14.1 mg, 0.13 mmol) in DCM (0.5 mL) was added to the mixture in one portion. Then the mixture was stirred at -20 °C for 12 hrs. The mixture was concentrated, the crude product was purified by flash chromatography on silica gel (eluent: PE/EA = 5:1 ~ 2:1, v/v) to afford the pure product **1N (**39.7 mg, 75% yield, 90% ee, yellow solid).

To a flame-dried 10-mL Schlenk flask charged with a magnetic stirring bar, 3,4-dimethoxyaniline **5b** (0.025 mmol), 5.0 mol% [PdCl(allyl)]$_2$, 10.0 mol% *(R)*-**6b**, alcohol **1N** (0.06 mmol), aldehyde **4** (0.05 mmol) and 50 mg 4 Å MS in 1 mL DCM were sequentially added at -10 °C. Diazoacetate **2** (0.06 mmol) and enamine **3** (0.06 mmol) dissolved in DCM (1.0 mL) were added by syringe pump over 180 mins. The mixture was stirred for 1.0 hour at -10 °C. After the completion of the reaction, the reaction mixture was filtrated and the filtrate was evaporated in vacuo to give the crude

product. And then the crude product was purified by flash chromatography on silica gel (eluent: PE/EA = 50:1 ~ 30:1, v/v) to give the pure product **92** (62% yield, 29.5 mg, 95:5 d.r., 92% ee, yellow solid).

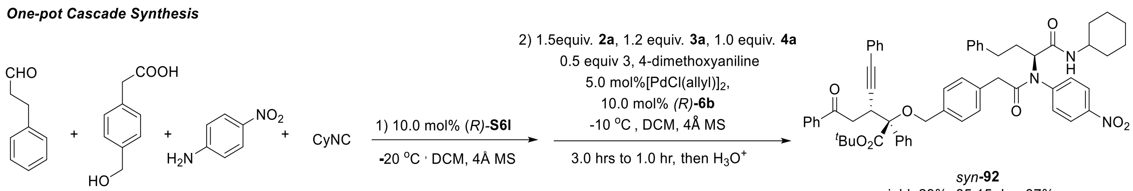

syn-**92**

yield: 29%, 85:15 d.r., 37% ee

To a flame-dried 10-mL Schlenk flask charged with a magnetic stirring bar, amine (0.065 mmol, 1.3 equiv.), acid (0.05 mmol, 1.0 equiv.), 10.0 mol% (*R*)-**S6l**, and dry DCM (1.0 mL) under argon. The resulting solution was stirred for 15 mins at -20 °C and then a solution of aldehyde (0.065 mmol) in DCM (0.5 mL) was added to the mixture in one portion. After 15 mins, a solution of isocyanide (0.065 mmol) in DCM (0.5 mL) was added to the mixture in one portion. Then the mixture was stirred at -20 °C for 12 hrs. Then 3, 4-dimethoxyaniline **5b** (0.025 mmol, 0.5 equiv.), 5.0 mol% $[PdCl(allyl)]_2$, 10.0 mol% **6b**, aldehyde **4a** (0.05 mmol, 1.0 equiv.) were sequentially added at -10 °C. Diazoacetate **2a** (0.075 mmol) and enamine **3a** (0.06 mmol, 1.2 equiv.) dissolved in DCM (0.5 mL) were added by syringe pump over 180 mins. The mixture was stirred for 1.0 hr at -10 °C. After the completion of the reaction, the reaction mixture was filtrated and the filtrate was evaporated in vacuo to give the crude product. And then the crude product was purified by flash chromatography on silica gel (eluent: PE/EA = 10:1 ~ 5:1, v/v) to give the pure product **92**.

2.17 Procedure for Synthesis of 93 from 38.

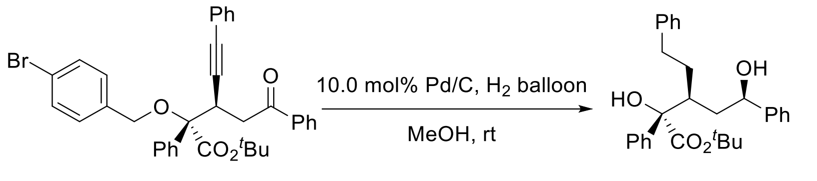

38 >20:1 d.r., 94 % ee

93 >20:1 d.r., 92% ee

To a flame-dried 10-mL Schlenk flask charged with a magnetic stirring bar, **38** (60.8 mg, 0.10mmol), Pd/C (1.1 mg, 10.0 mol%) in 4.0mL MeOH were sequentially added and stirred for overnight with a hydrogen-filled balloon. The reaction mixture the reaction mixture was filtered, and the filtrate were concentrated, the crude product was purified by flash chromatography on silica gel (eluent: PE/EA = 30:1 ~ 10:1, v/v) to afford the pure product **93 (**38.4 mg, 86% yield, >20:1 d.r., 92% ee, colorless oil).

2.18 Procedure for Synthesis of 94 from 42. [19]

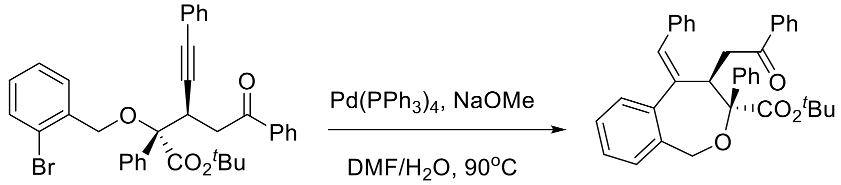

42 92:8 d.r., 88 % ee

94 92:8 d.r., 88% ee

To a flame-dried 10-mL Schlenk flask charged with a magnetic stirring bar, **42** (61.0 mg, 0.1 mmol, 1.0 equiv.), 0.5 mol% Pd(PPh_3)$_4$ (5.8 mg), NaOMe (16.2 mg, 0.3 mmol, 3.0 equiv.) in 5.0 mL DMF and H_2O (v_{DMF} : v_{H2O} =7:1) under argon, and the flask was heated to 90 °C under argon for 4.0 hrs. The mixture was extracted with ethyl acetate and the combined organic layers were washed with 2N HCl (× 3) and brine, dried with Na_2SO_4, the mixture was then cooled down and partitioned by CH_2Cl_2 and water, and the aqueous layer was extracted with CH_2Cl_2 (4.0 mL x 3). The organic layers were combined together and dried over Na_2SO_4. After filtration, the solvent was removed under vacuum, the crude product was purified by by flash chromatography on silica gel (eluent: PE/EA = 2:1 ~ 10:1, v/v) to afford the product **94 (**38.2 mg, 75% yield, 92:8

d.r., 88% ee, colorless oil).

2.19 Procedure for Synthesis of S26, S28 from 41. [20]

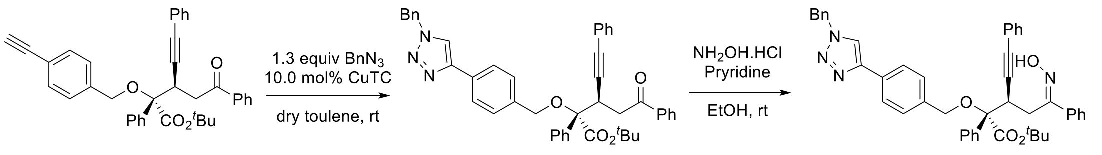

41 >20:1 d.r., 96% ee

S27 yield: 88%, >20:1 d.r., 94% ee

S28 yield: 90%, >20:1 d.r., 94% ee

To a flame-dried 10-mL Schlenk flask charged with a magnetic stirring bar, 10.0 mol% CuTC, **41** (55.4 mg, 0.10 mmol) in 2.0 mL of dry toluene under a dinitrogen atmosphere was at room temperature. BnN_3 (17.3mg, 0.13 mmol) in dry toluene (1.0 mL) were introduced by syringe pump over 10.0 mins and the reaction solution was stirred for another 2.0 hr. The reaction was concentrated in vacuo, the crude product was purified by flash chromatography on silica gel (eluent: PE/EA = 10:1 ~ 5:1, v/v) to afford the pure product **S27** (60.4 mg, 88% yield, 10:1 d.r, >20:1 d.r., 94% ee, colorless oil).

To a flame-dried 10-mL Schlenk flask charged with a magnetic stirring bar, **S27** (34.4 mg, 0.05 mmol, 1.0 equiv.) in ethanol was added pyridine (15.8 mg, 0.2 mmol, 4.0 equiv.) and hydroxylamine hydrochloride (13.9 mg, 0.2 mmol, 4.0 equiv.) at room temperature. The mixture was stirred overnight. The mixture was extracted with ethyl acetate and the combined organic layers were washed with 2.0 N HCl (× 3) and brine, dried with Na_2SO_4, the reaction mixture the reaction mixture was filtered, and the filtrate were concentrated, the crude product was purified by flash chromatography on silica gel (eluent: PE/EA = 5:1 ~ 2:1, v/v) to afford the pure product **S28 (**31.6 mg, 90% yield, >20:1 d.r., 94% ee, white solid).

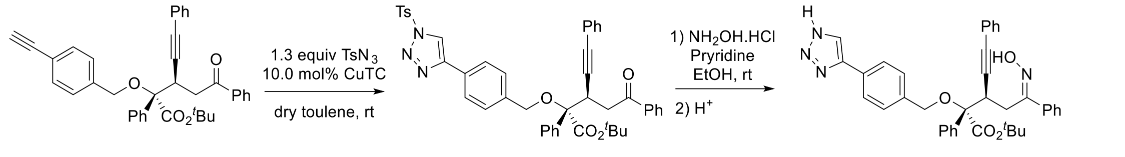

41
>20:1 d.r., 96% ee

S25
yield: 86%, >20:1 d.r., 94% ee

S26
yield: 75%, >20:1 d.r., 92% ee

To a flame-dried 10-mL Schlenk flask charged with a magnetic stirring bar, 10.0 mol% CuTC, **41** (55.4 mg, 0.10 mmol) in 2.0 mL of dry toluene under a dinitrogen atmosphere was at room temperature. TsN_3 (25.6 mg, 0.13 mmol) in dry toluene (1.0 mL) were introduced by syringe pump over 10.0 mins and the reaction solution was stirred for another 2.0 hr. The reaction was concentrated in vacuo, the crude product was purified by flash chromatography on silica gel (eluent: PE/EA = 10:1 ~ 5:1, v/v) to afford the pure product **S25** (64.6mg, 86% yield, 10:1 d.r, >20:1 d.r., 94% ee, colorless oil).

To a flame-dried 10-mL Schlenk flask charged with a magnetic stirring bar, **S25** (37.6 mg, 0.05 mmol, 1.0 equiv.) in ethanol was added pyridine (15.8 mg, 0.2 mmol, 4.0 equiv.) and hydroxylamine hydrochloride (13.9 mg, 0.2 mmol, 4.0 equiv.) at room temperature. The mixture was stirred overnight. The mixture was extracted with ethyl acetate and the combined organic layers were washed with 2.0 N HCl (× 3) and brine, dried with Na_2SO_4, The reaction mixture the reaction mixture was filtered, and the filtrate were concentrated, the crude product was purified by flash chromatography on silica gel (eluent: PE/EA = 5:1 ~ 2:1, v/v) to afford the pure product **S26 (**23.0 mg, 75% yield, >20:1 d.r., 92% ee, white solid).

2.20 Procedure for Synthesis of 95 and 95-b.

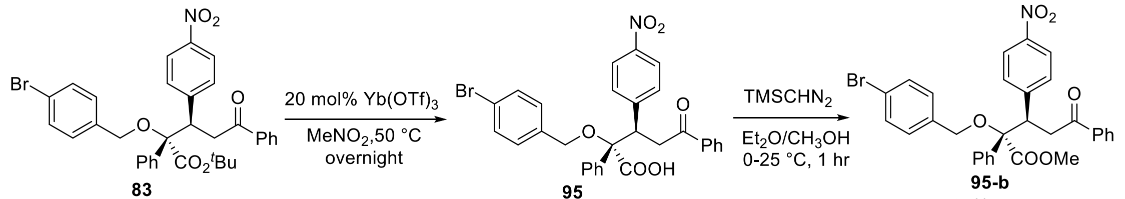

83
89% ee

95
75% yield

95-b
89% yield
89% ee

To a flame-dried 10-mL Schlenk flask charged with a magnetic stirring bar, **83** (62.9

mg, 0.1 mmol, 1.0 equiv.), 20.0 mol% $Yb(OTf)_3$ in 3 mL $MeNO_2$. The mixture was stirred at 50 °C for overnight. The mixture was concentrated, the crude product was purified by flash chromatography on silica gel (eluent: PE/EA = 2:1 ~ 1:2, v/v) to afford the pure product **95 (**43.1 mg, 75% yield).

To a flame-dried 10-mL Schlenk flask charged with a magnetic stirring bar, **95** (57.44 mg, 0.10 mmol) in 1.0 mL Et_2O/MeOH (v: v=1:1) at room temperature. $TMSCHN_2$ (3.0 equiv.) was added to the reaction slowly for 5.0 mins. The reaction solution was stirred for 1 hr. The reaction solution was slowly added saturated $NaHCO_3$(aq) before extracted with EtOAc (5 x 3 mL). The organic layers were washed with brine. After drying over $MgSO_4$ and concentration in vacuo, the crude product was purified by flash chromatography on silica gel (eluent: DCM/MeOH = 40:1 ~ 20:1, v/v) to afford the pure product **95-b (**52.4 mg, 70% yield, 89% ee, colorless oil).

2.21 Procedure for Synthesis of 96, 97, and 97-b.

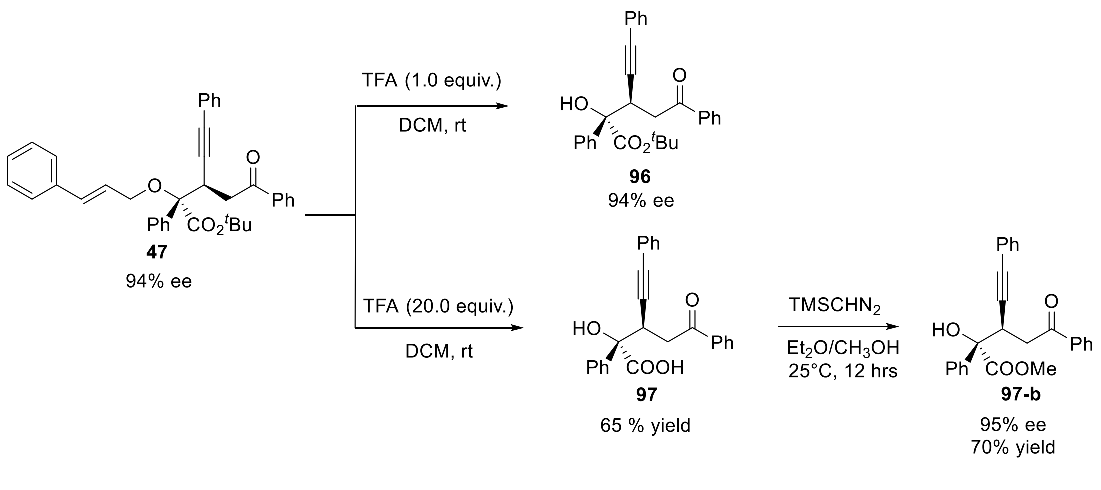

To a flame-dried 10-mL Schlenk flask charged with a magnetic stirring bar, **47** 55.6 mg (0.10 mmol) in 4.0 mL DCM at room temperature. TFA (1.0 equiv.) was added to the reaction slowly for 2.0 mins. The reaction solution was stirred for overnight. The reaction solution was slowly added saturated $NaHCO_3$(aq) before extracted with EtOAc (5 x 3 mL). The organic layers were washed with brine. After drying over $MgSO_4$ and

concentration in vacuo, the crude product was purified by flash chromatography on silica gel (eluent: PE/EA = 30:1 ~ 10:1, v/v) to afford the pure product **96 (**26.4 mg, 60% yield, >20:1 d.r., 94% ee, colorless oil).

To a flame-dried 10-mL Schlenk flask charged with a magnetic stirring bar, **47** 55.6 mg (0.10 mmol) in 4.0 mL DCM at room temperature. TFA (20.0 equiv.) was added to the reaction slowly for 2.0 mins. The reaction solution was stirred for overnight. The reaction solution was slowly added saturated NH_4Cl(aq) before extracted with EtOAc (5 x 3 mL). The organic layers were washed with brine. After drying over $MgSO_4$ and concentration in vacuo, the crude product was purified by flash chromatography on silica gel (eluent: PE/EA = 20:1 ~ 10:1, v/v) to afford the pure product **97 (**25.0 mg, 65% yield, >20:1 d.r., colorless oil).

To a flame-dried 10-mL Schlenk flask charged with a magnetic stirring bar, **97** 38.4 mg (0.10 mmol) in 1.0 mL Et_2O/MeOH (v: v=1:1) at room temperature. $TMSCHN_2$ (3.0 equiv.) was added to the reaction slowly for 5.0 mins. The reaction solution was stirred for 12 hrs. The reaction solution was slowly added saturated $NaHCO_3$(aq) before extracted with EtOAc (5 x 3 mL). The organic layers were washed with brine. After drying over $MgSO_4$ and concentration in vacuo, the crude product was purified by flash chromatography on silica gel (eluent: DCM/MeOH = 40:1 ~ 20:1, v/v) to afford the pure product **97-b (**27.9 mg, 70% yield, >20:1 d.r., 95% ee, colorless oil).

2.22 Conclusion of 6CRs/7CRs design and synthetic applications.

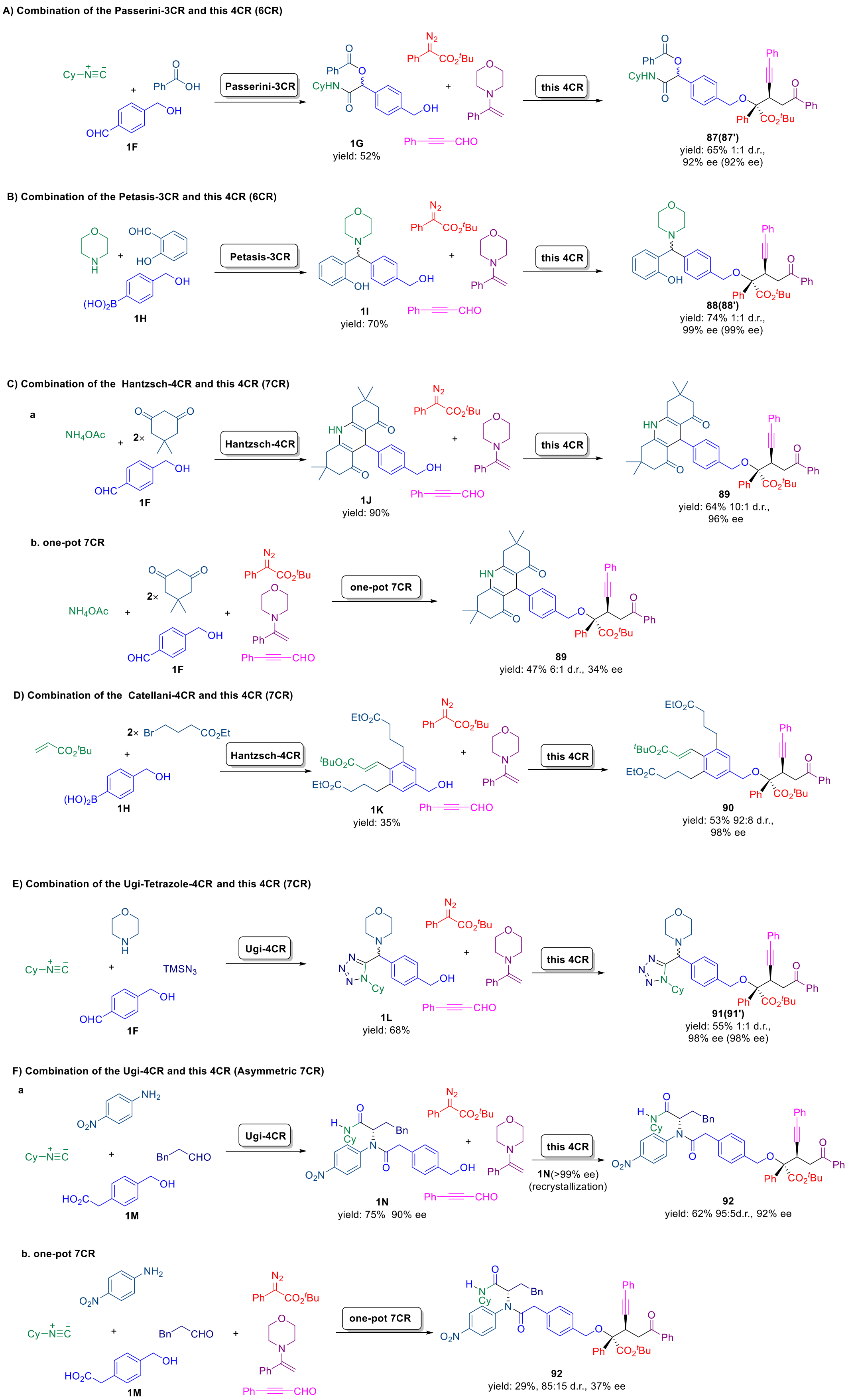

Supplementary Figure 3. Higher-order MCRs design

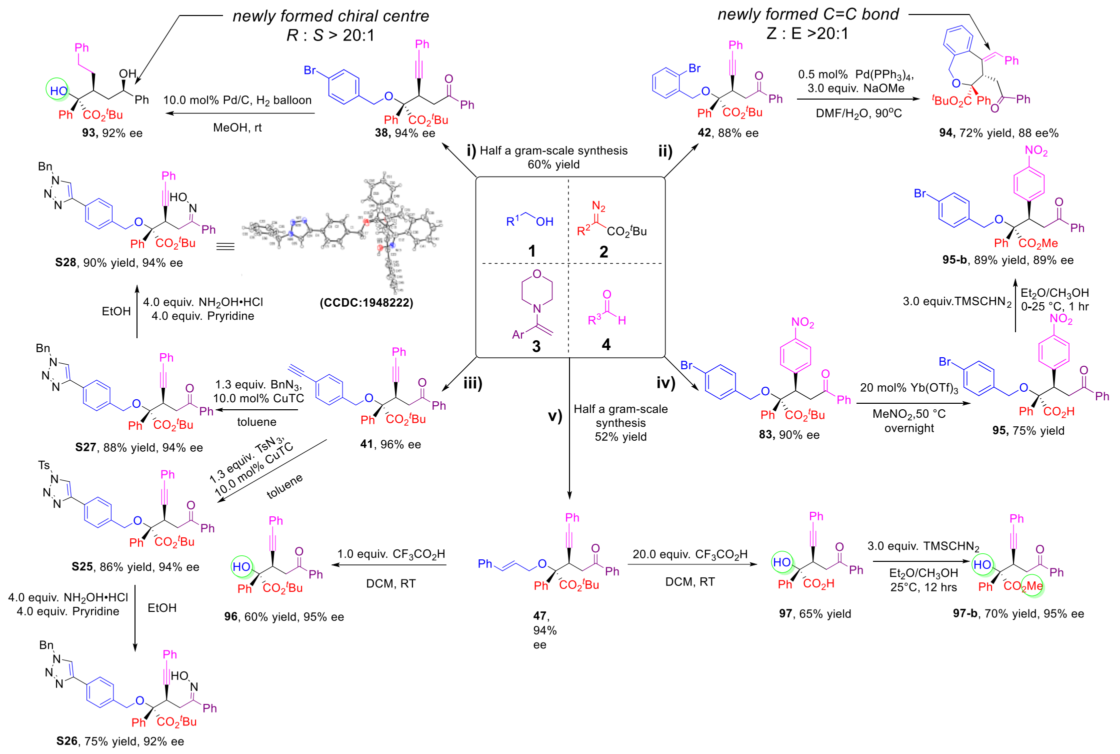

Supplementary Figure 4. Synthetic application

2.23 Preparation of [Pd(allyl)(*R*)-6b].[21]

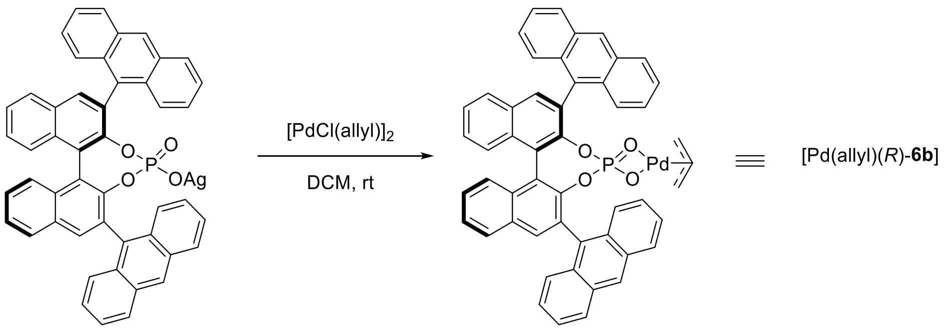

To a flame-dried 25-mL Schlenk flask charged with a magnetic stirring bar, silver phosphate (161.5 mg, 0.2 mmol, 1.0 equiv.) was dissolved in 5.0 mL DCM and then Schlenk flask was placed in dark. Subsequently $[PdCl(allyl)]_2$ (161.5 mg, 0.2 mmol, 1.0 equiv.) was added. The resulting mixture was stirred overnight at room temperature. The mixture was filtered and the filtrate was concentrated to give a fluffy yellowish powder [Pd(allyl)(*R*)-**6b**] (118.47 mg, 70% yield).

3. Supplementary Table 1-2 for Detailed Racemic Condition Optimizations

Supplementary Table 1. Condition Optimization of Racemic Four-component Reaction [a]

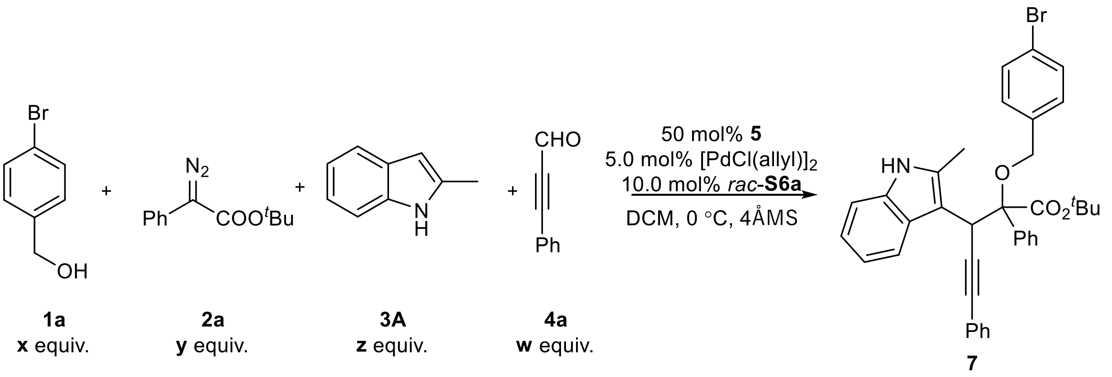

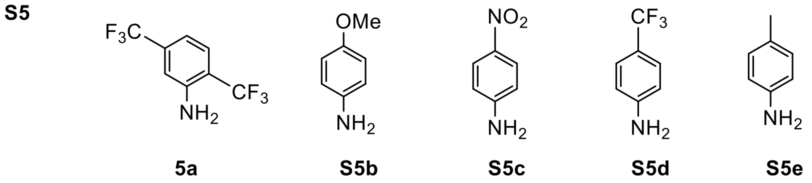

Entry	III-5	x (equiv.)	y (equiv.)	z (equiv.)	w (equiv.)	dr[b]	Yield (%)[c]
1	**5a**	1.2	1.5	1.2	1.0	>20:1	25
2	**S5b**	1.2	1.5	1.2	1.0	-	ND
3	**S5c**	1.2	1.5	1.2	1.0	-	ND
4	**S5d**	1.2	1.5	1.2	1.0	-	ND
5	**S5e**	1.2	1.5	1.2	1.0	-	ND
6	**5a**	1.2	2.0	1.2	1.0	>20:1	20
7	**5a**	1.2	1.5	1.0	3.0	>20:1	44
8	**5a**	1.2	1.5	1.0	4.0	>20:1	42
9	**5a**	1.5	1.5	1.0	3.0	>20:1	42
10	**5a**	2.0	1.5	1.0	3.0	>20:1	54

11	**5a**	3.0	1.5	1.0	3.0	>20:1	63
12	**5a**	4.0	1.5	1.0	3.0	>20:1	56

[a] Standard conditions: **2b** and **3A** in 1.0 mL DCM was added into a solution of **1a**, **4a**, **5**, $[Pd(allyl)Cl]_2$ (5 mol%), *rac*-**S6a** (10 mol%) and 4 Å MS (70 mg) in 1.0 mL DCM via a syringe pump for 60 mins, and the resulting mixture was stirred for another 6 hr. [b] Determined by ^{1}H NMR spectroscopy analyses. [c] Determined by ^{1}H NMR spectroscopy analyses using 1, 3, 5-trimethoxybenzene as an internal standard.

Procedure

To a flame-dried 10-mL Schlenk flask charged with a magnetic stirring bar, alcohol **1a** (0.15 mmol), aldehyde **4a** (0.15 mmol), **5** (50 mol%), $[PdCl(allyl)]_2$ (5 mol%), *rac*-**S6a** (10 mol%) and 4Å MS (70 mg) in DCM (1.0 mL), was added a mixture of diazoacetate **2a** (0.075 mmol) and indole **3A** (0.05 mmol) in DCM (1.0 mL) for 1.0 h via a syringe pump at 0 °C. The mixture was stirred for additional 6 hrs under these conditions. After the completion of the reaction, the reaction mixture was filtrated and the filtrate was evaporated in vacuo to give the crude product. And then the crude product was purified by flash chromatography on silica gel (eluent: PE: EA = 20 : 1) to afford the pure products **7**.

Supplementary Table 2. Racemic Condition Optimization [a]

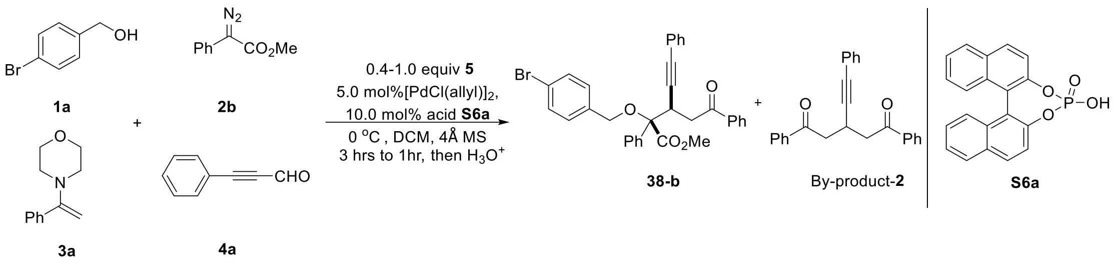

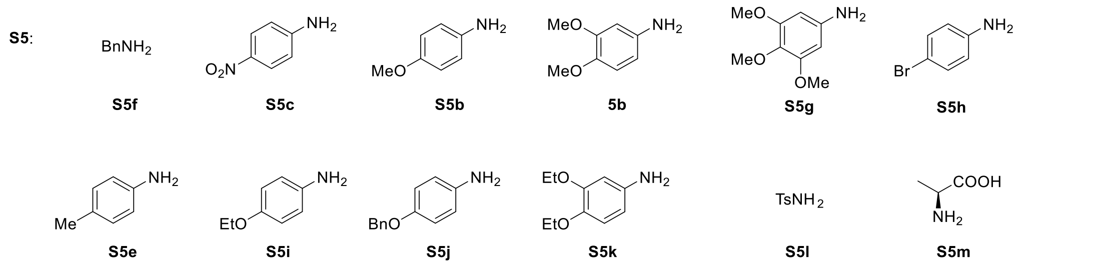

entry	**S5(equiv.)**	Acid	Yield of **7-b**/%[b]	d.r.[c]	Yield of by-product-**2**/%[b]
1	**S5f**(0.5)	**S6a**	0	ND	81
2	**S5c**(0.5)	**S6a**	13	3:1	70
3	**S5b**(0.5)	**S6a**	32	3:1	47
4	**5b**(0.5)	**S6a**	45	3:1	39
5	**S5g**(0.5)	**S6a**	35	3:1	41
6	**S5h**(0.5)	**S6a**	16	3:1	62
7	**S5e**(0.5)	**S6a**	31	3:1	52
8	**S5i**(0.5)	**S6a**	33	3:1	46
9	**S5j**(0.5)	**S6a**	36	3:1	42
10	**S5k**(0.5)	**S6a**	35	3:1	41
11	**S5l**(0.5)	**S6a**	33	4:1	45
12	**S5m**(0.5)	**S6a**	39	2:1	45
13	**5b**(0.5)	TsOH	7	ND	61
14	**5b**(0.5)	TFOH	0	ND	< 5
15	**5b**(0.5)	HCOOH	13(14)[d]	3:1	64(69)[e]
16	**5b**(0.5)	EtCOOH	10	3:1	67

17	**5a**(0.5)	4-NO_2PhCOOH	14	3:1	63
18	**5a**(0.4)	**S6a**	30	3:1	43
19	**5a**(1.0)	**S6a**	39	3:1	40

[a] Standard condition: **1a**/**2b**/**3a**/**4a**/**5**/[PdCl(allyl)]$_2$/acid = 0.18/0.18/0.18/0.15/0.06-0.15/0.0075/0.015-0.03 mmol, **2b** and **3a** in 1.0 mL dry DCM were added into a solution of **1a**, **4a**, [PdCl(allyl)]$_2$, acid, **5** and 100 mg 4 Å MS in 2.0 mL dry DCM via a syringe pump under a dinitrogen atmosphere for 180 mins, and the resulting mixture was stirred for another 1.0 hr. [b] Isolated yield. [c] Determined by ^{1}H NMR spectroscopy analyses. [d] Adding amount of HCOOH is 50.0 mol%.

Procedure

To a flame-dried 10-mL Schlenk flask charged with a magnetic stirring bar, **5** (0.075 mmol), 5.0 mol% [PdCl(allyl)]$_2$, 10.0-50.0 mol% acid, alcohol **1a** (0.18 mmol), aldehyde **4a** (0.15 mmol) and 100 mg 4 Å MS in 2.0 mL DCM were sequentially added at 0 °C. Diazoacetate **2b** (0.18 mmol) and enamine **3a** (0.18 mmol) dissolved in DCM (1.0 mL) were added by syringe pump over 180 mins. The mixture was stirred for 1.0 hr at 0 °C. After the completion of the reaction, the reaction mixture was filtrated and the filtrate was evaporated in vacuo to give the crude product. And then the crude product was purified by flash chromatography on silica gel (eluent: PE/EA = 50:1 ~ 30:1, v/v) to give the mixed products **38-b** containing *anti*-product and *syn*-product or *anti*-product.

4. Supplementary Table 3-4 for Detailed Condition Optimizations

Supplementary Table 3 Condition Optimization of Asymmetric Four-component Reaction [a]

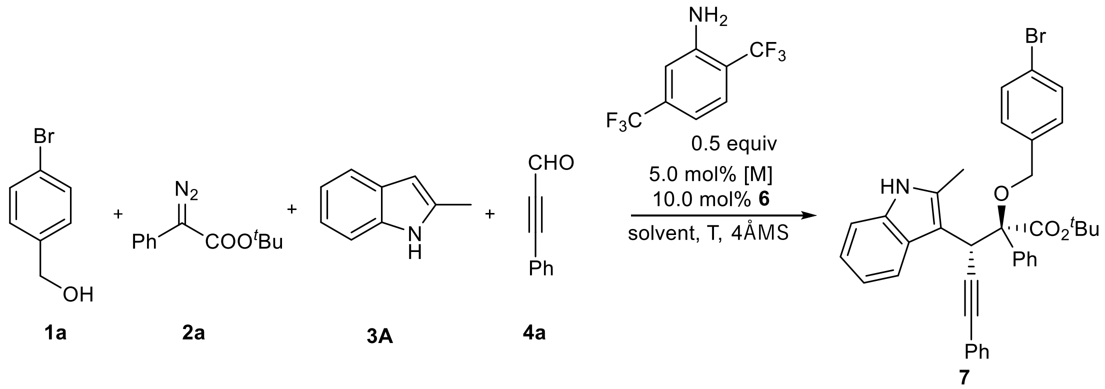

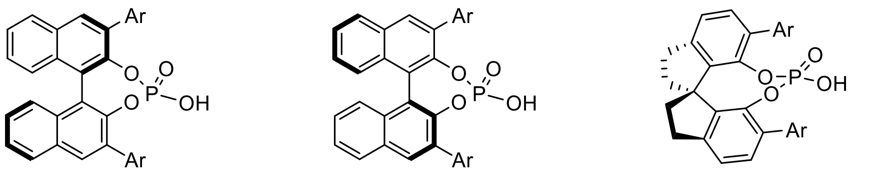

(R)-**6c**: Ar=C_6H_5
(R)-**6b**: Ar=9-anthryl
(R)-**S6b**: Ar=4-CF_3-C_6H_4

(S)-**S6c**: Ar=4-OMe-C_6H_4
(S)-**S6d**: Ar=$SiPh_3$
(S)-**S6e**: Ar=9-phenanthryl

(S)-**S6f**: Ar=$SiPh_3$
(S)-**S6g**: Ar=9-anthryl
(S)-**6a**: Ar=1-pyrenyl

Entry	**6**	[M]	Solvent	T/°C	dr[b]	Yield (%)[c]	ee (%)[d]
1	(*R*)-**6c**	$[PdCl(allyl)]_2$	DCM	0	>20:1	68	-36
2	(*R*)-**6b**	$[PdCl(allyl)]_2$	DCM	0	>20:1	70	-60
3	(*R*)-**S6b**	$[PdCl(allyl)]_2$	DCM	0	>20:1	73	-53
4	*(S)*-**S6c**	$[PdCl(allyl)]_2$	DCM	0	>20:1	40	47
5	*(S)*-**S6d**	$[PdCl(allyl)]_2$	DCM	0	-	ND	-
6	*(S)*-**S6e**	$[PdCl(allyl)]_2$	DCM	0	>20:1	69	84
7	*(S)*-**S6f**	$[PdCl(allyl)]_2$	DCM	0	-	ND	-
8	*(S)*-**S6g**	$[PdCl(allyl)]_2$	DCM	0	>20:1	51	87
9	*(S)*-**6a**	$[PdCl(allyl)]_2$	DCM	0	>20:1	50	92
10	*(S)*-**6a**	$[PdCl(2\text{-}butenyl)]_2$	DCM	0	>20:1	52	93
11	*(S)*-**6a**	$[PdCl(cinnyl)]_2$	DCM	0	>20:1	56	93
12	*(S)*-**6a**	$Pd(CH_3CN)_2Cl_2$	DCM	0	>20:1	64	93
13	*(S)*-**6a**	$Pd(C_6H_5CN)_2Cl_2$	DCM	0	>20:1	59	92

14	*(S)*-**6a**	$Pd(CH_3CN)_2Cl_2$	DCE	0	>20:1	71	93
15	*(S)*-**6a**	$Pd(CH_3CN)_2Cl_2$	PhCl	0	>20:1	60	90
16	*(S)*-**6a**	$Pd(CH_3CN)_2Cl_2$	MTBE	0	-	ND	-
17[e]	*(S)*-**6a**	$Pd(CH_3CN)_2Cl_2$	DCE	-10	>20:1	78	94
18[e]	*(S)*-**6a**	$Pd(CH_3CN)_2Cl_2$	DCE	-20	>20:1	83	94
19[e]	*(S)*-**6a**	$Pd(CH_3CN)_2Cl_2$	DCE	-30	>20:1	78	93

[a] Standard conditions: **1a/2a/3A/4a/5a/[M]/6** = 0.15/0.075/0.05/0.15/0.025/0.0025/0.005 mmol, **2a, 3A** in 1.0 mL solvent was added into a solution of **1a**, **4a**, **5a** (50 mol%), [M] (5 mol%), 6 (10 mol%), and 4 Å MS (50 mg) in 1.0 mL solvent via a syringe pump for 60 mins, and the resulting mixture was stirred for another 12 hours. [b] Determined by ^1H NMR spectroscopy analyses. [c] Determined by ^1H NMR spectroscopy analyses using 1, 3, 5-trimethoxybenzene as an internal standard. [d] Determined by HPLC analyses using a chiral stationary phase. [e] 10 mol% $Pd(CH_3CN)_2Cl_2$

Procedure

To a flame-dried 10-mL Schlenk flask charged with a magnetic stirring bar, alcohol **1a** (0.15 mmol), aldehyde **4a** (0.15 mmol), 2,5-bis(trifluoromethyl)aniline **5a** (50 mol%), [Pd] (5 mol%), **6** (10 mol%) and 4Å MS (50 mg) in solvent (1.0 mL), was added a mixture of diazoacetate **2a** (0.075 mmol) and indole **3A** (0.05mmol) in solvent (1.0 mL) for 1.0 h via a syringe pump at -20 ~ 0 ℃. The mixture was stirred for additional 12 hours under these conditions. After the completion of the reaction, the reaction mixture was filtrated and the filtrate was evaporated in vacuo to give the crude product. And then the crude product was purified by flash chromatography on silica gel (eluent: PE: EA = 20 : 1) to afford the pure products.

Supplementary Table 4. Condition Optimization for Four-component Reactions [a]

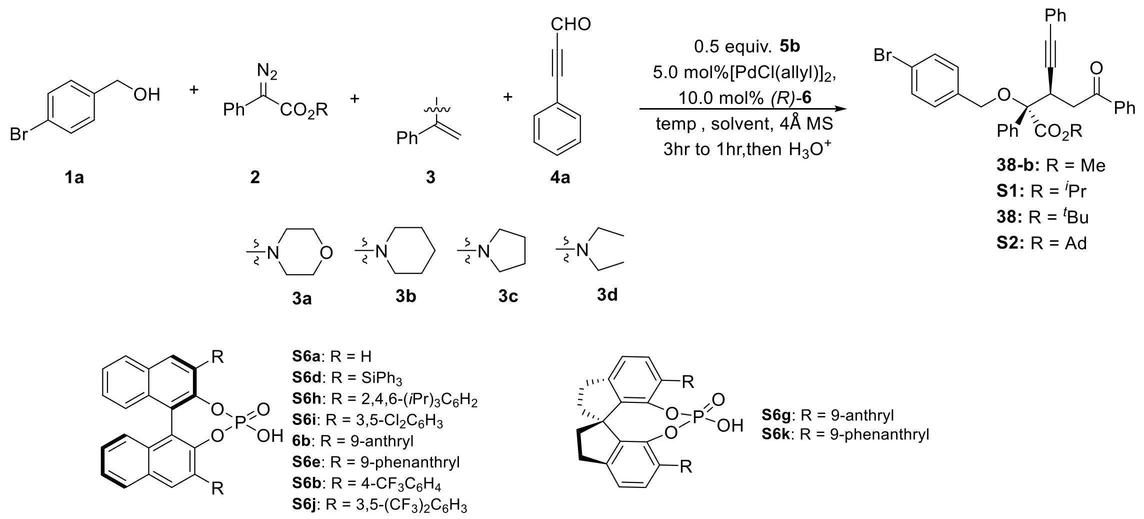

entry	S6	R	Temp/°C	Solvent	3	Product	Yield/%[b]	d.r.[c]	ee/%[d]
1	**S6a**	Me	0	DCM	**3a**	**38-b**	55	86:14	5
2	**S6d**	Me	0	DCM	**3a**	**38-b**	46	94:6	25
3	**S6h**	Me	0	DCM	**3a**	**38-b**	41	94:6	27
4	**S6i**	Me	0	DCM	**3a**	**38-b**	57	94:6	23
5	**6b**	Me	0	DCM	**3a**	**38-b**	60	96:4	52
6	**S6e**	Me	0	DCM	**3a**	**38-b**	51	96:4	53
7	**S6b**	Me	0	DCM	**3a**	**38-b**	60	95:5	33
8	**S6j**	Me	0	DCM	**3a**	**38-b**	42	90:10	11
9	**S6g**	Me	0	DCM	**3a**	**38-b**	46	90:10	13
10	**S6k**	Me	0	DCM	**3a**	**38-b**	43	98:2	38
11	**6b**	Me	-10	DCM	**3a**	**38-b**	67	94:6	46
12	**6b**	^{i}Pr	-10	DCM	**3a**	**S1**	63	96:4	80
13	**6b**	^{t}Bu	-10	DCM	**3a**	**38**	72	98:2	98
14	**6b**	Ad	-10	DCM	**3a**	**S2**	55	94:6	94
15	**6b**	^{t}Bu	-20	DCM	**3a**	**38**	46	95:5	98
16	**6b**	^{t}Bu	-10	DCE	**3a**	**38**	53	95:5	98

17	**6b**	^{t}Bu	-10	toluene	**3a**	**38**	62	96:4	98
18	**6b**	^{t}Bu	-10	$CHCl_3$	**3a**	**38**	44	98:2	95
19	**6b**	^{t}Bu	-10	DCM	**3b**	**38**	8	81:29	46
20	**6b**	^{t}Bu	-10	DCM	**3c**	**38**	< 5	-	-
21	**6b**	^{t}Bu	-10	DCM	**3d**	**38**	< 5	-	-

[a] Standard condition: **1a**/**2**/**3a**/**4a**/**5b**/[PdCl(allyl)]$_2$/**6** = 0.18/0.18/0.18/0.15/0.075/0.0075/0.015 mmol, **2** and **3a** in 1.0 mL dry solvent were added into a solution of **1a**, **4a**, [PdCl(allyl)]$_2$, **6**, aniline **5b** and 100 mg 4 Å MS in 1.5 mL dry solvent via a syringe pump under a nitrogen atmosphere for 3 hours, and the resulting mixture was stirred for another 1.0 hour. [b] Isolated yield. [c] Determined by ^{1}H NMR spectroscopy analyses or HPLC. [d] Determined by HPLC analyses using a chiral stationary phase.

Procedure

To a flame-dried 10-mL Schlenk flask charged with a magnetic stirring bar, 3,4-dimethoxyaniline **5b** (0.10 mmol), 5.0 mol% [PdCl(allyl)]$_2$, 10.0 mol% *(R)*-**6**, alcohol **1a** (0.18 mmol), aldehyde **4a** (0.15 mmol) and 100 mg 4 Å MS in 2.0 mL of solvent were sequentially added at -20～0 °C. Diazoacetate **2** (0.18 mmol) and enamine **3**(0.18 mmol) dissolved in solvent (1.0 mL) were added by syringe pump over 3 hours. The mixture was stirred for 1.0 hour at -20～0 °C. After the completion of the reaction, the reaction mixture was filtrated and the filtrate was evaporated in vacuo to give the crude product. And then the crude product was purified by flash chromatography on silica gel (eluent: PE/EA = 50:1 ~ 30:1, v/v) to give the product.

II. Supplementary Discussion

5. X-ray Diffraction Parameters and Data

1) The crystal structure of compound **21** has been deposited at the Cambridge

Crystallographic Data Centre (CCDC **2156063**). The data is available free of charge at www.ccdc.cam.ac.uk/conts/retrieving.html.

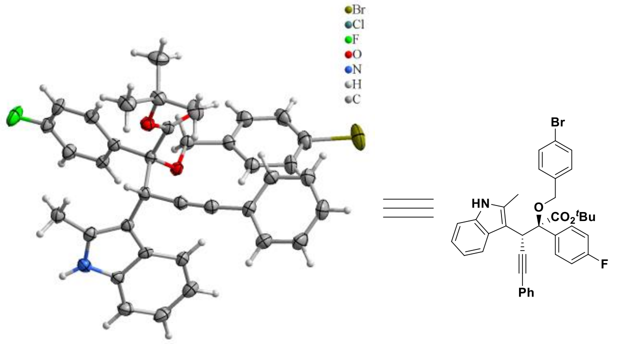


```
Bond precision:   C-C = 0.0079 A           Wavelength=1.54184

Cell:              a=11.9443(1)      b=18.1049(3)      c=31.5163(4)
                   alpha=90          beta=90           gamma=90
Temperature:       100 K

                  Calculated                    Reported
Volume            6815.41(15)                   6815.41(15)
Space group       P 21 21 21                    P 21 21 21
Hall group        P 2ac 2ab                     P 2ac 2ab
Moiety formula    2(C37 H33 Br F N O3), C H     2(C37 H33 Br F N O3), C H
                  Cl3                           Cl3
Sum formula       C75 H67 Br2 Cl3 F2 N2 O6      C75 H67 Br2 Cl3 F2 N2 O6
Mr                1396.46                       1396.47
Dx,g cm-3         1.361                         1.361
Z                 4                             4
Mu (mm-1)         3.068                         3.068
F000              2872.0                        2872.0
F000'             2878.12
h,k,lmax          15,23,40                      15,23,39
Nref              14768[ 8107]                  14446
Tmin,Tmax         0.604,0.736                   0.621,1.000
Tmin'             0.516

Correction method= # Reported T Limits: Tmin=0.621 Tmax=1.000
AbsCorr = MULTI-SCAN

Data completeness= 1.78/0.98        Theta(max)= 79.235

                                                 wR2(reflections)=
R(reflections)= 0.0564( 12951)                   0.1596( 14446)
S = 1.051                   Npar= 819

Alert level C
PLAT341_ALERT_3_C Low Bond Precision on  C-C Bonds ...............    0.00787 Ang.
PLAT420_ALERT_2_C D-H Bond Without Acceptor  N00E     --H00E     .     Please Check
PLAT420_ALERT_2_C D-H Bond Without Acceptor  N00F     --H00F     .     Please Check
PLAT601_ALERT_2_C Unit Cell Contains Solvent Accessible VOIDS of .         71 Ang**3
PLAT934_ALERT_3_C Number of (Iobs-Icalc)/Sigma(W) > 10 Outliers ..          1 Check

Alert level G
PLAT007_ALERT_5_G Number of Unrefined Donor-H Atoms ..............          2 Report
PLAT083_ALERT_2_G SHELXL Second Parameter in WGHT  Unusually Large       5.09 Why ?
PLAT371_ALERT_2_G Long   C(sp2)-C(sp1) Bond  C00O     - C00V     .       1.44 Ang.
PLAT371_ALERT_2_G Long   C(sp2)-C(sp1) Bond  C00P     - C01K     .       1.43 Ang.
PLAT434_ALERT_2_G Short Inter HL..HL Contact Br02        ..Cl2   .       3.47 Ang.
                                  1-x,-1/2+y,1/2-z  =           3_645 Check
PLAT720_ALERT_4_G Number of Unusual/Non-Standard Labels .........        154 Note
PLAT791_ALERT_4_G Model has Chirality at C00J          (Sohnke SpGr)       R Verify
PLAT791_ALERT_4_G Model has Chirality at C00M          (Sohnke SpGr)       R Verify
PLAT791_ALERT_4_G Model has Chirality at C00X          (Sohnke SpGr)       R Verify
PLAT791_ALERT_4_G Model has Chirality at C016          (Sohnke SpGr)       R Verify
PLAT912_ALERT_4_G Missing # of FCF Reflections Above STh/L=  0.600        86 Note
PLAT978_ALERT_2_G Number C-C Bonds with Positive Residual Density.         2 Info

   0 ALERT level A = Most likely a serious problem - resolve or explain
   0 ALERT level B = A potentially serious problem, consider carefully
   5 ALERT level C = Check. Ensure it is not caused by an omission or oversight
  12 ALERT level G = General information/check it is not something unexpected

   0 ALERT type 1 CIF construction/syntax error, inconsistent or missing data
   8 ALERT type 2 Indicator that the structure model may be wrong or deficient
   2 ALERT type 3 Indicator that the structure quality may be low
   6 ALERT type 4 Improvement, methodology, query or suggestion
   1 ALERT type 5 Informative message, check
```

Supplementary Figure 5. X-ray Diffraction Parameters and Data of **21**

2) The crystal structure of compound **S28** has been deposited at the Cambridge Crystallographic Data Centre (CCDC **1948222**). The data is available free of charge at www.ccdc.cam.ac.uk/conts/retrieving.html.

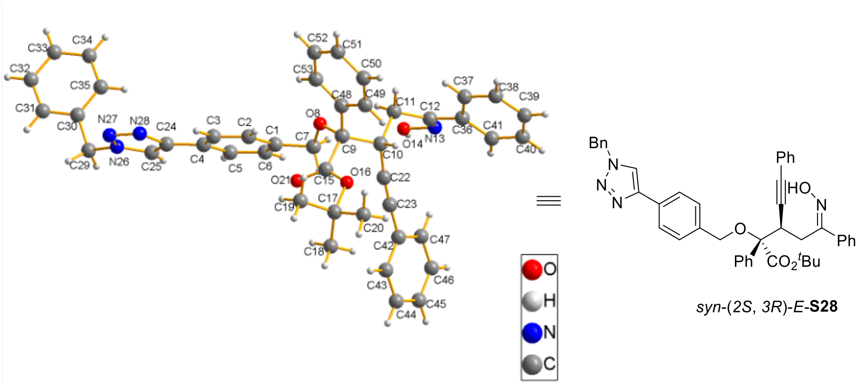

syn-(2*S*, 3*R*)-*E*-**S28**

The structure and absolute stereochemistry of ketoxime **S28** was confirmed by X-ray crystallographic analysis, which also could determine the structure and absolute stereochemistry of **41**.

```
Bond precision:   C-C = 0.0047 A          Wavelength=1.54184

Cell:             a=11.2326(2)     b=15.9229(2)     c=21.1892(3)
                  alpha=90         beta=90          gamma=90
Temperature:      100 K

                 Calculated                 Reported
Volume           3789.81(10)                3789.81(10)
Space group      P 21 21 21                 P 21 21 21
Hall group       P 2ac 2ab                  P 2ac 2ab
Moiety formula   C45 H42 N4 O4              C45 H42 N4 O4
Sum formula      C45 H42 N4 O4              C45 H42 N4 O4
Mr               702.83                     702.82
Dx,g cm-3        1.232                      1.232
Z                4                          4
Mu (mm-1)        0.631                      0.631
F000             1488.0                     1488.0
F000'            1492.30
h,k,lmax         14,20,26                   13,19,26
Nref             8021[ 4470]                7799
Tmin,Tmax        0.939,0.939                0.685,1.000
Tmin'            0.939

Correction method= # Reported T Limits: Tmin=0.685 Tmax=1.000
AbsCorr = MULTI-SCAN

Data completeness= 1.74/0.97       Theta(max)= 77.092

R(reflections)= 0.0439( 6912)      wR2(reflections)= 0.1199( 7799)

S = 1.044                    Npar= 482
```

```
Alert level C
PLAT340_ALERT_3_C Low Bond Precision on  C-C Bonds ...............    0.00465 Ang.

Alert level G
PLAT007_ALERT_5_G Number of Unrefined Donor-H Atoms ..............          1 Report
PLAT371_ALERT_2_G Long   C(sp2)-C(sp1) Bond  C23      - C42     .        1.43 Ang.
PLAT791_ALERT_4_G Model has Chirality at C9            (Chiral SPGR)        S Verify
PLAT791_ALERT_4_G Model has Chirality at C10           (Chiral SPGR)        R Verify
PLAT883_ALERT_1_G No Info/Value for _atom_sites_solution_primary .     Please Do !
PLAT912_ALERT_4_G Missing # of FCF Reflections Above STh/L=  0.600         58 Note
PLAT978_ALERT_2_G Number C-C Bonds with Positive Residual Density.          5 Info
PLAT992_ALERT_5_G Repd & Actual _reflns_number_gt Values Differ by          1 Check

 0 ALERT level A = Most likely a serious problem - resolve or explain
 0 ALERT level B = A potentially serious problem, consider carefully
 1 ALERT level C = Check. Ensure it is not caused by an omission or oversight
 8 ALERT level G = General information/check it is not something unexpected

 1 ALERT type 1 CIF construction/syntax error, inconsistent or missing data
 2 ALERT type 2 Indicator that the structure model may be wrong or deficient
 1 ALERT type 3 Indicator that the structure quality may be low
 3 ALERT type 4 Improvement, methodology, query or suggestion
 2 ALERT type 5 Informative message, check
```

Supplementary Figure 6. X-ray Diffraction Parameters and Data of **S28**

6. ECD Parameters and Data of 93

The calculated and experimental ECD spectra are shown as below (to get better accuracy, the experimental data are not processed). Obviously, the calculated ECD spectra for the SSR diastereomer are more consistent with the experimental data. Therefore, we think the SSR one is more likely to be the actual product.

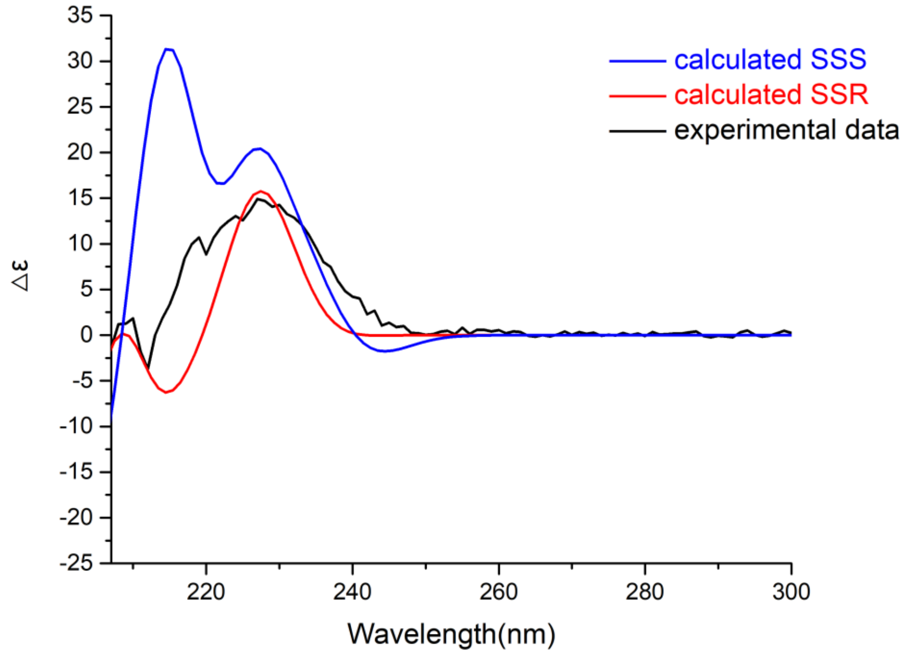

Supplementary Figure 7. Comparision of B3LPY/6-311G(d,p)-calculated ECD with the experimental data in MeOH

Computational Methods.

MMFF and DFT/TDDFT calculations were perform with the molclus[22] software package and Gaussion 09[23] software package. The MMFF calculation was to do the conformational search and obtain 10 of the lowest configurations. Their geometries were then optimized at the B3LYP[242]/6-311G(d,p) level of theory combined with the SMD[25] model in MeOH. The TDDFT calculation were performed with the same level

to get the ECD data. Finally, the Boltzmann weighted summation of spectra data were form by using SpecDis[26] software.

7. Control Experiments

7.1 Control Experiment 1

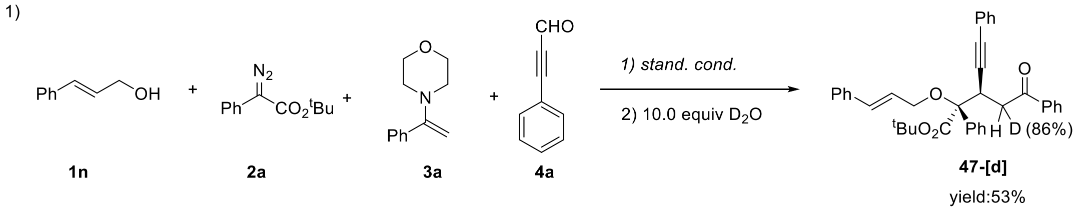

To a flame-dried 10-mL Schlenk flask charged with a magnetic stirring bar, 3,4-dimethoxyaniline (0.10 mmol), 5.0 mol% $[PdCl(allyl)]_2$, 10.0 mol% *(R)*- **6b**, alcohol **1n** (0.24 mmol), aldehyde **4a** (0.20 mmol) and 100 mg 4 Å MS in 2.0 mL DCM were sequentially added at -10 ºC. Diazoacetate **2a** (0.24 mmol) and enamine **3a** (0.24 mmol) dissolved in DCM (1.0 mL) were added by syringe pump over 60 mins. The mixture was stirred for 1.0 hr at -10 ºC. After the completion of the reaction, D_2O (10.0 equiv.) was added in one portionthe and stirred for 2.0 hrs. The reaction mixture was filtrated and the filtrate was evaporated in vacuo to give the crude product. And then the crude product was purified by flash chromatography on silica gel (eluent: PE/EA = 50:1 ~ 30:1, v/v) to give the product **47**-[d] (59.0 mg, 53% yield). The yield of deuterated product **47**-[d] was detected by ^{1}H NMR (86%).

7.2 Control Experiment 2

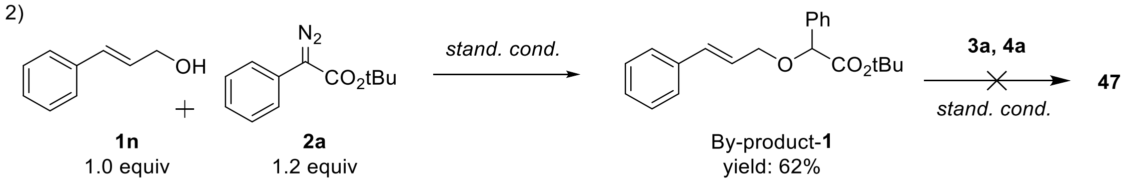

To a flame-dried 10-mL Schlenk flask charged with a magnetic stirring bar, 2.0 mol% $Rh_2(OAc)_4$, cinnamyl alcohol **1n** (0.24 mmol) and 100 mg 4 Å MS in 2.0 mL DCM

were sequentially added at -10 °C. Diazoacetate **2a** (0.24 mmol) dissolved in DCM (1.0 mL) were added by syringe pump over 60 mins. The mixture was stirred for 1.0 hr at -10 °C. After the completion of the reaction, the reaction mixture was filtrated and the filtrate was evaporated in vacuo to give the crude product. And then the crude product was purified by flash chromatography on silica gel (eluent: PE/EA = 50:1 ~ 30:1, v/v) to give the pure By-product-**1**.

To a flame-dried 10-mL Schlenk flask charged with a magnetic stirring bar, 3,4-dimethoxyaniline (0.10 mmol), 5.0 mol% [PdCl(allyl)]$_2$, 10.0 mol% *(R)*-**6b**, By-product-**1** (0.24 mmol), aldehyde **4a** (0.20 mmol) and 100 mg 4 Å MS in 2.0 mL DCM were sequentially added at -10 °C. Enamine **3a** (0.24 mmol) dissolved in DCM (1.0 mL) were added by syringe pump over 60 mins. The mixture was stirred for 1.0 hr at -10 °C. After the completion of the reaction, the target **47** could not be detected by LC-MS.

7.3 Control Experiment 3

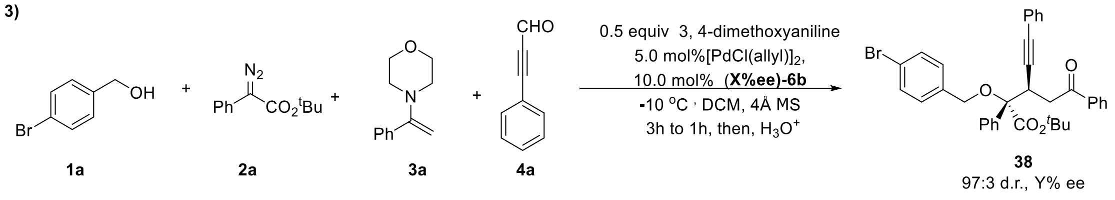

To a flame-dried 10-mL Schlenk flask charged with a magnetic stirring bar, 3,4-dimethoxyaniline **5b** (0.10 mmol), 5.0 mol% [PdCl(allyl)]$_2$, 10.0 mol% *(*X%ee*)*-**6b**, alcohol **1a** (0.24 mmol), aldehyde **4a** (0.20 mmol) and 100 mg 4 Å MS in 2.0 mL DCM were sequentially added at -10 °C. Diazoacetate **2a** (0.24 mmol) and enamine **3a** (0.24 mmol) dissolved in DCM (1.0 mL) were added by syringe pump over 60 mins. The mixture was stirred for 1.0 hr at -10 °C. After the completion of the reaction, the ee value of **38** was determined by HPLC analyses using a chiral stationary phase. The linear effect result: $y = 1.0036x + 1.0587$, $R^2 = 0.9969$.

Result:

Entry	x<br>*ee* of CPA-**6b**/%	y<br>*ee* of product **38**/%
1	0	0
2	4	3
3	6	8
4	24	25
5	42	44
6	66	71
7	74	78
8	96	95
9	99	98

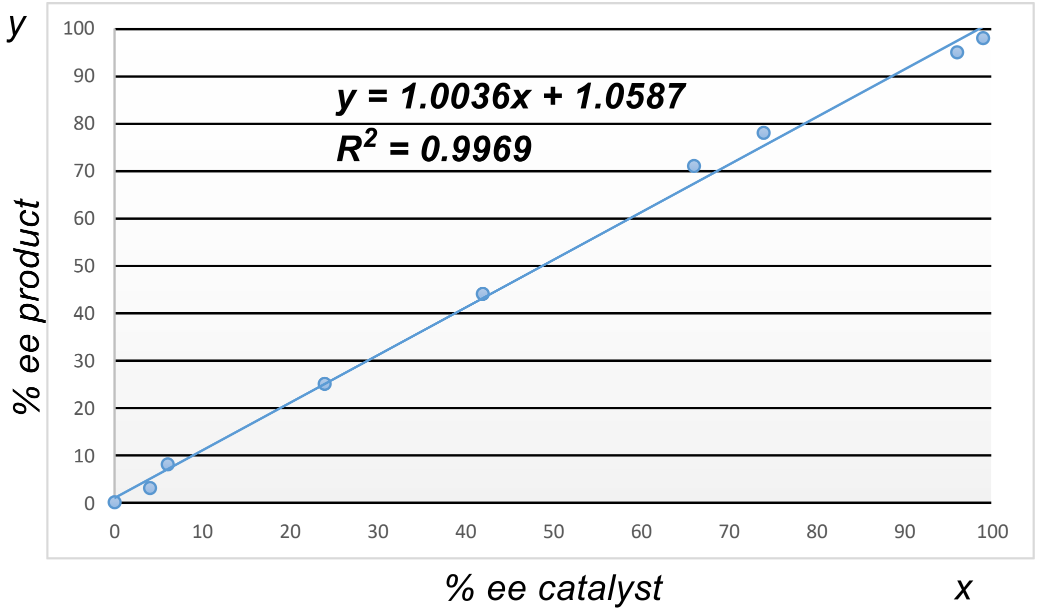

7.4 Control Experiment 4

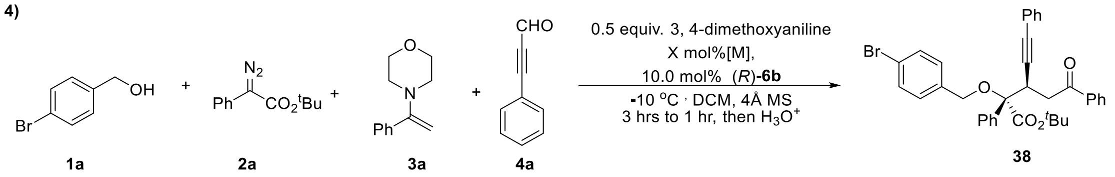

To a flame-dried 10-mL Schlenk flask charged with a magnetic stirring bar, 3,4-dimethoxyaniline **5b** (0.10 mmol), x mol% [M], 10.0 mol% *(R)*-**6b**, alcohol **1a** (0.24

mmol), aldehyde **4a** (0.20 mmol) and 100 mg 4 Å MS in 2.0 mL DCM were sequentially added at -10 ºC. Diazoacetate **2a** (0.24 mmol) and enamine **3a** (0.24 mmol) dissolved in DCM (1.0 mL) were added by syringe pump over 180 mins. The mixture was stirred for 1.0 hr at -10 ºC. After the completion of the reaction, he reaction mixture was filtrated and the filtrate was evaporated in vacuo to give the crude product. And then the crude product was purified by flash chromatography on silica gel (eluent: PE/EA = 50:1 ~ 30:1, v/v) to give the product **38**, and d.r and ee value of **38** were determined by HPLC analyses using a chiral stationary phase.

Result:

Entry	[M]/mol%	yield of **38**/%	d.r. of **38**	ee of **38**/%
1	$[PdCl(allyl)]_2$/(5.0)	72	98:2	98
2	$[PdCl(2\text{-butenyl})]_2$/(5.0)	55	98:2	98
3	$[PdCl(cinnyl)]_2$/(5.0)	61	96:4	96
4	$[PdOAc(allyl)]_2$/(5.0)	40	80:20	39
5	$[PdTFA(allyl)]_2$/(5.0)	53	90:10	60
6	$Rh_2(OAc)_4$/(2.0)	37	98:2	98
7	$Rh_2(Oct)_4$/(2.0)	28	98:2	98
8	$Rh_2(esp)_2$/(1.0)	30	98:2	98
9	$[Ir(COD)Cl]_2$/(5.0)	0	ND	ND
10	CP*Ru(COD)Cl/(5.0)	0	ND	ND
11	CuOTf/(5.0)	0	ND	ND
12	$Cu(MeCN)_4PF_6$/(5.0)	0	ND	ND

7.5 NMR Tube Experiments

(1) The effect of CPA for the formation of imines

a. Absence of 6b(CPA)

To a flame-dried NMR tube charged with 0.1 mmol **3a**, 0.1 mmol **5b** in 1.0 mL of CD_2Cl_2. The mixture was shocked by ultrasonic cleaning machine for several times. The reaction progress was monitoring at 5 mins, 20 mins, 40 mins, 60 mins by ^{1}H NMR respectively.

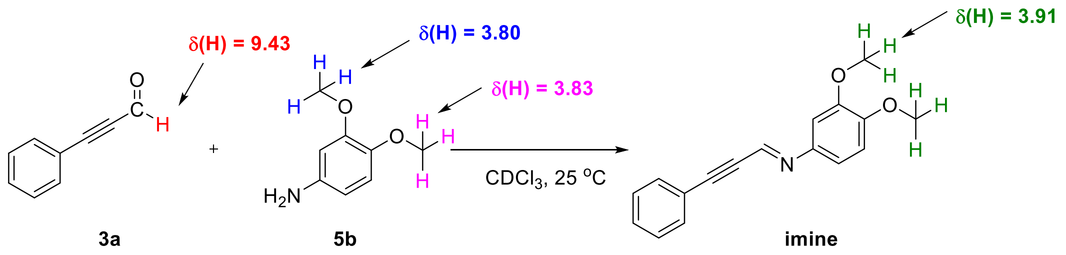

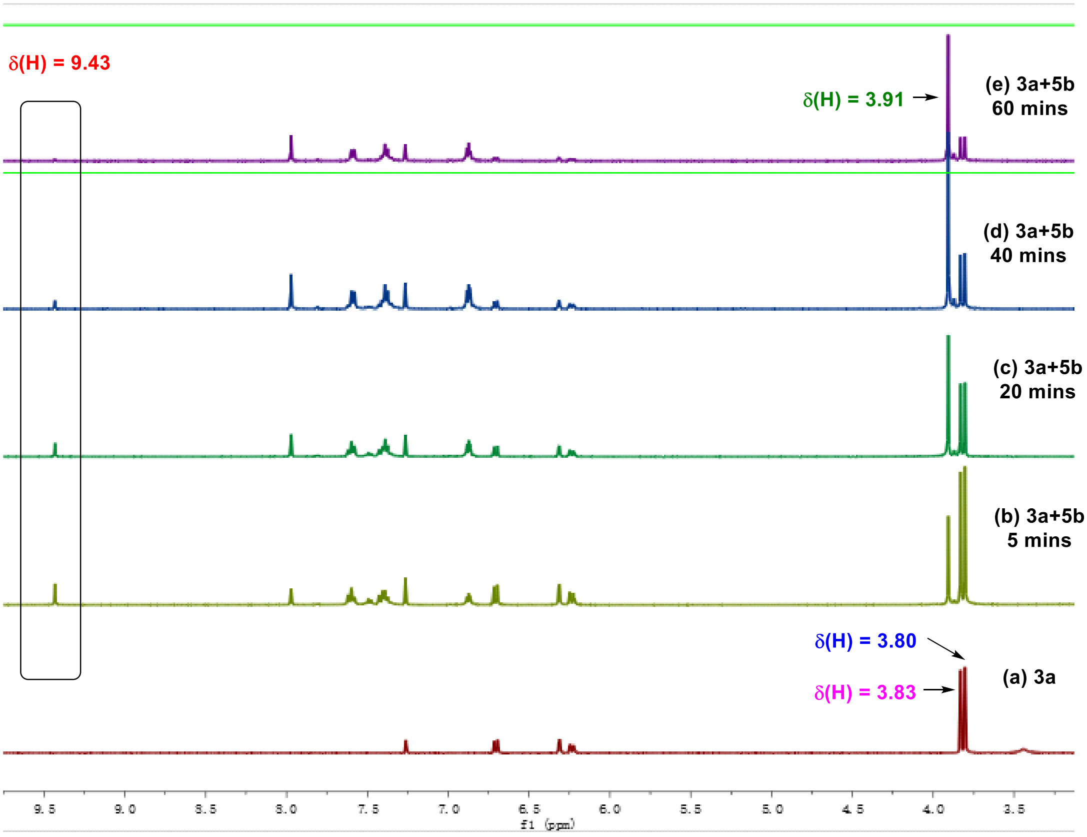

b. Presence of 6b (CPA)

To a flame-dried NMR tube charged with 0.1 mmol **3a**, 0.1 mmol **5b,** 0.01 mmol **6b** in 1.0 mL of CD_2Cl_2. The mixture was shocked by ultrasonic cleaning machine for several

times. The reaction progress was monitoring at 5 mins, 15 mins by ^{1}H NMR respectively.

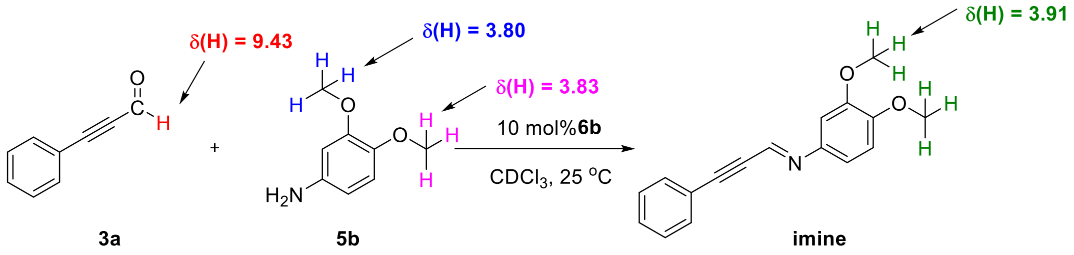

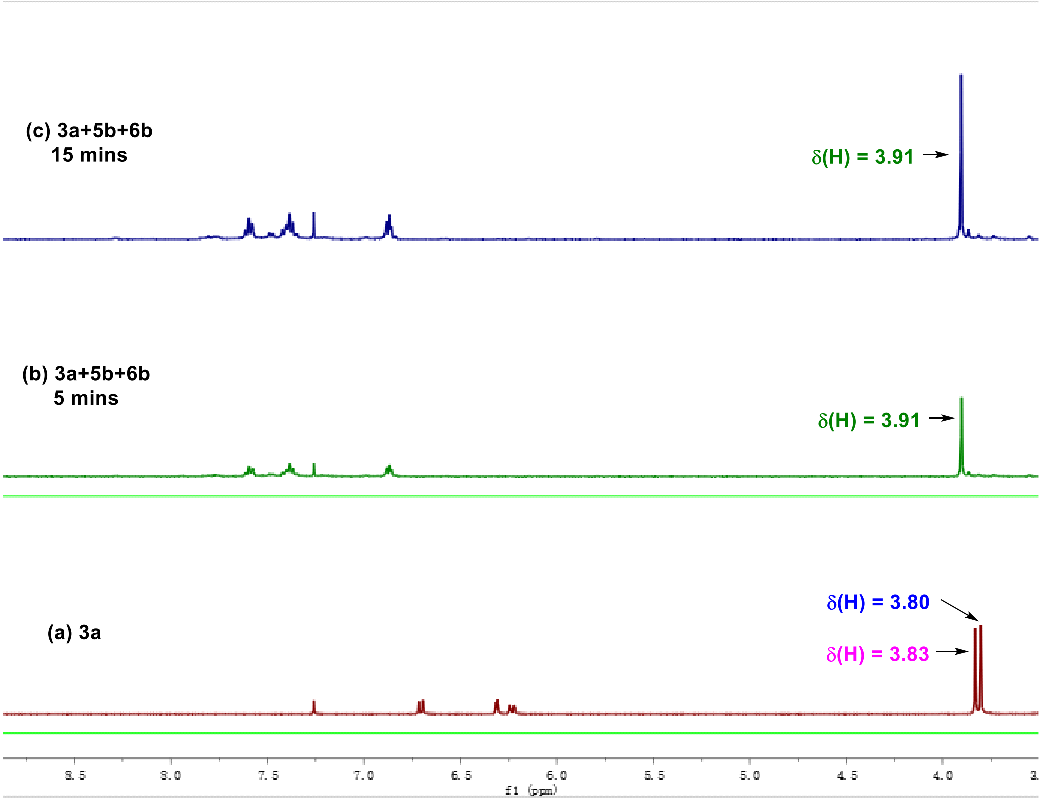

(2) The reaction of [Pd], CPA, imine

a. Absence of imines

To a flame-dried NMR tube charged with 0.01 mmol $[PdCl(allyl)]_2$, 0.01 mmol *(R)*-**6b** in 1.0 mL of CD_2Cl_2. The mixture was shocked by ultrasonic cleaning machine for several times. The reaction progress was monitoring at 1.0 hr, 4.0 hrs and overnight by ^{1}H NMR and ^{31}P NMR respectively.

Result:

^{1}H NMR

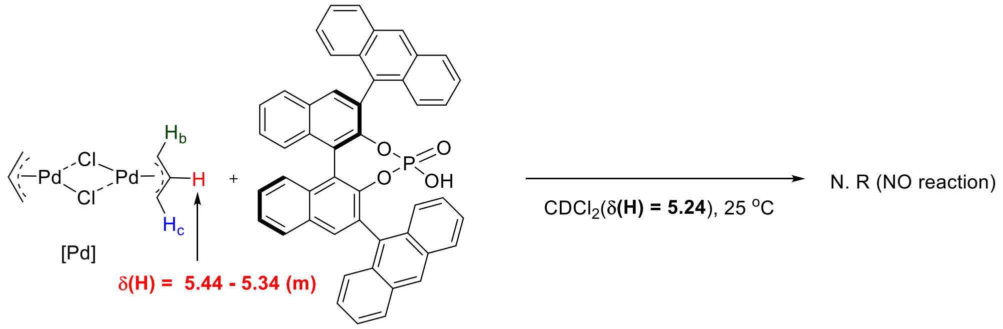

(*R*)-6b

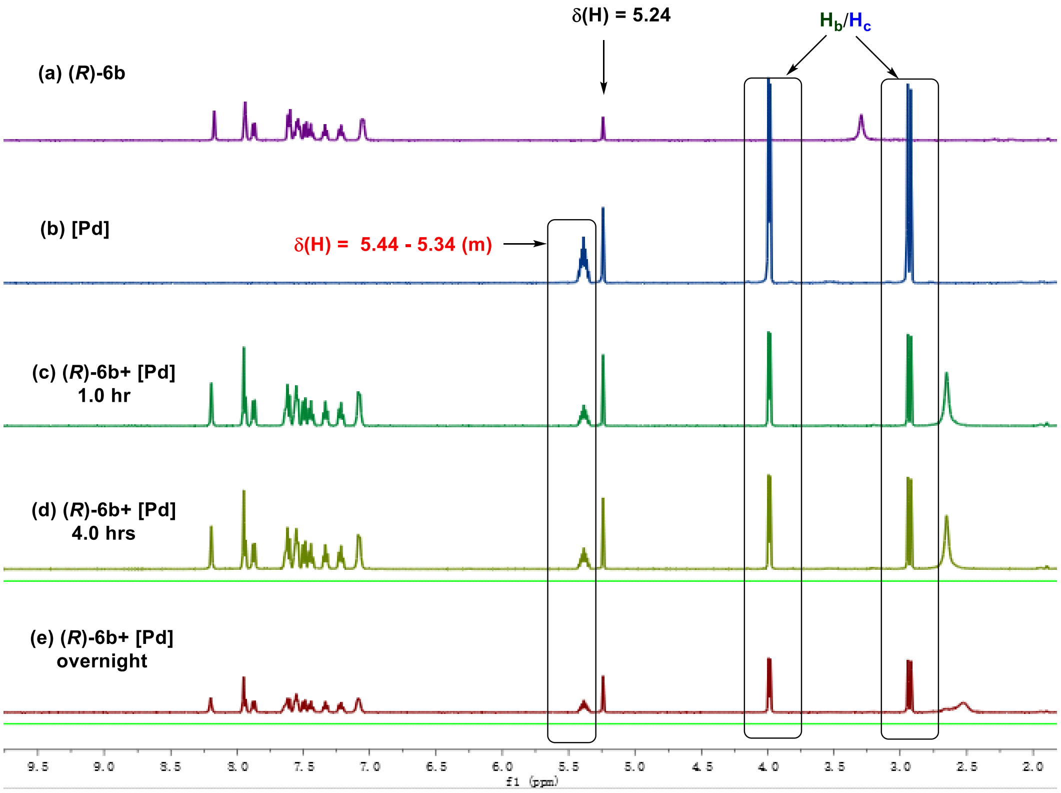

^{31}P NMR

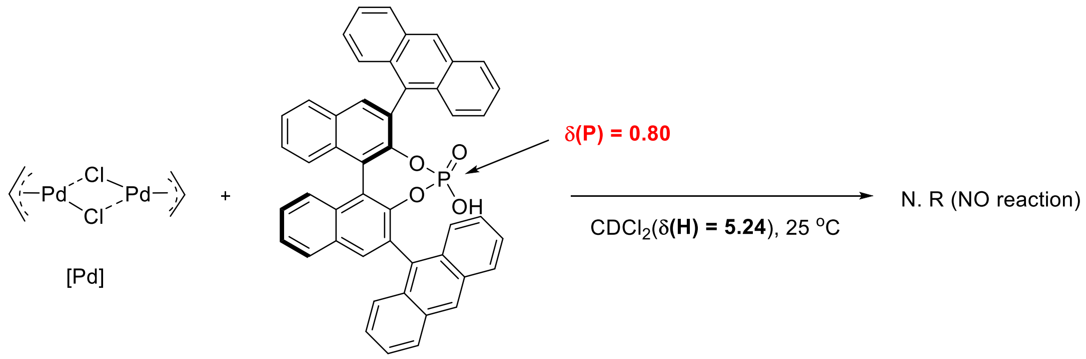

(R)-6b

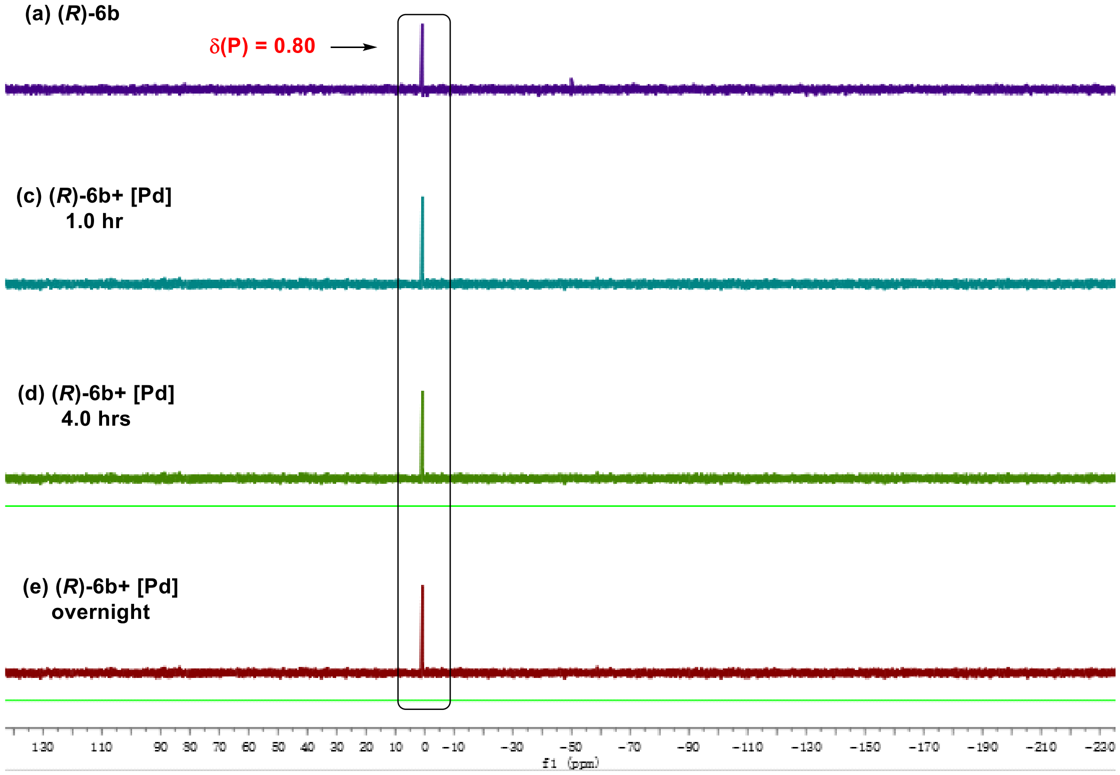

Results of both ^{1}H NMR and ^{31}P NMR illustrated that no chiral palladium(II) phosphate complex existed.

b. Presence of imines

To a flame-dried NMR tube charged with 0.01 mmol $[PdCl(allyl)]_2$, 0.01 mmol *(R)*-**6b**, 0.03 mmol **4a**, 0.03 mmol **5b** in 1.0 mL of CD_2Cl_2. The mixture was shocked by ultrasonic cleaning machine for several times. The reaction progress was monitoring at

5 mins, 30 mins, 60 mins by ^{1}H NMR and ^{31}P NMR respectively.

Result:

^{1}H NMR

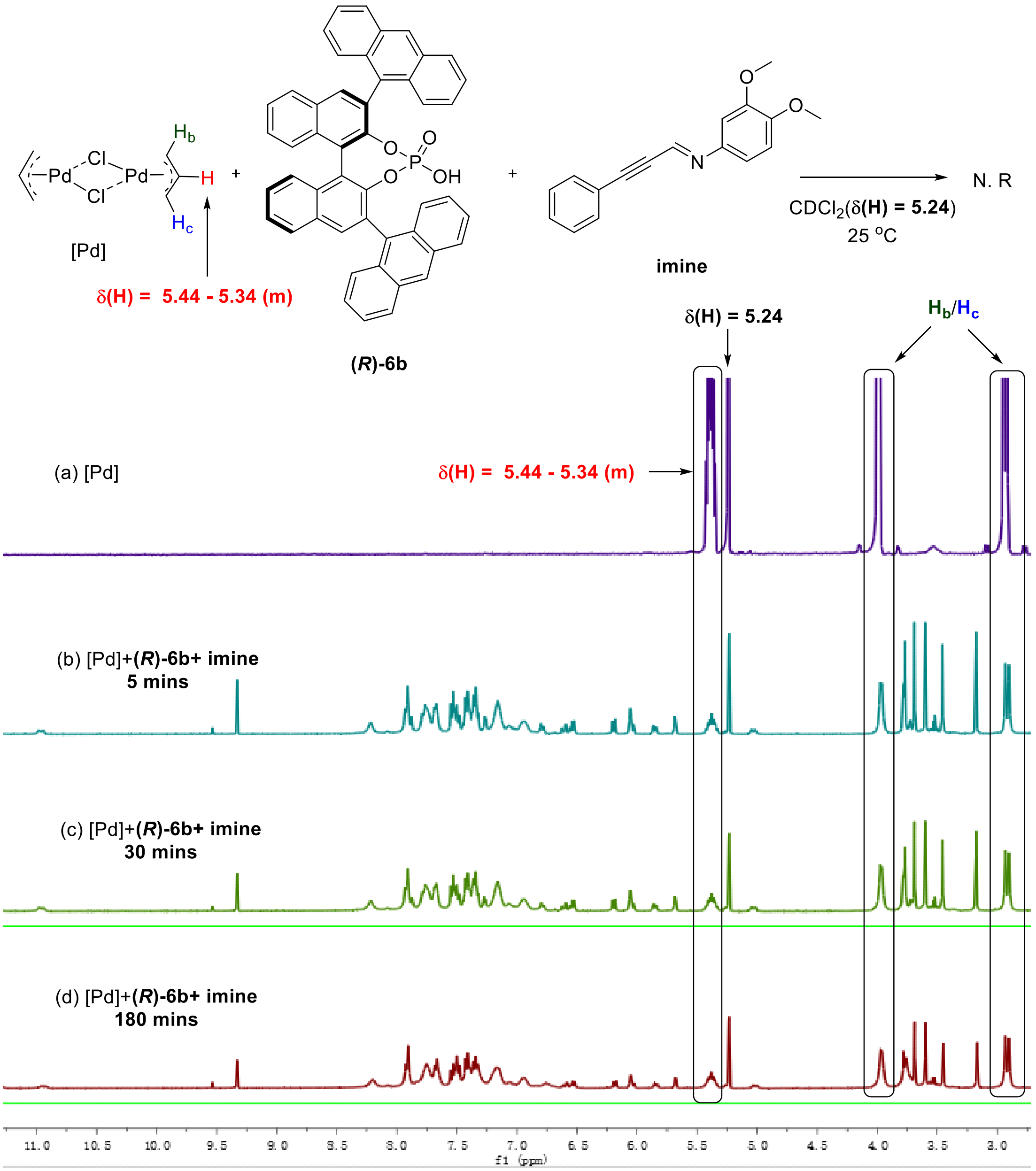

^{31}P NMR

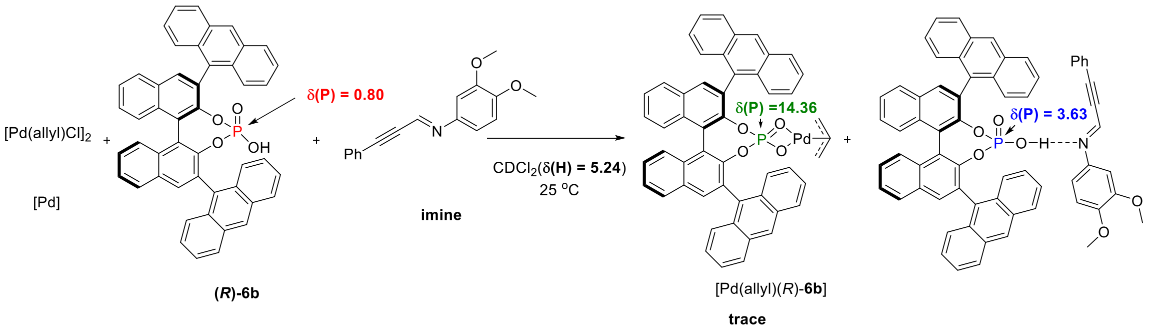

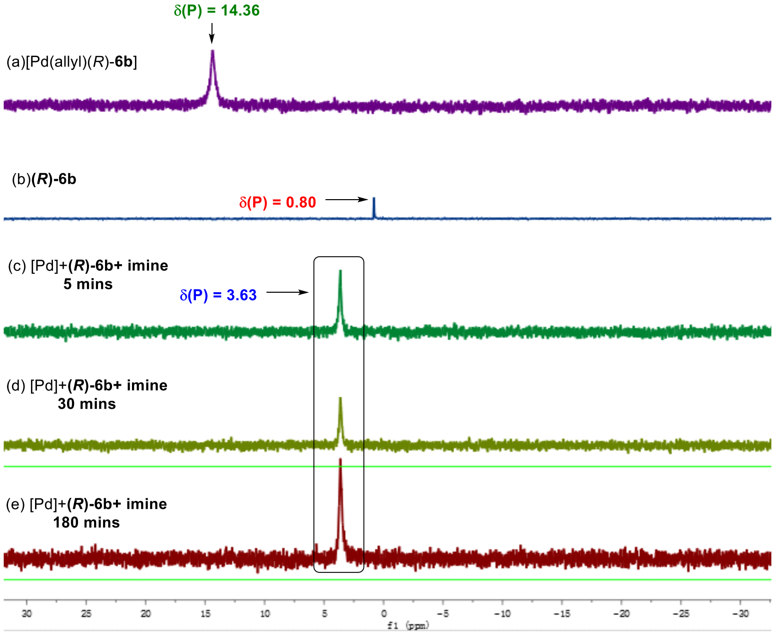

Results of both ^{1}H NMR and ^{31}P NMR illustrated that no chiral palladium(II) phosphate complex existed in the both absence and presence of imines.

7.6 High-resolution Mass Spectrometry (HRMS) Analysis

In order to deeply investigate the reaction mechanism, the follow reaction was analyzed by High-resolution mass spectrometry. The result illustrated that ion pair existed.

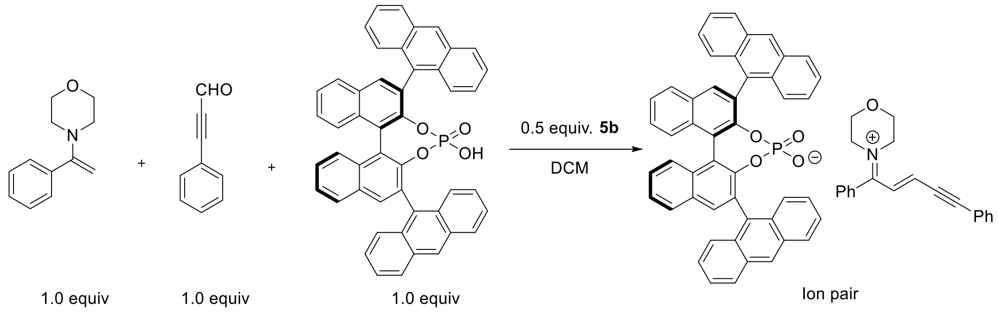

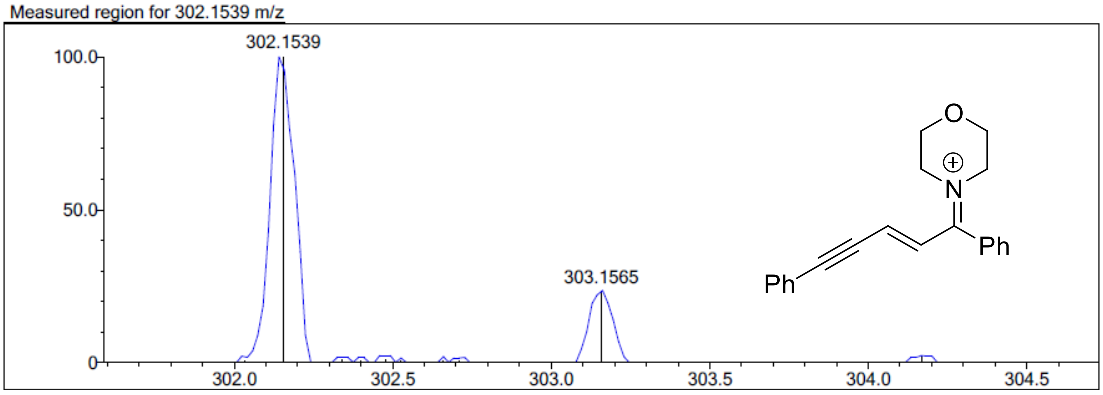

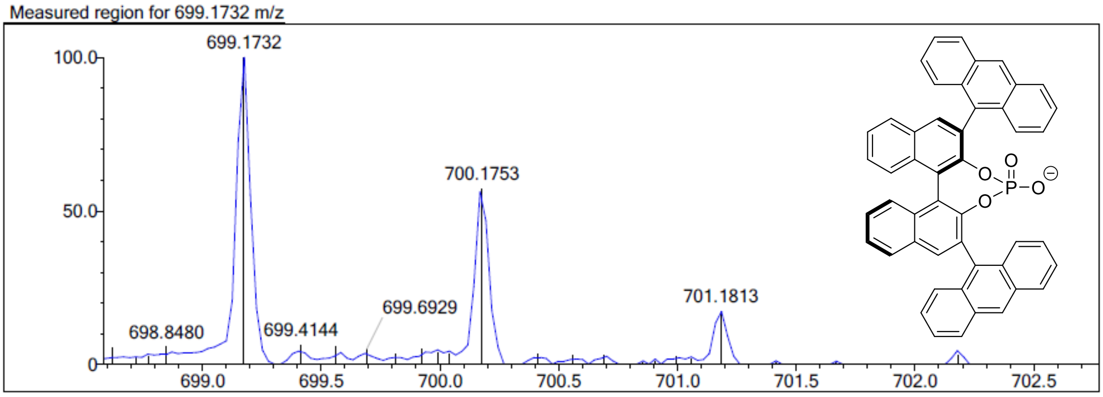

7.7 Control Experiments for DFT

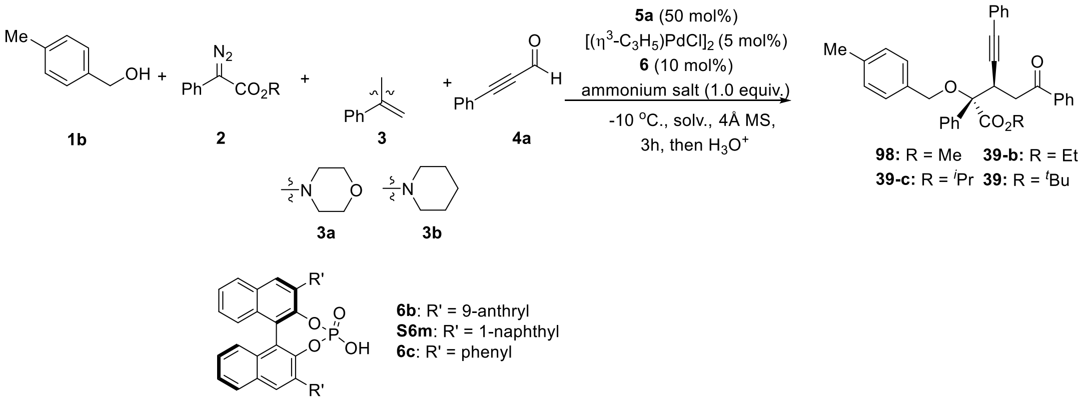

entry	R	**6**	Solvent	Ammonium salt (1.0 equiv.)	**3**	Product	Yield/%[b]	d.r.[c]	ee/%[d]
1	Me	**6b**	DCM	-	**3a**	**98**	53	83:17	21
2	Et	**6b**	DCM	-	**3a**	**39-b**	50	85:15	33
3	^{i}Pr	**6b**	DCM	-	**3a**	**39-c**	54	89:11	56

4	^{t}Bu	**6b**	DCM	-	**3a**	**39**	65	94:6	92
5	^{t}Bu	**S6m**	DCM	-	**3a**	**39**	43	89:11	67
6	^{t}Bu	**6c**	DCM	-	**3a**	**39**	47	84:16	6
7	^{t}Bu	**6b**	DCE	-	**3a**	**39**	42	90:10	92
8	^{t}Bu	**6b**	$CHCl_3$	-	**3a**	**39**	35	90:10	92
9	^{t}Bu	**6b**	toluene	-	**3a**	**39**	53	90:10	94
10	^{t}Bu	**6b**	EA	-	**3a**	**39**	30	92:8	97
11	^{t}Bu	**6b**	MeOH	-	**3a**	**39**	< 5		
12	^{t}Bu	**6b**	DMF	-	**3a**	**39**	< 5		
13	^{t}Bu	**6b**	THF	-	**3a**	**39**	8	92:8	95
14	^{t}Bu	**6b**	DCM/THF (1:9)	-	**3a**	**39**	22	91:9	96
15	^{t}Bu	**6b**	DCM/THF (1:4)	-	**3a**	**39**	25	95:5	97
16	^{t}Bu	**6b**	DCM/MeOH (1:9)	-	**3a**	**39**	6	99:1	94
17	^{t}Bu	**6b**	DCM	$(^nBu)_4N^+Br^-$	**3a**	**39**	27	80:20	56
18	^{t}Bu	**6b**	DCM	-	**3b**	**39**	11	80:20	46

[a] Standard condition: **1a**/**2**/**3a**/**4a**/**5**/$[PdCl(allyl)]_2$/**6** = 0.18/0.18/0.18/0.15/0.075/0.0075/0.015 mmol, **2** and **3a** in 1.0 mL dry solvent were added into a solution of **1a**, **4a**, $[PdCl(allyl)]_2$, PA**6**, aniline **5b** and 100 mg 4 Å MS in 1.5 mL dry solvent via a syringe pump under a dinitrogen atmosphere for 180 mins, and the resulting mixture was stirred for another 1.0 hr. [b] Isolated yield. [c] Determined by ^{1}H NMR spectroscopy analyses or HPLC. [d] Determined by HPLC analyses using a chiral stationary phase.

7.8 Control Experiment of Investigation Pathway of 4x.[27]

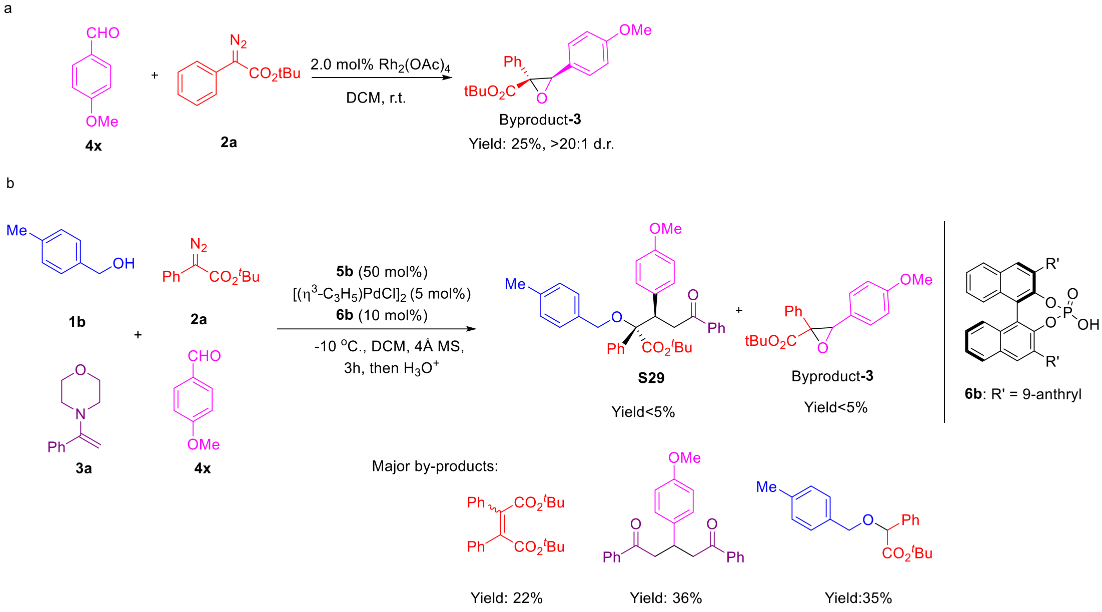

To a flame-dried 10-mL Schlenk flask charged with a magnetic stirring bar, 2.0 mol% $Rh_2(OAc)_4$, **4x** (0.20 mmol) and 100 mg 4 Å MS in 2.0 mL DCM were sequentially added at room temperature. Diazoacetate **2a** (0.24 mmol) dissolved in DCM (1.0 mL) were added by syringe pump over 60 mins. The mixture was stirred for 5.0 hrs. After the completion of the reaction, the reaction mixture was filtrated and the filtrate was evaporated in vacuo to give the crude product. And then the crude product was purified by flash chromatography on silica gel (eluent: PE/EA = 20:1 ~ 10:1, v/v) to give the by-product-**3** (25% yield, > 20:1 d.r.)

To a flame-dried 10-mL Schlenk flask charged with a magnetic stirring bar, 3,4-dimethoxyaniline (0.10 mmol), 5.0 mol% $[PdCl(allyl)]_2$, 10.0 mol% *(R)*-**6b**, **1b** (0.12 mmol), aldehyde **4x** (0.10 mmol) and 100 mg 4 Å MS in 1.0 mL DCM were sequentially added at -10 °C. Enamine **3a** (0.12 mmol), and **2a** (0.12 mmol) dissolved in DCM (1.0 mL) were added by syringe pump over 180 mins. The mixture was stirred for 1.0 hr at -10 °C. After the completion of the reaction, the target **S29** could not be detected by LC-MS and ^1H NMR.

7.9 Control Experiments

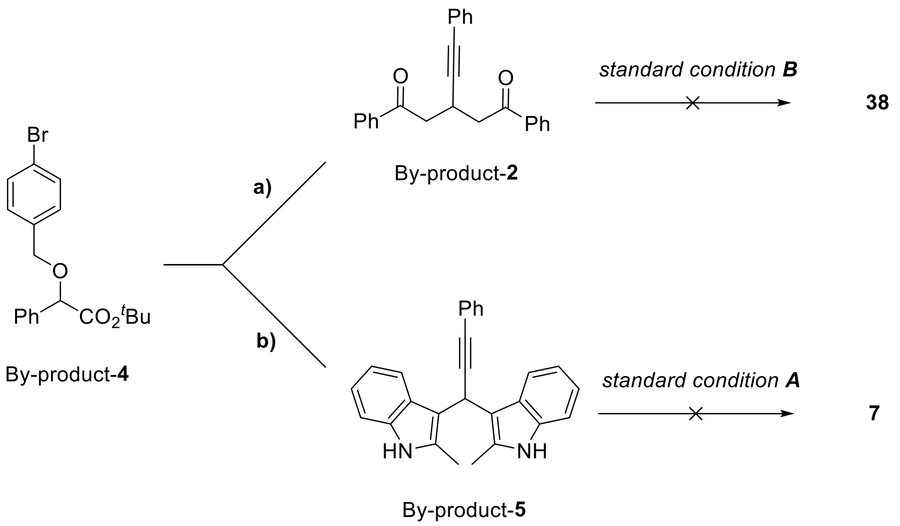

To a flame-dried 10-mL Schlenk flask charged with a magnetic stirring bar, By-product-**4** (0.1 mmol), and By-product-**2** (0.1 mmol) or By-product-5 (0.1 mmol) were dissolved in 1.0 mL solvent. The mixture was conducted under the condition A or B. After the completion of the reaction, neither the target **7** or **38** could not be detected by LC-MS.

7.10 Control Experiments

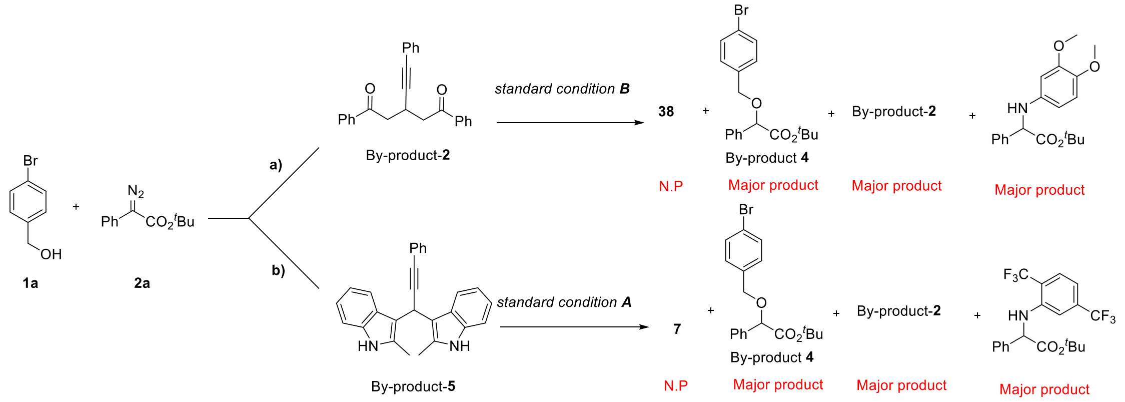

NOTE: N.P is short for no product.

To a flame-dried 10-mL Schlenk flask charged with a magnetic stirring bar, By-product-**1a** (0.12 mmol), and By-product-**2** (0.1 mmol) or By-product-5 (0.1 mmol) were dissolved in 1.0 mL solvent. Diazoacetate **2a** (0.12 mmol) dissolved in solvent (1.0 mL)

were added by syringe pump over 180 mins. The mixture was conducted under the condition A or B. After the completion of the reaction, neither the target **7**/**38** could not be detected by LC-MS.

7.11 Control Experiment

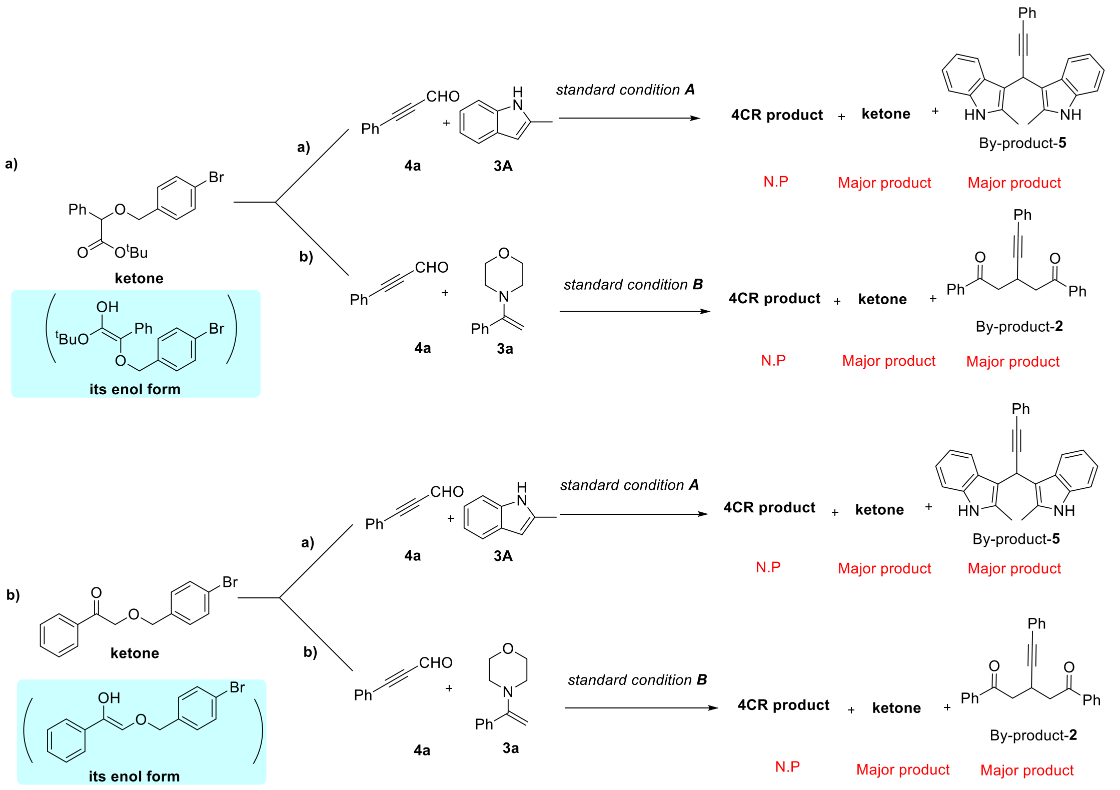

NOTE: N.P. is short for no product.

To a flame-dried 10-mL Schlenk flask charged with a magnetic stirring bar, 3,4-dimethoxyaniline (0.10 mmol), 5.0 mol% $[PdCl(allyl)]_2$, 10.0 mol% *(R)*-**6b**, ketone (0.24 mmol), aldehyde **4a** (0.20 mmol) and 100 mg 4 Å MS in 2.0 mL DCM were sequentially added at -10 °C. Enamine **3a** (0.24 mmol) dissolved in DCM (1.0 mL) were added by syringe pump over 60 mins. The mixture was stirred for 1.0 hr at -10 °C. After the completion of the reaction, neither the 4CR (four-component reaction) product could not be detected by LC-MS.

7.12 Control Experiment

a)

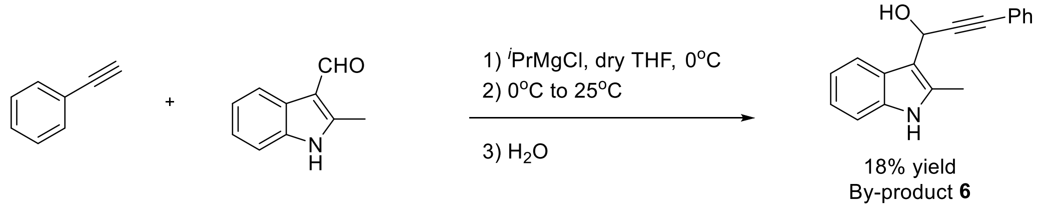

b)

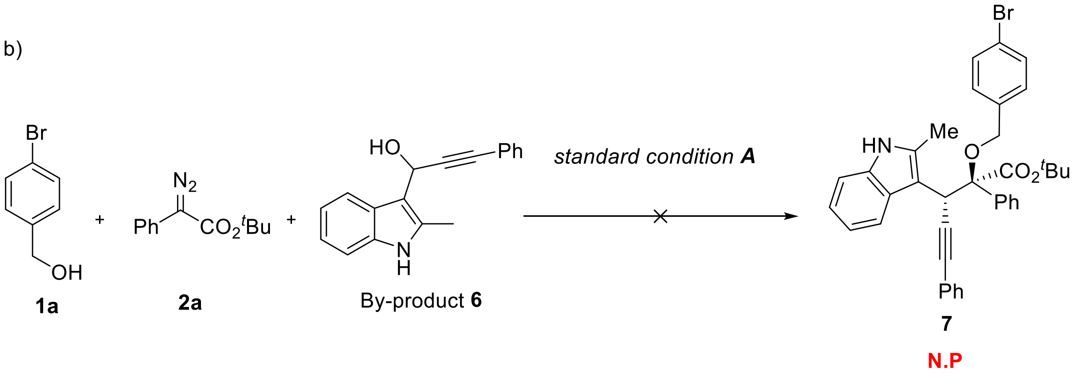

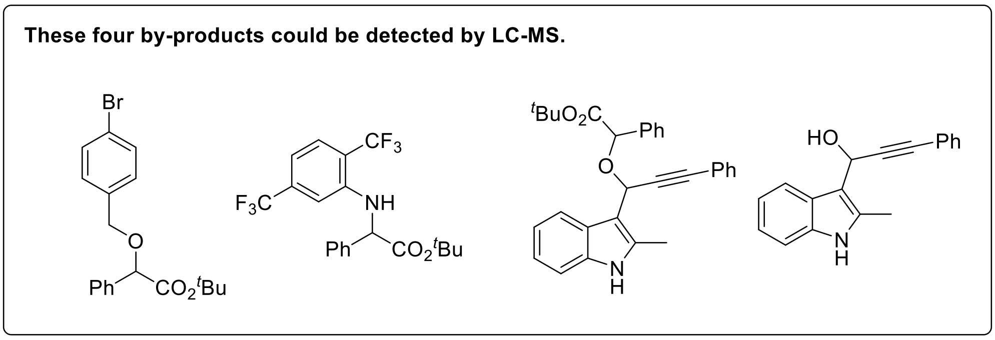

NOTE: N.P is short for no product.

^{i}PrMgCl (2 M in hexane, 8.0 mL, 16 mmol) was added dropwise to a solution of phenyl acetylene (1.7 ml, 15 mmol) in dry THF (10 mL) at 0 ℃. The reaction mixture was stirred for 30 min at 0 ℃, then at room temperature for 1 h. A solution of 2-methyl-1*H* indole-3-carboxaldehyde (800 mg, 5.0 mmol) in dry THF (20 mL) was added slowly to the mentioned above Grignard solution at 0 ℃. The resulting mixture was stirred at 0 ℃ for 20 min, then at room temperature for 20 h. H_2O was used to quench the reaction. The reaction mixture was extracted with Et_2O (3 x 30 mL) and the combined organic layer were dried over Na_2SO_4. The crude product was purified by flash chromatography (SiO_2 (NEt_3), PE: EtOAc = 5:1) to yield by-product-**6** in 22% yield (290 mg, 1.08 mmol).

To a flame-dried 10-mL Schlenk flask charged with a magnetic stirring bar, **5a** (0.10 mmol), 5.0 mol% [Pd], 10.0 mol% *(R)*-**6a**, By-product-**6** (0.24 mmol), **1a** (0.24 mmol) and 100 mg 4 Å MS in 2.0 mL DCM were sequentially added at -10 °C. **2a** (0.24 mmol) dissolved in DCM (1.0 mL) were added by syringe pump over 60 mins. The mixture was stirred for 1.0 hr at -10 °C. After the completion of the reaction, the target **7** could not be detected by LC-MS.

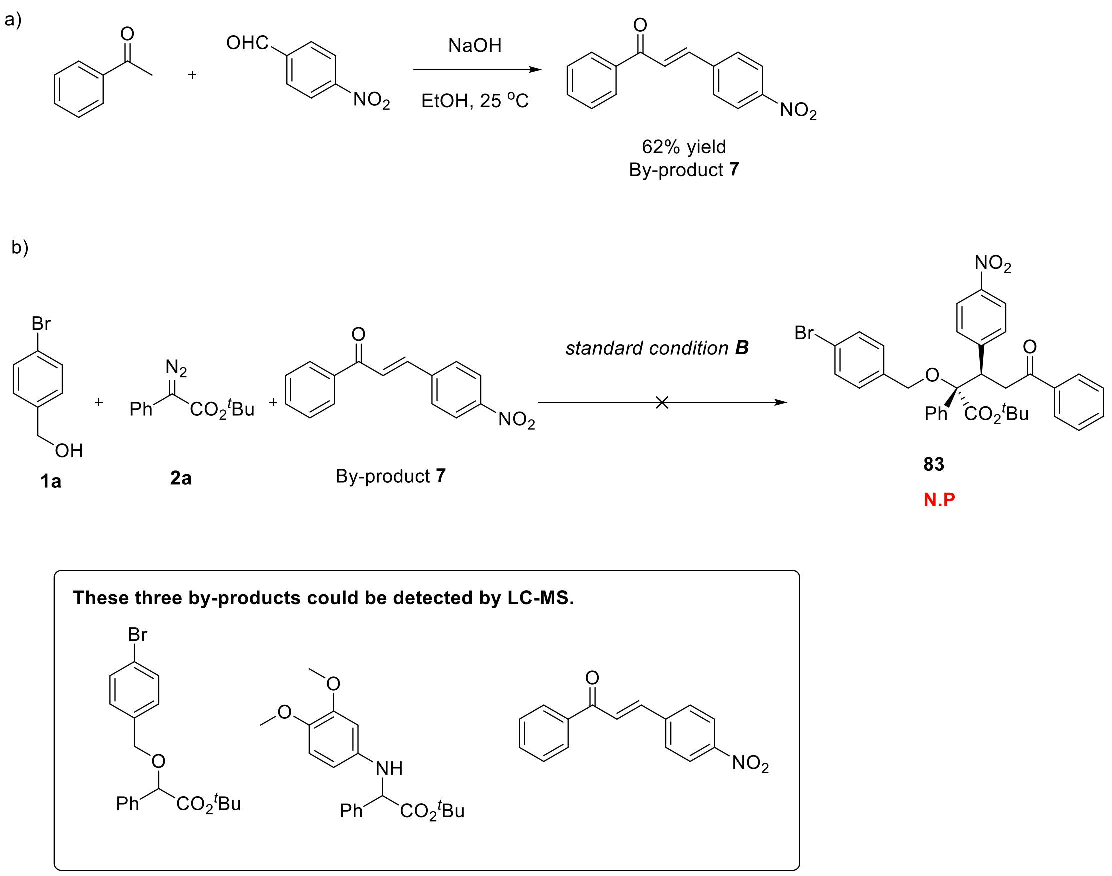

NOTE: N.P is short for no product.

Acetophenone (5 mmol) and aromatic aldehyde (5 mmol) were dissolved in 3 mL of ethanol and 10% NaOH was added, the mixture was stirred for 5 min. After completion of the reaction, the mixture was poured into ice; the precipitate was filtered and recrystallized with ethanol, to get pure by-product-**7** (62% yield).

To a flame-dried 10-mL Schlenk flask charged with a magnetic stirring bar, 3,4-dimethoxyaniline (0.10 mmol), 5.0 mol% $[PdCl(allyl)]_2$, 10.0 mol% *(R)*-**6b**, by-product-**7** (0.24 mmol), **1a** (0.24 mmol) and 100 mg 4 Å MS in 2.0 mL DCM were sequentially added at -10 °C. **2a** (0.24 mmol) dissolved in DCM (1.0 mL) were added by syringe pump over 60 mins. The mixture was stirred for 1.0 hr at -10 °C. After the completion of the reaction, the target **83** could not be detected by LC-MS.

8. DFT Calculation

DFT calculations on the mechanism and stereoselectivity of this four-component reaction have been conducted with Gaussian 09.[30] Geometry optimizations were carried out at the B3LYP level of theory[31,32], and LANL2DZ pseudo-potential[33,34] was used for Pd and 6-31G(d) basis set (5D keyword used) for all the other atoms in dichloromethane using the CPCM solvation model.[35-37] Vibrational frequencies were computed at the same level to verify that optimized structures are local minima or transition states and to evaluate zero-point vibrational energies and thermal corrections at 298 K, 1 atm. For transition states, intrinsic reaction coordinate (IRC)[38] was calculated to confirm that they indeed connected between correct minima. Single-point energy calculations were performed at the M06-2X level of theory[39] with SDD[40] for Pd and the 6-311+G(d,p) basis set (5D keyword used) for other atoms using the CPCM solvation model. The three-dimensional structures were visualized utilizing CYLview.[41]

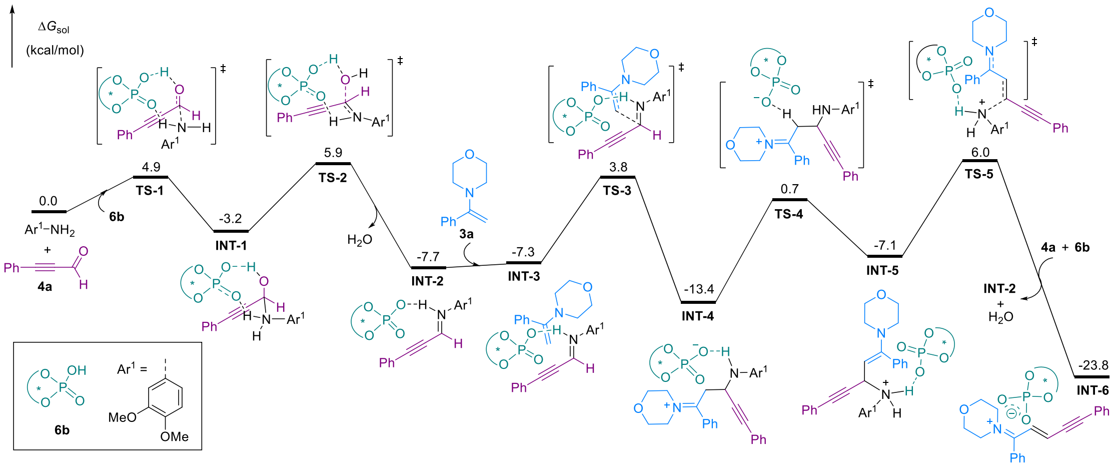

Supplementary Figure 8. Free energy profiles for the CPA catalyzed iminium formation. The Gibbs free energies were computed with CPCM(DCM)-M06-2X/6-311+G(d,p)//CPCM(DCM)-B3LYP/6-31G(d).

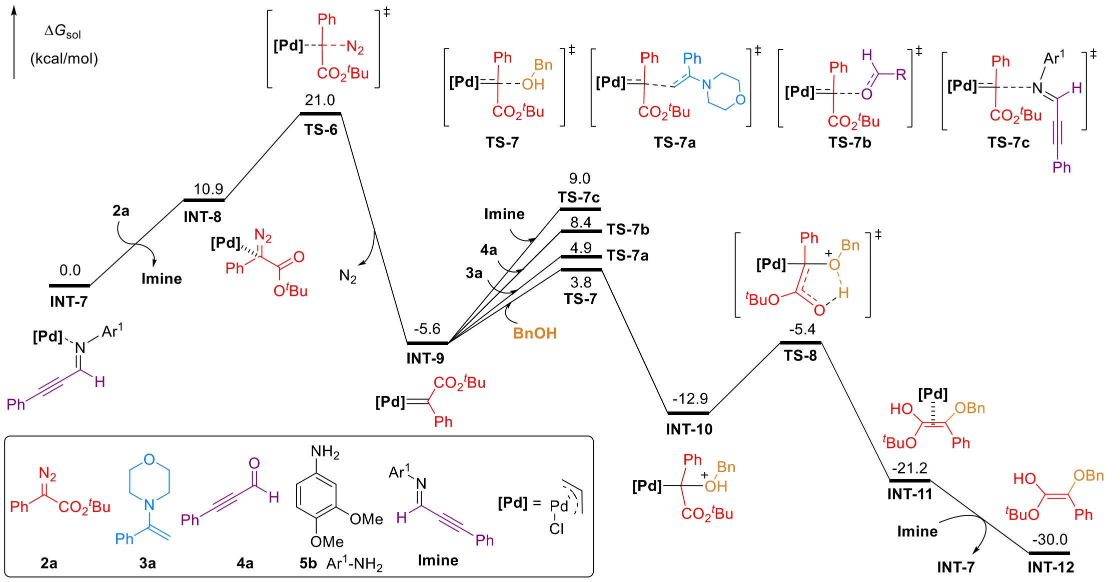

Supplementary Figure 9. Free energy profiles for the palladium catalyzed enol formation. The Gibbs free energies were computed with CPCM(DCM)-M06-2X/6-311+G(d,p)[SDD for Pd]//CPCM(DCM)-B3LYP/6-31G(d)[LANL2DZ for Pd].

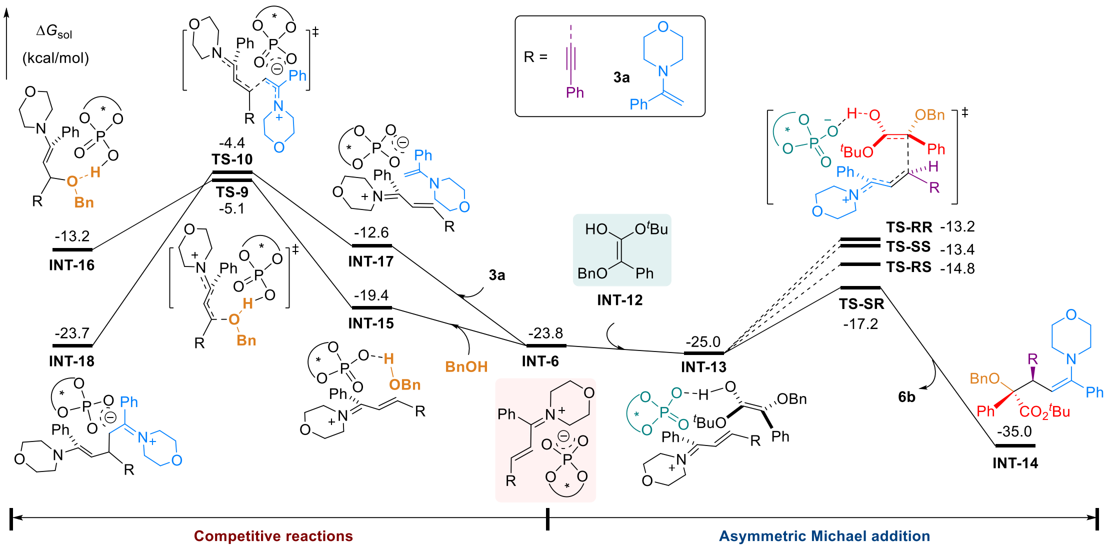

Supplementary Figure 10. Free energy profiles for the asymmetric Michael addition and competitive side reactions. The Gibbs free energies were computed with CPCM(DCM)-M06-2X/6-311+G(d,p)//CPCM(DCM)-B3LYP/6-31G(d).

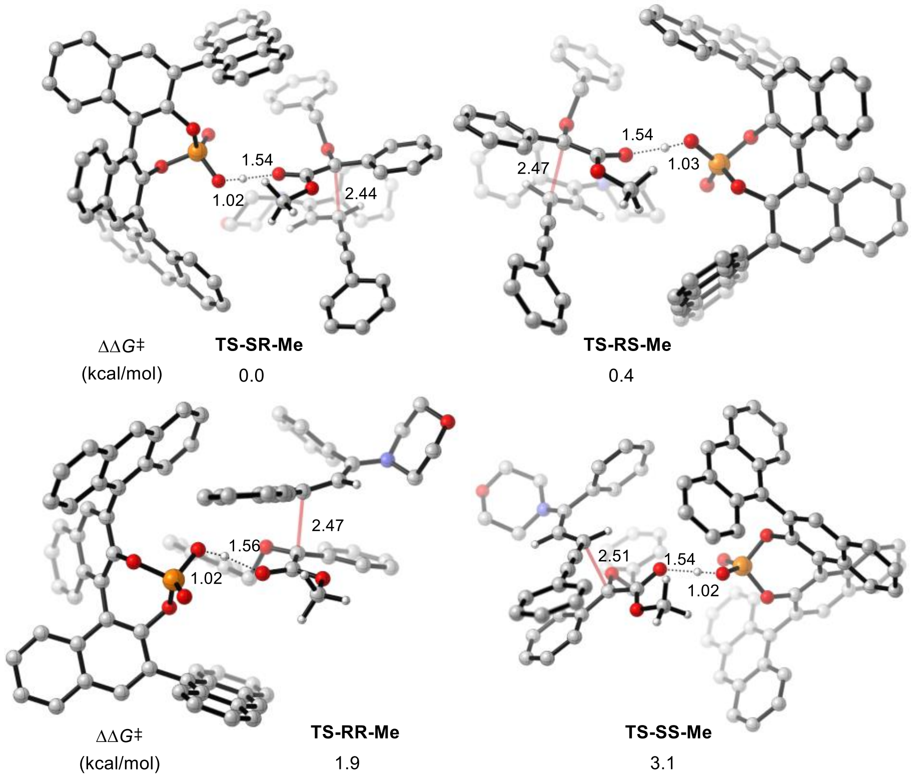

Supplementary Figure 11. Optimized transition state geometries of symmetric Michael addition using CPA **6b** and diazoester **2b**. The Gibbs free energies were computed with CPCM(DCM)-M06-2X/6-311+G(d,p)//CPCM(DCM)-B3LYP/6-31G(d).

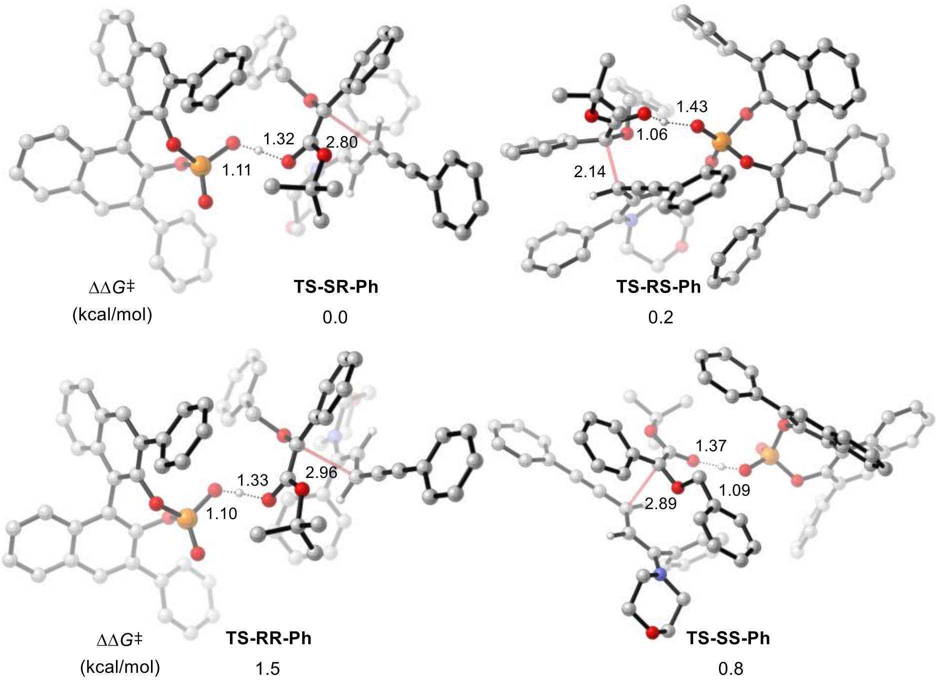

Supplementary Figure 12. Optimized transition state geometries of symmetric Michael addition using CPA **6c** and diazoester **2a**. The Gibbs free energies were computed with CPCM(DCM)-M06-2X/6-311+G(d,p)//CPCM(DCM)-B3LYP/6-31G(d).

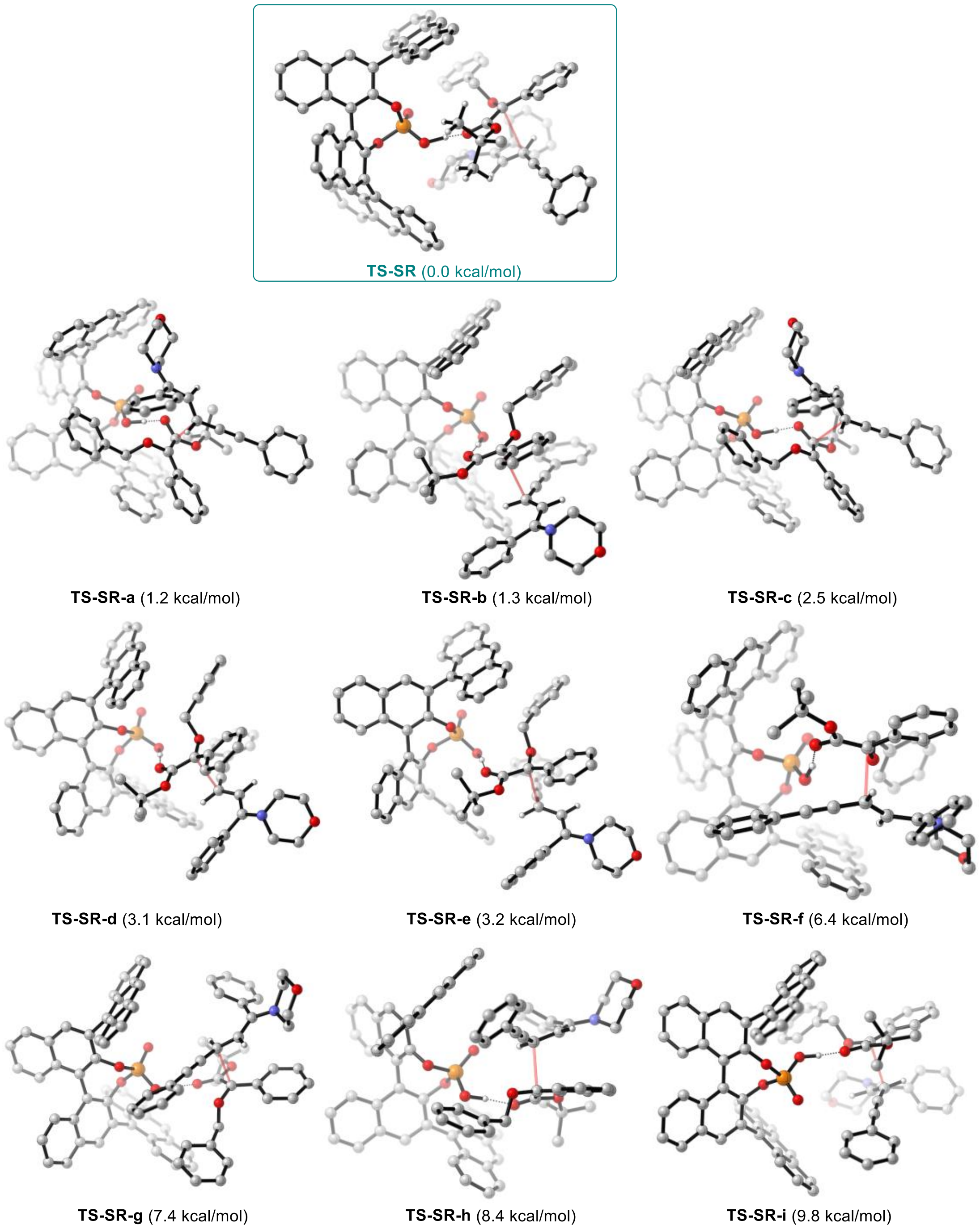

Supplementary Figure 13. The different conformers of the stereocontrolling transition states **TS-SR** for the asymmetric four-component reaction using CPA **6b** and diazoester **2a**. The Gibbs free energies were computed with CPCM(DCM)-M06-2X/6-311+G(d,p)//CPCM(DCM)-B3LYP/6-31G(d).

III. Supplementary Data

9. Analytical Data for the Products

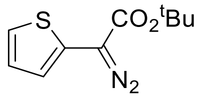

2h

^{1}H NMR (400 MHz, $CDCl_3$) δ 7.30 (dd, *J* = 5.2, 1.2 Hz, 1H), 7.02 (dd, *J* = 5.2, 3.7 Hz, 1H), 6.76 (dd, *J* = 3.7, 1.2 Hz, 1H), 1.55 (s, 1H). ^{13}C NMR (101 MHz, $CDCl_3$) δ 164.4, 126.8, 125.4, 120.3, 82.9, 55.8, 34.9, 28.4, 25.5.

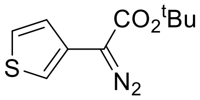

2i

^{1}H NMR (400 MHz, $CDCl_3$) δ 7.39 – 7.34 (m, 2H), 7.01 (dd, *J* = 5.0, 1.4 Hz, 1H), 1.55 (s, 9H). ^{13}C NMR (101 MHz, $CDCl_3$) δ 164.8, 126.2, 123.8, 122.5, 117.5, 37.2, 28.4, 28.1.

N,2-Dimethyl-N-phenyl-1*H*-indol-5-amine (3H)

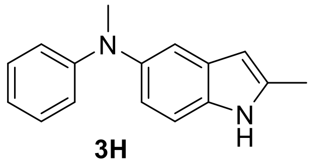

3H

Brown solid, mp: 91.8 – 92.3 °C; 885.5 mg, 75% yield; ^{1}H NMR (400 MHz, $CDCl_3$) δ 7.77 (s, 1H), 7.32 (d, *J* = 1.6 Hz, 1H), 7.23 (d, *J* = 8.6 Hz, 1H), 7.19 – 7.12 (m, 2H), 6.93 (dd, *J* = 8.5, 2.0 Hz, 1H), 6.78 – 6.67 (m, 3H), 6.17 (s, 1H), 3.30 (s, 3H), 2.41 (s, 3H). ^{13}C NMR (126 MHz, $CDCl_3$) δ 150.6, 141.8, 136.0, 133.8, 130.0, 128.8, 120.4, 117.3, 117.2, 114.5, 111.1, 100.6, 40.9, 13.8. HRMS(ESI) $[M + H]^+$ calcd for $C_{16}H_{17}N_2{}^+$, 237.1386, found 237.1388.

2-Methyl-5-(*p*-tolylthio)-1*H*-indole (3I)

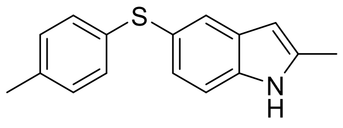

3l

Yellow solid, mp: 116.6 – 117.5 °C; 911.1 mg, 72% yield; ^{1}H NMR (400 MHz, $CDCl_3$) δ 7.89 (s, 1H), 7.66 (s, 1H), 7.22 – 7.19 (m, 2H), 7.09 (d, J = 8.2 Hz, 2H), 7.01 (d, J = 8.1 Hz, 2H), 6.18 (s, 1H), 2.42 (s, 3H), 2.27 (s, 3H). ^{13}C NMR (126 MHz, $CDCl_3$) δ 136.1, 136.0, 135.8, 135.3, 130.0, 129.6, 128.4, 126.8, 125.6, 123.6, 111.2, 100.5, 21.0, 13.7. HRMS(ESI) $[M + H]^+$ calcd for $C_{16}H_{16}NS^+$, 254.0998, found 254.1001.

***tert*-Butyl (2*R*,3*R*)-2-((4-bromobenzyl)oxy)-3-(2-methyl-1*H*-indol-3-yl)-2,5-diphenylpent-4-ynoate (7)**

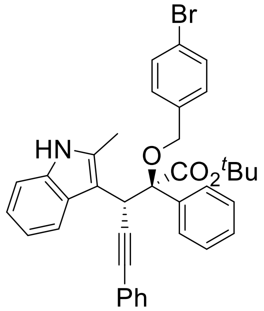

Yellow oil; 107.7 mg, 87% yield, >20:1 d.r., 94% *ee*, $[\alpha]_D^{20}$ = 3.76 (c = 0.033, MeOH); ^{1}H NMR (400 MHz, $CDCl_3$) δ 7.89 (d, J = 5.6 Hz, 1H), 7.62 (s, 1H), 7.46 – 7.42 (m, 2H), 7.34 (d, J = 8.3 Hz, 2H), 7.28 (s, 4H), 7.22 – 7.14 (m, 3H), 7.08 (d, J = 4.3 Hz, 4H), 7.06 – 7.01 (m, 1H), 6.91 (t, J = 7.5 Hz, 1H), 4.81 (d, J = 13.0 Hz, 1H), 4.74 (s, 1H), 4.29 (d, J = 13.0 Hz, 1H), 1.57 (s, 3H), 1.53 (s, 9H). ^{13}C NMR (126 MHz, $CDCl_3$) δ 170.9, 139.3, 136.1, 135.0, 134.3, 131.7, 131.0, 128.9, 128.5, 128.3, 128.0, 127.9, 127.7, 127.5, 124.1, 120.8, 120.3, 119.1, 109.8, 105.9, 89.7, 89.6, 83.2, 83.0, 67.7, 41.6, 28.2, 11.2. HRMS(ESI) $[M+Na]^+$ calcd for $C_{37}H_{34}BrNO_3Na^+$, 642.1614, found 642.1614. (Chiral IA, λ= 254 nm, *n*-hexane/2-propanol = 98/2, Flow rate = 1.0 mL/min), t_R = 17.637 min, 20.256 min (major).

HPLC chromatogram of racemic 7

Condition: n-hexane/2-propanol = 98:2

Flow rate = 1.0 mL/min

λ = 254 nm

Chiral IA

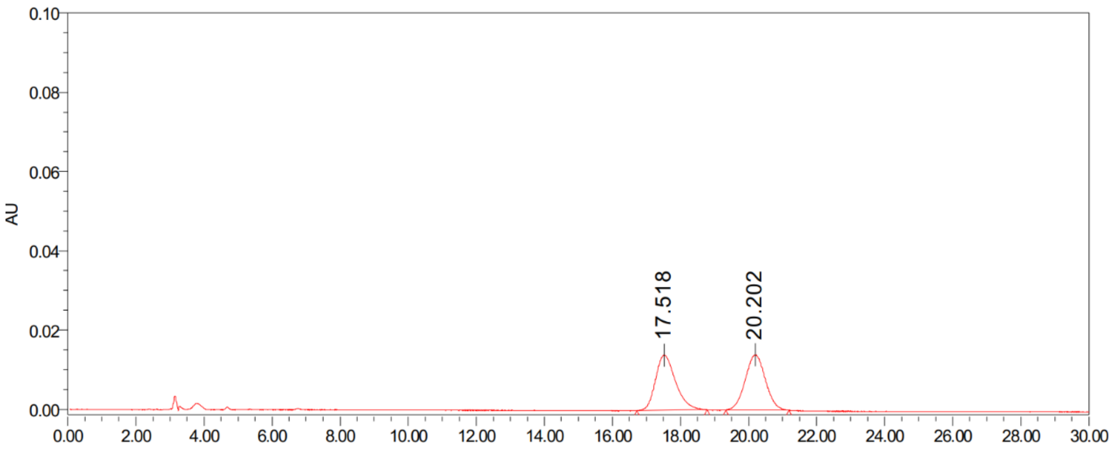

Entry	Retention Time/min	Area	Height	Area(%)
1	17.518	566658	13898	49.97
2	20.202	567229	13895	50.03

Supplementary Figure 14. Chiral HPLC analysis of racemic **7**

HPLC chromatogram of chiral 7

Condition: n-hexane/2-propanol = 98:2

Flow rate = 1.0 mL/min

λ = 254 nm

Chiral IA

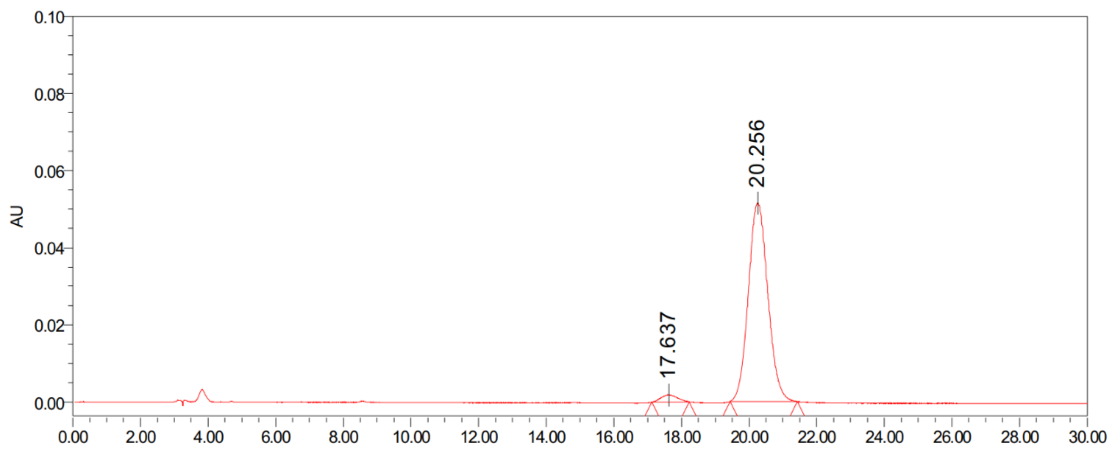

Entry	Retention Time/min	Area	Height	Area(%)
1	17.637	61443	1816	2.92
2	20.256	2040876	51444	97.08

Supplementary Figure 15. Chiral HPLC analysis of chiral **7**

tert-Butyl (2*R*,3*R*)-2-((4-cyanobenzyl)oxy)-3-(2-methyl-1*H*-indol-3-yl)-2,5-diphenylpent-4-ynoate (8)

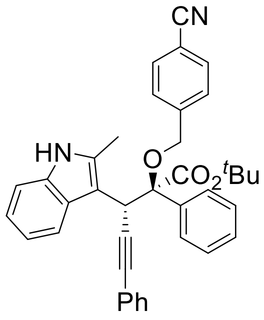

8

Yellow oil; 88.3 mg, 78% yield, >20:1 d.r., 92% *ee*, $[\alpha]_D^{20}$ = -15.48 (c = 0.033, MeOH); ^{1}H NMR (400 MHz, $CDCl_3$) δ 7.95 (s, 1H), 7.72 (s, 1H), 7.47 (ddd, *J* = 9.6, 9.1, 5.2 Hz, 6H), 7.32 – 7.28 (m, 3H), 7.21 (dd, *J* = 12.9, 7.3 Hz, 2H), 7.10 (t, *J* = 7.6 Hz, 2H), 7.08 – 7.02 (m, 3H), 6.92 (t, *J* = 7.4 Hz, 1H), 4.89 (d, *J* = 14.2 Hz, 1H), 4.74 (s, 1H), 4.40 (d, *J* = 14.2 Hz, 1H), 1.53 (s, 12H). ^{13}C NMR (101 MHz, $CDCl_3$) δ 170.8, 146.1, 135.9, 135.1, 134.4, 131.8, 131.6, 128.8, 128.3, 128.2, 127.9, 127.8, 127.6, 127.1, 123.9, 121.7, 120.9, 119.4, 119.1, 110.2, 109.9, 105.7, 89.9, 89.5, 83.3, 83.2, 67.7, 41.5, 28.2, 11.0.

HRMS(ESI) $[M + Na]^+$ calcd for $C_{38}H_{34}N_2O_3Na^+$, 589.2462, found 589.2465. (Chiral IA, λ = 254 nm, *n*-hexane/2-propanol = 95/5, Flow rate = 1.0 mL/min), t_R =17.555 min, 19.020 min (major).

HPLC chromatogram of racemic 8

Condition: n-hexane/2-propanol = 95:5

Flow rate = 1.0 mL/min

λ = 254 nm

Chiral IA

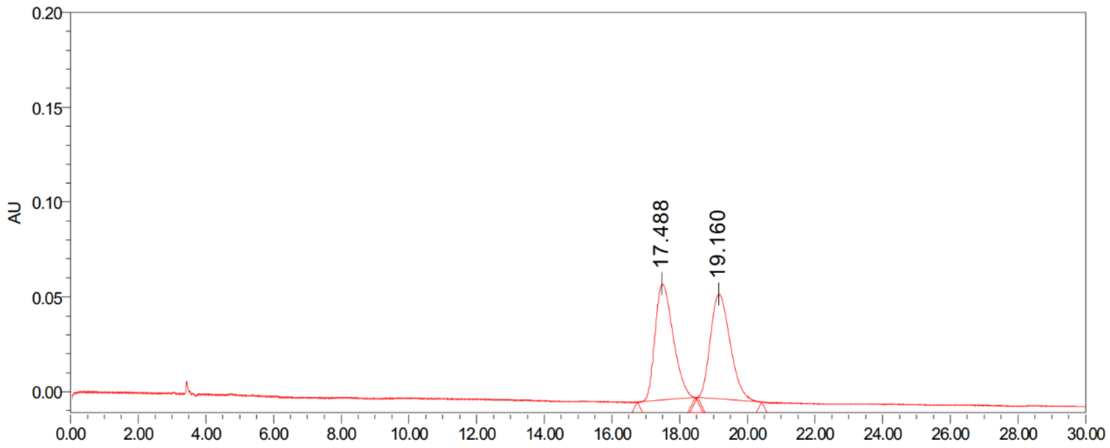

Entry	Retention Time/min	Area	Height	Area(%)
1	17.488	2270400	61235	50.30
2	19.160	2243630	55219	49.70

Supplementary Figure 16. Chiral HPLC analysis of racemic **8**

HPLC chromatogram of chiral 8

Condition: n-hexane/2-propanol = 95:5

Flow rate = 1.0 mL/min

λ = 254 nm

Chiral IA

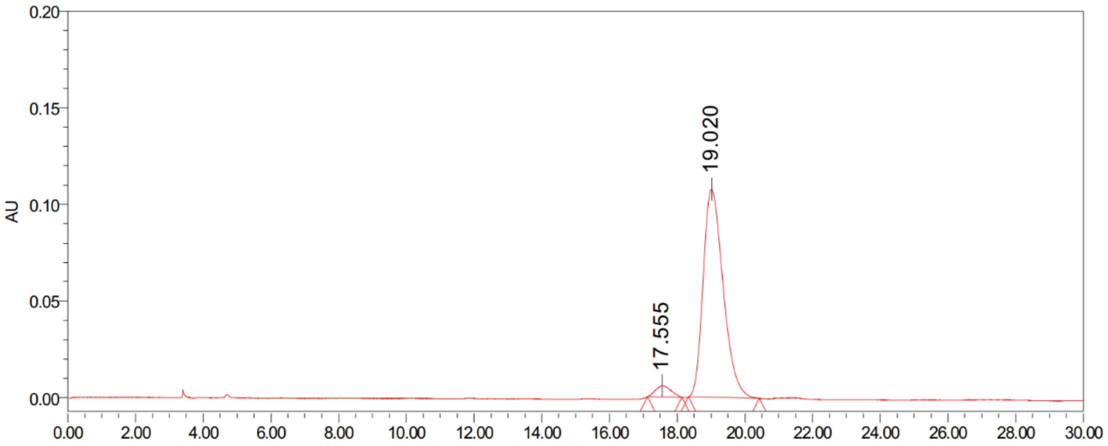

Entry	Retention Time/min	Area	Height	Area(%)
1	17.555	182943	5711	3.95
2	19.020	4444458	107820	96.05

Supplementary Figure 17. Chiral HPLC analysis of chiral **8**

tert-Butyl (2_R_,3_R_)-2-((4-ethynylbenzyl)oxy)-3-(2-methyl-1_H_-indol-3-yl)-2,5-diphenylpent-4-ynoate (9)

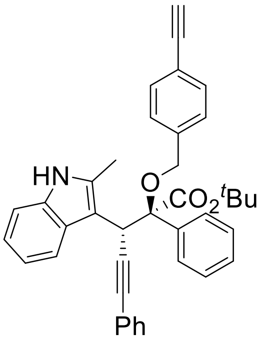

9

Yellow oil; 59.9 mg, 53% yield, >20:1 d.r., 94% *ee*, $[\alpha]_D^{20}$ = -15.37 (c = 0.033, MeOH); ^{1}H NMR (500 MHz, $CDCl_3$) δ 7.90 (s, 1H), 7.64 (s, 1H), 7.45 (d, *J* = 6.7 Hz, 2H), 7.37 (s, 4H), 7.29 (d, *J* = 6.2 Hz, 3H), 7.23 – 7.15 (m, 2H), 7.09 (d, *J* = 3.8 Hz, 4H), 7.03 (t, *J* = 7.5 Hz, 1H), 6.91 (t, *J* = 7.5 Hz, 1H), 4.86 (d, *J* = 13.3 Hz, 1H), 4.75 (s, 1H), 4.36 (d, *J* = 13.3 Hz, 1H), 3.03 (s, 1H), 1.59 (s, 3H), 1.51 (s, 9H). ^{13}C NMR (126 MHz, $CDCl_3$) δ 171.0, 141.3, 136.2, 135.0, 134.3, 131.8, 131.7, 128.9, 128.2, 128.0, 127.9, 127.7, 127.4, 126.6, 124.1, 120.8, 120.1, 119.1, 109.7, 106.0, 89.7, 89.6, 84.1, 83.1,

83.0, 76.6, 68.0, 41.6, 28.2, 11.2. HRMS(ESI) $[M+Na]^+$ calcd for $C_{39}H_{35}NO_3Na^+$, 588.2509, found 588.2512. (Chiral IA, λ = 254 nm, *n*-hexane/2-propanol = 90/10, Flow rate = 1.0 mL/min), t_R = 5.332 min, 5.933 min (major).

HPLC chromatogram of racemic 9

Condition: n-hexane/2-propanol = 90:10

Flow rate = 1.0 mL/min

λ = 254 nm

Chiral IA

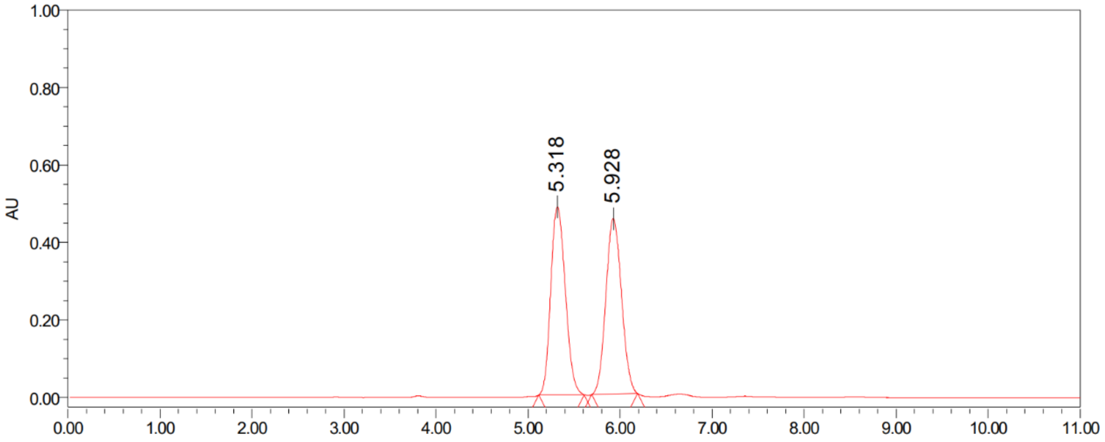

Entry	Retention Time/min	Area	Height	Area(%)
1	5.318	5362827	485056	50.02
2	5.928	5359577	452626	49.98

Supplementary Figure 18. Chiral HPLC analysis of racemic **9**

HPLC chromatogram of chiral 9

Condition: n-hexane/2-propanol = 90:10

Flow rate = 1.0 mL/min

λ = 254 nm

Chiral IA

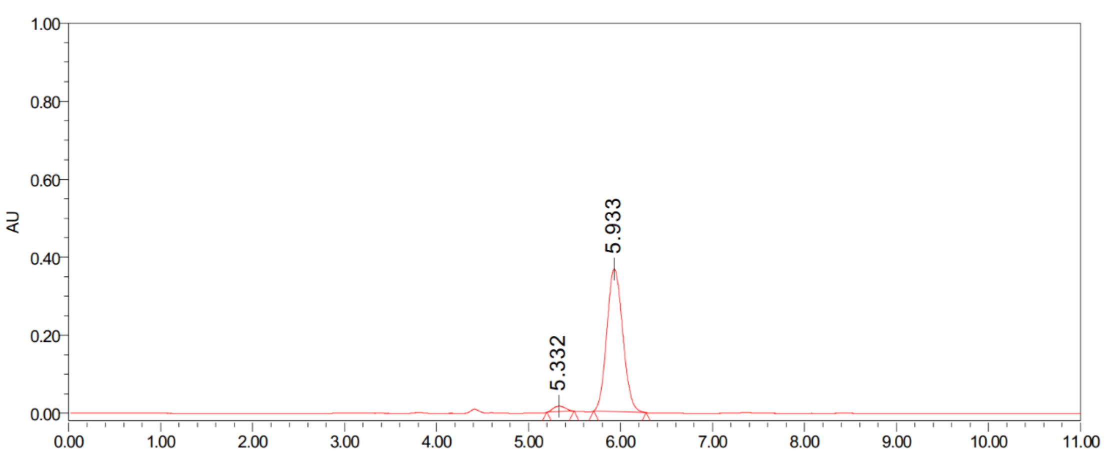

Entry	Retention Time/min	Area	Height	Area(%)
1	5.332	134818	14395	2.97
2	5.933	4406257	365597	97.03

Supplementary Figure 19. Chiral HPLC analysis of chiral **9**

***tert*-Butyl (2*R*,3*R*)-3-(2-methyl-1*H*-indol-3-yl)-2-((3-methylbenzyl)oxy)-2,5-diphenylpent-4-ynoate (10)**

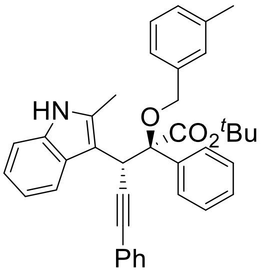

10

Yellow oil; 98.8 mg, 89% yield, >20:1 d.r., 91% *ee*, $[\alpha]_D^{20}$ = 30.22 (c = 0.033, MeOH); ^{1}H NMR (500 MHz, $CDCl_3$) δ 7.89 (s, 1H), 7.62 (s, 1H), 7.45 (d, J = 7.0 Hz, 2H), 7.26 (d, J = 5.9 Hz, 3H), 7.21 – 7.12 (m, 6H), 7.09 (dd, J = 14.4, 7.2 Hz, 3H), 7.01 (dd, J = 13.6, 6.6 Hz, 2H), 6.89 (t, J = 7.5 Hz, 1H), 4.88 (d, J = 12.6 Hz, 1H), 4.78 (s, 1H), 4.35 (d, J = 12.6 Hz, 1H), 2.21 (s, 3H), 1.61 (s, 3H), 1.52 (s, 9H). ^{13}C NMR (126 MHz, $CDCl_3$) δ 171.1, 140.1, 137.4, 136.4, 135.0, 134.4, 131.8, 128.9, 128.2, 128.0, 127.9, 127.7, 127.6, 127.4, 127.3, 124.3, 123.8, 120.7, 119.1, 109.7, 106.1, 89.8, 89.5, 83.1, 82.8, 68.3, 41.6, 28.2, 21.4, 11.3. HRMS(ESI) $[M+Na]^+$ calcd for $C_{38}H_{37}NO_3Na^+$,

578.2666, found 578.2665. (Chiral IA, λ = 254 nm, *n*-hexane/2-propanol = 90/10, Flow rate = 1.0 mL/min), t_R =4.869 min, 11.056 min (major).

HPLC chromatogram of racemic 10

Condition: n-hexane/2-propanol = 90:10

Flow rate = 1.0 mL/min

λ = 254 nm

Chiral IA

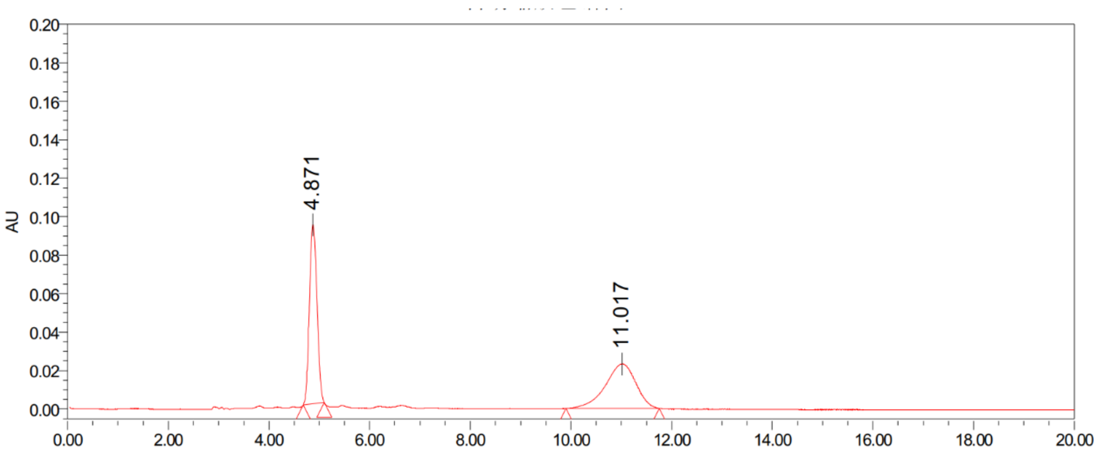

Entry	Retention Time/min	Area	Height	Area(%)
1	4.871	971838	93046	50.06
2	11.017	969335	23124	49.94

Supplementary Figure 20. Chiral HPLC analysis of racemic **10**

HPLC chromatogram of chiral 10

Condition: n-hexane/2-propanol = 90:10

Flow rate = 1.0 mL/min

λ = 254 nm

Chiral IA

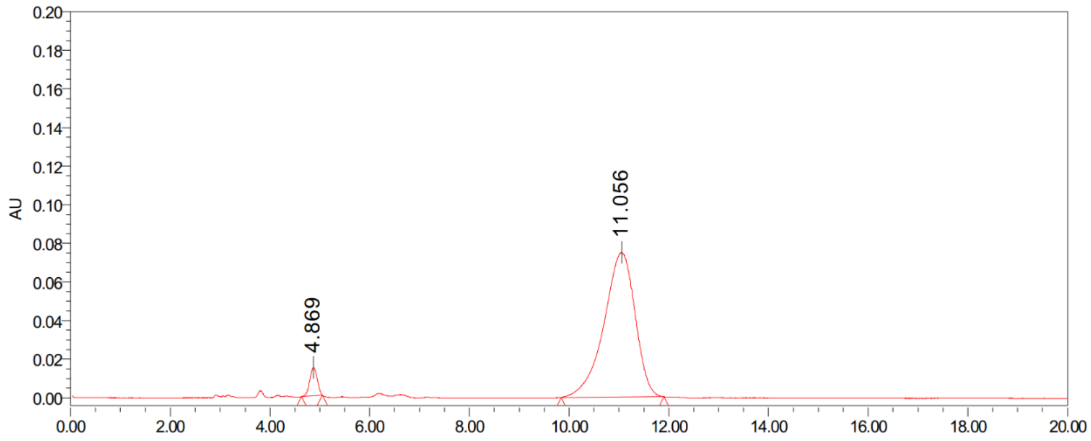

Entry	Retention Time/min	Area	Height	Area(%)
1	4.869	153051	14659	4.52
2	11.056	3234379	74842	95.48

Supplementary Figure 21. Chiral HPLC analysis of chiral **10**

***tert*-Butyl (2*R*,3*R*)-2-(furan-2-ylmethoxy)-3-(2-methyl-1*H*-indol-3-yl)-2,5-diphenylpent-4-ynoate (11)**

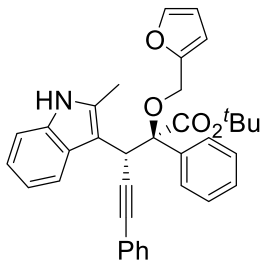

11

Yellow oil; 86.1 mg, 81% yield, >20:1 d.r., 88% *ee*, $[\alpha]_D^{20}$ = 63.29 (c = 0.033, MeOH); ^{1}H NMR (500 MHz, $CDCl_3$) δ 7.67 (s, 1H), 7.63 (s, 1H), 7.41 – 7.38 (m, 2H), 7.28 (s, 1H), 7.25 (dt, *J* = 8.5, 4.3 Hz, 3H), 7.20 – 7.17 (m, 1H), 7.15 (d, *J* = 7.9 Hz, 3H), 7.09 (t, *J* = 7.6 Hz, 2H), 7.00 (t, *J* = 7.5 Hz, 1H), 6.84 (t, *J* = 7.5 Hz, 1H), 6.26 (s, 1H), 6.18 (d, *J* = 3.0 Hz, 1H), 4.97 (d, *J* = 12.5 Hz, 1H), 4.70 (s, 1H), 4.25 (d, *J* = 12.5 Hz, 1H), 1.67 (s, 3H), 1.63 (s, 9H). ^{13}C NMR (126 MHz, $CDCl_3$) δ 171.1, 153.0, 141.7, 136.0, 134.8, 134.3, 131.7, 129.0, 128.1, 127.9, 127.5, 127.4, 124.3, 120.5, 118.9, 110.1, 109.4, 108.0, 106.1, 89.6, 89.4, 82.9, 82.8, 61.3, 41.4, 28.3, 11.3. HRMS(ESI) $[M+Na]^+$ calcd

for $C_{35}H_{33}NO_4Na^+$, 554.2302, found 554.2300. (Chiral IA, λ = 254 nm, *n*-hexane/2-propanol = 95/5, Flow rate = 1.0 mL/min), t_R =7.959 min, 13.869 min (major).

HPLC chromatogram of racemic 11

Condition: n-hexane/2-propanol = 95:5

Flow rate = 1.0 mL/min

λ = 254 nm

Chiral IA

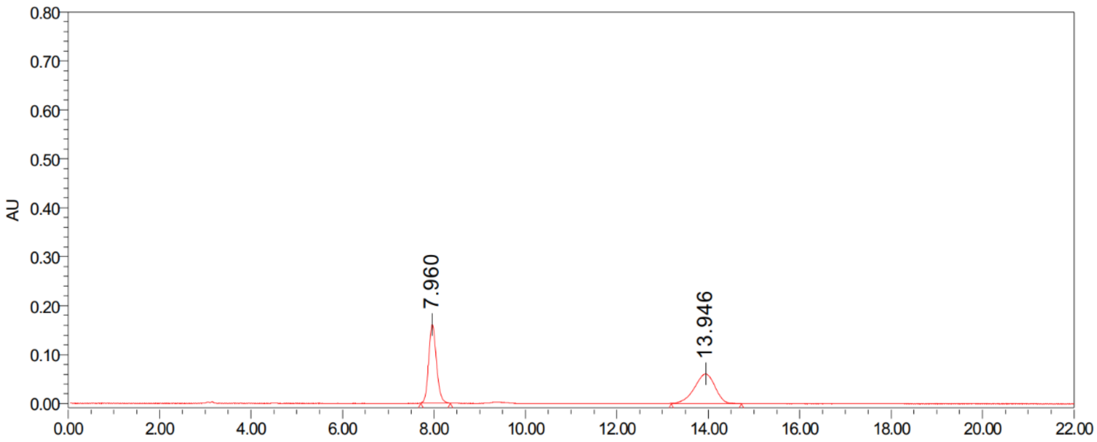

Entry	Retention Time/min	Area	Height	Area(%)
1	7.960	1888114	160626	49.90
2	13.946	1895507	60340	50.10

Supplementary Figure 22. Chiral HPLC analysis of racemic **11**

HPLC chromatogram of chiral 11

Condition: n-hexane/2-propanol = 95:5

Flow rate = 1.0 mL/min

λ = 254 nm

Chiral IA

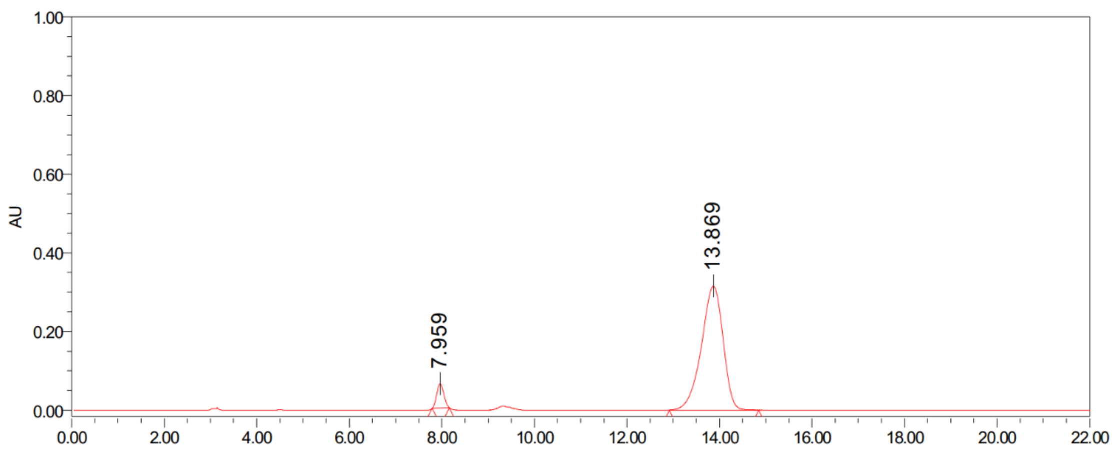

Entry	Retention Time/min	Area	Height	Area(%)
1	7.959	653936	61580	5.98
2	13.869	10276348	315178	94.02

Supplementary Figure 23. Chiral HPLC analysis of chiral **11**

tert-Butyl (2*R*,3*R*)-2-(benzo[b]thiophen-2-ylmethoxy)-3-(2-methyl-1*H*-indol-3-yl)-2,5-diphenylpent-4-ynoate (12)

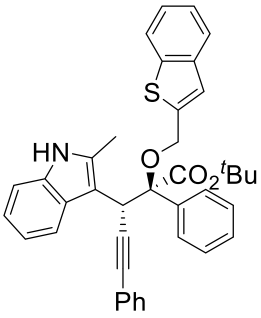

12

Brown solid, mp: 91.9 – 92.8 °C; 78.8 mg, 66% yield, >20:1 d.r., 94% *ee*, $[\alpha]_D^{20}$ = -3.59 (c = 0.033, MeOH); ^{1}H NMR (500 MHz, $CDCl_3$) δ 7.92 (s, 1H), 7.75 (d, *J* = 7.6 Hz, 1H), 7.61 (s, 1H), 7.56 (d, *J* = 7.5 Hz, 1H), 7.49 (d, *J* = 4.0 Hz, 2H), 7.27 (d, *J* = 5.0 Hz, 4H), 7.23 – 7.07 (m, 8H), 7.02 (t, *J* = 7.4 Hz, 1H), 6.87 (t, *J* = 7.4 Hz, 1H), 5.13 (d, *J* = 13.0 Hz, 1H), 4.76 (s, 1H), 4.57 (d, *J* = 13.1 Hz, 1H), 1.64 (s, 3H), 1.59 (s, 9H). ^{13}C NMR (126 MHz, $CDCl_3$) δ 170.9, 143.9, 139.9, 139.7, 136.0, 135.0, 134.4, 131.8, 129.0, 128.2, 128.1, 127.8, 127.63, 127.55, 124.2, 123.9, 123.6, 123.2, 122.2, 120.9,

120.7, 119.2, 109.7, 105.9, 89.8, 89.5, 83.2, 83.1, 64.9, 41.5, 28.3, 11.4. HRMS(ESI) $[M+Na]^+$ calcd for $C_{39}H_{35}NO_3SNa^+$, 620.2230, found 620.2231. (Chiral IA, λ = 254 nm, *n*-hexane/2-propanol = 95/5, Flow rate = 1.0 mL/min), t_R = 9.245 min, 11.002 min (major).

HPLC chromatogram of racemic 12

Condition: n-hexane/2-propanol = 95:5

Flow rate = 1.0 mL/min

λ = 254 nm

Chiral IA

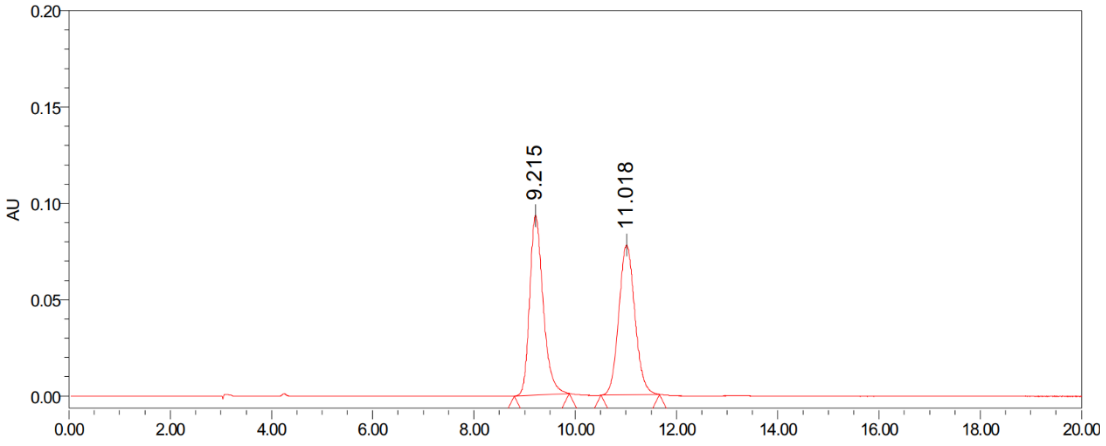

Entry	Retention Time/min	Area	Height	Area(%)
1	9.215	1708962	93171	50.04
2	11.018	1706154	77828	49.96

Supplementary Figure 24. Chiral HPLC analysis of racemic **12**

HPLC chromatogram of chiral 12

Condition: n-hexane/2-propanol = 95:5

Flow rate = 1.0 mL/min

λ = 254 nm

Chiral IA

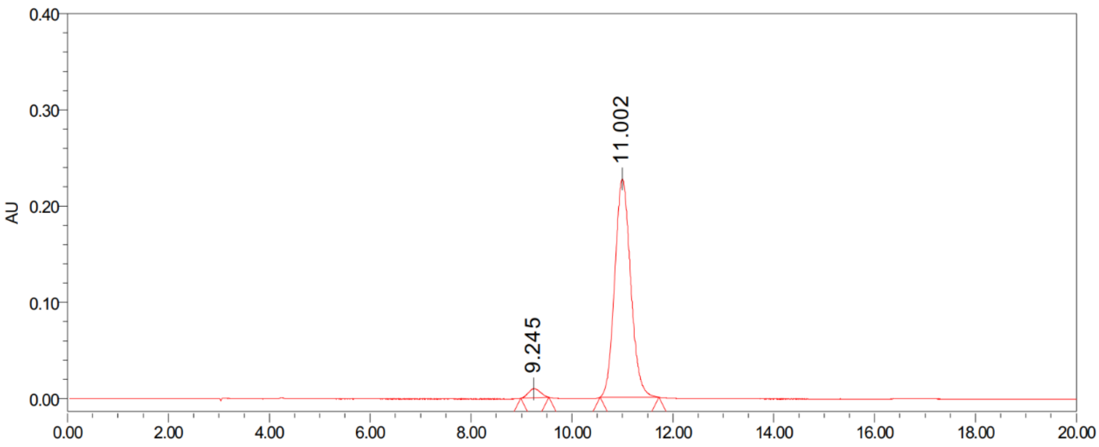

The larger version of HPLC chromatogram of chiral 12

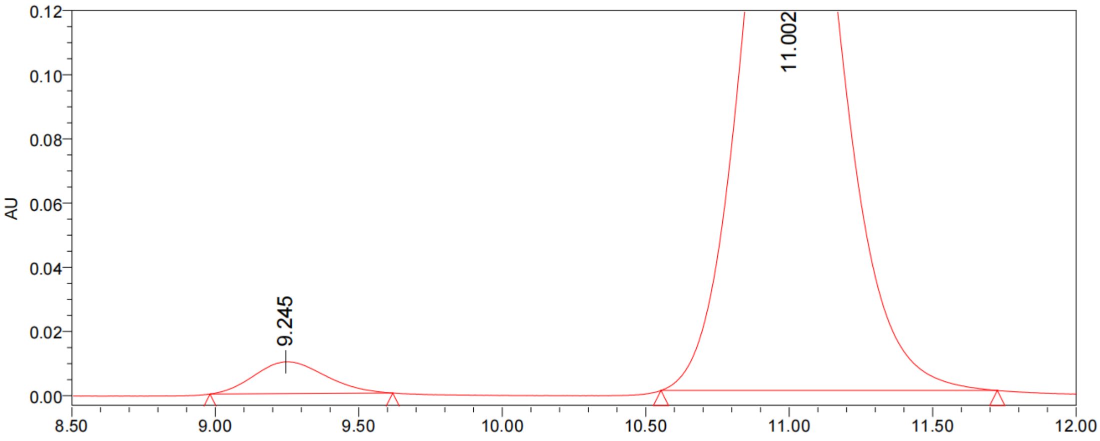

Entry	Retention Time/min	Area	Height	Area(%)
1	9.245	156854	9561	3.08
2	11.002	4940033	226728	96.92

Supplementary Figure 25. Chiral HPLC analysis of chiral **12**

***tert*-Butyl (2*R*,3*R*)-2-(cinnamyloxy)-3-(2-methyl-1*H*-indol-3-yl)-2,5-diphenylpent-4-ynoate (13)**

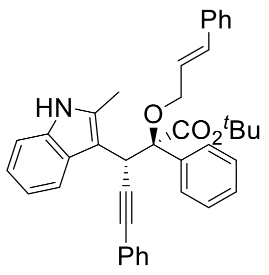

13

Yellow oil; 77.1 mg, 68% yield, >20:1 d.r., 90% *ee*, $[\alpha]_D^{20}$ = -6.77 (c = 0.033, MeOH); ^{1}H NMR (500 MHz, $CDCl_3$) δ 8.05 (s, 1H), 7.62 (s, 1H), 7.43 (dd, *J* = 6.5, 3.0 Hz, 2H), 7.24 (dd, *J* = 9.7, 5.1 Hz, 5H), 7.18 (dd, *J* = 6.3, 5.2 Hz, 5H), 7.12 – 7.05 (m, 5H), 7.01 (t, *J* = 7.4 Hz, 1H), 6.59 (d, *J* = 16.0 Hz, 1H), 6.20 (dt, *J* = 16.0, 4.8 Hz, 1H), 4.70 (s, 1H), 4.56 (ddd, *J* = 14.1, 4.6, 1.5 Hz, 1H), 3.94 (ddd, *J* = 14.2, 4.8, 1.4 Hz, 1H), 1.63 (s, 9H), 1.56 (s, 3H). ^{13}C NMR (126 MHz, $CDCl_3$) δ 171.3, 137.6, 136.5, 135.1, 134.3, 131.8, 129.9, 129.1, 128.5, 128.2, 127.9, 127.84, 127.78, 127.6, 127.4, 127.1, 126.4, 124.3, 120.8, 119.1, 109.9, 106.2, 89.8, 89.7, 83.0, 82.9, 67.5, 41.6, 28.4, 11.1. HRMS(ESI) $[M+Na]^+$ calcd for $C_{39}H_{37}NO_3Na^+$, 590.2666, found 590.2665. (Chiral IA, λ = 254 nm, *n*-hexane/2-propanol = 98/2, Flow rate = 1.0 mL/min), t_R = 23.652 min, 27.172 min (major).

HPLC chromatogram of racemic 13

Condition: n-hexane/2-propanol = 98:2

Flow rate = 1.0 mL/min

λ = 254 nm

Chiral IA

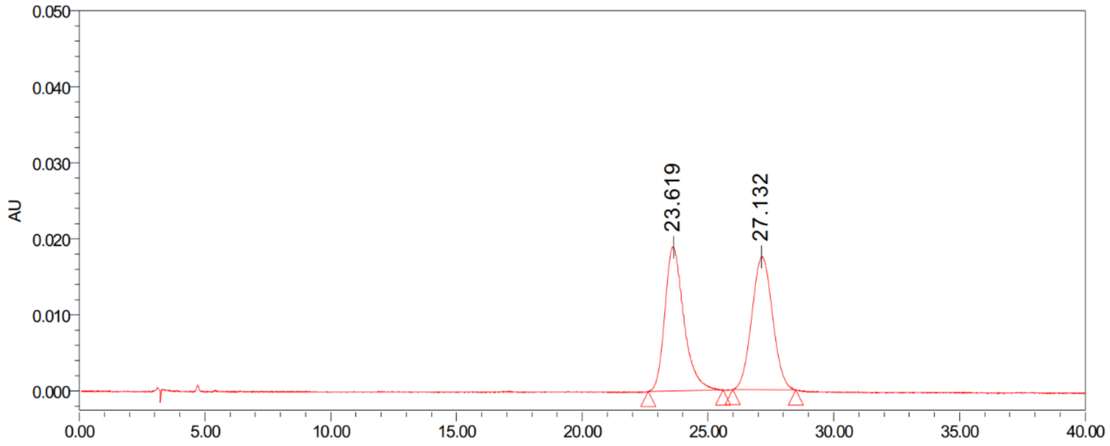

Entry	Retention Time/min	Area	Height	Area(%)
1	23.619	1003153	18908	50.00
2	27.132	1003050	17482	50.00

Supplementary Figure 26. Chiral HPLC analysis of racemic **13**

HPLC chromatogram of chiral 13

Condition: n-hexane/2-propanol = 98:2

Flow rate = 1.0 mL/min

λ = 254 nm

Chiral IA

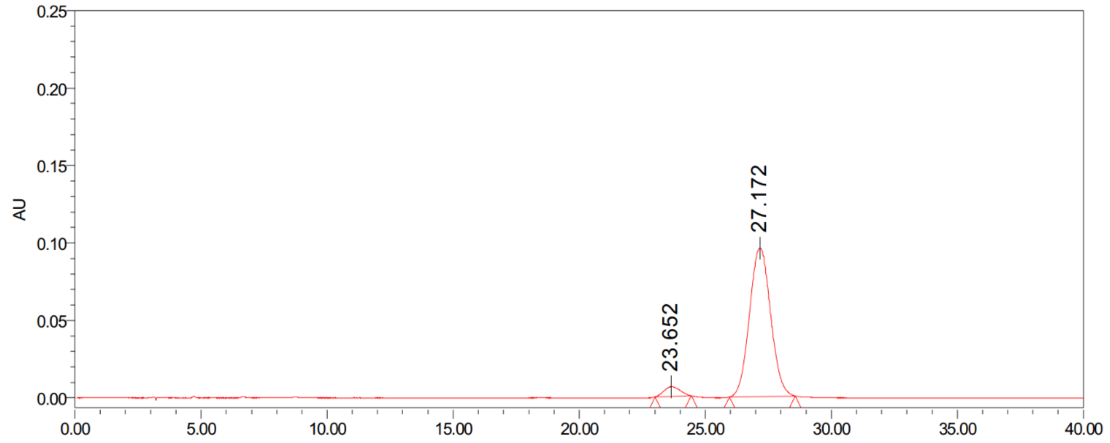

Entry	Retention Time/min	Area	Height	Area(%)
1	23.652	279299	6244	4.80

2	27.172	5534956	95859	95.20

Supplementary Figure 27. Chiral HPLC analysis of chiral **13**

***tert*-Butyl (2*R*,3*R*)-2-(allyloxy)-3-(2-methyl-1*H*-indol-3-yl)-2,5-diphenylpent-4-ynoate (14)**

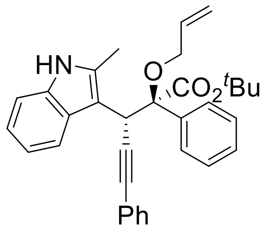

14

Yellow solid, mp: 117.4 – 118.4 °C; 49.1 mg, 50% yield, >20:1 d.r., 68% *ee*, $[\alpha]_D^{20}$ = 45.29 (c = 0.033, MeOH); ^{1}H NMR (500 MHz, $CDCl_3$) δ 7.92 (s, 1H), 7.64 (s, 1H), 7.43 – 7.39 (m, 2H), 7.28 – 7.22 (m, 3H), 7.18 (dd, *J* = 10.0, 6.6 Hz, 2H), 7.06 (dd, *J* = 15.5, 6.2 Hz, 5H), 6.99 (t, *J* = 7.4 Hz, 1H), 5.98 – 5.81 (m, 1H), 5.26 (dd, *J* = 17.3, 1.8 Hz, 1H), 5.03 (dd, *J* = 10.6, 1.6 Hz, 1H), 4.69 (s, 1H), 4.36 (ddd, *J* = 13.4, 2.8, 1.9 Hz, 1H), 3.82 – 3.75 (m, 1H), 1.64 (s, 3H), 1.62 (s, 9H). ^{13}C NMR (126 MHz, $CDCl_3$) δ 171.1, 136.5, 136.0, 135.0, 134.2, 131.7, 129.0, 128.1, 127.9, 127.8, 127.5, 127.3, 124.3, 120.7, 119.0, 115.0, 109.6, 106.3, 89.7, 89.3, 82.8, 82.7, 67.9, 41.4, 28.3, 11.3. HRMS(ESI) $[M+Na]^+$ calcd for $C_{33}H_{33}NO_3Na^+$, 514.2353, found 514.2352. (Chiral IA, λ = 254 nm, *n*-hexane/2-propanol = 95/5, Flow rate = 1.0 mL/min), t_R = 6.917 min, 9.166 min (major).

HPLC chromatogram of racemic 14

Condition: n-hexane/2-propanol = 95:5

Flow rate = 1.0 mL/min

λ = 254 nm

Chiral IA

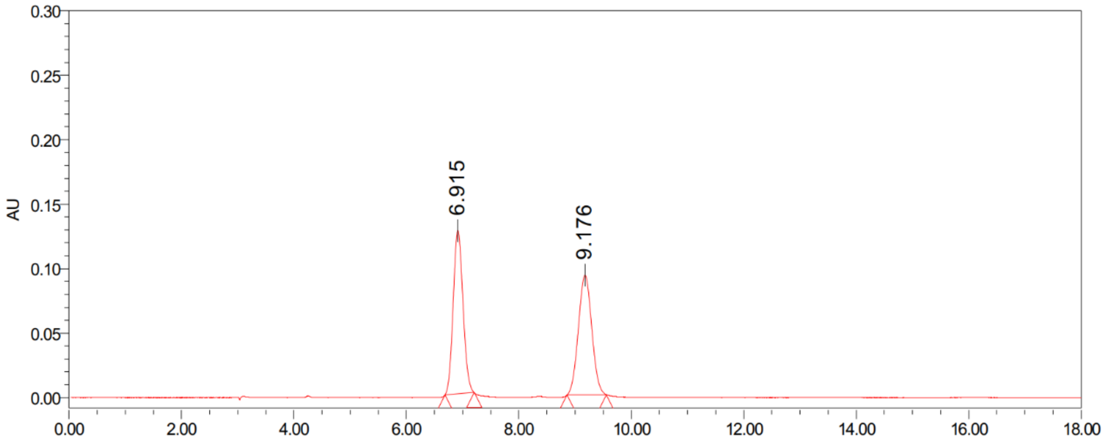

Entry	Retention Time/min	Area	Height	Area(%)
1	6.915	1537068	126405	49.91
2	9.176	1542638	92762	50.09

Supplementary Figure 28. Chiral HPLC analysis of racemic **14**

HPLC chromatogram of chiral 14

Condition: n-hexane/2-propanol = 95:5

Flow rate = 1.0 mL/min

λ = 254 nm

Chiral IA

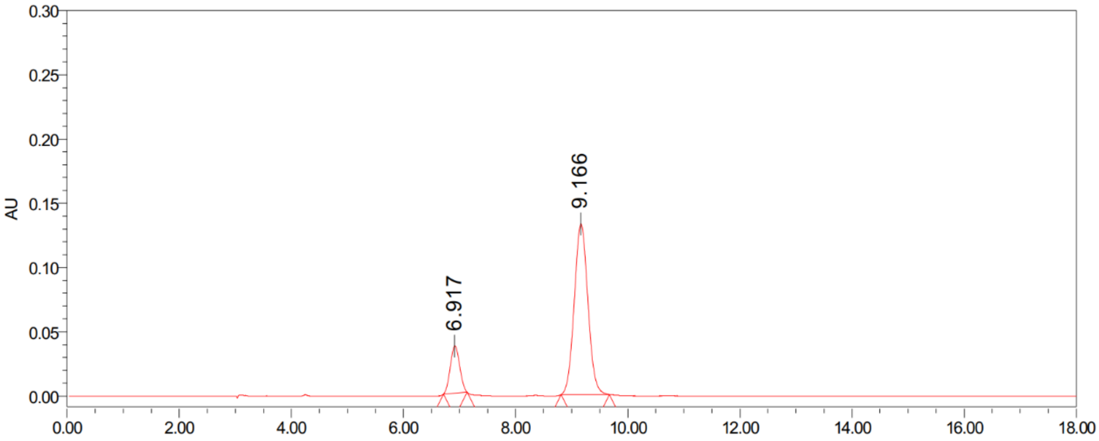

Entry	Retention Time/min	Area	Height	Area(%)
1	6.917	426024	36603	15.91
2	9.166	2251782	132731	84.09

Supplementary Figure 29. Chiral HPLC analysis of chiral **14**

tert-Butyl (2*R*,3*R*)-3-(2-methyl-1*H*-indol-3-yl)-2,5-diphenyl-2-((3-phenylprop-2-yn-1-yl)oxy)pent-4-ynoate (15)

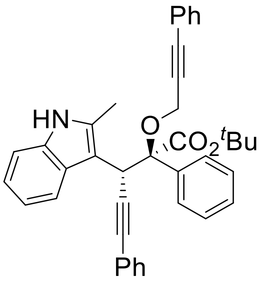

15

Yellow oil; 50.9 mg, 45% yield, >20:1 d.r., 91% *ee*, $[\alpha]_D^{20}$ = 15.74 (c = 0.033, MeOH); ^{1}H NMR (500 MHz, $CDCl_3$) δ 7.92 (s, 1H), 7.64 (s, 1H), 7.45 – 7.42 (m, 2H), 7.30 – 7.26 (m, 5H), 7.26 – 7.22 (m, 3H), 7.16 (dd, *J* = 9.6, 4.9 Hz, 4H), 7.08 (t, *J* = 7.6 Hz, 2H), 7.02 (t, *J* = 7.4 Hz, 1H), 6.96 (t, *J* = 7.4 Hz, 1H), 4.86 (d, *J* = 15.6 Hz, 1H), 4.69 (s, 1H), 4.10 (d, *J* = 15.6 Hz, 1H), 1.78 (s, 3H), 1.64 (s, 9H). ^{13}C NMR (126 MHz, $CDCl_3$) δ 171.0, 135.8, 134.9, 134.3, 131.8, 131.6, 129.0, 128.13, 128.08, 128.03, 127.96, 127.7, 127.5, 124.2, 123.3, 120.6, 119.1, 109.5, 106.1, 89.9, 89.5, 87.0, 85.2, 83.0, 82.8, 56.0, 41.3, 28.3, 11.5. HRMS(ESI) $[M+Na]^+$ calcd for $C_{39}H_{35}NO_3Na^+$,

588.2509, found 588.2513. (Chiral IA, λ = 254 nm, *n*-hexane/2-propanol = 95/5, Flow rate = 1.0 mL/min), t_R = 9.774 min, 13.980 min (major).

HPLC chromatogram of racemic 15

Condition: n-hexane/2-propanol = 95:5

Flow rate = 1.0 mL/min

λ = 254 nm

Chiral IA

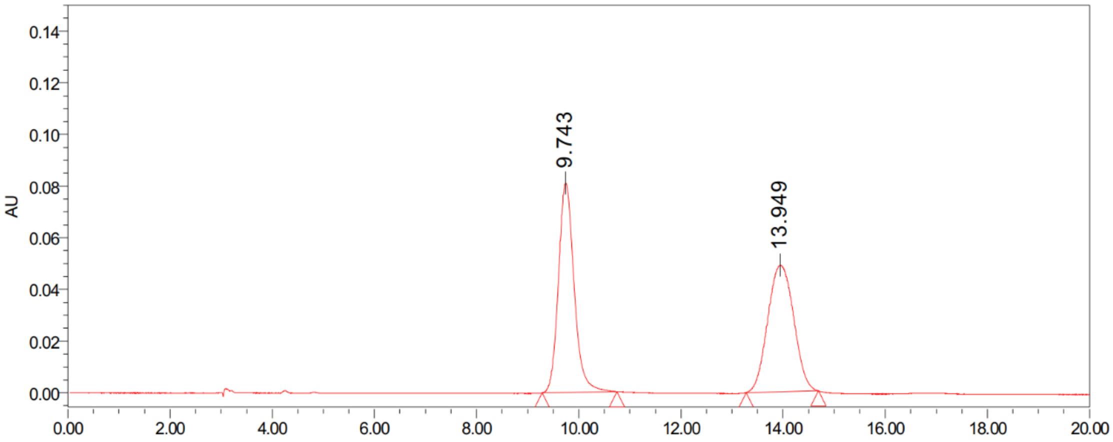

Entry	Retention Time/min	Area	Height	Area(%)
1	9.743	1731460	80994	50.09
2	13.949	1725249	48998	49.91

Supplementary Figure 30. Chiral HPLC analysis of racemic **15**

HPLC chromatogram of chiral 15

Condition: n-hexane/2-propanol = 95:5

Flow rate = 1.0 mL/min

λ = 254 nm

Chiral IA

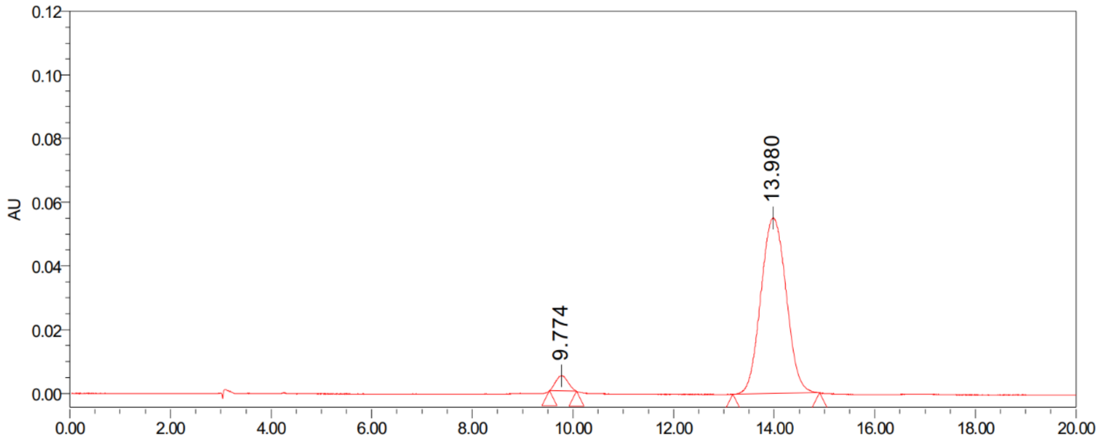

The larger version of HPLC chromatogram of chiral 15

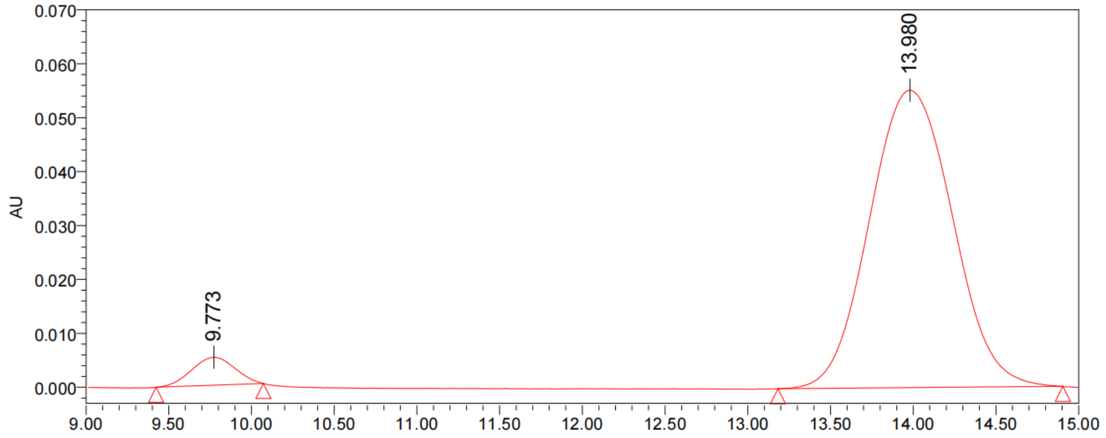

Entry	Retention Time/min	Area	Height	Area(%)
1	9.774	93920	5193	4.45
2	13.980	1926825	55178	95.55

Supplementary Figure 31. Chiral HPLC analysis of chiral **15**

***tert*-Butyl (2*R*,3*R*)-2-((4-bromobenzyl)oxy)-2-(2-fluorophenyl)-3-(2-methyl-1*H*-indol-3-yl)-5-phenylpent-4-ynoate (16)**

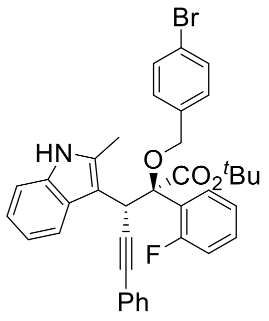

16

Yellow oil; 104.5 mg, 82% yield, >20:1 d.r., 93% *ee*, $[\alpha]_D^{20}$ = 27.69 (c = 0.033, MeOH); ^{1}H NMR (500 MHz, $CDCl_3$) δ 7.60 (s, 1H), 7.49 (d, *J* = 8.0 Hz, 1H), 7.41 (d, *J* = 8.4 Hz, 2H), 7.37 (s, 2H), 7.37 – 7.35 (m, 2H), 7.29 – 7.26 (m, 3H), 7.25 (s, 1H), 7.14 (dd, *J* = 10.4, 4.3 Hz, 1H), 7.10 (d, *J* = 8.0 Hz, 1H), 6.97 (t, *J* = 7.5 Hz, 1H), 6.89 (t, *J* = 7.6 Hz, 1H), 6.80 (t, *J* = 7.5 Hz, 1H), 6.74 (dd, *J* = 11.3, 8.3 Hz, 1H), 5.17 (s, 1H), 5.00 (d, *J* = 12.2 Hz, 1H), 4.60 (d, *J* = 12.2 Hz, 1H), 2.10 (s, 3H), 1.39 (s, 9H). ^{13}C NMR (126 MHz, $CDCl_3$) δ 168.6, δ 160.1 (d, *J* = 247.0 Hz), 138.2, 134.9, 133.7, 131.6, 131.3, 129.52 (d, *J* = 23.9 Hz), 129.50 (d, *J* = 11.3 Hz), 129.0, 128.5, 128.2, 127.6, 126.4 (d, *J* = 11.3 Hz), 124.2, 123.2 (d, *J* = 2.5 Hz), 121.0, 120.7, 120.5, 118.8, 115.3 (d, *J* = 22.7 Hz), 109.5, 106.8, 89.6, 85.7, 83.2, 82.7, 67.3, 37.6, 27.8, 12.2. ^{19}F NMR (376 MHz, $CDCl_3$) δ -109.74. HRMS(ESI) $[M+Na]^+$ calcd for $C_{37}H_{33}BrFNO_3Na^+$, 660.1520, found 660.1524. (Chiral IC, λ = 254 nm, *n*-hexane/2-propanol = 98/2, Flow rate = 1.0 mL/min), t_R = 7.585 min, 12.561 min (major).

HPLC chromatogram of racemic 16

Condition: n-hexane/2-propanol = 98:2

Flow rate = 1.0 mL/min

λ = 254 nm

Chiral IC

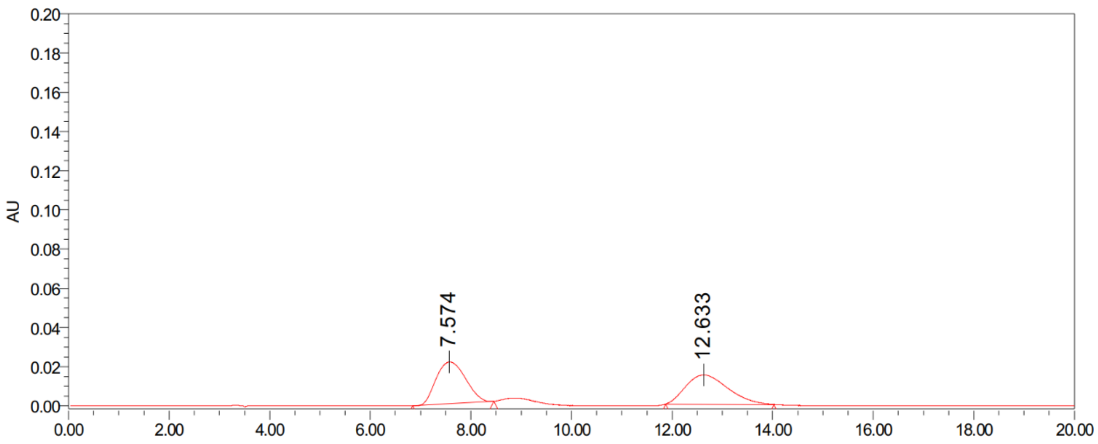

Entry	Retention Time/min	Area	Height	Area(%)
1	7.574	871264	21326	49.96
2	12.633	872552	14880	50.04

Supplementary Figure 32. Chiral HPLC analysis of racemic **16**

HPLC chromatogram of chiral 16

Condition: n-hexane/2-propanol = 98:2

Flow rate = 1.0 mL/min

λ = 254 nm

Chiral IC

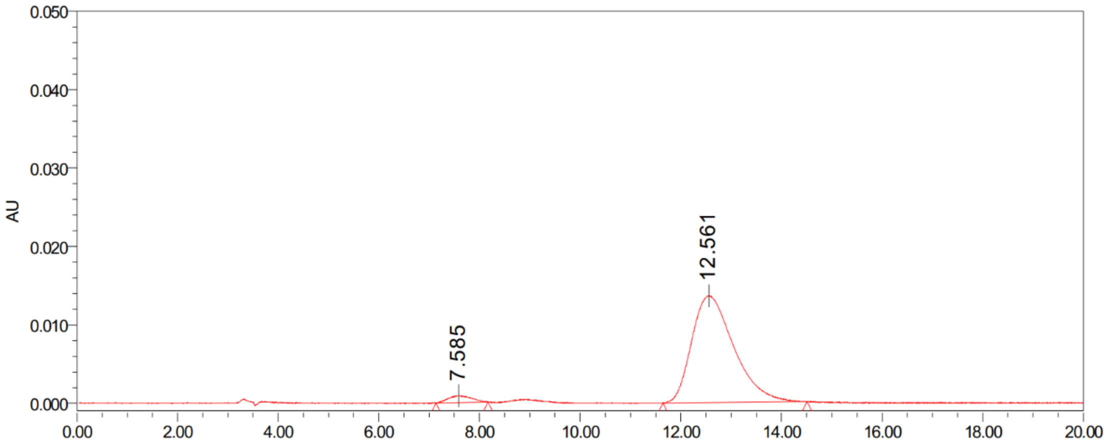

The larger version of HPLC chromatogram of chiral 16

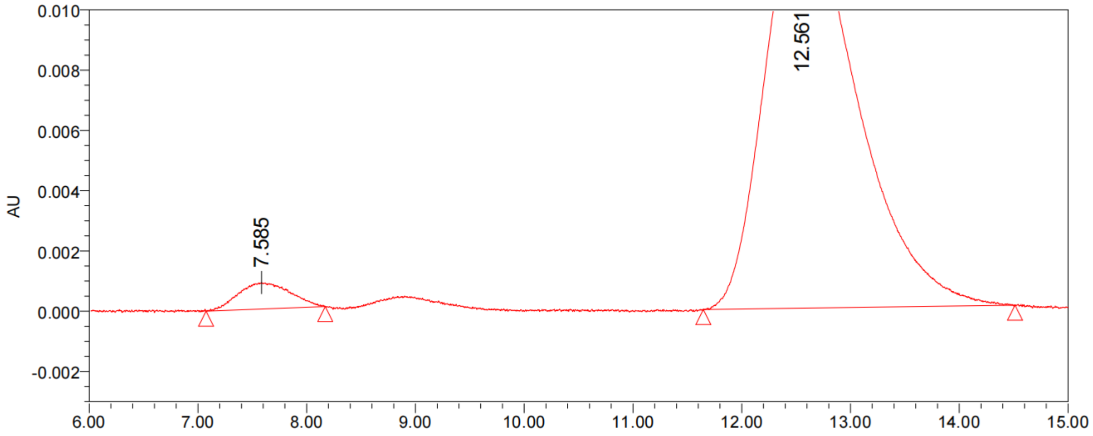

Entry	Retention Time/min	Area	Height	Area(%)
1	7.585	27962	862	3.48
2	12.561	788644	13587	96.52

Supplementary Figure 33. Chiral HPLC analysis of chiral **16**

***tert*-Butyl (2*R*,3*R*)-2-((4-bromobenzyl)oxy)-3-(2-methyl-1*H*-indol-3-yl)-2-(naphthalen-2-yl)-5-phenylpent-4-ynoate (17)**

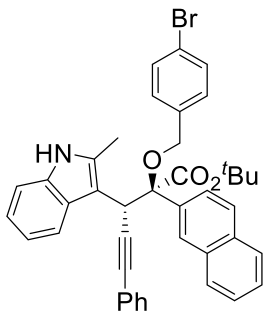

17

Yellow solid, mp: 99.1 – 99.9 °C; 89.7 mg, 67% yield, >20:1 d.r., 92% *ee*, $[\alpha]_D^{20}$ = 42.19 (c = 0.033, MeOH); ^{1}H NMR (400 MHz, $CDCl_3$) δ 7.98 (d, *J* = 6.9 Hz, 1H), 7.87 (s, 1H), 7.73 – 7.69 (m, 1H), 7.68 – 7.64 (m, 1H), 7.58 (s, 1H), 7.43 (ddd, *J* = 6.5, 5.4, 2.9 Hz, 5H), 7.34 (d, *J* = 8.4 Hz, 2H), 7.30 – 7.25 (m, 5H), 7.18 (d, *J* = 8.0 Hz, 1H), 7.04 (t, *J* = 7.3 Hz, 1H), 6.89 (dd, *J* = 13.8, 7.3 Hz, 2H), 4.85 (s, 1H), 4.82 (d, *J* = 13.3 Hz, 1H), 4.30 (d, *J* = 12.9 Hz, 1H), 1.57 (s, 9H), 1.40 (s, 3H). ^{13}C NMR (126 MHz, $CDCl_3$) δ 171.0, 139.2, 135.1, 134.1, 134.1, 132.8, 132.6, 131.7, 131.0, 128.8, 128.6, 128.5, 128.3, 127.8, 127.4, 127.3, 126.5, 126.4, 125.9, 125.8, 124.1, 120.9, 120.3, 119.2, 109.8, 106.1, 89.8, 89.6, 83.3, 83.1, 67.9, 41.7, 28.3, 11.2. HRMS(ESI) $[M+Na]^+$ calcd for $C_{41}H_{36}BrNO_3Na^+$, 692.1771, found 692.1770. (Chiral IA, λ = 254 nm, *n*-hexane/2-propanol = 95/5, Flow rate = 1.0 mL/min), t_R = 8.828 min, 9.773 min (major).

HPLC chromatogram of racemic 17

Condition: n-hexane/2-propanol = 95:5

Flow rate = 1.0 mL/min

λ = 254 nm

Chiral IA

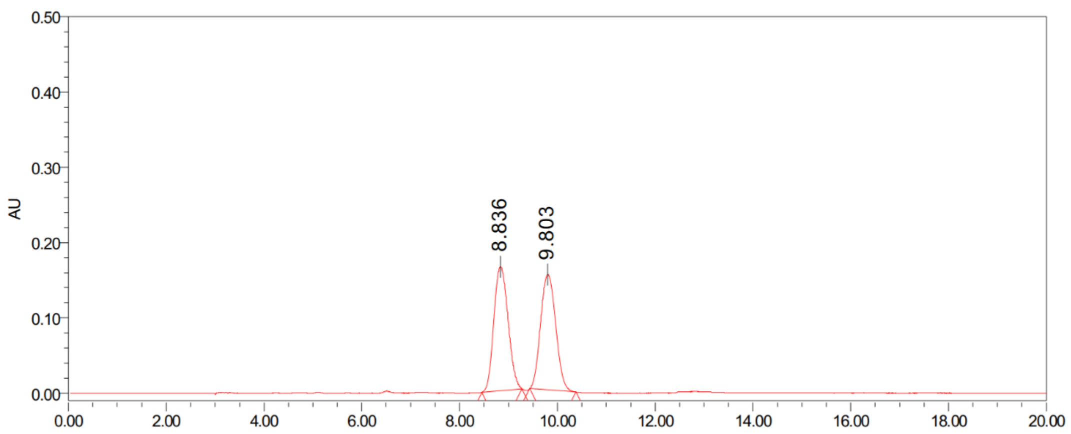

Entry	Retention Time/min	Area	Height	Area(%)
1	8.836	3298871	164440	49.93
2	9.803	3308060	153201	50.07

Supplementary Figure 34. Chiral HPLC analysis of racemic **17**

HPLC chromatogram of chiral 17

Condition: n-hexane/2-propanol = 95:5

Flow rate = 1.0 mL/min

λ = 254 nm

Chiral IA

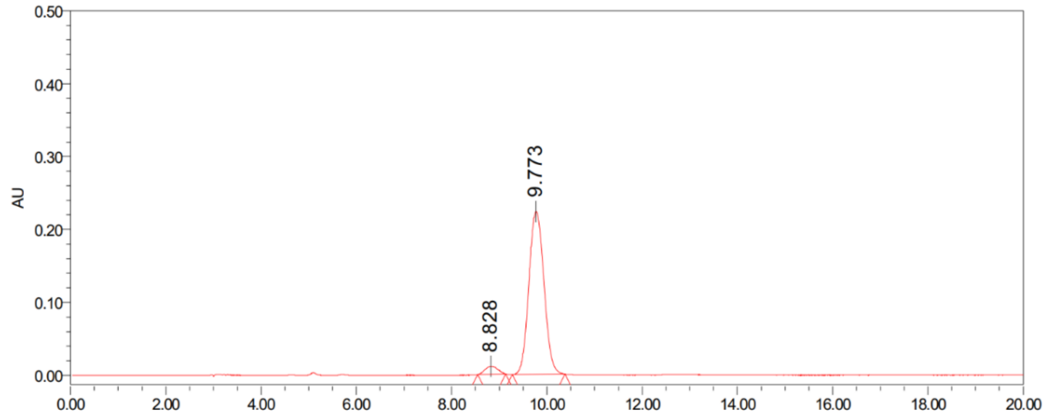

Entry	Retention Time/min	Area	Height	Area(%)
1	8.828	202709	10981	3.94
2	9.773	4936347	223403	96.06

Supplementary Figure 35. Chiral HPLC analysis of chiral **17**

tert-Butyl (2*R*,3*R*)-2-((4-bromobenzyl)oxy)-3-(2-methyl-1*H*-indol-3-yl)-5-phenyl-2-(*m*-tolyl)pent-4-ynoate (18)

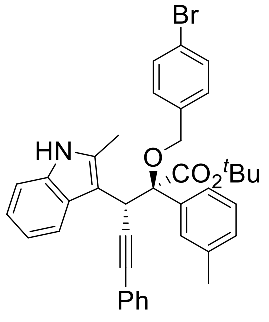

18

Yellow oil; 88.6 mg, 70% yield, >20:1 d.r., 92% *ee*, $[\alpha]_D^{20}$ = -6.24 (c = 0.033, MeOH); ^{1}H NMR (400 MHz, $CDCl_3$) δ 7.82 (d, *J* = 5.5 Hz, 1H), 7.66 (s, 1H), 7.46 – 7.42 (m, 2H), 7.34 (d, *J* = 8.4 Hz, 2H), 7.31 – 7.25 (m, 5H), 7.18 (d, *J* = 8.0 Hz, 1H), 7.00 (ddd, *J* = 18.3, 13.3, 7.3 Hz, 3H), 6.88 (dd, *J* = 15.9, 8.2 Hz, 3H), 4.81 (d, *J* = 13.0 Hz, 1H), 4.73 (s, 1H), 4.29 (d, *J* = 13.0 Hz, 1H), 2.10 (s, 3H), 1.65 (s, 3H), 1.53 (s,9H). ^{13}C NMR (126 MHz, $CDCl_3$) δ 171.0, 139.4, 137.0, 136.0, 135.0, 134.3, 131.6, 130.9, 129.0, 128.7, 128.58, 128.55, 128.2, 127.7, 127.3, 125.0, 124.1, 120.7, 120.2, 119.1, 109.7, 106.1, 89.7, 89.6, 83.1, 82.9, 67.7, 41.4, 28.2, 21.5, 11.3. HRMS(ESI) $[M+Na]^+$ calcd for $C_{38}H_{36}BrNO_3Na^+$, 656.1771, found 656.1771. (Chiral IA, λ = 254 nm, *n*-hexane/2-propanol = 95/5, Flow rate = 1.0 mL/min), t_R =6.970 min, 8.280 min (major).

HPLC chromatogram of racemic 18

Condition: n-hexane/2-propanol = 95:5

Flow rate = 1.0 mL/min

λ = 254 nm

Chiral IA

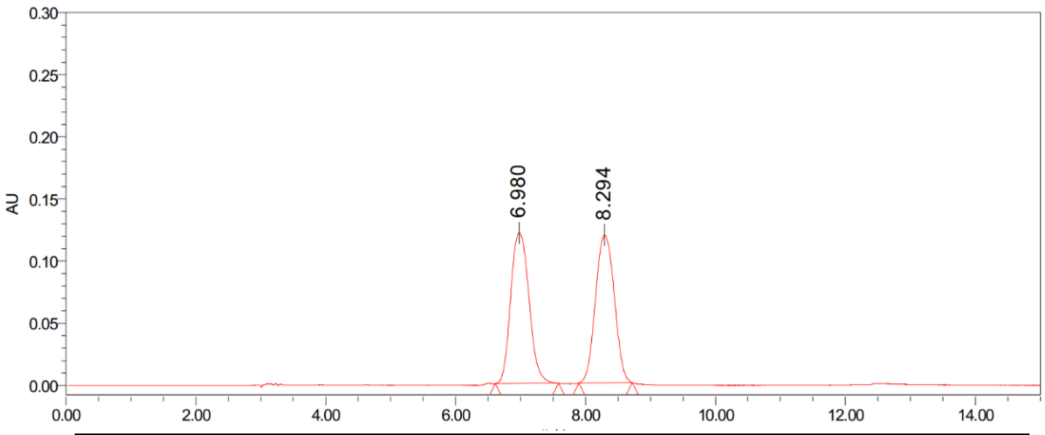

Entry	Retention Time/min	Area	Height	Area(%)
1	6.980	2374601	121233	49.92
2	8.294	2382686	118785	50.08

Supplementary Figure 36. Chiral HPLC analysis of racemic **18**

HPLC chromatogram of chiral 18

Condition: n-hexane/2-propanol = 95:5

Flow rate = 1.0 mL/min

λ = 254 nm

Chiral IA

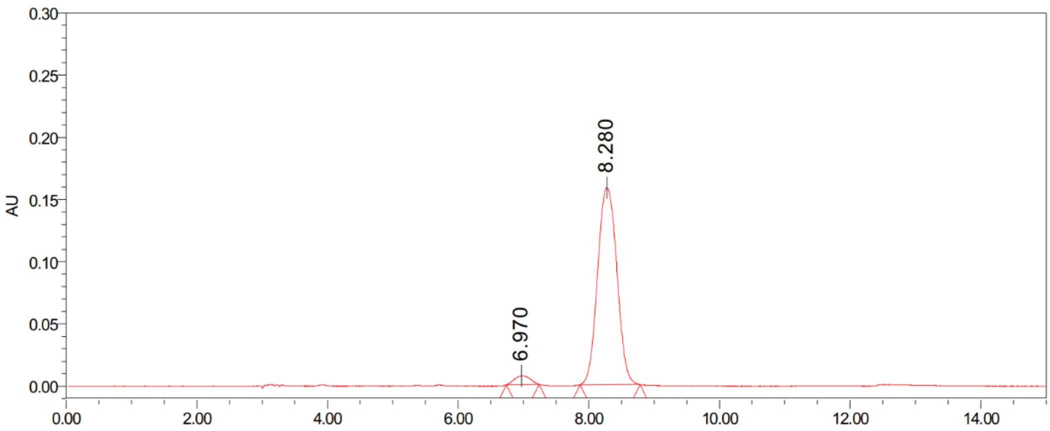

Entry	Retention Time/min	Area	Height	Area(%)
1	6.970	117329	6866	3.82
2	8.280	3228799	158349	96.18

Supplementary Figure 37. Chiral HPLC analysis of chiral **18**

***tert*-Butyl (2*R*,3*R*)-2-((4-bromobenzyl)oxy)-3-(2-methyl-1*H*-indol-3-yl)-5-phenyl-2-(*p*-tolyl)pent-4-ynoate (19)**

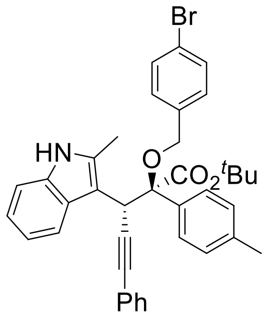

19

Yellow oil; 69.7 mg, 55% yield, >20:1 d.r., 92% *ee*, $[\alpha]_D^{20}$ = 6.58 (c = 0.033, MeOH); ^{1}H NMR (400 MHz, $CDCl_3$) δ 7.92 (d, *J* = 7.0 Hz, 1H), 7.67 (s, 1H), 7.44 (dd, *J* = 7.0, 2.4 Hz, 2H), 7.33 (d, *J* = 8.4 Hz, 2H), 7.28 (dd, *J* = 9.2, 5.4 Hz, 5H), 7.19 (d, *J* = 8.0 Hz, 1H), 7.04 (t, *J* = 7.4 Hz, 1H), 6.96 (d, *J* = 8.0 Hz, 2H), 6.91 (t, *J* = 7.1 Hz, 3H), 4.79 (d, *J* = 13.0 Hz, 1H), 4.72 (s, 1H), 4.28 (d, *J* = 13.0 Hz, 1H), 2.27 (s, 3H), 1.59 (s, 3H), 1.53 (s, 9H). ^{13}C NMR (126 MHz, $CDCl_3$) δ 171.1, 155.3, 139.4, 137.7, 135.0, 134.3, 133.1, 131.6, 130.9, 128.9, 128.5, 128.2, 128.1, 127.8, 127.7, 124.1, 120.7, 120.2, 119.1, 109.7, 106.1, 89.7, 89.6, 83.0, 82.8, 67.6, 41.6, 28.2, 21.1, 11.2. HRMS(ESI) $[M+Na]^+$ calcd for $C_{38}H_{36}BrNO_3Na^+$, 656.1771, found 656.1772. (Chiral IA, λ = 254 nm, *n*-hexane/2-propanol = 98/2, Flow rate = 1.0 mL/min), t_R = 16.428 min, 19.302 min (major).

HPLC chromatogram of racemic 19

Condition: n-hexane/2-propanol = 98:2

Flow rate = 1.0 mL/min

λ = 254 nm

Chiral IA

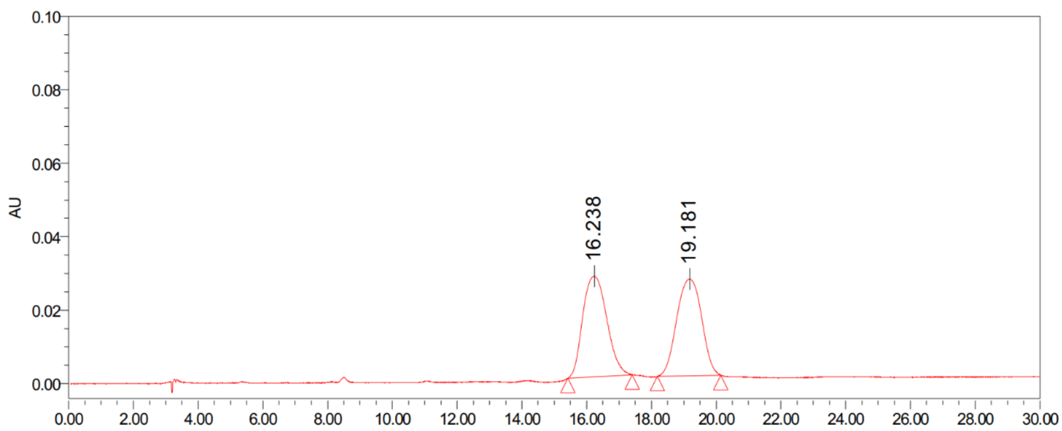

Entry	Retention Time/min	Area	Height	Area(%)
1	16.238	1412539	27362	50.02
2	19.181	1411551	26327	49.98

Supplementary Figure 38. Chiral HPLC analysis of racemic **19**

HPLC chromatogram of chiral 19

Condition: n-hexane/2-propanol = 98:2

Flow rate = 1.0 mL/min

λ = 254 nm

Chiral IA

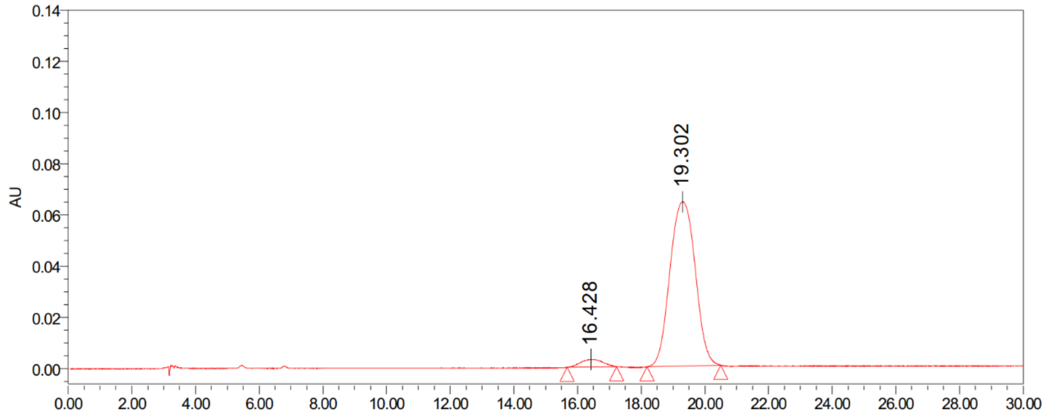

Entry	Retention Time/min	Area	Height	Area(%)
1	16.428	144041	2950	3.90
2	19.302	3549106	64172	96.10

Supplementary Figure 39. Chiral HPLC analysis of chiral **19**

***tert*-Butyl (2*R*,3*R*)-2-((4-bromobenzyl)oxy)-2-(4-methoxyphenyl)-3-(2-methyl-1*H*-indol-3-yl)-5-phenylpent-4-ynoate (20)**

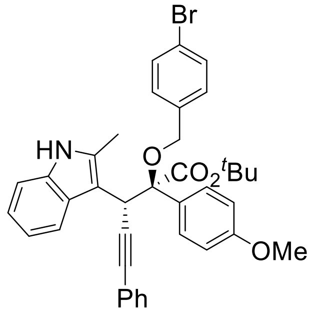

20

Yellow solid, mp: 94.7 - 95.6 °C; 79.2 mg, 61% yield, >20:1 d.r., 91% *ee*, $[\alpha]_D^{20}$ = 15.87 (c = 0.033, MeOH); ^{1}H NMR (400 MHz, $CDCl_3$) δ 7.91 (d, *J* = 6.0 Hz, 1H), 7.67 (s, 1H), 7.46 – 7.42 (m, 2H), 7.33 (d, *J* = 8.4 Hz, 2H), 7.30 – 7.27 (m, 5H), 7.17 (d, *J* = 8.0 Hz, 1H), 7.04 (t, *J* = 7.3 Hz, 1H), 6.98 (d, *J* = 8.5 Hz, 2H), 6.91 (t, *J* = 7.5 Hz, 1H), 6.62 (d, *J* = 9.0 Hz, 2H), 4.78 (d, *J* = 13.0 Hz, 1H), 4.70 (s, 1H), 4.25 (d, *J* = 13.0 Hz, 1H), 3.72 (s, 3H), 1.62 (s, 3H), 1.54 (s, 9H). ^{13}C NMR (126 MHz, $CDCl_3$) δ 171.2, 159.4, 139.3, 135.0, 134.3, 131.7, 130.9, 129.2, 128.8, 128.5, 128.3, 128.2, 127.7, 124.1, 120.8, 120.2, 119.1, 112.7, 109.8, 106.0, 89.7, 89.3, 83.1, 82.9, 67.5, 55.3, 41.6, 28.2, 11.3. HRMS(ESI) $[M+Na]^+$ calcd for $C_{38}H_{36}BrNO_4Na^+$, 672.1720, found 672.1724. (Chiral IA, λ = 254 nm, *n*-hexane/2-propanol = 95/5, Flow rate = 1.0 mL/min), t_R = 10.092 min, 10.901 min (major).

HPLC chromatogram of racemic 20

Condition: n-hexane/2-propanol = 95:5

Flow rate = 1.0 mL/min

λ = 254 nm

Chiral IA

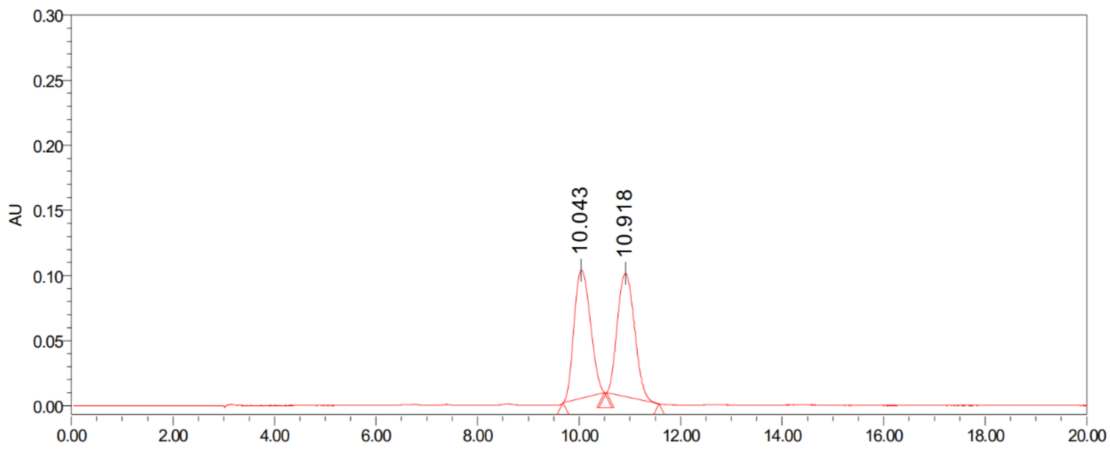

Entry	Retention Time/min	Area	Height	Area(%)
1	10.043	2222108	98520	49.96
2	10.918	2225835	94643	50.04

Supplementary Figure 40. Chiral HPLC analysis of racemic **20**

HPLC chromatogram of chiral 20

Condition: n-hexane/2-propanol = 95:5

Flow rate = 1.0 mL/min

λ = 254 nm

Chiral IA

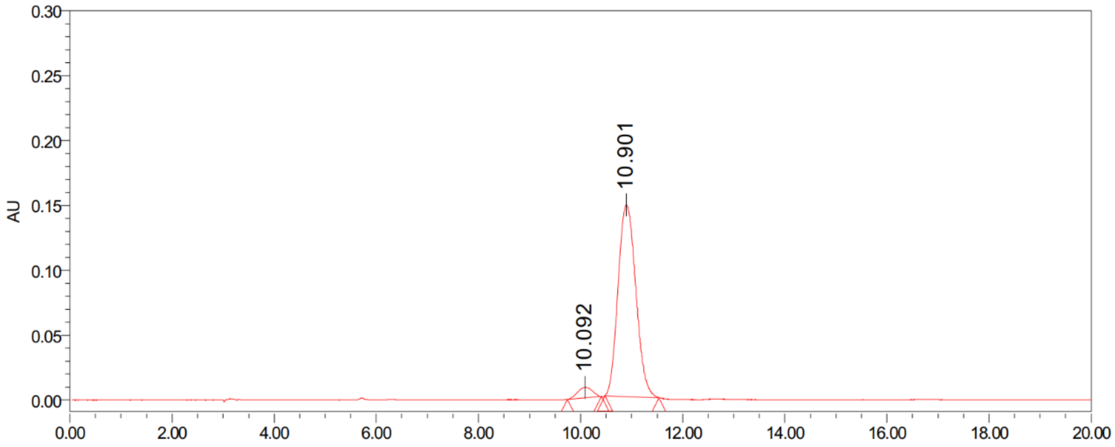

The larger version of HPLC chromatogram of chiral 20

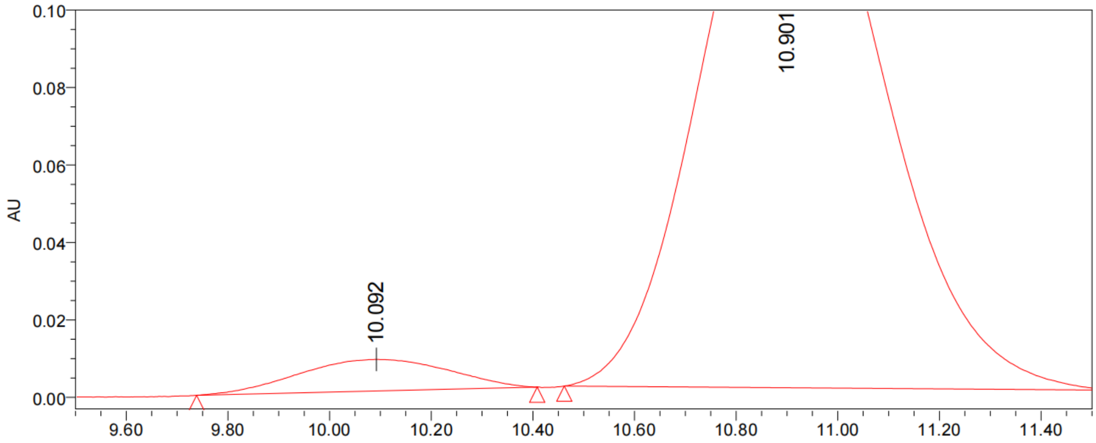

Entry	Retention Time/min	Area	Height	Area(%)
1	10.092	166761	8170	4.47
2	10.901	3565018	147644	95.53

Supplementary Figure 41. Chiral HPLC analysis of chiral **20**

***tert*-Butyl (2*R*,3*R*)-2-((4-bromobenzyl)oxy)-2-(4-fluorophenyl)-3-(2-methyl-1*H*-indol-3-yl)-5-phenylpent-4-ynoate (21)**

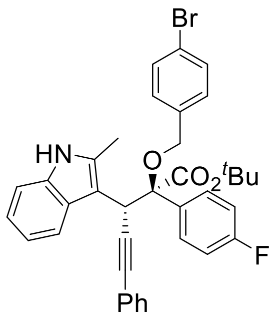

21

White solid, mp: 135.5 – 136.4 °C; 89.2 mg, 70% yield, >20:1 d.r., 93% *ee*, $[\alpha]_D^{20}$ = 12.38 (c = 0.033, MeOH); ^{1}H NMR (500 MHz, $CDCl_3$) δ 7.84 (s, 1H), 7.66 (s, 1H), 7.43 (s, 2H), 7.35 (s, 3H), 7.28 (s, 4H), 7.17 (d, *J* = 4.2 Hz, 1H), 7.06 (s, 3H), 6.91 (d, *J* = 2.5 Hz, 1H), 6.78 (s, 2H), 4.79 (d, *J* = 12.7 Hz, 1H), 4.72 (s, 1H), 4.24 (d, *J* = 9.7 Hz, 1H), 1.66 (s, 3H), 1.53 (s, 9H). ^{13}C NMR (126 MHz, $CDCl_3$) δ 170.8, δ 162.6 (d, *J* = 247.0 Hz), 138.9, 135.0, 134.3, 132.0, 131.6, 131.0, 129.8 (d, *J* = 8.8 Hz), 128.7, 128.6, 128.3, 127.8, 124.0, 120.9, 120.4, 119.2, 114.2 (d, *J* = 20.2 Hz), 109.9, 105.8, 89.2, 89.1, 83.4, 83.2, 67.7, 41.6, 28.2, 11.3. ^{19}F NMR (376 MHz, $CDCl_3$) δ -114.12. HRMS(ESI) $[M+Na]^+$ calcd for $C_{37}H_{33}BrFNO_3Na^+$, 660.1520, found 660.1522. (Chiral IA, λ = 254 nm, *n*-hexane/2-propanol = 95/5, Flow rate = 1.0 mL/min), t_R = 7.466 min, 8.638 min (major).

HPLC chromatogram of racemic 21

Condition: n-hexane/2-propanol = 95:5

Flow rate = 1.0 mL/min

λ = 254 nm

Chiral IA

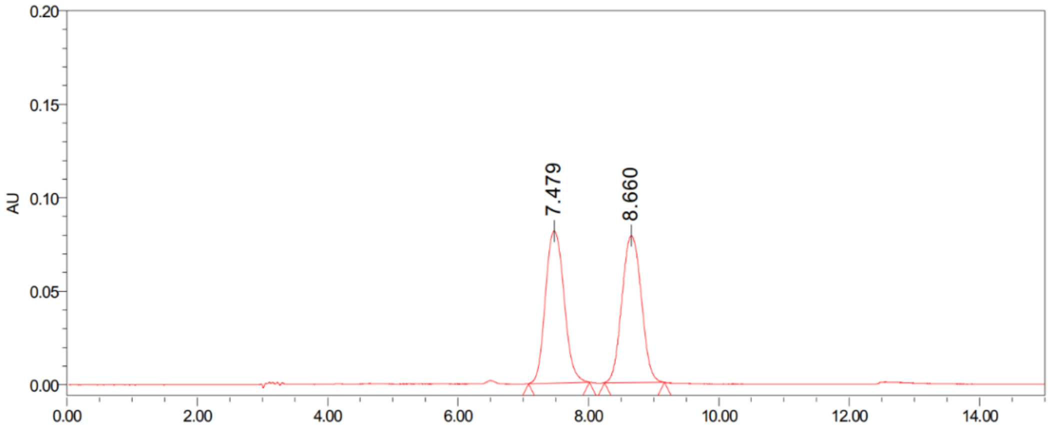

Entry	Retention Time/min	Area	Height	Area(%)
1	7.479	1614729	81105	50.01
2	8.660	1613921	78487	49.99

Supplementary Figure 42. Chiral HPLC analysis of racemic **21**

HPLC chromatogram of chiral 21

Condition: n-hexane/2-propanol = 95:5

Flow rate = 1.0 mL/min

λ = 254 nm

Chiral IA

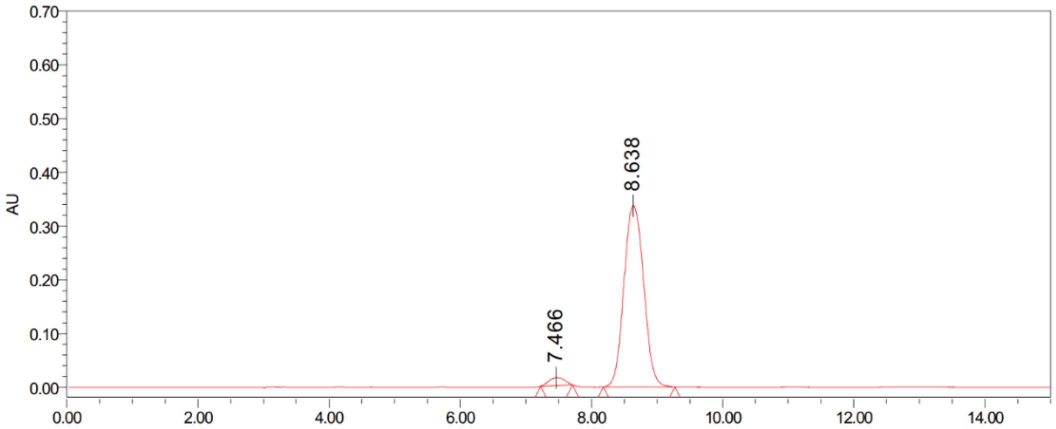

Entry	Retention Time/min	Area	Height	Area(%)
1	7.466	251734	14895	3.47
2	8.638	7001045	336533	96.53

Supplementary Figure 43. Chiral HPLC analysis of chiral **21**

tert-Butyl (2_R_,3_R_)-2-((4-bromobenzyl)oxy)-3-(2-ethyl-1_H_-indol-3-yl)-2,5-diphenylpent-4-ynoate (22)

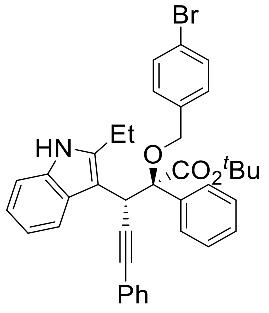

22

Yellow oil; 88.6 mg, 70% yield, >20:1 d.r., 95% *ee*, $[\alpha]_D^{20}$ = 6.33 (c = 0.033, MeOH); ^{1}H NMR (500 MHz, $CDCl_3$) δ 7.89 (s, 1H), 7.72 (s, 1H), 7.47 – 7.41 (m, 2H), 7.35 (d, *J* = 8.3 Hz, 2H), 7.32 – 7.26 (m, 5H), 7.22 – 7.15 (m, 2H), 7.10 – 7.01 (m, 5H), 6.90 (t, *J* = 7.4 Hz, 1H), 4.78 (d, *J* = 14.6 Hz, 2H), 4.29 (d, *J* = 13.0 Hz, 1H), 2.24 – 2.13 (m, 1H), 1.98 – 1.79 (m, 1H), 1.53 (s, 9H), 0.85 (t, *J* = 7.5 Hz, 3H). ^{13}C NMR (126 MHz, $CDCl_3$) δ 171.0, 139.5, 139.3, 136.1, 135.1, 131.7, 131.0, 128.7, 128.6, 128.3, 128.0, 127.9, 127.7, 127.5, 124.1, 120.8, 120.3, 119.2, 109.9, 105.3, 89.8, 89.7, 82.99, 82.97, 67.7, 41.3, 28.2, 18.5, 12.9. HRMS(ESI) $[M+Na]^+$ calcd for $C_{38}H_{36}BrNO_3Na^+$, 656.1771, found 656.1771. (Chiral IA, λ = 254 nm, *n*-hexane/2-propanol = 95/5, Flow rate = 1.0 mL/min), t_R = 6.171 min, 6.733 min (major).

HPLC chromatogram of racemic 22

Condition: n-hexane/2-propanol = 95:5

Flow rate = 1.0 mL/min

λ = 254 nm

Chiral IA

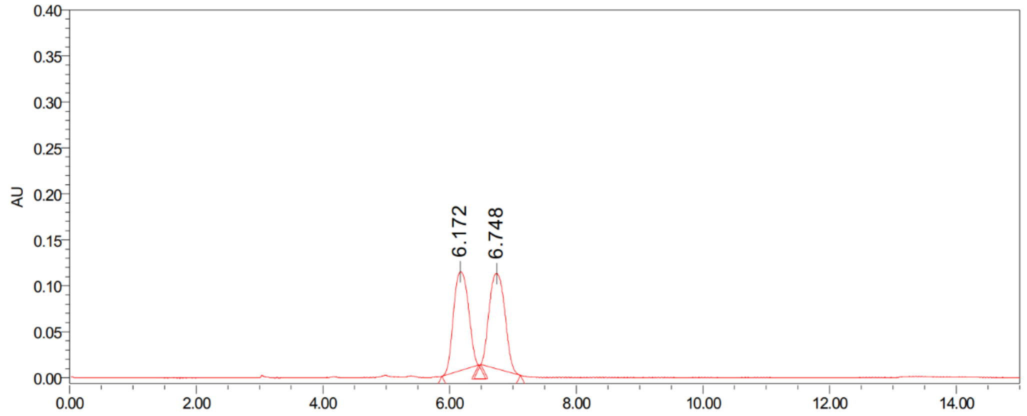

Entry	Retention Time/min	Area	Height	Area(%)
1	6.172	1765567	107679	49.96
2	6.748	1768302	103663	50.04

Supplementary Figure 44. Chiral HPLC analysis of racemic **22**

HPLC chromatogram of chiral 22

Condition: n-hexane/2-propanol = 95:5

Flow rate = 1.0 mL/min

λ = 254 nm

Chiral IA

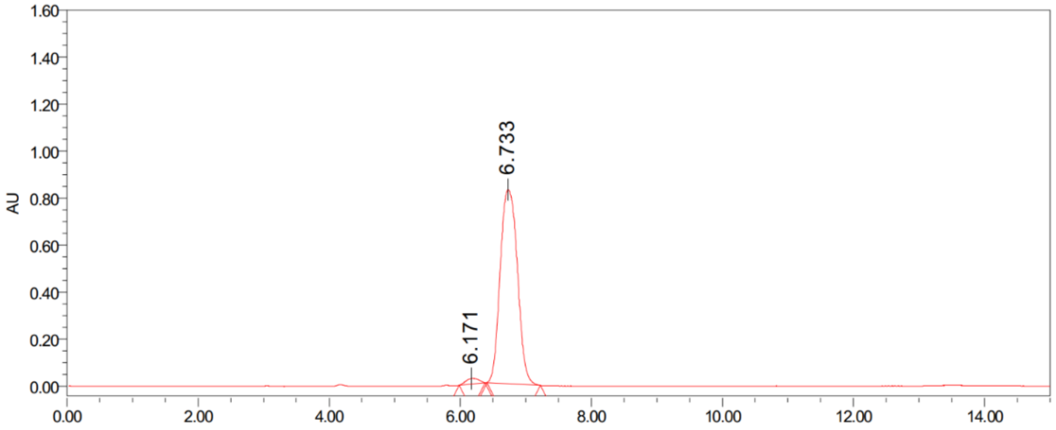

Entry	Retention Time/min	Area	Height	Area(%)
1	6.171	309901	22565	2.48
2	6.733	14923309	825029	97.52

Supplementary Figure 45. Chiral HPLC analysis of chiral **22**

***tert*-Butyl (2*R*,3*R*)-2-((4-bromobenzyl)oxy)-3-(5-fluoro-2-methyl-1*H*-indol-3-yl)-2,5-diphenylpent-4-ynoate (23)**

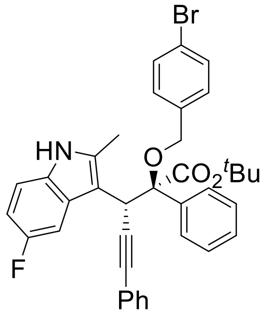

23

White solid, mp: 133.3 – 134.1 °C; 94.3 mg, 74% yield, >20:1 d.r., 87% *ee*, $[\alpha]_D^{20}$ = -6.25 (c = 0.033, MeOH); ^{1}H NMR (500 MHz, $CDCl_3$) δ 7.57 (s, 1H), 7.45 (d, *J* = 7.3 Hz, 1H), 7.35 (dd, *J* = 7.2, 2.3 Hz, 2H), 7.28 (s, 1H), 7.26 (s, 1H), 7.23 – 7.19 (m, 3H), 7.17 (d, *J* = 8.3 Hz, 2H), 7.15 – 7.10 (m, 1H), 7.04 – 6.98 (m, 4H), 6.95 (dd, *J* = 8.7, 4.4 Hz, 1H), 6.67 (td, *J* = 9.0, 2.5 Hz, 1H), 4.75 (d, *J* = 12.8 Hz, 1H), 4.60 (s, 1H), 4.21 (d, *J* = 12.8 Hz, 1H), 1.47 (s, 3H), 1.45 (s, 9H). ^{13}C NMR (126 MHz, $CDCl_3$) δ 170.8, δ 157.5 (d, *J* = 233.1 Hz), 139.0, 136.3, 136.0, 131.6, 131.4, 131.0, 129.4 (d, *J* = 10.1 Hz), 128.6, 128.3, 128.1, 127.9, 127.8, 127.5, 123.8, 120.5, 110.2 (d, *J* = 8.8 Hz), 108.9 (d, *J* = 26.5 Hz), 106.3 (d, *J* = 5.0 Hz), 89.6, 89.1, 83.4, 83.1, 67.9, 41.5, 28.2, 11.3. ^{19}F NMR (376 MHz, $CDCl_3$) δ -125.10. HRMS(ESI) $[M+Na]^+$ calcd for $C_{37}H_{33}BrFNO_3Na^+$, 660.1520, found 660.1519. (Chiral IF-3, λ = 254 nm, *n*-hexane/2-propanol = 95/5, Flow rate = 1.0 mL/min), t_R = 6.931 min, 8.017 min (major).

HPLC chromatogram of racemic 23

Condition: n-hexane/2-propanol = 95:5

Flow rate = 1.0 mL/min

λ = 254 nm

Chiral IF-3

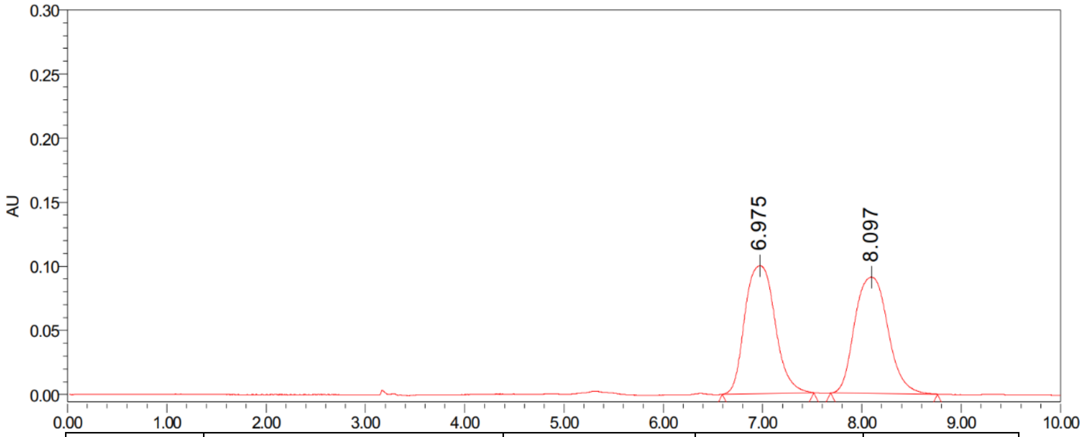

Entry	Retention Time/min	Area	Height	Area(%)
1	6.975	2111947	99711	50.04
2	8.097	2108204	90715	49.96

Supplementary Figure 46. Chiral HPLC analysis of racemic **23**

HPLC chromatogram of chiral 23

Condition: n-hexane/2-propanol = 95:5

Flow rate = 1.0 mL/min

λ = 254 nm

Chiral IF-3

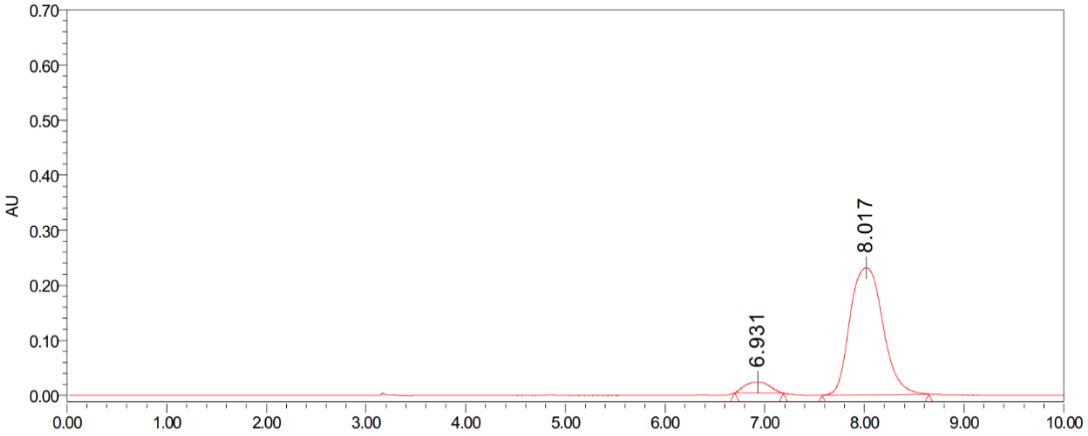

The larger version of HPLC chromatogram of chiral 23

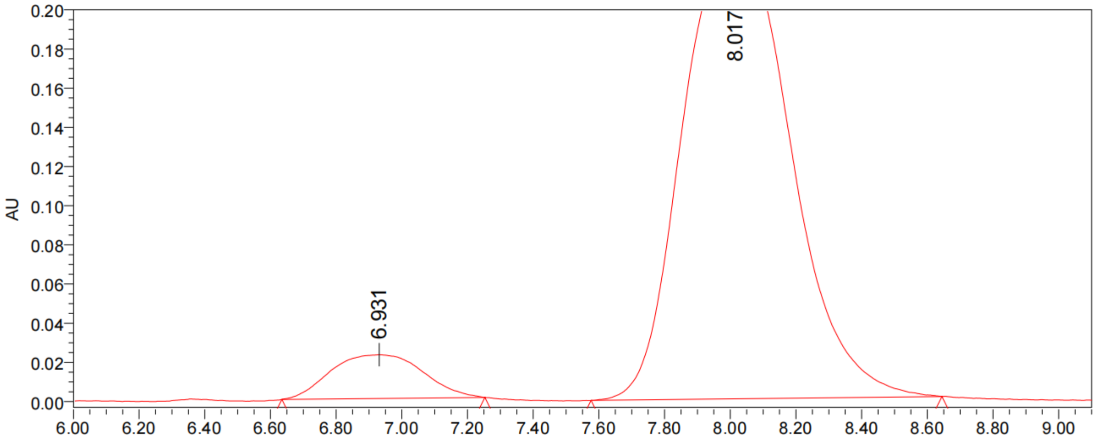

Entry	Retention Time/min	Area	Height	Area(%)
1	6.931	435765	22359	6.52
2	8.017	5270484	229930	93.48

Supplementary Figure 47. Chiral HPLC analysis of chiral **23**

***tert*-Butyl (2*R*,3*R*)-2-((4-bromobenzyl)oxy)-3-(5-isopropyl-2-methyl-1*H*-indol-3-yl)-2,5-diphenylpent-4-ynoate (24):**

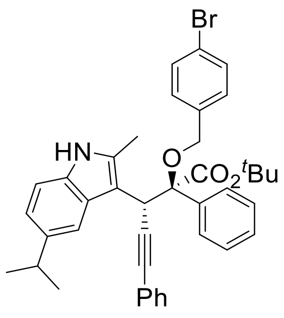

24

Yellow oil; 117.7 mg, 89% yield, >20:1 d.r., 97% *ee*, $[\alpha]_D^{20}$ = -21.34 (c = 0.033, MeOH); ^{1}H NMR (400 MHz, $CDCl_3$) δ 7.74 (s, 1H), 7.55 (s, 1H), 7.47 (d, *J* = 4.7 Hz, 2H), 7.34 (s, 4H), 7.29 (d, *J* = 4.7 Hz, 3H), 7.20 (d, *J* = 4.8 Hz, 1H), 7.13 – 7.05 (m, 5H), 6.91 (d, *J* = 8.1 Hz, 1H), 4.78 (d, *J* = 12.7 Hz, 1H), 4.70 (s, 1H), 4.27 (d, *J* = 12.7 Hz, 1H), 2.75 (dt, *J* = 13.2, 6.5 Hz, 1H), 1.55 (s, 12H), 1.08 (d, *J* = 6.7 Hz, 3H), 1.02 (d, *J* = 6.7 Hz, 3H). ^{13}C NMR (126 MHz, $CDCl_3$) δ 171.0, 139.5, 139.4, 136.0, 134.5, 133.6, 131.6, 131.0, 129.0, 128.6, 128.2, 128.00, 127.98, 127.7, 127.4, 124.2, 120.3, 120.0, 109.4, 105.7, 89.8, 89.6, 83.2, 83.0, 67.6, 41.7, 34.2, 28.3, 24.51, 24.46, 11.2. HRMS(ESI) $[M+Na]^+$ calcd for $C_{40}H_{40}BrNO_3Na^+$, 684.2084, found 684.2083. (Chiral IA, λ = 254 nm, *n*-hexane/2-propanol = 95/5, Flow rate = 1.0 mL/min), t_R = 6.140 min (major), 6.886 min.

HPLC chromatogram of racemic 24

Condition: n-hexane/2-propanol = 95:5

Flow rate = 1.0 mL/min

λ = 254 nm

Chiral IA

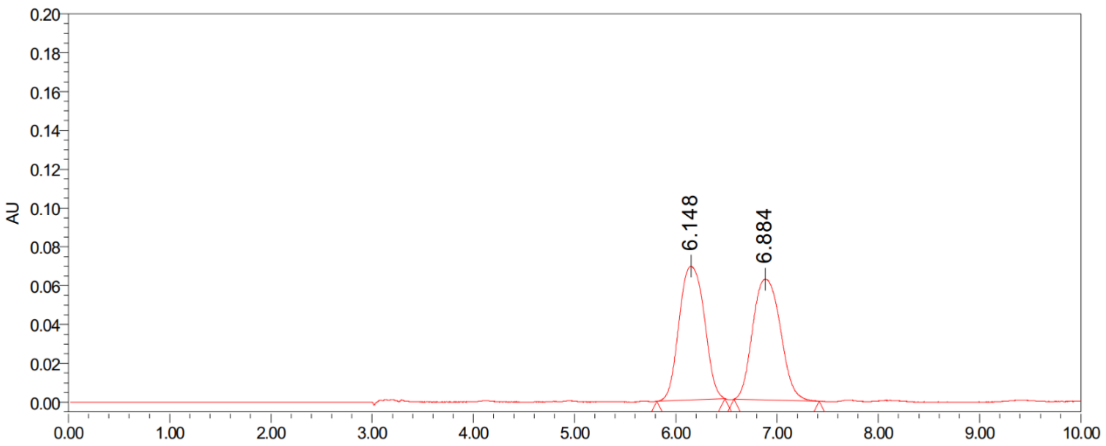

Entry	Retention Time/min	Area	Height	Area(%)
1	6.148	1195845	68821	50.09
2	6.884	1191637	62268	49.91

Supplementary Figure 48. Chiral HPLC analysis of racemic **24**

HPLC chromatogram of chiral 24

Condition: n-hexane/2-propanol = 95:5

Flow rate = 1.0 mL/min

λ = 254 nm

Chiral IA

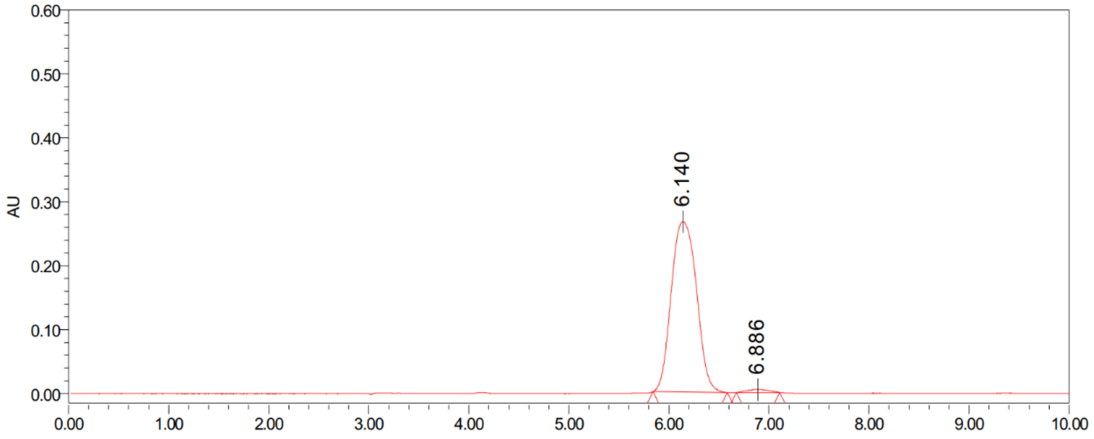

The larger version of HPLC chromatogram of chiral 24

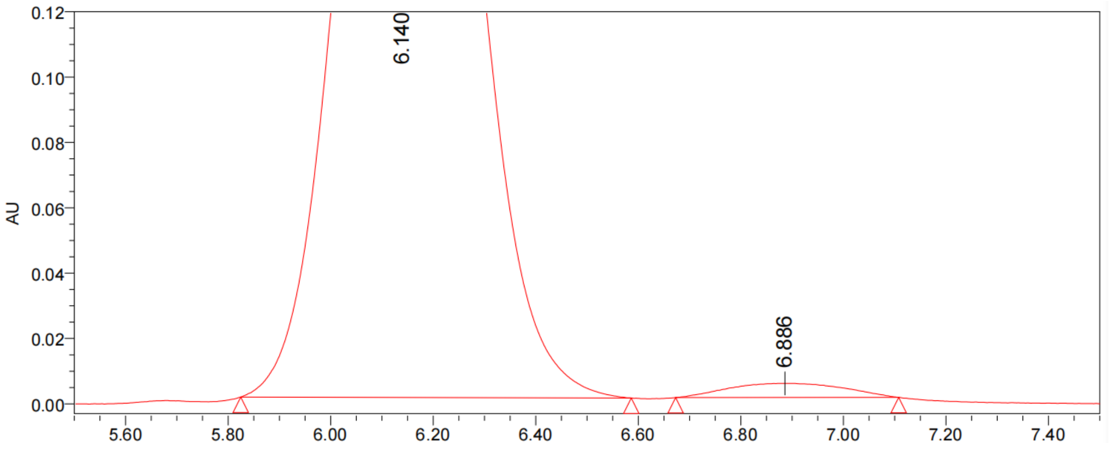

Entry	Retention Time/min	Area	Height	Area(%)
1	6.140	4677048	266246	98.50
2	6.886	68585	4332	1.50

Supplementary Figure 49. Chiral HPLC analysis of chiral **24**

***tert*-Butyl (2*R*,3*R*)-2-((4-bromobenzyl)oxy)-3-(2-methyl-5-phenyl-1*H*-indol-3-yl)-2,5-diphenylpent-4-ynoate (25)**

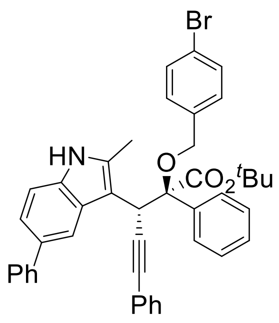

25

Yellow oil; 86.2 mg, 62% yield, >20:1 d.r., 92% *ee*, $[\alpha]_D^{20}$ = -96.31 (c = 0.033, MeOH); ^{1}H NMR (400 MHz, $CDCl_3$) δ 8.21 (s, 1H), 7.55 (s, 1H), 7.34 – 7.29 (m, 2H), 7.21 (t, *J* = 8.5 Hz, 6H), 7.18 – 7.08 (m, 10H), 7.04 (d, *J* = 3.8 Hz, 4H), 4.70 (d, *J* = 12.5 Hz, 1H), 4.62 (s, 1H), 4.18 (d, *J* = 12.5 Hz, 1H), 1.48 (s, 9H), 1.42 (s, 3H). ^{13}C NMR (101 MHz, $CDCl_3$) δ 171.0, 142.6, 139.0, 135.9, 135.0, 134.7, 132.4, 131.7, 131.0, 129.3, 128.8, 128.5, 128.2, 128.1, 128.0, 127.8, 127.5, 127.1, 126.0, 123.9, 120.5, 120.4, 110.0, 106.4, 89.7, 89.6, 83.5, 83.1, 67.7, 41.9, 28.3, 11.0. HRMS(ESI) $[M+Na]^+$ calcd for $C_{43}H_{38}BrNO_3Na^+$, 718.1927, found 718.1929. (Chiral IA, λ = 254 nm, *n*-hexane/2-propanol = 95/5, Flow rate = 1.0 mL/min), t_R = 12.060 min, 20.226 min (major).

HPLC chromatogram of racemic 25

Condition: n-hexane/2-propanol = 95:5

Flow rate = 1.0 mL/min

λ = 254 nm

Chiral IA

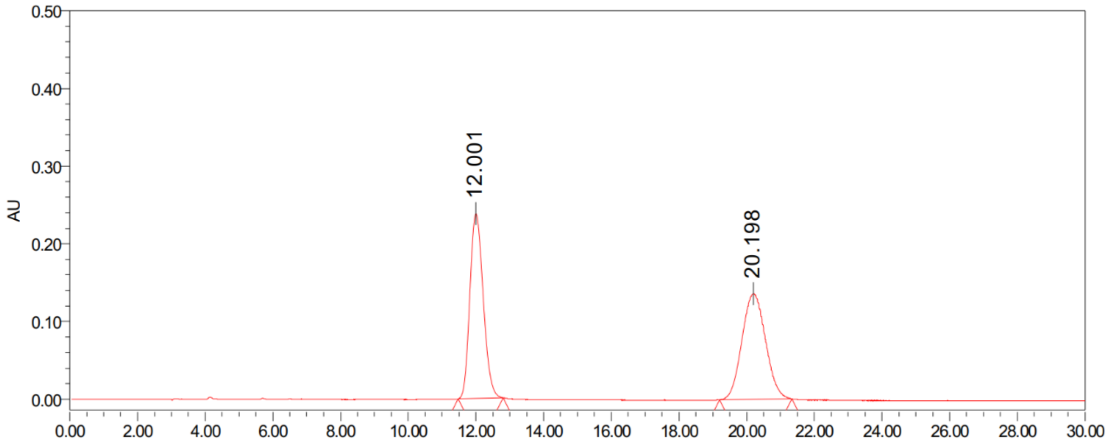

Entry	Retention Time/min	Area	Height	Area(%)
1	12.001	6528807	237595	50.03
2	20.198	6521436	135809	49.97

Supplementary Figure 50. Chiral HPLC analysis of racemic **25**

HPLC chromatogram of chiral 25

Condition: n-hexane/2-propanol = 95:5

Flow rate = 1.0 mL/min

λ = 254 nm

Chiral IA

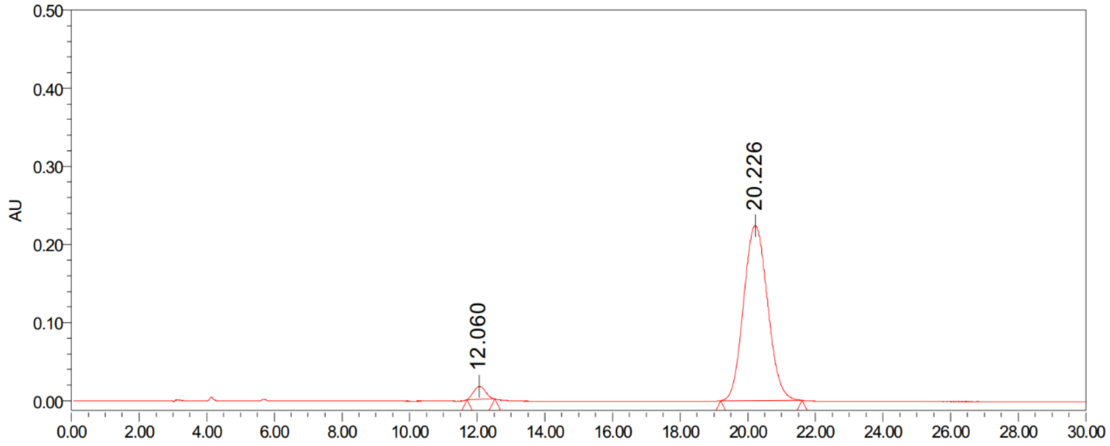

The larger version of HPLC chromatogram of chiral 25

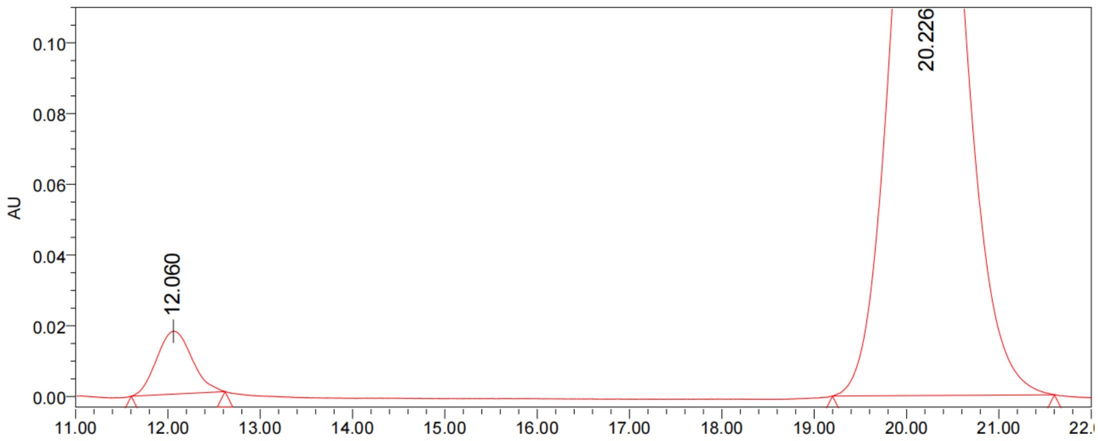

Entry	Retention Time/min	Area	Height	Area(%)
1	12.060	399221	16434	3.61
2	20.226	10971568	223996	96.39

Supplementary Figure 51. Chiral HPLC analysis of chiral **25**

***tert*-Butyl (2*R*,3*R*)-2-((4-bromobenzyl)oxy)-3-(2-methyl-5-(4,4,5,5-tetramethyl-1,3,2-dioxaborolan-2-yl)-1*H*-indol-3-yl)-2,5-diphenylpent-4-ynoate (26)**

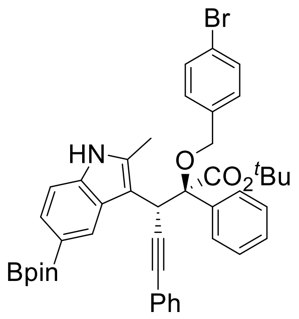

26

Yellow oil; 89.4 mg, 60% yield, >20:1 d.r., 82% *ee*, $[\alpha]_D^{20}$ = -48.49 (c = 0.033, MeOH); ^{1}H NMR (400 MHz, $CDCl_3$) δ 8.74 (s, 1H), 7.73 (s, 1H), 7.65 – 7.60 (m, 2H), 7.51 (d, *J* = 8.1 Hz, 1H), 7.35 – 7.28 (m, 7H), 7.25 – 7.18 (m, 1H), 7.15 (d, *J* = 8.1 Hz, 1H), 7.09 (d, *J* = 4.3 Hz, 4H), 4.76 (d, *J* = 13.4 Hz, 1H), 4.72 (s, 1H), 4.33 (d, *J* = 13.4 Hz, 1H), 1.51 (s, 9H), 1.47 (s, 3H), 1.32 (s, 6H), 1.26 (s, 6H). ^{13}C NMR (126 MHz, $CDCl_3$) δ 171.0, 139.4, 137.2, 136.2, 134.3, 131.9, 130.8, 128.5, 128.3, 128.1, 128.0, 127.9, 127.7, 127.4, 127.0, 124.2, 120.0, 109.3, 106.3, 89.7, 89.6, 83.6, 83.1, 83.0, 67.7, 41.7, 28.2, 25.0, 24.8, 10.9. HRMS(ESI) $[M+Na]^+$ calcd for $C_{43}H_{45}BBrNO_5Na^+$, 768.2474, found 768.2475. (Chiral IA, λ = 254 nm, *n*-hexane/2-propanol = 95/5, Flow rate = 1.0 mL/min), t_R = 5.973 min (major), 7.064 min.

HPLC chromatogram of racemic 26

Condition: n-hexane/2-propanol = 95:5

Flow rate = 1.0 mL/min

λ = 254 nm

Chiral IA

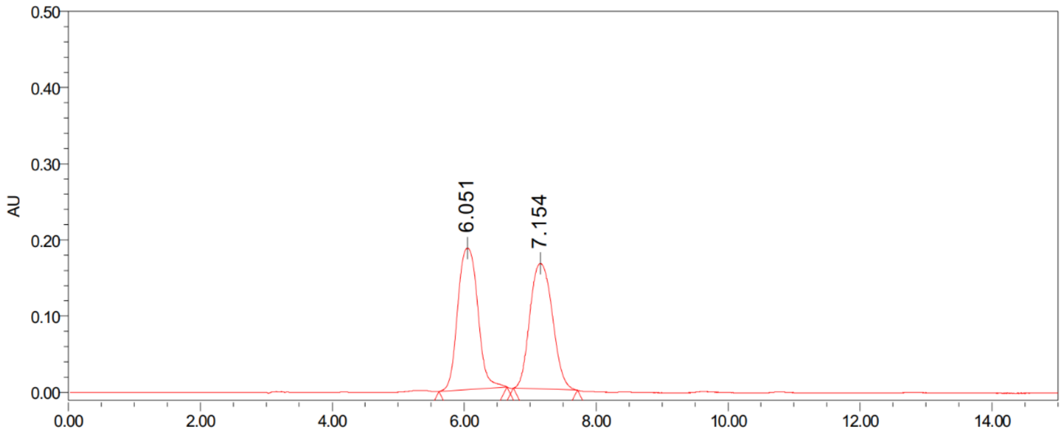

Entry	Retention Time/min	Area	Height	Area(%)
1	6.051	3938892	185725	50.03
2	7.154	3934803	164784	49.97

Supplementary Figure 52. Chiral HPLC analysis of racemic **26**

HPLC chromatogram of chiral 26

Condition: n-hexane/2-propanol = 95:5

Flow rate = 1.0 mL/min

λ = 254 nm

Chiral IA

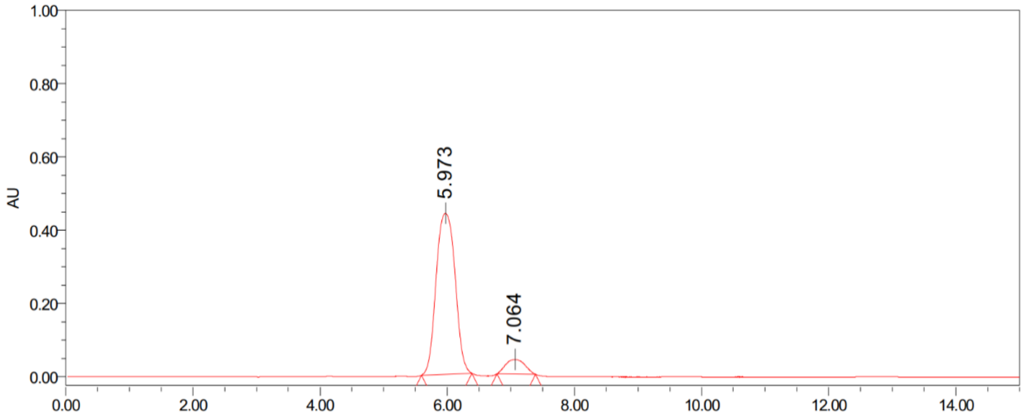

Entry	Retention Time/min	Area	Height	Area(%)
1	5.973	9118990	438707	91.31
2	7.064	845605	39943	8.69

Supplementary Figure 53. Chiral HPLC analysis of chiral **26**

tert-Butyl (2*R*,3*R*)-2-((4-bromobenzyl)oxy)-3-(2,6-dimethyl-1*H*-indol-3-yl)-2,5-diphenylpent-4-ynoate (27)

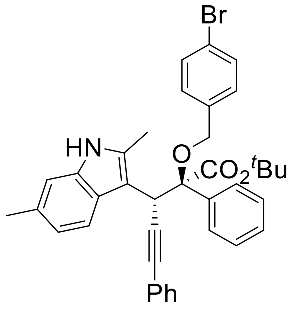

27

Yellow oil; 44.3 mg, 35% yield, >20:1 d.r., 93% *ee*, $[\alpha]_D^{20}$ = -3.18 (c = 0.033, MeOH); ^{1}H NMR (500 MHz, $CDCl_3$) δ 7.82 (d, *J* = 6.3 Hz, 1H), 7.52 (s, 1H), 7.45 (dd, *J* = 7.3, 2.0 Hz, 2H), 7.35 (d, *J* = 8.4 Hz, 2H), 7.32 – 7.28 (m, 5H), 7.22 (t, *J* = 6.8 Hz, 1H), 7.14 – 7.08 (m, 4H), 6.99 (s, 1H), 6.76 (d, *J* = 8.1 Hz, 1H), 4.79 (d, *J* = 13.0 Hz, 1H), 4.70 (s, 1H), 4.30 (d, *J* = 13.0 Hz, 1H), 2.41 (s, 3H), 1.53 (s, 12H). ^{13}C NMR (126 MHz, $CDCl_3$) δ 170.9, 139.3, 136.2, 135.5, 133.5, 131.6, 130.9, 130.4, 128.5, 128.20, 127.93, 127.89, 127.7, 127.4, 126.7, 124.1, 120.8, 120.2, 109.7, 105.8, 89.7, 89.6, 83.0, 82.9, 67.7, 41.6, 28.2, 21.7, 11.0. HRMS(ESI) $[M+Na]^+$ calcd for $C_{38}H_{36}BrNO_3Na^+$, 656.1771, found 656.1770. (Chiral IA, λ = 254 nm, *n*-hexane/2-propanol = 95/5, Flow rate = 1.0 mL/min), t_R =10.696 min, 11.494 min (major).

HPLC chromatogram of racemic 27

Condition: n-hexane/2-propanol = 95:5

Flow rate = 1.0 mL/min

λ = 254 nm

Chiral IA

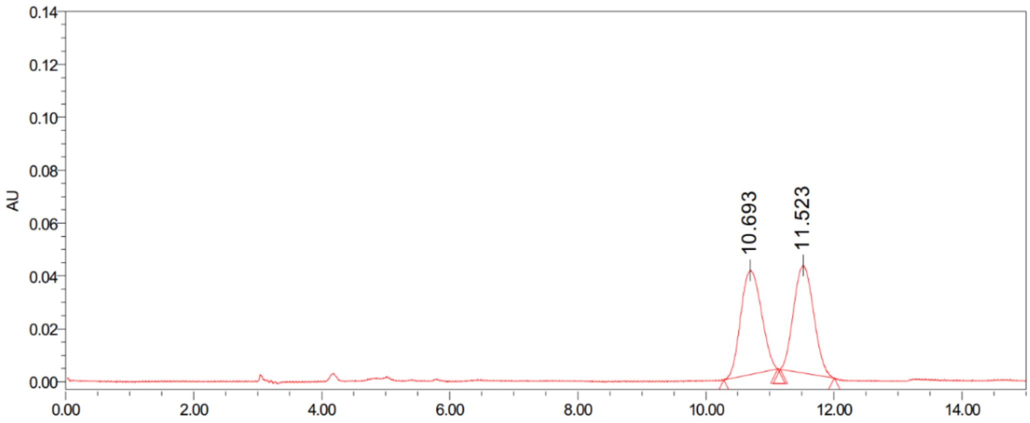

Entry	Retention Time/min	Area	Height	Area(%)
1	10.693	882614	39485	49.99
2	11.523	882923	40622	50.01

Supplementary Figure 54. Chiral HPLC analysis of racemic **27**

HPLC chromatogram of chiral 27

Condition: n-hexane/2-propanol = 95:5

Flow rate = 1.0 mL/min

λ = 254 nm

Chiral IA

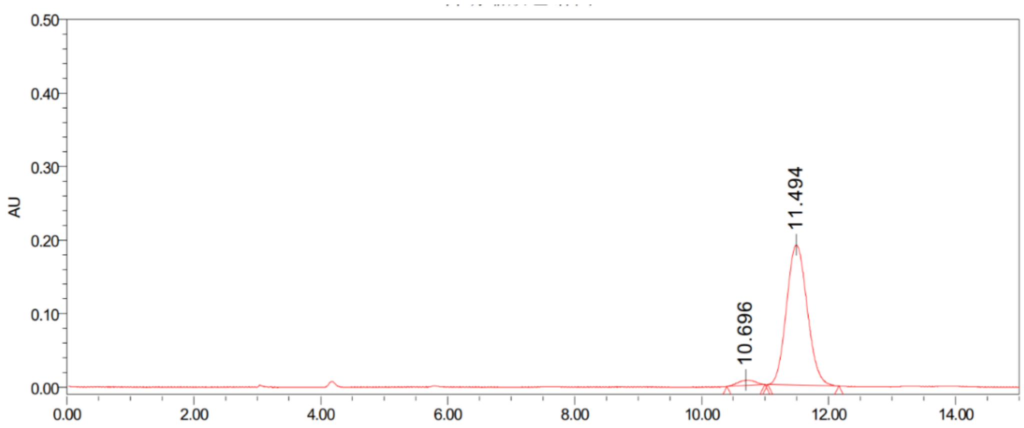

Entry	Retention Time/min	Area	Height	Area(%)
1	10.696	135962	7299	3.52
2	11.494	4328022	190371	96.48

Supplementary Figure 55. Chiral HPLC analysis of chiral **27**

tert-Butyl (2*R*,3*R*)-2-((4-bromobenzyl)oxy)-3-(2-methyl-5-(methyl(phenyl)amino)-1*H*-indol-3-yl)-2,5-diphenylpent-4-ynoate (28)

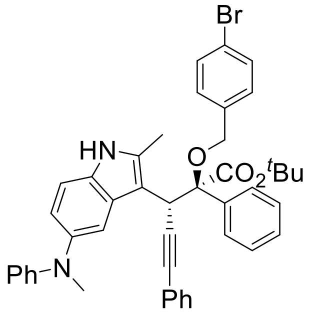

28

Yellow oil; 113.0 mg, 78% yield, >20:1 d.r., 97% *ee*, $[\alpha]_D^{20}$ = 96.58 (c = 0.033, MeOH); ^{1}H NMR (400 MHz, $CDCl_3$) δ 7.65 (s, 1H), 7.30 (d, *J* = 7.1 Hz, 2H), 7.25 (s, 4H), 7.20 (dt, *J* = 14.2, 7.0 Hz, 5H), 7.12 (d, *J* = 5.8 Hz, 5H), 7.06 (t, *J* = 7.8 Hz, 2H), 6.84 (d, *J* = 8.2 Hz, 1H), 6.65 (t, *J* = 7.8 Hz, 3H), 4.78 (d, *J* = 12.7 Hz, 1H), 4.69 (s, 1H), 4.27 (d, *J* = 12.7 Hz, 1H), 2.94 (s, 3H), 1.65 (s, 3H), 1.52 (s, 9H). ^{13}C NMR (126 MHz, $CDCl_3$) δ 170.9, 150.6, 141.1, 139.1, 136.0, 135.3, 132.7, 131.6, 131.1, 123.0, 128.7, 128.6, 128.3, 128.1, 127.9, 127.7, 127.5, 123.8, 120.4, 117.0, 114.5, 110.5, 106.2, 89.43, 89.38, 83.3, 83.1, 67.7, 41.6, 40.5, 28.2, 11.4. HRMS(ESI) $[M+Na]^+$ calcd for $C_{44}H_{41}BrN_2O_3Na^+$, 747.2193, found 747.2195. (Chiral IA, λ = 254 nm, *n*-hexane/2-propanol = 95/5, Flow rate = 1.0 mL/min), t_R = 8.621 min (major), 27.352 min.

HPLC chromatogram of racemic 28

Condition: n-hexane/2-propanol = 95:5

Flow rate = 1.0 mL/min

λ = 254 nm

Chiral IA

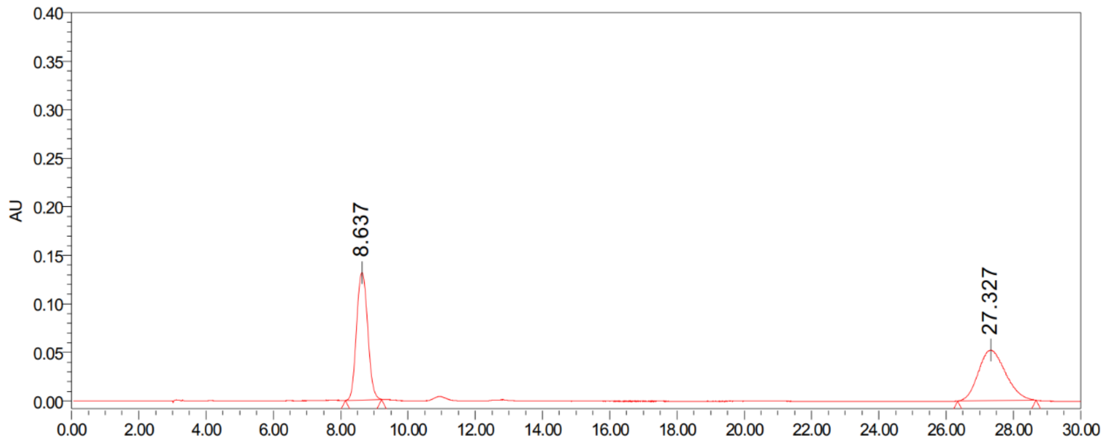

Entry	Retention Time/min	Area	Height	Area(%)
1	8.637	3023567	130993	50.93
2	27.327	2912808	52055	49.07

Supplementary Figure 56. Chiral HPLC analysis of racemic **28**

HPLC chromatogram of chiral 28

Condition: n-hexane/2-propanol = 95:5

Flow rate = 1.0 mL/min

λ = 254 nm

Chiral IA

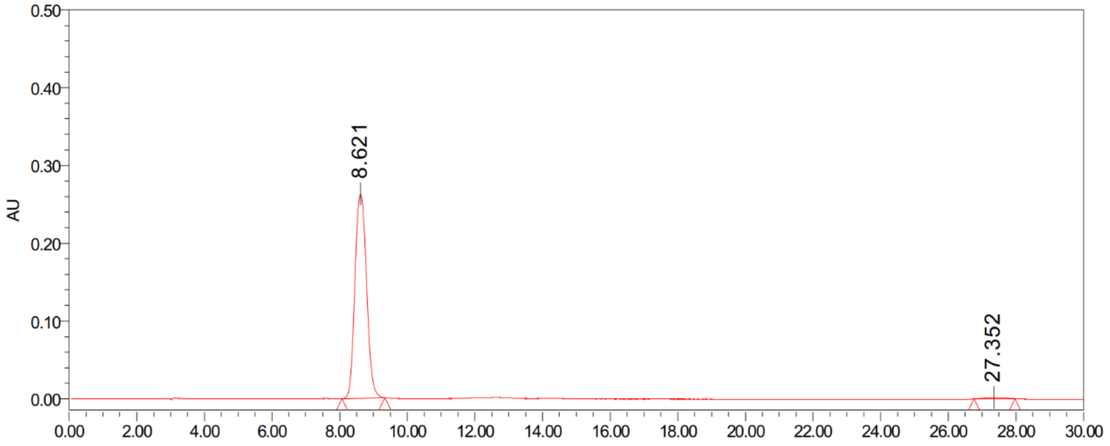

The larger version of HPLC chromatogram of chiral 28

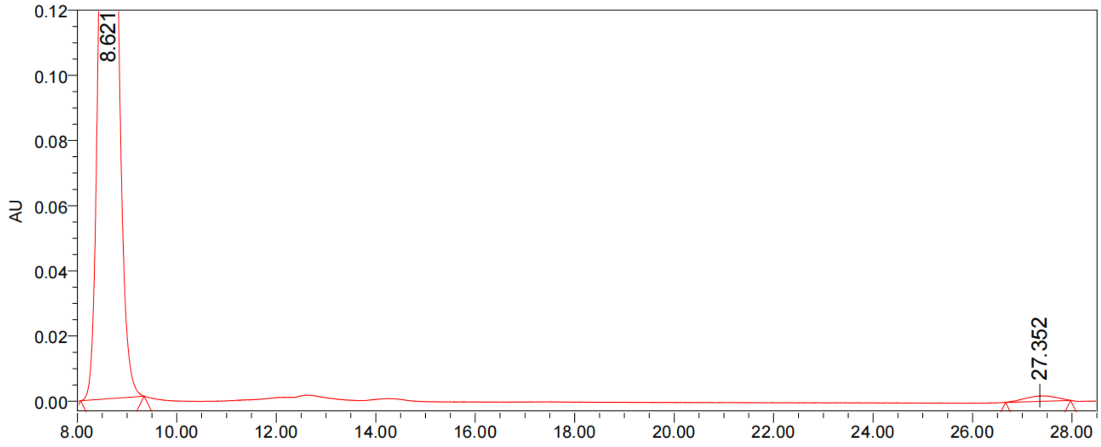

Entry	Retention Time/min	Area	Height	Area(%)
1	8.621	6282115	262286	98.50
2	27.352	73288	1726	1.50

Supplementary Figure 57. Chiral HPLC analysis of chiral **28**

***tert*-Butyl (2*R*,3*R*)-2-((4-bromobenzyl)oxy)-3-(2-methyl-5-(*p*-tolylt*hi*o)-1*H*-indol-3-yl)-2,5-diphenylpent-4-ynoate (29)**

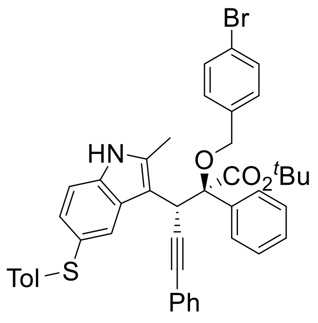

29

Yellow oil; 56.3 mg, 38% yield, >20:1 d.r., 86% *ee*, $[\alpha]_D^{20}$ = 75.26 (c = 0.033, MeOH); ^{1}H NMR (400 MHz, $CDCl_3$) δ 8.14 (s, 1H), 7.77 (s, 1H), 7.37 – 7.33 (m, 2H), 7.30 (d, *J* = 8.4 Hz, 2H), 7.26 – 7.15 (m, 6H), 7.14 (d, *J* = 1.5 Hz, 2H), 7.10 (d, *J* = 4.3 Hz, 4H), 6.96 (d, *J* = 8.2 Hz, 2H), 6.87 (d, *J* = 8.0 Hz, 2H), 4.84 (d, *J* = 12.8 Hz, 1H), 4.75 (s, 1H), 4.28 (d, *J* = 12.8 Hz, 1H), 2.22 (s, 3H), 1.63 (s, 3H), 1.53 (s, 9H). ^{13}C NMR (126 MHz, $CDCl_3$) δ 170.8, 138.8, 136.4, 136.1, 135.4, 134.8, 134.7, 131.6, 131.0, 129.8, 129.4, 128.5, 128.2, 128.1, 127.8, 127.74, 127.71, 127.5, 127.1, 123.7, 122.5, 120.4, 110.9, 106.3, 89.5, 89.2, 83.4, 83.1, 67.9, 41.5, 28.2, 21.0, 11.3. HRMS(ESI) $[M+Na]^+$ calcd for $C_{44}H_{40}BrNO_3SNa^+$, 764.1804, found 764.1803. (Chiral IA, λ = 254 nm, *n*-hexane/2-propanol = 95/5, Flow rate = 1.0 mL/min), t_R = 9.819 min (major), 12.248 min.

HPLC chromatogram of racemic 29

Condition: n-hexane/2-propanol = 95:5

Flow rate = 1.0 mL/min

λ = 254 nm

Chiral IA

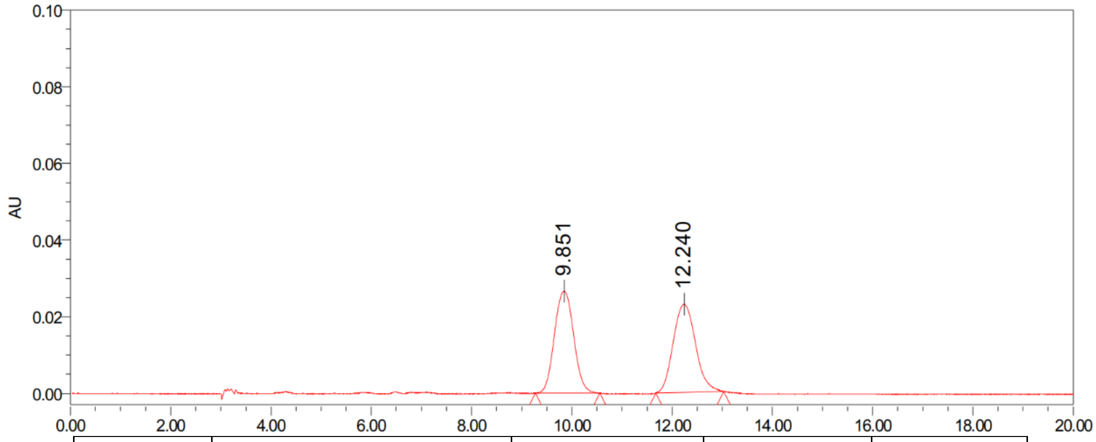

Entry	Retention Time/min	Area	Height	Area(%)
1	9.851	712557	26653	50.01
2	12.240	712298	23027	49.99

Supplementary Figure 58. Chiral HPLC analysis of racemic **29**

HPLC chromatogram of chiral 29

Condition: n-hexane/2-propanol = 95:5

Flow rate = 1.0 mL/min

λ = 254 nm

Chiral IA

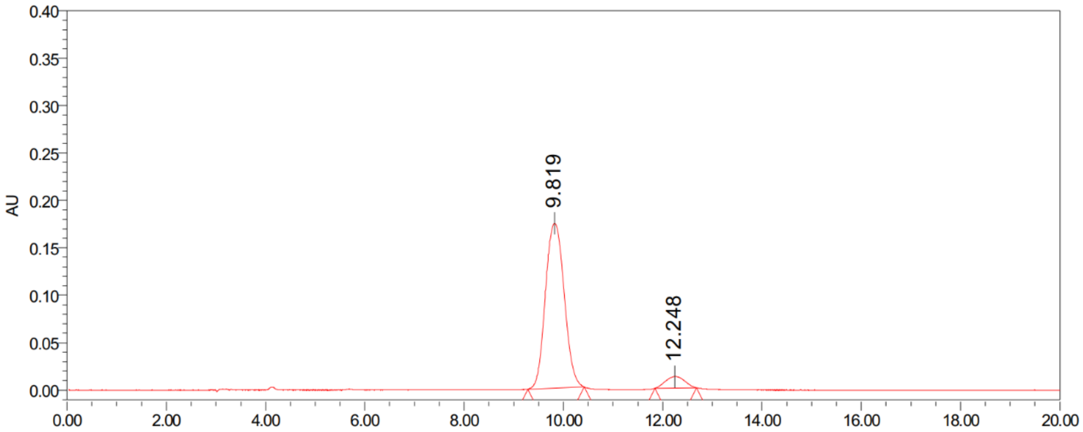

Entry	Retention Time/min	Area	Height	Area(%)
1	9.819	4579504	173870	93.44
2	12.248	321292	12113	6.56

Supplementary Figure 59. Chiral HPLC analysis of chiral **29**

***tert*-Butyl (2*R*,3*R*)-2-((4-bromobenzyl)oxy)-5-(4-chlorophenyl)-3-(2-methyl-1*H*-indol-3-yl)-2-phenylpent-4-ynoate (30)**

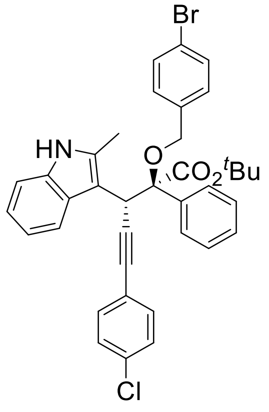

30

Yellow oil; 98.0 mg, 75% yield, >20:1 d.r., 92% *ee*, $[\alpha]_D^{20}$ = 18.39 (c = 0.033, MeOH); ^{1}H NMR (500 MHz, $CDCl_3$) δ 7.87 (s, 1H), 7.66 (s, 1H), 7.36 – 7.32 (m, 4H), 7.25 (t, *J* = 8.2 Hz, 4H), 7.22 – 7.18 (m, 1H), 7.17 (d, *J* = 8.0 Hz, 1H), 7.07 (td, *J* = 15.3, 7.8 Hz, 5H), 6.91 (t, *J* = 7.5 Hz, 1H), 4.81 (d, *J* = 12.9 Hz, 1H), 4.72 (s, 1H), 4.28 (d, *J* = 12.9 Hz, 1H), 1.56 (s, 3H), 1.53 (s, 9H). ^{13}C NMR (126 MHz, $CDCl_3$) δ 170.9, 139.2, 136.0, 135.0, 134.4, 133.7, 132.9, 131.0, 128.8, 128.61, 128.55, 128.1, 127.9, 127.5, 122.5, 120.9, 120.4, 119.2, 109.9, 105.7, 90.8, 89.6, 83.1, 82.0, 67.8, 41.6, 28.2, 11.1. HRMS(ESI) $[M+Na]^+$ calcd for $C_{37}H_{33}BrClNO_3Na^+$, 676.1225, found 676.1228. (Chiral IA, λ = 254 nm, *n*-hexane/2-propanol = 95/5, Flow rate = 1.0 mL/min), t_R = 9.207 min, 10.215 min (major).

HPLC chromatogram of racemic 30

Condition: n-hexane/2-propanol = 95:5

Flow rate = 1.0 mL/min

λ = 254 nm

Chiral IA

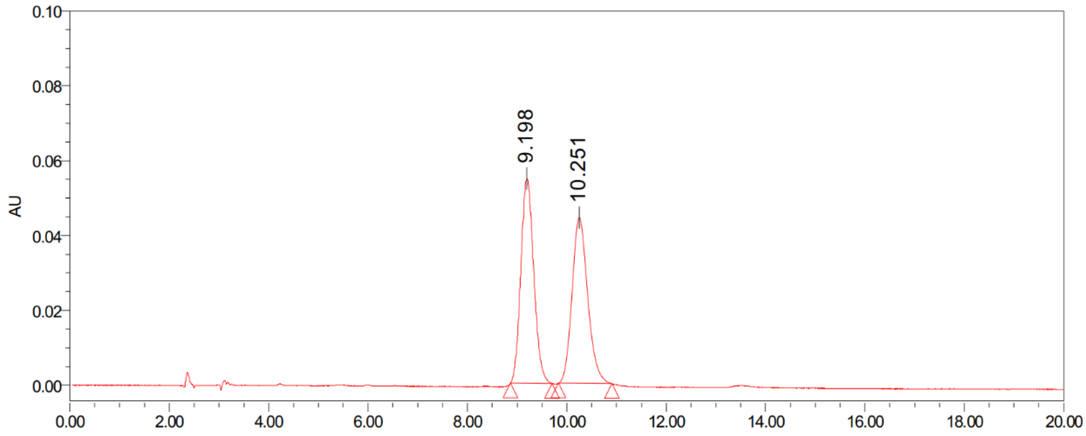

Entry	Retention Time/min	Area	Height	Area(%)
1	9.198	977570	54746	50.09
2	10.251	973947	44326	49.91

Supplementary Figure 60. Chiral HPLC analysis of racemic **30**

HPLC chromatogram of chiral 30

Condition: n-hexane/2-propanol = 95:5

Flow rate = 1.0 mL/min

λ = 254 nm

Chiral IA

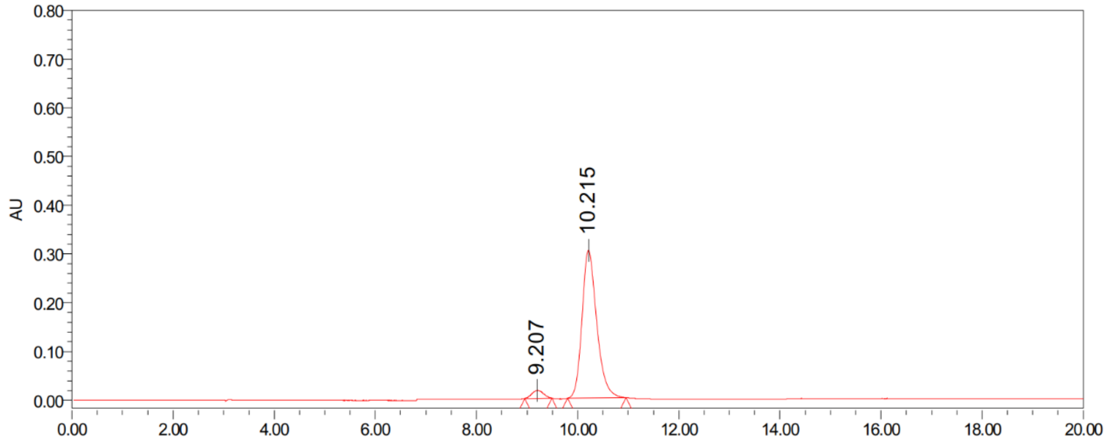

The larger version of HPLC chromatogram of chiral 30

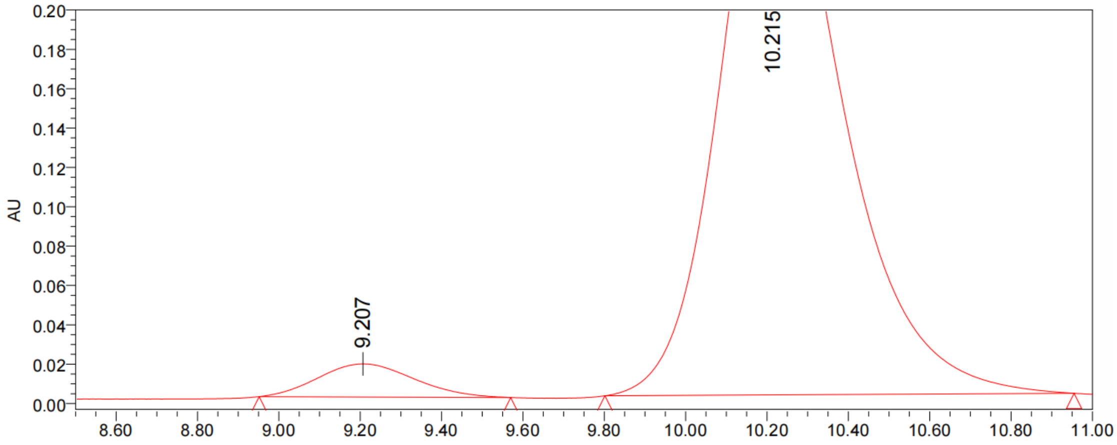

Entry	Retention Time/min	Area	Height	Area(%)
1	9.207	254822	16285	3.98
2	10.215	6146368	303188	96.02

Supplementary Figure 61. Chiral HPLC analysis of chiral **30**

***tert*-Butyl (2*R*,3*R*)-2-((4-bromobenzyl)oxy)-3-(2-methyl-1*H*-indol-3-yl)-2-phenyl-5-(*p*-tolyl)pent-4-ynoate (31)**

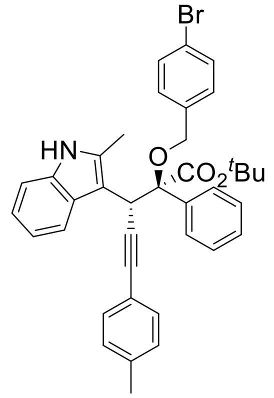

31

Yellow oil; 106.4 mg, 84% yield, >20:1 d.r., 94% *ee*, $[\alpha]_D^{20}$ = 12.27 (c = 0.033, MeOH); ^{1}H NMR (500 MHz, $CDCl_3$) δ 7.89 (s, 1H), 7.63 (s, 1H), 7.34 (d, *J* = 7.9 Hz, 4H), 7.28 (d, *J* = 8.1 Hz, 2H), 7.20 (dd, *J* = 9.3, 5.1 Hz, 1H), 7.16 (d, *J* = 8.0 Hz, 1H), 7.12 – 7.06 (m, 6H), 7.03 (t, *J* = 7.5 Hz, 1H), 6.90 (t, *J* = 7.5 Hz, 1H), 4.80 (d, *J* = 13.0 Hz, 1H), 4.73 (s, 1H), 4.29 (d, *J* = 13.0 Hz, 1H), 2.33 (s, 3H), 1.57 (s, 3H), 1.53 (s, 9H). ^{13}C NMR (126 MHz, $CDCl_3$) δ 171.0, 139.3, 137.7, 136.2, 135.0, 134.3, 131.5, 130.9, 129.0, 128.9, 128.5, 128.0, 127.9, 127.4, 121.0, 120.8, 120.3, 119.1, 109.7, 106.0, 89.7, 88.8, 83.2, 83.0, 67.7, 41.6, 28.2, 21.5, 11.2. HRMS(ESI) $[M+Na]^+$ calcd for $C_{38}H_{36}BrNO_3Na^+$, 656.1771, found 656.1771. (Chiral IA, λ = 254 nm, *n*-hexane/2-propanol = 95/5, Flow rate = 1.0 mL/min), t_R = 8.939 min, 11.703 min (major).

HPLC chromatogram of racemic 31

Condition: n-hexane/2-propanol = 95:5

Flow rate = 1.0 mL/min

λ = 254 nm

Chiral IA

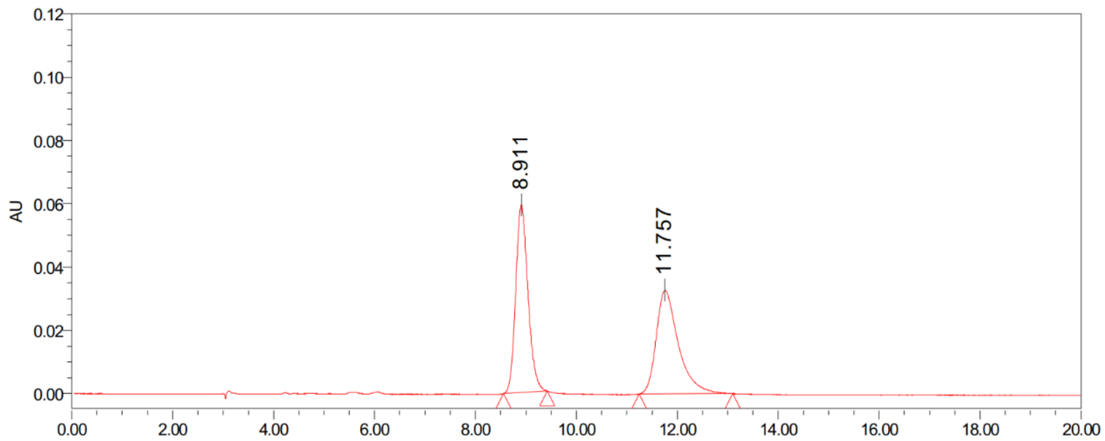

Entry	Retention Time/min	Area	Height	Area(%)
1	8.911	996920	59333	49.93
2	11.757	999586	32728	50.07

Supplementary Figure 62. Chiral HPLC analysis of racemic **31**

HPLC chromatogram of chiral 31

Condition: n-hexane/2-propanol = 95:5

Flow rate = 1.0 mL/min

λ = 254 nm

Chiral IA

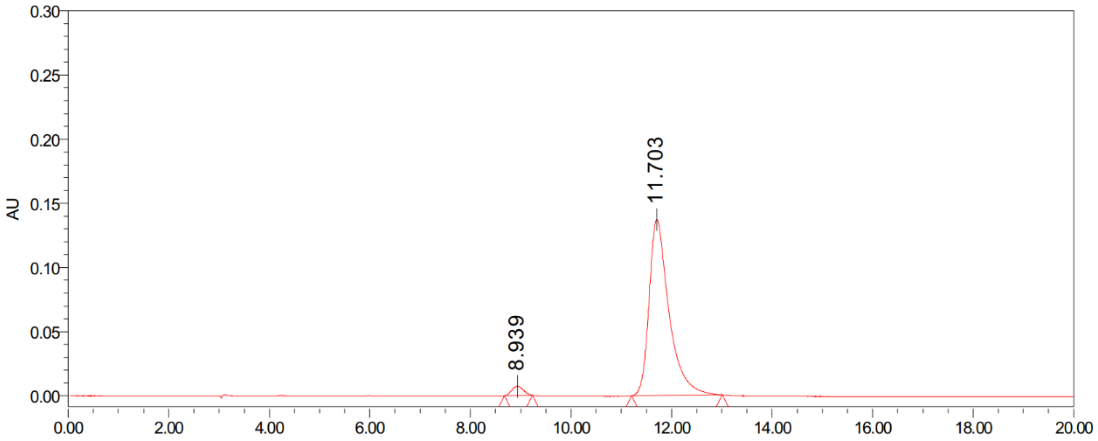

The larger version of HPLC chromatogram of chiral 31

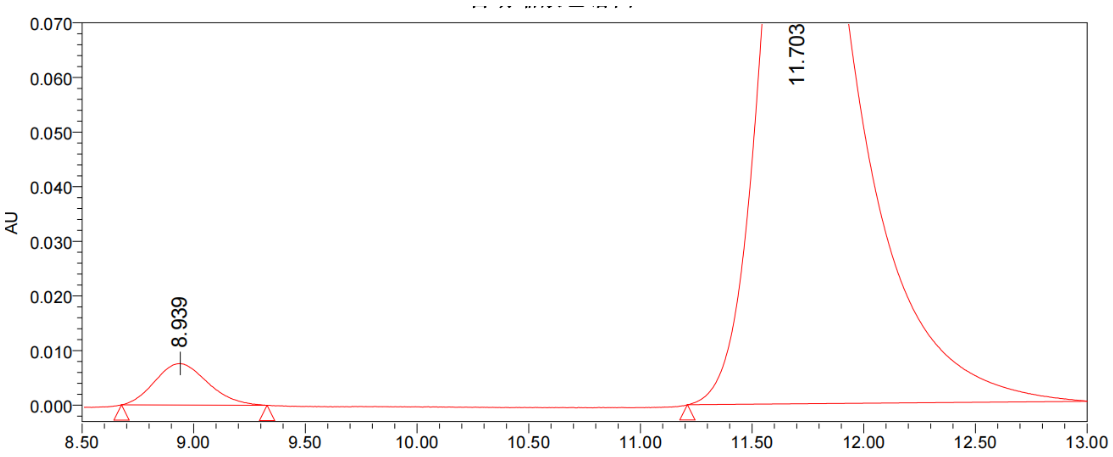

Entry	Retention Time/min	Area	Height	Area(%)
1	8.939	115788	7387	2.98
2	11.703	3768296	137193	97.02

Supplementary Figure 63. Chiral HPLC analysis of chiral **31**

***tert*-Butyl (2*R*,3*R*)-5-([1,1'-biphenyl]-4-yl)-2-((4-bromobenzyl)oxy)-3-(2-methyl-1*H*-indol-3-yl)-2-phenylpent-4-ynoate (32)**

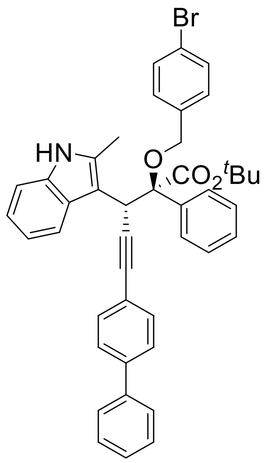

32

Yellow oil; 123.7 mg, 89% yield, >20:1 d.r., 93% *ee*, $[\alpha]_D^{20}$ = 45.81 (c = 0.033, MeOH); ^{1}H NMR (500 MHz, $CDCl_3$) δ 7.91 (s, 1H), 7.63 (s, 1H), 7.58 (d, *J* = 7.6 Hz, 2H), 7.52 (q, *J* = 8.2 Hz, 4H), 7.42 (t, *J* = 7.6 Hz, 2H), 7.32 (dt, *J* = 16.0, 8.2 Hz, 5H), 7.19 (d, *J* = 3.7 Hz, 1H), 7.16 (d, *J* = 8.0 Hz, 1H), 7.09 (d, *J* = 4.1 Hz, 4H), 7.04 (t, *J* = 7.5 Hz, 1H), 6.92 (t, *J* = 7.5 Hz, 1H), 4.83 (d, *J* = 13.0 Hz, 1H), 4.77 (s, 1H), 4.30 (d, *J* = 13.0 Hz, 1H), 1.58 (s, 3H), 1.55 (s, 9H). ^{13}C NMR (126 MHz, $CDCl_3$) δ 171.0, 140.6, 140.4, 139.3, 136.2, 135.1, 134.4, 132.1, 131.0, 128.9, 128.6, 128.0, 127.9, 127.6, 127.5, 127.1, 127.0, 123.1, 120.8, 120.3, 119.2, 109.8, 106.0, 90.4, 89.7, 83.0, 67.8, 41.7, 28.3, 11.2. HRMS(ESI) $[M+Na]^+$ calcd for $C_{43}H_{38}BrNO_3Na^+$, 718.1927, found 718.1926. (Chiral IA, λ = 254 nm, *n*-hexane/2-propanol = 95/5, Flow rate = 1.0 mL/min), t_R = 12.425 min, 18.333 min (major).

HPLC chromatogram of racemic 32

Condition: n-hexane/2-propanol = 95:5

Flow rate = 1.0 mL/min

λ = 254 nm

Chiral IA

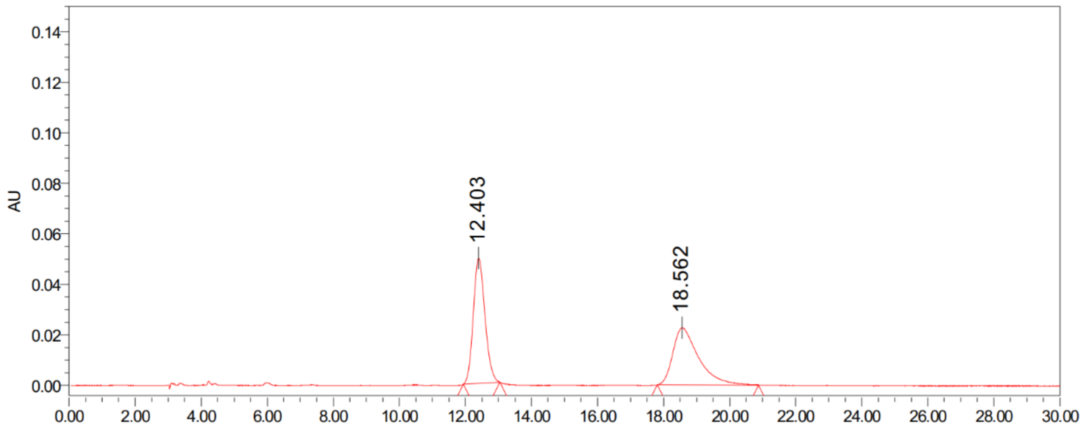

Entry	Retention Time/min	Area	Height	Area(%)
1	12.403	1220788	49556	50.04
2	18.562	1218617	22597	49.96

Supplementary Figure 64. Chiral HPLC analysis of racemic **32**

HPLC chromatogram of chiral 32

Condition: n-hexane/2-propanol = 95:5

Flow rate = 1.0 mL/min

λ = 254 nm

Chiral IA

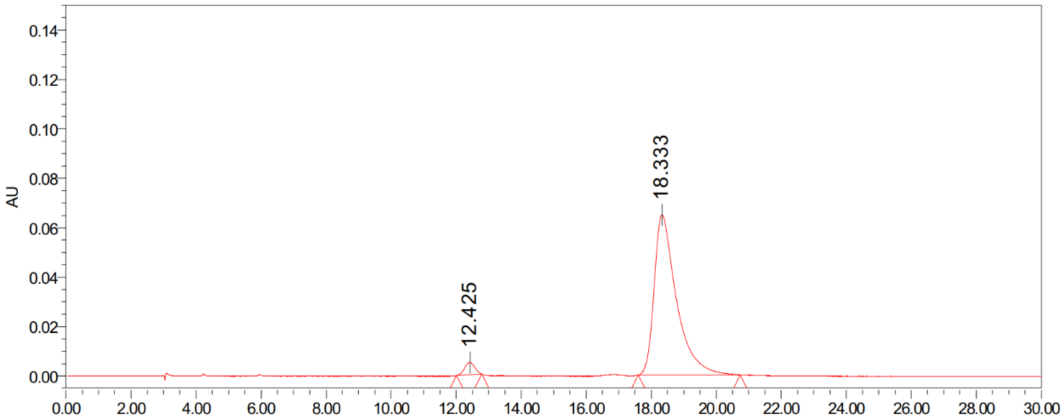

Entry	Retention Time/min	Area	Height	Area(%)
1	12.425	110267	5022	3.49
2	18.333	3099510	64842	96.51

Supplementary Figure 65. Chiral HPLC analysis of chiral **32**

tert-Butyl (2_R_,3_R_)-2-((4-bromobenzyl)oxy)-3-(2-methyl-1_H_-indol-3-yl)-2-phenyl-5-(_m_-tolyl)pent-4-ynoate (33)

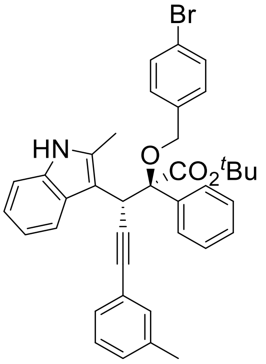

33

Yellow oil; 97.5 mg, 77% yield, >20:1 d.r., 95% *ee*, $[\alpha]_D^{20}$ = 6.83 (c = 0.033, MeOH); ^{1}H NMR (500 MHz, $CDCl_3$) δ 7.88 (s, 1H), 7.64 (s, 1H), 7.34 (d, *J* = 8.0 Hz, 2H), 7.27 (dd, *J* = 17.0, 8.8 Hz, 4H), 7.22 – 7.14 (m, 3H), 7.08 (d, *J* = 2.9 Hz, 5H), 7.03 (t, *J* = 7.4 Hz, 1H), 6.90 (t, *J* = 7.4 Hz, 1H), 4.81 (d, *J* = 13.0 Hz, 1H), 4.74 (s, 1H), 4.30 (d, *J* = 13.0 Hz, 1H), 2.31 (s, 3H), 1.57 (s, 3H), 1.53 (s, 9H). ^{13}C NMR (126 MHz, $CDCl_3$) δ 170.9, 139.3, 137.8, 136.2, 135.0, 134.3, 132.3, 131.0, 128.9, 128.7, 128.6, 128.6, 128.2, 128.0, 127.9, 127.5, 123.9, 120.8, 120.3, 119.1, 109.8, 106.0, 89.7, 89.2, 83.3, 83.0, 67.7, 41.5, 28.2, 21.3, 11.2. HRMS(ESI) $[M+Na]^+$ calcd for $C_{38}H_{36}BrNO_3Na^+$, 656.1771, found 656.1771. (Chiral IA, λ = 254 nm, *n*-hexane/2-propanol = 95/5, Flow rate = 1.0 mL/min), t_R = 7.470 min, 8.958 min (major).

HPLC chromatogram of racemic 33

Condition: n-hexane/2-propanol = 95:5

Flow rate = 1.0 mL/min

λ = 254 nm

Chiral IA

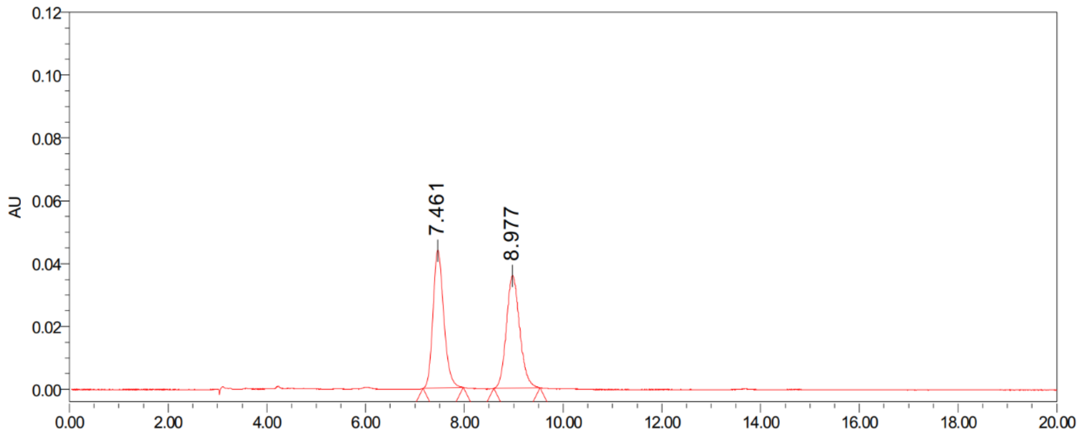

Entry	Retention Time/min	Area	Height	Area(%)
1	7.461	669454	43804	50.09
2	8.977	667011	35772	49.91

Supplementary Figure 66. Chiral HPLC analysis of racemic **33**

HPLC chromatogram of chiral 33

Condition: n-hexane/2-propanol = 95:5

Flow rate = 1.0 mL/min

λ = 254 nm

Chiral IA

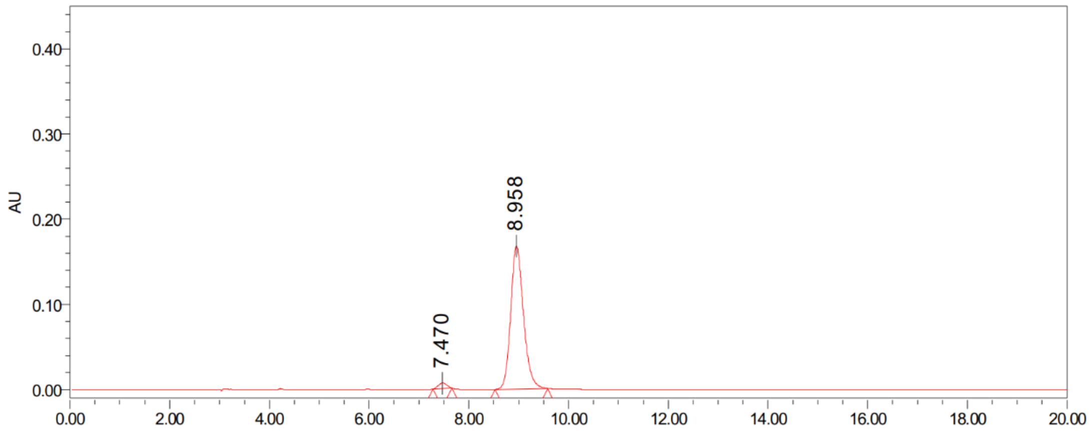

The larger version of HPLC chromatogram of chiral 33

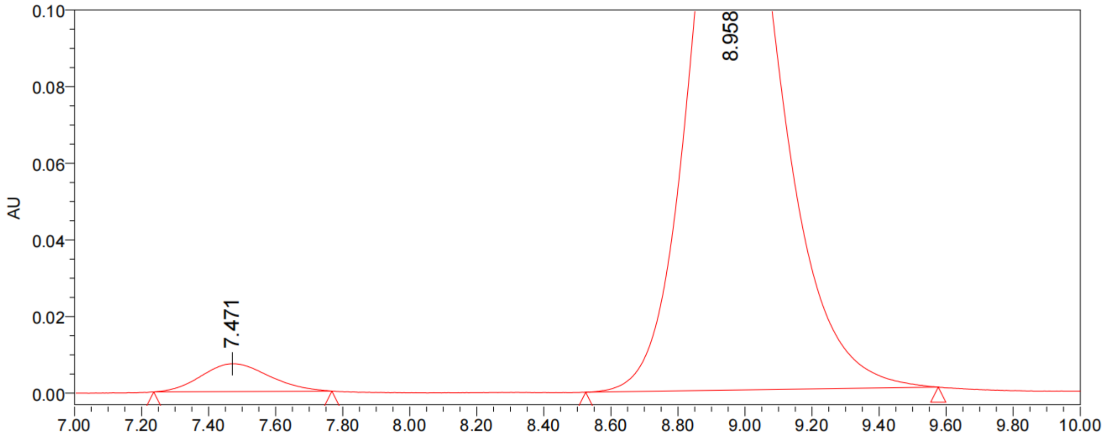

Entry	Retention Time/min	Area	Height	Area(%)
1	7.470	75358	6271	2.48
2	8.958	2959512	167864	97.52

Supplementary Figure 67. Chiral HPLC analysis of chiral **33**

***tert*-Butyl (2*R*,3*R*)-2-((4-bromobenzyl)oxy)-5-(2-fluorophenyl)-3-(2-methyl-1*H*-indol-3-yl)-2-phenylpent-4-ynoate (34)**

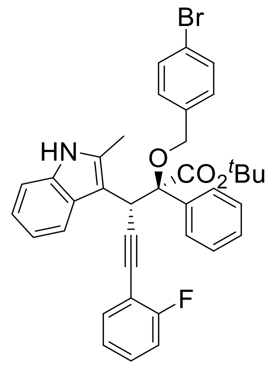

34

Yellow oil; 86.7 mg, 68% yield, >20:1 d.r., 94% *ee*, $[\alpha]_D^{20}$ = 9.58 (c = 0.033, MeOH); ^{1}H NMR (400 MHz, $CDCl_3$) δ 7.86 (d, *J* = 5.7 Hz, 1H), 7.64 (s, 1H), 7.44 – 7.35 (m, 3H), 7.31 (d, *J* = 8.4 Hz, 2H), 7.28 – 7.24 (m, 1H), 7.23 – 7.19 (m, 1H), 7.17 (d, *J* = 8.0 Hz, 1H), 7.12 – 7.07 (m, 4H), 7.06 (d, *J* = 8.1 Hz, 2H), 7.02 (d, *J* = 7.6 Hz, 1H), 6.89 (t, *J* = 7.4 Hz, 1H), 4.82 (d, *J* = 14.1 Hz, 2H), 4.32 (d, *J* = 12.9 Hz, 1H), 1.61 (s, 3H), 1.51 (s, 9H). ^{13}C NMR (126 MHz, $CDCl_3$) δ 170.9, δ 141.7 (d, *J* = 192.8 Hz), 139.2, 136.1, 135.0, 134.4, 133.9, 130.9, 129.4 (d, *J* = 7.6 Hz), 128.8, 128.6, 128.0, 127.9, 127.4, 123.8 (d, *J* = 3.8 Hz), 120.8, 120.3, 119.1, 115.5 (d, *J* = 21.4 Hz), 112.5 (d, *J* = 16.4 Hz), 109.7, 105.7, 94.8, 89.5, 83.1, 76.6, 67.8, 41.6, 28.1, 11.2. ^{19}F NMR (376 MHz, $CDCl_3$) δ -109.85. HRMS(ESI) $[M+Na]^+$ calcd for $C_{37}H_{33}BrFNO_3Na^+$, 660.1520, found 660.1522. (Chiral IA, λ = 254 nm, *n*-hexane/2-propanol = 98/2, Flow rate = 1.0 mL/min), t_R = 17.431 min, 19.478 min (major).

HPLC chromatogram of racemic 34

Condition: n-hexane/2-propanol = 98:2

Flow rate = 1.0 mL/min

λ = 254 nm

Chiral IA

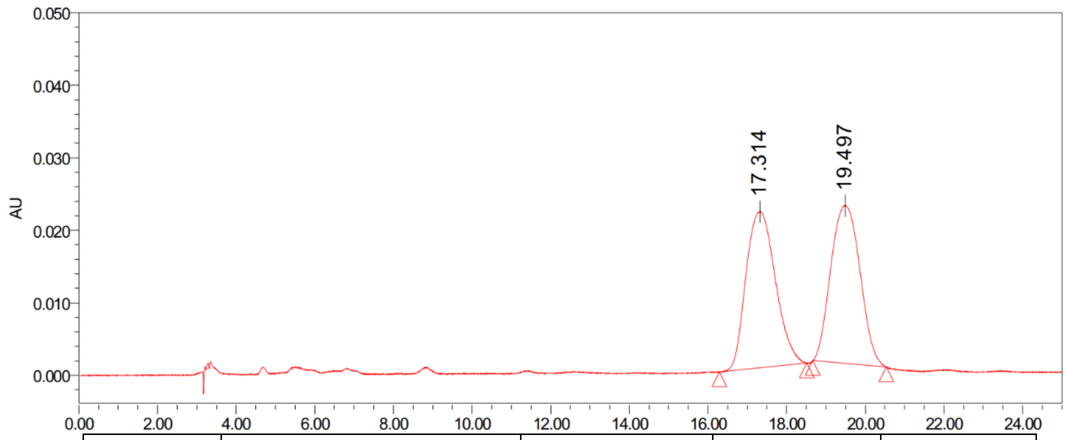

Entry	Retention Time/min	Area	Height	Area(%)
1	17.314	1120039	21525	49.94
2	19.497	1122854	21773	50.06

Supplementary Figure 68. Chiral HPLC analysis of racemic **34**

HPLC chromatogram of chiral 34

Condition: n-hexane/2-propanol = 98:2

Flow rate = 1.0 mL/min

λ = 254 nm

Chiral IA

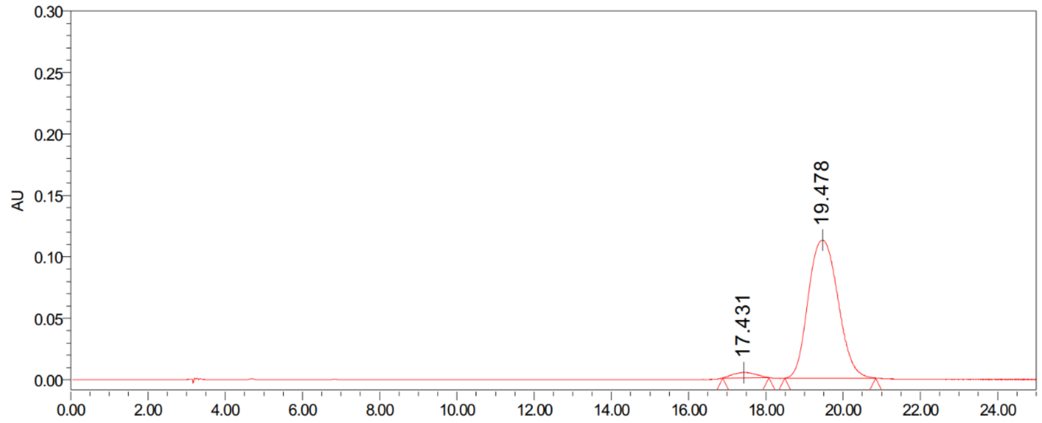

Entry	Retention Time/min	Area	Height	Area(%)
1	17.431	179090	4259	2.90
2	19.478	5991215	112243	97.10

Supplementary Figure 69. Chiral HPLC analysis of chiral **34**

***tert*-Butyl (2*R*,3*R*)-2-((4-bromobenzyl)oxy)-3-(2-methyl-1*H*-indol-3-yl)-5-(naphthalen-1-yl)-2-phenylpent-4-ynoate (35)**

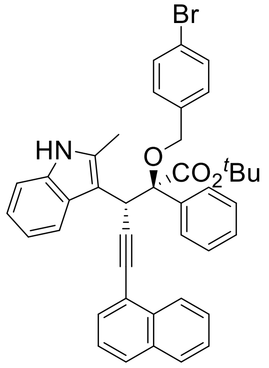

35

Yellow oil; 103.1 mg, 77% yield, >20:1 d.r., 95% *ee*, $[\alpha]_D^{20}$ = 54.41 (c = 0.033, MeOH); ^{1}H NMR (500 MHz, $CDCl_3$) δ 8.48 (d, *J* = 8.4 Hz, 1H), 7.94 (s, 1H), 7.81 (d, *J* = 8.2 Hz, 1H), 7.76 (d, *J* = 8.2 Hz, 1H), 7.64 (s, 1H), 7.61 (d, *J* = 7.1 Hz, 1H), 7.47 (t, *J* = 7.5 Hz, 1H), 7.36 (dd, *J* = 14.9, 7.4 Hz, 3H), 7.33 – 7.27 (m, 3H), 7.21 (dd, *J* = 7.2, 4.7 Hz, 1H), 7.17 (d, *J* = 8.0 Hz, 1H), 7.14 – 7.09 (m, 4H), 7.03 (t, *J* = 7.5 Hz, 1H), 6.88 (t, *J* = 7.5 Hz, 1H), 4.92 (s, 1H), 4.88 (d, *J* = 12.5 Hz, 1H), 4.32 (d, *J* = 12.5 Hz, 1H), 1.63 (s, 3H), 1.48 (s, 9H). ^{13}C NMR (126 MHz, $CDCl_3$) δ 171.1, 138.9, 136.1, 135.0, 134.5, 133.8, 133.2, 131.1, 130.2, 129.0, 128.8, 128.09, 128.06, 128.05, 128.0, 127.5, 127.1, 126.5, 126.3, 125.3, 121.8, 120.9, 120.5, 119.1, 109.8, 106.0, 94.4, 89.8, 83.1, 81.4, 68.0, 41.8, 28.2, 11.3. HRMS(ESI) $[M+Na]^+$ calcd for $C_{41}H_{36}BrNO_3Na^+$, 692.1771, found 692.1770. (Chiral IC, λ = 254 nm, *n*-hexane/2-propanol = 95/5, Flow rate = 1.0 mL/min), t_R = 4.731 min, 5.691 min (major).

HPLC chromatogram of racemic 35

Condition: n-hexane/2-propanol = 95:5

Flow rate = 1.0 mL/min

λ = 254 nm

Chiral IC

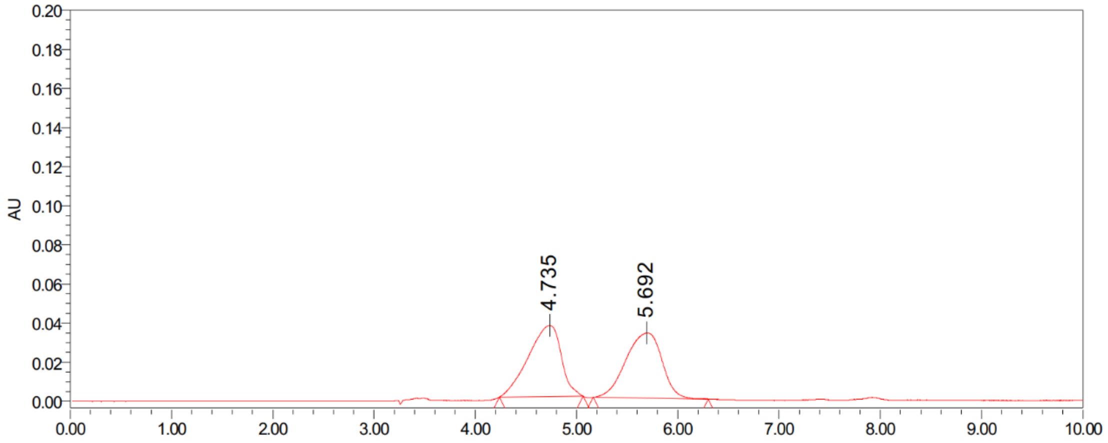

Entry	Retention Time/min	Area	Height	Area(%)
1	4.735	823336	36408	49.94
2	5.692	825343	33356	50.06

Supplementary Figure 70. Chiral HPLC analysis of racemic **35**

HPLC chromatogram of chiral 35

Condition: n-hexane/2-propanol = 95:5

Flow rate = 1.0 mL/min

λ = 254 nm

Chiral IC

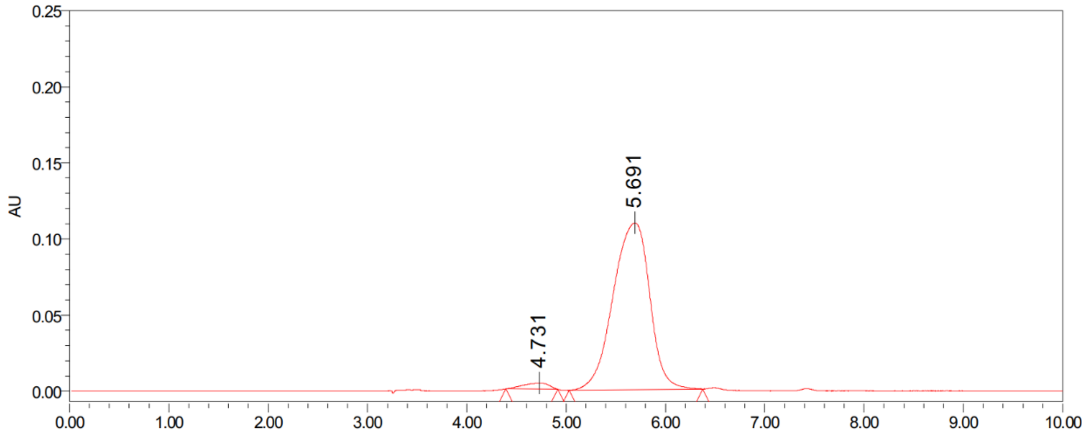

The larger version of HPLC chromatogram of chiral 35

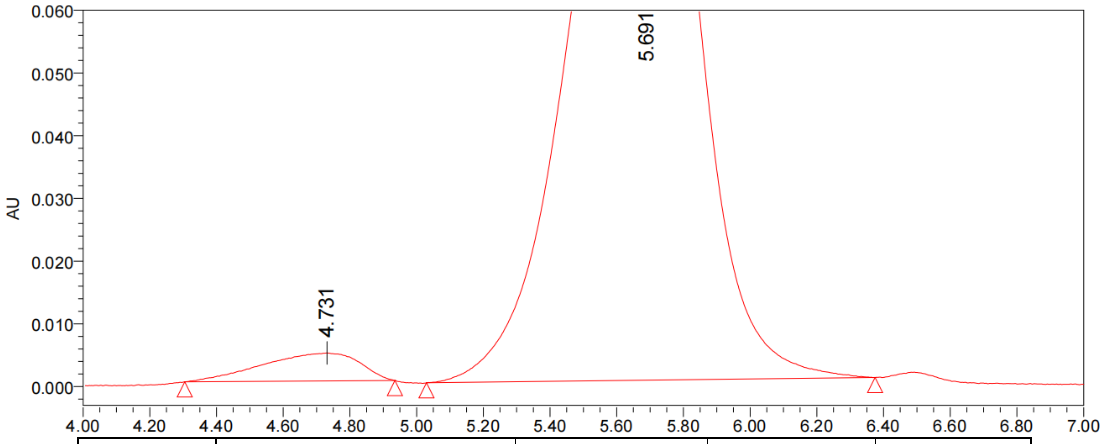

Entry	Retention Time/min	Area	Height	Area(%)
1	4.731	71946	4014	2.50
2	5.691	2801541	109530	97.50

Supplementary Figure 71. Chiral HPLC analysis of chiral **35**

***tert*-Butyl (2*R*,3*R*)-2-((4-bromobenzyl)oxy)-3-(2-methyl-1*H*-indol-3-yl)-2-phenyl-5-(thiophen-2-yl)pent-4-ynoate (36)**

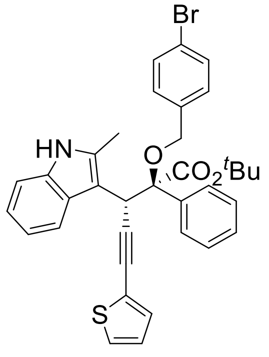

36

Yellow oil; 96.3 mg, 77% yield, >20:1 d.r., 95% *ee*, $[\alpha]_D^{20}$ = -9.36 (c = 0.033, MeOH); ^{1}H NMR (500 MHz, $CDCl_3$) δ 7.83 (s, 1H), 7.64 (s, 1H), 7.37 (d, *J* = 8.2 Hz, 2H), 7.28 (d, *J* = 8.2 Hz, 2H), 7.22 – 7.17 (m, 2H), 7.14 (dd, *J* = 10.5, 5.7 Hz, 2H), 7.10 – 7.05 (m, 4H), 7.02 (t, *J* = 7.5 Hz, 1H), 6.95 – 6.92 (m, 1H), 6.90 (t, *J* = 7.5 Hz, 1H), 4.78 (d, *J* = 13.1 Hz, 1H), 4.75 (s, 1H), 4.30 (d, *J* = 13.1 Hz, 1H), 1.57 (s, 3H), 1.53 (s, 9H). ^{13}C NMR (126 MHz, $CDCl_3$) δ 170.9, 139.3, 136.0, 135.0, 134.4, 131.2, 131.0, 128.8, 128.5, 128.1, 127.9, 127.5, 126.9, 126.3, 124.2, 120.8, 120.3, 119.2, 109.8, 105.6, 93.7, 89.6, 83.1, 76.5, 67.7, 41.9, 28.2, 11.2. HRMS(ESI) $[M+Na]^+$ calcd for $C_{35}H_{32}BrNO_3SNa^+$, 648.1178, found 648.1175. (Chiral IA, λ = 254 nm, *n*-hexane/2-propanol = 99/1, Flow rate = 1.0 mL/min), t_R = 39.538 min, 43.205 min (major).

HPLC chromatogram of racemic 36

Condition: n-hexane/2-propanol = 99:1

Flow rate = 1.0 mL/min

λ = 254 nm

Chiral IA

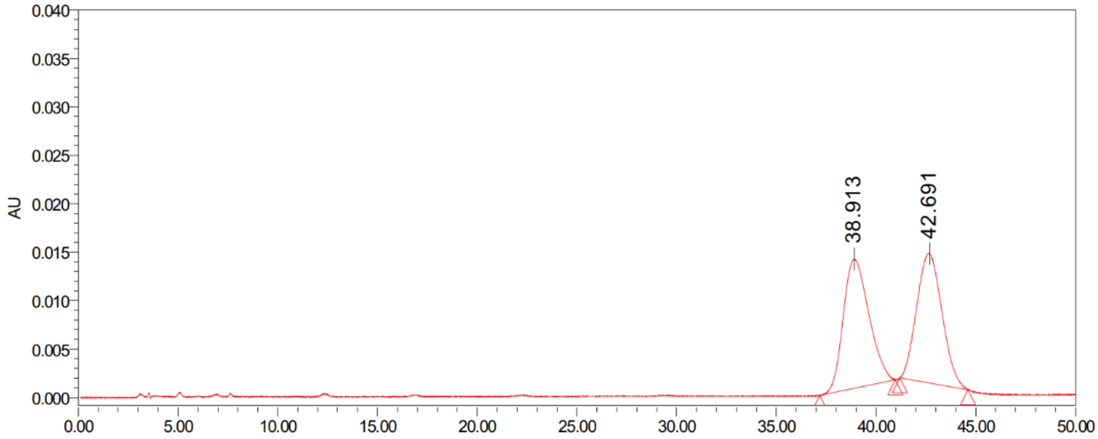

Entry	Retention Time/min	Area	Height	Area(%)
1	38.913	1184311	13330	50.03
2	42.691	1183023	13360	49.97

Supplementary Figure 72. Chiral HPLC analysis of racemic **36**

HPLC chromatogram of chiral 36

Condition: n-hexane/2-propanol = 99:1

Flow rate = 1.0 mL/min

λ = 254 nm

Chiral IA

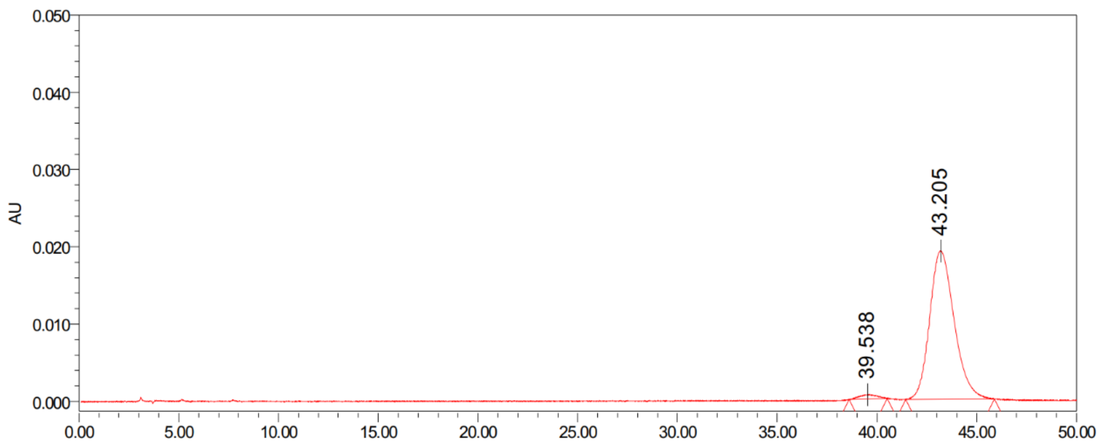

The larger version of HPLC chromatogram of chiral 36

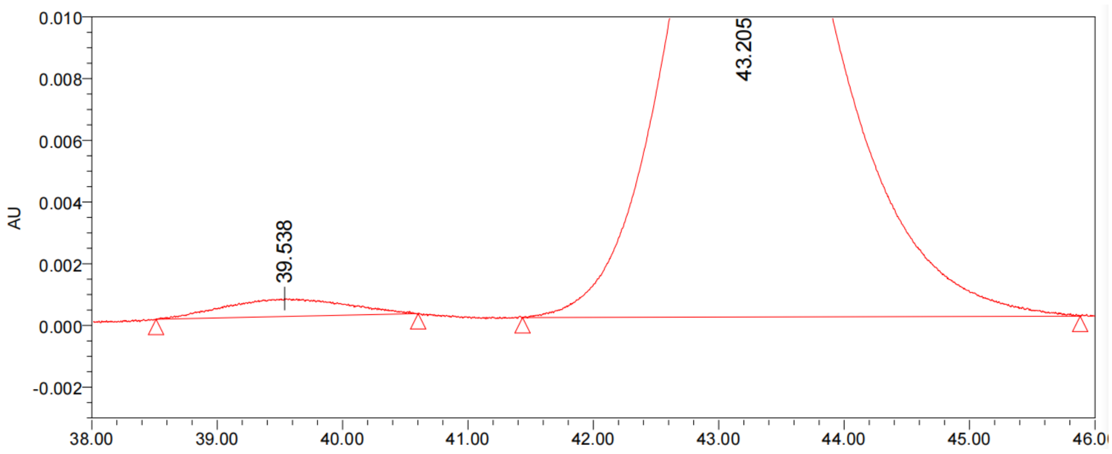

Entry	Retention Time/min	Area	Height	Area(%)
1	39.538	34253	548	2.51
2	43.205	1647070	19207	97.49

Supplementary Figure 73. Chiral HPLC analysis of chiral **36**

***tert*-Butyl (2*R*,3*R*)-2-((4-bromobenzyl)oxy)-3-(2-methyl-1*H*-indol-3-yl)-2-phenyl-5-(thiophen-3-yl)pent-4-ynoate (37)**

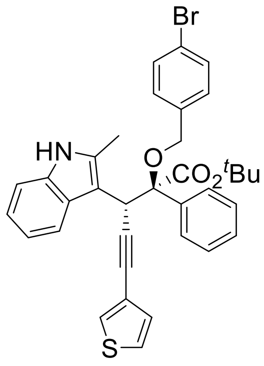

37

Yellow oil; 102.5 mg, 82% yield, >20:1 d.r., 94% *ee*, $[\alpha]_D^{20}$ = -12.15 (c = 0.033, MeOH); ^{1}H NMR (500 MHz, $CDCl_3$) δ 7.88 (s, 1H), 7.64 (s, 1H), 7.34 (d, *J* = 8.6 Hz, 3H), 7.25 (d, *J* = 8.1 Hz, 2H), 7.23 – 7.21 (m, 1H), 7.18 (d, *J* = 8.0 Hz, 1H), 7.15 (d, *J* = 8.0 Hz, 1H), 7.11 – 7.06 (m, 5H), 7.03 (t, *J* = 7.5 Hz, 1H), 6.90 (t, *J* = 7.5 Hz, 1H), 4.81 (d, *J* = 13.0 Hz, 1H), 4.71 (s, 1H), 4.28 (d, *J* = 13.0 Hz, 1H), 1.55 (s, 3H), 1.53 (s, 9H). ^{13}C NMR (126 MHz, $CDCl_3$) δ 171.0, 139.3, 136.1, 135.1, 134.3, 130.9, 130.0, 128.8, 128.5, 128.0, 127.87, 127.86, 127.5, 125.1, 123.1, 120.8, 120.3, 119.1, 109.8, 105.8, 89.7, 89.0, 83.0, 78.4, 67.7, 41.6, 28.2, 11.1. HRMS(ESI) $[M+Na]^+$ calcd for $C_{35}H_{32}BrNO_3SNa^+$, 648.1178, found 648.1179. (Chiral IA, λ = 254 nm, *n*-hexane/2-propanol = 95/5, Flow rate = 1.0 mL/min), t_R = 10.349 min, 12.001 min (major).

HPLC chromatogram of racemic 37

Condition: n-hexane/2-propanol = 95:5

Flow rate = 1.0 mL/min

λ = 254 nm

Chiral IA

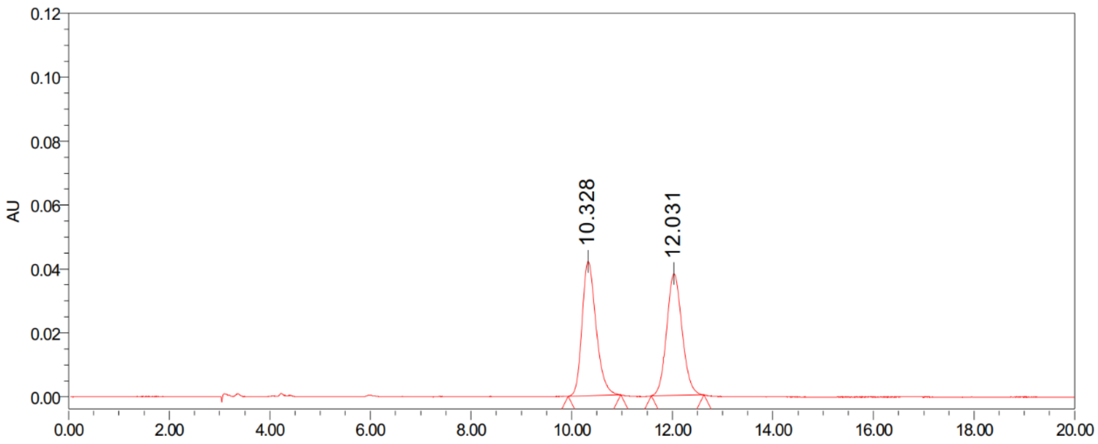

Entry	Retention Time/min	Area	Height	Area(%)
1	10.328	813577	42019	50.07
2	12.031	811280	37995	49.93

Supplementary Figure 74. Chiral HPLC analysis of racemic **37**

HPLC chromatogram of chiral 37

Condition: n-hexane/2-propanol = 95:5

Flow rate = 1.0 mL/min

λ = 254 nm

Chiral IA

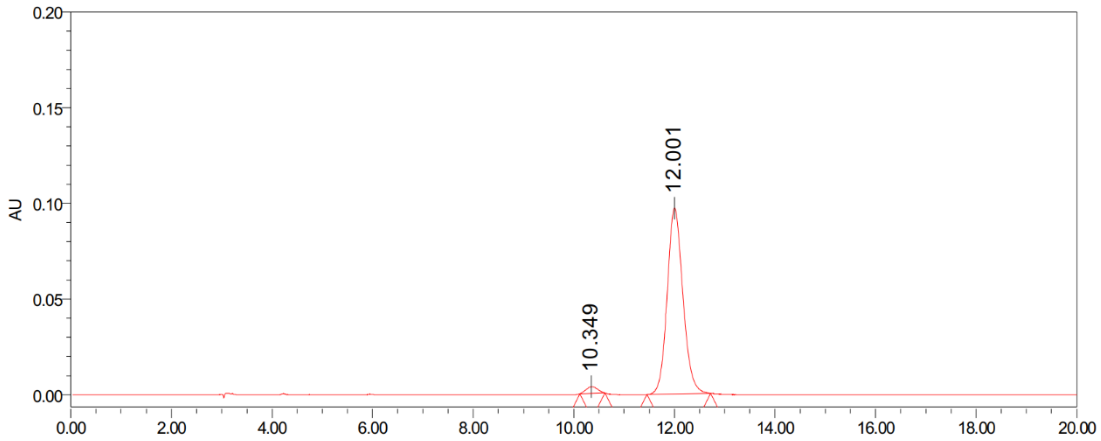

The larger version of HPLC chromatogram of chiral 37

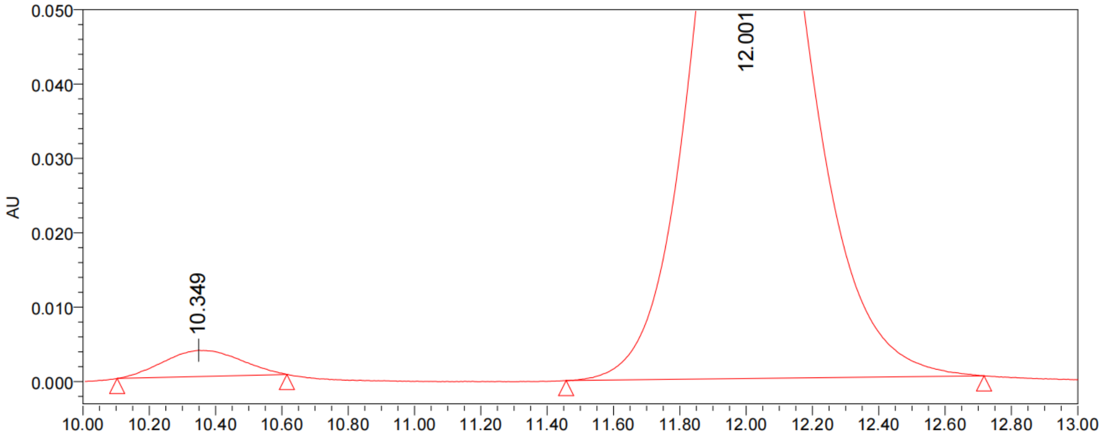

Entry	Retention Time/min	Area	Height	Area(%)
1	10.349	54960	3473	2.63
2	12.001	2117462	97155	97.37

Supplementary Figure 75. Chiral HPLC analysis of chiral **37**

Tert-butyl (2S,3R)-2-((4-bromobenzyl)oxy)-3-(2-oxo-2-phenylethyl)-2,5-diphenylpent-4-ynoate(38)

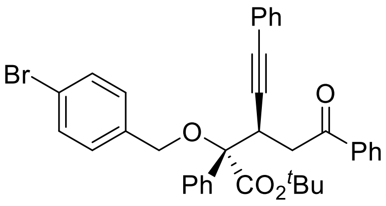

38

Colorless oil; 65.7 mg, 72% yield, 98:2 d.r., 98% *ee*, $[\alpha]_D^{20}$ = -14.66 (c = 0.1, CH_2Cl_2);

^{1}H NMR (400 MHz, $CDCl_3$) δ 7.87 – 7.82 (m, 2H), 7.71 – 7.67 (m, 2H), 7.56 – 7.50 (m, 1H), 7.49 – 7.46 (m, 2H), 7.42 (d, *J* = 7.9 Hz, 2H), 7.38 (d, *J* = 8.5 Hz, 3H), 7.35 – 7.31 (m, 2H), 7.24 (d, *J* = 8.2 Hz, 5H), 5.05 (d, *J* = 11.7 Hz, 1H), 4.90 (d, *J* = 11.7 Hz, 1H), 4.41 (dd, *J* = 10.2, 3.1 Hz, 1H), 3.32 (dd, *J* = 16.6, 10.2 Hz, 1H), 3.09 (dd, *J* = 16.6, 3.1 Hz, 1H), 1.51 (s, 9H). ^{13}C NMR (101 MHz, $CDCl_3$) δ 197.4, 169.4, 138.3, 138.1, 136.9, 133.1, 131.4, 129.2, 128.6, 128.6, 128.3, 128.3, 128.3, 128.2, 127.9, 126.7, 123.4, 121.2, 89.7, 85.9, 84.1, 83.0, 68.0, 39.2, 36.6, 28.1. HRMS (ESI) $[M+Na]^+$ calcd for $C_{36}H_{33}O_4BrNa^+$, 631.1454, found 631.1455. (Chiral IA, λ= 254 nm, *n*-hexane/2-propanol= 49/1, Flow rate = 1.0 mL/min), t_R = 10.370 min(major), 18.293 min.

HPLC chromatogram of racemic 38

Condition: n-hexane/2-propanol =49:1

Flow rate =1.0 mL/min

λ= 254 nm

Chiral IA

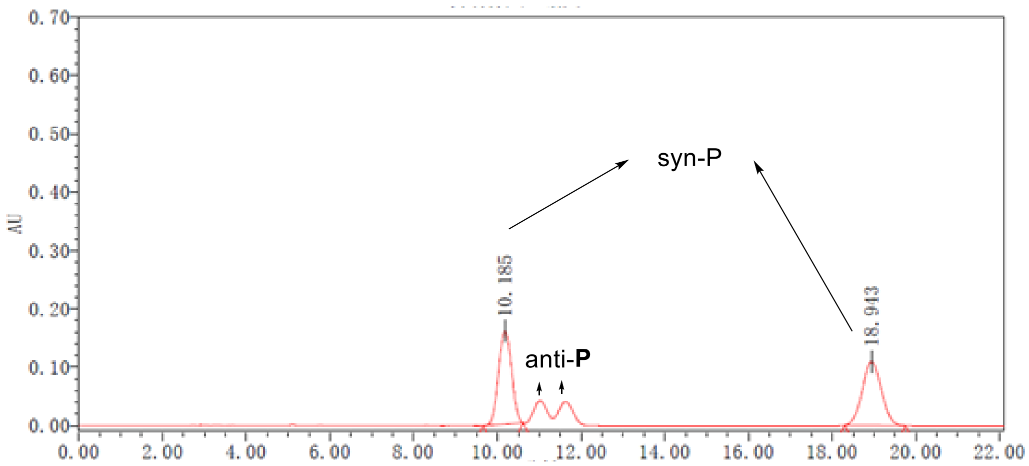

Entry	Retention Time/min	Area	Height	Area(%)
1	10.185	3452482	159302	49.84
2	18.943	3475146	109538	50.16

Supplementary Figure 76. Chiral HPLC analysis of racemic **38**

HPLC chromatogram of chiral 38

Condition: n-hexane/2-propanol =49:1

Flow rate =1.0 mL/min

λ= 254 nm

Chiral IA

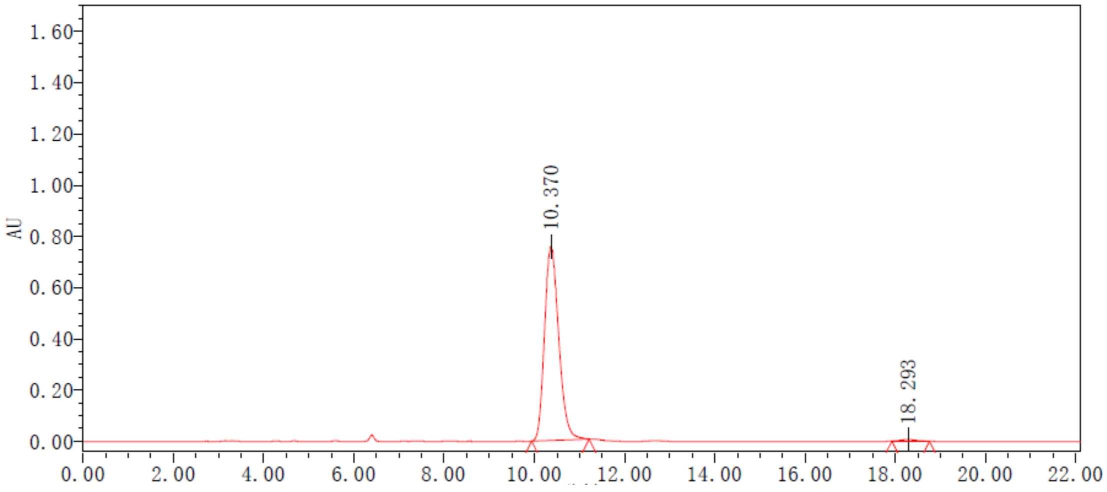

The larger version of HPLC chromatogram of chiral 38

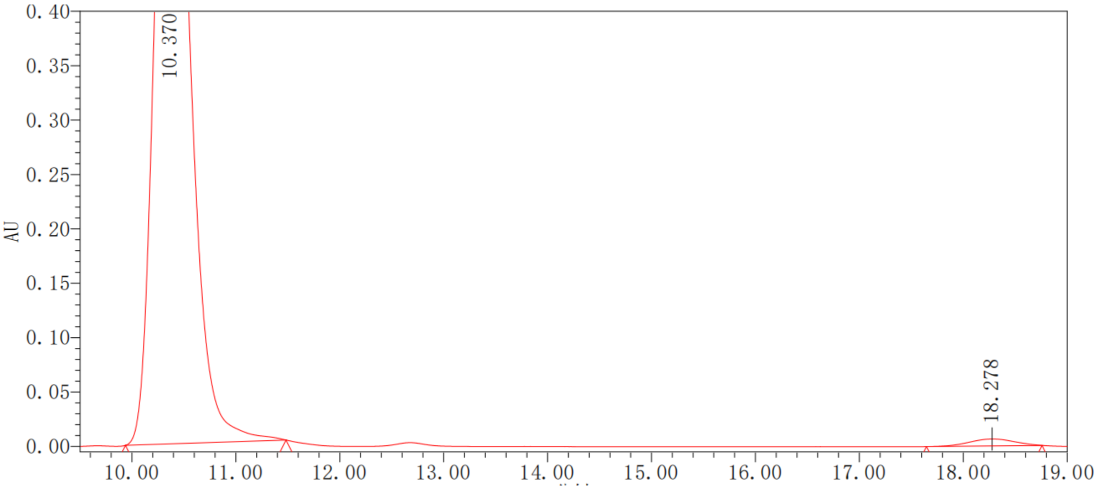

Entry	Retention Time/min	Area	Height	Area(%)
1	10.370	16899995	758510	99.01
2	18.278	190997	6399	0.99

Supplementary Figure 77. Chiral HPLC analysis of chiral **38**

HPLC chromatogram of 38 (half a gram-scale synthesis)

HPLC chromatogram of racemic 38

Condition: n-hexane/2-propanol =49:1

Flow rate =1.0 mL/min

λ= 254 nm

Chiral IA

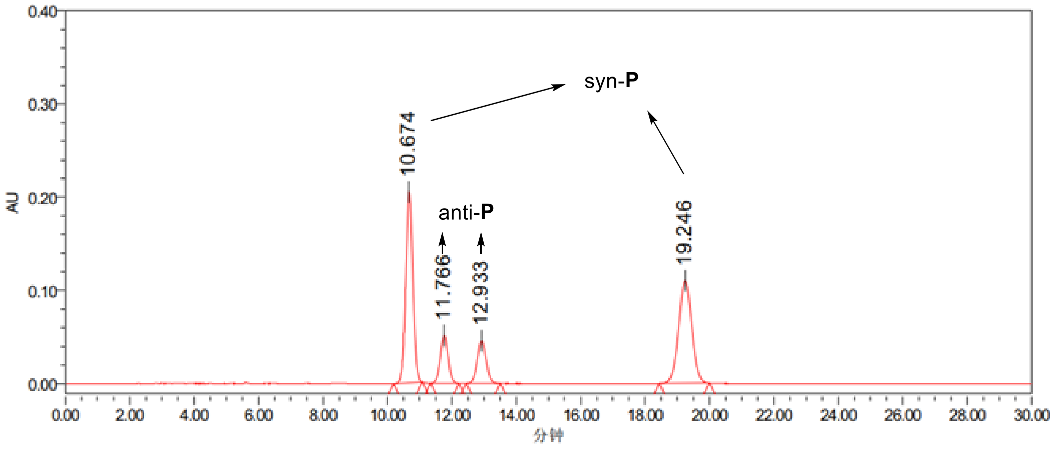

Entry	Retention Time/min	Area	Height	Area(%)
1	10.674	3126283	204889	38.60
2	11.766	867633	51348	10.72
3	12.933	884701	45933	10.92
4	19.246	3130920	109766	38.66

Supplementary Figure 78. Chiral HPLC analysis of racemic **38 (half a gram-scale synthesis)**

HPLC chromatogram of chiral 38

Condition: n-hexane/2-propanol =49:1

Flow rate =1.0 mL/min

λ= 254 nm

Chiral IA

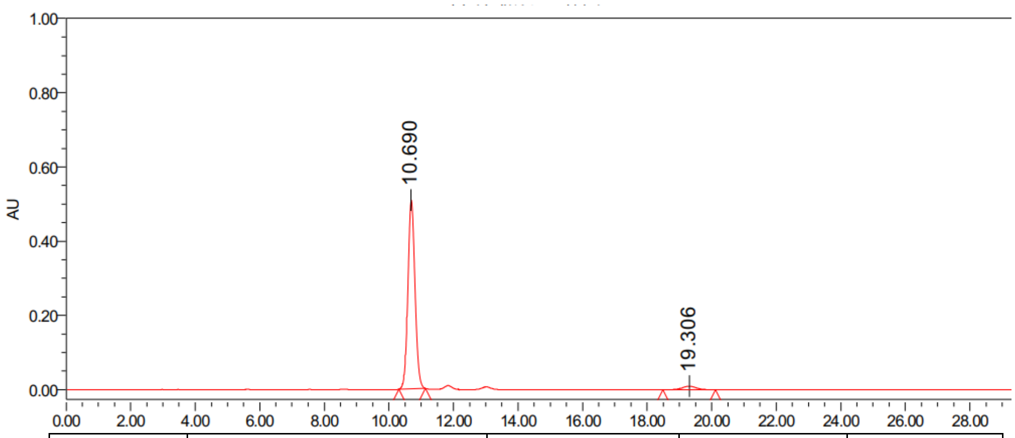

Entry	Retention Time/min	Area	Height	Area(%)
1	10.690	7766989	508858	96.74
2	19.306	262150	9198	3.26

Supplementary Figure 79. Chiral HPLC analysis of chiral **38 (half a gram-scale synthesis)**

Methyl (2*S*,3*R*)-2-((4-bromobenzyl)oxy)-3-(2-oxo-2-phenylethyl)-2,5-diphenylpent-4-ynoate(syn-38-b)

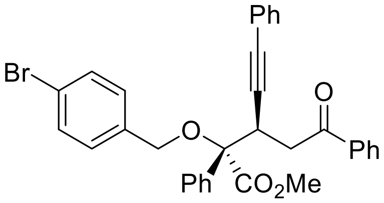

syn-**38-b**

Colorless oil; 56.9 mg, 67% yield, 94:6 d.r., 46% *ee*; ^{1}H NMR (400 MHz, $CDCl_3$) δ 7.86 – 7.82 (m, 2H), 7.68 – 7.64 (m, 2H), 7.53 (t, *J* = 7.4 Hz, 1H), 7.47 (dd, *J* = 8.5, 4.0 Hz, 2H), 7.42 – 7.34 (m, 7H), 7.24 (d, *J* = 6.0 Hz, 5H), 5.03 (d, *J* = 11.6 Hz, 1H), 4.83 (d, *J* = 11.6 Hz, 1H), 4.48 (dd, *J* = 10.0, 3.2 Hz, 1H), 3.85 (s, 3H), 3.29 (dd, *J* = 16.7, 10.0 Hz, 1H), 3.18 – 3.11 (m, 1H). ^{13}C NMR (101 MHz, $CDCl_3$) δ ^{13}C NMR (101 MHz, $CDCl_3$) δ 197.2, 171.2, 137.7, 136.8, 133.2, 131.5, 131.5, 129.3, 128.8, 128.6, 128.5, 128.2, 128.2, 128.2, 128.0, 126.7, 123.2, 121.4, 89.0, 86.2, 84.5, 68.2, 52.6, 39.3, 36.5. HRMS (ESI) $[M+Na]^+$ calcd for $C_{33}H_{27}O_4BrNa^+$, 589.0985, found 589.0985. (Chiral

IA-3, λ= 254 nm, *n*-hexane/2-propanol= 49/1, Flow rate = 1.0 mL/min), t_R = 23.807 min(major), 35.090 min.

Methyl (2R,3R)-2-((4-bromobenzyl)oxy)-3-(2-oxo-2-phenylethyl)-2,5-diphenylpent-4-ynoate(anti-38-b)

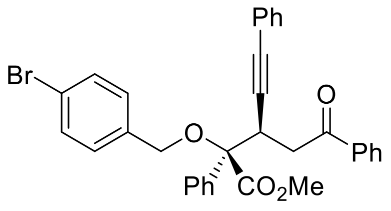

anti-**38-b**

^{1}H NMR (400 MHz, $CDCl_3$) δ 7.88 – 7.84 (m, 2H), 7.61 (dd, *J* = 8.0, 1.6 Hz, 2H), 7.52 (t, *J* = 7.4 Hz, 1H), 7.45 (d, *J* = 8.4 Hz, 2H), 7.42 (d, *J* = 8.0 Hz, 3H), 7.39 (d, *J* = 1.9 Hz, 2H), 7.26 – 7.22 (m, 7H), 4.57 (d, *J* = 12.1 Hz, 1H), 4.49 (d, *J* = 12.4 Hz, 1H), 3.83 (s, 3H), 3.54 (s, 1H), 3.15 (d, *J* = 5.3 Hz, 2H). ^{13}C NMR (101 MHz, $CDCl_3$) δ 197.3, 171.0, 137.2, 136.9, 135.5, 133.1, 131.5, 131.4, 128.8, 128.7, 128.6, 128.5, 128.4, 128.2, 128.1, 128.0, 123.2, 121.3, 88.6, 87.1, 84.3, 67.4, 52.6, 39.7, 37.3. HRMS (ESI) $[M+Na]^+$ calcd for $C_{33}H_{27}O_4BrNa^+$, 589.0985, found 589.0985.

HPLC chromatogram of racemic 38-b

Condition: n-hexane/2-propanol =49:1

Flow rate =1.0 mL/min

λ= 254 nm

Chiral IA-3

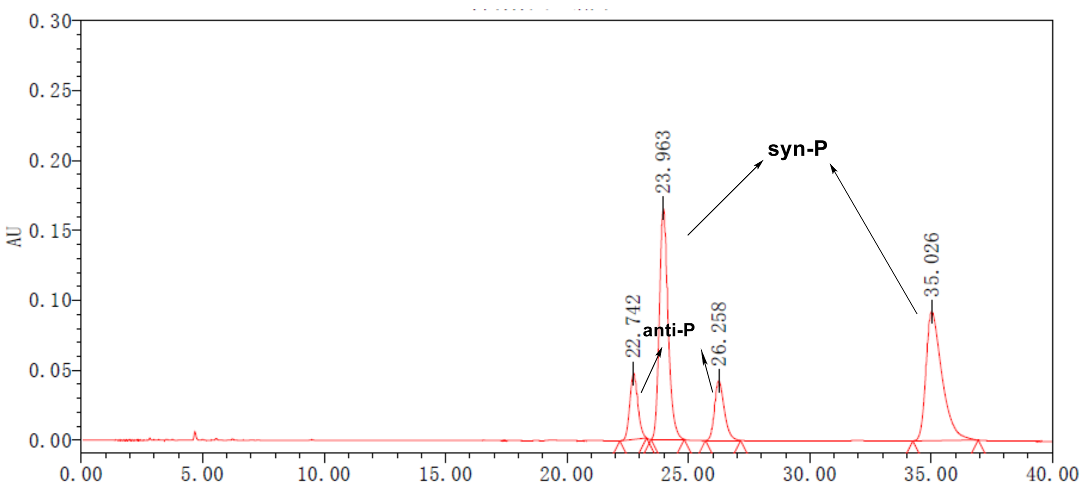

Entry	Retention Time/min	Area	Height	Area(%)
1	22.742	1103292	47006	10.22
2	23.963	4220677	165268	39.10
3	26.258	1201744	42685	11.13
4	35.026	4267537	92238	39.54

Supplementary Figure 80. Chiral HPLC analysis of racemic **38-b**

HPLC chromatogram of racemic anti-38-b

Condition: n-hexane/2-propanol =49:1

Flow rate =1.0 mL/min

λ= 254 nm

Chiral IA-3

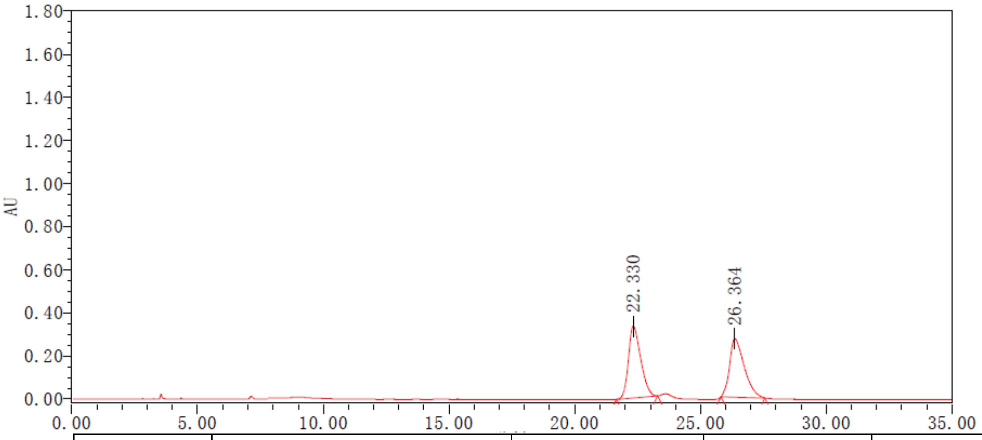

Entry	Retention Time/min	Area	Height	Area(%)
1	22.330	11113205	333082	50.34
2	26.364	10962316	271172	49.66

Supplementary Figure 81. Chiral HPLC analysis of racemic **anti-38-b**

HPLC chromatogram of chiral 38-b

Condition: n-hexane/2-propanol =49:1

Flow rate =1.0 mL/min

λ= 254 nm

Chiral IA-3

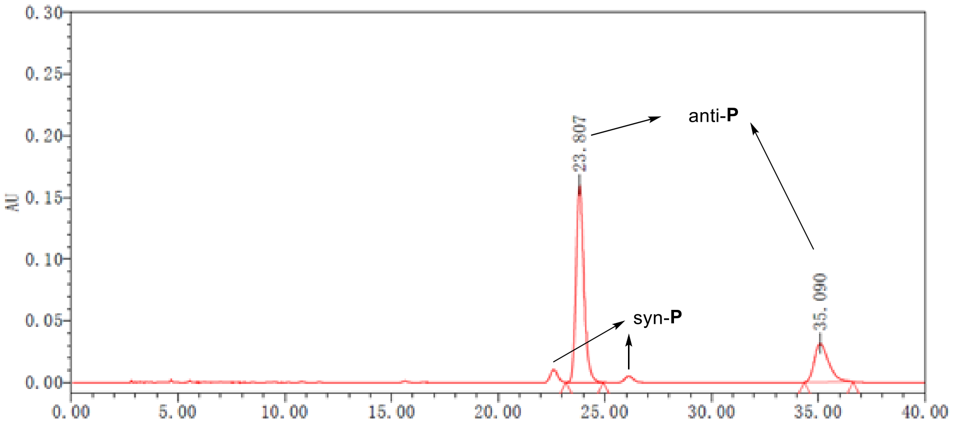

Entry	Retention Time/min	Area	Height	Area(%)
1	23.807	4122942	160048	73.78
2	35.090	1464980	31383	26.22

Supplementary Figure 82. Chiral HPLC analysis of chiral **38-b**

Tert-butyl (2S,3R)-2-((4-methylbenzyl)oxy)-3-(2-oxo-2-phenylethyl)-2,5-diphenylpent-4-ynoate (39)

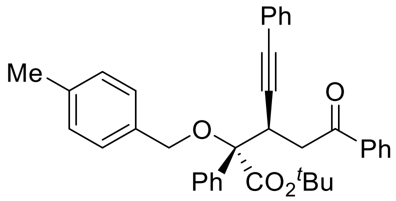

39

Colorless oil; 53.0 mg, 65% yield, >20:1 d.r., 91% *ee*, $[\alpha]_D^{20}$ = -12.61 (c = 0.1, CH_2Cl_2); ^{1}H NMR (500 MHz, $CDCl_3$) δ 7.85 (d, *J* = 7.5 Hz, 2H), 7.71 (d, *J* = 7.6 Hz, 2H), 7.52 (t, *J* = 7.3 Hz, 1H), 7.43 – 7.39 (m, 3H), 7.37 (d, *J* = 6.6 Hz, 1H), 7.35 (d, *J* = 7.8 Hz, 2H), 7.30 (d, *J* = 7.3 Hz, 2H), 7.26 – 7.22 (m, 4H), 7.18 (d, *J* = 7.8 Hz, 2H), 5.08 (d, *J* = 11.1 Hz, 1H), 4.92 (d, *J* = 11.1 Hz, 1H), 4.40 (dd, *J* = 10.3, 2.7 Hz, 1H), 3.36 (dd, *J* = 16.7, 10.4 Hz, 1H), 3.10 (dd, *J* = 16.6, 2.6 Hz, 1H), 2.36 (s, 3H), 1.52 (s, 9H). ^{13}C NMR (126 MHz, $CDCl_3$) δ 197.6, 169.6, 138.7, 137.1, 136.0, 136.0, 133.0, 131.4, 129.0, 128.5, 128.2, 128.2, 128.2, 128.1, 127.8, 127.7, 126.8, 123.5, 89.9, 85.7, 84.1, 82.7, 68.5, 39.2, 36.6, 28.1, 21.3. HRMS (ESI) $[M+Na]^+$ calcd for $C_{37}H_{36}O_4Na^+$, 567.2506, found 567.2509. (Chiral IE-3, λ= 254 nm, *n*-hexane/2-propanol= 97/3, Flow rate = 1.0 mL/min), t_R = 13.596 min(major), 30.967 min.

HPLC chromatogram of racemic 39

Condition: n-hexane/2-propanol =97:3

Flow rate =1.0 mL/min

λ= 254 nm

Chiral IE-3

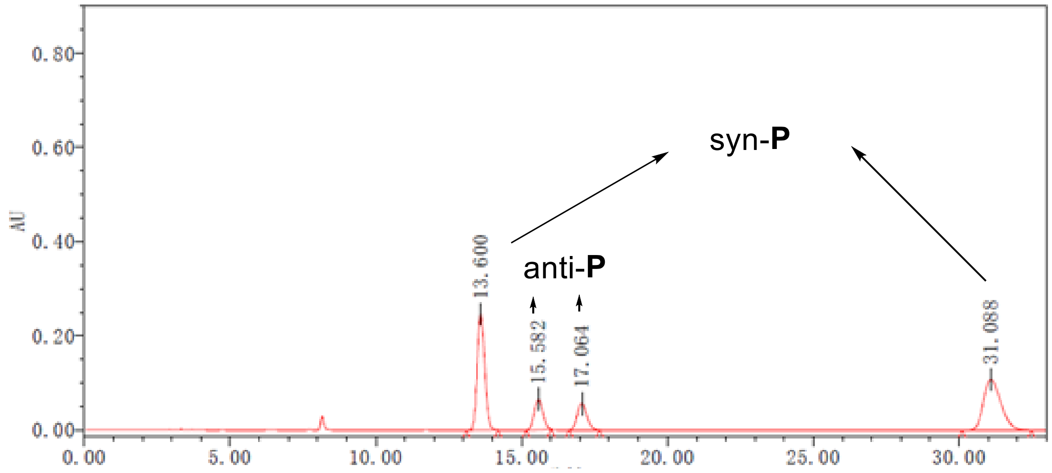

Entry	Retention Time/min	Area	Height	Area(%)
1	13.600	4423931	246453	38.31
2	15.582	1316349	64450	11.40
3	17.064	1316626	56624	11.40
4	31.088	4489356	108334	38.88

Supplementary Figure 83. Chiral HPLC analysis of racemic **39**

HPLC chromatogram of chiral 39

Condition: n-hexane/2-propanol =97:3

Flow rate =1.0 mL/min

λ= 254 nm

Chiral IE-3

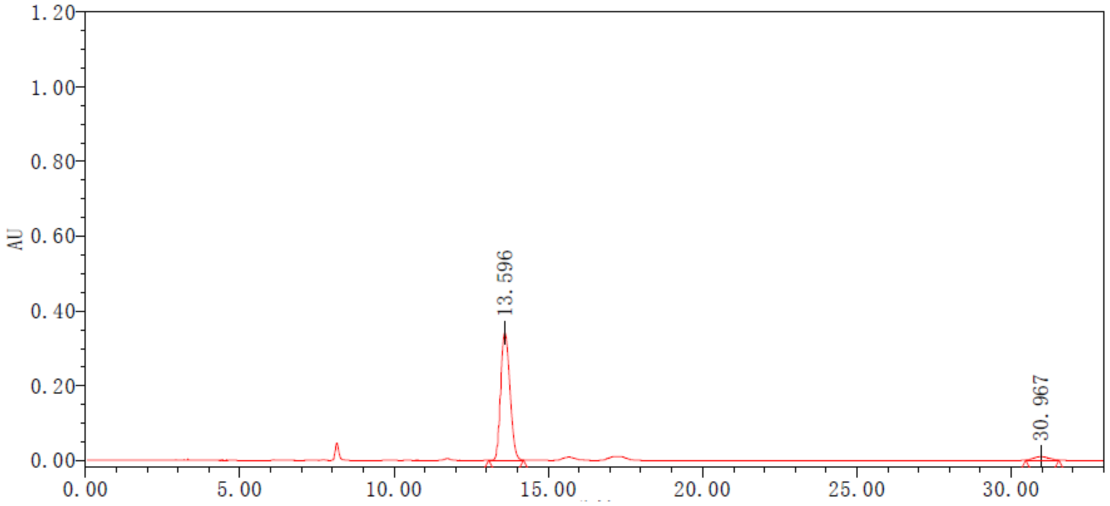

The larger version of HPLC chromatogram of chiral 39

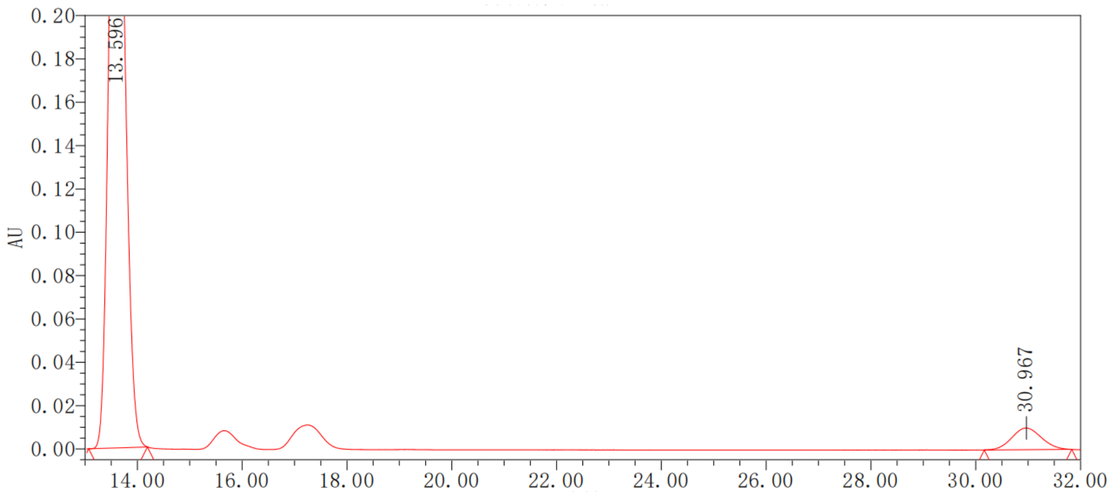

Entry	Retention Time/min	Area	Height	Area(%)
1	13.596	7092744	341401	95.51
2	30.967	392804	10012	4.49

Supplementary Figure 84. Chiral HPLC analysis of chiral **39**

Ethyl (2S,3R)-2-((4-methylbenzyl)oxy)-3-(2-oxo-2-phenylethyl)-2,5-diphenylpent-4-ynoate (*syn*-39-b)

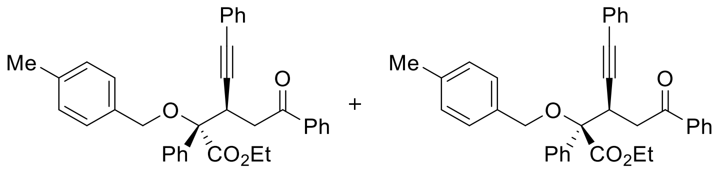

syn-**39-b**(major) *anti*-**39-b**(minor)

Colorless oil; Combined in 50% yield (35.6mg), 85:15 d.r, 33% ee. Composite NMR signals of *syn*-**39-b** (major) and *anti*-**39-b** (minor) (The ratio of two diasteroisomers in ^{1}H NMR is *syn*-**39-b** : *anti*-**39-b** (minor) = 3.00 : 0.60). ^{1}H NMR (500 MHz, $CDCl_3$) δ 7.85 (comp, 2.47H), 7.70 (d, *J* = 7.6 Hz, 2.06H), 7.66 (d, *J* = 7.4 Hz, 0.43H), 7.52 (comp, 1.35H), 7.39 (comp, 7.58H), 7.33 – 7.28 (comp, 1.54H), 7.23 (comp, 6.53H), 7.18 (d, *J* = 7.8 Hz, 2.07H), 7.15 (d, *J* = 7.9 Hz, 0.42H), 5.08 (d, *J* = 11.1 Hz, 1H), 4.88 (d, *J* = 11.1 Hz, 1H), 4.55 (q, *J* = 11.4 Hz, 0.43H), 4.46 (dd, *J* = 10.3, 2.7 Hz, 1H), 4.38 – 4.28 (comp, 2.52H), 3.33 (dd, *J* = 16.6, 10.3 Hz, 1.02H), 3.21 (dd, *J* = 16.5, 3.1 Hz, 0.21H), 3.13 (dd, *J* = 16.7, 2.9 Hz, 1.01H), 3.04 (dd, *J* = 16.5, 10.3 Hz, 0.21H), 2.36 (s, 3H), 2.34 (s, 0.6H), 1.32 (comp, 3.60H). ^{13}C NMR (126 MHz, $CDCl_3$) δ 197.5, 170.8, 138.2, 137.2, 136.9, 135.7, 133.1, 131.5, 131.5, 129.0, 128.5, 128.3, 128.3, 128.2, 128.1, 127.9, 127.7, 127.3, 126.8, 123.4, 89.4, 86.6, 85.8, 84.3, 69.4, 68.5, 67.9, 61.7, 39.3, 36.6, 29.7, 21.2, 14.3. HRMS (ESI) $[M+Na]^+$ calcd for $C_{35}H_{32}O_4Na^+$, 539.2193, found 539.2197. (Chiral IA, λ= 254 nm, *n*-hexane/2-propanol= 19/1, Flow rate = 1.0 mL/min), t_R = 12.128 min (major), 16.115 min.

HPLC chromatogram of racemic 39-b

Condition: n-hexane/2-propanol = 19:1

Flow rate =1.0 mL/min

λ= 254 nm

Chiral IA

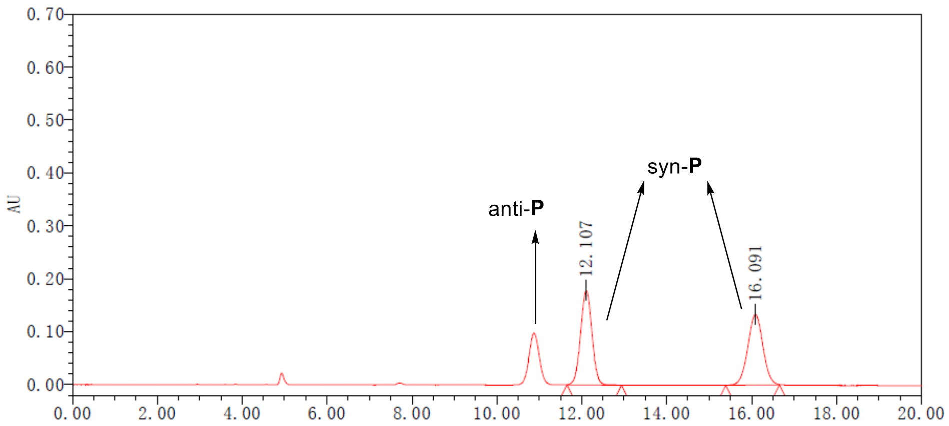

Entry	Retention Time/min	Area	Height	Area(%)
1	12.107	3290381	178678	50.06
2	16.091	3282907	133193	49.94

Supplementary Figure 85. Chiral HPLC analysis of racemic **39-b**

HPLC chromatogram of chiral 39-b

Condition: n-hexane/2-propanol =19:1

Flow rate =1.0 mL/min

λ= 254 nm

Chiral IA

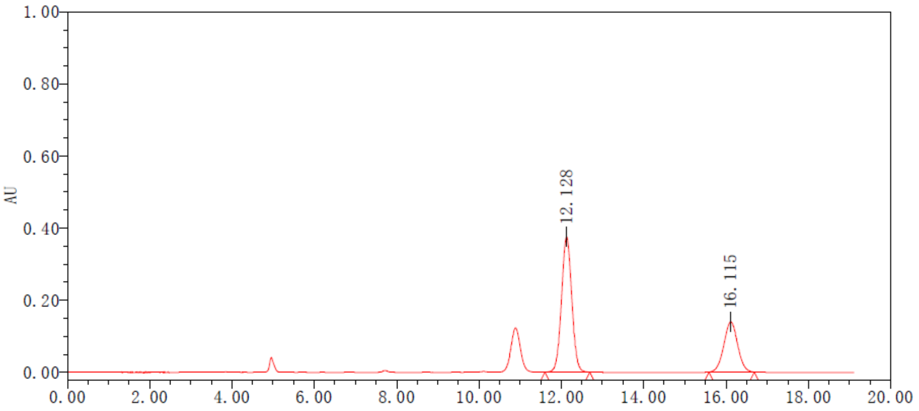

Entry	Retention Time/min	Area	Height	Area(%)
1	12.128	6442648	375471	66.50
2	16.115	3244931	139136	33.50

Supplementary Figure 86. Chiral HPLC analysis of chiral **39-b**

Isopropyl (2S,3R)-2-((4-methylbenzyl)oxy)-3-(2-oxo-2-phenylethyl)-2,5-diphenylpent-4-ynoate (*syn*-39-c)

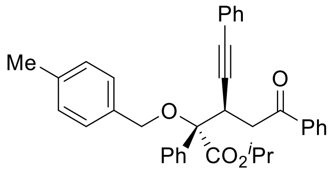

syn-**39-c**

Colorless oil; 54% yield (32.2mg), 89:11 d.r, 60% ee. ^{1}H NMR (500 MHz, $CDCl_3$) δ 7.85 (d, *J* = 7.7 Hz, 2H), 7.71 (d, *J* = 7.5 Hz, 2H), 7.51 (t, *J* = 7.4 Hz, 1H), 7.42 (d, *J* = 5.4 Hz, 2H), 7.40 (d, *J* = 4.9 Hz, 2H), 7.37 (d, *J* = 7.1 Hz, 1H), 7.34 (d, *J* = 7.8 Hz, 2H), 7.30 (d, *J* = 7.1 Hz, 1H), 7.25 – 7.22 (m, 4H), 7.18 (d, *J* = 7.8 Hz, 2H), 5.21 (dt, *J* = 12.5, 6.3 Hz, 1H), 5.09 (d, *J* = 11.1 Hz, 1H), 4.92 (d, *J* = 11.1 Hz, 1H), 4.45 (dd, *J* = 10.3, 2.9 Hz, 1H), 3.35 (dd, *J* = 16.7, 10.4 Hz, 1H), 3.10 (dd, *J* = 16.7, 2.9 Hz, 1H), 2.37 (s, 3H), 1.31 (d, *J* = 6.3 Hz, 3H), 1.29 (d, *J* = 6.1 Hz, 3H). ^{13}C NMR (126 MHz, $CDCl_3$) δ 197.5, 170.1, 138.4, 137.2, 135.8, 133.1, 131.4, 129.0, 128.5, 128.2, 128.1, 127.9, 127.8, 127.3, 126.8, 123.4, 89.6, 85.6, 84.2, 69.6, 68.7, 39.3, 36.5, 21.8, 21.8, 21.2. HRMS (ESI) $[M+Na]^+$ calcd for $C_{36}H_{34}O_4Na^+$, 553.2349, found 553.2351. (Chiral IA, λ= 254 nm, *n*-hexane/2-propanol= 49/1, Flow rate = 1.0 mL/min), t_R = 15.207 min (major), 26.479 min.

HPLC chromatogram of racemic 39-c

Condition: n-hexane/2-propanol =49:1

Flow rate =1.0 mL/min

λ= 254 nm

Chiral IA

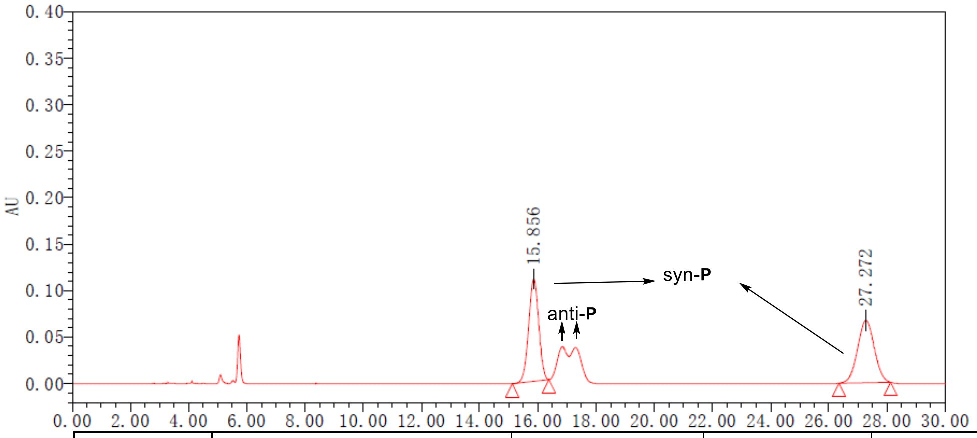

Entry	Retention Time/min	Area	Height	Area(%)
1	15.856	2647839	110026	49.75
2	27.272	2674916	66984	50.25

Supplementary Figure 87. Chiral HPLC analysis of racemic **39-c**

HPLC chromatogram of chiral 39-c

Condition: n-hexane/2-propanol =49:1

Flow rate =1.0 mL/min

λ= 254 nm

Chiral IA

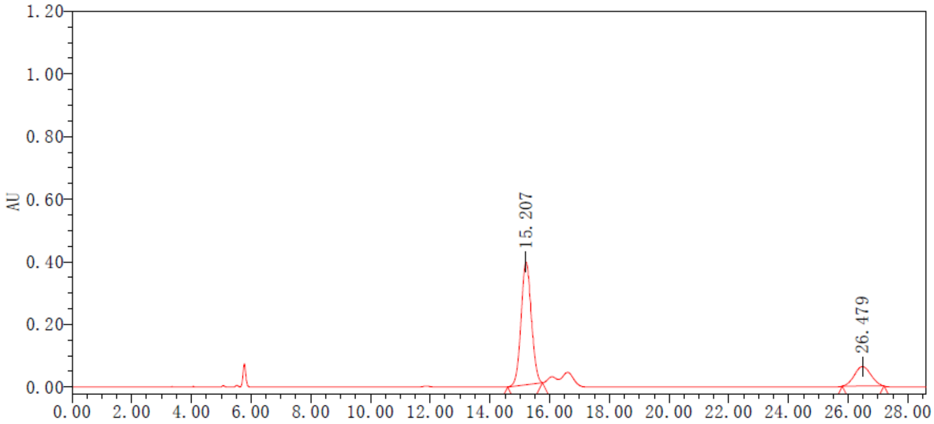

Entry	Retention Time/min	Area	Height	Area(%)
1	15.207	9413437	391747	79.70
2	26.479	2398163	62428	20.30

Supplementary Figure 88. Chiral HPLC analysis of chiral **39-c**

Tert-butyl (2S,3R)-3-(2-oxo-2-phenylethyl)-2,5-diphenyl-2-((4-(trifluoromethyl)benzyl)oxy)pent-4-ynoate(40)

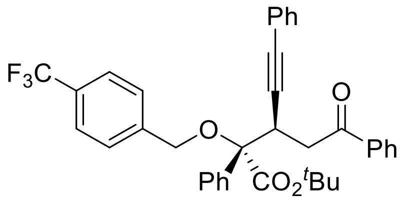

40

Colorless oil; 53.9 mg, 60% yield, >20:1 d.r., 96% *ee*, $[\alpha]_D^{20}$ = -13.91 (c = 0.1, CH_2Cl_2); ^{1}H NMR (400 MHz, $CDCl_3$) δ 7.87 – 7.83 (m, 2H), 7.72 – 7.68 (m, 2H), 7.64 – 7.59 (m, 4H), 7.53 (t, *J* = 7.4 Hz, 1H), 7.44 – 7.36 (m, 4H), 7.33 (d, *J* = 7.1 Hz, 1H), 7.25 (d, *J* = 6.3 Hz, 5H), 5.16 (d, *J* = 12.3 Hz, 1H), 5.02 (d, *J* = 12.2 Hz, 1H), 4.44 (dd, *J* = 10.1, 3.1 Hz, 1H), 3.34 (dd, *J* = 16.7, 10.1 Hz, 1H), 3.11 (dd, *J* = 16.7, 3.0 Hz, 1H), 1.51 (s, 9H). ^{13}C NMR (101 MHz, $CDCl_3$) δ 197.3, 169.4, 143.1, 137.5 (d, *J* = 130.6 Hz), 133.2, 131.4, 131.4, 128.6, 128.5, 128.4, 128.2, 128.2, 128.0, 128.0, 127.5, 126.7, 125.2 (q, *J* = 3.7 Hz), 123.3, 89.6, 86.0, 84.2, 83.1, 68.0, 39.2, 36.6, 28.1. ^{19}F NMR (376 MHz, $CDCl_3$) δ -62.38. HRMS (ESI) $[M+Na]^+$ calcd for $C_{37}H_{33}O_4F_3Na^+$, 621.2223, found 621.2225. (Chiral IA, λ= 254 nm, *n*-hexane/2-propanol= 19/1, Flow rate = 1.0 mL/min), t_R = 9.784 min(major), 15.402 min.

HPLC chromatogram of racemic 40

Condition: n-hexane/2-propanol =19:1

Flow rate =1.0 mL/min

λ= 254 nm

Chiral IA

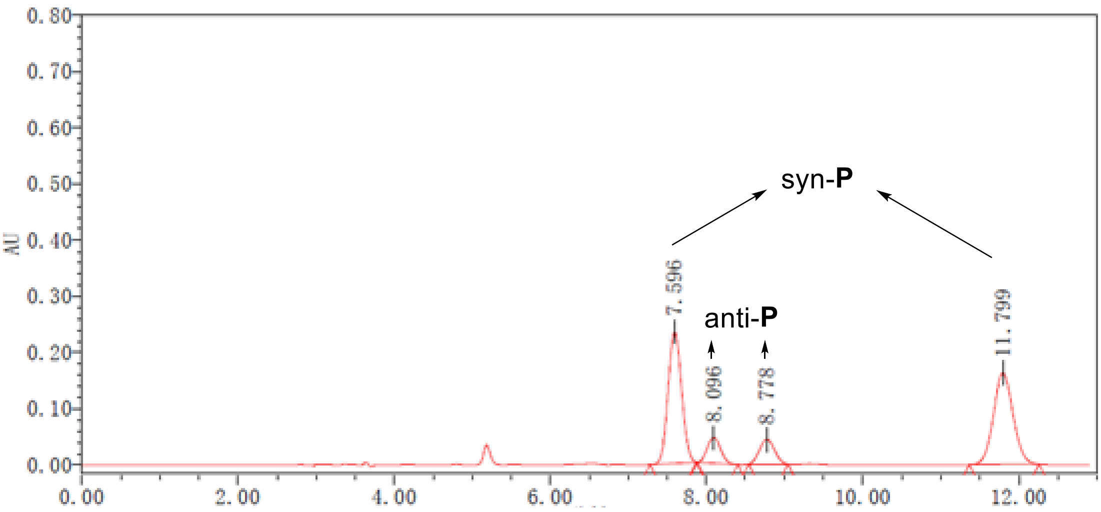

Entry	Retention Time/min	Area	Height	Area(%)
1	7.596	2821506	233593	41.39
2	8.096	550314	45273	8.07
3	8.778	584575	43290	8.58
4	11.799	2860608	162948	41.96

Supplementary Figure 89. Chiral HPLC analysis of racemic **40**

HPLC chromatogram of chiral 40

Condition: n-hexane/2-propanol =19:1

Flow rate =1.0 mL/min

λ= 254 nm

Chiral IA

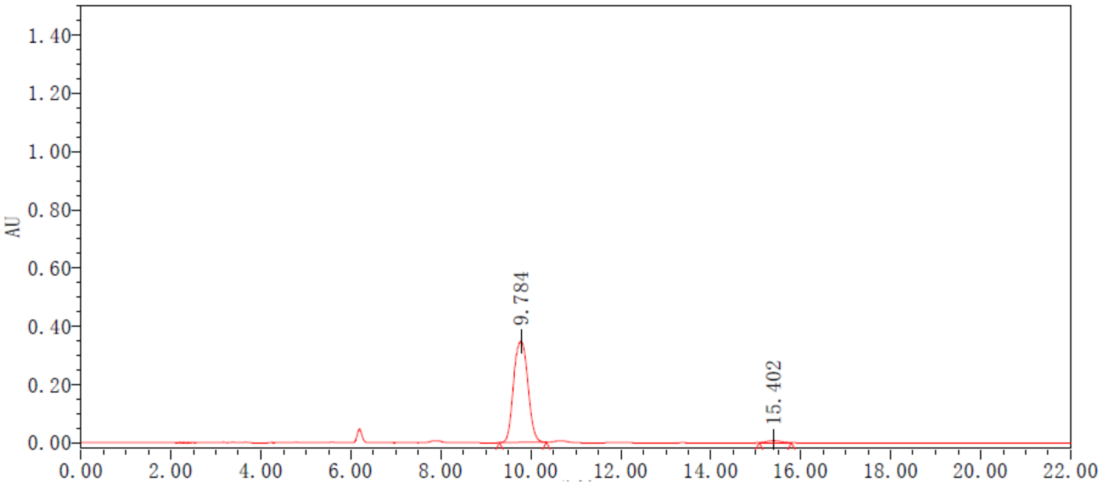

The larger version of HPLC chromatogram of chiral 40

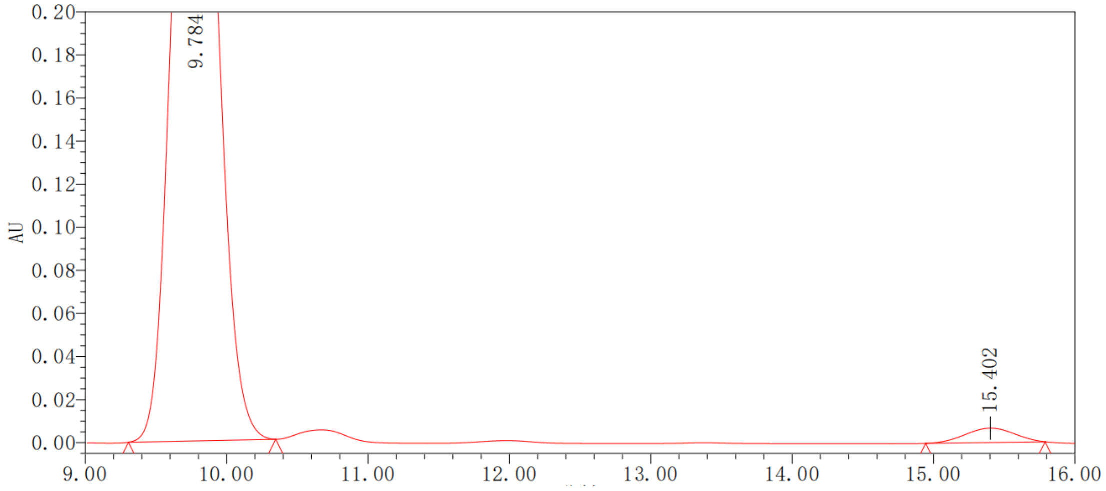

Entry	Retention Time/min	Area	Height	Area(%)
1	9.784	7756472	347620	98.21
2	15.402	141404	6245	1.79

Supplementary Figure 90. Chiral HPLC analysis of chiral **40**

Tert-butyl (2S,3R)-2-((4-ethynylbenzyl)oxy)-3-(2-oxo-2-phenylethyl)-2,5-diphenylpent-4-ynoate (41)

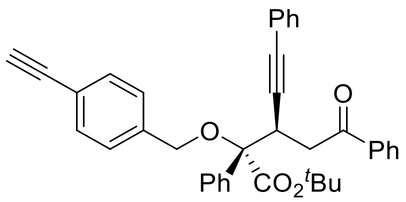

41

Colorless oil; 45.6 mg, 55% yield, >20:1 d.r., 96% *ee*, $[\alpha]_D^{20}$ = -12.11 (c = 0.1, CH_2Cl_2); ^1H NMR (400 MHz, $CDCl_3$) δ 7.86 – 7.81 (m, 2H), 7.72 – 7.68 (m, 2H), 7.53 – 7.49 (m, 1H), 7.47 (d, *J* = 4.7 Hz, 3H), 7.43 – 7.34 (m, 5H), 7.32 (d, *J* = 7.1 Hz, 1H), 7.24 (d, *J* = 9.1 Hz, 5H), 5.10 (d, *J* = 12.1 Hz, 1H), 4.95 (d, *J* = 12.1 Hz, 1H), 4.41 (dd, *J* = 10.1, 3.1 Hz, 1H), 3.33 (dd, *J* = 16.6, 10.2 Hz, 1H), 3.10 (dd, *J* = 16.6, 3.1 Hz, 1H), 3.07 (s, 1H), 1.51 (s, 9H). ^{13}C NMR (126 MHz, $CDCl_3$) δ 197.5, 169.4, 139.9, 138.3, 136.9, 133.1, 132.1, 131.4, 128.5, 128.3, 128.2, 128.2, 128.2, 127.9, 127.3, 126.7, 123.4, 121.0, 89.7, 85.9, 84.1, 83.7, 82.9, 68.3, 39.2, 36.7, 28.1. HRMS (ESI) $[M+Na]^+$ calcd for $C_{38}H_{34}O_4Na^+$, 577.2349, found 577.2346. (Chiral IA-3, λ= 254 nm, *n*-hexane/2-propanol= 49/1, Flow rate = 1.0 mL/min), t_R = 12.667 min(major), 26.065 min.

HPLC chromatogram of racemic 41

Condition: n-hexane/2-propanol =49:1

Flow rate =1.0 mL/min

λ= 254 nm

Chiral IA-3

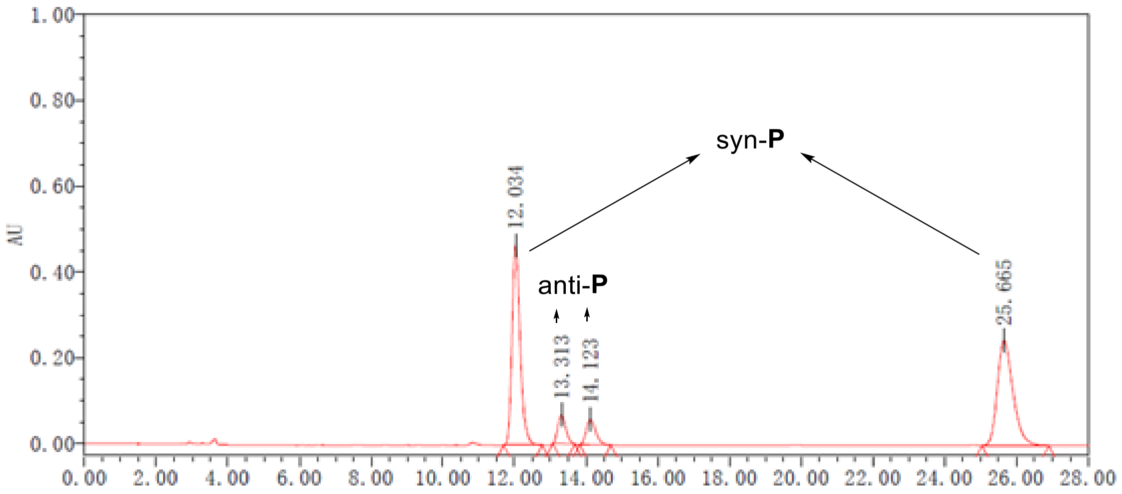

Entry	Retention Time/min	Area	Height	Area(%)
1	12.034	7234797	465971	43.58
2	13.313	1048221	66826	6.31
3	14.123	1047789	58802	6.31
4	25.665	7269537	244342	43.79

Supplementary Figure 91. Chiral HPLC analysis of racemic **41**

HPLC chromatogram of chiral 41

Condition: n-hexane/2-propanol =49:1

Flow rate =1.0 mL/min

λ= 254 nm

Chiral IA-3

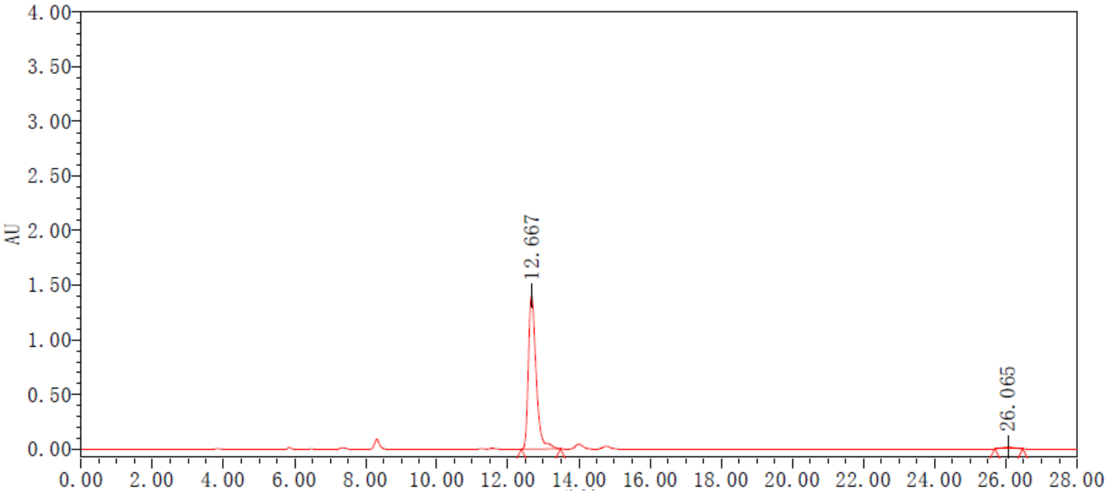

The larger version of HPLC chromatogram of chiral 41

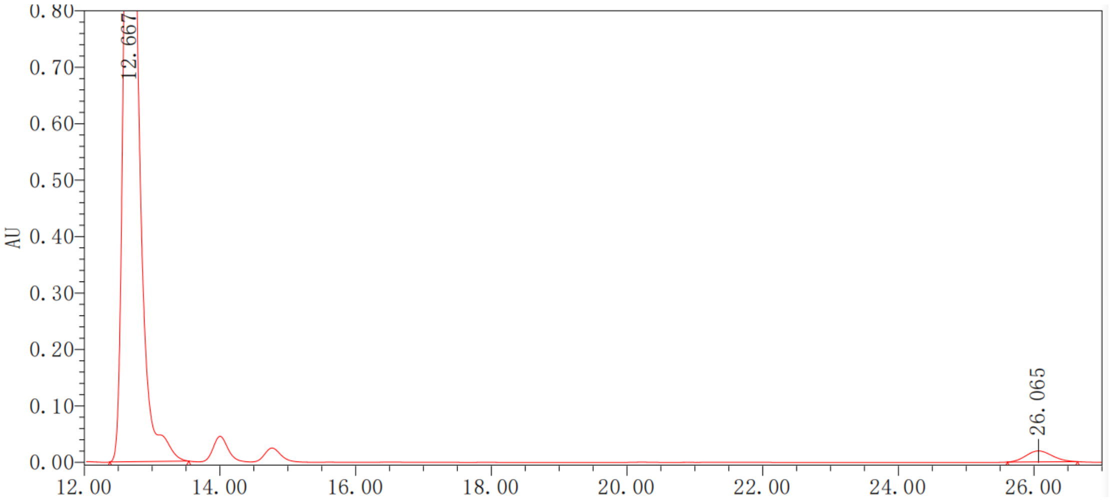

Entry	Retention Time/min	Area	Height	Area(%)
1	12.667	20886858	1407348	98.01
2	26.065	424180	17606	1.99

Supplementary Figure 92. Chiral HPLC analysis of chiral **41**

Tert-butyl (2S,3R)-2-((2-bromobenzyl)oxy)-3-(2-oxo-2-phenylethyl)-2,5-diphenylpent-4-ynoate (42)

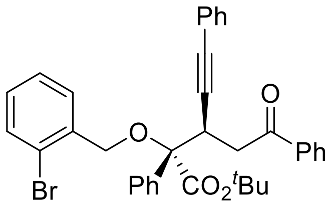

42

Colorless oil; 45.6 mg, 50% yield, 92:8 d.r., 88% *ee*, $[\alpha]_D^{20}$ = -13.11 (c = 0.1, CH_2Cl_2); ^1H NMR (500 MHz, $CDCl_3$) δ 7.92 – 7.84 (m, 3H), 7.74 (d, *J* = 7.5 Hz, 2H), 7.55 – 7.50 (m, 2H), 7.43 – 7.36 (m, 5H), 7.34 (d, *J* = 6.3 Hz, 1H), 7.31 (dd, *J* = 7.5, 2.6 Hz, 2H), 7.23 (dt, *J* = 2.6, 2.2 Hz, 3H), 7.16 (t, *J* = 8.3 Hz, 1H), 5.27 (d, *J* = 13.1 Hz, 1H), 5.04 (d, *J* = 13.1 Hz, 1H), 4.44 (dd, *J* = 10.2, 3.0 Hz, 1H), 3.45 (dd, *J* = 16.8, 10.3 Hz, 1H), 3.10 (dd, *J* = 16.7, 2.9 Hz, 1H), 1.48 (s, 9H). ^{13}C NMR (126 MHz, $CDCl_3$) δ 197.5, 169.3, 138.5, 138.4, 136.9, 133.1, 132.2, 131.6, 129.0, 128.6, 128.5, 128.3, 128.2, 128.1, 128.1, 127.8, 127.5, 126.6, 123.4, 121.9, 89.7, 86.1, 84.3, 82.9, 68.2, 39.2, 36.7, 28.0. HRMS (ESI) $[M+Na]^+$ calcd for $C_{36}H_{33}O_4BrNa^+$, 631.1454, found 631.1459. (Chiral IE-3, λ= 254 nm, *n*-hexane/2-propanol= 97/3, Flow rate = 1.0 mL/min), t_R = 14.832 min(major), 33.652 min.

HPLC chromatogram of racemic 42

Condition: n-hexane/2-propanol =97:3

Flow rate =1.0 mL/min

λ= 254 nm

Chiral IE-3

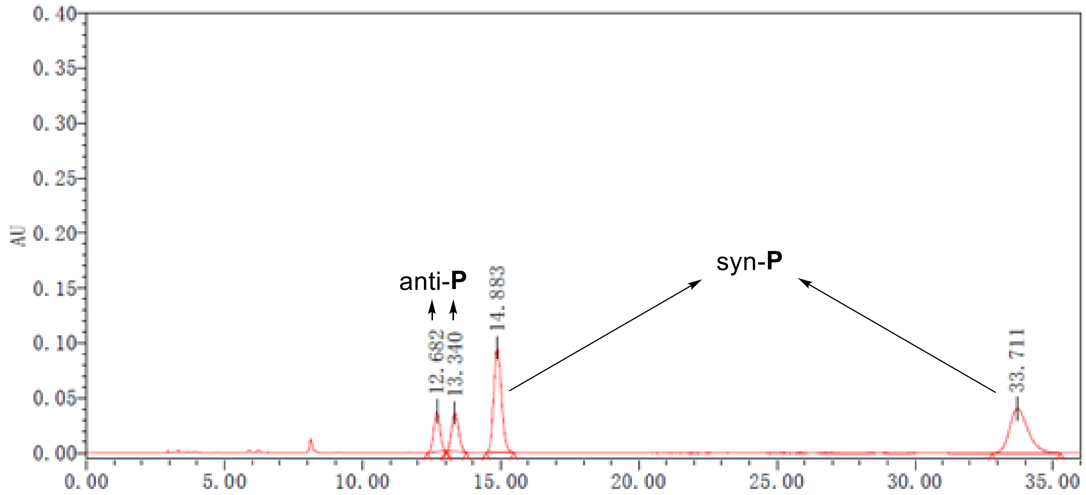

Entry	Retention Time/min	Area	Height	Area(%)
1	12.682	596062	37195	11.95
2	13.340	602382	34973	12.08
3	14.883	1894469	95327	38.00
4	33.711	1893011	40773	37.97

Supplementary Figure 93. Chiral HPLC analysis of racemic **42**

HPLC chromatogram of chiral 42

Condition: n-hexane/2-propanol =97:3

Flow rate =1.0 mL/min

λ= 254 nm

Chiral IE-3

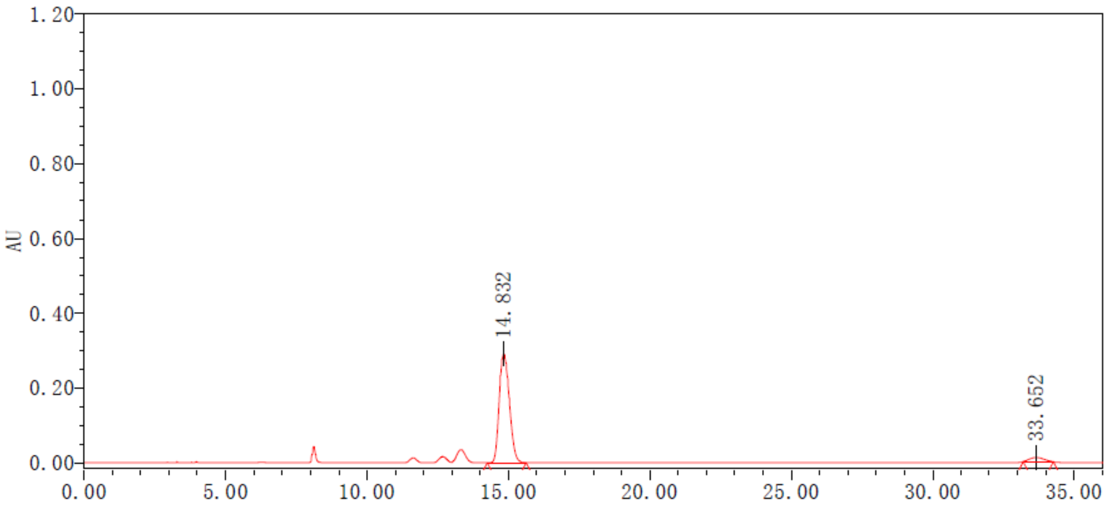

The larger version of HPLC chromatogram of chiral 42

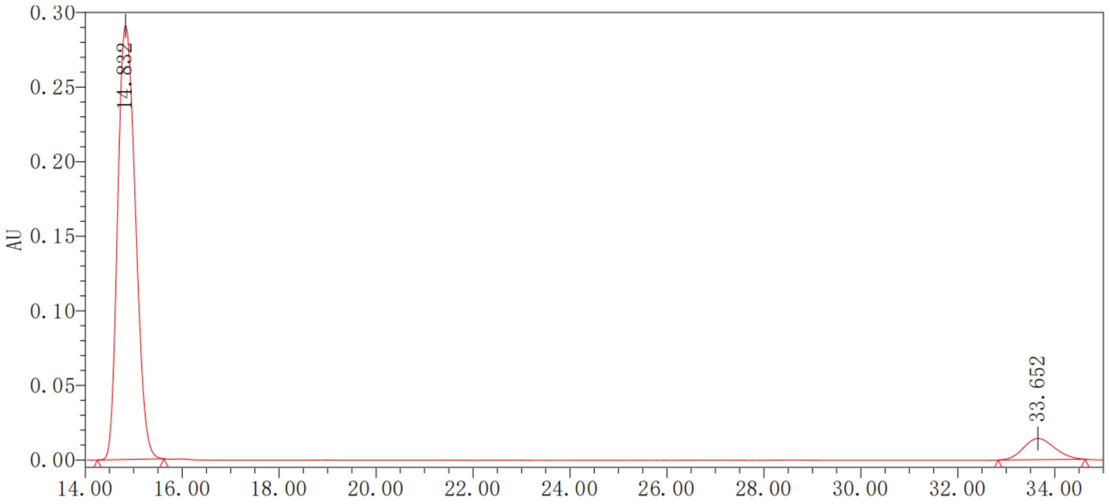

Entry	Retention Time/min	Area	Height	Area(%)
1	14.832	6906065	290795	94.12
2	33.652	431065	11047	5.88

Supplementary Figure 94. Chiral HPLC analysis of chiral **42**

Tert-butyl (2S,3R)-2-(furan-2-ylmethoxy)-3-(2-oxo-2-phenylethyl)-2,5-diphenylpent-4-ynoate(43)

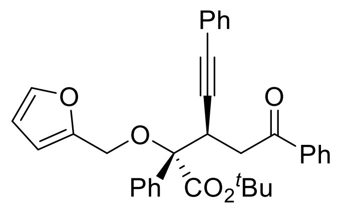

43

Colorless oil; 54.6 mg, 70% yield, >20:1 d.r., 99% *ee*; ^{1}H NMR (400 MHz, $CDCl_3$) δ 7.90 (d, *J* = 7.4 Hz, 2H), 7.70 (d, *J* = 7.4 Hz, 2H), 7.53 (t, *J* = 7.3 Hz, 1H), 7.42 (dd, *J* = 13.2, 5.4 Hz, 3H), 7.35 (t, *J* = 7.4 Hz, 2H), 7.30 (d, *J* = 7.1 Hz, 1H), 7.24 (d, *J* = 13.0 Hz, 5H), 6.38 (dd, *J* = 9.0, 2.3 Hz, 2H), 5.16 (d, *J* = 12.1 Hz, 1H), 4.91 (d, *J* = 12.1 Hz, 1H), 4.38 (dd, *J* = 10.3, 2.7 Hz, 1H), 3.46 (dd, *J* = 16.7, 10.4 Hz, 1H), 3.08 (dd, *J* = 16.7, 2.7 Hz, 1H), 1.53 (s, 9H). ^{13}C NMR (101 MHz, $CDCl_3$) δ 197.6, 169.3, 152.3, 142.6, 138.3, 137.0, 133.1, 131.4, 128.5, 128.3, 128.2, 128.2, 128.1, 127.8, 126.8, 123.4, 110.3, 108.9, 89.8, 85.5, 84.1, 82.9, 61.2, 39.0, 36.5, 28.1. HRMS (ESI) $[M+Na]^+$ calcd for $C_{34}H_{32}O_5Na^+$, 543.2140, found 543.2142. (Chiral IA, λ= 254 nm, *n*-hexane/2-propanol= 19/1, Flow rate = 1.0 mL/min), t_R = 7.586 min(major), 15.075 min.

HPLC chromatogram of racemic 43

Condition: n-hexane/2-propanol =19:1

Flow rate =1.0 mL/min

λ= 254 nm

Chiral IA

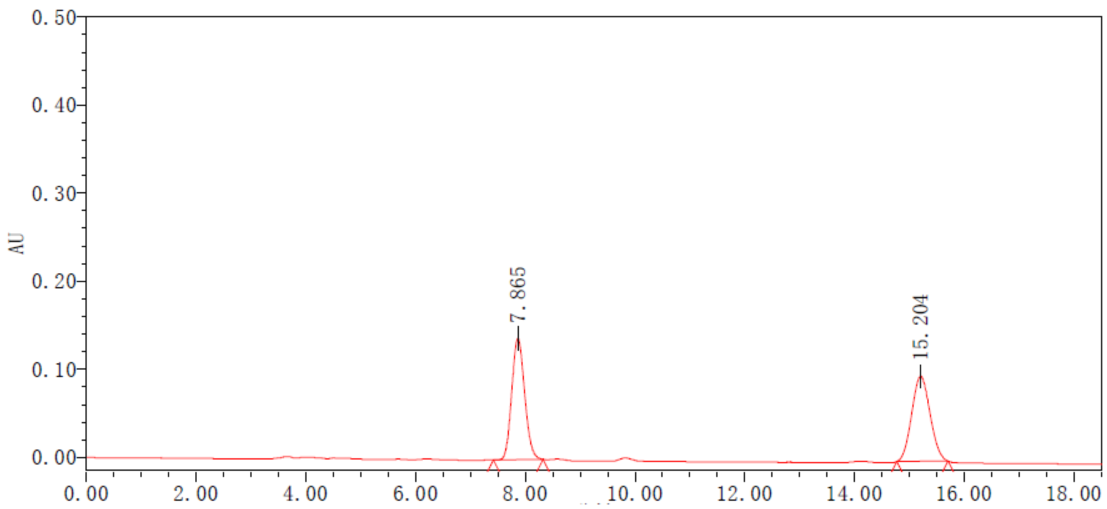

Entry	Retention Time/min	Area	Height	Area(%)
1	7.865	2225887	137741	49.74
2	15.204	2249077	96528	50.26

Supplementary Figure 95. Chiral HPLC analysis of racemic **43**

HPLC chromatogram of chiral 43

Condition: n-hexane/2-propanol =19:1

Flow rate =1.0 mL/min

λ= 254 nm

Chiral IA

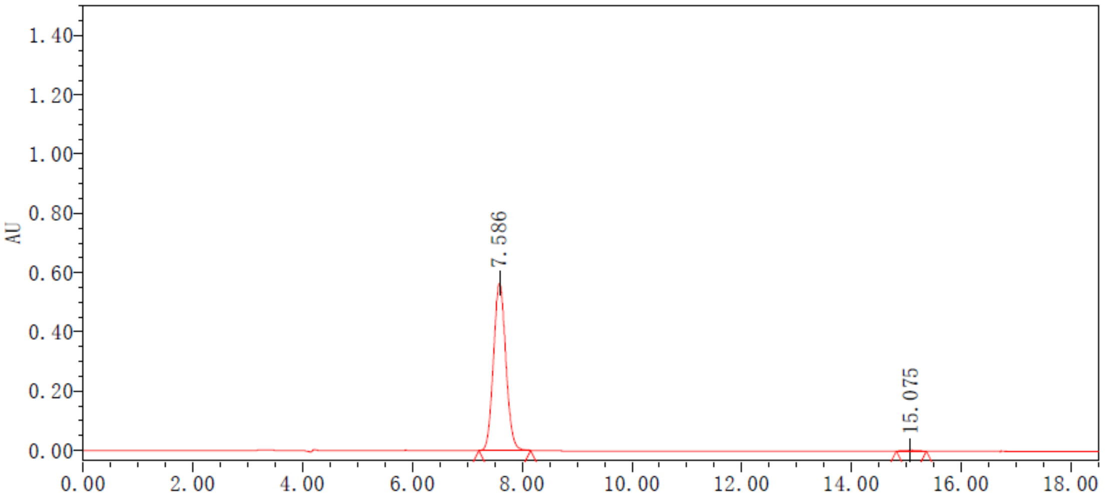

The larger version of HPLC chromatogram of chiral 43

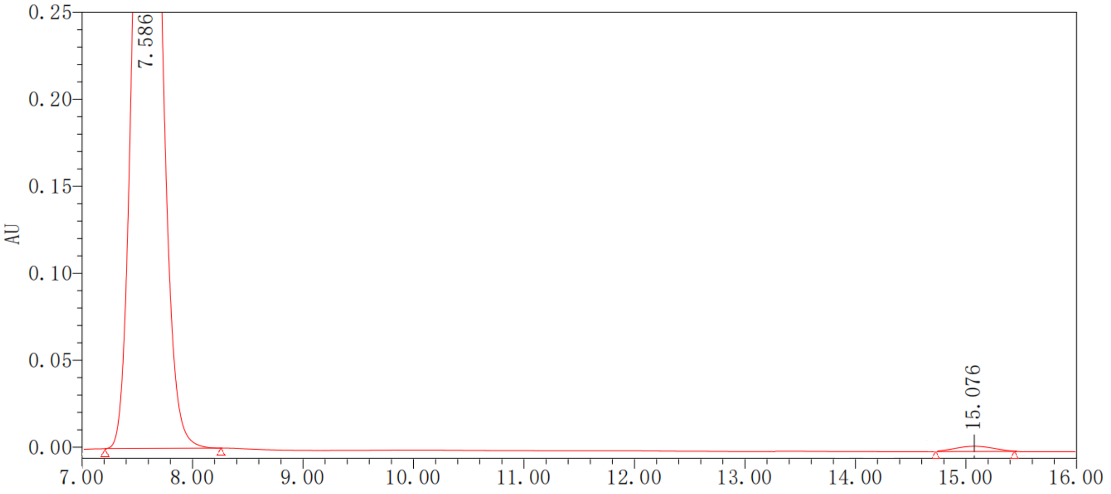

Entry	Retention Time/min	Area	Height	Area(%)
1	7.586	8927372	564755	99.51
2	15.075	44057	2263	0.49

Supplementary Figure 96. Chiral HPLC analysis of chiral **43**

Tert-butyl (2S,3R)-3-(2-oxo-2-phenylethyl)-2,5-diphenyl-2-(thiophen-2-ylmethoxy)pent-4-ynoate (44)

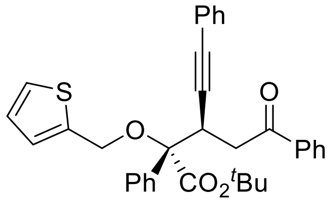

44

Colorless oil; 57.7 mg, 72% yield, >20:1 d.r., 99% *ee*; ^{1}H NMR (400 MHz, $CDCl_3$) δ 7.91 – 7.84 (m, 2H), 7.77 – 7.68 (m, 2H), 7.52 (t, *J* = 7.4 Hz, 1H), 7.44 – 7.33 (m, 4H), 7.32 – 7.29 (m, 2H), 7.26 – 7.19 (m, 5H), 7.06 (d, *J* = 2.7 Hz, 1H), 6.99 (dd, *J* = 5.0, 3.5 Hz, 1H), 5.37 (d, *J* = 11.7 Hz, 1H), 5.14 (d, *J* = 11.7 Hz, 1H), 4.41 (dd, *J* = 10.4, 2.8 Hz, 1H), 3.42 (dd, *J* = 16.8, 10.4 Hz, 1H), 3.09 (dd, *J* = 16.8, 2.8 Hz, 1H), 1.53 (s, 9H). ^{13}C NMR (101 MHz, $CDCl_3$) δ 197.5, 169.3, 141.9, 138.4, 136.9, 133.1, 131.4, 128.6, 128.3, 128.3, 128.2, 128.2, 127.9, 126.7, 126.5, 125.6, 125.5, 123.4, 89.7, 85.9, 84.1, 83.0, 64.1, 39.2, 36.5, 28.1. HRMS (ESI) $[M+Na]^+$ calcd for $C_{34}H_{32}O_4SNa^+$, 559.1914, found 559.1910. (Chiral IE-3, λ= 254 nm, *n*-hexane/2-propanol= 19/1, Flow rate = 1.0 mL/min), t_R = 10.844 min(major), 26.285 min.

HPLC chromatogram of racemic 44

Condition: n-hexane/2-propanol =19:1

Flow rate =1.0 mL/min

λ= 254 nm

Chiral IE-3

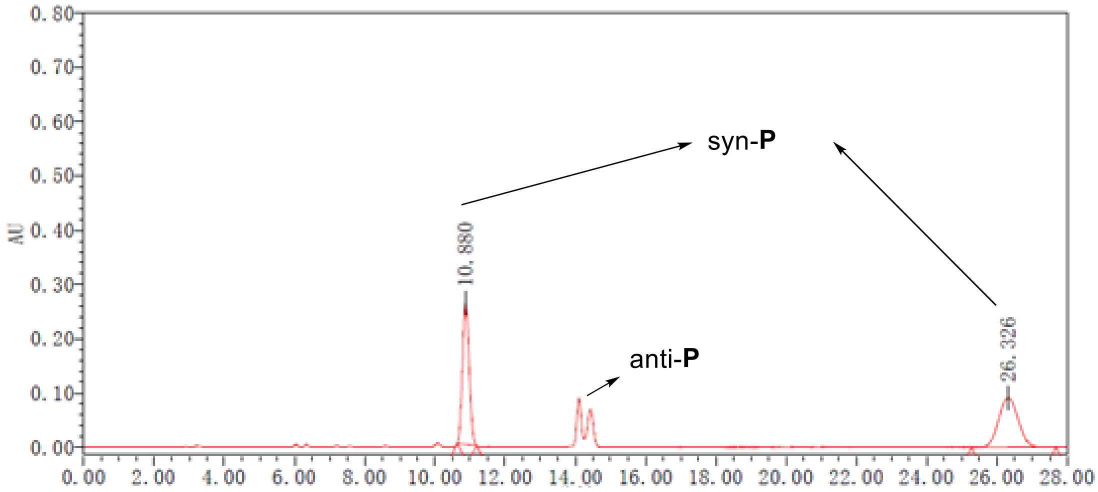

Entry	Retention Time/min	Area	Height	Area(%)
1	10.880	3522490	261335	50.08
2	26.326	3511789	91478	49.92

Supplementary Figure 97. Chiral HPLC analysis of racemic **44**

HPLC chromatogram of chiral 44

Condition: n-hexane/2-propanol =19:1

Flow rate =1.0 mL/min

λ= 254 nm

Chiral IE-3

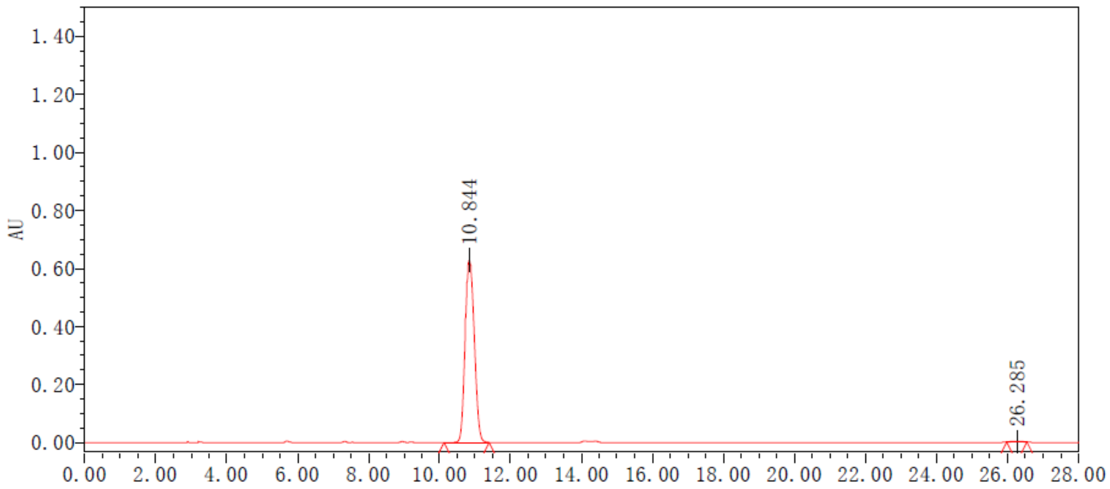

The larger version of HPLC chromatogram of chiral 44

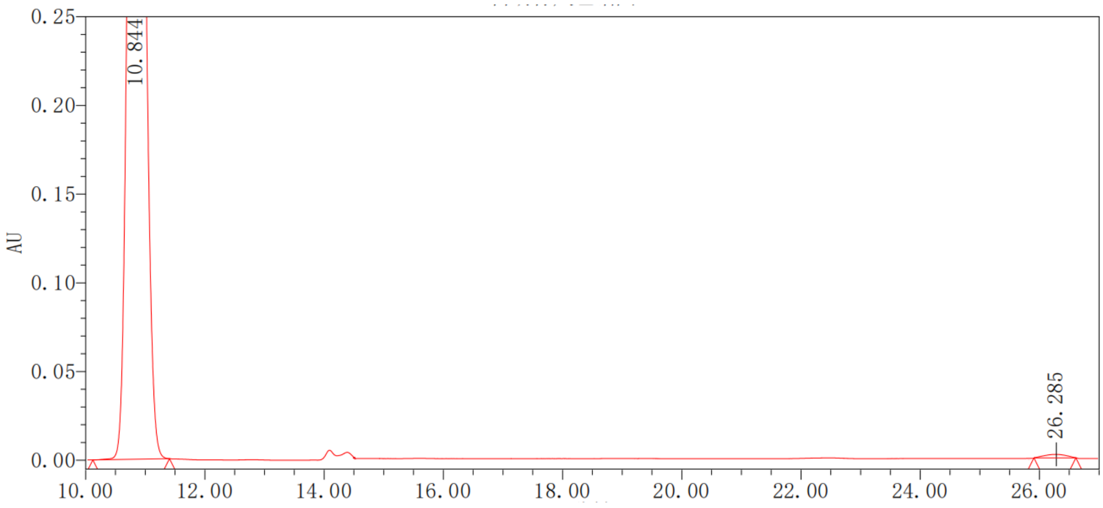

Entry	Retention Time/min	Area	Height	Area(%)
1	10.844	11578063	628323	99.52
2	26.285	31953	1475	0.48

Supplementary Figure 98. Chiral HPLC analysis of chiral **44**

Tert-butyl (2S,3R)-2-(benzofuran-2-ylmethoxy)-3-(2-oxo-2-phenylethyl)-2,5-diphenylpent-4-ynoate(45)

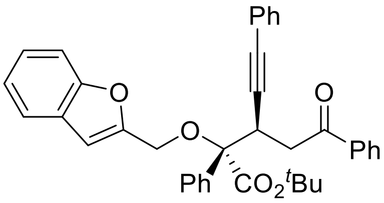

45

Colorless oil; 61.5 mg, 72% yield, >20:1 d.r., 99% *ee*; ^{1}H NMR (400 MHz, $CDCl_3$) δ 7.96 – 7.91 (m, 2H), 7.80 – 7.71 (m, 2H), 7.60 (dd, *J* = 7.6, 0.7 Hz, 1H), 7.53 (ddd, *J* = 10.8, 10.3, 3.5 Hz, 2H), 7.41 (dd, *J* = 15.7, 7.9 Hz, 4H), 7.36 – 7.31 (m, 2H), 7.30 – 7.24 (m, 6H), 6.83 (s, 1H), 5.40 (d, *J* = 12.6 Hz, 1H), 5.14 (d, *J* = 12.6 Hz, 1H), 4.47 (dd, *J* = 10.3, 2.9 Hz, 1H), 3.58 (dd, *J* = 16.8, 10.3 Hz, 1H), 3.13 (dd, *J* = 16.7, 2.9 Hz, 1H), 1.59 (s, 9H). ^{13}C NMR (101 MHz, $CDCl_3$) δ 197.6, 169.3, 155.1, 138.2, 137.0, 133.1, 131.4, 128.5, 128.4, 128.3, 128.3, 128.3, 128.2, 128.1, 127.9, 126.8, 124.2, 123.4, 122.7, 121.1, 111.3, 105.2, 89.7, 85.8, 84.2, 83.0, 61.9, 39.0, 36.7, 28.1. HRMS (ESI) $[M+Na]^+$ calcd for $C_{38}H_{34}O_5Na^+$, 593.2298, found 593.2293. (Chiral IE-3, λ= 254 nm, *n*-hexane/2-propanol= 19/1, Flow rate = 1.0 mL/min), t_R = 10.681 min(major), 22.058 min.

HPLC chromatogram of racemic 45

Condition: n-hexane/2-propanol =19:1

Flow rate =1.0 mL/min

λ= 254 nm

Chiral IE-3

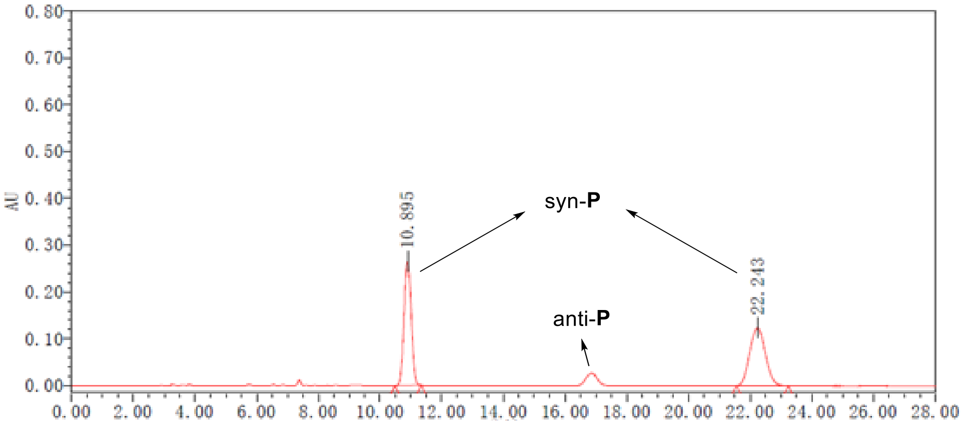

Entry	Retention Time/min	Area	Height	Area(%)
1	10.895	4418288	264820	50.13
2	22.243	4395186	123830	49.87

Supplementary Figure 99. Chiral HPLC analysis of racemic **45**

HPLC chromatogram of chiral 45

Condition: n-hexane/2-propanol =19:1

Flow rate =1.0 mL/min

λ= 254 nm

Chiral IE-3

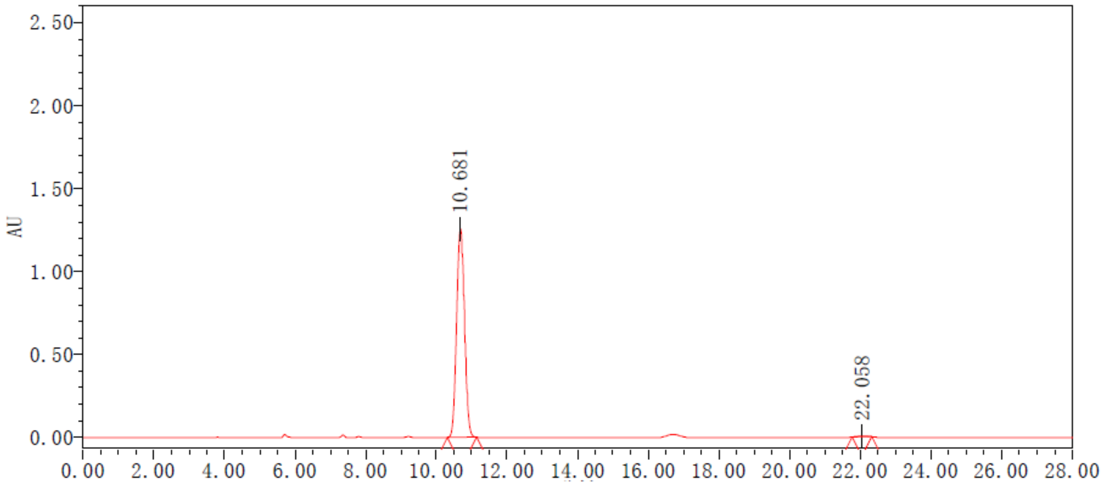

The larger version of HPLC chromatogram of chiral 45

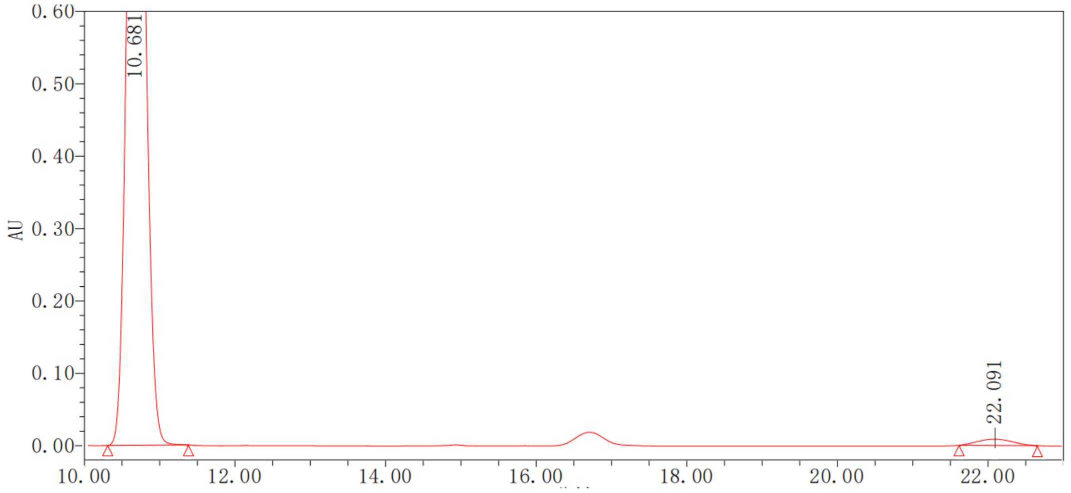

Entry	Retention Time/min	Area	Height	Area(%)
1	10.681	19757193	1256331	99.51
2	22.091	96684	4552	0.49

Supplementary Figure 100. Chiral HPLC analysis of chiral **45**

Tert-butyl (2S,3R)-2-(benzo[b]thiophen-2-ylmethoxy)-3-(2-oxo-2-phenylethyl)-2,5-diphenylpent-4-ynoate (46)

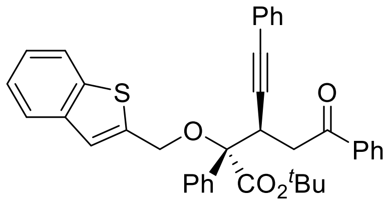

46

Colorless oil; 60.2 mg, 74% yield, >20:1 d.r., 93% *ee*; ^{1}H NMR (400 MHz, $CDCl_3$) δ 7.88 – 7.81 (m, 3H), 7.76 – 7.69 (m, 3H), 7.53 – 7.47 (m, 1H), 7.41 – 7.36 (m, 4H), 7.34 (dd, *J* = 3.8, 1.6 Hz, 1H), 7.33 – 7.30 (m, 2H), 7.28 – 7.23 (m, 6H), 5.47 (d, *J* = 12.2 Hz, 1H), 5.25 (d, *J* = 12.1 Hz, 1H), 4.44 (dd, *J* = 10.4, 2.9 Hz, 1H), 3.45 (dd, *J* = 16.7, 10.4 Hz, 1H), 3.08 (dd, *J* = 16.7, 2.9 Hz, 1H), 1.54 (s, 9H). ^{13}C NMR (101 MHz, $CDCl_3$) δ 197.5, 169.3, 143.0, 140.2, 139.5, 138.2, 136.9, 133.1, 131.5, 128.5, 128.4, 128.3, 128.3, 128.2, 128.1, 127.9, 126.7, 124.2, 124.1, 123.5, 123.4, 122.4, 121.7, 89.7, 86.0, 84.2, 83.1, 64.9, 39.1, 36.6, 28.1. HRMS (ESI) $[M+Na]^+$ calcd for $C_{38}H_{34}O_4SNa^+$, 609.2070, found 609.2065. (Chiral IA, λ= 254 nm, *n*-hexane/2-propanol= 49/1, Flow rate = 1.0 mL/min), t_R = 13.028 min(major), 25.316 min.

HPLC chromatogram of racemic 46

Condition: n-hexane/2-propanol =49:1

Flow rate =1.0 mL/min

λ= 254 nm

Chiral IA

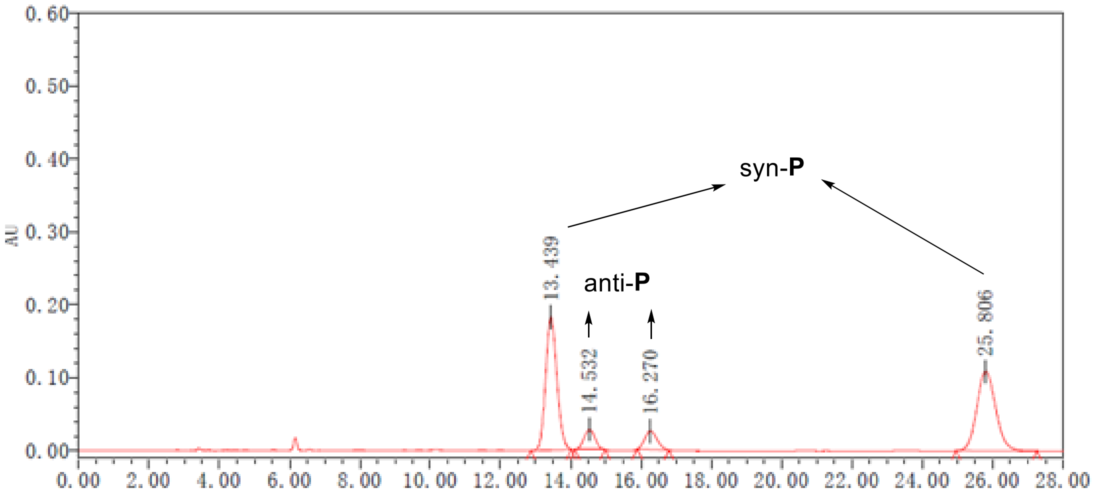

Entry	Retention Time/min	Area	Height	Area(%)
1	13.439	4072895	183009	43.59
2	14.532	608324	27386	6.51
3	16.270	623506	25297	6.67
4	25.806	4039690	108681	43.23

Supplementary Figure 101. Chiral HPLC analysis of racemic **46**

HPLC chromatogram of chiral 46

Condition: n-hexane/2-propanol = 49:1

Flow rate =1.0 mL/min

λ= 254 nm

Chiral IA

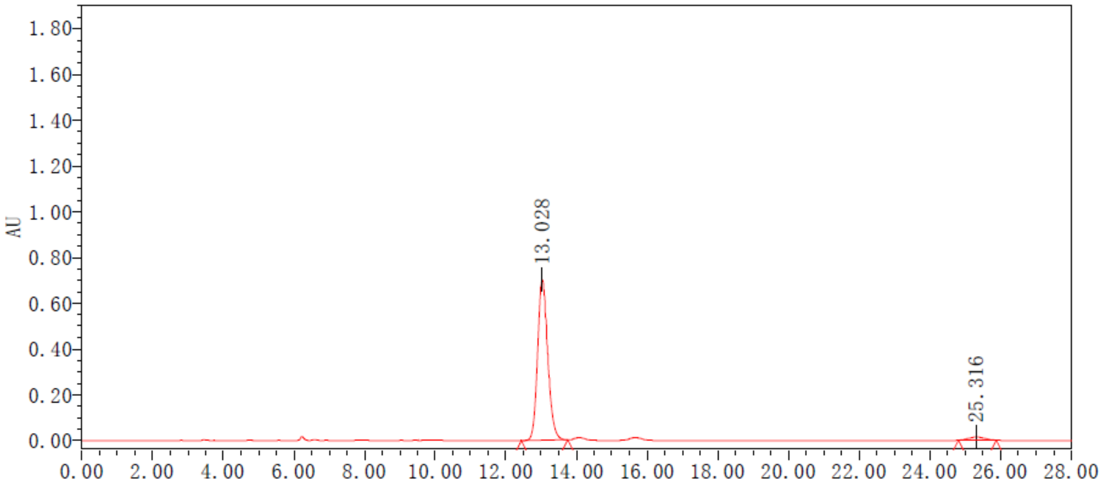

The larger version of HPLC chromatogram of chiral 46

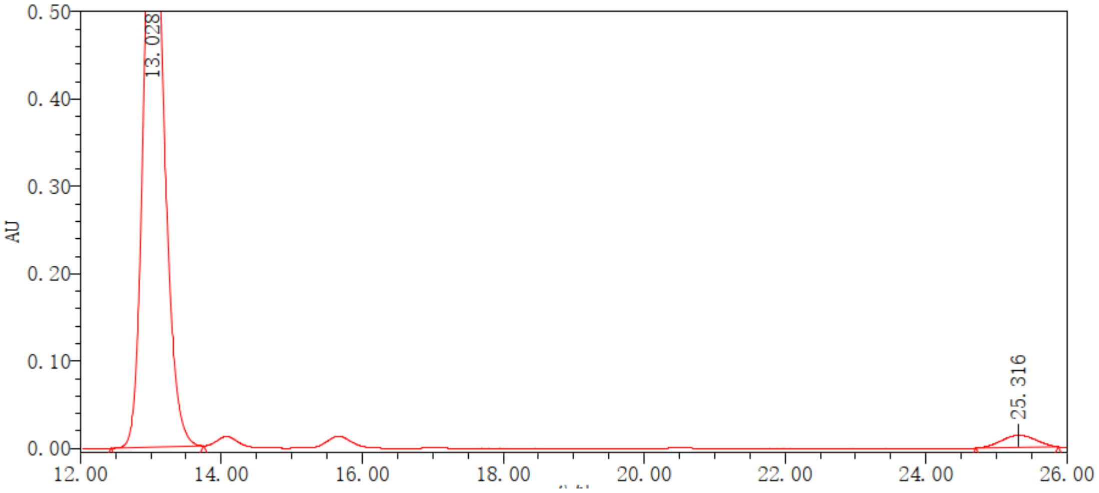

Entry	Retention Time/min	Area	Height	Area(%)
1	13.028	14190254	701421	96.51
2	25.316	444194	13487	3.49

Supplementary Figure 102. Chiral HPLC analysis of chiral **46**

Tert-butyl (2S,3R)-2-(cinnamyloxy)-3-(2-oxo-2-phenylethyl)-2,5-diphenylpent-4-ynoate (47)

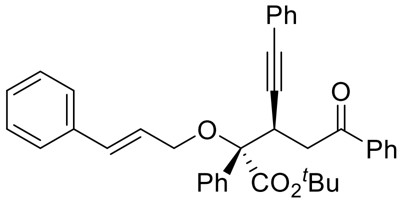

47

Colorless oil; 45.9 mg, 55% yield, >20:1 d.r., 93% *ee*, $[\alpha]_D^{20}$ = -12.79 (c = 0.1, CH_2Cl_2); ^1H NMR (400 MHz, $CDCl_3$) δ 7.90 (d, *J* = 7.4 Hz, 2H), 7.71 (d, *J* = 7.3 Hz, 2H), 7.51 (t, *J* = 7.4 Hz, 1H), 7.38 (dd, *J* = 15.2, 8.2 Hz, 6H), 7.31 (t, *J* = 7.3 Hz, 3H), 7.26 – 7.20 (m, 6H), 6.71 (d, *J* = 15.9 Hz, 1H), 6.42 (dt, *J* = 15.9, 5.6 Hz, 1H), 4.70 (dd, *J* = 12.7, 5.8 Hz, 1H), 4.55 (dd, *J* = 12.6, 5.0 Hz, 1H), 4.37 (dd, *J* = 10.1, 2.9 Hz, 1H), 3.38 (dd, *J* = 16.6, 10.2 Hz, 1H), 3.15 (dd, *J* = 16.6, 2.8 Hz, 1H), 1.53 (s, 9H). ^{13}C NMR (126 MHz, $CDCl_3$) δ 197.6, 169.6, 138.6, 137.0, 137.0, 133.1, 131.5, 131.4, 128.5, 128.3, 128.2, 128.2, 128.2, 127.8, 127.6, 126.8, 126.7, 126.5, 123.5, 89.7, 85.8, 84.0, 82.8, 67.6, 39.3, 36.7, 28.1. HRMS (ESI) $[M+Na]^+$ calcd for $C_{38}H_{36}O_4Na^+$, 579.2506, found 579.2504. (Chiral IE-3, λ= 254 nm, *n*-hexane/2-propanol= 19/1, Flow rate = 1.0 mL/min), t_R = 11.529 min(major), 22.634 min.

HPLC chromatogram of racemic 47

Condition: n-hexane/2-propanol =19:1

Flow rate =1.0 mL/min

λ= 254 nm

Chiral IE-3

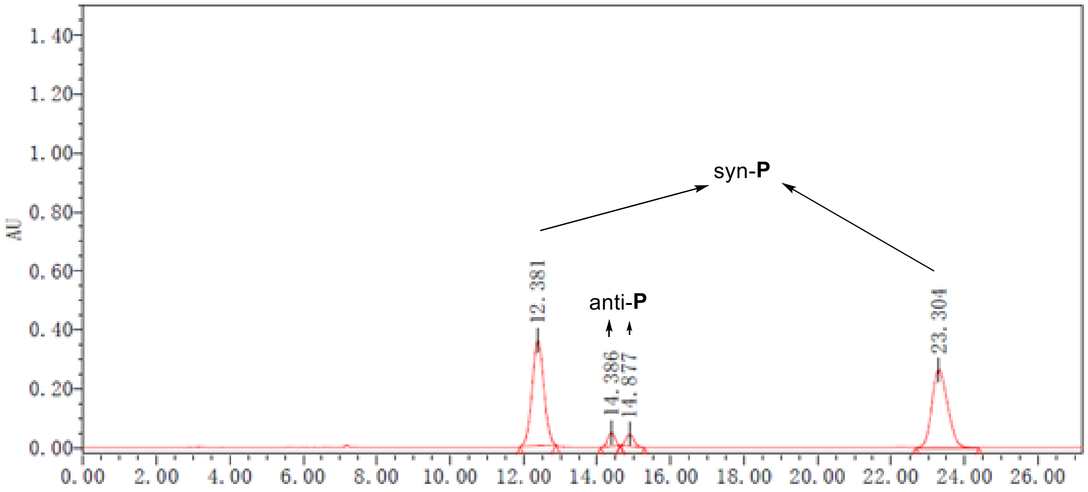

Entry	Retention Time/min	Area	Height	Area(%)
1	12.381	7891813	358649	46.27
2	14.386	650976	48062	3.82
3	14.877	633398	41112	3.71
4	23.304	7880382	265218	46.20

Supplementary Figure 103. Chiral HPLC analysis of racemic **47**

HPLC chromatogram of chiral 47

Condition: n-hexane/2-propanol =19:1

Flow rate =1.0 mL/min

λ= 254 nm

Chiral IE-3

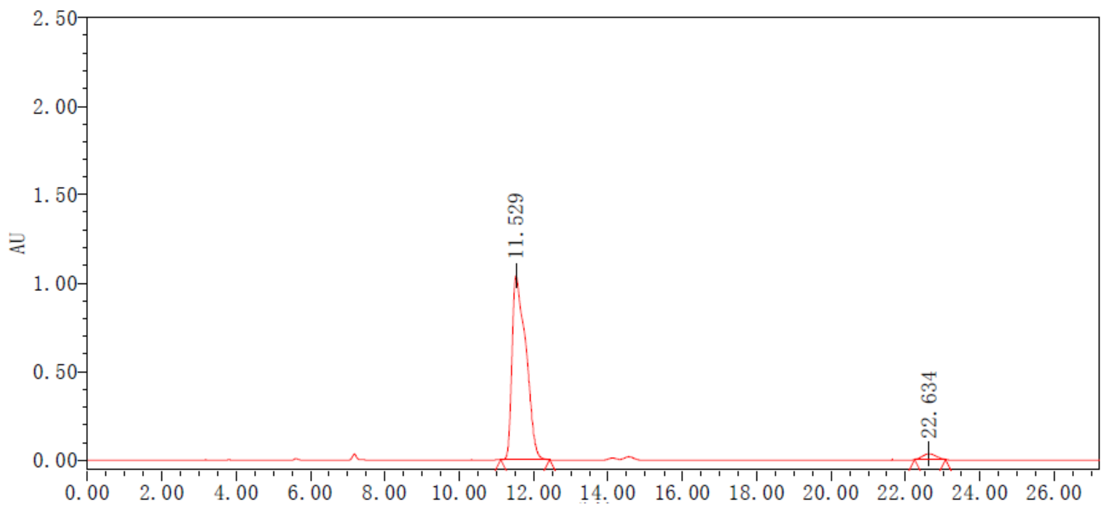

The larger version of HPLC chromatogram of chiral 47

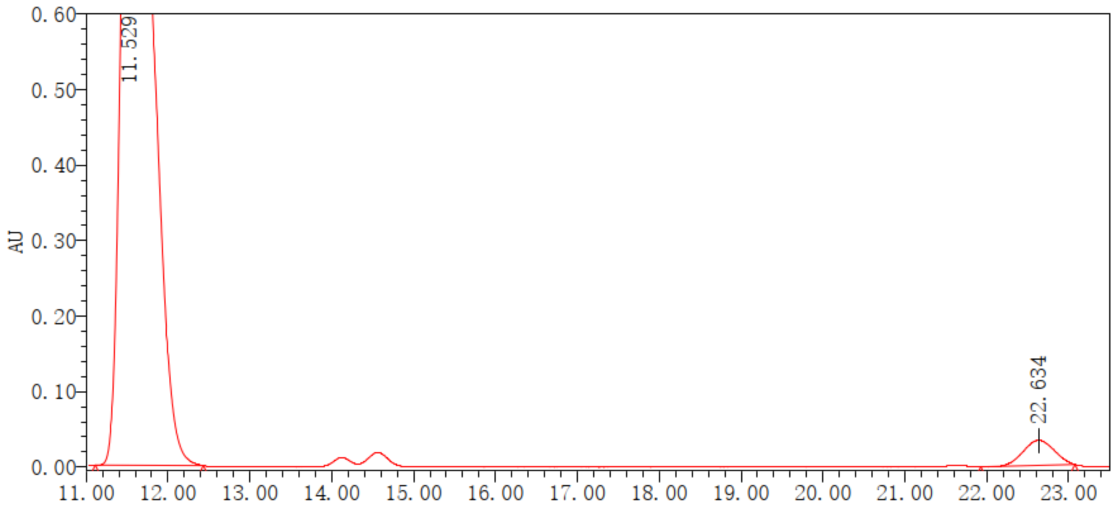

Entry	Retention Time/min	Area	Height	Area(%)
1	11.529	26532083	1040734	96.51
2	22.634	875925	33169	3.49

Supplementary Figure 104. Chiral HPLC analysis of chiral **47**

Tert-butyl (2S,3R)-2-(cinnamyloxy)-3-(2-oxo-2-phenylethyl-1-d)-2,5-diphenylpent-4-ynoate(47-[D])

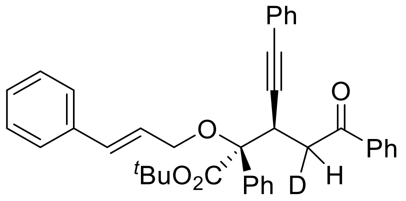

47-[D]

^{1}H NMR (400 MHz, $CDCl_3$) δ 7.90 (d, *J* = 7.8 Hz, 2H), 7.71 (d, *J* = 7.8 Hz, 2H), 7.50 (d, *J* = 7.1 Hz, 1H), 7.38 (dd, *J* = 14.3, 7.6 Hz, 6H), 7.31 (t, *J* = 7.4 Hz, 3H), 7.23 (d, *J* = 7.7 Hz, 6H), 6.71 (d, *J* = 15.9 Hz, 1H), 6.42 (dt, *J* = 15.9, 5.6 Hz, 1H), 4.68 (d, *J* = 6.0 Hz, 1H), 4.55 (dd, *J* = 12.4, 4.9 Hz, 1H), 4.36 (d, *J* = 2.4 Hz, 1H), 3.38 (dd, *J* = 16.6, 10.3 Hz, 0.14H), 3.14 (dd, *J* = 15.0, 4.7 Hz, 1H), 1.53 (s, 9H). ^{13}C NMR (101 MHz, $CDCl_3$) δ 197.7, 169.6, 138.6, 137.1, 137.0, 133.1, 131.5, 131.4, 128.5, 128.3, 128.2, 128.2, 128.1, 127.8, 127.6, 126.8, 126.7, 126.5, 123.5, 89.7, 85.8, 84.0, 82.8, 67.6, 39.3, 39.2, 39.0, 36.7, 28.1. HRMS (ESI) $[M+Na]^+$ calcd for $C_{38}H_{35}DO_4Na^+$, 580.2569, found 580.2566.

Tert-butyl (2S,3R)-2-(((E)-3-(4-methoxyphenyl)allyl)oxy)-3-(2-oxo-2-phenylethyl)-2,5-diphenylpent-4-ynoate (48)

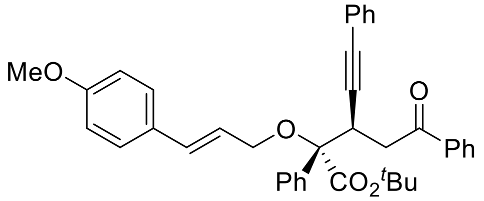

48

Colorless oil; 51.0 mg, 58% yield, >20:1 d.r., 97% *ee*; ^{1}H NMR (400 MHz, $CDCl_3$) δ 8.03 (d, *J* = 7.7 Hz, 1H), 7.90 (d, *J* = 7.7 Hz, 2H), 7.71 (d, *J* = 7.7 Hz, 2H), 7.50 (dd, *J* = 15.4, 7.7 Hz, 2H), 7.41 – 7.36 (m, 3H), 7.35 – 7.31 (m, 3H), 7.25 – 7.21 (m, 4H), 6.85 (d, *J* = 8.4 Hz, 2H), 6.64 (d, *J* = 15.8 Hz, 1H), 6.29 (dt, *J* = 15.8, 5.8 Hz, 1H), 4.67 (dd, *J* = 12.2, 6.3 Hz, 1H), 4.53 (dd, *J* = 12.1, 5.3 Hz, 1H), 4.36 (dd, *J* = 10.0, 2.7 Hz, 1H), 3.80 (s, 3H), 3.38 (dd, *J* = 16.5, 10.2 Hz, 1H), 3.15 (dd, *J* = 16.6, 2.6 Hz, 1H), 1.53 (s,

9H). ^{13}C NMR (126 MHz, $CDCl_3$) δ 197.7, 169.6, 159.2, 138.6, 137.0, 133.3, 133.1, 131.7, 131.4, 131.3, 129.8, 128.7, 128.5, 128.3, 128.3, 128.2, 128.1, 128.1, 127.8, 127.7, 126.8, 124.4, 123.5, 114.0, 89.8, 85.7, 84.0, 82.7, 67.9, 55.3, 39.3, 36.7, 28.1. HRMS (ESI) $[M+Na]^+$ calcd for $C_{39}H_{38}O_5Na^+$, 609.2611, found 609.2610. (Chiral IA, λ= 254 nm, *n*-hexane/2-propanol= 19/1, Flow rate = 1.0 mL/min), t_R = 9.753 min(major), 17.375 min.

HPLC chromatogram of racemic 48

Condition: n-hexane/2-propanol =19:1

Flow rate =1.0 mL/min

λ= 254 nm

Chiral IA

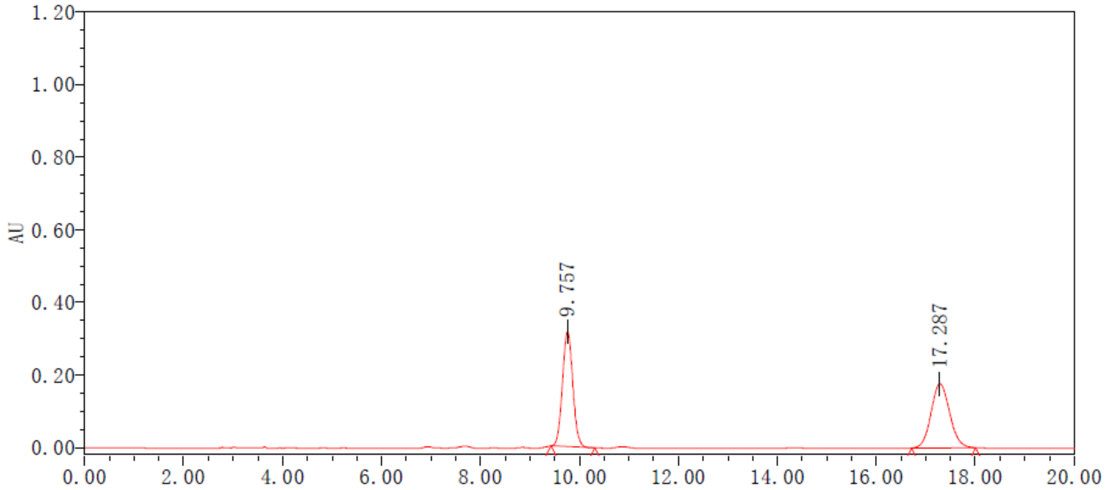

Entry	Retention Time/min	Area	Height	Area(%)
1	9.757	4594856	317072	49.98
2	17.287	4599083	176656	50.02

Supplementary Figure 105. Chiral HPLC analysis of racemic **48**

HPLC chromatogram of chiral 48

Condition: n-hexane/2-propanol =19:1

Flow rate =1.0 mL/min

λ= 254 nm

Chiral IA

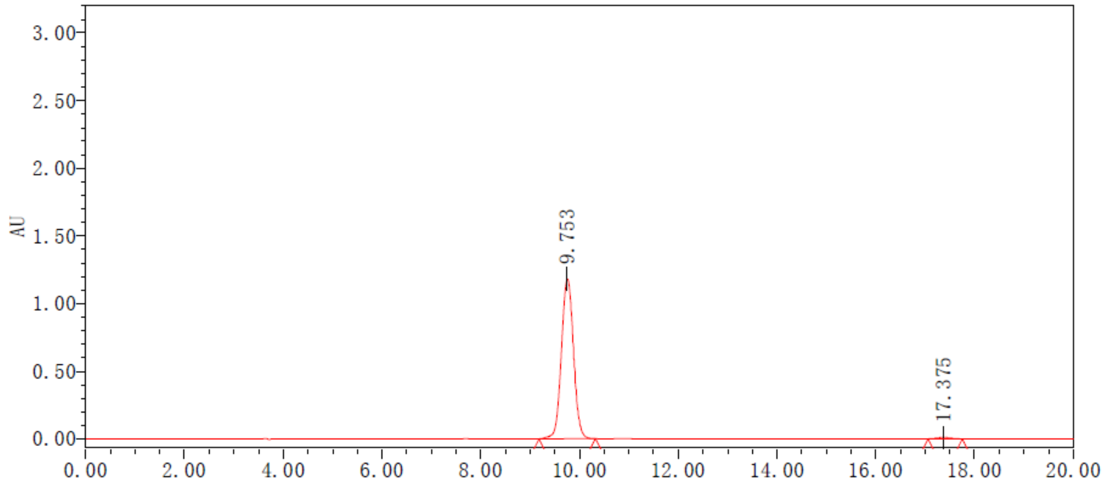

The larger version of HPLC chromatogram of chiral 48

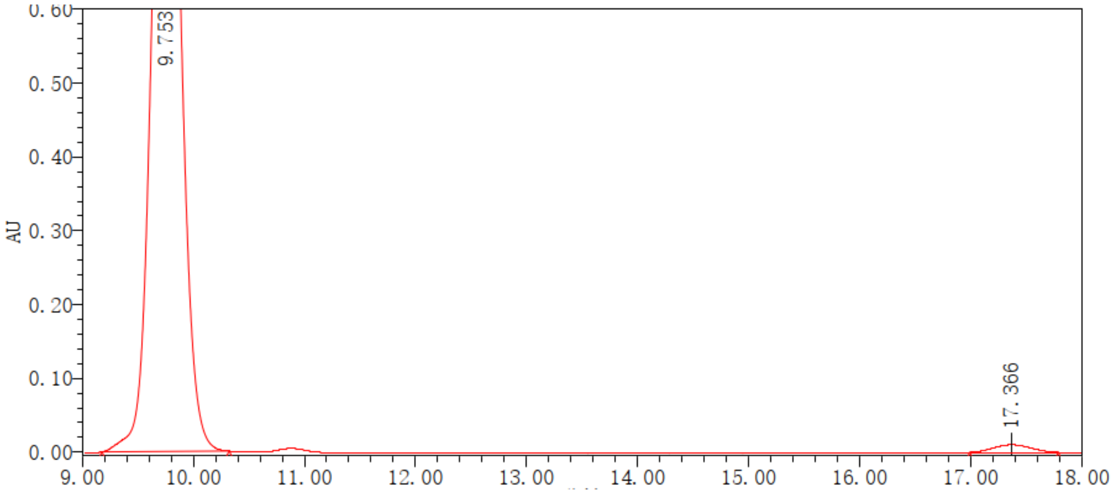

Entry	Retention Time/min	Area	Height	Area(%)
1	9.753	20121122	1180943	98.50
2	17.366	247194	10441	2.50

Supplementary Figure 106. Chiral HPLC analysis of chiral **48**

Tert-butyl (2S,3R)-2-(((E)-3-(4-bromophenyl)allyl)oxy)-3-(2-oxo-2-phenylethyl)-2,5-diphenylpent-4-ynoate (49)

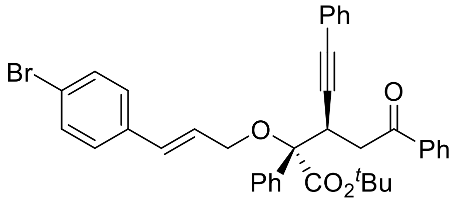

49

Colorless oil; 58.0 mg, 61% yield, >20:1 d.r., 97% *ee*; ^{1}H NMR (400 MHz, $CDCl_3$) δ 7.90 (d, *J* = 7.9 Hz, 2H), 7.70 (d, *J* = 8.0 Hz, 2H), 7.52 (t, *J* = 7.3 Hz, 1H), 7.44 – 7.35 (m, 6H), 7.32 (d, *J* = 7.0 Hz, 1H), 7.25 – 7.20 (m, 7H), 6.65 (d, *J* = 15.9 Hz, 1H), 6.40 (dt, *J* = 15.9, 5.4 Hz, 1H), 4.66 (dd, *J* = 12.9, 5.8 Hz, 1H), 4.53 (dd, *J* = 12.9, 5.0 Hz, 1H), 4.37 (dd, *J* = 10.0, 2.8 Hz, 1H), 3.35 (dd, *J* = 16.6, 10.1 Hz, 1H), 3.16 (dd, *J* = 16.5, 2.8 Hz, 1H), 1.53 (s, 9H). ^{13}C NMR (126 MHz, $CDCl_3$) δ 197.5, 169.5, 138.4, 137.0, 136.0, 133.1, 131.6, 131.4, 130.2, 128.6, 128.2, 128.2, 128.2, 128.1, 127.9, 127.6, 126.8, 123.5, 121.3, 89.7, 85.8, 84.0, 82.8, 67.4, 39.3, 36.7, 28.1. HRMS (ESI) $[M+Na]^+$ calcd for $C_{38}H_{35}O_4BrNa^+$, 657.1611, found 657.1606. (Chiral IE-3, λ= 254 nm, *n*-hexane/2-propanol= 19/1, Flow rate = 1.0 mL/min), t_R = 11.987 min(major), 23.436 min.

HPLC chromatogram of racemic 49

Condition: n-hexane/2-propanol =19:1

Flow rate =1.0 mL/min

λ= 254 nm

Chiral IE-3

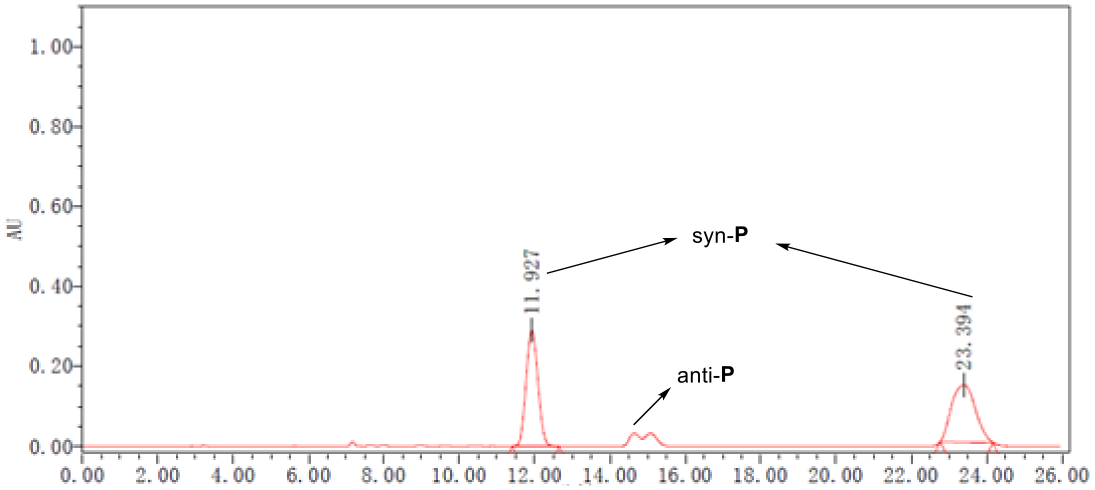

Entry	Retention Time/min	Area	Height	Area(%)
1	11.927	6199542	290636	49.91
2	23.394	6222741	142333	50.09

Supplementary Figure 107. Chiral HPLC analysis of racemic **49**

HPLC chromatogram of chiral 49

Condition: n-hexane/2-propanol =19:1

Flow rate =1.0 mL/min

λ= 254 nm

Chiral IE-3

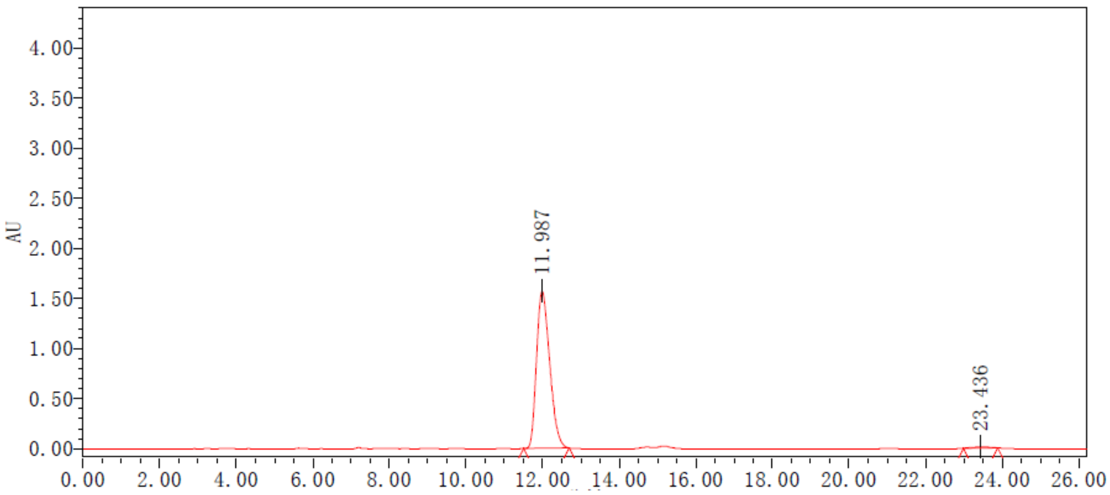

The larger version of HPLC chromatogram of chiral 49

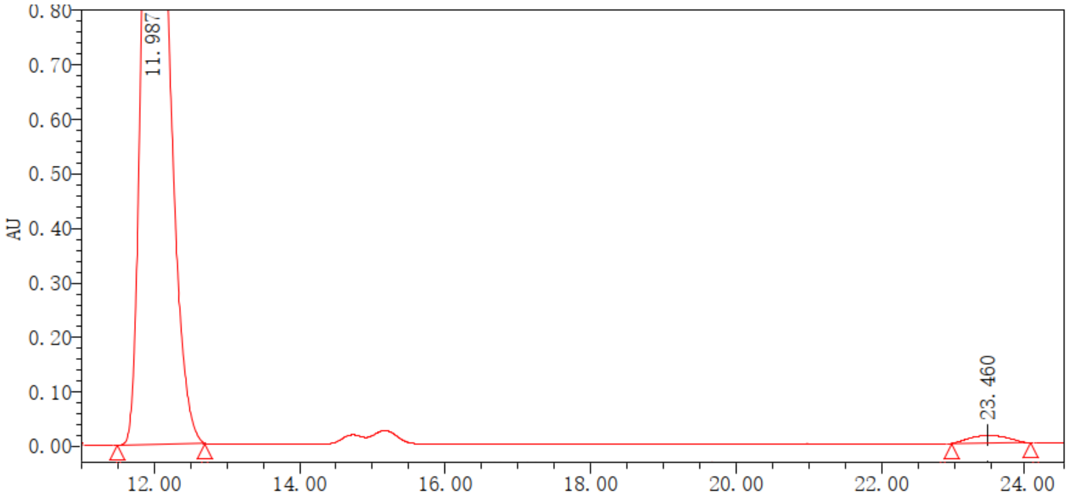

Entry	Retention Time/min	Area	Height	Area(%)
1	11.987	37048978	1569398	98.51
2	23.436	505333	13705	1.49

Supplementary Figure 108. Chiral HPLC analysis of chiral **49**

Tert-butyl (2S,3R)-2-(((E)-3-(4-nitrophenyl)allyl)oxy)-3-(2-oxo-2-phenylethyl)-2,5-diphenylpent-4-ynoate (50)

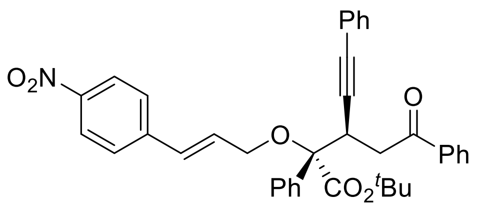

50

Colorless oil; 53.2 mg, 59% yield, >20:1 d.r., 99% *ee*; ^{1}H NMR (400 MHz, $CDCl_3$) δ 8.17 (d, *J* = 8.8 Hz, 2H), 7.94 – 7.86 (m, 2H), 7.73 – 7.66 (m, 2H), 7.54 (t, *J* = 7.4 Hz, 1H), 7.48 (d, *J* = 8.8 Hz, 2H), 7.41 (dd, *J* = 16.2, 7.9 Hz, 4H), 7.36 – 7.32 (m, 1H), 7.25 (d, *J* = 9.4 Hz, 5H), 6.81 (d, *J* = 16.0 Hz, 1H), 6.57 (dt, *J* = 16.0, 5.1 Hz, 1H), 4.71 (ddd, *J* = 13.9, 5.4, 1.3 Hz, 1H), 4.58 (dd, *J* = 13.9, 3.3 Hz, 1H), 4.40 (dd, *J* = 9.9, 3.2 Hz, 1H), 3.35 (dd, *J* = 16.7, 9.9 Hz, 1H), 3.18 (dd, *J* = 16.7, 3.2 Hz, 1H), 1.54 (s, 9H). ^{13}C NMR (126 MHz, $CDCl_3$) δ 197.4, 169.4, 146.9, 143.6, 138.1, 136.9, 133.2, 131.9, 131.4, 128.8, 128.6, 128.4, 128.3, 128.2, 128.2, 127.9, 127.0, 126.8, 124.0, 89.5, 85.9, 84.1, 83.0, 66.9, 39.3, 36.7, 28.1. HRMS (ESI) $[M+Na]^+$ calcd for $C_{38}H_{35}O_6Na^+$, 624.2357, found 624.2352. (Chiral IA, λ= 254 nm, *n*-hexane/2-propanol= 19/1, Flow rate = 1.0 mL/min), t_R = 20.694 min(major), 37.037 min.

HPLC chromatogram of racemic 50

Condition: n-hexane/2-propanol =19:1

Flow rate =1.0 mL/min

λ= 254 nm

Chiral IA

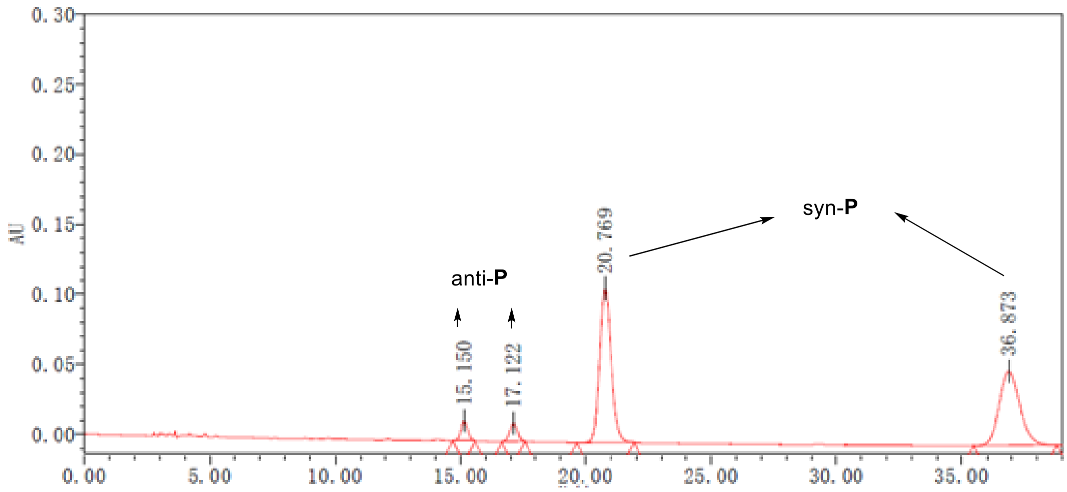

Entry	Retention Time/min	Area	Height	Area(%)
1	15.150	266982	14198	3.79
2	17.122	265085	12700	3.77
3	20.769	3491967	109886	49.63
4	36.873	3012578	52919	42.81

Supplementary Figure 109. Chiral HPLC analysis of racemic **50**

HPLC chromatogram of chiral 50

Condition: n-hexane/2-propanol =19:1

Flow rate =1.0 mL/min

λ= 254 nm

Chiral IA

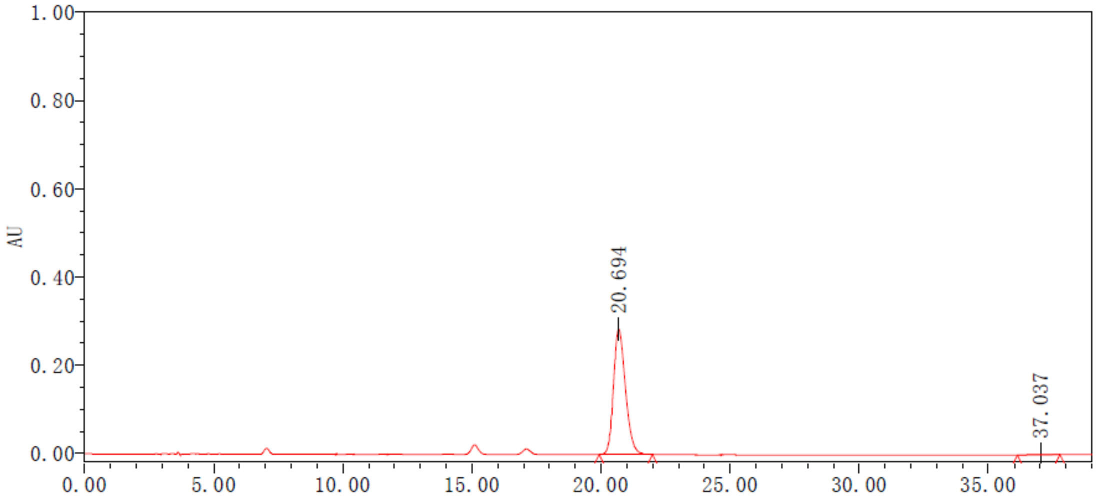

The larger version of HPLC chromatogram of chiral 50

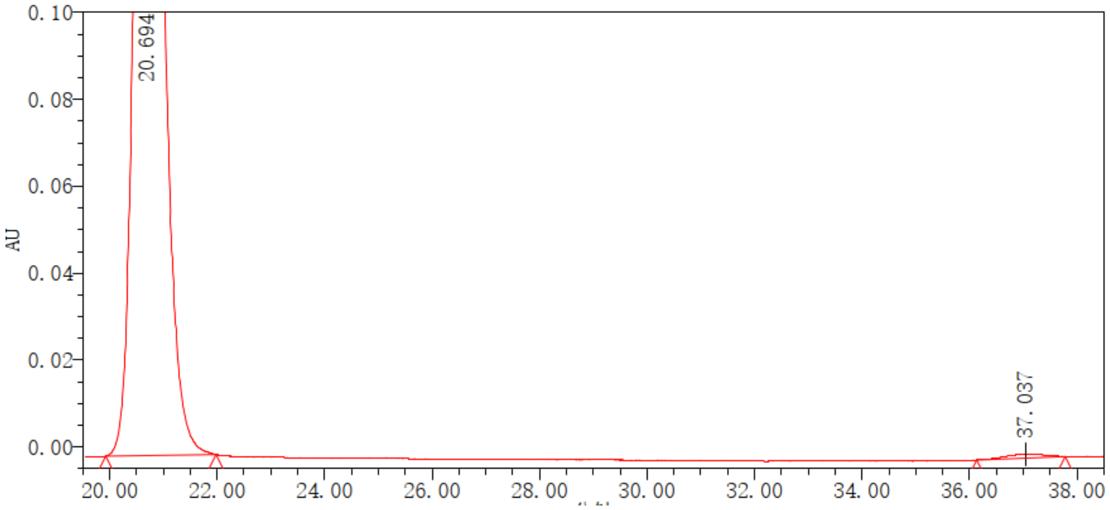

Entry	Retention Time/min	Area	Height	Area(%)
1	20.694	9076478	284505	99.50
2	37.037	45354	927	0.50

Supplementary Figure 110. Chiral HPLC analysis of chiral **50**

Tert-butyl (2S,3R)-2-(((E)-3-(2-bromophenyl)allyl)oxy)-3-(2-oxo-2-phenylethyl)-2,5-diphenylpent-4-ynoate (51)

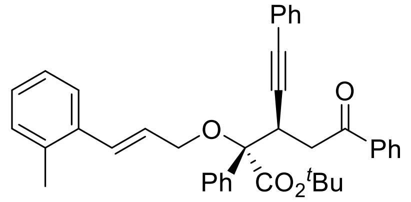

51

Colorless oil; 51.3 mg, 60% yield, >20:1 d.r., 93% *ee*; ^{1}H NMR (400 MHz, $CDCl_3$) δ 7.90 (d, *J* = 7.6 Hz, 2H), 7.72 (d, *J* = 7.7 Hz, 2H), 7.50 (dd, *J* = 14.9, 7.8 Hz, 2H), 7.38 (dd, *J* = 14.4, 7.5 Hz, 4H), 7.32 (d, *J* = 7.2 Hz, 1H), 7.26 – 7.19 (m, 5H), 7.15 (dd, *J* = 8.2, 3.0 Hz, 3H), 6.92 (d, *J* = 15.8 Hz, 1H), 6.32 (dt, *J* = 15.8, 5.7 Hz, 1H), 4.72 (dd, *J* = 12.5, 6.0 Hz, 1H), 4.55 (dd, *J* = 12.5, 5.1 Hz, 1H), 4.36 (dd, *J* = 10.1, 2.9 Hz, 1H), 3.39 (dd, *J* = 16.6, 10.2 Hz, 1H), 3.16 (dd, *J* = 16.5, 2.8 Hz, 1H), 2.31 (s, 3H), 1.54 (s, 9H). ^{13}C NMR (101 MHz, $CDCl_3$) δ 197.6, 169.6, 138.6, 137.0, 136.0, 135.5, 133.1, 131.4, 130.3, 129.3, 128.5, 128.2, 128.2, 128.2, 128.1, 128.0, 127.8, 127.5, 126.9, 126.1, 125.8, 123.5, 89.8, 85.8, 84.0, 82.7, 67.9, 39.4, 36.8, 28.1, 19.8. HRMS (ESI) $[M+Na]^+$ calcd for $C_{39}H_{38}O_4Na^+$, 593.2756, found 593.2756. (Chiral IE-3, λ= 254 nm, *n*-hexane/2-propanol= 19/1, Flow rate = 1.0 mL/min), t_R = 10.751 min(major), 22.224 min.

HPLC chromatogram of racemic 51

Condition: n-hexane/2-propanol =19:1

Flow rate =1.0 mL/min

λ= 254 nm

Chiral IE-3

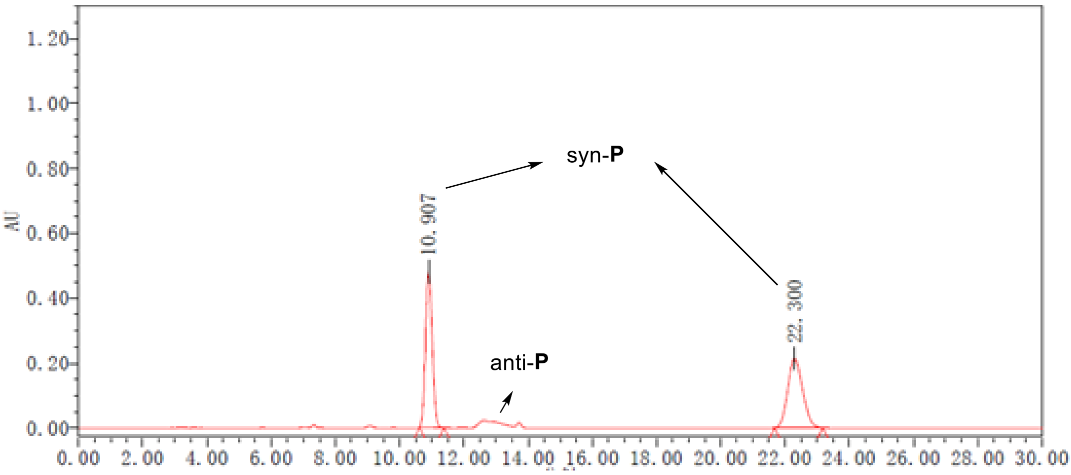

Entry	Retention Time/min	Area	Height	Area(%)
1	10.907	7022469	479488	50.28
2	22.300	6944345	213967	49.72

Supplementary Figure 111. Chiral HPLC analysis of racemic **51**

HPLC chromatogram of chiral 51

Condition: n-hexane/2-propanol =19:1

Flow rate =1.0 mL/min

λ= 254 nm

Chiral IE-3

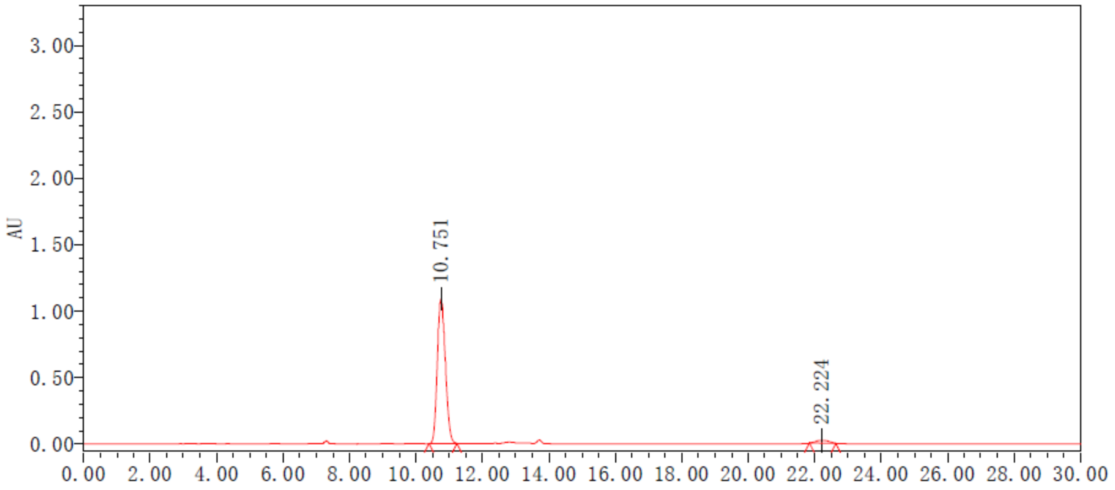

The larger version of HPLC chromatogram of chiral 51

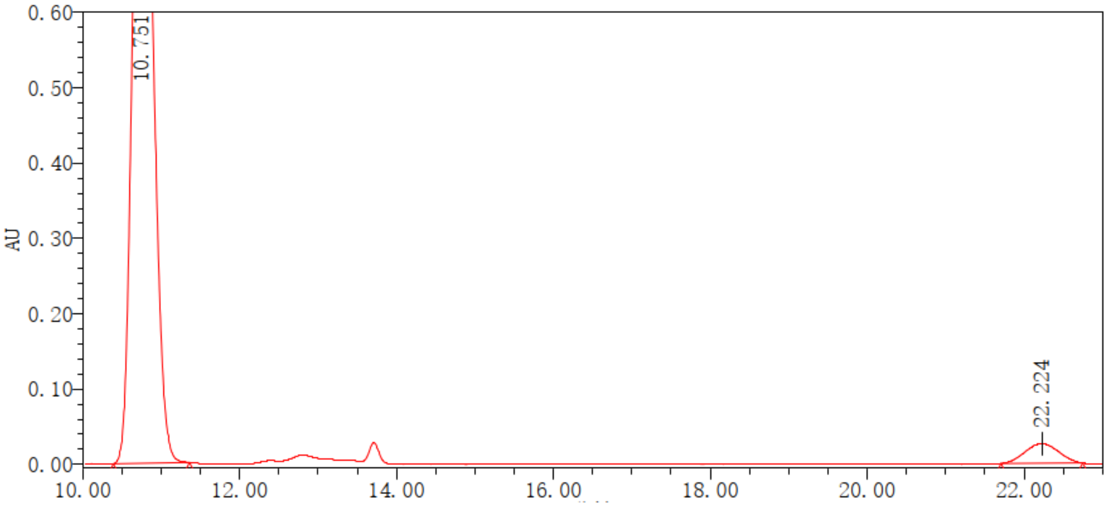

Entry	Retention Time/min	Area	Height	Area(%)
1	10.751	18803712	1090492	96.52
2	22.224	597231	22671	3.48

Supplementary Figure 112. Chiral HPLC analysis of chiral **51**

Tert-butyl (2S,3R)-2-(((E)-2-methyl-3-phenylallyl)oxy)-3-(2-oxo-2-phenylethyl)-2,5-diphenylpent-4-ynoate (52)

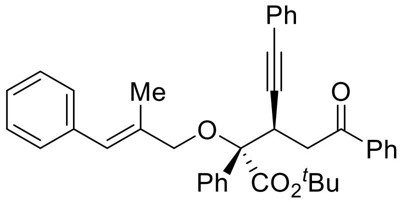

52

Colorless oil; 53.9 mg, 63% yield, >20:1 d.r., 94% *ee*; ^{1}H NMR (400 MHz, $CDCl_3$) δ 7.92 – 7.87 (m, 2H), 7.76 – 7.71 (m, 2H), 7.51 (dd, *J* = 10.5, 4.2 Hz, 1H), 7.42 – 7.35 (m, 4H), 7.33 – 7.28 (m, 5H), 7.26 – 7.19 (m, 6H), 6.67 (s, 1H), 4.63 (d, *J* = 12.0 Hz, 1H), 4.46 – 4.35 (m, 2H), 3.50 – 3.36 (m, 1H), 3.16 (dd, *J* = 16.6, 3.0 Hz, 1H), 1.99 (d, *J* = 0.9 Hz, 3H), 1.53 (s, 9H). ^{13}C NMR (126 MHz, $CDCl_3$) δ 197.6, 169.5, 138.7, 137.8, 137.0, 135.6, 133.1, 131.4, 129.0, 128.6, 128.2, 128.2, 128.2, 128.1, 127.8, 126.8, 126.4, 126.0, 123.5, 89.8, 85.5, 84.0, 82.7, 72.1, 39.4, 36.6, 28.1, 15.8. HRMS (ESI) $[M+Na]^+$ calcd for $C_{39}H_{38}O_4Na^+$, 593.2662, found 593.2662. (Chiral IC, λ= 254 nm, *n*-hexane/2-propanol= 49/1, Flow rate = 1.0 mL/min), t_R = 7.560 min(major), 11.924 min.

HPLC chromatogram of racemic 52

Condition: n-hexane/2-propanol =49:1

Flow rate =1.0 mL/min

λ= 254 nm

Chiral IC

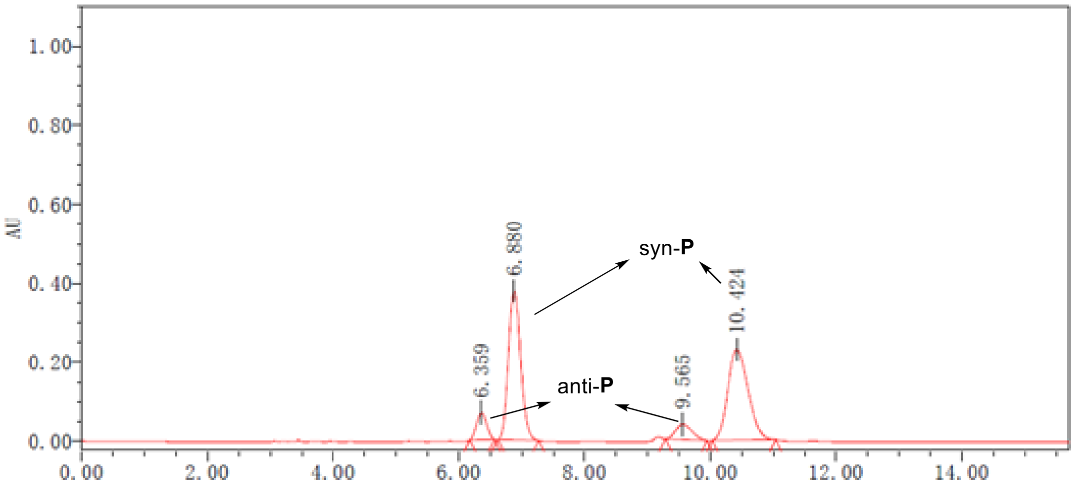

Entry	Retention Time/min	Area	Height	Area(%)
1	6.359	781102	69069	6.61
2	6.880	5154728	378200	43.63
3	9.565	722113	39855	6.11
4	10.424	5155801	230506	43.64

Supplementary Figure 113. Chiral HPLC analysis of racemic **52**

HPLC chromatogram of chiral 52

Condition: n-hexane/2-propanol =49:1

Flow rate =1.0 mL/min

λ= 254 nm

Chiral IC

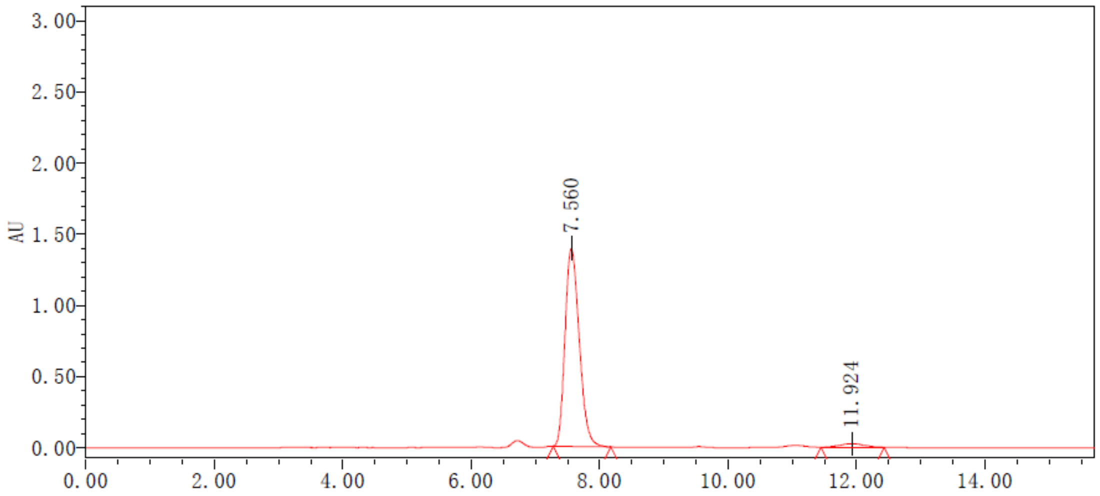

The larger version of HPLC chromatogram of chiral 52

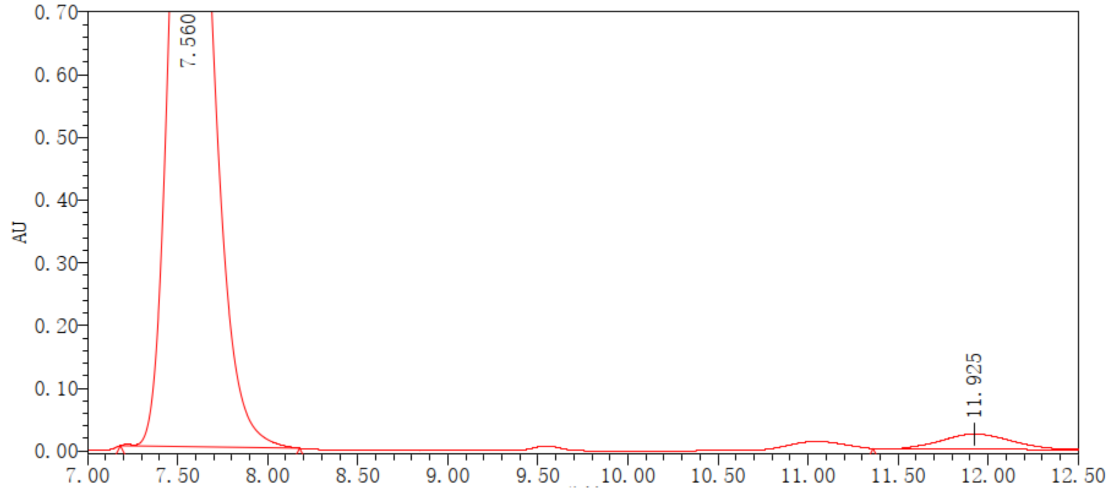

Entry	Retention Time/min	Area	Height	Area(%)
1	7.560	21270615	1391012	97.02
2	11.924	653961	24333	2.98

Supplementary Figure 114. Chiral HPLC analysis of chiral **52**

Tert-butyl (2S,3R)-2-(((E)-3-(furan-2-yl)allyl)oxy)-3-(2-oxo-2-phenylethyl)-2,5-diphenylpent-4-ynoate (53)

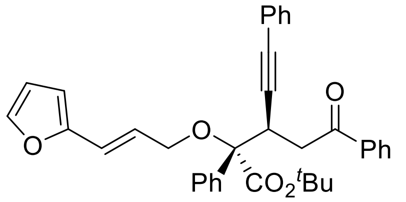

53

Colorless oil; 48.7 mg, 55% yield, >20:1 d.r., 93% *ee*; ^{1}H NMR (400 MHz, $CDCl_3$) δ 7.90 (d, *J* = 7.8 Hz, 2H), 7.70 (d, *J* = 7.8 Hz, 2H), 7.52 (t, *J* = 7.4 Hz, 1H), 7.44 – 7.34 (m, 1H), 7.31 (d, *J* = 7.0 Hz, 5H), 7.24 (d, *J* = 10.5 Hz, 1H), 6.57 (d, *J* = 15.9 Hz, 5H), 6.35 (dt, *J* = 18.9, 5.5 Hz, 1H), 6.21 (d, *J* = 3.1 Hz, 2H), 4.68 (dd, *J* = 13.3, 5.7 Hz, 1H), 4.52 (dd, *J* = 13.3, 4.9 Hz, 1H), 4.34 (dd, *J* = 10.1, 2.9 Hz, 1H), 3.37 (dd, *J* = 16.6, 10.2 Hz, 1H), 3.14 (dd, *J* = 16.5, 2.8 Hz, 1H), 1.52 (s, 9H). ^{13}C NMR (126 MHz, $CDCl_3$) δ 197.6, 169.5, 152.7, 141.9, 138.5, 137.0, 133.1, 131.4, 128.5, 128.2, 128.2, 128.2, 128.1, 127.8, 126.8, 125.4, 123.5, 119.6, 111.3, 107.78, 89.7, 85.7, 84.0, 82.8, 67.0, 39.3, 36.8, 28.1. HRMS (ESI) $[M+Na]^+$ calcd for $C_{36}H_{34}O_5Na^+$, 569.2298, found 569.2294. (Chiral IE-3, λ= 254 nm, *n*-hexane/2-propanol= 19/1, Flow rate = 1.0 mL/min), t_R = 11.457 min(major), 22.875 min.

HPLC chromatogram of racemic 53

Condition: n-hexane/2-propanol =19:1

Flow rate =1.0 mL/min

λ= 254 nm

Chiral IE-3

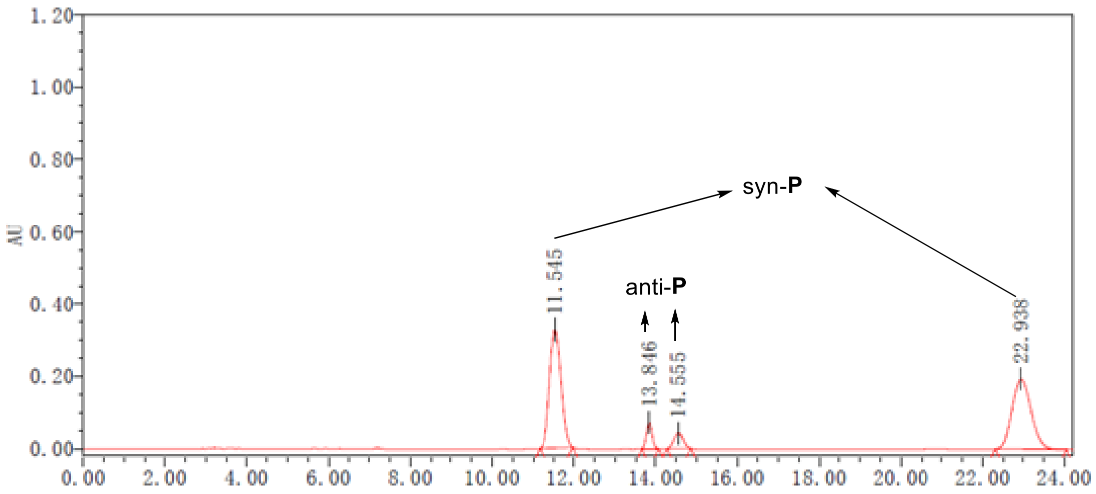

Entry	Retention Time/min	Area	Height	Area(%)
1	11.545	6404622	326793	45.35
2	13.846	699675	73171	4.95
3	14.555	673424	42324	4.77
4	22.938	6345621	194513	44.93

Supplementary Figure 115. Chiral HPLC analysis of racemic **53**

HPLC chromatogram of chiral 53

Condition: n-hexane/2-propanol =19:1

Flow rate =1.0 mL/min

λ= 254 nm

Chiral IE-3

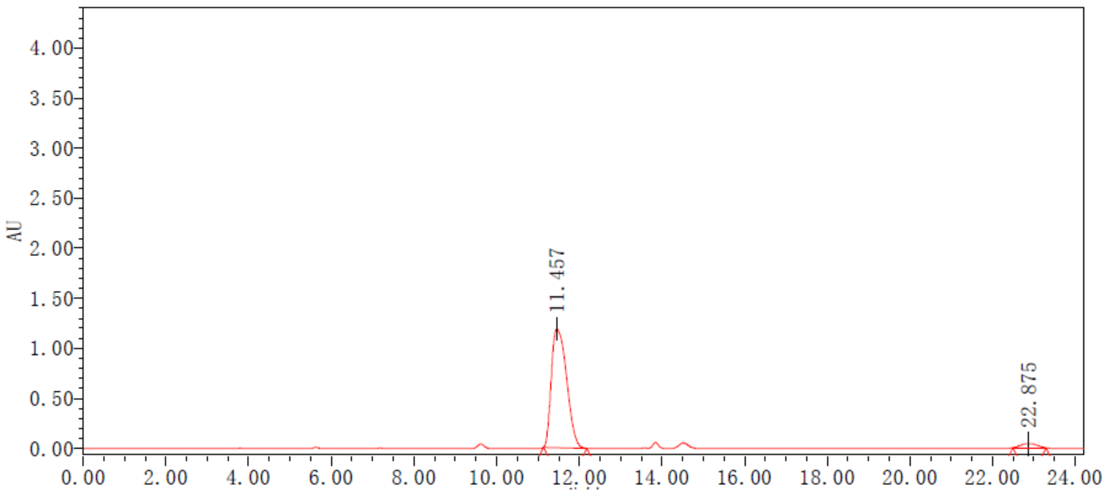

The larger version of HPLC chromatogram of chiral 53

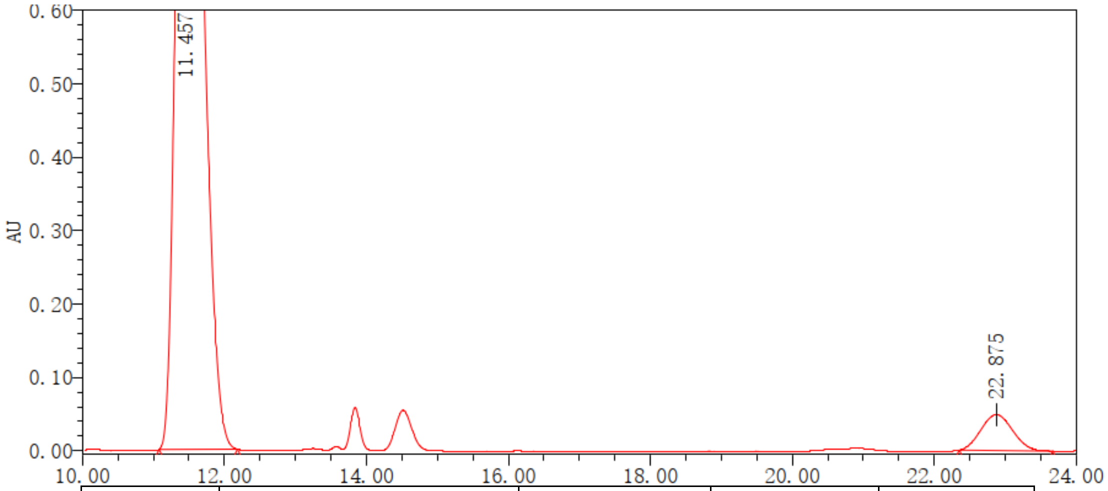

Entry	Retention Time/min	Area	Height	Area(%)
1	11.457	30481356	1184631	96.48
2	22.875	1105463	41600	3.52

Supplementary Figure 116. Chiral HPLC analysis of chiral **53**

Tert-butyl (2S,3R)-2-((3-(4-chlorophenyl)prop-2-yn-1-yl)oxy)-3-(2-oxo-2-phenylethyl)-2,5-diphenylpent-4-ynoate (54)

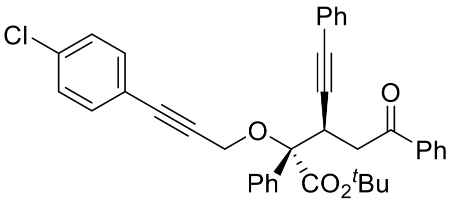

54

Colorless oil; 45.9 mg, 52% yield, >20:1 d.r., 92% *ee*; ^{1}H NMR (400 MHz, $CDCl_3$) δ 7.93 – 7.87 (m, 2H), 7.74 – 7.69 (m, 2H), 7.51 (t, *J* = 7.4 Hz, 1H), 7.38 (dd, *J* = 5.8, 4.1 Hz, 2H), 7.36 – 7.31 (m, 5H), 7.27 – 7.24 (m, 4H), 7.24 – 7.20 (m, 3H), 5.07 (d, *J* = 15.7 Hz, 1H), 4.79 (d, *J* = 15.7 Hz, 1H), 4.40 (dd, *J* = 10.5, 2.8 Hz, 1H), 3.62 (dd, *J* = 16.9, 10.5 Hz, 1H), 3.05 (dd, *J* = 16.8, 2.8 Hz, 1H), 1.52 (s, 9H). ^{13}C NMR (101 MHz, $CDCl_3$) δ 197.6, 169.2, 137.9, 137.0, 134.4, 133.1, 133.1, 131.5, 128.6, 128.5, 128.4, 128.2, 128.2, 127.9, 126.7, 123.3, 121.4, 89.4, 87.5, 86.2, 84.7, 84.0, 83.1, 56.3, 39.1, 36.4, 28.0. Peak overlapping was observed. HRMS (ESI) $[M+Na]^+$ calcd for $C_{38}H_{33}O_4ClNa^+$, 611.1960, found 611.1960. (Chiral IA, λ= 254 nm, *n*-hexane/2-propanol= 19/1, Flow rate = 1.0 mL/min), t_R = 7.254 min(major), 14.901 min.

HPLC chromatogram of racemic 54

Condition: n-hexane/2-propanol =19:1

Flow rate =1.0 mL/min

λ= 254 nm

Chiral IA

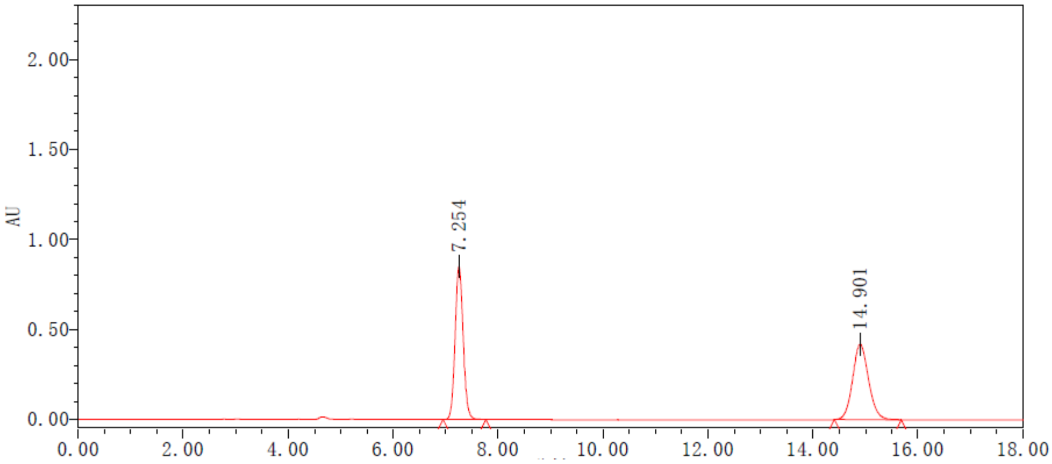

Entry	Retention Time/min	Area	Height	Area(%)
1	7.254	7.254	851140	50.28
2	14.901	14.901	419548	49.72

Supplementary Figure 117. Chiral HPLC analysis of racemic **54**

HPLC chromatogram of chiral 54

Condition: n-hexane/2-propanol =19:1

Flow rate =1.0 mL/min

λ= 254 nm

Chiral IA

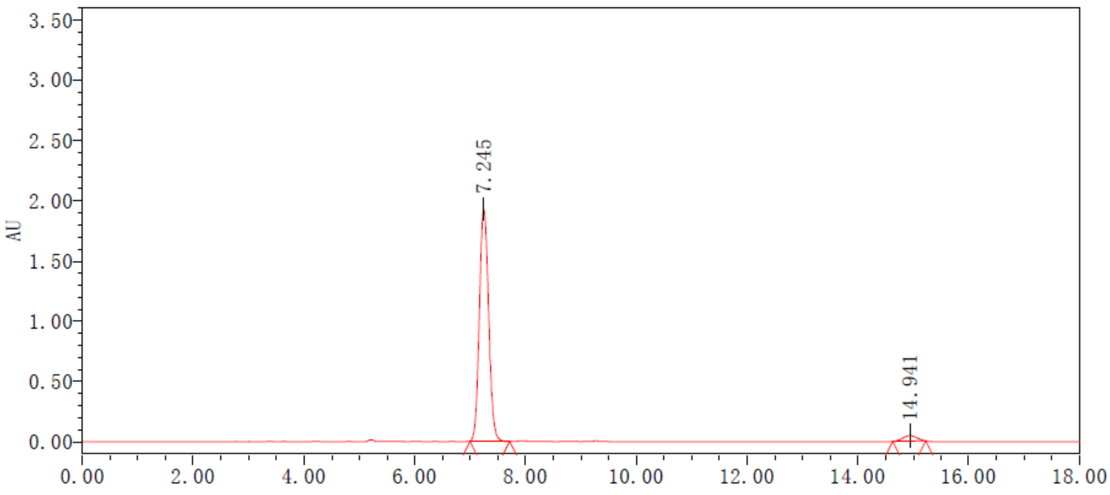

The larger version of HPLC chromatogram of chiral 54

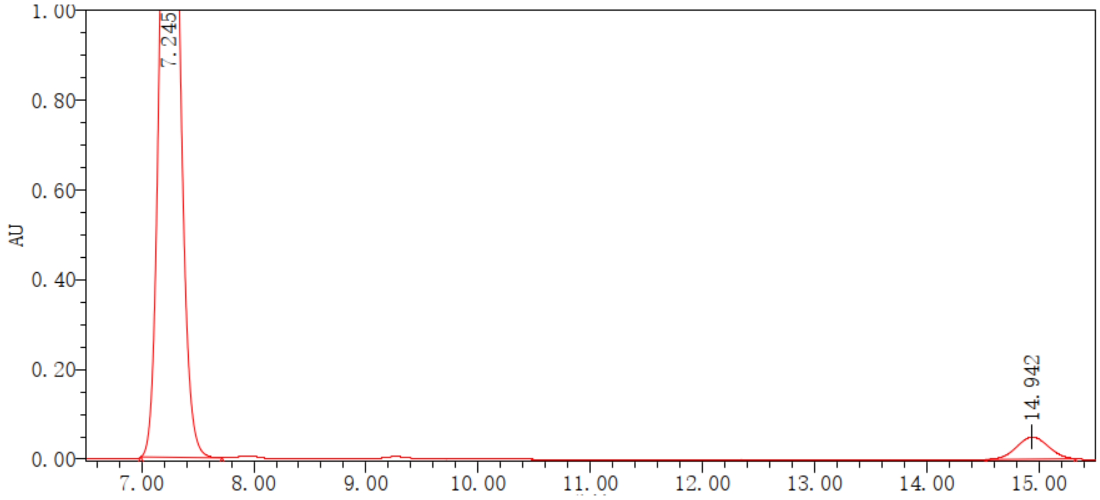

Entry	Retention Time/min	Area	Height	Area(%)
1	7.245	22158810	1925063	96.37
2	14.941	810673	45317	3.63

Supplementary Figure 118. Chiral HPLC analysis of chiral **54**

Tert-butyl (2S,3R)-2-(allyloxy)-3-(2-oxo-2-phenylethyl)-2,5-diphenylpent-4-ynoate (55)

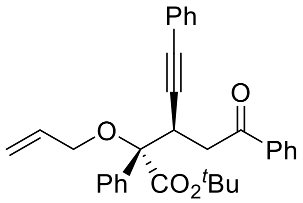

55

Colorless oil; 45.3 mg, 63% yield, >20:1 d.r., 89% *ee*; ^{1}H NMR (400 MHz, $CDCl_3$) δ 7.90 (d, J = 7.9 Hz, 2H), 7.68 (d, J = 8.0 Hz, 2H), 7.53 (t, J = 7.3 Hz, 1H), 7.42 (t, J = 7.6 Hz, 2H), 7.36 (t, J = 7.4 Hz, 2H), 7.31 (d, J = 7.1 Hz, 1H), 7.24 (d, J = 13.6 Hz, 5H), 6.05 (ddd, J = 16.0, 10.3, 5.1 Hz, 1H), 5.41 (d, J = 17.2 Hz, 1H), 5.20 (d, J = 10.4 Hz, 1H), 4.54 (dd, J = 12.7, 5.2 Hz, 1H), 4.35 (ddd, J = 13.1, 11.6, 3.6 Hz, 2H), 3.34 (dd, J = 16.6, 10.2 Hz, 1H), 3.13 (dd, J = 16.6, 2.7 Hz, 1H), 1.51 (s, 9H). ^{13}C NMR (126 MHz, $CDCl_3$) δ 197.6, 169.5, 138.6, 137.0, 135.2, 133.1, 131.4, 128.5, 128.2, 128.2, 128.1, 127.8, 126.8, 123.5, 116.0, 89.7, 85.6, 83.9, 82.7, 67.7, 39.3, 36.7, 28.1. HRMS (ESI) $[M+Na]^+$ calcd for $C_{32}H_{32}O_4Na^+$, 503.2193, found 503.2189. (Chiral IE-3, λ= 254 nm, *n*-hexane/2-propanol= 19/1, Flow rate = 1.0 mL/min), t_R = 8.725 min(major), 19.158 min.

HPLC chromatogram of racemic 55

Condition: n-hexane/2-propanol =19:1

Flow rate =1.0 mL/min

λ= 254 nm

Chiral IE-3

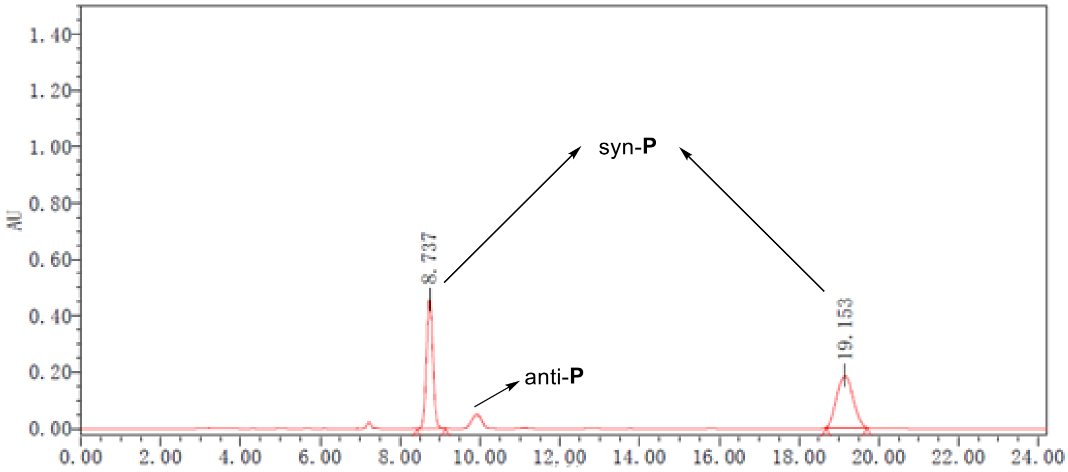

Entry	Retention Time/min	Area	Height	Area(%)
1	8.737	5225981	459523	49.91
2	19.153	5244627	184171	50.09

Supplementary Figure 119. Chiral HPLC analysis of racemic **55**

HPLC chromatogram of chiral 55

Condition: n-hexane/2-propanol =19:1

Flow rate =1.0 mL/min

λ= 254 nm

Chiral IE-3

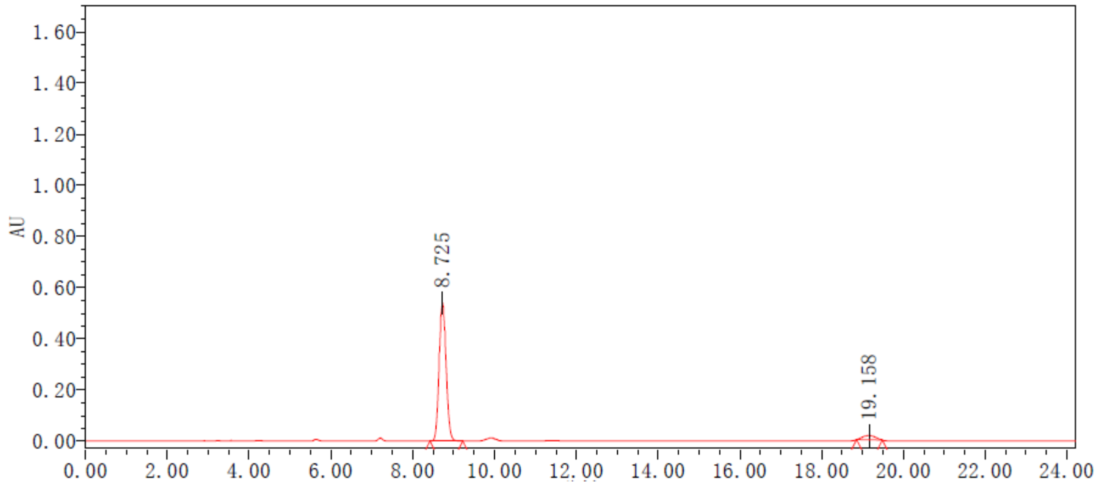

The larger version of HPLC chromatogram of chiral 55

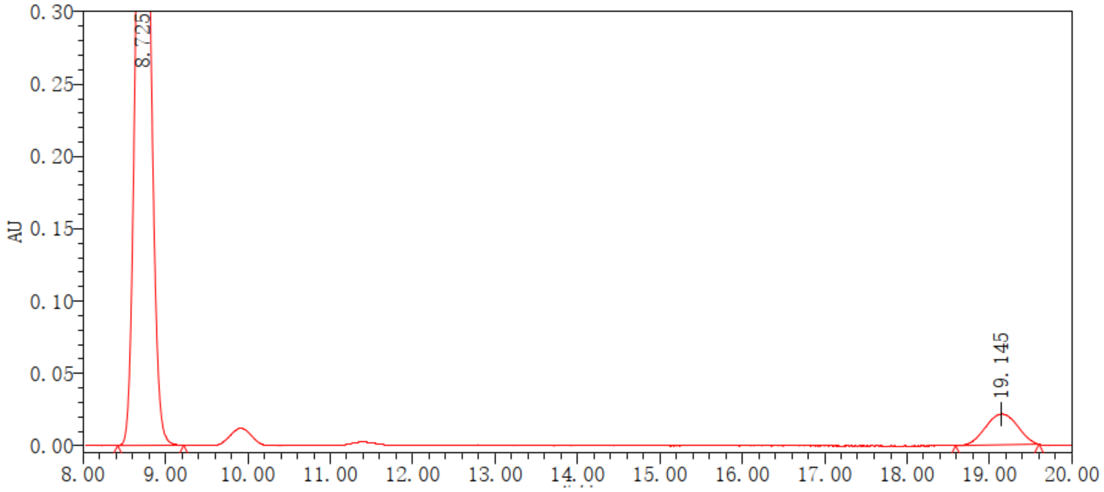

Entry	Retention Time/min	Area	Height	Area(%)
1	8.725	6624480	539623	94.48
2	19.158	365027	16480	5.52

Supplementary Figure 120. Chiral HPLC analysis of chiral **55**

Tert-butyl (2S,3R)-2-(but-3-en-1-yloxy)-3-(2-oxo-2-phenylethyl)-2,5-diphenylpent-4-ynoate (56)

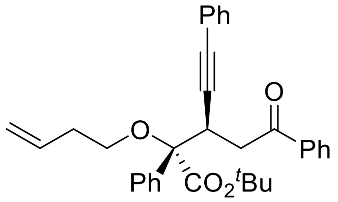

56

Colorless oil; 45.2 mg, 62% yield, >20:1 d.r., 90% *ee*; ^{1}H NMR (400 MHz, $CDCl_3$) δ 7.89 (d, *J* = 7.6 Hz, 2H), 7.66 (d, *J* = 7.7 Hz, 2H), 7.53 (t, *J* = 7.3 Hz, 1H), 7.42 (t, *J* = 7.6 Hz, 2H), 7.35 (t, *J* = 7.5 Hz, 2H), 7.30 (d, *J* = 7.1 Hz, 1H), 7.24 (d, *J* = 12.9 Hz, 5H), 5.95 (ddt, *J* = 17.0, 10.2, 6.7 Hz, 1H), 5.14 (d, *J* = 17.2 Hz, 1H), 5.06 (d, *J* = 10.5 Hz, 1H), 4.31 (dd, *J* = 10.0, 2.8 Hz, 1H), 4.05 (dd, *J* = 15.3, 6.8 Hz, 1H), 3.88 (dd, *J* = 15.2, 6.7 Hz, 1H), 3.29 (dd, *J* = 16.6, 10.1 Hz, 1H), 3.12 (dd, *J* = 16.6, 2.8 Hz, 1H), 2.48 (dd, *J* = 13.1, 6.5 Hz, 2H), 1.51 (s, 9H). ^{13}C NMR (126 MHz, $CDCl_3$) δ 197.7, 169.5, 138.8, 137.0, 135.6, 133.1, 131.4, 128.5, 128.2, 128.1, 128.1, 128.0, 127.7, 126.7, 123.6, 116.4, 89.8, 85.3, 83.9, 82.6, 65.8, 35.0, 28.1. HRMS (ESI) $[M+Na]^+$ calcd for $C_{33}H_{34}O_4Na^+$, 517.2349, found 517.2346. (Chiral IA, λ= 254 nm, *n*-hexane/2-propanol= 49/1, Flow rate = 1.0 mL/min), t_R = 7.426 min(major), 14.607 min.

HPLC chromatogram of racemic 56

Condition: n-hexane/2-propanol =49:1

Flow rate =1.0 mL/min

λ= 254 nm

Chiral IA

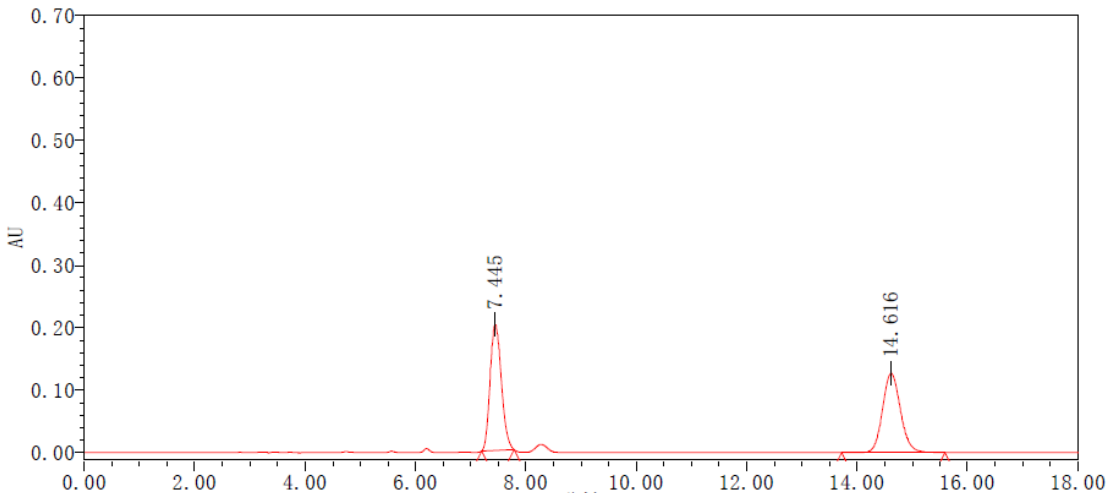

Entry	Retention Time/min	Area	Height	Area(%)
1	7.445	2893437	201301	50.30
2	14.616	2858742	127090	49.70

Supplementary Figure 121. Chiral HPLC analysis of racemic **56**

HPLC chromatogram of chiral 56

Condition: n-hexane/2-propanol =49:1

Flow rate =1.0 mL/min

λ= 254 nm

Chiral IA

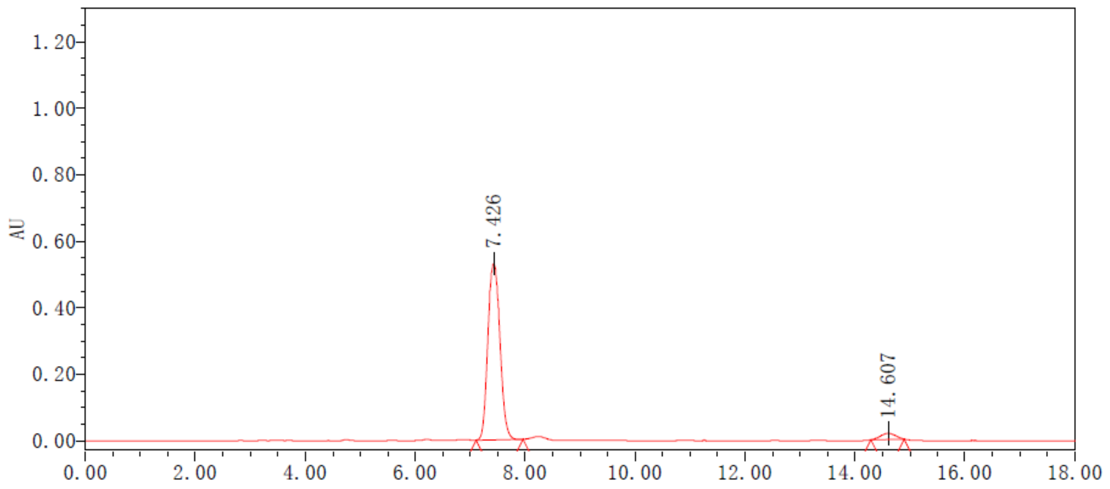

The larger version of HPLC chromatogram of chiral 56

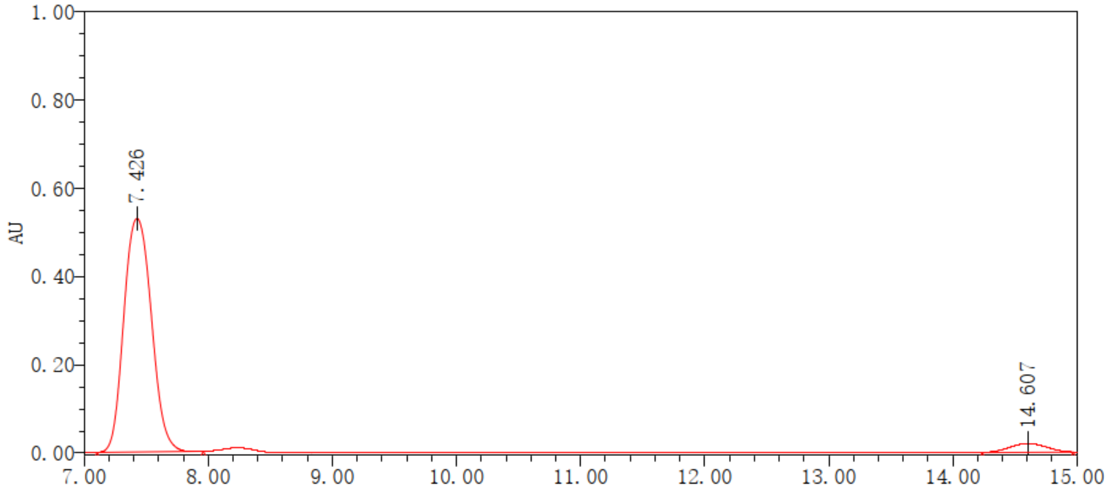

Entry	Retention Time/min	Area	Height	Area(%)
1	7.426	8186509	529686	95.14
2	14.607	345935	18208	4.86

Supplementary Figure 122. Chiral HPLC analysis of chiral **56**

Tert-butyl (2S,3R)-3-(2-oxo-2-phenylethyl)-2-(pent-4-en-1-yloxy)-2,5-diphenylpent-4-ynoate (57)

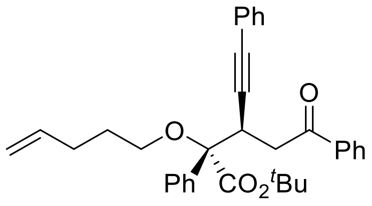

57

Colorless oil; 45.2 mg, 52% yield, >20:1 d.r., 90% *ee*; ^{1}H NMR (400 MHz, $CDCl_3$) δ 7.92 – 7.87 (m, 2H), 7.69 – 7.64 (m, 2H), 7.54 (t, *J* = 7.4 Hz, 1H), 7.43 (t, *J* = 7.6 Hz, 2H), 7.36 (t, *J* = 7.4 Hz, 2H), 7.30 (d, *J* = 7.2 Hz, 1H), 7.24 (d, *J* = 14.6 Hz, 5H), 5.87 (ddt, *J* = 16.9, 10.2, 6.6 Hz, 1H), 5.04 (ddd, *J* = 17.1, 3.3, 1.6 Hz, 1H), 4.96 (dd, *J* = 10.2, 1.8 Hz, 1H), 4.31 (dd, *J* = 10.1, 3.2 Hz, 1H), 3.98 (dt, *J* = 8.8, 6.6 Hz, 1H), 3.81 (dt, *J* = 8.9, 6.4 Hz, 1H), 3.27 (dd, *J* = 16.6, 10.1 Hz, 1H), 3.12 (dd, *J* = 16.6, 3.1 Hz, 1H), 2.24 (dd, *J* = 14.2, 7.4 Hz, 2H), 1.89 – 1.73 (m, 2H), 1.50 (s, 9H). ^{13}C NMR (126 MHz, $CDCl_3$) δ 197.7, 169.6, 138.9, 138.6, 137.0, 133.0, 131.4, 128.5, 128.2, 128.1, 128.1, 128.0, 127.7, 126.8, 123.6, 114.7, 89.8, 85.2, 83.9, 82.5, 65.8, 39.3, 36.3, 30.5, 29.73, 28.1. HRMS (ESI) $[M+Na]^+$ calcd for $C_{34}H_{36}O_4Na^+$, 531.2509, found 531.2506. (Chiral IC, λ= 254 nm, *n*-hexane/2-propanol= 49/1, Flow rate = 1.0 mL/min), t_R = 7.432 min(major), 12.362 min.

HPLC chromatogram of racemic 57

Condition: n-hexane/2-propanol =49:1

Flow rate =1.0 mL/min

λ= 254 nm

Chiral IC

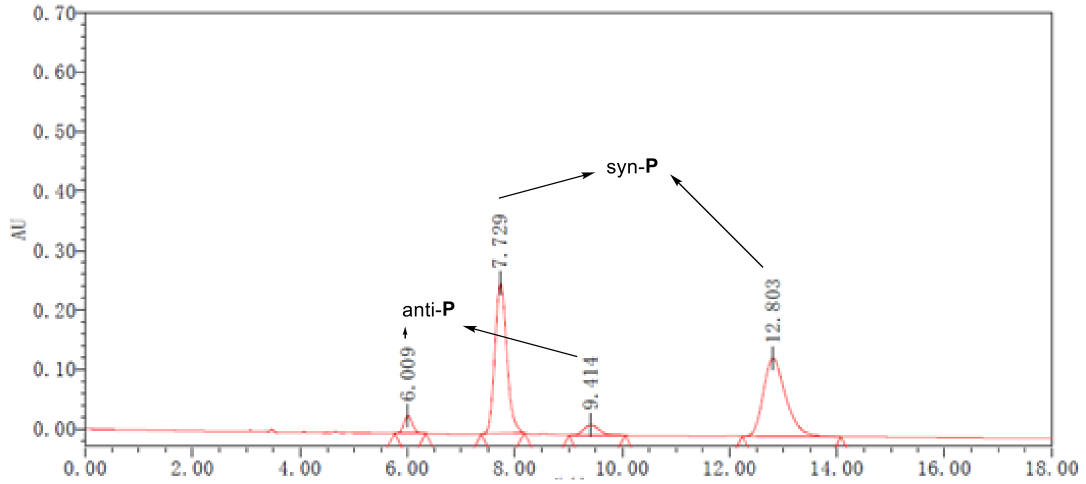

Entry	Retention Time/min	Area	Height	Area(%)
1	6.009	336436	29596	4.03
2	7.729	3848417	252827	46.12
3	9.414	342179	16979	4.10
4	12.803	3817076	131176	45.75

Supplementary Figure 123. Chiral HPLC analysis of racemic **57**

HPLC chromatogram of chiral 57

Condition: n-hexane/2-propanol =49:1

Flow rate =1.0 mL/min

λ= 254 nm

Chiral IC

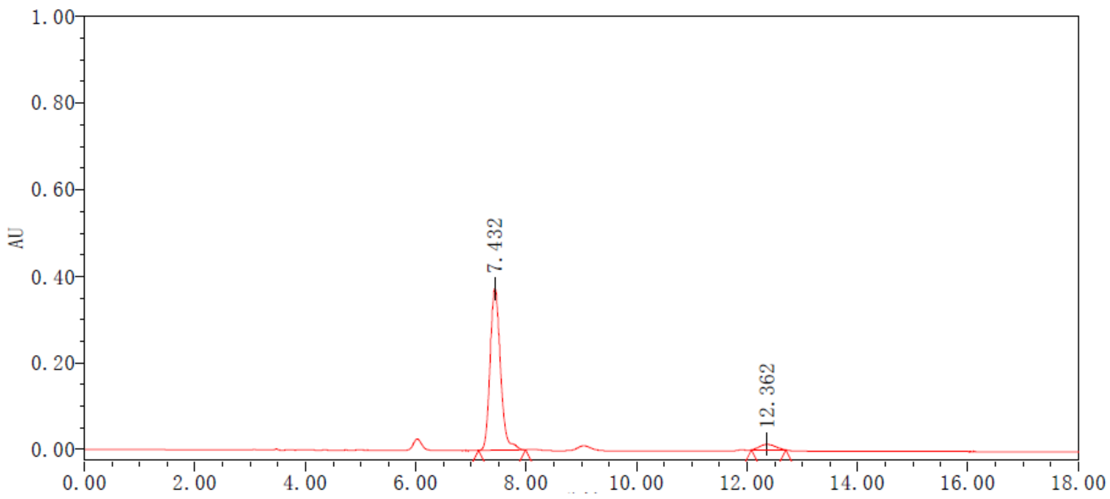

Entry	Retention Time/min	Area	Height	Area(%)
1	7.432	4948850	373399	95.02
2	12.362	259610	12978	4.98

Supplementary Figure 124. Chiral HPLC analysis of chiral **57**

Tert-butyl (2S,3R)-2-(hex-5-en-1-yloxy)-3-(2-oxo-2-phenylethyl)-2,5-diphenylpent-4-ynoate (58)

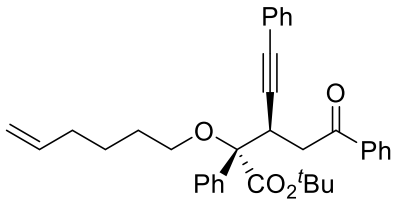

58

Colorless oil; 39.2 mg, 50% yield, >20:1 d.r., 91% *ee*; ^{1}H NMR (400 MHz, $CDCl_3$) δ 7.92 – 7.88 (m, 2H), 7.68 – 7.64 (m, 2H), 7.56 – 7.50 (m, 1H), 7.43 (t, *J* = 7.6 Hz, 2H), 7.36 (t, *J* = 7.4 Hz, 2H), 7.30 (d, *J* = 7.2 Hz, 1H), 7.24 (d, *J* = 14.4 Hz, 5H), 5.81 (ddt, *J* = 16.9, 10.2, 6.6 Hz, 1H), 5.00 (ddd, *J* = 17.1, 3.4, 1.6 Hz, 1H), 4.94 (dd, *J* = 10.2, 1.0 Hz, 1H), 4.30 (dd, *J* = 9.8, 3.3 Hz, 1H), 3.96 (dt, *J* = 8.8, 6.5 Hz, 1H), 3.26 (dd, *J* = 16.6, 9.9 Hz, 1H), 3.15 (dd, *J* = 16.6, 3.3 Hz, 1H), 2.10 (dd, *J* = 14.4, 7.1 Hz, 2H), 1.71 (dt, *J* = 12.7, 6.6 Hz, 2H), 1.61 – 1.56 (m, 2H), 1.51 (s, 9H). ^{13}C NMR (101 MHz, $CDCl_3$) δ 197.7, 169.7, 138.9, 138.9, 137.0, 133.0, 131.4, 128.5, 128.2, 128.1, 128.0,

128.0, 127.7, 126.8, 123.6, 114.5, 89.8, 85.2, 83.8, 82.5, 66.2, 39.4, 36.4, 33.7, 30.0, 28.1, 25.6. HRMS (ESI) $[M+Na]^+$ calcd for $C_{35}H_{38}O_4Na^+$, 545.2662, found 545.2663. (Chiral IC, λ= 254 nm, *n*-hexane/2-propanol/EtOH= 197/1/2, Flow rate = 1.0 mL/min), t_R = 6.752 min(major), 14.838 min.

HPLC chromatogram of racemic 58

Condition: n-hexane/2-propanol/EtOH = 197:1:2

Flow rate =1.0 mL/min

λ= 254 nm

Chiral IC

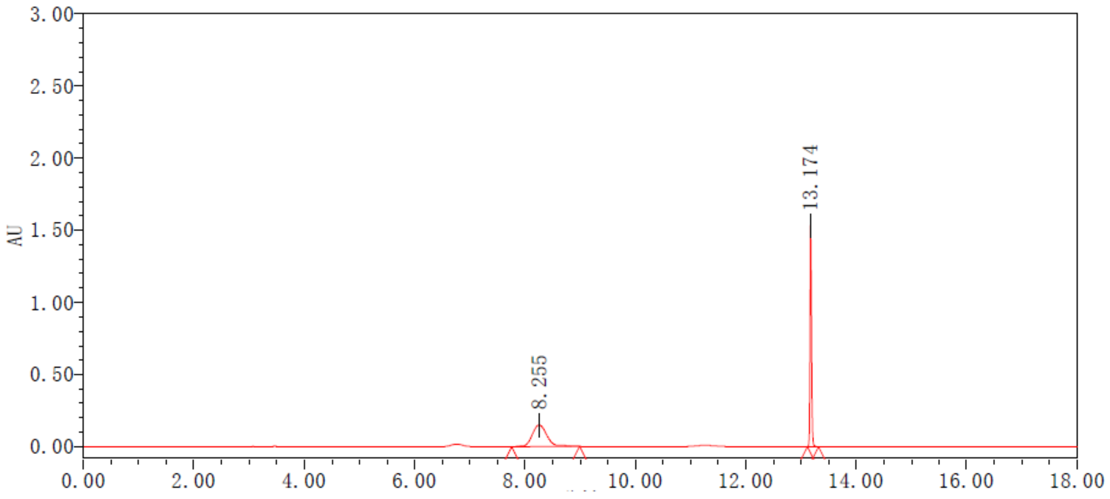

Entry	Retention Time/min	Area	Height	Area(%)
1	8.255	2783408	149850	50.23
2	13.174	2758327	1532847	49.77

Supplementary Figure 125. Chiral HPLC analysis of racemic **58**

HPLC chromatogram of chiral 58

Condition: n-hexane/2-propanol/EtOH = 197:1:2

Flow rate =1.0 mL/min

λ= 254 nm

Chiral IC

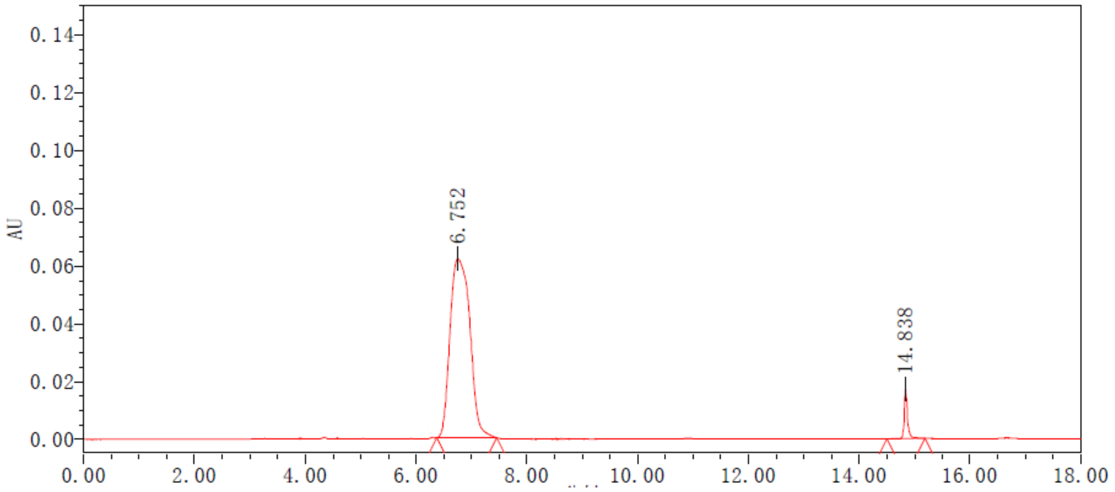

Entry	Retention Time/min	Area	Height	Area(%)
1	6.752	1545035	61969	95.52
2	14.838	70082	17161	4.48

Supplementary Figure 126. Chiral HPLC analysis of chiral **58**

Tert-butyl (2S,3R)-2-(((Z)-3,8-dimethylnona-2,7-dien-1-yl)oxy)-3-(2-oxo-2-phenylethyl)-2,5-diphenylpent-4-ynoate (59)

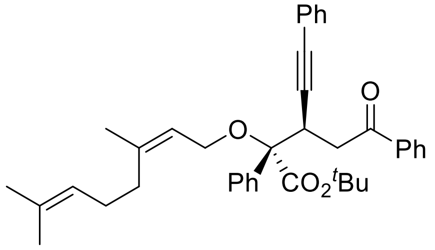

59

Colorless oil; 52.7 mg, 61% yield, 9:1 d.r., 90% *ee*; ^{1}H NMR (400 MHz, $CDCl_3$) δ 7.92 – 7.88 (m, 2H), 7.70 – 7.65 (m, 2H), 7.52 (dd, *J* = 10.5, 4.2 Hz, 1H), 7.42 (t, *J* = 7.6 Hz, 2H), 7.35 (dd, *J* = 8.2, 6.6 Hz, 2H), 7.29 (d, *J* = 7.2 Hz, 1H), 7.24 – 7.19 (m, 5H), 5.51 (t, *J* = 6.1 Hz, 1H), 5.02 (dd, *J* = 6.4, 5.2 Hz, 1H), 4.55 (dd, *J* = 11.1, 6.9 Hz, 1H), 4.35 – 4.25 (m, 2H), 3.32 (dd, *J* = 16.7, 10.2 Hz, 1H), 3.15 (dd, *J* = 16.7, 3.0 Hz, 1H), 2.07 (td, *J* = 8.7, 4.5 Hz, 3H), 2.02 (d, *J* = 3.3 Hz, 1H), 1.77 (t, *J* = 2.5 Hz, 3H), 1.61 (s, 1H),

1.59 (s, 3H), 1.55 (s, 3H), 1.51 (s, 9H), 1.35 (dd, J = 30.2, 12.4 Hz, 1H). ^{13}C NMR (101 MHz, $CDCl_3$) δ 197.7, 169.7, 139.5, 138.8, 137.1, 133.0, 131.8, 131.4, 128.5, 128.2, 128.1, 128.0, 127.7, 126.9, 123.9, 123.6, 122.4, 121.6, 100.1, 89.8, 85.4, 83.9, 82.5, 63.4, 39.4, 36.5, 32.5, 28.1, 28.0, 26.8, 25.7, 23.5, 17.7. HRMS (ESI) $[M+Na]^+$ calcd for $C_{39}H_{44}O_4Na^+$, 599.3132, found 599.3128. (Chiral ID-3, λ= 254 nm, *n*-hexane/2-propanol= 49/1, Flow rate = 1.0 mL/min), t_R = 7.648 min(major), 20.584 min.

HPLC chromatogram of racemic 59

Condition: n-hexane/2-propanol =49:1

Flow rate =1.0 mL/min

λ= 254 nm

Chiral ID-3

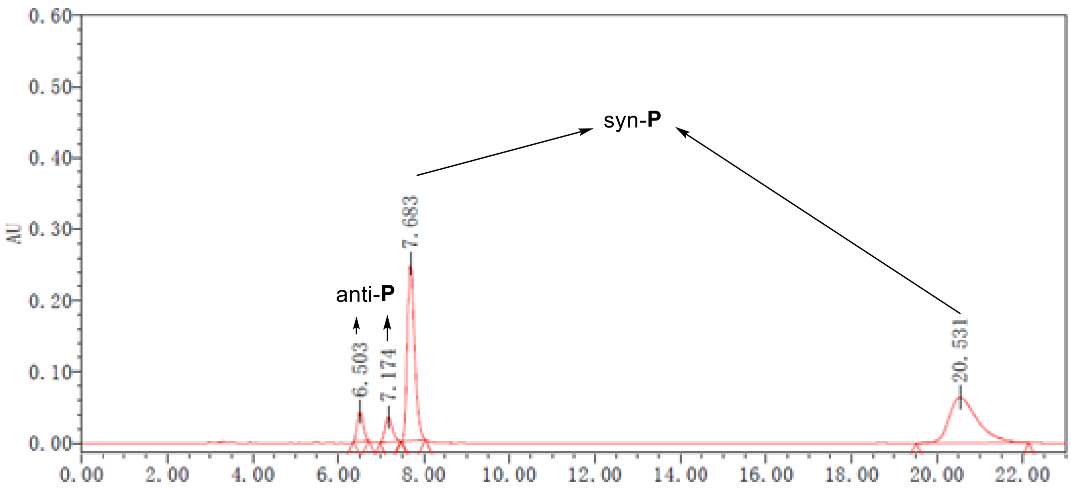

Entry	Retention Time/min	Area	Height	Area(%)
1	6.503	410294	42068	6.12
2	7.174	411303	34923	6.13
3	7.683	2945133	247583	43.92
4	20.531	2938239	63610	43.82

Supplementary Figure 127. Chiral HPLC analysis of racemic **59**

HPLC chromatogram of chiral 59

Condition: n-hexane/2-propanol =49:1

Flow rate =1.0 mL/min

λ= 254 nm

Chiral ID-3

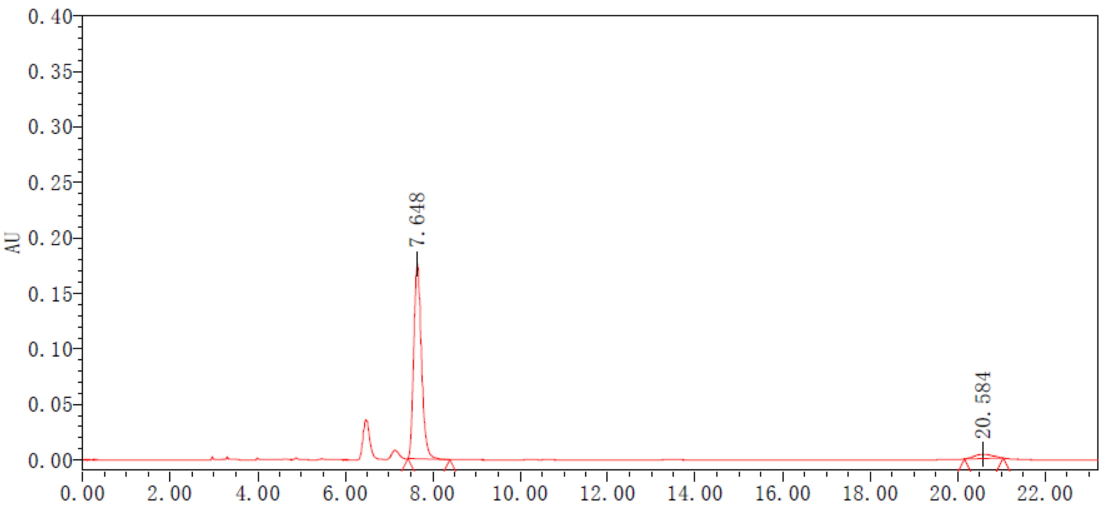

The larger version of HPLC chromatogram of chiral 59

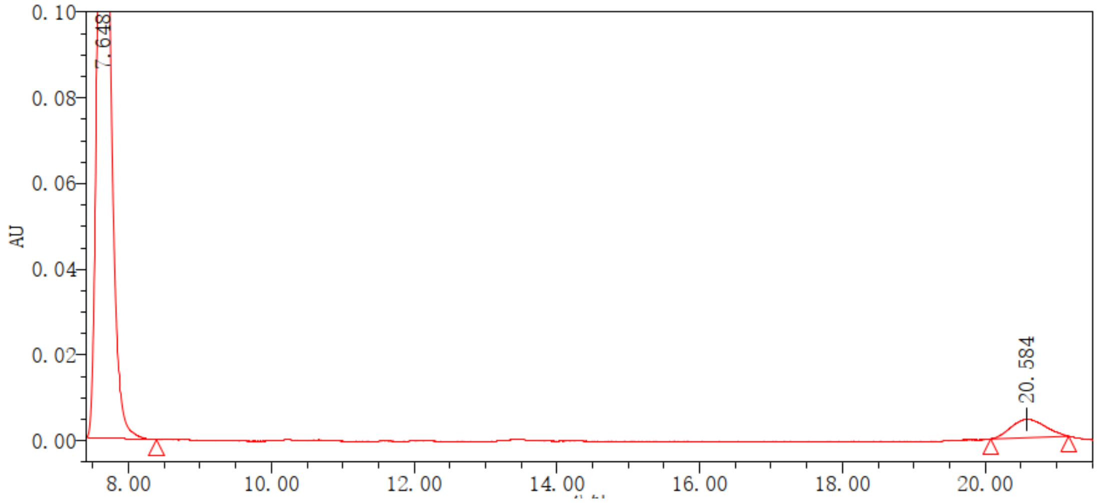

Entry	Retention Time/min	Area	Height	Area(%)
1	7.648	2200522	175662	95.25
2	20.584	109760	3728	4.75

Supplementary Figure 128. Chiral HPLC analysis of chiral **59**

Tert-butyl (2S,3R)-2-(2-(4-(benzyloxy)-4-oxobutoxy)ethoxy)-3-(2-oxo-2-

phenylethyl)-2,5-diphenylpent-4-ynoate (*syn*-60)

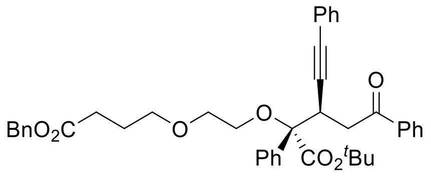

syn-**60**

Colorless oil; 53% yield (17.5 mg), >20:1 d.r., 97% ee. ^{1}H NMR (500 MHz, $CDCl_3$) δ 7.91 (d, *J* = 7.5 Hz, 2H), 7.68 (d, *J* = 7.6 Hz, 2H), 7.50 (t, *J* = 7.3 Hz, 1H), 7.41 (dd, *J* = 15.8, 7.8 Hz, 3H), 7.36 – 7.30 (m, 8H), 7.21 (d, *J* = 2.5 Hz, 4H), 5.08 (s, 2H), 4.32 (dd, *J* = 10.2, 2.8 Hz, 1H), 4.19 (dd, *J* = 9.5, 5.1 Hz, 1H), 4.03 – 3.95 (m, 1H), 3.70 (dd, *J* = 11.8, 7.2 Hz, 1H), 3.56 (t, *J* = 6.2 Hz, 2H), 3.41 (dd, *J* = 16.7, 10.3 Hz, 1H), 3.08 (dd, *J* = 16.7, 2.6 Hz, 1H), 2.46 (dd, *J* = 13.5, 7.2 Hz, 2H), 1.93 (dd, *J* = 12.3, 6.1 Hz, 2H), 1.50 (s, 9H). ^{13}C NMR (126 MHz, $CDCl_3$) δ 197.7, 173.4, 173.3, 169.4, 138.6, 137.0, 136.1, 133.1, 131.4, 128.6, 128.5, 128.4, 128.3, 128.2, 128.2, 128.2, 128.1, 128.1, 128.1, 127.8, 127.2, 126.8, 123.5, 89.9, 85.3, 82.7, 81.7, 81.6, 70.6, 70.2, 70.1, 68.8, 66.2, 65.9, 39.0, 31.0, 28.1, 27.9, 25.2. HRMS (ESI) $[M+Na]^+$ calcd for $C_{42}H_{44}O_7Na^+$, 683.2979, found 683.2975. (Chiral IA-3, λ= 254 nm, *n*-hexane/2-propanol= 9/1, Flow rate = 1.0 mL/min), t_R = 7.954 min (major), 13.564 min.

HPLC chromatogram of racemic 60

Condition: n-hexane/2-propanol =9:1

Flow rate =1.0 mL/min

λ= 254 nm

Chiral IA-3

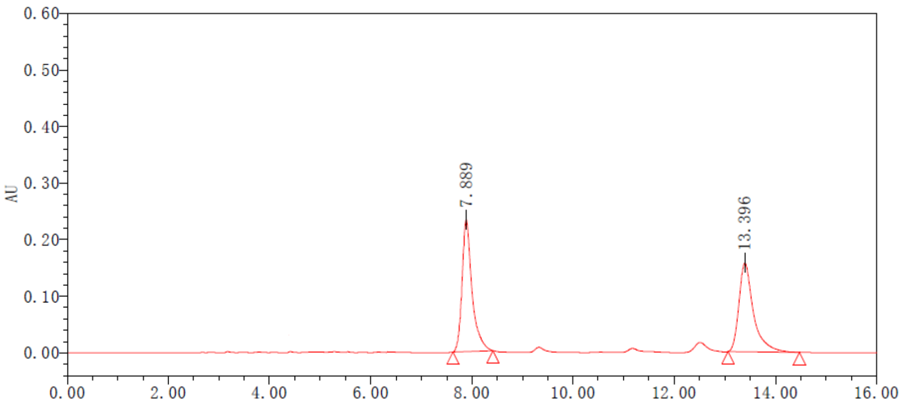

Entry	Retention Time/min	Area	Height	Area(%)
1	7.889	3170989	232747	50.19
2	13.396	3146358	157120	49.81

Supplementary Figure 129. Chiral HPLC analysis of racemic **60**

HPLC chromatogram of chiral 60

Condition: n-hexane/2-propanol =9:1

Flow rate =1.0 mL/min

λ= 254 nm

Chiral IA-3

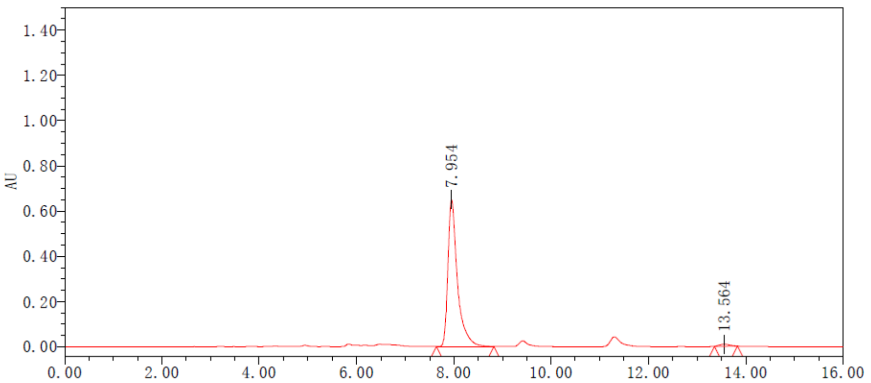

Entry	Retention Time/min	Area	Height	Area(%)
1	7.954	8774656	650002	98.53
2	13.564	130850	9043	1.47

Supplementary Figure 130. Chiral HPLC analysis of chiral **60**

Tert-butyl (2S,3R)-2-((4-bromobenzyl)oxy)-2-(3,4-dichlorophenyl)-3-(2-oxo-2-phenylethyl)-5-phenylpent-4-ynoate (61)

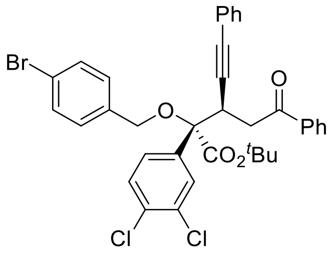

61

Colorless oil; 65.9 mg, 65% yield, >20:1 d.r., >99% *ee*, $[\alpha]_D^{20}$ = -12.36 (c = 0.1, CH_2Cl_2); ^1H NMR (400 MHz, $CDCl_3$) δ 7.85 (d, *J* = 7.6 Hz, 3H), 7.55 (t, *J* = 8.3 Hz, 1H), 7.52 (s, 1H), 7.49 (d, *J* = 8.5 Hz, 2H), 7.46 – 7.41 (m, 3H), 7.34 (d, *J* = 8.2 Hz, 2H), 7.26 (d, *J* = 9.1 Hz, 5H), 4.99 (d, *J* = 11.5 Hz, 1H), 4.87 (d, *J* = 11.5 Hz, 1H), 4.35 (dd, *J* = 9.7, 3.2 Hz, 1H), 3.31 (dd, *J* = 16.7, 9.8 Hz, 1H), 3.09 (dd, *J* = 16.7, 3.1 Hz, 1H), 1.52 (s, 9H). ^{13}C NMR (101 MHz, $CDCl_3$) δ 197.0, 168.5, 138.7, 137.3, 136.7, 133.3, 132.6, 131.5, 131.4, 130.2, 129.3, 128.7, 128.3, 128.2, 128.2, 126.1, 123.0, 121.6, 88.8, 85.1, 84.6, 83.7, 68.2, 39.1, 36.6, 28.1, 28.0. HRMS (ESI) $[M+Na]^+$ calcd for $C_{36}H_{31}O_4Cl_2BrNa^+$, 699.0675, found 699.0674. (Chiral IE-3, λ= 254 nm, *n*-hexane/2-propanol= 97/3, Flow rate = 1.0 mL/min), t_R = 7.401 min(major), 10.092 min.

HPLC chromatogram of racemic 61

Condition: n-hexane/2-propanol =97:3

Flow rate =1.0 mL/min

λ= 254 nm

Chiral IE-3

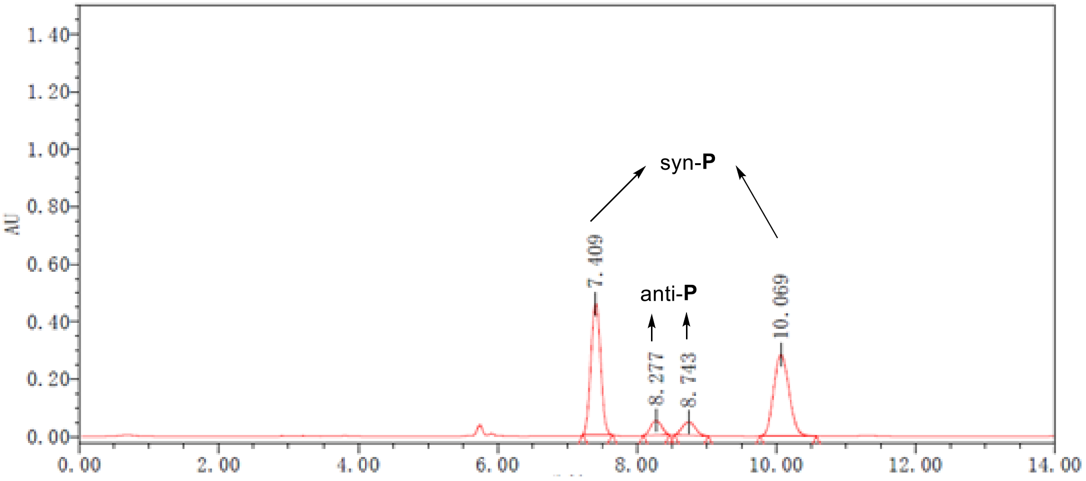

Entry	Retention Time/min	Area	Height	Area(%)
1	7.409	4374540	458002	44.26
2	8.277	559177	50697	5.66
3	8.743	613385	46416	6.21
4	10.069	4337024	283557	43.88

Supplementary Figure 131. Chiral HPLC analysis of racemic **61**

HPLC chromatogram of chiral 61

Condition: n-hexane/2-propanol =97:3

Flow rate =1.0 mL/min

λ= 254 nm

Chiral IE-3

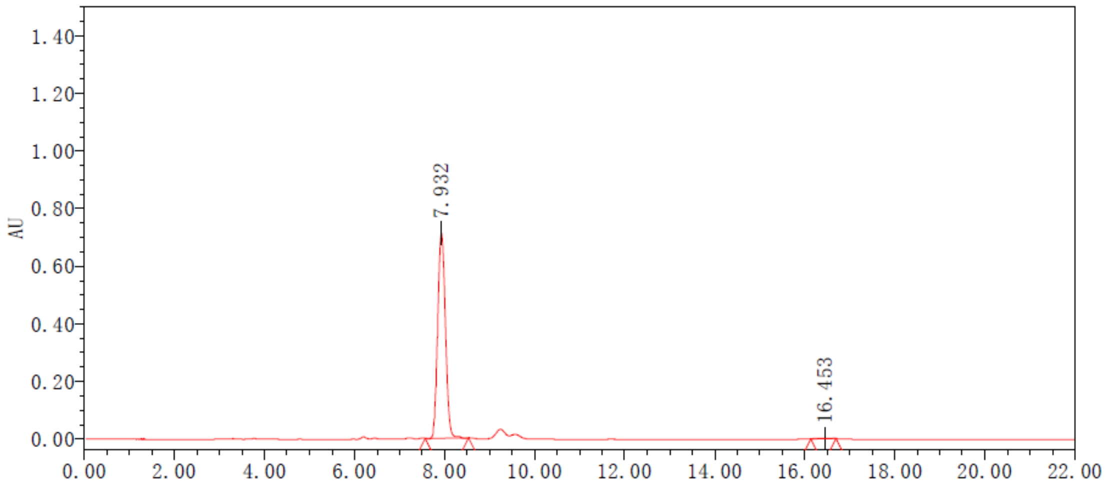

The larger version of HPLC chromatogram of chiral 61

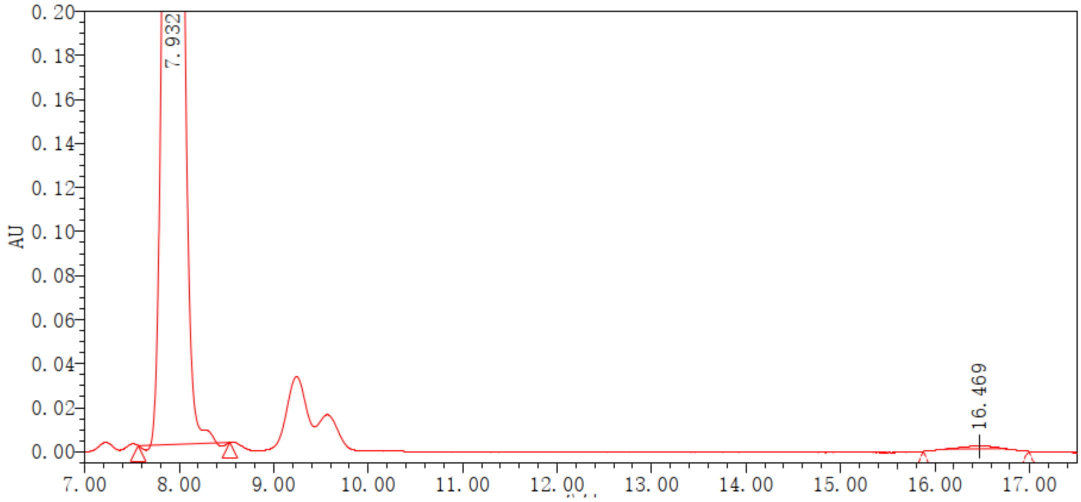

Entry	Retention Time/min	Area	Height	Area(%)
1	7.932	8397971	711773	99.76
2	16.453	19993	970	0.24

Supplementary Figure 132. Chiral HPLC analysis of chiral **61**

Tert-butyl (2S,3R)-2-((4-bromobenzyl)oxy)-2-(4-methoxyphenyl)-3-(2-oxo-2-phenylethyl)-5-phenylpent-4-ynoate (62)

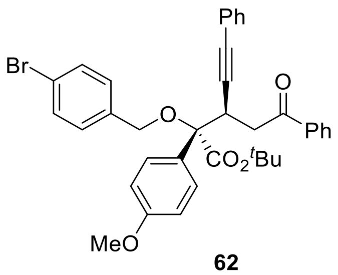

62

Colorless oil; 47.9 mg, 50% yield, >20:1 d.r., >99% *ee,* $[\alpha]_D^{20}$ = -12.98 (c = 0.1, CH_2Cl_2); ^1H NMR (400 MHz, $CDCl_3$) δ 7.85 (d, *J* = 7.3 Hz, 2H), 7.60 (d, *J* = 8.9 Hz, 2H), 7.53 (t, *J* = 7.4 Hz, 1H), 7.47 (d, *J* = 8.4 Hz, 2H), 7.42 (d, *J* = 7.8 Hz, 2H), 7.38 (t, *J* = 6.9 Hz, 2H), 7.25 (d, *J* = 9.0 Hz, 5H), 6.88 (d, *J* = 9.0 Hz, 2H), 5.02 (d, *J* = 11.8 Hz, 1H), 4.88 (d, *J* = 11.8 Hz, 1H), 4.38 (dd, *J* = 10.1, 3.0 Hz, 1H), 3.80 (s, 3H), 3.32 (dd, *J* = 16.7, 10.1 Hz, 1H), 3.11 (dd, *J* = 16.6, 3.0 Hz, 1H), 1.51 (s, 9H). ^{13}C NMR (101 MHz, $CDCl_3$) δ 197.5, 169.6, 159.4, 138.1, 136.9, 133.1, 132.4, 131.4, 130.2, 129.2, 128.6, 128.2, 128.2, 128.0, 127.9, 123.4, 121.2, 113.6, 89.8, 85.6, 82.8, 67.9, 55.3, 39.2, 36.6, 28.1. HRMS (ESI) $[M+Na]^+$ calcd for $C_{37}H_{35}O_5BrNa^+$, 661.1560, found 661.1560. (Chiral IB-3, λ= 254 nm, *n*-hexane/2-propanol= 19/1, Flow rate = 1.0 mL/min), t_R = 14.080 min(major), 33.833 min.

HPLC chromatogram of racemic 62

Condition: n-hexane/2-propanol =19:1

Flow rate =1.0 mL/min

λ= 254 nm

Chiral IB-3

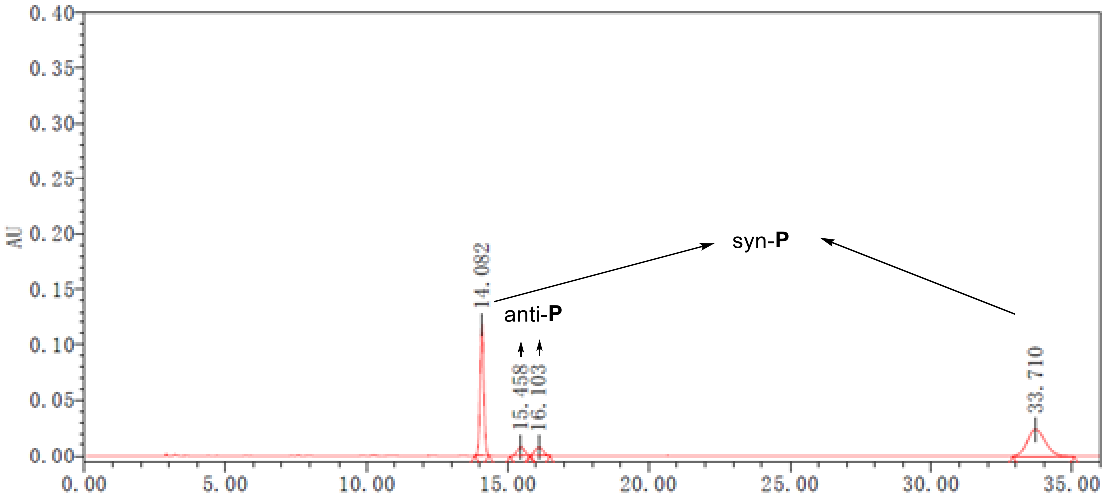

Entry	Retention Time/min	Area	Height	Area(%)
1	14.082	1057188	117486	44.60
2	15.458	132000	7632	5.57
3	16.103	131926	7025	5.57
4	33.710	1049386	23871	44.27

Supplementary Figure 133. Chiral HPLC analysis of racemic **62**

HPLC chromatogram of chiral 62

Condition: n-hexane/2-propanol =19:1

Flow rate =1.0 mL/min

λ= 254 nm

Chiral IB-3

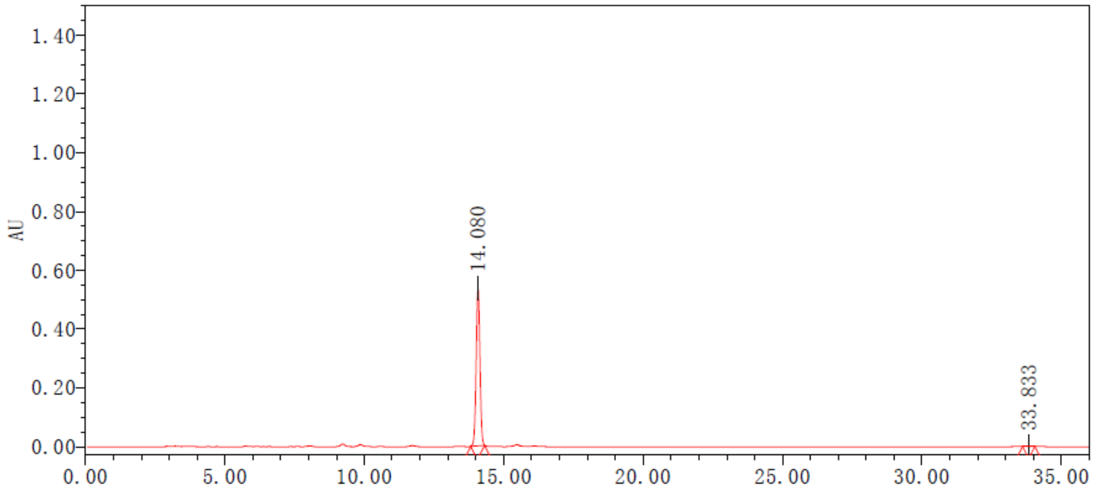

Entry	Retention Time/min	Area	Height	Area(%)
1	14.080	4949393	536766	99.92
2	33.833	4088	257	0.08

Supplementary Figure 134. Chiral HPLC analysis of chiral **62**

Tert-butyl (2S,3R)-2-((4-bromobenzyl)oxy)-2-(4-fluorophenyl)-3-(2-oxo-2-phenylethyl)-5-phenylpent-4-ynoate (63)

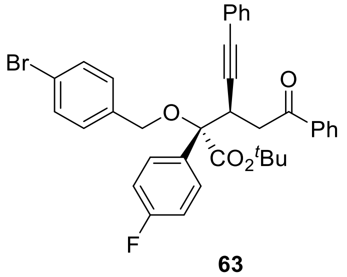

63

Colorless oil; 59.2 mg, 63% yield, >20:1 d.r., 97% *ee*; ^{1}H NMR (500 MHz, $CDCl_3$) δ 7.85 (d, *J* = 7.4 Hz, 2H), 7.67 (dd, *J* = 8.8, 5.3 Hz, 2H), 7.54 (t, *J* = 7.4 Hz, 1H), 7.48 (d, *J* = 8.3 Hz, 2H), 7.42 (t, *J* = 7.7 Hz, 2H), 7.37 (d, *J* = 8.3 Hz, 2H), 7.25 (d, *J* = 6.8 Hz, 5H), 7.05 (t, *J* = 8.7 Hz, 2H), 5.02 (d, *J* = 11.6 Hz, 1H), 4.89 (d, *J* = 11.6 Hz, 1H), 4.40 (dd, *J* = 9.9, 3.0 Hz, 1H), 3.32 (dd, *J* = 16.7, 10.0 Hz, 1H), 3.09 (dd, *J* = 16.7, 2.9 Hz, 1H), 1.51 (s, 9H). ^{13}C NMR (126 MHz, $CDCl_3$) δ 197.3, 169.2, 162.5 (d, *J* = 247.6 Hz), 137.8, 136.8, 134.2, 134.1, 133.2, 131.5, 131.4, 129.2, 128.7 (d, *J* = 8.2 Hz), 128.2, 128.2, 128.0, 122.3 (d, *J* = 230.4 Hz), 115.3, 115.1, 89.4, 85.5, 84.3, 83.2, 68.0, 39.2,

36.5, 28.1. ^{19}F NMR (471 MHz, $CDCl_3$) δ -113.92. HRMS (ESI) $[M+Na]^+$ calcd for $C_{36}H_{32}O_4FBrNa^+$, 649.1560, found 649.1356. (Chiral IE-3, λ= 254 nm, *n*-hexane/2-propanol= 97/3, Flow rate = 1.0 mL/min), t_R = 9.275 min(major), 24.098 min.

HPLC chromatogram of racemic 63

Condition: n-hexane/2-propanol =97:3

Flow rate =1.0 mL/min

λ= 254 nm

Chiral IE-3

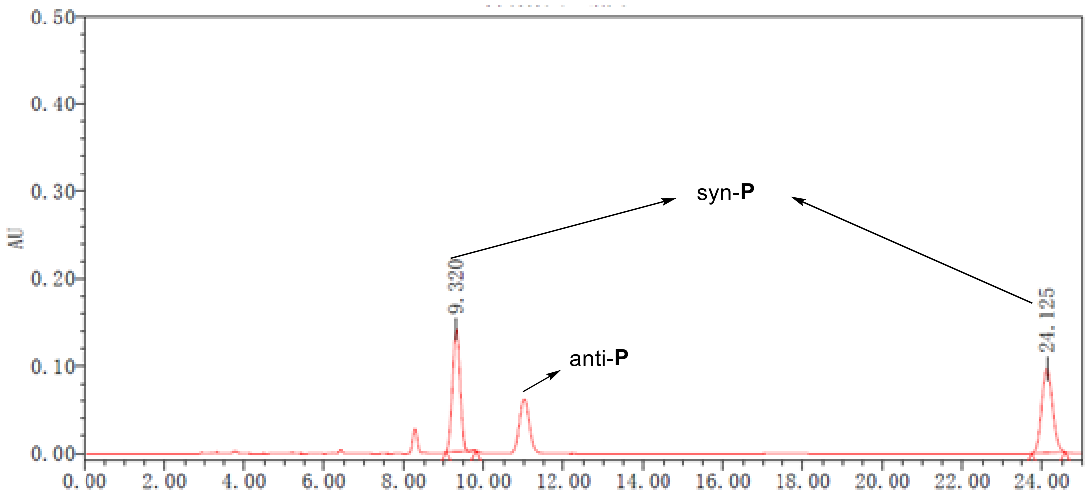

Entry	Retention Time/min	Area	Height	Area(%)
1	9.320	1813011	140041	50.29
2	24.125	1792365	96391	49.71

Supplementary Figure 135. Chiral HPLC analysis of racemic **63**

HPLC chromatogram of chiral 63

Condition: n-hexane/2-propanol =97:3

Flow rate =1.0 mL/min

λ= 254 nm

Chiral IE-3

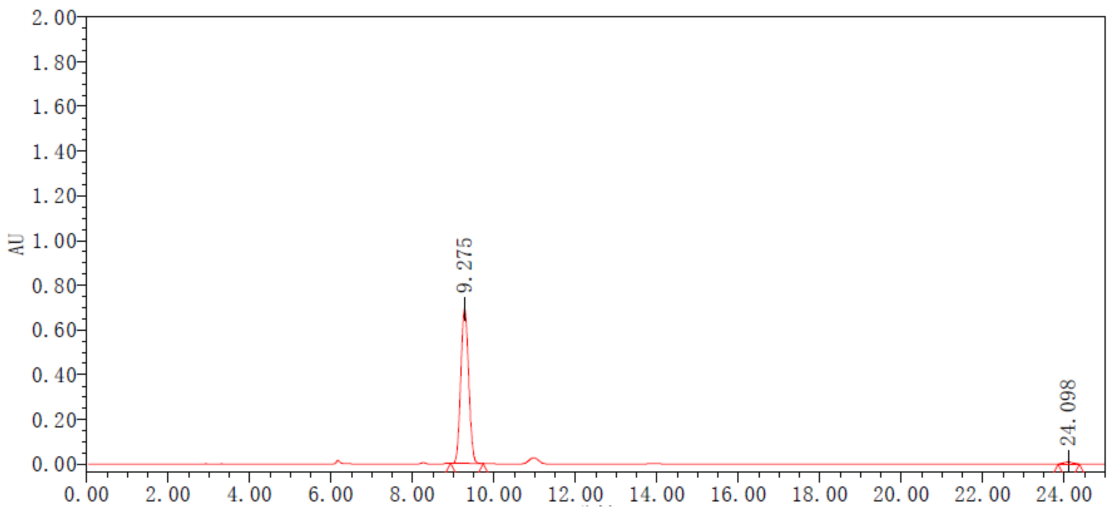

The larger version of HPLC chromatogram of chiral 63

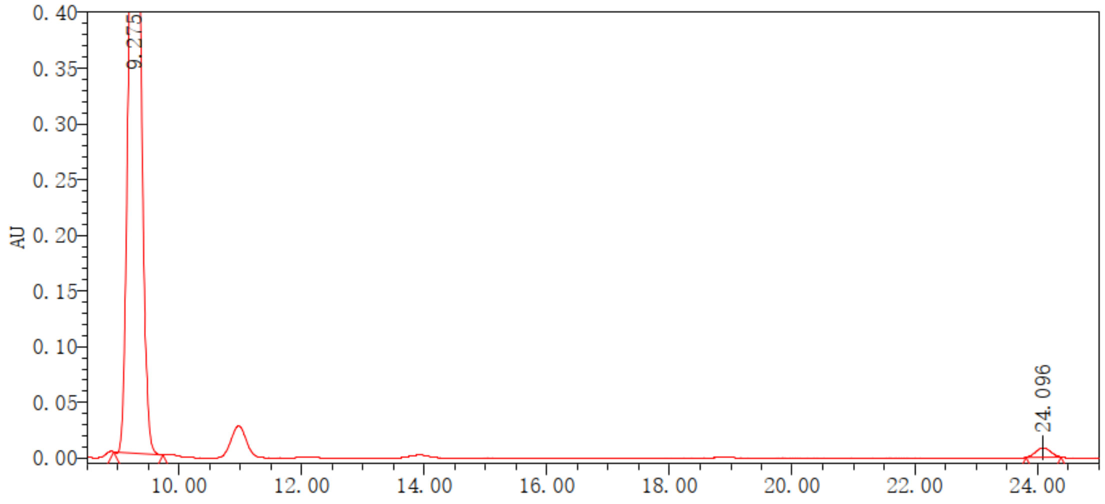

Entry	Retention Time/min	Area	Height	Area(%)
1	9.275	9168282	689687	98.51
2	24.096	131642	8103	1.49

Supplementary Figure 136. Chiral HPLC analysis of chiral **63**

Tert-butyl (2R,3R)-2-((4-bromobenzyl)oxy)-3-(2-oxo-2-phenylethyl)-5-phenyl-2-(thiophen-2-yl)pent-4-ynoate (64)

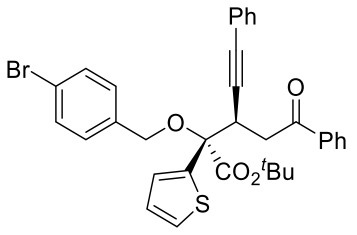

64

Colorless oil; 39.6 mg, 43% yield, >20:1 d.r., 99% *ee*; ^{1}H NMR (400 MHz, $CDCl_3$) δ 7.91 – 7.85 (m, 2H), 7.54 (t, *J* = 7.4 Hz, 1H), 7.43 (dd, *J* = 15.7, 7.9 Hz, 4H), 7.35 (d, *J* = 8.4 Hz, 2H), 7.30 (dd, *J* = 5.1, 1.1 Hz, 1H), 7.28 – 7.22 (m, 6H), 6.99 (dd, *J* = 5.1, 3.7 Hz, 1H), 4.94 (d, *J* = 11.9 Hz, 1H), 4.85 (d, *J* = 11.9 Hz, 1H), 4.29 (dd, *J* = 8.4, 4.7 Hz, 1H), 3.34 (dd, *J* = 6.5, 4.5 Hz, 2H), 1.56 (s, 9H). ^{13}C NMR (101 MHz, $CDCl_3$) δ 197.2, 168.4, 141.7, 137.8, 136.9, 133.1, 131.5, 131.3, 129.0, 128.6, 128.2, 128.2, 128.0, 126.6, 126.4, 123.2, 121.1, 112.7, 93.2, 88.8, 85.0, 83.5, 81.1, 77.3, 77.0, 76.7, 69.4, 67.9, 51.3, 39.1, 39.2, 38.3, 28.0, 25.0. HRMS (ESI) $[M+Na]^+$ calcd for $C_{34}H_{31}O_4SBrNa^+$, 637.1019, found 637.1019. (Chiral ID-3, λ= 254 nm, *n*-hexane/2-propanol= 49/1, Flow rate = 1.0 mL/min), t_R = 9.650 min(major), 22.555 min.

HPLC chromatogram of racemic 64

Condition: n-hexane/2-propanol =49:1

Flow rate =1.0 mL/min

λ= 254 nm

Chiral ID-3

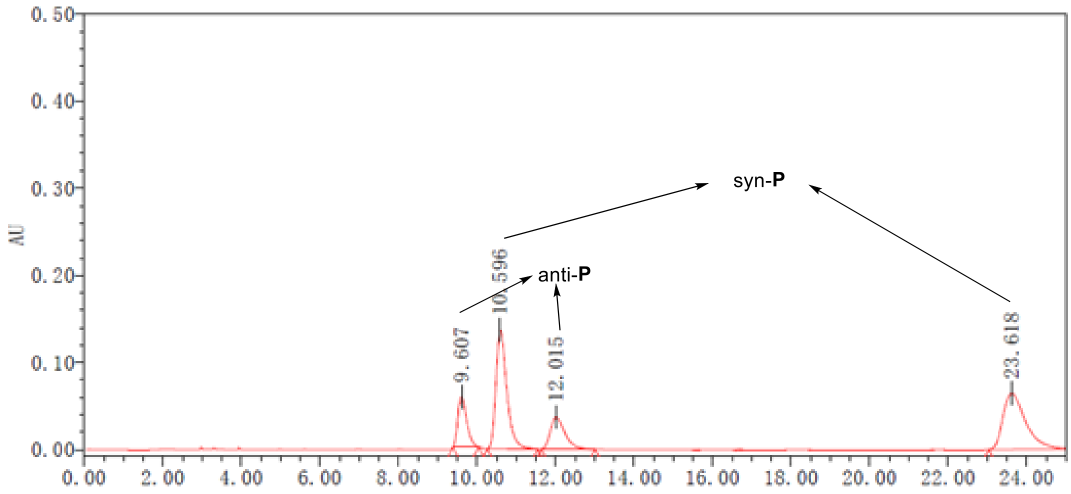

Entry	Retention Time/min	Area	Height	Area(%)
1	9.607	909105	57501	12.93
2	10.596	2610966	135845	37.14
3	12.015	926181	36593	13.17
4	23.618	2584403	64137	36.76

Supplementary Figure 137. Chiral HPLC analysis of racemic **64**

HPLC chromatogram of chiral 64

Condition: n-hexane/2-propanol =49:1

Flow rate =1.0 mL/min

λ= 254 nm

Chiral ID-3

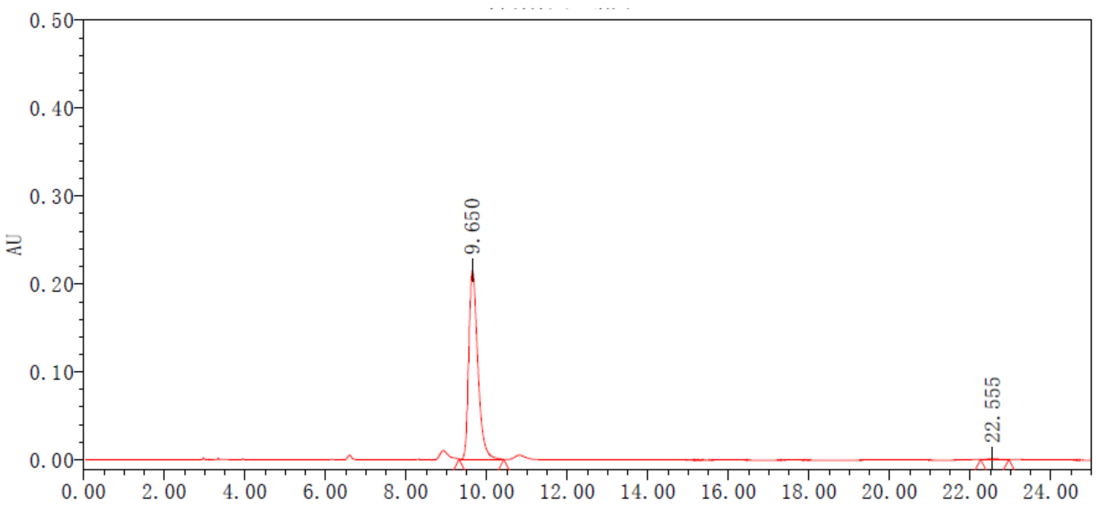

The larger version of HPLC chromatogram of chiral 64

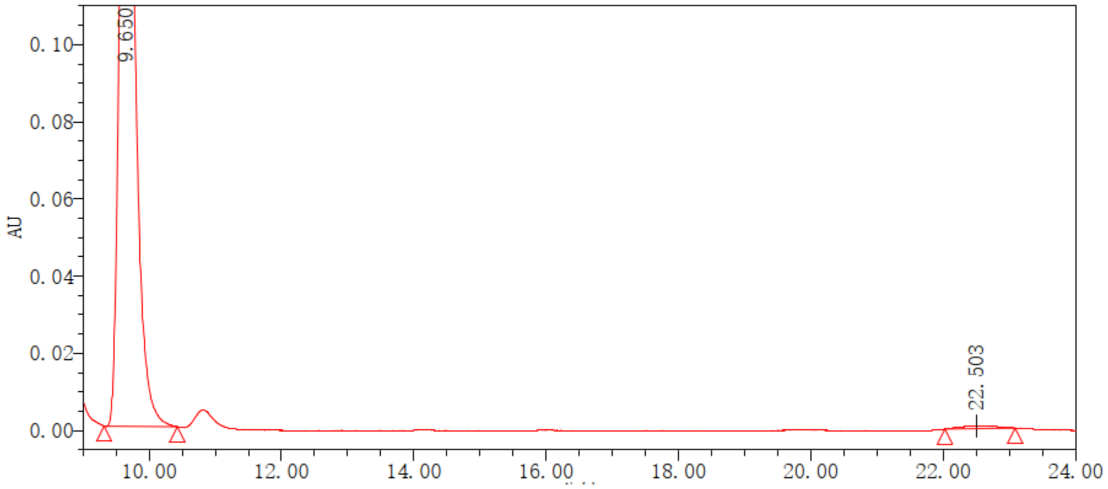

Entry	Retention Time/min	Area	Height	Area(%)
1	9.650	3331433	214864	99.51
2	22.503	9234	350	0.49

Supplementary Figure 138. Chiral HPLC analysis of chiral **64**

Tert-butyl (2S,3R)-2-((4-bromobenzyl)oxy)-3-(2-oxo-2-phenylethyl)-5-phenyl-2-(thiophen-3-yl)pent-4-ynoate (65)

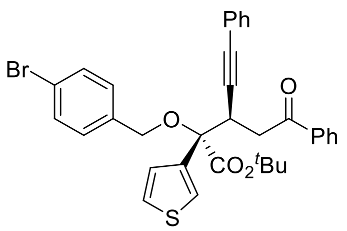

65

Colorless oil; 47.9 mg, 52% yield, >20:1 d.r., 92% *ee*; ^{1}H NMR (500 MHz, $CDCl_3$) δ 7.87 (d, *J* = 7.5 Hz, 2H), 7.58 – 7.51 (m, 2H), 7.48 – 7.39 (m, 4H), 7.34 (d, *J* = 7.7 Hz, 2H), 7.26 (d, *J* = 15.8 Hz, 4H), 7.24 (d, *J* = 4.3 Hz, 4H), 4.93 (d, *J* = 11.8 Hz, 1H), 4.81 (d, *J* = 11.8 Hz, 1H), 4.29 (d, *J* = 8.5 Hz, 1H), 3.34 (dd, *J* = 16.6, 9.6 Hz, 1H), 3.21 (dd, *J* = 33.2, 12.4 Hz, 1H), 1.54 (s, 9H). ^{13}C NMR (126 MHz, $CDCl_3$) δ 197.4, 169.1, 139.5, 138.0, 136.9, 133.1, 131.4, 131.4, 129.1, 128.6, 128.2, 127.9, 126.8, 125.6, 124.2, 123.3, 121.2, 89.2, 84.8, 84.2, 83.04, 67.8, 39.1, 37.6, 28.1, 28.1. Peak overlapping was observed. HRMS (ESI) $[M+Na]^+$ calcd for $C_{34}H_{31}O_4SBrNa^+$, 637.1019, found 637.1017. (Chiral IE-3, λ= 254 nm, *n*-hexane/2-propanol= 49/1, Flow rate = 1.0 mL/min), t_R = 16.568 min(major), 36.556 min.

HPLC chromatogram of racemic 65

Condition: n-hexane/2-propanol = 49:1

Flow rate =1.0 mL/min

λ= 254 nm

Chiral IE-3

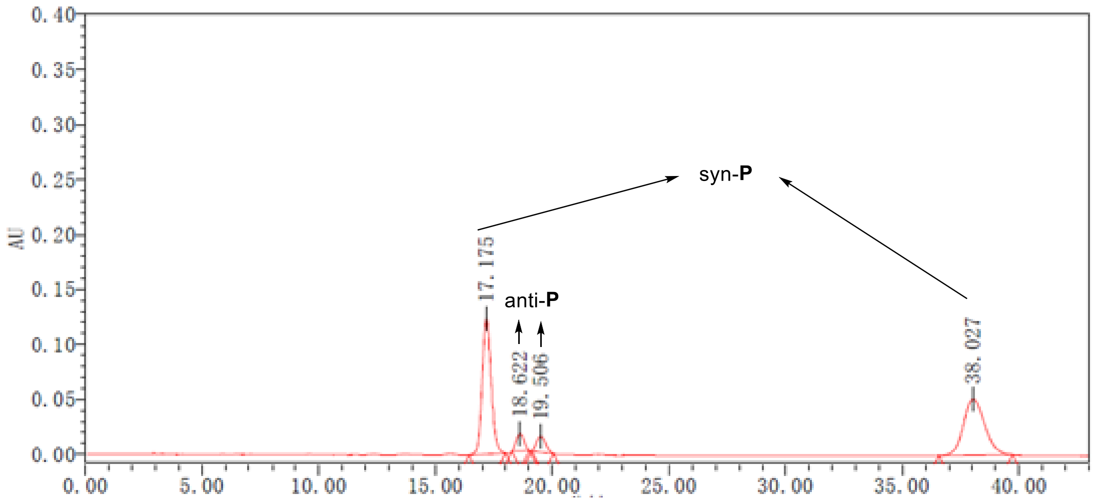

Entry	Retention Time/min	Area	Height	Area(%)
1	17.175	3318551	123719	45.02
2	18.622	379526	16077	5.15
3	19.506	385317	14468	5.23
4	38.027	3287821	51212	44.60

Supplementary Figure 139. Chiral HPLC analysis of racemic **65**

HPLC chromatogram of chiral 65

Condition: n-hexane/2-propanol =49:1

Flow rate =1.0 mL/min

λ= 254 nm

Chiral IE-3

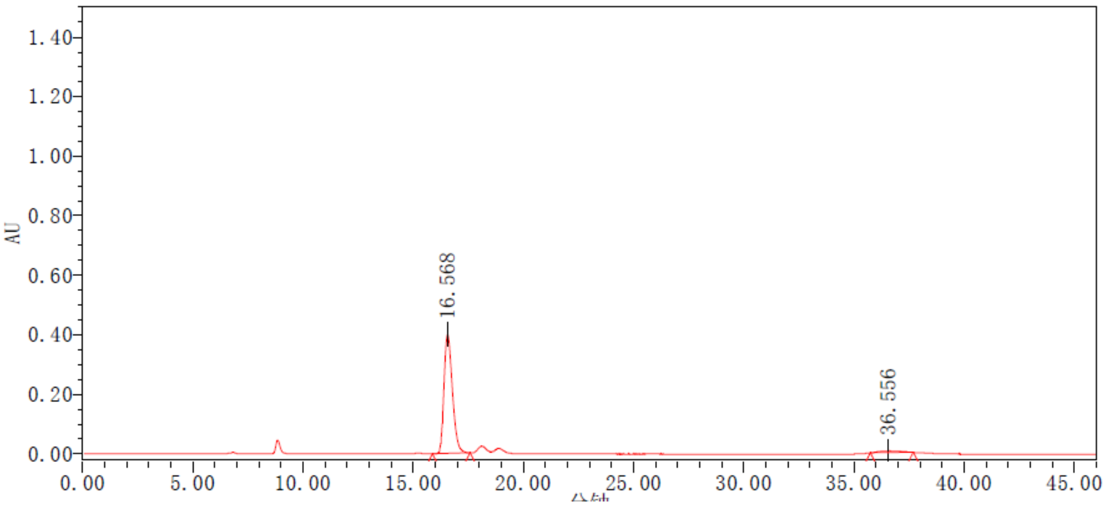

Entry	Retention Time/min	Area	Height	Area(%)
1	16.568	10475819	399381	96.15
2	36.556	419706	6221	3.85

Supplementary Figure 140. Chiral HPLC analysis of chiral **65**

Tert-butyl (2R,3R)-2-benzyl-2-((4-bromobenzyl)oxy)-3-(2-oxo-2-phenylethyl)-5-phenylpent-4-ynoate (66)

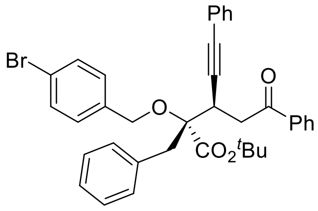

66

Colorless oil; 39.2 mg, 42% yield, >20:1 d.r., 92% *ee*; ^{1}H NMR (400 MHz, $CDCl_3$) δ 7.96 – 7.89 (m, 2H), 7.57 (t, *J* = 7.4 Hz, 1H), 7.47 – 7.42 (m, 5H), 7.36 – 7.32 (m, 4H), 7.30 – 7.28 (m, 5H), 7.23 (d, *J* = 8.4 Hz, 2H), 4.98 (d, *J* = 11.4 Hz, 1H), 4.75 (d, *J* = 11.4 Hz, 1H), 3.96 (dd, *J* = 8.8, 3.9 Hz, 1H), 3.67 (dd, *J* = 17.0, 3.9 Hz, 1H), 3.39 (dd, *J* = 16.9, 8.0 Hz, 2H), 3.36 – 3.32 (m, 1H), 1.50 (s, 9H). ^{13}C NMR (101 MHz, $CDCl_3$) δ 197.6, 169.9, 137.4, 137.0, 135.7, 133.1, 131.5, 131.3, 130.7, 129.1, 128.6, 128.2, 128.2, 128.1, 128.0, 126.9, 123.2, 121.2, 91.8, 89.2, 88.2, 84.6, 83.1, 82.5, 66.8, 39.7, 39.1, 36.1, 34.2, 28.2. HRMS (ESI) $[M+Na]^+$ calcd for $C_{37}H_{35}O_4BrNa^+$, 645.1611,

found 645.1614. (Chiral IA-3, λ= 254 nm, *n*-hexane/2-propanol= 49/1, Flow rate = 1.0 mL/min), t_R = 10.805 min(major), 14.844 min.

HPLC chromatogram of racemic 66

Condition: n-hexane/2-propanol =49:1

Flow rate =1.0 mL/min

λ= 254 nm

Chiral IA-3

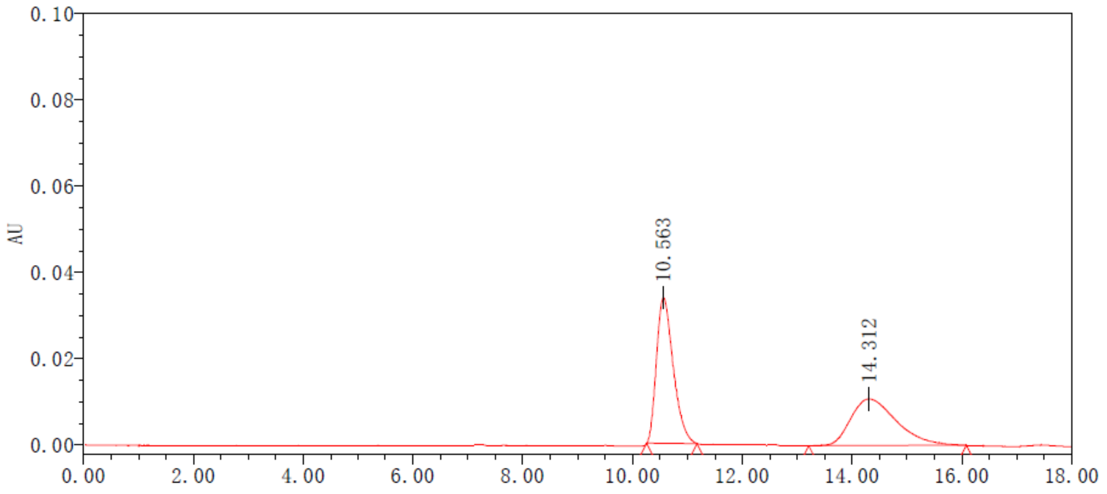

Entry	Retention Time/min	Area	Height	Area(%)
1	10.563	668100	33750	50.42
2	14.312	667613	10779	49.58

Supplementary Figure 141. Chiral HPLC analysis of racemic **66**

HPLC chromatogram of chiral 66

Condition: n-hexane/2-propanol =49:1

Flow rate =1.0 mL/min

λ= 254 nm

Chiral IA-3

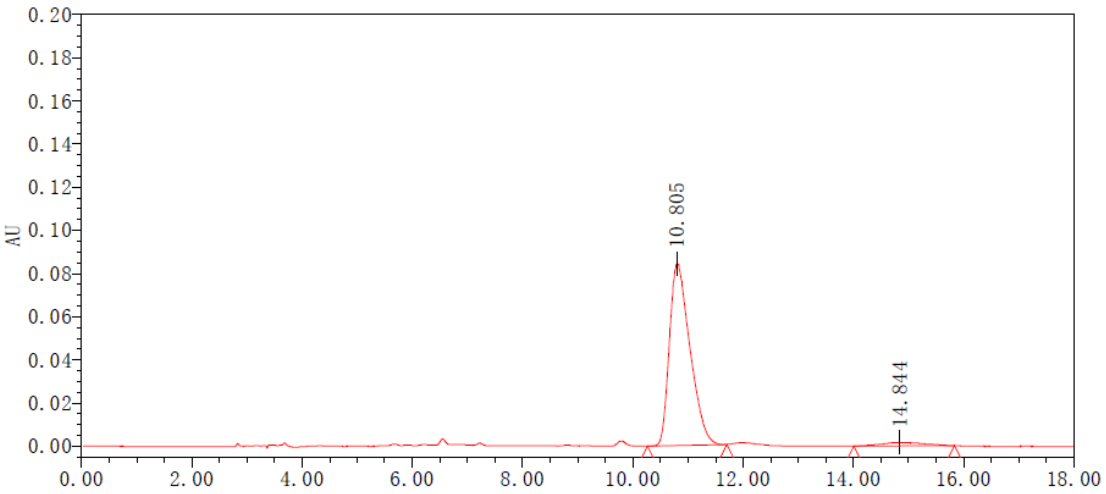

Entry	Retention Time/min	Area	Height	Area(%)
1	10.805	2191029	84327	96.20
2	14.844	86562	1602	3.80

Supplementary Figure 142. Chiral HPLC analysis of chiral **66**

Tert-butyl (2R,3R)-2-((4-bromobenzyl)oxy)-2-methyl-3-(2-oxo-2-phenylethyl)-5-phenylpent-4-ynoate (67)

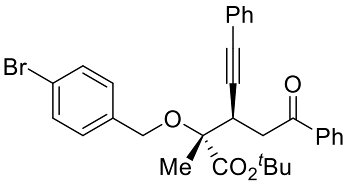

67

Colorless oil; 50.0 mg, 61% yield, >20:1 d.r., 98% *ee*; ^{1}H NMR (400 MHz, $CDCl_3$) δ 7.98 – 7.93 (m, 2H), 7.58 – 7.52 (m, 1H), 7.43 (t, *J* = 6.7 Hz, 2H), 7.40 – 7.36 (m, 2H), 7.31 – 7.27 (m, 2H), 7.26 – 7.20 (m, 5H), 4.58 – 4.44 (m, 2H), 3.87 (dd, *J* = 8.8, 4.2 Hz, 1H), 3.61 (dd, *J* = 16.7, 4.2 Hz, 1H), 3.28 (dd, *J* = 16.7, 8.8 Hz, 1H), 1.67 (s, 3H), 1.52 (s, 9H). ^{13}C NMR (101 MHz, $CDCl_3$) δ 197.8, 170.9, 137.5, 137.1, 133.0, 131.6, 131.3, 129.1, 128.5, 128.3, 128.1, 127.9, 123.4, 121.2, 88.7, 83.6, 82.1, 81.9, 66.4, 39.2, 37.2, 29.7, 28.1, 20.8. HRMS (ESI) $[M+Na]^+$ calcd for $C_{31}H_{31}O_4BrNa^+$, 569.1299, found 569.1298. (Chiral IA-3, λ= 254 nm, *n*-hexane/2-propanol= 99/1, Flow rate = 1.0

mL/min), t_R = 11.569 min(major), 13.093 min.

HPLC chromatogram of racemic 67

Condition: n-hexane/2-propanol =99:1

Flow rate =1.0 mL/min

λ= 254 nm

Chiral IA-3

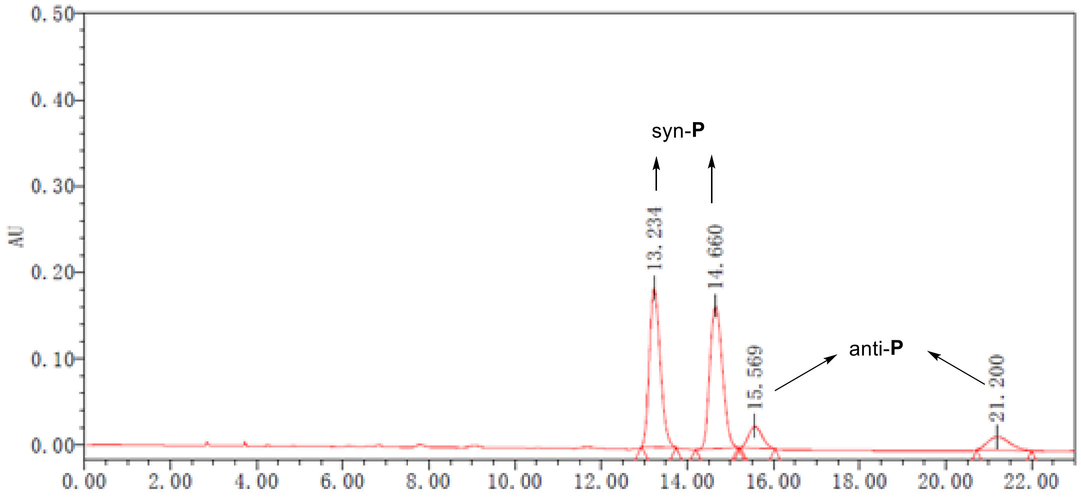

Entry	Retention Time/min	Area	Height	Area(%)
1	13.234	13.234	184637	43.12
2	14.660	14.660	165217	42.71
3	15.569	15.569	25358	7.17
4	21.200	21.200	15303	7.00

Supplementary Figure 143. Chiral HPLC analysis of racemic **67**

HPLC chromatogram of chiral 67

Condition: n-hexane/2-propanol =99:1

Flow rate =1.0 mL/min

λ= 254 nm

Chiral IA-3

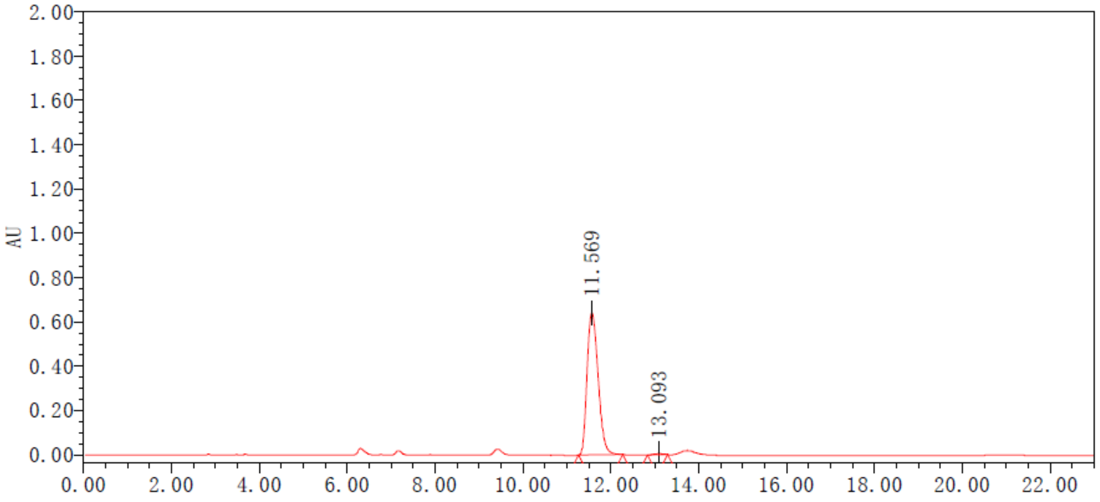

The larger version of HPLC chromatogram of chiral 67

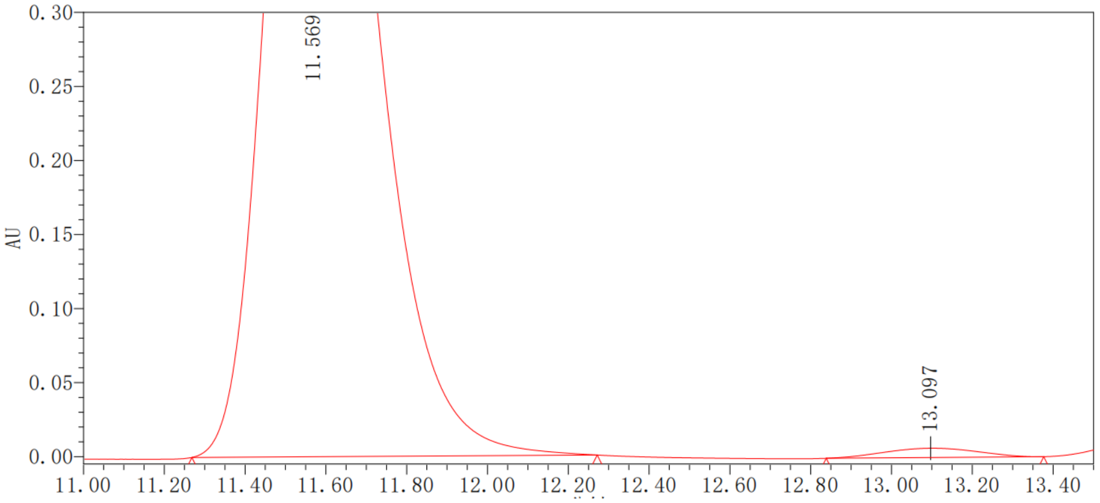

Entry	Retention Time/min	Area	Height	Area(%)
1	11.569	11115192	642444	99.27
2	13.097	82114	5594	0.73

Supplementary Figure 144. Chiral HPLC analysis of chiral **67**

Tert-butyl (2S,3R)-2-((4-bromobenzyl)oxy)-3-(2-(4-fluorophenyl)-2-oxoethyl)-2,5-diphenylpent-4-ynoate (68)

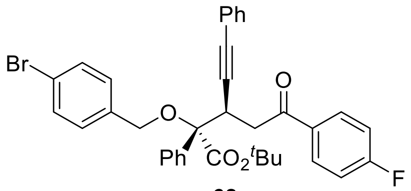

68

Colorless oil; 60.1 mg, 64% yield, >20:1 d.r., 99% *ee*, $[\alpha]_D^{20}$ = -14.39 (c = 0.1, CH_2Cl_2); ^1H NMR (400 MHz, $CDCl_3$) δ 7.89 – 7.83 (m, 2H), 7.70 – 7.66 (m, 2H), 7.50 – 7.46 (m, 2H), 7.37 (dd, *J* = 7.8, 5.3 Hz, 4H), 7.32 (d, *J* = 7.1 Hz, 1H), 7.25 (d, *J* = 4.0 Hz, 1H), 7.24 (s, 4H), 7.10 – 7.04 (m, 2H), 5.04 (d, *J* = 11.7 Hz, 1H), 4.89 (d, *J* = 11.7 Hz, 1H), 4.38 (dd, *J* = 10.1, 3.2 Hz, 1H), 3.26 (dd, *J* = 16.5, 10.1 Hz, 1H), 3.13 – 3.00 (m, 1H), 1.51 (s, 9H). ^{13}C NMR (101 MHz, $CDCl_3$) δ 195.9, 169.4, 165.8 (d, *J* = 254.8 Hz), 138.2 (d, *J* = 22.7 Hz), 133.3 (d, *J* = 2.8 Hz), 131.4 (d, *J* = 4.5 Hz), 130.9, 130.8, 129.2, 128.3, 128.2, 128.0, 126.7, 123.3, 121.3, 115.8, 115.5, 89.5, 85.9, 84.2, 83.0, 68.0, 39.1, 36.7, 28.1. Peak overlapping was observed. ^{19}F NMR (376 MHz, $CDCl_3$) δ -105.15. HRMS (ESI) $[M+Na]^+$ calcd for $C_{36}H_{32}O_4FBrNa^+$, 649.1360, found 649.1356. (Chiral IE-3, λ= 254 nm, *n*-hexane/2-propanol= 19/1, Flow rate = 1.0 mL/min), t_R = 10.750 min(major), 16.307 min.

HPLC chromatogram of racemic 68

Condition: n-hexane/2-propanol =19:1

Flow rate =1.0 mL/min

λ= 254 nm

Chiral IE-3

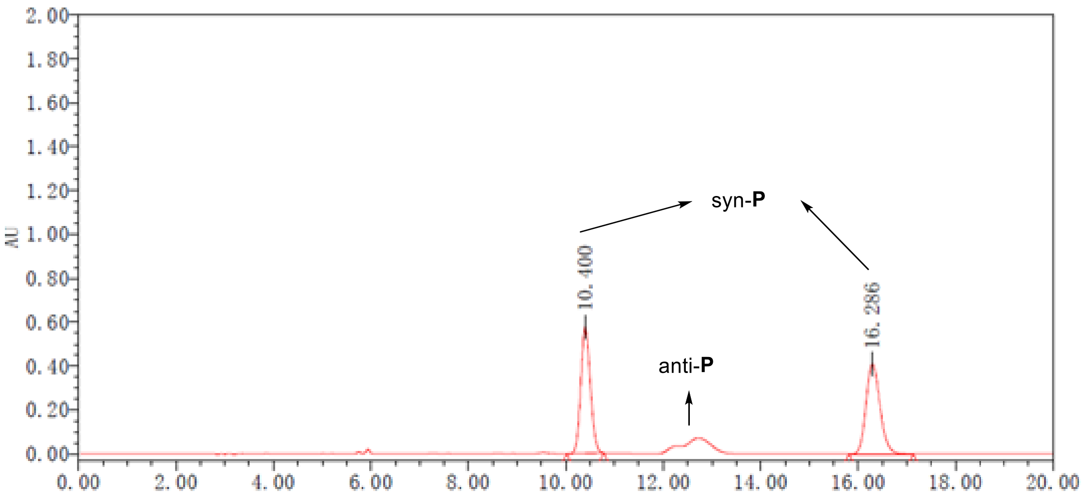

Entry	Retention Time/min	Area	Height	Area(%)
1	10.400	8098660	577025	49.91
2	16.286	8129458	408821	50.09

Supplementary Figure 145. Chiral HPLC analysis of racemic **68**

HPLC chromatogram of chiral 68

Condition: n-hexane/2-propanol =19:1

Flow rate =1.0 mL/min

λ= 254 nm

Chiral IE-3

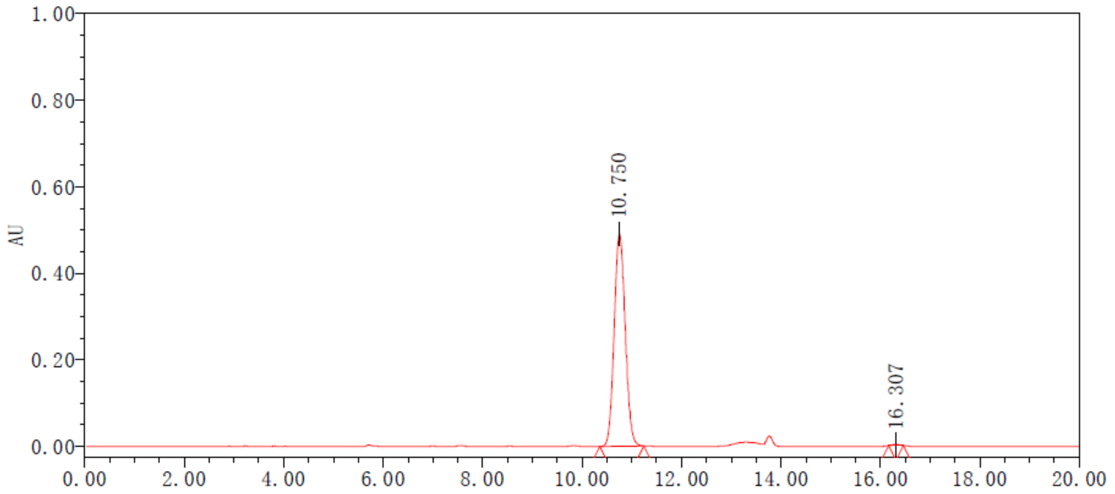

The larger version of HPLC chromatogram of chiral 68

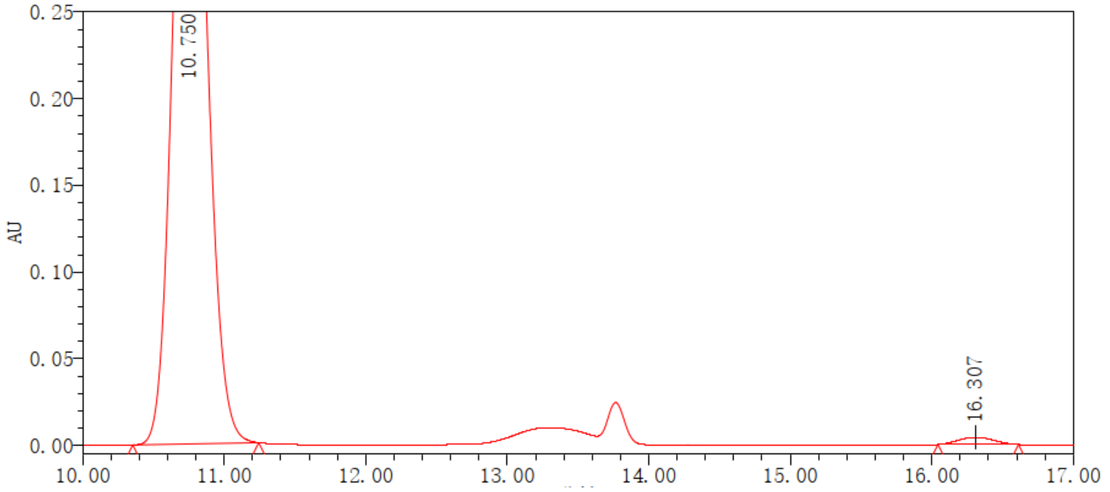

Entry	Retention Time/min	Area	Height	Area(%)
1	10.750	7559970	489917	99.51
2	16.307	25774	2317	0.49

Supplementary Figure 146. Chiral HPLC analysis of chiral **68**

Tert-butyl (2S,3R)-2-((4-bromobenzyl)oxy)-3-(2-(4-chlorophenyl)-2-oxoethyl)-2,5-diphenylpent-4-ynoate (69)

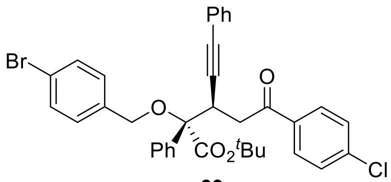

69

Colorless oil; 65.5 mg, 68% yield, >20:1 d.r., >99% *ee*; ^{1}H NMR (400 MHz, $CDCl_3$) δ 7.78 – 7.74 (m, 2H), 7.70 – 7.65 (m, 2H), 7.48 (d, *J* = 8.4 Hz, 2H), 7.38 (d, *J* = 1.4 Hz, 3H), 7.36 (d, *J* = 1.1 Hz, 2H), 7.33 (dd, *J* = 6.5, 1.8 Hz, 1H), 7.25 (d, *J* = 5.6 Hz, 6H), 5.03 (d, *J* = 11.7 Hz, 1H), 4.88 (d, *J* = 11.7 Hz, 1H), 4.37 (dd, *J* = 10.0, 3.3 Hz, 1H), 3.25 (dd, *J* = 16.5, 10.0 Hz, 1H), 3.08 (dd, *J* = 16.5, 3.2 Hz, 1H), 1.51 (s, 9H). ^{13}C NMR (101 MHz, $CDCl_3$) δ 197.0, 169.4, 143.9, 138.4, 138.1, 134.5, 131.4, 129.2, 128.3, 128.3, 128.2, 128.2, 127.9, 126.7, 123.4, 121.2, 89.8, 85.9, 84.1, 82.9, 68.0, 39.1, 36.6, 28.1, 21.7. Peak overlapping was observed. HRMS (ESI) $[M+Na]^+$ calcd for $C_{36}H_{32}O_4ClBrNa^+$, 665.1065, found 665.1069. (Chiral IE-3, λ= 254 nm, *n*-hexane/2-propanol= 19/1, Flow rate = 1.0 mL/min), t_R = 7.401 min(major), 10.092 min.

HPLC chromatogram of racemic 69

Condition: n-hexane/2-propanol =19:1

Flow rate =1.0 mL/min

λ= 254 nm

Chiral IE-3

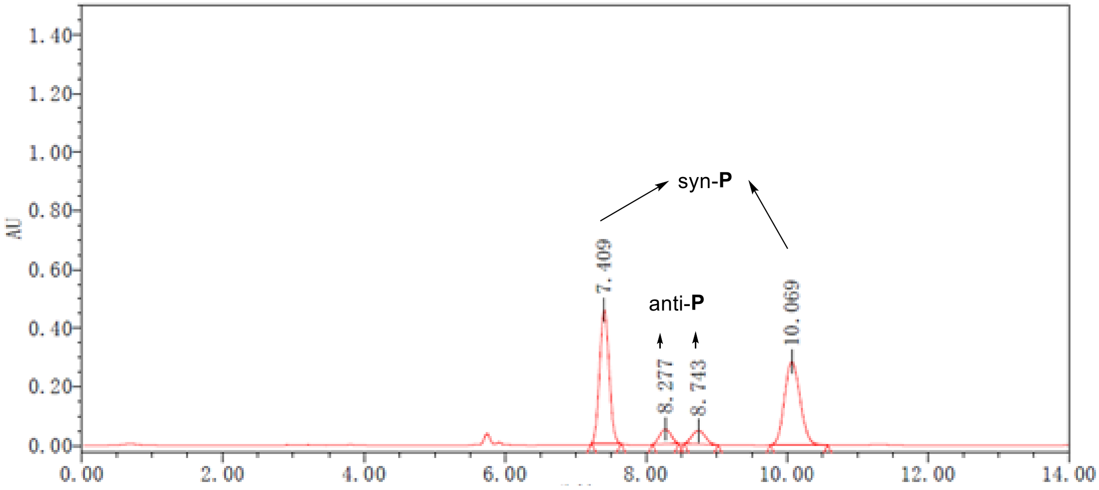

Entry	Retention Time/min	Area	Height	Area(%)
1	7.409	4374540	458002	44.26
2	8.277	559177	50697	5.66
3	8.743	613385	46416	6.21
4	10.069	4337024	283557	43.88

Supplementary Figure 147. Chiral HPLC analysis of racemic **69**

HPLC chromatogram of chiral 69

Condition: n-hexane/2-propanol =19:1

Flow rate =1.0 mL/min

λ= 254 nm

Chiral IE-3

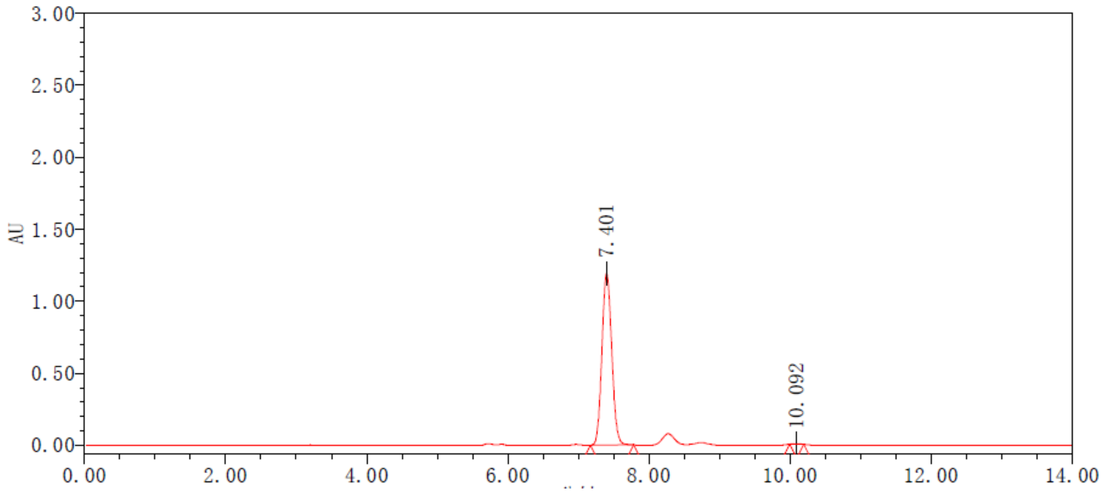

The larger version of HPLC chromatogram of chiral 69

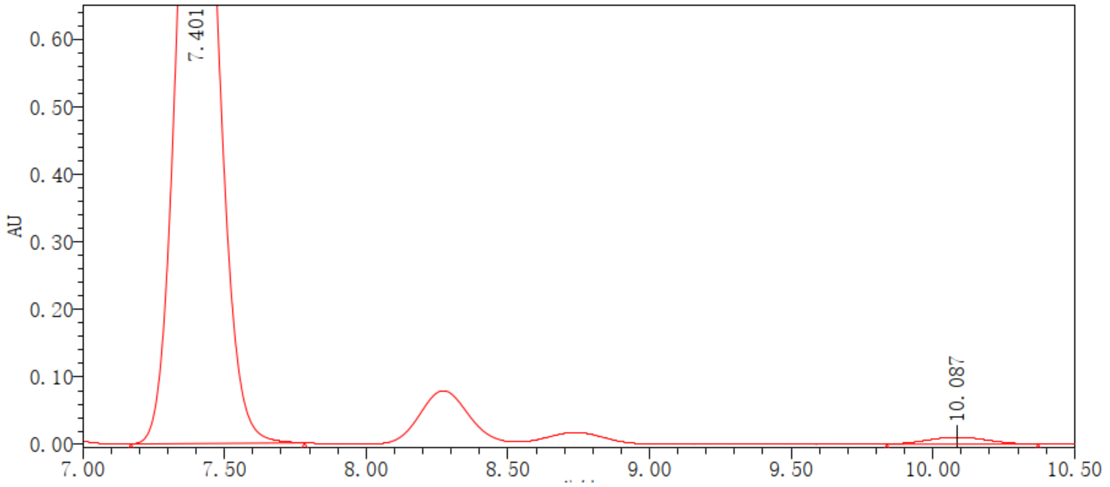

Entry	Retention Time/min	Area	Height	Area(%)
1	7.401	11401645	1193220	99.73
2	10.092	30567	3945	0.27

Supplementary Figure 148. Chiral HPLC analysis of chiral **69**

Tert-butyl (2S,3R)-2-((4-bromobenzyl)oxy)-3-(2-oxo-2-(p-tolyl)ethyl)-2,5-diphenylpent-4-ynoate (70)

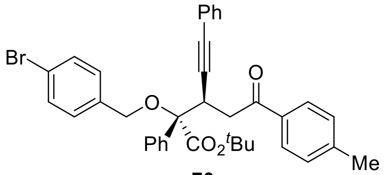

70

Colorless oil; 87.1 mg, 70% yield, >20:1 d.r., 99% *ee*; ^{1}H NMR (400 MHz, $CDCl_3$) δ 7.74 (d, *J* = 8.2 Hz, 2H), 7.71 – 7.66 (m, 2H), 7.48 (d, *J* = 8.4 Hz, 2H), 7.40 – 7.36 (m, 3H), 7.35 – 7.29 (m, 2H), 7.24 (d, *J* = 8.4 Hz, 5H), 7.20 (d, *J* = 8.0 Hz, 2H), 5.05 (d, *J* = 11.7 Hz, 1H), 4.91 (d, *J* = 11.7 Hz, 1H), 4.41 (dd, *J* = 10.2, 3.0 Hz, 1H), 3.30 (dd, *J* = 16.6, 10.2 Hz, 1H), 3.06 (dd, *J* = 16.5, 3.0 Hz, 1H), 2.38 (s, 3H), 1.51 (s, 9H). ^{13}C NMR (101 MHz, $CDCl_3$) δ 197.0, 169.4, 143.9, 138.4, 138.1, 134.5, 131.4, 129.2, 128.3, 128.3, 128.2, 128.2, 127.9, 126.7, 123.4, 121.2, 89.8, 85.9, 84.1, 82.9, 68.0, 39.1, 36.6, 28.1, 21.7. Peak overlapping was observed. HRMS (ESI) $[M+Na]^+$ calcd for $C_{37}H_{35}O_4BrNa^+$, 645.1611, found 645.1610. (Chiral IE-3, λ= 254 nm, *n*-hexane/2-propanol= 19/1, Flow rate = 1.0 mL/min), t_R = 7.581 min(major), 11.439 min.

HPLC chromatogram of racemic 70

Condition: n-hexane/2-propanol =19:1

Flow rate =1.0 mL/min

λ= 254 nm

Chiral IE-3

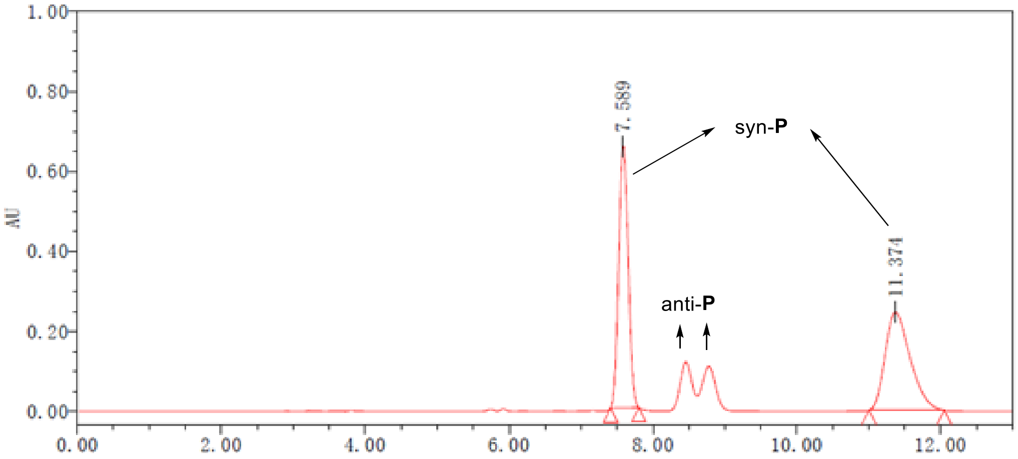

Entry	Retention Time/min	Area	Height	Area(%)
1	7.589	5946947	654224	50.04
2	11.374	5936607	245522	49.96

Supplementary Figure 149. Chiral HPLC analysis of racemic **70**

HPLC chromatogram of chiral 70

Condition: n-hexane/2-propanol =19:1

Flow rate =1.0 mL/min

λ= 254 nm

Chiral IE-3

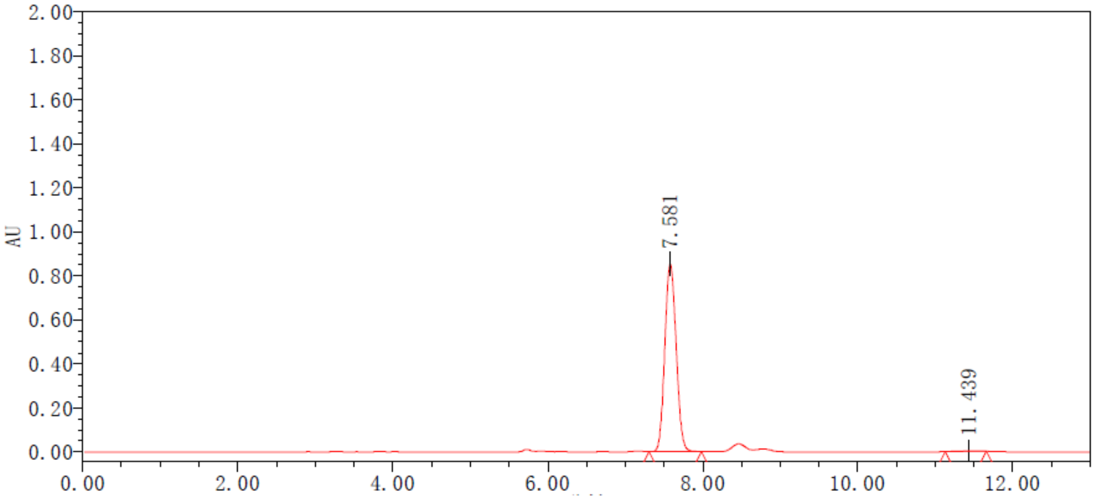

Entry	Retention Time/min	Area	Height	Area(%)
1	7.581	8606054	852567	99.51
2	11.439	35308	1514	0.49

Supplementary Figure 150. Chiral HPLC analysis of chiral **70**

Tert-butyl (2S,3R)-2-((4-bromobenzyl)oxy)-5-(3-chloro-2-fluorophenyl)-3-(2-oxo-2-phenylethyl)-2-phenylpent-4-ynoate (71)

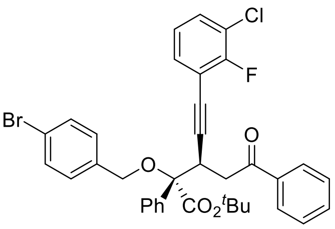

71

Colorless oil; 72.2 mg, 73% yield, >20:1 d.r., 99% *ee*, $[\alpha]_D^{20}$ = -12.68 (c = 0.1, CH_2Cl_2); ^1H NMR (500 MHz, $CDCl_3$) δ 7.86 – 7.81 (m, 2H), 7.68 (d, *J* = 7.5 Hz, 2H), 7.53 (t, *J*

= 7.4 Hz, 1H), 7.49 (d, *J* = 8.3 Hz, 2H), 7.43 – 7.38 (m, 5H), 7.36 (d, *J* = 7.8 Hz, 1H), 7.32 (d, *J* = 7.2 Hz, 1H), 7.30 – 7.26 (m, 1H), 7.16 – 7.12 (m, 1H), 6.95 (t, *J* = 7.9 Hz, 1H), 5.07 (d, *J* = 11.8 Hz, 1H), 4.92 (d, *J* = 11.8 Hz, 1H), 4.46 (dd, *J* = 10.2, 2.8 Hz, 1H), 3.35 (dd, *J* = 16.9, 10.2 Hz, 1H), 3.10 (dd, *J* = 16.9, 2.8 Hz, 1H), 1.50 (s, 9H). ^{13}C NMR (126 MHz, $CDCl_3$) δ 197.1, 169.3, 158.5 (d, *J* = 253.0 Hz), 138.1 (d, *J* = 26.7 Hz), 136.7, 133.2, 131.7, 131.4, 130.3, 129.1, 128.6, 128.4, 128.4, 128.2, 126.6, 124.2, 124.2, 121.4, 121.2 (d, *J* = 3.8 Hz), 113.4, 96.5, 96.4, 85.8, 83.2, 68.1, 39.1, 36.6, 28.0. ^{19}F NMR (471 MHz, $CDCl_3$) δ -112.4. HRMS (ESI) $[M+Na]^+$ calcd for $C_{36}H_{31}O_4FClBrNa^+$, 683.0970, found 683.0967. (Chiral IA-3, λ= 254 nm, *n*-hexane/2-propanol/EtOH= 98/1/1, Flow rate = 1.0 mL/min), t_R = 9.178 min(major), 10.953 min.

HPLC chromatogram of racemic 71

Condition: n-hexane/2-propanol/EtOH = 98:1:1

Flow rate =1.0 mL/min

λ= 254 nm

Chiral IA-3

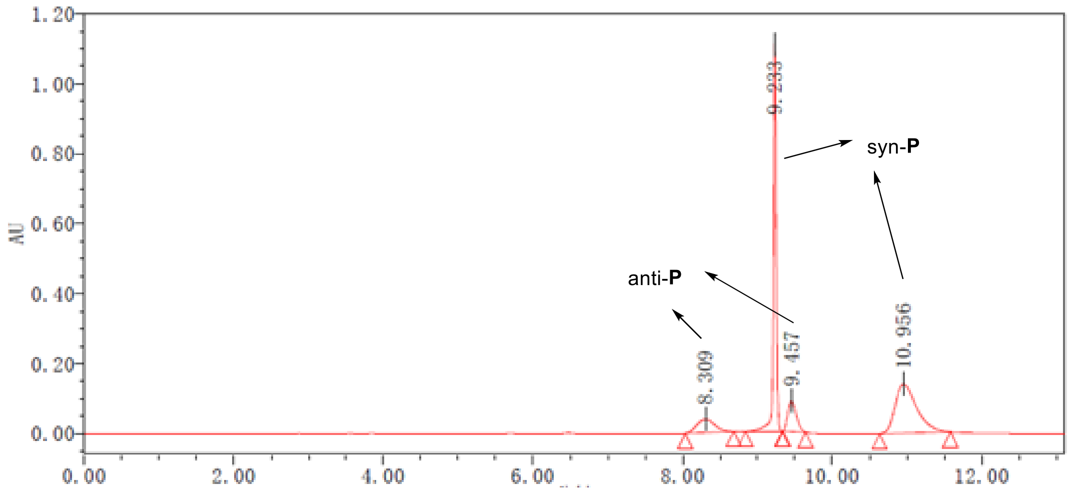

Entry	Retention Time/min	Area	Height	Area(%)
1	8.309	658634	38234	9.44
2	9.233	2810523	1109878	40.26
3	9.457	688344	88087	9.86
4	10.956	2822567	139899	40.44

Supplementary Figure 151. Chiral HPLC analysis of racemic **71**

HPLC chromatogram of chiral 71

Condition: n-hexane/2-propanol/EtOH = 98:1:1

Flow rate =1.0 mL/min

λ= 254 nm

Chiral IA-3

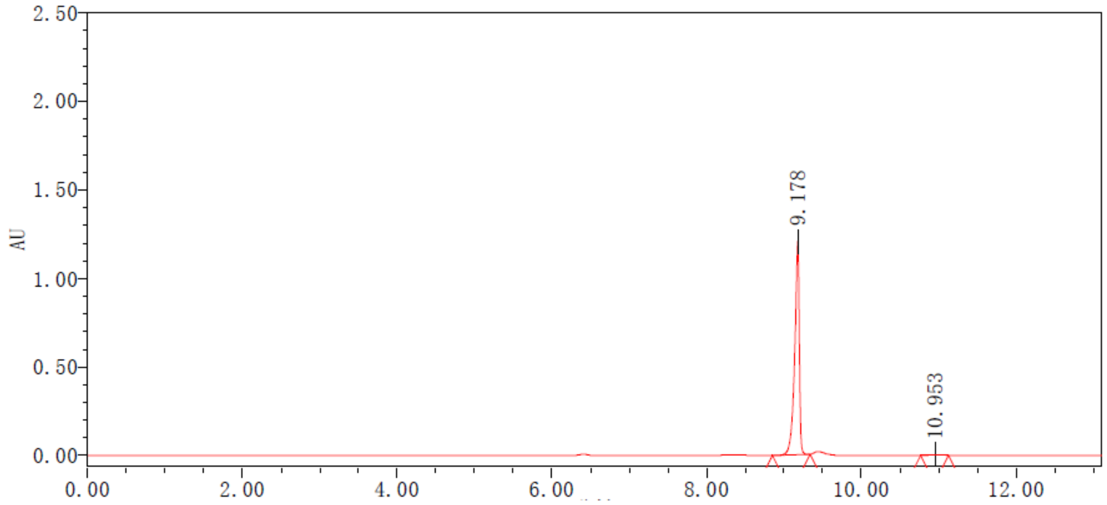

The larger version of HPLC chromatogram of chiral 71

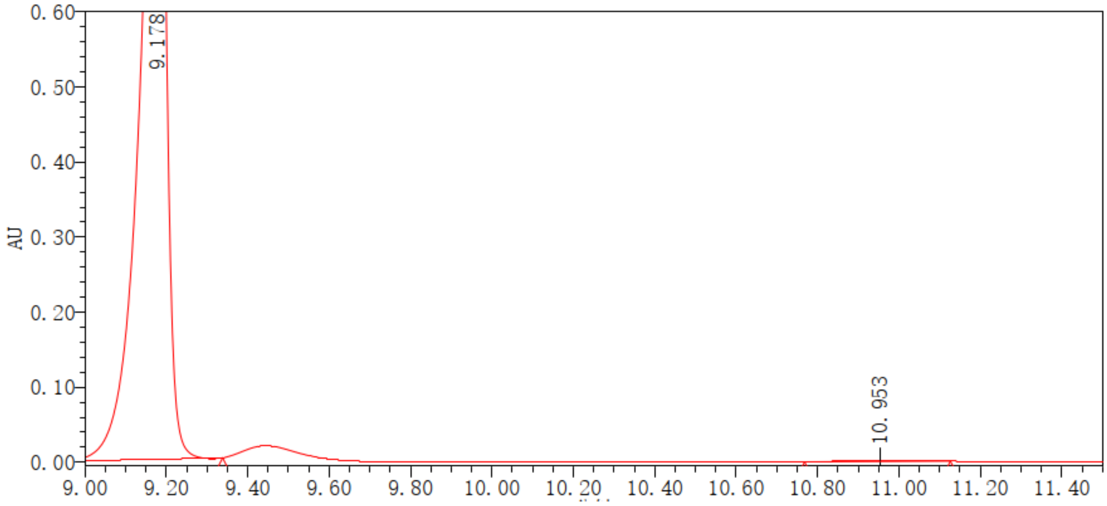

Entry	Retention Time/min	Area	Height	Area(%)
1	9.178	4898082	1206046	99.53
2	10.953	23259	1895	0.47

Supplementary Figure 152. Chiral HPLC analysis of chiral **71**

Tert-butyl (2S,3R)-2-((4-bromobenzyl)oxy)-5-(2,6-difluorophenyl)-3-(2-oxo-2-phenylethyl)-2-phenylpent-4-ynoate (72)

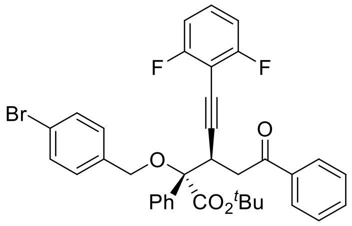

72

Colorless oil; 52.3 mg, 52% yield, >20:1 d.r., 97% *ee*, $[\alpha]_D^{20}$ = -14.33 (c = 0.1, CH_2Cl_2); ^1H NMR (400 MHz, $CDCl_3$) δ 7.85 – 7.79 (m, 2H), 7.72 – 7.66 (m, 2H), 7.53 (d, *J* = 7.4 Hz, 1H), 7.51 – 7.47 (m, 2H), 7.41 (d, *J* = 8.7 Hz, 3H), 7.39 – 7.34 (m, 2H), 7.31 (d, *J* = 7.1 Hz, 1H), 7.26 (s, 1H), 7.19 (tt, *J* = 8.4, 6.3 Hz, 1H), 6.87 – 6.79 (m, 1H), 5.11 (d, *J* = 11.9 Hz, 1H), 4.98 (d, *J* = 11.8 Hz, 1H), 4.52 (dd, *J* = 10.1, 3.0 Hz, 1H), 3.36 (dd, *J* = 17.0, 10.1 Hz, 1H), 3.07 (dd, *J* = 17.0, 3.0 Hz, 1H), 1.49 (s, 9H). ^{13}C NMR (101 MHz, $CDCl_3$) δ 197.1, 169.2, 138.2 (d, *J* = 22.7 Hz), 136.8, 133.1, 131.4, 129.28, 129.1, 128.5, 128.4, 128.3, 128.2, 128.2, 126.5, 121.2, 111.2 (d, *J* = 5.7 Hz), 111.2 (d, *J* = 5.7 Hz), 100.0, 85.7, 83.2, 68.2, 39.1, 36.5, 27.9. ^{19}F NMR (376 MHz, $CDCl_3$) δ -107.72. HRMS (ESI) $[M+Na]^+$ calcd for $C_{36}H_{31}O_4F_2BrNa^+$, 667.1266, found 667.1269. (Chiral IA-3, λ= 254 nm, *n*-hexane/2-propanol/EtOH= 197/1/2, Flow rate = 1.0 mL/min), t_R = 12.593 min(major), 14.042 min.

HPLC chromatogram of racemic 72

Condition: n-hexane/2-propanol/EtOH =197:1:2

Flow rate =1.0 mL/min

λ= 254 nm

Chiral IA-3

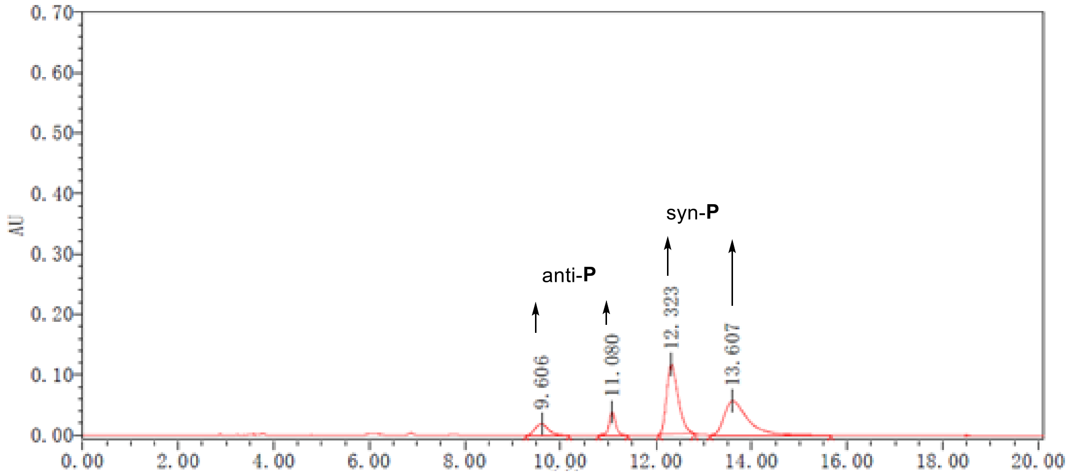

Entry	Retention Time/min	Area	Height	Area(%)
1	9.606	367764	19093	7.96
2	11.080	367126	38276	7.94
3	12.323	1949814	115556	42.18
4	13.607	1937580	56523	41.92

Supplementary Figure 153. Chiral HPLC analysis of racemic **72**

HPLC chromatogram of chiral 72

Condition: n-hexane/2-propanol/EtOH =197:1:2

Flow rate =1.0 mL/min

λ= 254 nm

Chiral IA-3

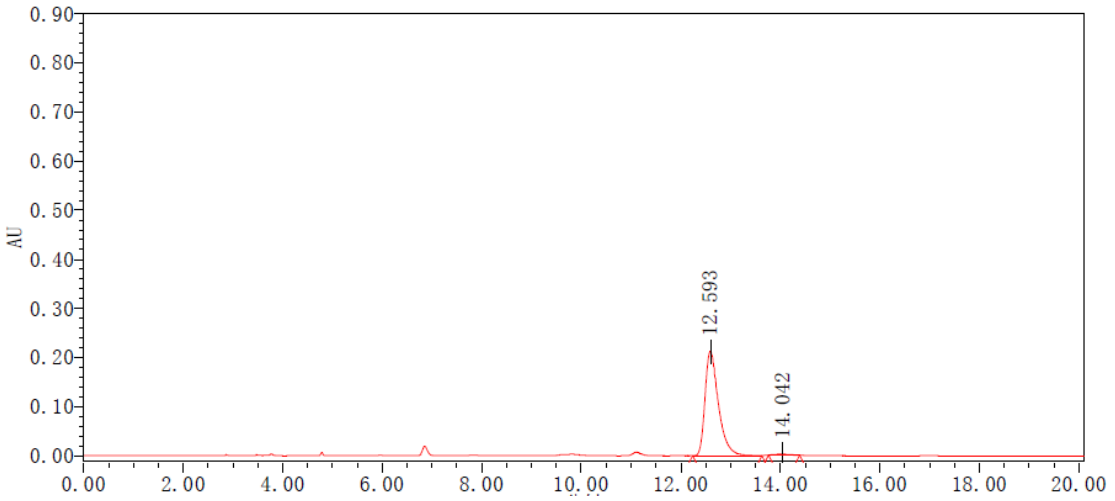

The larger version of HPLC chromatogram of chiral 72

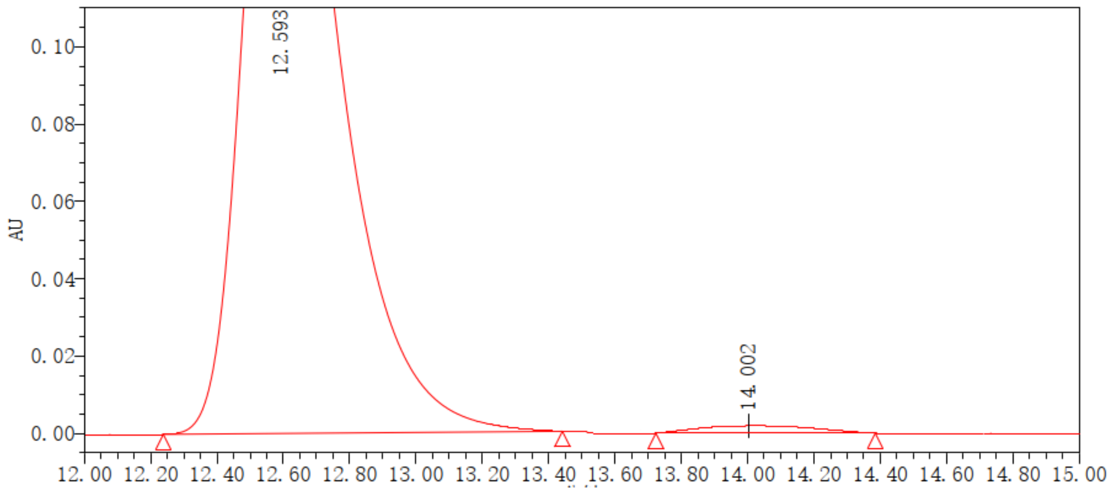

Entry	Retention Time/min	Area	Height	Area(%)
1	12.593	3997058	212561	98.51
2	14.042	39426	1713	1.49

Supplementary Figure 154. Chiral HPLC analysis of chiral **72**

Tert-butyl (2S,3R)-2-((4-bromobenzyl)oxy)-5-(4-methoxyphenyl)-3-(2-oxo-2-phenylethyl)-2-phenylpent-4-ynoate (73)

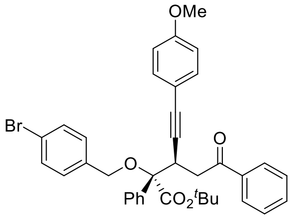

73

Colorless oil; 53.6 mg, 56% yield, >20:1 d.r., >99% *ee*; ^{1}H NMR (500 MHz, $CDCl_3$) δ 7.83 (d, *J* = 7.4 Hz, 2H), 7.69 (d, *J* = 7.8 Hz, 2H), 7.52 (t, *J* = 7.1 Hz, 1H), 7.48 (d, *J* = 7.2 Hz, 2H), 7.37 (dd, *J* = 16.0, 8.1 Hz, 7H), 7.17 (d, *J* = 7.7 Hz, 2H), 6.76 (d, *J* = 7.7 Hz, 2H), 5.06 (d, *J* = 11.8 Hz, 1H), 4.91 (d, *J* = 11.8 Hz, 1H), 4.39 (d, *J* = 9.8 Hz, 1H), 3.76 (s, 3H), 3.31 (dd, *J* = 16.4, 10.2 Hz, 1H), 3.06 (d, *J* = 16.4 Hz, 1H), 1.51 (s, 9H). ^{13}C NMR (126 MHz, $CDCl_3$) δ 197.5, 169.5, 159.3, 138.4, 138.1, 137.0, 133.1, 132.7, 131.4, 129.2, 128.5, 128.3, 128.2, 126.7, 126.7, 121.2, 115.6, 113.8, 88.1, 85.9, 840, 82.9, 68.0, 55.3, 39.3, 36.6, 28.1. HRMS (ESI) $[M+Na]^+$ calcd for $C_{37}H_{35}O_5BrNa^+$, 661.1560, found 661.1558. (Chiral IA, λ= 254 nm, *n*-hexane/2-propanol= 19/1, Flow rate = 1.0 mL/min), t_R = 10.250 min(major), 19.466 min.

HPLC chromatogram of racemic 73

Condition: n-hexane/2-propanol =19:1

Flow rate =1.0 mL/min

λ= 254 nm

Chiral IA

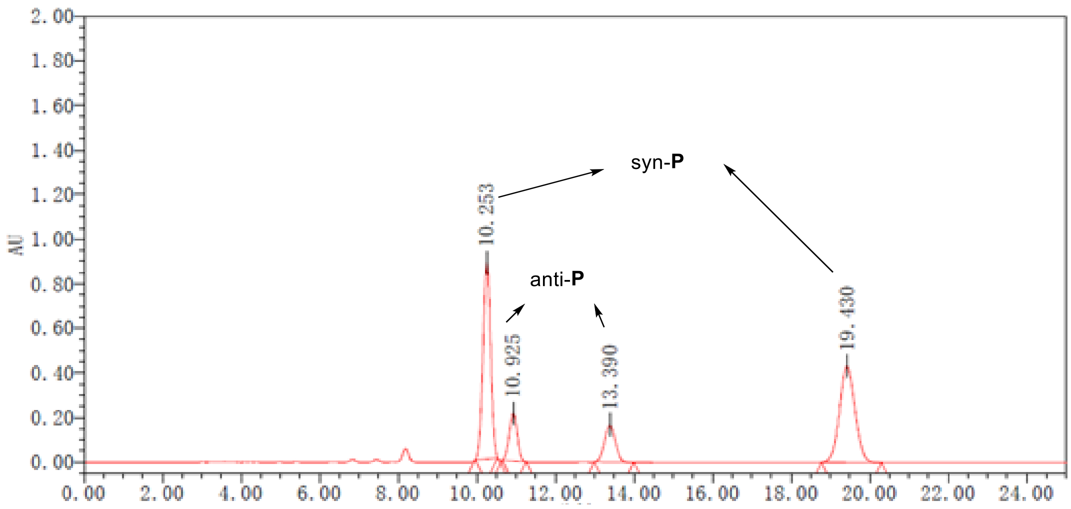

Entry	Retention Time/min	Area	Height	Area(%)
1	10.253	12066968	880501	39.96
2	10.925	3086483	213067	10.22
3	13.390	3125223	165652	10.35
4	19.430	11917599	433062	39.47

Supplementary Figure 155. Chiral HPLC analysis of racemic **73**

HPLC chromatogram of chiral 73

Condition: n-hexane/2-propanol =19:1

Flow rate =1.0 mL/min

λ= 254 nm

Chiral IA

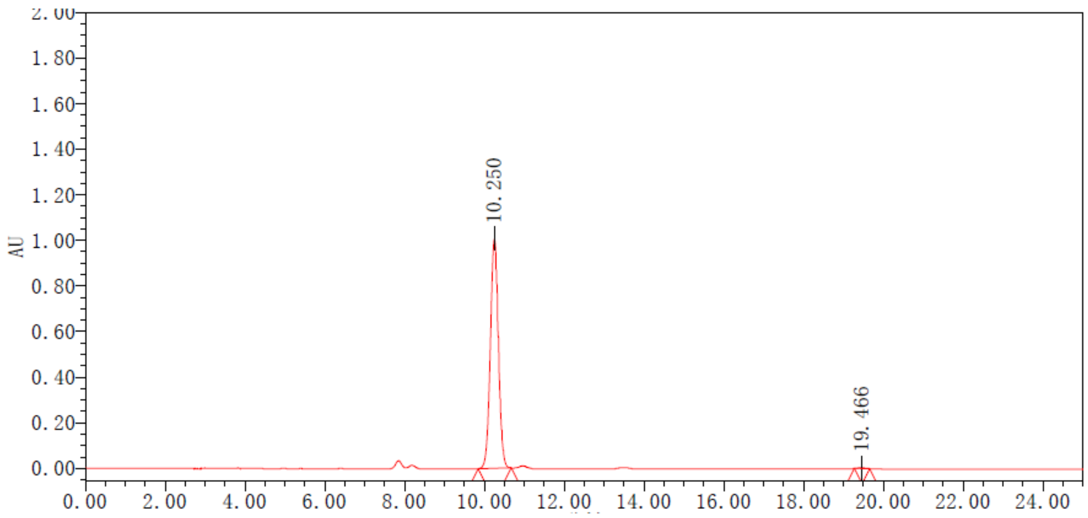

The larger version of HPLC chromatogram of chiral 73

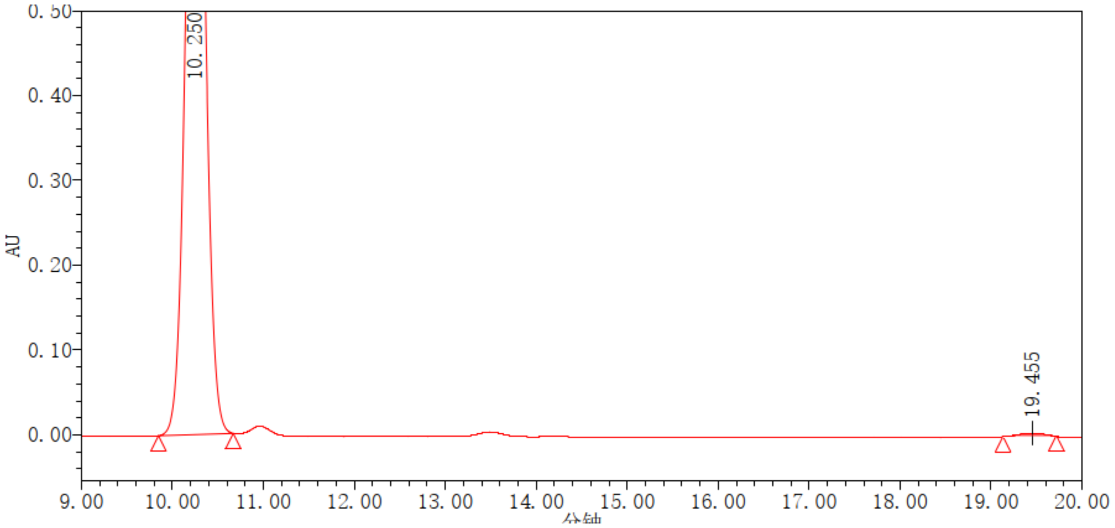

Entry	Retention Time/min	Area	Height	Area(%)
1	10.250	14322650	1007259	99.76
2	19.466	34586	2328	0.24

Supplementary Figure 156. Chiral HPLC analysis of chiral **73**

Tert-butyl (2S,3R)-2-((4-bromobenzyl)oxy)-3-(2-oxo-2-phenylethyl)-2-phenyl-5-(4-(trifluoromethyl)phenyl)pent-4-ynoate (74)

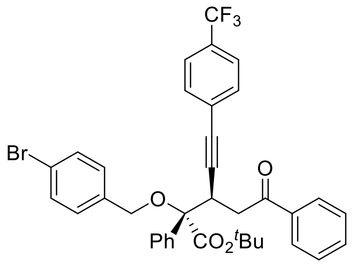

74

Colorless oil; 65.0 mg, 62% yield, >20:1 d.r., 98% *ee*; ^{1}H NMR (400 MHz, $CDCl_3$) δ 7.88 – 7.84 (m, 2H), 7.68 – 7.64 (m, 2H), 7.54 (t, *J* = 7.4 Hz, 1H), 7.50 – 7.45 (m, 4H), 7.43 (d, *J* = 7.8 Hz, 2H), 7.39 (d, *J* = 7.1 Hz, 2H), 7.36 (t, *J* = 2.5 Hz, 2H), 7.33 (d, *J* = 2.3 Hz, 1H), 7.30 (d, *J* = 8.3 Hz, 2H), 4.99 (d, *J* = 11.7 Hz, 1H), 4.80 (d, *J* = 11.6 Hz, 1H), 4.41 (dd, *J* = 10.1, 3.1 Hz, 1H), 3.33 (dd, *J* = 16.7, 10.1 Hz, 1H), 3.18 (dd, *J* = 16.7, 3.1 Hz, 1H), 1.52 (s, 9H). ^{13}C NMR (101 MHz, $CDCl_3$) δ 197.2, 169.3, 137.9 (d, *J* = 14.8 Hz), 136.8, 133.3, 131.6, 131.4, 129.1, 128.6, 128.6, 128.5, 128.4, 128.4, 128.2, 127.21 (d, *J* = 11.6 Hz), 126.8, 125.2 (q, *J* = 3.7 Hz), 121.3, 92.5, 85.9, 83.1, 67.9, 39.1, 36.9, 28.1. ^{19}F NMR (376 MHz, $CDCl_3$) δ -62.80. HRMS (ESI) $[M+Na]^+$ calcd for $C_{37}H_{32}O_4F_3BrNa^+$, 699.1328, found 699.1328. (Chiral IA, λ= 254 nm, *n*-hexane/2-propanol= 19/1, Flow rate = 1.0 mL/min), t_R = 6.894 min (major), 11.067 min.

HPLC chromatogram of racemic 74

Condition: n-hexane/2-propanol =19:1

Flow rate =1.0 mL/min

λ= 254 nm

Chiral IA

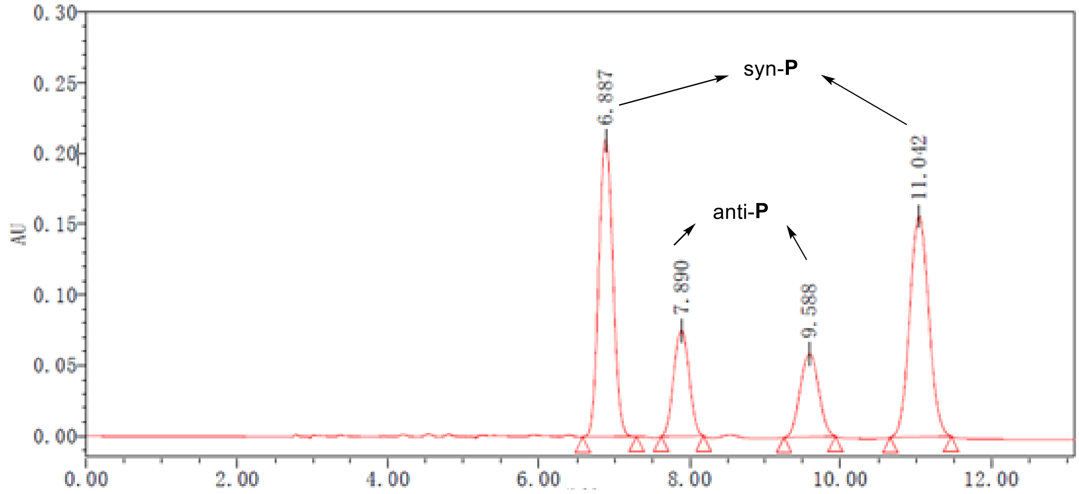

Entry	Retention Time/min	Area	Height	Area(%)
1	6.887	2774985	210225	36.38
2	7.890	1075225	75013	14.10
3	9.588	1014480	59184	13.30
4	11.042	2763395	156333	36.23

Supplementary Figure 157. Chiral HPLC analysis of racemic **74**

HPLC chromatogram of chiral 74

Condition: n-hexane/2-propanol =19:1

Flow rate =1.0 mL/min

λ= 254 nm

Chiral IA

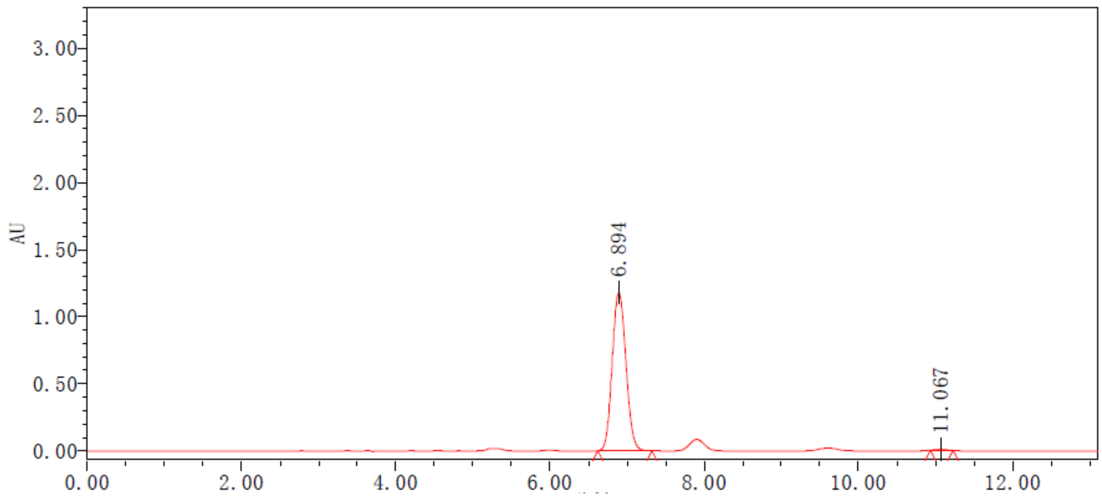

The larger version of HPLC chromatogram of chiral 74

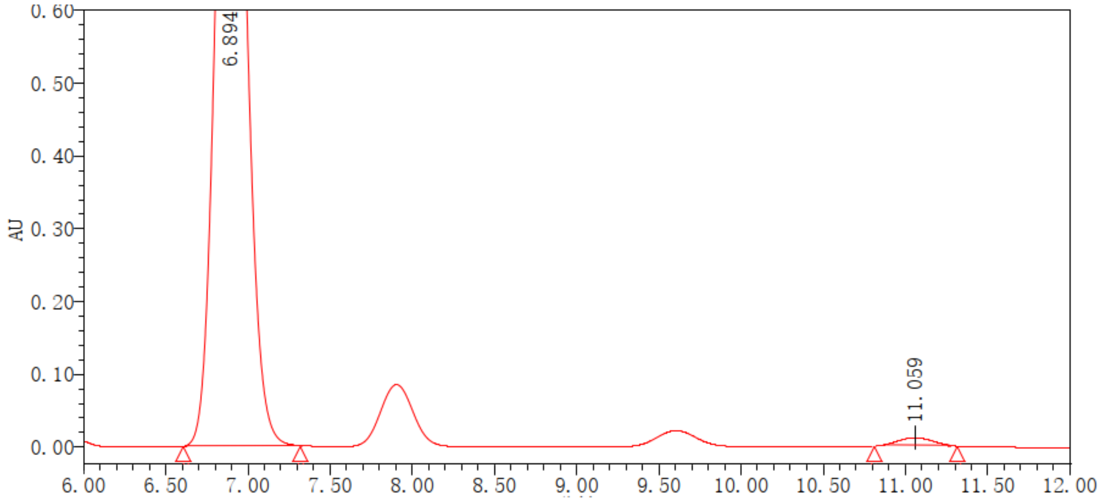

Entry	Retention Time/min	Area	Height	Area(%)
1	6.894	14644587	1176627	99.18
2	11.059	74337	6962	0.82

Supplementary Figure 158. Chiral HPLC analysis of chiral **74**

Tert-butyl (2S,3R)-2-((4-bromobenzyl)oxy)-5-(4-cyanophenyl)-3-(2-oxo-2-phenylethyl)-2-phenylpent-4-ynoate (75)

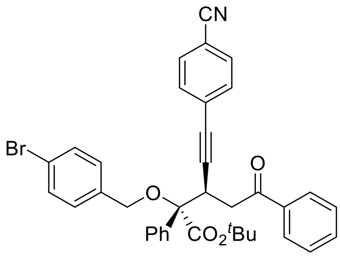

75

Colorless oil; 46.7 mg, 49% yield, >20:1 d.r., 98% *ee*; ^{1}H NMR (400 MHz, $CDCl_3$) δ 7.88 – 7.83 (m, 2H), 7.64 (d, *J* = 7.1 Hz, 2H), 7.57 – 7.53 (m, 1H), 7.52 – 7.47 (m, 3H), 7.45 (d, *J* = 4.2 Hz, 2H), 7.42 (d, *J* = 7.7 Hz, 2H), 7.38 (d, *J* = 7.7 Hz, 2H), 7.32 (d, *J* = 8.3 Hz, 2H), 7.26 (d, *J* = 7.6 Hz, 2H), 4.95 (d, *J* = 11.6 Hz, 1H), 4.74 (d, *J* = 11.6 Hz, 1H), 4.40 (dd, *J* = 9.8, 3.3 Hz, 1H), 3.32 (dd, *J* = 16.7, 9.9 Hz, 1H), 3.23 (dd, *J* = 16.7, 3.3 Hz, 1H), 1.52 (s, 9H). ^{13}C NMR (101 MHz, $CDCl_3$) δ 197.0, 169.3, 137.8, 137.7, 136.7, 133.3, 131.9, 131.9, 131.4, 129.1, 128.6, 128.5, 128.4, 128.3, 128.2, 126.8, 121.4, 118.5, 111.3, 94.7, 85.9, 83.2, 82.6, 67.9, 39.1, 37.1, 28.1. HRMS (ESI) $[M+Na]^+$ calcd for $C_{37}H_{32}NO_4BrNa^+$, 656.1407, found 656.1411. (Chiral IA-3, λ= 254 nm, *n*-hexane/2-propanol= 19/1, Flow rate = 1.0 mL/min), t_R = 13.971 min(major), 27.226 min.

HPLC chromatogram of racemic 75

Condition: n-hexane/2-propanol =19:1

Flow rate =1.0 mL/min

λ= 254 nm

Chiral IA-3

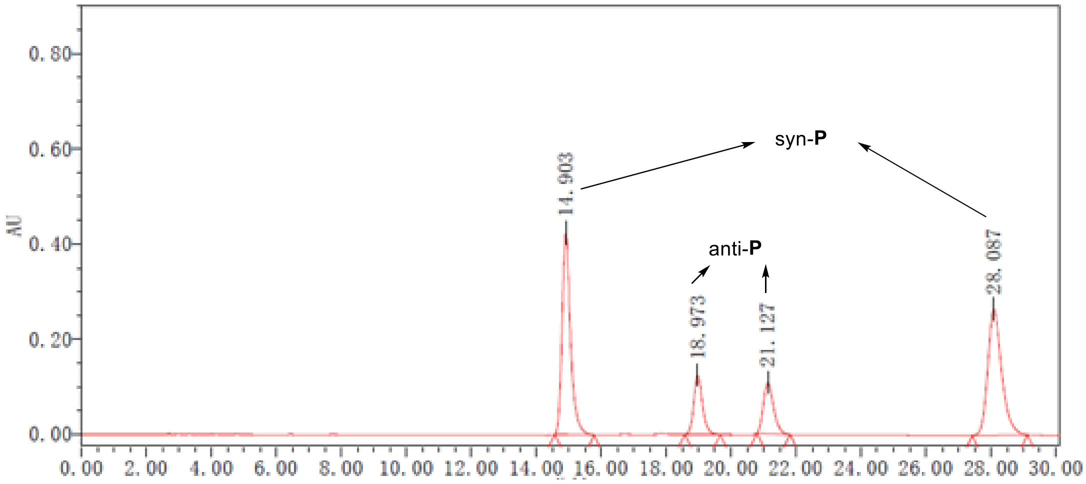

Entry	Retention Time/min	Area	Height	Area(%)
1	14.903	7931507	424450	38.32
2	18.973	2414402	124514	11.67
3	21.127	2348469	107737	11.35
4	28.087	8001633	264366	38.66

Supplementary Figure 159. Chiral HPLC analysis of racemic **75**

HPLC chromatogram of chiral 75

Condition: n-hexane/2-propanol =19:1

Flow rate =1.0 mL/min

λ= 254 nm

Chiral IA-3

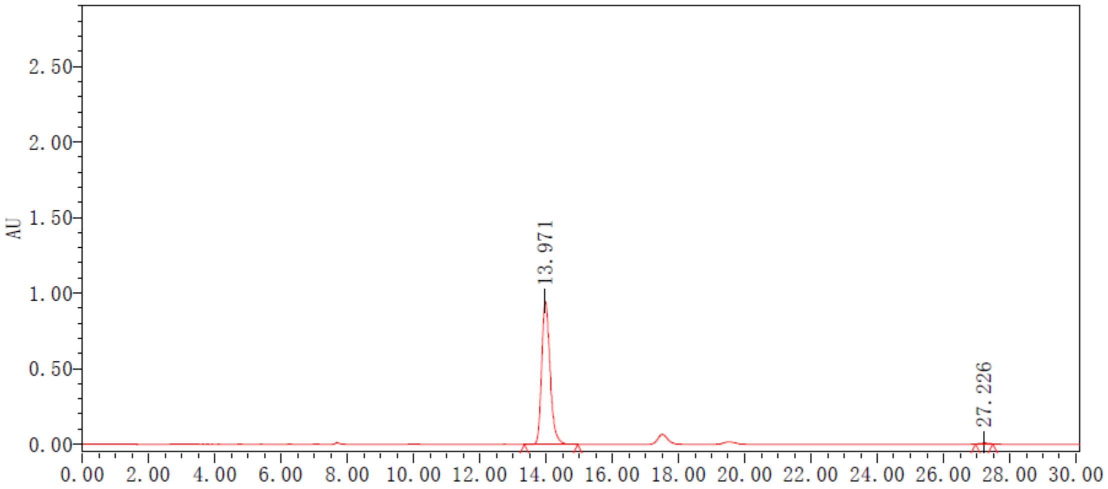

The larger version of HPLC chromatogram of chiral 75

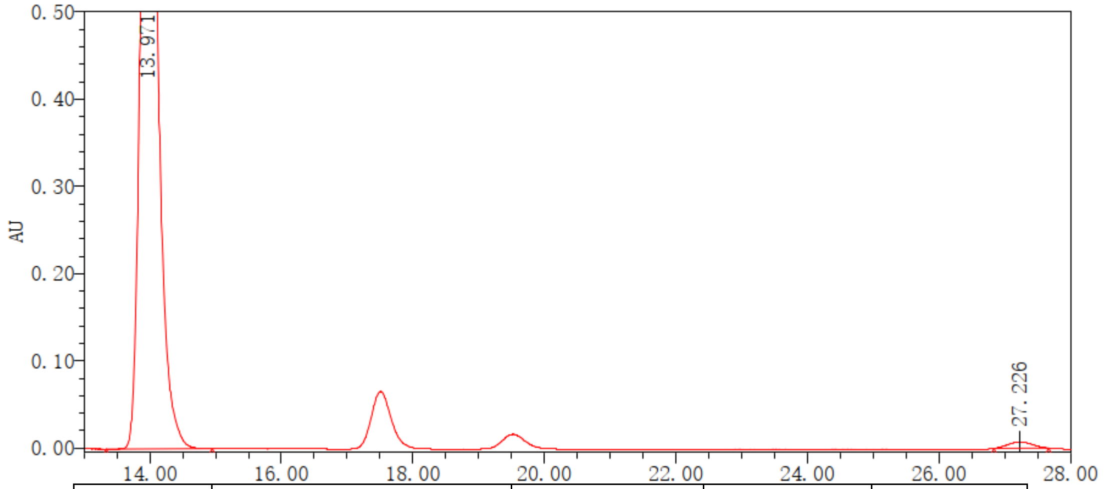

Entry	Retention Time/min	Area	Height	Area(%)
1	13.971	16765775	944933	99.08
2	27.226	87866	4621	0.92

Supplementary Figure 160. Chiral HPLC analysis of chiral **75**

Tert-butyl (2S,3R)-2-((4-bromobenzyl)oxy)-5-(furan-2-yl)-3-(2-oxo-2-phenylethyl)-2-phenylpent-4-ynoate (76)

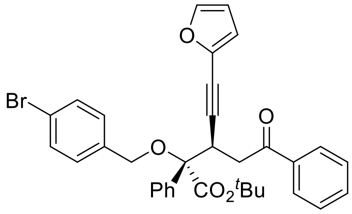

76

Colorless oil; 39.6 mg, 47% yield, >20:1 d.r., 98% *ee*; ^{1}H NMR (500 MHz, $CDCl_3$) δ 7.82 (d, *J* = 7.4 Hz, 2H), 7.66 (d, *J* = 7.5 Hz, 2H), 7.52 (t, *J* = 7.4 Hz, 1H), 7.48 (d, *J* = 8.3 Hz, 2H), 7.42 – 7.39 (m, 3H), 7.38 (t, *J* = 3.6 Hz, 1H), 7.35 (d, *J* = 7.8 Hz, 2H), 7.31 (d, *J* = 7.2 Hz, 1H), 7.29 (d, *J* = 1.2 Hz, 1H), 6.39 (d, *J* = 3.3 Hz, 1H), 6.31 (dd, *J* = 3.2, 1.9 Hz, 1H), 5.00 (d, *J* = 11.7 Hz, 1H), 4.86 (d, *J* = 11.7 Hz, 1H), 4.45 (dd, *J* = 10.0, 2.9 Hz, 1H), 3.31 (dd, *J* = 17.1, 10.1 Hz, 1H), 3.10 (dd, *J* = 17.1, 2.8 Hz, 1H), 1.53 (s, 9H). ^{13}C NMR (126 MHz, $CDCl_3$) δ 196.9, 169.2, 143.1, 138.1, 138.0, 137.1, 136.7, 133.2, 131.4, 129.3, 128.6, 128.4, 128.3, 128.2, 126.6, 121.3, 114.4, 110.7, 94.0, 85.7, 83.2, 74.5, 68.0, 39.0, 36.4, 28.0. HRMS (ESI) $[M+Na]^+$ calcd for $C_{34}H_{31}O_5BrNa^+$, 621.1247, found 621.1247. (Chiral IE-3, λ= 254 nm, *n*-hexane/2-propanol= 19/1, Flow rate = 1.0 mL/min), t_R = 11.605 min(major), 27.521 min.

HPLC chromatogram of racemic 76

Condition: n-hexane/2-propanol =19:1

Flow rate =1.0 mL/min

λ= 254 nm

Chiral IE-3

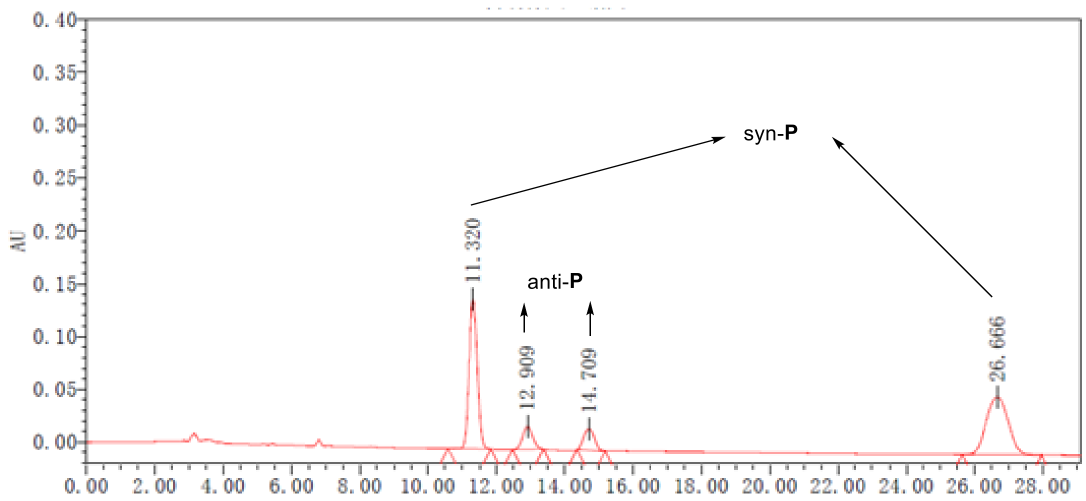

Entry	Retention Time/min	Area	Height	Area(%)
1	11.320	2444372	141407	42.33
2	12.909	459230	21538	7.95
3	14.709	449122	19961	7.78
4	26.666	2421323	54570	41.93

Supplementary Figure 161. Chiral HPLC analysis of racemic **76**

HPLC chromatogram of chiral 76

Condition: n-hexane/2-propanol =19:1

Flow rate =1.0 mL/min

λ= 254 nm

Chiral IE-3

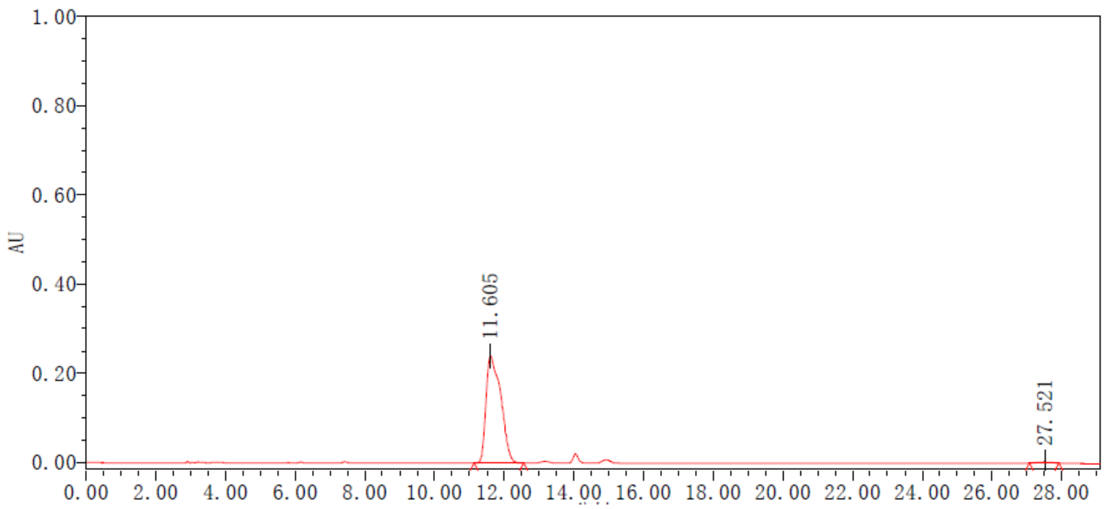

Entry	Retention Time/min	Area	Height	Area(%)
1	11.605	6999862	240744	99.06
2	27.521	66662	2155	0.94

Supplementary Figure 162. Chiral HPLC analysis of chiral **76**

Tert-butyl (2S,3R,E)-2-((4-bromobenzyl)oxy)-3-(2-oxo-2-phenylethyl)-2,7-diphenylhept-6-en-4-ynoate (77)

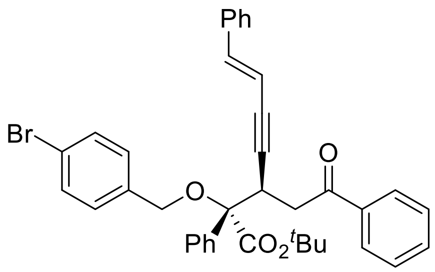

77

Colorless oil; 50.4 mg, 53% yield, >20:1 d.r., 97% *ee*; ^{1}H NMR (500 MHz, $CDCl_3$) δ 7.86 – 7.82 (m, 2H), 7.67 (d, *J* = 7.5 Hz, 2H), 7.53 (t, *J* = 7.4 Hz, 1H), 7.49 (d, *J* = 8.4 Hz, 2H), 7.43 – 7.34 (m, 6H), 7.34 – 7.27 (m, 5H), 7.26 – 7.24 (m, 1H), 6.71 (d, *J* = 16.2 Hz, 1H), 6.07 (dd, *J* = 16.2, 2.0 Hz, 1H), 5.01 (d, *J* = 11.7 Hz, 1H), 4.87 (d, *J* = 11.7 Hz, 1H), 4.44 – 4.32 (m, 1H), 3.28 (dd, *J* = 16.8, 10.3 Hz, 1H), 3.08 (dd, *J* = 16.8, 2.9 Hz, 1H), 1.53 (s, 9H). ^{13}C NMR (126 MHz, $CDCl_3$) δ 197.4, 169.4, 140.7, 138.3, 138.1, 136.8, 136.3, 133.2, 131.4, 129.2, 128.7, 128.6, 128.5, 128.3, 128.3, 128.2, 126.7,

126.1, 121.3, 108.3, 91.9, 85.9, 83.4, 83.0, 67.9, 39.2, 36.7, 28.1. HRMS (ESI) $[M+Na]^+$ calcd for $C_{38}H_{35}O_4BrNa^+$, 657.1611, found 657.1612. (Chiral IE-3, λ= 254 nm, *n*-hexane/2-propanol= 19/1, Flow rate = 1.0 mL/min), t_R = 15.071 min(major), 21.541 min.

HPLC chromatogram of racemic 77

Condition: n-hexane/2-propanol =19:1

Flow rate =1.0 mL/min

λ= 254 nm

Chiral IE-3

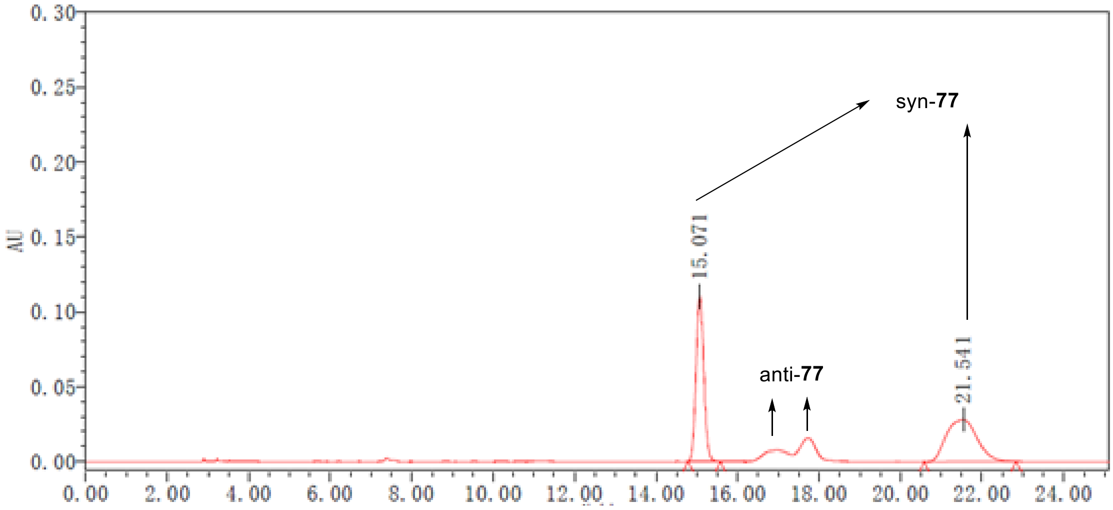

Entry	Retention Time/min	Area	Height	Area(%)
1	15.071	1489715	110158	50.18
2	21.541	1478921	28046	49.82

Supplementary Figure 163. Chiral HPLC analysis of racemic **77**

HPLC chromatogram of chiral 77

Condition: n-hexane/2-propanol =19:1

Flow rate =1.0 mL/min

λ= 254 nm

Chiral IE-3

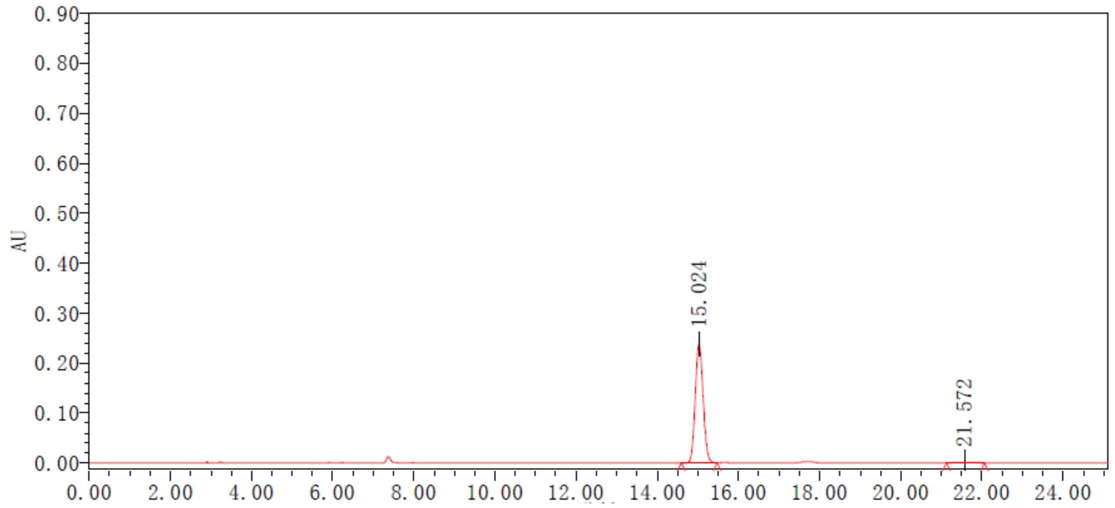

The larger version of HPLC chromatogram of chiral 77

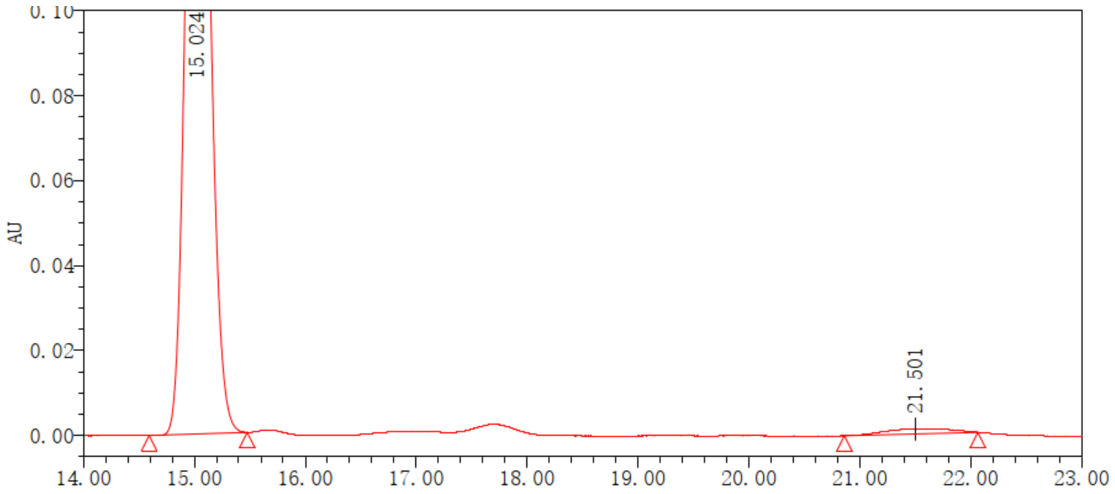

Entry	Retention Time/min	Area	Height	Area(%)
1	15.024	3211828	238201	98.48
2	21.051	33087	901	1.52

Supplementary Figure 164. Chiral HPLC analysis of chiral **77**

Tert-butyl (2S,3R,E)-2-((4-bromobenzyl)oxy)-6-(4-methoxyphenyl)-6-oxo-3-(2-oxo-2-phenylethyl)-2-phenylhex-4-enoate (78)

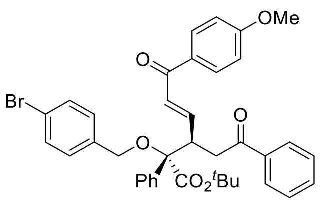

78

Colorless oil; 55.1 mg, 55% yield, >20:1 d.r., 89% *ee*; ^{1}H NMR (400 MHz, $CDCl_3$) δ 7.90 (d, *J* = 7.2 Hz, 2H), 7.71 (d, *J* = 8.9 Hz, 2H), 7.57 (t, *J* = 7.0 Hz, 3H), 7.47 – 7.41 (m, 5H), 7.36 (dd, *J* = 14.5, 7.4 Hz, 2H), 7.22 (d, *J* = 8.3 Hz, 2H), 6.94 (dd, *J* = 15.5, 8.4 Hz, 1H), 6.87 (d, *J* = 8.9 Hz, 2H), 6.70 (d, *J* = 15.6 Hz, 1H), 4.73 (d, *J* = 11.8 Hz, 1H), 4.30 (d, *J* = 11.8 Hz, 1H), 4.09 – 3.99 (m, 1H), 3.87 (s, 3H), 3.57 (dd, *J* = 16.7, 2.7 Hz, 1H), 3.15 (dd, *J* = 16.7, 9.6 Hz, 1H), 1.57 (s, 9H). ^{13}C NMR (126 MHz, $CDCl_3$) δ 197.9, 188.8, 169.8, 163.2, 145.6, 137.8, 137.3, 136.9, 133.2, 131.3, 130.9, 130.6, 128.9, 128.6, 128.5, 128.3, 128.1, 127.7, 121.1, 113.6, 87.1, 83.4, 67.4, 55.5, 48.2, 38.7, 29.7, 28.2. HRMS (ESI) $[M+Na]^+$ calcd for $C_{38}H_{36}O_6BrNa^+$, 691.1666, found 691.1662. (Chiral IA, λ= 254 nm, *n*-hexane/2-propanol= 9/1, Flow rate = 1.0 mL/min), t_R = 20.334 min(major), 24.716 min.

HPLC chromatogram of racemic 78

Condition: n-hexane/2-propanol =9:1

Flow rate =1.0 mL/min

λ= 254 nm

Chiral IA

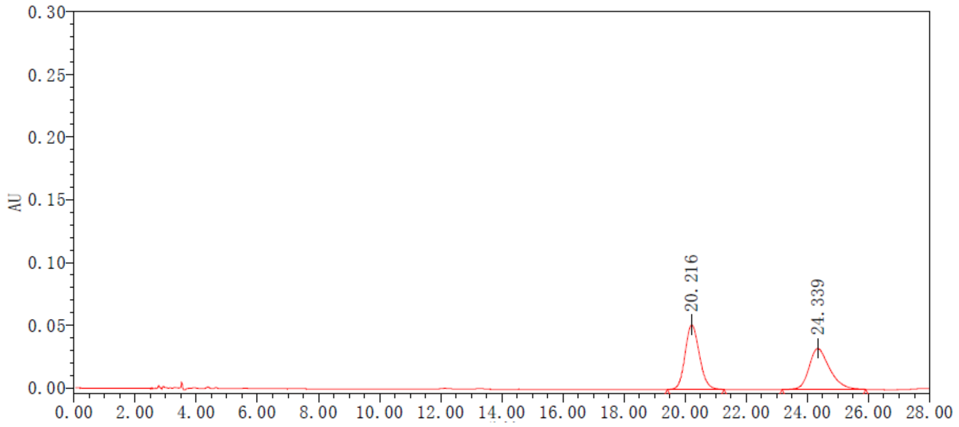

Entry	Retention Time/min	Area	Height	Area(%)
1	20.216	1602120	51569	50.40
2	24.339	1592157	32701	49.60

Supplementary Figure 165. Chiral HPLC analysis of racemic **78**

HPLC chromatogram of chiral 78

Condition: n-hexane/2-propanol =9:1

Flow rate =1.0 mL/min

λ= 254 nm

Chiral IA

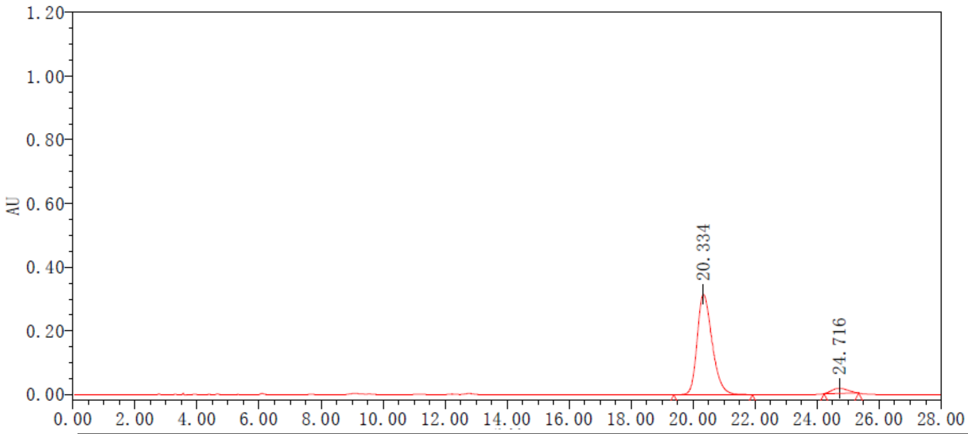

Entry	Retention Time/min	Area	Height	Area(%)
1	20.334	10474346	314667	94.47
2	24.716	570770	15766	5.53

Supplementary Figure 166. Chiral HPLC analysis of chiral **78**

Tert-butyl (2S,3R,E)-2-((4-bromobenzyl)oxy)-6-(4-bromophenyl)-6-oxo-3-(2-oxo-2-phenylethyl)-2-phenylhex-4-enoate (79)

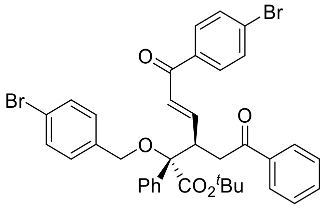

79

Colorless oil; 46.2 mg, 43% yield, >20:1 d.r., 82% *ee*; ^{1}H NMR (400 MHz, $CDCl_3$) δ 7.88 (d, *J* = 7.4 Hz, 1H), 7.66 (d, *J* = 8.5 Hz, 1H), 7.58 – 7.48 (m, 5H), 7.45 – 7.34 (m, 5H), 7.18 (d, *J* = 8.3 Hz, 1H), 6.92 (dd, *J* = 15.6, 8.4 Hz, 1H), 6.57 (d, *J* = 15.6 Hz, 1H), 4.70 (d, *J* = 11.8 Hz, 1H), 4.25 (d, *J* = 11.8 Hz, 1H), 3.99 (t, *J* = 8.1 Hz, 1H), 3.59 (dd, *J* = 16.7, 2.6 Hz, 1H), 3.12 (dd, *J* = 16.7, 9.7 Hz, 1H), 1.55 (s, 9H). ^{13}C NMR (126 MHz, $CDCl_3$) δ 197.7, 189.7, 180.0, 169.7, 165.4, 147.4, 145.2, 137.7, 136.4, 133.3, 131.7, 131.4, 130.1, 128.9, 128.7, 128.6, 128.2, 128.1, 127.7, 122.4, 121.1, 87.1, 83.5, 67.5, 48.5, 38.6, 28.2. HRMS (ESI) $[M+Na]^+$ calcd for $C_{37}H_{35}O_5Br_2Na^+$, 739.0665, found 739.0660. (Chiral IC, λ= 254 nm, *n*-hexane/2-propanol= 9/1, Flow rate = 1.0 mL/min), t_R = 15.663 min(major), 16.958 min.

HPLC chromatogram of racemic 79

Condition: n-hexane/2-propanol =9:1

Flow rate =1.0 mL/min

λ= 254 nm

Chiral IC

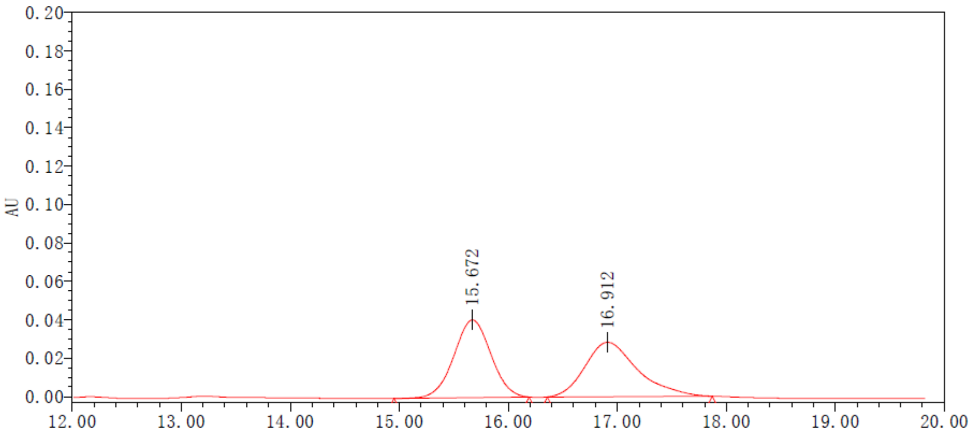

Entry	Retention Time/min	Area	Height	Area(%)
1	15.672	953526	40431	50.22
2	16.912	945207	28267	49.78

Supplementary Figure 167. Chiral HPLC analysis of racemic **79**

HPLC chromatogram of chiral 79

Condition: n-hexane/2-propanol =9:1

Flow rate =1.0 mL/min

λ= 254 nm

Chiral IC

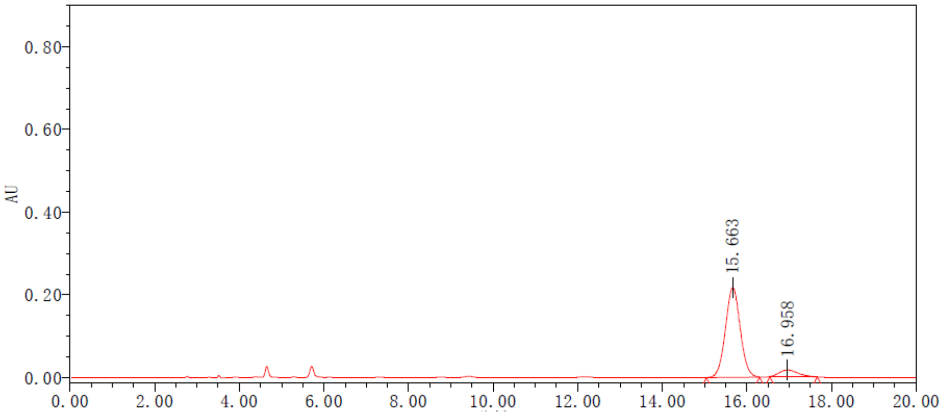

Entry	Retention Time/min	Area	Height	Area(%)
1	15.663	5262189	216676	91.12
2	16.958	512656	16602	8.88

Supplementary Figure 168. Chiral HPLC analysis of chiral **79**

Tert-butyl (2S,3R,E)-2-((4-bromobenzyl)oxy)-6-(naphthalen-1-yl)-6-oxo-3-(2-oxo-2-phenylethyl)-2-phenylhex-4-enoate (80)

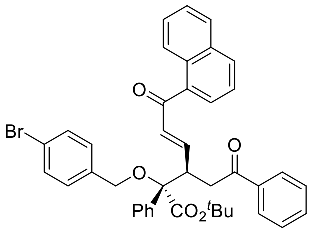

80

Colorless oil; 43.3 mg, 42% yield, >20:1 d.r., 87% *ee*; ^{1}H NMR (400 MHz, $CDCl_3$) δ 8.11 (s, 1H), 7.90 (d, *J* = 7.2 Hz, 2H), 7.87 – 7.78 (m, 4H), 7.55 (p, *J* = 6.9 Hz, 6H), 7.41 (dd, *J* = 7.2, 5.0 Hz, 4H), 7.36 (d, *J* = 7.1 Hz, 1H), 7.20 (d, *J* = 8.3 Hz, 2H), 7.00 (dd, *J* = 15.6, 8.4 Hz, 2H), 6.79 (d, *J* = 15.6 Hz, 1H), 4.73 (d, *J* = 11.9 Hz, 1H), 4.29 (d, *J* = 11.8 Hz, 1H), 4.10 – 4.03 (m, 1H), 3.60 (dd, *J* = 16.7, 2.7 Hz, 1H), 3.16 (dd, *J* = 16.7, 9.6 Hz, 1H), 1.55 (s, 9H). ^{13}C NMR (101 MHz, $CDCl_3$) δ 197.8, 190.5, 169.8, 146.7, 137.8, 137.3, 136.9, 135.4, 135.0, 133.2, 132.4, 131.3, 130.7, 130.2, 129.5, 128.9, 128.7, 128.6, 128.6, 128.3, 128.3, 128.2, 127.9, 127.8, 126.6, 124.5, 121.1, 87.2, 83.5, 67.5, 48.4, 38.7, 29.7, 28.2. HRMS (ESI) $[M+Na]^+$ calcd for $C_{41}H_{37}O_5Na^+$, 711.1717, found 711.1716. (Chiral IA, λ= 254 nm, *n*-hexane/2-propanol= 9/1, Flow rate = 1.0 mL/min), t_R = 17.147 min(major), 27.484 min.

HPLC chromatogram of racemic 80

Condition: n-hexane/2-propanol =9:1

Flow rate =1.0 mL/min

λ= 254 nm

Chiral IA

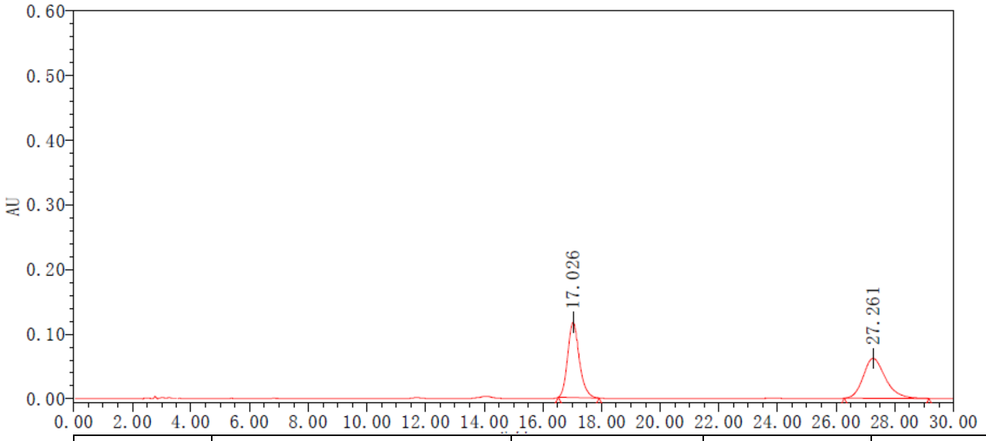

Entry	Retention Time/min	Area	Height	Area(%)
1	17.026	3239325	116348	49.98
2	27.261	3242134	61685	50.02

Supplementary Figure 169. Chiral HPLC analysis of racemic **80**

HPLC chromatogram of chiral 80

Condition: n-hexane/2-propanol =9:1

Flow rate =1.0 mL/min

λ= 254 nm

Chiral IA

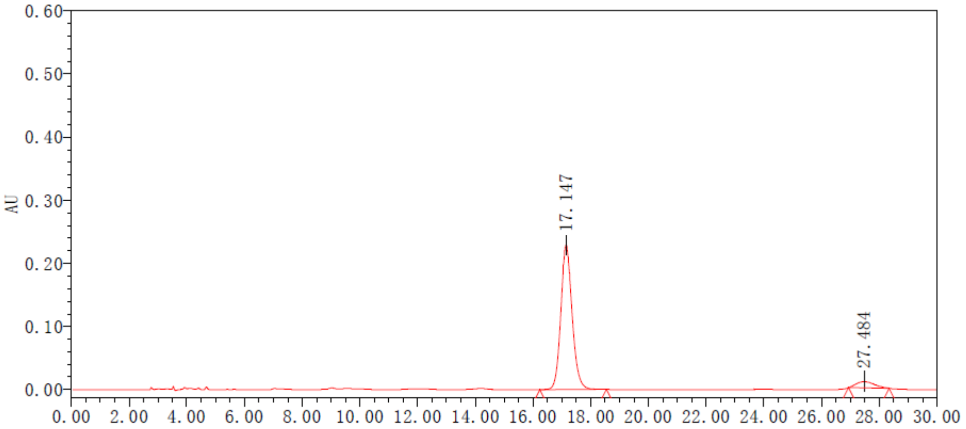

Entry	Retention Time/min	Area	Height	Area(%)
1	17.147	6289544	228806	93.51
2	27.484	436666	9969	6.49

Supplementary Figure 170. Chiral HPLC analysis of chiral **80**

Tert-butyl (2S,3R,E)-2-((4-bromobenzyl)oxy)-6-oxo-3-(2-oxo-2-phenylethyl)-2-phenyl-6-(thiophen-2-yl)hex-4-enoate (81)

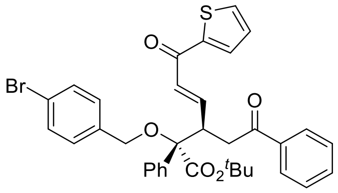

81

Colorless oil; 48.3 mg, 50% yield, >20:1 d.r., 80% *ee*; ^{1}H NMR (500 MHz, $CDCl_3$) δ 7.87 (d, *J* = 7.8 Hz, 2H), 7.60 – 7.56 (m, 1H), 7.54 (dd, *J* = 13.6, 7.4 Hz, 3H), 7.48 (d, *J* = 3.6 Hz, 1H), 7.44 – 7.36 (m, 6H), 7.33 (d, *J* = 7.0 Hz, 1H), 7.19 (d, *J* = 7.8 Hz, 2H), 7.06 (t, *J* = 3.9 Hz, 1H), 7.02 (dd, *J* = 15.5, 8.3 Hz, 1H), 6.60 (d, *J* = 15.4 Hz, 1H), 4.71 (d, *J* = 11.8 Hz, 1H), 4.28 (d, *J* = 11.8 Hz, 1H), 4.02 (t, *J* = 8.6 Hz, 1H), 3.56 (d, *J* = 16.5 Hz, 1H), 3.13 (dd, *J* = 16.7, 9.6 Hz, 1H), 1.56 (s, 9H). ^{13}C NMR (126 MHz, $CDCl_3$) δ 197.8, 182.0, 169.7, 146.1, 145.0, 137.8, 137.2, 136.8, 133.8, 133.2, 132.1, 131.3, 128.9, 128.6, 128.6, 128.5, 128.1, 128.1, 127.8, 127.7, 121.1, 87.1, 83.5, 67.5, 48.0, 38.7, 28.2. HRMS (ESI) $[M+Na]^+$ calcd for $C_{35}H_{33}O_5SBrNa^+$, 667.1124, found 667.1128. (Chiral IC, λ= 254 nm, *n*-hexane/2-propanol= 9/1, Flow rate = 1.0 mL/min), t_R = 18.306 min, 23.095 min (major).

HPLC chromatogram of racemic 81

Condition: n-hexane/2-propanol =9:1

Flow rate =1.0 mL/min

λ= 254 nm

Chiral IC

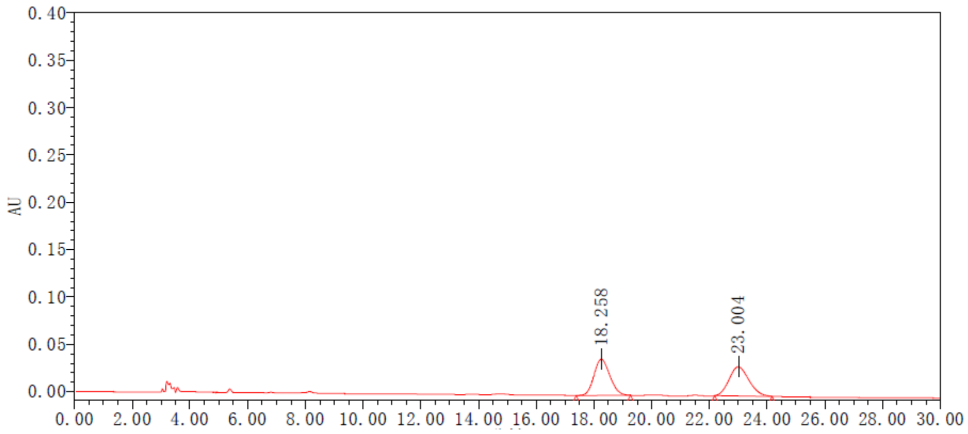

Entry	Retention Time/min	Area	Height	Area(%)
1	18.258	1464581	38159	49.79
2	23.004	1477072	30848	50.21

Supplementary Figure 171. Chiral HPLC analysis of racemic **81**

HPLC chromatogram of chiral 81

Condition: n-hexane/2-propanol =9:1

Flow rate =1.0 mL/min

λ= 254 nm

Chiral IC

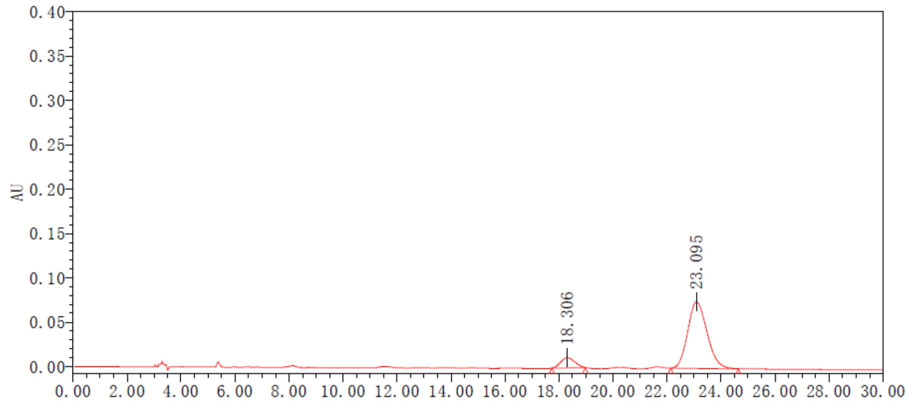

Entry	Retention Time/min	Area	Height	Area(%)
1	18.306	406258	11289	9.82
2	23.095	3730119	75142	90.18

Supplementary Figure 172. Chiral HPLC analysis of chiral **81**

6-(tert-butyl) 1-ethyl (4R,5S,E)-5-((4-bromobenzyl)oxy)-4-(2-oxo-2-phenylethyl)-5-phenylhex-2-enedioate (82)

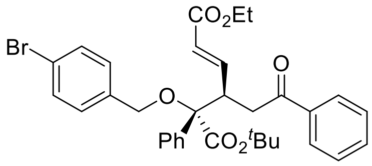

82

Colorless oil; 45.5 mg, 50% yield, >20:1 d.r., 87% *ee*; ^{1}H NMR (500 MHz, $CDCl_3$) δ 7.86 (d, *J* = 7.3 Hz, 2H), 7.54 (d, *J* = 7.5 Hz, 1H), 7.49 (d, *J* = 7.3 Hz, 2H), 7.43 – 7.40 (m, 4H), 7.35 (dd, *J* = 16.3, 7.4 Hz, 3H), 7.18 (d, *J* = 8.3 Hz, 2H), 6.94 (dd, *J* = 15.8, 8.0 Hz, 1H), 5.66 (d, *J* = 15.8 Hz, 1H), 4.68 (d, *J* = 11.8 Hz, 1H), 4.23 (d, *J* = 11.8 Hz, 1H), 4.08 (q, *J* = 7.2 Hz, 2H), 3.94 (d, *J* = 8.8 Hz, 1H), 3.51 (dd, *J* = 17.0, 2.6 Hz, 1H), 3.06 (dd, *J* = 17.0, 9.5 Hz, 1H), 1.56 (s, 9H), 1.20 (t, *J* = 7.1 Hz, 3H). ^{13}C NMR (126 MHz, $CDCl_3$) δ 197.5, 169.7, 166.2, 146.7, 137.8, 137.1, 136.7, 133.2, 131.3, 128.9, 128.6, 128.5, 128.4, 128.1, 127.6, 123.5, 121.1, 86.9, 83.4, 67.3, 60.2, 38.4, 28.2, 14.2. HRMS (ESI) $[M+Na]^+$ calcd for $C_{33}H_{35}O_6BrNa^+$, 629.1509, found 629.1507. (Chiral IA-3, λ= 254 nm, *n*-hexane/2-propanol= 19/1, Flow rate = 1.0 mL/min), t_R = 11.584 min(major), 15.677 min.

HPLC chromatogram of racemic 82

Condition: n-hexane/2-propanol =49:1

Flow rate =1.0 mL/min

λ= 254 nm

Chiral IA-3

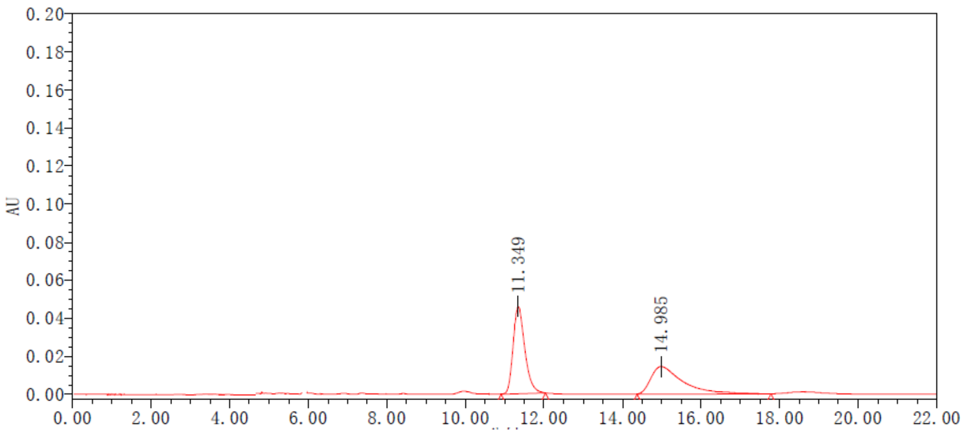

Entry	Retention Time/min	Area	Height	Area(%)
1	11.349	883243	45701	50.08
2	14.985	882743	14418	49.92

Supplementary Figure 173. Chiral HPLC analysis of racemic **82**

HPLC chromatogram of chiral 82

Condition: n-hexane/2-propanol =49:1

Flow rate =1.0 mL/min

λ= 254 nm

Chiral IA-3

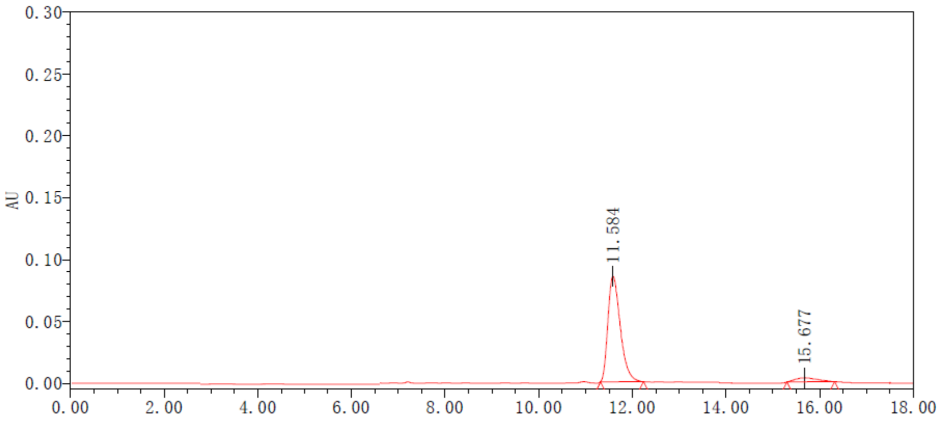

Entry	Retention Time/min	Area	Height	Area(%)
1	11.584	1565221	85549	93.50
2	15.677	108861	3232	6.50

Supplementary Figure 174. Chiral HPLC analysis of chiral **82**

Tert-butyl (2*R*,3*S*)-2-((4-bromobenzyl)oxy)-3-(4-nitrophenyl)-5-oxo-2,5-diphenylpentanoate (83)

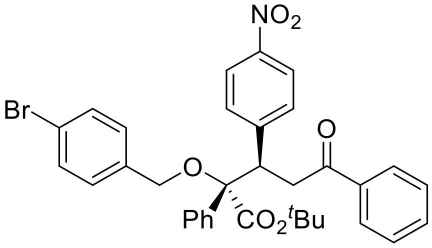

83

Colorless oil; 57.6 mg, 61% yield, >20:1 d.r., 89% *ee,* $[\alpha]_D^{20}$ = -12.45 (c = 0.1, CH_2Cl_2); ^{1}H NMR (400 MHz, $CDCl_3$) δ 8.01 (d, *J* = 8.8 Hz, 2H), 7.71 (d, *J* = 7.3 Hz, 2H), 7.55 – 7.47 (m, 5H), 7.42 (d, *J* = 8.7 Hz, 2H), 7.39 – 7.33 (m, 5H), 7.26 (d, *J* = 7.4 Hz, 2H), 4.86 (d, *J* = 12.4 Hz, 1H), 4.51 (d, *J* = 12.4 Hz, 1H), 4.27 (dd, *J* = 10.0, 3.7 Hz, 1H), 3.53 (qd, *J* = 17.2, 6.9 Hz, 2H), 1.37 (s, 9H). ^{13}C NMR (101 MHz, $CDCl_3$) δ 197.4, 169.5, 147.8, 146.8, 137.9, 136.6, 136.4, 133.2, 131.6, 131.4, 128.6, 128.5, 128.5, 128.4, 127.9, 127.8, 122.6, 121.3, 87.9, 83.5, 67.7, 51.3, 39.6, 28.0. HRMS (ESI) $[M+Na]^+$ calcd for $C_{34}H_{32}NO_6BrNa^+$, 652.1305, found 652.1303. HPLC (Chiral IA, λ= 254 nm, *n*-hexane/2-propanol= 9/1, Flow rate = 1.0 mL/min), t_R = 12.087 min (major), 10.037 min.

HPLC chromatogram of racemic 83

Condition: n-hexane/2-propanol =9:1

Flow rate =1.0 mL/min

λ= 254 nm

Chiral IA

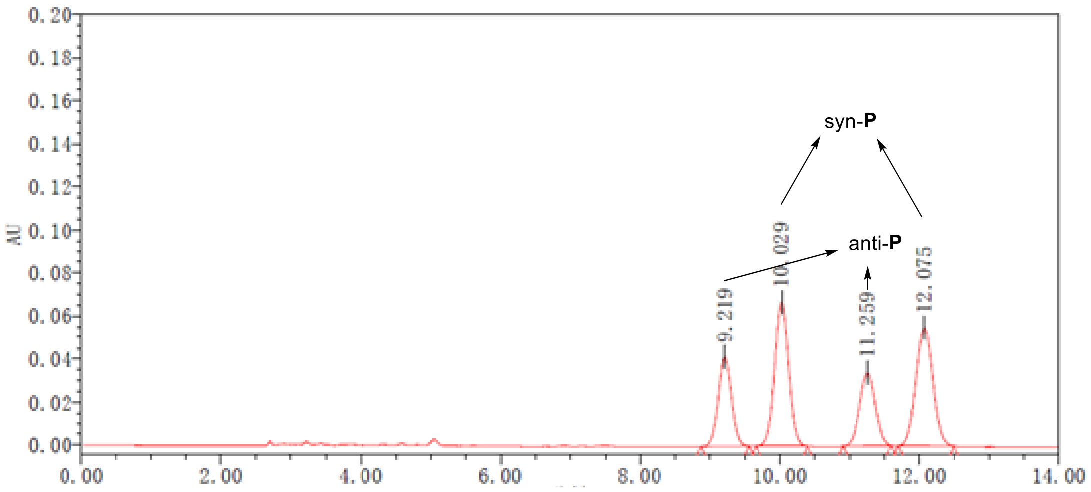

Entry	Retention Time/min	Area	Height	Area(%)
1	9.219	541702	41400	18.58
2	10.029	926669	66778	31.79
3	11.259	527070	33905	18.08
4	12.075	919516	54869	31.54

Supplementary Figure 175. Chiral HPLC analysis of racemic **83**

HPLC chromatogram of chiral 83

Condition: n-hexane/2-propanol =9:1

Flow rate =1.0 mL/min

λ= 254 nm

Chiral IA

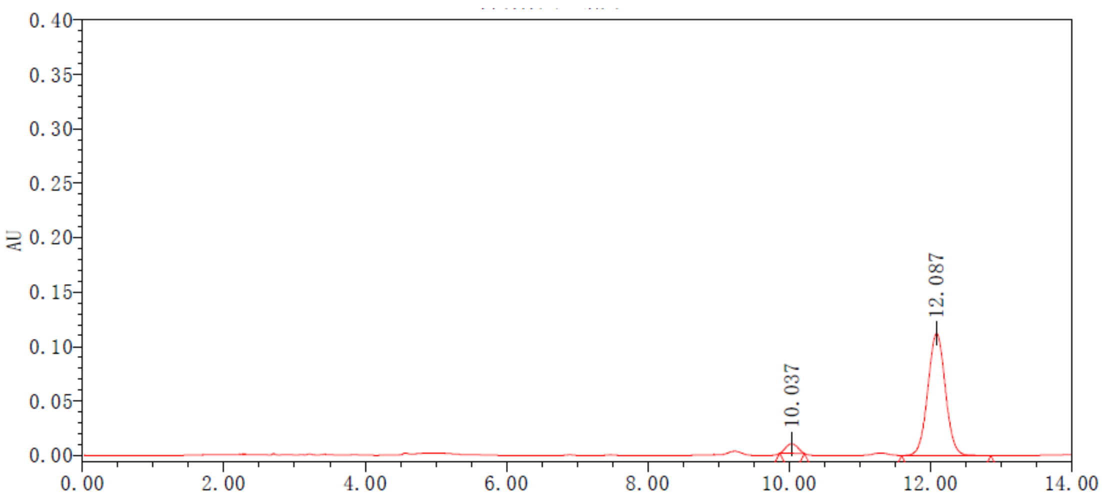

Entry	Retention Time/min	Area	Height	Area(%)
1	10.037	102657	8926	5.51
2	12.087	1924279	112089	94.49

Supplementary Figure 176. Chiral HPLC analysis of chiral **83**

Tert-butyl (2S,3R)-2-((4-bromobenzyl)oxy)-3-(3-methyl-4-nitrophenyl)-5-oxo-2,5-diphenylpentanoate (84)

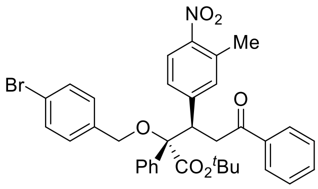

84

Colorless oil; 52.1 mg, 54% yield, >20:1 d.r., 90% *ee*, $[\alpha]_D^{20}$ = -13.78 (c = 0.1, CH_2Cl_2); ^{1}H NMR (500 MHz, $CDCl_3$) δ 7.81 (d, *J* = 8.3 Hz, 1H), 7.73 (d, *J* = 7.5 Hz, 2H), 7.51 (t, *J* = 6.2 Hz, 5H), 7.41 – 7.36 (m, 3H), 7.35 (d, *J* = 1.3 Hz, 2H), 7.27 – 7.25 (m, 2H), 7.21 (d, *J* = 10.1 Hz, 2H), 4.83 (d, *J* = 12.4 Hz, 1H), 4.52 (d, *J* = 12.4 Hz, 1H), 4.20 (dd, *J* = 10.0, 3.4 Hz, 1H), 3.54 (dd, *J* = 17.2, 10.1 Hz, 1H), 3.47 (dd, *J* = 17.2, 3.5 Hz, 1H), 2.49 (s, 3H), 1.37 (s, 9H). ^{13}C NMR (126 MHz, $CDCl_3$) δ 197.5, 169.5, 147.7, 145.8, 137.9, 136.7, 136.5, 135.2, 133.2, 132.7, 131.6, 128.7, 128.6, 128.6, 128.4, 128.3, 127.9, 127.9, 123.9, 121.2, 87.9, 83.3, 67.6, 51.0, 39.4, 28.0, 20.7. HRMS (ESI) $[M+Na]^+$

calcd for $C_{35}H_{34}NO_6BrNa^+$, 666.1462, found 666.1459. HPLC (Chiral IA, λ= 254 nm, *n*-hexane/2-propanol= 19/1, Flow rate = 1.0 mL/min), t_R = 9.344 min, 11.525 min(major).

HPLC chromatogram of racemic 84

Condition: n-hexane/2-propanol =19:1

Flow rate =1.0 mL/min

λ= 254 nm

Chiral IA

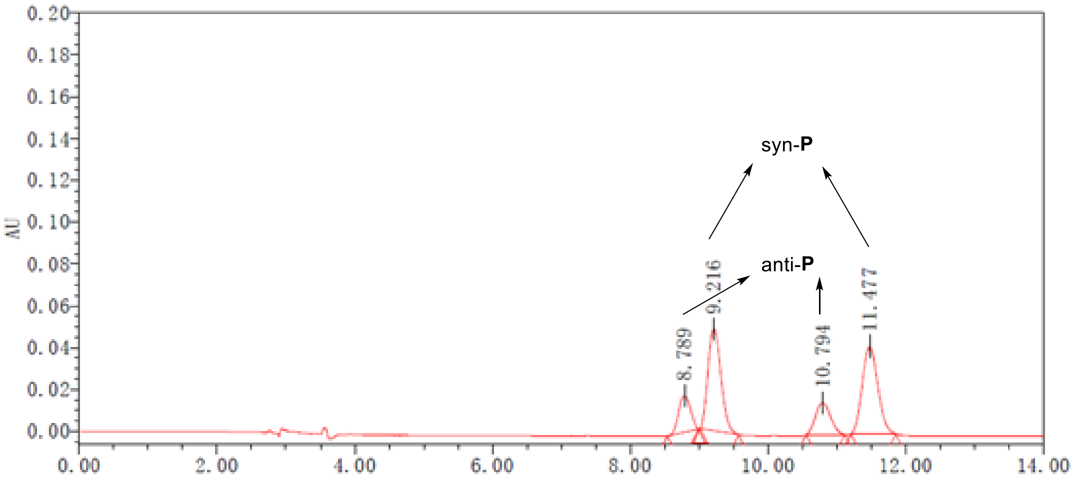

Entry	Retention Time/min	Area	Height	Area(%)
1	8.789	208251	17259	11.94
2	9.216	644543	48750	36.95
3	10.794	230009	15158	13.19
4	11.477	661659	41630	37.93

Supplementary Figure 177. Chiral HPLC analysis of racemic **84**

HPLC chromatogram of chiral 84

Condition: n-hexane/2-propanol =19:1

Flow rate =1.0 mL/min

λ= 254 nm

Chiral IA

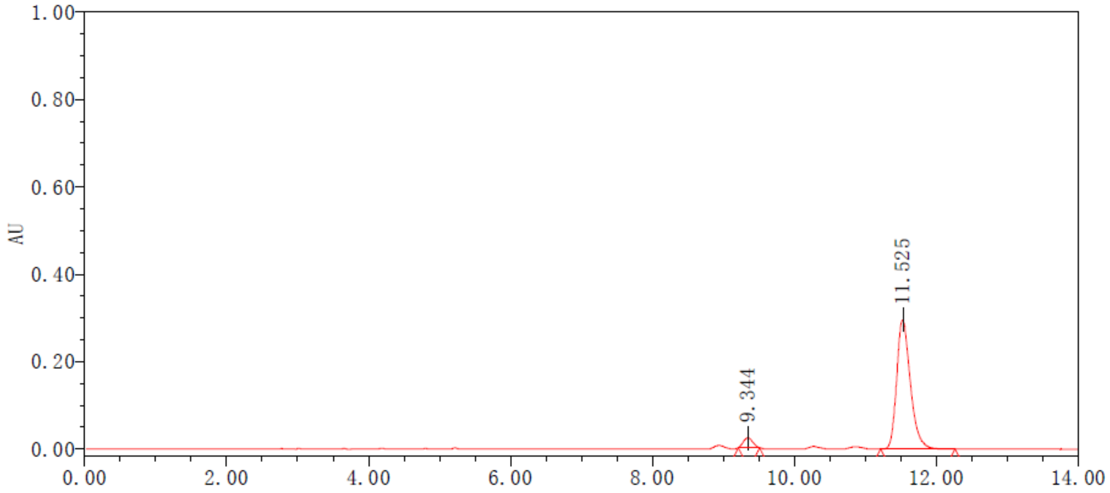

Entry	Retention Time/min	Area	Height	Area(%)
1	9.344	206914	22766	4.94
2	11.525	3978778	295461	95.06

Supplementary Figure 178. Chiral HPLC analysis of chiral **84**

Tert-butyl (2S,3R)-2-(cinnamyloxy)-3-(4-nitrophenyl)-5-oxo-2,5-diphenylpentanoate (85)

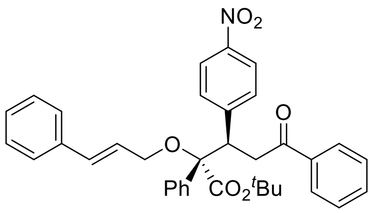

85

Colorless oil; 57.1 mg, 66% yield, >20:1 d.r., 93% *ee*, $[\alpha]_D^{20}$ = -11.09 (c = 0.1, CH_2Cl_2); ^1H NMR (500 MHz, $CDCl_3$) δ 8.03 (d, *J* = 8.7 Hz, 2H), 7.74 (d, *J* = 7.5 Hz, 2H), 7.57 (d, *J* = 7.3 Hz, 2H), 7.50 (dd, *J* = 7.7, 5.8 Hz, 3H), 7.44 (d, *J* = 7.5 Hz, 2H), 7.41 – 7.33 (m, 7H), 7.28 (t, *J* = 7.3 Hz, 1H), 6.69 (d, *J* = 16.0 Hz, 1H), 6.40 (dt, *J* = 15.9, 5.4 Hz,

1H), 4.52 (dd, J = 7.3, 6.1 Hz, 1H), 4.22 (td, J = 10.2, 4.6 Hz, 2H), 3.60 (dd, J = 17.3, 10.6 Hz, 1H), 3.46 (dd, J = 17.2, 3.1 Hz, 1H), 1.37 (s, 9H). ^{13}C NMR (126 MHz, $CDCl_3$) δ 197.6, 169.7, 148.1, 146.8, 136.9, 136.8, 136.7, 133.2, 131.4, 131.2, 128.7, 128.6, 128.3, 128.30, 127.9, 127.8, 127.7, 126.6, 126.5, 122.6, 87.8, 83.2, 67.7, 51.5, 39.6, 28.0. HRMS (ESI) [M+Na]$^+$ calcd for $C_{36}H_{35}NO_6Na^+$, 600.2357, found 600.2358. HPLC (Chiral IE-3, λ= 254 nm, *n*-hexane/2-propanol= 17/3, Flow rate = 1.0 mL/min), t_R = 9.798 min, 16.248 min(major).

HPLC chromatogram of racemic 85

Condition: n-hexane/2-propanol =17:3

Flow rate =1.0 mL/min

λ= 254 nm

Chiral IE-3

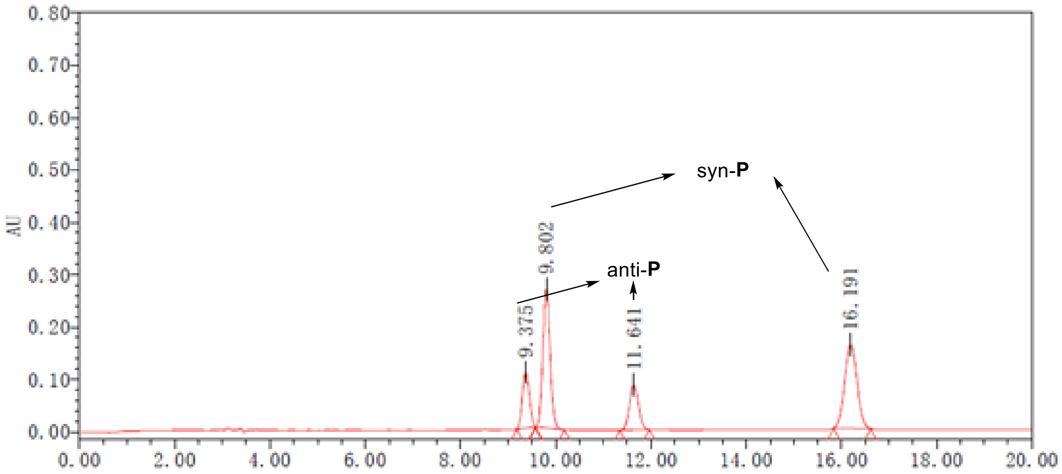

Entry	Retention Time/min	Area	Height	Area(%)
1	9.375	1070069	106241	13.49
2	9.802	2859038	264703	36.03
3	11.641	1108093	85549	13.97
4	16.191	2897132	159691	36.51

Supplementary Figure 179. Chiral HPLC analysis of racemic **85**

HPLC chromatogram of chiral 85

Condition: n-hexane/2-propanol =17:3

Flow rate =1.0 mL/min

λ= 254 nm

Chiral IE-3

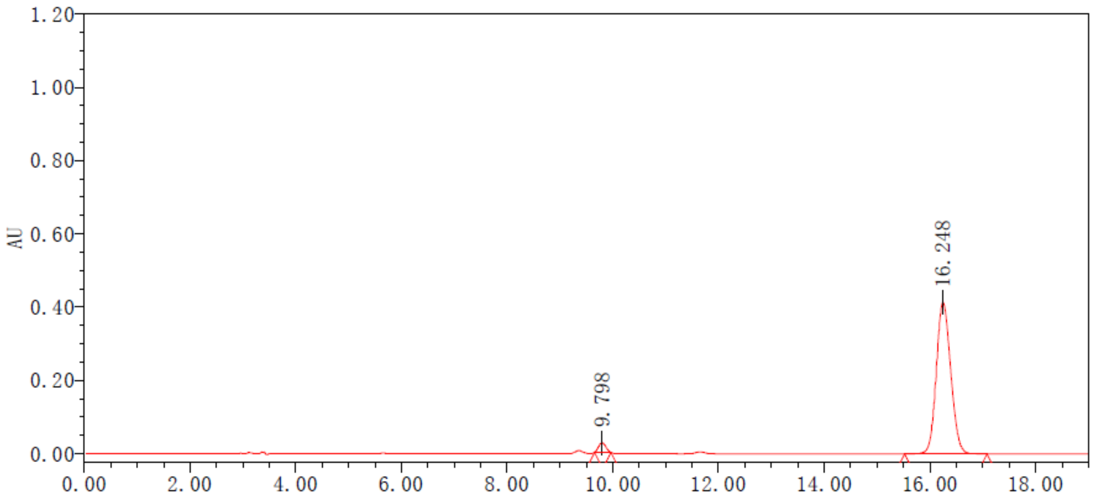

Entry	Retention Time/min	Area	Height	Area(%)
1	9.798	255068	26006	3.47
2	16.248	7853260	413978	96.53

Supplementary Figure 180. Chiral HPLC analysis of chiral **85**

Tert-butyl (2S,3R)-2-(furan-2-ylmethoxy)-3-(4-nitrophenyl)-5-oxo-2,5-diphenylpentanoate (86)

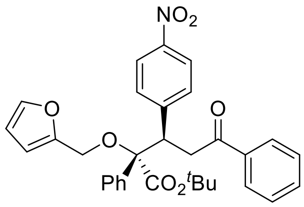

86

Colorless oil; 52.7 mg, 65% yield, >20:1 d.r., 93% *ee*, $[\alpha]_D^{20}$ = -14.12 (c = 0.1, CH_2Cl_2); ^{1}H NMR (400 MHz, $CDCl_3$) δ 8.06 – 7.95 (m, 2H), 7.73 – 7.63 (m, 4H), 7.54 – 7.49

(m, 3H), 7.49 – 7.45 (m, 1H), 7.43 – 7.31 (m, 5H), 6.48 – 6.30 (m, 2H), 4.95 (d, *J* = 12.4 Hz, 1H), 4.55 (d, *J* = 12.4 Hz, 1H), 4.15 (dd, *J* = 10.5, 3.2 Hz, 1H), 3.54 (dd, *J* = 17.3, 10.5 Hz, 1H), 3.41 (dd, *J* = 17.3, 3.2 Hz, 1H), 1.36 (s, 9H). ^{13}C NMR (126 MHz, $CDCl_3$) δ 197.7, 169.6, 152.3, 148.3, 146.8, 142.7, 136.7, 136.6, 133.1, 131.4, 128.5, 128.4, 128.4, 127.9, 127.7, 122.5, 110.4, 108.6, 87.6, 83.4, 61.3, 51.5, 39.5, 27.9. HRMS (ESI) $[M+Na]^+$ calcd for $C_{32}H_{31}NO_7Na^+$, 564.1993, found 564.1996. (Chiral IA, λ= 254 nm, *n*-hexane/2-propanol= 49/1, Flow rate = 1.0 mL/min), t_R = 16.664 min, 24.094 min(major)

HPLC chromatogram of racemic 86

Condition: n-hexane/2-propanol =49:1

Flow rate =1.0 mL/min

λ= 254 nm

Chiral IA

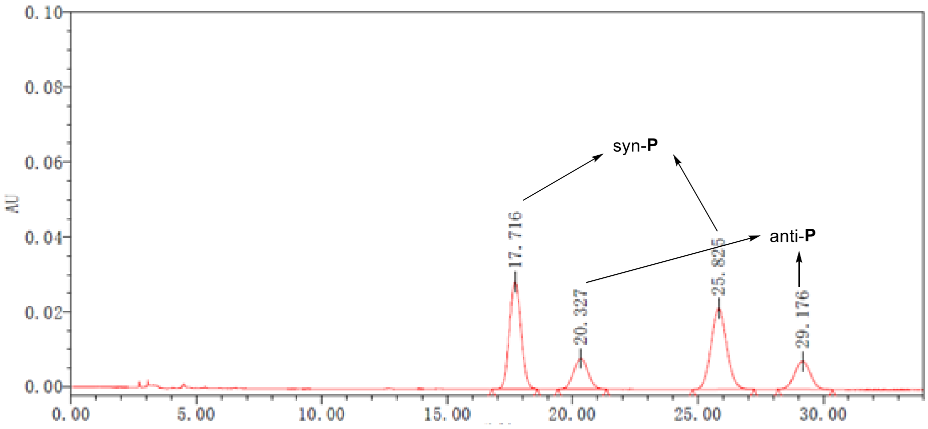

Entry	Retention Time/min	Area	Height	Area(%)
1	17.716	932158	28631	36.63
2	20.327	335866	8223	13.20
3	25.825	944625	21582	37.12
4	29.176	332399	7539	13.06

Supplementary Figure 181. Chiral HPLC analysis of racemic **86**

HPLC chromatogram of chiral 86

Condition: n-hexane/2-propanol =49:1

Flow rate =1.0 mL/min

λ= 254 nm

Chiral IA

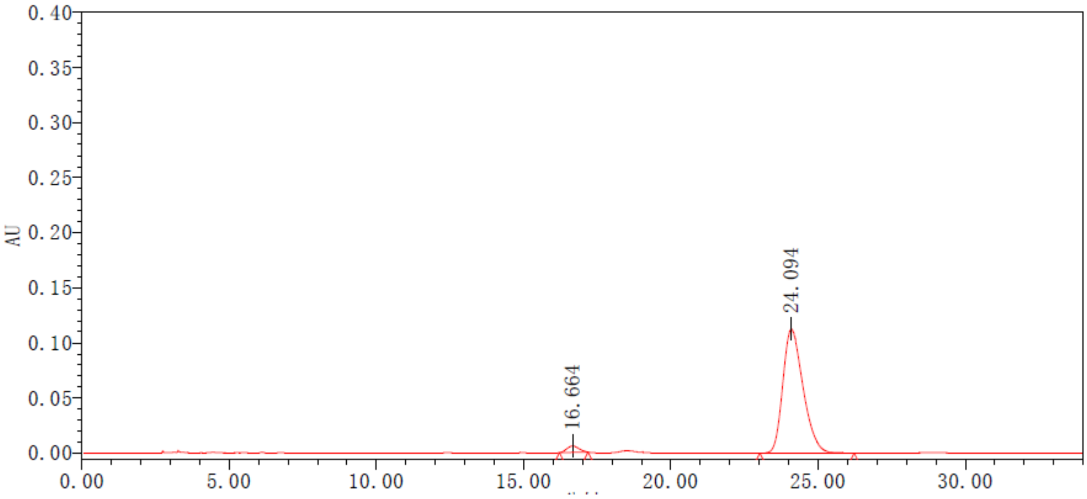

Entry	Retention Time/min	Area	Height	Area(%)
1	16.664	168212	5826	3.53
2	24.094	5381329	112554	96.47

Supplementary Figure 182. Chiral HPLC analysis of chiral **86**

2-(Cyclohexylamino)-1-(4-(hydroxymethyl)phenyl)-2-oxoethyl benzoate (racemic-1G)

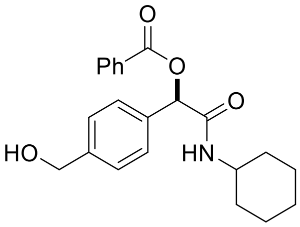

1G

White solid; 52% yield (572.5 mg). ^{1}H NMR (400 MHz, $CDCl_3$) δ 8.12 – 8.05 (m, 2H), 7.61 (t, *J* = 7.4 Hz, 1H), 7.50 (d, *J* = 4.9 Hz, 1H), 7.48 (d, *J* = 4.4 Hz, 2H), 7.44 (d, *J* =

14.9 Hz, 1H), 7.36 (d, *J* = 8.1 Hz, 2H), 6.28 (s, 1H), 6.15 (d, *J* = 8.1 Hz, 1H), 4.66 (s, 2H), 3.82 (ddd, *J* = 21.0, 11.3, 7.2 Hz, 1H), 2.33 (s, 1H), 1.94 – 1.86 (m, 2H), 1.67 (t, *J* = 11.9 Hz, 2H), 1.62 – 1.57 (m, 2H), 1.38 – 1.32 (m, 2H), 1.18 – 1.13 (m, 2H). ^{13}C NMR (101 MHz, $CDCl_3$) δ 167.4, 165.0, 142.0, 135.0, 133.7, 129.8, 129.3, 128.7, 127.6, 127.3, 126.1, 75.7, 64.8, 48.3, 32.9, 32.8, 25.4, 24.7, 24.7. HRMS (ESI) $[M+H]^+$ calcd for $C_{22}H_{26}O_4^+$, 368.1856, found 368.1856.

1-(4-((((2S,3R)-1-(tert-butoxy)-1-oxo-3-(2-oxo-2-phenylethyl)-2,5-diphenylpent-4-yn-2-yl)oxy)methyl)phenyl)-2-(cyclohexylamino)-2-oxoethyl benzoate (87)

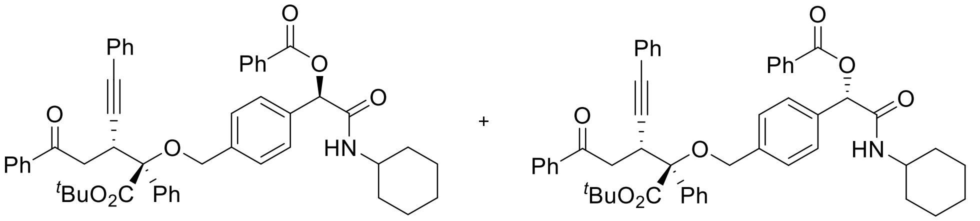

***syn*-87** ***syn*-87'**

White solid; 65% yield (25.6 mg), 1:1 d.r., 92% ee, 92% ee. Composite NMR signals of *syn*-**87** and *syn*-**87'** (All spectral peaks of two diasteroisomers *syn*-**87** and *syn*-**87'** are overlapping in ^{1}H NMR and ^{13}C NMR). ^{1}H NMR (400 MHz, $CDCl_3$) δ 8.09 (d, *J* = 8.2 Hz, 2H), 7.85 (d, *J* = 7.3 Hz, 2H), 7.70 (d, *J* = 7.5 Hz, 2H), 7.58 (d, *J* = 7.3 Hz, 2H), 7.54 (d, *J* = 5.1 Hz, 2H), 7.51 – 7.43 (m, 5H), 7.41 – 7.33 (m, 6H), 7.22 (d, *J* = 2.9 Hz, 3H), 6.32 (s, 1H), 6.11 (d, *J* = 8.1 Hz, 1H), 5.13 (d, *J* = 11.6 Hz, 1H), 4.96 (d, *J* = 11.7 Hz, 1H), 4.41 (dd, *J* = 10.2, 2.8 Hz, 1H), 3.85 – 3.79 (m, 1H), 3.36 (dd, *J* = 16.6, 10.2 Hz, 1H), 3.11 (dd, *J* = 16.6, 2.9 Hz, 1H), 1.93 (dd, *J* = 32.9, 16.4 Hz, 2H), 1.67 (dd, *J* = 13.2, 11.1 Hz, 4H), 1.51 (s, 9H), 1.32 (dd, *J* = 10.9, 5.1 Hz, 2H), 1.16 (dd, *J* = 16.2, 7.9 Hz, 2H). ^{13}C NMR (101 MHz, $CDCl_3$) δ 197.5, 169.4, 167.4, 165.0, 139.9, 138.4, 136.9, 134.9, 133.7, 133.1, 131.4, 129.8, 128.7, 128.6, 128.5, 128.3, 128.2, 128.2, 127.8, 127.6, 127.5, 127.5, 127.3, 126.8, 123.4, 89.8, 85.8, 84.1, 82.9, 75.9, 70.6, 68.2, 48.3, 39.2, 33.0, 32.9, 28.1, 27.9, 25.5, 24.8, 24.7. HRMS (ESI) $[M+Na]^+$ calcd for

$C_{51}H_{51}NO_7Na^+$, 812.3558, found 812.3562. (Chiral IC, λ= 254 nm, *n*-hexane/2-propanol= 4/1, Flow rate = 1.0 mL/min), t_R = 17.339 min (major), 24.262 min; $t_{R'}$ = 31.982 min (major), 45.486 min.

HPLC chromatogram of racemic 87

Condition: n-hexane/2-propanol =4:1

Flow rate =1.0 mL/min

λ= 254.0 nm

Chiral IC

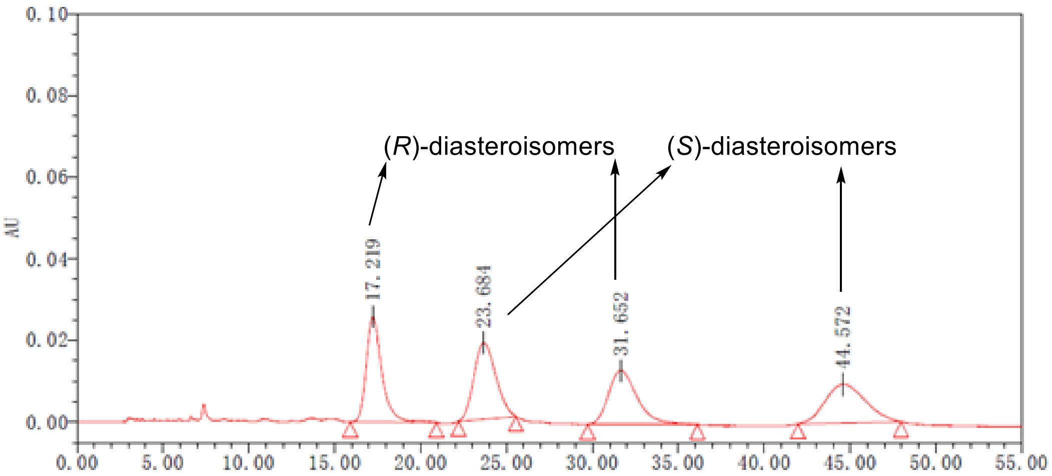

Entry	Retention Time/min	Area	Height	Area(%)
1	17.219	1645637	25910	25.86
2	23.684	1657749	18758	26.05
3	31.652	1524635	13187	23.96
4	44.572	1535964	9468	24.14

Supplementary Figure 183. Chiral HPLC analysis of racemic **87**

HPLC chromatogram of chiral 87 and 87’

Condition: n-hexane/2-propanol =4:1

Flow rate =1.0 mL/min

λ= 254.0 nm

Chiral IC

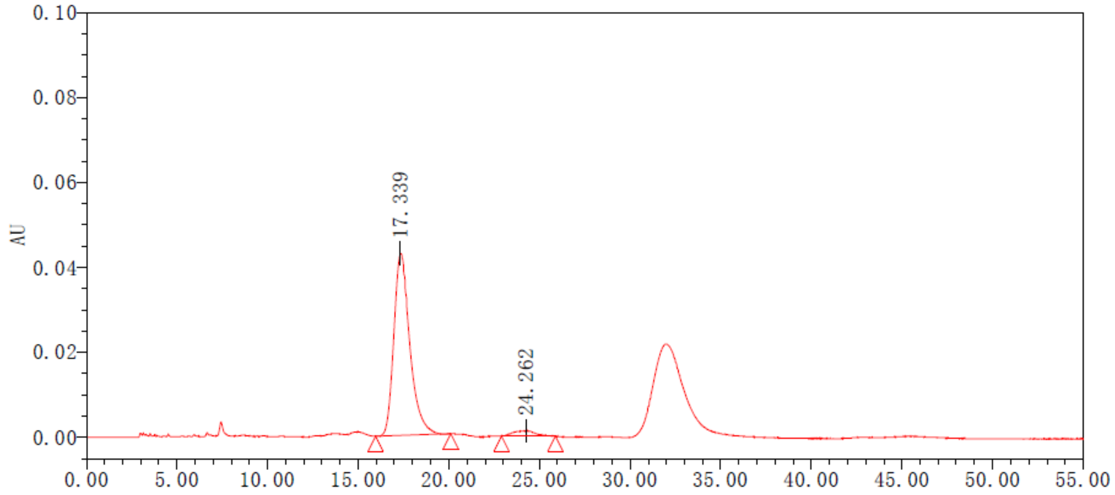

Entry	Retention Time/min	Area	Height	Area(%)
1	17.339	2641026	42907	96.09
2	24.262	107348	1285	3.91

Supplementary Figure 184. Chiral HPLC analysis of chiral **87**

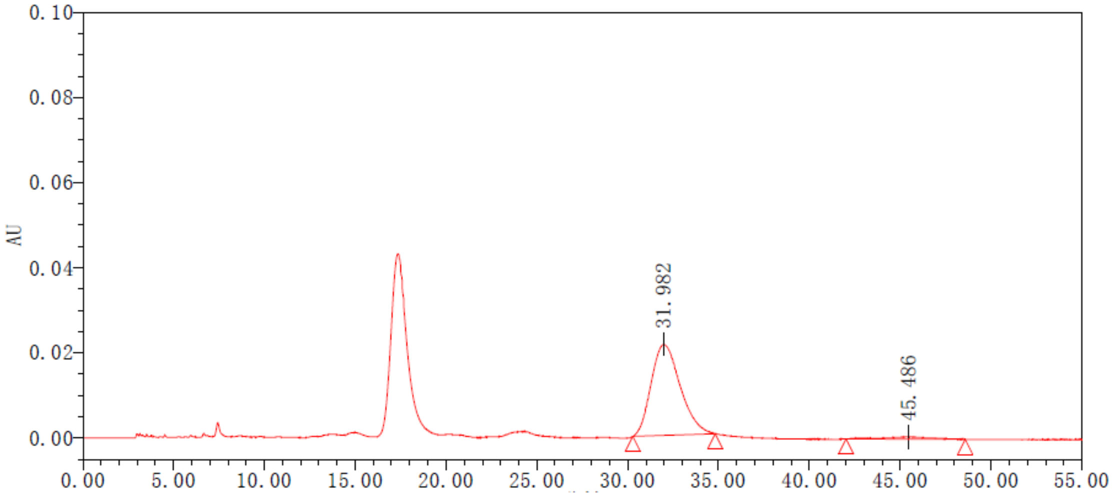

The larger version of HPLC chromatogram of chiral 87'

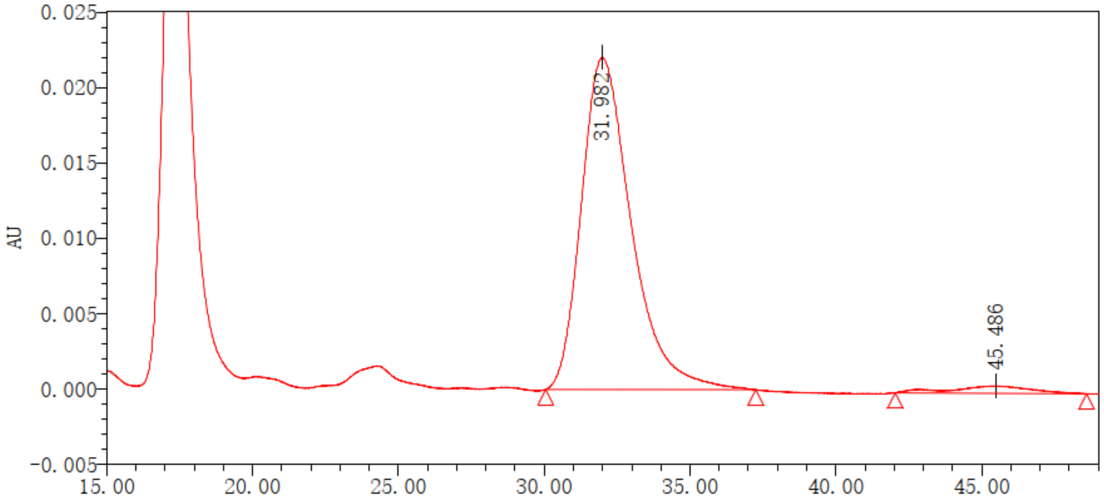

Entry	Retention Time/min	Area	Height	Area(%)
1	31.982	2381648	21429	96.25
2	45.486	92669	485	3.75

Supplementary Figure 185. Chiral HPLC analysis of chiral **87'**

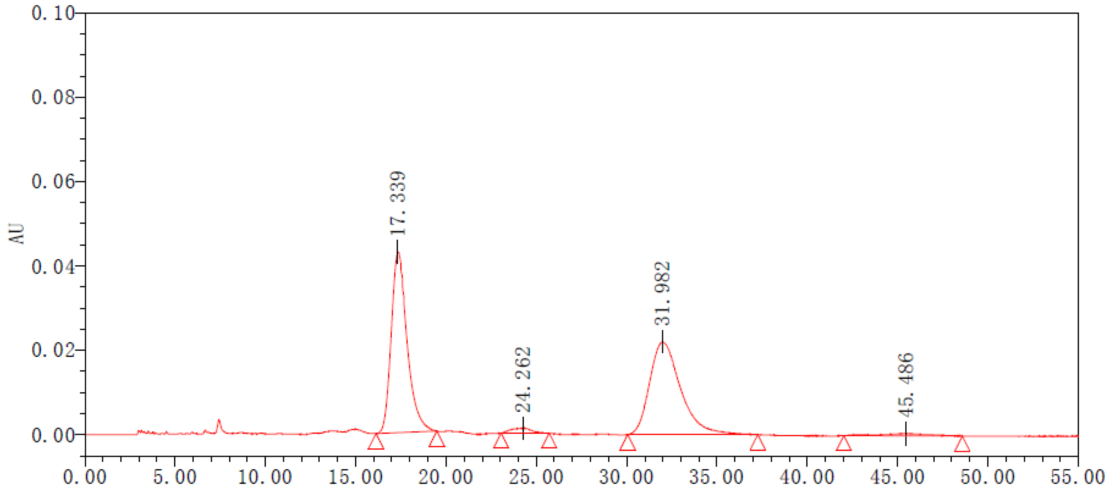

The larger version of HPLC chromatogram of chiral chiral 87 and 87’

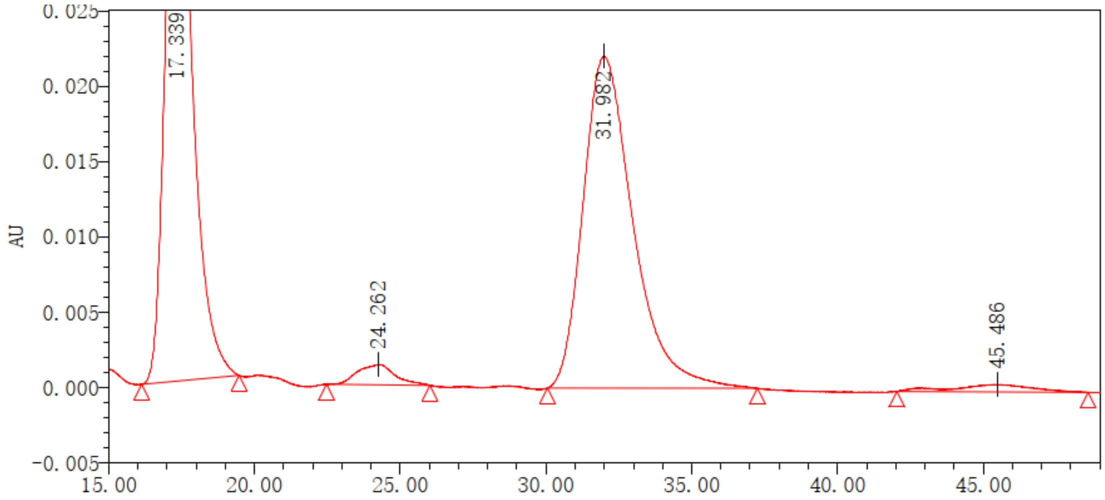

Entry	Retention Time/min	Area	Height	Area(%)
1	17.339	2631702	42882	48.36
2	24.262	93357	1201	1.72
3	31.982	2624189	22021	48.22
4	45.486	92669	485	1.70

Supplementary Figure 186. Chiral HPLC analysis of chiral **87 and 87’**

2-((4-(Hydroxymethyl)phenyl)(morpholino)methyl)phenol (racemic-1I)

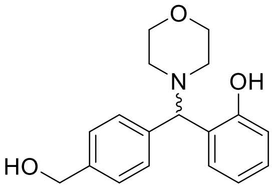

1l

Colorless oil; 70% yield (210 mg). ^{1}H NMR (400 MHz, $CDCl_3$) δ 11.72 (s, 1H), 7.39 (d, *J* = 7.8 Hz, 2H), 7.27 (d, *J* = 8.3 Hz, 2H), 7.12 – 7.05 (m, 1H), 6.91 (dd, *J* = 7.6, 1.3 Hz, 1H), 6.83 (dd, *J* = 8.1, 0.7 Hz, 1H), 6.69 (td, *J* = 7.5, 1.0 Hz, 1H), 4.58 (s, 2H), 4.38 (s, 1H), 3.69 (dd, *J* = 10.9, 6.0 Hz, 4H), 2.62 – 2.35 (m, 4H). ^{13}C NMR (101 MHz, $CDCl_3$) δ 156.0, 141.0, 138.6, 129.3, 128.8, 127.6, 124.8, 119.7, 117.1, 76.5, 66.9, 64.7, 52.2. HRMS (ESI) $[M+H]^+$ calcd for $C_{18}H_{22}NO_3^+$, 300.1594, found 300.1593.

Tert-butyl (2S,3R)-2-((4-((R)-(2-hydroxyphenyl)(morpholino)methyl)benzyl)oxy)-3-(2-oxo-2-phenylethyl)-2,5-diphenylpent-4-ynoate (*syn*-88)

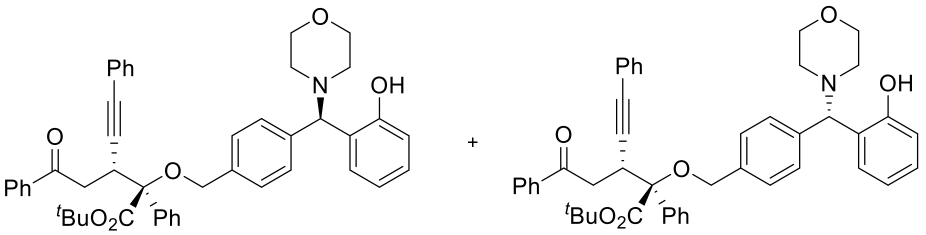

***syn*-88** ***syn*-88'**

White solid; 74% yield (26.7 mg), 1:1 d.r., 98% ee, 98% ee. Composite NMR signals of *syn*-**88** and *syn*-**88’** (All spectral peaks of two diasteroisomers *syn*-**88** and *syn*-**88’** are overlapping in ^{1}H NMR and ^{13}C NMR). ^{1}H NMR (500 MHz, $CDCl_3$) δ 11.69 (s, 1H), 7.84 (d, *J* = 7.5 Hz, 2H), 7.69 (d, *J* = 7.7 Hz, 2H), 7.51 (t, *J* = 6.9 Hz, 1H), 7.45 (t, *J* = 8.8 Hz, 3H), 7.40 – 7.33 (m, 6H), 7.30 (d, *J* = 7.2 Hz, 1H), 7.24 (d, *J* = 6.6 Hz, 1H), 7.23 (d, *J* = 7.7 Hz, 3H), 7.12 (t, *J* = 7.7 Hz, 1H), 6.95 (d, *J* = 6.9 Hz, 1H), 6.86 (d, *J* = 8.1 Hz, 1H), 6.73 (t, *J* = 7.4 Hz, 1H), 5.06 (d, *J* = 11.7 Hz, 1H), 4.91 (d, *J* = 11.6 Hz, 1H), 4.42 (s, 1H), 4.41 (dd, *J* = 11.4, 4.0 Hz, 1H), 3.75 (d, *J* = 3.9 Hz, 4H), 3.33 (dd, *J* = 16.7, 10.3 Hz, 1H), 3.17 – 3.06 (m, 1H), 2.54 (d, *J* = 61.0 Hz, 4H), 1.50 (s, 9H). ^{13}C NMR (126 MHz, $CDCl_3$) δ 197.4, 169.4, 156.1, 139.0, 138.4, 136.9, 133.1, 131.5, 131.4, 129.4, 128.7, 128.5, 128.5, 128.3, 128.2, 128.2, 128.2, 128.1, 128.1, 128.0, 127.9, 126.8, 124.9, 123.4, 119.6, 117.1, 89.7, 85.8, 84.1, 82.9, 76.6, 68.2, 66.9, 39.3, 36.5, 28.1.

HRMS (ESI) $[M+Na]^+$ calcd for $C_{47}H_{47}NO_6Na^+$, 744.3296, found 744.3298. (Chiral IA-3, λ= 254 nm, *n*-hexane/2-propanol= 17/3, Flow rate = 1.0 mL/min), t_R = 7.732 min (major), 12.434 min; $t_{R'}$ = 8.782 min (major), 15.130 min.

HPLC chromatogram of racemic 88

Condition: n-hexane/2-propanol =17:3

Flow rate =1.0 mL/min

λ= 254.0 nm

Chiral IA-3

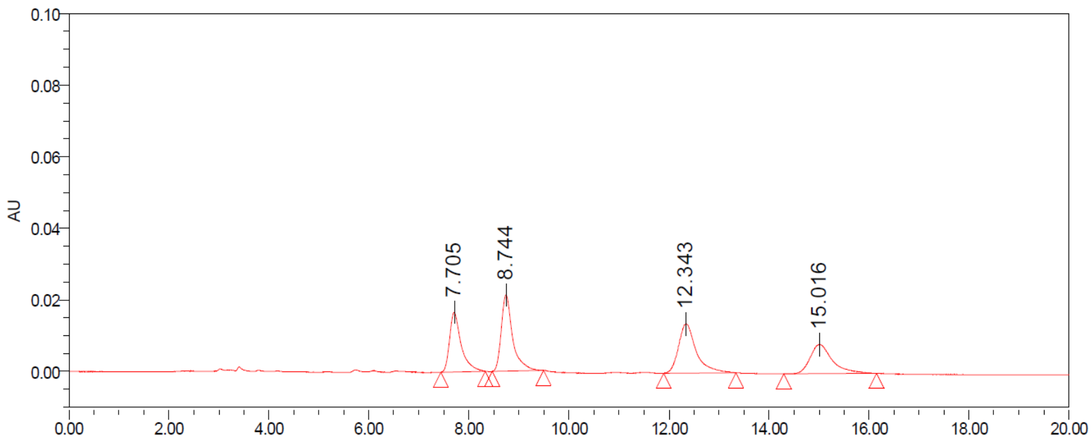

Entry	Retention Time/min	Area	Height	Area(%)
1	7.705	267422	16613	22.37
2	8.744	337897	21296	28.26
3	12.343	336987	13741	28.19
4	15.016	253249	8181	21.18

Supplementary Figure 187. Chiral HPLC analysis of racemic **88**

HPLC chromatogram of chiral 88 and 88'

Condition: n-hexane/2-propanol =17:3

Flow rate =1.0 mL/min

λ= 254.0 nm

Chiral IA-3

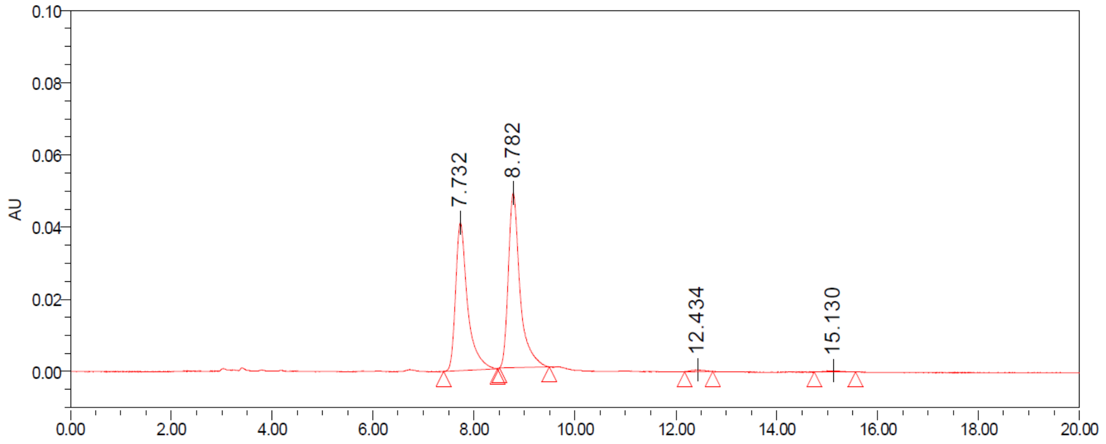

The larger version of HPLC chromatogram of chiral 88 and 88'

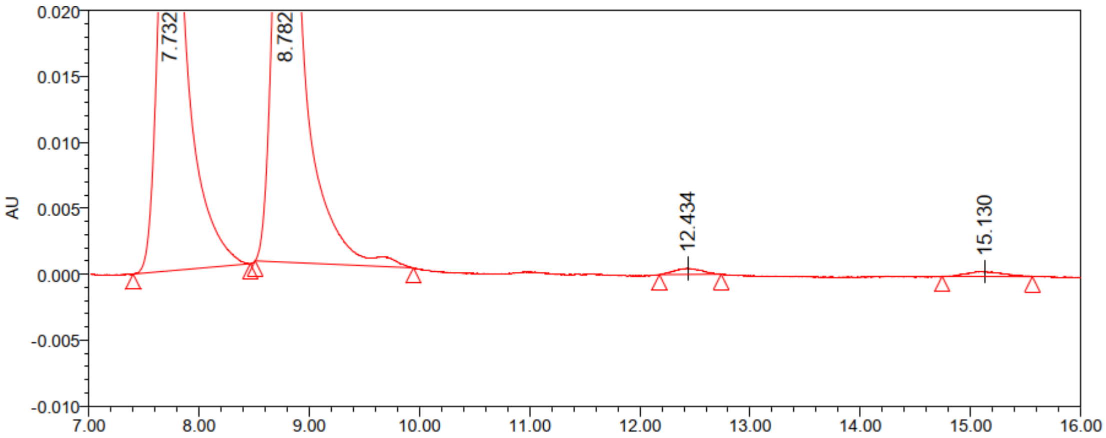

Entry	Retention Time/min	Area	Height	Area(%)
1	7.732	675372	40954	45.96
2	8.782	777546	48362	52.92
3	12.434	7977	464	0.54
4	15.130	8465	365	0.58

Supplementary Figure 188. Chiral HPLC analysis of chiral **88 and 88'**

9-(4-(Hydroxymethyl)phenyl)-3,3,6,6-tetramethyl-3,4,6,7,9,10-hexahydroacridine-1,8(2H,5H)-dione(1J)

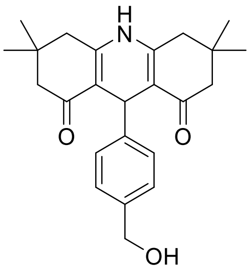

1J

Colorless oil; 90% yield (68.2 mg).^{1}H NMR (400 MHz, $CDCl_3$) δ 11.89 (s, 1H), 7.23 (d, *J* = 8.1 Hz, 2H), 7.05 (d, *J* = 7.9 Hz, 2H), 5.50 (s, 1H), 5.26 (s, 1H), 4.56 (s, 2H), 2.37 (dq, *J* = 35.3, 17.8 Hz, 8H), 1.21 (s, 6H), 1.08 (s, 6H). ^{13}C NMR (101 MHz, $CDCl_3$) δ 190.6, 189.5, 138.7, 137.3, 127.0, 126.9, 115.6, 64.7, 47.0, 46.4, 32.6, 31.4, 29.6, 27.4. HRMS (ESI) $[M+Na]^+$ calcd for $C_{24}H_{29}NO_3Na^+$, 402.2040, found 402.2040.

Tert-butyl (2S,3R)-3-(2-oxo-2-phenylethyl)-2,5-diphenyl-2-((4-(3,3,6,6-tetramethyl-1,8-dioxo-1,2,3,4,5,6,7,8,9,10-decahydroacridin-9-yl)benzyl)oxy)pent-4-ynoate (*syn*-89)

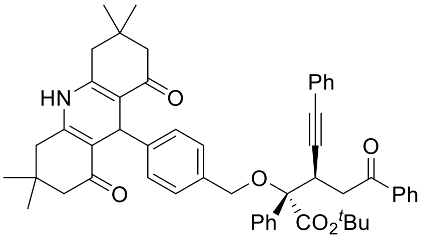

syn-**89**

Colorless oil; 64% yield (25.6 mg), 10:1 d.r., 96% ee. ^{1}H NMR (400 MHz, $CDCl_3$) δ 11.96 (s, 1H), 7.86 (d, *J* = 7.5 Hz, 2H), 7.71 (d, *J* = 7.5 Hz, 2H), 7.52 (t, *J* = 7.3 Hz, 1H), 7.45 – 7.39 (m, 4H), 7.38 (d, *J* = 4.0 Hz, 1H), 7.35 (d, *J* = 7.8 Hz, 2H), 7.30 (d, *J* = 7.4 Hz, 1H), 7.23 (d, *J* = 2.5 Hz, 4H), 7.10 (d, *J* = 8.1 Hz, 2H), 5.54 (s, 1H), 5.08 (d, *J* = 11.4 Hz, 1H), 4.94 (d, *J* = 11.4 Hz, 1H), 4.39 (dd, *J* = 10.3, 2.7 Hz, 1H), 3.37 (dd, *J* = 16.7, 10.3 Hz, 1H), 3.09 (dd, *J* = 16.7, 2.6 Hz, 1H), 2.51 – 2.27 (m, 8H), 1.50 (s, 9H), 1.24 (s, 6H), 1.10 (s, 6H). ^{13}C NMR (101 MHz, $CDCl_3$) δ 197.6, 190.5, 189.4, 169.5, 138.6, 137.2, 136.9, 136.5, 133.1, 131.5, 131.4, 128.5, 128.5, 128.3, 128.2, 128.2, 128.2, 128.1, 127.8, 127.7, 127.5, 126.8, 123.5, 115.7, 115.6, 89.9, 85.7, 84.0, 82.8, 68.3, 47.1, 46.5, 39.2, 36.6, 32.6, 31.4, 29.7, 28.1, 27.4. HRMS (ESI) $[M+Na]^+$ calcd for $C_{53}H_{55}NO_6Na^+$, 802.4102, found 802.4104. (Chiral IA-3, λ= 254 nm, *n*-hexane/2-propanol= 4/1, Flow rate = 1.0 mL/min), t_R = 11.547 min (major), 20.882 min.

HPLC chromatogram of racemic 89

Condition: n-hexane/2-propanol =4:1

Flow rate =1.0 mL/min

λ= 254 nm

Chiral IA-3

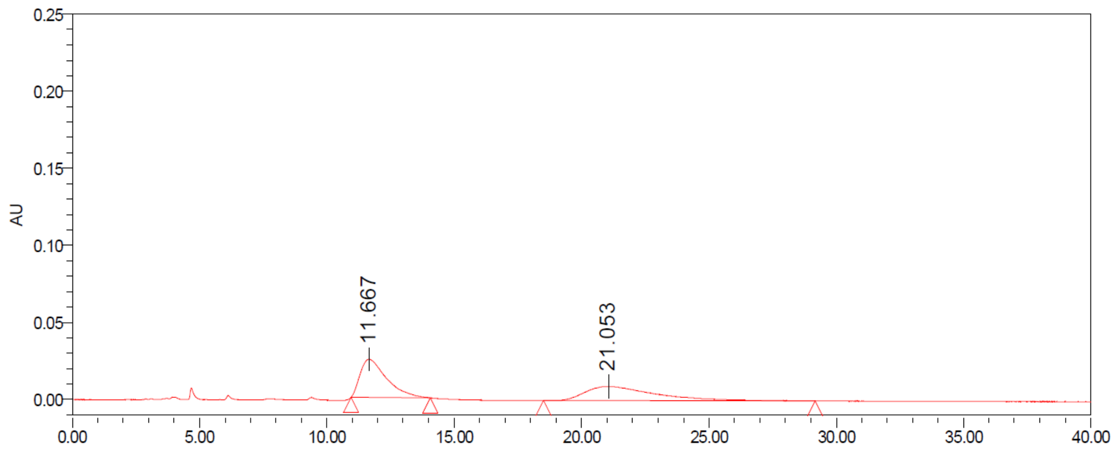

Entry	Retention Time/min	Area	Height	Area(%)
1	11.667	1802681	24754	50.56
2	21.053	1762595	9139	49.44

Supplementary Figure 189. Chiral HPLC analysis of racemic **89**

HPLC chromatogram of chiral 89

Condition: n-hexane/2-propanol =4:1

Flow rate =1.0 mL/min

λ= 254 nm

Chiral IA-3

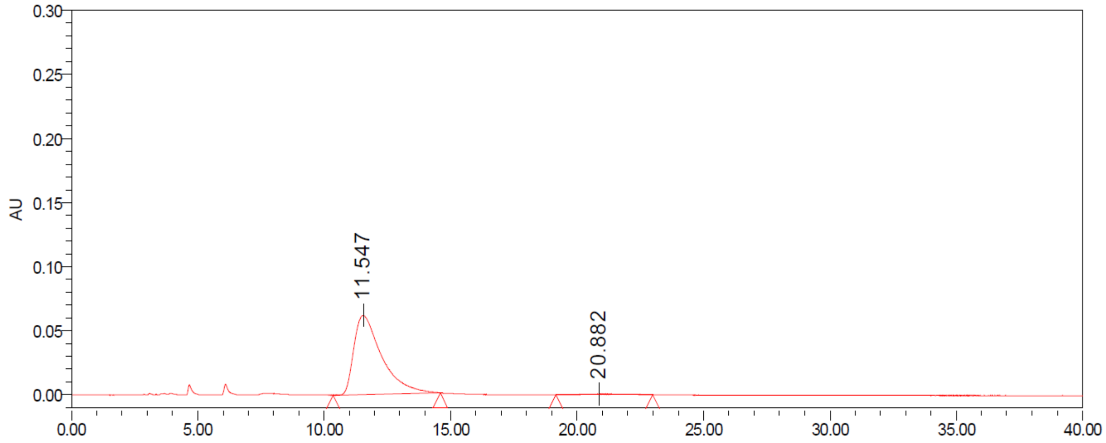

The larger version of HPLC chromatogram of chiral 89

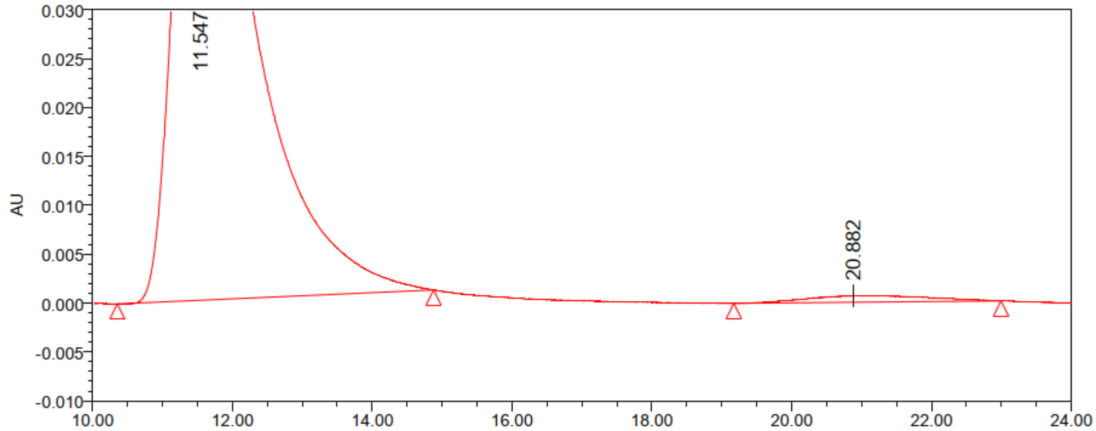

Entry	Retention Time/min	Area	Height	Area(%)
1	11.547	4709094	61522	98.33
2	20.882	79826	675	1.67

Supplementary Figure 190. Chiral HPLC analysis of chiral **89**

Diethyl 4,4'-(5-((((2S,3R)-1-(tert-butoxy)-1-oxo-3-(2-oxo-2-phenylethyl)-2,5-diphenylpent-4-yn-2-yl)oxy)methyl)-2-((E)-3-(tert-butoxy)-3-oxoprop-1-en-1-yl)-1,3-phenylene)dibutyrate (*syn*-90)

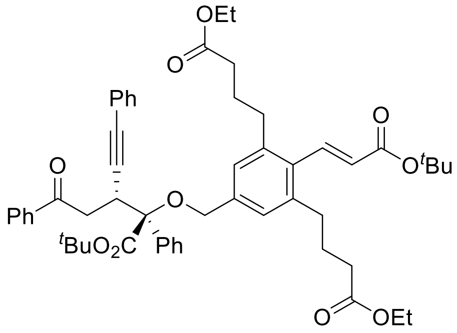

syn-**90**

Colorless oil; 53% yield (25.3 mg), 92:8 d.r., 98% ee. ^{1}H NMR (400 MHz, $CDCl_3$) δ 7.86 (d, *J* = 7.5 Hz, 2H), 7.75 (d, *J* = 9.8 Hz, 1H), 7.71 (d, *J* = 7.6 Hz, 2H), 7.52 (t, *J* = 7.1 Hz, 1H), 7.43 – 7.36 (m, 4H), 7.32 (d, *J* = 7.2 Hz, 1H), 7.27 – 7.19 (m, 7H), 5.91 (d, *J* = 16.3 Hz, 1H), 5.05 (d, *J* = 11.6 Hz, 1H), 4.90 (d, *J* = 11.6 Hz, 1H), 4.42 (dd, *J* = 10.1, 2.6 Hz, 1H), 4.10 (q, *J* = 7.1 Hz, 4H), 3.35 (dd, *J* = 16.6, 10.2 Hz, 1H), 3.13 (dd, *J* = 20.6, 4.2 Hz, 1H), 2.68 – 2.61 (m, 4H), 2.29 (t, *J* = 7.4 Hz, 4H), 1.90 – 1.80 (m, 4H), 1.55 (s, 9H), 1.52 (s, 9H), 1.22 (t, *J* = 7.1 Hz, 6H). ^{13}C NMR (101 MHz, $CDCl_3$) δ 197.5, 173.3, 169.4, 165.7, 142.0, 139.9, 138.5, 137.0, 133.4, 133.1, 131.5, 131.4, 128.5, 128.5, 128.2, 128.2, 128.2, 127.8, 126.8, 126.5, 89.8, 85.8, 84.1, 82.8, 80.7, 68.3, 60.3, 39.3, 36.5, 33.9, 33.0, 28.2, 28.1, 26.1, 14.3. HRMS (ESI) $[M+Na]^+$ calcd for $C_{55}H_{64}O_{10}Na^+$, 907.4392, found 907.4392. (Chiral IC, λ= 254 nm, *n*-hexane/2-propanol= 9/1, Flow rate = 1.0 mL/min), t_R = 17.577 min (major), 29.700 min.

HPLC chromatogram of racemic 90

Condition: n-hexane/2-propanol =9:1

Flow rate =1.0 mL/min

λ= 254.0 nm

Chiral IC

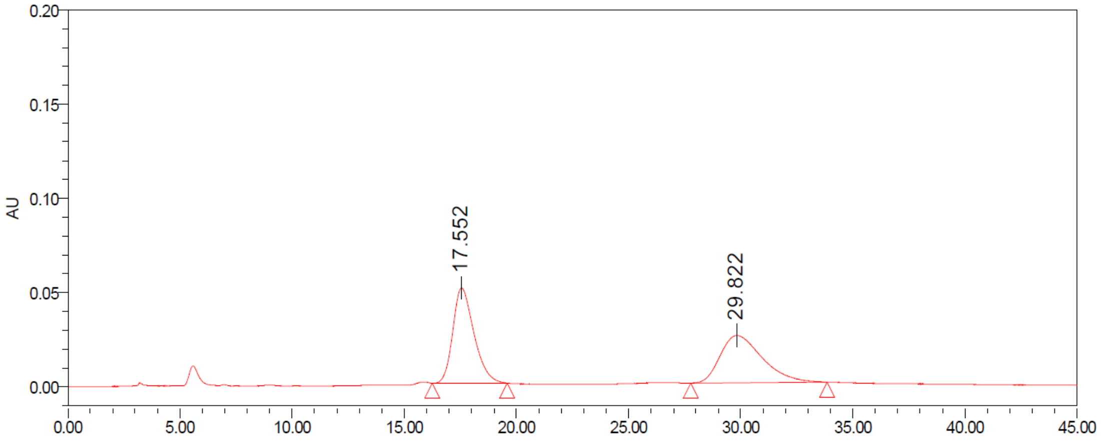

Entry	Retention Time/min	Area	Height	Area(%)
1	17.552	3321391	50547	50.36
2	29.822	3273941	25125	49.64

Supplementary Figure 191. Chiral HPLC analysis of racemic **90**

HPLC chromatogram of chiral 90

Condition: n-hexane/2-propanol =9:1

Flow rate =1.0 mL/min

λ= 254.0 nm

Chiral IC

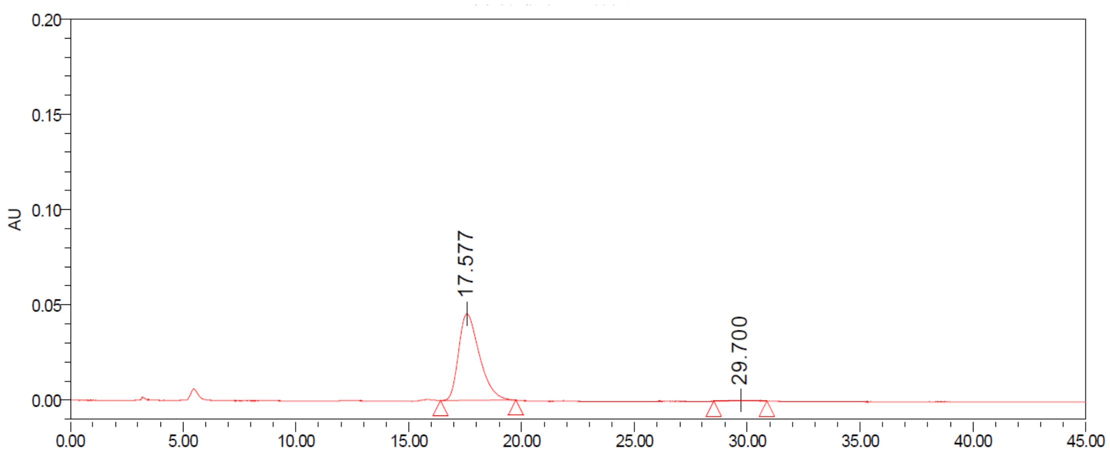

The larger version of HPLC chromatogram of chiral 90

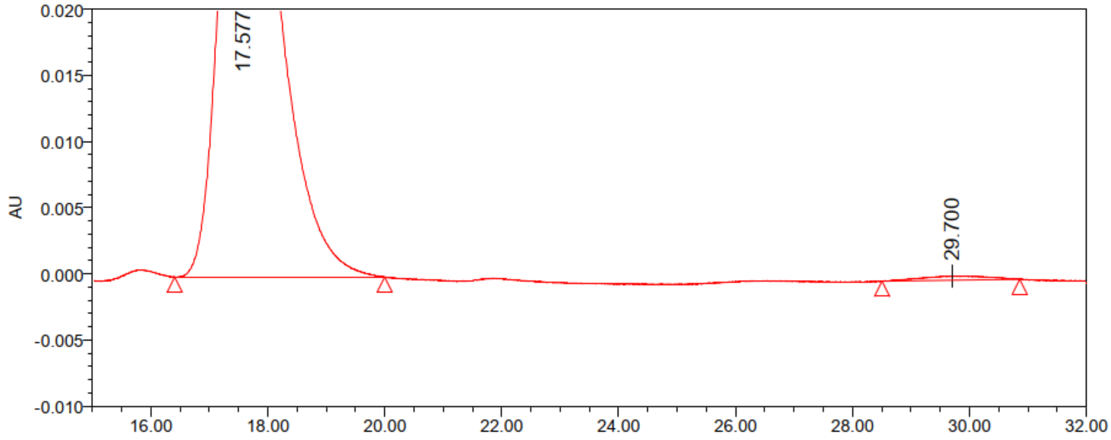

Entry	Retention Time/min	Area	Height	Area(%)
1	17.577	2934094	45448	99.17
2	29.700	24603	327	0.83

Supplementary Figure 192. Chiral HPLC analysis of chiral **90**

(4-((1-Cyclohexyl-1H-tetrazol-5-yl)(morpholino)methyl)phenyl)methanol

(racemic-1L)

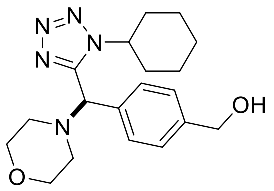

1L

Colorless oil; 68% yield (243 mg). ^{1}H NMR (400 MHz, $CDCl_3$) δ 7.39 (d, *J* = 8.2 Hz, 2H), 7.35 (d, *J* = 8.2 Hz, 2H), 4.96 (s, 1H), 4.67 (s, 2H), 4.60 – 4.52 (m, 1H), 4.03 (s, 1H), 3.69 (t, *J* = 4.5 Hz, 4H), 2.57 (dd, *J* = 11.0, 4.5 Hz, 2H), 2.44 – 2.33 (m, 2H), 1.86 (dd, *J* = 9.8, 7.2 Hz, 4H), 1.74 (d, *J* = 7.9 Hz, 2H), 1.59 (d, *J* = 10.0 Hz, 1H), 1.44 – 1.19 (m, 3H). ^{13}C NMR (101 MHz, $CDCl_3$) δ 153.3, 142.2, 133.4, 128.7, 127.2, 66.7, 65.0, 63.9, 58.0, 51.6, 32.8, 32.7, 25.3, 25.2, 24.7. HRMS (ESI) $[M+H]^+$ calcd for $C_{19}H_{28}N_5O_2^+$, 358.2238, found 358.2236.

Tert-butyl (2S,3R)-2-((4-((R)-(1-cyclohexyl-1H-tetrazol-5-yl)(morpholino)methyl)benzyl)oxy)-3-(2-oxo-2-phenylethyl)-2,5-diphenylpent-4-ynoate (*syn*-91)

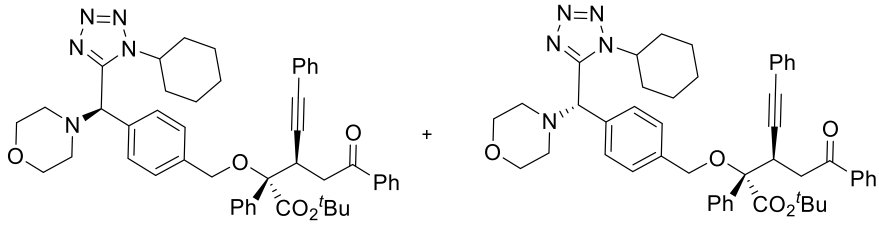

syn-**91** *syn*-**91'**

White solid; 55% yield (21.4 mg), 1:1 d.r., 98% ee, 98% ee. Composite NMR signals of *syn*-**91** and *syn*-**91’** (All spectral peaks of two diasteroisomers *syn*-**91** and *syn*-**91’** are overlapping in ^{1}H NMR and ^{13}C NMR). ^{1}H NMR (400 MHz, $CDCl_3$) δ 7.85 (d, *J* = 7.7 Hz, 2H), 7.68 (d, *J* = 7.7 Hz, 2H), 7.53 – 7.48 (m, 3H), 7.45 – 7.38 (m, 6H), 7.37 – 7.32 (m, 4H), 7.26 – 7.21 (m, 6H), 5.07 (d, *J* = 11.7 Hz, 1H), 4.94 (d, *J* = 4.3 Hz, 1H), 4.93 – 4.89 (m, 1H), 4.53 (d, *J* = 8.1 Hz, 1H), 4.42 (d, *J* = 8.3 Hz, 1H), 3.75 (s, 4H), 3.33 (dd, *J* = 16.7, 10.2 Hz, 1H), 3.13 (dd, *J* = 14.2, 5.3 Hz, 1H), 2.71 – 2.58 (m, 2H), 2.43 – 2.34 (m, 2H), 1.89 (dd, *J* = 10.6, 8.2 Hz, 4H), 1.69 (dd, *J* = 24.5, 5.7 Hz, 4H), 1.52

(d, J = 1.5 Hz, 2H), 1.51 (s, 9H). ^{13}C NMR (101 MHz, $CDCl_3$) δ 197.4, 169.4, 153.2, 139.8, 138.3, 136.9, 133.9, 133.2, 133.1, 131.5, 131.4, 128.6, 128.6, 128.5, 128.4, 128.3, 128.2, 128.2, 128.1, 128.0, 127.9, 126.7, 123.4, 89.6, 85.8, 82.9, 68.0, 66.9, 65.5, 58.1, 52.0, 39.3, 36.5, 32.8, 29.7, 28.1, 25.5, 25.4, 24.8. HRMS (ESI) $[M+Na]^+$ calcd for $C_{48}H_{53}N_5O_5Na^+$, 802.3939, found 802.3944. (Chiral IA-3, λ= 254 nm, *n*-hexane/2-propanol=19/1, Flow rate = 1.0 mL/min), (*R*)- 102 (98% ee) and 102' (98% ee): t_R = 43.447 min (major), 102.904 min, $t_{R'}$ = 55.800 min (major), 113.780 min. (*S*)- 102 (98% ee) and 102' (98% ee): t_R = 44.192 min, 103.376 min(major), $t_{R'}$ = 57.050 min, 114.206 min (major).

HPLC chromatogram of racemic 91

Condition: n-hexane/2-propanol =19:1

Flow rate =1.0 mL/min

λ= 254 nm

Chiral IA-3

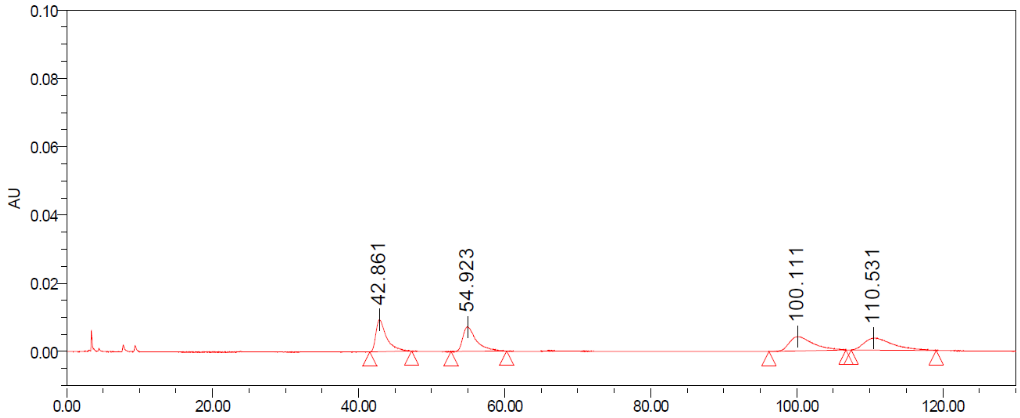

Entry	Retention Time/min	Area	Height	Area(%)
1	42.861	947265	9351	25.29
2	54.923	929508	7204	24.82
3	100.111	936319	4180	25.00
4	110.531	931922	3459	24.88

Supplementary Figure 193. Chiral HPLC analysis of racemic **91**

HPLC chromatogram of (*R*)- 91 and 91’

Condition: n-hexane/2-propanol =19:1

Flow rate =1.0 mL/min

λ= 254 nm

Chiral IA-3

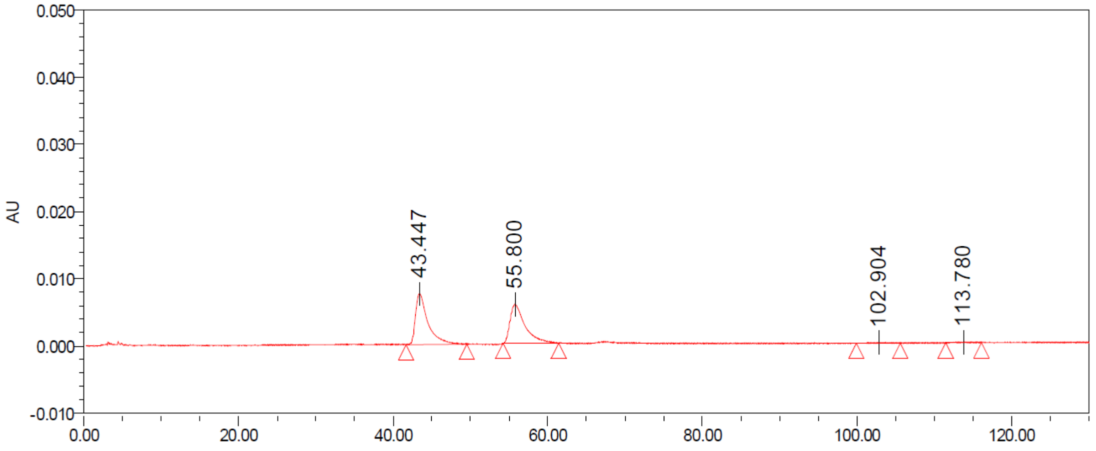

The larger version of HPLC chromatogram of (*R*)- 91 and 91’

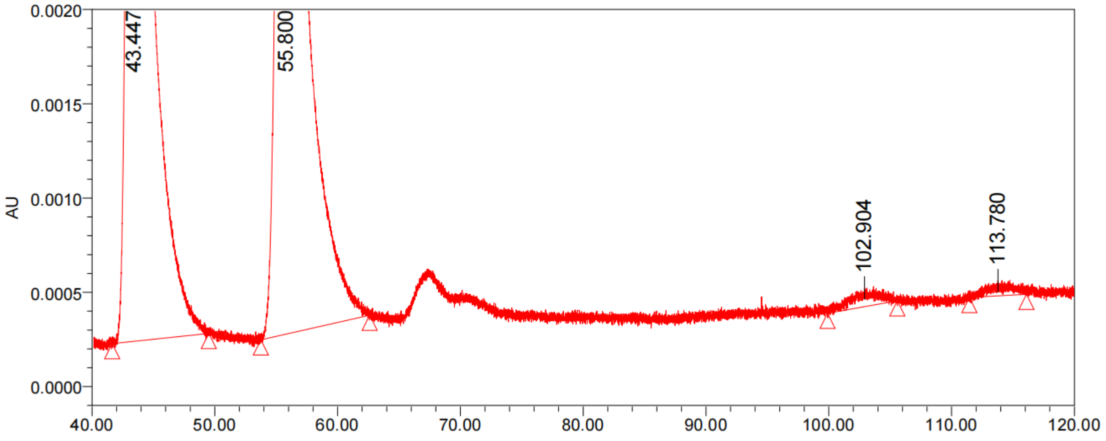

Entry	Retention Time/min	Area	Height	Area(%)
1	43.447	826278	7549	50.99
2	55.800	772051	5763	47.64
3	102.904	13469	100	0.83
4	113.780	8814	80	0.54

Supplementary Figure 194. Chiral HPLC analysis of **(*R*)- 91 and 91’**

HPLC chromatogram of (*S*)-91 and 91’

Condition: n-hexane/2-propanol =19:1

Flow rate =1.0 mL/min

λ= 254 nm

Chiral IA-3

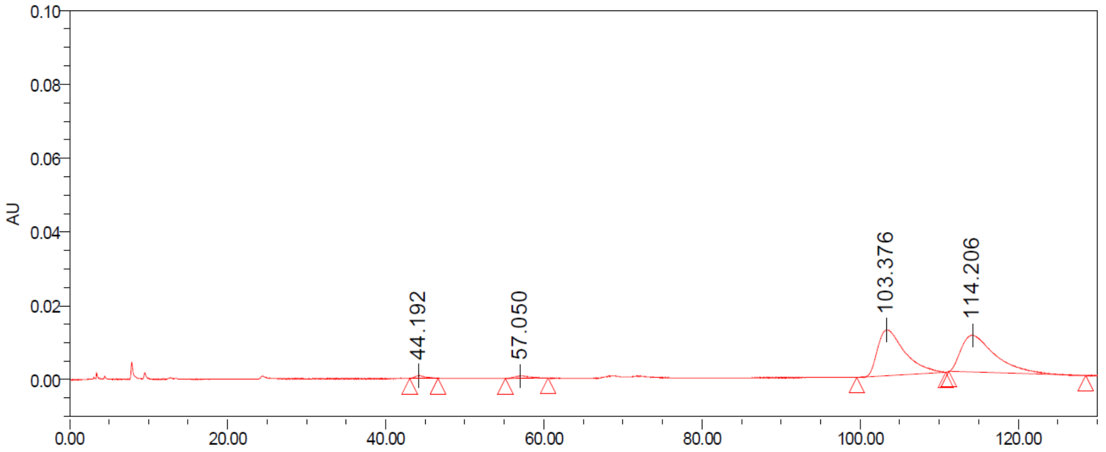

The larger version of HPLC chromatogram of (*S*)-91 and 91’

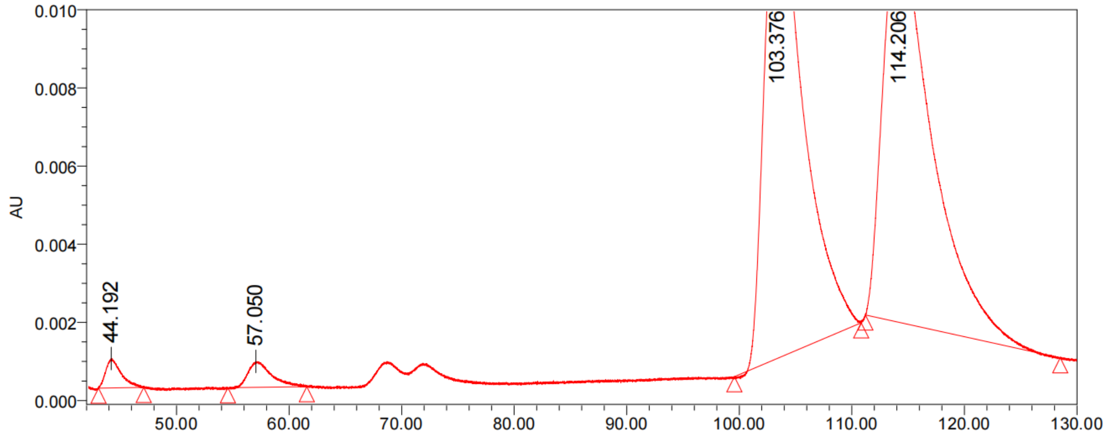

Entry	Retention Time/min	Area	Height	Area(%)
1	44.192	66773	743	1.09
2	57.050	81867	639	1.34
3	103.376	2951437	12376	48.14
4	114.206	3030941	10015	49.44

Supplementary Figure 195. Chiral HPLC analysis of **(*S*)-91 and 91’**

(S)-N-cyclohexyl-2-(2-(4-(hydroxymethyl)phenyl)-N-(4-nitrophenyl)acetamido)-4-phenylbutanamide (1N)

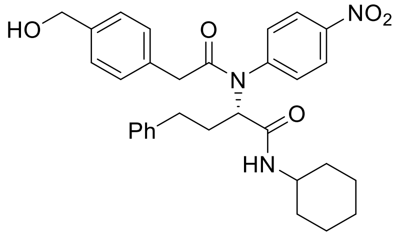

1N

Yellow solid; 75% yield (39.7 mg), 90% ee. ^{1}H NMR (400 MHz, $CDCl_3$) δ 8.20 (d, *J* = 9.0 Hz, 2H), 7.31 (d, *J* = 9.0 Hz, 2H), 7.26 – 7.21 (m, 4H), 7.18 (d, *J* = 7.2 Hz, 1H), 7.06 (d, *J* = 7.0 Hz, 2H), 6.93 (d, *J* = 8.0 Hz, 2H), 6.43 (d, *J* = 7.5 Hz, 1H), 5.01 (dd, *J* = 9.4, 5.8 Hz, 1H), 4.64 (s, 2H), 3.82 – 3.71 (m, 1H), 3.49 – 3.35 (m, 2H), 2.62 (ddd, *J* = 14.8, 9.6, 5.5 Hz, 1H), 2.54 – 2.45 (ddd, *J* = 14.8, 9.6, 5.5 Hz, 1H), 1.92 – 1.83 (m, 3H), 1.75 – 1.63 (m, 2H), 1.60 – 1.51 (m, 2H), 1.40 – 1.32 (m, 2H), 1.23 – 1.16 (m, 2H), 1.13 – 1.05 (m, 1H). ^{13}C NMR (101 MHz, $CDCl_3$) δ 171.5, 168.9, 147.5, 144.5, 140.5, 140.0, 133.2, 131.0, 129.5, 128.9, 128.6, 128.4, 127.2, 126.3, 124.5, 64.7, 58.2, 48.4, 41.7, 32.9, 32.8, 32.4, 31.0, 25.5, 24.7. HRMS (ESI) $[M+Na]^+$ calcd for $C_{31}H_{35}N_3O_5Na^+$, 530.2649, found 530.2648. (Chiral IC, λ= 254 nm, *n*-hexane/2-propanol= 4/1, Flow rate = 1.0 mL/min), t_R = 25.257 min (major), 47.611 min.

HPLC chromatogram of racemic 1N

Condition: n-hexane/2-propanol =4:1

Flow rate =1.0 mL/min

λ= 224.5 nm

Chiral IC

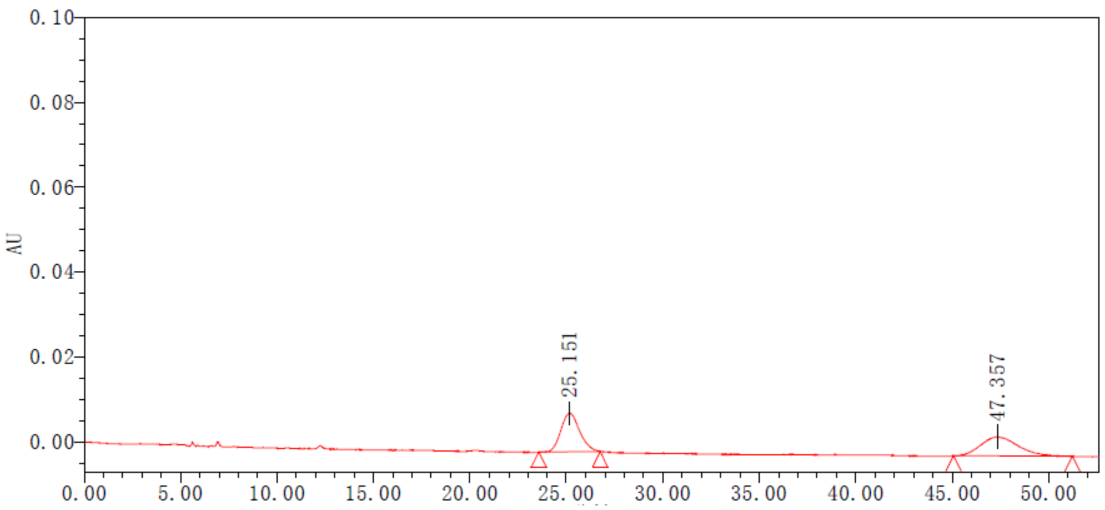

Entry	Retention Time/min	Area	Height	Area(%)
1	25.151	613419	9096	49.99
2	47.357	613670	4506	50.01

Supplementary Figure 196. Chiral HPLC analysis of racemic **1N**

HPLC chromatogram of chiral 1N

Condition: n-hexane/2-propanol =4:1

Flow rate =1.0 mL/min

λ= 224.5 nm

Chiral IC

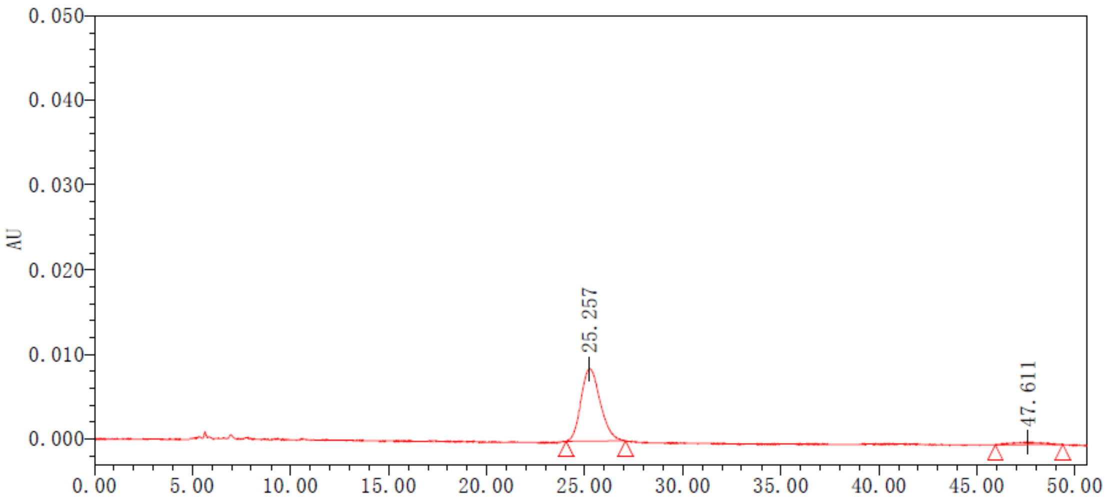

Entry	Retention Time/min	Area	Height	Area(%)
1	25.257	583278	8532	94.95
2	47.611	31052	279	5.05

Supplementary Figure 197. Chiral HPLC analysis of chiral **1N**

Tert-butyl (2S,3R)-2-((4-(2-(((S)-1-(cyclohexylamino)-1-oxo-4-phenylbutan-2-yl)(4-nitrophenyl)amino)-2-oxoethyl)benzyl)oxy)-3-(2-oxo-2-phenylethyl)-2,5-diphenylpent-4-ynoate (*syn*-92)

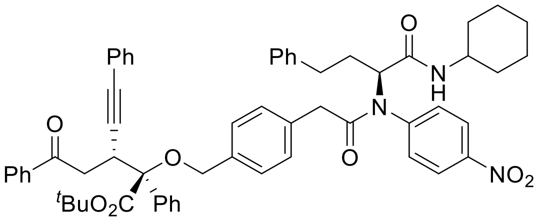

syn-**92**

Yellow solid; 62% yield (29.5 mg), 95:5 d.r., 92% ee. ^{1}H NMR (500 MHz, $CDCl_3$) δ 8.21 (d, *J* = 9.0 Hz, 2H), 7.86 (d, *J* = 7.7 Hz, 2H), 7.68 (d, *J* = 7.5 Hz, 2H), 7.52 (t, *J* = 7.4 Hz, 1H), 7.41 (t, *J* = 7.8 Hz, 2H), 7.39 – 7.35 (m, 4H), 7.33 – 7.28 (m, 3H), 7.25 – 7.22 (m, 7H), 7.17 (t, *J* = 7.3 Hz, 1H), 7.06 (d, *J* = 7.2 Hz, 2H), 6.94 (d, *J* = 8.0 Hz, 2H), 6.41 (s, 1H), 5.04 (td, *J* = 11.6, 4.9 Hz, 2H), 4.90 (d, *J* = 11.4 Hz, 1H), 4.41 (dd, *J* = 10.3, 2.8 Hz, 1H), 3.85 – 3.69 (m, 1H), 3.49 – 3.39 (m, 2H), 3.34 (dd, *J* = 16.5, 10.4 Hz, 1H), 3.10 (d, *J* = 16.0 Hz, 1H), 2.68 – 2.59 (m, 1H), 2.55 – 2.46 (m, 1H), 1.88 (td, *J* = 9.4, 4.7 Hz, 2H), 1.82 (d, *J* = 15.8 Hz, 1H), 1.59 (dd, *J* = 20.8, 14.8 Hz, 4H), 1.51 (s, 9H), 1.37 – 1.31 (m, 4H), 0.88 (t, *J* = 6.9 Hz, 1H). ^{13}C NMR (126 MHz, $CDCl_3$) δ 197.4, 171.5, 169.4, 168.9, 147.5, 144.6, 140.5, 138.4, 138.1, 136.9, 133., 131.5, 131.4, 131.0, 128.7, 128.7, 128.6, 128.5, 128.4, 128.2, 128.2, 128.2, 128.2, 128.1, 127.9, 127.8, 127.8, 127.3, 126.7, 126.3, 124.5, 123.4, 89.7, 85.8, 84.1, 82.8, 68.2, 58.2, 48.3, 41.7, 39.2, 36.5, 32.9, 32.8, 32.4, 31.9, 31.6, 31.5, 30.9, 30.3, 30.2, 29.7, 29.7, 29.4, 28.1, 25.5, 24.7, 22.7, 14.1. HRMS (ESI) $[M+Na]^+$ calcd for $C_{60}H_{61}N_3O_7Na^+$, 974.4351, found 974.4355. (Chiral IA-3, λ= 254 nm, *n*-hexane/2-propanol= 17/3, Flow rate = 1.0 mL/min), t_R = 23.126 min (major), 41.012 min.

HPLC chromatogram of racemic 92

Condition: n-hexane/2-propanol =17:3

Flow rate =1.0 mL/min

λ= 254.0 nm

Chiral IA-3

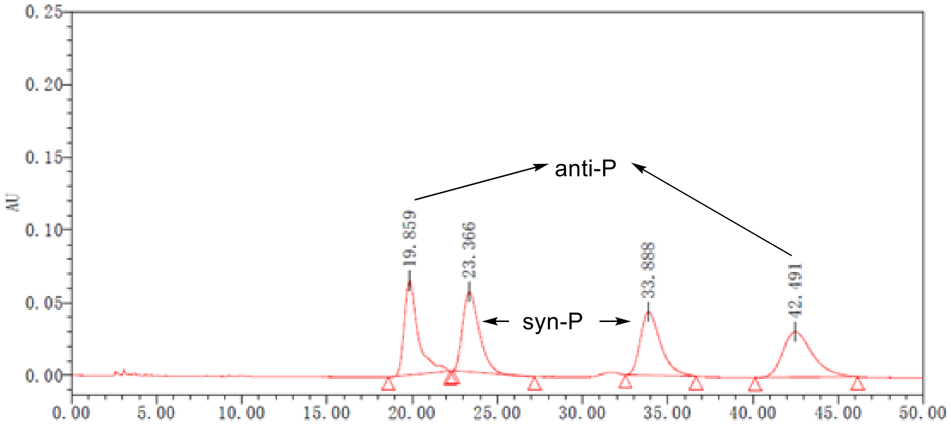

Entry	Retention Time/min	Area	Height	Area(%)
1	19.859	3732170	64728	25.10
2	23.366	3743187	55078	25.17
3	33.888	3593935	43530	24.17
4	42.491	3799582	31233	25.55

Supplementary Figure 198. Chiral HPLC analysis of racemic **92**

HPLC chromatogram of chiral 92

Condition: n-hexane/2-propanol =17:3

Flow rate =1.0 mL/min

λ= 254.0 nm

Chiral IA-3

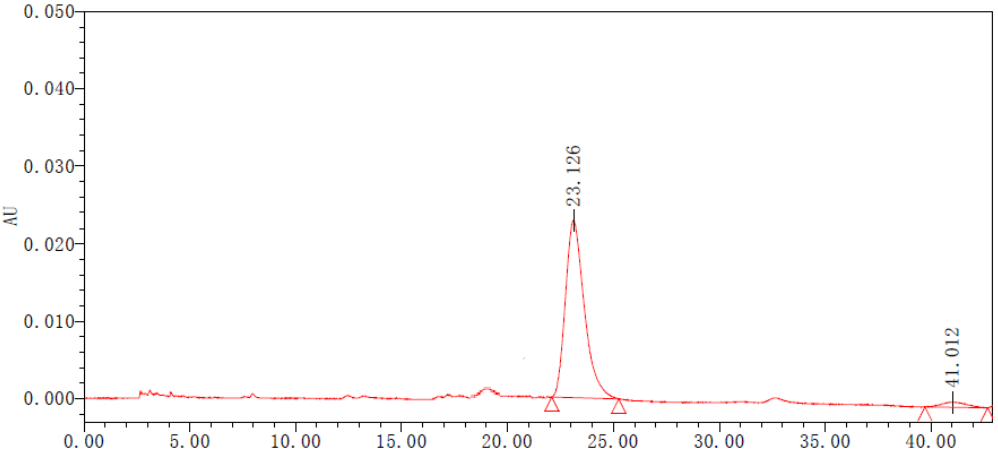

Entry	Retention Time/min	Area	Height	Area(%)
1	23.126	1416957	22886	96.24
2	41.012	55343	657	3.76

Supplementary Figure 199. Chiral HPLC analysis of chiral **92**

Tert-butyl (2S,3S,5R)-2,5-dihydroxy-3-phenethyl-2,5-diphenylpentanoate(93)

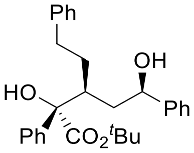

93

Colorless oil, 38.4 mg, 86% yield, >20:1 d.r., 92% ee. ^{1}H NMR (400 MHz, $CDCl_3$) δ 7.67 (d, *J* = 7.2 Hz, 2H), 7.36 (dd, *J* = 8.4, 6.6 Hz, 2H), 7.33 (d, *J* = 7.3 Hz, 2H), 7.29 (d, *J* = 6.9 Hz, 1H), 7.25 – 7.20 (m, 5H), 7.14 (t, *J* = 7.3 Hz, 1H), 6.95 (d, *J* = 7.1 Hz, 2H), 3.86 (s, 1H), 2.92 – 2.82 (m, 1H), 2.69 (ddd, *J* = 13.7, 10.3, 6.7 Hz, 1H), 2.61 – 2.51 (m, 1H), 2.43 (dd, *J* = 13.1, 8.4 Hz, 1H), 2.32 – 2.22 (m, 1H), 1.98 – 1.87 (m, 1H), 1.79 – 1.70 (m, 1H), 1.70 – 1.62 (m, 1H), 1.54 (ddd, *J* = 14.4, 8.5, 4.4 Hz, 1H), 1.45 (s, 9H). ^{13}C NMR (126 MHz, $CDCl_3$) δ 174.8, 142.8, 142.6, 141.6, 128.5, 128.4, 128.3, 128.2, 128.0, 127.5, 126.0, 125.9, 125.6, 83.42, 82.02, 44.44, 34.40, 34.14, 32.86, 30.87, 27.77. HRMS (ESI) $[M+Na]^+$ calcd for $C_{29}H_{34}O_4Na^+$, 469.2377, found 469.2377. (Chiral IE-3, λ= 254 nm, *n*-hexane/2-propanol= 99/1, Flow rate = 1.0 mL/min), t_R = 14.319 min, 15.178 min (major).

HPLC chromatogram of racemic 93

Condition: n-hexane/2-propanol = 99:1

Flow rate =1.0 mL/min

λ= 254 nm

Chiral IE-3

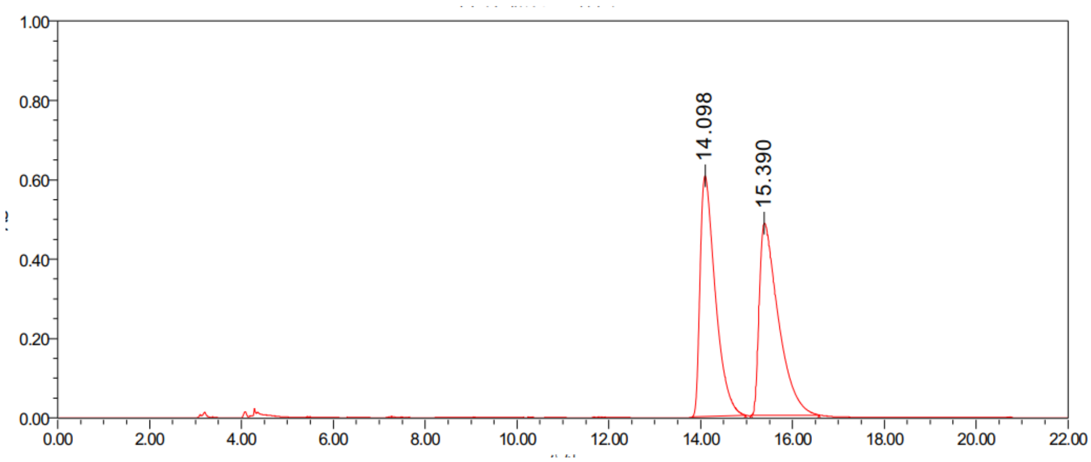

Entry	Retention Time/min	Area	Height	Area(%)
1	14.098	14012053	605562	49.91
2	15.390	14065380	484444	50.09

Supplementary Figure 200. Chiral HPLC analysis of racemic **93**

HPLC chromatogram of chiral 93

Condition: n-hexane/2-propanol =99:1

Flow rate =1.0 mL/min

λ= 254 nm

Chiral IE-3

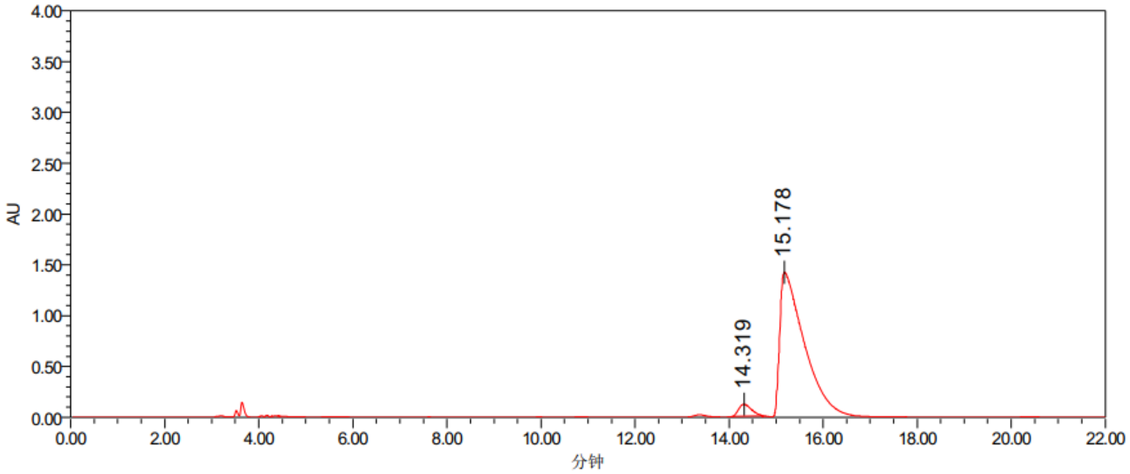

Entry	Retention Time/min	Area	Height	Area(%)
1	14.319	2147628	116439	4.00
2	15.178	51512268	142107	96.00

Supplementary Figure 201. Chiral HPLC analysis of chiral **93**

Tert-butyl (3S,4R)-5-((E)-benzylidene)-4-(2-oxo-2-phenylethyl)-3-phenyl-1,3,4,5-tetrahydrobenzo[c]oxepine-3-carboxylate (94)

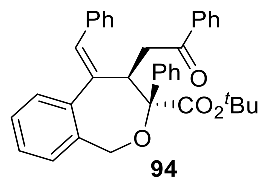

94

Colorless oil; 38.2 mg, 72% yield, 92:8 d.r., 88% *ee*; ^{1}H NMR (400 MHz, $CDCl_3$) δ 7.93 (d, *J* = 7.6 Hz, 2H), 7.62 (d, *J* = 7.1 Hz, 2H), 7.48 (t, *J* = 7.7 Hz, 2H), 7.40 (d, *J* = 8.1 Hz, 3H), 7.37 – 7.27 (m, 3H), 7.24 – 7.14 (m, 5H), 7.05 (t, *J* = 7.4 Hz, 1H), 6.97 (d, *J* = 6.9 Hz, 1H), 6.72 (s, 1H), 5.48 – 5.30 (m, 2H), 4.90 – 4.73 (m, 1H), 3.01 (dd, *J* = 17.5, 9.9 Hz, 1H), 2.41 (dd, *J* = 17.5, 3.5 Hz, 1H), 0.98 (s, 9H).^{13}C NMR (101 MHz, $CDCl_3$) δ 199.8, 169.8, 143.9, 140.6, 140.05, 138.0, 137.3, 136.2, 134.0, 132.6, 130.5, 129.7, 128.5, 128.4, 128.1, 128.1, 127.9, 127.58, 127.5, 127.3, 127.1, 126.0, 100.0, 88.1, 82.2, 70.2, 40.3, 37.9, 29.7, 27.2. HRMS (ESI) $[M+Na]^+$ calcd for $C_{36}H_{34}O_4Na^+$, 553.2349, found 553.2349. (Chiral IE-3, λ= 254 nm, *n*-hexane/2-propanol= 9/1, Flow rate = 1.0 mL/min), t_R = 6.570 min(major), 8.019 min.

HPLC chromatogram of racemic 94

Condition: n-hexane/2-propanol =9:1

Flow rate =1.0 mL/min

λ= 254 nm

Chiral IE-3

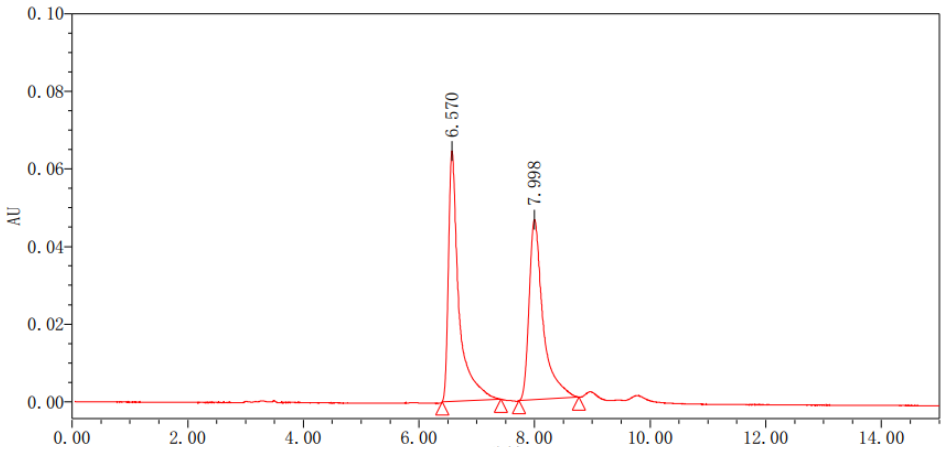

Entry	Retention Time/min	Area	Height	Area(%)
1	6.570	814480	64518	51.01
2	7.998	782255	46420	48.99

Supplementary Figure 202. Chiral HPLC analysis of racemic **94**

HPLC chromatogram of chiral 94

Condition: n-hexane/2-propanol =9:1

Flow rate =1.0 mL/min

λ= 254 nm

Chiral IE-3

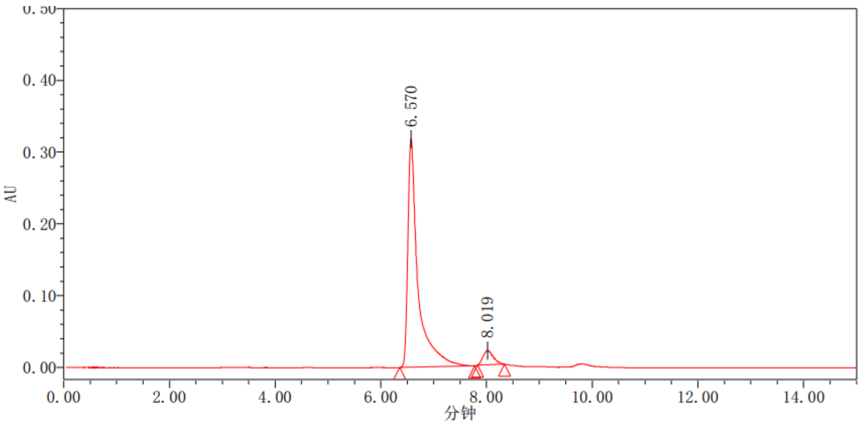

Entry	Retention Time/min	Area	Height	Area(%)
1	6.570	4250038	317623	94.05
2	8.019	268945	19813	5.95

Supplementary Figure 203. Chiral HPLC analysis of chiral **94**

(2S,3R)-2-((4-Bromobenzyl)oxy)-3-(4-nitrophenyl)-5-oxo-2,5-diphenylpentanoic acid (95)

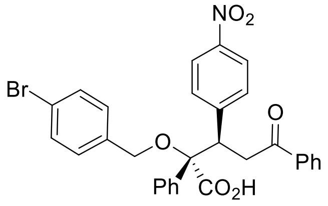

95

Colorless oil; 43.1 mg, 75% yield. ^{1}H NMR (400 MHz, $CDCl_3$) δ 7.96 (d, *J* = 8.8 Hz, 2H), 7.76 – 7.70 (m, 2H), 7.54 – 7.51 (m, 2H), 7.51 – 7.48 (m, 2H), 7.39 – 7.37 (m, 5H), 7.35 – 7.33 (m, 3H), 7.21 (d, *J* = 8.4 Hz, 2H), 6.19 (s, 1H), 4.74 (d, *J* = 12.0 Hz, 1H), 4.47 (d, *J* = 12.0 Hz, 1H), 4.34 (dd, *J* = 8.6, 5.2 Hz, 1H), 3.65 – 3.49 (m, 2H). ^{13}C NMR (101 MHz, $CDCl_3$) δ 197.3, 147.0, 146.7, 137.1, 136.5, 135.1, 133.4, 131.7, 131.6, 131.2, 129.0, 128.9, 128.8, 128.7, 128.6, 128.0, 128.0, 127.8, 122.6, 121.6, 87.8, 67.9, 51.2, 39.2, 29.7. HRMS (ESI) $[M+Na]^+$ calcd for $C_{30}H_{24}NO_6BrNa^+$, 596.0679, found 596.0675.

Methyl (2S,3R)-2-((4-bromobenzyl)oxy)-3-(4-nitrophenyl)-5-oxo-2,5-diphenylpentanoate (95-b)

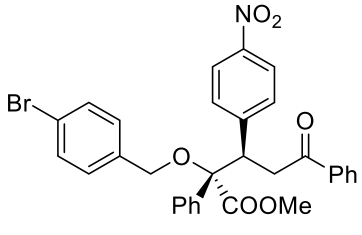

95-b

Colorless oil; 52.4 mg, 70% yield, 89% ee. ^{1}H NMR (400 MHz, $CDCl_3$) δ 8.01 (d, *J* = 8.8 Hz, 2H), 7.75 (d, *J* = 7.3 Hz, 2H), 7.53 – 7.47 (m, 5H), 7.42 – 7.29 (m, 8H), 7.22 (s, 1H), 4.74 (d, *J* = 12.2 Hz, 1H), 4.49 (d, *J* = 12.2 Hz, 1H), 4.33 (dd, *J* = 9.7, 4.2 Hz, 1H), 3.69 (s, 3H), 3.60 (dd, *J* = 17.3, 9.7 Hz, 1H), 3.53 (dd, *J* = 17.4, 4.2 Hz, 1H). ^{13}C

NMR (101 MHz, $CDCl_3$) δ 196.1, 170.0, 146.1, 145.9, 136.4, 135.5, 134.8, 132.2, 130.6, 130.0, 127.7, 127.6, 127.4, 126.9, 126.7, 121.5, 120.4, 87.1, 66.9, 51.2, 50.3, 38.1. HRMS (ESI) $[M+Na]^+$ calcd for $C_{31}H_{26}NO_6BrNa^+$, 610.0836, found 610.0840. (Chiral IA, λ= 254 nm, *n*-hexane/2-propanol= 9/1, Flow rate = 1.0 mL/min), t_R = 13.333 min, 21.811 min (major).

HPLC chromatogram of racemic 95-b

Condition: n-hexane/2-propanol =9:1

Flow rate =1.0 mL/min

λ= 254 nm

Chiral IA

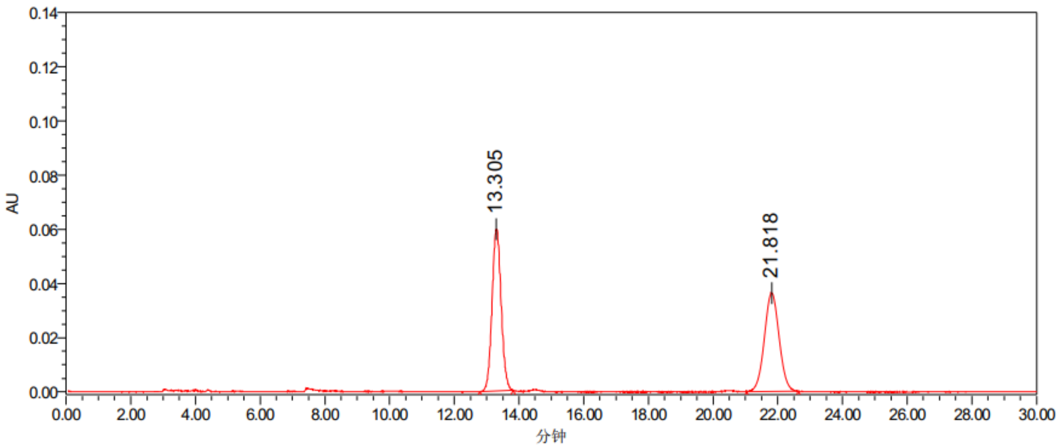

Entry	Retention Time/min	Area	Height	Area(%)
1	13.305	1157118	59812	50.05
2	21.818	1154629	36356	49.95

Supplementary Figure 204. Chiral HPLC analysis of racemic **95-b**

HPLC chromatogram of chiral 95-b

Condition: n-hexane/2-propanol =9:1

Flow rate =1.0 mL/min

λ= 254 nm

Chiral IA

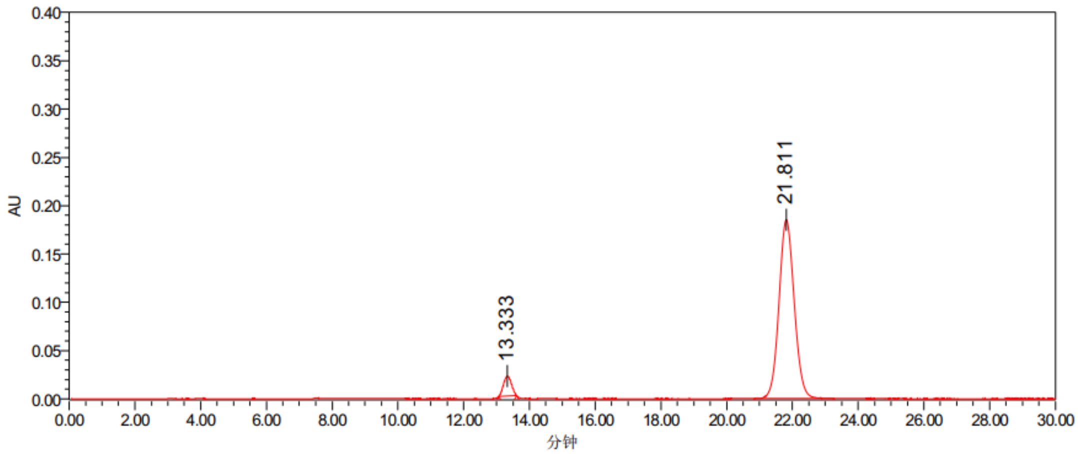

Entry	Retention Time/min	Area	Height	Area(%)
1	13.333	342502	20612	5.49
2	21.811	5965331	18497	94.51

Supplementary Figure 205. Chiral HPLC analysis of chiral **95-b**

Tert-butyl (2S,3R)-2-hydroxy-3-(2-oxo-2-phenylethyl)-2,5-diphenylpent-4-ynoate (96)

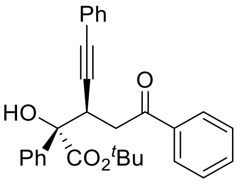

96

Colorless oil; 26.4 mg, 60% yield, >20:1 d.r., 95% *ee*; ^{1}H NMR (500 MHz, $CDCl_3$) δ 7.90 (d, *J* = 7.7 Hz, 2H), 7.74 (d, *J* = 7.8 Hz, 2H), 7.52 (t, *J* = 7.4 Hz, 1H), 7.41 (t, *J* = 7.7 Hz, 2H), 7.36 (t, *J* = 7.6 Hz, 2H), 7.29 (d, *J* = 6.9 Hz, 3H), 7.23 (d, *J* = 6.6 Hz, 3H), 4.29 – 4.27 (m, 1H), 4.26 (s, 1H), 3.54 (dd, *J* = 17.1, 10.6 Hz, 1H), 2.65 (dd, *J* = 17.1, 2.8 Hz, 1H), 1.49 (s, 9H). ^{13}C NMR (101 MHz, $CDCl_3$) δ 198.0, 172.9, 138.5, 136.9, 133.1, 131.7, 128.5, 128.4, 128.3, 128.09, 128.1, 127.9, 126.3, 123.1, 88.3, 84.1, 83.3, 79.9, 38.4, 37.5, 27.8. HRMS (ESI) $[M+Na]^+$ calcd for $C_{29}H_{28}O_4Na^+$, 463.1880, found

463.1883. (Chiral IE-3, λ= 254 nm, *n*-hexane/2-propanol= 49/1, Flow rate = 1.0 mL/min), t_R = 10.371 min(major), 15.249 min.

HPLC chromatogram of racemic 96

Condition: n-hexane/2-propanol =49:1

Flow rate =1.0 mL/min

λ= 254 nm

Chiral IE-3

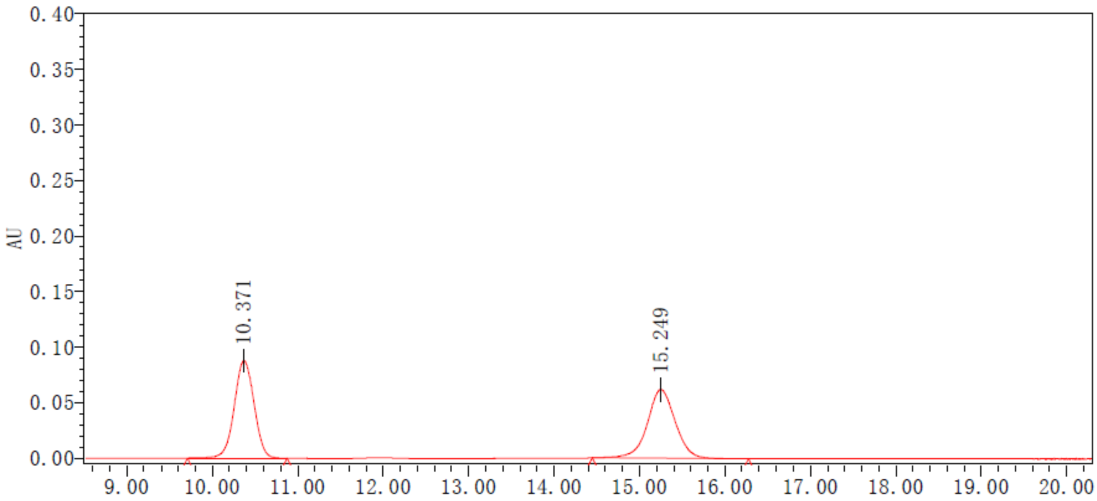

Entry	Retention Time/min	Area	Height	Area(%)
1	10.371	1415136	87699	50.21
2	15.249	1403227	61924	49.79

Supplementary Figure 206. Chiral HPLC analysis of racemic **96**

HPLC chromatogram of chiral 96

Condition: n-hexane/2-propanol =49:1

Flow rate =1.0 mL/min

λ= 254 nm

Chiral IE-3

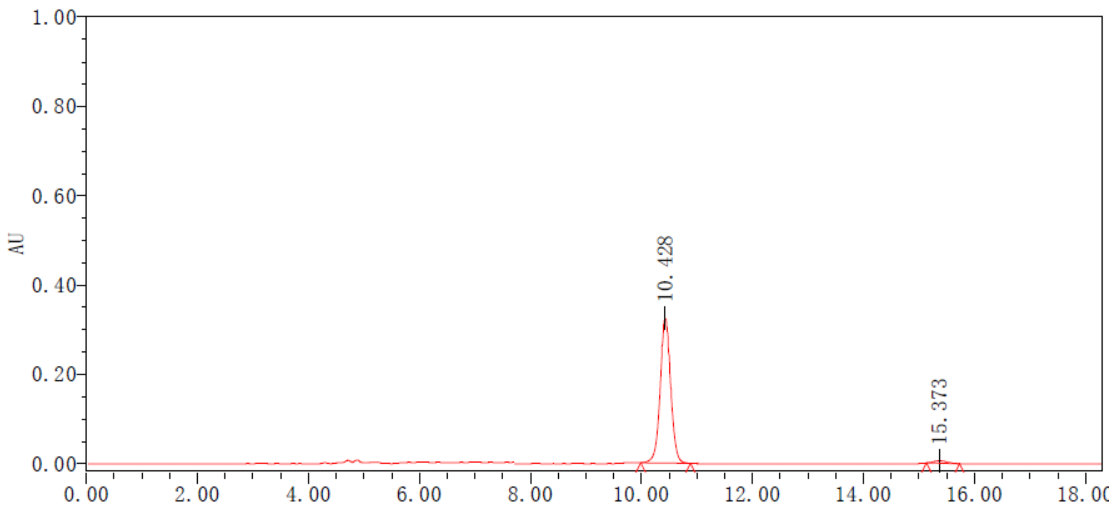

Entry	Retention Time/min	Area	Height	Area(%)
1	10.428	4300933	325068	97.48
2	15.373	90142	5299	2.52

Supplementary Figure 207. Chiral HPLC analysis of chiral **96**

(2S,3R)-2-hydroxy-3-(2-oxo-2-phenylethyl)-2,5-diphenylpent-4-ynoic acid (97)

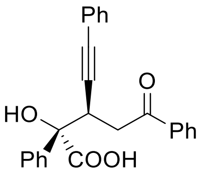

97

Colorless oil, 25.0 mg, 65% yield, >20:1 d.r.; ^{1}H NMR (500 MHz, Acetone-d6) δ 7.89 (dd, *J* = 19.3, 7.6 Hz, 4H), 7.57 (t, *J* = 7.3 Hz, 1H), 7.46 (t, *J* = 7.5 Hz, 2H), 7.25 (t, *J* = 7.4 Hz, 2H), 7.21 – 7.11 (m, 6H), 4.16 (d, *J* = 10.5 Hz, 1H), 3.37 (dd, *J* = 15.6, 11.2 Hz, 1H), 2.58 (d, *J* = 15.7 Hz, 1H). ^{13}C NMR (126 MHz, Acetone-d6) δ 198.4, 177.6, 143.6, 137.6, 132.7, 131.5, 128.5, 128.0, 127.3, 127.2, 126.6, 126.3, 124.4, 91.8, 81.9, 79.8, 39.1, 39.0. HRMS (ESI) $[M+Na]^+$ calcd for $C_{25}H_{20}O_4Na^+$, 407.1254, found 407.1254.

Methyl (2S,3R)-2-hydroxy-3-(2-oxo-2-phenylethyl)-2,5-diphenylpent-4-ynoate (97-b)

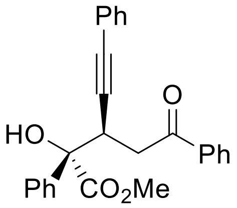

97-b

Colorless oil; 26.4 mg, 27.9 mg, 70% yield, >20:1 d.r., 95% ee; ^{1}H NMR (500 MHz, $CDCl_3$) δ 7.89 (d, *J* = 7.8 Hz, 2H), 7.75 (d, *J* = 7.8 Hz, 2H), 7.52 (t, *J* = 7.2 Hz, 1H), 7.44 – 7.35 (m, 5H), 7.31 (d, *J* = 6.4 Hz, 3H), 7.24 (d, *J* = 6.7 Hz, 2H), 4.37 (d, *J* = 10.0 Hz, 1H), 4.18 (s, 1H), 3.87 (s, 3H), 3.52 (dd, *J* = 17.2, 10.5 Hz, 1H), 2.70 (d, *J* = 17.3 Hz, 1H). ^{13}C NMR (126 MHz, $CDCl_3$) δ 197.7, 174.4, 137.7, 136.8, 133.2, 131.8, 128.6, 128.5, 128.4, 128.2, 128.1, 128.1, 126.3, 122.9, 87.9, 83.7, 80.4, 53.8, 38.2, 37.6. HRMS (ESI) $[M+Na]^+$ calcd for $C_{25}H_{22}O_4Na^+$, 421.1410, found 421.1413. (Chiral IE-3, λ= 254 nm, *n*-hexane/2-propanol= 9/1, Flow rate = 1.0 mL/min), t_R = 16.330 min(major), 26.039 min.

HPLC chromatogram of racemic 97-b

Condition: n-hexane/2-propanol =9:1

Flow rate =1.0 mL/min

λ= 254 nm

Chiral IE-3

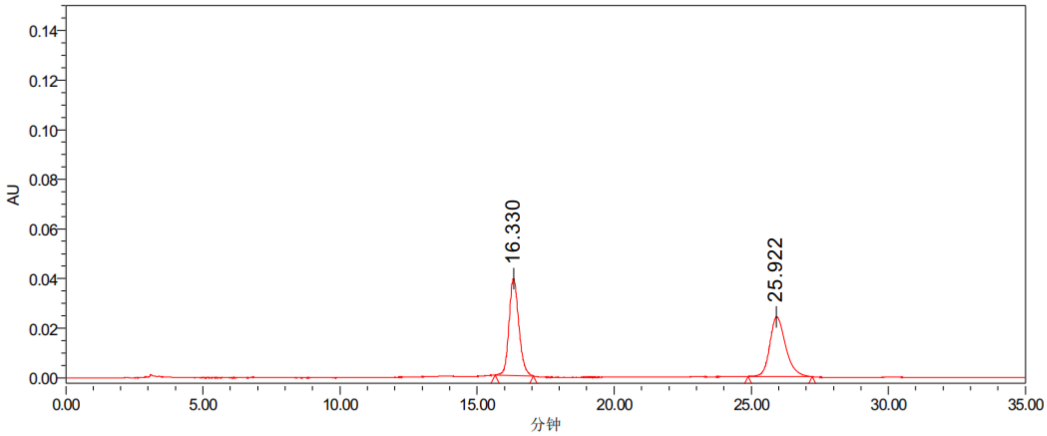

Entry	Retention Time/min	Area	Height	Area(%)
1	16.330	959906	39092	50.04
2	25.922	958252	24055	49.96

Supplementary Figure 208. Chiral HPLC analysis of racemic **97-b**

HPLC chromatogram of chiral 97-b

Condition: n-hexane/2-propanol =9:1

Flow rate =1.0 mL/min

λ= 254 nm

Chiral IE-3

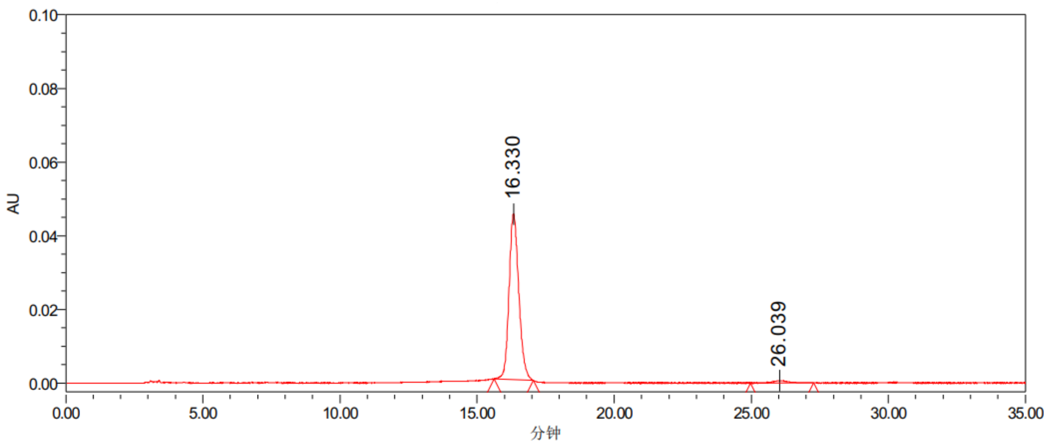

The larger version of HPLC chromatogram of chiral 97-b

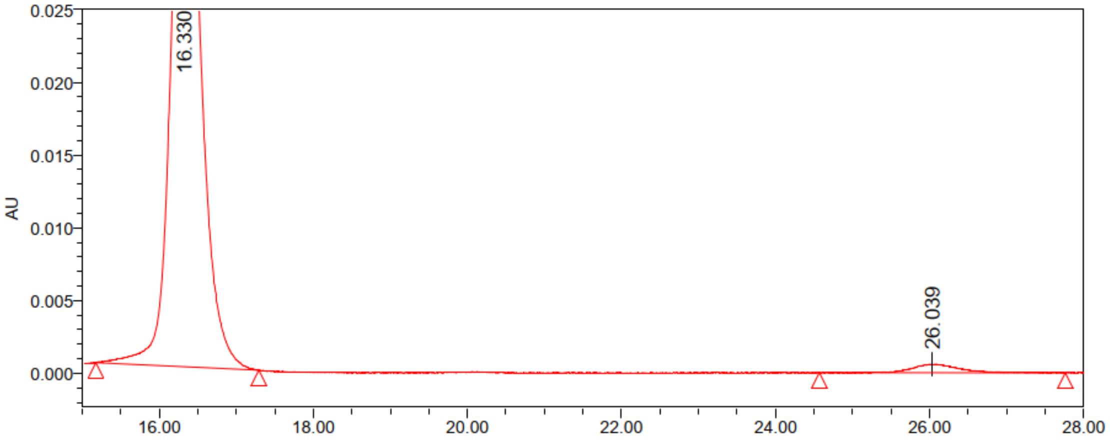

Entry	Retention Time/min	Area	Height	Area(%)
1	16.330	1100042	44912	97.51
2	26.039	25198	594	2.49

Supplementary Figure 209. Chiral HPLC analysis of chiral **97-b**

Methyl (2S,3R)-2-((4-methylbenzyl)oxy)-3-(2-oxo-2-phenylethyl)-2,5-diphenylpent-4-ynoate (*syn*-98)

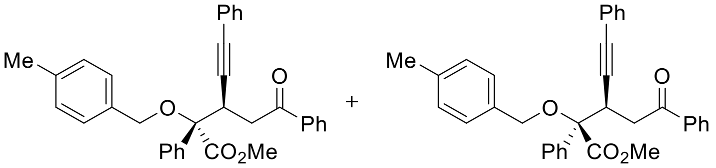

syn-**98**(major) *anti*-**98**(minor)

Colorless oil; Combined in 53% yield (39.9mg), 83:17 d.r, 21% ee. Composite NMR signals of *syn*-**98**(major) and *anti*-**98** (minor) (The ratio of two diasteroisomers in ^{1}H

NMR is *syn*-**98** : *anti*-**98** (minor) = 3.00 : 0.72). ^{1}H NMR (400 MHz, $CDCl_3$) δ 7.87 – 7.81 (comp, 2.80H), 7.70 – 7.62 (comp, 2.93H), 7.55 – 7.47 (comp, 1.72H), 7.43 – 7.29 (comp, 9.95H), 7.27 – 7.13 (comp, 11.35H), 5.06 (d, *J* = 11.1 Hz, 1.06H), 4.84 (d, *J* = 11.1 Hz, 1.09H), 4.54 (d, *J* = 2.9 Hz, 0.39H), 4.46 (comp, 1.30H), 3.85 (s, 3.02H), 3.83 (s, 0.72H), 3.32 (dd, *J* = 16.7, 10.2 Hz, 1.13H), 3.22 (dd, *J* = 16.5, 3.2 Hz, 0.33H), 3.14 (dd, *J* = 16.7, 3.1 Hz, 1.12H), 3.03 (dd, *J* = 16.5, 10.2 Hz, 0.36H), 2.36 (s, 2.98H), 2.34 (s, 0.79H). ^{13}C NMR (101 MHz, $CDCl_3$) δ 197.3, 171.4, 138.1, 137.2, 136.9, 135.6, 1345.0, 133.1, 131.5, 131.5, 129.0, 128.5, 128.4, 128.3, 128.2, 128.1, 127.9, 127.8, 127.7, 127.3, 126.8, 123.4, 89.2, 88.7, 86.7, 86.0, 84.4, 68.7, 68.0, 52.5, 39.3, 37.1, 36.6, 29.7, 21.2. HRMS (ESI) $[M+Na]^+$ calcd for $C_{34}H_{30}O_4Na^+$, 525.2036, found 525.2039. (Chiral IA, λ= 254 nm, *n*-hexane/2-propanol= 19/1, Flow rate = 1.0 mL/min), t_R = 11.159 min (major), 15.918 min.

HPLC chromatogram of racemic 98

Condition: n-hexane/2-propanol = 19:1

Flow rate =1.0 mL/min

λ= 254 nm

Chiral IA

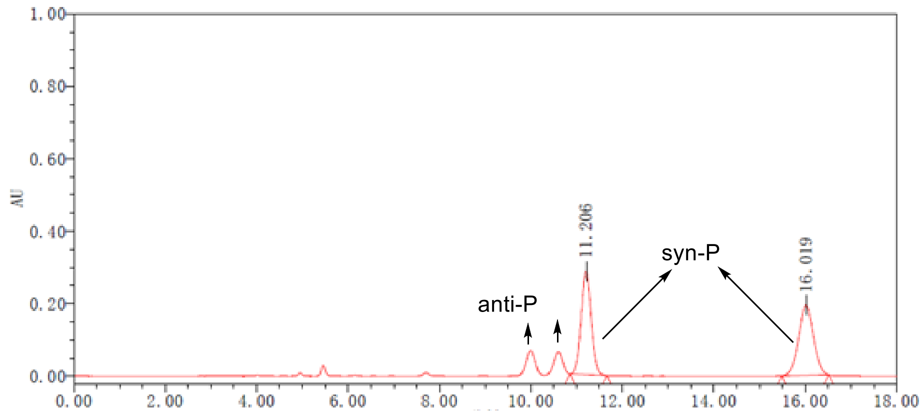

Entry	Retention Time/min	Area	Height	Area(%)
1	11.206	4409358	285693	50.09
2	16.019	4392774	193748	49.91

Supplementary Figure 210. Chiral HPLC analysis of racemic **98**

HPLC chromatogram of chiral 98

Condition: n-hexane/2-propanol =19:1

Flow rate =1.0 mL/min

λ= 254 nm

Chiral IA

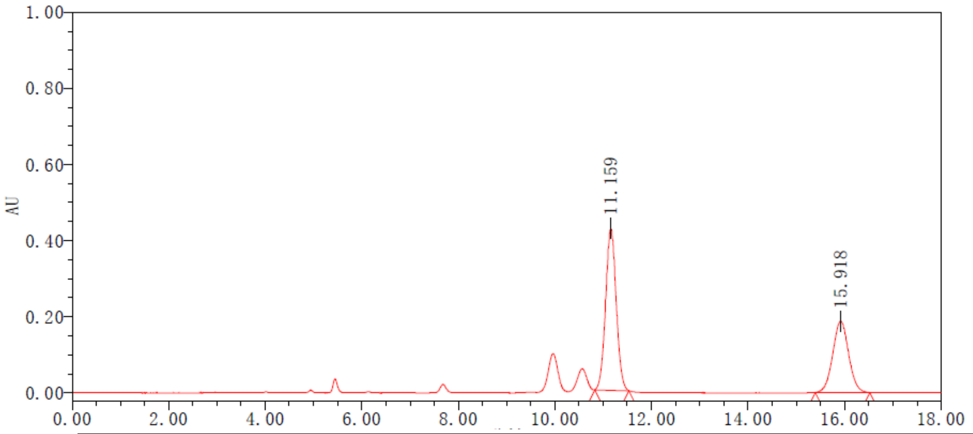

Entry	Retention Time/min	Area	Height	Area(%)
1	11.159	6535501	425356	60.47
2	15.918	4272173	187338	39.53

Supplementary Figure 211. Chiral HPLC analysis of chiral **98**

Isopropyl (2S,3R)-2-((4-bromobenzyl)oxy)-3-(2-oxo-2-phenylethyl)-2,5-diphenylpent-4-ynoate (S1)

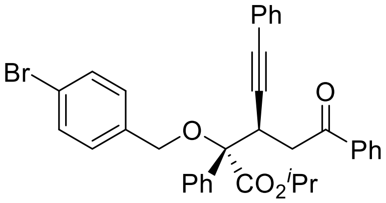

S1

Colorless oil; 55.3 mg, 62% yield, 96:4 d.r., 80% *ee*; ^{1}H NMR (400 MHz, $CDCl_3$) δ 7.86 – 7.81 (m, 2H), 7.69 (d, *J* = 7.2 Hz, 2H), 7.53 (t, *J* = 7.4 Hz, 1H), 7.47 (t, *J* = 6.8 Hz, 2H), 7.44 – 7.40 (m, 2H), 7.41 – 7.36 (m, 3H), 7.33 (dd, *J* = 9.3, 4.7 Hz, 2H), 7.26 – 7.21 (m, 5H), 5.20 (dt, *J* = 12.5, 6.2 Hz, 1H), 5.06 (d, *J* = 11.7 Hz, 1H), 4.90 (d, *J* = 11.7 Hz, 1H), 4.46 (dd, *J* = 10.2, 3.1 Hz, 1H), 3.31 (dd, *J* = 16.6, 10.2 Hz, 1H), 3.10 (dd, *J* = 16.6, 3.1 Hz, 1H), 1.29 (dd, *J* = 14.0, 6.3 Hz, 6H). ^{13}C NMR (101 MHz, $CDCl_3$) δ 197.3, 167.0, 138.0, 137.9, 136.9, 133.1, 131.4, 131.4, 129.3, 128.6, 128.5, 128.4, 128.3, 128.2, 127.9, 126.7, 123.3, 121.3, 89.4, 85.8, 84.3, 69.7, 68.1, 45.7, 39.2, 36.5, 21.8, 21.7. 89.4, 85.8, 84.3, 69.7, 68.1, 45.7, 39.2, 36.5, 21.8, 21.7. HRMS (ESI) $[M+Na]^+$ calcd for $C_{35}H_{31}O_4BrNa^+$, 617.1298, found 617.1302. (Chiral IA-3, λ= 254 nm, *n*-hexane/2-propanol= 49/1, Flow rate = 1.0 mL/min), t_R = 19.432 min(major), 32.117 min.

HPLC chromatogram of racemic S1

Condition: n-hexane/2-propanol =49:1

Flow rate =1.0 mL/min

λ= 254 nm

Chiral IA-3

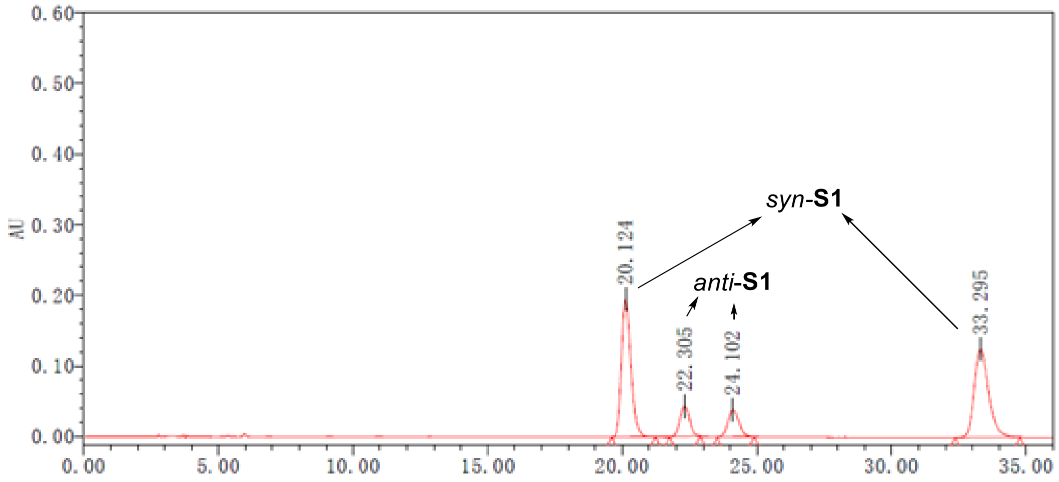

Entry	Retention Time/min	Area	Height	Area(%)
1	20.124	4594035	195267	40.42
2	22.305	1066902	43195	9.39
3	24.102	1059536	37963	9.32
4	33.295	4645914	124850	40.87

Supplementary Figure 212. Chiral HPLC analysis of racemic **S1**

HPLC chromatogram of chiral S1

Condition: n-hexane/2-propanol =49:1

Flow rate =1.0 mL/min

λ= 254 nm

Chiral IA-3

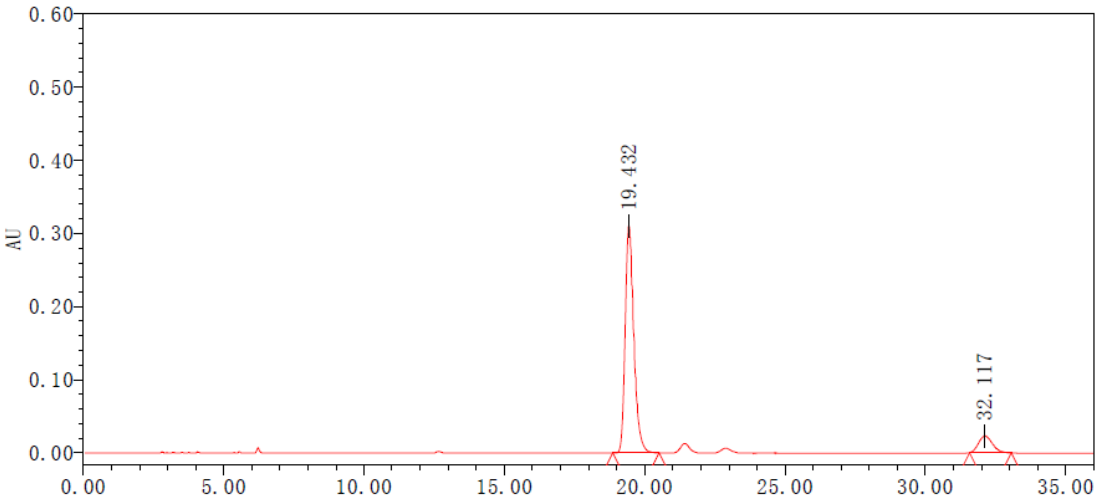

Entry	Retention Time/min	Area	Height	Area(%)
1	19.432	6748195	310324	89.53
2	32.117	789232	23019	10.47

Supplementary Figure 213. Chiral HPLC analysis of chiral **S1**

(3S')-adamantan-1-yl (2S,3R)-2-((4-bromobenzyl)oxy)-3-(2-oxo-2-phenylethyl)-2,5-diphenylpent-4-ynoate(S2)

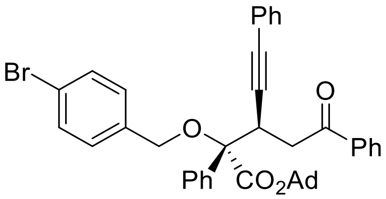

S2

Colorless oil; 57.8 mg, 55% yield, 94:6 d.r., 94% *ee*; ^{1}H NMR (400 MHz, $CDCl_3$) δ 7.88 (d, *J* = 7.2 Hz, 3H), 7.72 (d, *J* = 7.3 Hz, 2H), 7.56 (t, *J* = 7.4 Hz, 1H), 7.50 (d, *J* = 8.4 Hz, 2H), 7.47 – 7.43 (m, 2H), 7.42 – 7.37 (m, 6H), 7.38 – 7.33 (m, 2H), 7.29 (d, *J* = 9.0 Hz, 1H), 5.05 (d, *J* = 11.7 Hz, 1H), 4.88 (d, *J* = 11.7 Hz, 1H), 4.42 (dd, *J* = 10.1, 3.1 Hz, 1H), 3.77 (s, 1H), 3.34 (dd, *J* = 16.5, 10.1 Hz, 1H), 3.16 (dd, *J* = 16.5, 3.0 Hz, 1H), 2.29 (s, 1H), 2.20 (d, *J* = 9.7 Hz, 7H), 1.74 (s, 1H), 1.68 (s, 5H). ^{13}C NMR (101 MHz, $CDCl_3$) δ 197.5, 169.1, 138.3, 138.1, 137.0, 133.1, 131.6, 131.4, 131.4, 129.2, 128.6, 128.2, 128.2, 128.2, 127.8, 126.8, 123.5, 121.2, 89.7, 85.9, 84.1, 83.11, 67.9,

67.5, 67.3, 66.8, 66.2, 41.8, 41.3, 39.4, 36.8, 36.2, 36.1, 30.9. HRMS (ESI) $[M+Na]^+$ calcd for $C_{42}H_{39}O_4BrNa^+$, 709.1924, found 709.1920. (Chiral IE-3, λ= 254 nm, *n*-hexane/2-propanol= 19/1, Flow rate = 1.0 mL/min), t_R = 11.866 min(major), 23.080 min.

HPLC chromatogram of racemic S2

Condition: n-hexane/2-propanol =19:1

Flow rate =1.0 mL/min

λ= 254 nm

Chiral IE-3

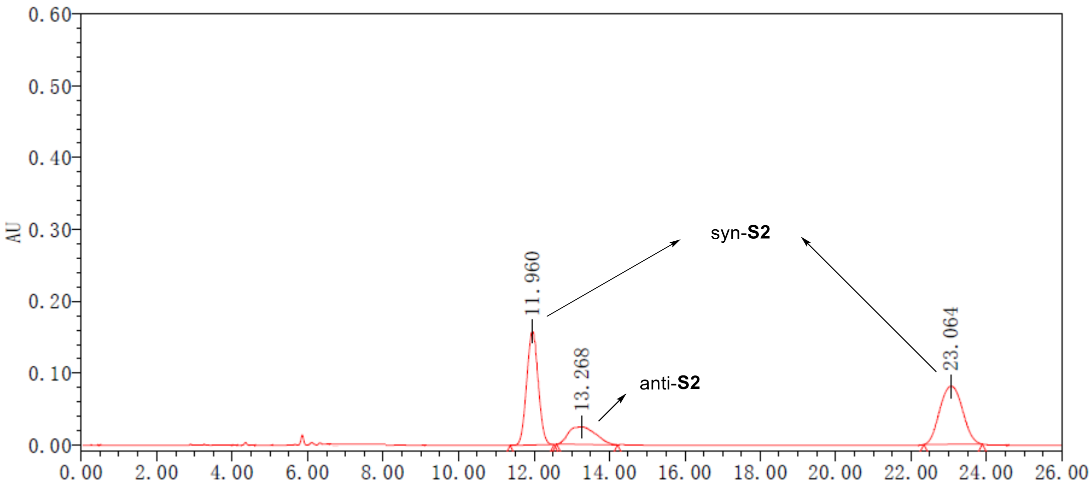

Entry	Retention Time/min	Area	Height	Area(%)
1	11.960	3399610	158056	42.40
2	13.268	1224706	24329	15.27
3	23.064	3394051	81034	42.33

Supplementary Figure 214. Chiral HPLC analysis of racemic **S2**

HPLC chromatogram of chiral S2

Condition: n-hexane/2-propanol =19:1

Flow rate =1.0 mL/min

λ= 254 nm

Chiral IE-3

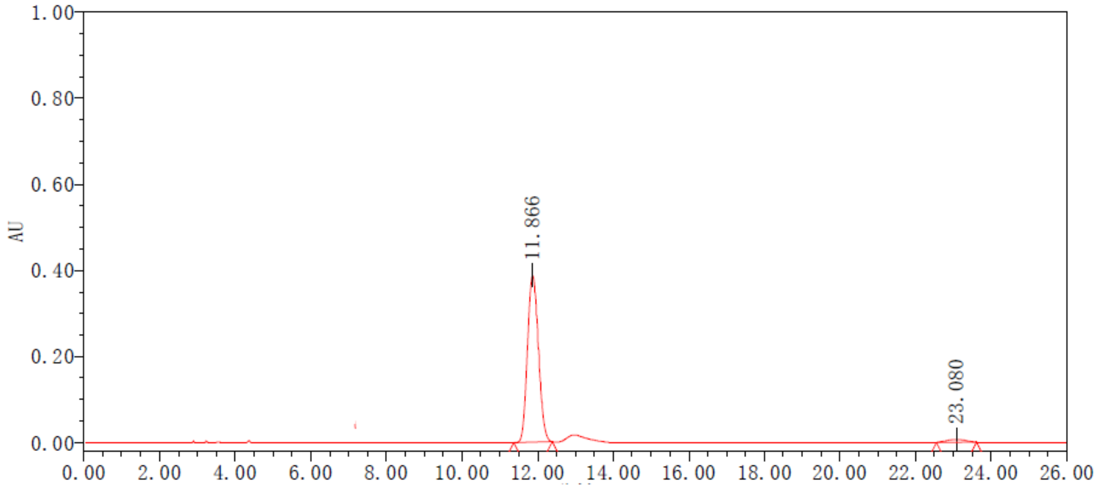

Entry	Retention Time/min	Area	Height	Area(%)
1	11.866	7844491	387110	97.22
2	23.080	224168	6254	2.78

Supplementary Figure 215. Chiral HPLC analysis of chiral **S2**

Tert-butyl (2S,3R)-2-((4-iodobenzyl)oxy)-3-(2-oxo-2-phenylethyl)-2,5-diphenylpent-4-ynoate (S3)

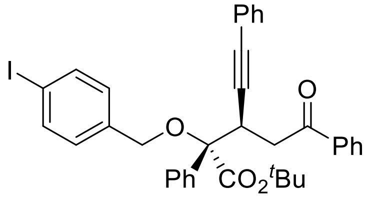

S3

Colorless oil; 52.8 mg, 53% yield, >20:1 d.r., 98% *ee*; ^{1}H NMR (400 MHz, $CDCl_3$) δ 7.87 – 7.82 (m, 2H), 7.68 (dd, *J* = 8.1, 1.6 Hz, 4H), 7.53 (t, *J* = 7.4 Hz, 1H), 7.41 (t, *J* = 7.8 Hz, 2H), 7.37 (t, *J* = 7.4 Hz, 2H), 7.32 (d, *J* = 7.1 Hz, 1H), 7.26 (d, *J* = 6.0 Hz, 2H), 7.24 (d, *J* = 5.7 Hz, 5H), 5.04 (d, *J* = 11.8 Hz, 1H), 4.90 (d, *J* = 11.8 Hz, 1H), 4.41 (dd, *J* = 10.2, 3.1 Hz, 1H), 3.32 (dd, *J* = 16.7, 10.2 Hz, 1H), 3.08 (dd, *J* = 16.6, 3.0 Hz, 1H), 1.51 (s, 9H). ^{13}C NMR (101 MHz, $CDCl_3$) δ 197.4, 169.4, 138.7, 138.3, 137.4,

136.9, 133.1, 131.4, 129.5, 128.6, 128.3, 128.3, 128.2, 128.2, 128.2, 127.9, 126.7, 123.4, 92.8, 89.7, 85.9, 84.1, 83.0, 68.1, 39.2, 36.6, 28.1. HRMS (ESI) $[M+Na]^+$ calcd for $C_{36}H_{33}O_4INa^+$, 679.1316, found 679.1312. (Chiral IE-3, λ= 254 nm, *n*-hexane/2-propanol= 9/1, Flow rate = 1.0 mL/min), t_R = 7.571 min(major), 13.849 min.

HPLC chromatogram of racemic S3

Condition: n-hexane/2-propanol =9:1

Flow rate =1.0 mL/min

λ= 254 nm

Chiral IE-3

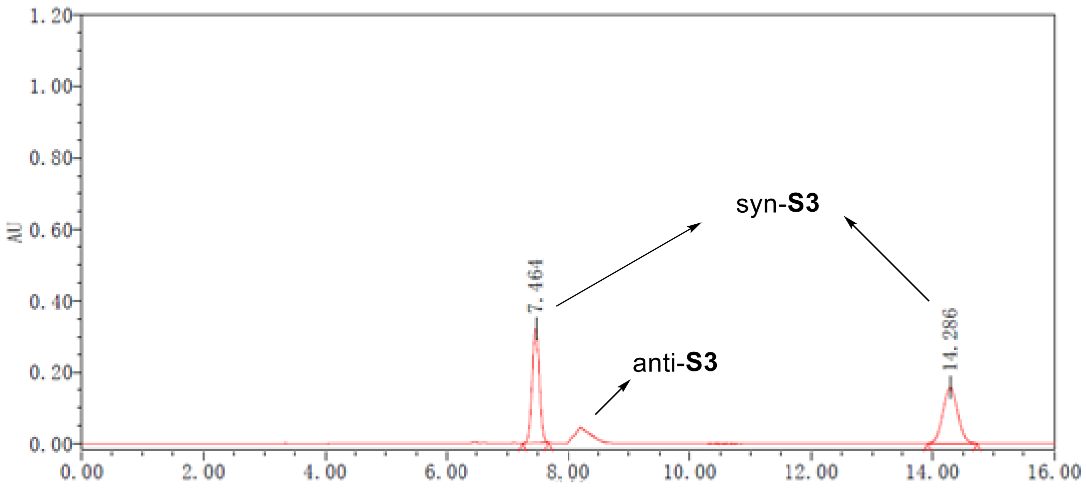

Entry	Retention Time/min	Area	Height	Area(%)
1	7.464	2651099	320271	50.20
2	14.286	2630463	155712	49.80

Supplementary Figure 216. Chiral HPLC analysis of racemic **S3**

HPLC chromatogram of chiral S3

Condition: n-hexane/2-propanol =9:1

Flow rate =1.0 mL/min

λ= 254 nm

Chiral IE-3

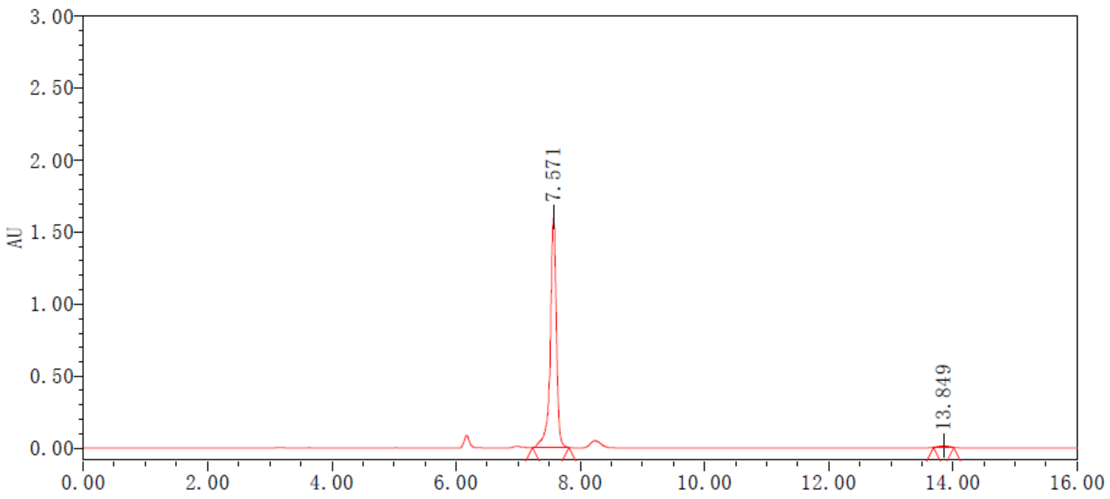

The larger version of HPLC chromatogram of chiral S3

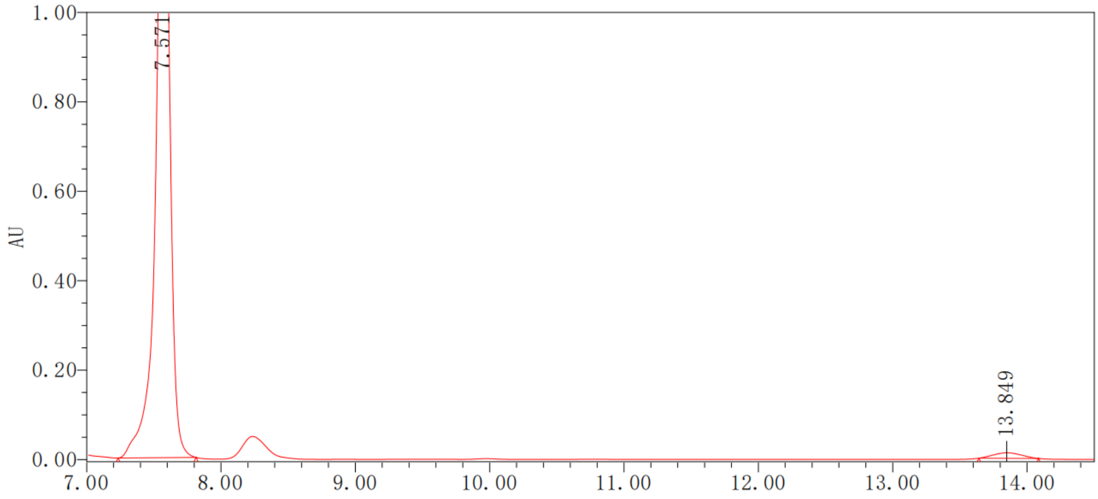

Entry	Retention Time/min	Area	Height	Area(%)
1	7.571	11058138	11058138	99.02
2	13.849	109255	109255	0.98

Supplementary Figure 217. Chiral HPLC analysis of chiral **S3**

Tert-butyl (2S,3R)-2-(naphthalen-2-ylmethoxy)-3-(2-oxo-2-phenylethyl)-2,5-diphenylpent-4-ynoate (S4)

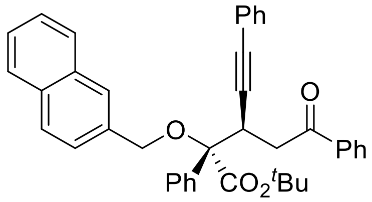

S4

Colorless oil; 55.7 mg, 64% yield, >20:1 d.r., 96% *ee*; ^{1}H NMR (400 MHz, $CDCl_3$) δ 7.93 (s, 1H), 7.86 – 7.80 (m, 4H), 7.78 (d, *J* = 3.4 Hz, 1H), 7.75 (d, *J* = 7.3 Hz, 2H), 7.67 (dd, *J* = 8.5, 1.4 Hz, 1H), 7.51 – 7.43 (m, 3H), 7.39 – 7.30 (m, 5H), 7.27 – 7.21 (m, 5H), 5.29 (d, *J* = 11.7 Hz, 1H), 5.13 (d, *J* = 11.6 Hz, 1H), 4.45 (dd, *J* = 10.2, 3.0 Hz, 1H), 3.39 (dd, *J* = 16.6, 10.3 Hz, 1H), 3.11 (dd, *J* = 16.6, 3.0 Hz, 1H), 1.53 (s, 9H). ^{13}C NMR (126 MHz, $CDCl_3$) δ 197.6, 169.6, 138.6, 136.9, 136.5, 133.3, 133.1, 133.0, 131.5, 128.5, 128.3, 128.2, 128.2, 128.2, 128.0, 128.0, 127.9, 127.7, 126.8, 126.1, 126.0, 125.9, 125.8, 123.5, 89.8, 85.9, 84.1, 82.9, 68.8, 39.2, 36.7, 28.1. HRMS (ESI) $[M+Na]^+$ calcd for $C_{40}H_{36}O_4Na^+$, 603.2506, found 603.2503. (Chiral IE-3, λ= 254 nm, *n*-hexane/2-propanol= 9/1, Flow rate = 1.0 mL/min), t_R = 9.133 min(major), 16.891 min.

HPLC chromatogram of racemic S4

Condition: n-hexane/2-propanol =9:1

Flow rate =1.0 mL/min

λ= 254 nm

Chiral IE-3

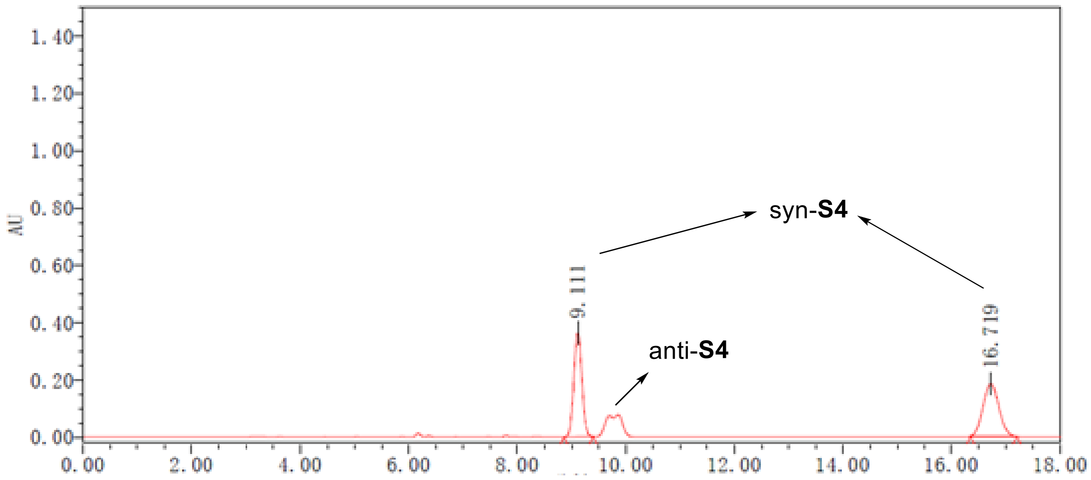

Entry	Retention Time/min	Area	Height	Area(%)
1	9.111	3709717	363477	49.66
2	16.719	3759995	184301	50.34

Supplementary Figure 218. Chiral HPLC analysis of racemic **S4**

HPLC chromatogram of chiral S4

Condition: n-hexane/2-propanol =9:1

Flow rate =1.0 mL/min

λ= 254 nm

Chiral IE-3

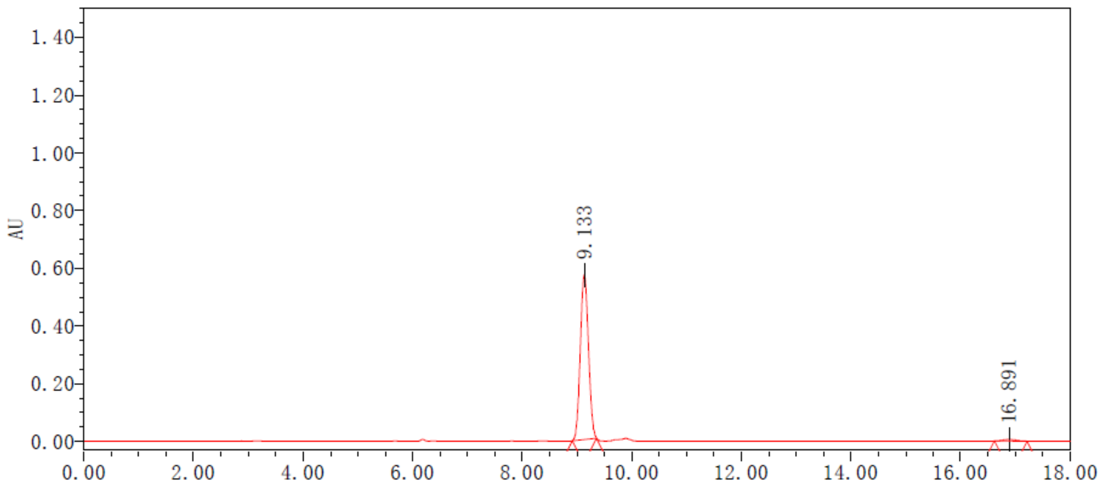

The larger version of HPLC chromatogram of chiral S4

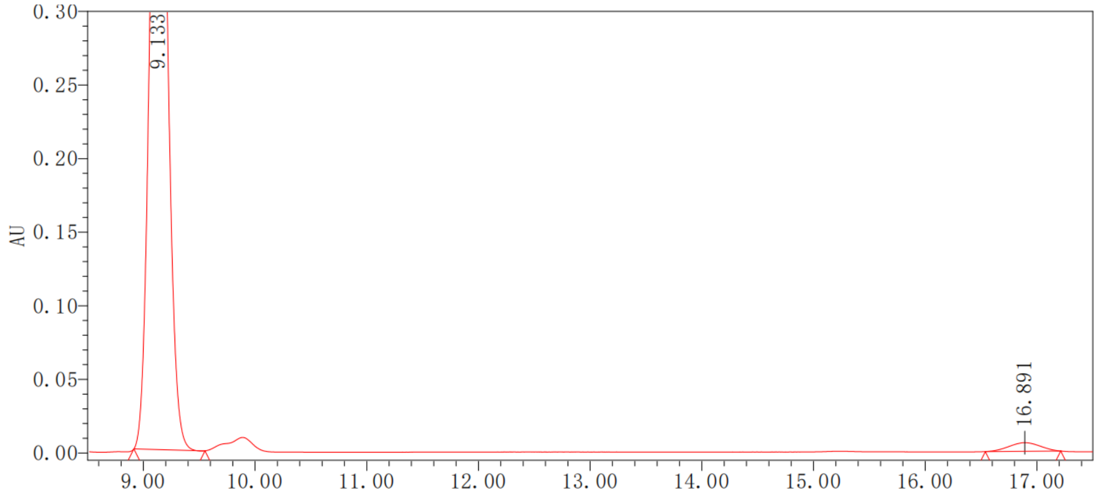

Entry	Retention Time/min	Area	Height	Area(%)
1	9.133	5809594	570886	98.29
2	16.891	100823	5493	1.71

Supplementary Figure 219. Chiral HPLC analysis of chiral **S4**

Tert-butyl (2S,3R)-2-((5-bromothiophen-2-yl)methoxy)-3-(2-oxo-2-phenylethyl)-2,5-diphenylpent-4-ynoate(S5)

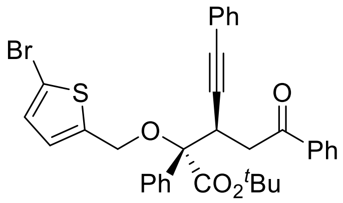

S5

Colorless oil; 54.8 mg, 59% yield, >20:1 d.r., 98% *ee*; ^{1}H NMR (400 MHz, $CDCl_3$) δ 7.90 – 7.86 (m, 2H), 7.70 – 7.65 (m, 2H), 7.55 – 7.50 (m, 1H), 7.42 (t, *J* = 7.6 Hz, 2H), 7.37 (t, *J* = 7.4 Hz, 2H), 7.31 (d, *J* = 7.1 Hz, 1H), 7.26 – 7.20 (m, 5H), 6.92 (d, *J* = 3.7 Hz, 1H), 6.78 (d, *J* = 3.7 Hz, 1H), 5.25 (d, *J* = 11.9 Hz, 1H), 5.05 (d, *J* = 11.9 Hz, 1H), 4.40 (dd, *J* = 10.3, 2.9 Hz, 1H), 3.37 (dd, *J* = 16.7, 10.3 Hz, 1H), 3.06 (dd, *J* = 16.7, 2.9 Hz, 1H), 1.53 (s, 9H). ^{13}C NMR (126 MHz, $CDCl_3$) δ 197.4, 169.2, 143.8, 138.1, 136.9, 133.2, 131.4, 129.2, 128.6, 128.4, 128.3, 128.2, 128.2, 127.9, 126.6, 125.6, 123.3, 112.3, 89.5, 86.0, 84.2, 83.1, 64.4, 39.1, 36.4, 28.1. HRMS (ESI) $[M+Na]^+$ calcd for $C_{34}H_{31}O_4SNa^+$, 637.1022, found 637.1019. (Chiral IE-3, λ= 254 nm, *n*-hexane/2-propanol= 49/1, Flow rate = 1.0 mL/min), t_R = 11.816 min(major), 26.455 min.

HPLC chromatogram of racemic S5

Condition: n-hexane/2-propanol =49:1

Flow rate =1.0 mL/min

λ= 254 nm

Chiral IE-3

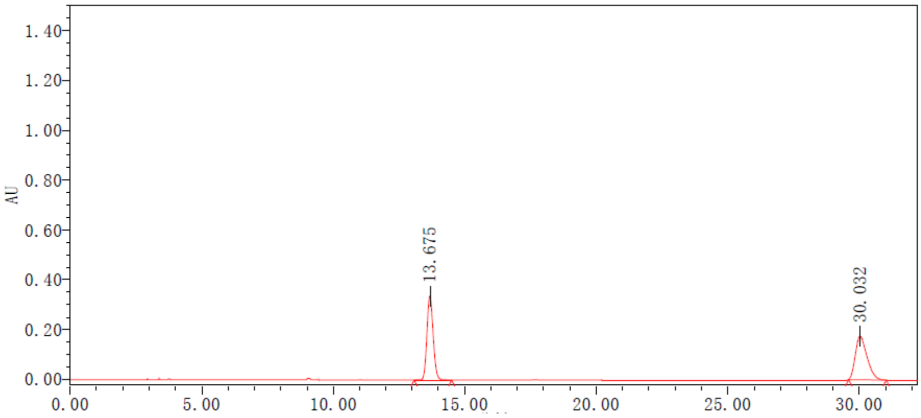

Entry	Retention Time/min	Area	Height	Area(%)
1	13.675	5450368	336283	50.13
2	30.032	5422602	175098	49.87

Supplementary Figure 220. Chiral HPLC analysis of racemic **S5**

HPLC chromatogram of chiral S5

Condition: n-hexane/2-propanol =49:1

Flow rate =1.0 mL/min

λ= 254 nm

Chiral IE-3

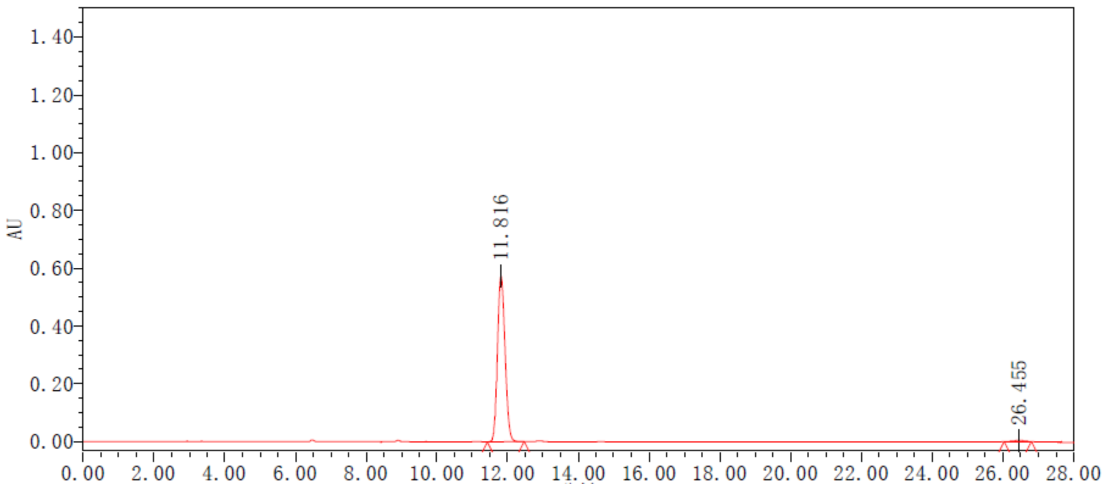

The larger version of HPLC chromatogram of chiral S5

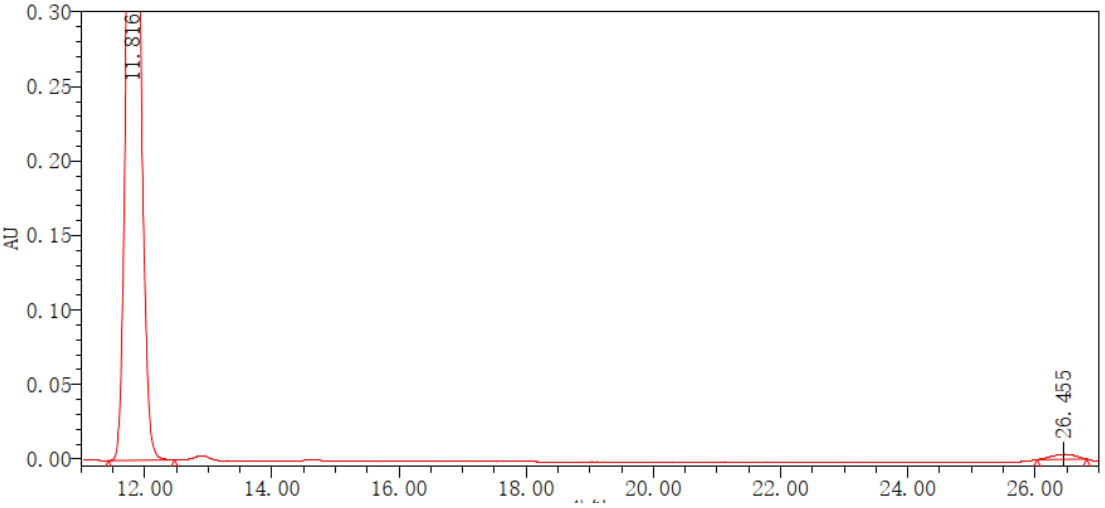

Entry	Retention Time/min	Area	Height	Area(%)
1	11.816	8420273	574023	98.91
2	26.455	92707	3488	1.09

Supplementary Figure 221. Chiral HPLC analysis of chiral **S5**

Tert-butyl (2S,3R)-3-(2-oxo-2-phenylethyl)-2,5-diphenyl-2-(((E)-3-(p-tolyl)allyl)oxy)pent-4-ynoate (S6)

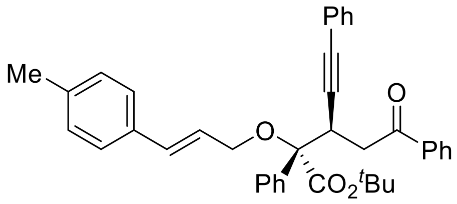

S6

Colorless oil; 57.3 mg, 67% yield, >20:1 d.r., 95% *ee*; ^{1}H NMR (400 MHz, $CDCl_3$) δ 7.94 (d, *J* = 7.7 Hz, 2H), 7.75 (d, *J* = 7.7 Hz, 2H), 7.55 (t, *J* = 7.3 Hz, 1H), 7.42 (dd, *J* = 17.1, 7.9 Hz, 4H), 7.37 – 7.31 (m, 3H), 7.28 (dd, *J* = 7.9, 3.6 Hz, 5H), 7.16 (d, *J* = 7.8 Hz, 2H), 6.71 (d, *J* = 15.9 Hz, 1H), 6.42 (dt, *J* = 15.9, 5.7 Hz, 1H), 4.72 (dd, *J* = 12.5, 6.1 Hz, 1H), 4.58 (dd, *J* = 12.4, 5.1 Hz, 1H), 4.40 (dd, *J* = 10.1, 2.8 Hz, 1H), 3.42 (dd, *J* = 16.6, 10.2 Hz, 1H), 3.19 (dd, *J* = 16.6, 2.7 Hz, 1H), 2.37 (s, 3H), 1.57 (s, 9H). ^{13}C NMR (126 MHz, $CDCl_3$) δ 197.7, 169.6, 138.6, 137.4, 137.0, 134.2, 133.1, 131.5, 131.4, 129.3, 128.5, 128.3, 128.2, 128.1, 127.8, 126.8, 126.5, 125.6, 123.5, 89.8, 85.7, 84.0, 82.7, 67.8, 39.3, 36.7, 28.1, 21.3. HRMS (ESI) $[M+Na]^+$ calcd for $C_{39}H_{38}O_4Na^+$, 593.2662, found 593.2660. (Chiral IE-3, λ= 254 nm, *n*-hexane/2-propanol= 19/1, Flow rate = 1.0 mL/min), t_R = 11.699 min(major), 22.829 min.

HPLC chromatogram of racemic S6

Condition: n-hexane/2-propanol =19:1

Flow rate =1.0 mL/min

λ= 254 nm

Chiral IE-3

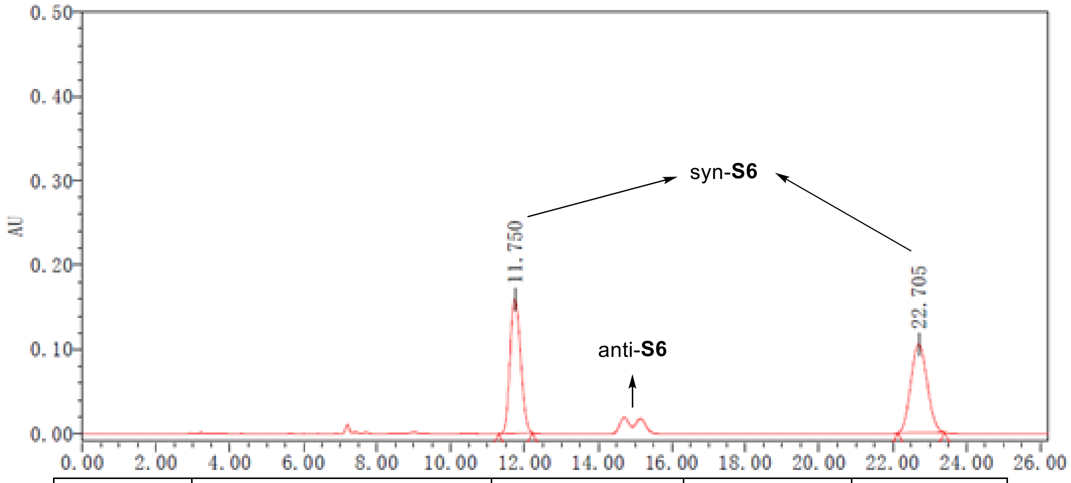

Entry	Retention Time/min	Area	Height	Area(%)
1	11.750	3169783	159177	49.73
2	22.705	3203819	104988	50.27

Supplementary Figure 222. Chiral HPLC analysis of racemic **S6**

HPLC chromatogram of chiral S6

Condition: n-hexane/2-propanol =19:1

Flow rate =1.0 mL/min

λ= 254 nm

Chiral IE-3

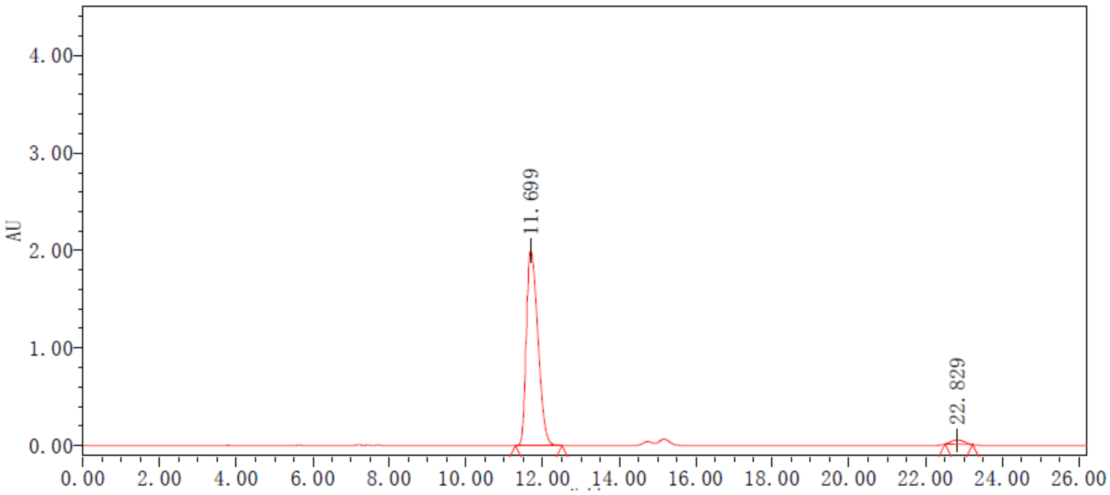

The larger version of HPLC chromatogram of chiral S6

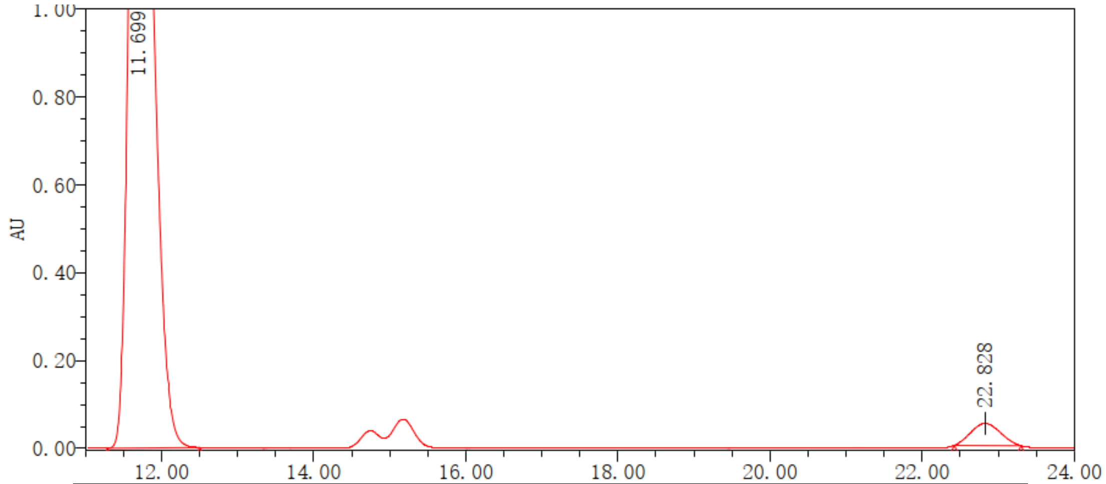

Entry	Retention Time/min	Area	Height	Area(%)
1	11.699	42219463	1996671	97.52
2	22.829	1071593	43996	2.48

Supplementary Figure 223. Chiral HPLC analysis of chiral **S6**

Tert-butyl (2S,3R)-2-(((E)-3-(4-chlorophenyl)allyl)oxy)-3-(2-oxo-2-phenylethyl)-2,5-diphenylpent-4-ynoate (S7)

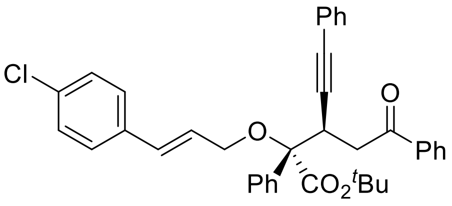

S7

Colorless oil; 48.7 mg, 55% yield, >20:1 d.r., 96% *ee*; ^{1}H NMR (400 MHz, $CDCl_3$) δ 7.90 (d, *J* = 7.8 Hz, 2H), 7.70 (d, *J* = 7.8 Hz, 2H), 7.52 (t, *J* = 7.3 Hz, 1H), 7.39 (dd, *J* = 16.5, 8.0 Hz, 4H), 7.30 (dd, *J* = 15.9, 6.2 Hz, 4H), 7.24 (d, *J* = 8.7 Hz, 5H), 6.66 (d, *J* = 16.0 Hz, 1H), 6.39 (dt, *J* = 15.9, 5.5 Hz, 1H), 4.67 (dd, *J* = 12.9, 5.8 Hz, 1H), 4.53 (dd, *J* = 12.9, 5.1 Hz, 1H), 4.37 (dd, *J* = 10.0, 2.9 Hz, 1H), 3.36 (dd, *J* = 16.6, 10.1 Hz, 1H), 3.16 (dd, *J* = 16.6, 2.9 Hz, 1H), 1.53 (s, 9H). ^{13}C NMR (126 MHz, $CDCl_3$) δ 197.5, 169.5, 138.4, 137.0, 135.5, 133.1, 131.4, 130.2, 128.7, 128.6, 128.2, 128.2, 127.8, 127.7, 127.4, 126.8, 123.5, 89.7, 85.8, 84.0, 82.8, 67.4, 39.3, 36.7, 28.1. Peak overlapping was observed. HRMS (ESI) $[M+Na]^+$ calcd for $C_{38}H_{35}O_4ClNa^+$, 613.2116 found 613.2117. (Chiral IA, λ= 254 nm, *n*-hexane/2-propanol= 49/1, Flow rate = 1.0 mL/min), t_R = 14.402 min(major), 26.664 min.

HPLC chromatogram of racemic S7

Condition: n-hexane/2-propanol =49:1

Flow rate =1.0 mL/min

λ= 254 nm

Chiral IA

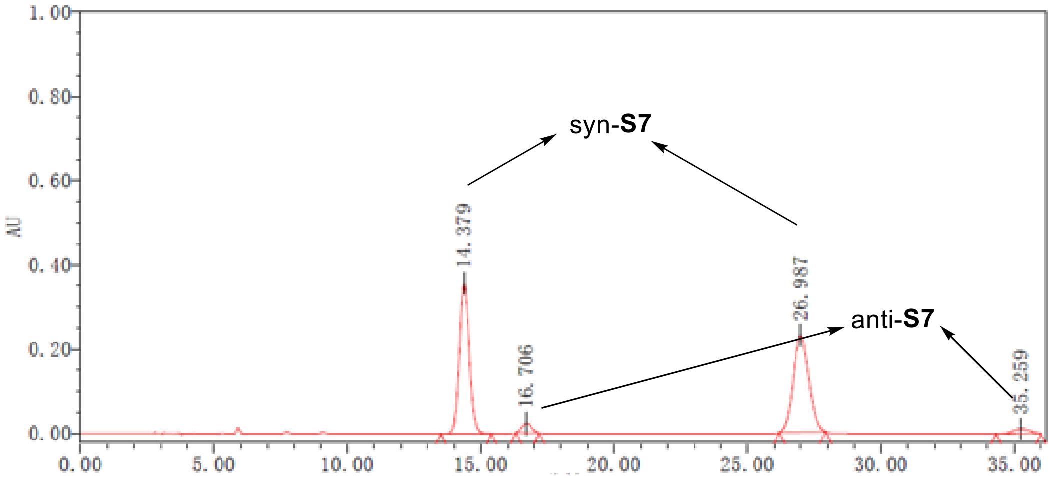

Entry	Retention Time/min	Area	Height	Area(%)
1	14.379	9188676	357265	46.81
2	16.706	561913	21788	2.86
3	26.987	9386302	230325	47.81
4	35.259	493876	11178	2.52

Supplementary Figure 224. Chiral HPLC analysis of racemic **S7**

HPLC chromatogram of chiral S7

Condition: n-hexane/2-propanol =49:1

Flow rate =1.0 mL/min

λ= 254 nm

Chiral IA

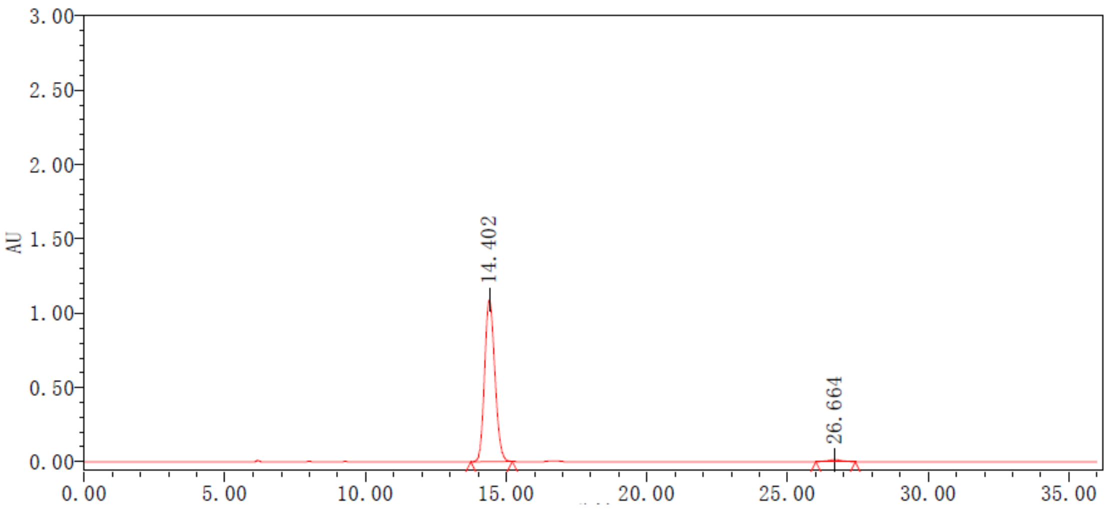

The larger version of HPLC chromatogram of chiral S7

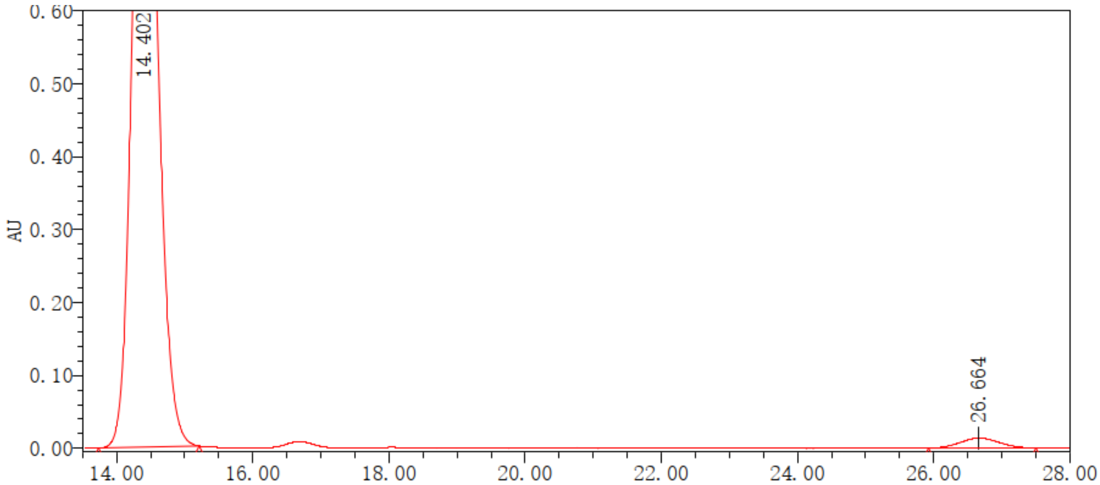

Entry	Retention Time/min	Area	Height	Area(%)
1	14.402	27235904	1088645	98.15
2	26.664	514553	13291	1.85

Supplementary Figure 225. Chiral HPLC analysis of chiral **S7**

Tert-butyl (2S,3R)-2-(((2E,5E)-hepta-2,5-dien-1-yl)oxy)-3-(2-oxo-2-phenylethyl)-2,5-diphenylpent-4-ynoate (S8)

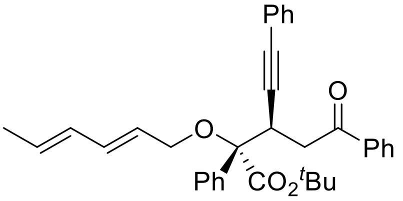

S8

Colorless oil; 47.6 mg, 61% yield, >20:1 d.r., 96% *ee*; ^{1}H NMR (400 MHz, $CDCl_3$) δ 7.92 – 7.86 (m, 2H), 7.70 – 7.66 (m, 2H), 7.53 (dd, *J* = 8.3, 6.4 Hz, 1H), 7.42 (t, *J* = 7.6 Hz, 2H), 7.35 (t, *J* = 7.4 Hz, 2H), 7.30 (d, *J* = 7.2 Hz, 1H), 7.23 (d, *J* = 13.3 Hz, 5H), 6.31 (dd, *J* = 15.2, 10.5 Hz, 1H), 6.10 (ddd, *J* = 14.7, 10.5, 1.3 Hz, 1H), 5.82 – 5.74 (m, 1H), 5.68 (dt, *J* = 13.6, 6.8 Hz, 1H), 4.54 (dd, *J* = 12.4, 6.3 Hz, 1H), 4.40 (dd, *J* = 12.4, 5.5 Hz, 1H), 4.31 (dd, *J* = 10.3, 3.0 Hz, 1H), 3.34 (dd, *J* = 16.6, 10.3 Hz, 1H), 3.11 (dd, *J* = 16.6, 3.0 Hz, 1H), 1.76 (d, *J* = 6.6 Hz, 3H), 1.51 (s, 9H). ^{13}C NMR (101 MHz, $CDCl_3$) δ 197.7, 169.6, 138.7, 137.0, 133.0, 132.3, 131.4, 131.1, 129.7, 128.5, 128.3, 128.1, 128.1, 128.1, 127.8, 127.3, 126.8, 123.5, 89.8, 85.6, 83.9, 82.6, 67.4, 39.3, 36.7, 28.1, 18.2. HRMS (ESI) $[M+Na]^+$ calcd for $C_{35}H_{36}O_4Na^+$, 543.2506, found 543.2511. (Chiral IE-3, λ= 254 nm, *n*-hexane/2-propanol= 19/1, Flow rate = 1.0 mL/min), t_R = 12.180 min(major), 29.777 min.

HPLC chromatogram of racemic S8

Condition: n-hexane/2-propanol =19:1

Flow rate =1.0 mL/min

λ= 254 nm

Chiral IE-3

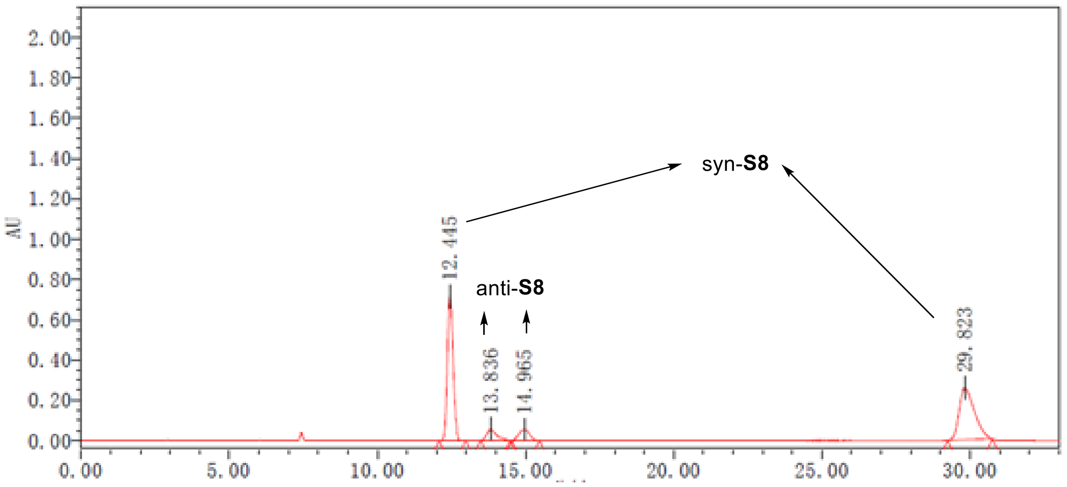

Entry	Retention Time/min	Area	Height	Area(%)
1	12.445	9526285	714973	44.16
2	13.836	1264612	54689	5.86
3	14.965	1295139	53772	6.00
4	29.823	9488227	258792	43.98

Supplementary Figure 226. Chiral HPLC analysis of racemic **S8**

HPLC chromatogram of chiral S8

Condition: n-hexane/2-propanol =19:1

Flow rate =1.0 mL/min

λ= 254 nm

Chiral IE-3

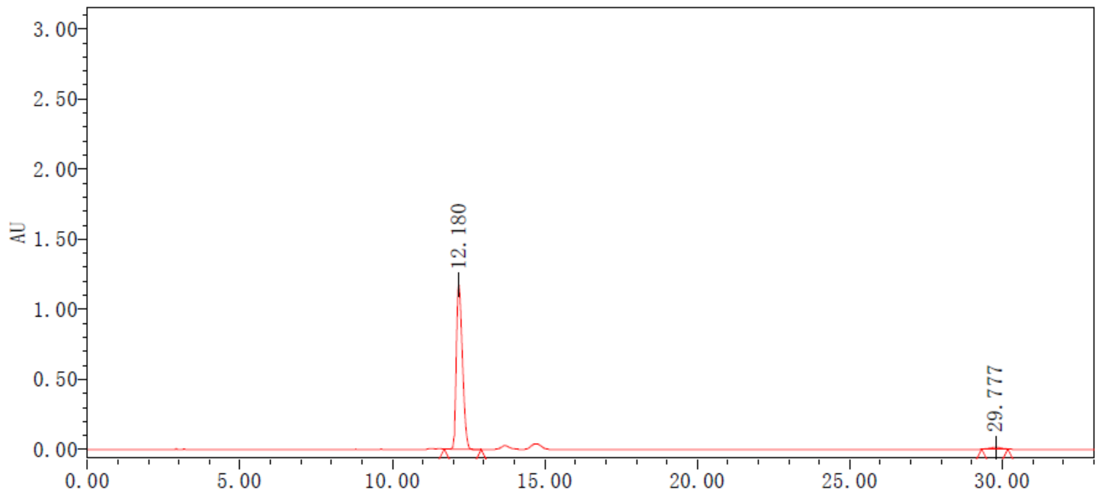

The larger version of HPLC chromatogram of chiral S8

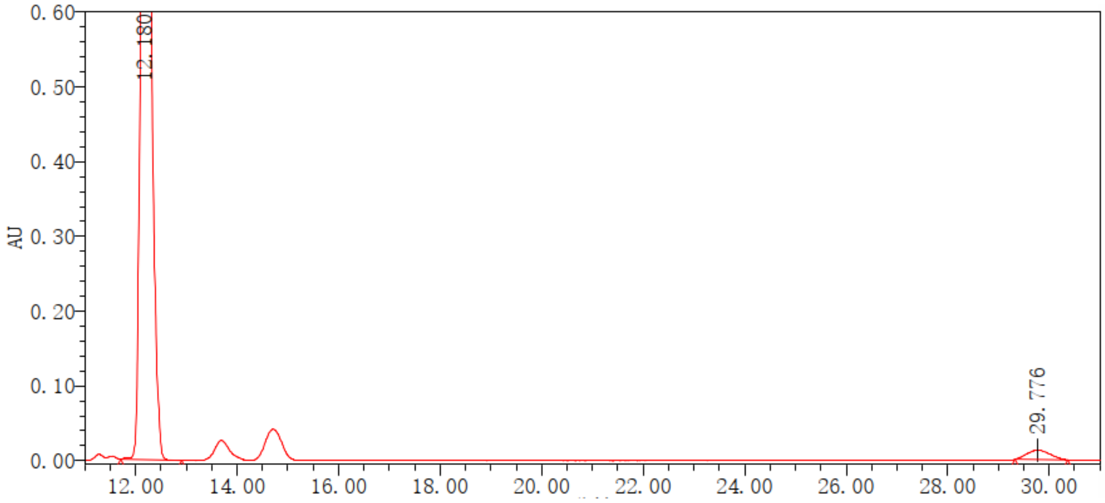

Entry	Retention Time/min	Area	Height	Area(%)
1	12.180	16129059	1172400	98.07
2	29.776	316675	11032	1.93

Supplementary Figure 227. Chiral HPLC analysis of chiral **S8**

Tert-butyl (2S,3R)-2-butoxy-3-(2-oxo-2-phenylethyl)-2,5-diphenylpent-4-ynoate (S9)

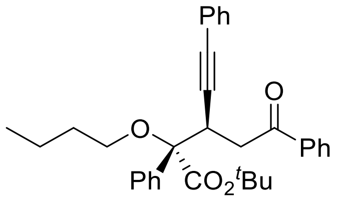

S9

Colorless oil; 44.6 mg, 60% yield, >20:1 d.r., 92% *ee*; ^{1}H NMR (400 MHz, $CDCl_3$) δ 7.92 – 7.87 (m, 2H), 7.68 – 7.65 (m, 2H), 7.56 – 7.51 (m, 1H), 7.42 (dd, *J* = 10.4, 4.8 Hz, 2H), 7.38 – 7.32 (m, 2H), 7.30 (dt, *J* = 4.8, 1.9 Hz, 1H), 7.27 – 7.21 (m, 5H), 4.30 (dt, *J* = 5.7, 3.1 Hz, 1H), 3.96 (dt, *J* = 8.8, 6.5 Hz, 1H), 3.77 (dt, *J* = 8.8, 6.4 Hz, 1H), 3.27 (dd, *J* = 16.6, 9.9 Hz, 1H), 3.16 (dd, *J* = 16.6, 3.3 Hz, 1H), 1.73 – 1.64 (m, 2H), 1.56 (d, *J* = 12.4 Hz, 2H), 1.51 (s, 9H), 0.95 (t, *J* = 7.4 Hz, 3H). ^{13}C NMR (101 MHz, $CDCl_3$) δ 197.7, 169.7, 139.0, 137.1, 133.0, 131.4, 128.5, 128.2, 128.1, 128.0, 127.9, 127.7, 126.8, 123.6, 89.8, 85.2, 83.8, 82.4, 66.1, 39.4, 36.4, 32.6, 28.1, 19.5, 14.1. HRMS (ESI) $[M+Na]^+$ calcd for $C_{33}H_{36}O_4Na^+$, 519.2506, found 519.2506. (Chiral IC, λ= 254 nm, *n*-hexane/2-propanol= 49/1, Flow rate = 1.0 mL/min), t_R = 6.216 min(major), 10.255 min.

HPLC chromatogram of racemic S9

Condition: n-hexane/2-propanol =49:1

Flow rate =1.0 mL/min

λ= 254 nm

Chiral IC

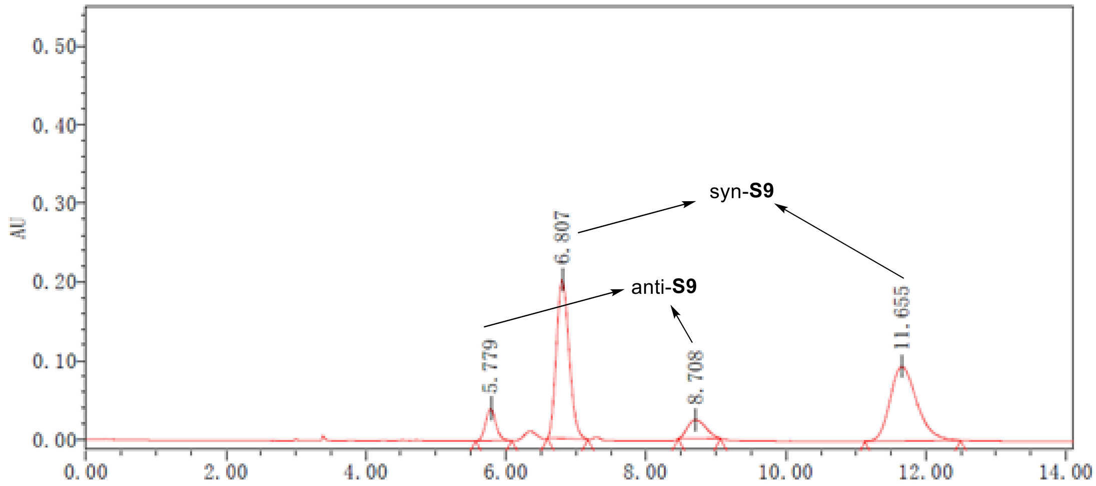

Entry	Retention Time/min	Area	Height	Area(%)
1	5.779	424595	40448	7.25
2	6.807	2499194	201354	42.69
3	8.708	437991	23845	7.48
4	11.655	2492428	94442	42.57

Supplementary Figure 228. Chiral HPLC analysis of racemic **S9**

HPLC chromatogram of chiral S9

Condition: n-hexane/2-propanol =49:1

Flow rate =1.0 mL/min

λ= 254 nm

Chiral IC

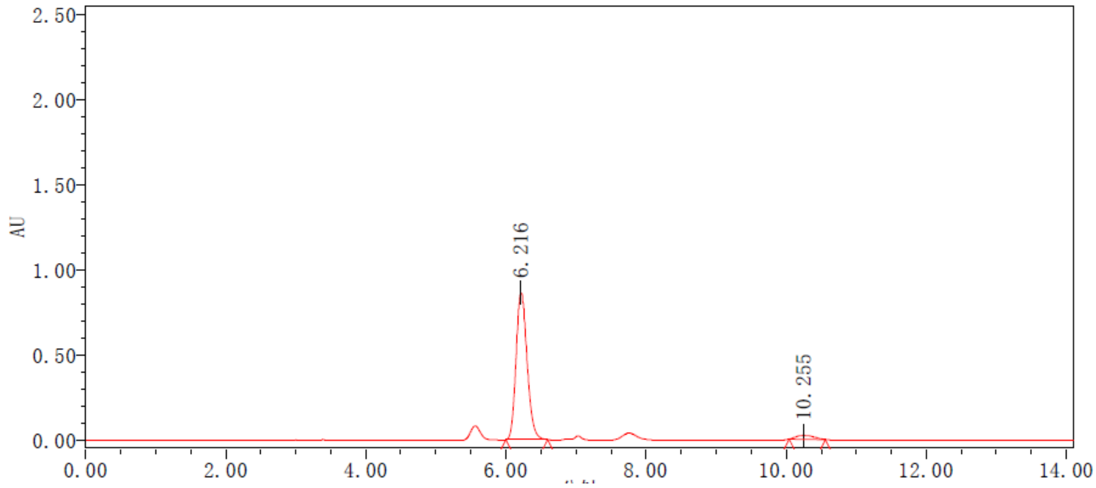

Entry	Retention Time/min	Area	Height	Area(%)
1	6.216	9553422	865316	95.99
2	10.255	398966	22998	4.01

Supplementary Figure 229. Chiral HPLC analysis of chiral **S9**

Tert-butyl (2S,3R)-2-((4-bromobenzyl)oxy)-2-(4-bromophenyl)-3-(2-oxo-2-phenylethyl)-5-phenylpent-4-ynoate (S10)

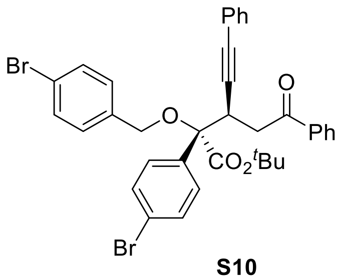

S10

Colorless oil; 66.9 mg, 65% yield, >20:1 d.r., 98% *ee*; ^{1}H NMR (400 MHz, $CDCl_3$) δ 7.87 (d, *J* = 7.7 Hz, 2H), 7.60 (d, *J* = 8.5 Hz, 2H), 7.56 (d, *J* = 6.4 Hz, 1H), 7.53 – 7.49 (m, 3H), 7.45 (t, *J* = 7.5 Hz, 3H), 7.38 (d, *J* = 8.0 Hz, 2H), 7.29 (d, *J* = 12.4 Hz, 5H), 5.04 (d, *J* = 11.6 Hz, 1H), 4.91 (d, *J* = 11.6 Hz, 1H), 4.42 (dd, *J* = 9.7, 2.4 Hz, 1H), 3.34 (dd, *J* = 16.6, 9.9 Hz, 1H), 3.11 (dd, *J* = 16.6, 2.3 Hz, 1H), 1.54 (s, 9H). ^{13}C NMR (126 MHz, $CDCl_3$) δ 197.2, 169.0, 137.7, 137.5, 136.8, 133.3, 131.5, 131.4, 131.4, 129.2, 128.6, 128.6, 128.3, 128.2, 128.1, 123.2, 122.6, 121.4, 89.2, 85.5, 84.4, 83.3, 68.0, 39.2, 36.4, 28.1. HRMS (ESI) $[M+Na]^+$ calcd for $C_{36}H_{32}O_4Br_2Na^+$, 709.0560, found

709.0556. (Chiral IB-3, λ= 254 nm, *n*-hexane/2-propanol= 19/1, Flow rate = 1.0 mL/min), t_R = 7.844 min(major), 19.889 min.

HPLC chromatogram of racemic S10

Condition: n-hexane/2-propanol =19:1

Flow rate =1.0 mL/min

λ= 254 nm

Chiral IB-3

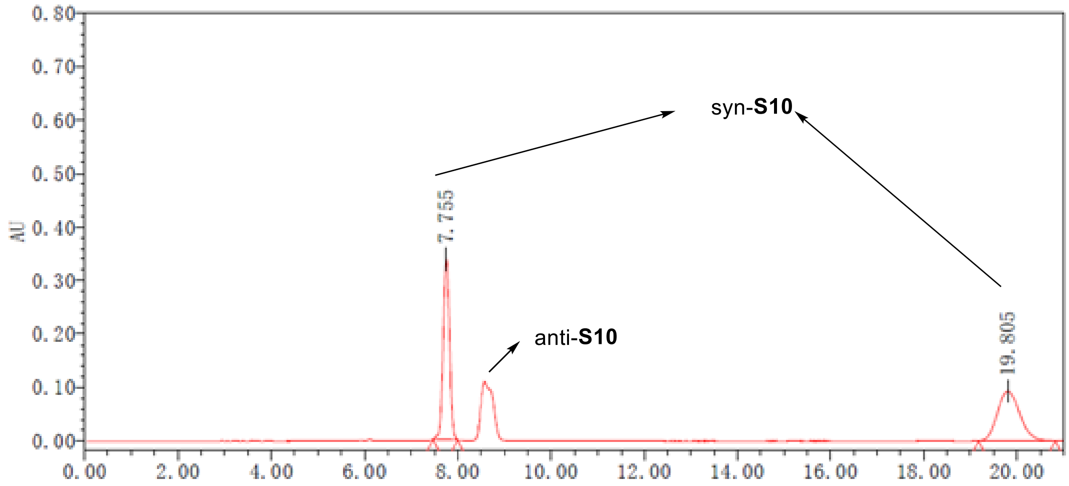

Entry	Retention Time/min	Area	Height	Area(%)
1	7.755	3102464	336655	50.22
2	19.805	3074689	92541	49.78

Supplementary Figure 230. Chiral HPLC analysis of racemic **S10**

HPLC chromatogram of chiral S10

Condition: n-hexane/2-propanol =19:1

Flow rate =1.0 mL/min

λ= 254 nm

Chiral IB-3

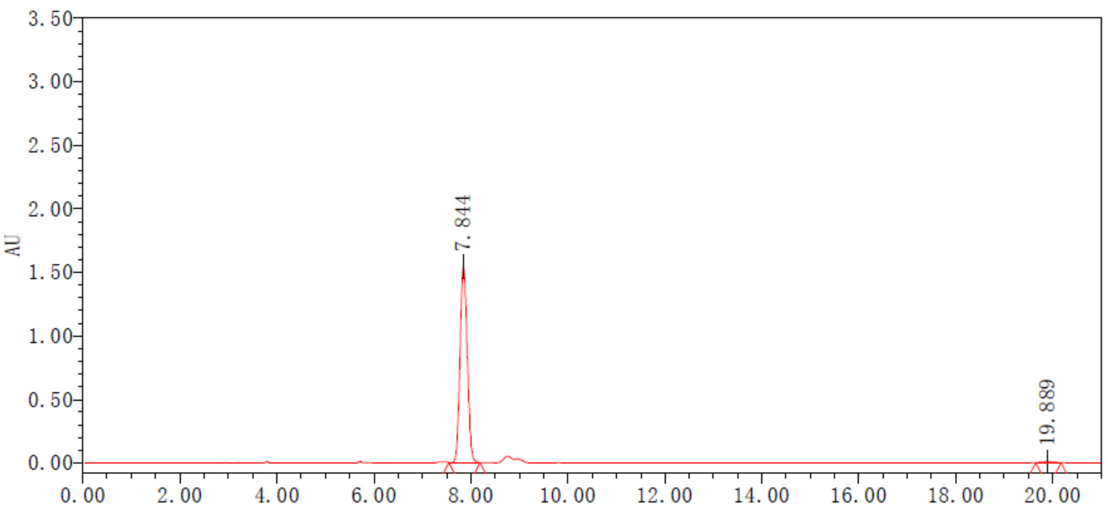

The larger version of HPLC chromatogram of chiral S10

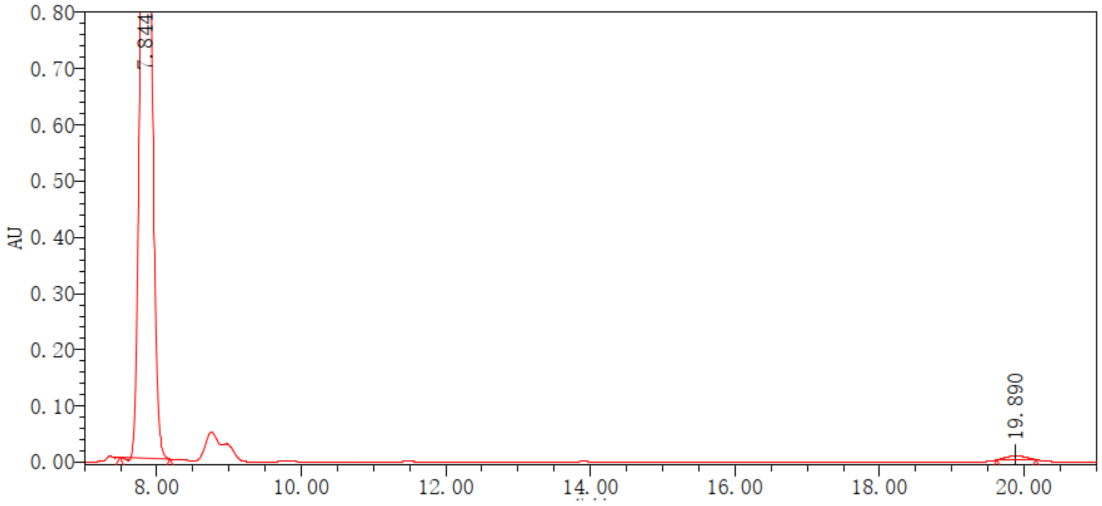

Entry	Retention Time/min	Area	Height	Area(%)
1	7.844	7.844	1540462	99.26
2	19.889	19.889	6348	0.74

Supplementary Figure 231. Chiral HPLC analysis of chiral **S10**

Tert-butyl (2S,3R)-2-((4-bromobenzyl)oxy)-2-(4-chlorophenyl)-3-(2-oxo-2-phenylethyl)-5-phenylpent-4-ynoate (S11)

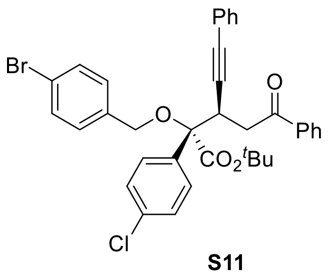

S11

Colorless oil; 59.7 mg, 62% yield, >20:1 d.r., 99% *ee*; ^{1}H NMR (400 MHz, $CDCl_3$) δ 7.94 – 7.90 (m, 2H), 7.55 (dd, *J* = 10.5, 4.3 Hz, 1H), 7.46 – 7.40 (m, 3H), 7.30 (dd, *J* = 7.2, 3.7 Hz, 2H), 7.28 – 7.24 (m, 8H), 7.20 (d, *J* = 8.4 Hz, 2H), 4.93 (d, *J* = 11.4 Hz, 1H), 4.72 (d, *J* = 11.4 Hz, 1H), 3.91 (dd, *J* = 8.6, 4.0 Hz, 1H), 3.65 (dd, *J* = 17.1, 4.0 Hz, 1H), 3.35 (t, *J* = 4.2 Hz, 1H), 1.48 (s, 9H). ^{13}C NMR (101 MHz, $CDCl_3$) δ 197.5, 169.7, 137.2, 136.9, 134.2, 133.2, 132.3, 132.0, 131.5, 131.4, 131.4, 129.1, 128.6, 128.3, 128.2, 128.1, 121.4, 121.3, 89.0, 84.4, 82.8, 66.8, 39.1, 34.0, 28.2. HRMS (ESI) $[M+Na]^+$ calcd for $C_{36}H_{32}O_4ClBrNa^+$, 665.1065, found 665.1063. (Chiral IE-3, λ= 254 nm, *n*-hexane/2-propanol= 19/1, Flow rate = 1.0 mL/min), t_R = 7.627 min(major), 18.483 min.

HPLC chromatogram of racemic S11

Condition: n-hexane/2-propanol =19:1

Flow rate =1.0 mL/min

λ= 254 nm

Chiral IE-3

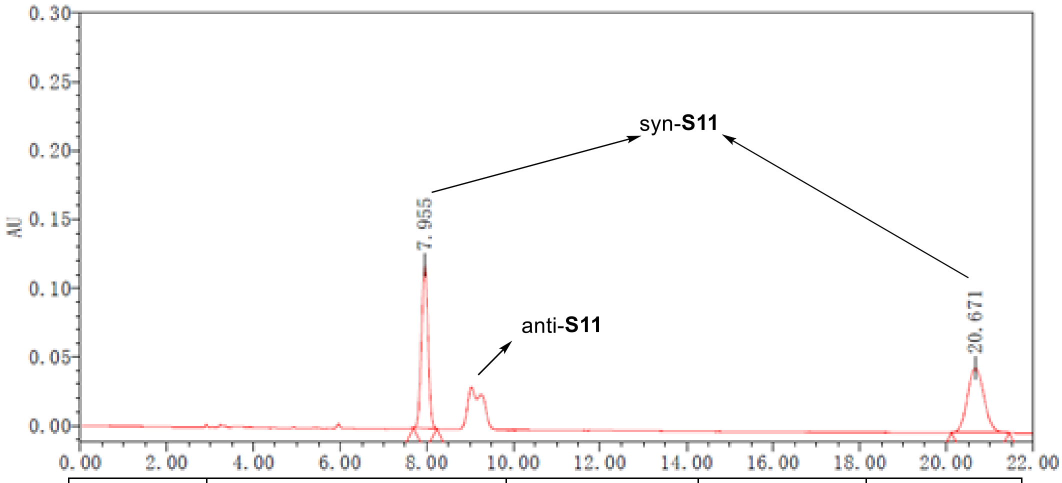

Entry	Retention Time/min	Area	Height	Area(%)
1	7.955	1251355	118414	50.23
2	20.671	1239917	46930	49.77

Supplementary Figure 232. Chiral HPLC analysis of racemic **S11**

HPLC chromatogram of chiral S11

Condition: n-hexane/2-propanol =19:1

Flow rate =1.0 mL/min

λ= 254 nm

Chiral IE-3

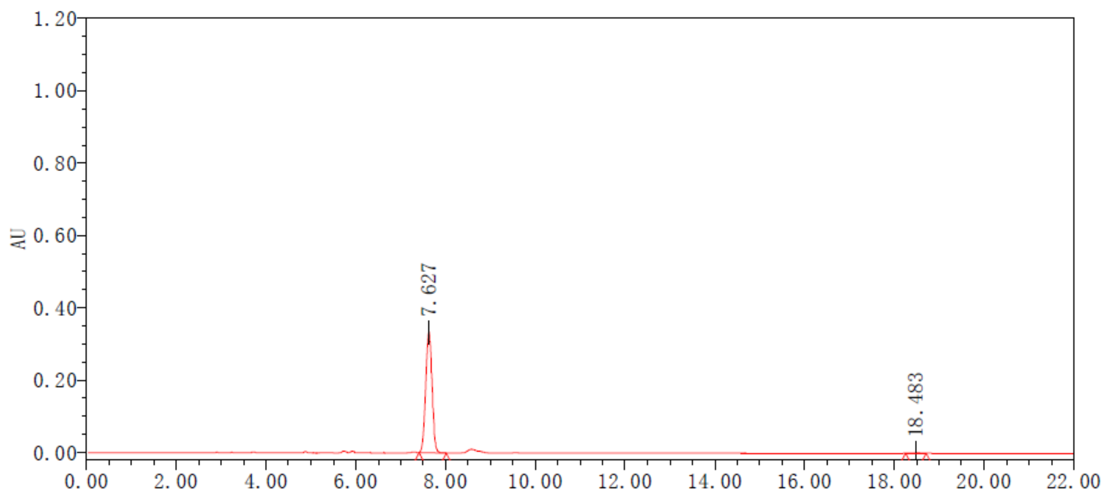

The larger version of HPLC chromatogram of chiral S11

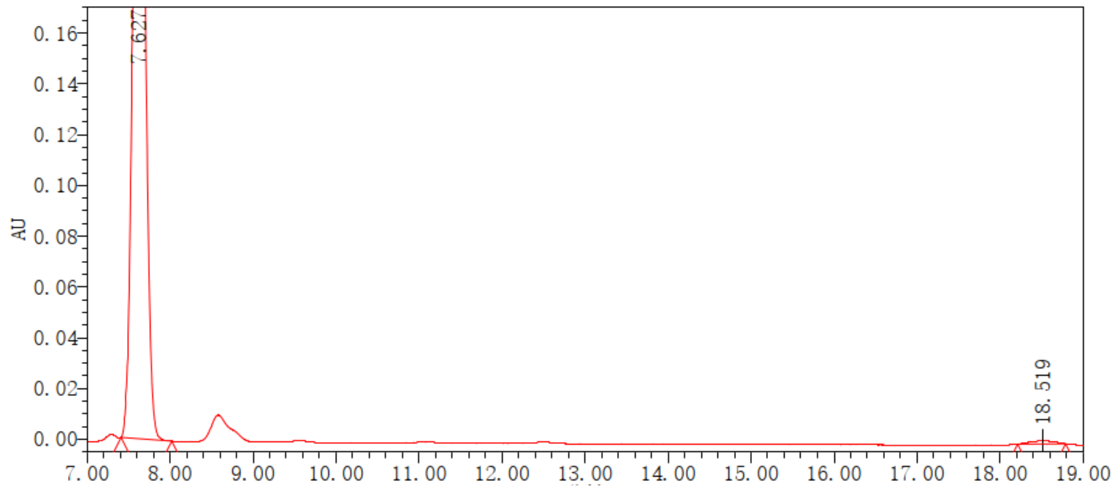

Entry	Retention Time/min	Area	Height	Area(%)
1	7.627	3295096	332931	99.54
2	18.519	15133	888	0.46

Supplementary Figure 233. Chiral HPLC analysis of chiral **S11**

Tert-butyl (2S,3R)-2-((4-bromobenzyl)oxy)-2-(3-bromophenyl)-3-(2-oxo-2-phenylethyl)-5-phenylpent-4-ynoate (S12)

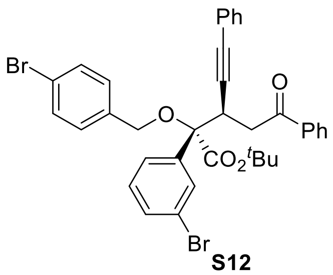

S12

Colorless oil; 64.8 mg, 63% yield, >20:1 d.r., 98% *ee*; ^{1}H NMR (400 MHz, $CDCl_3$) δ 7.94 – 7.85 (m, 3H), 7.63 (d, *J* = 7.9 Hz, 1H), 7.56 (d, *J* = 7.3 Hz, 1H), 7.53 – 7.43 (m, 5H), 7.39 (d, *J* = 8.1 Hz, 2H), 7.26 (d, *J* = 9.1 Hz, 6H), 5.05 (d, *J* = 11.6 Hz, 1H), 4.90 (d, *J* = 11.6 Hz, 1H), 4.38 (dd, *J* = 9.9, 2.9 Hz, 1H), 3.34 (dd, *J* = 16.6, 10.0 Hz, 1H), 3.12 (dd, *J* = 16.6, 2.9 Hz, 1H), 1.55 (s, 9H). ^{13}C NMR (126 MHz, $CDCl_3$) δ 197.1, 168.8, 140.7, 137.6, 136.8, 133.2, 131.5, 131.4, 131.4, 130.2, 129.8, 129.3, 128.6, 128.2, 128.2, 128.1, 125.3, 123.1, 122.6, 121.5, 89.0, 85.4, 84.5, 83.4, 68.1, 39.1, 36.8, 28.1. HRMS (ESI) $[M+Na]^+$ calcd for $C_{36}H_{32}O_4Br_2Na^+$, 709.0560, found 709.0556. (Chiral IB-3, λ= 254 nm, *n*-hexane/2-propanol= 19/1, Flow rate = 1.0 mL/min), t_R = 8.016 min(major), 13.851 min.

HPLC chromatogram of racemic S12

Condition: n-hexane/2-propanol =19:1

Flow rate =1.0 mL/min

λ= 254 nm

Chiral IB-3

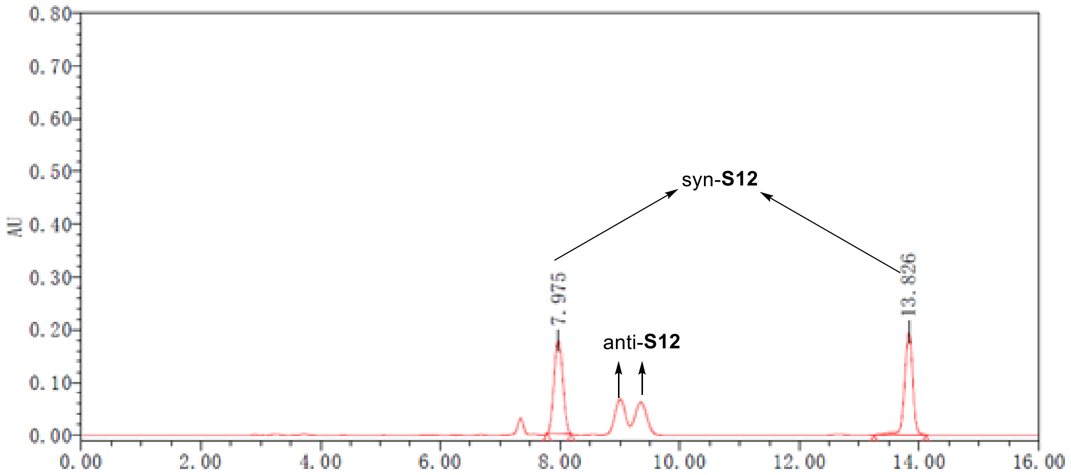

Entry	Retention Time/min	Area	Height	Area(%)
1	7.975	1794228	176673	50.07
2	13.826	1789114	194895	49.93

Supplementary Figure 234. Chiral HPLC analysis of racemic **S12**

HPLC chromatogram of chiral S12

Condition: n-hexane/2-propanol =19:1

Flow rate =1.0 mL/min

λ= 254 nm

Chiral IB-3

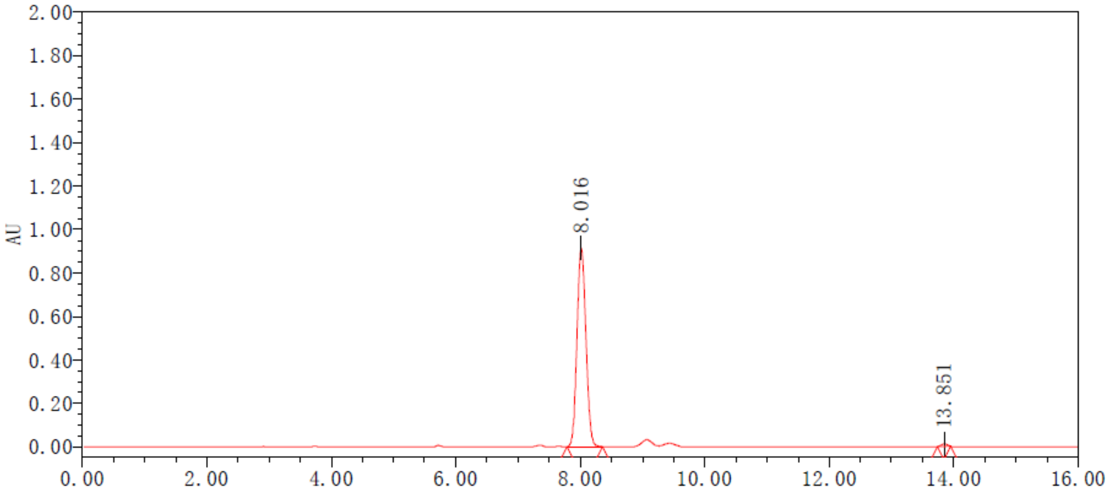

The larger version of HPLC chromatogram of chiral S12

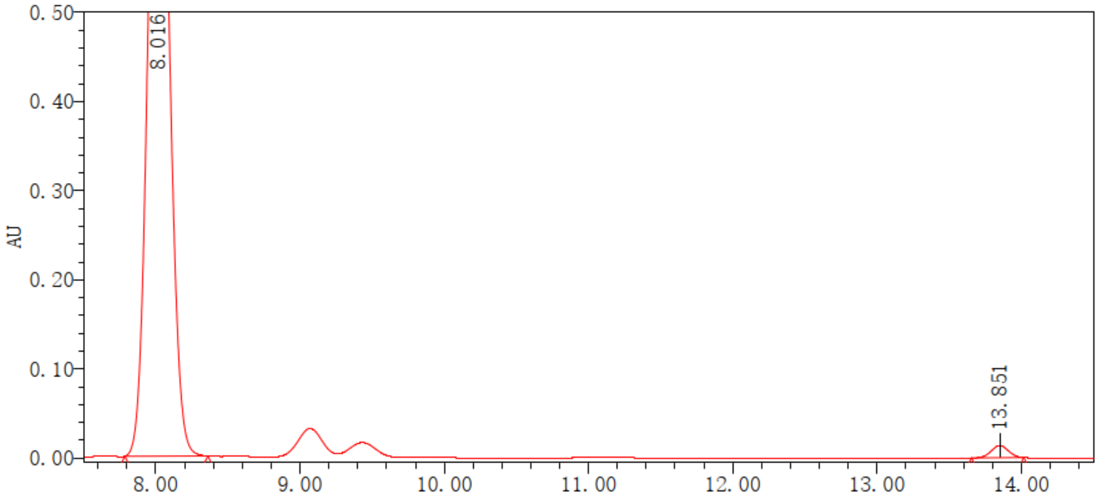

Entry	Retention Time/min	Area	Height	Area(%)
1	8.016	8985138	912719	99.13
2	13.851	79285	11462	0.87

Supplementary Figure 235. Chiral HPLC analysis of chiral **S12**

Tert-butyl (2S,3R)-2-((4-bromobenzyl)oxy)-2-(3-methoxyphenyl)-3-(2-oxo-2-phenylethyl)-5-phenylpent-4-ynoate (S13)

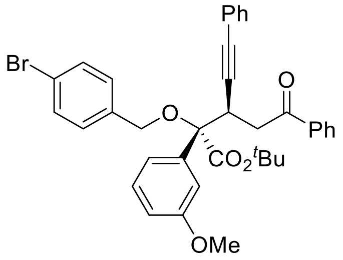

S13

Colorless oil; 53.6 mg, 56% yield, >20:1 d.r., 98% *ee*; ^{1}H NMR (400 MHz, $CDCl_3$) δ 7.84 (d, *J* = 7.8 Hz, 2H), 7.53 (t, *J* = 7.3 Hz, 1H), 7.47 (d, *J* = 8.2 Hz, 2H), 7.40 (dd, *J* = 14.9, 7.8 Hz, 5H), 7.28 – 7.21 (m, 7H), 6.85 (d, *J* = 7.4 Hz, 1H), 5.06 (d, *J* = 11.8 Hz, 1H), 4.91 (d, *J* = 11.8 Hz, 1H), 4.38 (dd, *J* = 10.1, 2.8 Hz, 1H), 3.76 (s, 3H), 3.31 (dd, *J* = 16.5, 10.2 Hz, 1H), 3.10 (dd, *J* = 16.6, 2.7 Hz, 1H), 1.52 (s, 9H). ^{13}C NMR (126 MHz, $CDCl_3$) δ 197.4, 169.3, 159.5, 139.9, 138.1, 136.9, 133.1, 131.4, 129.3, 129.2, 128.6, 128.2, 127.9, 123.4, 121.2, 119.0, 113.8, 112.6, 89.7, 85.8, 84.1, 83.0, 68.0, 55.2, 39.2, 36.7, 28.1. Peak overlapping was observed. HRMS (ESI) $[M+Na]^+$ calcd for $C_{37}H_{35}O_5BrNa^+$, 661.1560, found 661.1562. (Chiral IA, λ= 254 nm, *n*-hexane/2-propanol= 49/1, Flow rate = 1.0 mL/min), t_R = 13.274 min(major), 20.423 min.

HPLC chromatogram of racemic S13

Condition: n-hexane/2-propanol =49:1

Flow rate =1.0 mL/min

λ= 254 nm

Chiral IA

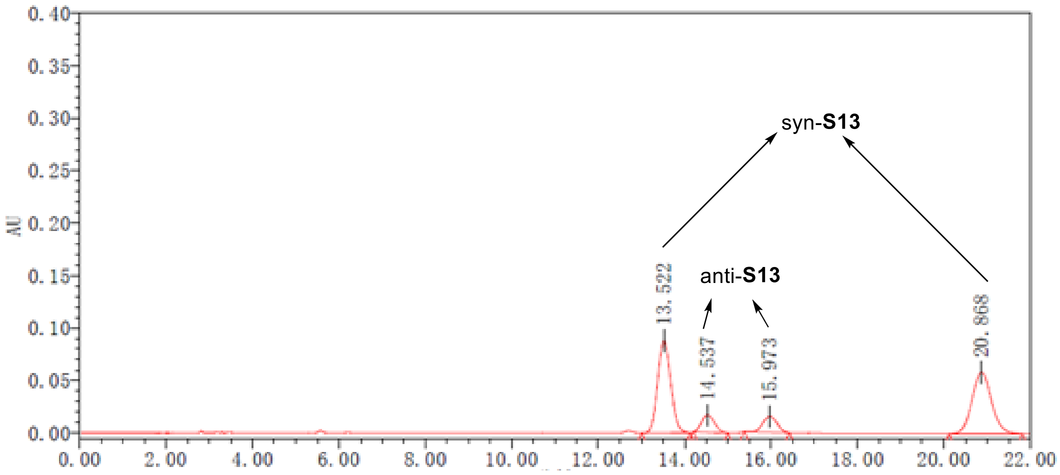

Entry	Retention Time/min	Area	Height	Area(%)
1	13.522	1839535	88180	42.01
2	14.537	347844	16736	7.94
3	15.973	352199	15306	8.04
4	20.868	1838956	58725	42.00

Supplementary Figure 236. Chiral HPLC analysis of racemic **S13**

HPLC chromatogram of chiral S13

Condition: n-hexane/2-propanol =49:1

Flow rate =1.0 mL/min

λ= 254 nm

Chiral IA

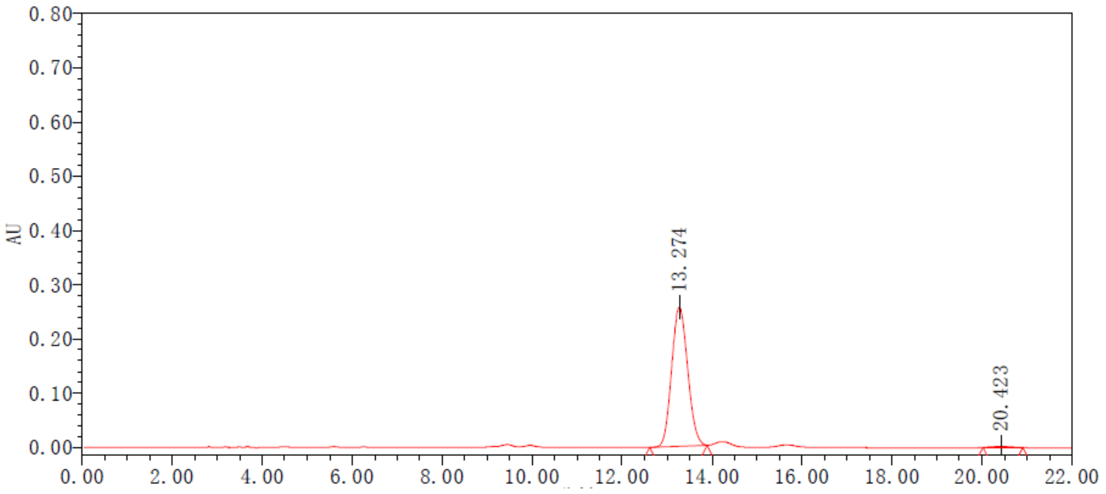

The larger version of HPLC chromatogram of chiral S13

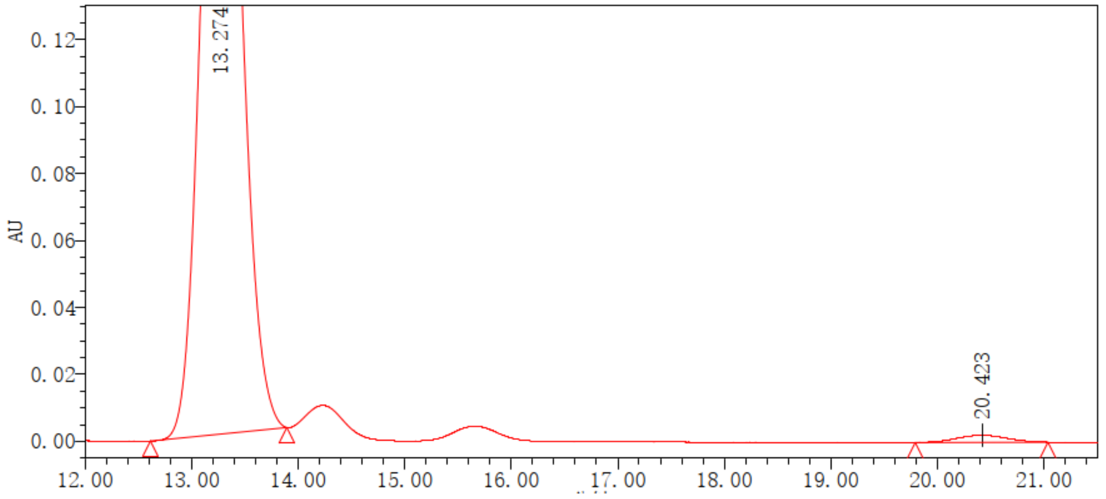

Entry	Retention Time/min	Area	Height	Area(%)
1	13.274	6383182	255928	99.19
2	20.423	51804	1808	0.81

Supplementary Figure 237. Chiral HPLC analysis of chiral **S13**

Tert-butyl (2R,3R)-2-((4-bromobenzyl)oxy)-2-(4-chlorobenzyl)-3-(2-oxo-2-phenylethyl)-5-phenylpent-4-ynoate (S14)

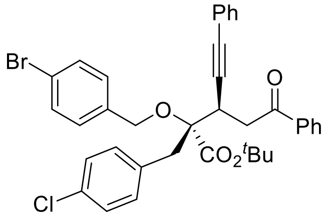

S14

Colorless oil; 39.4 mg, 40% yield, >20:1 d.r., 92% *ee*; ^{1}H NMR (400 MHz, $CDCl_3$) δ 7.93 – 7.89 (m, 2H), 7.58 – 7.53 (m, 1H), 7.46 – 7.41 (m, 4H), 7.30 (d, *J* = 3.5 Hz, 2H), 7.27 – 7.24 (m, 7H), 7.20 (d, *J* = 8.4 Hz, 2H), 4.93 (d, *J* = 11.4 Hz, 1H), 4.72 (d, *J* = 11.4 Hz, 1H), 3.91 (dd, *J* = 8.6, 4.0 Hz, 1H), 3.65 (dd, *J* = 17.1, 4.0 Hz, 1H), 3.39 – 3.35 (m, 1H), 3.30 (dd, *J* = 31.9, 9.3 Hz, 2H), 1.48 (s, 9H). ^{13}C NMR (101 MHz, $CDCl_3$) δ 197.5, 169.6, 137.2, 136.9, 134.2, 133.1, 132.8, 132.0, 131.5, 131.4, 129.0, 128.6, 128.2, 128.2, 128.1, 123.1, 123.0, 121.3, 88.9, 84.4, 82.8, 66.8, 39.1, 34.1, 28.2. HRMS (ESI) $[M+Na]^+$ calcd for $C_{37}H_{34}O_4ClBrNa^+$, 679.1220, found 679.1220. (Chiral IA-3, λ= 254 nm, *n*-hexane/2-propanol= 49/1, Flow rate = 1.0 mL/min), t_R = 12.288 min(major), 15.866 min.

HPLC chromatogram of racemic S14

Condition: n-hexane/2-propanol =49:1

Flow rate =1.0 mL/min

λ= 254 nm

Chiral IA-3

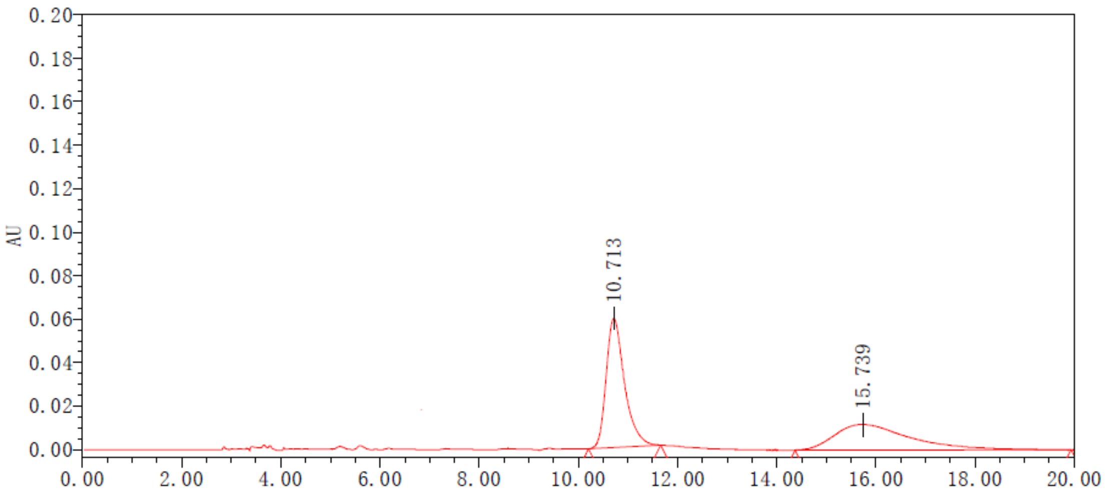

Entry	Retention Time/min	Area	Height	Area(%)
1	10.713	1376091	59478	49.57
2	15.739	1393337	11704	50.43

Supplementary Figure 238. Chiral HPLC analysis of racemic **S14**

HPLC chromatogram of chiral S14

Condition: n-hexane/2-propanol =49:1

Flow rate =1.0 mL/min

λ= 254 nm

Chiral IA-3

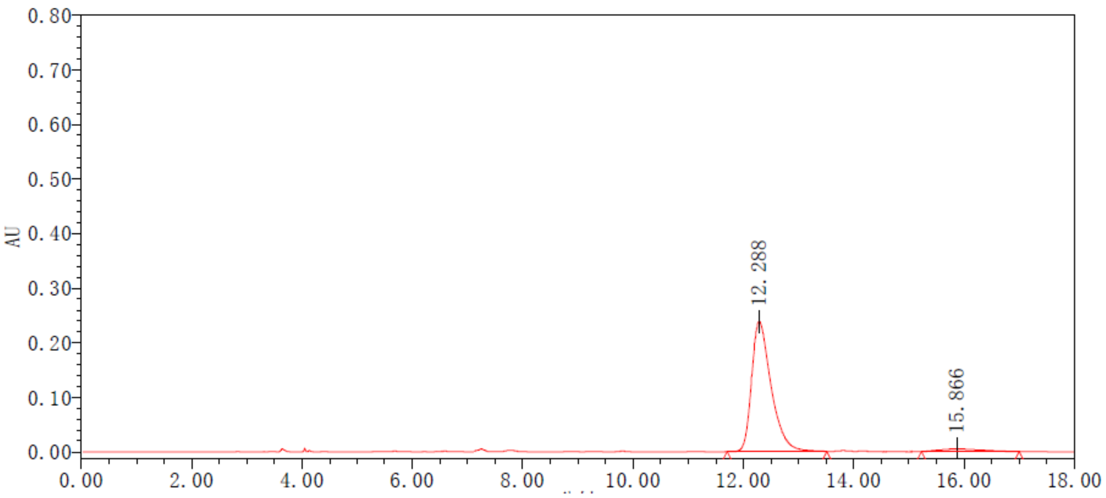

Entry	Retention Time/min	Area	Height	Area(%)
1	12.288	5870048	5870048	96.00
2	15.866	244783	244783	4.00

Supplementary Figure 239. Chiral HPLC analysis of chiral **S14**

Tert-butyl (2S,3R)-2-((4-bromobenzyl)oxy)-3-(2-oxo-2-phenylethyl)-2-phenyl-5-(p-tolyl)pent-4-ynoate (S15)

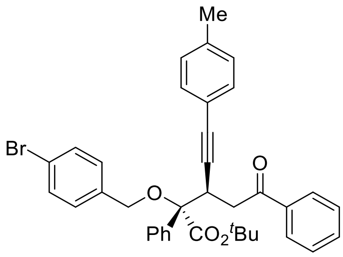

S15

Colorless oil; 51.3 mg, 55% yield, >20:1 d.r., 99% *ee*; ^{1}H NMR (400 MHz, $CDCl_3$) δ 7.86 – 7.80 (m, 2H), 7.71 – 7.67 (m, 2H), 7.52 (t, *J* = 7.4 Hz, 1H), 7.49 – 7.46 (m, 2H), 7.40 (dd, *J* = 7.3, 3.3 Hz, 3H), 7.38 – 7.33 (m, 3H), 7.32 – 7.29 (m, 1H), 7.13 (d, *J* = 8.1 Hz, 2H), 7.03 (d, *J* = 7.9 Hz, 2H), 5.06 (d, *J* = 11.8 Hz, 1H), 4.91 (d, *J* = 11.8 Hz, 1H), 4.40 (dd, *J* = 10.1, 3.0 Hz, 1H), 3.31 (dd, *J* = 16.6, 10.2 Hz, 1H), 3.07 (dd, *J* = 16.6, 3.1 Hz, 1H), 2.30 (s, 3H), 1.51 (s, 9H). ^{13}C NMR (101 MHz, $CDCl_3$) δ 197.5, 169.4, 138.4, 138.1, 137.9, 137.0, 133.1, 131.4, 131.3, 129.2, 129.0, 128.5, 128.3, 128.2, 128.2, 126.7, 121.2, 120.3, 88.9, 85.9, 84.2, 82.9, 68.0, 39.2, 36.6, 28.1, 21.4. HRMS

(ESI) $[M+Na]^+$ calcd for $C_{37}H_{35}O_4BrNa^+$, 645.1611, found 645.1608. (Chiral IA-3, λ= 254 nm, *n*-hexane/2-propanol= 19/1, Flow rate = 1.0 mL/min), t_R = 8.054 min(major), 9.316 min.

HPLC chromatogram of racemic S15

Condition: n-hexane/2-propanol =19:1

Flow rate =1.0 mL/min

λ= 254 nm

Chiral IA-3

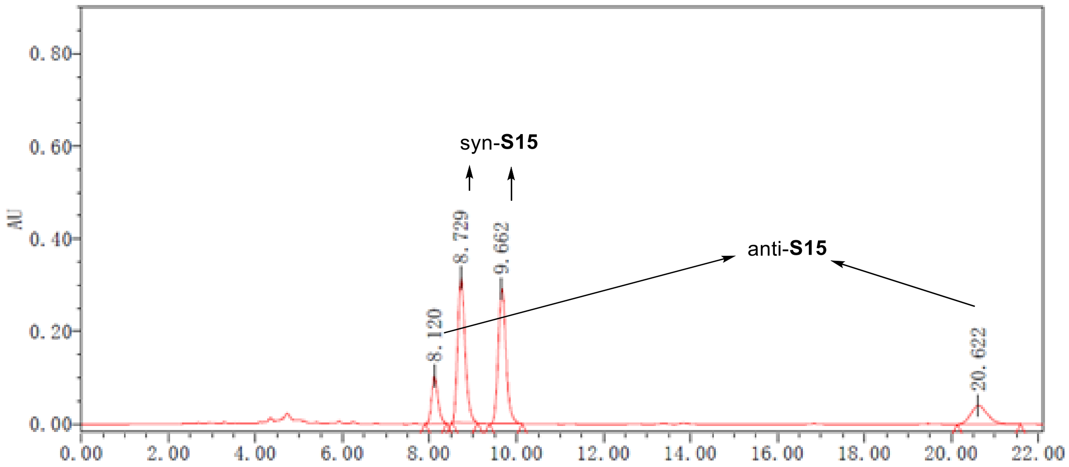

Entry	Retention Time/min	Area	Height	Area(%)
1	8.120	1046835	102902	11.07
2	8.729	3697670	312862	39.12
3	9.662	3661343	291250	38.73
4	20.622	1047061	39932	11.08

Supplementary Figure 240. Chiral HPLC analysis of racemic **S15**

HPLC chromatogram of chiral S15

Condition: n-hexane/2-propanol =19:1

Flow rate =1.0 mL/min

λ= 254 nm

Chiral IA-3

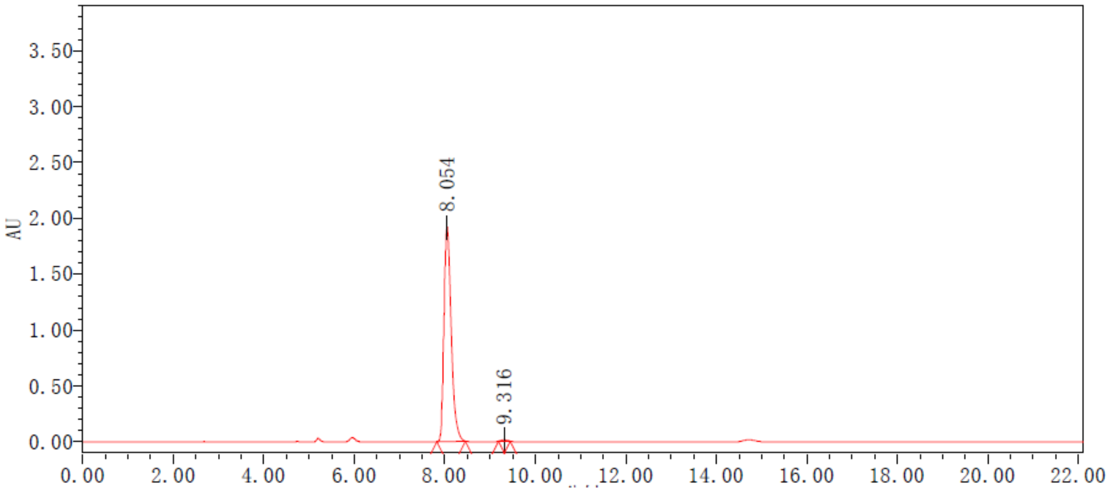

Entry	Retention Time/min	Area	Height	Area(%)
1	8.054	21441076	1915487	99.47
2	9.316	114358	12481	0.53

Supplementary Figure 241. Chiral HPLC analysis of chiral **S15**

Tert-butyl (2S,3R)-2-((4-bromobenzyl)oxy)-5-(4-chlorophenyl)-3-(2-oxo-2-phenylethyl)-2-phenylpent-4-ynoate (S16)

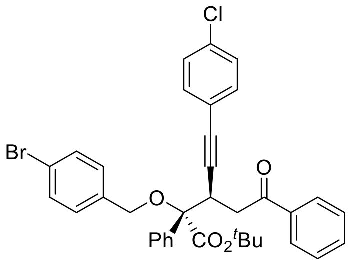

S16

Colorless oil; 57.8 mg, 60% yield, >20:1 d.r., 99% *ee*; ^{1}H NMR (500 MHz, $CDCl_3$) δ 7.85 (d, *J* = 7.3 Hz, 2H), 7.67 (d, *J* = 7.5 Hz, 2H), 7.53 (t, *J* = 7.4 Hz, 1H), 7.47 (d, *J* = 8.3 Hz, 2H), 7.41 (t, *J* = 7.7 Hz, 2H), 7.39 – 7.34 (m, 4H), 7.32 (t, *J* = 7.2 Hz, 1H), 7.20 (d, *J* = 8.4 Hz, 2H), 7.14 (d, *J* = 8.5 Hz, 2H), 5.00 (d, *J* = 11.7 Hz, 1H), 4.83 (d, *J* = 11.7 Hz, 1H), 4.39 (dd, *J* = 10.2, 3.0 Hz, 1H), 3.31 (dd, *J* = 16.7, 10.2 Hz, 1H), 3.13 (dd, *J*

= 16.6, 2.9 Hz, 1H), 1.51 (s, 9H). ^{13}C NMR (126 MHz, $CDCl_3$) δ 197.3, 169.4, 138.1, 137.9, 136.8, 133.9, 133.2, 132.6, 131.42, 129.2, 128.6, 128.5, 128.4, 128.3, 128.2, 126.8, 121.8, 121.3, 90.7, 85.9, 83.0, 67.9, 39.2, 36.8, 28.1. HRMS (ESI) $[M+Na]^+$ calcd for $C_{36}H_{32}O_4ClBrNa^+$, 665.1065, found 665.1065. (Chiral IA, λ= 254 nm, *n*-hexane/2-propanol= 19/1, Flow rate = 1.0 mL/min), t_R = 7.723 min(major), 12.895 min.

HPLC chromatogram of racemic S16

Condition: n-hexane/2-propanol =19:1

Flow rate =1.0 mL/min

λ= 254 nm

Chiral IA

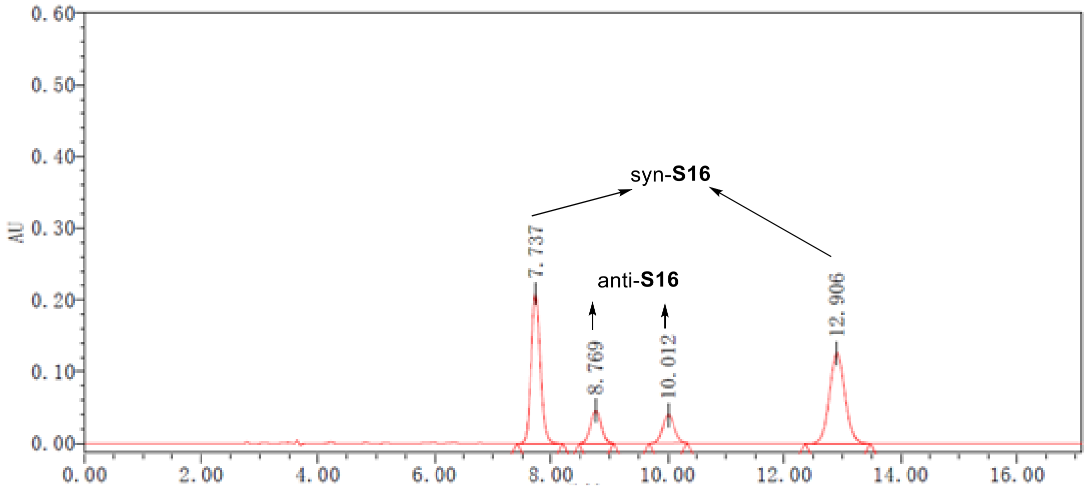

Entry	Retention Time/min	Area	Height	Area(%)
1	7.737	2307827	208651	40.28
2	8.769	563285	45762	9.83
3	10.012	556478	39555	9.71
4	12.906	2301769	125734	40.17

Supplementary Figure 242. Chiral HPLC analysis of racemic **S16**

HPLC chromatogram of chiral S16

Condition: n-hexane/2-propanol =19:1

Flow rate =1.0 mL/min

λ= 254 nm

Chiral IA

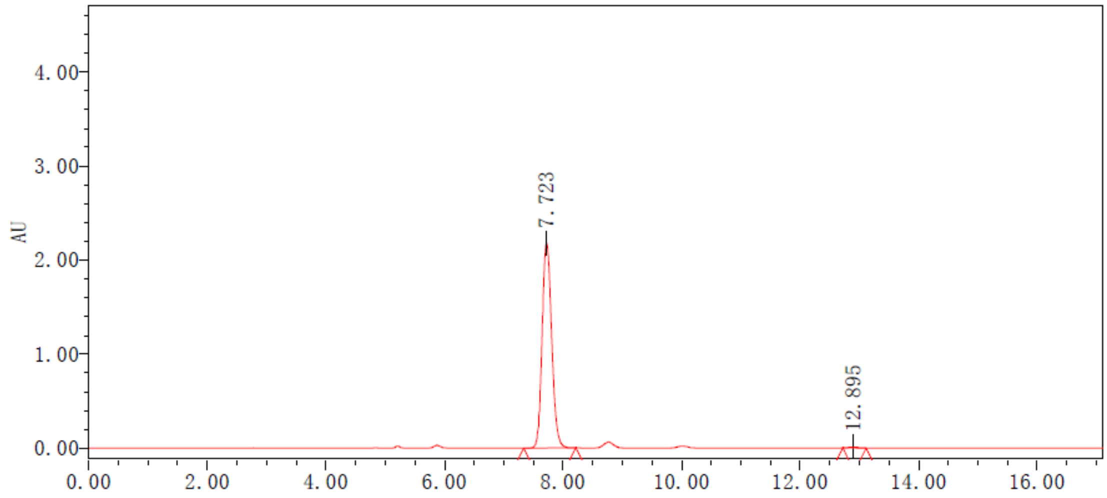

The larger version of HPLC chromatogram of chiral S16

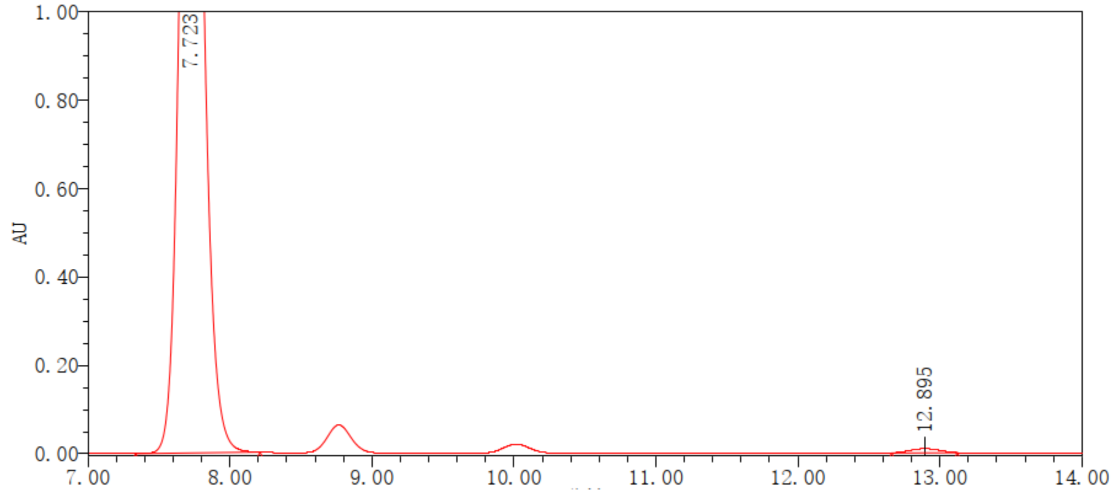

Entry	Retention Time/min	Area	Height	Area(%)
1	7.723	24139359	2179871	99.53
2	12.895	113041	8470	0.47

Supplementary Figure 243. Chiral HPLC analysis of chiral **S16**

Methyl 4-((3R,4S)-4-((4-bromobenzyl)oxy)-5-(tert-butoxy)-5-oxo-3-(2-oxo-2-phenylethyl)-4-phenylpent-1-yn-1-yl)benzoate (S17)

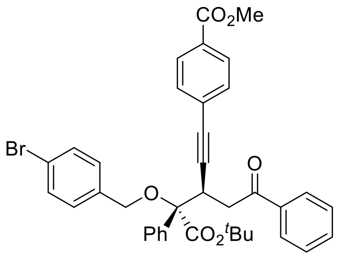

S17

Colorless oil; 56.6 mg, 57% yield, >20:1 d.r., 99% *ee*; ^{1}H NMR (500 MHz, $CDCl_3$) δ 7.90 (d, *J* = 8.4 Hz, 2H), 7.87 – 7.82 (m, 2H), 7.67 (d, *J* = 7.5 Hz, 2H), 7.54 (t, *J* = 7.4 Hz, 1H), 7.47 (d, *J* = 8.3 Hz, 2H), 7.43 (d, *J* = 7.9 Hz, 2H), 7.40 (d, *J* = 4.0 Hz, 1H), 7.36 (dd, *J* = 17.6, 8.8 Hz, 4H), 7.28 – 7.25 (m, 2H), 5.00 (d, *J* = 11.7 Hz, 1H), 4.83 (d, *J* = 11.7 Hz, 1H), 4.42 (dd, *J* = 10.2, 3.0 Hz, 1H), 3.89 (s, 3H), 3.33 (dd, *J* = 16.7, 10.2 Hz, 1H), 3.16 (dd, *J* = 16.7, 2.9 Hz, 1H), 1.51 (s, 9H). ^{13}C NMR (126 MHz, $CDCl_3$) δ 197.2, 169.3, 166.6, 138.0, 137.9, 136.8, 133.2, 131.4, 131.3, 129.4, 129.2, 129.1, 128.6, 128.4, 128.4, 128.2, 128.1, 126.8, 121.3, 93.1, 85.9, 83.5, 83.1, 68.0, 52.2, 39.1, 36.9, 28.1. HRMS (ESI) $[M+Na]^+$ calcd for $C_{38}H_{35}O_6BrNa^+$, 689.1509, found 689.1510. (Chiral IA-3, λ= 254 nm, *n*-hexane/2-propanol= 19/1, Flow rate = 1.0 mL/min), t_R = 10.543 min(major), 18.419 min.

HPLC chromatogram of racemic S17

Condition: n-hexane/2-propanol =19:1

Flow rate =1.0 mL/min

λ= 254 nm

Chiral IA-3

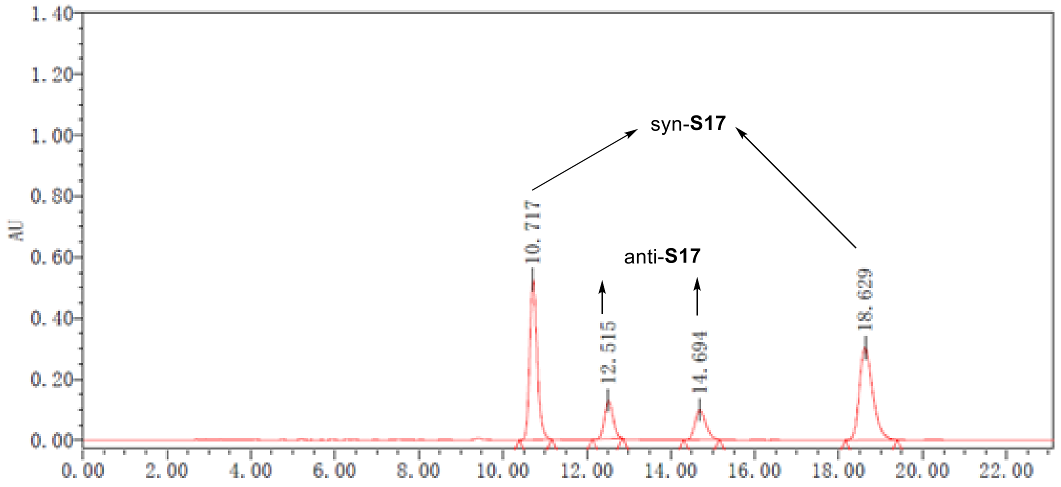

Entry	Retention Time/min	Area	Height	Area(%)
1	10.717	6550783	524511	39.14
2	12.515	1784250	126250	10.66
3	14.694	1757376	98365	10.50
4	18.629	6642978	303082	39.69

Supplementary Figure 244. Chiral HPLC analysis of racemic **S17**

HPLC chromatogram of chiral S17

Condition: n-hexane/2-propanol =19:1

Flow rate =1.0 mL/min

λ= 254 nm

Chiral IA-3

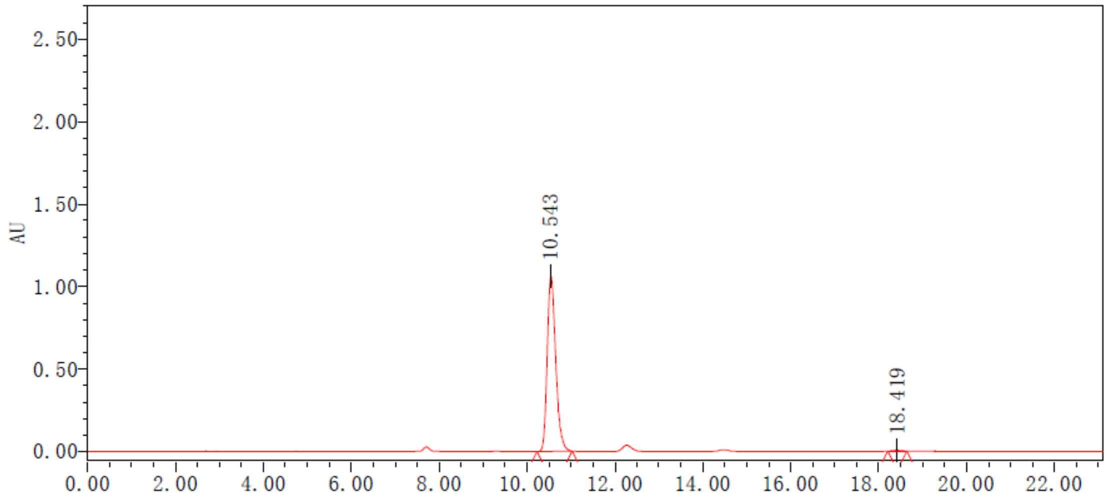

The larger version of HPLC chromatogram of chiral S17

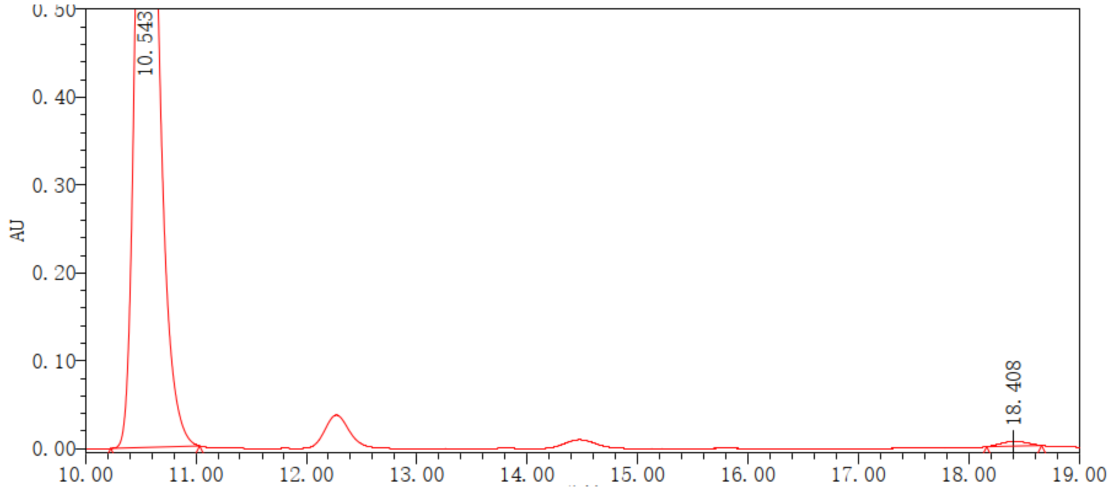

Entry	Retention Time/min	Area	Height	Area(%)
1	10.543	14064028	1061555	99.50
2	18.419	71343	4690	0.50

Supplementary Figure 245. Chiral HPLC analysis of chiral **S17**

Tert-butyl (2S,3R)-2-((4-bromobenzyl)oxy)-3-(2-oxo-2-phenylethyl)-2-phenyl-5-(thiophen-2-yl)pent-4-ynoate (S18)

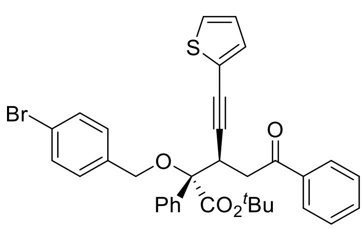

S18

Colorless oil; 38.7 mg, 42% yield, >20:1 d.r., 98% *ee*; ^{1}H NMR (500 MHz, $CDCl_3$) δ 7.83 (d, *J* = 7.3 Hz, 2H), 7.67 (d, *J* = 7.6 Hz, 2H), 7.53 (t, *J* = 7.4 Hz, 1H), 7.49 (d, *J* = 8.3 Hz, 2H), 7.39 (dt, *J* = 14.9, 7.8 Hz, 6H), 7.31 (t, *J* = 7.3 Hz, 1H), 7.16 (d, *J* = 5.1 Hz, 1H), 7.01 (d, *J* = 3.2 Hz, 1H), 6.90 (dd, *J* = 5.0, 3.7 Hz, 1H), 5.01 (d, *J* = 11.8 Hz, 1H), 4.88 (d, *J* = 11.8 Hz, 1H), 4.43 (dd, *J* = 10.1, 2.9 Hz, 1H), 3.31 (dd, *J* = 16.9, 10.1 Hz, 1H), 3.08 (dd, *J* = 16.8, 2.8 Hz, 1H), 1.52 (s, 9H). ^{13}C NMR (126 MHz, $CDCl_3$) δ 197.2, 169.3, 138.2, 138.0, 136.8, 133.2, 131.4, 131.3, 129.2, 128.6, 128.3, 128.3, 128.2, 126.8, 126.6, 126.5, 123.4, 121.2, 93.8, 85.9, 83.1, 68.0, 39.0, 36.7, 28.1. HRMS (ESI) $[M+Na]^+$ calcd for $C_{34}H_{31}O_4SBrNa^+$, 637.1019, found 637.1016. (Chiral IE-3, λ= 254 nm, *n*-hexane/2-propanol= 19/1, Flow rate = 1.0 mL/min), t_R = 11.160 min(major), 25.553 min.

HPLC chromatogram of racemic S18

Condition: n-hexane/2-propanol =19:1

Flow rate =1.0 mL/min

λ= 254 nm

Chiral IE-3

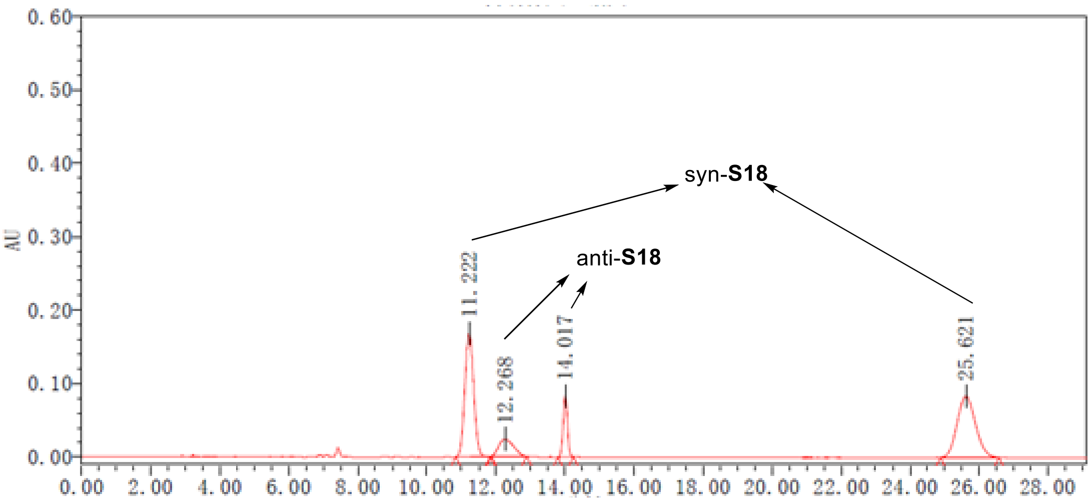

Entry	Retention Time/min	Area	Height	Area(%)
1	11.222	3070845	168973	40.51
2	12.268	704675	23572	9.30
3	14.017	710375	83303	9.37
4	25.621	3093886	83510	40.82

Supplementary Figure 246. Chiral HPLC analysis of racemic **S18**

HPLC chromatogram of chiral S18

Condition: n-hexane/2-propanol =19:1

Flow rate =1.0 mL/min

λ= 254 nm

Chiral IE-3

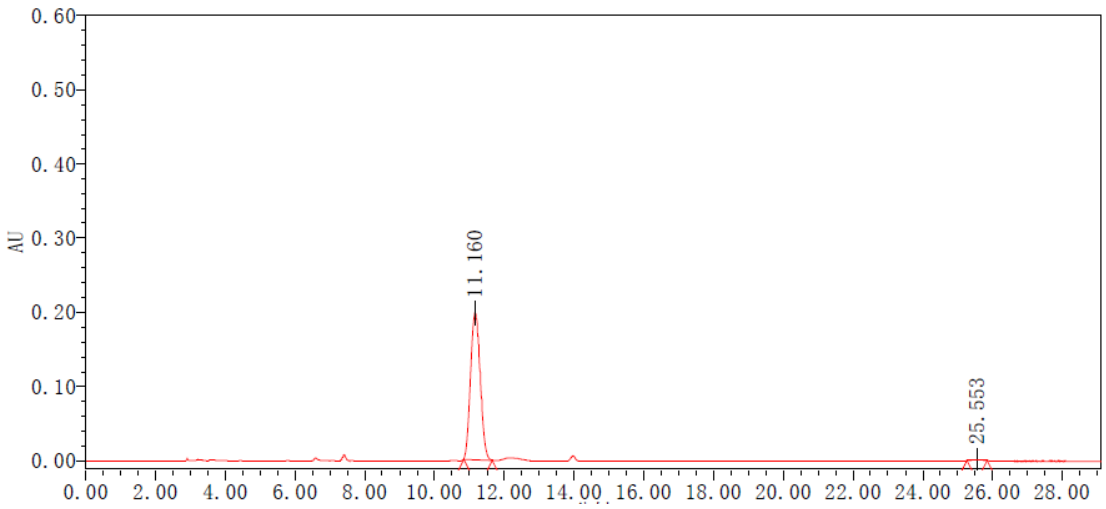

Entry	Retention Time/min	Area	Height	Area(%)
1	11.160	3883914	197424	99.50
2	25.553	19604	902	0.50

Supplementary Figure 247. Chiral HPLC analysis of chiral **S18**

Tert-butyl (2S,3R)-2-((4-bromobenzyl)oxy)-5-(5-methylthiophen-2-yl)-3-(2-oxo-2-phenylethyl)-2-phenylpent-4-ynoate (S19)

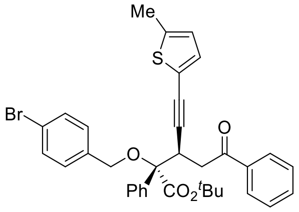

S19

Colorless oil; 43.4 mg, 46% yield, >20:1 d.r., 97% *ee*; ^{1}H NMR (500 MHz, $CDCl_3$) δ 7.85 – 7.79 (m, 2H), 7.69 – 7.64 (m, 2H), 7.53 (t, *J* = 7.4 Hz, 1H), 7.49 (d, *J* = 8.4 Hz, 2H), 7.43 – 7.38 (m, 4H), 7.36 (t, *J* = 7.5 Hz, 2H), 7.31 (d, *J* = 7.2 Hz, 1H), 6.81 (d, *J* = 3.5 Hz, 1H), 6.54 (dd, *J* = 3.5, 1.0 Hz, 1H), 5.02 (d, *J* = 11.8 Hz, 1H), 4.90 (d, *J* = 11.8 Hz, 1H), 4.41 (dd, *J* = 10.1, 2.9 Hz, 1H), 3.30 (dd, *J* = 16.8, 10.1 Hz, 1H), 3.05 (dd, *J* = 16.8, 2.9 Hz, 1H), 2.41 (s, 3H), 1.52 (s, 9H). ^{13}C NMR (126 MHz, $CDCl_3$) δ 197.3, 169.3, 141.2, 138.3, 138.1, 136.8, 133.1, 131.5, 131.4, 129.2, 128.5, 128.3, 128.3, 128.2, 126.6, 125.3, 121.2, 120.9, 92.8, 85.9, 83.0, 77.8, 77.3, 68.0, 39.0, 36.7, 28.1,

15.4. HRMS (ESI) $[M+Na]^+$ calcd for $C_{35}H_{33}O_4SBrNa^+$, 651.1175, found 651.1175. (Chiral IE-3, λ= 254 nm, *n*-hexane/2-propanol= 19/1, Flow rate = 1.0 mL/min), t_R = 11.793 min(major), 31.109 min.

HPLC chromatogram of racemic S19

Condition: n-hexane/2-propanol =19:1

Flow rate =1.0 mL/min

λ= 254 nm

Chiral IE-3

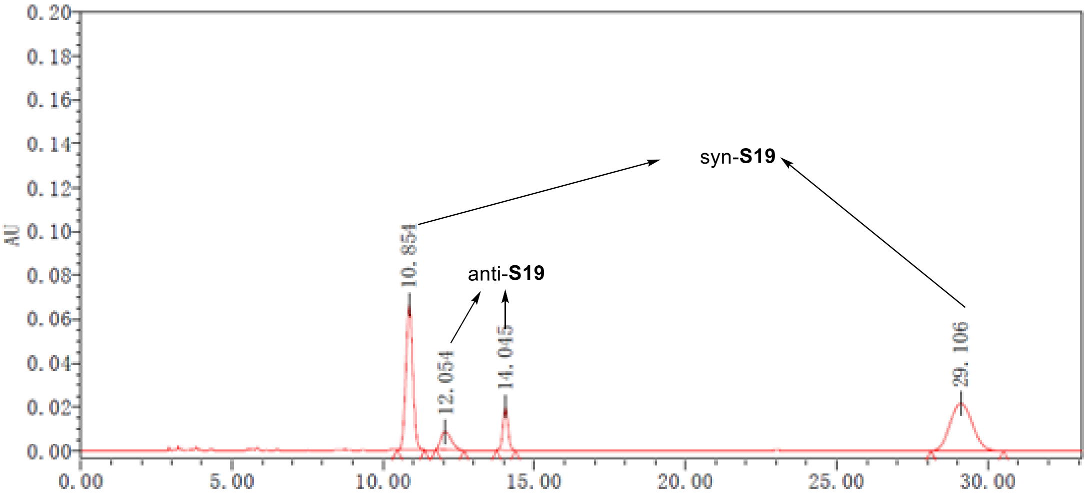

Entry	Retention Time/min	Area	Height	Area(%)
1	10.854	1114806	66456	41.90
2	12.054	204793	8366	7.70
3	14.045	226990	19828	8.53
4	29.106	1113738	21527	41.86

Supplementary Figure 248. Chiral HPLC analysis of racemic **S19**

HPLC chromatogram of chiral S19

Condition: n-hexane/2-propanol =19:1

Flow rate =1.0 mL/min

λ= 254 nm

Chiral IE-3

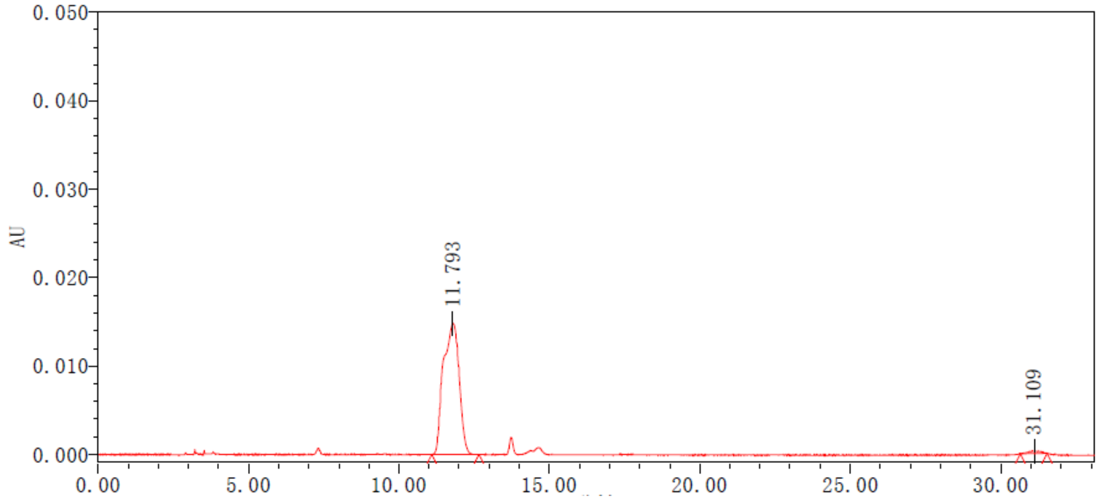

The larger version of HPLC chromatogram of chiral S19

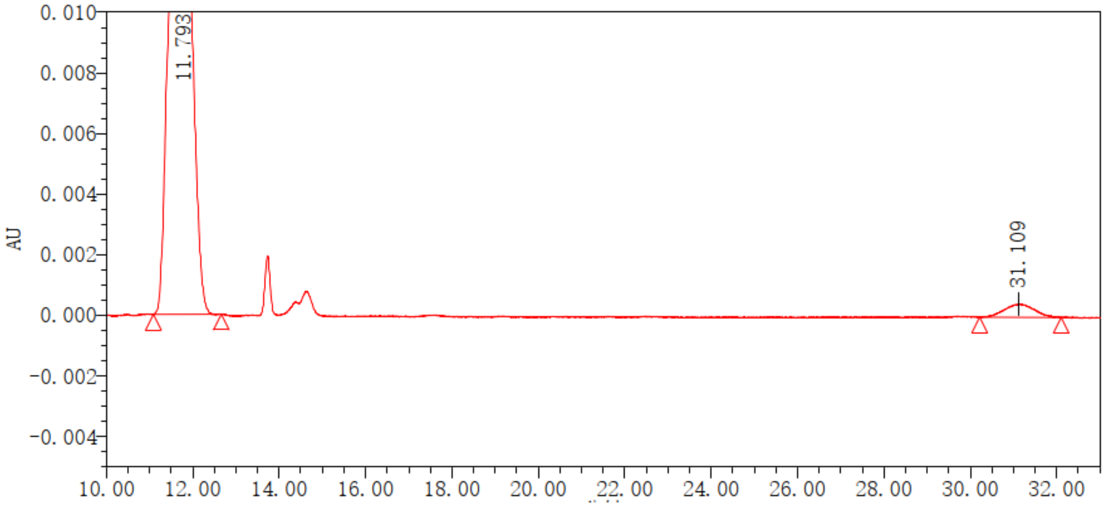

Entry	Retention Time/min	Area	Height	Area(%)
1	11.793	540927	14788	98.52
2	31.109	8101	259	1.48

Supplementary Figure 249. Chiral HPLC analysis of chiral **S19**

Tert-butyl (2S,3R)-2-((4-bromobenzyl)oxy)-3-(2-oxo-2-phenylethyl)-2-phenyl-5-(thiophen-3-yl)pent-4-ynoate (S20)

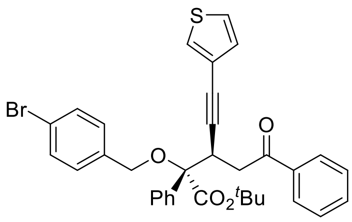

S20

Colorless oil; 43.3 mg, 47% yield, >20:1 d.r., 99% *ee*; ^{1}H NMR (400 MHz, $CDCl_3$) δ 7.87 – 7.80 (m, 2H), 7.70 – 7.64 (m, 2H), 7.55 – 7.50 (m, 1H), 7.47 (d, *J* = 1.8 Hz, 2H), 7.41 (d, *J* = 7.9 Hz, 2H), 7.37 (d, *J* = 8.6 Hz, 4H), 7.34 – 7.30 (m, 1H), 7.21 (dd, *J* = 2.9, 1.1 Hz, 1H), 7.17 (dd, *J* = 5.0, 3.0 Hz, 1H), 6.91 (dd, *J* = 5.0, 1.1 Hz, 1H), 5.03 (d, *J* = 11.8 Hz, 1H), 4.88 (d, *J* = 11.8 Hz, 1H), 4.39 (dd, *J* = 10.1, 3.0 Hz, 1H), 3.31 (dd, *J* = 16.7, 10.2 Hz, 1H), 3.09 (dd, *J* = 16.7, 3.1 Hz, 1H), 1.51 (s, 9H). ^{13}C NMR (101 MHz, $CDCl_3$) δ 197.4, 169.4, 138.3, 138.1, 136.9, 133.1, 131.4, 129.7, 129.2, 128.6, 128.3, 128.3, 128.2, 128.0, 126.7, 125.1, 122.4, 121.3, 89.1, 85.9, 83.0, 79.30, 68.0, 39.2, 36.6, 28.1. HRMS (ESI) $[M+Na]^+$ calcd for $C_{34}H_{31}O_4SBrNa^+$, 637.1019, found 637.1022.(Chiral IA, λ= 254 nm, *n*-hexane/2-propanol= 19/1, Flow rate = 1.0 mL/min), t_R = 8.550 min(major), 14.735 min.

HPLC chromatogram of racemic S20

Condition: n-hexane/2-propanol =19:1

Flow rate =1.0 mL/min

λ= 254 nm

Chiral IA

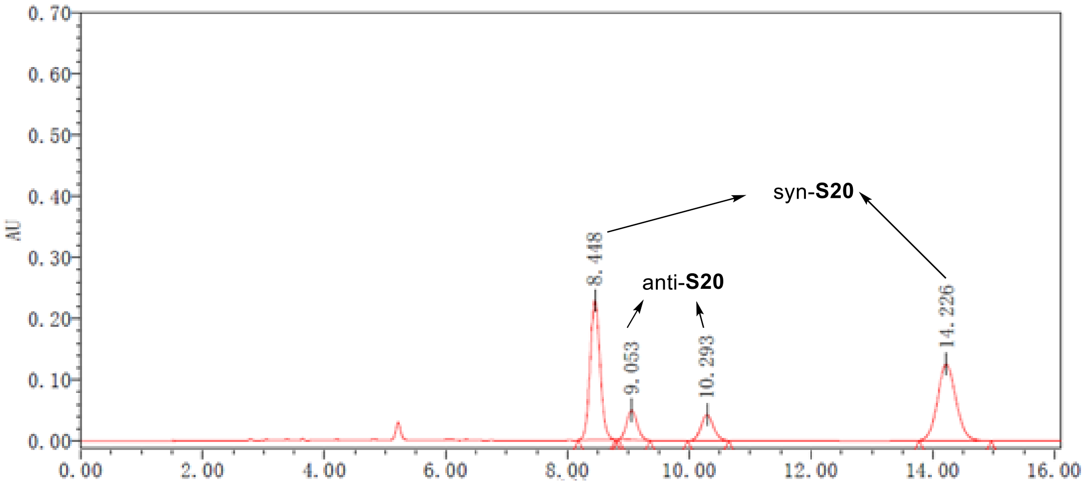

Entry	Retention Time/min	Area	Height	Area(%)
1	8.448	2620742	228355	40.67
2	9.053	586735	48986	9.11
3	10.293	606430	42645	9.41
4	14.226	2629601	124974	40.81

Supplementary Figure 250. Chiral HPLC analysis of racemic **S20**

HPLC chromatogram of chiral S20

Condition: n-hexane/2-propanol =19:1

Flow rate =1.0 mL/min

λ= 254 nm

Chiral IA

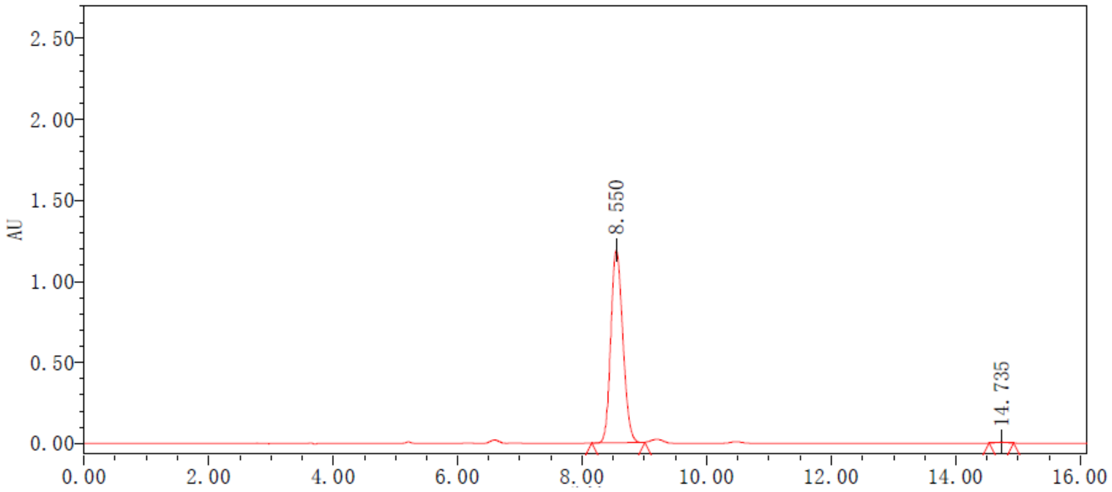

Entry	Retention Time/min	Area	Height	Area(%)
1	8.550	15829834	1188323	99.48
2	14.735	83433	5947	0.52

Supplementary Figure 251. Chiral HPLC analysis of chiral **S20**

Tert-butyl (2S,3R,E)-2-((4-bromobenzyl)oxy)-6-oxo-3-(2-oxo-2-phenylethyl)-2,6-diphenylhex-4-enoate (S21)

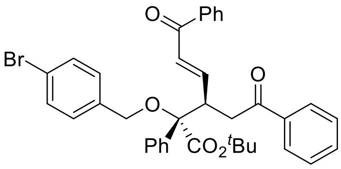

S21

Colorless oil; 52.7 mg, 55% yield, >20:1 d.r., 87% *ee*; ^{1}H NMR (400 MHz, $CDCl_3$) δ 7.92 – 7.87 (m, 2H), 7.68 – 7.62 (m, 2H), 7.53 (dd, *J* = 7.2, 1.5 Hz, 3H), 7.49 (t, *J* = 7.5 Hz, 1H), 7.44 – 7.39 (m, 6H), 7.38 – 7.34 (m, 3H), 7.19 (d, *J* = 8.3 Hz, 2H), 6.94 (dd, *J* = 15.6, 8.4 Hz, 1H), 6.66 (d, *J* = 15.9 Hz, 1H), 4.71 (d, *J* = 11.8 Hz, 1H), 4.26 (d, *J* = 11.8 Hz, 1H), 4.06 – 3.98 (m, 1H), 3.58 (dd, *J* = 16.7, 2.7 Hz, 1H), 3.13 (dd, *J* = 16.7, 9.7 Hz, 1H), 1.55 (s, 9H). ^{13}C NMR (101 MHz, $CDCl_3$) δ 197.8, 190.7, 169.7, 146.8, 137.8, 137.7, 137.3, 136.8, 133.2, 132.6, 131.3, 128.9, 128.7, 128.6, 128.6, 128.4, 128.1, 127.7, 121.1, 87.1, 83.5, 67.5, 48.3, 38.7, 28.2. HRMS (ESI) $[M+Na]^+$ calcd for

$C_{37}H_{35}O_5BrNa^+$, 661.1557, found 661.1560. (Chiral IE-3, λ= 254 nm, *n*-hexane/2-propanol= 23/2, Flow rate = 1.0 mL/min), t_R = 36.984 min(major), 41.330 min.

HPLC chromatogram of racemic S21

Condition: n-hexane/2-propanol =23:2

Flow rate =1.0 mL/min

λ= 254 nm

Chiral IE-3

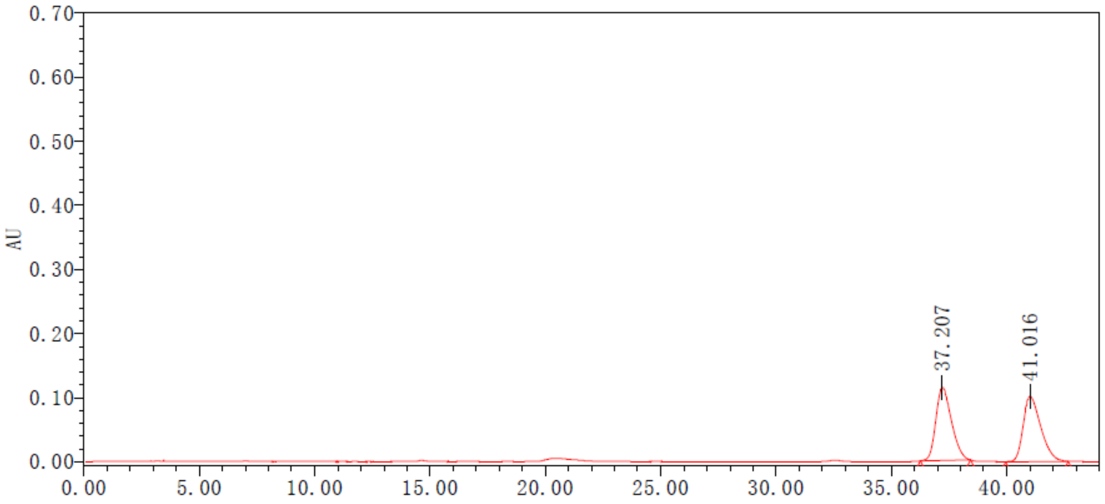

Entry	Retention Time/min	Area	Height	Area(%)
1	37.207	5419985	113974	50.04
2	41.016	5411834	101887	49.96

Supplementary Figure 252. Chiral HPLC analysis of racemic **S21**

HPLC chromatogram of chiral S21

Condition: n-hexane/2-propanol =23:2

Flow rate =1.0 mL/min

λ= 254 nm

Chiral IE-3

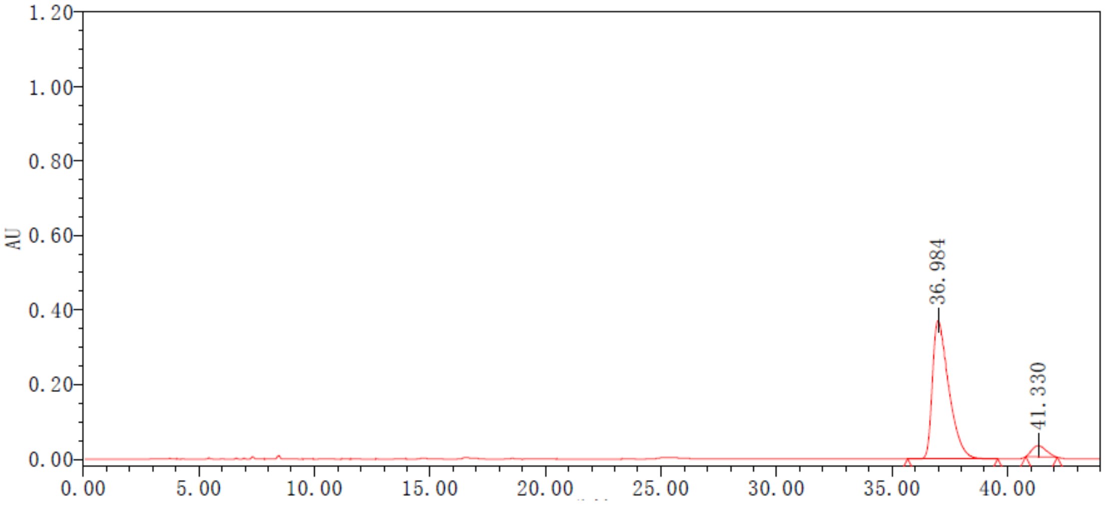

Entry	Retention Time/min	Area	Height	Area(%)
1	36.984	18333034	370985	93.51
2	41.330	1272020	29672	6.49

Supplementary Figure 253. Chiral HPLC analysis of chiral **S21**

Tert-butyl (2S,3R,E)-2-((4-bromobenzyl)oxy)-6-oxo-3-(2-oxo-2-phenylethyl)-2-phenyl-6-(p-tolyl)hex-4-enoate (S22)

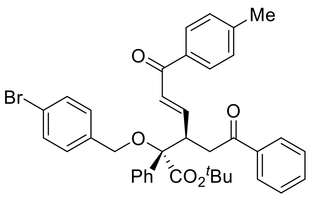

S22

Colorless oil; 46.0 mg, 47% yield, >20:1 d.r., 90% *ee*; ^{1}H NMR (400 MHz, $CDCl_3$) δ 7.90 – 7.85 (m, 2H), 7.58 – 7.51 (m, 5H), 7.40 (ddd, *J* = 6.7, 6.0, 3.4 Hz, 5H), 7.36 – 7.30 (m, 2H), 7.20 – 7.14 (m, 4H), 6.93 (dd, *J* = 15.6, 8.4 Hz, 1H), 6.66 (d, *J* = 15.4 Hz, 1H), 4.71 (d, *J* = 11.8 Hz, 1H), 4.27 (d, *J* = 11.8 Hz, 1H), 4.01 (dd, *J* = 12.8, 4.8 Hz, 1H), 3.55 (dd, *J* = 16.7, 2.7 Hz, 1H), 3.12 (dd, *J* = 16.7, 9.6 Hz, 1H), 2.37 (s, 3H), 1.55 (s, 9H). ^{13}C NMR (126 MHz, $CDCl_3$) δ 197.8, 190.2, 169.7, 146.2, 143.4, 140.9, 137.8, 137.30, 136.9, 135.1, 133.2, 131.3, 129.5, 129.1, 128.9, 128.7, 128.6, 128.5, 128.1, 127.7, 121.1, 87.1, 83.4, 67.5, 48.2, 38.7, 28.2, 21.6. HRMS (ESI) $[M+Na]^+$ calcd for

$C_{38}H_{37}O_5BrNa^+$,675.1717, found 675.1718. (Chiral IC, λ= 254 nm, *n*-hexane/2-propanol= 9/1, Flow rate = 1.0 mL/min), t_R = 13.163 min(major), 17.953 min.

HPLC chromatogram of racemic S22

Condition: n-hexane/2-propanol =9:1

Flow rate =1.0 mL/min

λ= 254 nm

Chiral IC

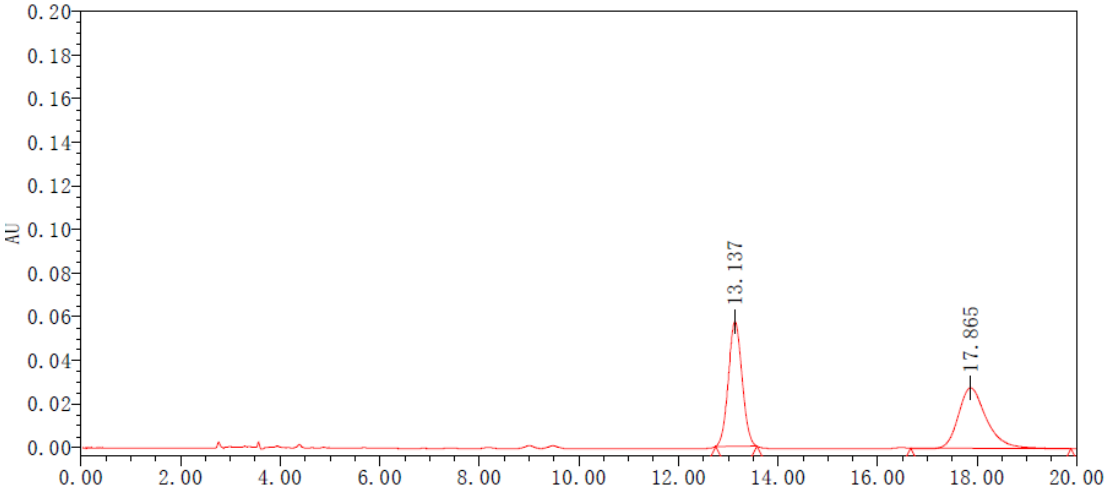

Entry	Retention Time/min	Area	Height	Area(%)
1	13.137	1097650	57261	50.31
2	17.865	1084313	27770	49.69

Supplementary Figure 254. Chiral HPLC analysis of racemic **S22**

HPLC chromatogram of chiral S22

Condition: n-hexane/2-propanol =9:1

Flow rate =1.0 mL/min

λ= 254 nm

Chiral IC

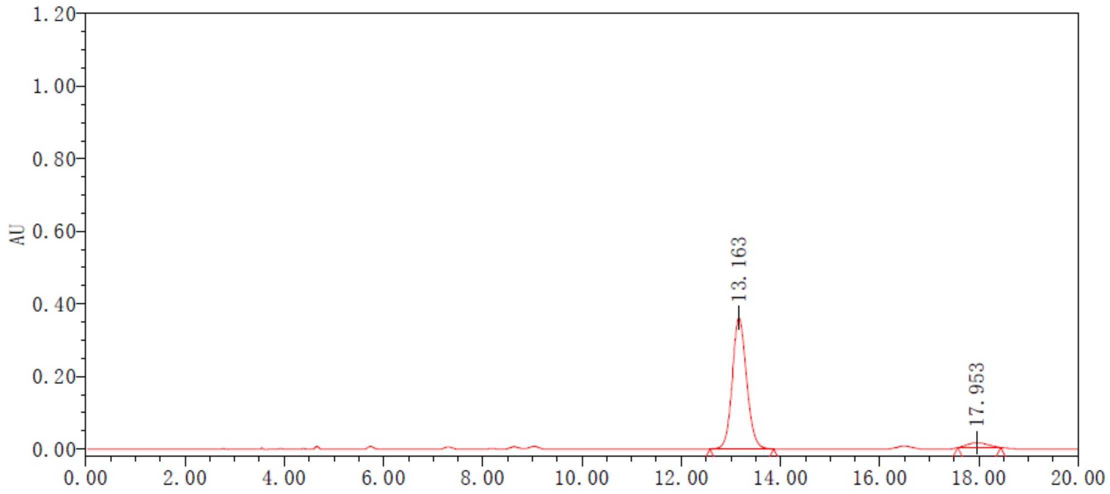

Entry	Retention Time/min	Area	Height	Area(%)
1	13.163	7224693	360847	94.93
2	17.953	385972	13380	5.07

Supplementary Figure 255. Chiral HPLC analysis of chiral **S22**

Tert-butyl (2S,3R)-3-(4-nitrophenyl)-5-oxo-2,5-diphenyl-2-(thiophen-2-ylmethoxy)pentanoate (S23)

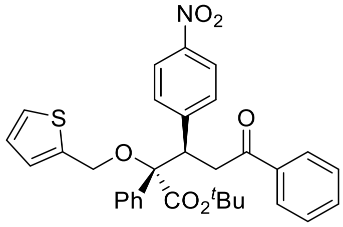

S23

Colorless oil; 56.0 mg, 67% yield, >20:1 d.r., 90% *ee*; ^{1}H NMR (400 MHz, $CDCl_3$) δ 8.03 (d, *J* = 8.8 Hz, 2H), 7.71 (d, *J* = 7.3 Hz, 2H), 7.62 (d, *J* = 6.8 Hz, 2H), 7.55 (d, *J* = 8.8 Hz, 2H), 7.49 (t, *J* = 7.4 Hz, 1H), 7.37 (dt, *J* = 11.1, 6.9 Hz, 6H), 7.05 (dd, *J* = 5.0, 3.5 Hz, 1H), 6.99 (d, *J* = 2.6 Hz, 1H), 5.16 (d, *J* = 12.2 Hz, 1H), 4.80 (d, *J* = 12.3 Hz, 1H), 4.19 (dd, *J* = 10.7, 3.0 Hz, 1H), 3.63 (dd, *J* = 17.3, 10.7 Hz, 1H), 3.43 (dd, *J* = 17.3, 3.1 Hz, 1H), 1.35 (s, 9H). ^{13}C NMR (101 MHz, $CDCl_3$) δ 197.6, 169.5, 148.1, 146.8, 142.0, 136.7, 136.4, 133.2 131.5, 128.6, 128.5, 127.9, 127.6, 126.7, 125.2, 124.7, 122.6, 87.8, 83.5, 64.1, 51.4, 39.5, 27.9. Peak overlapping was observed. HRMS (ESI)

[M+Na]$^+$ calcd for $C_{32}H_{31}NO_6SNa^+$, 580.1764, found 580.1770. (Chiral IA-3, λ= 254 nm, *n*-hexane/2-propanol= 9/1, Flow rate = 1.0 mL/min), t_R = 8.691 min, 10.772 min(major)

HPLC chromatogram of racemic S23

Condition: n-hexane/2-propanol =9:1

Flow rate =1.0 mL/min

λ= 254 nm

Chiral IA-3

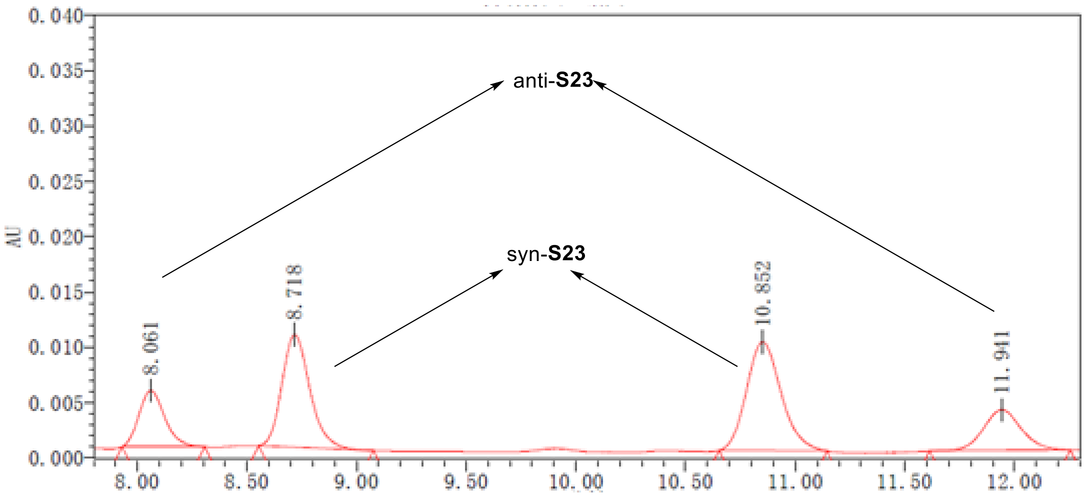

Entry	Retention Time/min	Area	Height	Area(%)
1	8.061	41857	5104	14.66
2	8.718	90696	10183	31.76
3	10.852	108117	9840	37.86
4	11.941	44866	3695	15.71

Supplementary Figure 256. Chiral HPLC analysis of racemic **S23**

HPLC chromatogram of chiral S23

Condition: n-hexane/2-propanol =9:1

Flow rate =1.0 mL/min

λ= 254 nm

Chiral IA-3

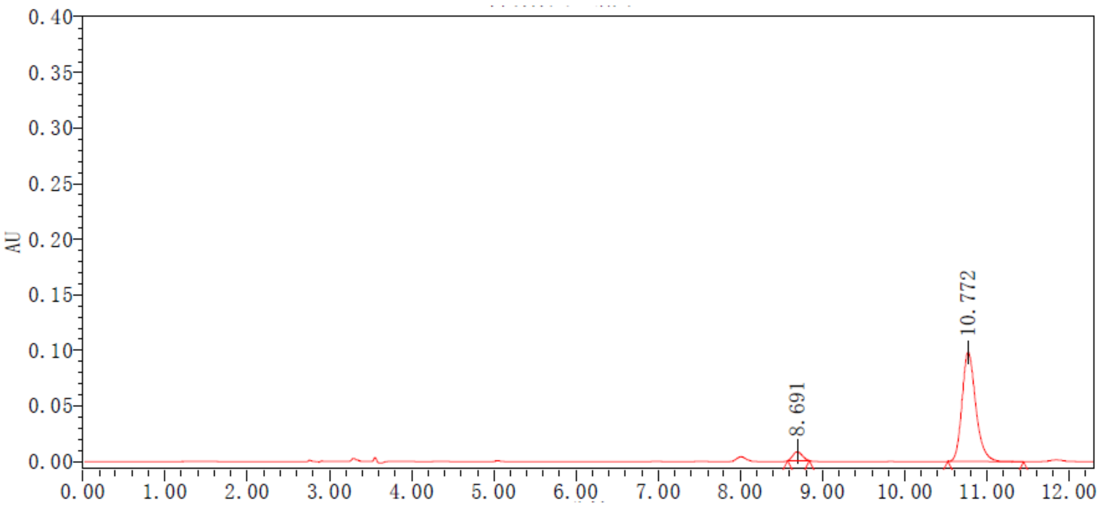

Entry	Retention Time/min	Area	Height	Area(%)
1	8.691	63031	7901	5.18
2	10.772	1153540	98382	94.82

Supplementary Figure 257. Chiral HPLC analysis of chiral **S23**

Tert-butyl (2S,3R)-2-((5-bromothiophen-2-yl)methoxy)-3-(4-nitrophenyl)-5-oxo-2,5-diphenylpentanoate (S24)

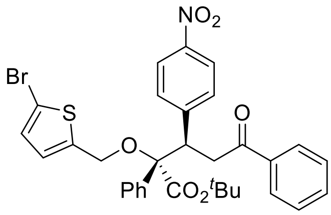

S24

Colorless oil; 52.4 mg, 55% yield, >20:1 d.r., 90% *ee*; ^{1}H NMR (500 MHz, $CDCl_3$) δ 8.04 (d, *J* = 8.7 Hz, 2H), 7.72 (d, *J* = 7.6 Hz, 2H), 7.59 (d, *J* = 7.4 Hz, 2H), 7.53 (d, *J* = 8.7 Hz, 2H), 7.50 (t, *J* = 7.6 Hz, 3H), 7.42 – 7.34 (m, 5H), 6.98 (d, *J* = 3.7 Hz, 1H), 6.73 (d, *J* = 3.6 Hz, 1H), 5.07 (d, *J* = 12.4 Hz, 1H), 4.70 (d, *J* = 12.4 Hz, 1H), 4.18 (dd, *J* = 10.6, 3.0 Hz, 1H), 3.61 (dd, *J* = 17.3, 10.7 Hz, 1H), 3.42 (dd, *J* = 17.3, 3.0 Hz, 1H),

1.35 (s, 9H). ^{13}C NMR (126 MHz, $CDCl_3$) δ 197.5, 169.4, 148.0, 146.8, 143.6, 136.6, 136.3, 133.2, 131.4, 129.5, 128.6, 128.6, 127.9, 127.5, 125.2, 122.7, 111.9, 87.9, 83.6, 64.3, 51.3, 39.4, 28.0. Peak overlapping was observed. HRMS (ESI) $[M+Na]^+$ calcd for $C_{32}H_{30}NO_6SBrNa^+$, 658.0869, found 658.0867. (Chiral IA-3, λ= 254 nm, *n*-hexane/2-propanol= 19/1, Flow rate = 1.0 mL/min), t_R = 10.980 min, 14.730 min(major)

HPLC chromatogram of racemic S24

Condition: n-hexane/2-propanol =19:1

Flow rate =1.0 mL/min

λ= 254 nm

Chiral IA-3

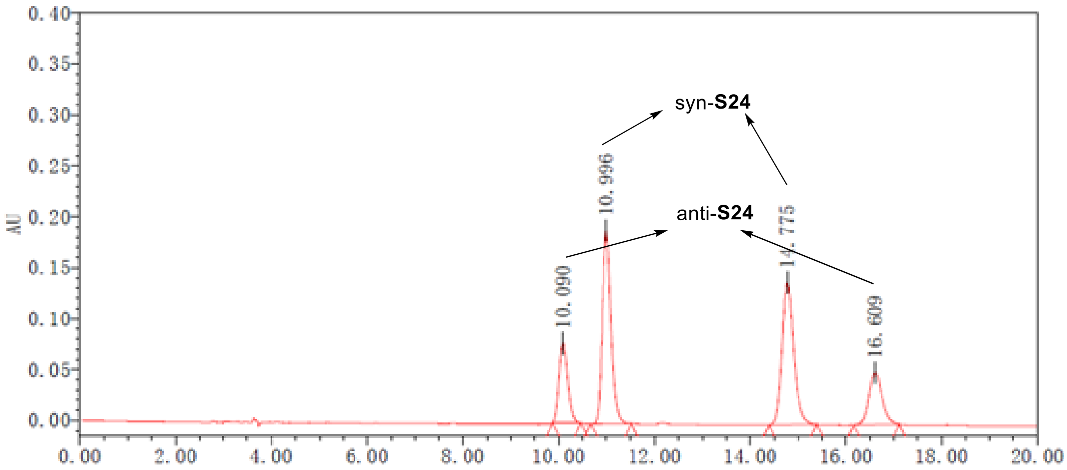

Entry	Retention Time/min	Area	Height	Area(%)
1	10.090	970254	970254	14.27
2	10.996	2461486	2461486	36.21
3	14.775	2391001	2391001	35.17
4	16.609	975821	975821	14.35

Supplementary Figure 258. Chiral HPLC analysis of racemic **S24**

HPLC chromatogram of chiral S24

Condition: n-hexane/2-propanol =19:1

Flow rate =1.0 mL/min

λ= 254 nm

Chiral IA-3

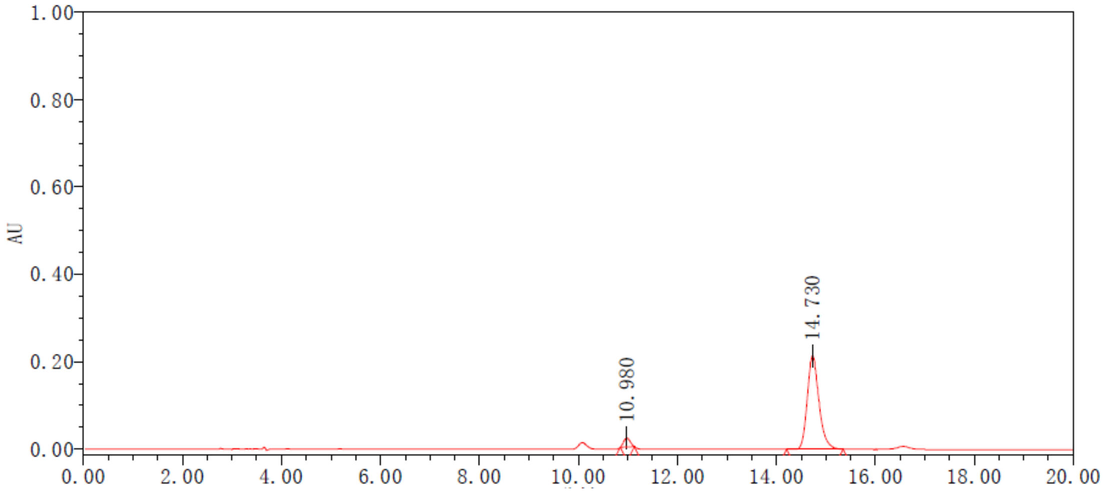

Entry	Retention Time/min	Area	Height	Area(%)
1	10.980	188538	20336	5.03
2	14.730	3557986	213954	94.97

Supplementary Figure 259. Chiral HPLC analysis of chiral **S24**

Tert-butyl (2S,3R)-3-(2-oxo-2-phenylethyl)-2,5-diphenyl-2-((4-(1-tosyl-1H-1,2,3-triazol-4-yl)benzyl)oxy)pent-4-ynoate (S25)

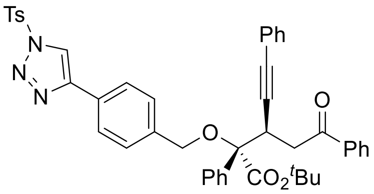

S25

Colorless oil; 64.6mg, 86% yield, >20:1 d.r., 94% ee. ^{1}H NMR (400 MHz, $CDCl_3$) δ 8.31 (s, 1H), 8.03 (d, *J* = 8.4 Hz, 2H), 7.89 – 7.79 (m, 4H), 7.71 (d, *J* = 7.4 Hz, 2H), 7.57 (d, *J* = 8.1 Hz, 2H), 7.51 (t, *J* = 7.4 Hz, 1H), 7.43 – 7.39 (m, 3H), 7.38 – 7.29 (m, 4H), 7.27 – 7.21 (m, 5H), 5.13 (d, *J* = 11.9 Hz, 1H), 4.98 (d, *J* = 11.9 Hz, 1H), 4.43 (dd,

J = 10.2, 2.9 Hz, 1H), 3.35 (dd, J = 16.6, 10.2 Hz, 1H), 3.12 (dd, J = 16.6, 2.9 Hz, 1H), 2.44 (s, 3H), 1.51 (s, 9H). ^{13}C NMR (101 MHz, $CDCl_3$) δ 197.5, 169.5, 147.4, 147.3, 140.1, 138.4, 136.9, 133.1, 131.4, 130.5, 128.7, 128.6, 128.3, 128.3, 128.2, 128.1, 128.0, 127.9, 126.8, 126.1, 123.4, 118.9, 89.7, 85.9, 84.2, 82.9, 68.3, 39.3, 36.6, 29.7, 28.1, 21.9. HRMS (ESI) $[M+Na]^+$ calcd for $C_{45}H_{41}N_3O_6SNa^+$, 774.2608, found 774.2606. (Chiral IA, λ= 254 nm, *n*-hexane/2-propanol= 17/3, Flow rate = 1.0 mL/min), t_R = 35.095 min(major), 67.281 min.

HPLC chromatogram of racemic S25

Condition: n-hexane/2-propanol = 17:3

Flow rate =1.0 mL/min

λ= 254 nm

Chiral IA

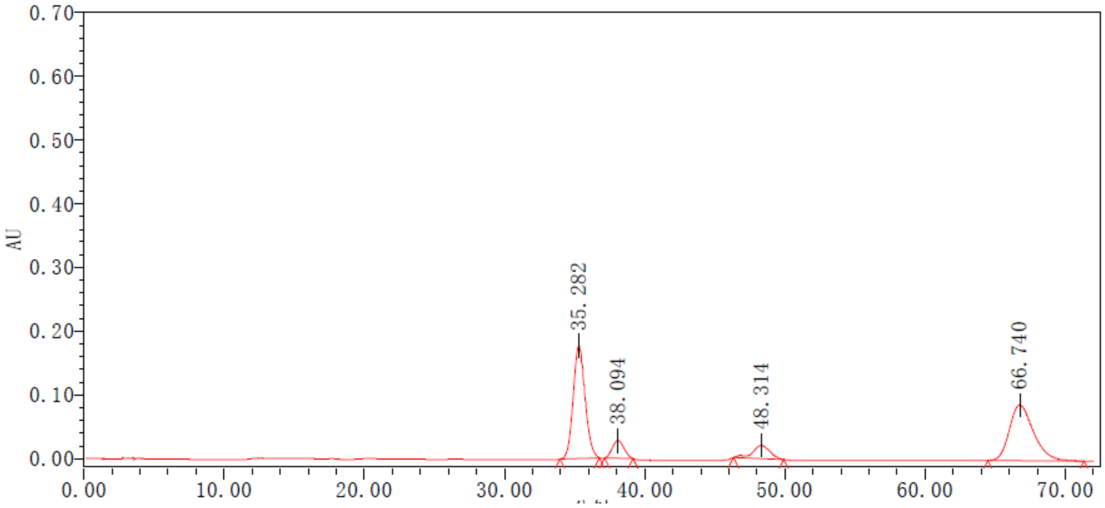

Entry	Retention Time/min	Area	Height	Area(%)
1	35.282	10540918	177043	43.35
2	38.094	1613880	28161	6.64
3	48.314	1742795	20630	7.17
4	66.740	10416644	87076	42.84

Supplementary Figure 260. Chiral HPLC analysis of racemic **S25**

HPLC chromatogram of chiral S25

Condition: n-hexane/2-propanol =17:3

Flow rate =1.0 mL/min

λ= 254 nm

Chiral IA

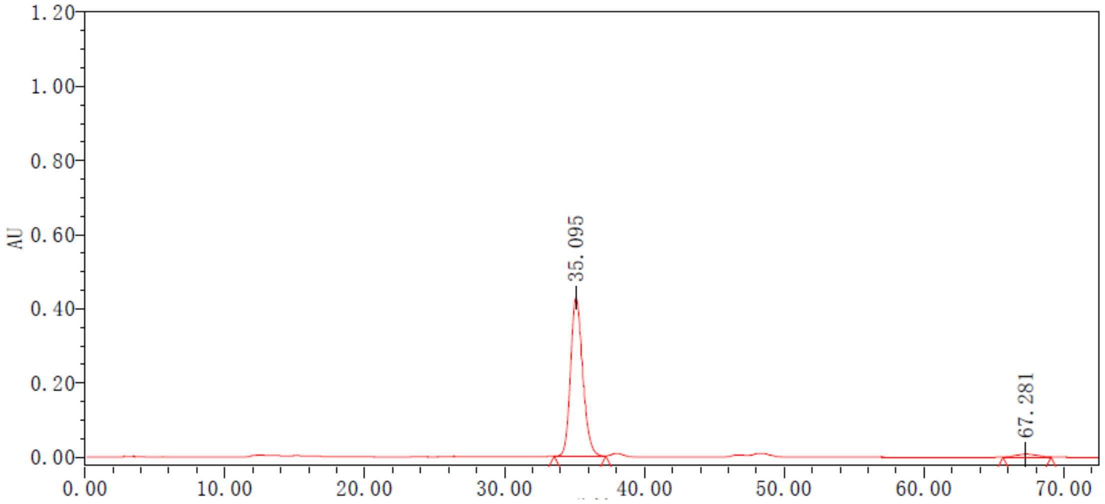

Entry	Retention Time/min	Area	Height	Area(%)
1	35.095	25922700	427421	96.96
2	67.281	811921	7766	3.04

Supplementary Figure 261. Chiral HPLC analysis of chiral **S25**

Tert-butyl (2S,3R)-2-((4-(1H-1,2,3-triazol-4-yl)benzyl)oxy)-3-((E)-2-(hydroxyimino)-2-phenylethyl)-2,5-diphenylpent-4-ynoate (S26)

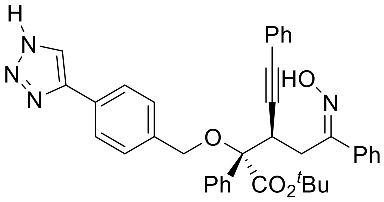

S26

White solid, 23.0 mg, 75% yield, >20:1 d.r., 92% ee. ^{1}H NMR (400 MHz, $CDCl_3$) δ 8.71 (s, 1H), 7.80 (d, J = 8.1 Hz, 2H), 7.70 (d, J = 7.0 Hz, 2H), 7.65 (d, J = 6.0 Hz, 1H), 7.57 – 7.50 (m, 4H), 7.37 (t, J = 5.6 Hz, 4H), 7.34 – 7.31 (m, 3H), 7.28 (d, J = 7.8 Hz,

4H), 7.19 – 7.12 (m, 3H), 7.04 – 7.00 (m, 2H), 5.57 (s, 2H), 5.16 (d, *J* = 11.8 Hz, 1H), 4.95 (d, *J* = 11.7 Hz, 1H), 4.19 (dd, *J* = 11.7, 4.1 Hz, 1H), 3.21 (t, *J* = 12.4 Hz, 1H), 2.90 (dd, *J* = 13.0, 4.1 Hz, 1H), 1.48 (s, 9H). ^{13}C NMR (101 MHz, $CDCl_3$) δ 169.6, 157.6, 148.2, 139.3, 138.5, 136.1, 134.7, 131.3, 131.3, 129.6, 129.2, 129.0, 128.8, 128.4, 128.3, 128.2, 128.1, 128.1, 128.1, 128.0, 127.8, 127.7, 127.4, 126.8, 126.7, 125.6, 123.5, 119.5, 89.4, 85.96, 84.8, 82.7, 68.4, 54.3, 37.5, 29.7, 28.0, 26.1. HRMS (ESI) $[M+Na]^+$ calcd for $C_{38}H_{36}N_4O_4Na^+$, 635.2629, found 635.2625. (Chiral IA, λ= 254 nm, *n*-hexane/2-propanol= 39/11, Flow rate = 1.0 mL/min), t_R = 13.353 min, 34.548 min (major).

HPLC chromatogram of racemic S26

Condition: n-hexane/2-propanol = 39:11

Flow rate =1.0 mL/min

λ= 254 nm

Chiral IA

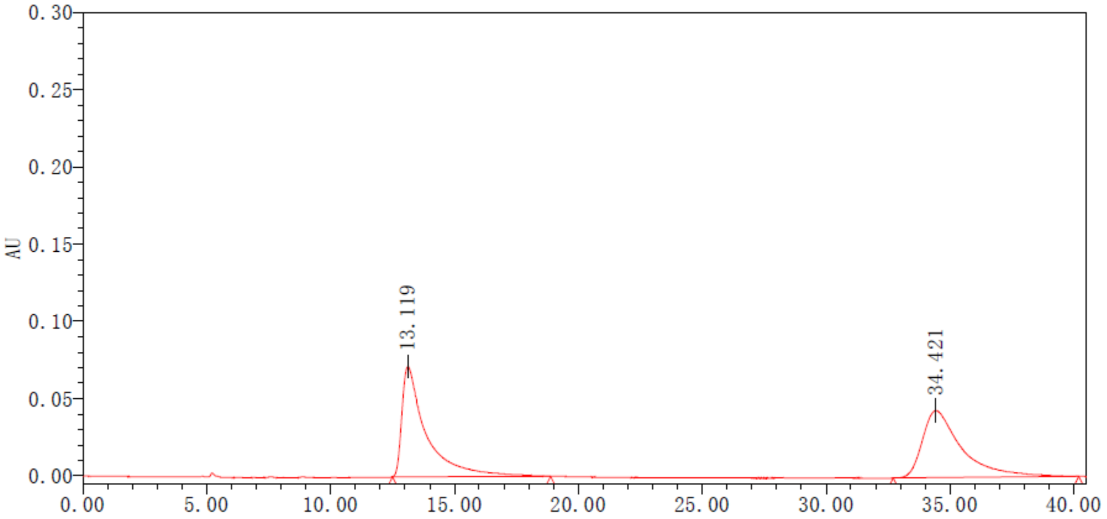

Entry	Retention Time/min	Area	Height	Area(%)
1	13.119	4815388	71330	50.28
2	34.421	4815388	43480	49.72

Supplementary Figure 262. Chiral HPLC analysis of racemic **S26**

HPLC chromatogram of chiral S26

Condition: n-hexane/2-propanol =39:11

Flow rate =1.0 mL/min

λ= 254 nm

Chiral IA

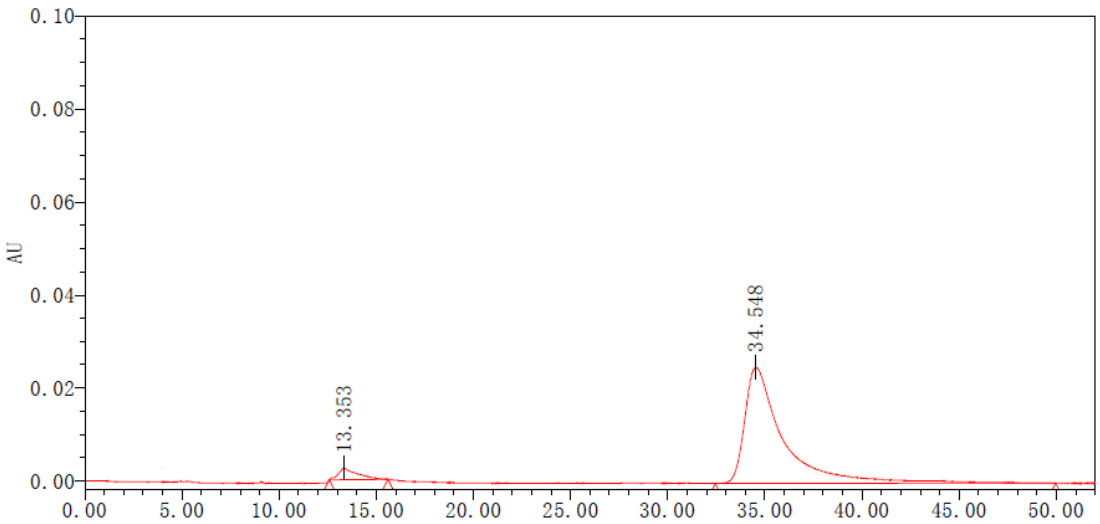

Entry	Retention Time/min	Area	Height	Area(%)
1	13.353	149153	2539	4.11
2	34.548	3479877	24976	95.89

Supplementary Figure 263. Chiral HPLC analysis of chiral **S26**

Tert-butyl (2S,3R)-2-((4-(1-benzyl-1H-1,2,3-triazol-4-yl)benzyl)oxy)-3-(2-oxo-2-phenylethyl)-2,5-diphenylpent-4-ynoate (S27)

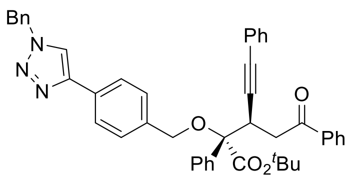

S27

Colorless oil; 60.4 mg, 88% yield, >20:1 d.r., 94% *ee*; ^{1}H NMR (500 MHz, $CDCl_3$) δ 7.84 (d, *J* = 7.4 Hz, 2H), 7.80 (d, *J* = 8.1 Hz, 2H), 7.71 (d, *J* = 7.5 Hz, 2H), 7.66 (s, 1H), 7.54 (d, *J* = 8.1 Hz, 2H), 7.50 (t, *J* = 7.4 Hz, 1H), 7.39 (t, *J* = 5.4 Hz, 4H), 7.37 – 7.34 (m, 3H), 7.33 – 7.28 (m, 3H), 7.26 – 7.20 (m, 5H), 5.58 (s, 2H), 5.13 (d, *J* = 11.7 Hz,

1H), 4.97 (d, J = 11.7 Hz, 1H), 4.41 (dd, J = 10.3, 2.9 Hz, 1H), 3.35 (dd, J = 16.6, 10.3 Hz, 1H), 3.10 (dd, J = 16.6, 2.9 Hz, 1H), 1.51 (s, 9H). ^{13}C NMR (126 MHz, $CDCl_3$) δ 197.5, 169.5, 148.2, 139.1, 138.5, 137.0, 134.7, 133.1, 131.4, 129.7, 129.2, 128.8, 128.5, 128.2, 128.2, 128.2, 128.2, 128.1, 128.0, 127.8, 126.8, 125.7, 123.5, 119.4, 89.8, 85.9, 84.1, 82.8, 68.4, 54.3, 39.2, 36.7, 28.1. HRMS (ESI) $[M+Na]^+$ calcd for $C_{45}H_{41}N_3O_4Na^+$, 710.2989, found 710.2987. (Chiral IA-3, λ= 254 nm, *n*-hexane/2-propanol= 17/1, Flow rate = 1.0 mL/min), t_R = 35.411 min(major), 60.438 min.

HPLC chromatogram of racemic S27

Condition: n-hexane/2-propanol =17:1

Flow rate =1.0 mL/min

λ= 254 nm

Chiral IA-3

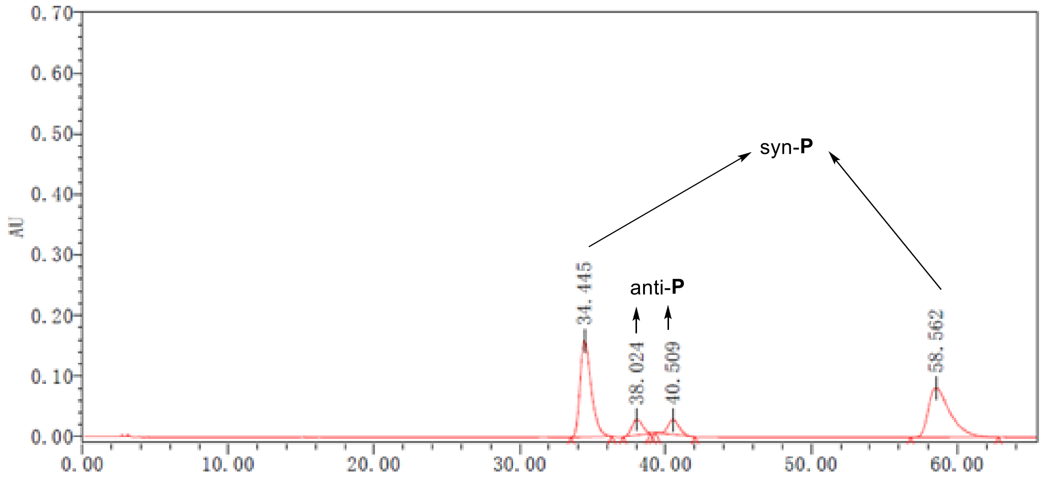

Entry	Retention Time/min	Area	Height	Area(%)
1	34.445	8393787	159635	43.27
2	38.024	1267538	25436	6.53
3	40.509	1267898	24111	6.54
4	58.562	8469407	81582	43.66

Supplementary Figure 264. Chiral HPLC analysis of racemic **S27**

HPLC chromatogram of chiral S27

Condition: n-hexane/2-propanol =17:1

Flow rate =1.0 mL/min

λ= 254 nm

Chiral IE-3

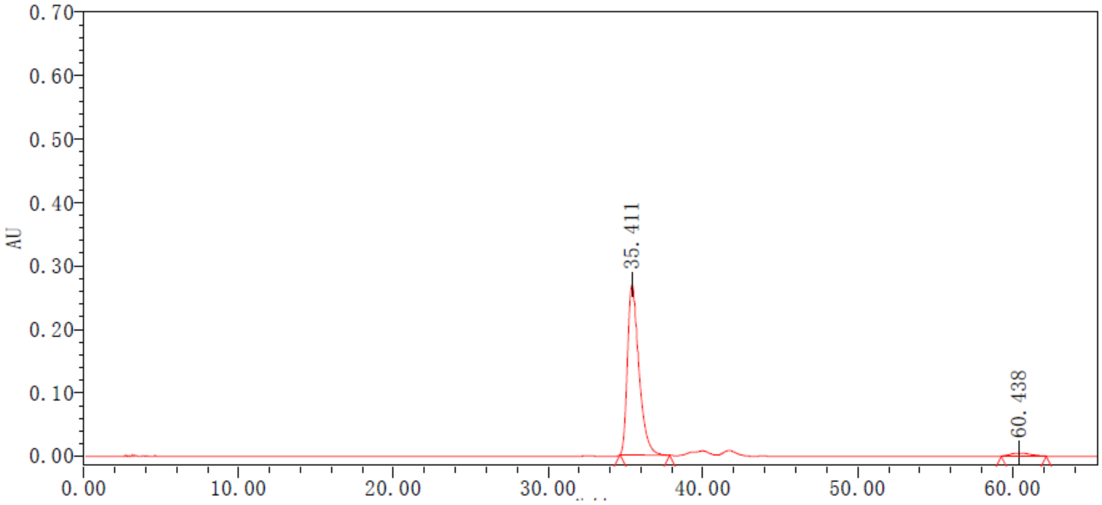

Entry	Retention Time/min	Area	Height	Area(%)
1	35.411	13996459	268264	97.04
2	60.438	426965	4857	2.96

Supplementary Figure 265. Chiral HPLC analysis of chiral **S27**

Tert-butyl (2S,3R)-2-((4-(1-benzyl-1H-1,2,3-triazol-4-yl)benzyl)oxy)-3-((E)-2-(hydroxyimino)-2-phenylethyl)-2,5-diphenylpent-4-ynoate (S28)

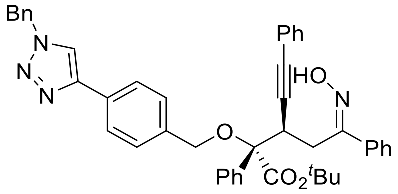

S28

White solid; 31.6 mg, 75% yield, >20:1 d.r., 94% *ee*; ^{1}H NMR (400 MHz, $CDCl_3$) δ 8.71 (s, 1H), 7.80 (d, *J* = 8.1 Hz, 2H), 7.70 (d, *J* = 7.0 Hz, 2H), 7.65 (d, *J* = 6.0 Hz, 1H), 7.57 – 7.50 (m, 4H), 7.37 (t, *J* = 5.6 Hz, 4H), 7.34 – 7.31 (m, 3H), 7.28 (d, *J* = 7.8 Hz,

4H), 7.19 – 7.12 (m, 3H), 7.04 – 7.00 (m, 2H), 5.57 (s, 2H), 5.16 (d, J = 11.8 Hz, 1H), 4.95 (d, J = 11.7 Hz, 1H), 4.19 (dd, J = 11.7, 4.1 Hz, 1H), 3.21 (t, J = 12.4 Hz, 1H), 2.90 (dd, J = 13.0, 4.1 Hz, 1H), 1.48 (s, 9H). ^{13}C NMR (101 MHz, $CDCl_3$) δ 169.6, 157.6, 148.2, 139.3, 138.5, 136.1, 134.7, 131.3, 129.6, 129.2, 129.0, 128.8, 128.4, 128.3, 128.2, 128.1, 128.1, 128.0, 127.8, 127.7, 127.4, 126.7, 125.6, 123.5, 119.5, 89.4, 86.0, 84.8, 82.7, 68.4, 54.3, 37.5, 29.7, 28.0, 26.1. HRMS (ESI) $[M+Na]^+$ calcd for $C_{45}H_{42}N_4O_4Na^+$, 725.3098, found 725.3096. (Chiral IA, λ= 254 nm, *n*-hexane/2-propanol= 4/1, Flow rate = 1.0 mL/min), t_R = 45.063 min(major), 74.588 min.

HPLC chromatogram of racemic S28

Condition: n-hexane/2-propanol = 4:1

Flow rate =1.0 mL/min

λ= 254 nm

Chiral IA

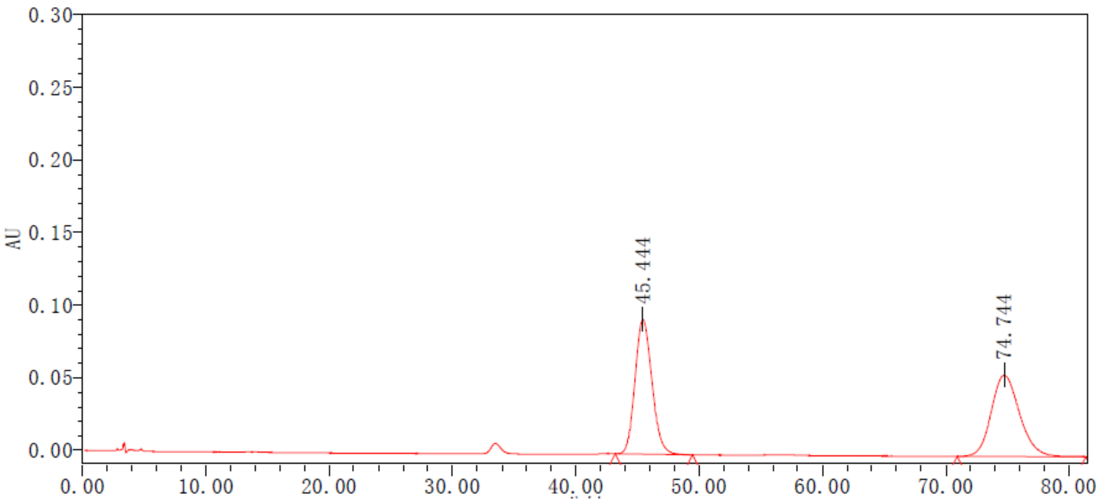

Entry	Retention Time/min	Area	Height	Area(%)
1	45.444	8793773	92761	49.71
2	74.744	8897465	55894	50.29

Supplementary Figure 266. Chiral HPLC analysis of racemic **S28**

HPLC chromatogram of chiral S28

Condition: n-hexane/2-propanol =4:1

Flow rate =1.0 mL/min

λ= 254 nm

Chiral IA

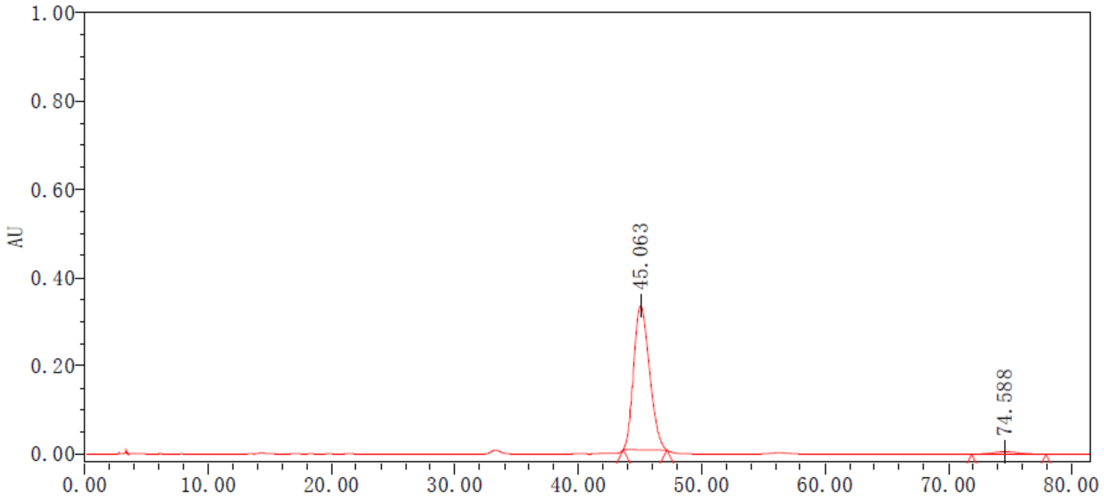

Entry	Retention Time/min	Area	Height	Area(%)
1	45.063	28916180	326482	97.19
2	74.588	835533	5543	2.81

Supplementary Figure 267. Chiral HPLC analysis of chiral **S28**

Tert-butyl 2-(cinnamyloxy)-2-phenylacetate (By-product-1)

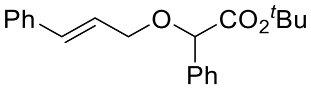

By-product-**1**

Colorless oil; 31.6 mg, 75% yield; ^{1}H NMR (400 MHz, $CDCl_3$) δ 7.46 (dd, *J* = 7.9, 1.2 Hz, 2H), 7.39 – 7.35 (m, 3H), 7.34 – 7.26 (m, 4H), 7.26 – 7.22 (m, 1H), 6.59 (d, *J* = 15.9 Hz, 1H), 6.32 (dt, *J* = 15.9, 6.2 Hz, 1H), 4.85 (s, 1H), 4.22 (dd, *J* = 6.2, 1.1 Hz, 2H), 1.38 (s, 9H). ^{13}C NMR (101 MHz, $CDCl_3$) δ 170.0, 137.0, 136.6, 133.2, 128.6, 128.5, 128.4, 127.8, 127.3, 126.6, 125.5, 81.8, 80.2, 77.4, 77.1, 76.8, 70.0, 27.9. HRMS (ESI) $[M+Na]^+$ calcd for $C_{21}H_{24}O_3Na^+$, 347.1620, found 347.1618.

1,5-diphenyl-3-(phenylethynyl)pentane-1,5-dione (By-product-2)

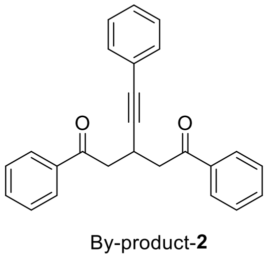

By-product-**2**

^{1}H NMR (400 MHz, $CDCl_3$) δ 8.07 – 7.99 (m, 4H), 7.60 – 7.53 (m, 2H), 7.48 (t, *J* = 7.6 Hz, 4H), 7.29 – 7.25 (m, 3H), 7.24 – 7.20 (m, 2H), 3.95 (p, *J* = 6.7 Hz, 1H), 3.47 (dd, *J* = 16.7, 6.7 Hz, 2H), 3.36 (dd, *J* = 16.7, 6.7 Hz, 2H). ^{13}C NMR (101 MHz, $CDCl_3$) δ 197.7, 136.9, 133.3, 131.7, 128.7, 128.3, 128.1, 127.8, 123.3, 100.0, 91.0, 43.1, 29.7, 24.2. HRMS (ESI) $[M+Na]^+$ calcd for $C_{25}H_{20}O_2Na^+$, 375.1356, found 375.1357.

Tert-butyl (2R,3R)-3-(4-methoxyphenyl)-2-phenyloxirane-2-carboxylate (By-product-3)

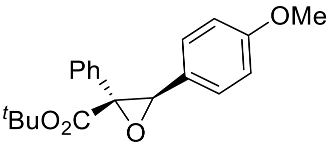

Byproduct-**3**

^{1}H NMR (500 MHz, $CDCl_3$) δ 7.65 – 7.59 (m, 1H), 7.41 (d, *J* = 1.7 Hz, 1H), 7.39 (d, *J* = 7.6 Hz, 1H), 7.37 (d, *J* = 7.0 Hz, 1H), 7.35 (d, *J* = 8.7 Hz, 2H), 6.89 (d, *J* = 8.7 Hz, 2H), 4.06 (s, 1H), 3.81 (s, 3H), 1.23 (s, 9H). ^{13}C NMR (101 MHz, $CDCl_3$) δ 165.7, 159.8, 135.4, 128.5, 127.6, 126.3, 126.1, 113.6, 82.7, 66.9, 65.6, 55.4, 27.7. HRMS (ESI) $[M+Na]^+$ calcd for $C_{20}H_{22}O_4Na^+$, 349.1410, found 349.1407.

Tert-Butyl 2-((4-bromobenzyl)oxy)-2-phenylacetate (By-product-4)

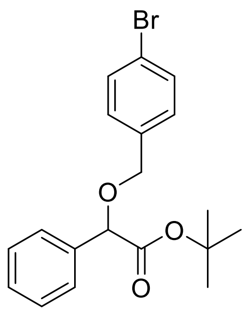

By-product-**4**

White solid, mp: 87.7 – 88.5 °C; 63.9 mg, 85% yield; ^{1}H NMR (400 MHz, $CDCl_3$) δ 7.49 – 7.41 (m, 4H), 7.39 – 7.32 (m, 3H), 7.25 (d, *J* = 8.6 Hz, 2H), 4.78 (s, 1H), 4.56 (d, *J* = 12.0 Hz, 1H), 4.51 (d, *J* = 12.0 Hz, 1H), 1.39 (s, 9H). ^{13}C NMR (126 MHz, $CDCl_3$) δ 169.8, 136.6, 136.6, 131.5, 129.6, 128.54, 128.52, 127.3, 121.7, 82.0, 80.3, 70.3, 27.9. HRMS(ESI) $[M + Na]^+$ calcd for $C_{19}H_{21}BrO_3Na^+$, 399.0566, found 399.0566.

3,3'-(3-Phenylprop-2-yne-1,1-diyl)bis(2-methyl-1*H*-indole) (By-product-5)

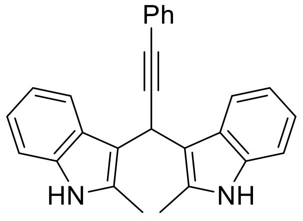

By-product-**5**

^{1}H NMR (400 MHz, Acetone) δ 8.95 (s, 2H), 6.89 (d, *J* = 6.7 Hz, 2H), 6.54 (s, 2H), 6.40 (s, 5H), 6.08 (dd, *J* = 21.4, 6.6 Hz, 4H), 5.00 (s, 1H), 1.60 (s, 6H). ^{13}C NMR (101 MHz, Acetone) δ 135.5, 131.6, 131.3, 128.5, 128.2, 127.7, 124.3, 120.3, 118.8, 118.6, 110.4, 110.3, 92.1, 81.5, 25.8, 11.7. HRMS(ESI) $[M + Na]^+$ calcd for $C_{27}H_{22}N_2Na^+$, 374.1675, found 374.1665.

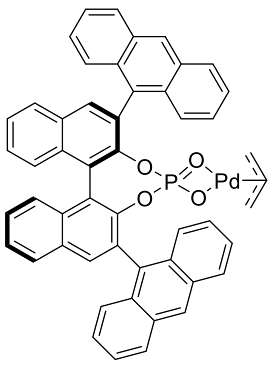

[Pd(allyl)(*R*)-**6b**]

Fluffy yellowish powder (118.47 mg, 70% yield).^{1}H NMR (500 MHz, $CDCl_3$) δ 8.48 – 8.14 (m, 2H), 8.00 – 7.76 (m, 9H), 7.60 (d, *J* = 7.7 Hz, 4H), 7.48 – 7.32 (m, 5H), 7.28 (s, 2H), 7.16 (d, *J* = 32.9 Hz, 6H), 4.57 (br, 1H), 3.09 (br, 2H), 1.96 (br, 1H). ^{13}C NMR (126 MHz, $CDCl_3$) δ 148.2, 133.1, 133.0, 131.7, 131.6, 131.5, 131.3, 131.4, 130.7, 129.8, 128.7, 128.7, 128.6, 128.5, 128.4, 128.2, 127.4, 127.3, 127.2, 127.1, 126.6, 126.3, 126.2, 125.9, 125.4, 125.1, 125.0, 124.9, 123.1, 110.3, 44.7, 3.75, 7.4. ^{31}P NMR (202 MHz, $CDCl_3$) δ 13.59. HRMS (ESI) $[M+Na]^+$ calcd for $C_{51}H_{35}O_4PPdNa^+$, 871.1218, found 871.1238.

10. NMR Spectra for the Products

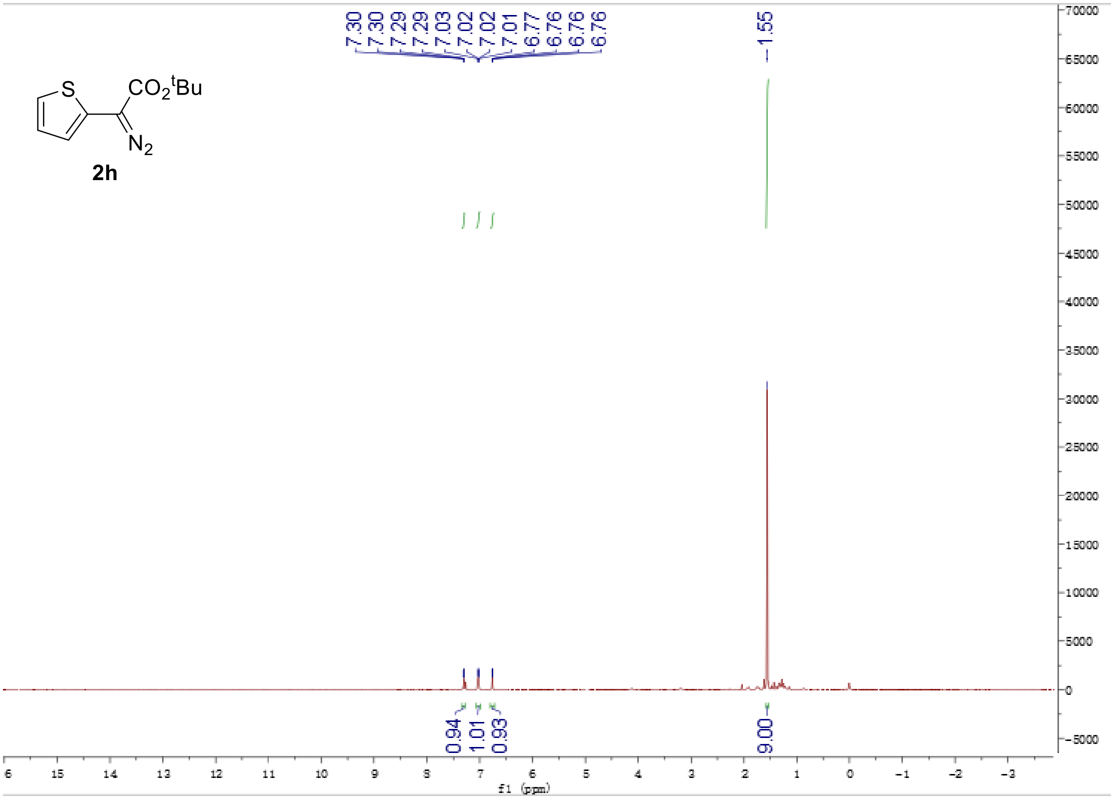

Supplementary Figure 268. ^{1}H NMR (400 MHz, $CDCl_3$) spectrum of **2h**.

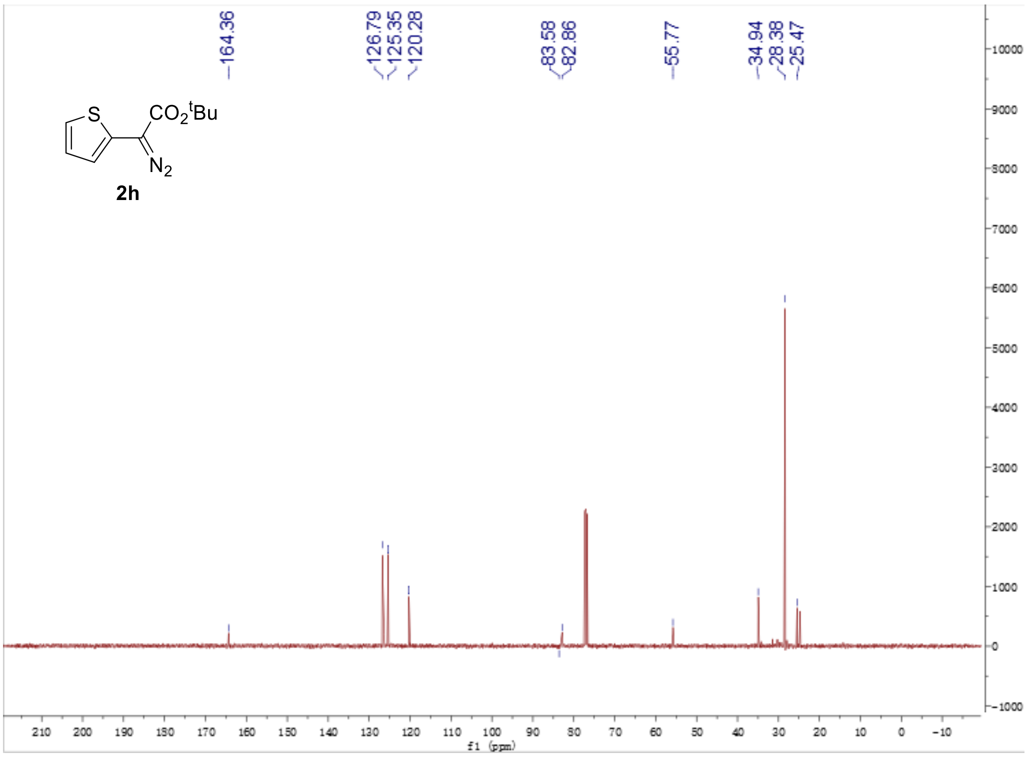

Supplementary Figure 269. ^{13}C NMR (101 MHz, $CDCl_3$) spectrum of **2h**.

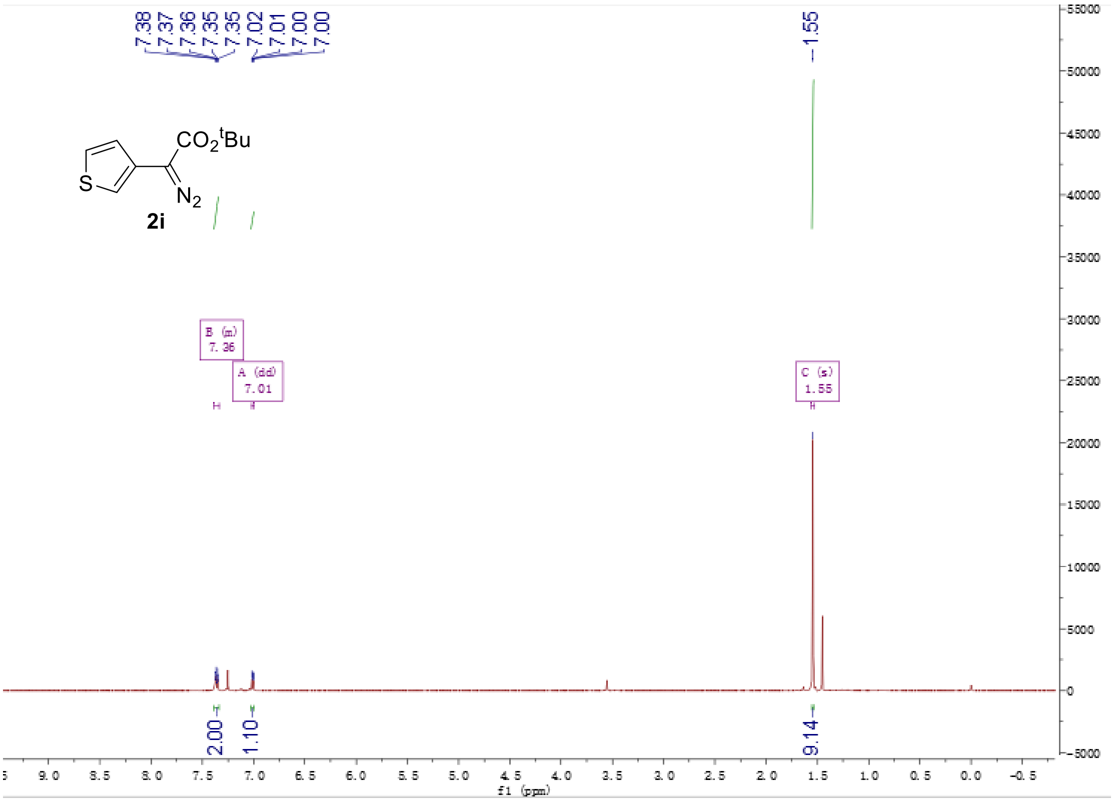

Supplementary Figure 270. ^{1}H NMR (400 MHz, $CDCl_3$) spectrum of **2i**.

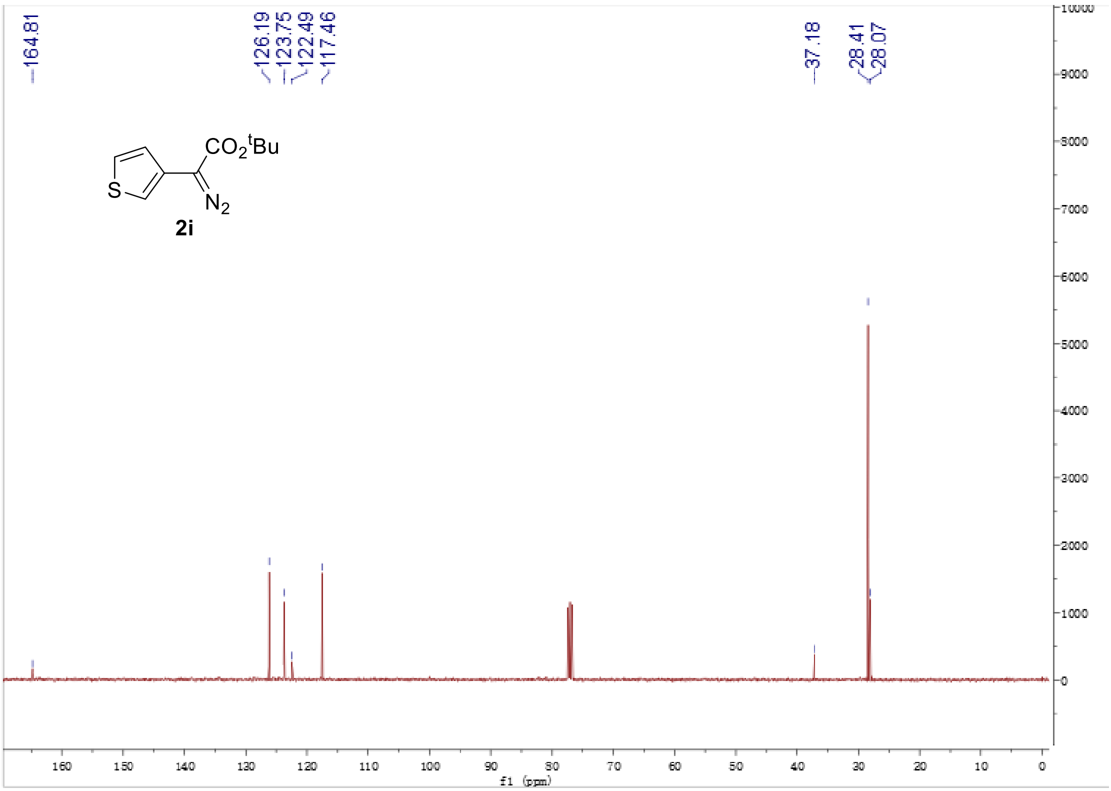

Supplementary Figure 271. ^{13}C NMR (101 MHz, $CDCl_3$) spectrum of **2i**.

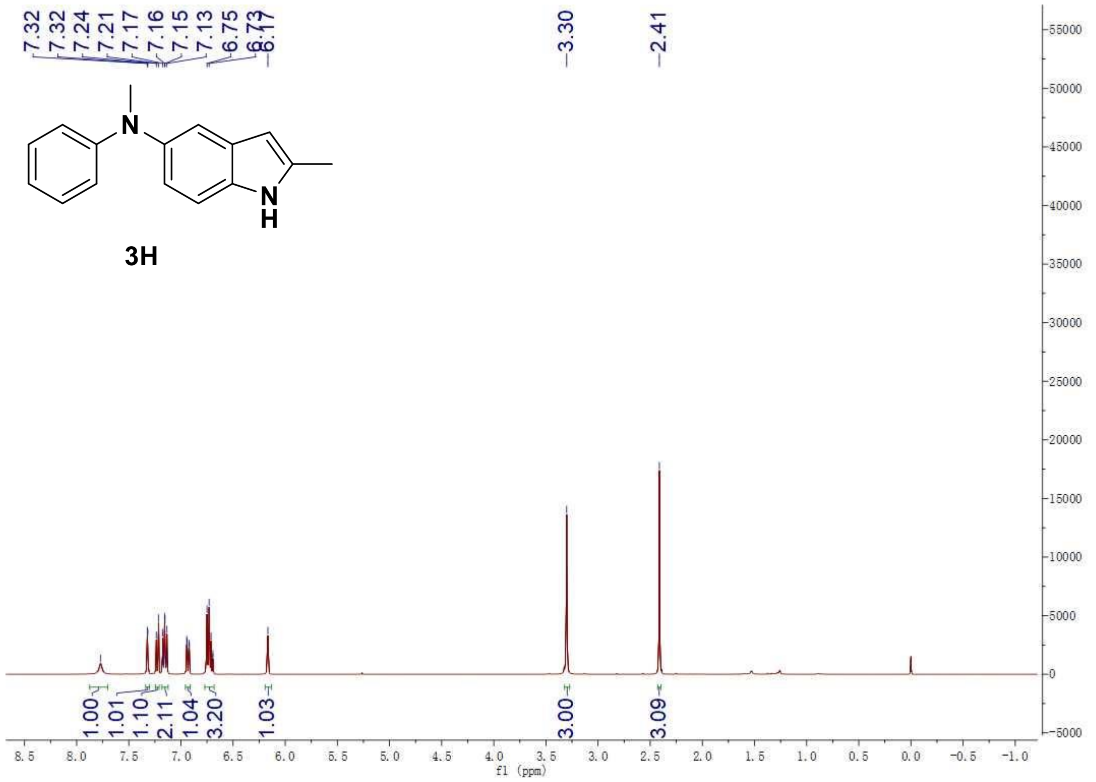

Supplementary Figure 272. ^{1}H NMR (400 MHz, $CDCl_3$) spectrum of **3H**.

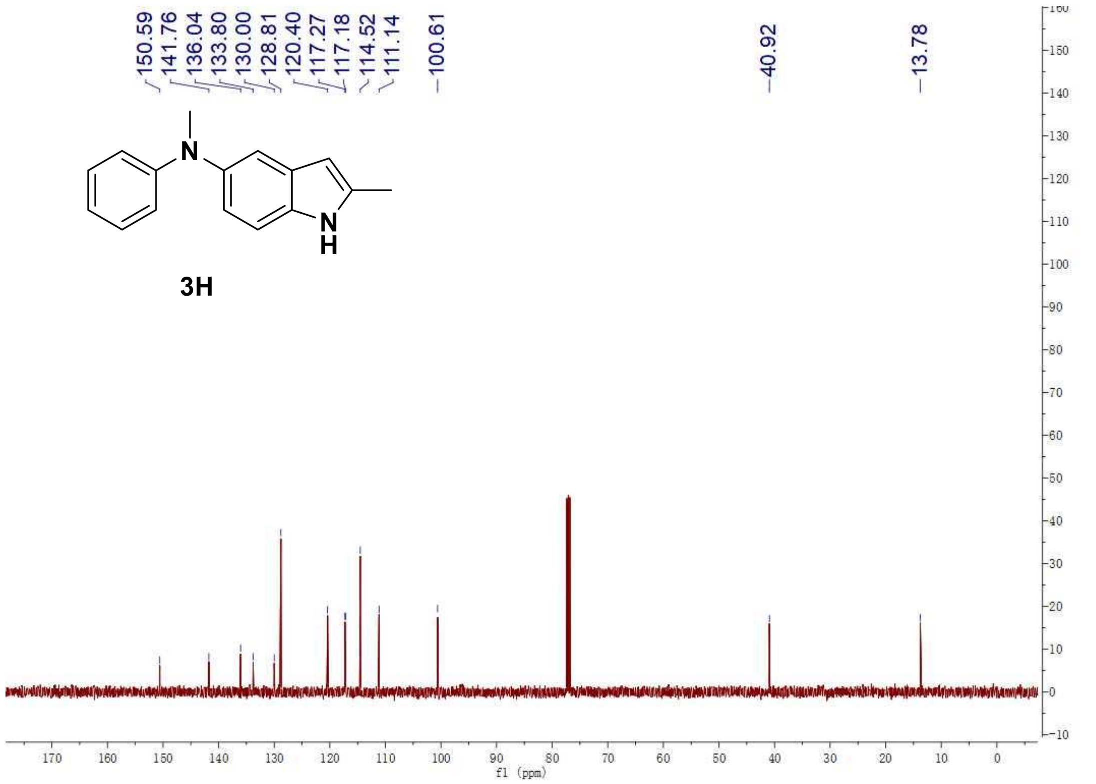

Supplementary Figure 273. ^{13}C NMR (101 MHz, $CDCl_3$) spectrum of **3H**.

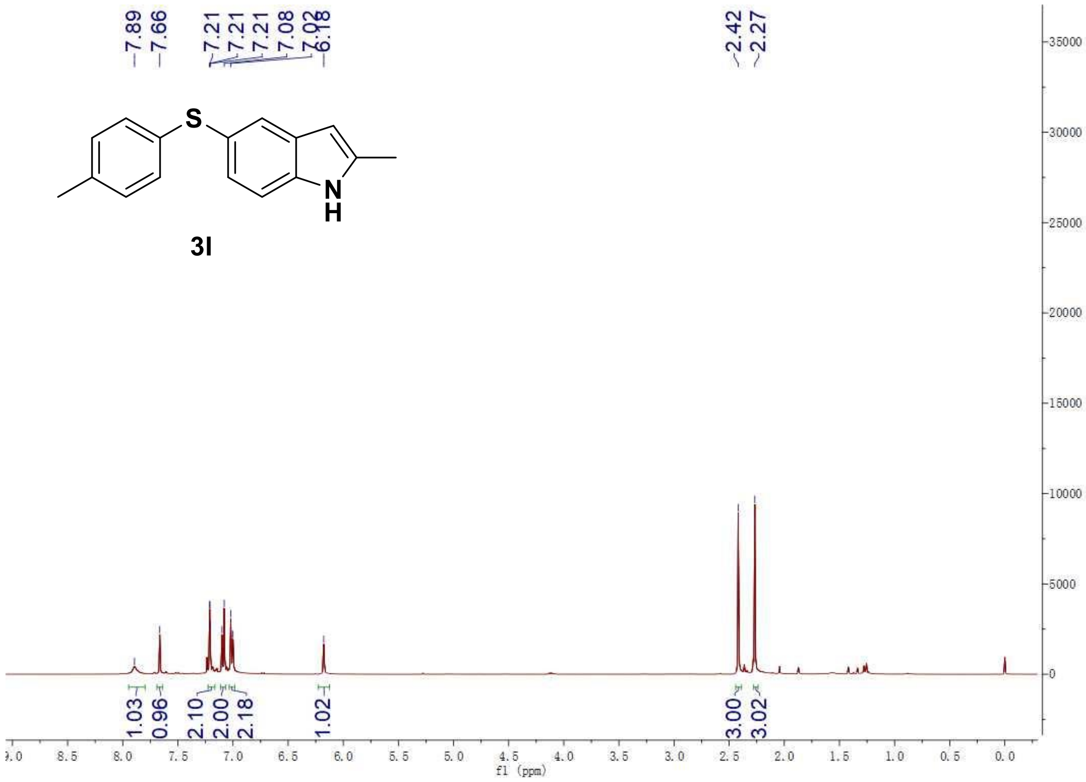

Supplementary Figure 274. ^{1}H NMR (400 MHz, $CDCl_3$) spectrum of **3l**.

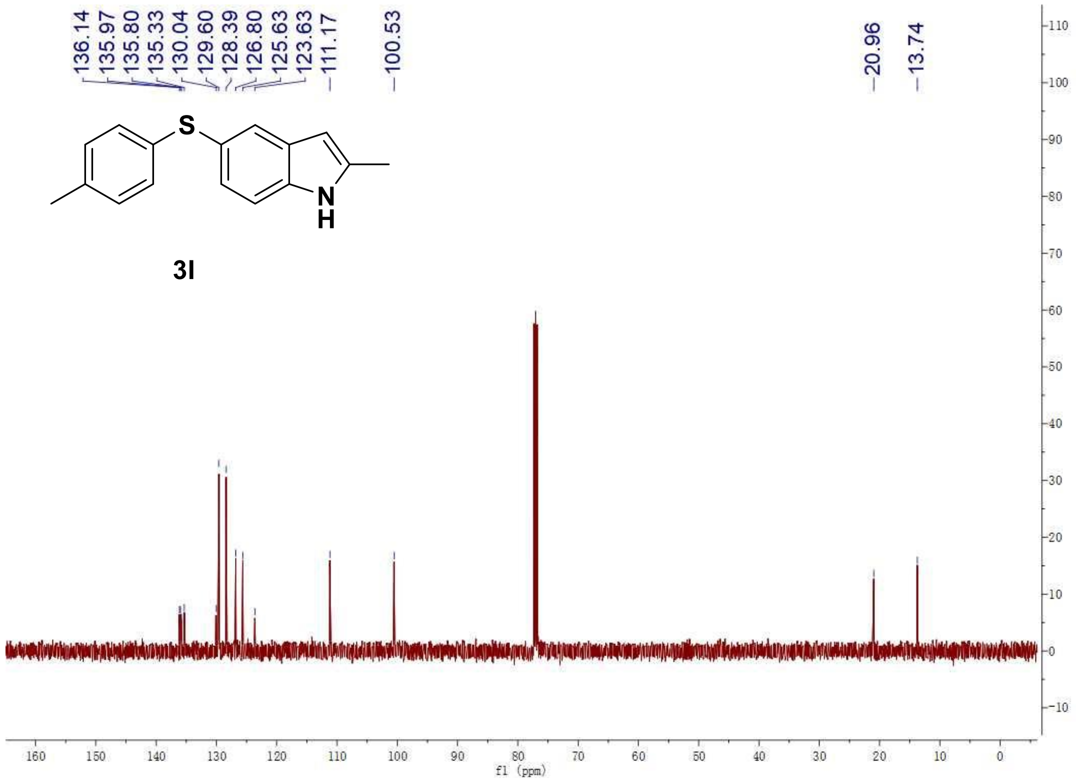

Supplementary Figure 275. ^{13}C NMR (101 MHz, $CDCl_3$) spectrum of **3l**.

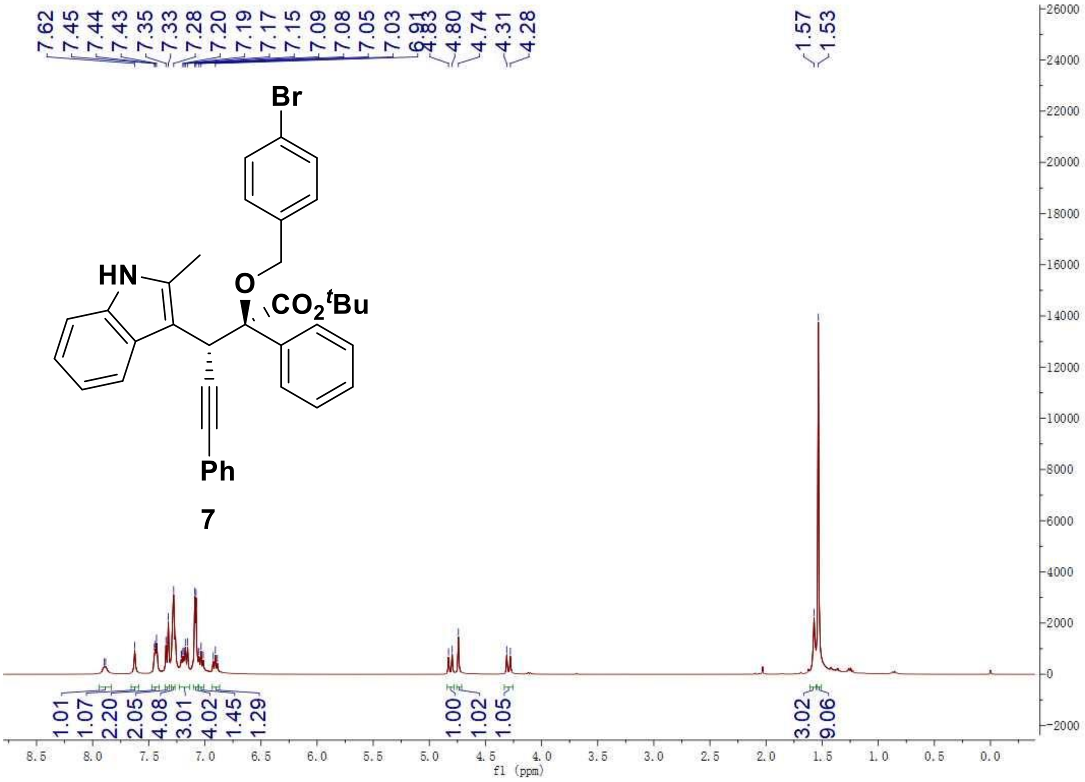

Supplementary Figure 276. ^{1}H NMR (400 MHz, $CDCl_3$) spectrum of **7**.

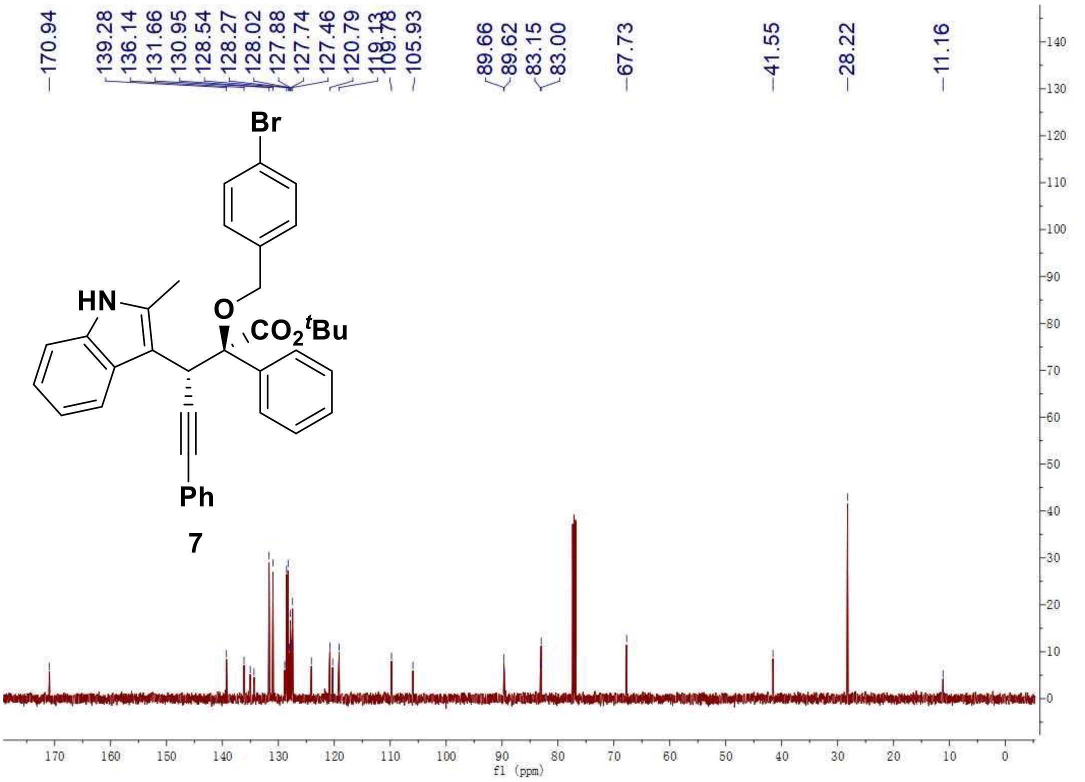

Supplementary Figure 277. ^{13}C NMR (101 MHz, $CDCl_3$) spectrum of **7**.

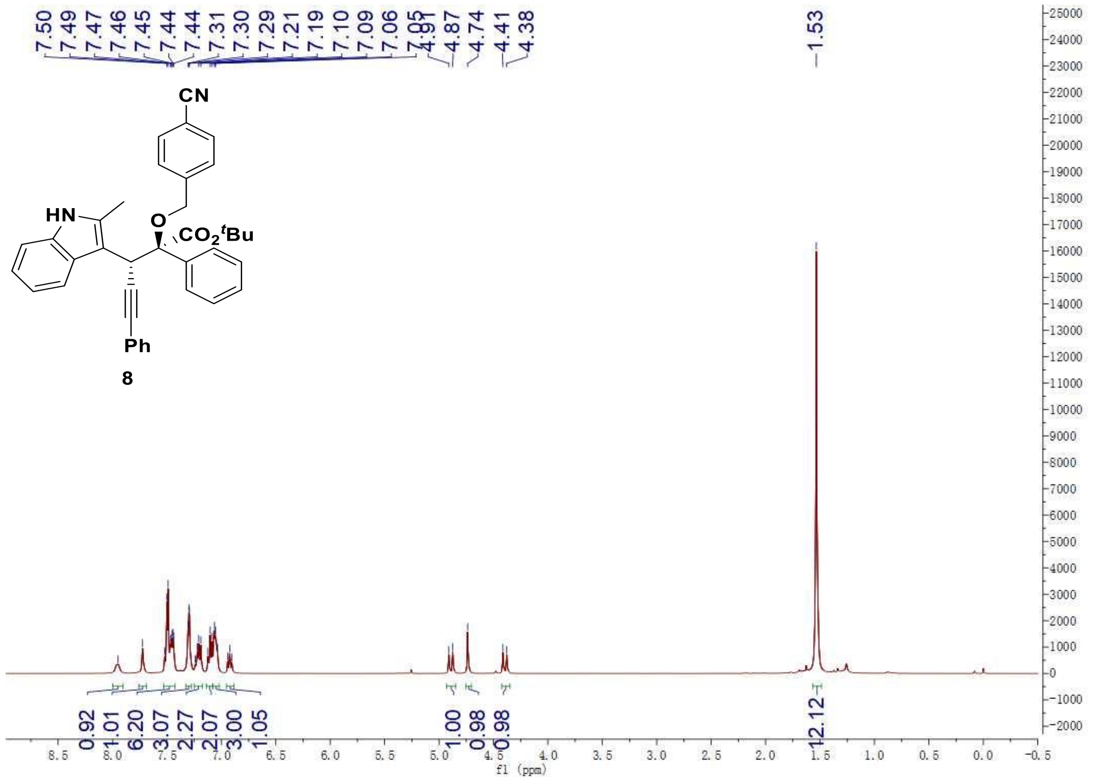

Supplementary Figure 278. ^{1}H NMR (400 MHz, $CDCl_3$) spectrum of **8**.

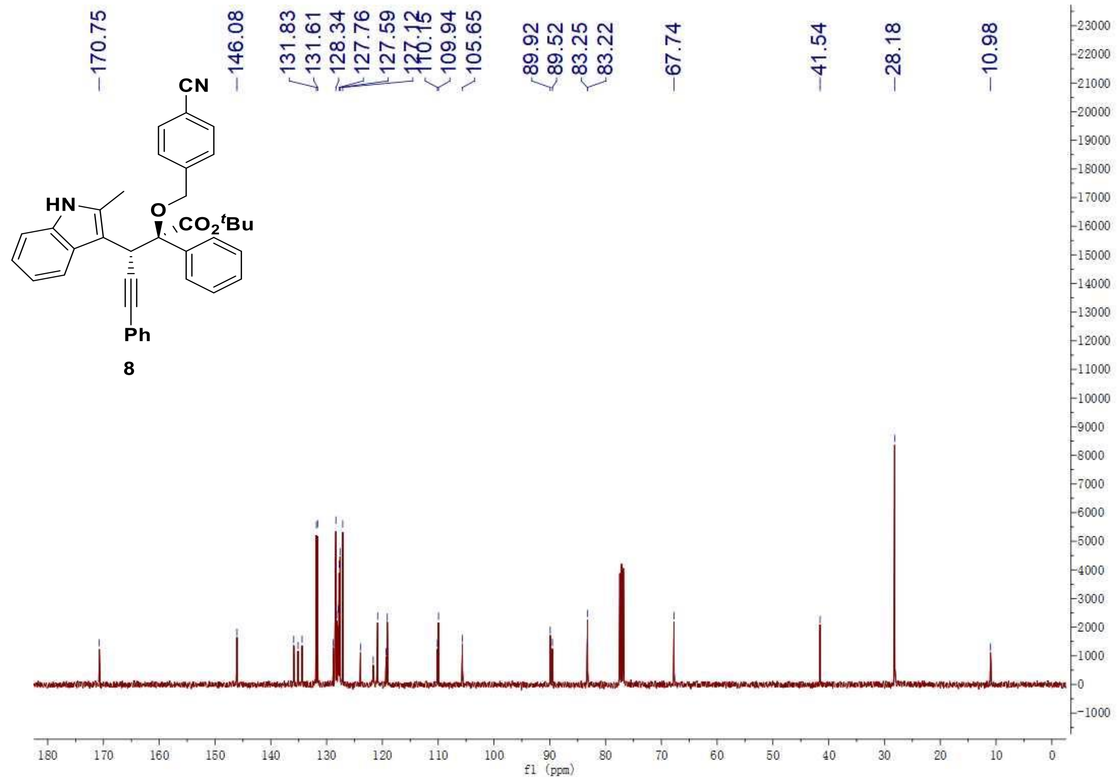

Supplementary Figure 279. ^{13}C NMR (101 MHz, $CDCl_3$) spectrum of **8**.

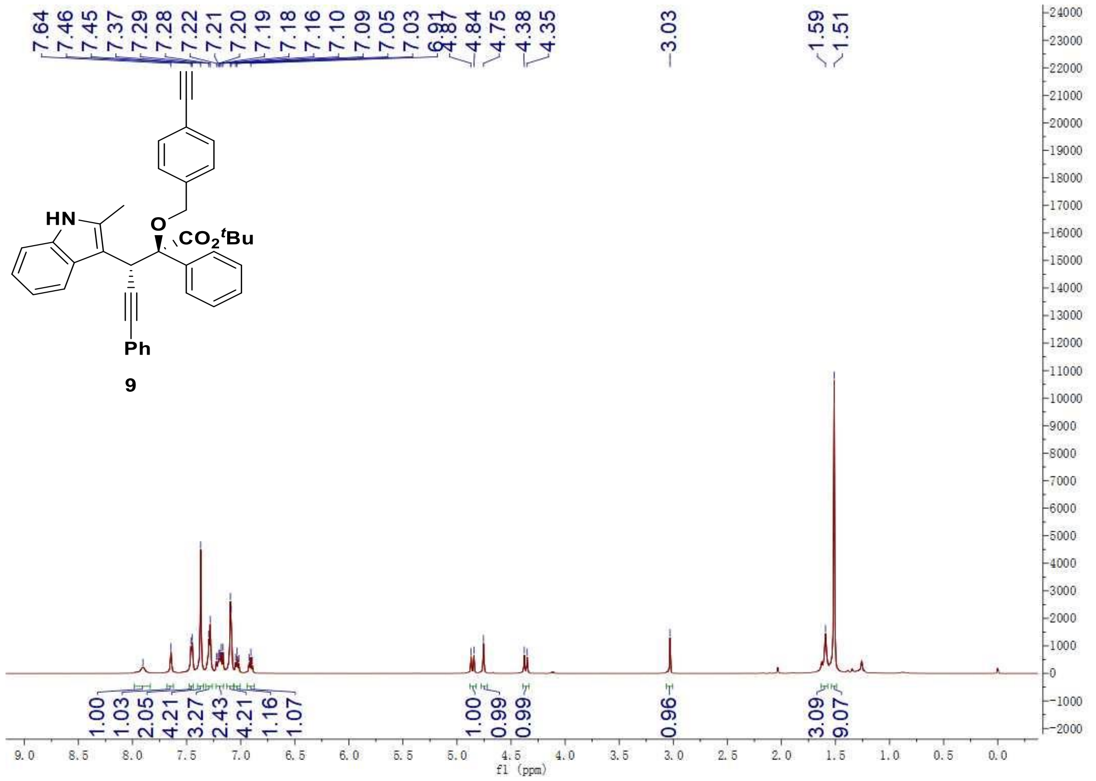

Supplementary Figure 280. ^{1}H NMR (500 MHz, $CDCl_3$) spectrum of **9**.

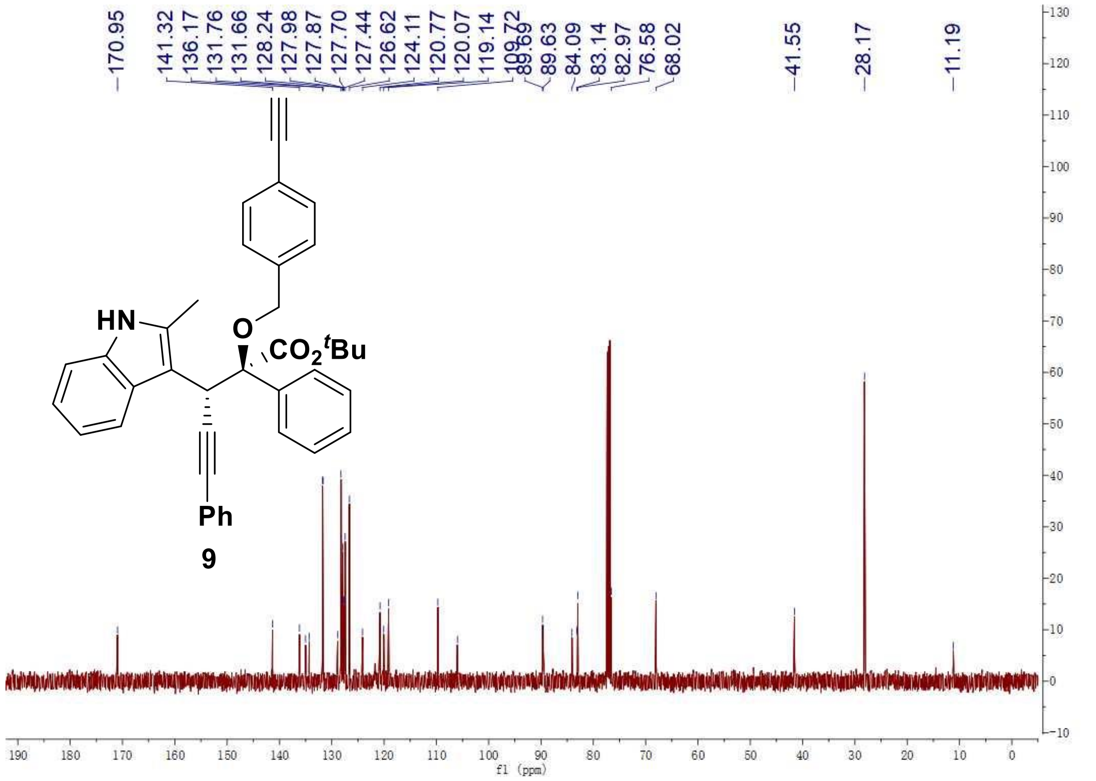

Supplementary Figure 281. ^{13}C NMR (126 MHz, $CDCl_3$) spectrum of **9**.

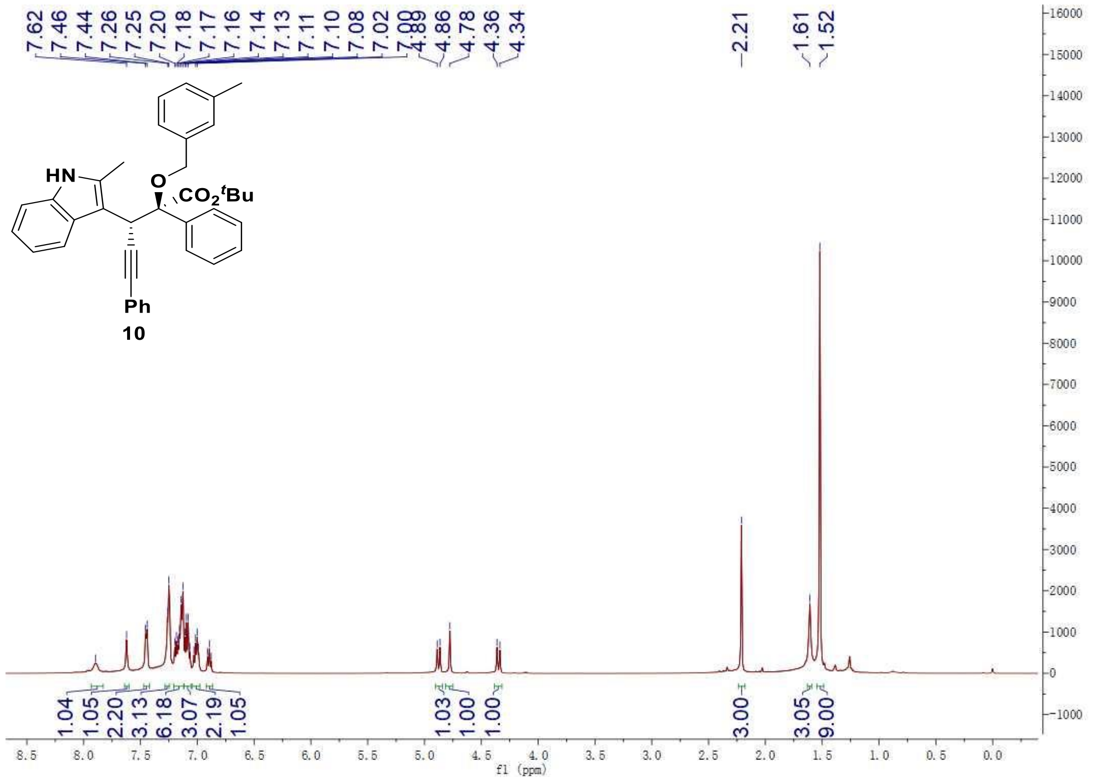

Supplementary Figure 282. ^{1}H NMR (500 MHz, $CDCl_3$) spectrum of **10**.

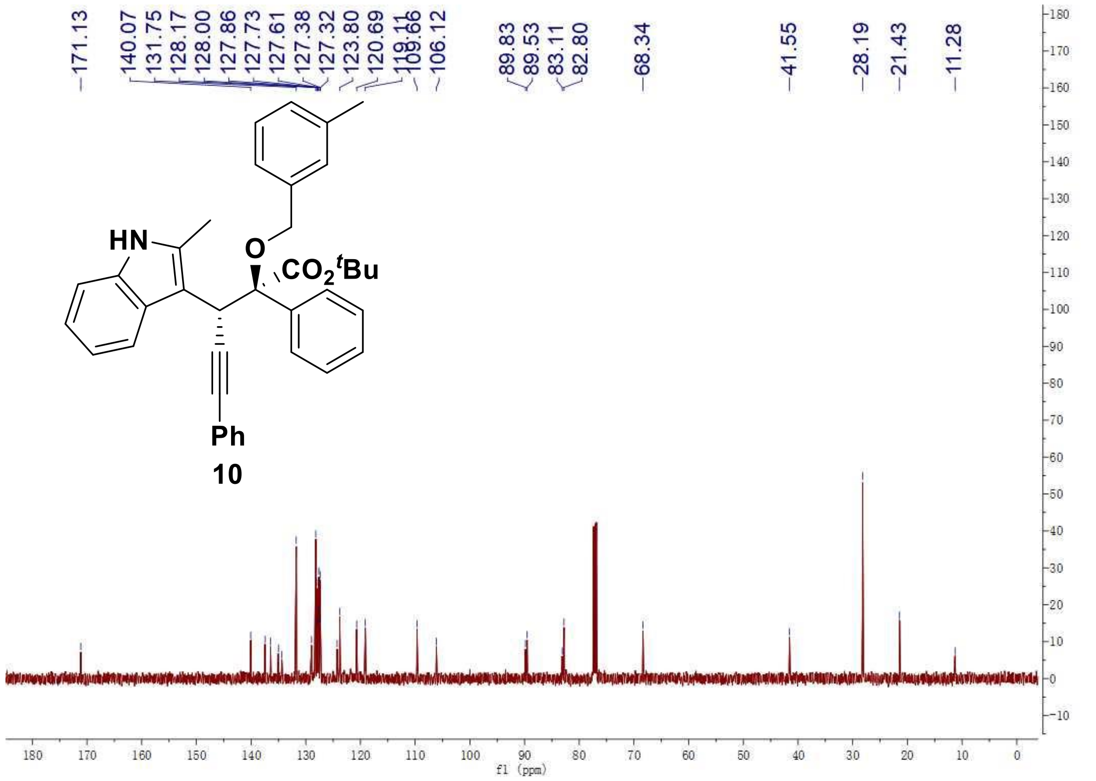

Supplementary Figure 283. ^{13}C NMR (126 MHz, $CDCl_3$) spectrum of **10**.

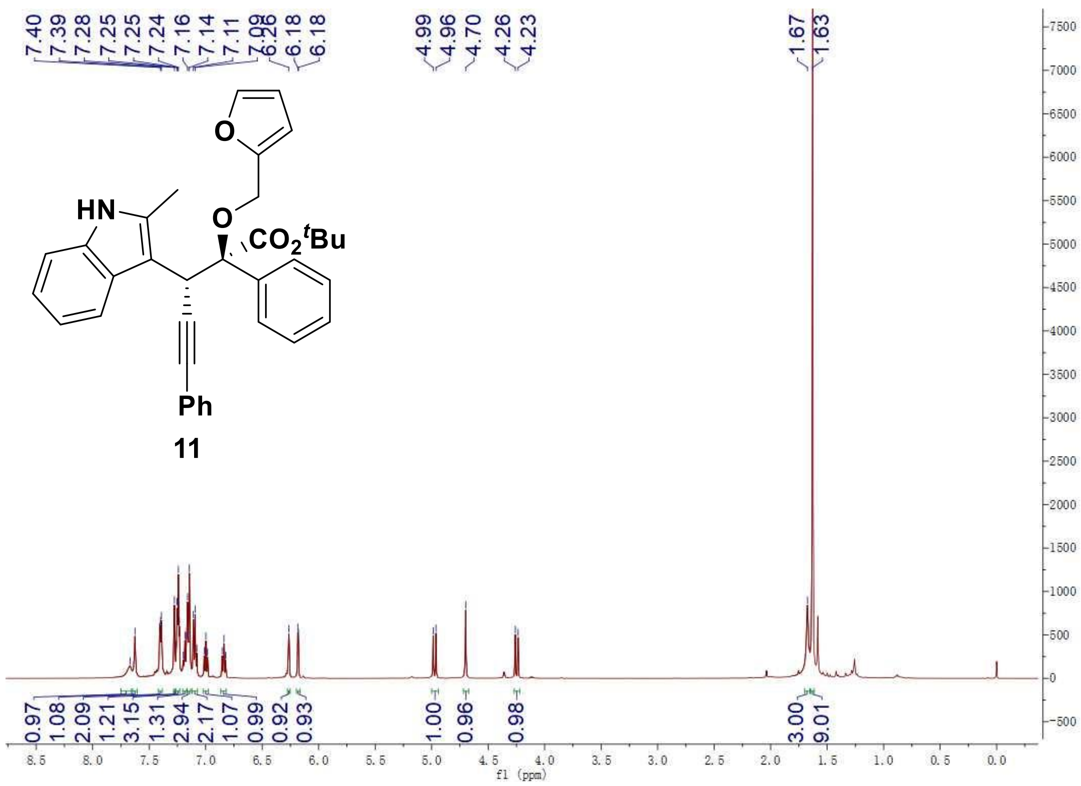

Supplementary Figure 284. ^{1}H NMR (500 MHz, $CDCl_3$) spectrum of **11**.

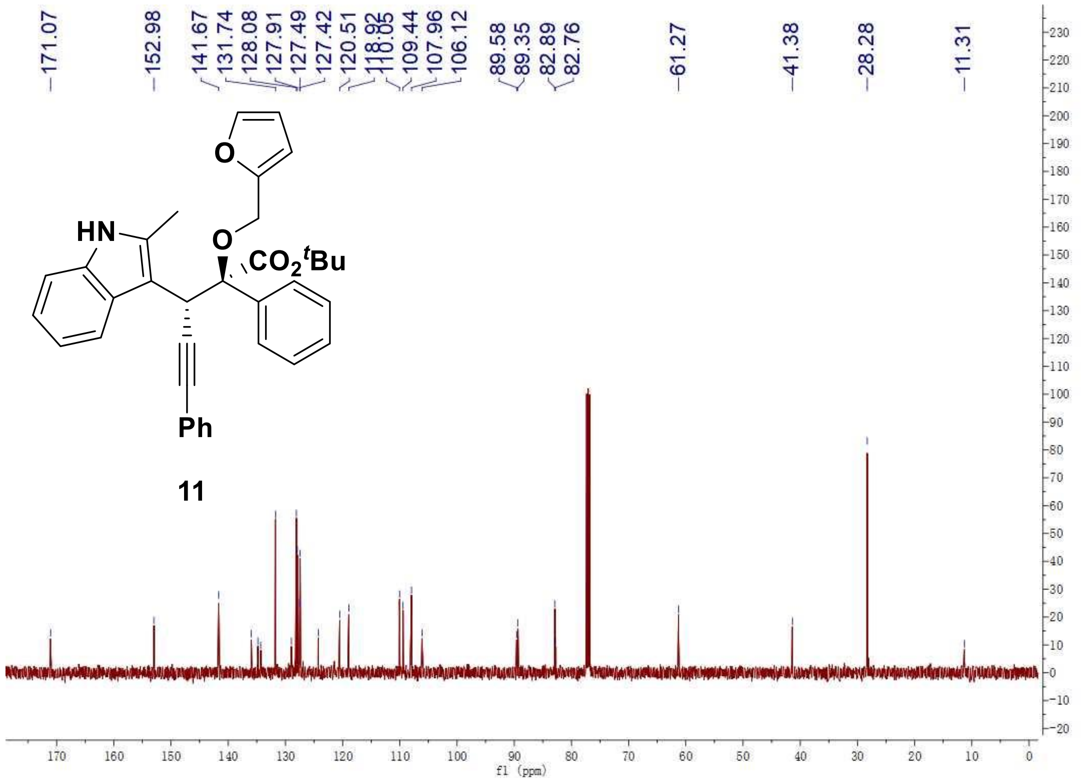

Supplementary Figure 285. ^{13}C NMR (126 MHz, $CDCl_3$) spectrum of **11**.

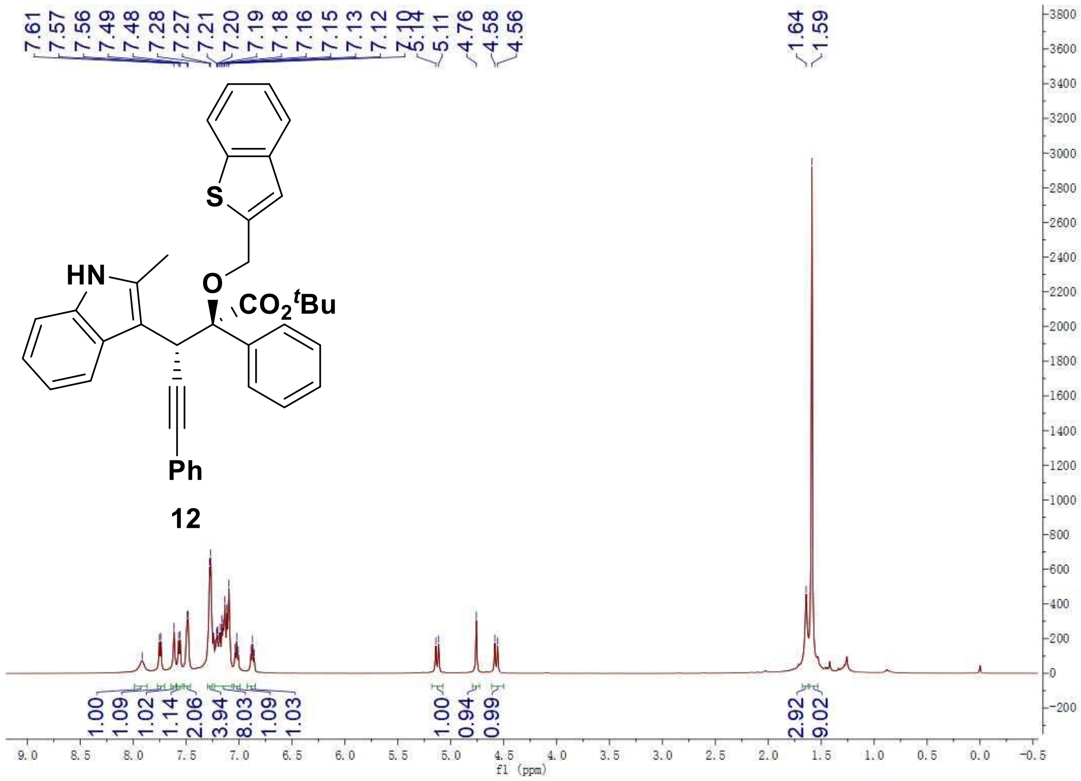

Supplementary Figure 286. ^{1}H NMR (500 MHz, $CDCl_3$) spectrum of **12**.

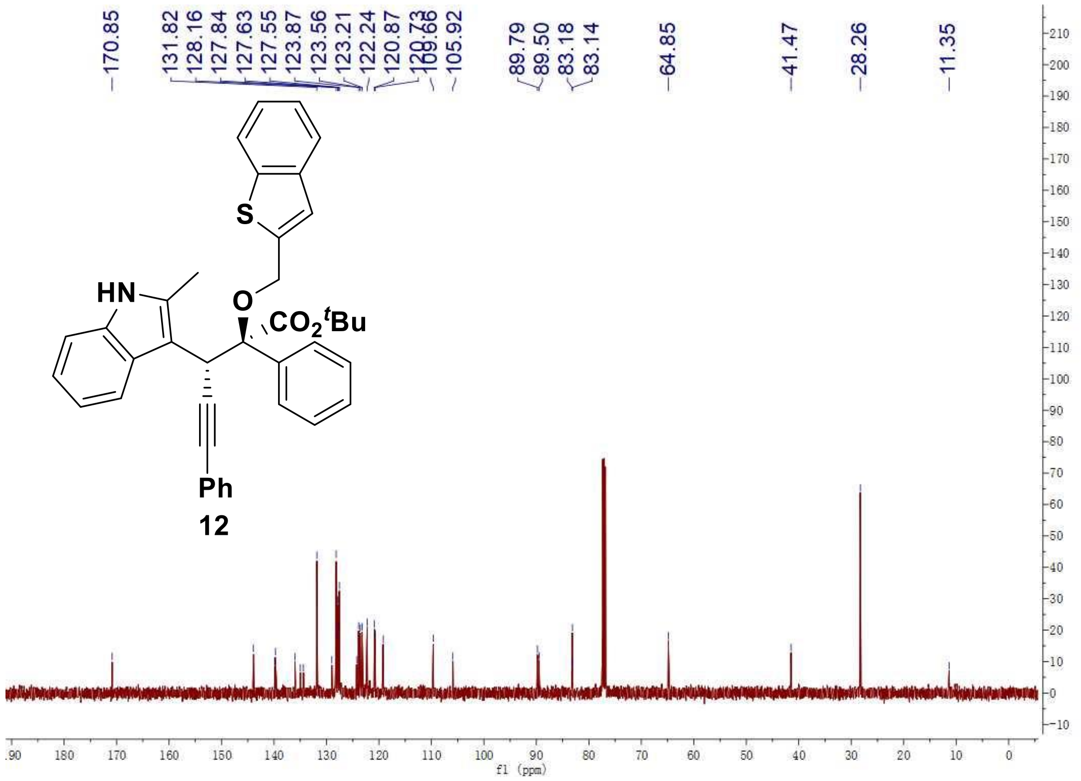

Supplementary Figure 287. ^{13}C NMR (126 MHz, $CDCl_3$) spectrum of **12**.

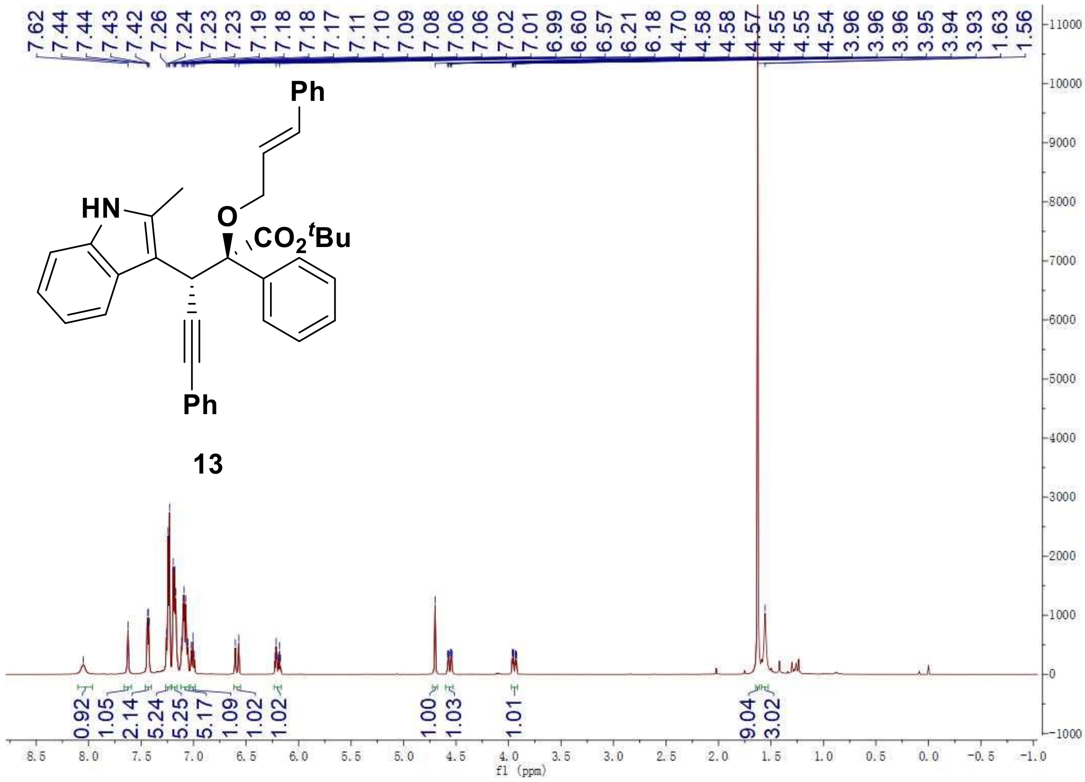

Supplementary Figure 288. ^{1}H NMR (500 MHz, $CDCl_3$) spectrum of **13**.

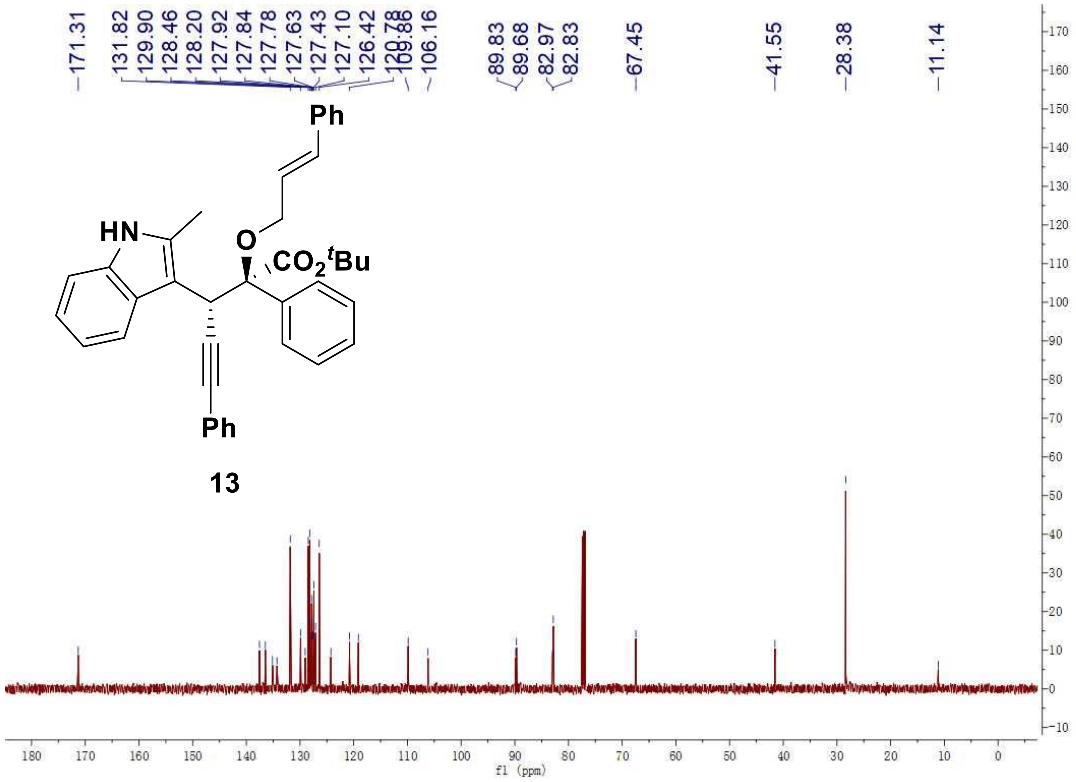

Supplementary Figure 289. ^{13}C NMR (126 MHz, $CDCl_3$) spectrum of **13**.

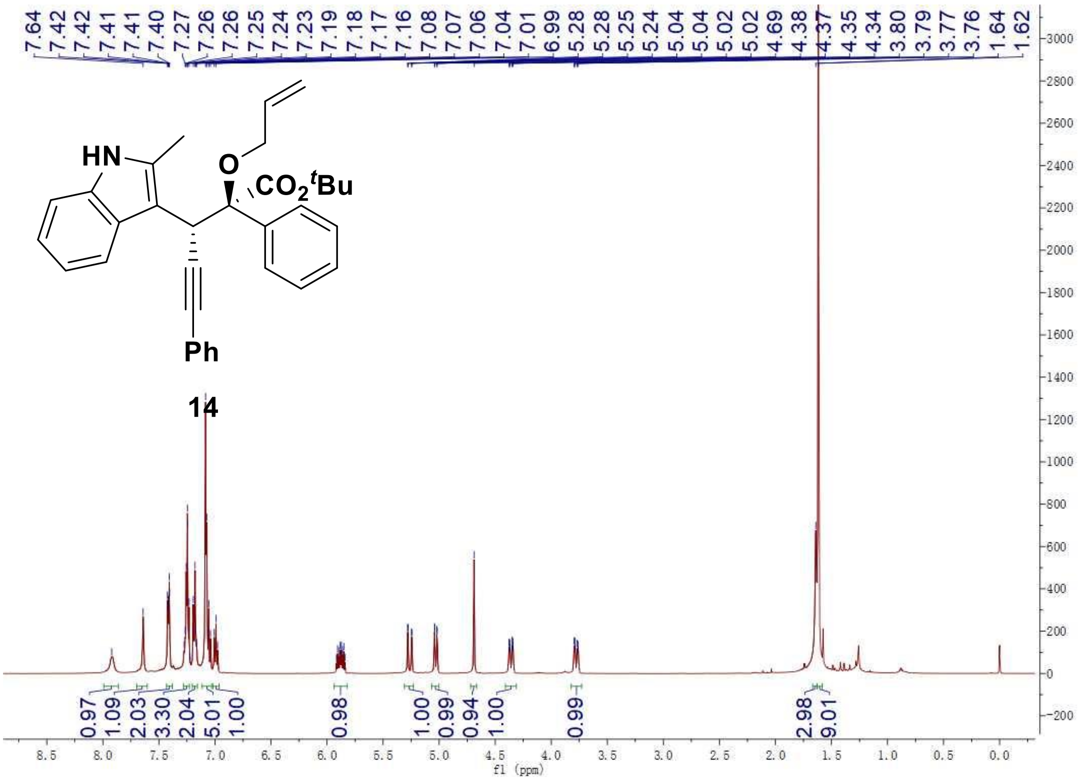

Supplementary Figure 290. ^{1}H NMR (500 MHz, $CDCl_3$) spectrum of **14**.

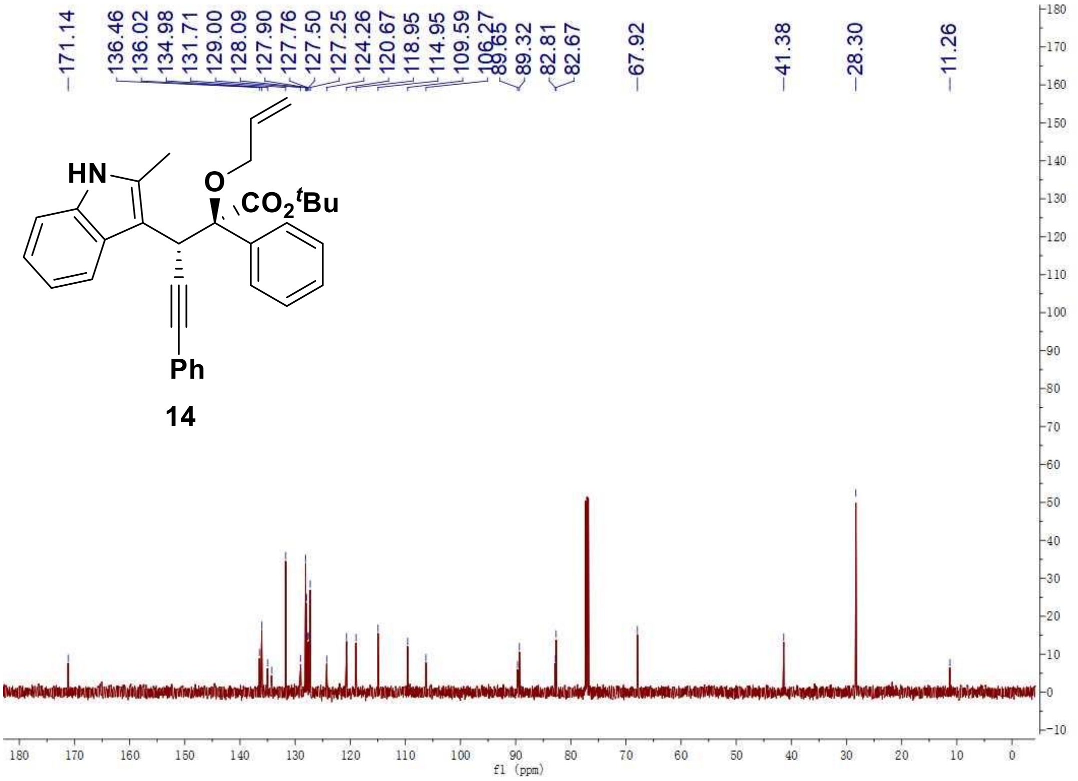

Supplementary Figure 291. ^{13}C NMR (126 MHz, $CDCl_3$) spectrum of **14**.

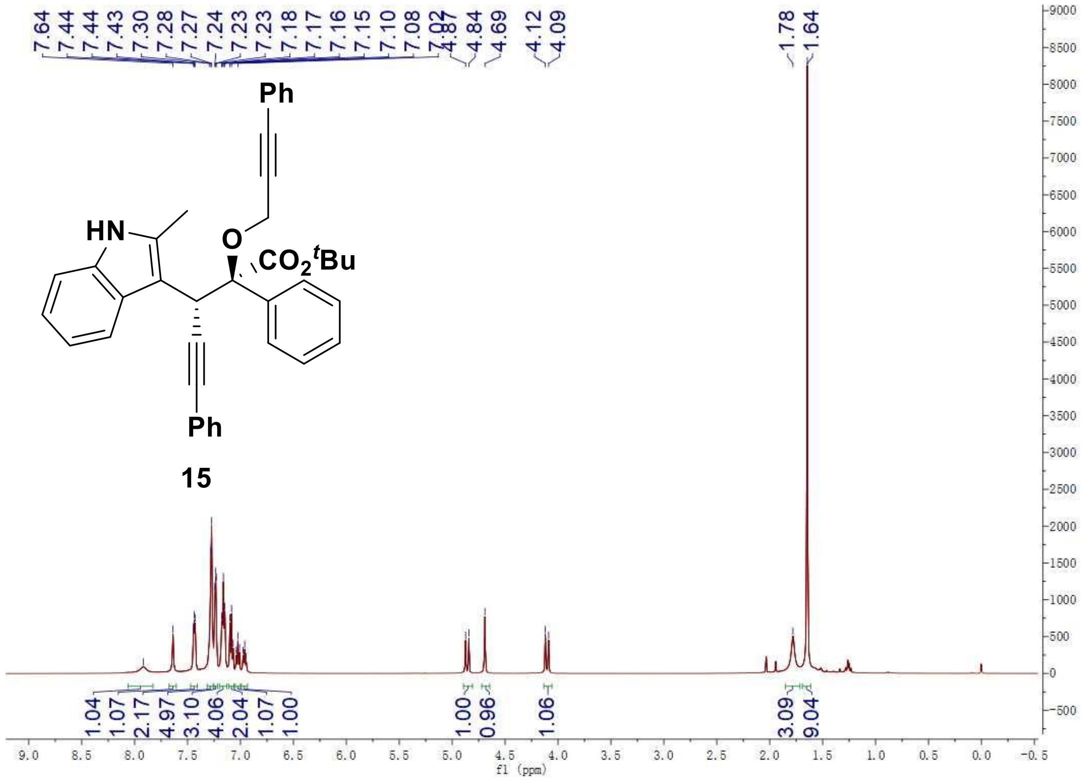

Supplementary Figure 292. ^{1}H NMR (500 MHz, $CDCl_3$) spectrum of **15**.

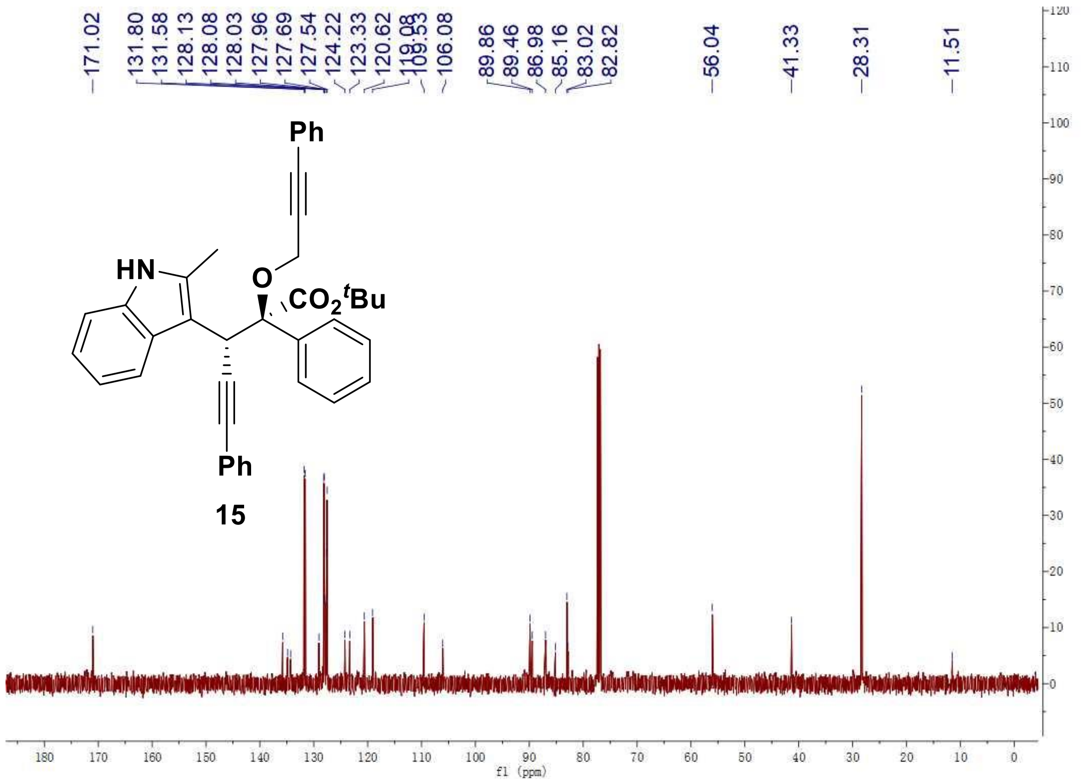

Supplementary Figure 293. ^{13}C NMR (126 MHz, $CDCl_3$) spectrum of **15**.

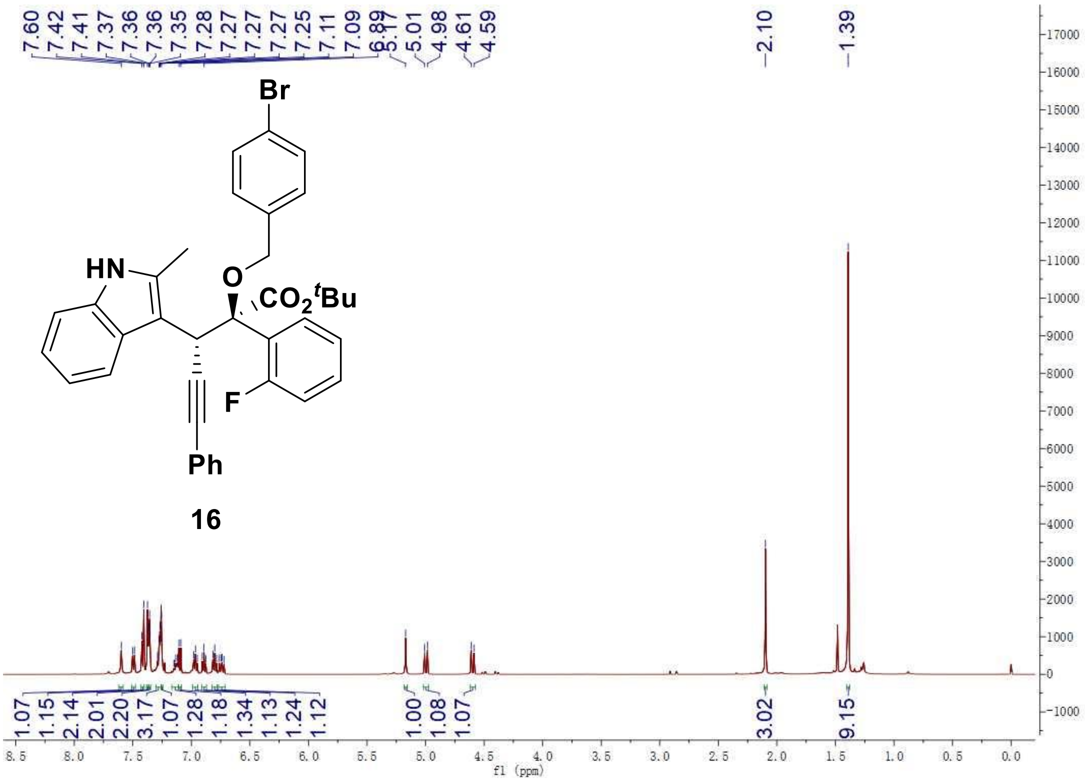

Supplementary Figure 294. ^{1}H NMR (500 MHz, $CDCl_3$) spectrum of **16**.

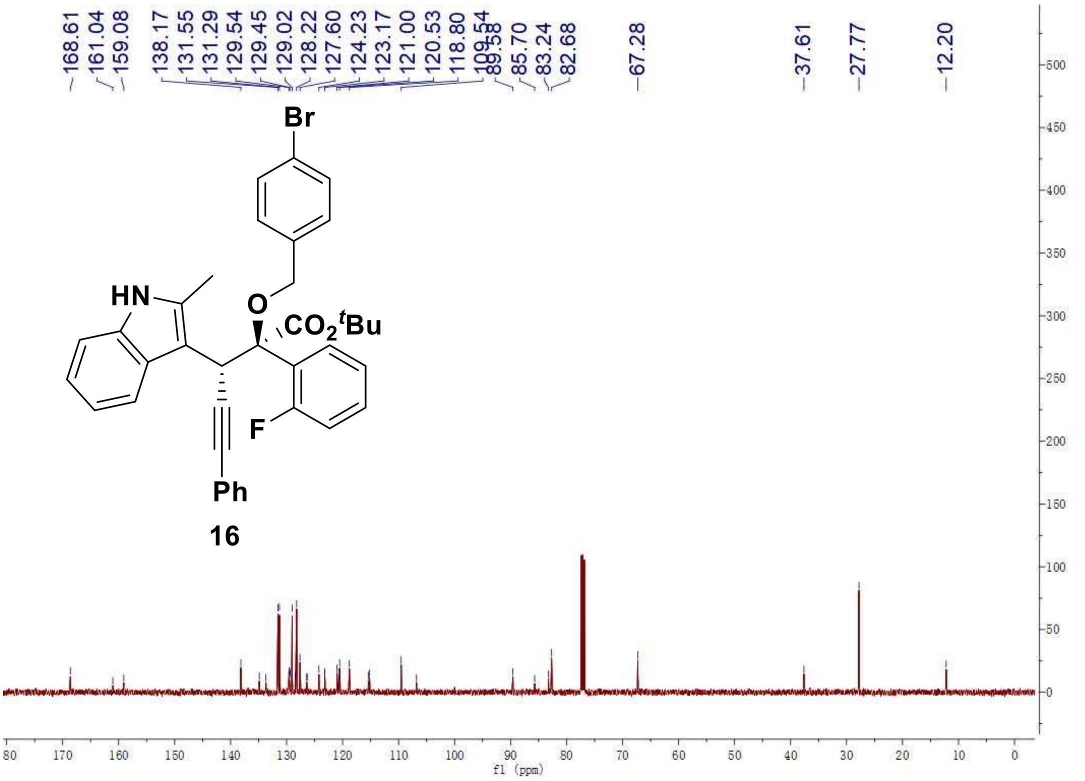

Supplementary Figure 295. ^{13}C NMR (126 MHz, $CDCl_3$) spectrum of **16**.

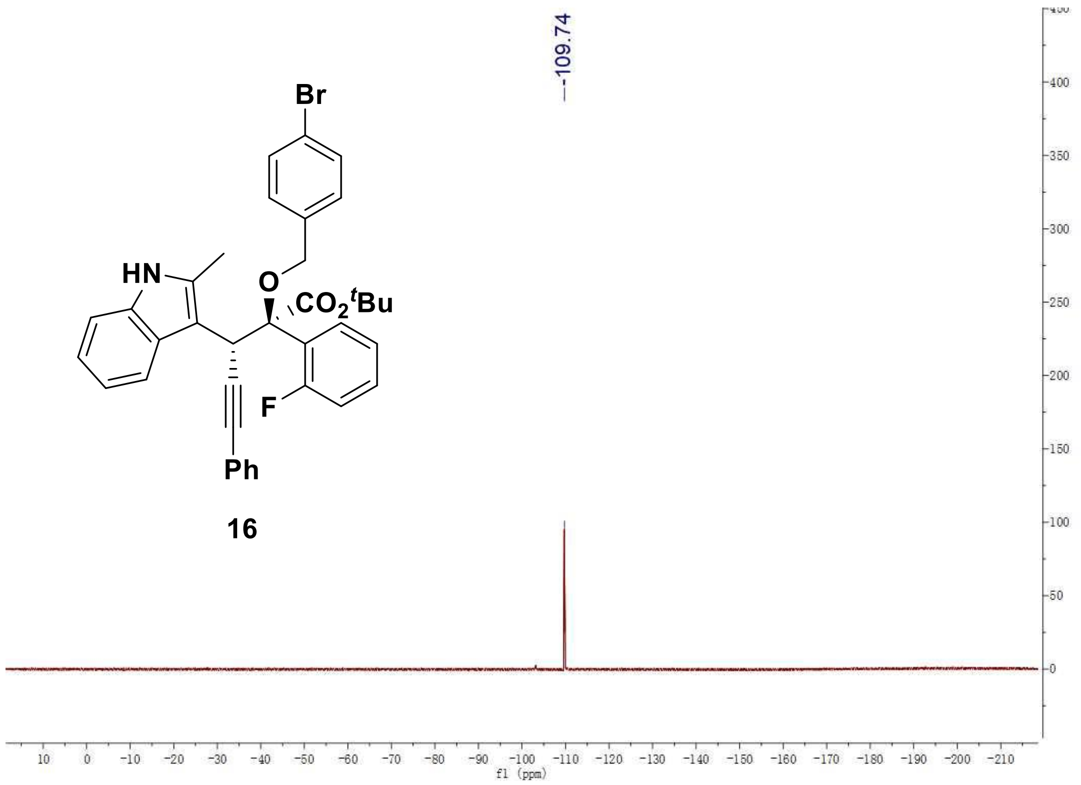

Supplementary Figure 296. ^{19}F NMR (471 MHz, $CDCl_3$) spectrum of **16**.

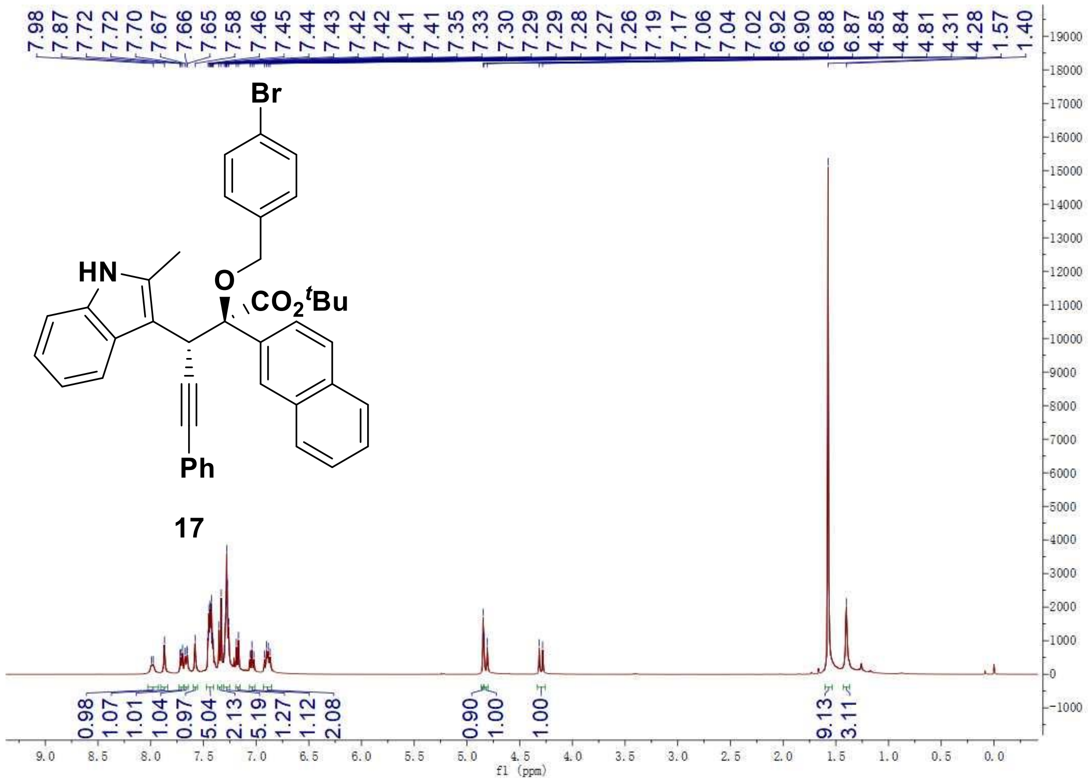

Supplementary Figure 297. ^{1}H NMR (400 MHz, $CDCl_3$) spectrum of **17**.

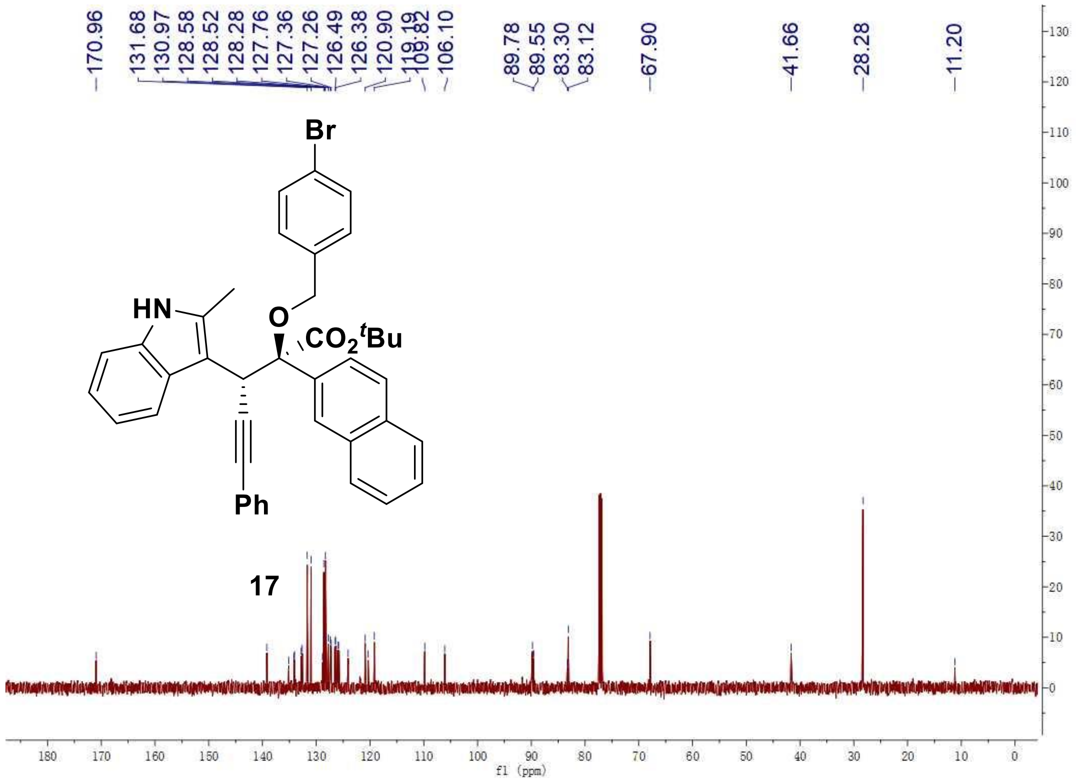

Supplementary Figure 298. ^{13}C NMR (101 MHz, $CDCl_3$) spectrum of **17**.

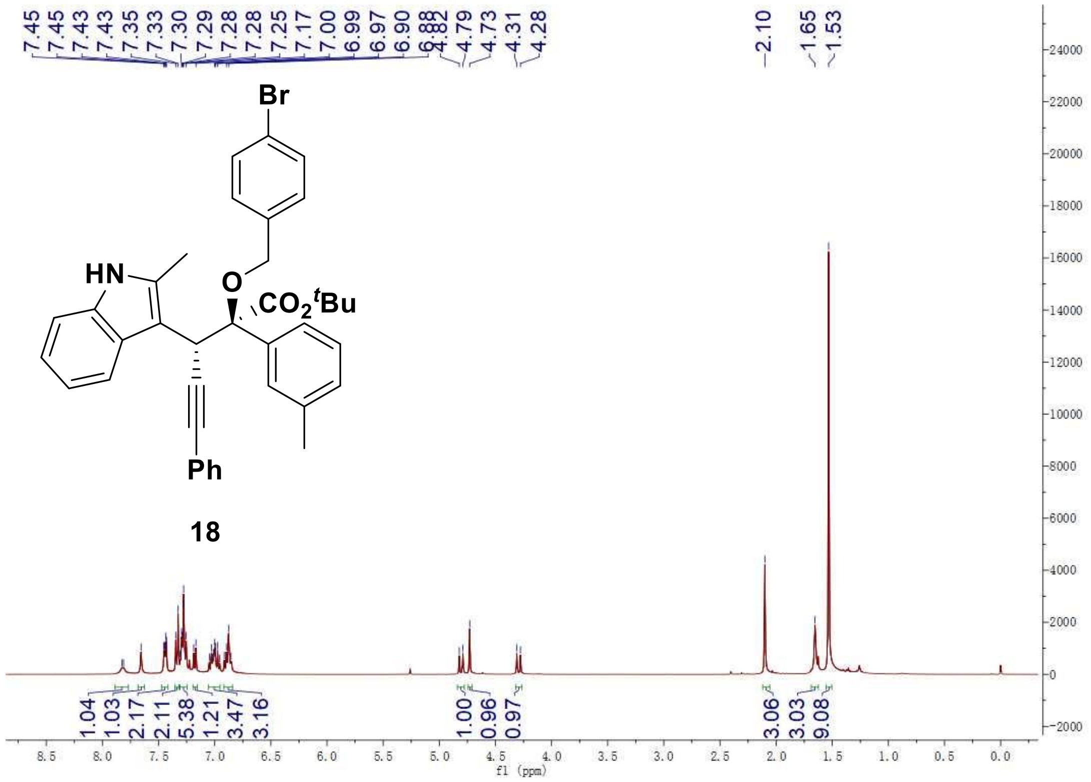

Supplementary Figure 299. ^{1}H NMR (400 MHz, $CDCl_3$) spectrum of **18**.

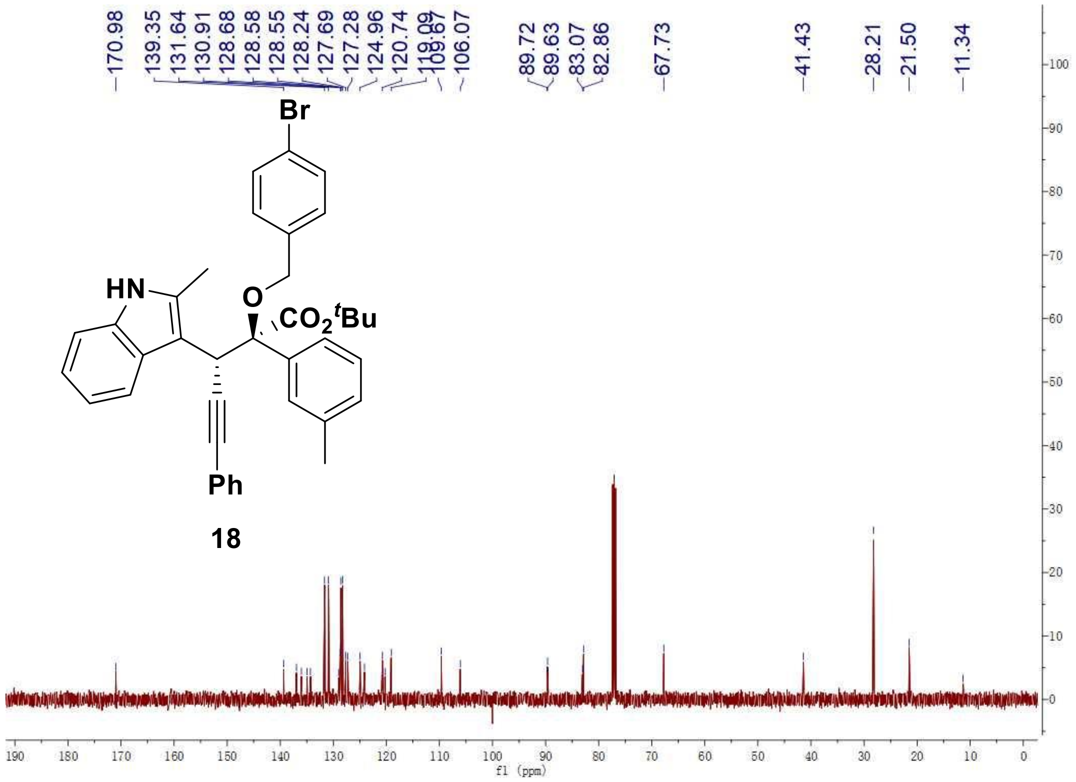

Supplementary Figure 300. ^{13}C NMR (101 MHz, $CDCl_3$) spectrum of **18**.

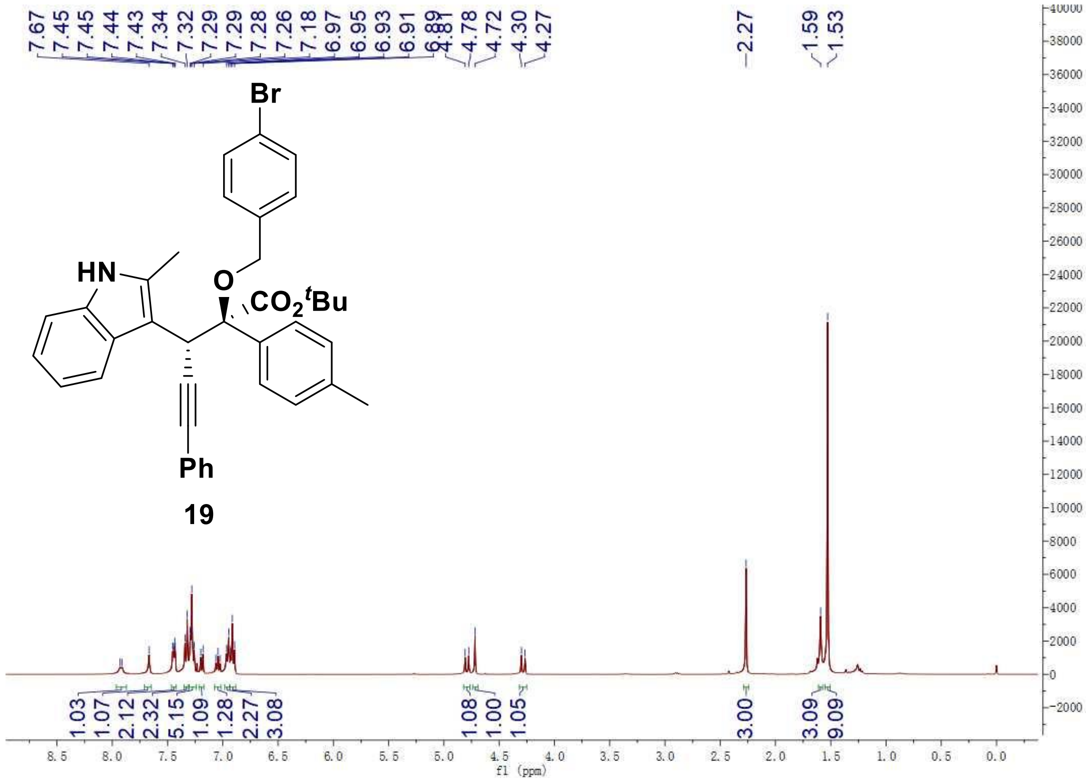

Supplementary Figure 301. ^{1}H NMR (400 MHz, $CDCl_3$) spectrum of **19**.

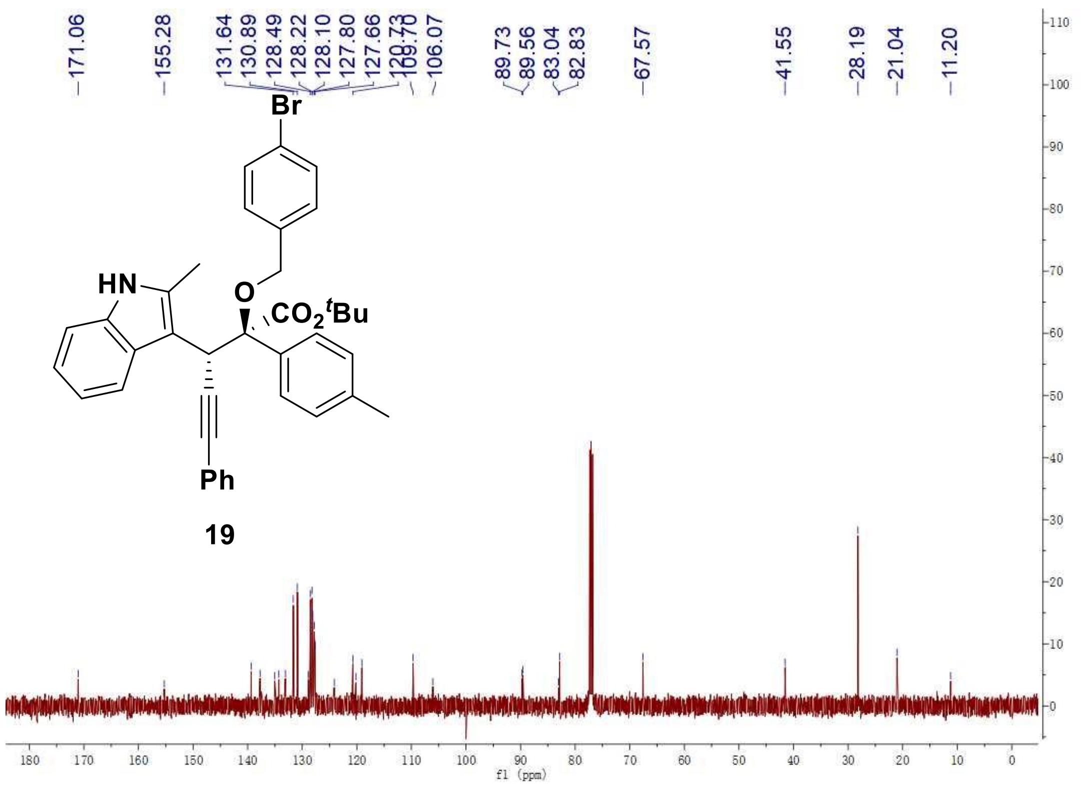

Supplementary Figure 302. ^{13}C NMR (101 MHz, $CDCl_3$) spectrum of **19**.

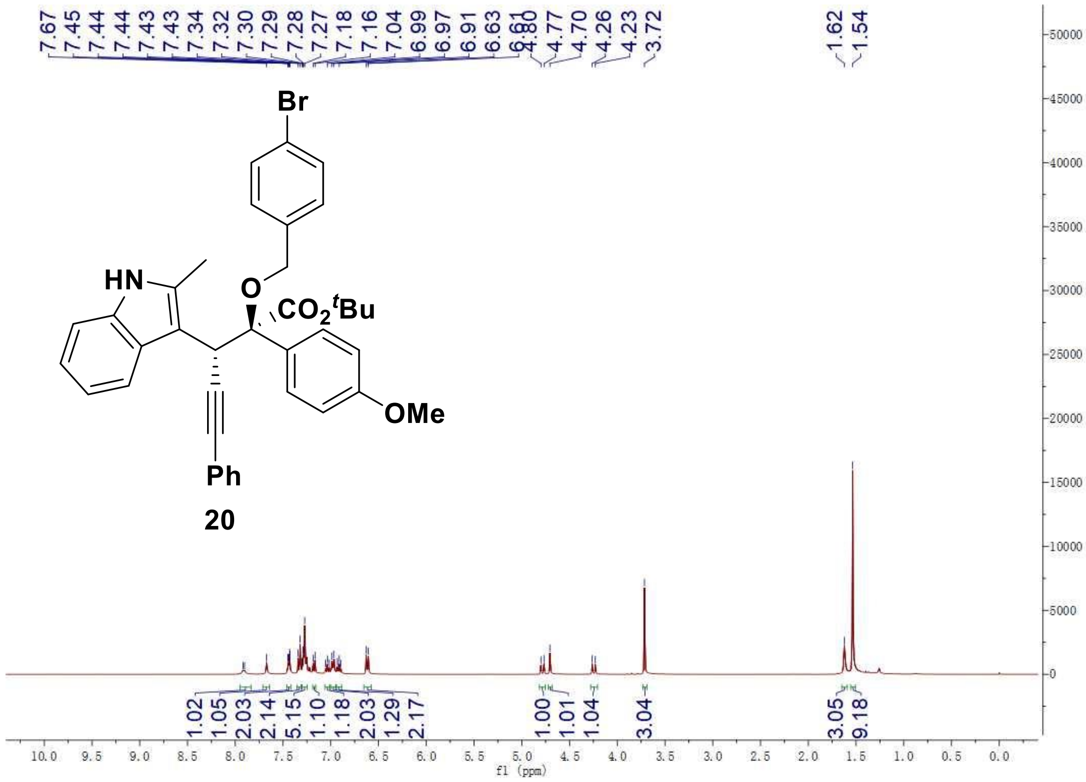

Supplementary Figure 303. ^{1}H NMR (400 MHz, $CDCl_3$) spectrum of **20**.

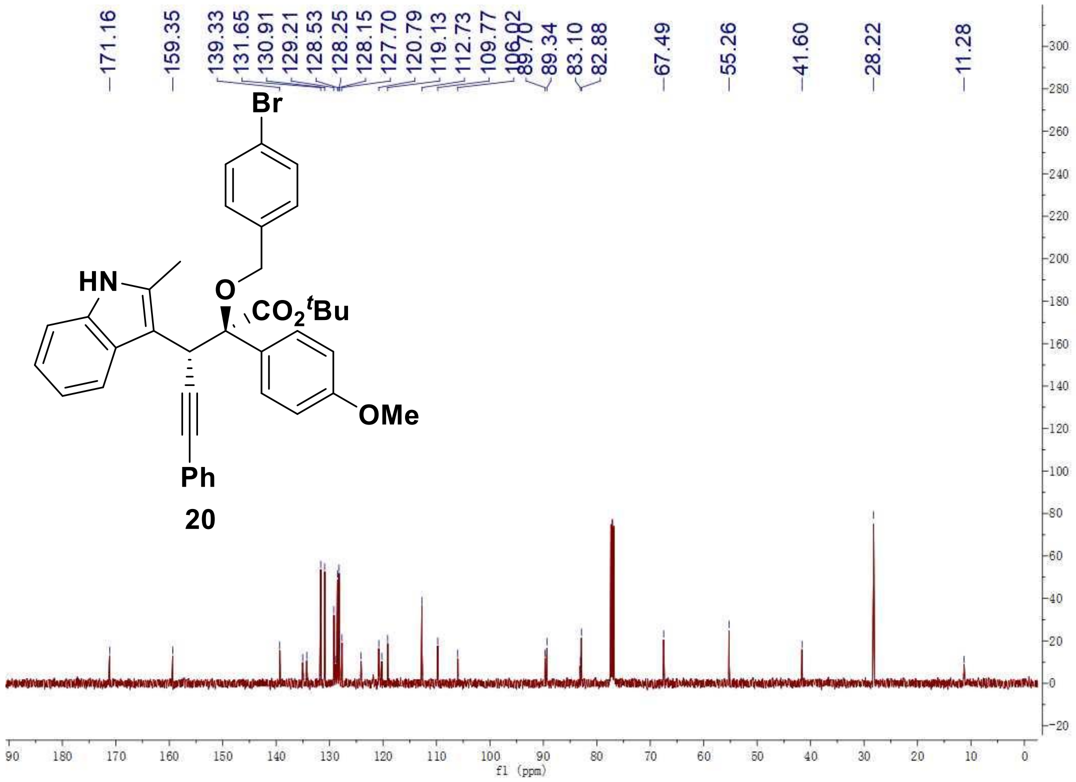

Supplementary Figure 304. ^{13}C NMR (101 MHz, $CDCl_3$) spectrum of **20**.

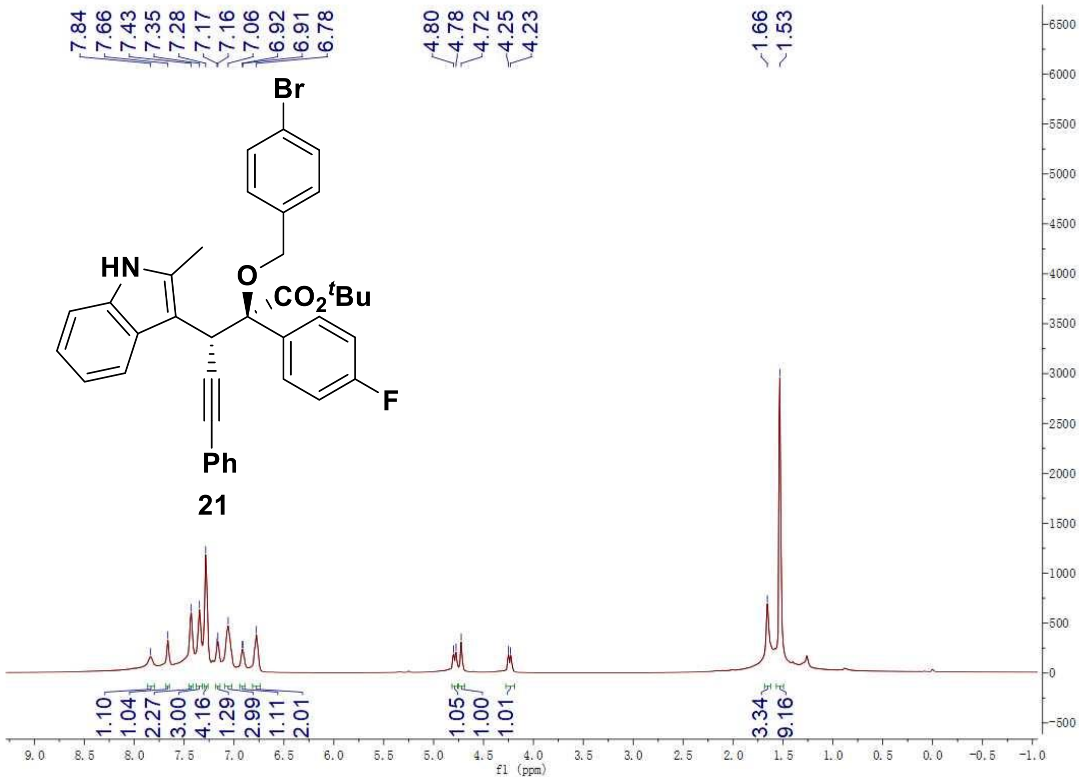

Supplementary Figure 305. ^{1}H NMR (500 MHz, $CDCl_3$) spectrum of **21**.

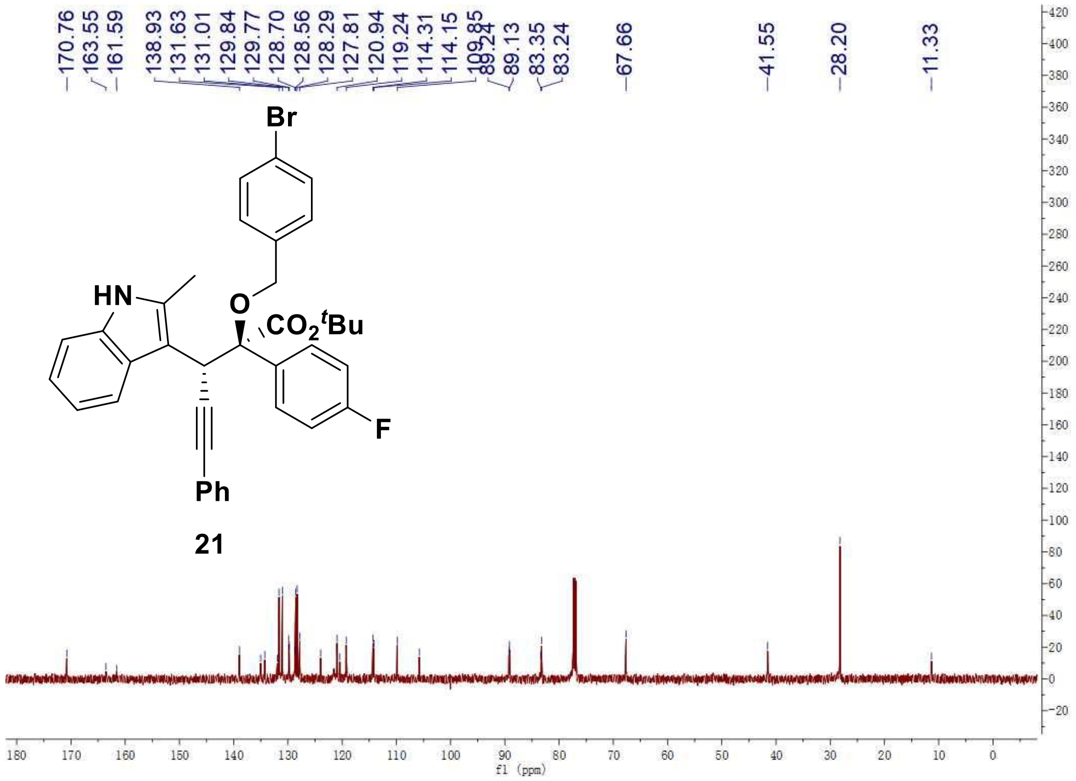

Supplementary Figure 306. ^{13}C NMR (126 MHz, $CDCl_3$) spectrum of **21**.

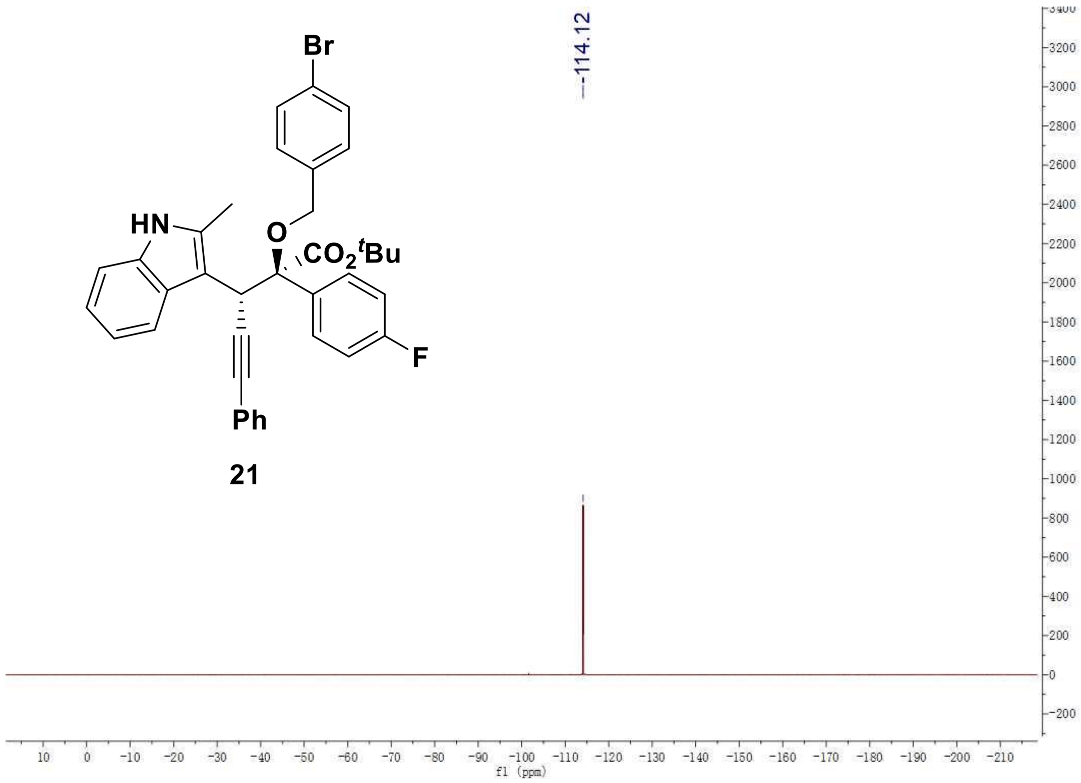

Supplementary Figure 307. ^{19}F NMR (471 MHz, $CDCl_3$) spectrum of **21**.

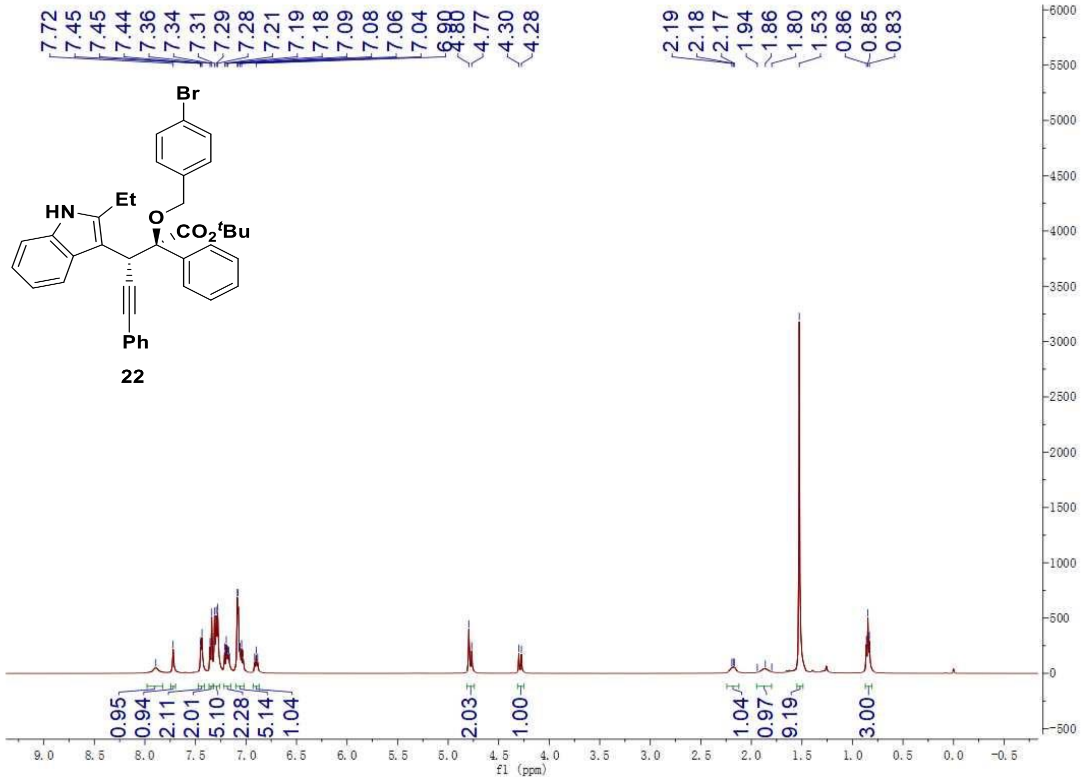

Supplementary Figure 308. ^{1}H NMR (500 MHz, $CDCl_3$) spectrum of **22**.

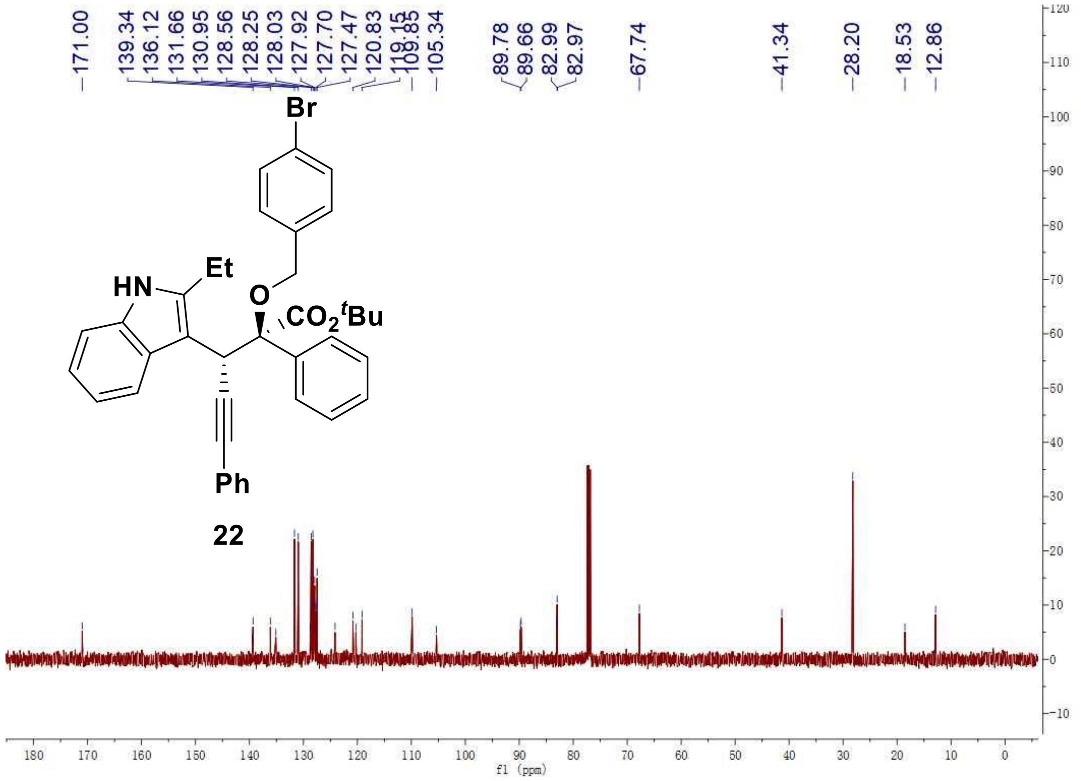

Supplementary Figure 309. ^{13}C NMR (126 MHz, $CDCl_3$) spectrum of **22**.

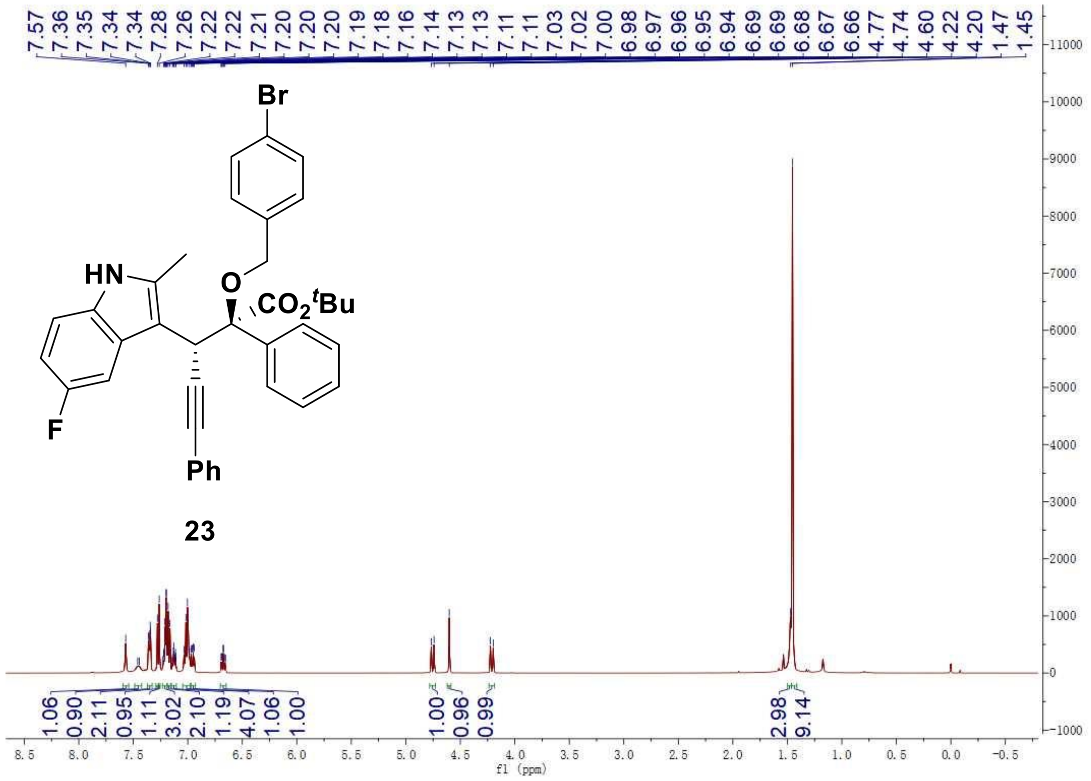

Supplementary Figure 310. ^{1}H NMR (500 MHz, $CDCl_3$) spectrum of **23**.

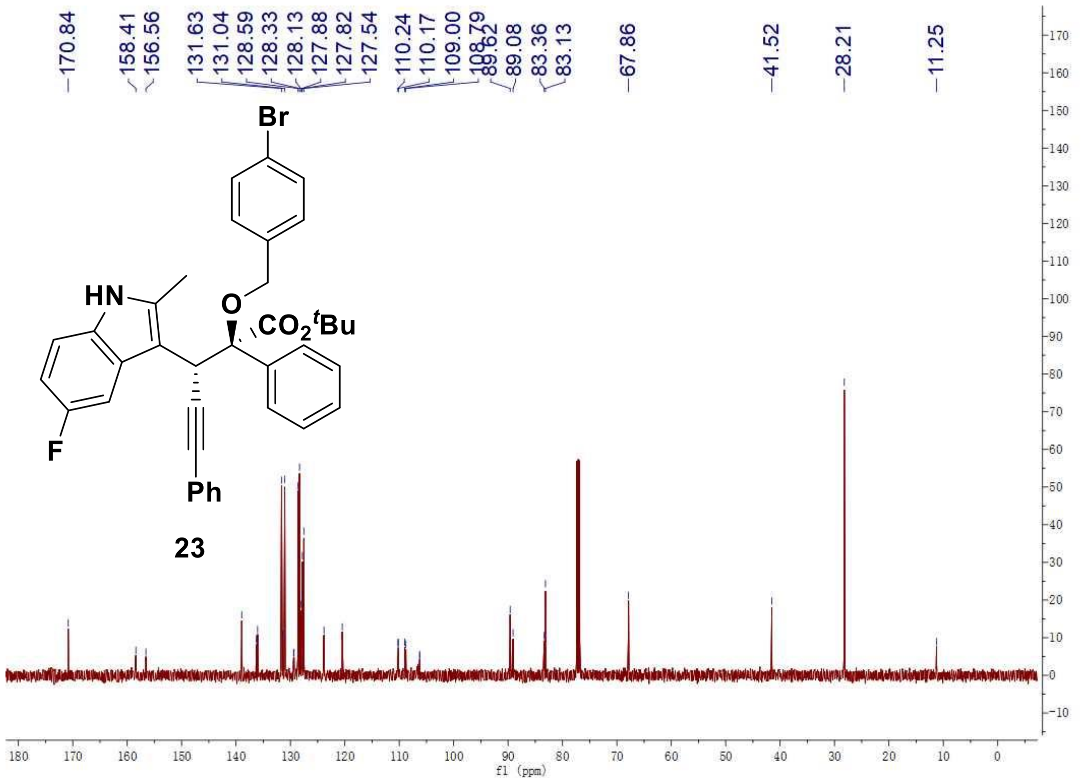

Supplementary Figure 311. ^{13}C NMR (126 MHz, $CDCl_3$) spectrum of **23**.

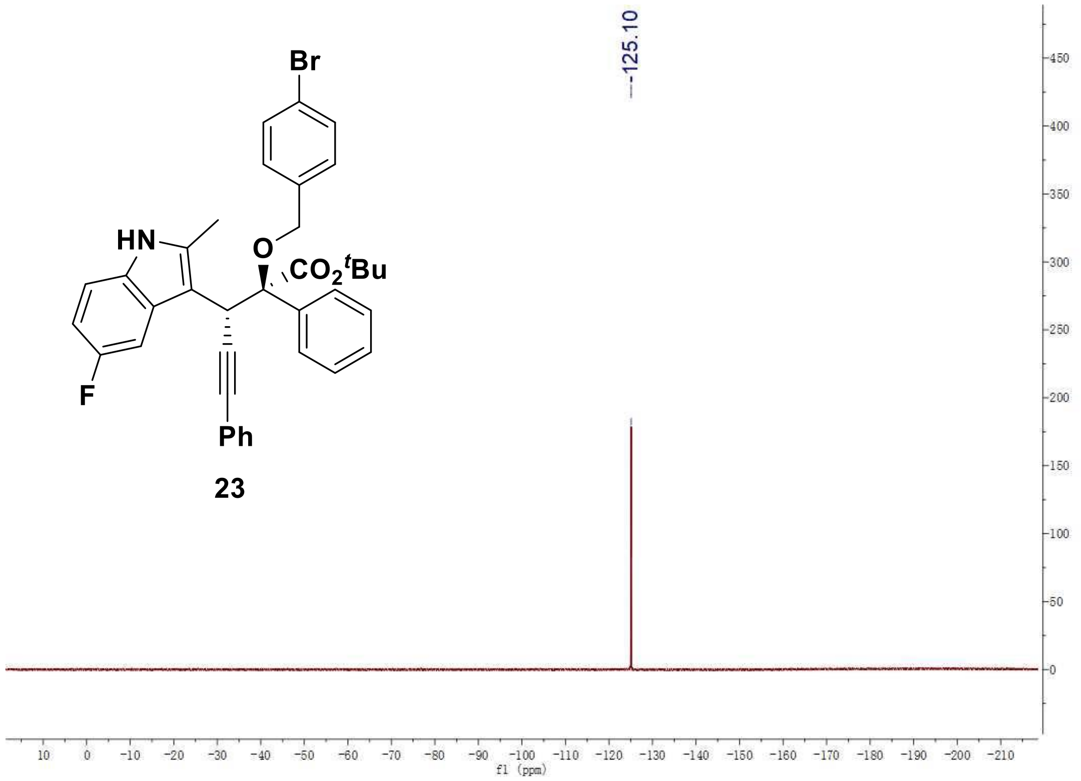

Supplementary Figure 312. ^{19}F NMR (471 MHz, $CDCl_3$) spectrum of **23**.

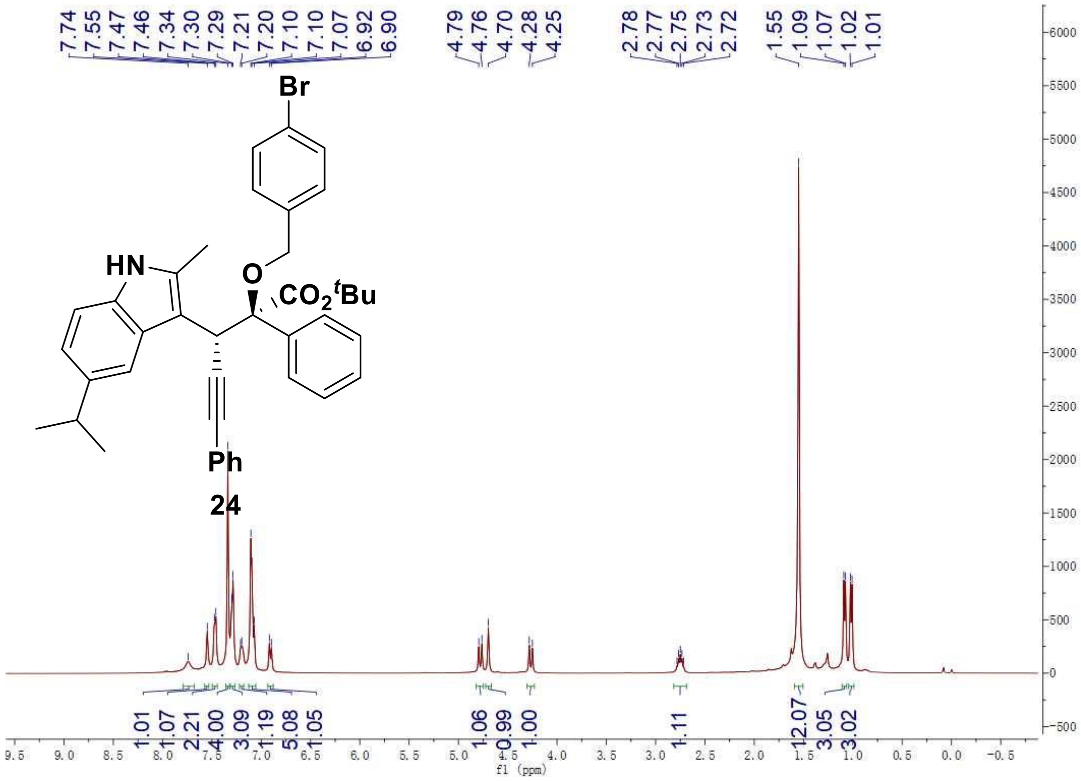

Supplementary Figure 313. ^{1}H NMR (400 MHz, $CDCl_3$) spectrum of **24**.

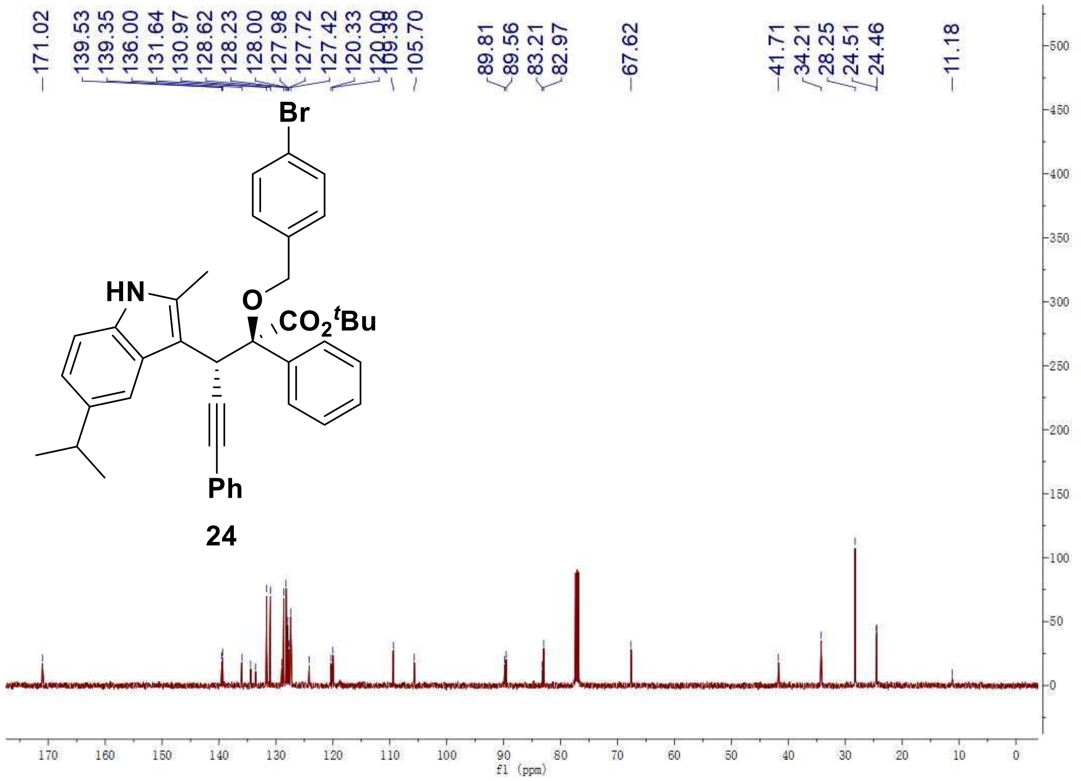

Supplementary Figure 314. ^{13}C NMR (101 MHz, $CDCl_3$) spectrum of **24**.

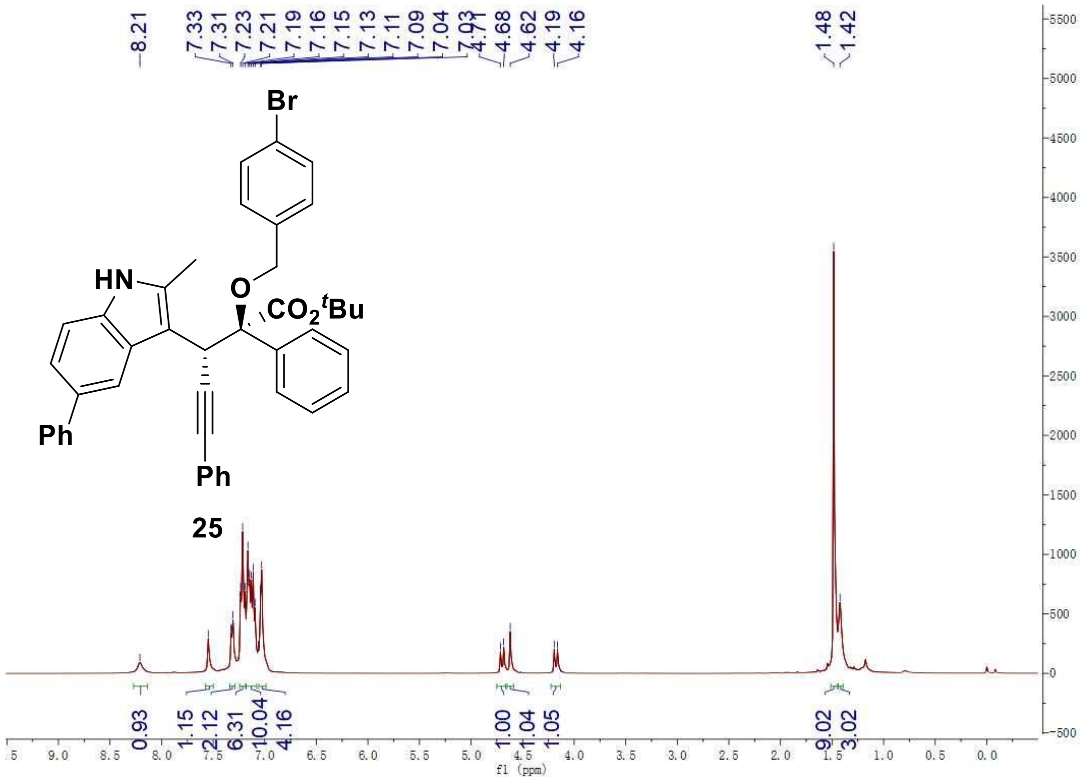

Supplementary Figure 315. ^{1}H NMR (400 MHz, $CDCl_3$) spectrum of **25**.

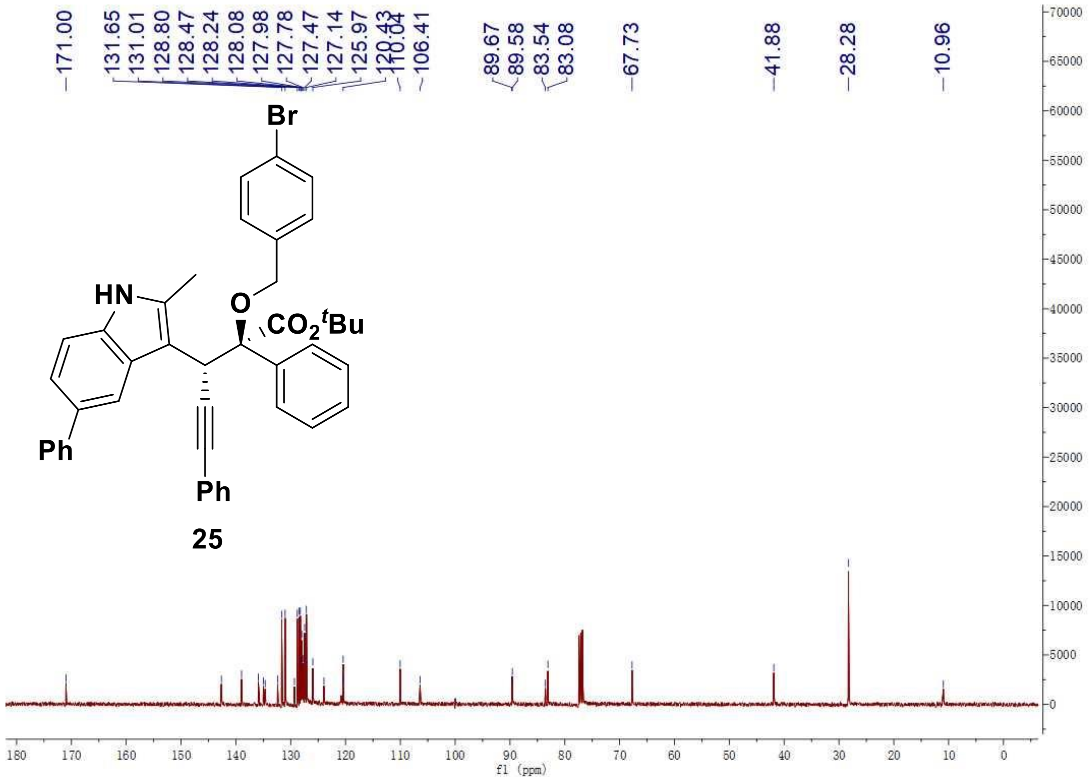

Supplementary Figure 316. ^{13}C NMR (101 MHz, $CDCl_3$) spectrum of **25**.

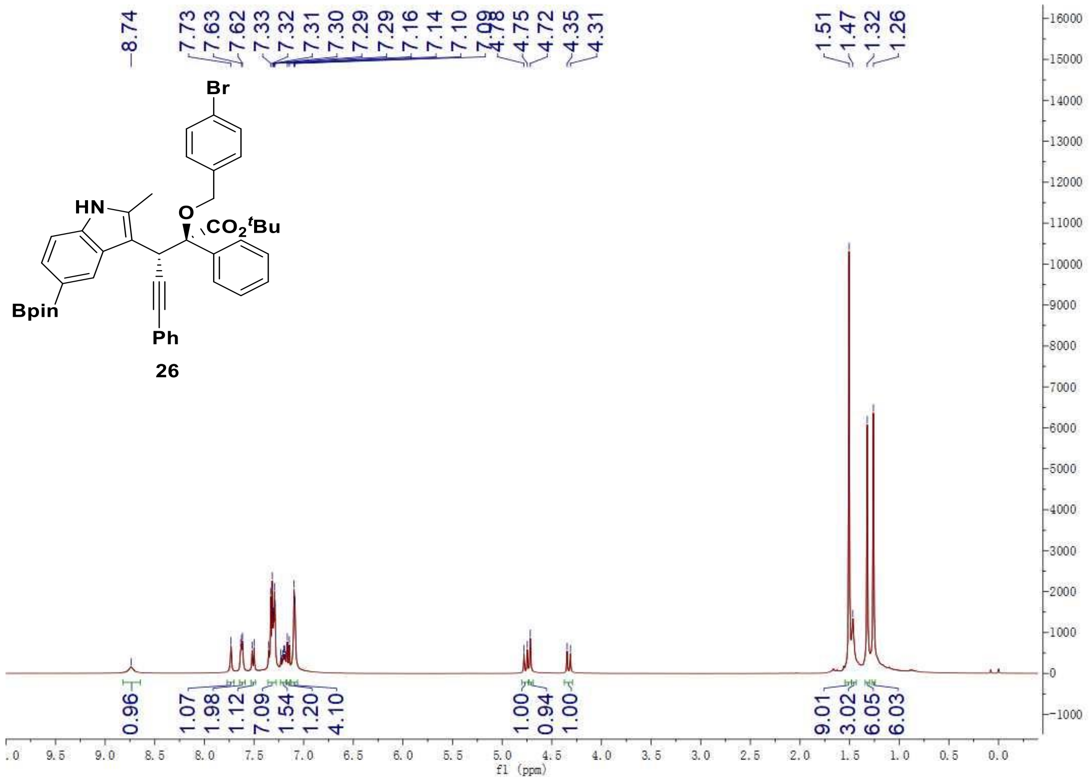

Supplementary Figure 317. ^{1}H NMR (400 MHz, $CDCl_3$) spectrum of **26**.

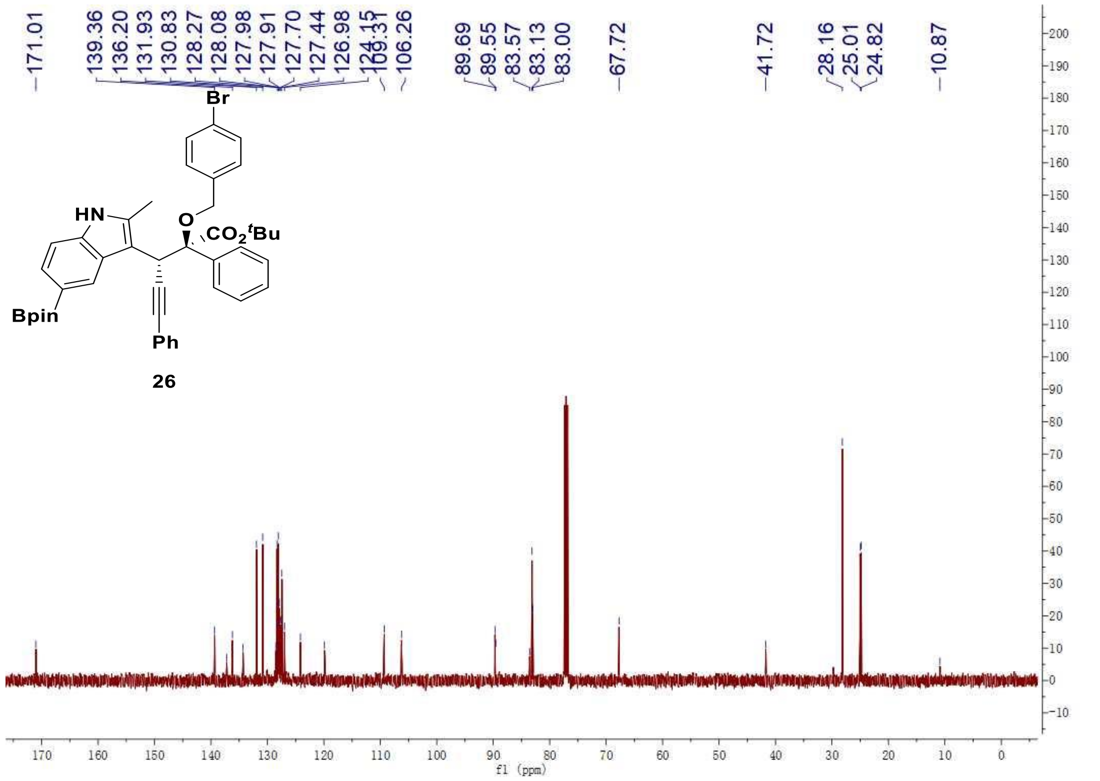

Supplementary Figure 318. ^{13}C NMR (101 MHz, $CDCl_3$) spectrum of **26**.

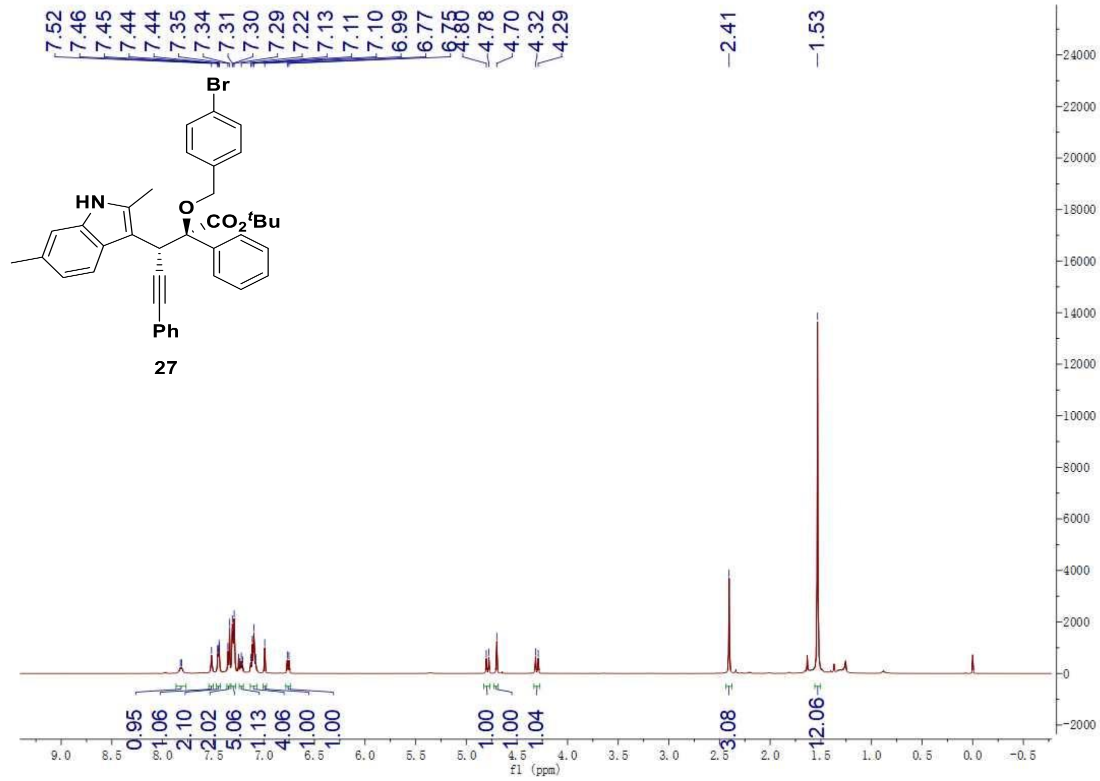

Supplementary Figure 319. ^{1}H NMR (500 MHz, $CDCl_3$) spectrum of **27**.

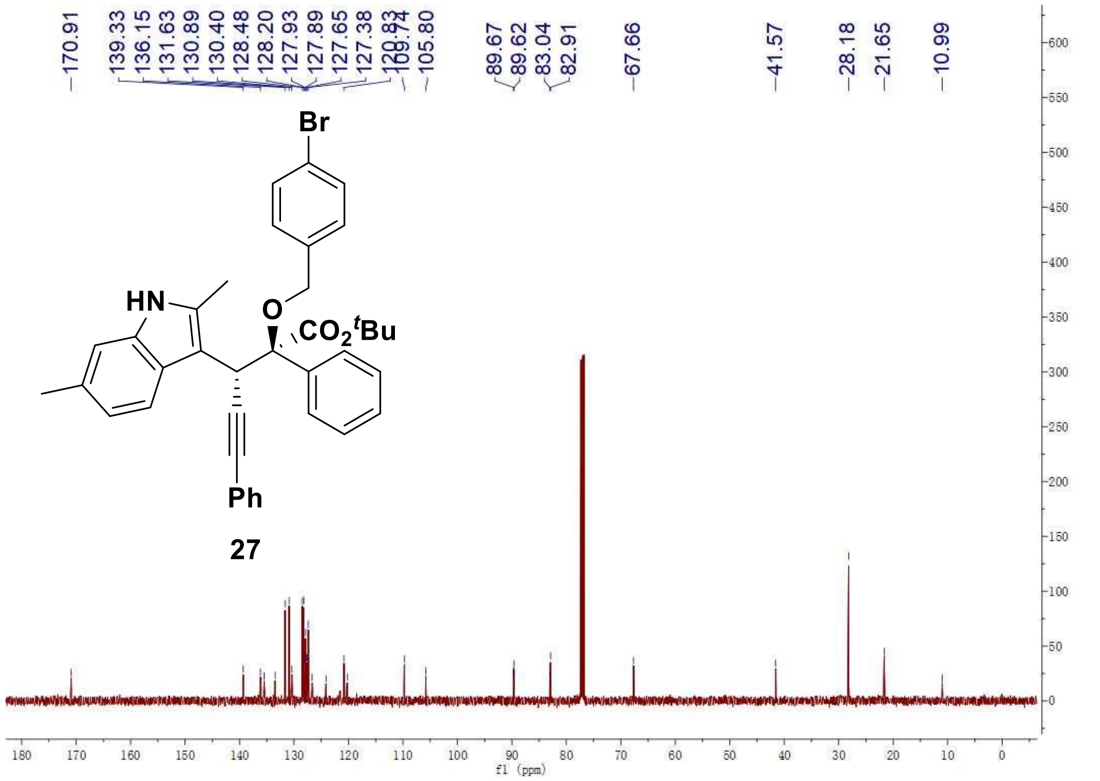

Supplementary Figure 320. ^{13}C NMR (126 MHz, $CDCl_3$) spectrum of **27**.

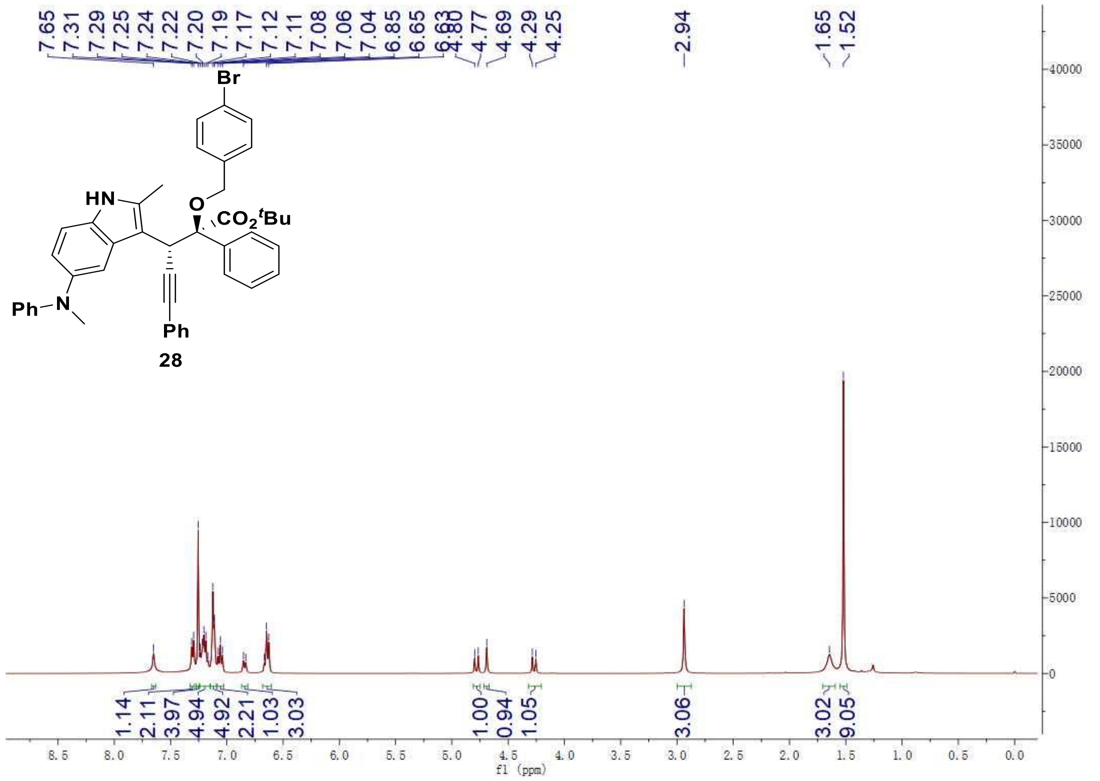

Supplementary Figure 321. ^1H NMR (400 MHz, $CDCl_3$) spectrum of **28**.

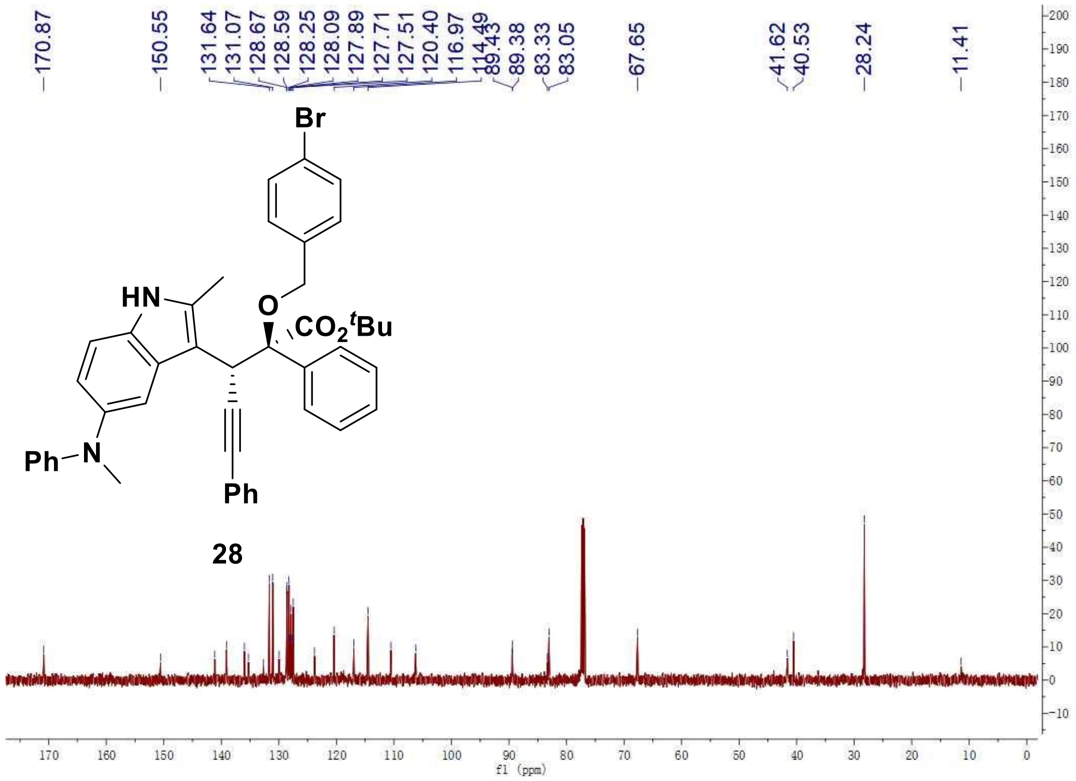

Supplementary Figure 322. ^{13}C NMR (101 MHz, $CDCl_3$) spectrum of **28**.

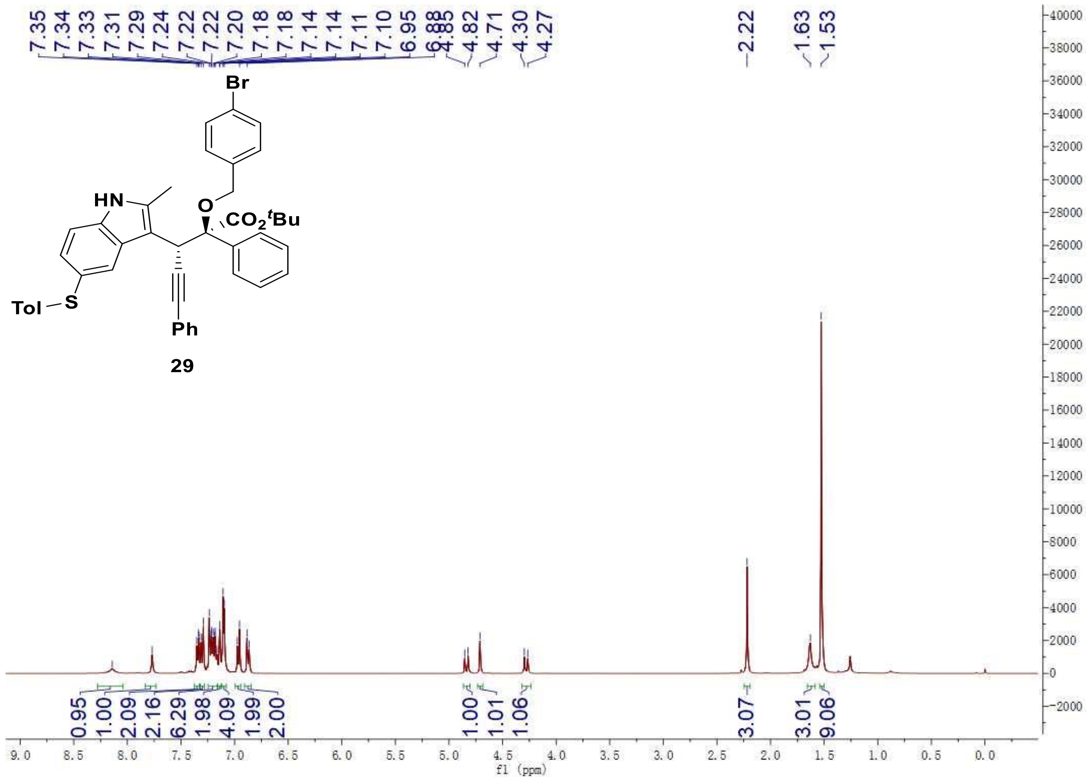

Supplementary Figure 323. ^{1}H NMR (400 MHz, $CDCl_3$) spectrum of **29**.

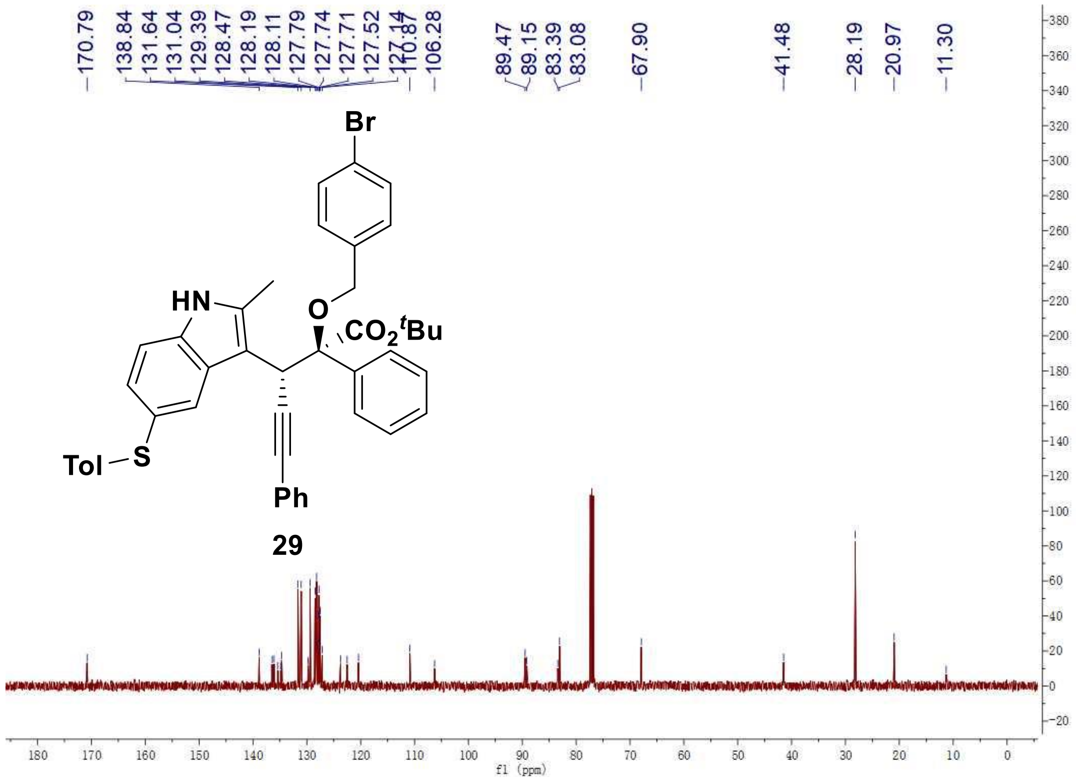

Supplementary Figure 324. ^{13}C NMR (101 MHz, $CDCl_3$) spectrum of **29**.

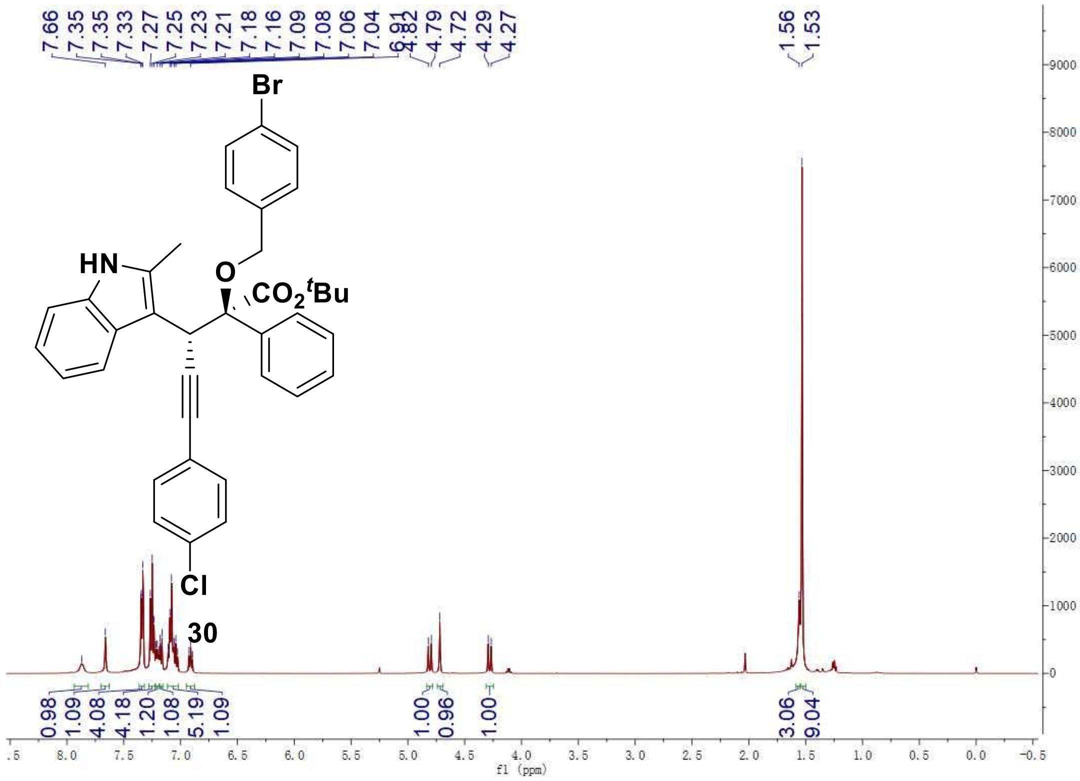

Supplementary Figure 325. ^{1}H NMR (500 MHz, $CDCl_3$) spectrum of **30**.

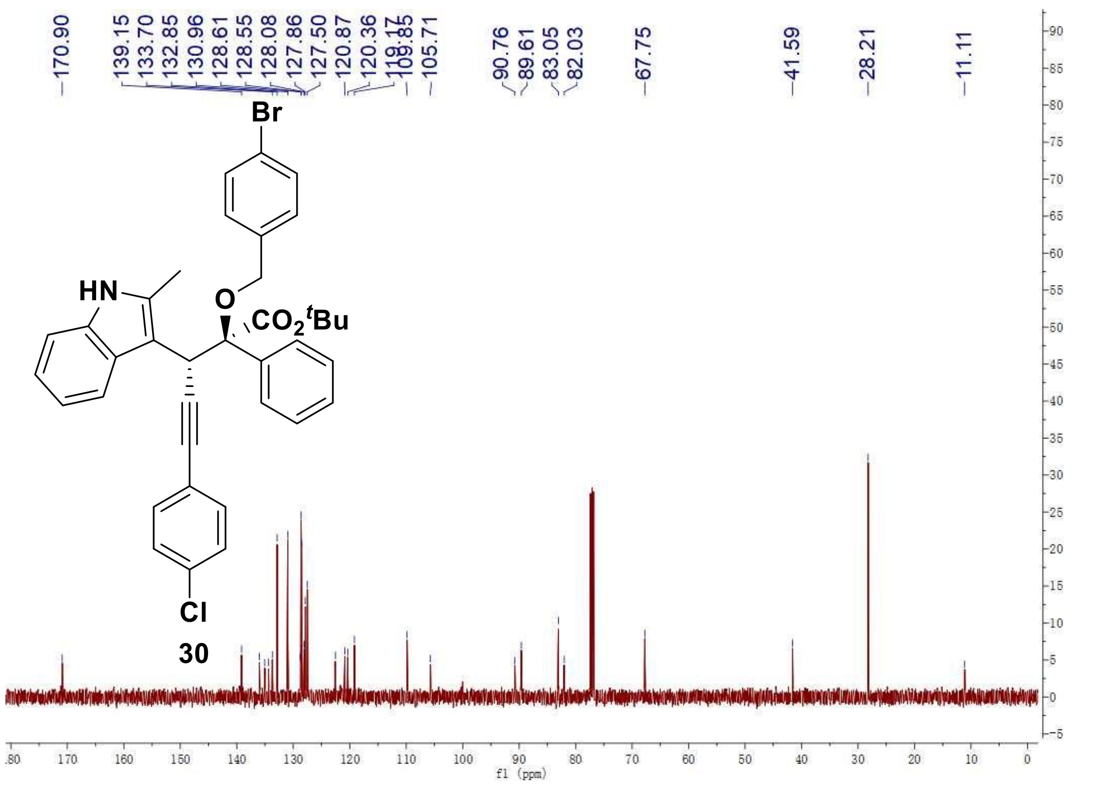

Supplementary Figure 326. ^{13}C NMR (126 MHz, $CDCl_3$) spectrum of **30**.

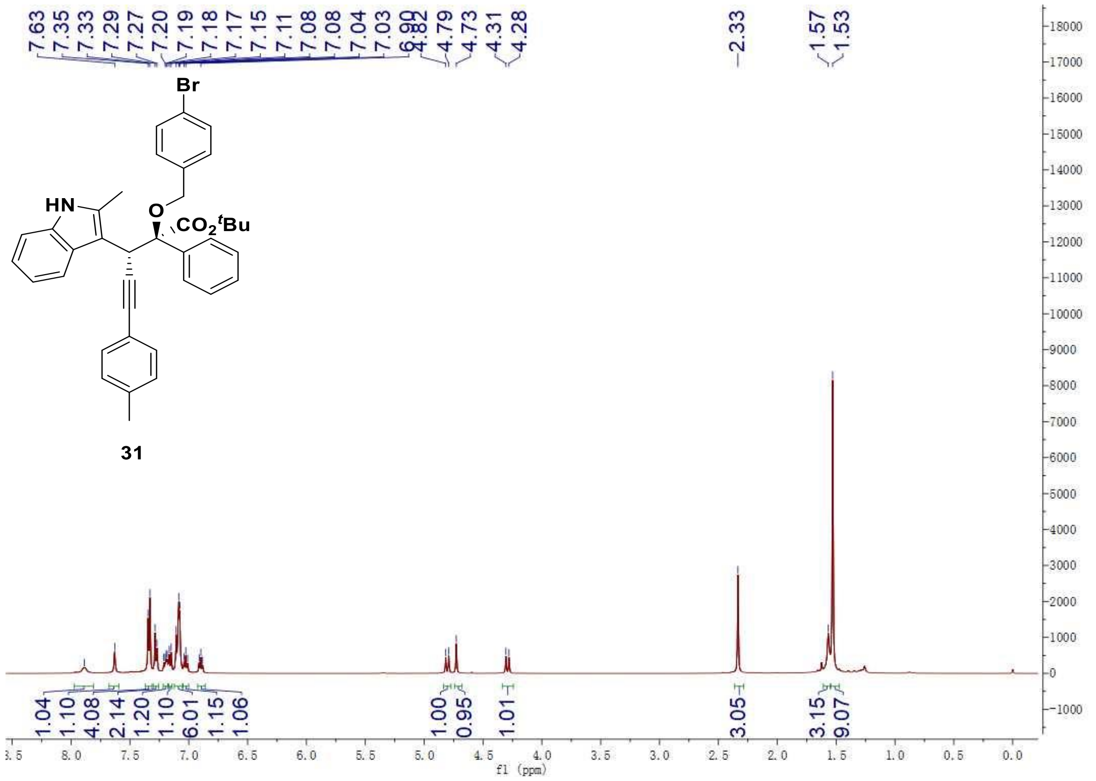

Supplementary Figure 327. ^{1}H NMR (500 MHz, $CDCl_3$) spectrum of **31**.

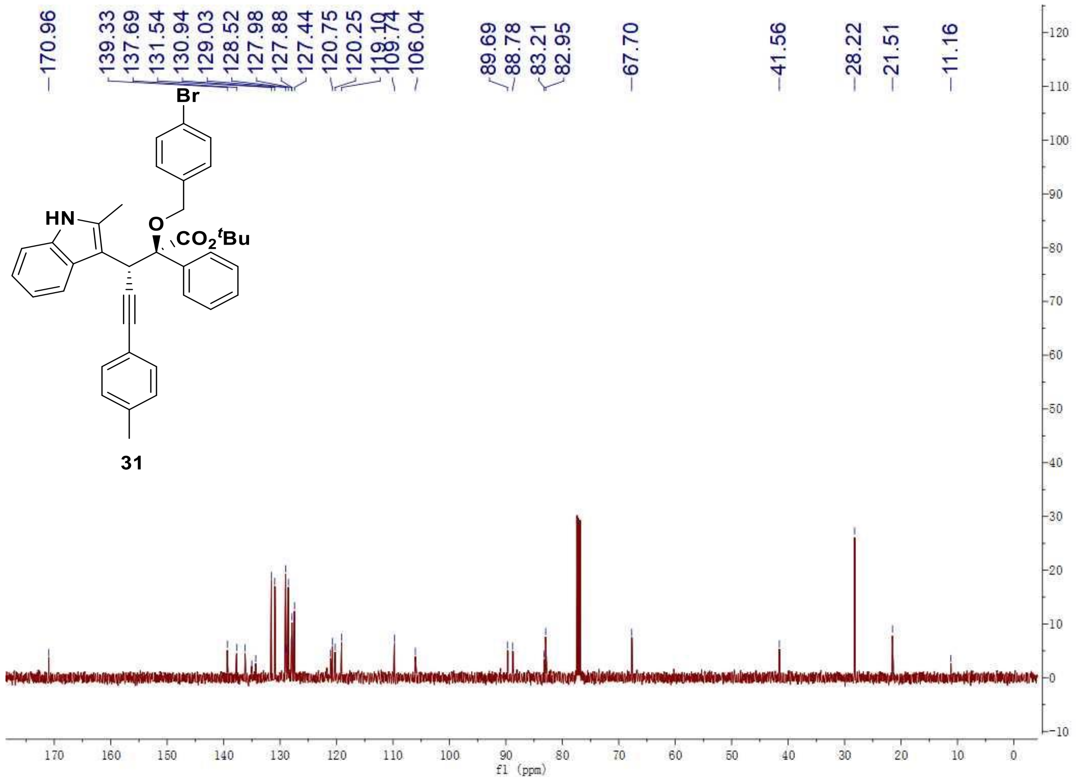

Supplementary Figure 328. ^{13}C NMR (126 MHz, $CDCl_3$) spectrum of **31**.

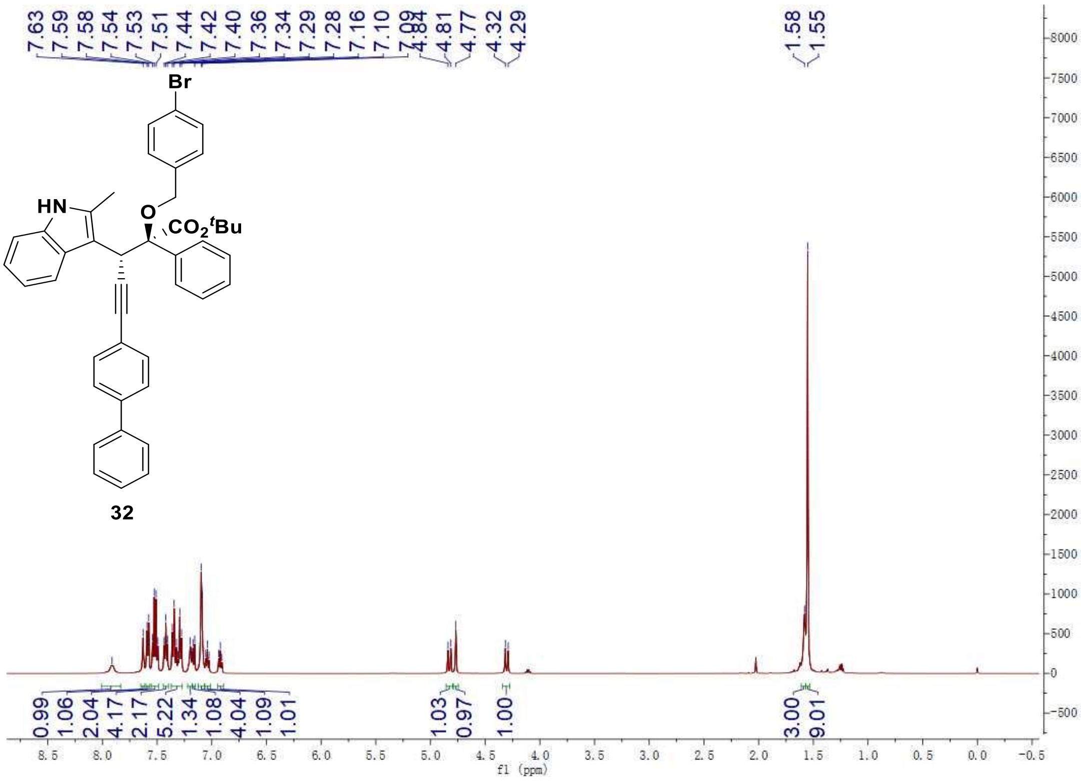

Supplementary Figure 329. ^{1}H NMR (500 MHz, $CDCl_3$) spectrum of **32**.

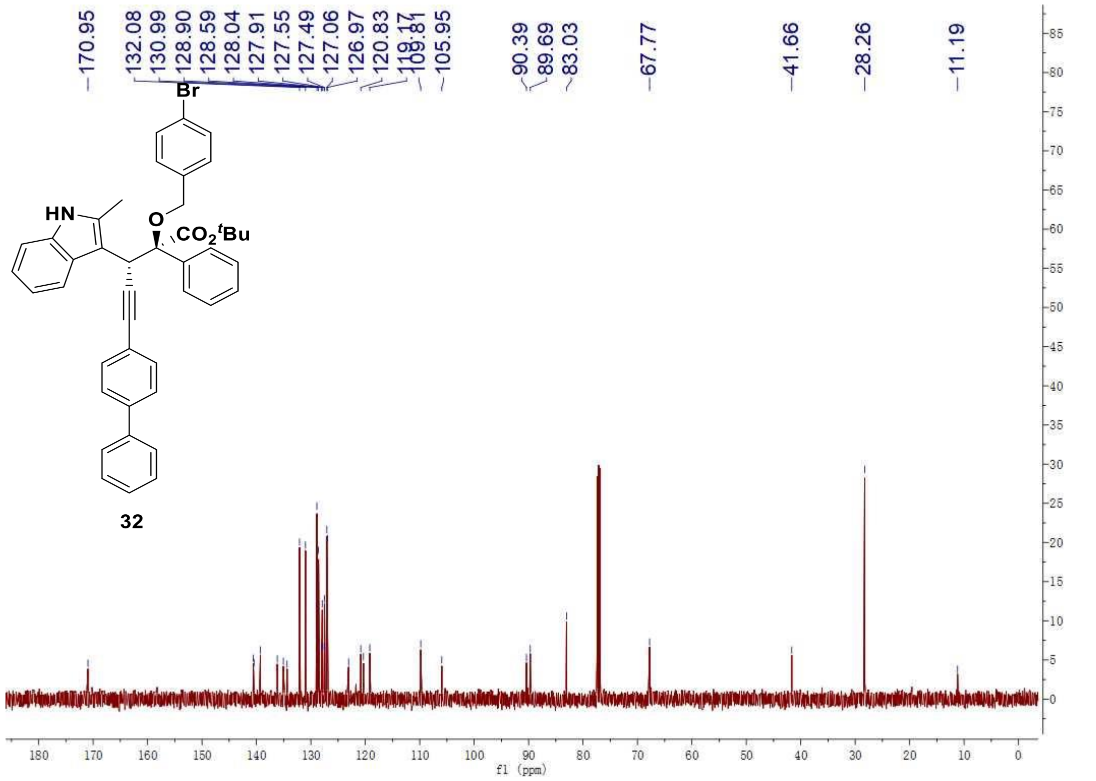

Supplementary Figure 330. ^{13}C NMR (126 MHz, $CDCl_3$) spectrum of **32**.

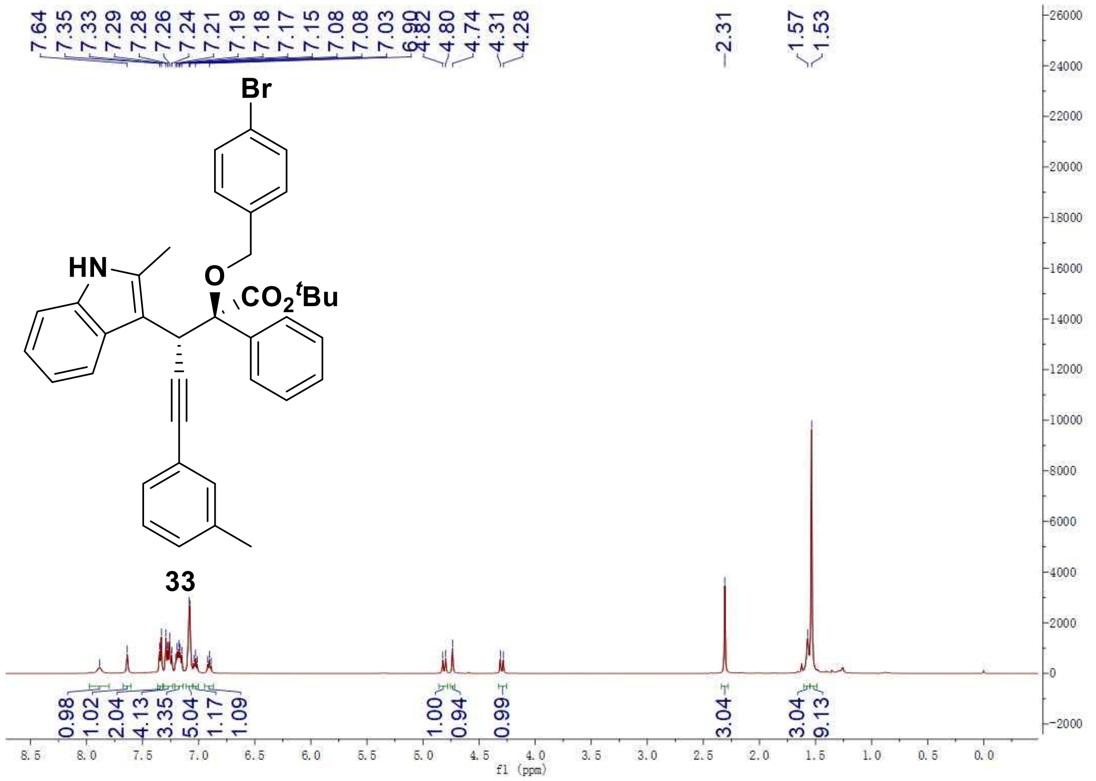

Supplementary Figure 331. ^{1}H NMR (500 MHz, $CDCl_3$) spectrum of **33**.

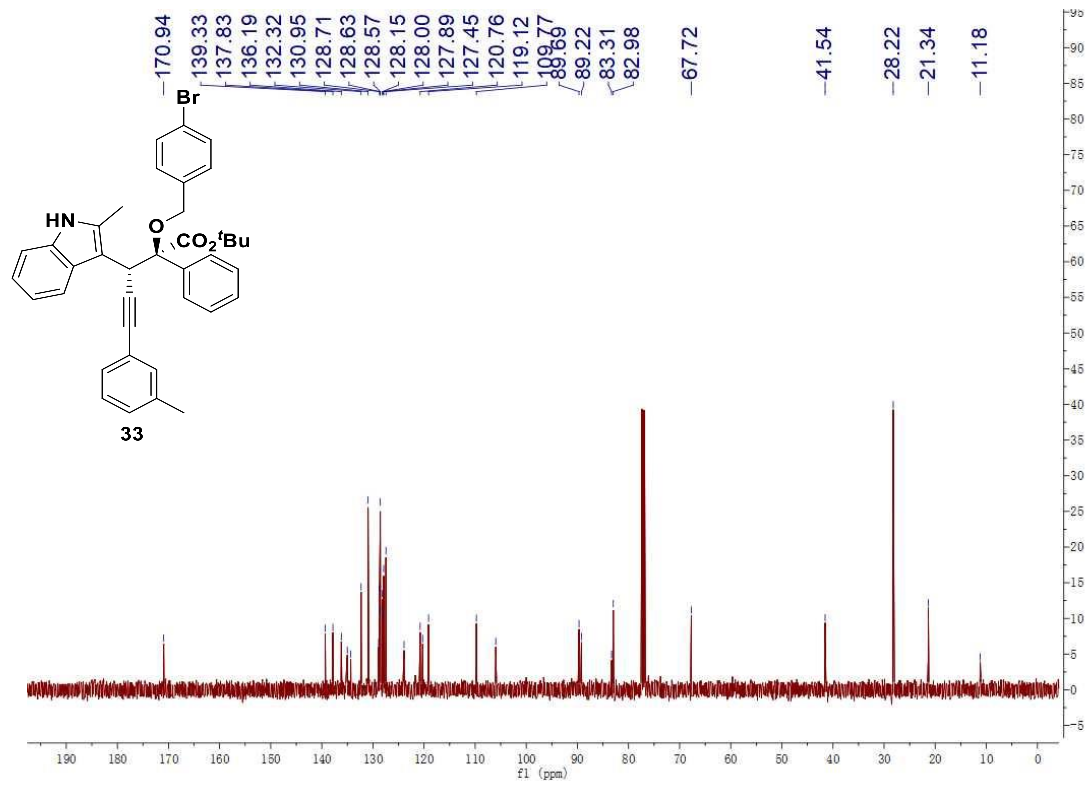

Supplementary Figure 332. ^{13}C NMR (126 MHz, $CDCl_3$) spectrum of **33**.

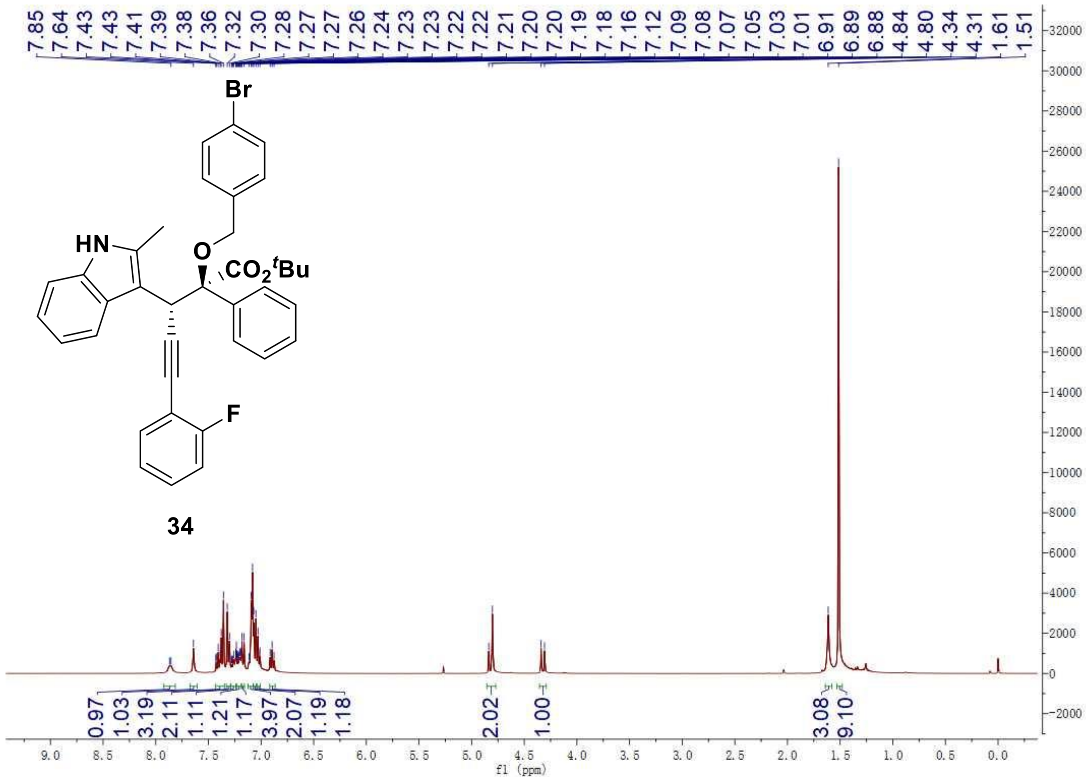

Supplementary Figure 333. ^{1}H NMR (400 MHz, $CDCl_3$) spectrum of **34**.

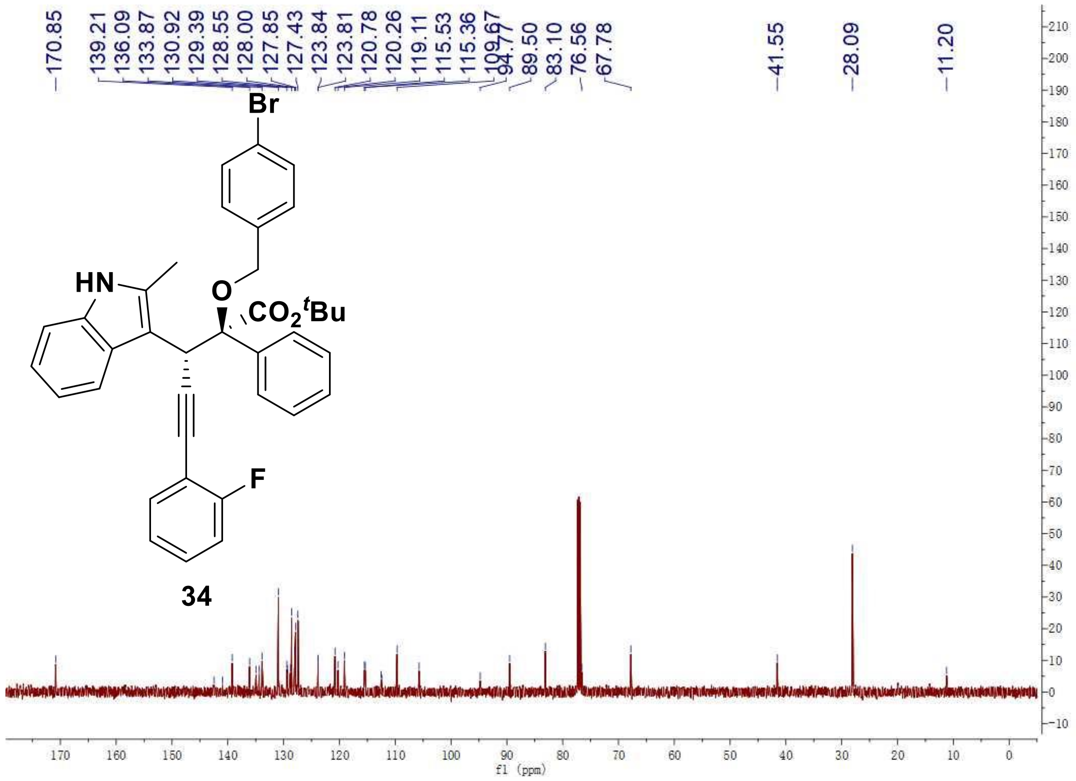

Supplementary Figure 334. ^{13}C NMR (101 MHz, $CDCl_3$) spectrum of **34**.

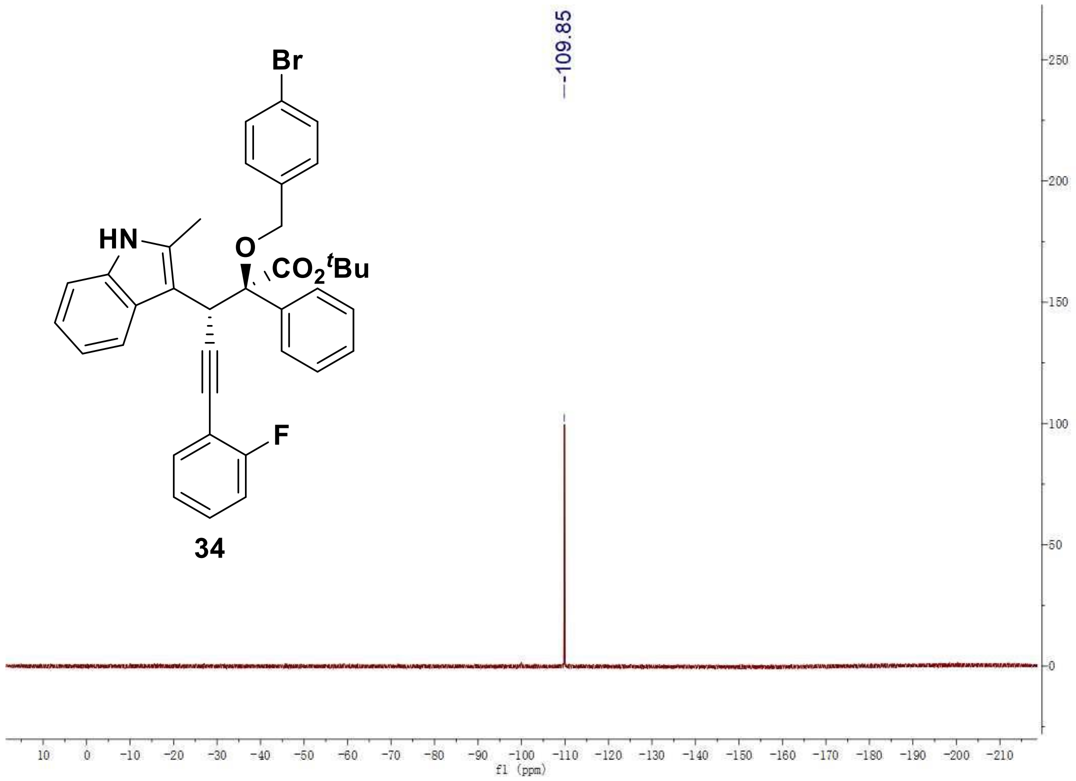

Supplementary Figure 335. ^{19}F NMR (376 MHz, $CDCl_3$) spectrum of **34**.

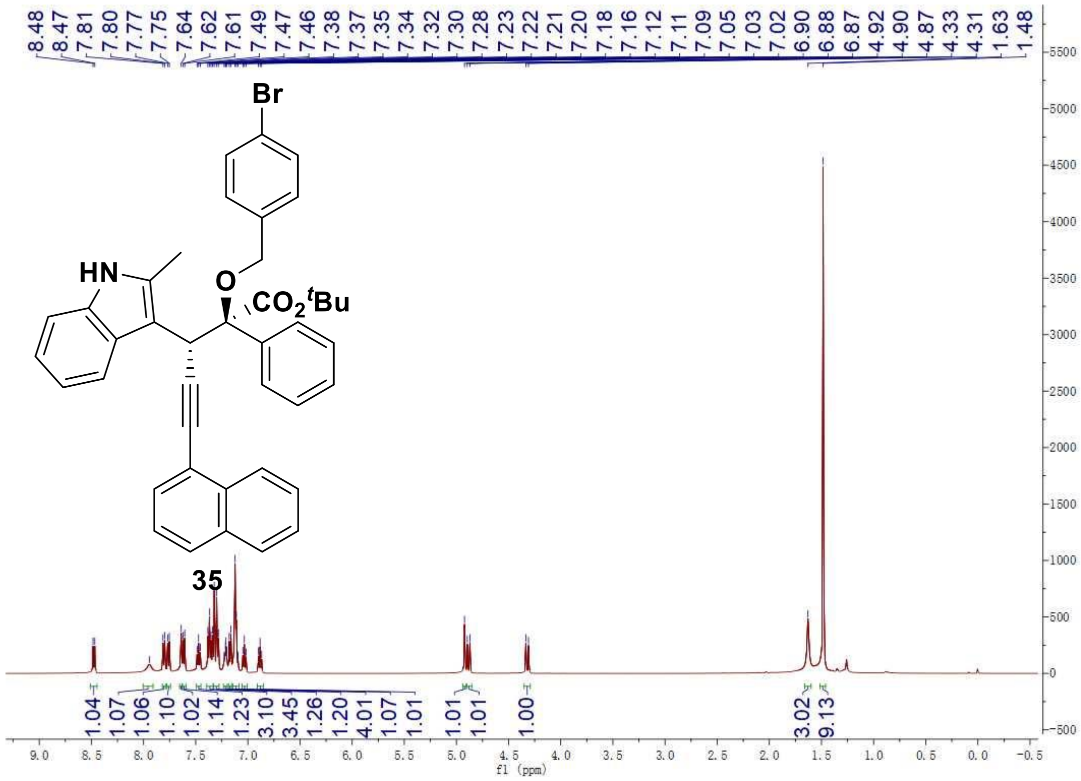

Supplementary Figure 336. ^{1}H NMR (500 MHz, $CDCl_3$) spectrum of **35**.

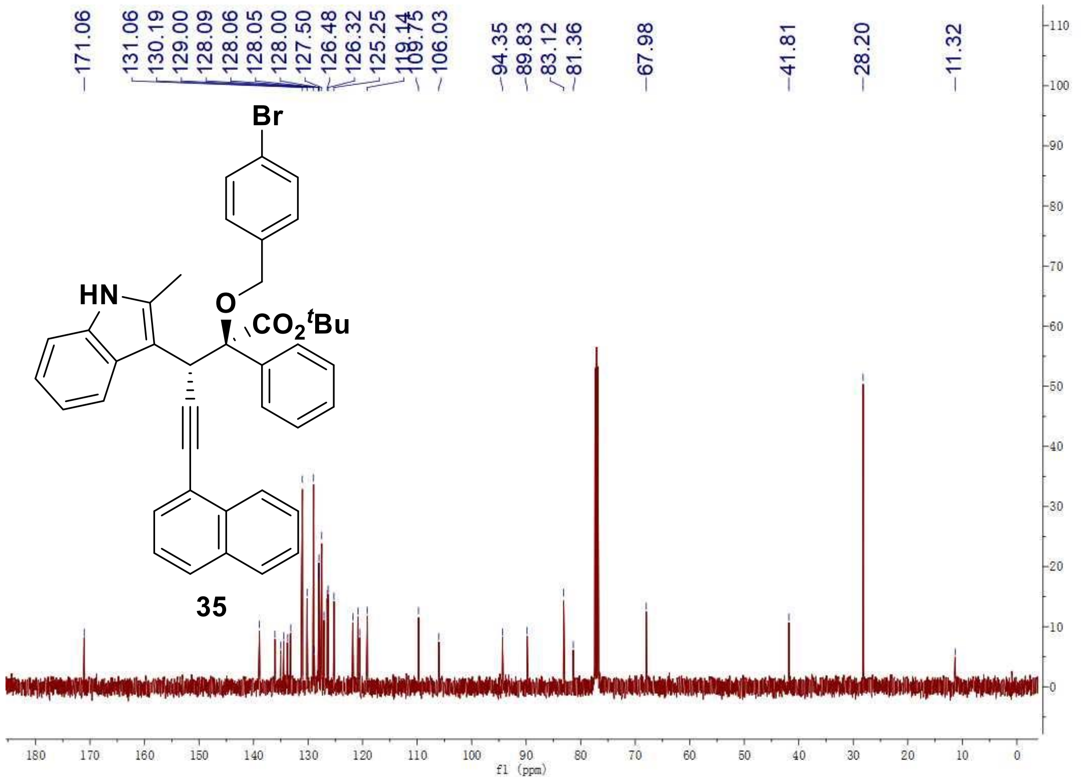

Supplementary Figure 337. ^{13}C NMR (126 MHz, $CDCl_3$) spectrum of **35**.

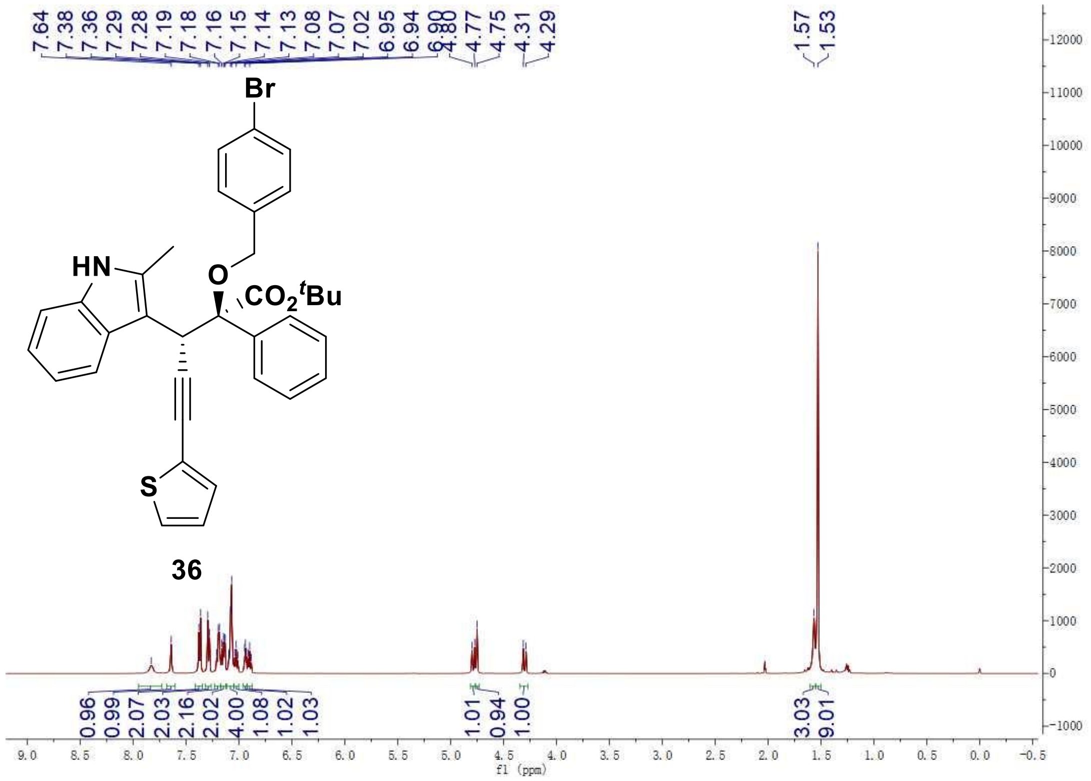

Supplementary Figure 338. ^{1}H NMR (500 MHz, $CDCl_3$) spectrum of **36**.

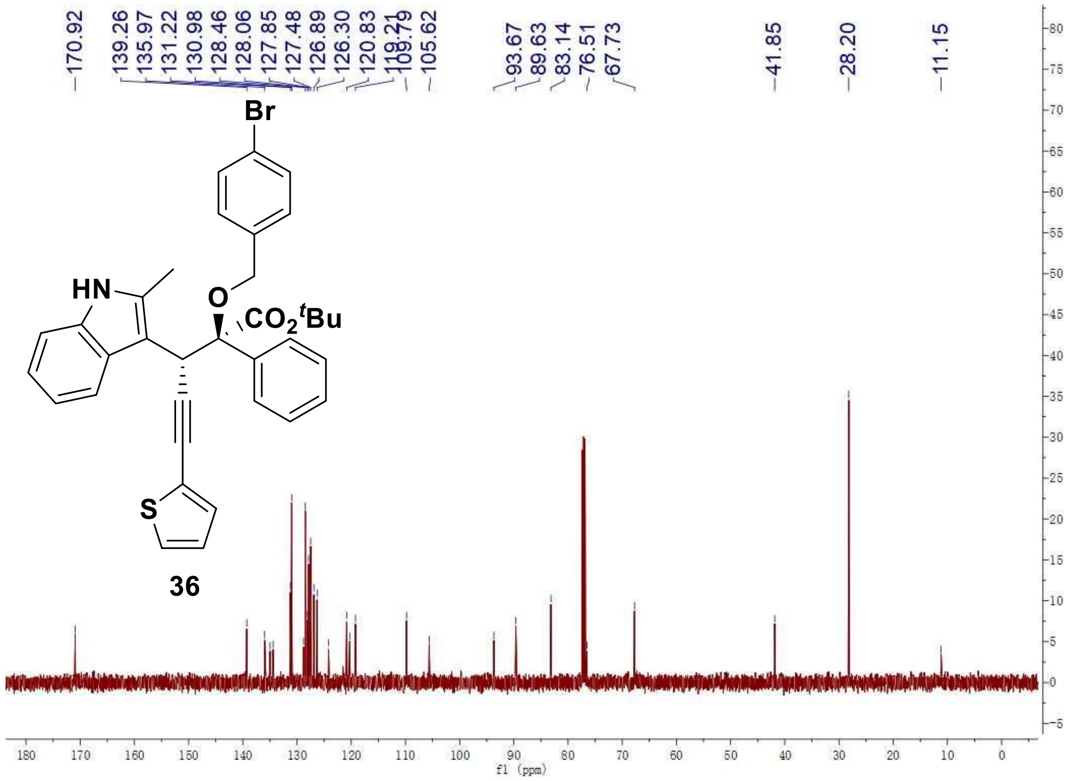

Supplementary Figure 339. ^{13}C NMR (126 MHz, $CDCl_3$) spectrum of **36**.

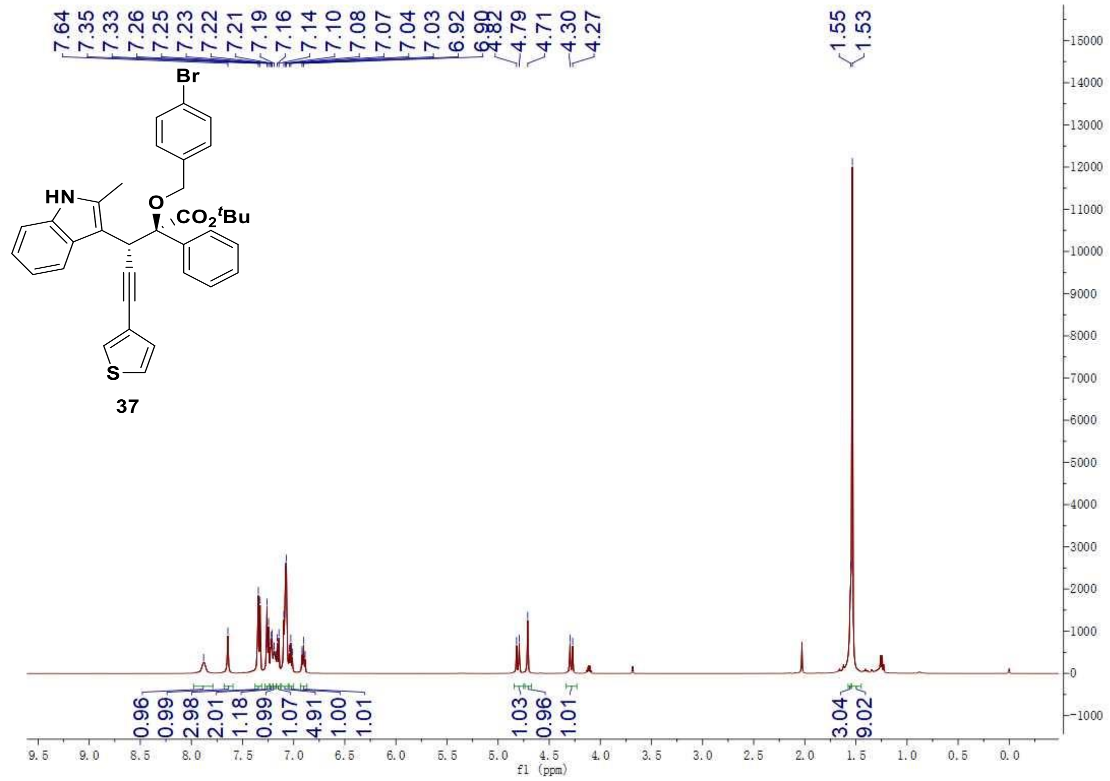

Supplementary Figure 340. ^{1}H NMR (500 MHz, $CDCl_3$) spectrum of **37**.

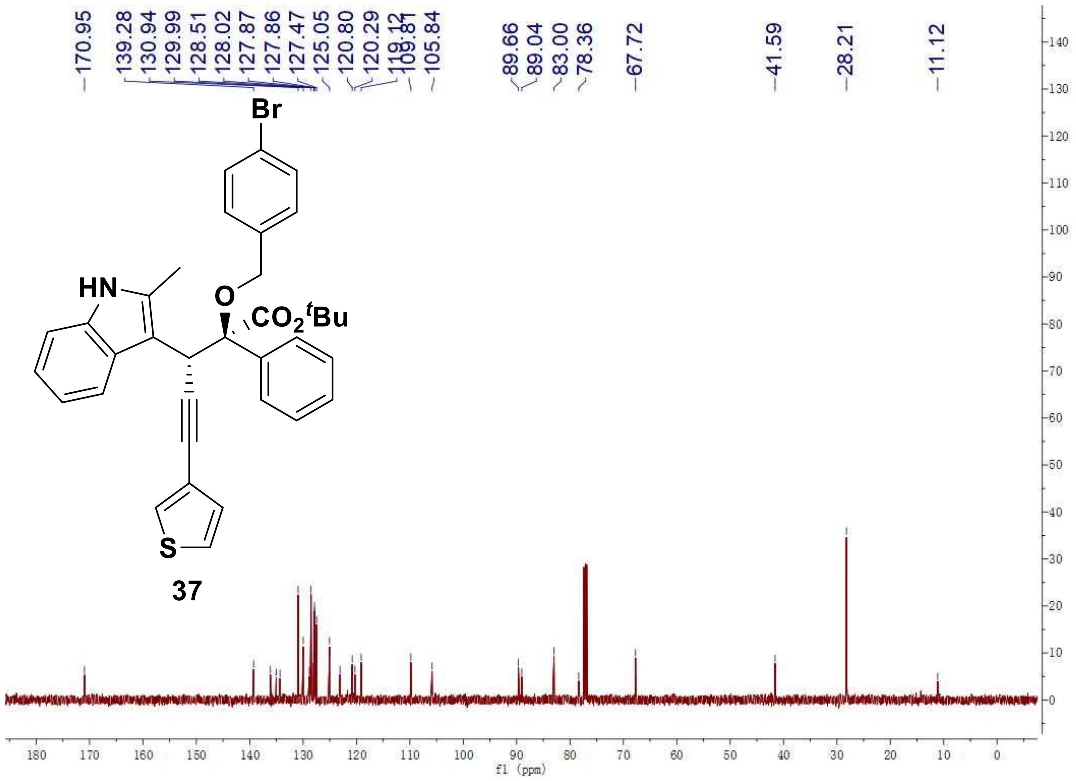

Supplementary Figure 341. ^{13}C NMR (126 MHz, $CDCl_3$) spectrum of **37**.

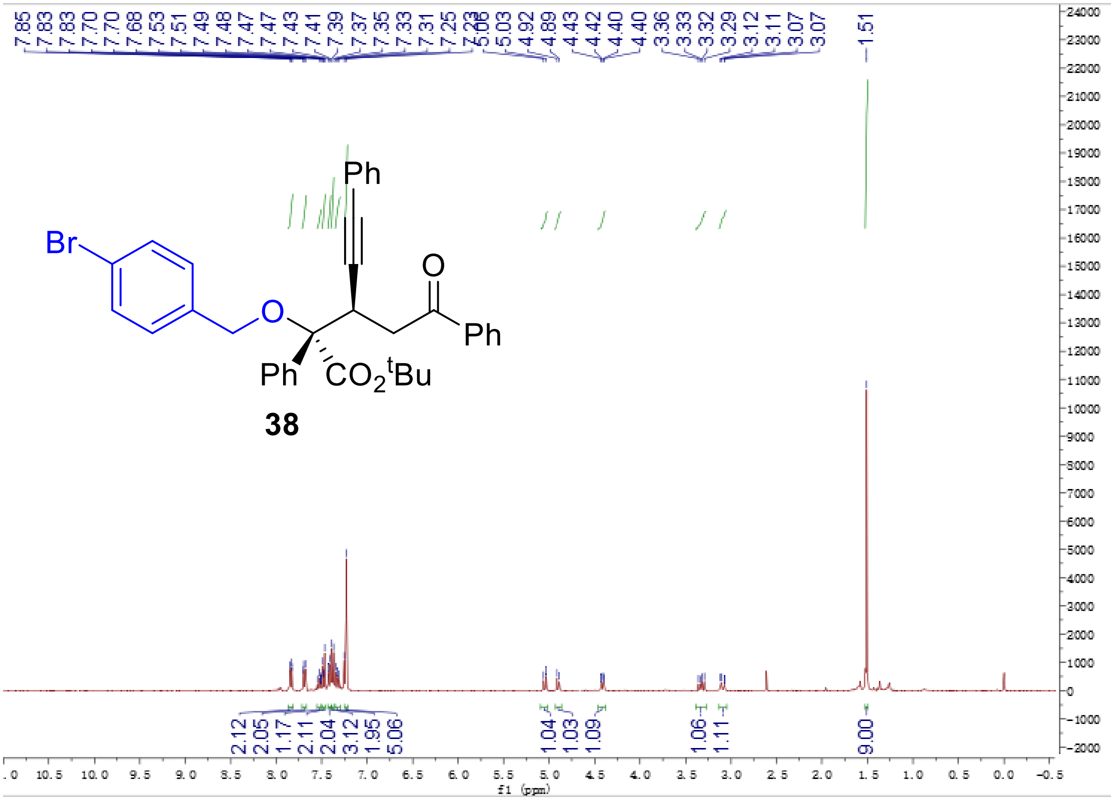

Supplementary Figure 342. ^{1}H NMR (400 MHz, $CDCl_3$) spectrum of **38**.

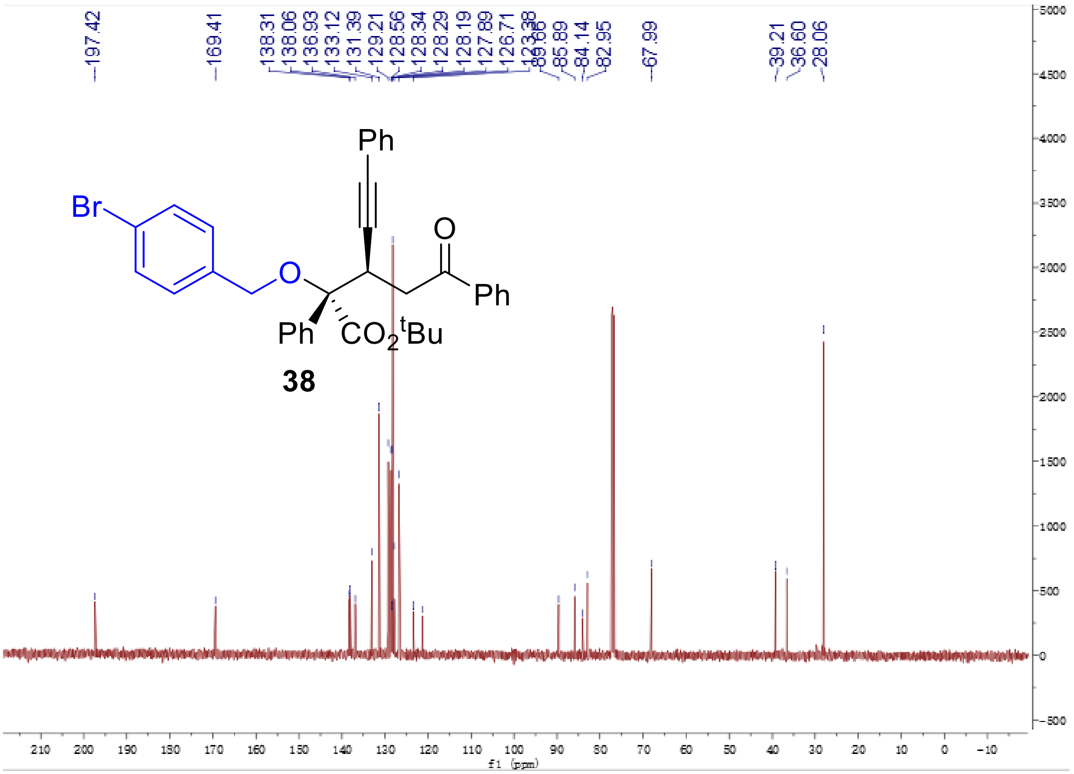

Supplementary Figure 343. ^{13}C NMR (101 MHz, $CDCl_3$) spectrum of **38**.

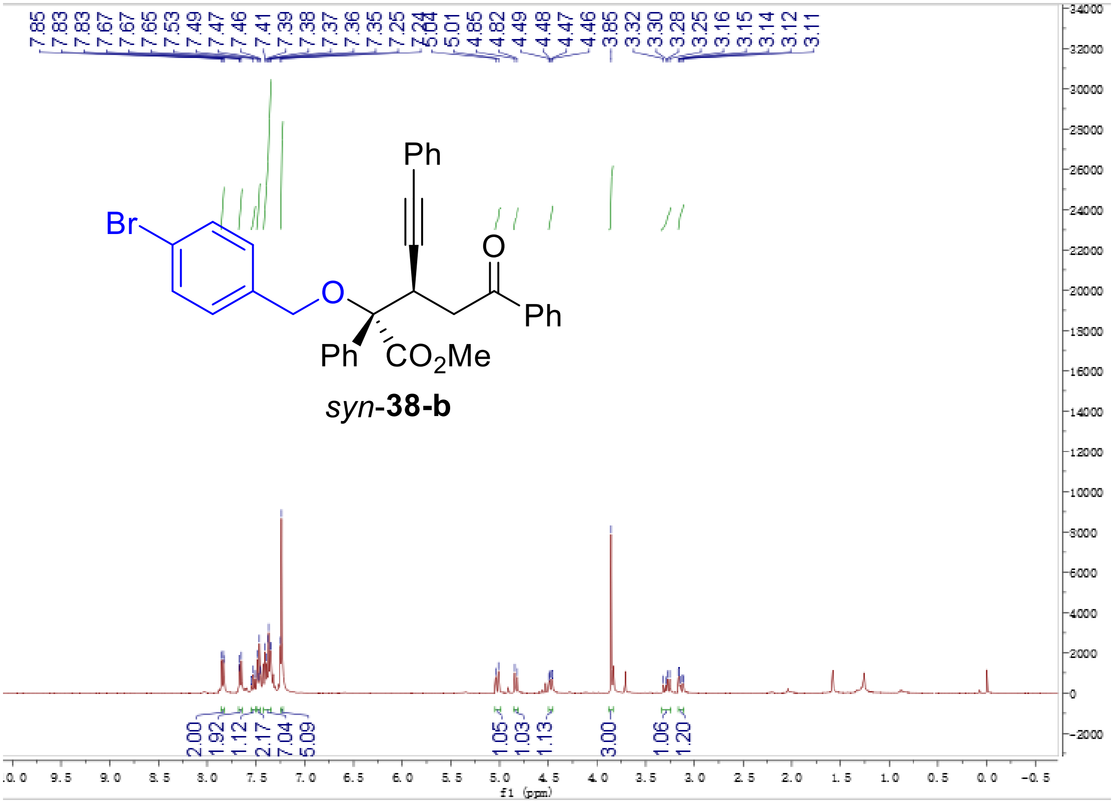

Supplementary Figure 344. ^{1}H NMR (400 MHz, $CDCl_3$) spectrum of **38-b**.

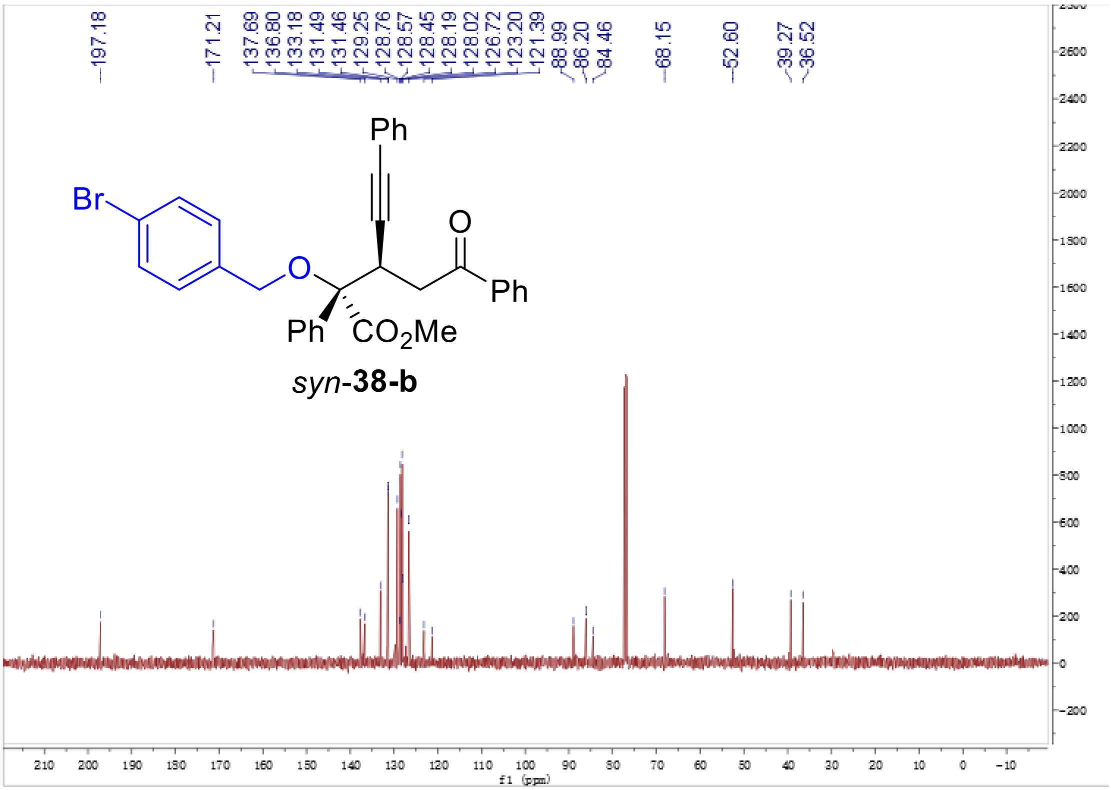

Supplementary Figure 345. ^{13}C NMR (101 MHz, $CDCl_3$) spectrum of **38-b**.

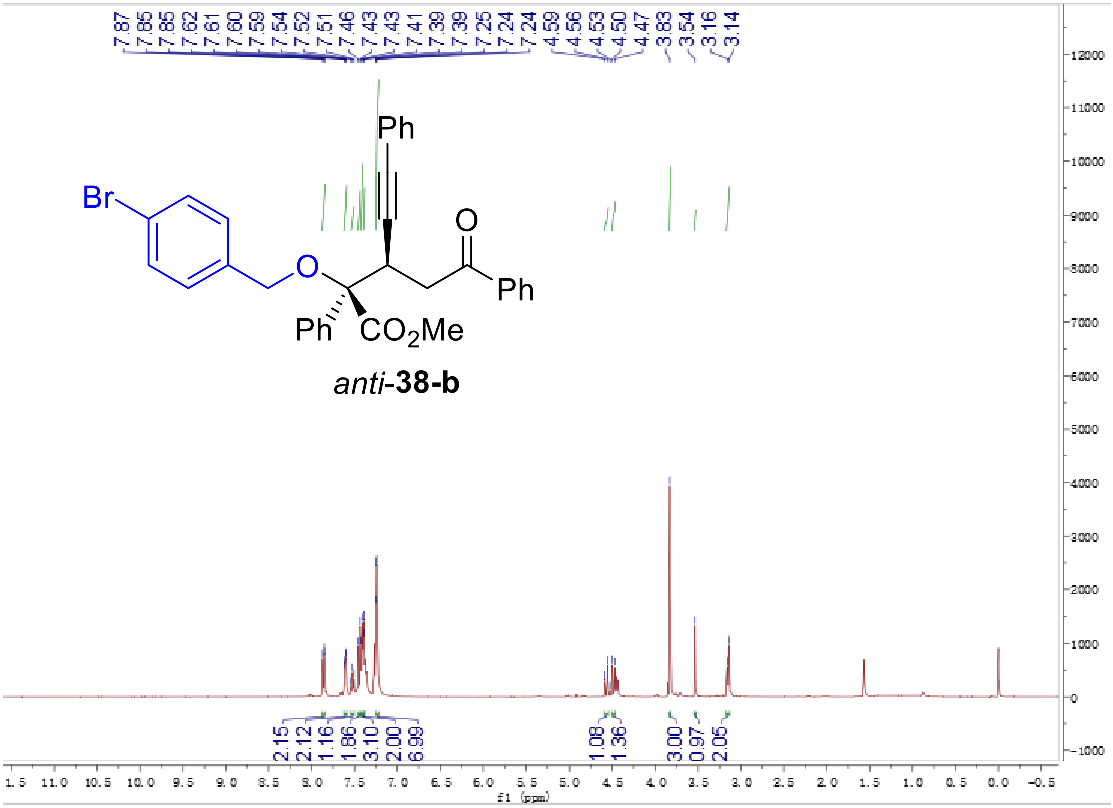

Supplementary Figure 346. ^{1}H NMR (400 MHz, $CDCl_3$) spectrum of **anti-38-b**.

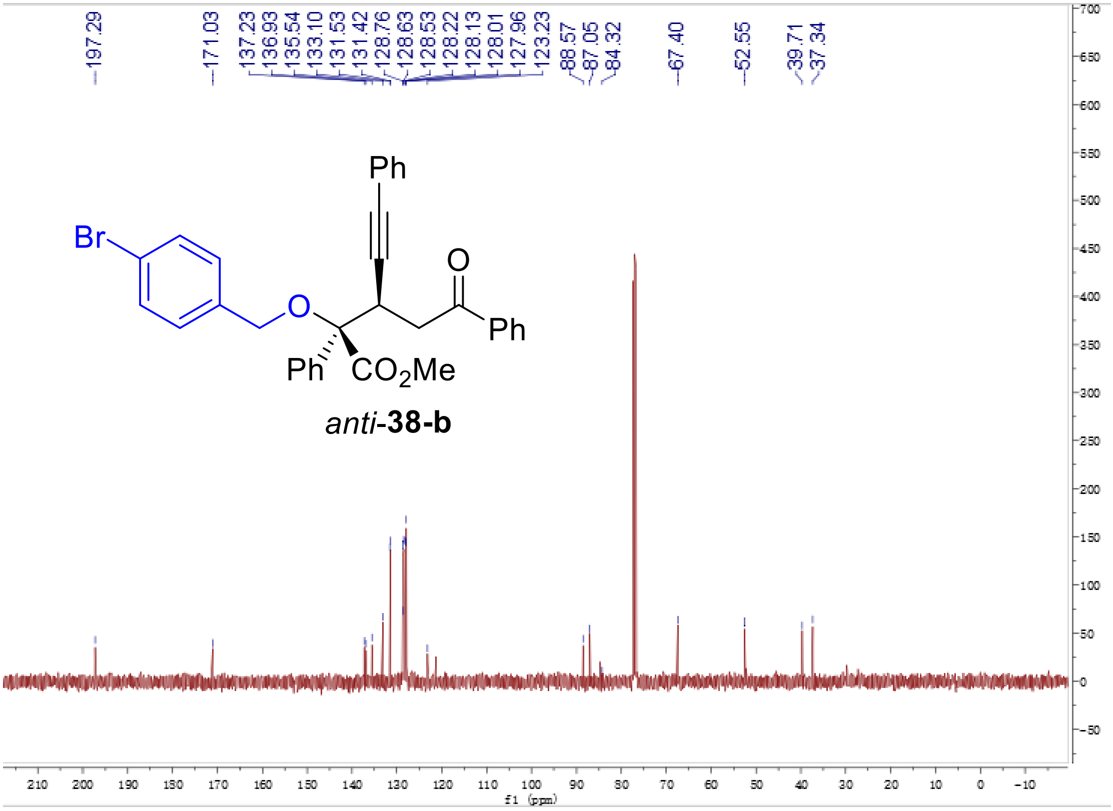

Supplementary Figure 347. ^{13}C NMR (101 MHz, $CDCl_3$) spectrum of **anti-38-b**.

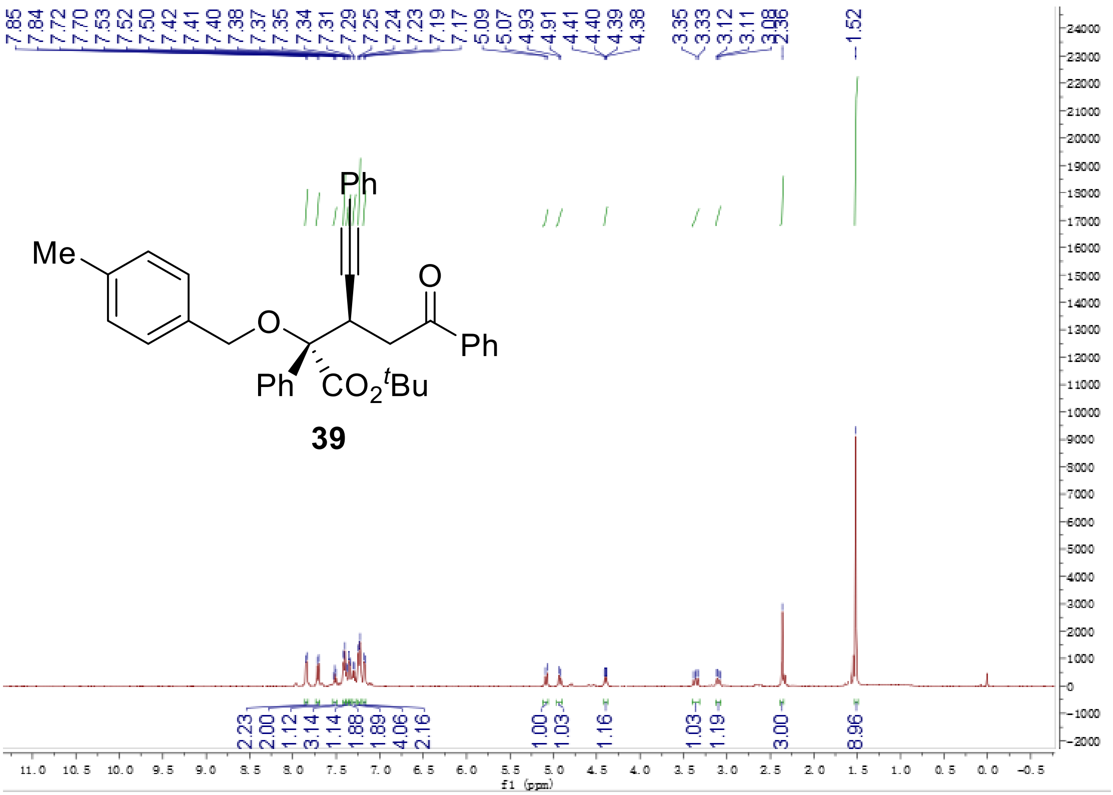

Supplementary Figure 348. ^{1}H NMR (500 MHz, $CDCl_3$) spectrum of **39**.

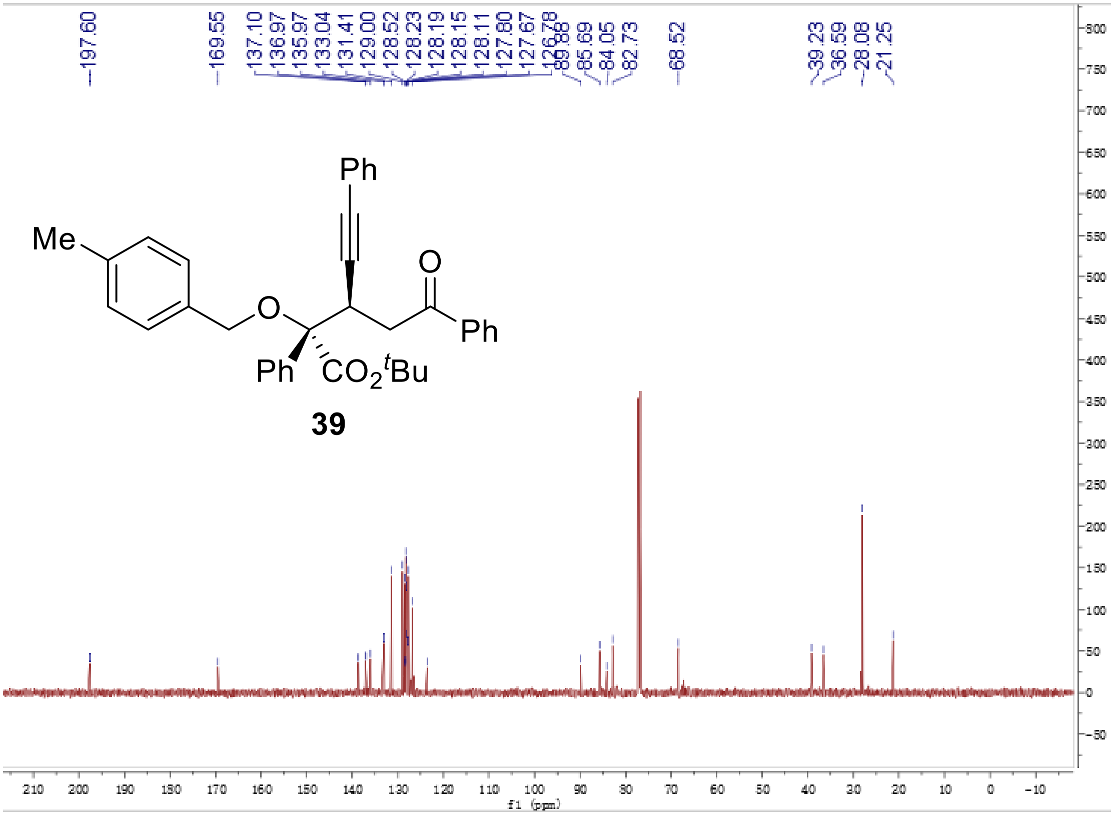

Supplementary Figure 349. ^{13}C NMR (126 MHz, $CDCl_3$) spectrum of **39**.

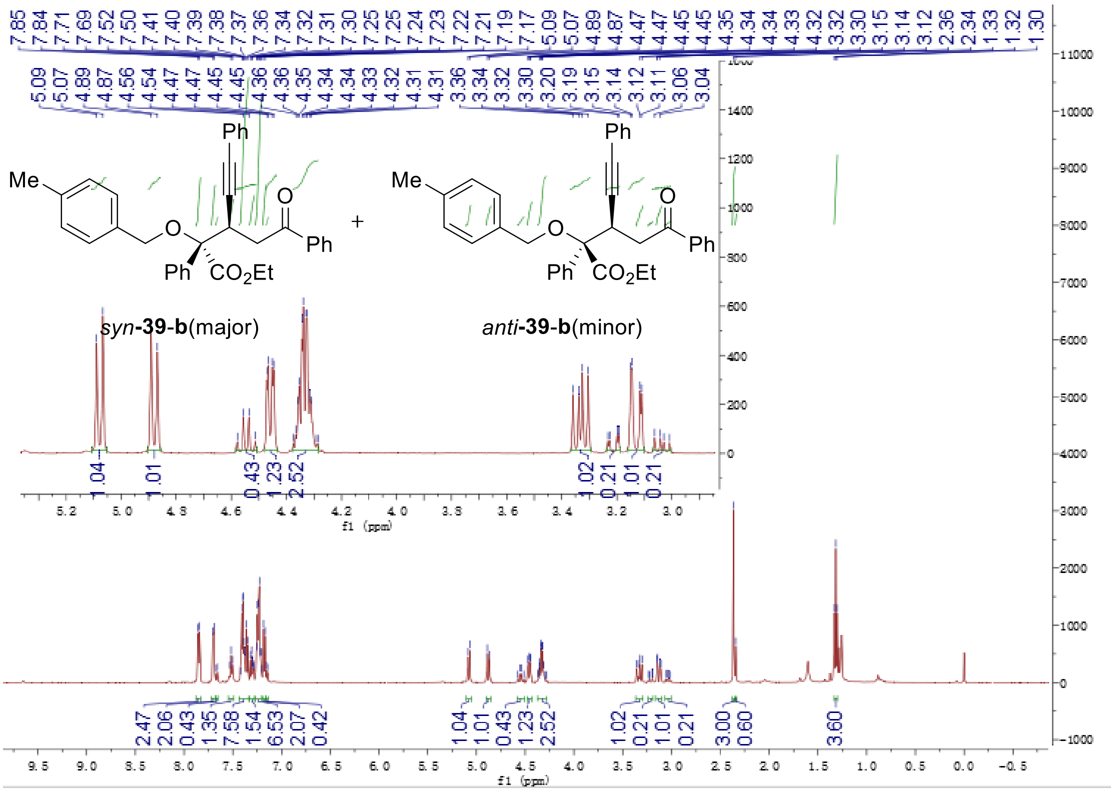

Supplementary Figure 350. ^{1}H NMR (500 MHz, $CDCl_3$) spectrum of **39-b**.

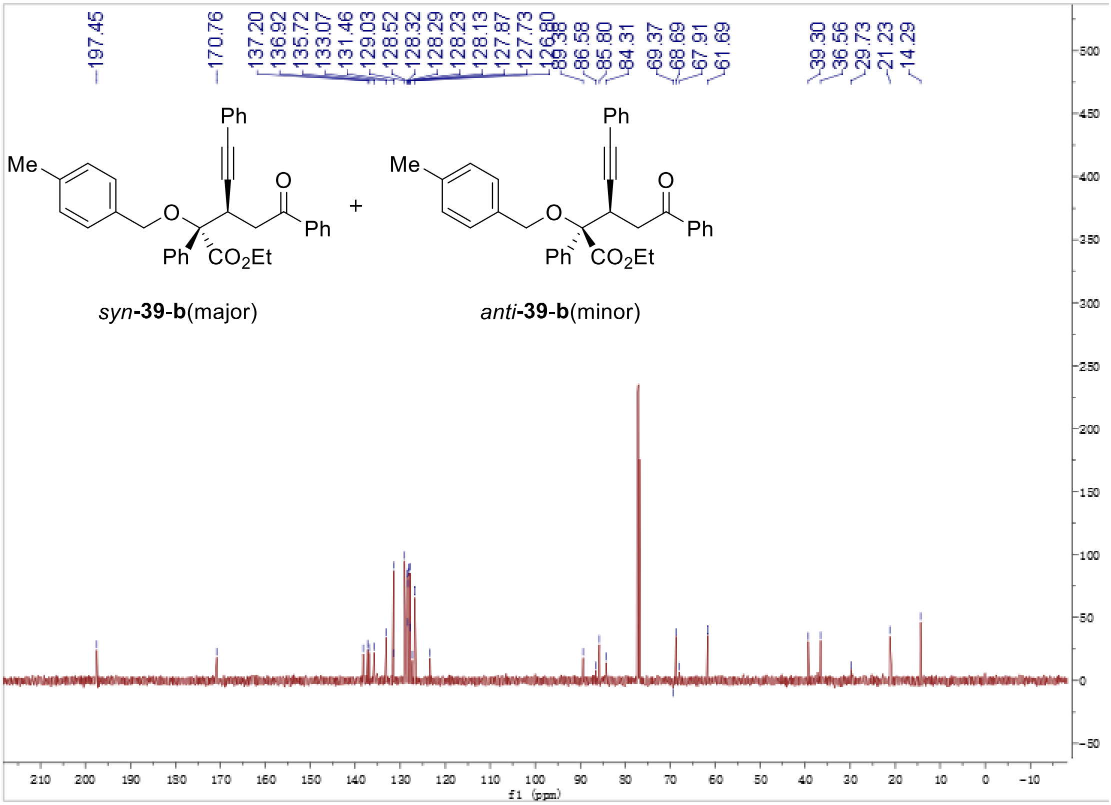

Supplementary Figure 351. ^{13}C NMR (126 MHz, $CDCl_3$) spectrum of **39-b**.

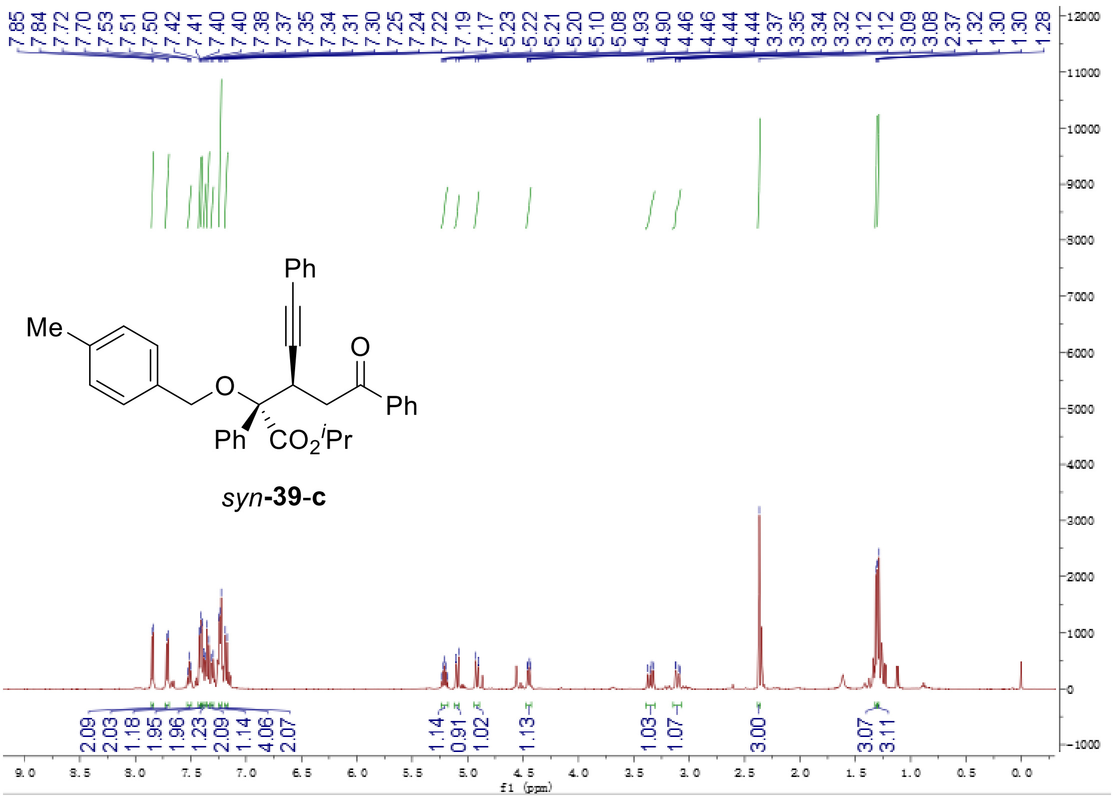

Supplementary Figure 352. ^{1}H NMR (500 MHz, $CDCl_3$) spectrum of **39-c**.

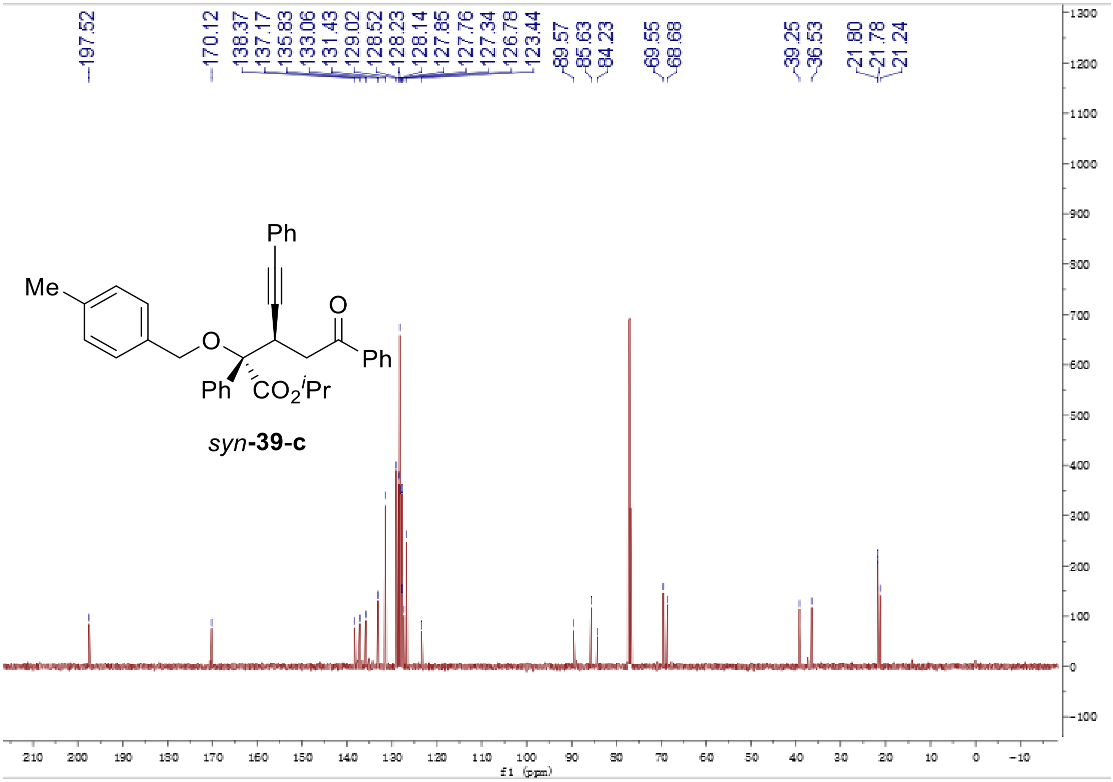

Supplementary Figure 353. ^{13}C NMR (126 MHz, $CDCl_3$) spectrum of **39-c**.

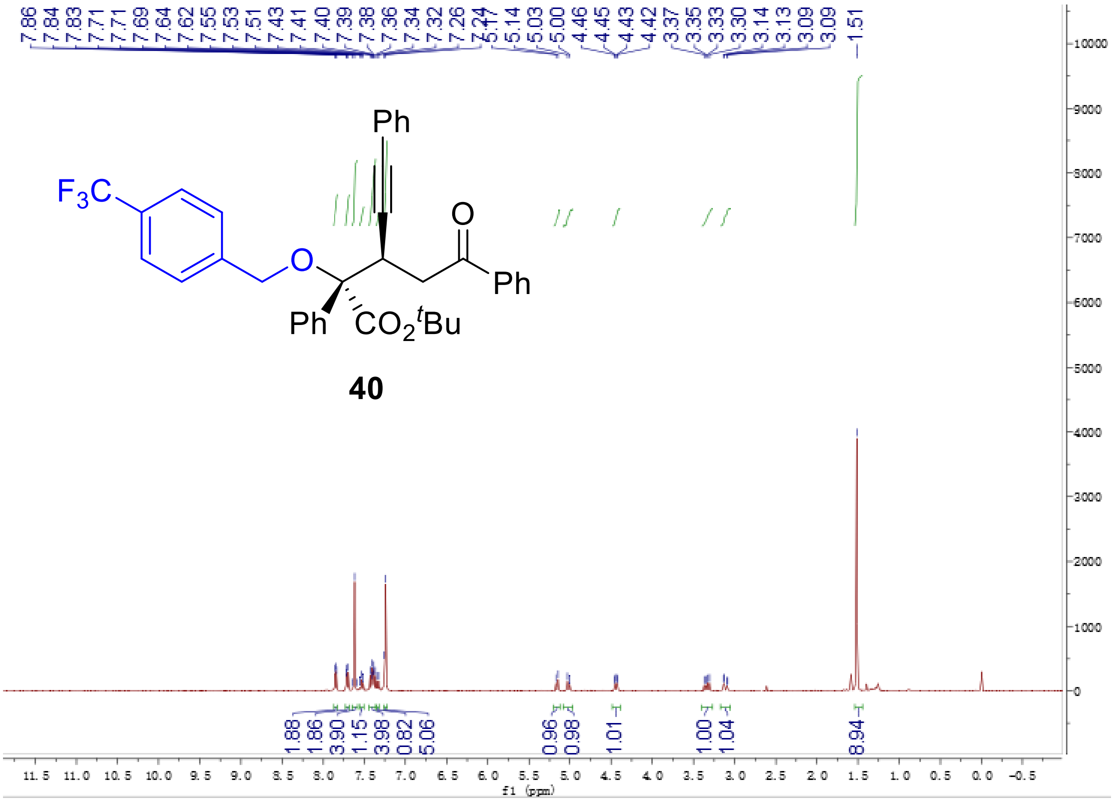

Supplementary Figure 354. ^{1}H NMR (400 MHz, $CDCl_3$) spectrum of **40**.

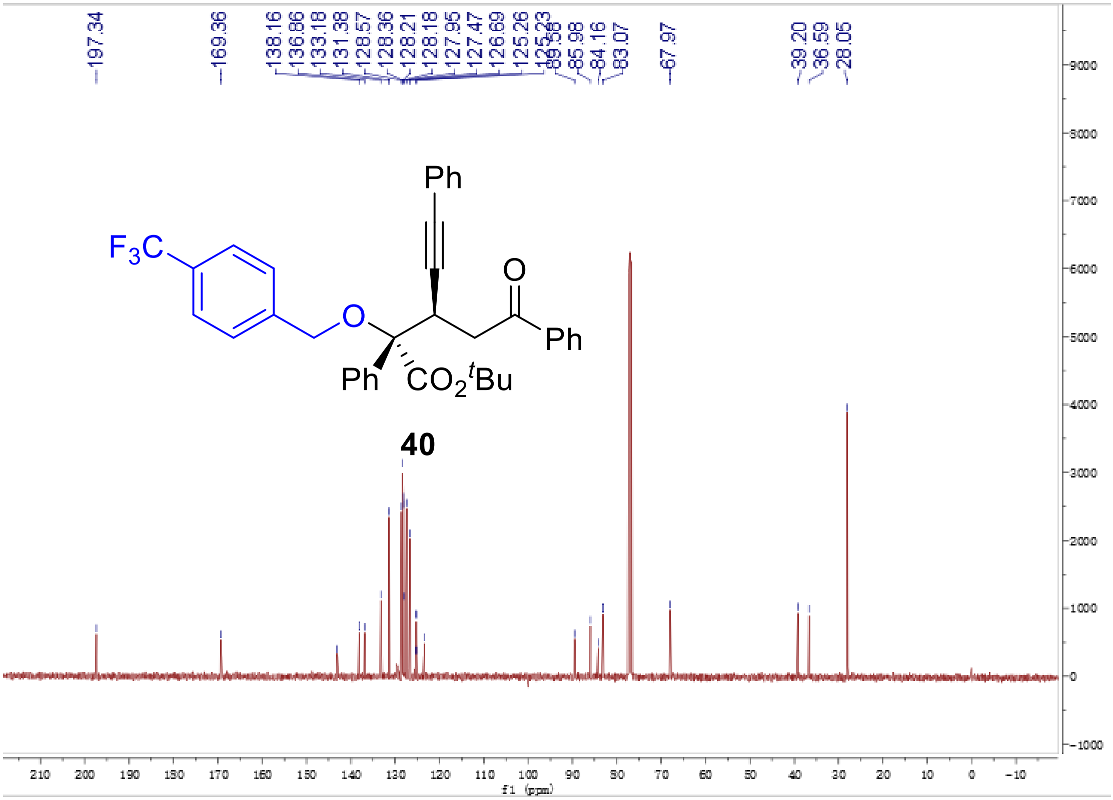

Supplementary Figure 355. ^{13}C NMR (101 MHz, $CDCl_3$) spectrum of **40**.

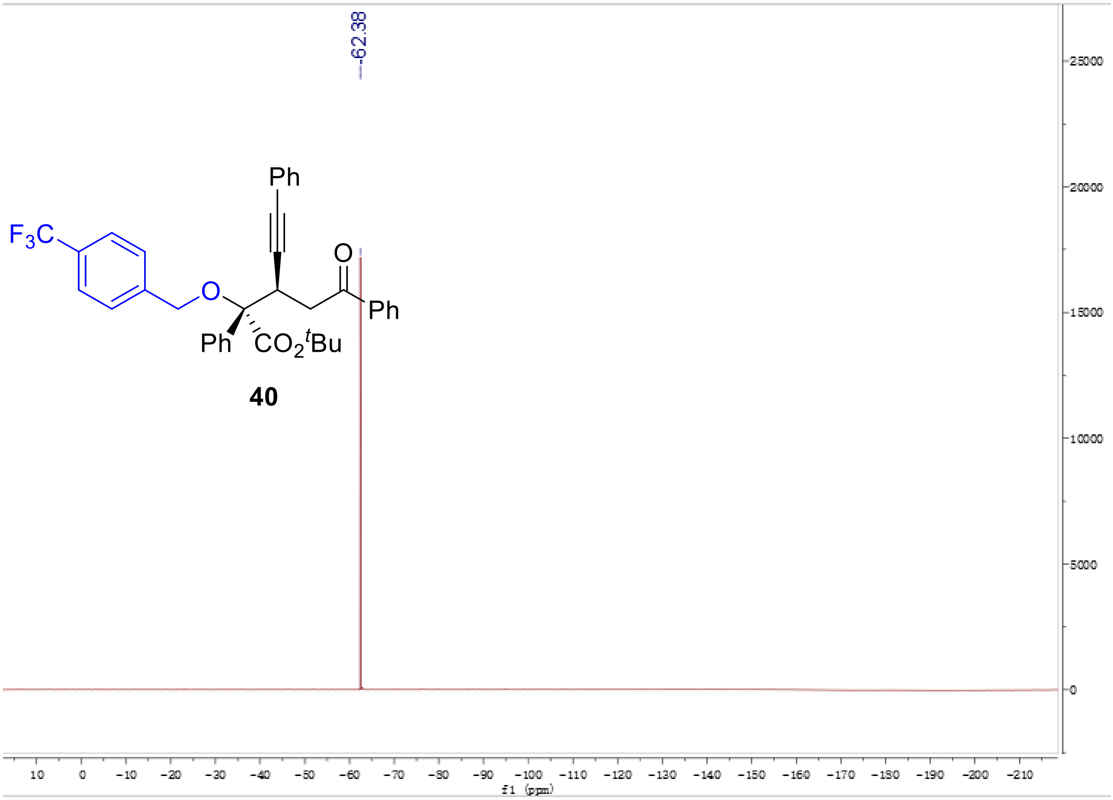

Supplementary Figure 356. ^{19}F NMR (376 MHz, $CDCl_3$) spectrum of **40**.

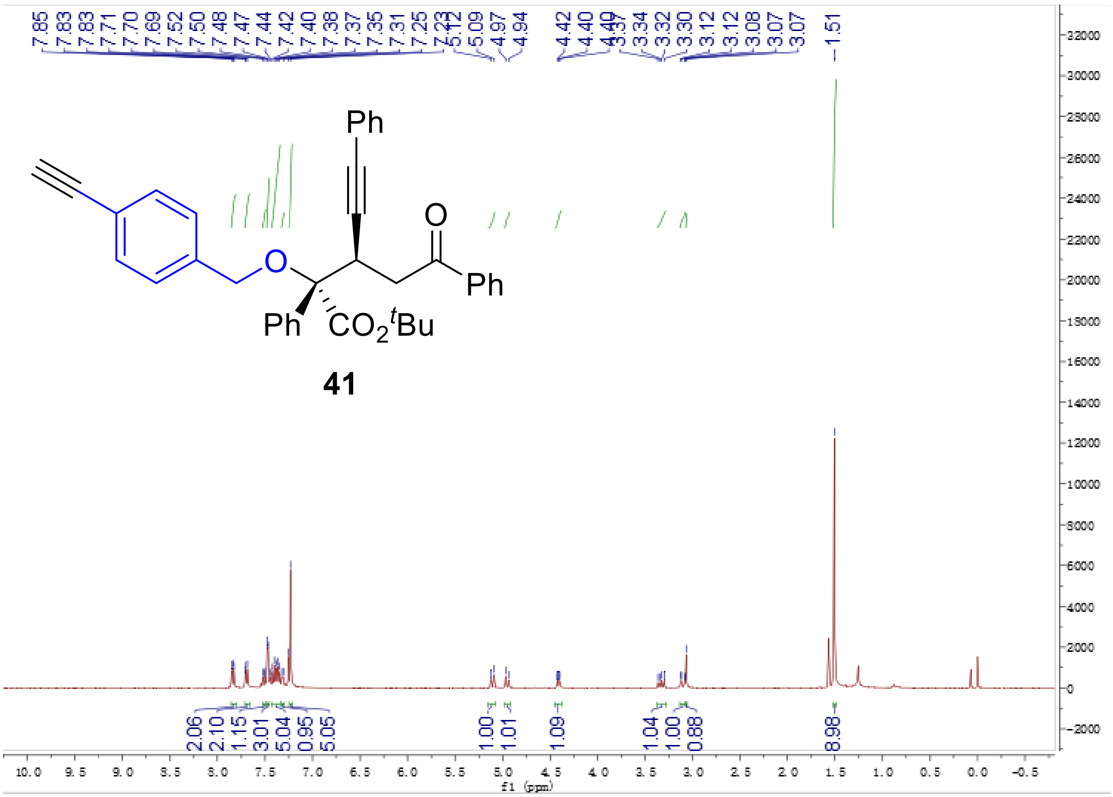

Supplementary Figure 357. ^{1}H NMR (400 MHz, $CDCl_3$) spectrum of **41**.

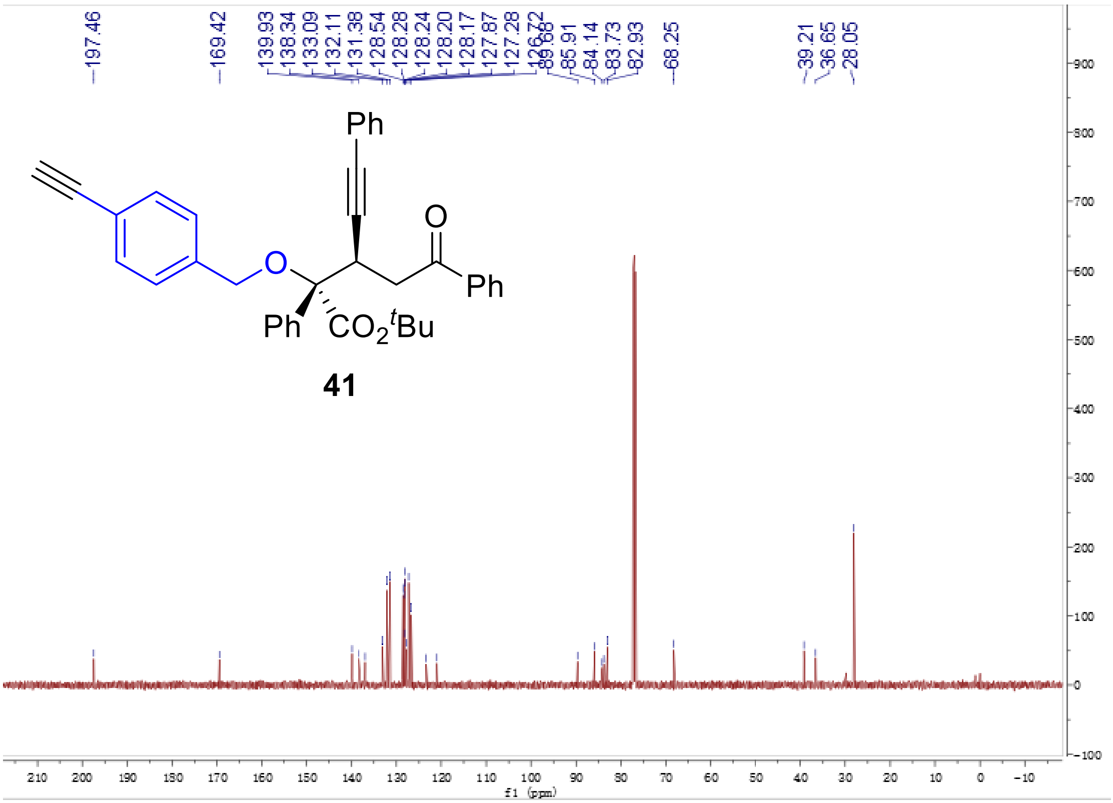

Supplementary Figure 358. ^{13}C NMR (101 MHz, $CDCl_3$) spectrum of **41**.

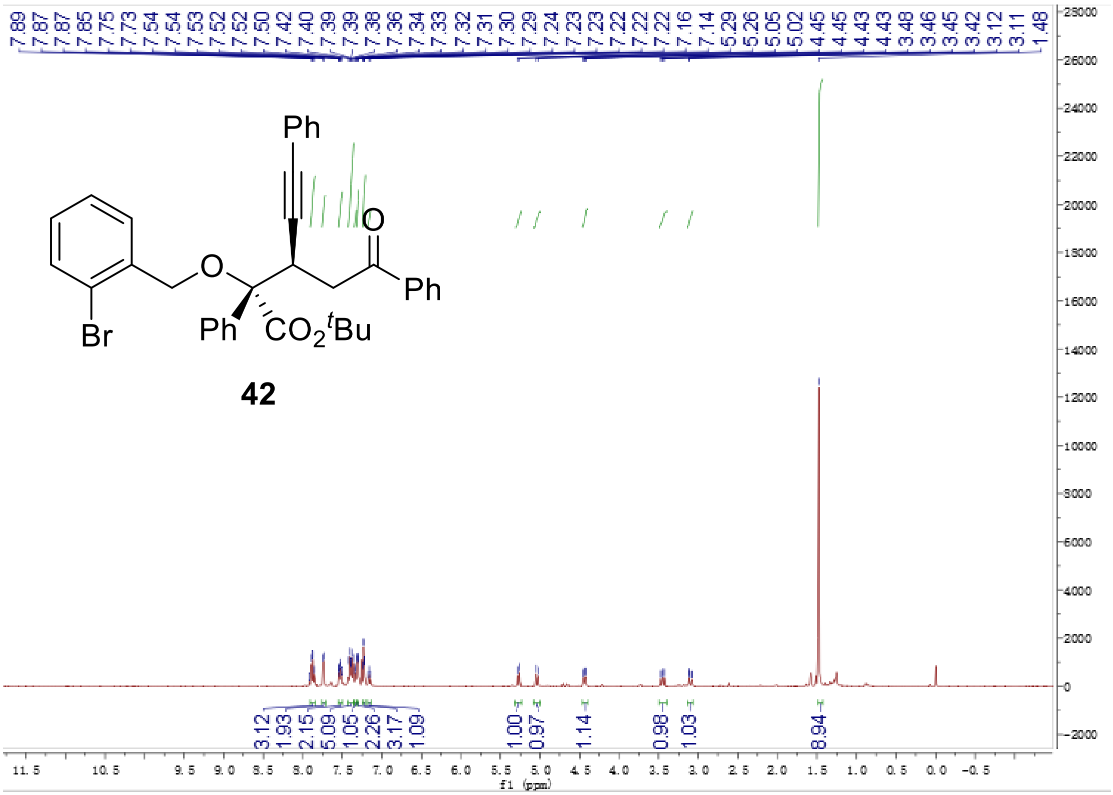

Supplementary Figure 359. ^{1}H NMR (500 MHz, $CDCl_3$) spectrum of **42**.

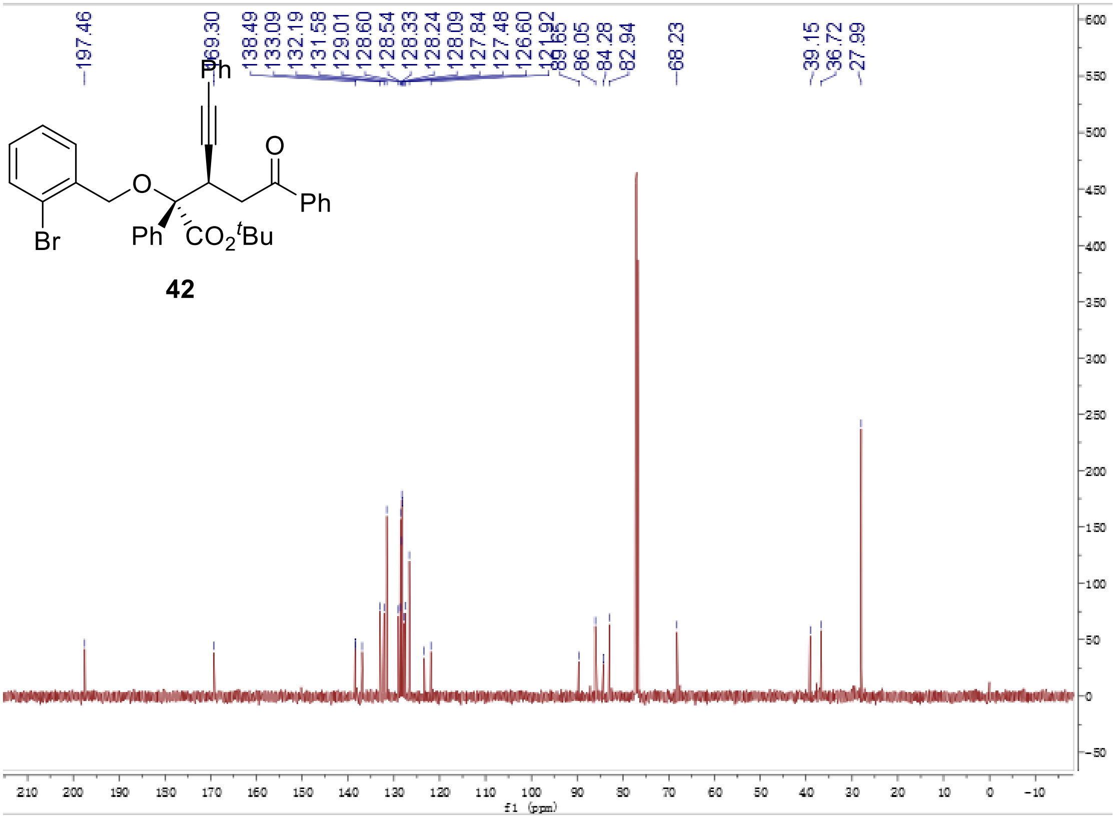

Supplementary Figure 360. ^{13}C NMR (126 MHz, $CDCl_3$) spectrum of **42**.

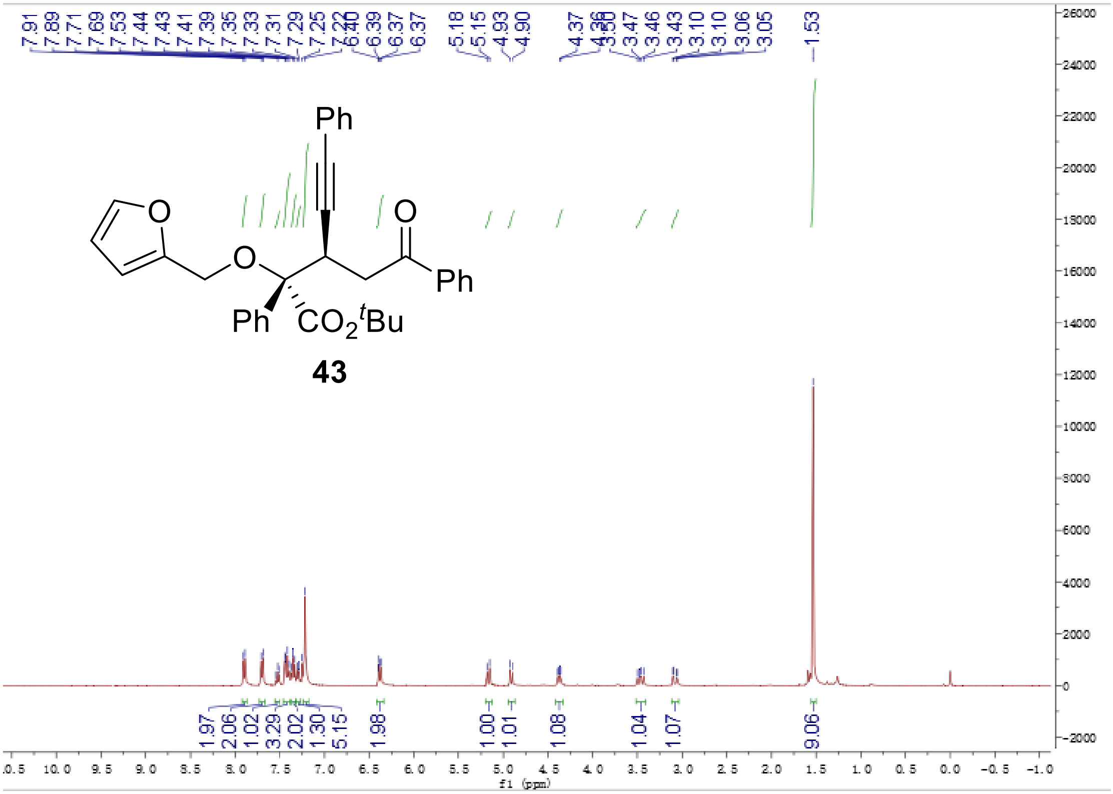

Supplementary Figure 361. ^{1}H NMR (400 MHz, $CDCl_3$) spectrum of **43**.

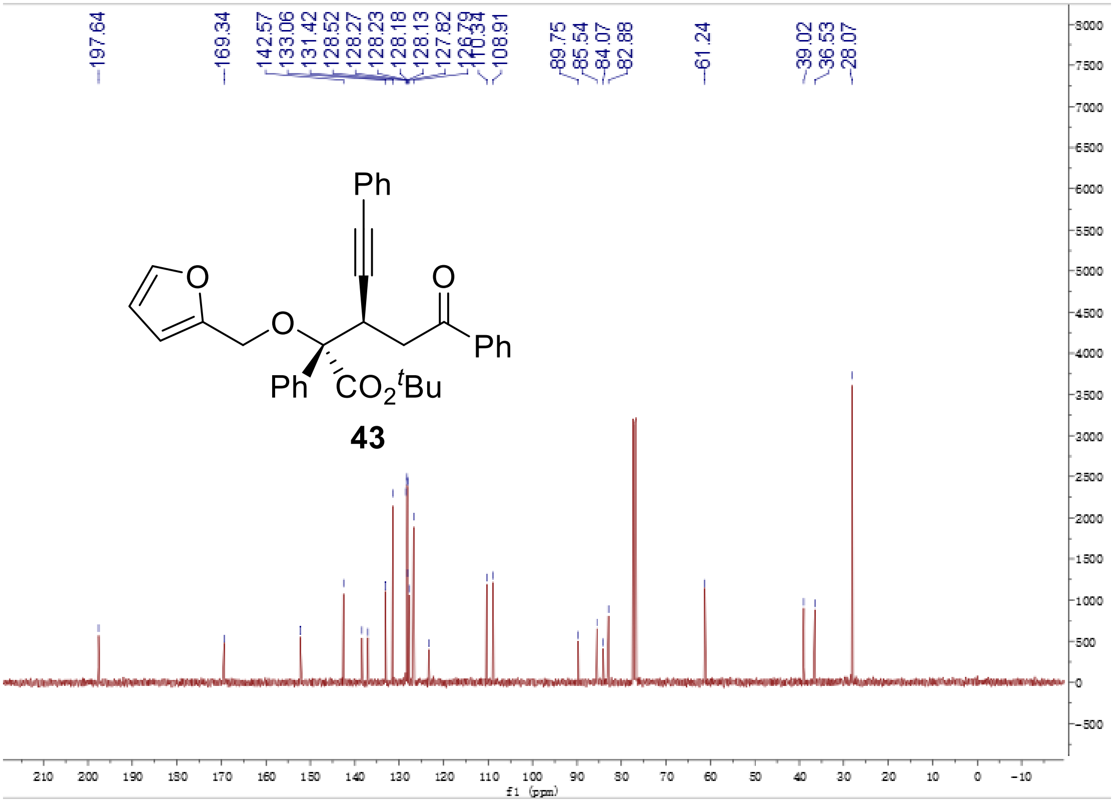

Supplementary Figure 362. ^{13}C NMR (101 MHz, $CDCl_3$) spectrum of **43**.

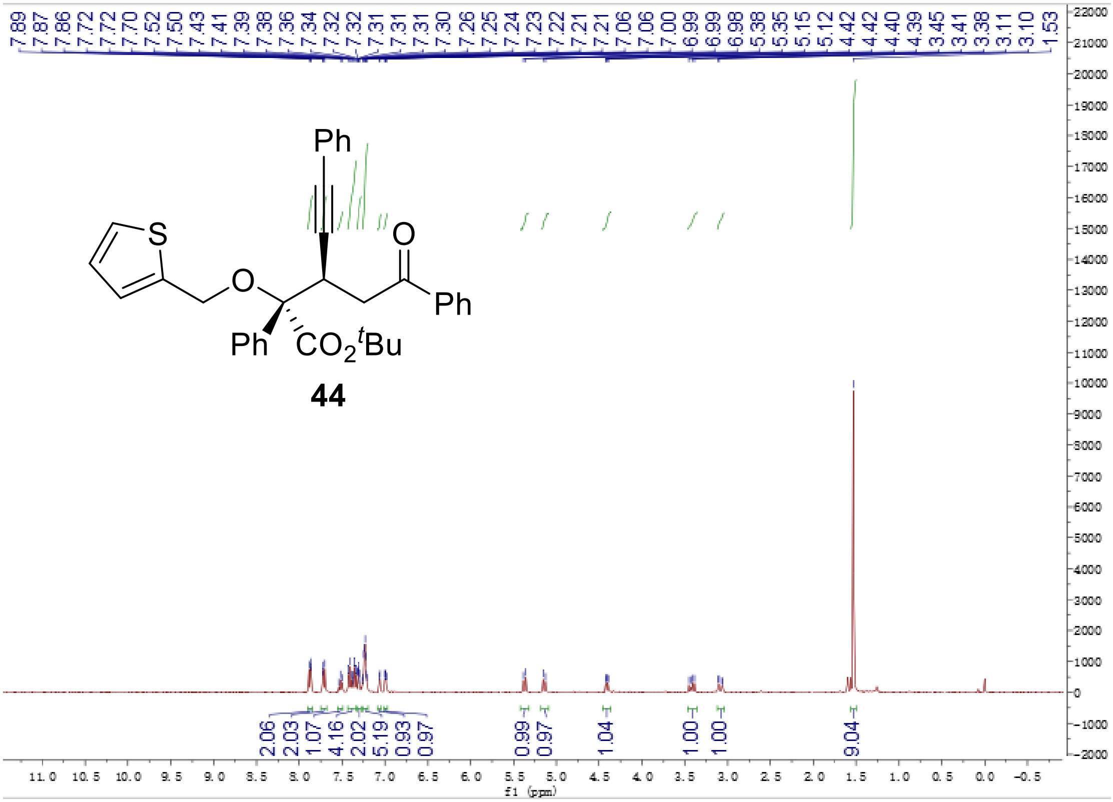

Supplementary Figure 363. ^{1}H NMR (400 MHz, $CDCl_3$) spectrum of **44**.

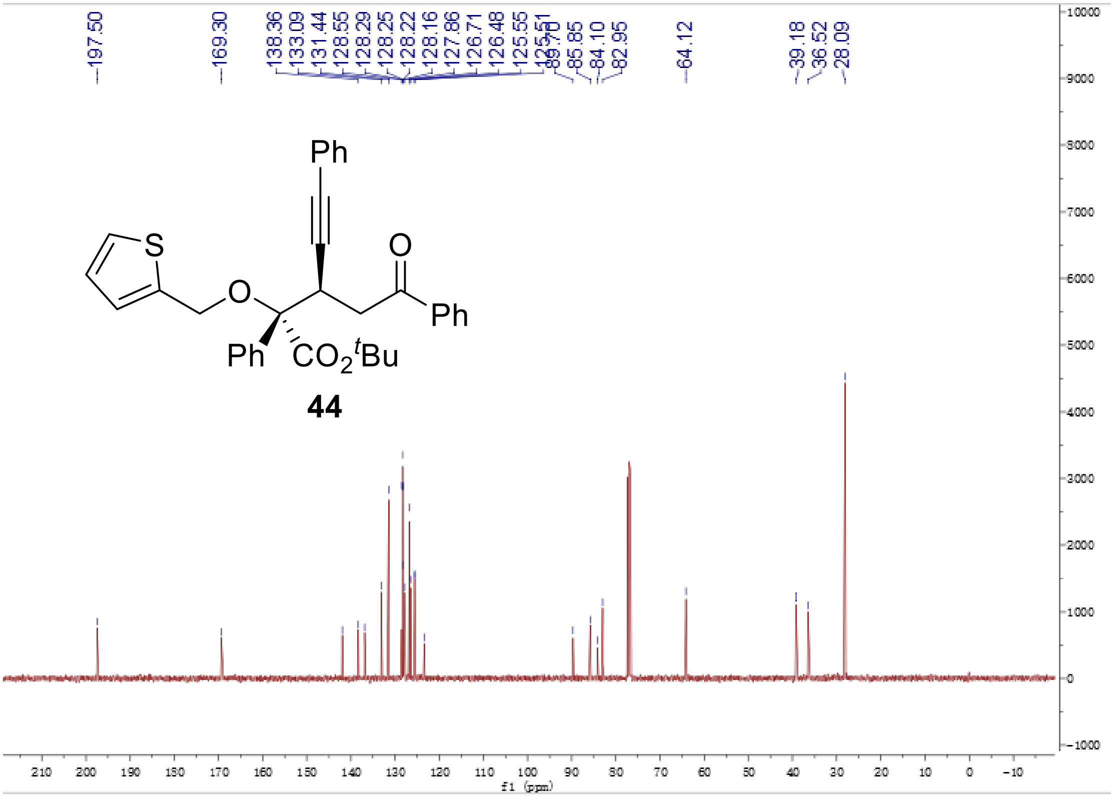

Supplementary Figure 364. ^{13}C NMR (101 MHz, $CDCl_3$) spectrum of **44**.

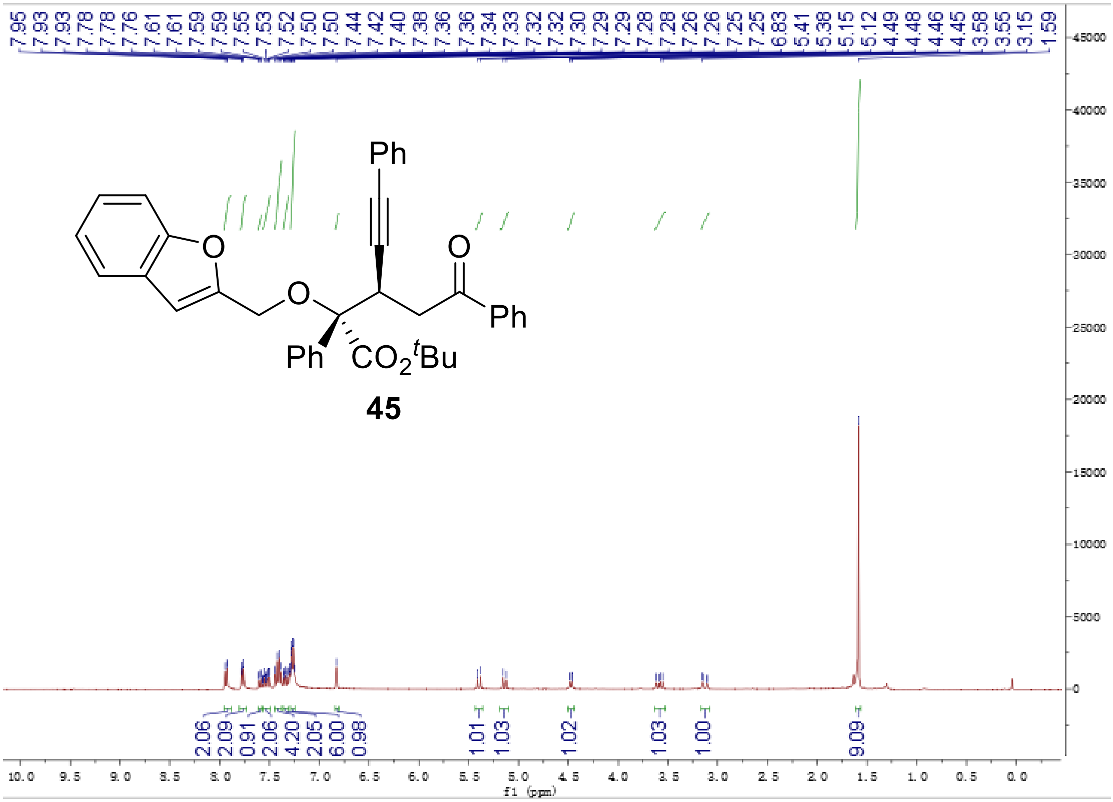

Supplementary Figure 365. ^{1}H NMR (400 MHz, $CDCl_3$) spectrum of **45**.

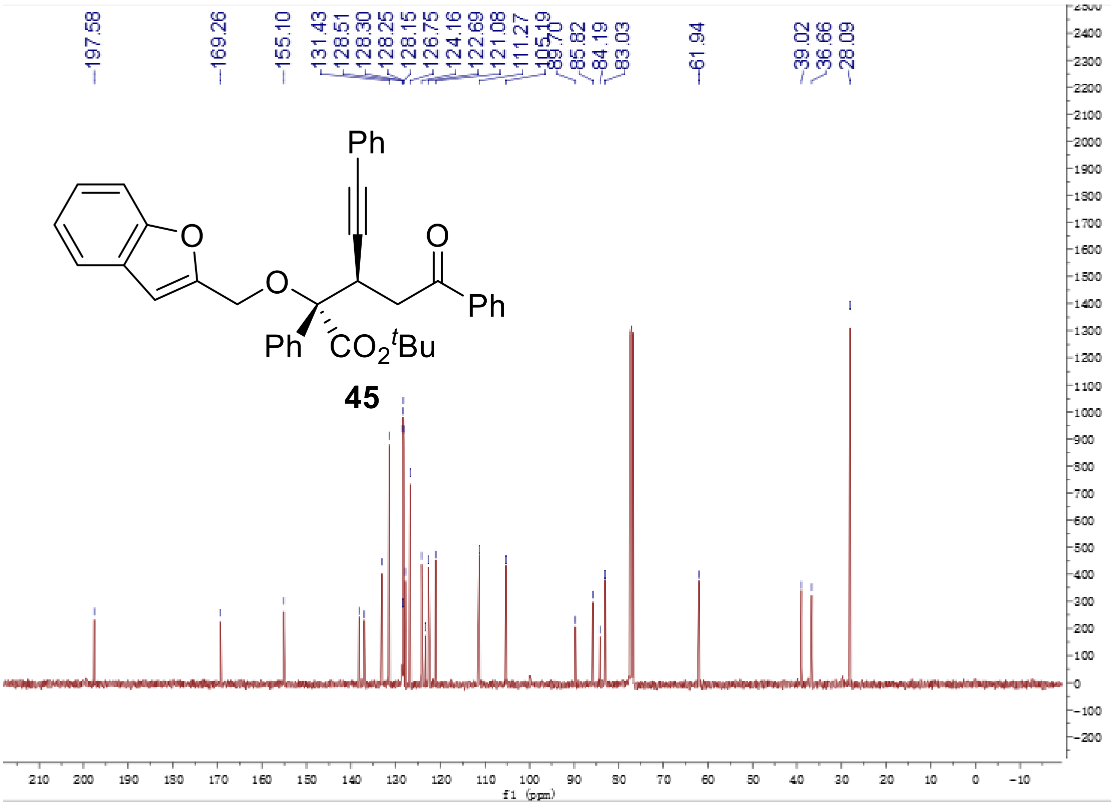

Supplementary Figure 366. ^{13}C NMR (101 MHz, $CDCl_3$) spectrum of **45**.

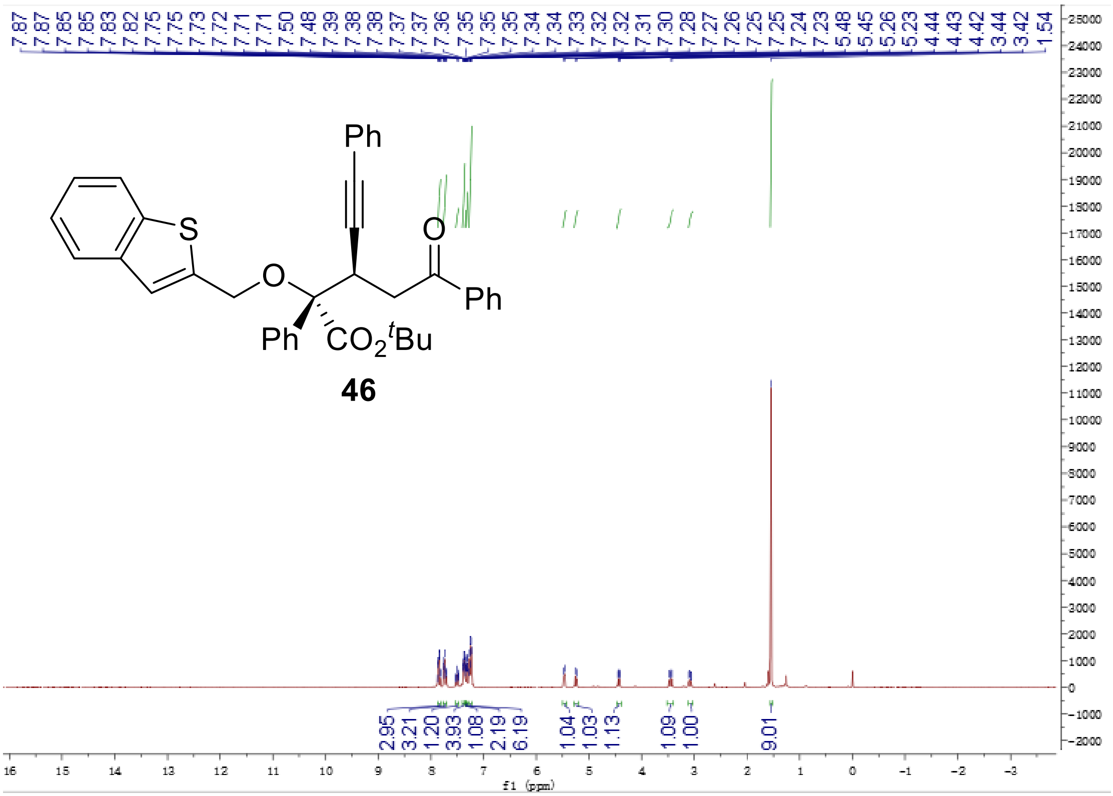

Supplementary Figure 367. ^{1}H NMR (400 MHz, $CDCl_3$) spectrum of **46**.

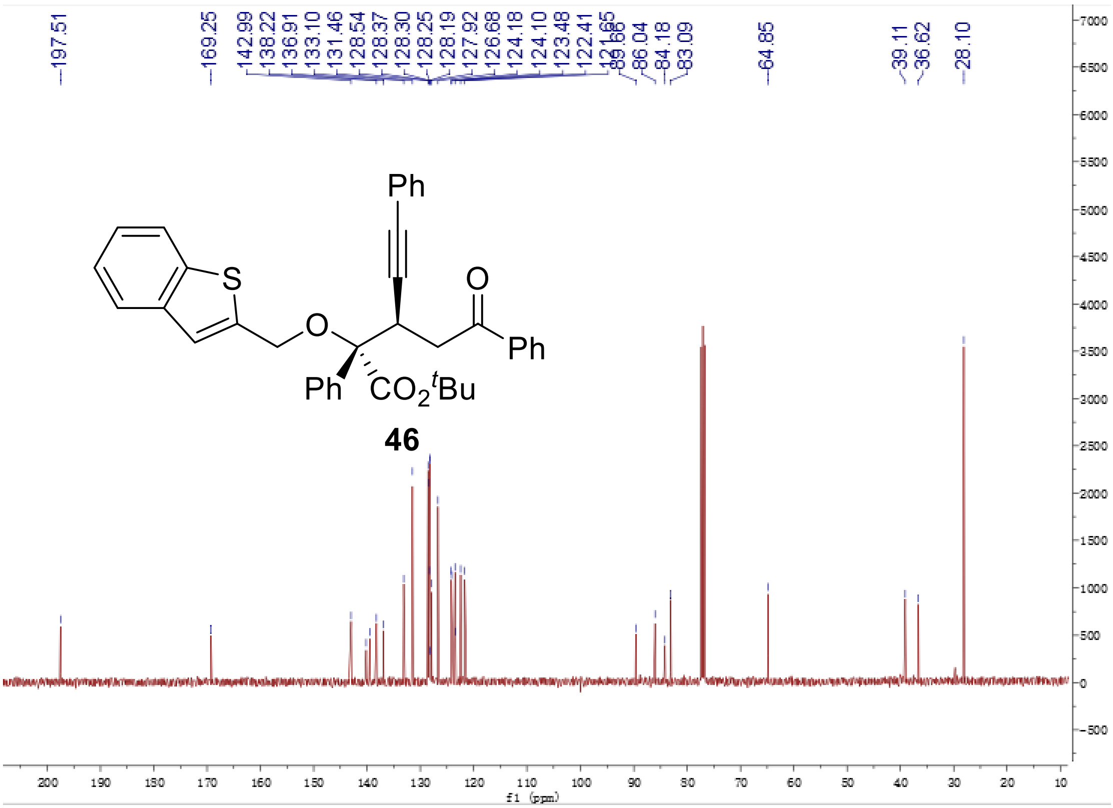

Supplementary Figure 368. ^{13}C NMR (101 MHz, $CDCl_3$) spectrum of **46**.

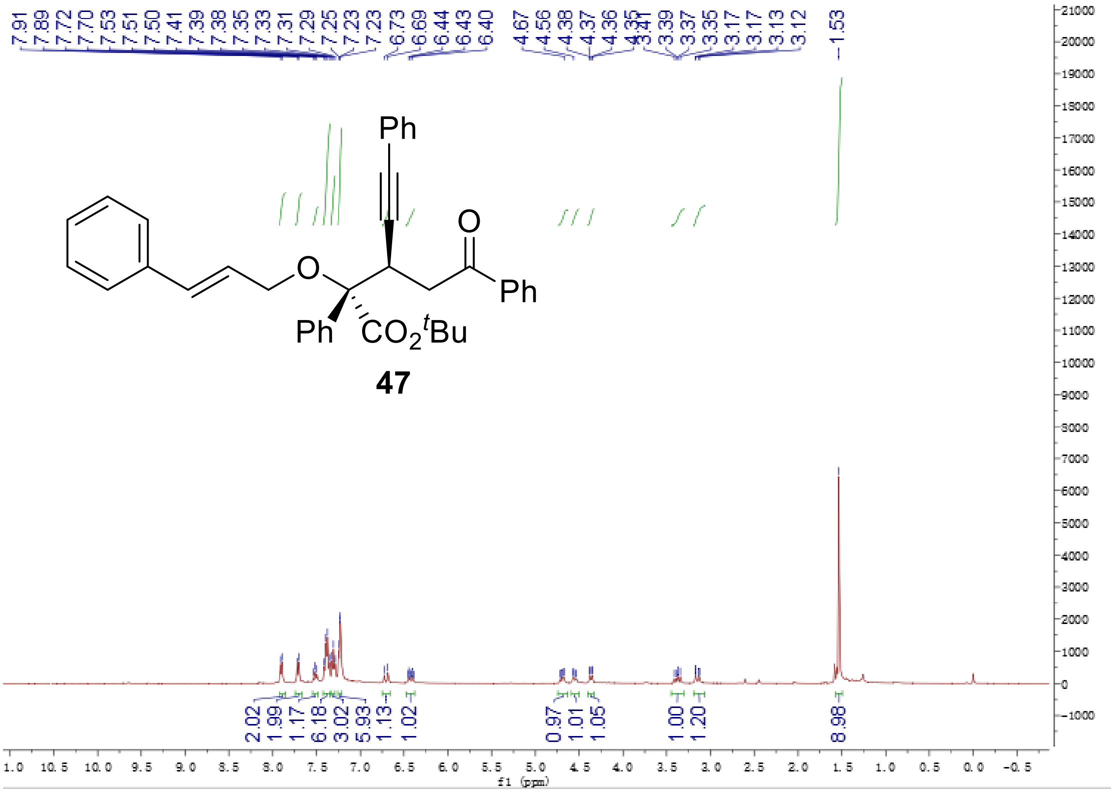

Supplementary Figure 369. ^{1}H NMR (400 MHz, $CDCl_3$) spectrum of **47**.

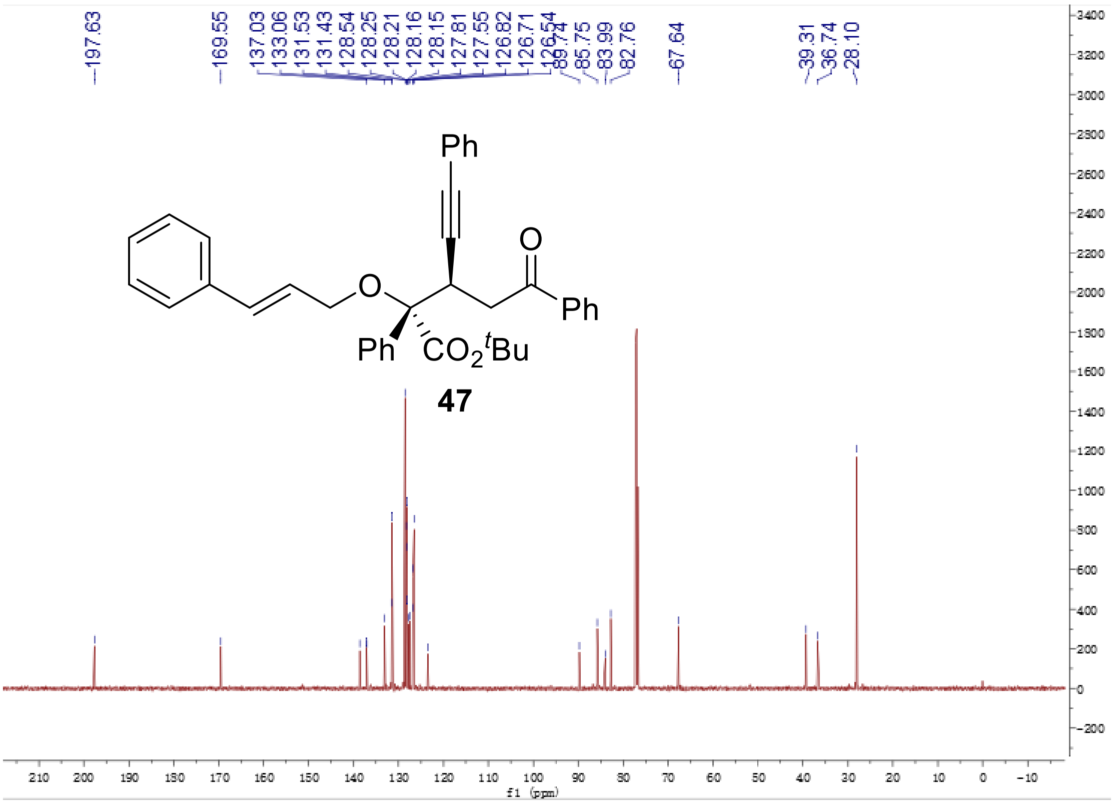

Supplementary Figure 370. ^{13}C NMR (101 MHz, $CDCl_3$) spectrum of **47**.

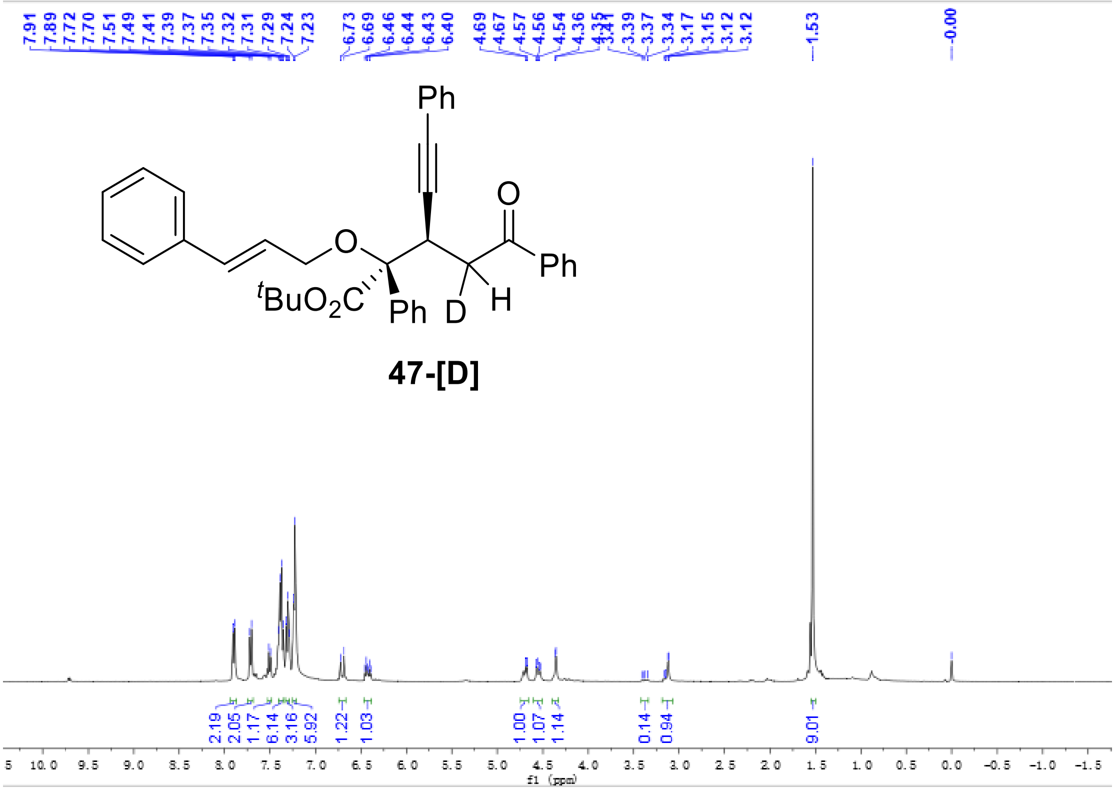

Supplementary Figure 371. ^{1}H NMR (400 MHz, $CDCl_3$) spectrum of **47-[D]**.

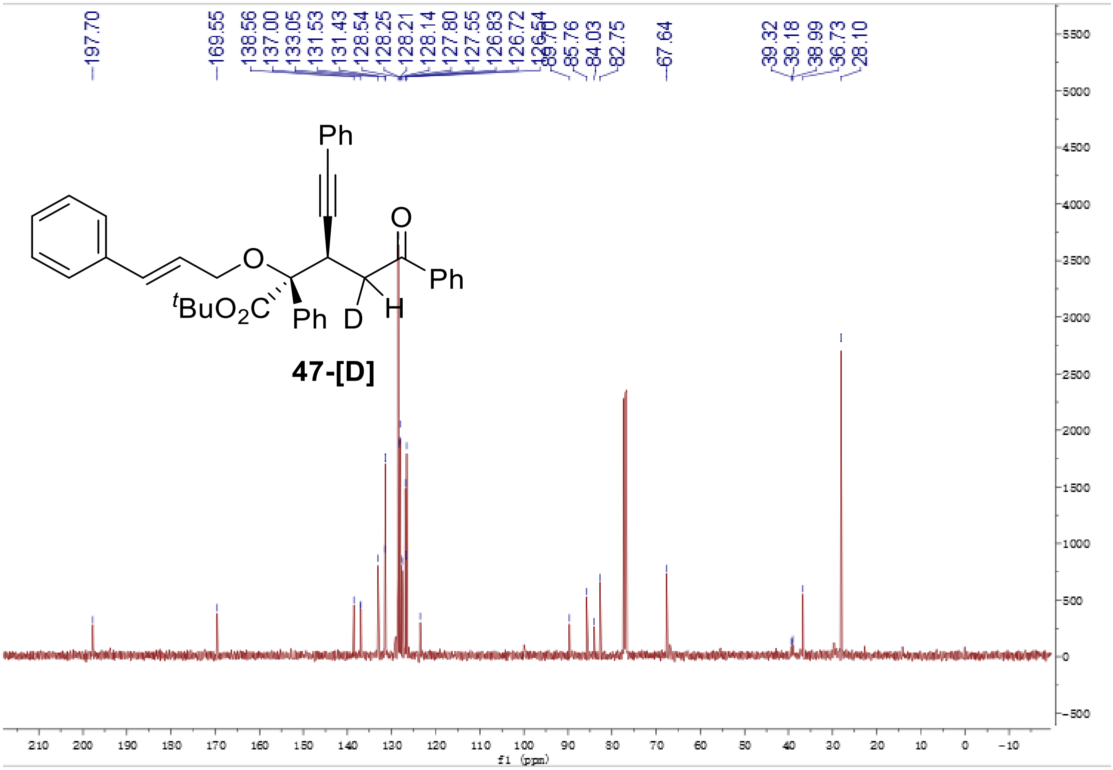

Supplementary Figure 372. ^{13}C NMR (101 MHz, $CDCl_3$) spectrum of **47-[D]**.

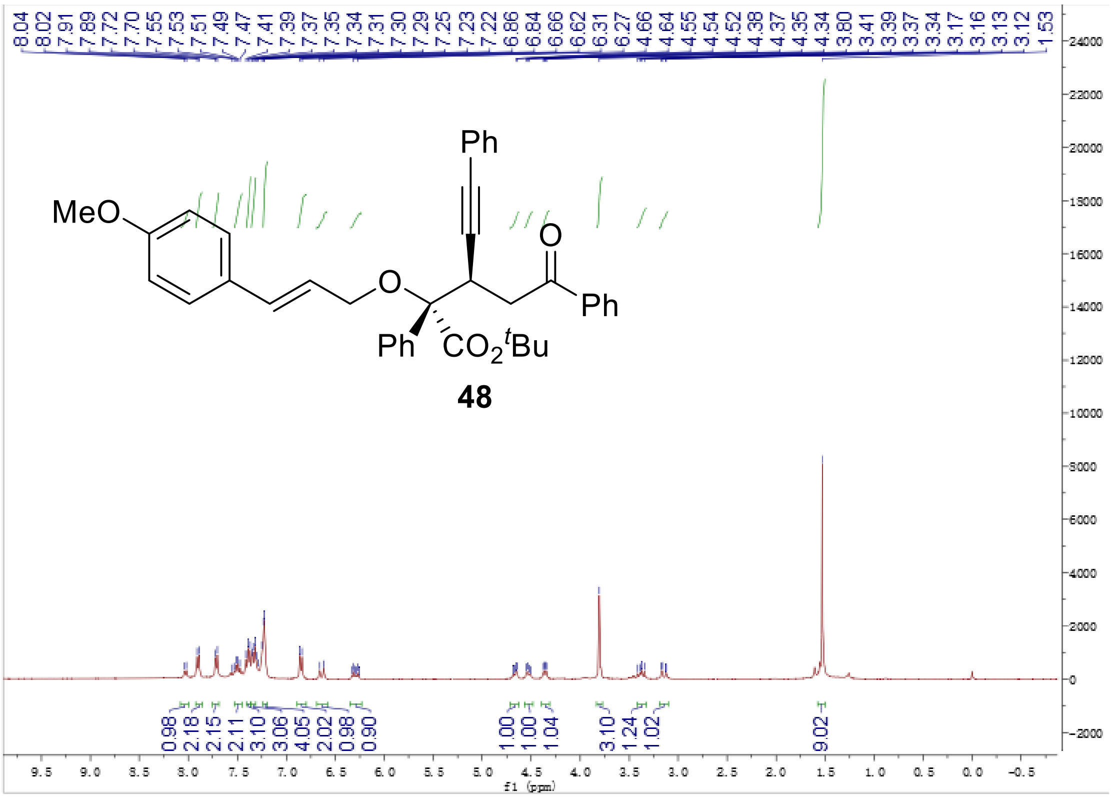

Supplementary Figure 373. ^{1}H NMR (400 MHz, $CDCl_3$) spectrum of **48**.

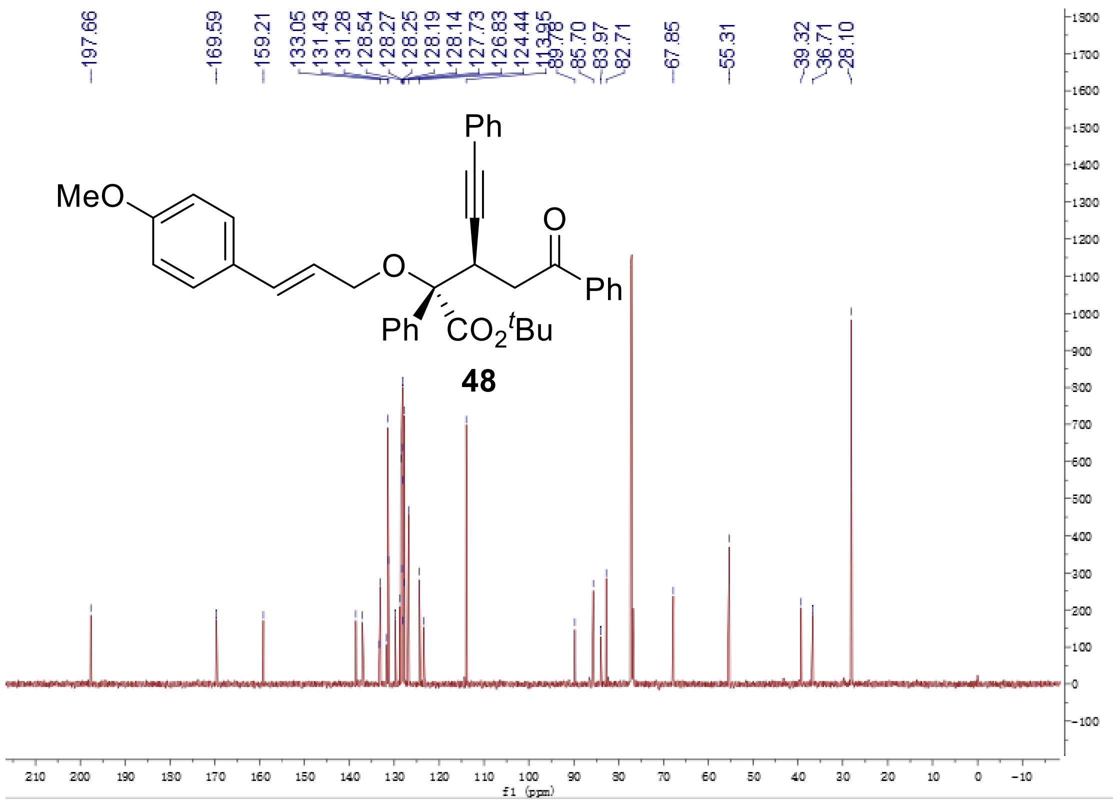

Supplementary Figure 374. ^{13}C NMR (101 MHz, $CDCl_3$) spectrum of **48**.

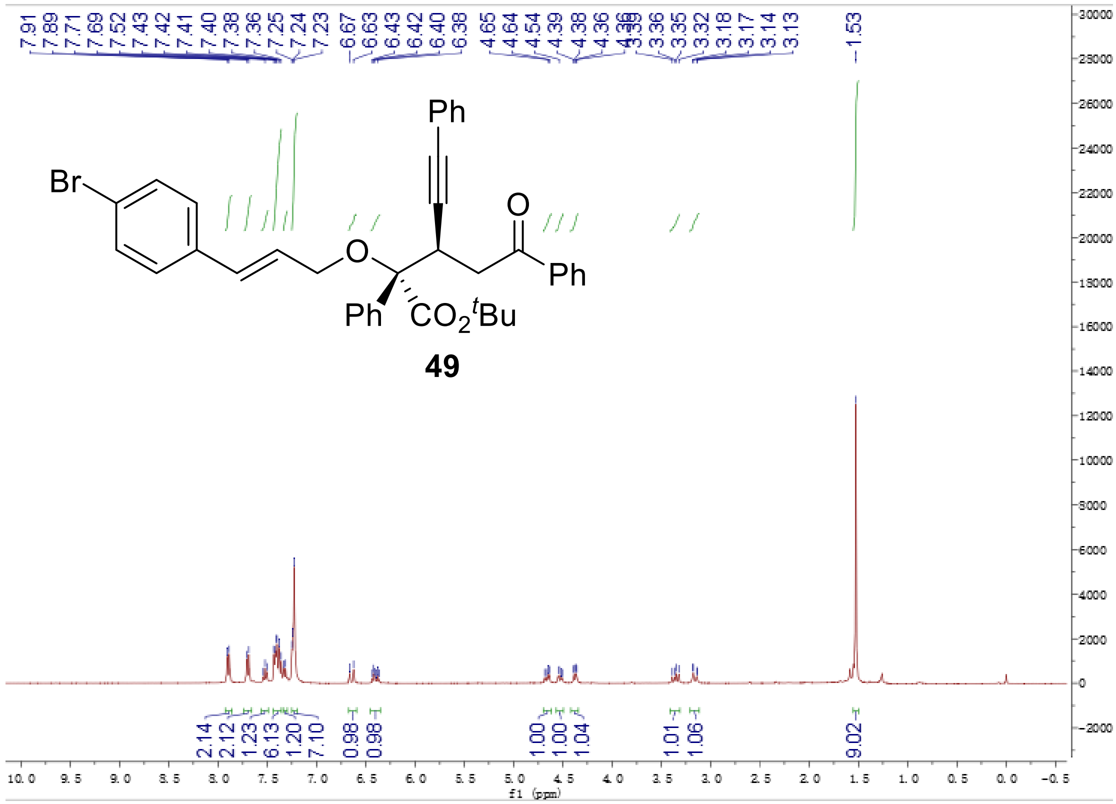

Supplementary Figure 375. ^{1}H NMR (400 MHz, $CDCl_3$) spectrum of **49**.

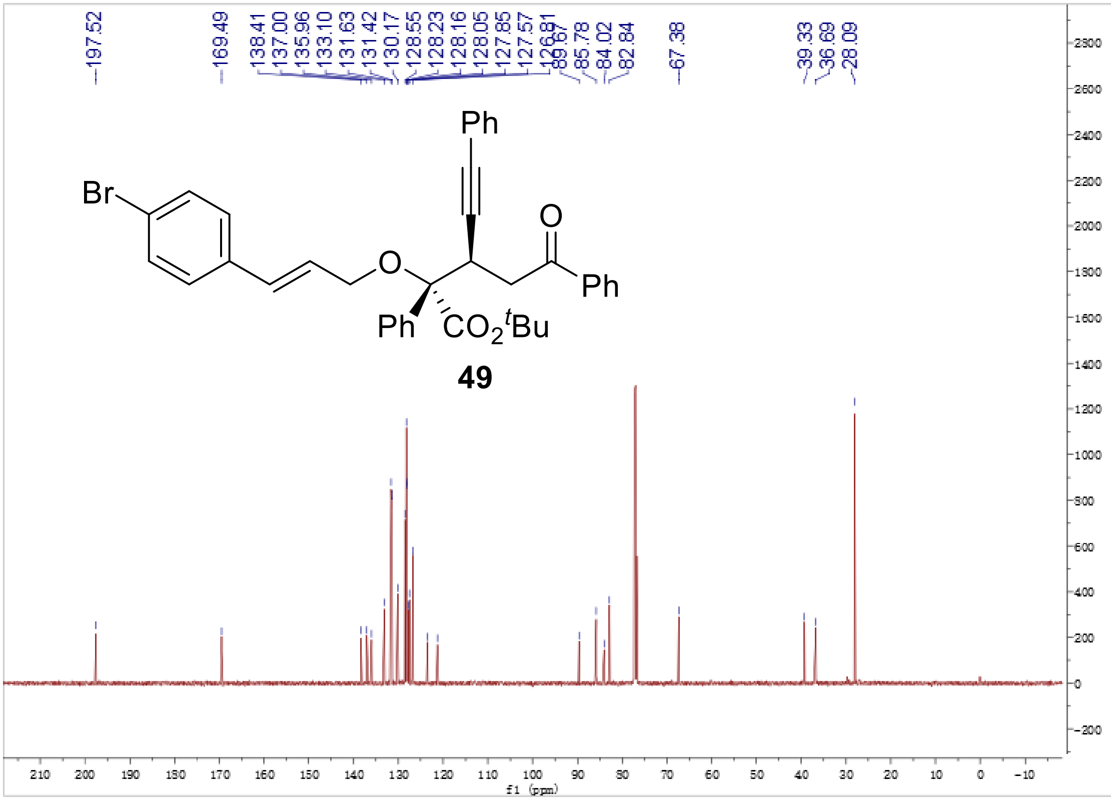

Supplementary Figure 376. ^{13}C NMR (101 MHz, $CDCl_3$) spectrum of **49**.

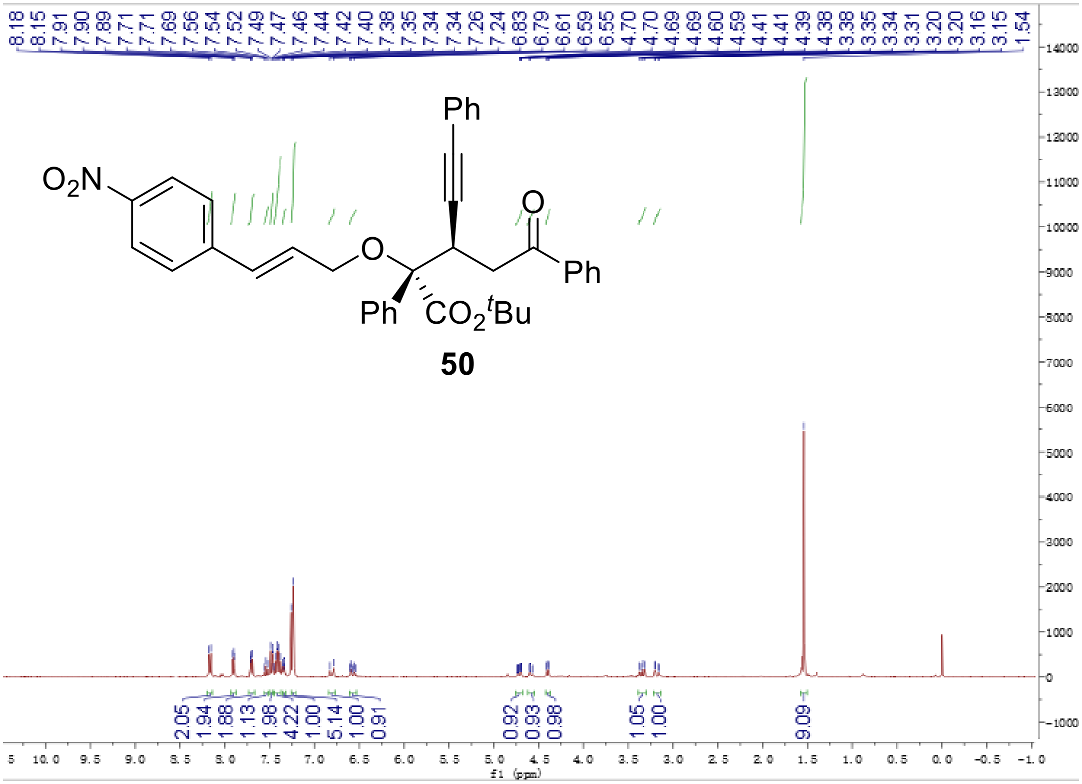

Supplementary Figure 377. ^{1}H NMR (400 MHz, $CDCl_3$) spectrum of **50**.

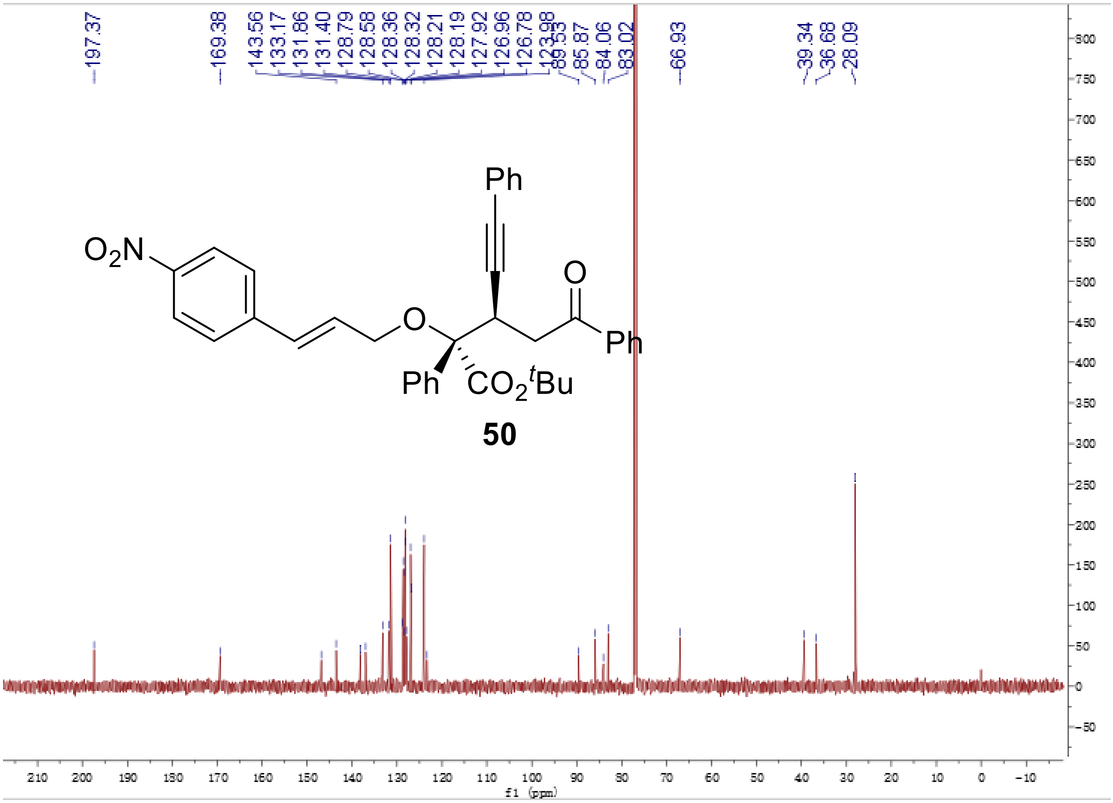

Supplementary Figure 378. ^{13}C NMR (101 MHz, $CDCl_3$) spectrum of **50**.

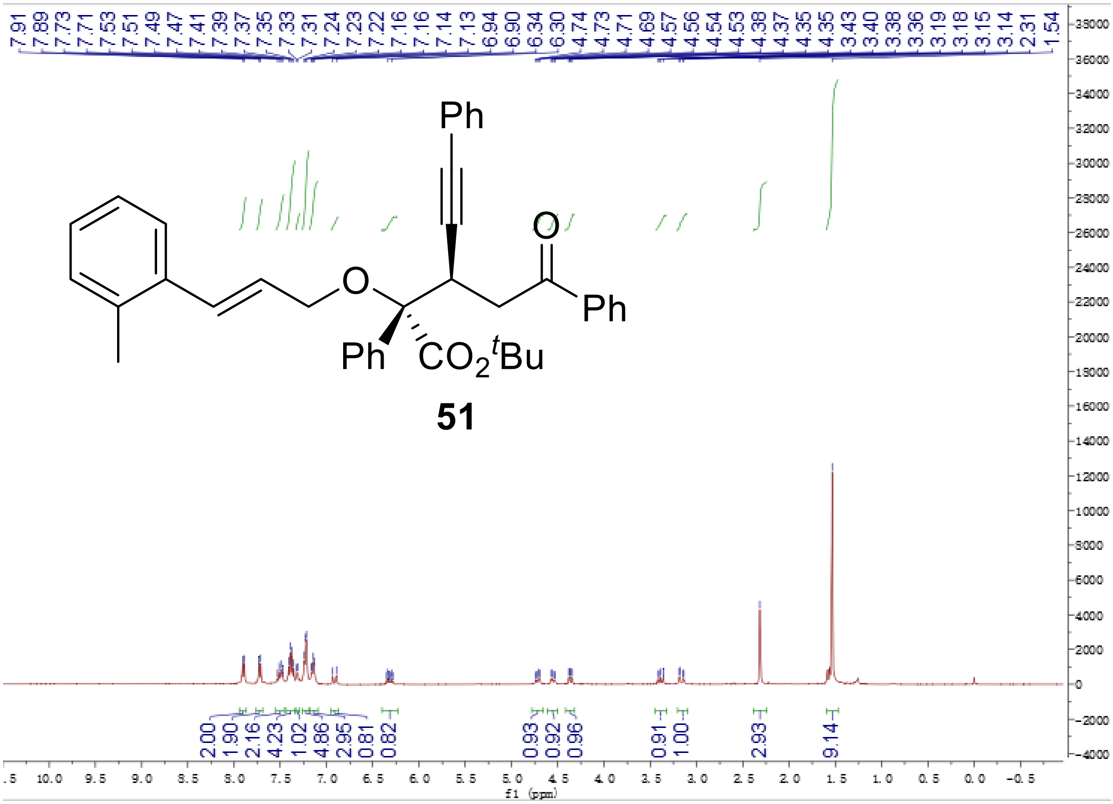

Supplementary Figure 379. ^{1}H NMR (400 MHz, $CDCl_3$) spectrum of **51**.

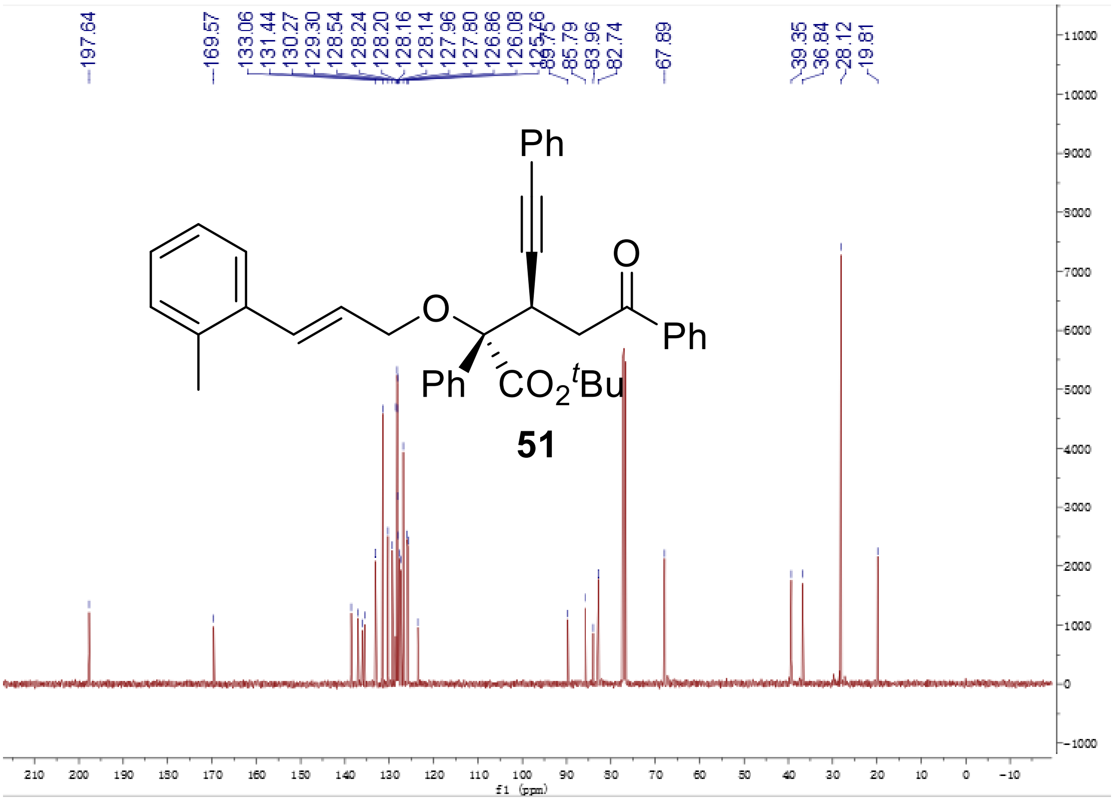

Supplementary Figure 380. ^{13}C NMR (101 MHz, $CDCl_3$) spectrum of **51**.

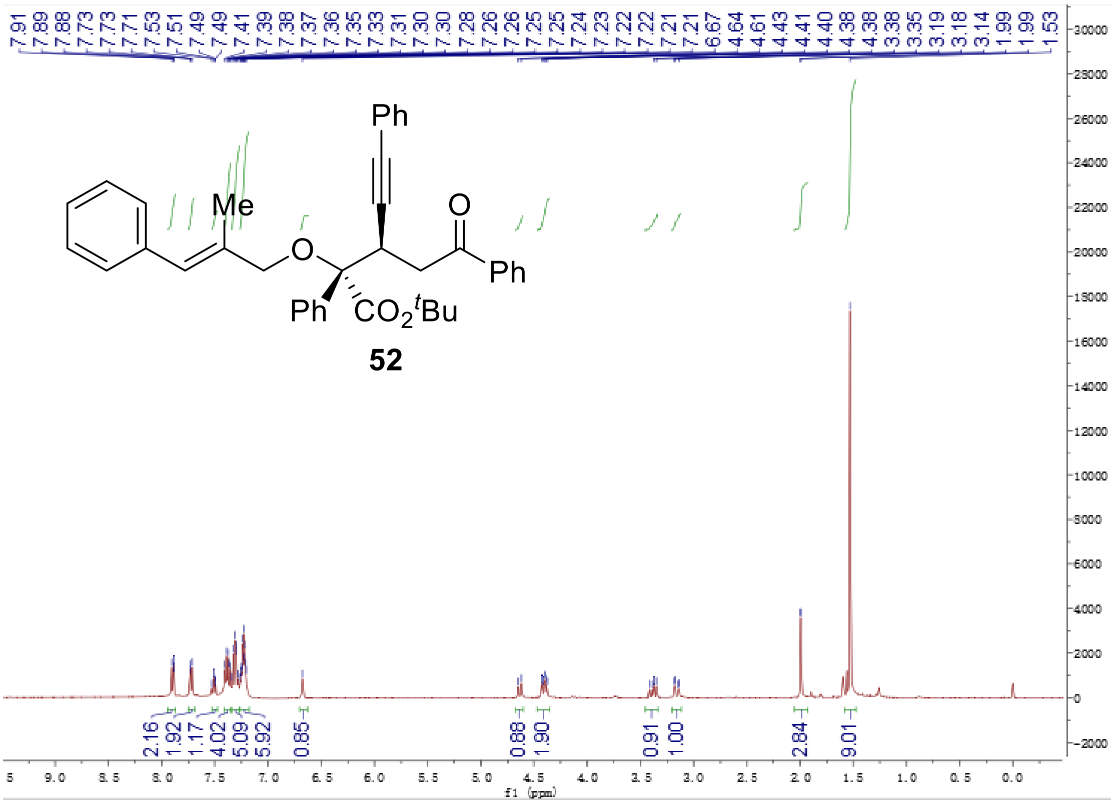

Supplementary Figure 381. ^{1}H NMR (400 MHz, $CDCl_3$) spectrum of **52**.

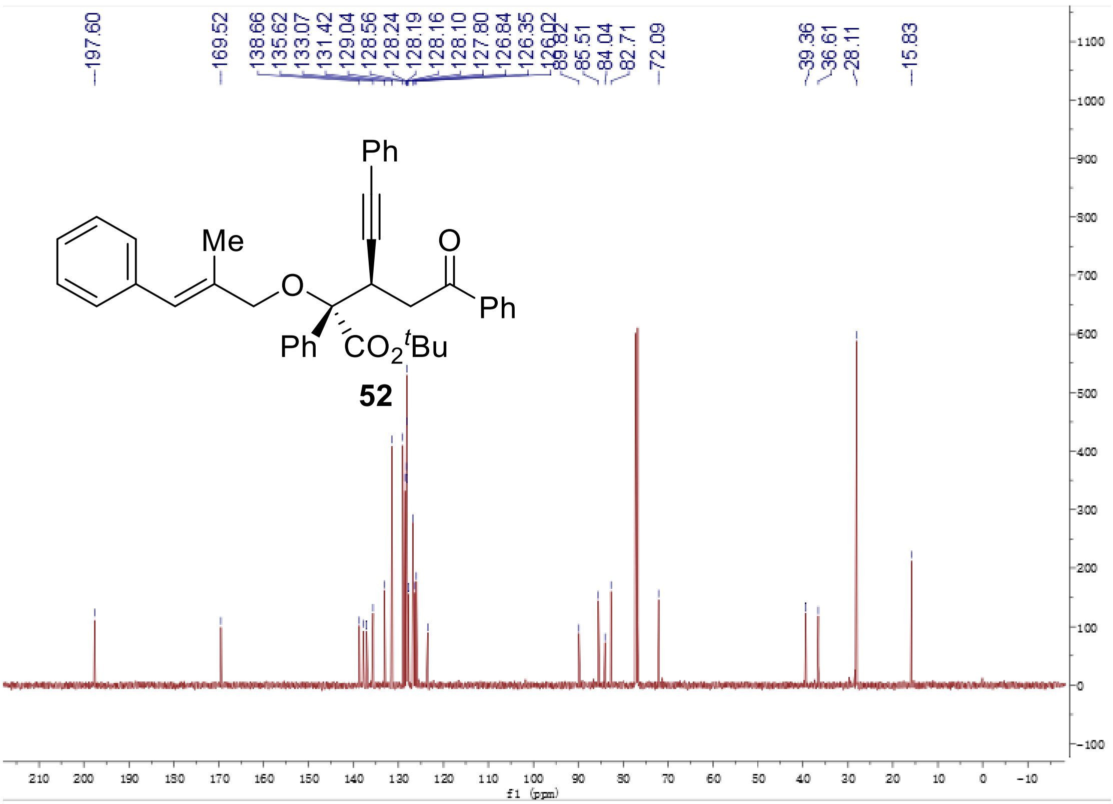

Supplementary Figure 382. ^{13}C NMR (101 MHz, $CDCl_3$) spectrum of **52**.

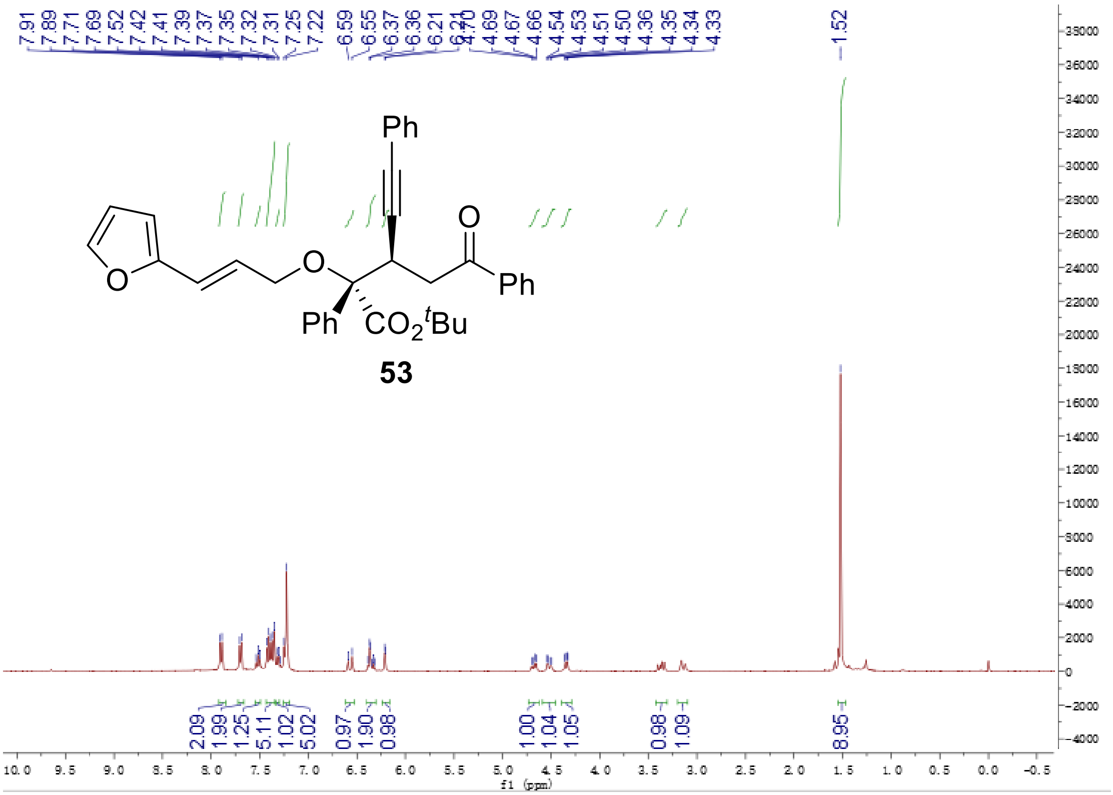

Supplementary Figure 383. ^{1}H NMR (400 MHz, $CDCl_3$) spectrum of **53**.

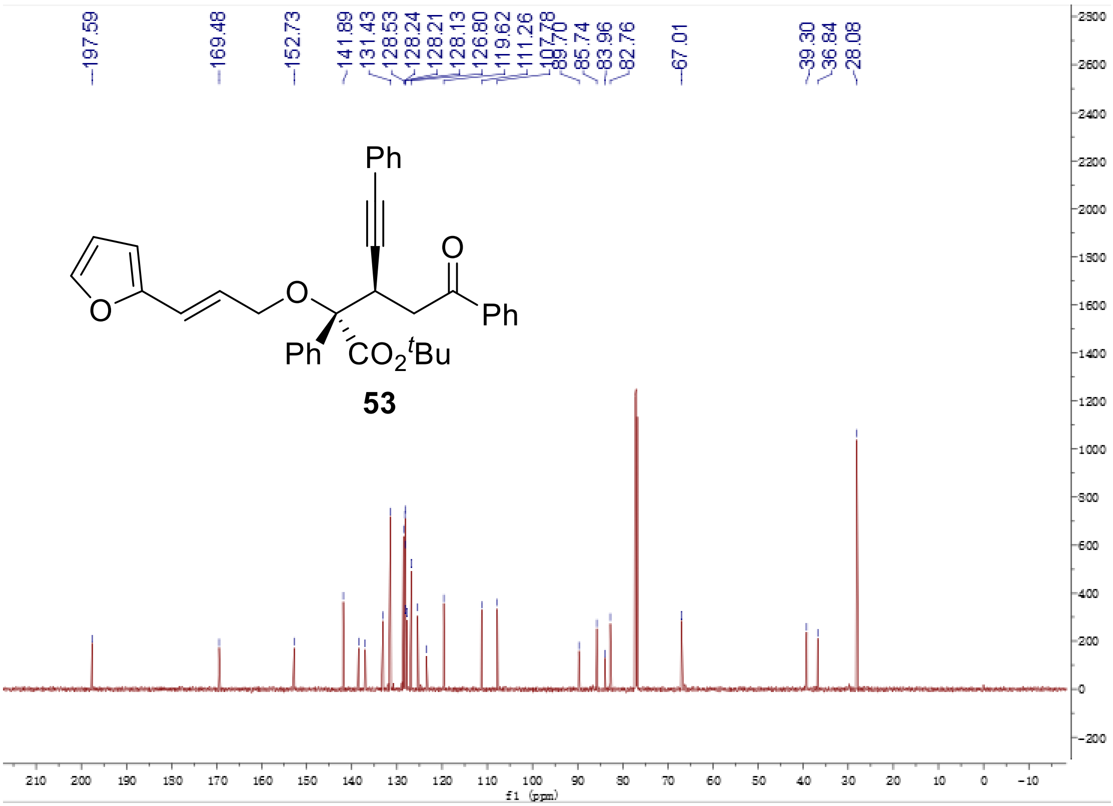

Supplementary Figure 384. ^{13}C NMR (101 MHz, $CDCl_3$) spectrum of **53**.

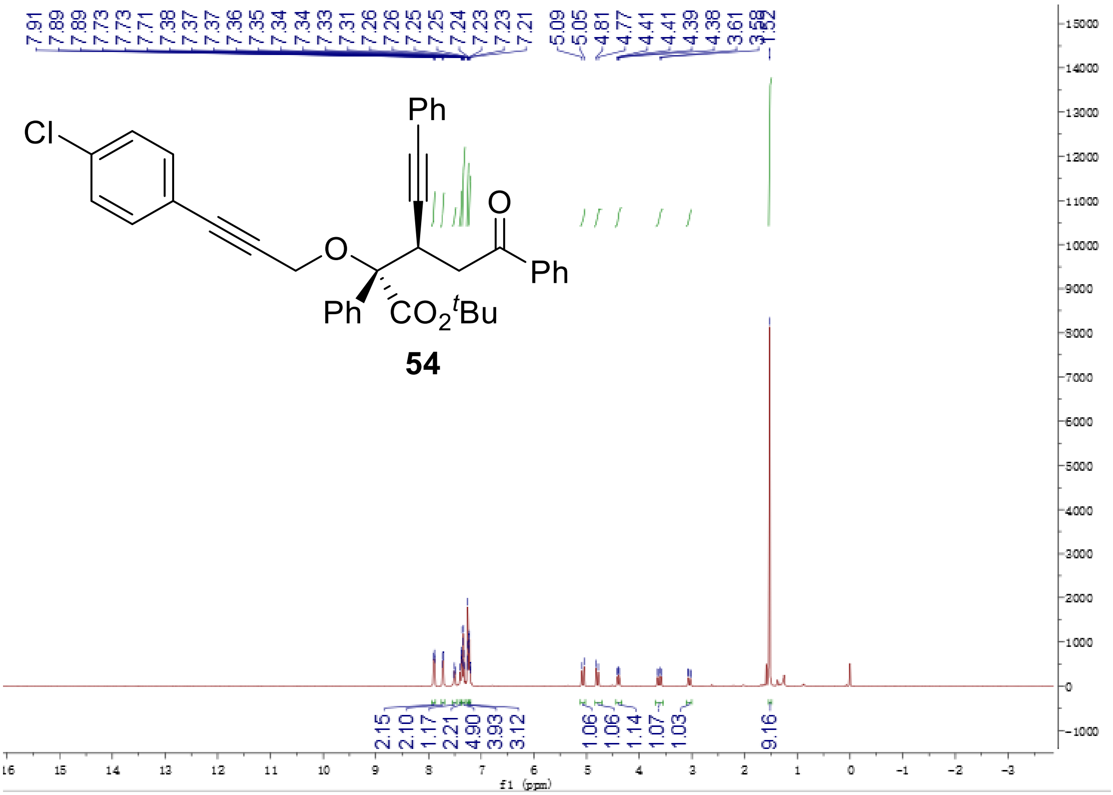

Supplementary Figure 385. ^{1}H NMR (400 MHz, $CDCl_3$) spectrum of **54**.

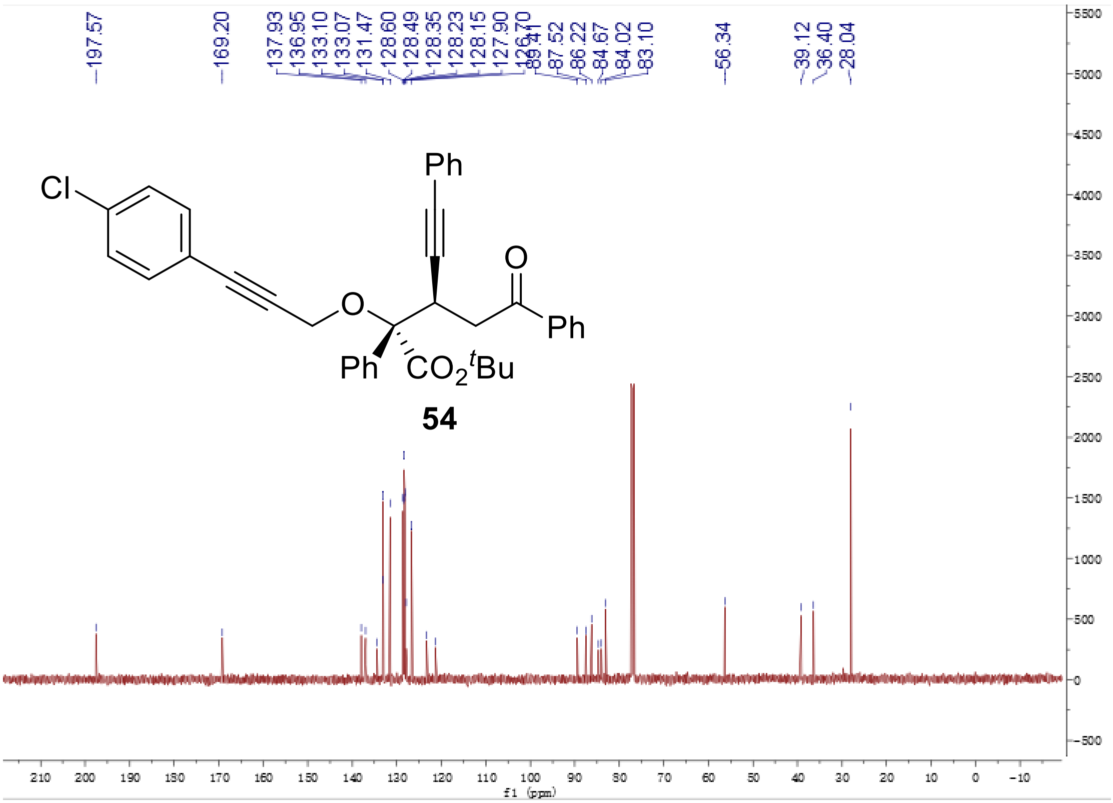

Supplementary Figure 386. ^{13}C NMR (101 MHz, CDCl$_3$) spectrum of **54**.

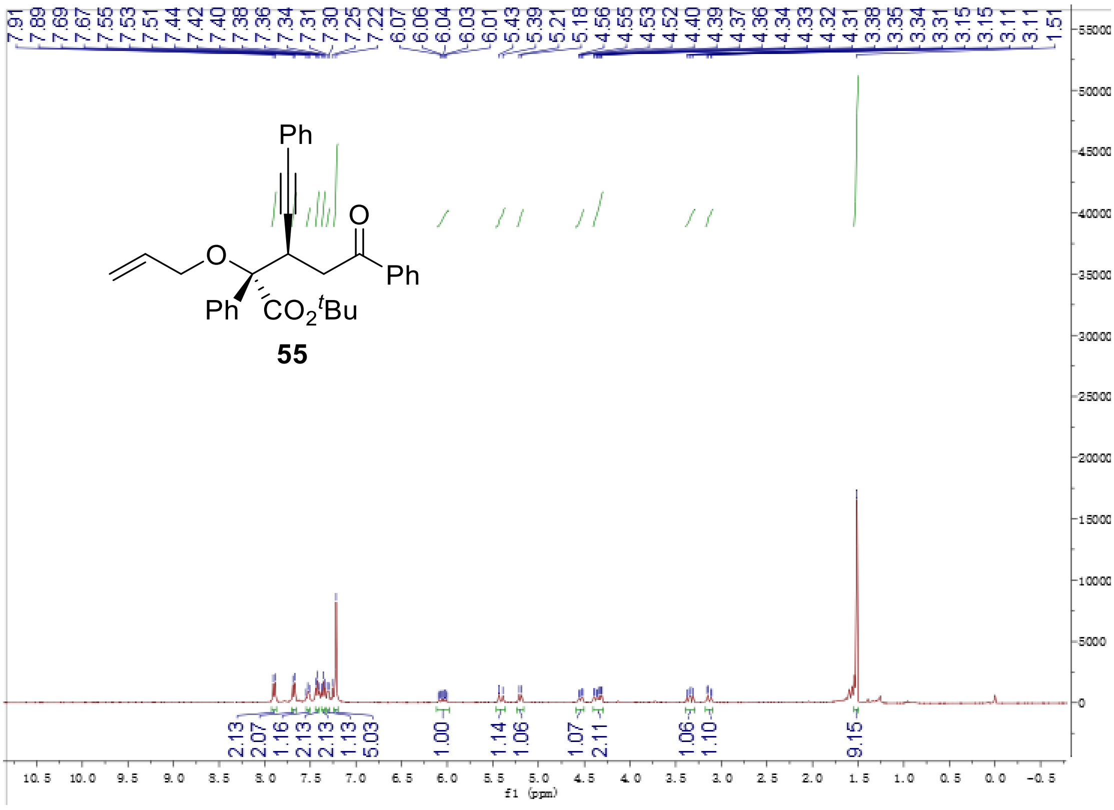

Supplementary Figure 387. ^{1}H NMR (400 MHz, CDCl$_3$) spectrum of **55**.

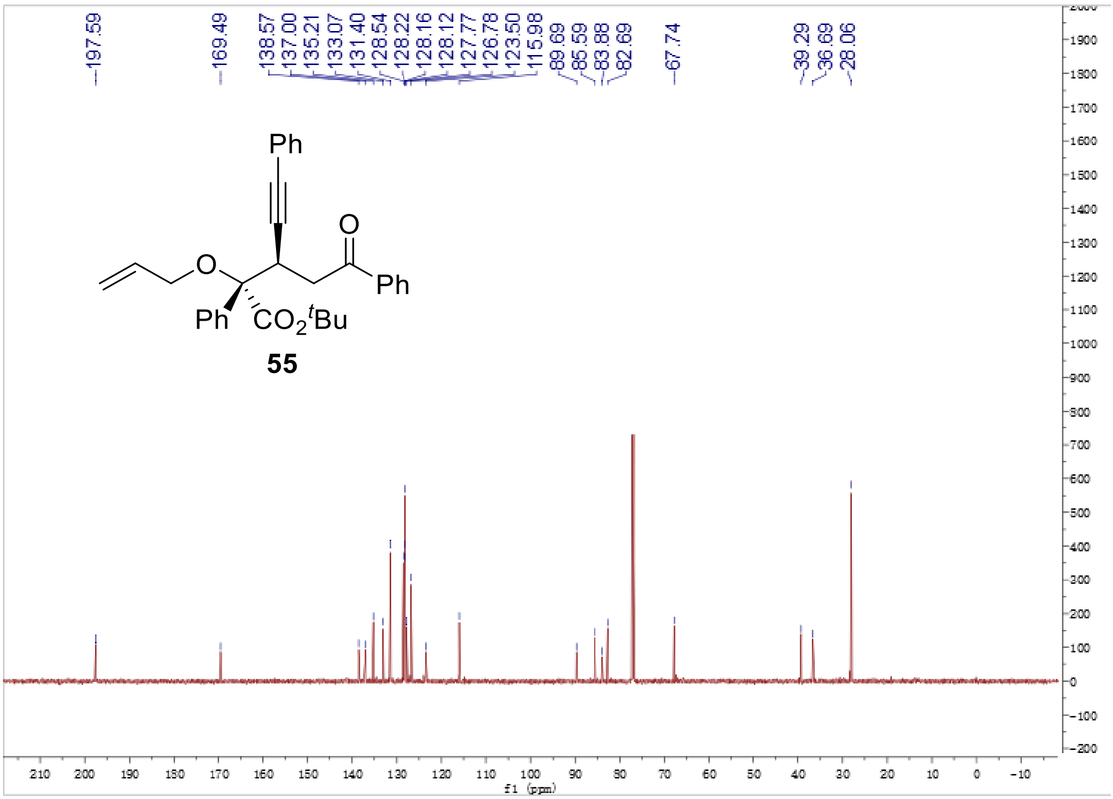

Supplementary Figure 388. ^{13}C NMR (101 MHz, $CDCl_3$) spectrum of **55**.

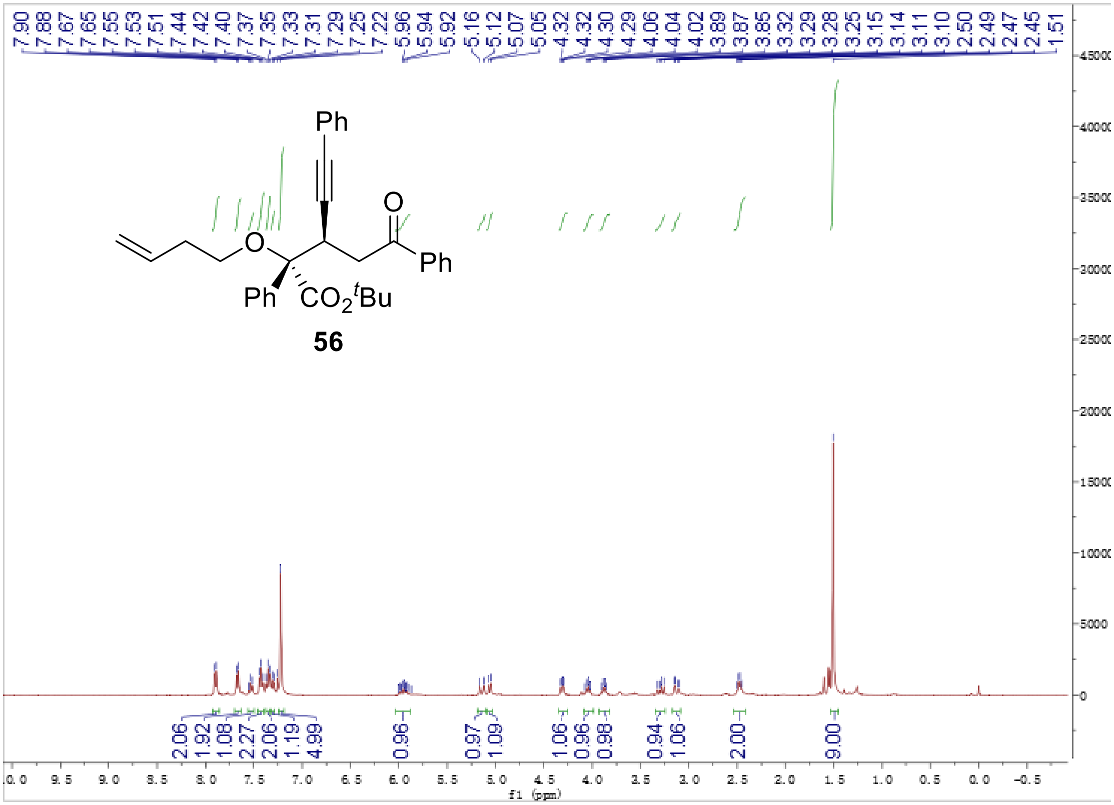

Supplementary Figure 389. ^{1}H NMR (400 MHz, $CDCl_3$) spectrum of **56**.

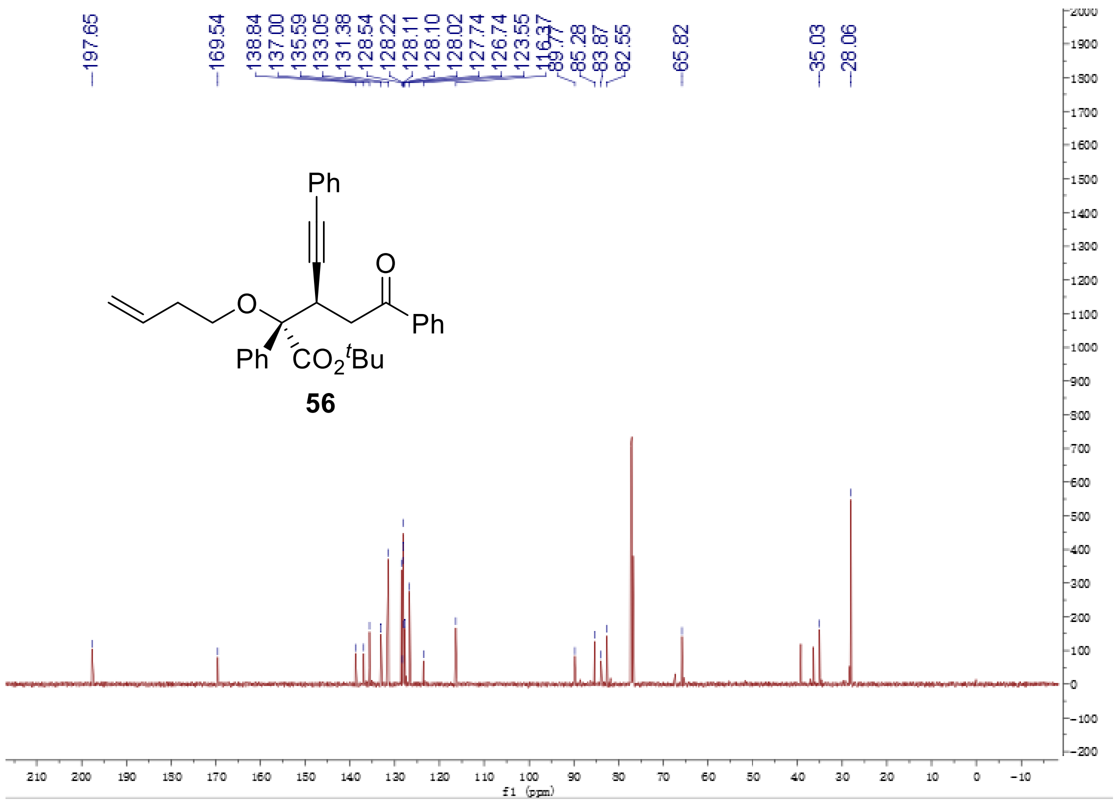

Supplementary Figure 390. ^{13}C NMR (101 MHz, $CDCl_3$) spectrum of **56**.

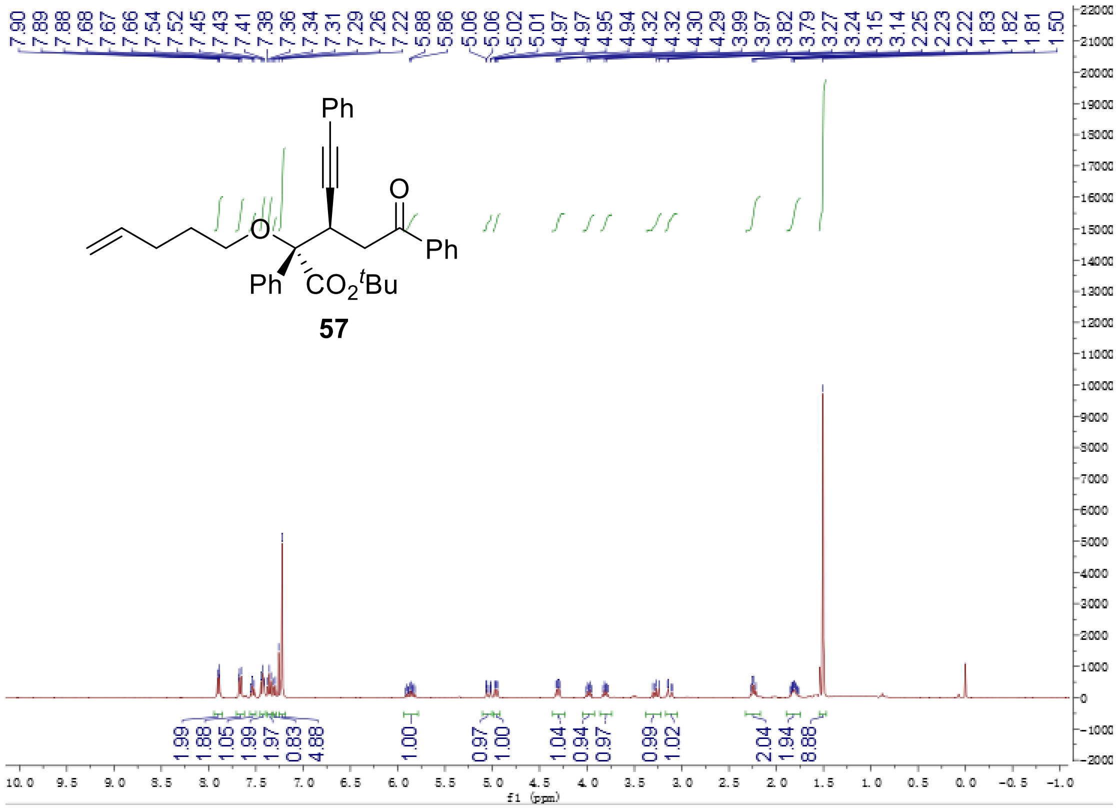

Supplementary Figure 391. ^{1}H NMR (400 MHz, $CDCl_3$) spectrum of **57**.

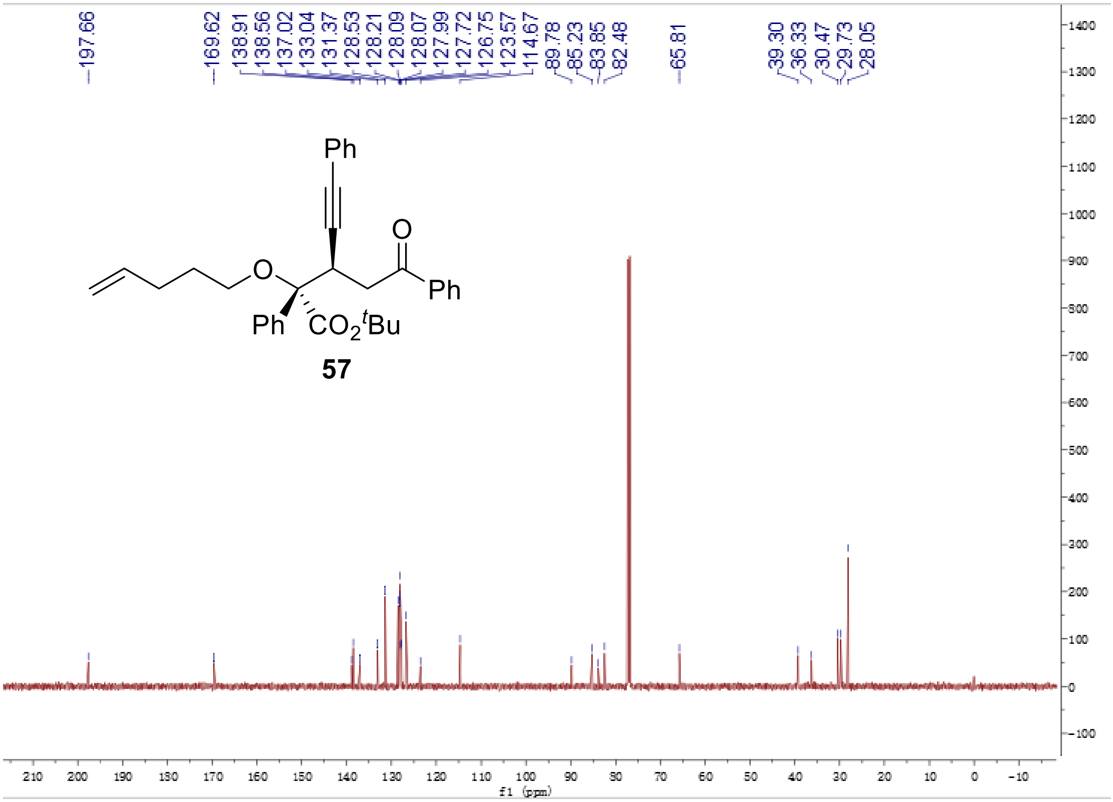

Supplementary Figure 392. ^{13}C NMR (101 MHz, $CDCl_3$) spectrum of **57**.

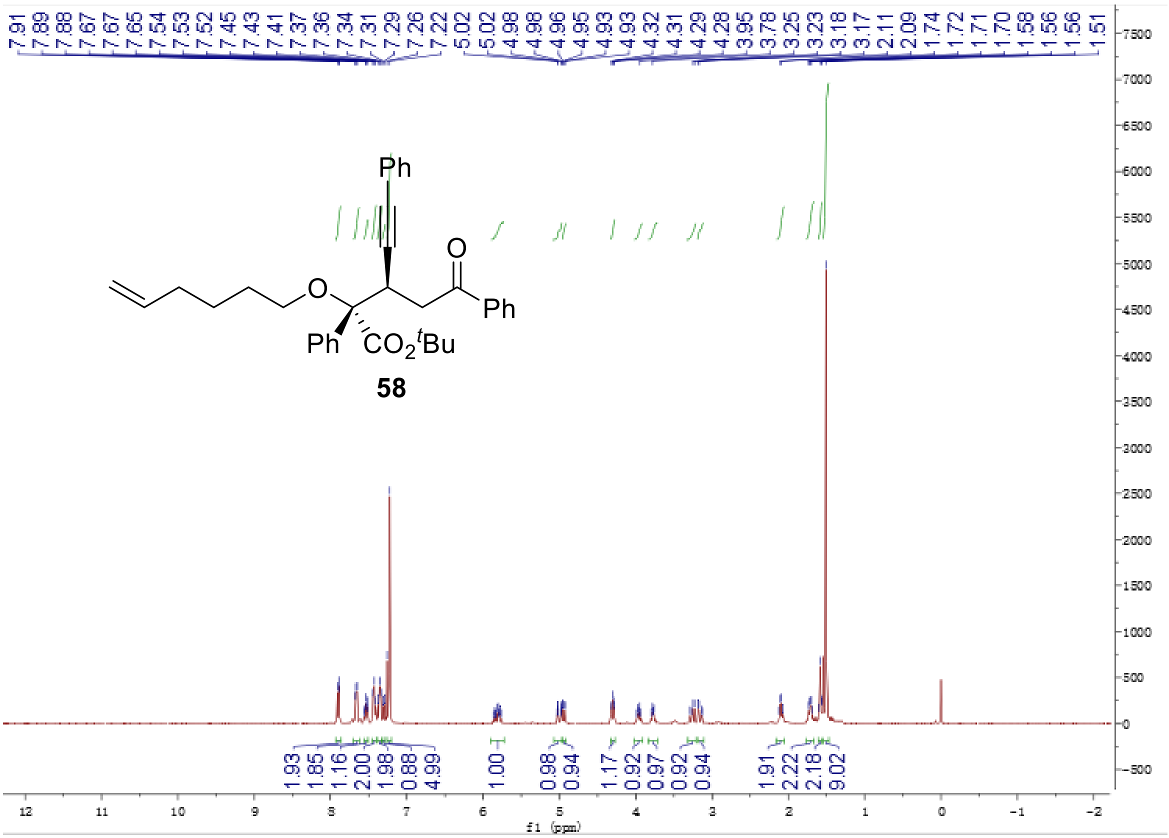

Supplementary Figure 393. ^{1}H NMR (400 MHz, $CDCl_3$) spectrum of **58**.

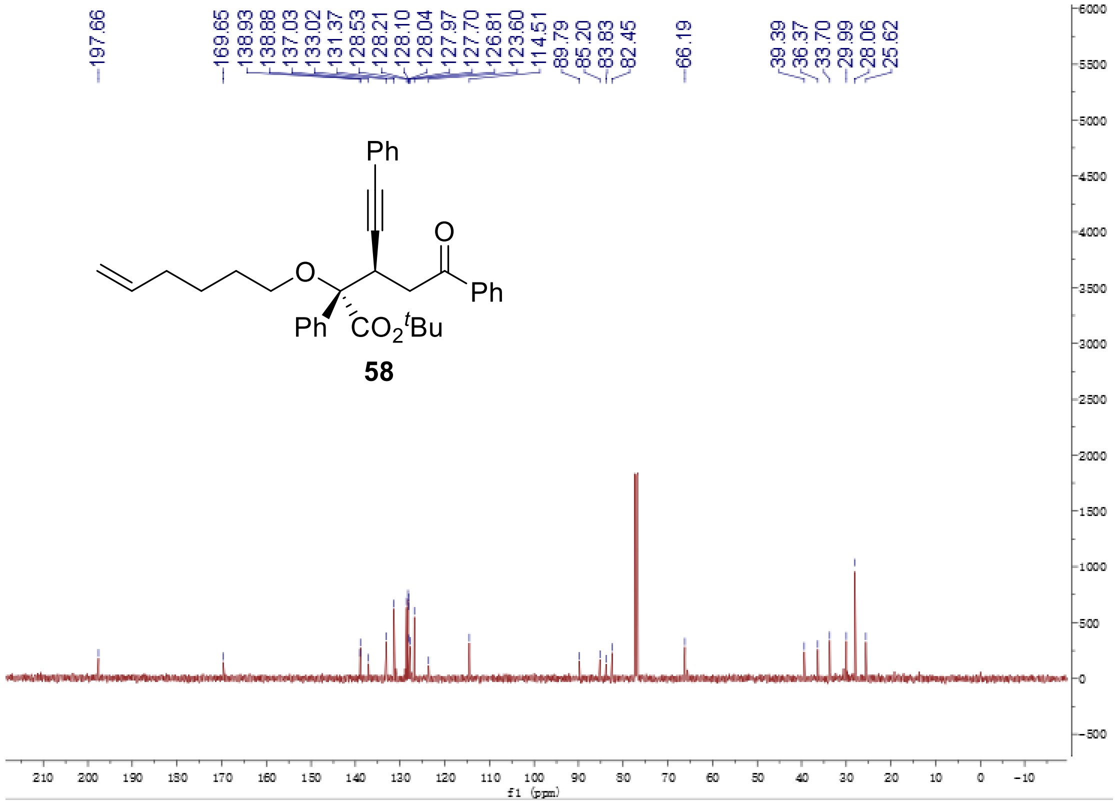

Supplementary Figure 394. ^{13}C NMR (101 MHz, $CDCl_3$) spectrum of **58**.

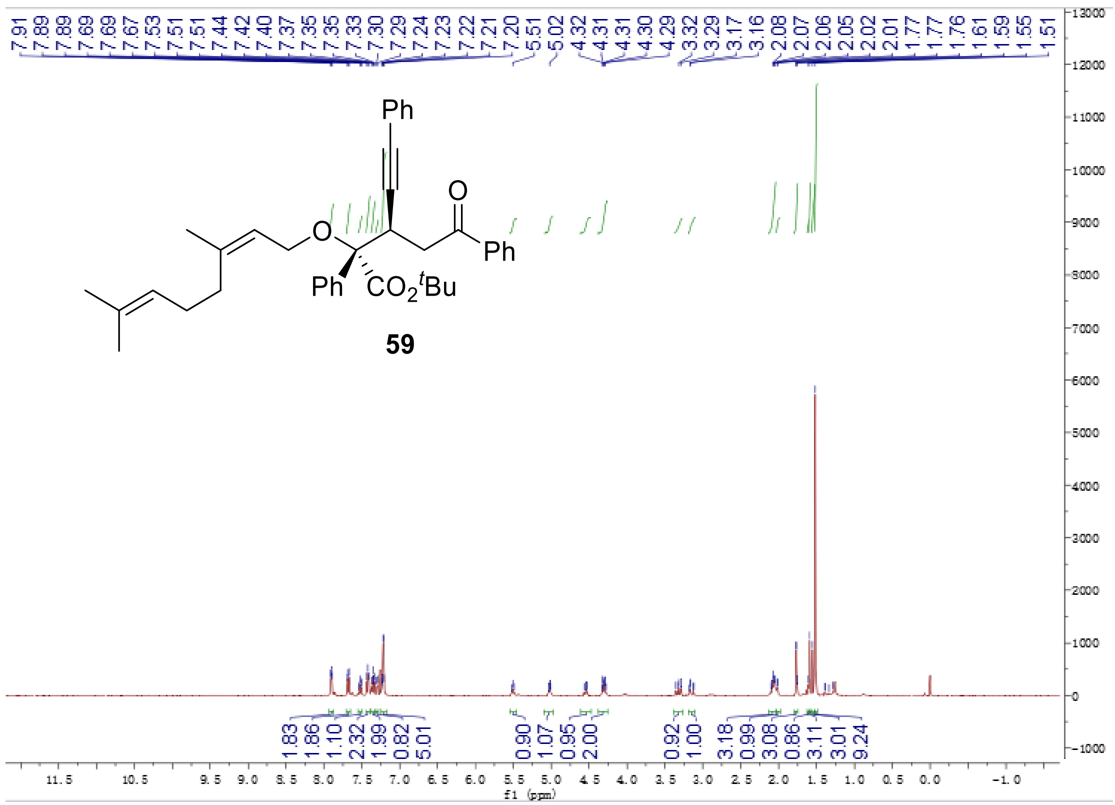

Supplementary Figure 395. ^{1}H NMR (400 MHz, $CDCl_3$) spectrum of **59**.

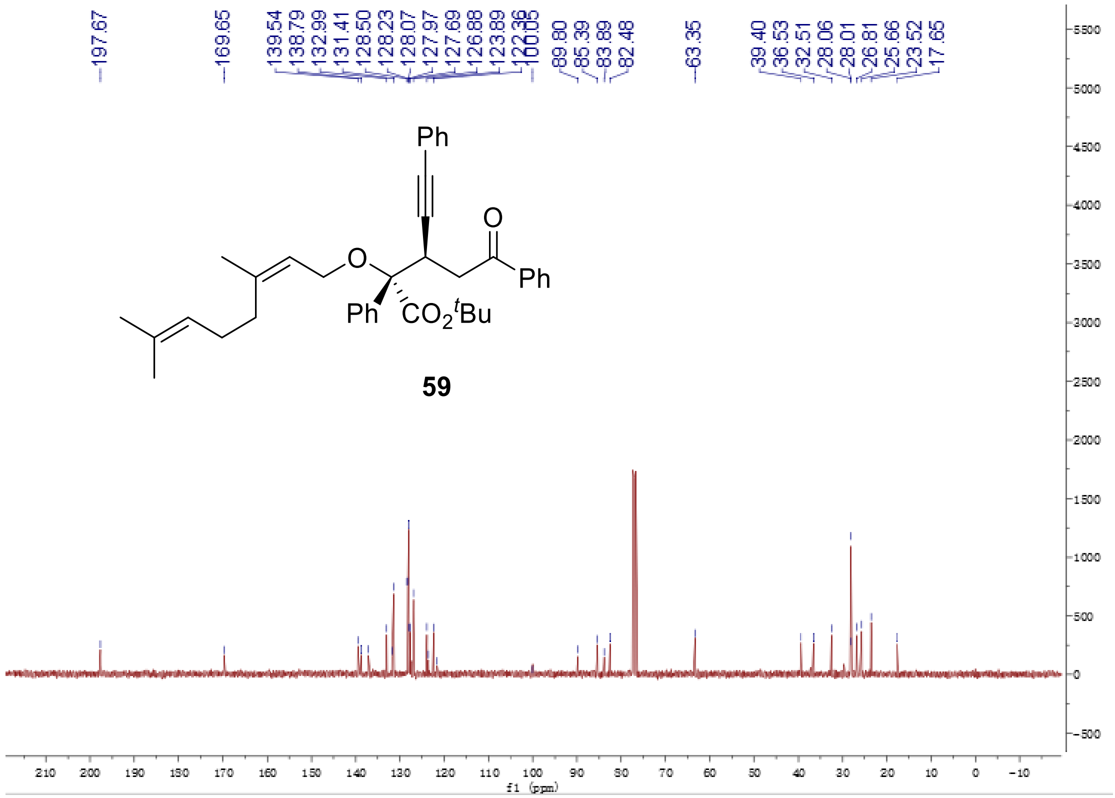

Supplementary Figure 396. ^{13}C NMR (101 MHz, $CDCl_3$) spectrum of **59**.

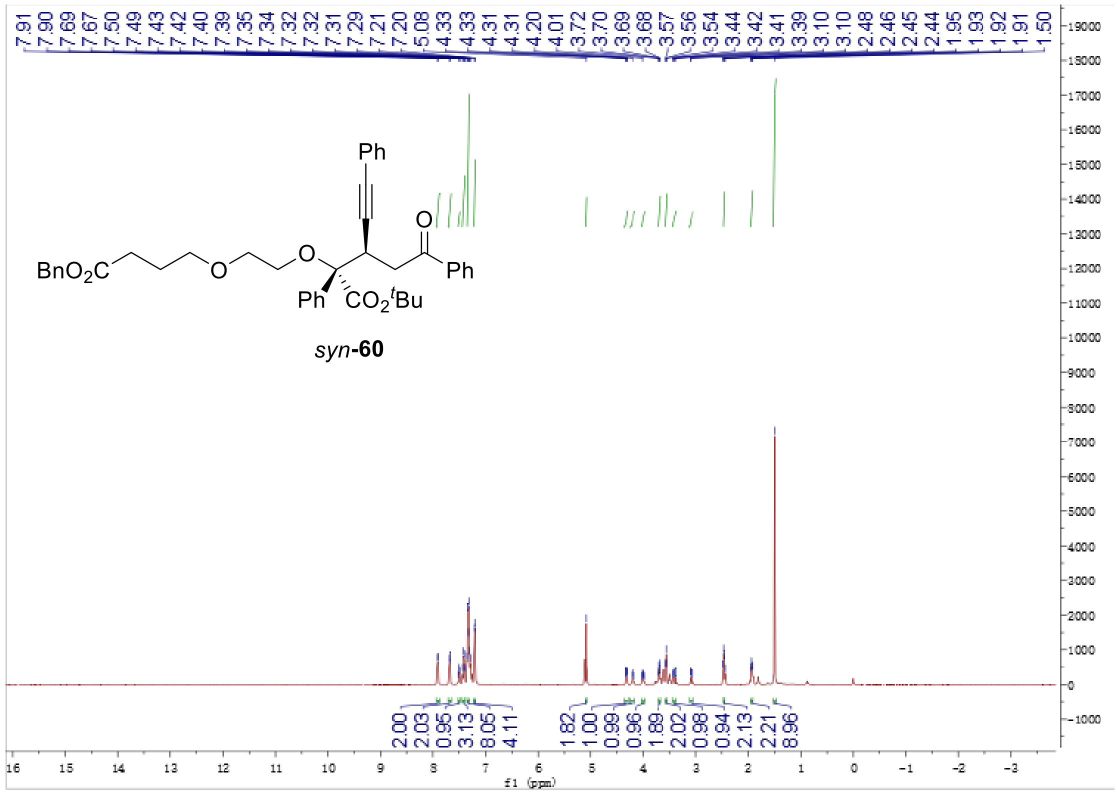

Supplementary Figure 397. ^{1}H NMR (500 MHz, $CDCl_3$) spectrum of **60**.

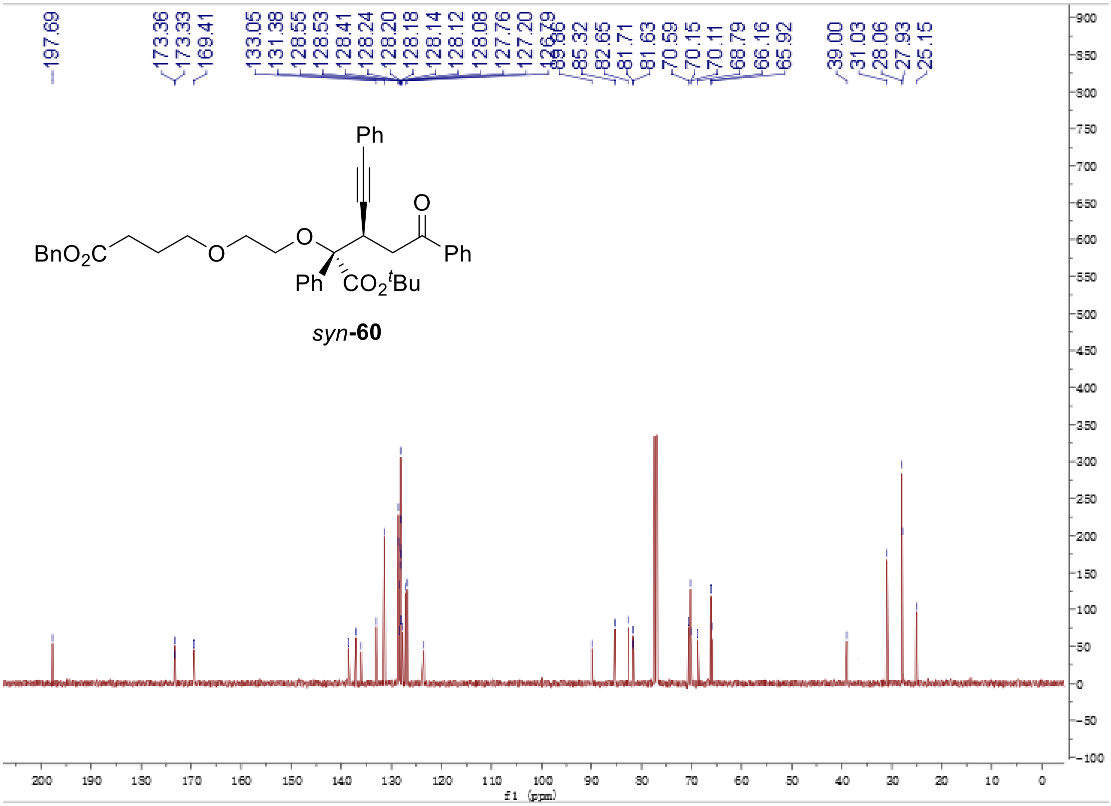

Supplementary Figure 398. ^{13}C NMR (126 MHz, $CDCl_3$) spectrum of **60**.

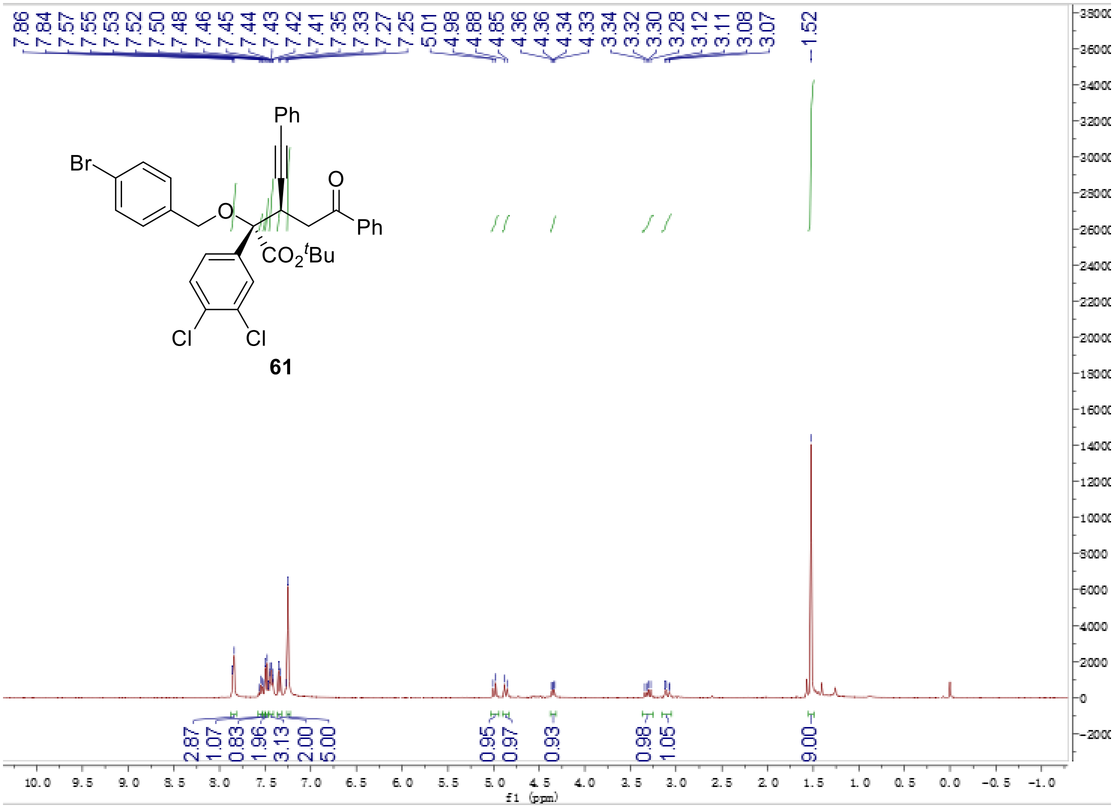

Supplementary Figure 399. ^{1}H NMR (400 MHz, $CDCl_3$) spectrum of **61**.

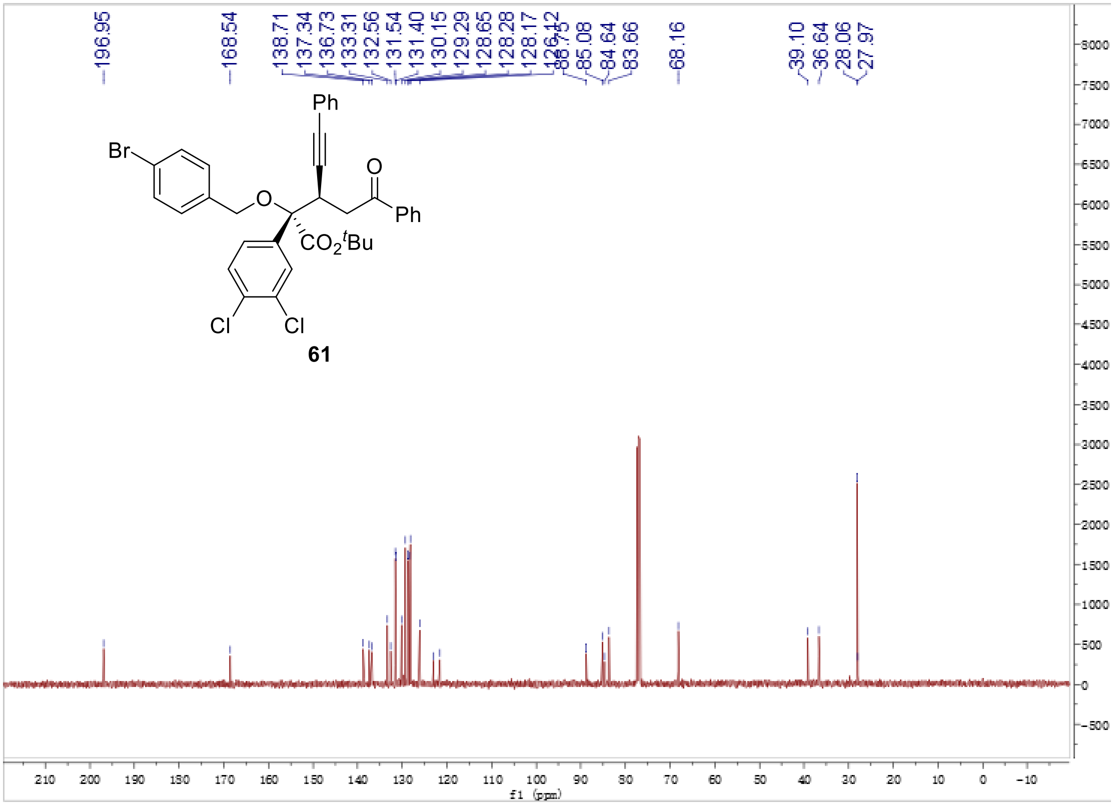

Supplementary Figure 400. ^{13}C NMR (101 MHz, $CDCl_3$) spectrum of **61**.

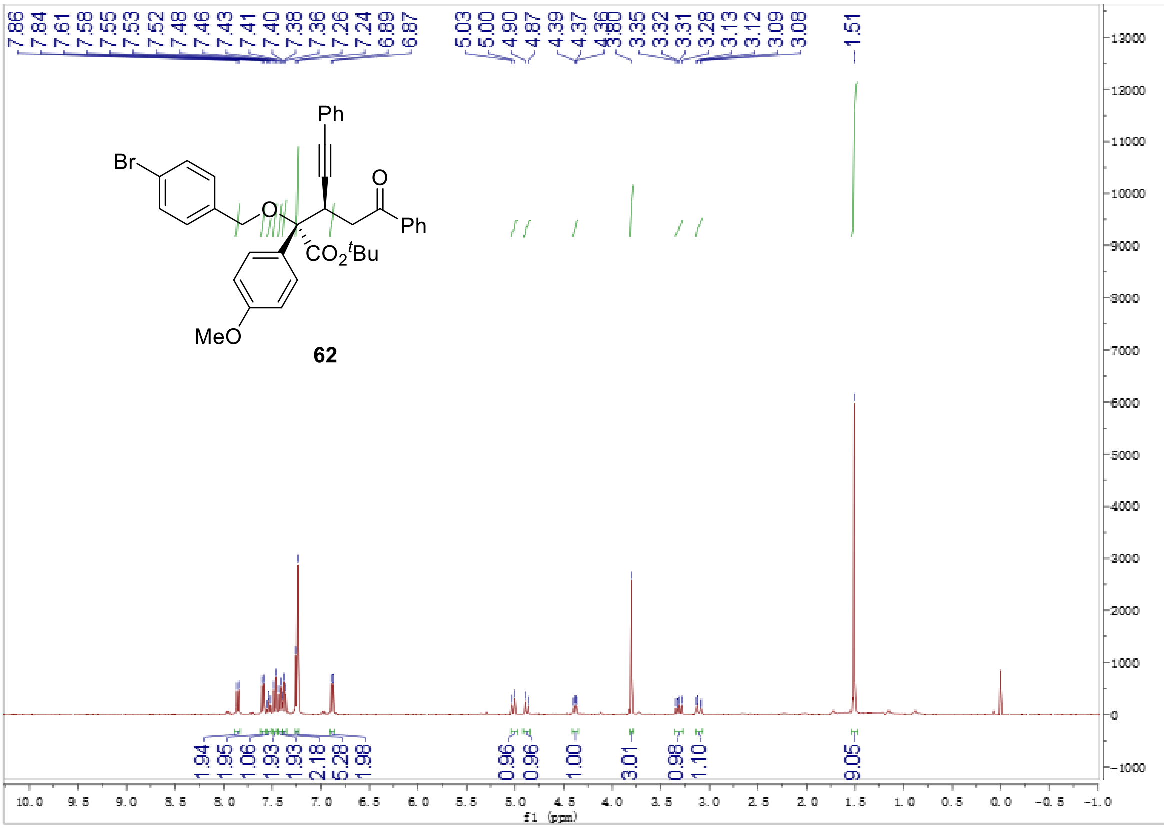

Supplementary Figure 401. ^{1}H NMR (400 MHz, $CDCl_3$) spectrum of **62**.

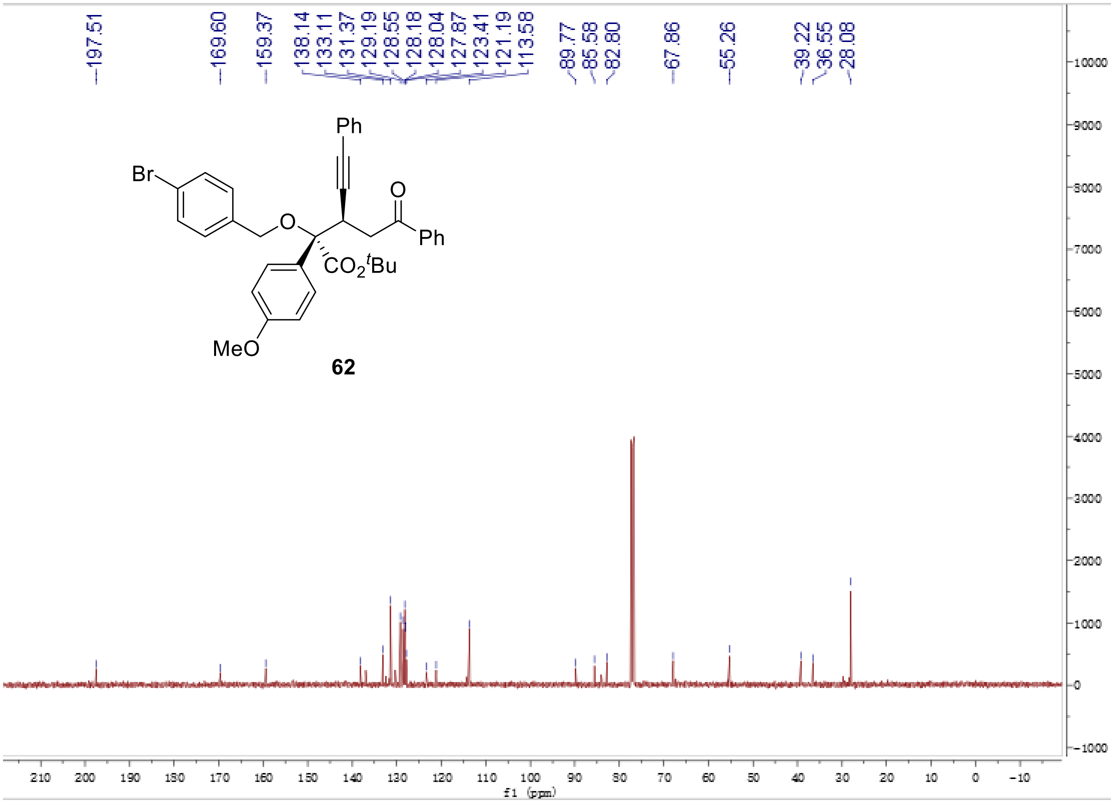

Supplementary Figure 402. ^{13}C NMR (101 MHz, $CDCl_3$) spectrum of **62**.

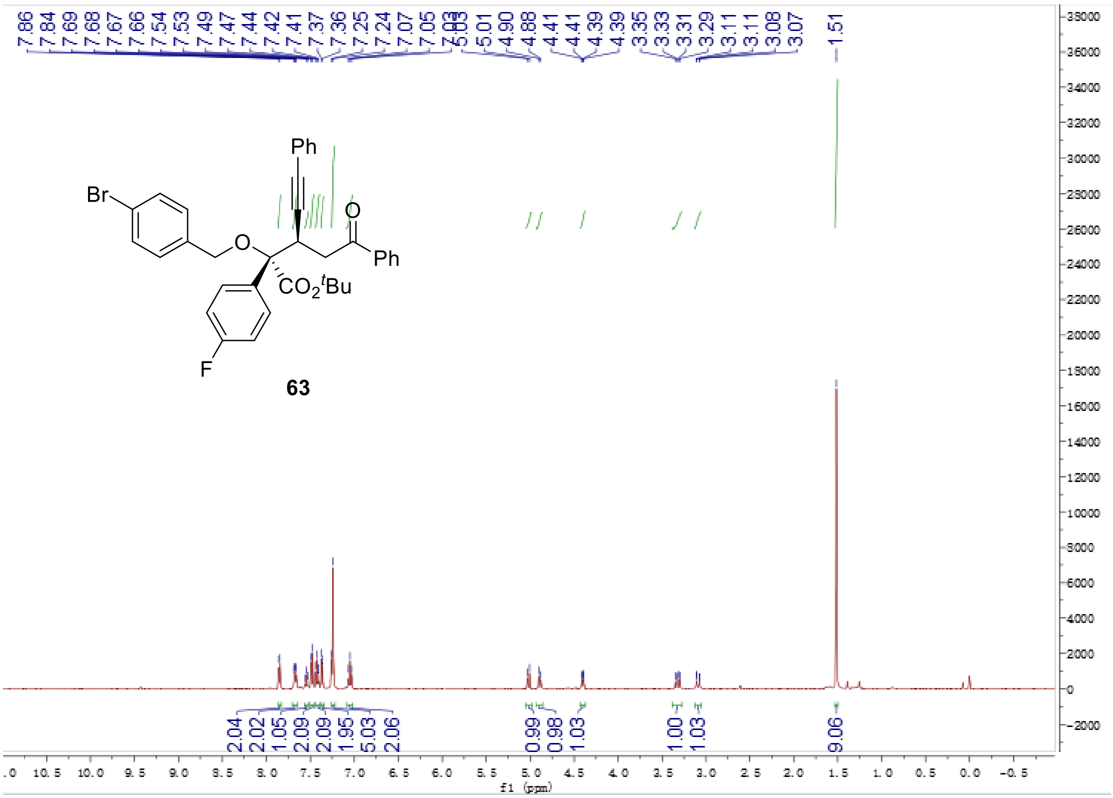

Supplementary Figure 403. ^{1}H NMR (500 MHz, $CDCl_3$) spectrum of **63**.

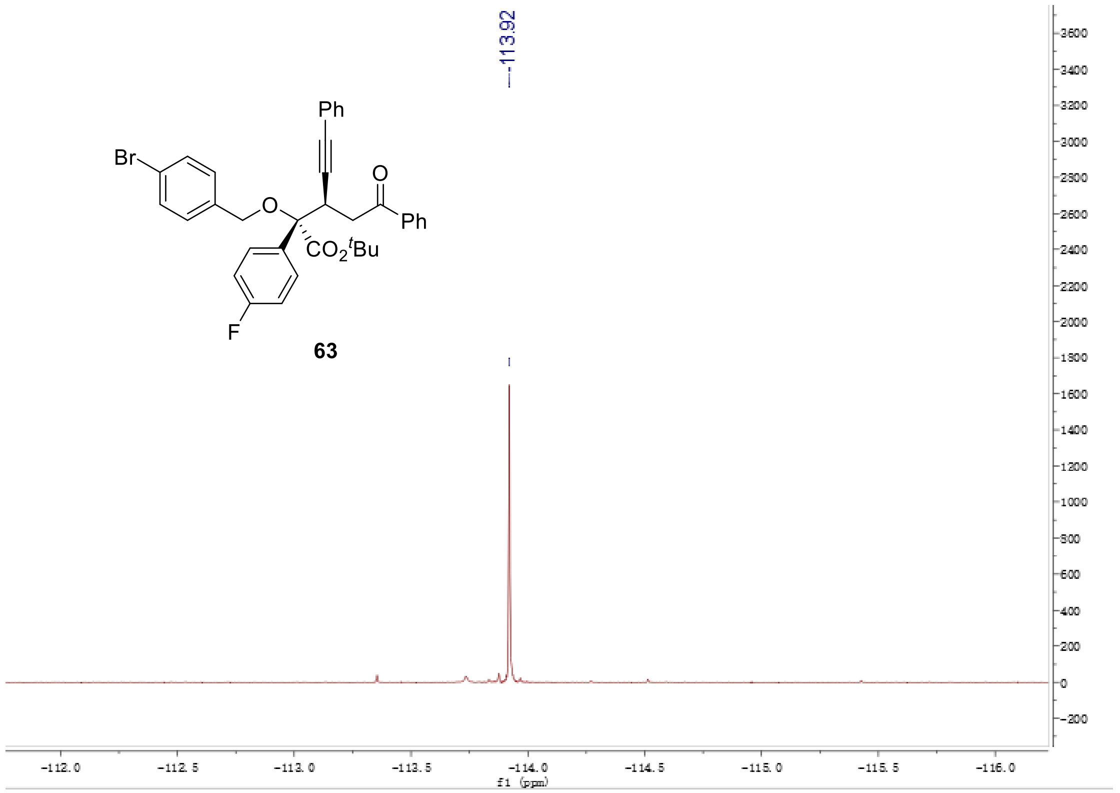

Supplementary Figure 404. ^{19}F NMR (471 MHz, $CDCl_3$) spectrum of **63**.

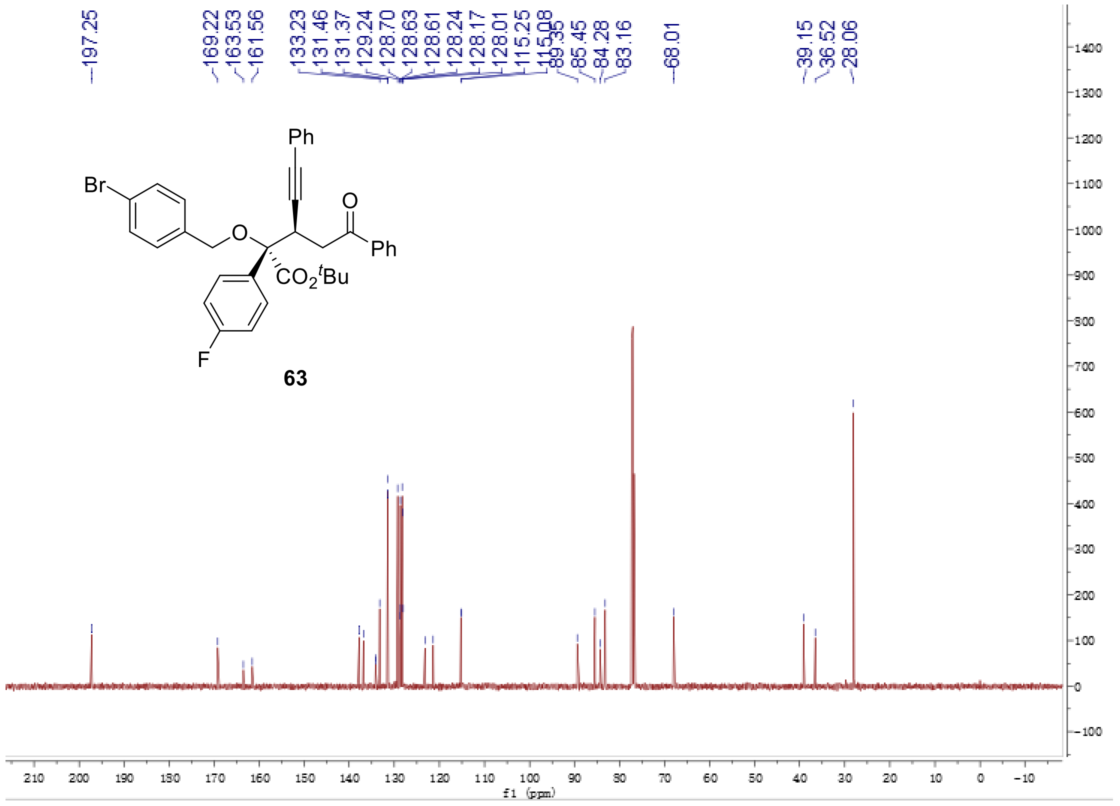

Supplementary Figure 405. ^{13}C NMR (126 MHz, $CDCl_3$) spectrum of **63**.

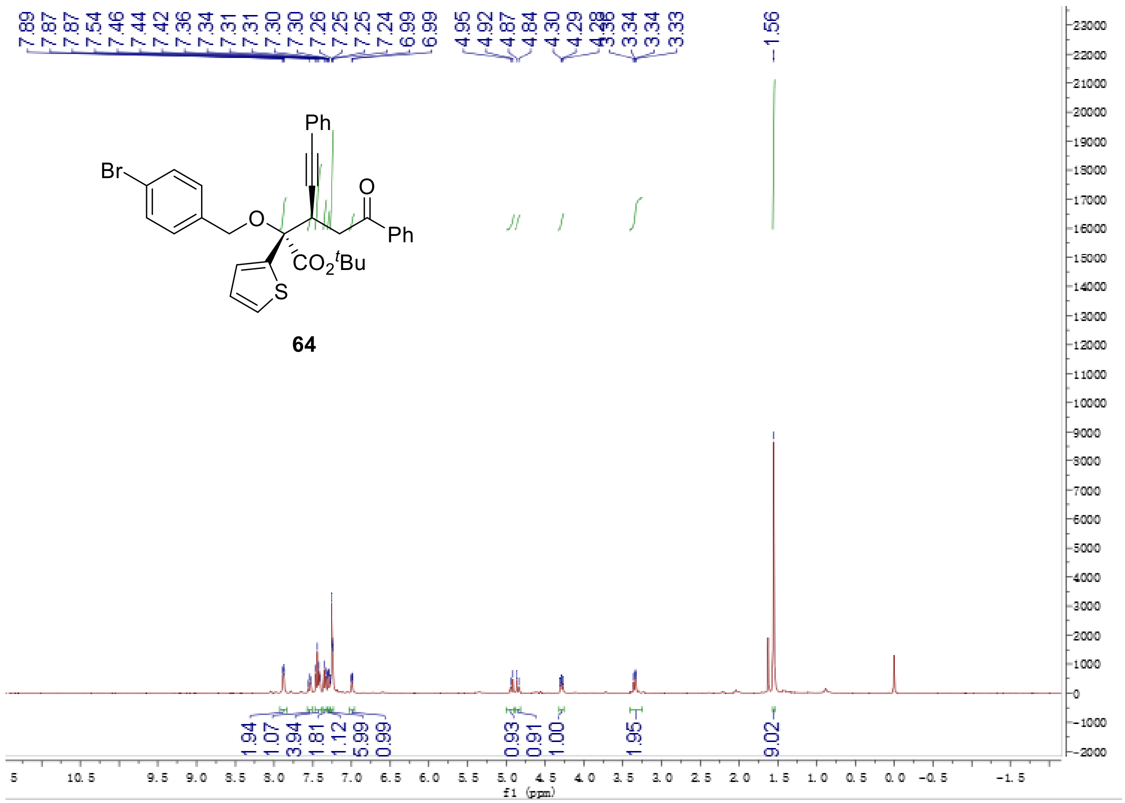

Supplementary Figure 406. ^{1}H NMR (400 MHz, $CDCl_3$) spectrum of **64**.

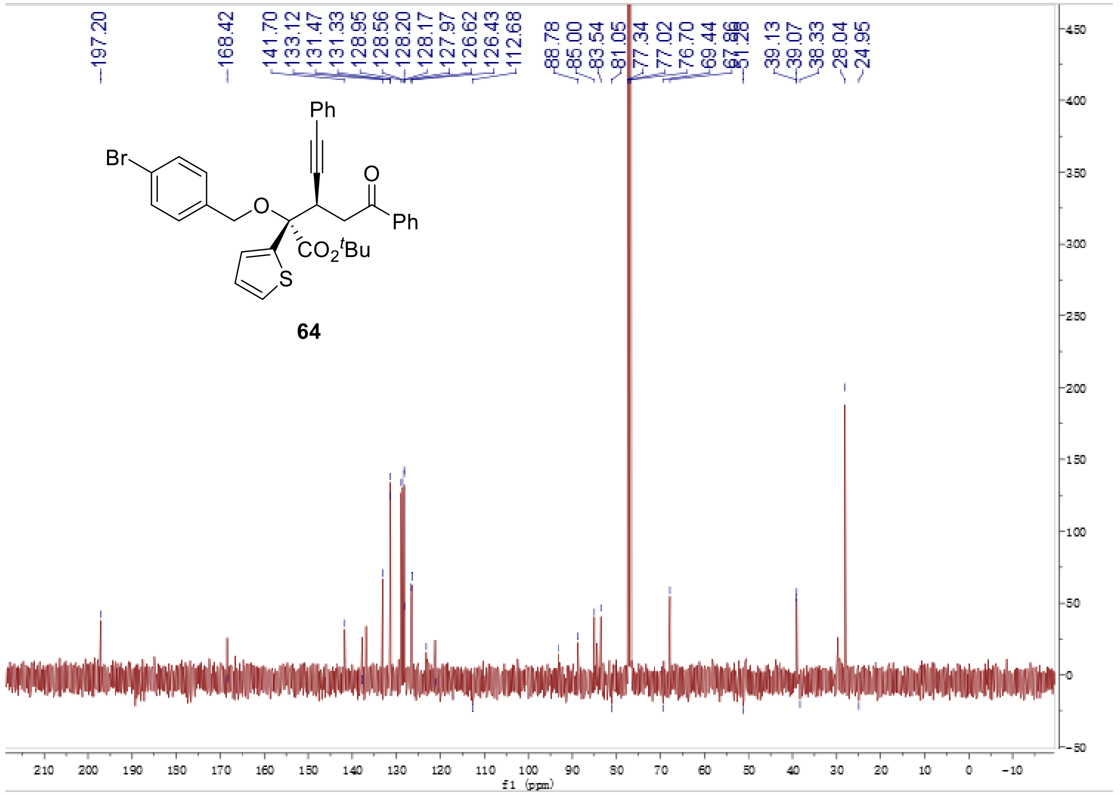

Supplementary Figure 407. ^{13}C NMR (101 MHz, $CDCl_3$) spectrum of **64**.

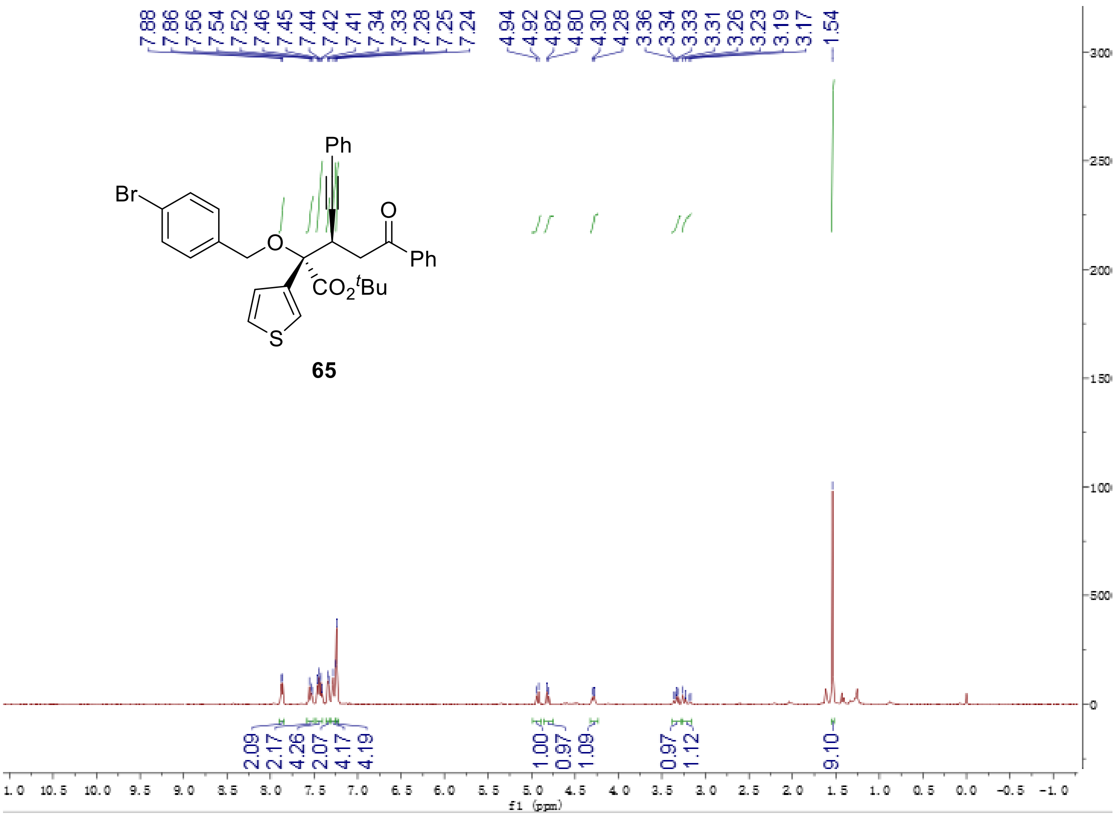

Supplementary Figure 408. ^{1}H NMR (500 MHz, $CDCl_3$) spectrum of **65**.

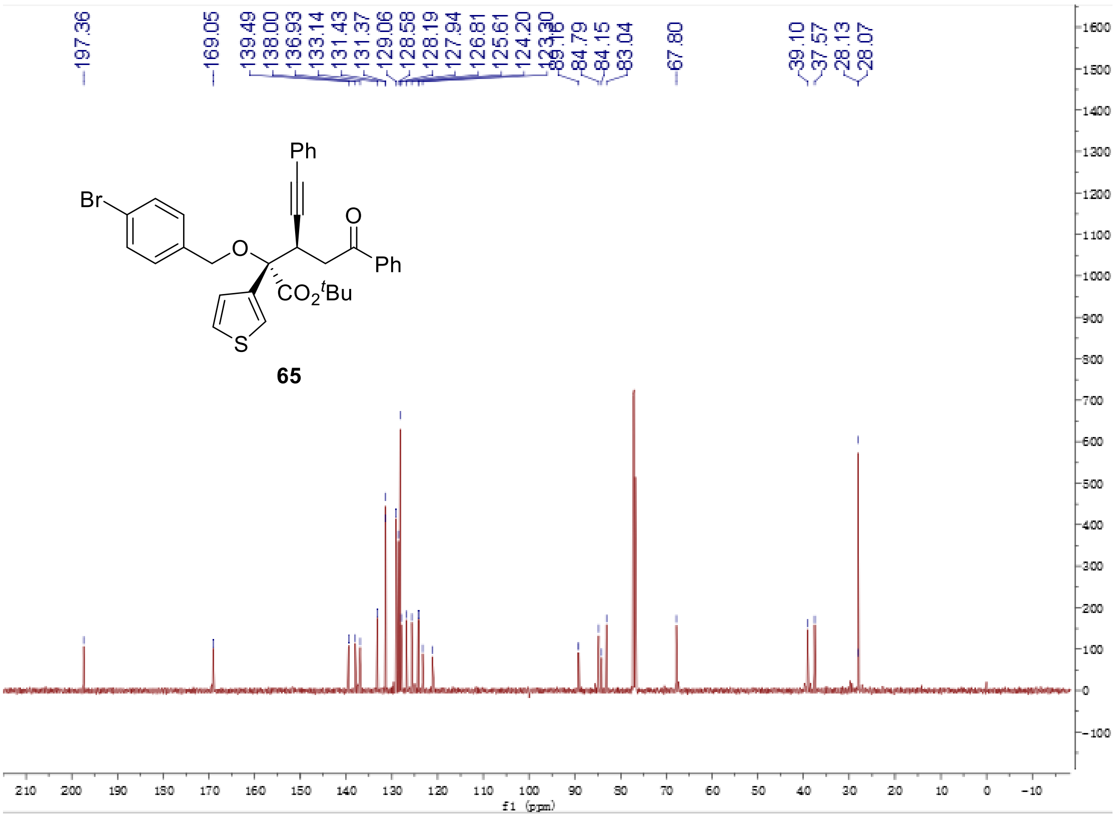

Supplementary Figure 409. ^{13}C NMR (126 MHz, $CDCl_3$) spectrum of **65**.

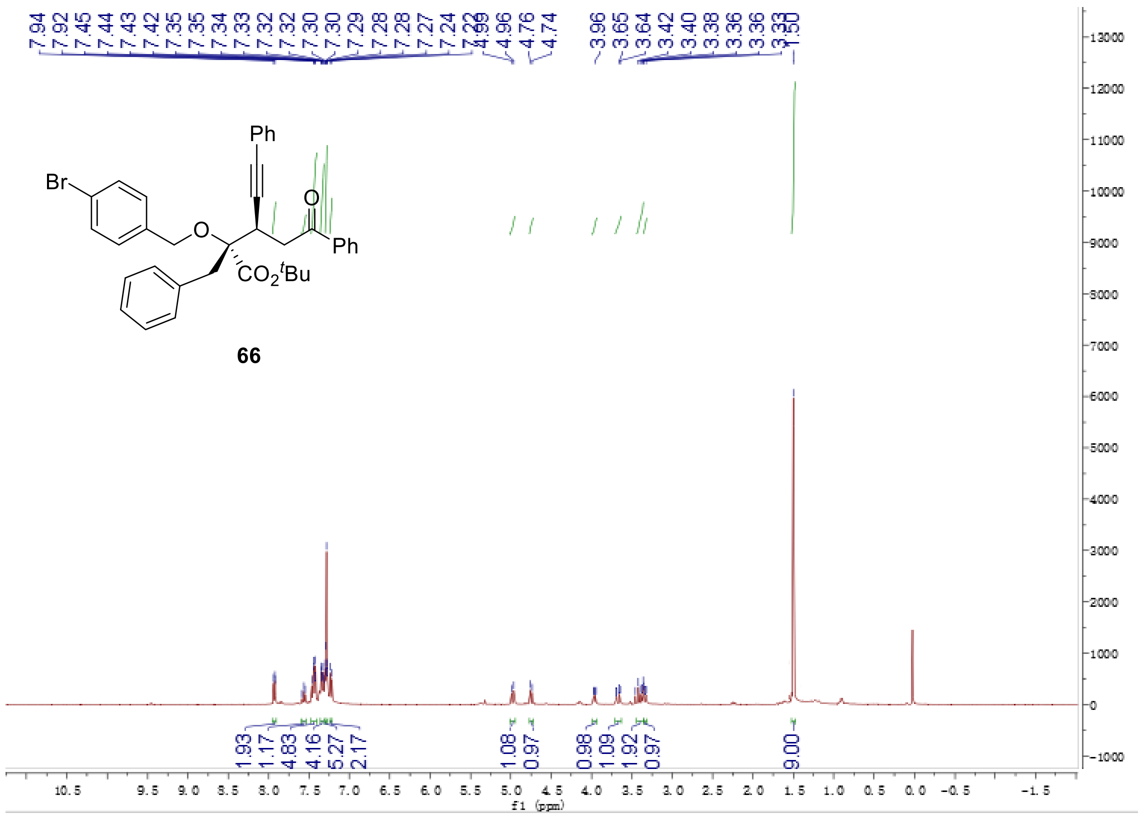

Supplementary Figure 410. ^{1}H NMR (400 MHz, $CDCl_3$) spectrum of **66**.

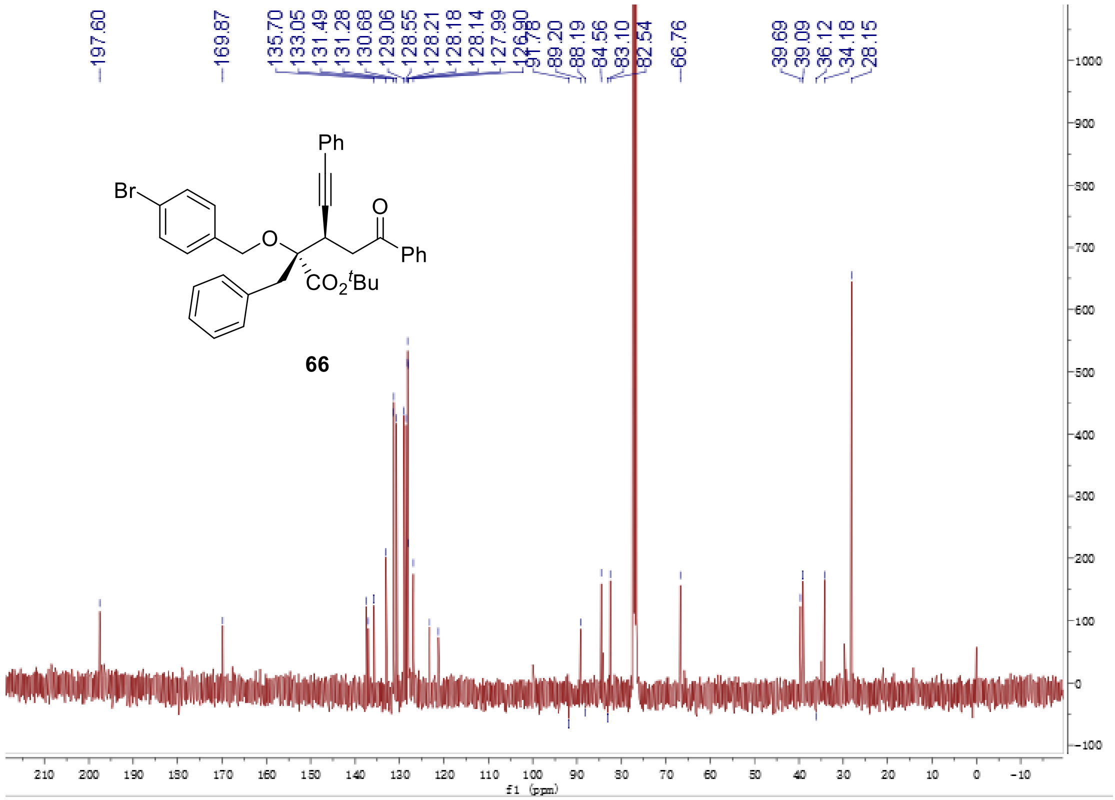

Supplementary Figure 411. ^{13}C NMR (101 MHz, $CDCl_3$) spectrum of **66**.

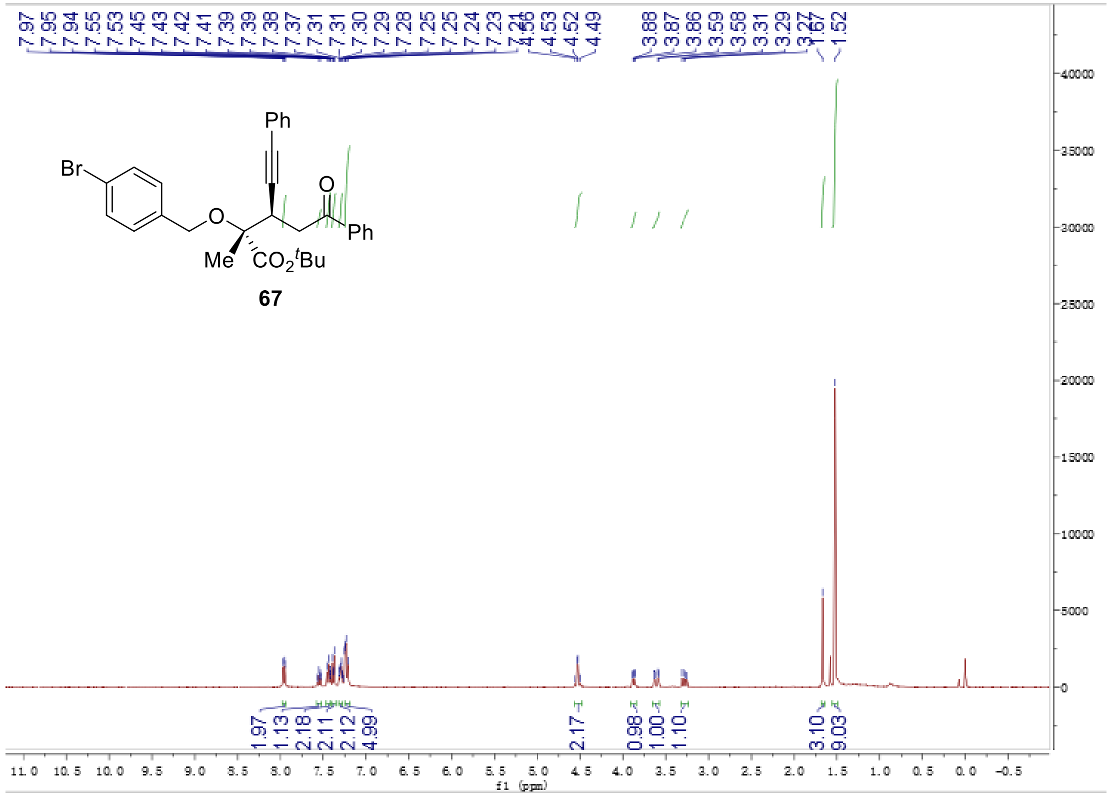

Supplementary Figure 412. ^{1}H NMR (400 MHz, $CDCl_3$) spectrum of **67**.

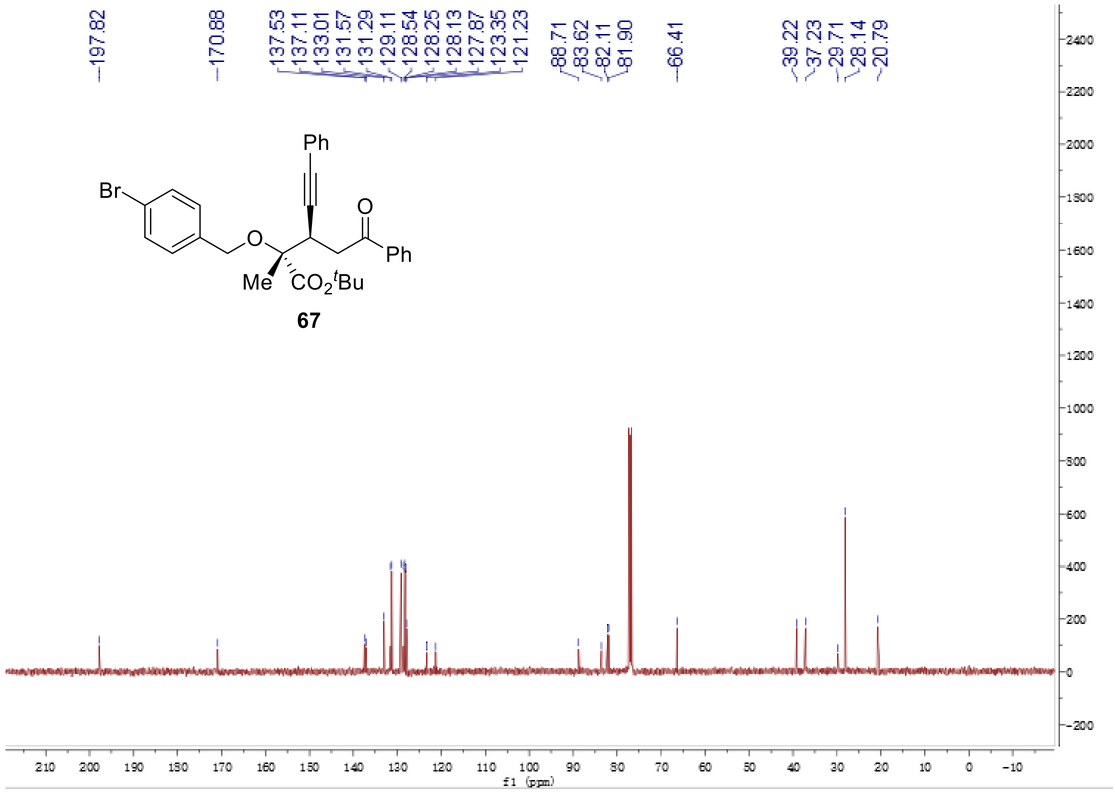

Supplementary Figure 413. ^{13}C NMR (101 MHz, $CDCl_3$) spectrum of **67**.

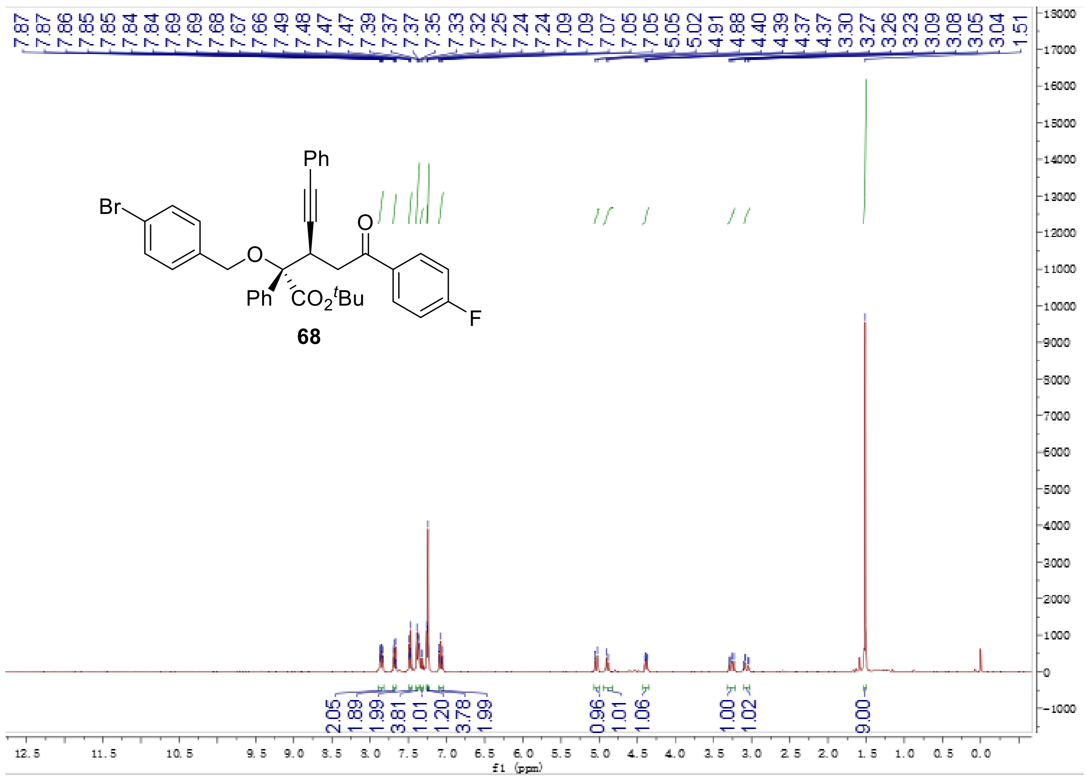

Supplementary Figure 414. ^{1}H NMR (400 MHz, $CDCl_3$) spectrum of **68**.

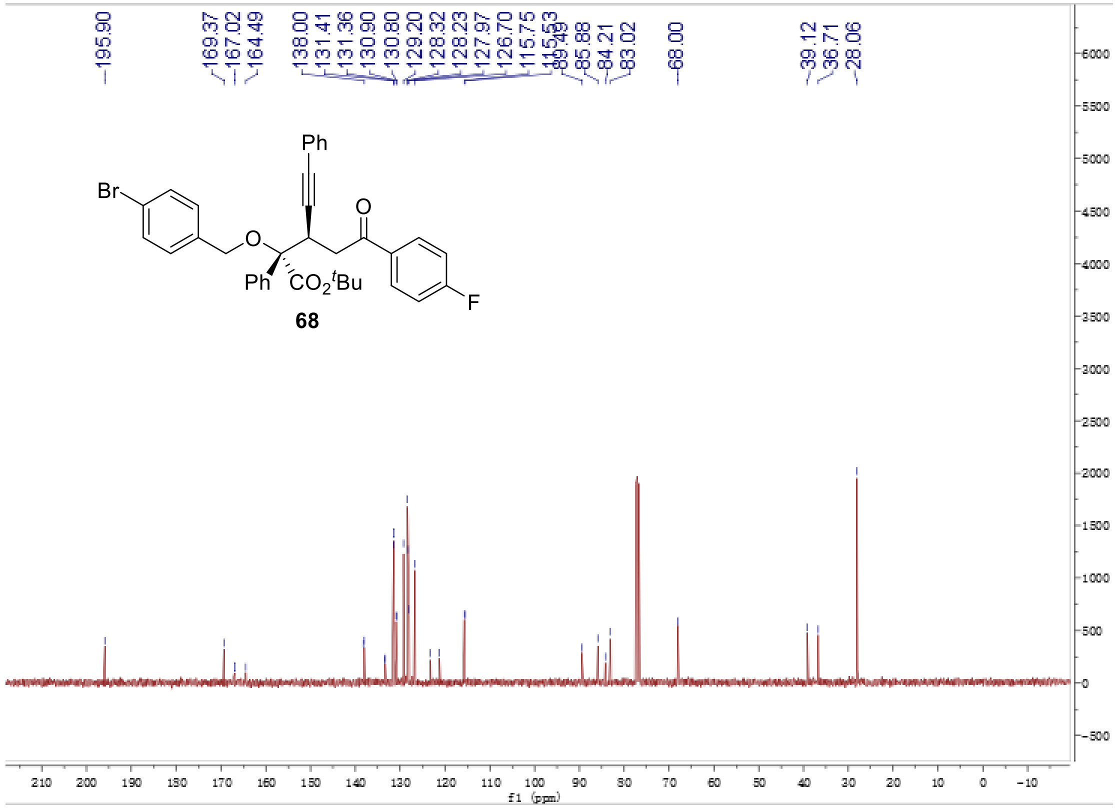

Supplementary Figure 415. ^{13}C NMR (101 MHz, $CDCl_3$) spectrum of **68**.

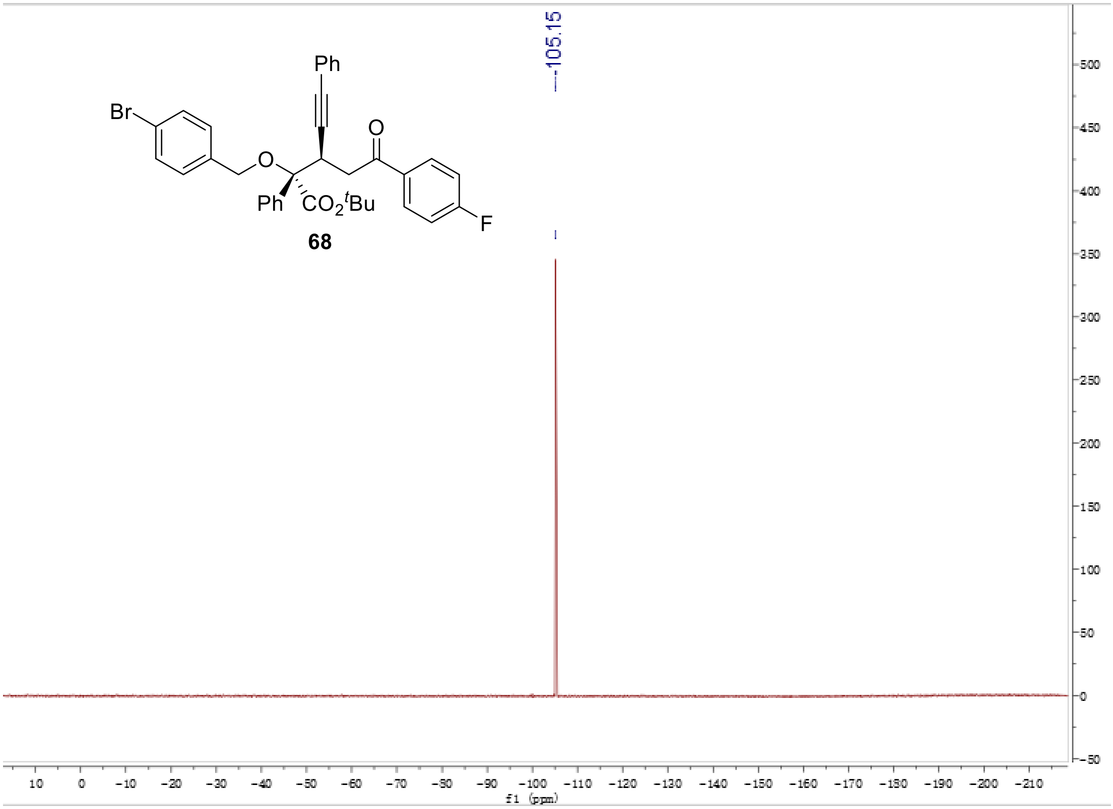

Supplementary Figure 416. ^{19}F NMR (376 MHz, $CDCl_3$) spectrum of **68**.

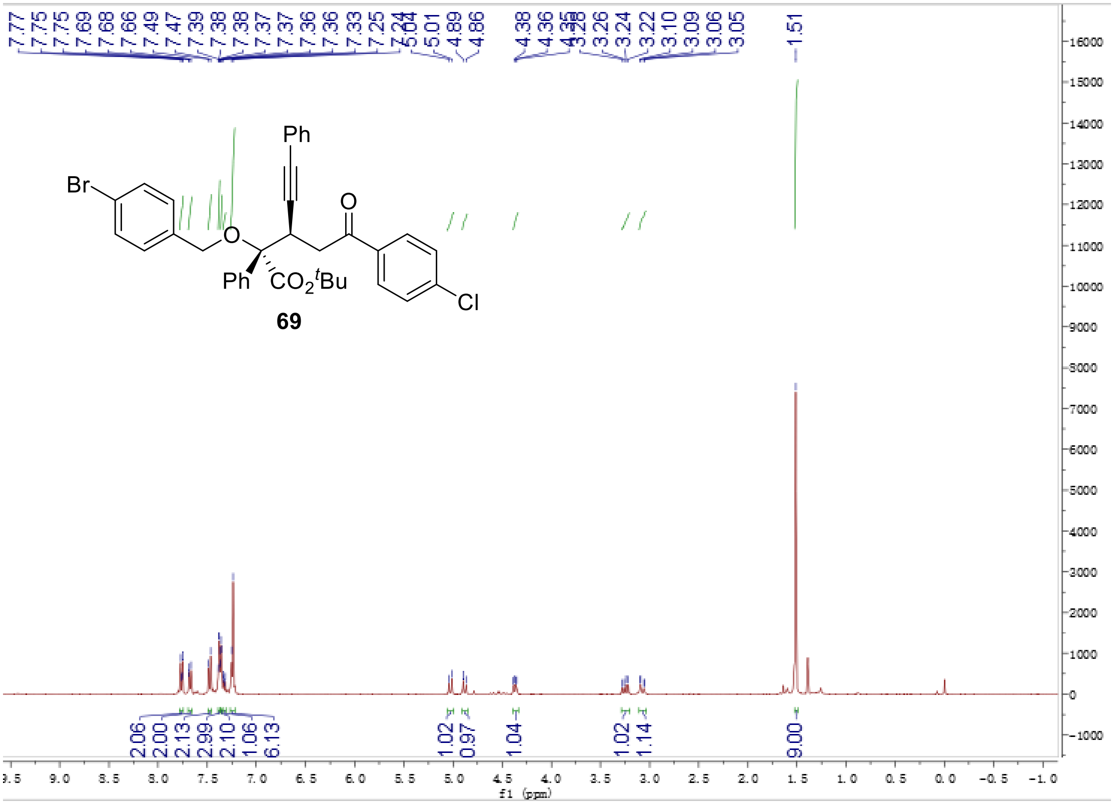

Supplementary Figure 417. ^{1}H NMR (400 MHz, $CDCl_3$) spectrum of **69**.

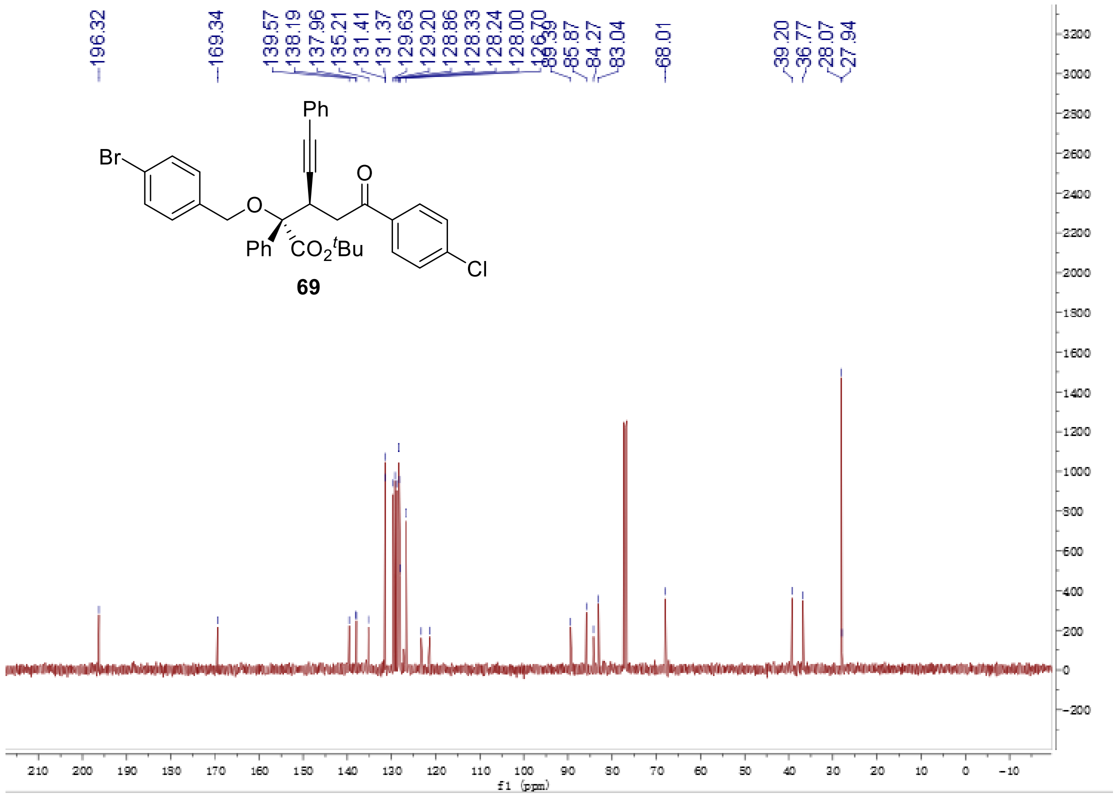

Supplementary Figure 418. ^{13}C NMR (101 MHz, $CDCl_3$) spectrum of **69**.

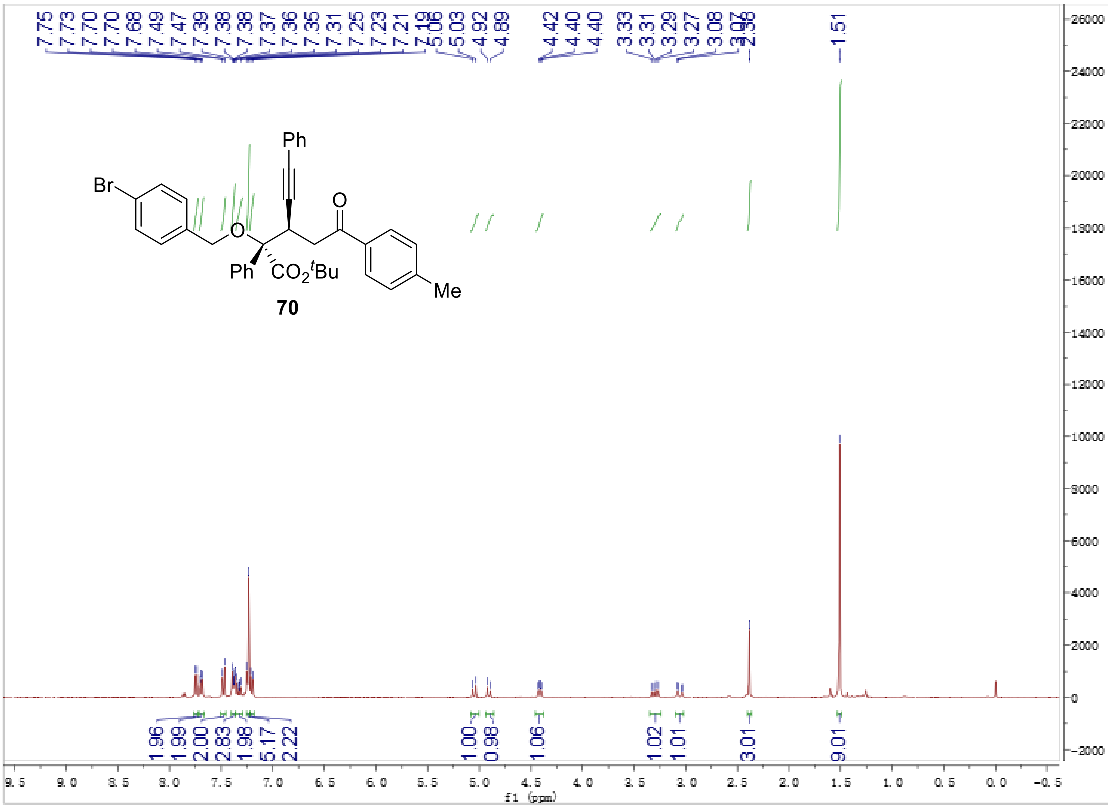

Supplementary Figure 419. ^{1}H NMR (400 MHz, $CDCl_3$) spectrum of **70**.

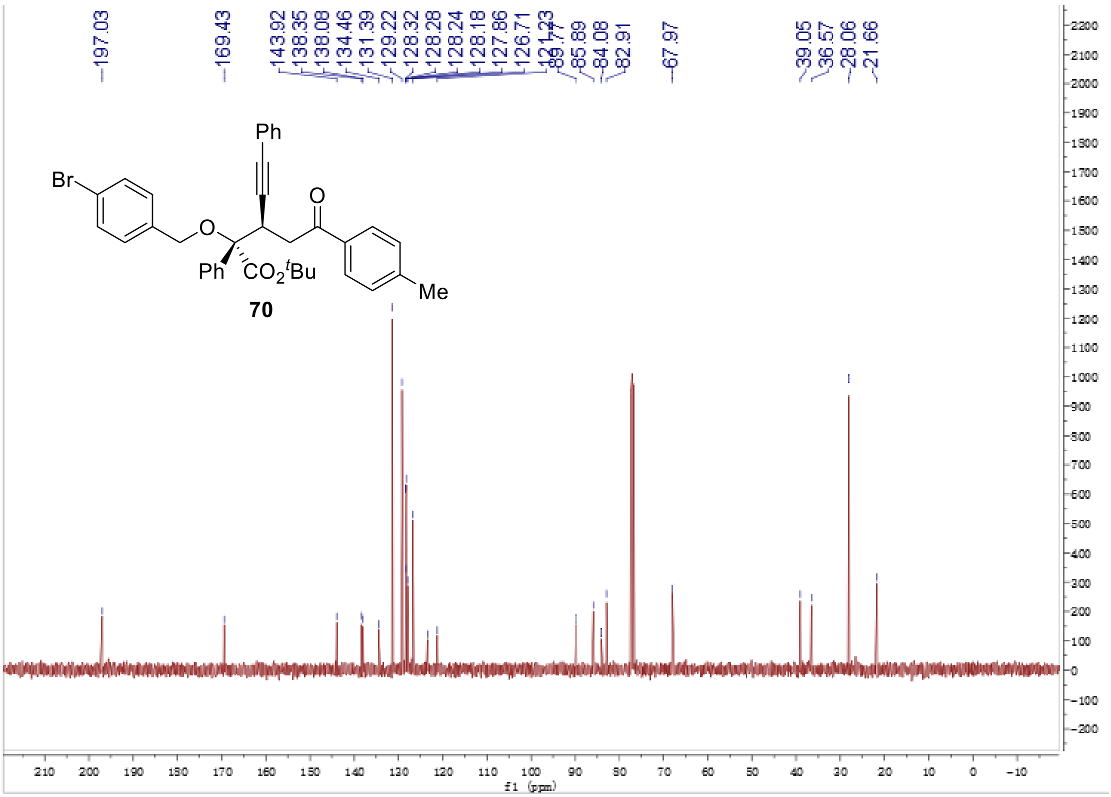

Supplementary Figure 420. ^{13}C NMR (101 MHz, $CDCl_3$) spectrum of **70**.

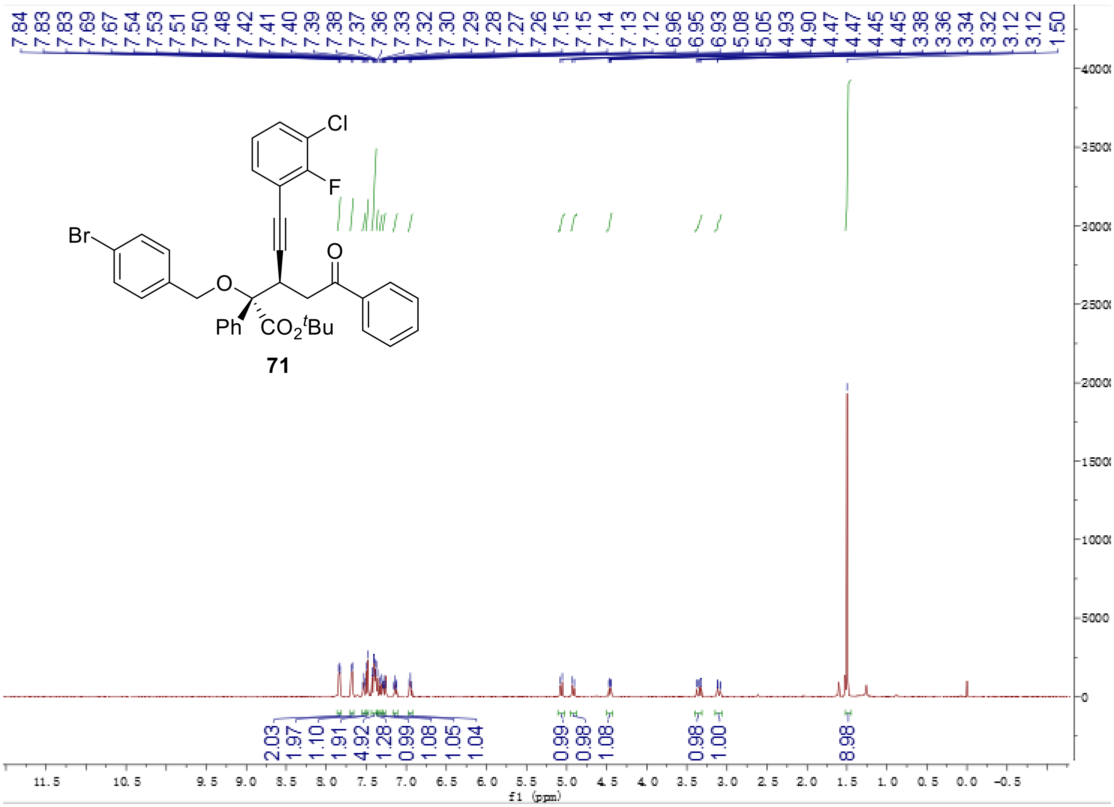

Supplementary Figure 421. ^{1}H NMR (500 MHz, $CDCl_3$) spectrum of **71**.

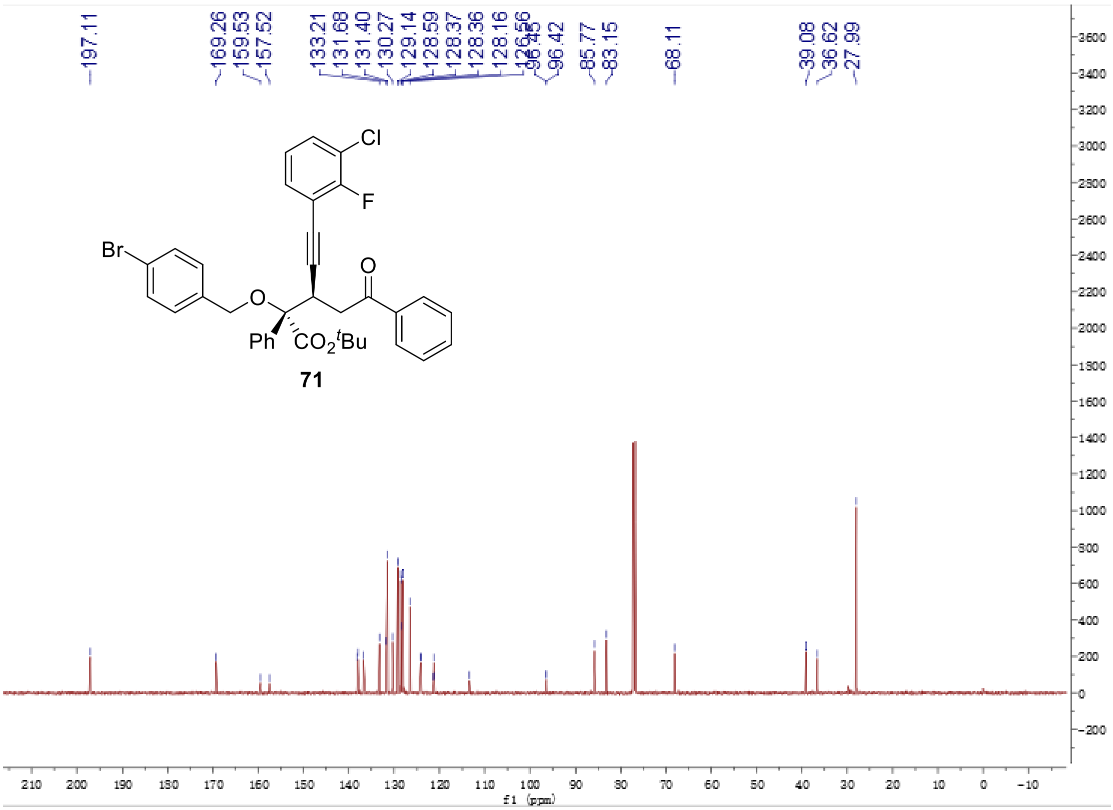

Supplementary Figure 422. ^{13}C NMR (126 MHz, $CDCl_3$) spectrum of **71**.

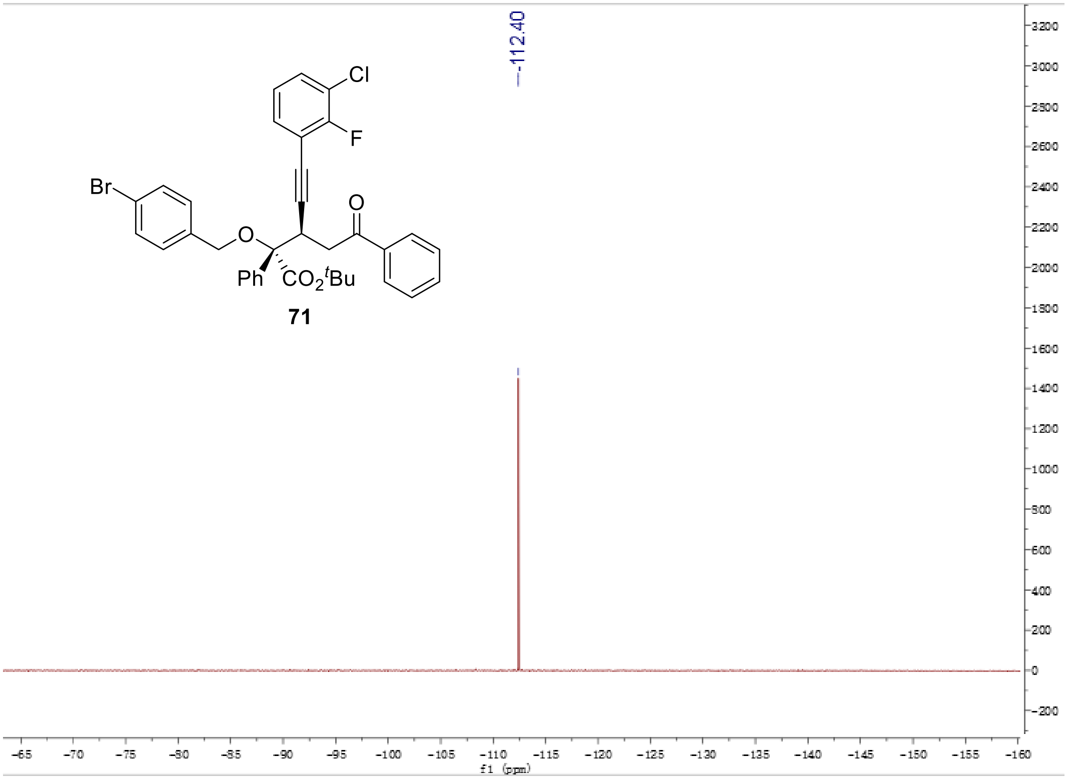

Supplementary Figure 423. ^{19}F NMR (471 MHz, $CDCl_3$) spectrum of **71**.

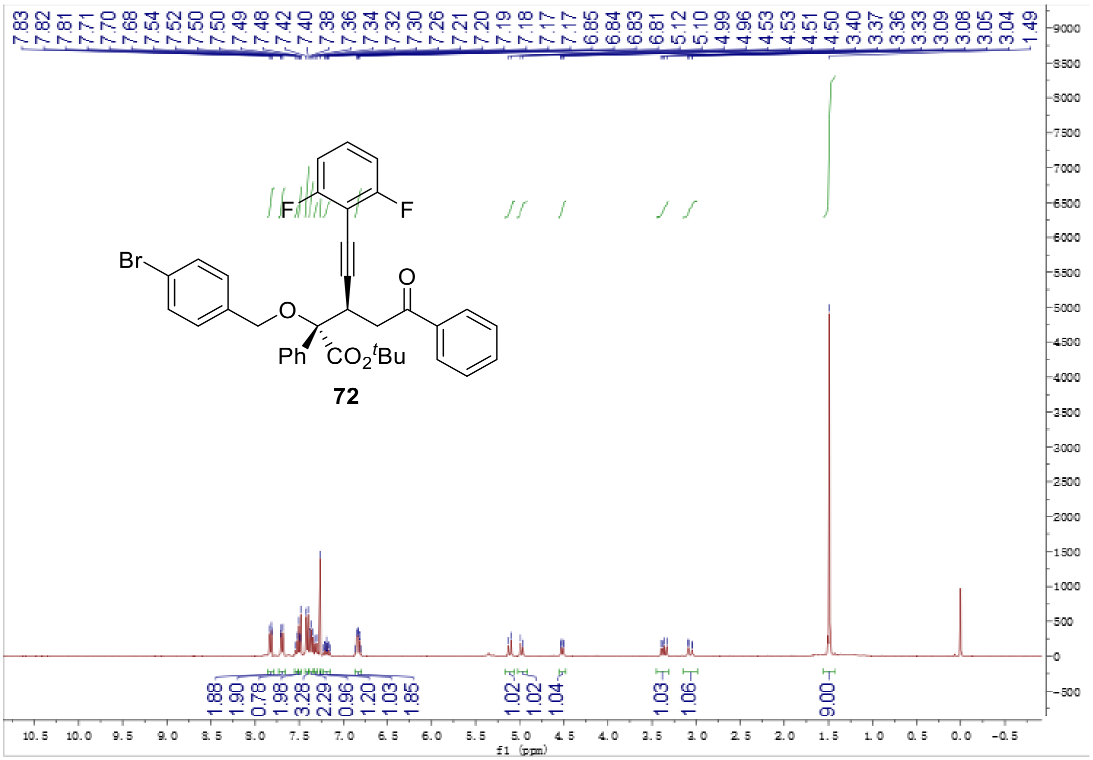

Supplementary Figure 424. ^{1}H NMR (400 MHz, $CDCl_3$) spectrum of **72**.

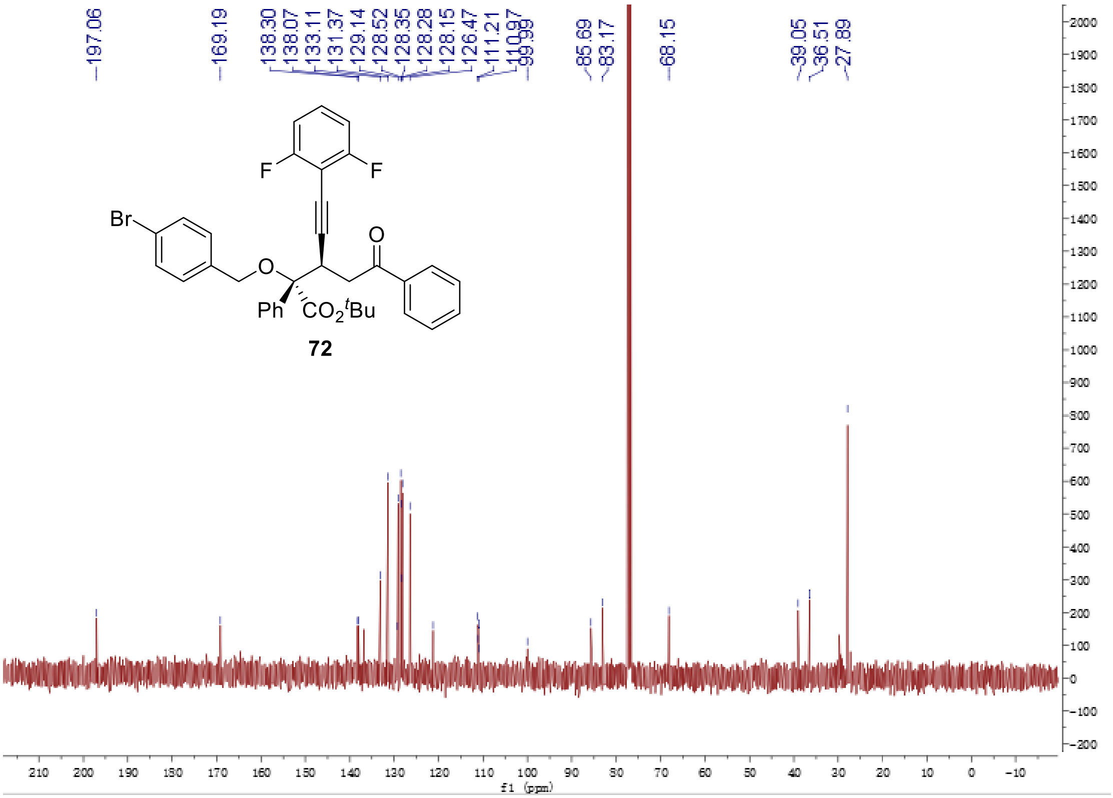

Supplementary Figure 425. ^{13}C NMR (101 MHz, $CDCl_3$) spectrum of **72**.

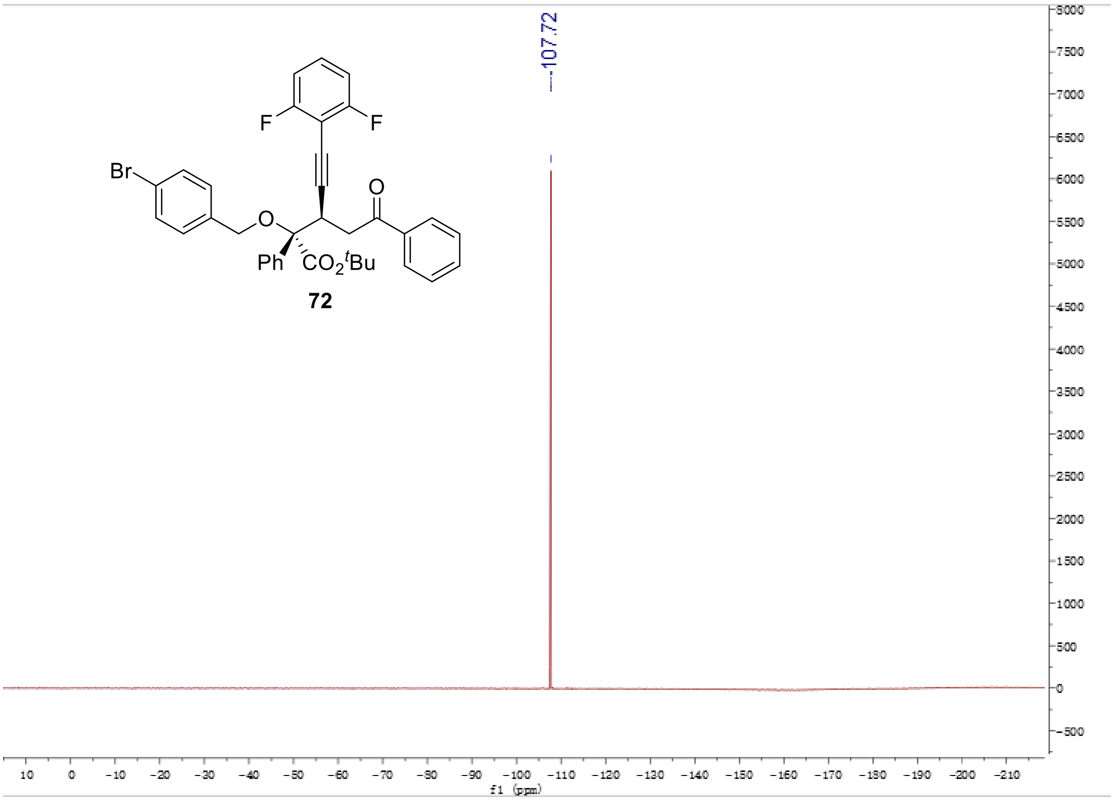

Supplementary Figure 426. ^{19}F NMR (376 MHz, $CDCl_3$) spectrum of **72**.

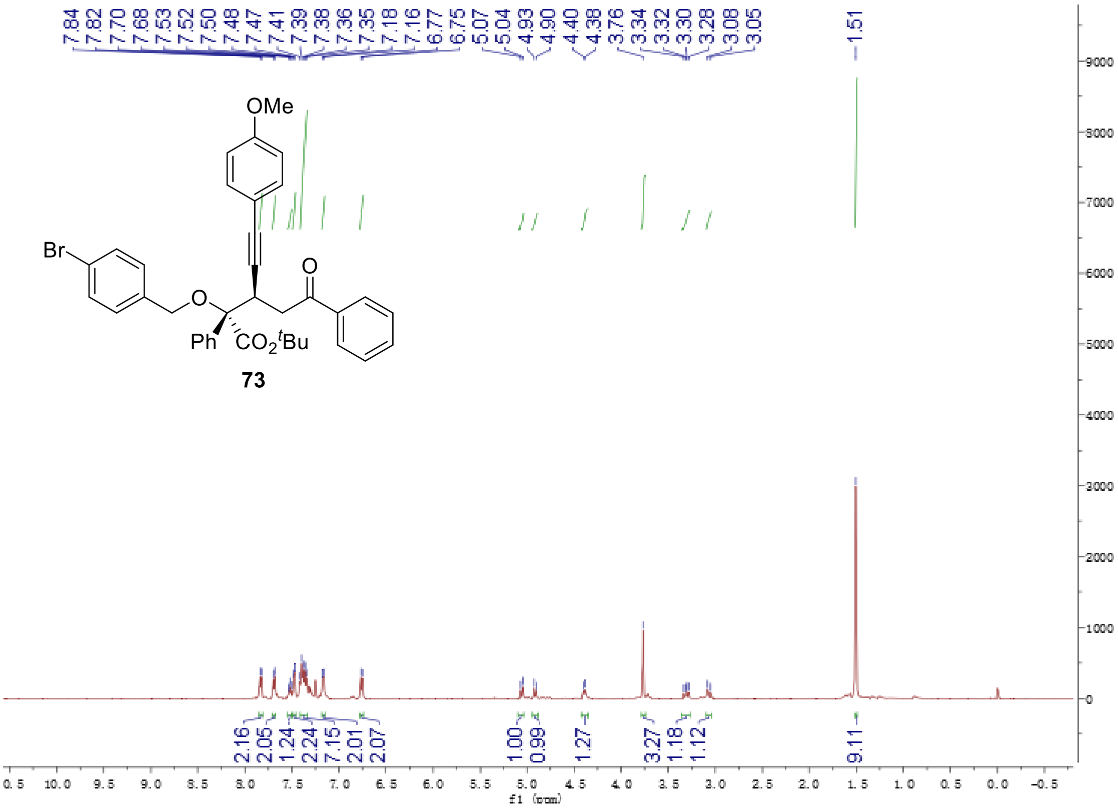

Supplementary Figure 427. ^{1}H NMR (500 MHz, $CDCl_3$) spectrum of **73**.

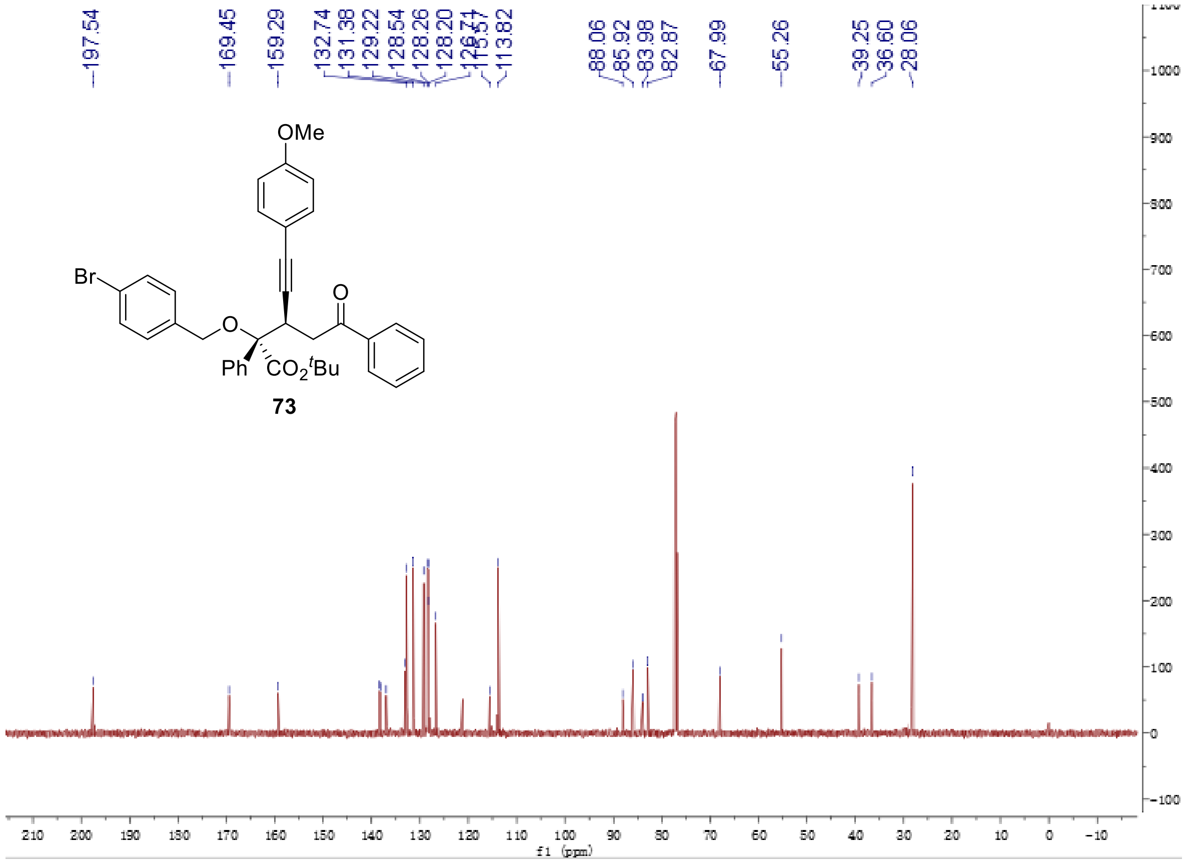

Supplementary Figure 428. ^{13}C NMR (126 MHz, $CDCl_3$) spectrum of **73**.

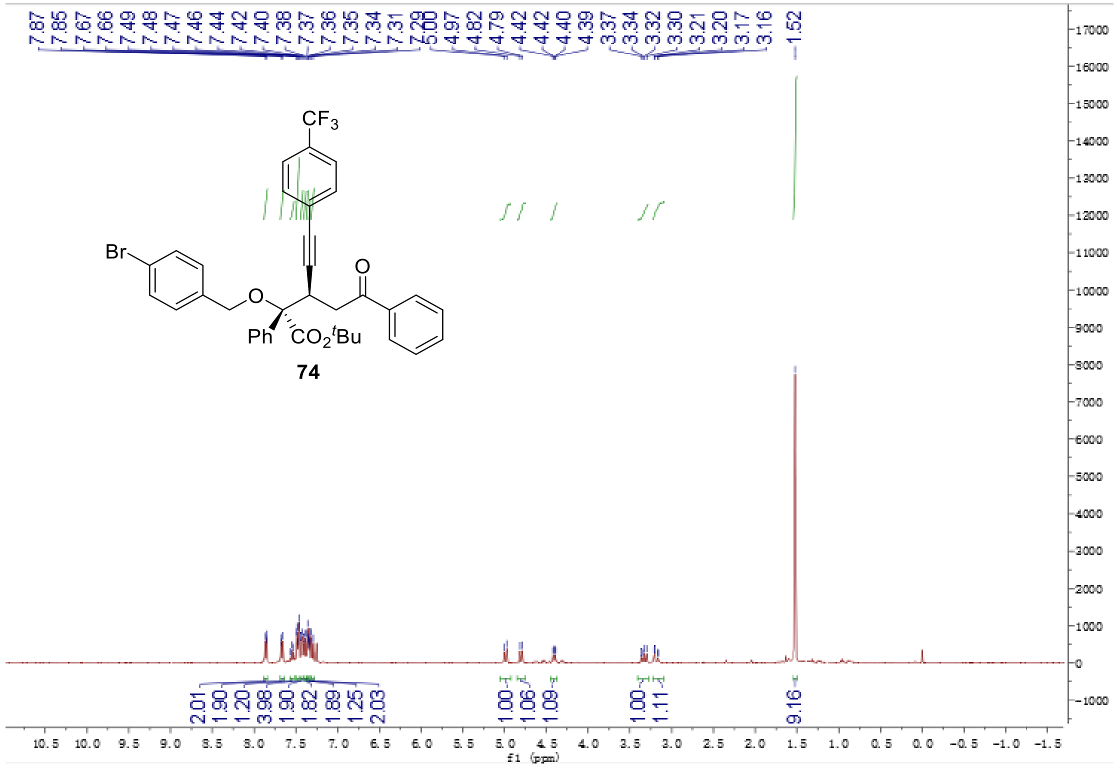

Supplementary Figure 429. ^{1}H NMR (400 MHz, $CDCl_3$) spectrum of **74**.

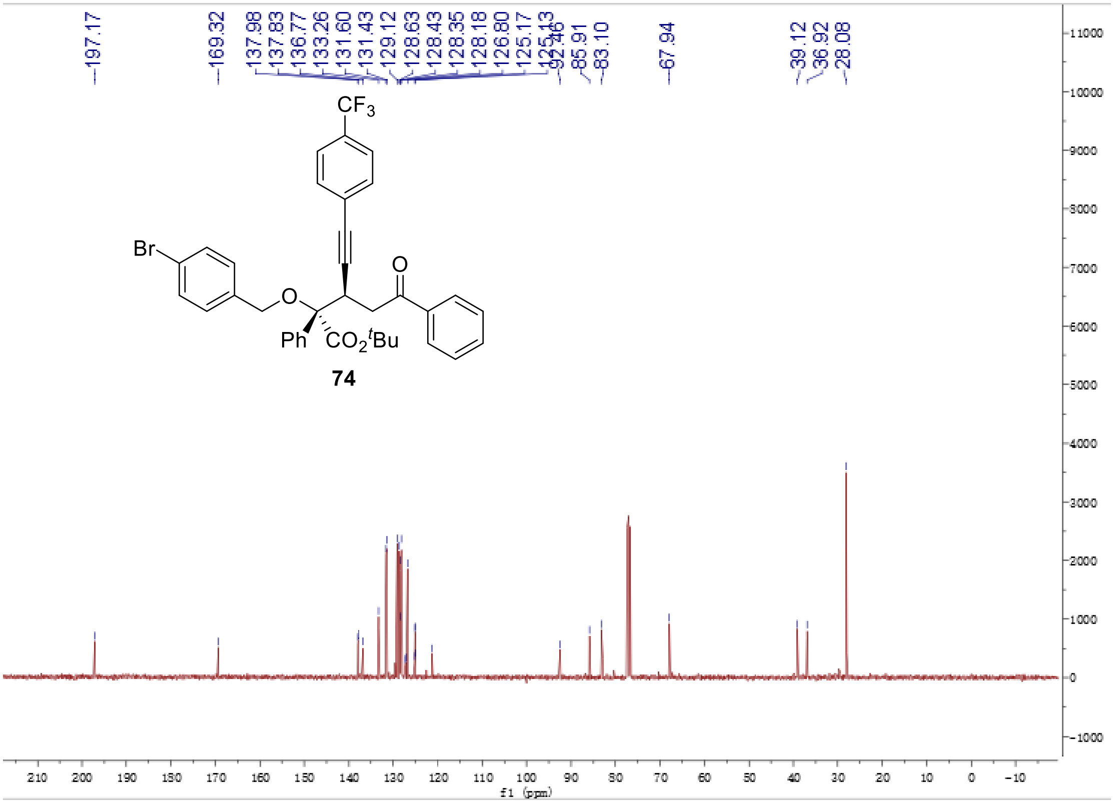

Supplementary Figure 430. ^{13}C NMR (101 MHz, $CDCl_3$) spectrum of **74**.

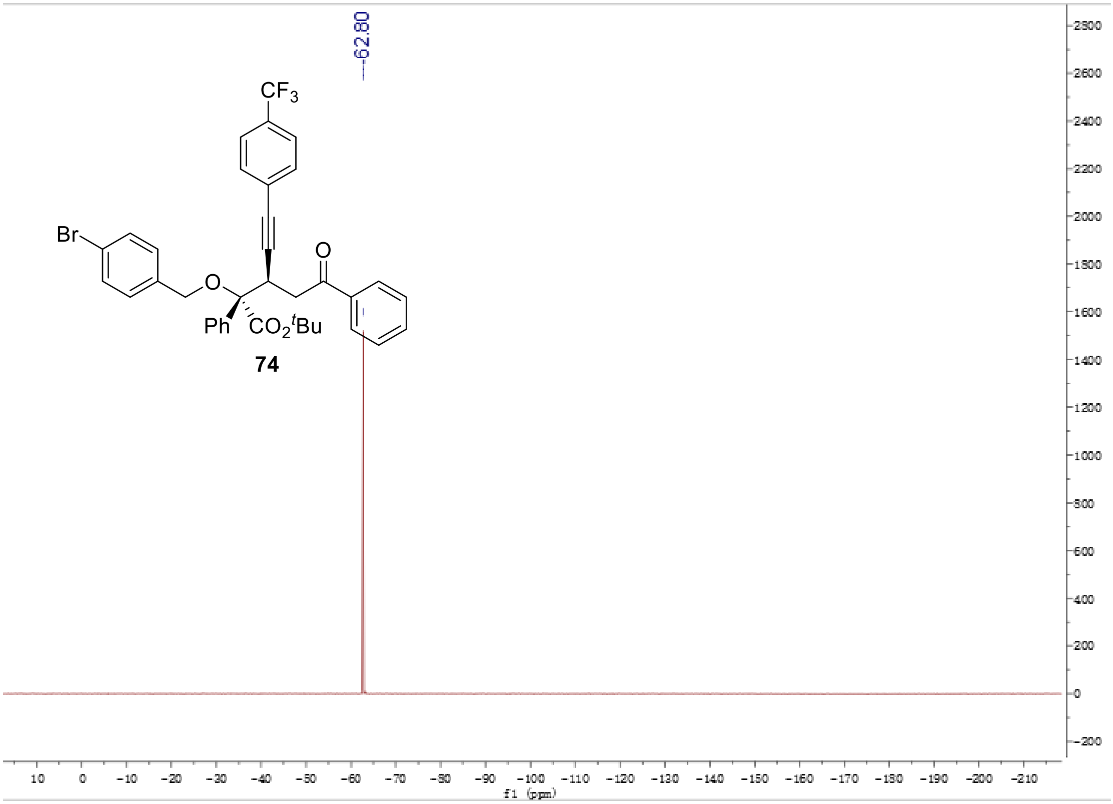

Supplementary Figure 431. ^{19}F NMR (376 MHz, $CDCl_3$) spectrum of **74**.

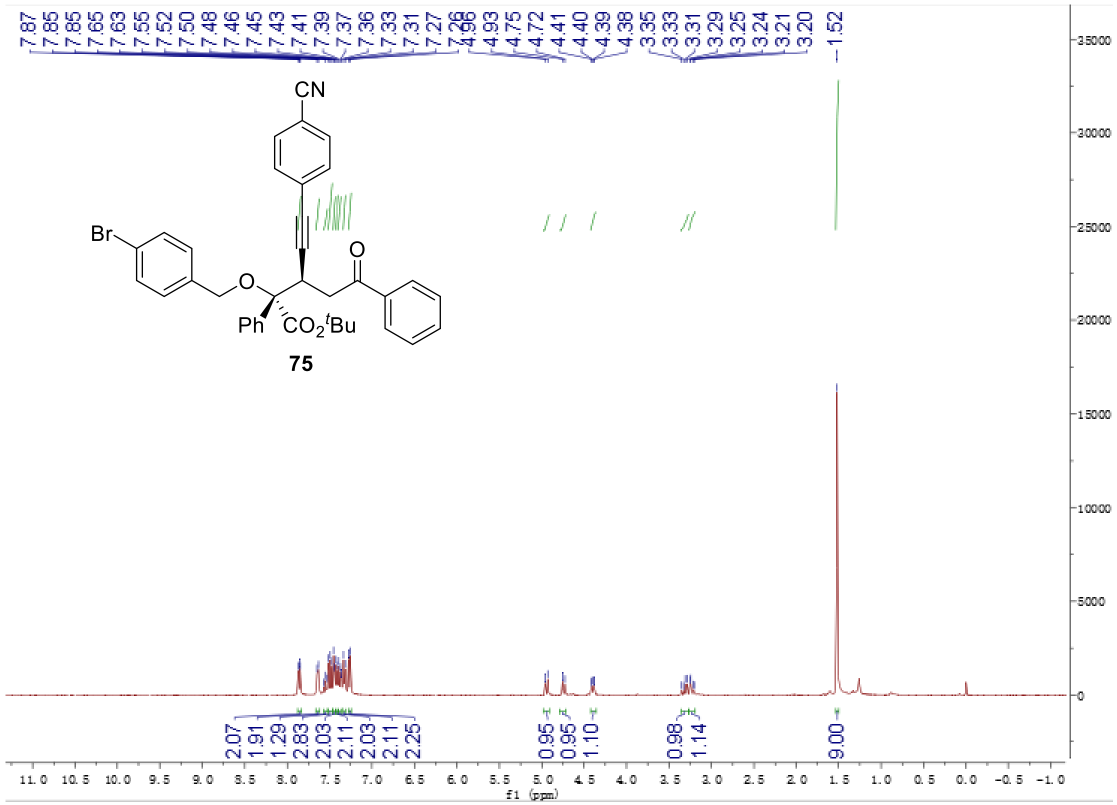

Supplementary Figure 432. ^{1}H NMR (400 MHz, $CDCl_3$) spectrum of **75**.

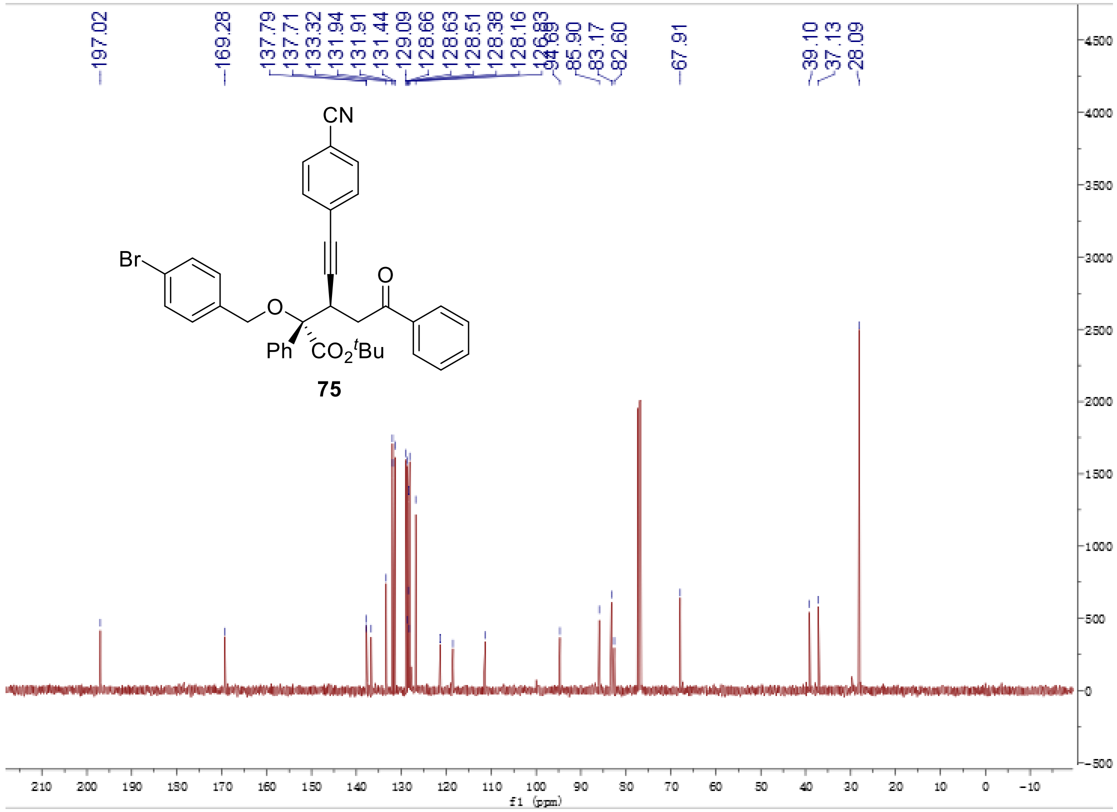

Supplementary Figure 433. ^{13}C NMR (101 MHz, $CDCl_3$) spectrum of **75**.

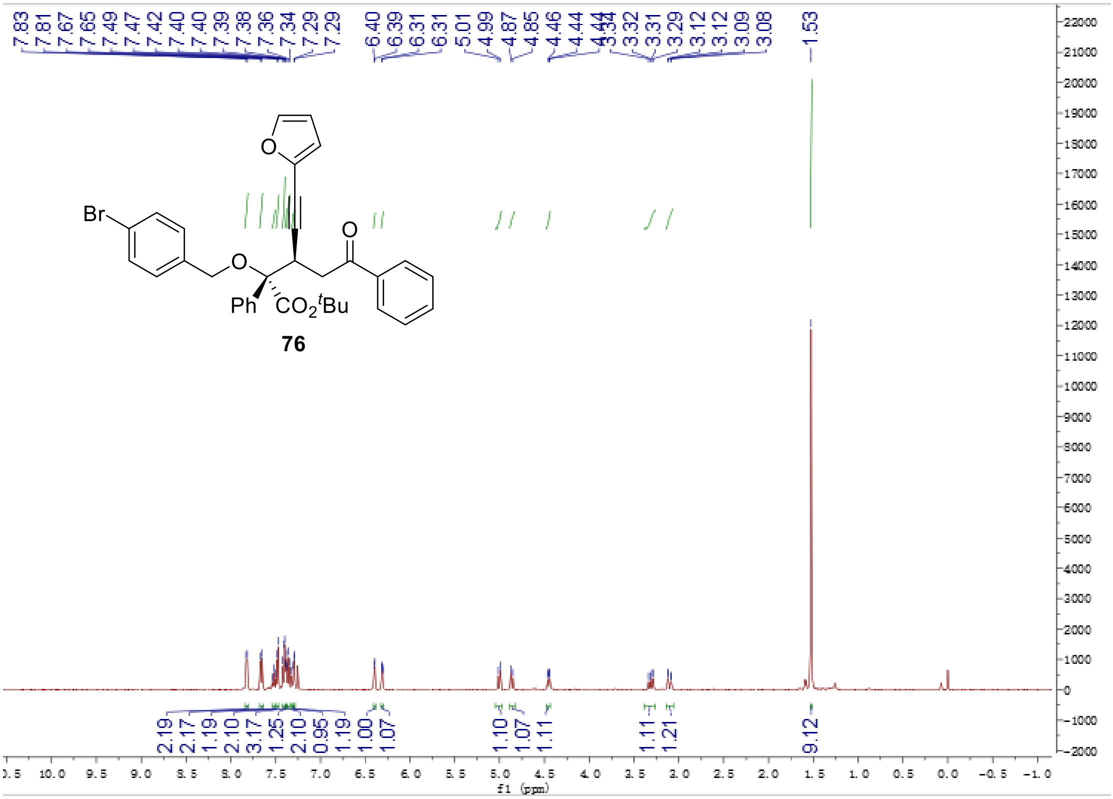

Supplementary Figure 434. ^{1}H NMR (500 MHz, $CDCl_3$) spectrum of **76**.

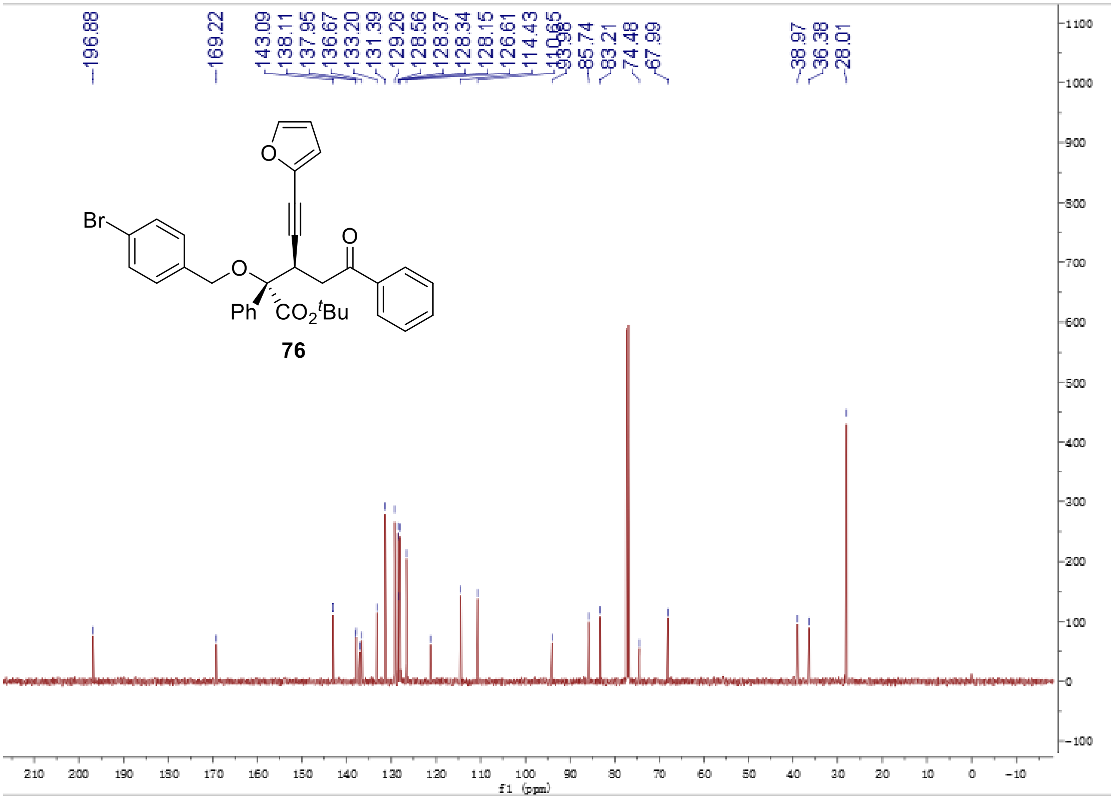

Supplementary Figure 435. ^{13}C NMR (126 MHz, $CDCl_3$) spectrum of **76**.

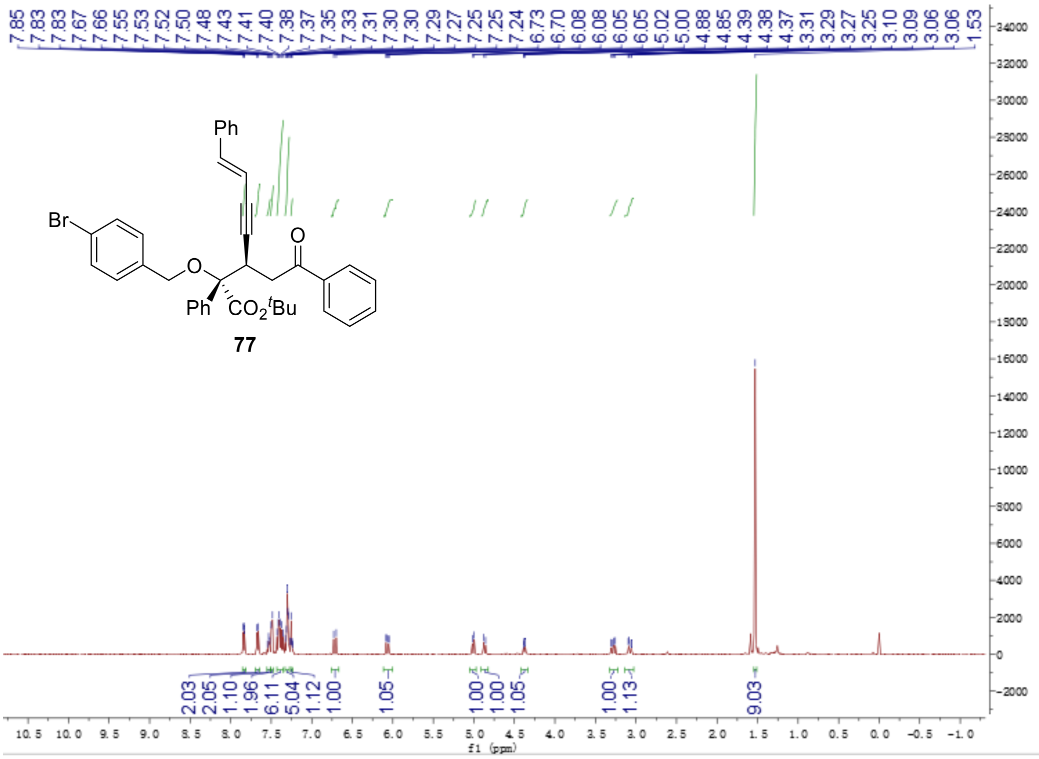

Supplementary Figure 436. ^{1}H NMR (500 MHz, $CDCl_3$) spectrum of **77**.

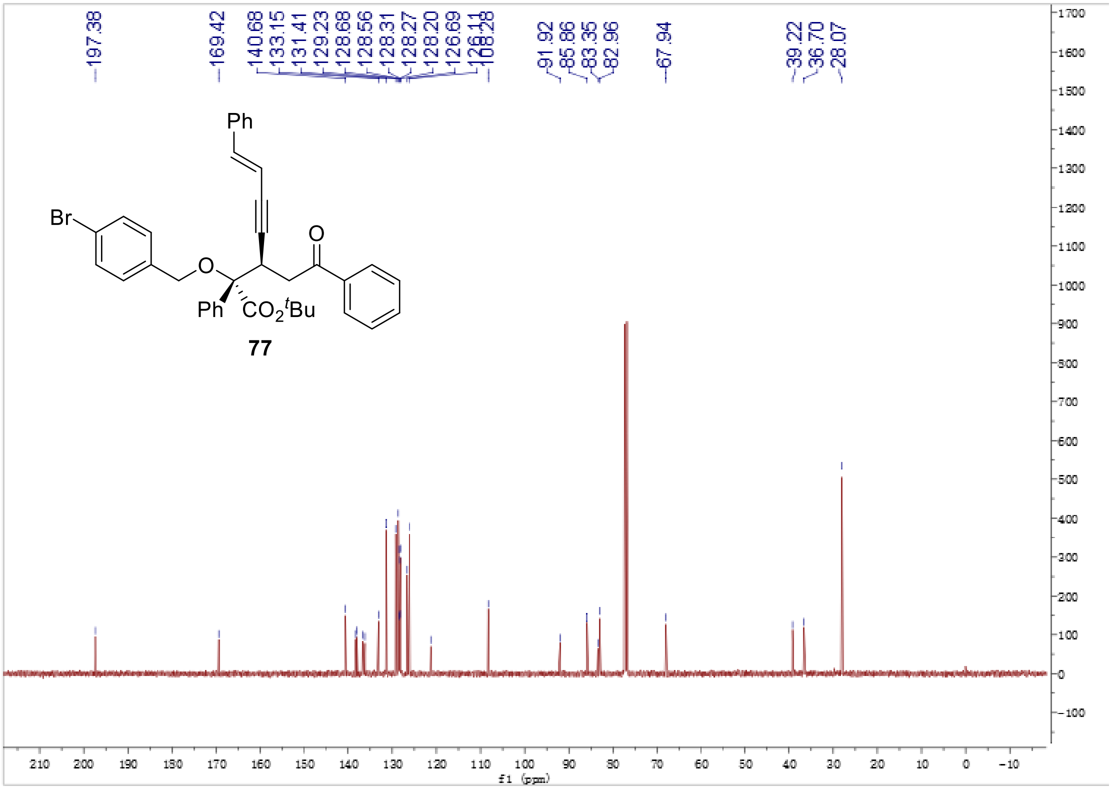

Supplementary Figure 437. ^{13}C NMR (126 MHz, $CDCl_3$) spectrum of **77**.

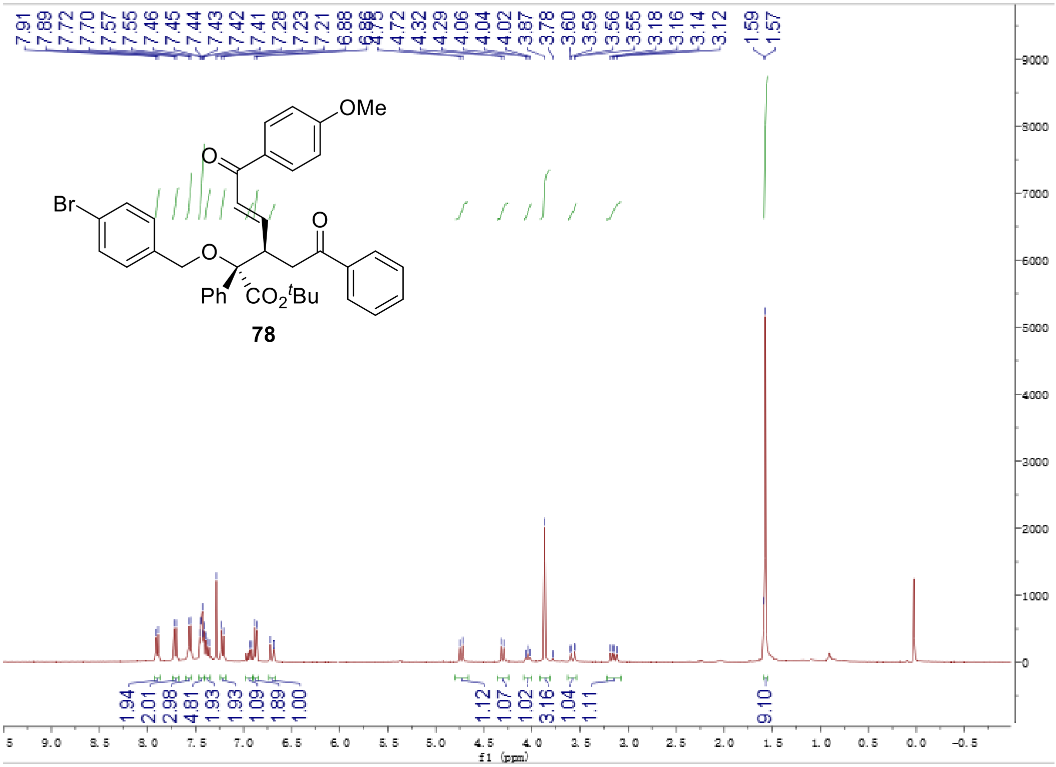

Supplementary Figure 438. ^{1}H NMR (400 MHz, $CDCl_3$) spectrum of **78**.

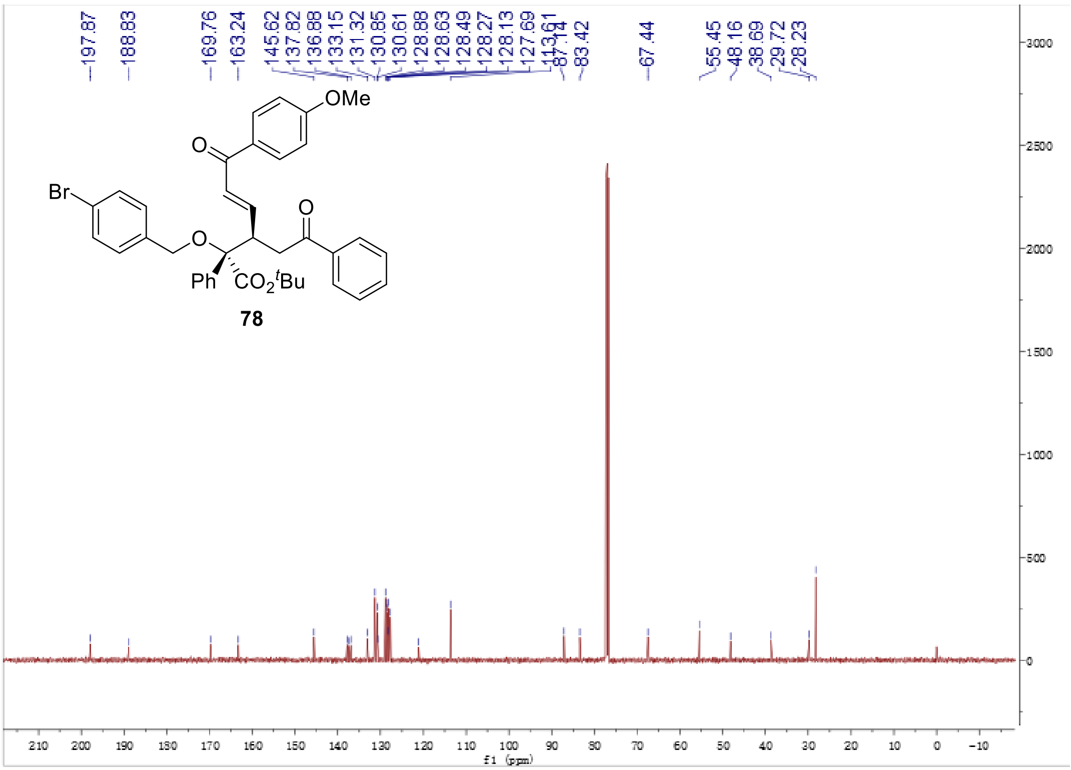

Supplementary Figure 439. ^{13}C NMR (101 MHz, $CDCl_3$) spectrum of **78**.

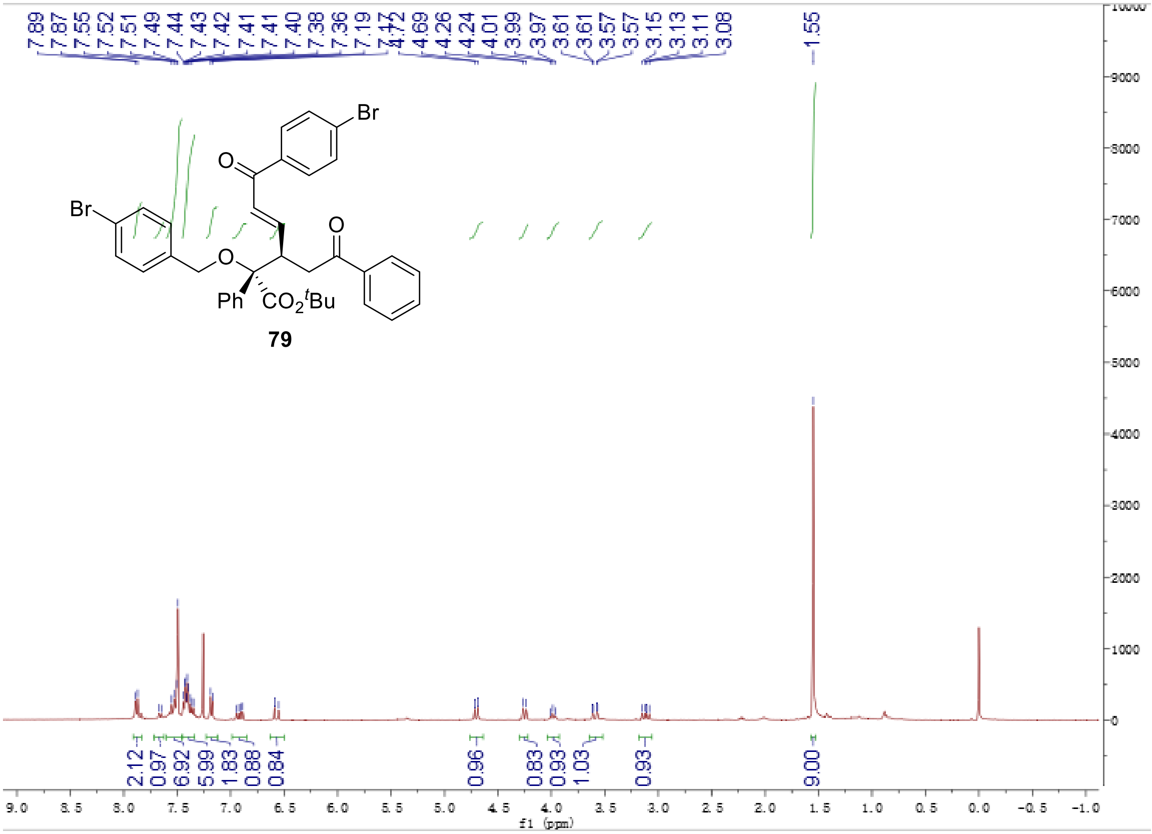

Supplementary Figure 440. ^{1}H NMR (400 MHz, $CDCl_3$) spectrum of **79**.

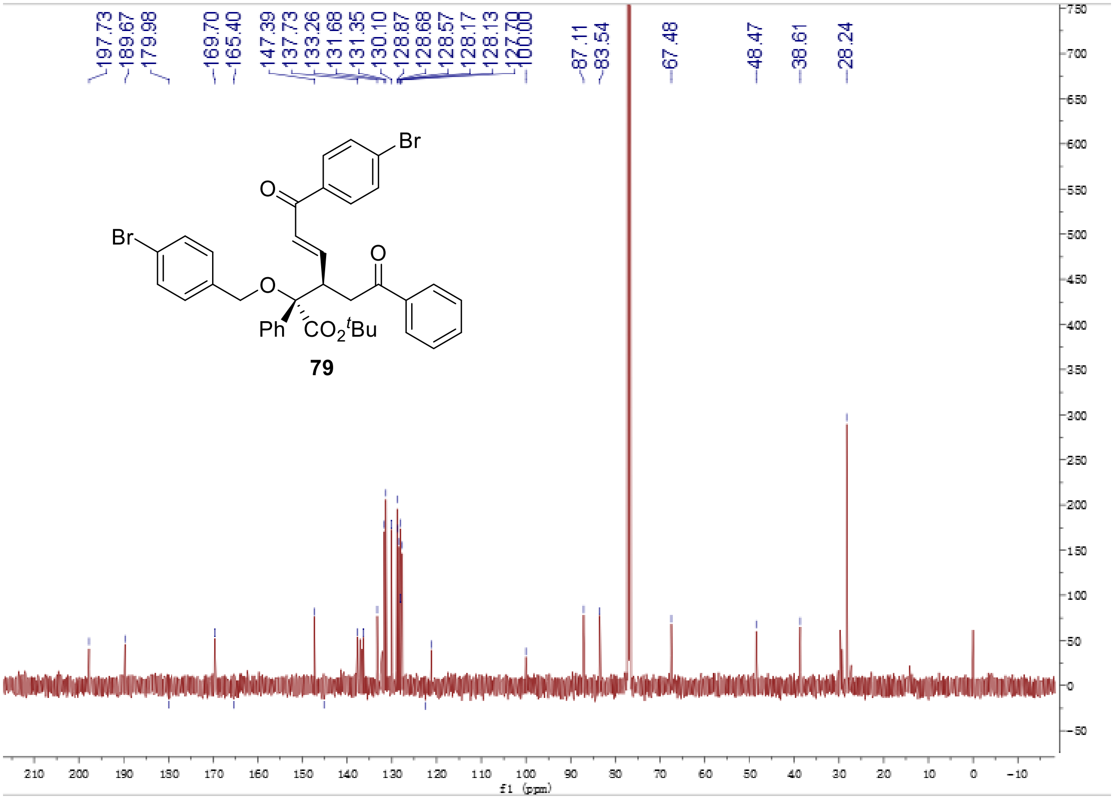

Supplementary Figure 441. ^{13}C NMR (101 MHz, $CDCl_3$) spectrum of **79**.

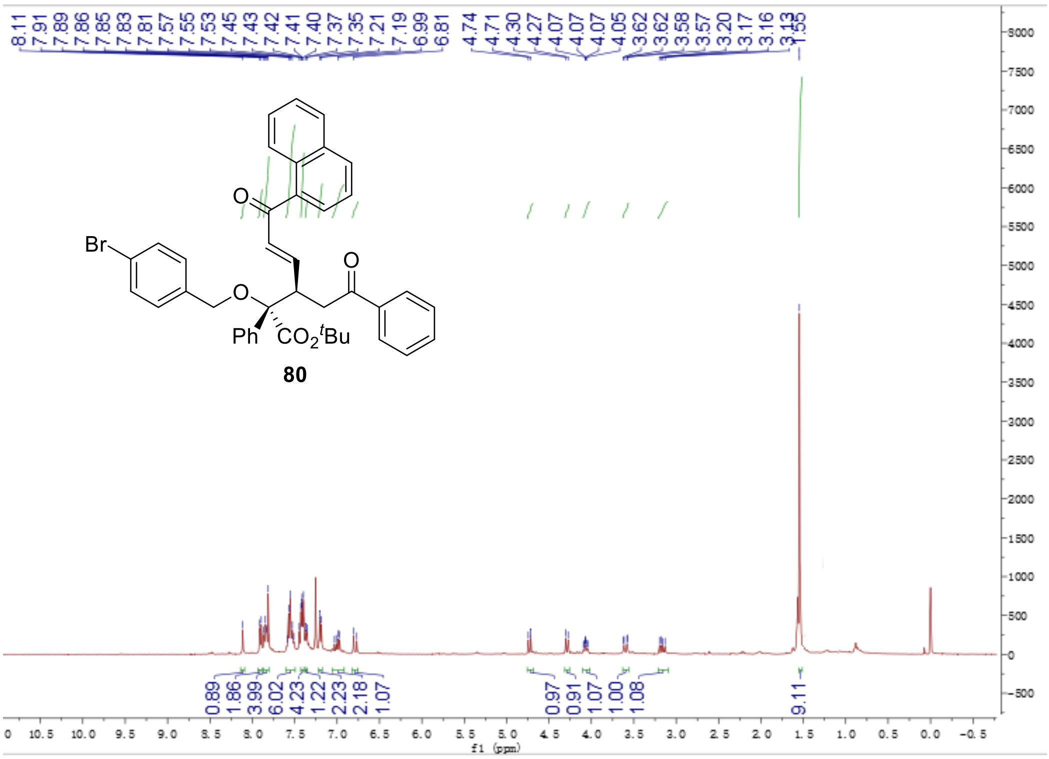

Supplementary Figure 442. ^{1}H NMR (400 MHz, $CDCl_3$) spectrum of **80**.

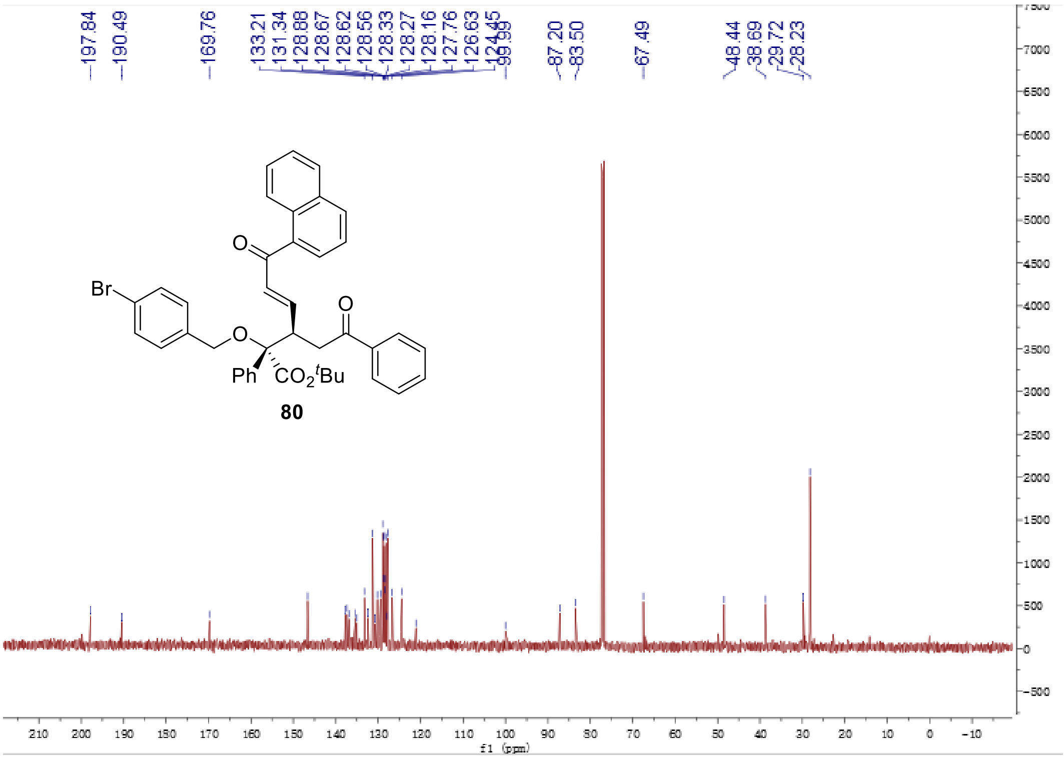

Supplementary Figure 443. ^{13}C NMR (101 MHz, $CDCl_3$) spectrum of **80**.

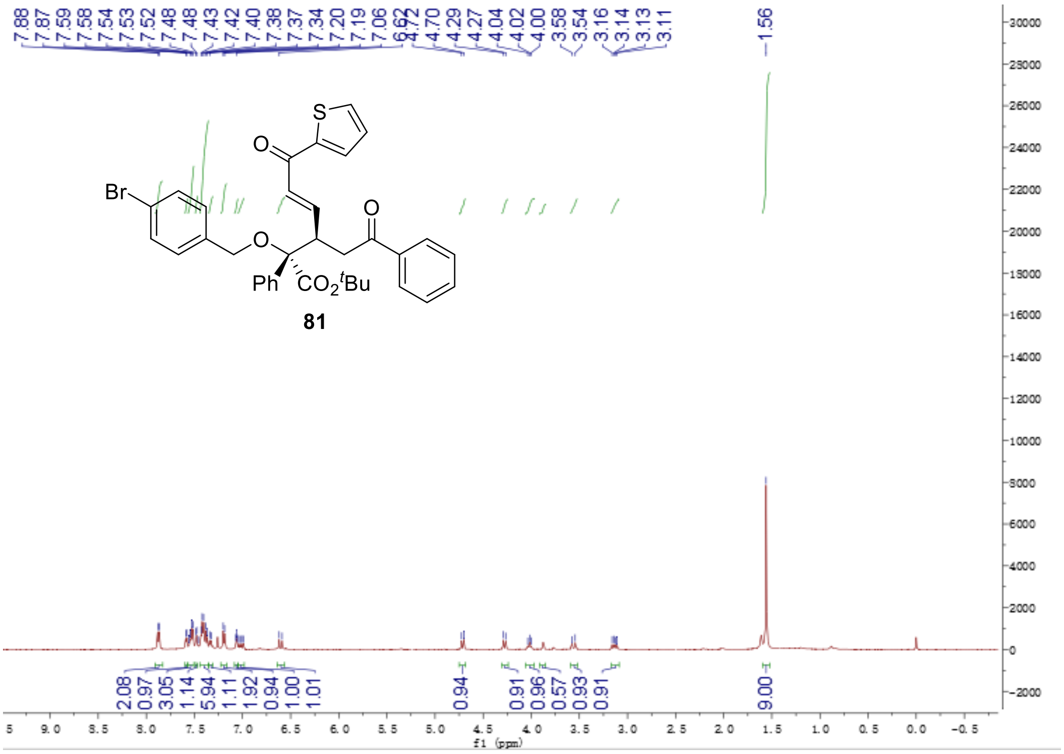

Supplementary Figure 444. ^{1}H NMR (500 MHz, $CDCl_3$) spectrum of **81**.

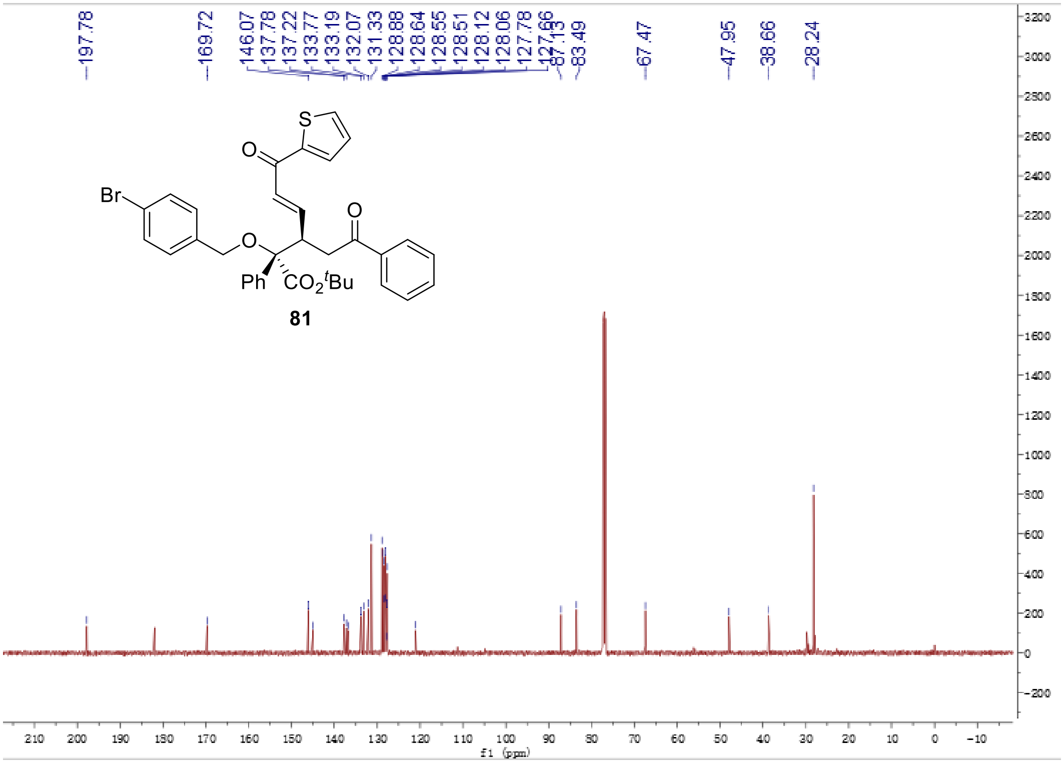

Supplementary Figure 445. ^{13}C NMR (126 MHz, $CDCl_3$) spectrum of **81**.

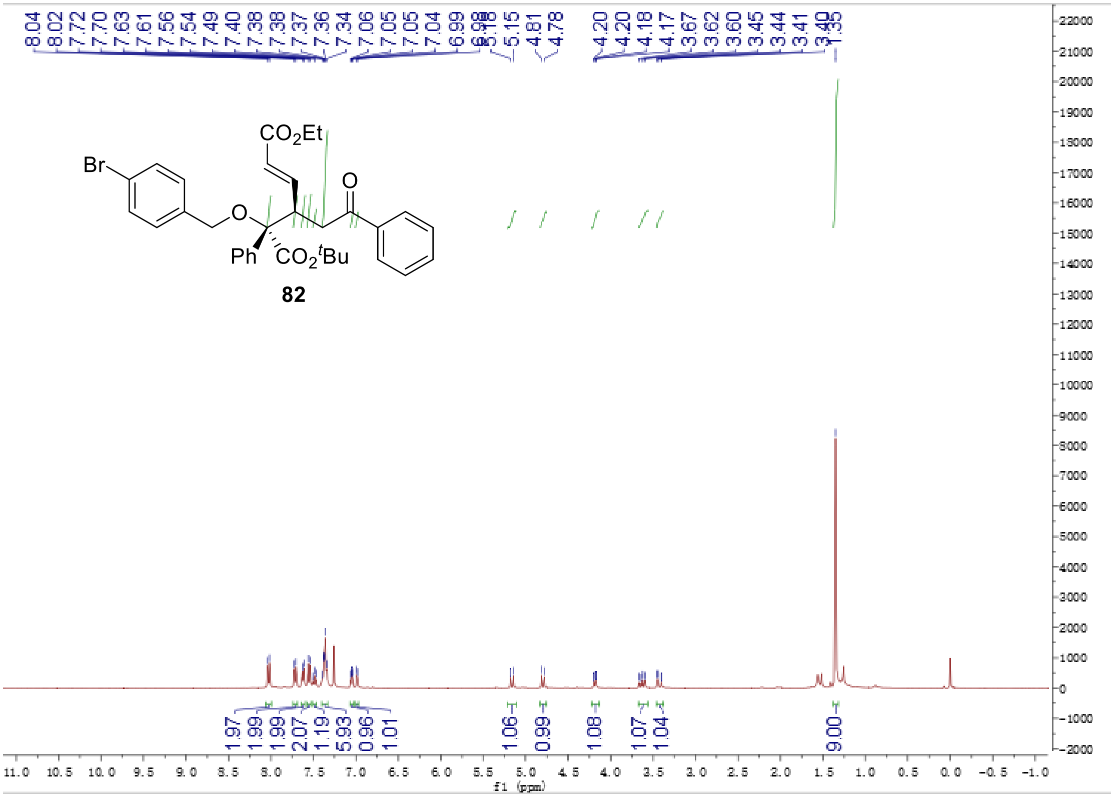

Supplementary Figure 446. ^{1}H NMR (500 MHz, $CDCl_3$) spectrum of **82**.

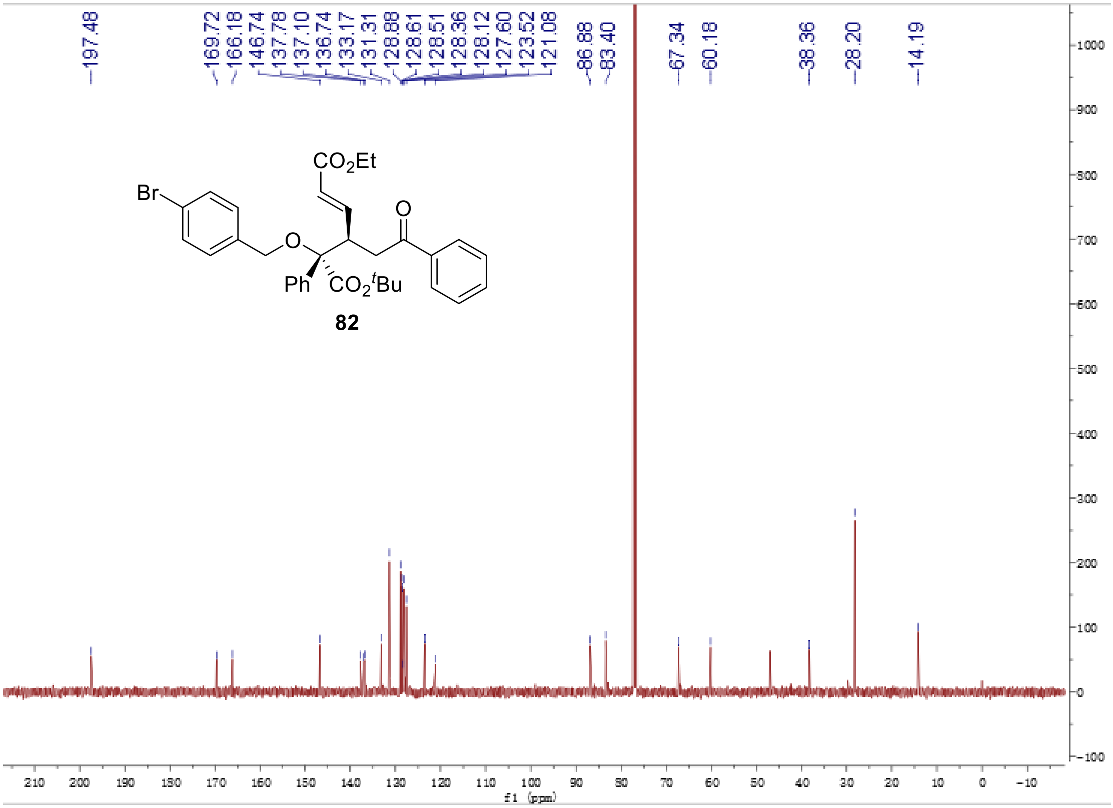

Supplementary Figure 447. ^{13}C NMR (126 MHz, $CDCl_3$) spectrum of **82**.

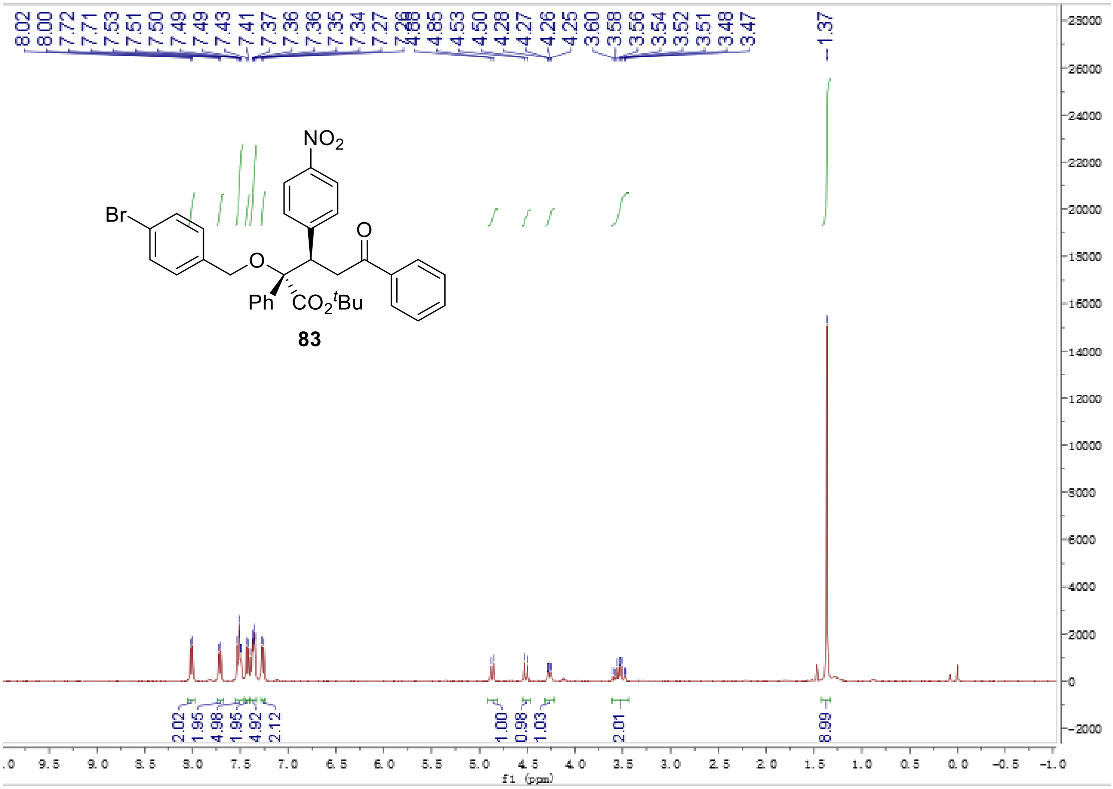

Supplementary Figure 448. ^{1}H NMR (400 MHz, $CDCl_3$) spectrum of **83**.

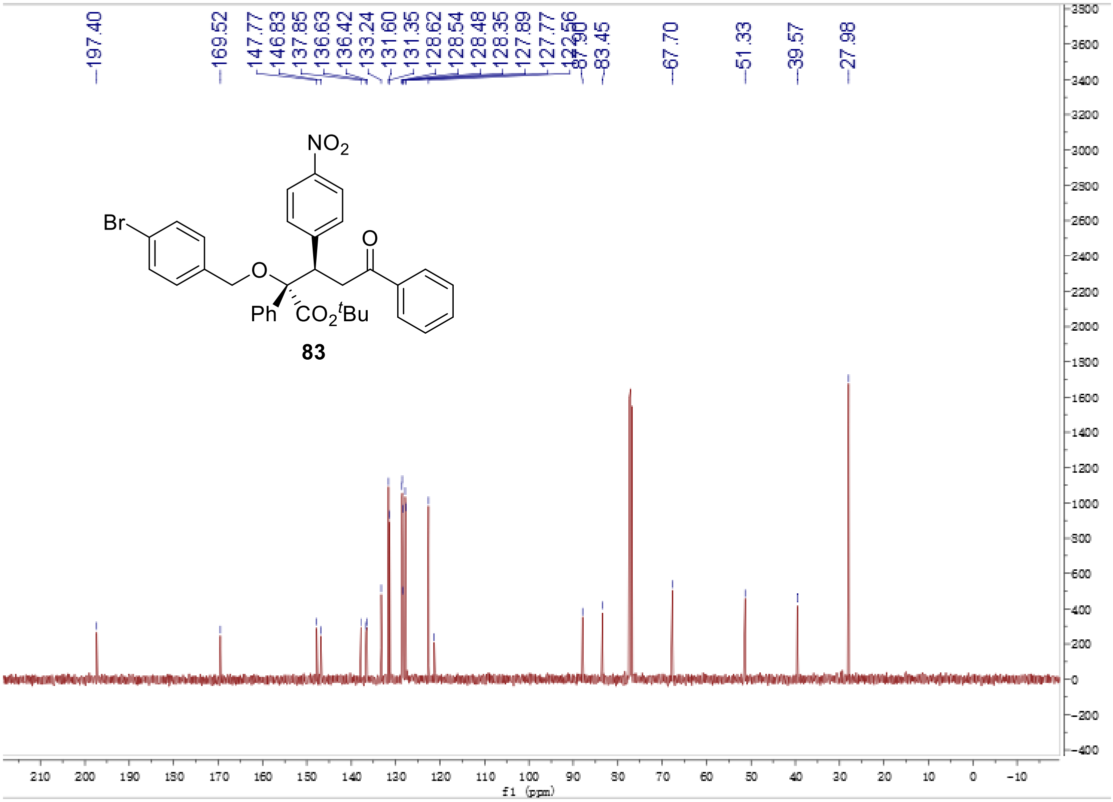

Supplementary Figure 449. ^{13}C NMR (101 MHz, $CDCl_3$) spectrum of **83**.

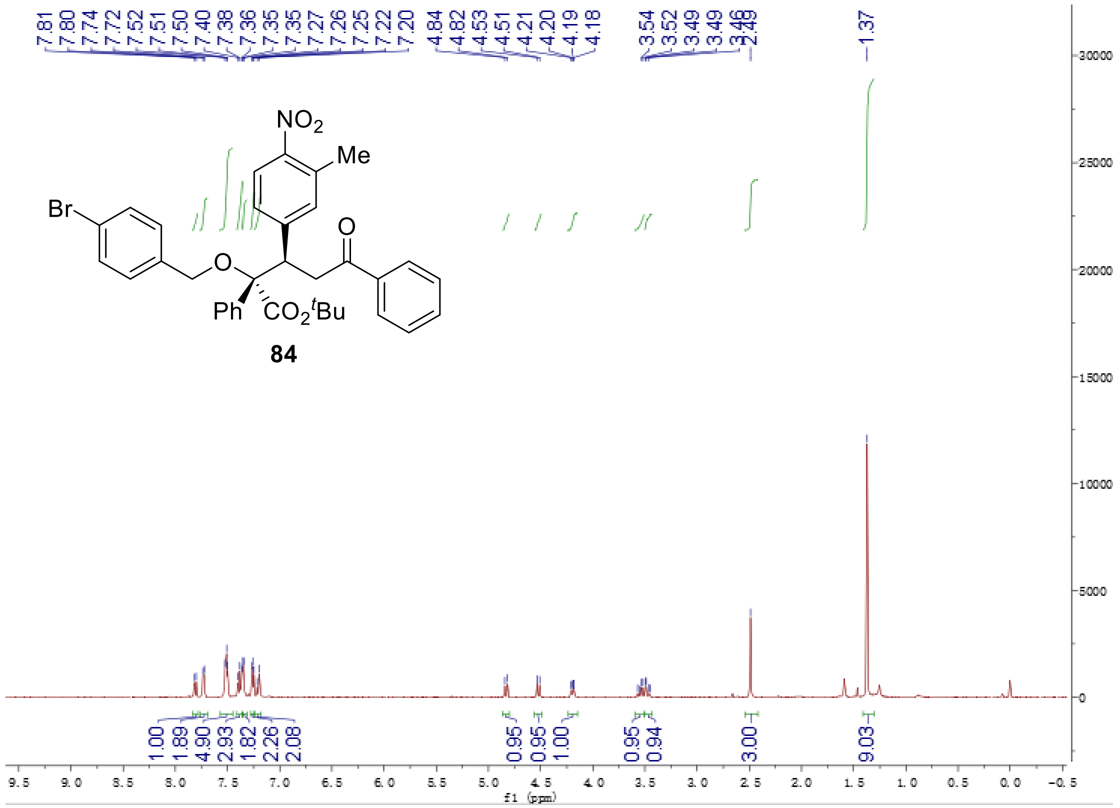

Supplementary Figure 450. ^{1}H NMR (500 MHz, $CDCl_3$) spectrum of **84**.

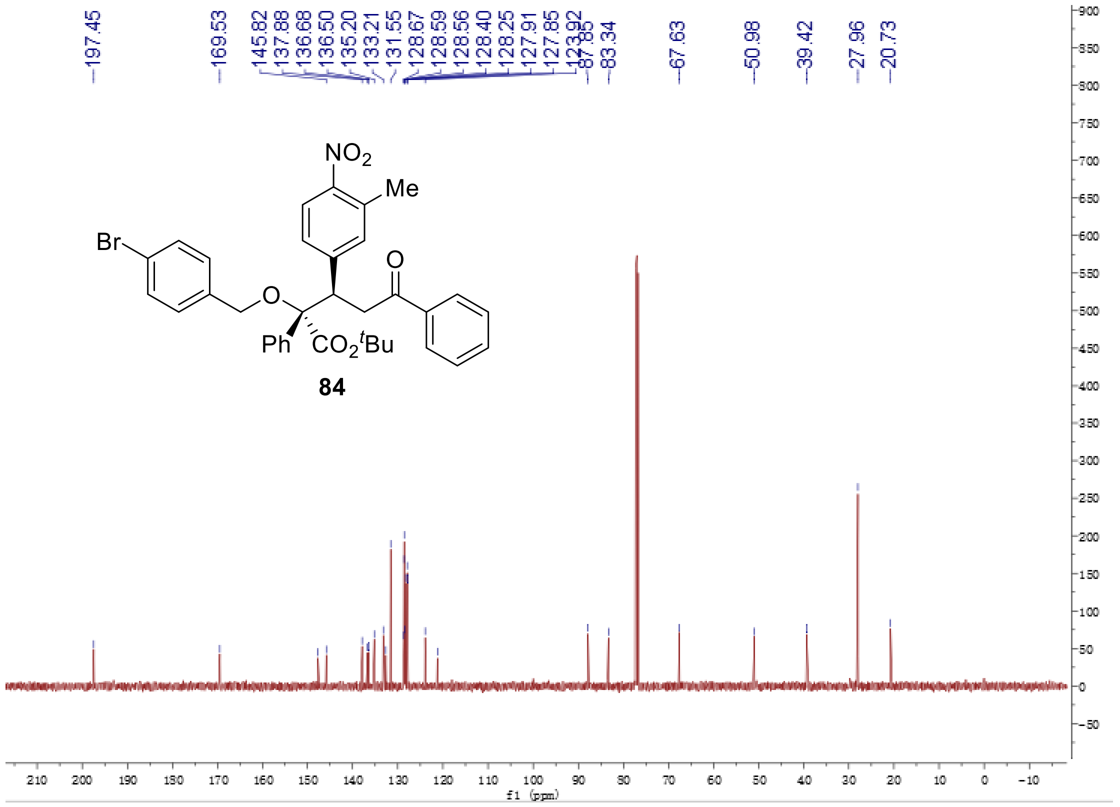

Supplementary Figure 451. ^{13}C NMR (126 MHz, $CDCl_3$) spectrum of **84**.

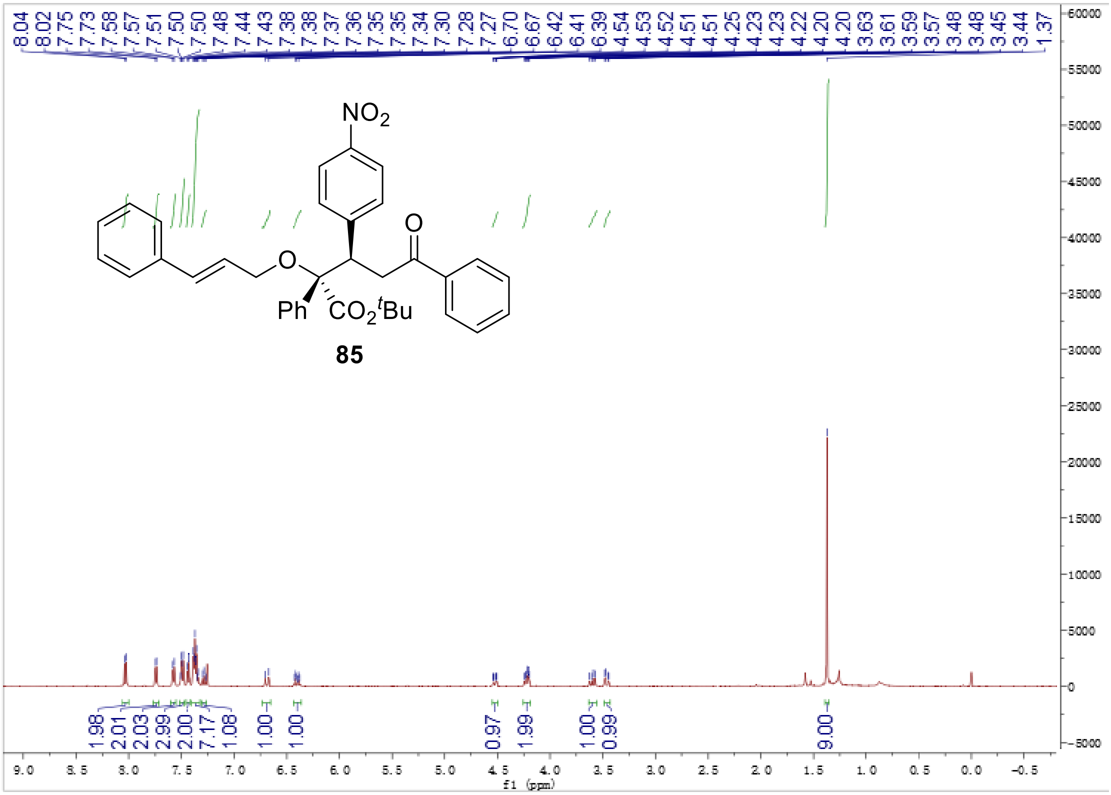

Supplementary Figure 452. ^{1}H NMR (500 MHz, $CDCl_3$) spectrum of **85**.

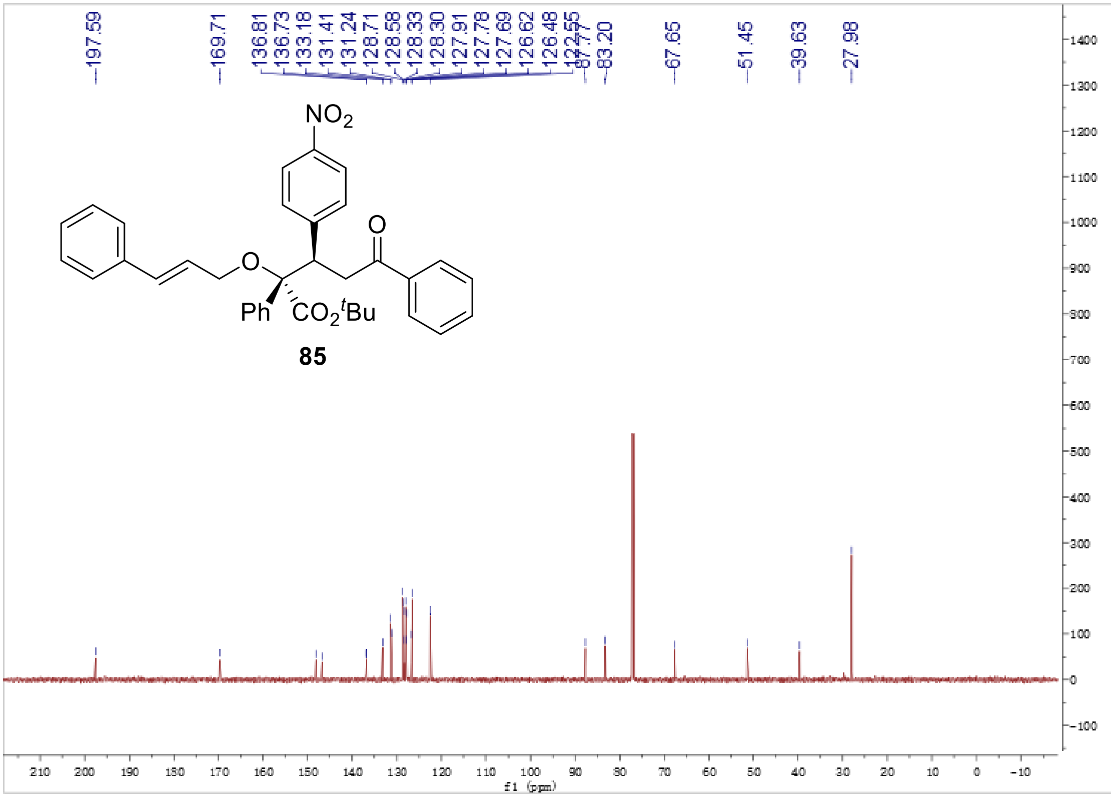

Supplementary Figure 453. ^{13}C NMR (126 MHz, $CDCl_3$) spectrum of **85**.

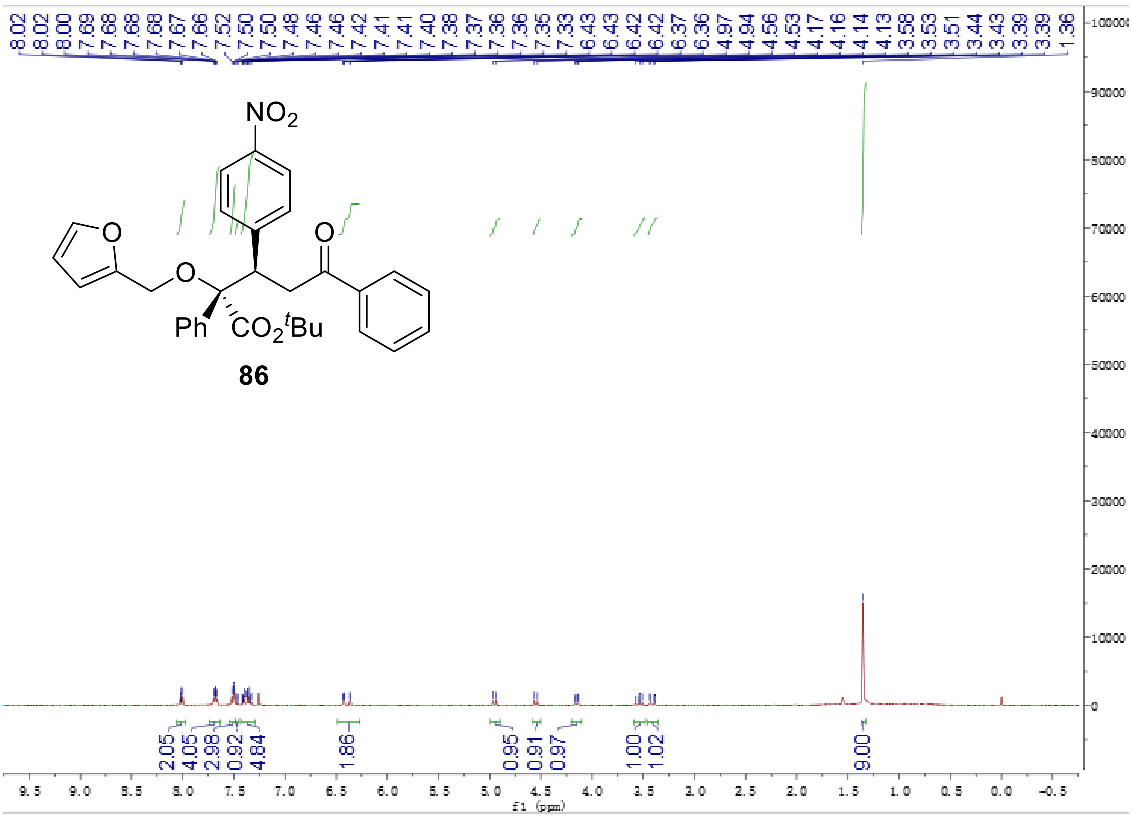

Supplementary Figure 454. ^{1}H NMR (400 MHz, $CDCl_3$) spectrum of **86**.

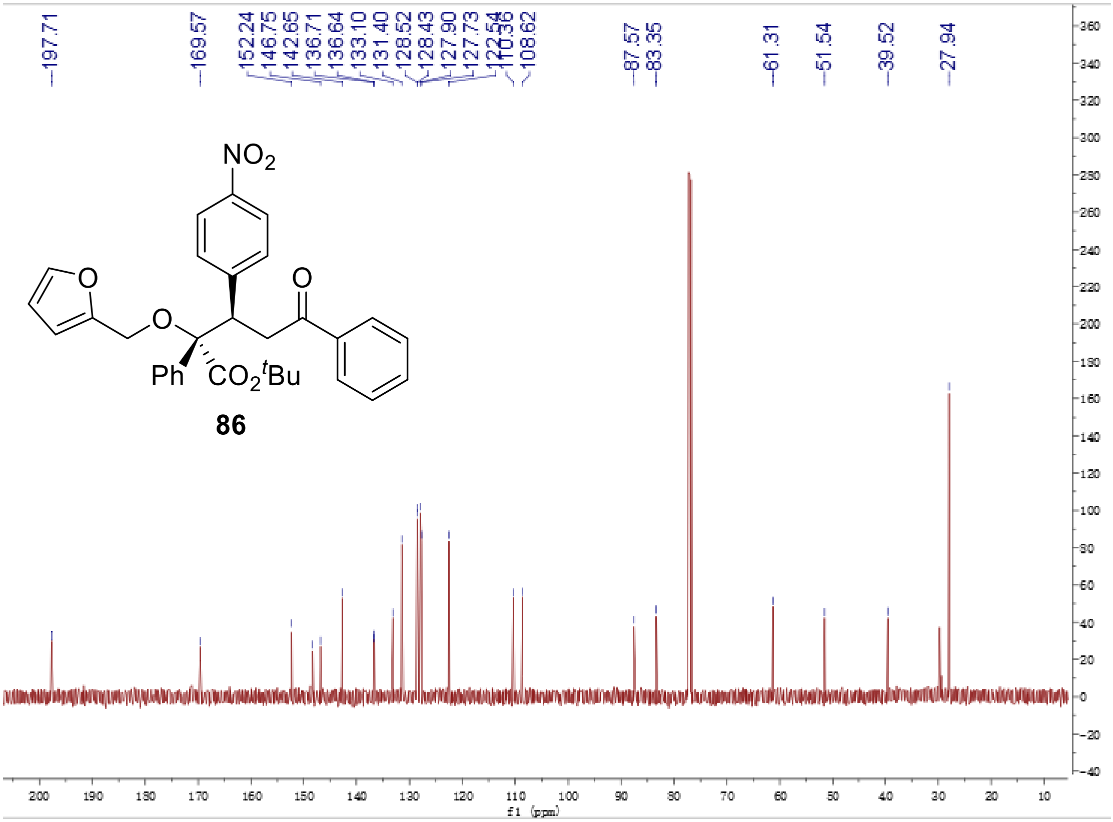

Supplementary Figure 455. ^{13}C NMR (101 MHz, $CDCl_3$) spectrum of **86**.

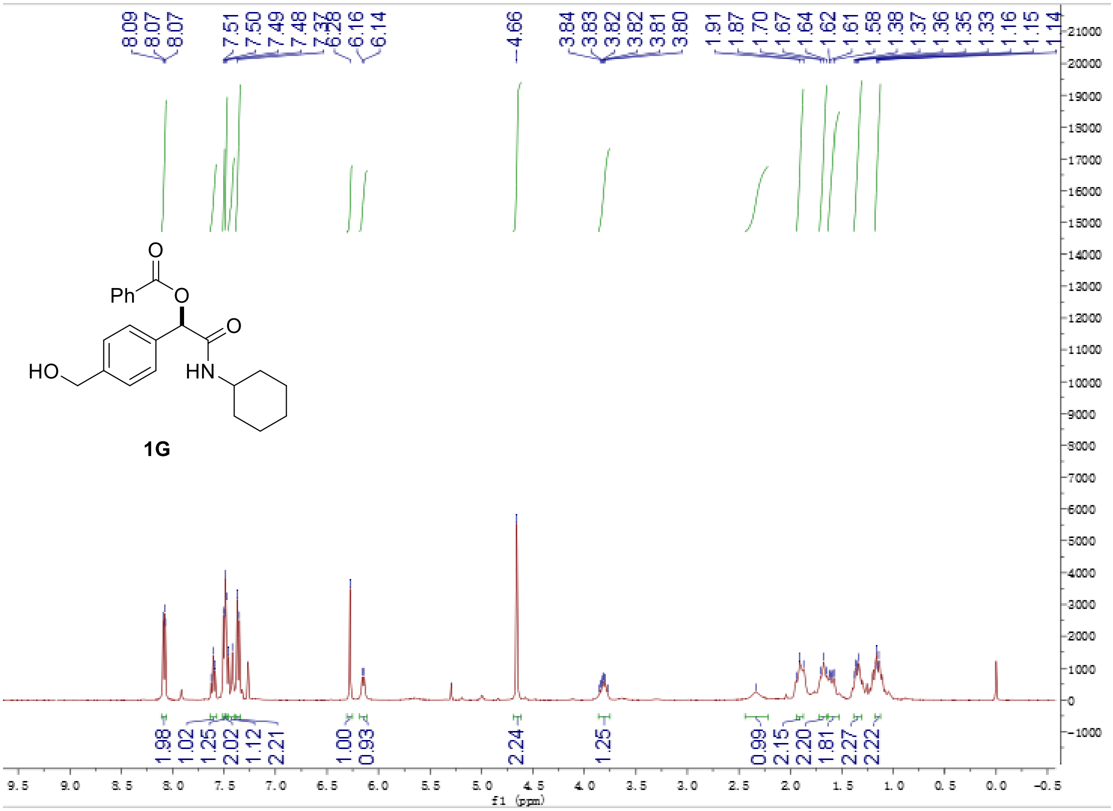

Supplementary Figure 456. ^{1}H NMR (400 MHz, $CDCl_3$) spectrum of **1G**.

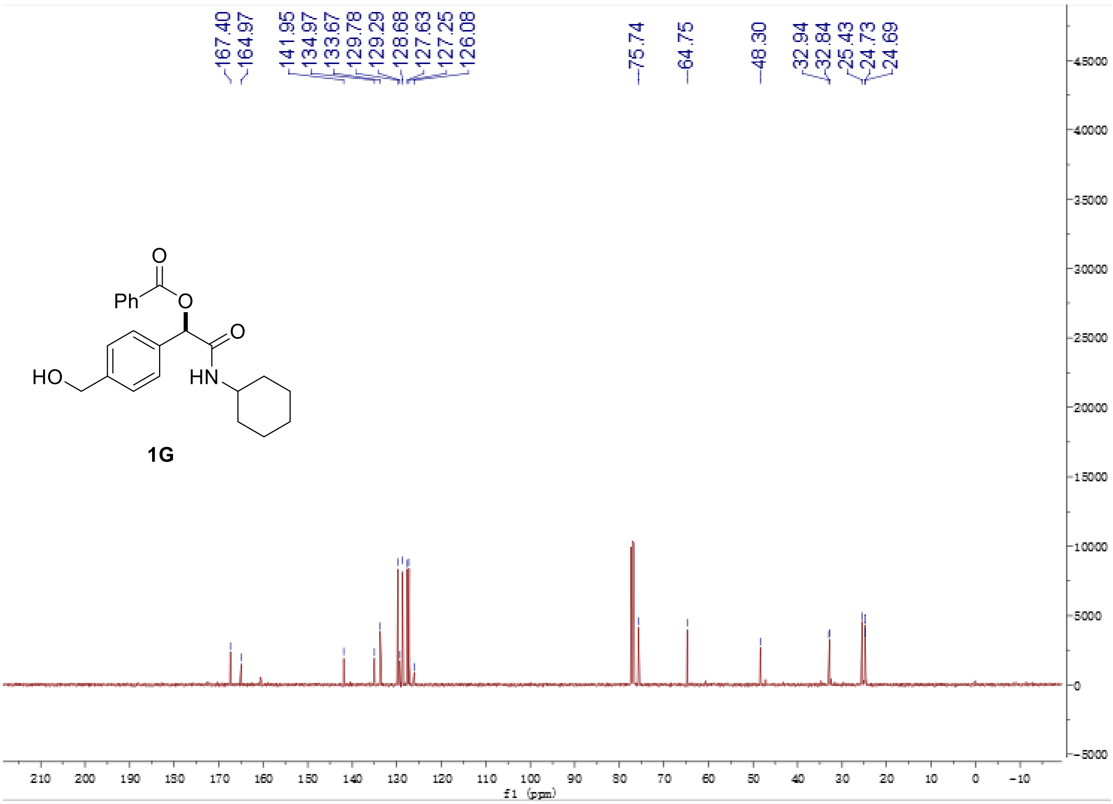

Supplementary Figure 457. ^{13}C NMR (101 MHz, $CDCl_3$) spectrum of **1G**.

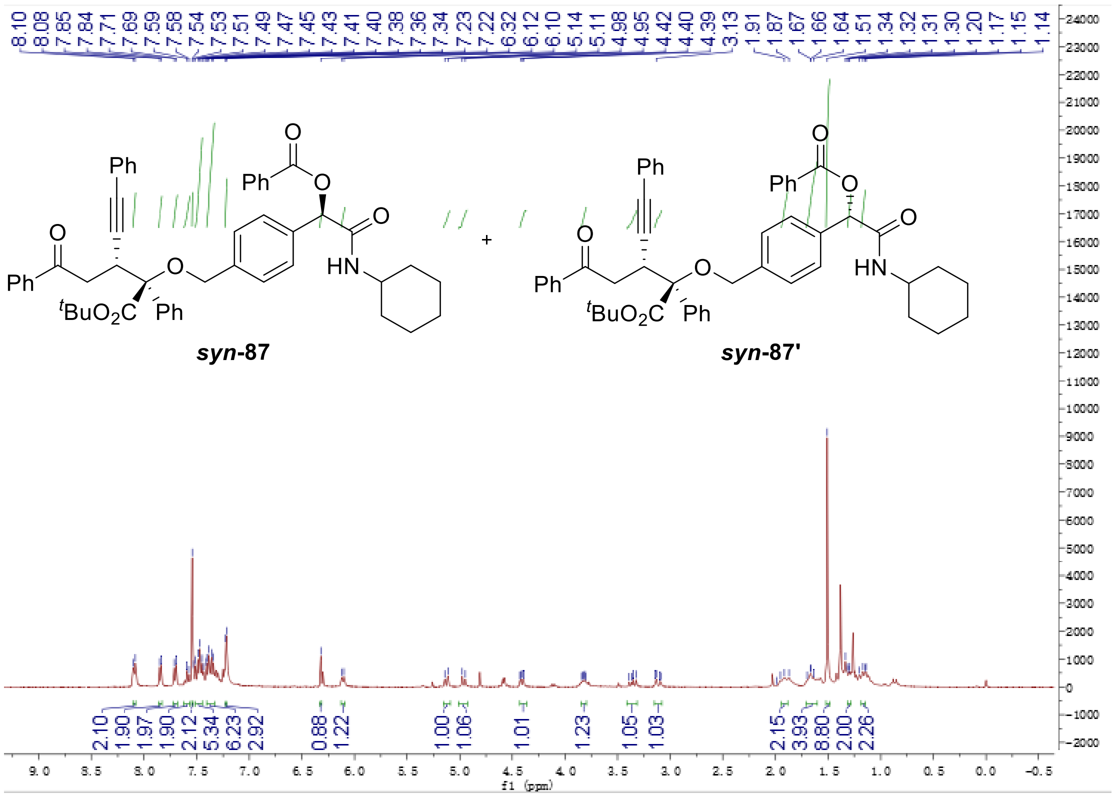

Supplementary Figure 458. ^{1}H NMR (400 MHz, $CDCl_3$) spectrum of **87**.

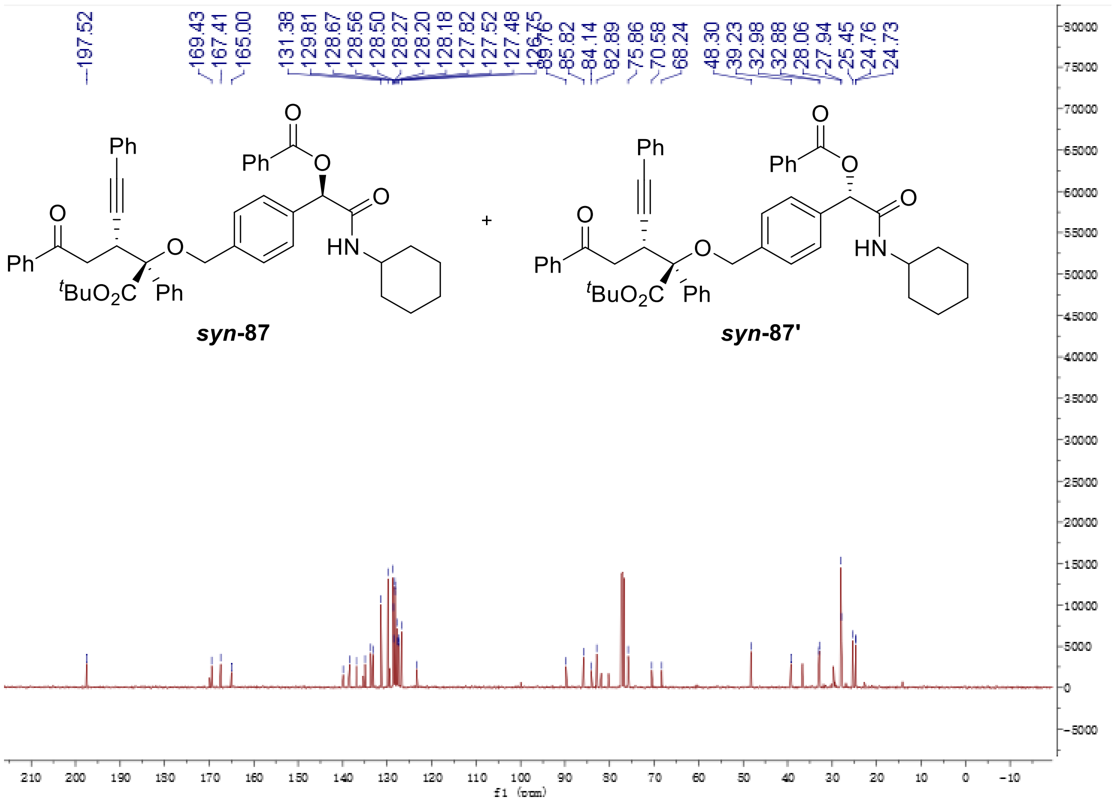

Supplementary Figure 459. ^{13}C NMR (101 MHz, $CDCl_3$) spectrum of **87**.

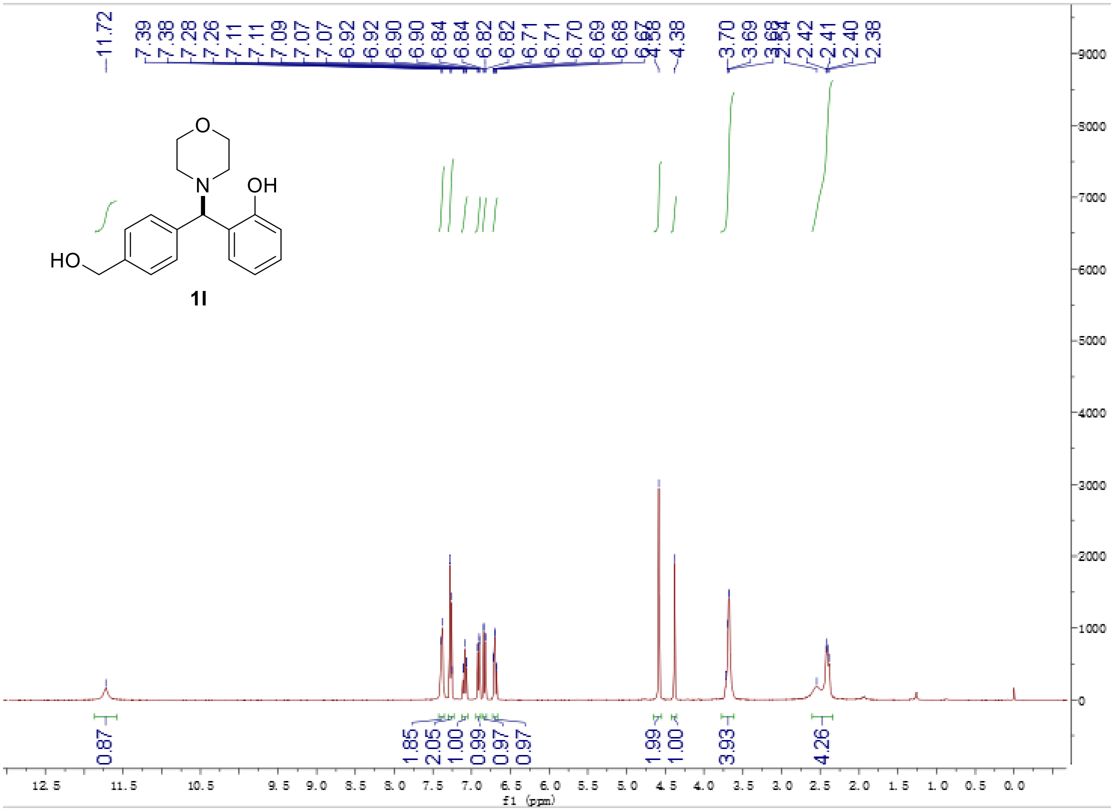

Supplementary Figure 460. ^{1}H NMR (400 MHz, $CDCl_3$) spectrum of **1l**.

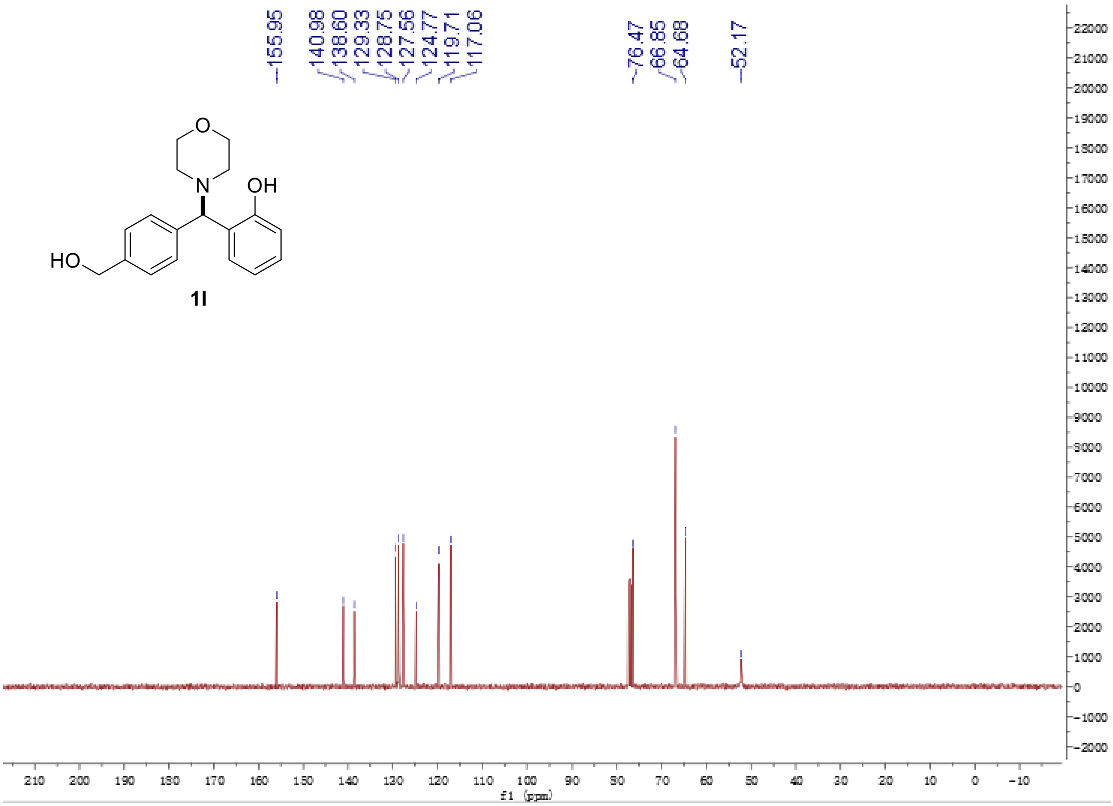

Supplementary Figure 461. ^{13}C NMR (101 MHz, $CDCl_3$) spectrum of **1l**.

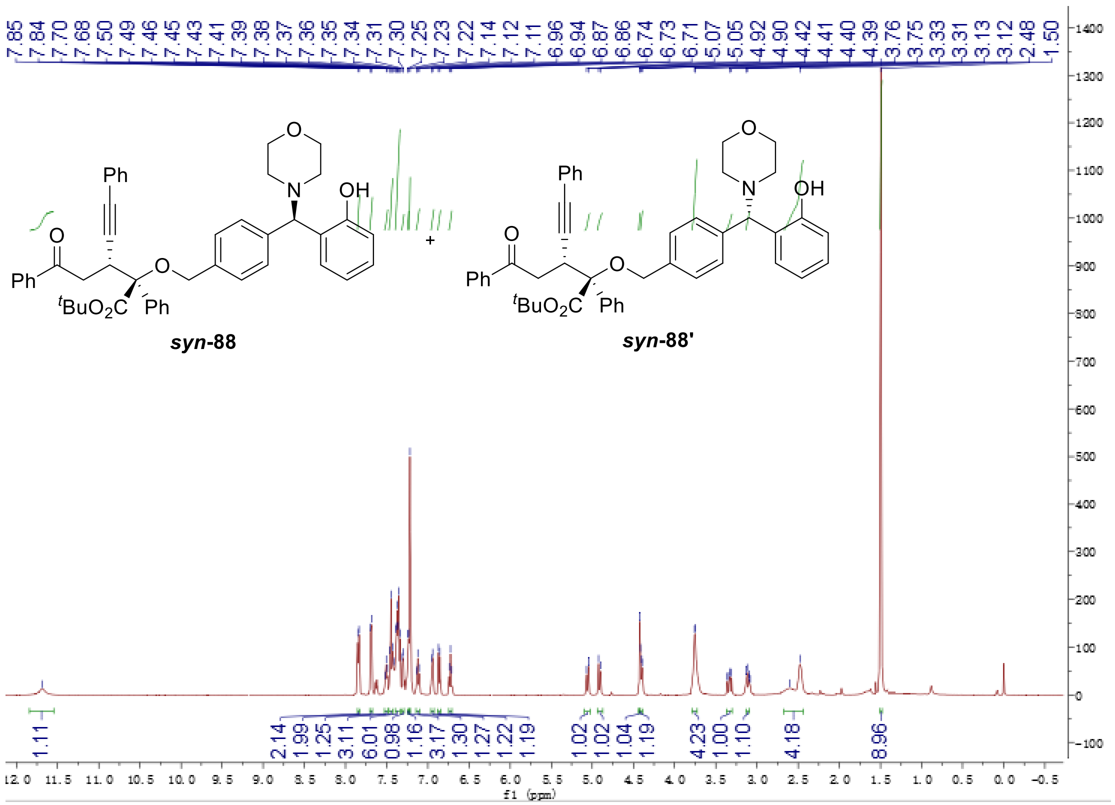

Supplementary Figure 462. ^{1}H NMR (500 MHz, $CDCl_3$) spectrum of **88**.

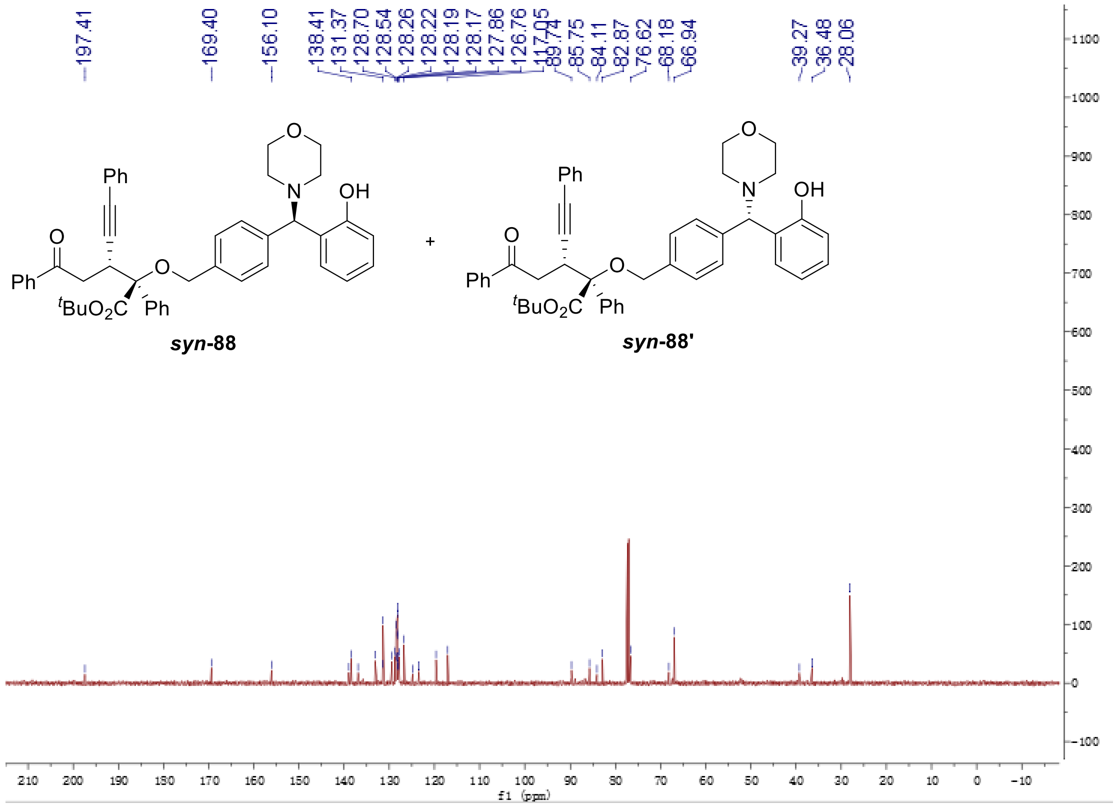

Supplementary Figure 463. ^{13}C NMR (126 MHz, $CDCl_3$) spectrum of **88**.

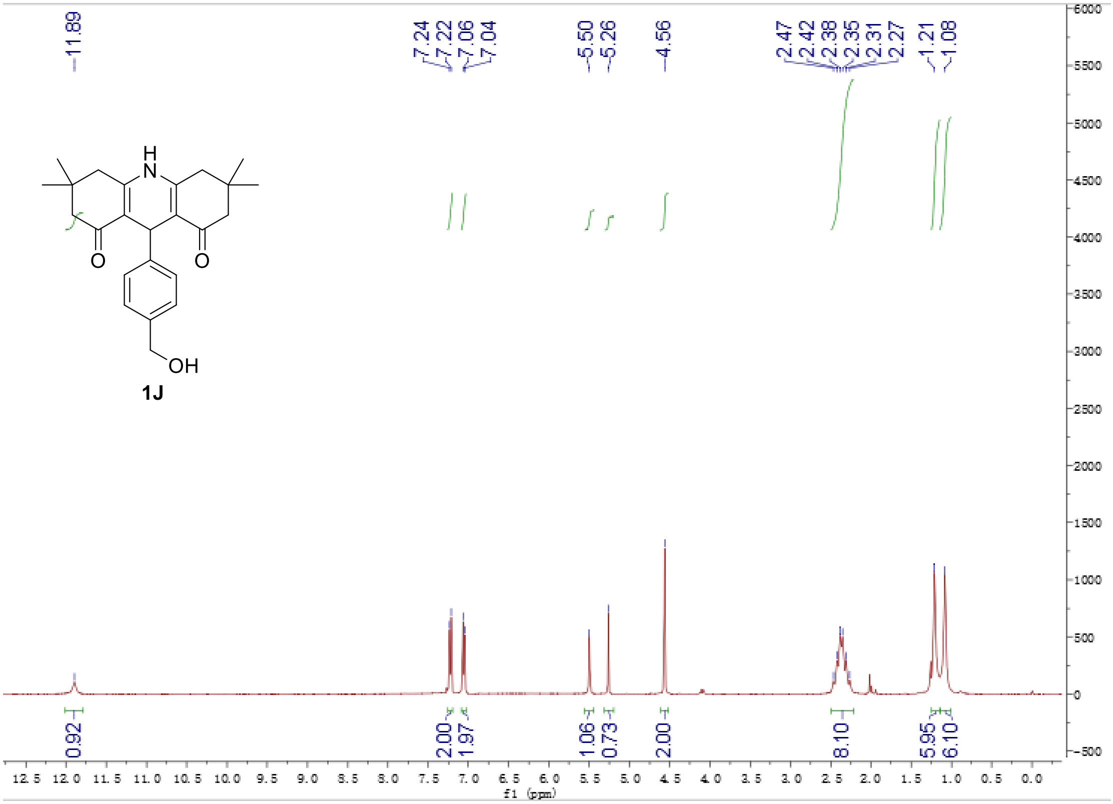

Supplementary Figure 564. ^{1}H NMR (400 MHz, $CDCl_3$) spectrum of **1J**.

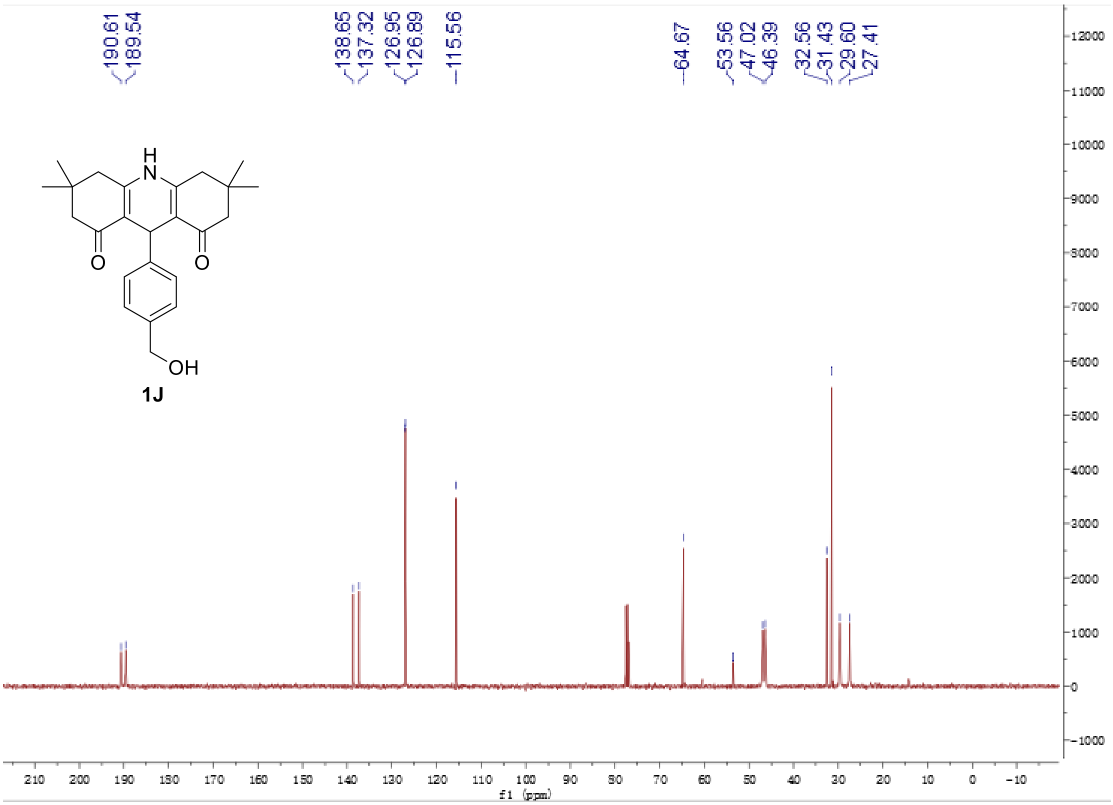

Supplementary Figure 465. ^{13}C NMR (101 MHz, $CDCl_3$) spectrum of **1J**.

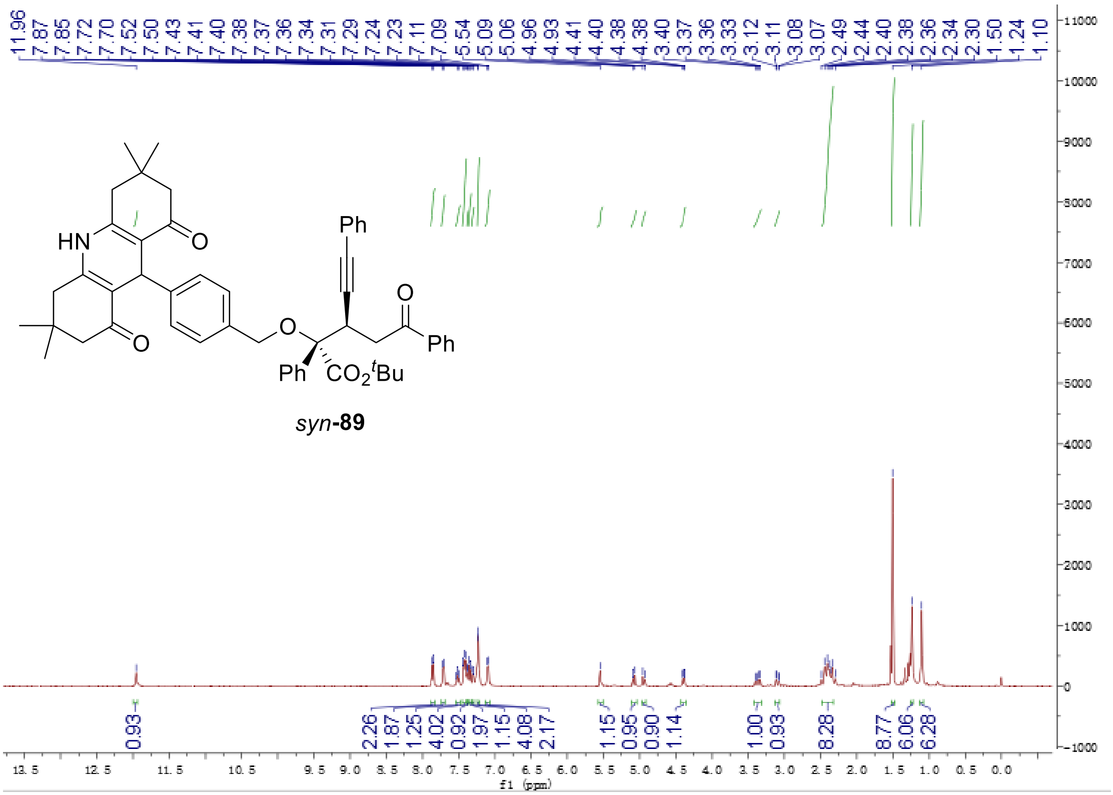

Supplementary Figure 466. ^{1}H NMR (400 MHz, $CDCl_3$) spectrum of **89**.

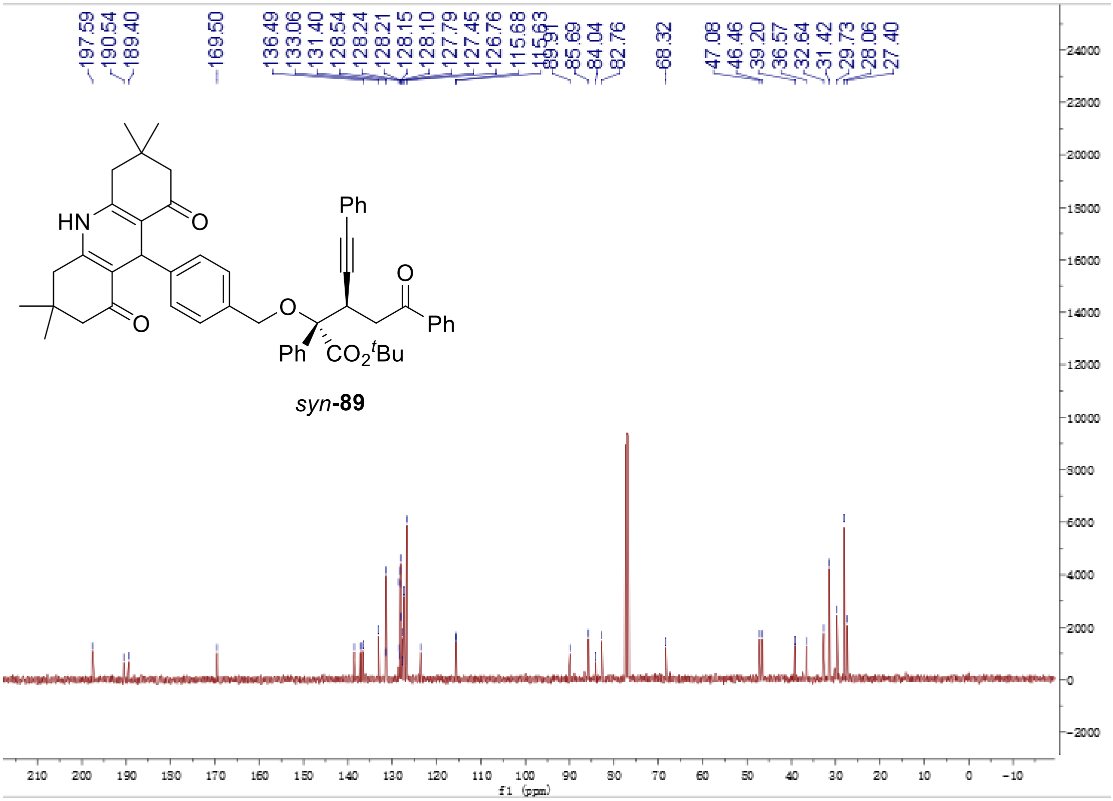

Supplementary Figure 467. ^{13}C NMR (101 MHz, $CDCl_3$) spectrum of **89**.

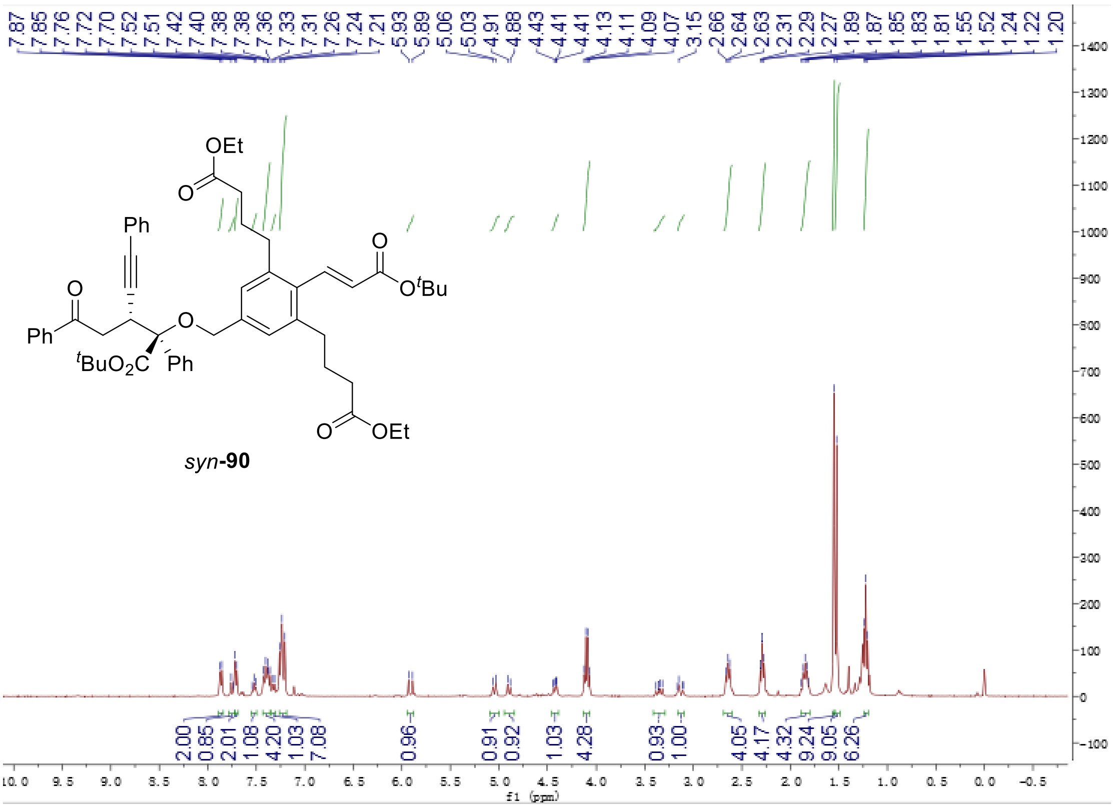

Supplementary Figure 468. ^{1}H NMR (400 MHz, $CDCl_3$) spectrum of **90**.

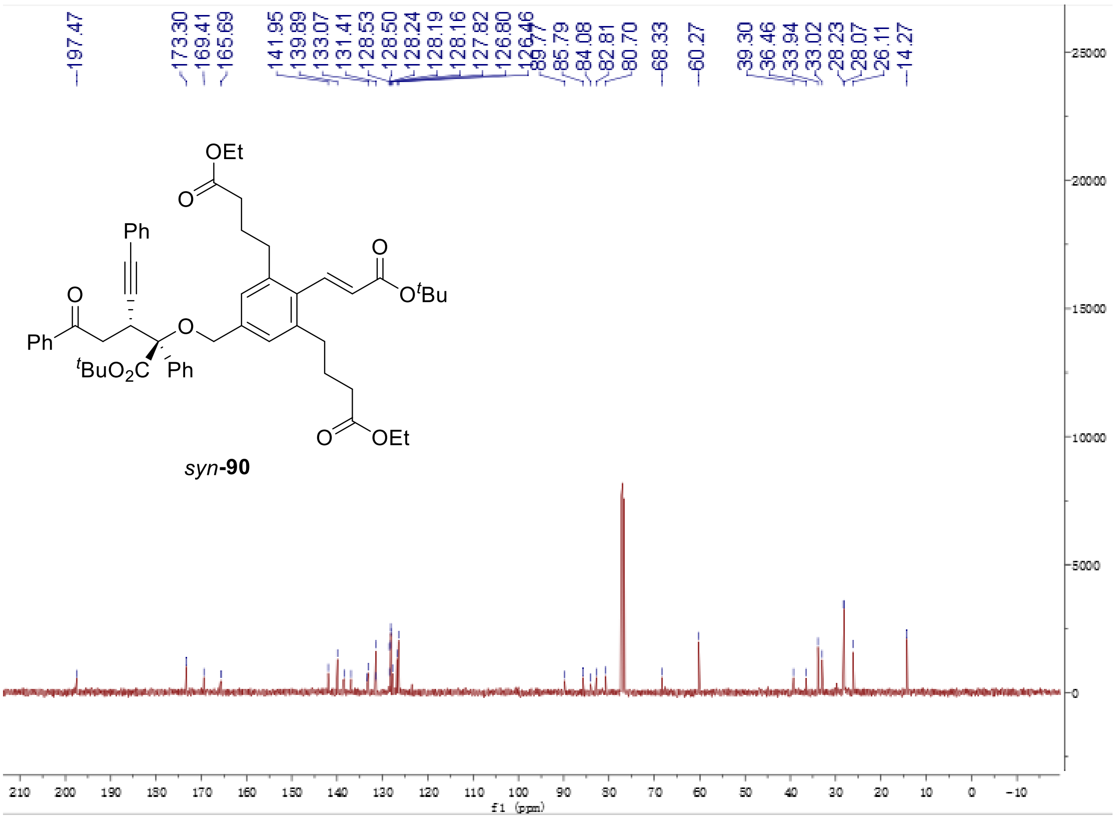

Supplementary Figure 469. ^{13}C NMR (101 MHz, $CDCl_3$) spectrum of **90**.

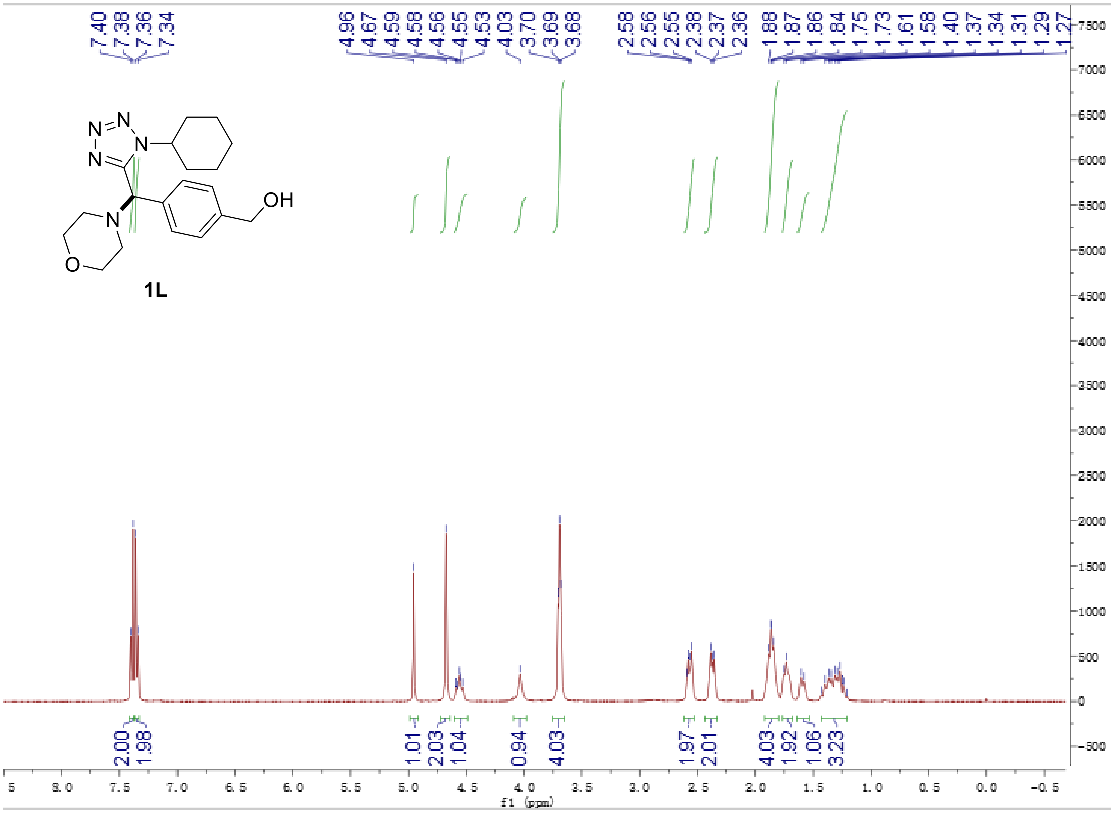

Supplementary Figure 470. ^{1}H NMR (400 MHz, $CDCl_3$) spectrum of **1L**.

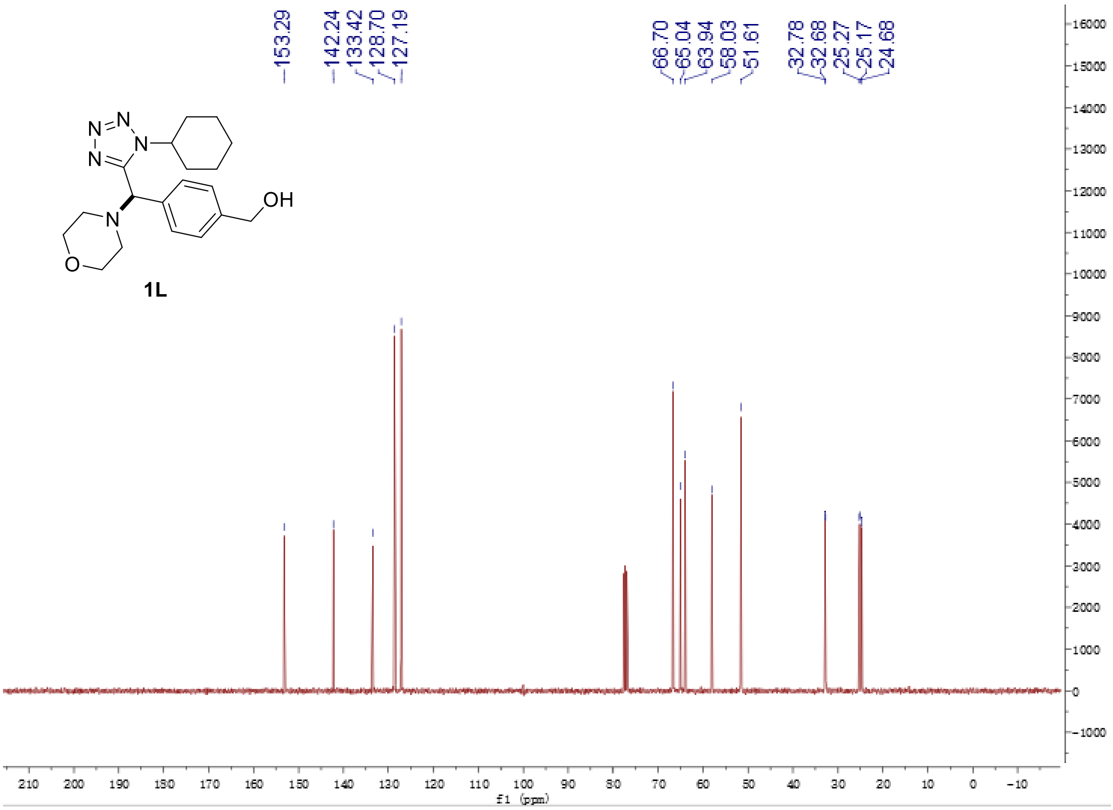

Supplementary Figure 471. ^{13}C NMR (101 MHz, $CDCl_3$) spectrum of **1L**.

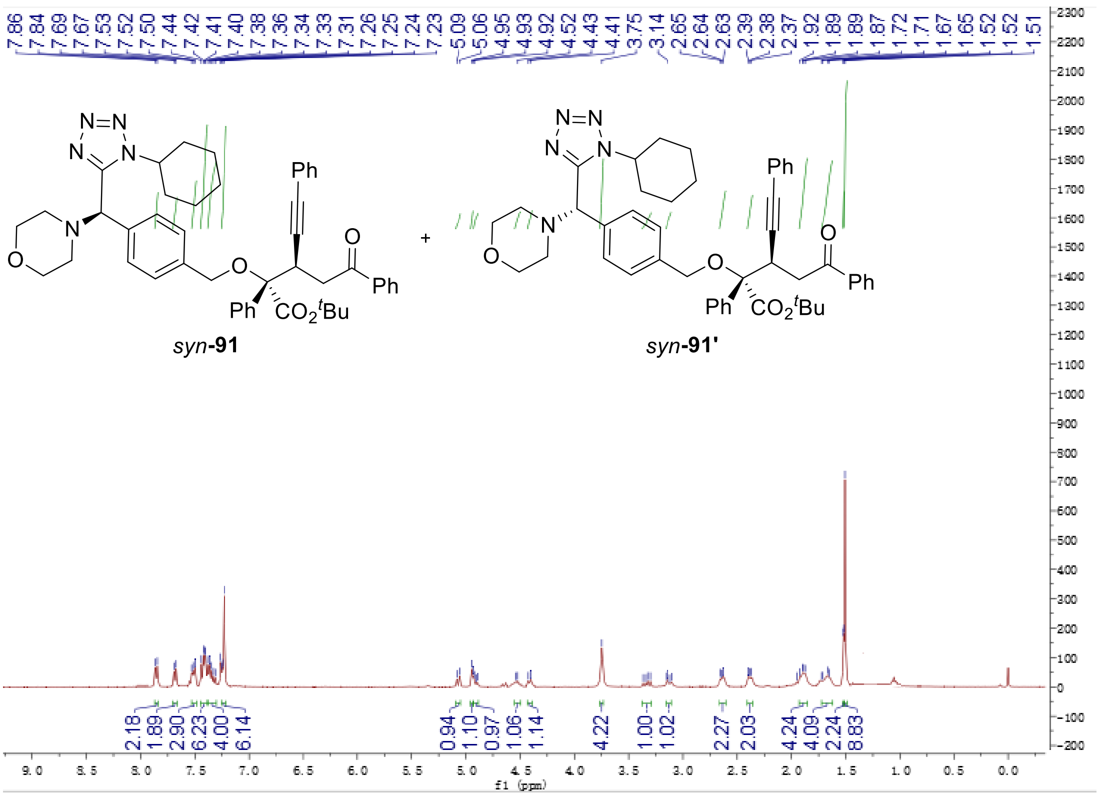

Supplementary Figure 472. ^{1}H NMR (400 MHz, $CDCl_3$) spectrum of **91**.

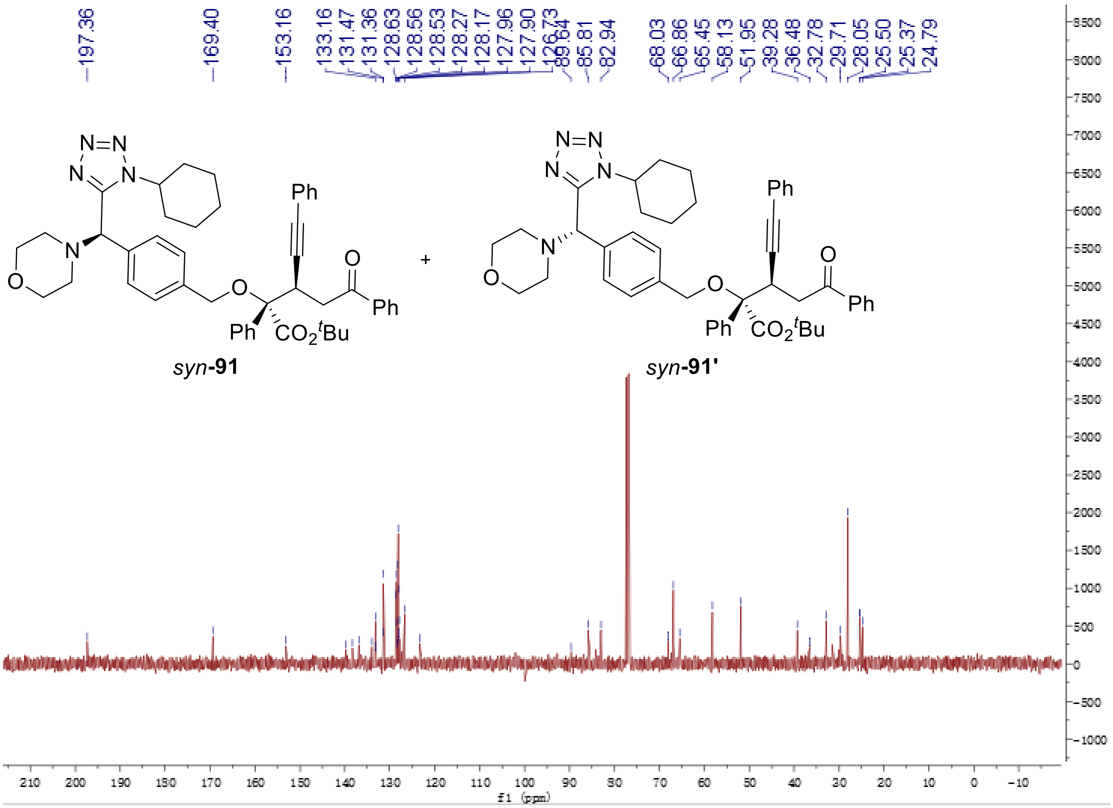

Supplementary Figure 473. ^{13}C NMR (101 MHz, $CDCl_3$) spectrum of **91**.

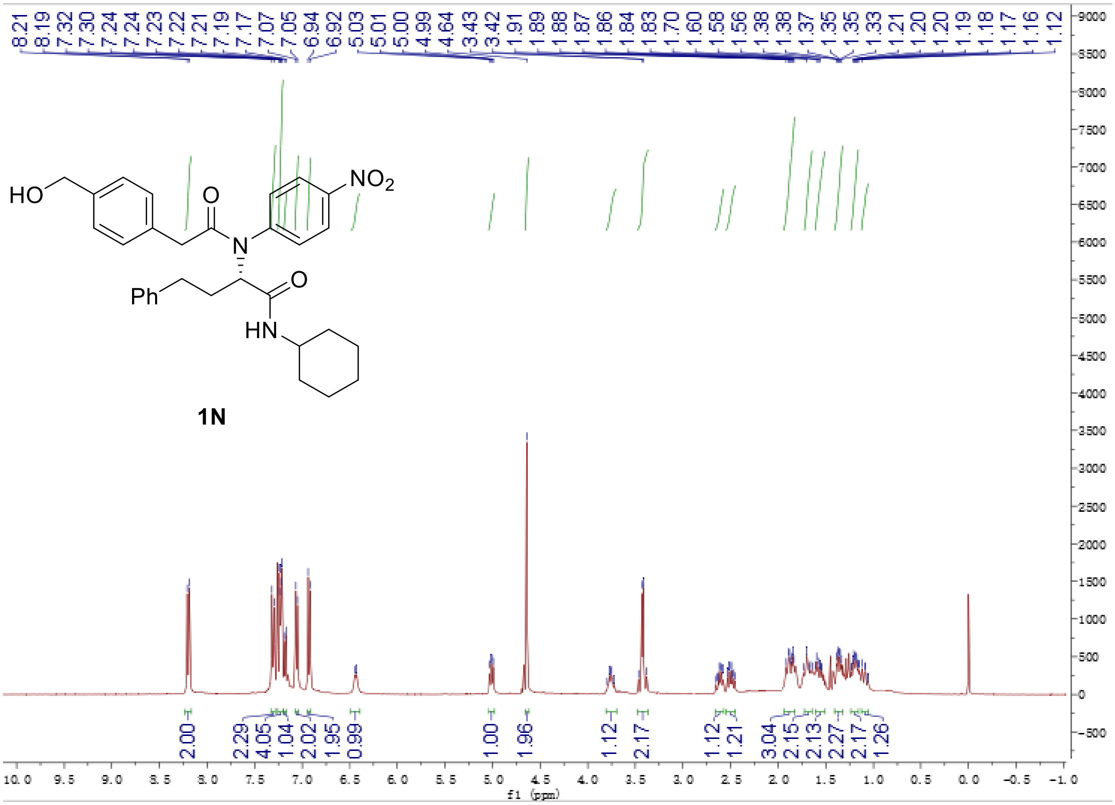

Supplementary Figure 474. ^{1}H NMR (400 MHz, $CDCl_3$) spectrum of **1N**.

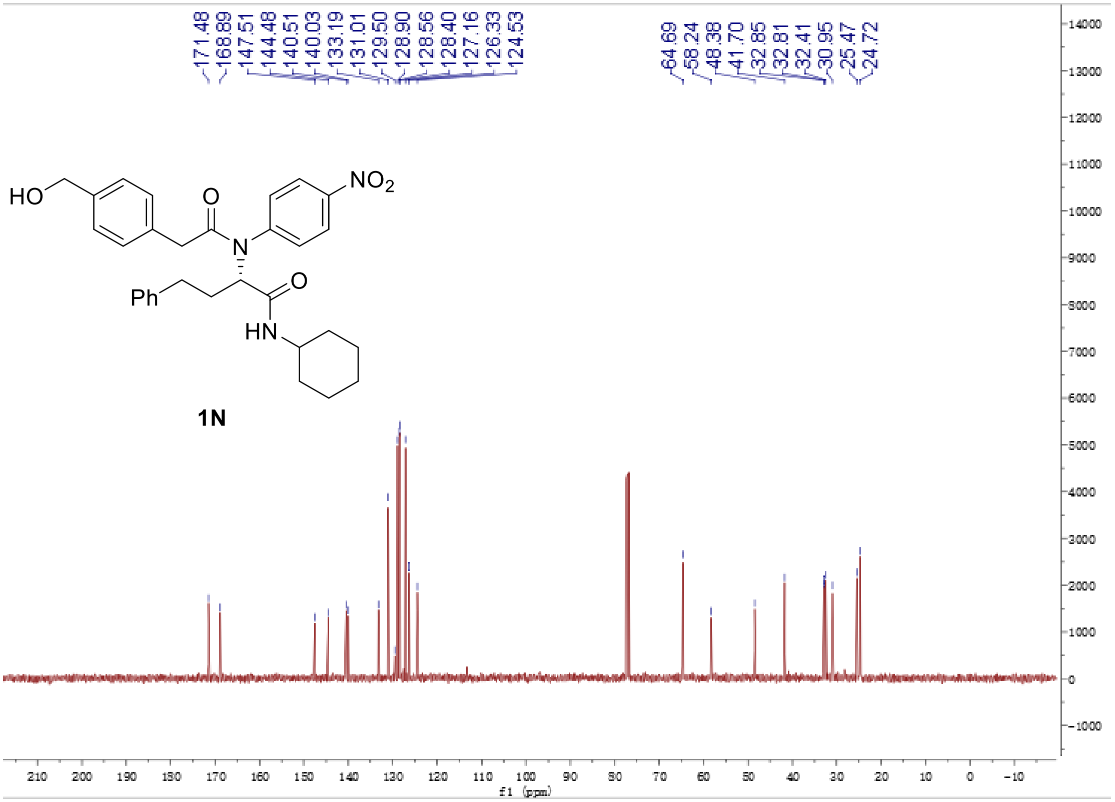

Supplementary Figure 475. ^{13}C NMR (101 MHz, $CDCl_3$) spectrum of **1N**.

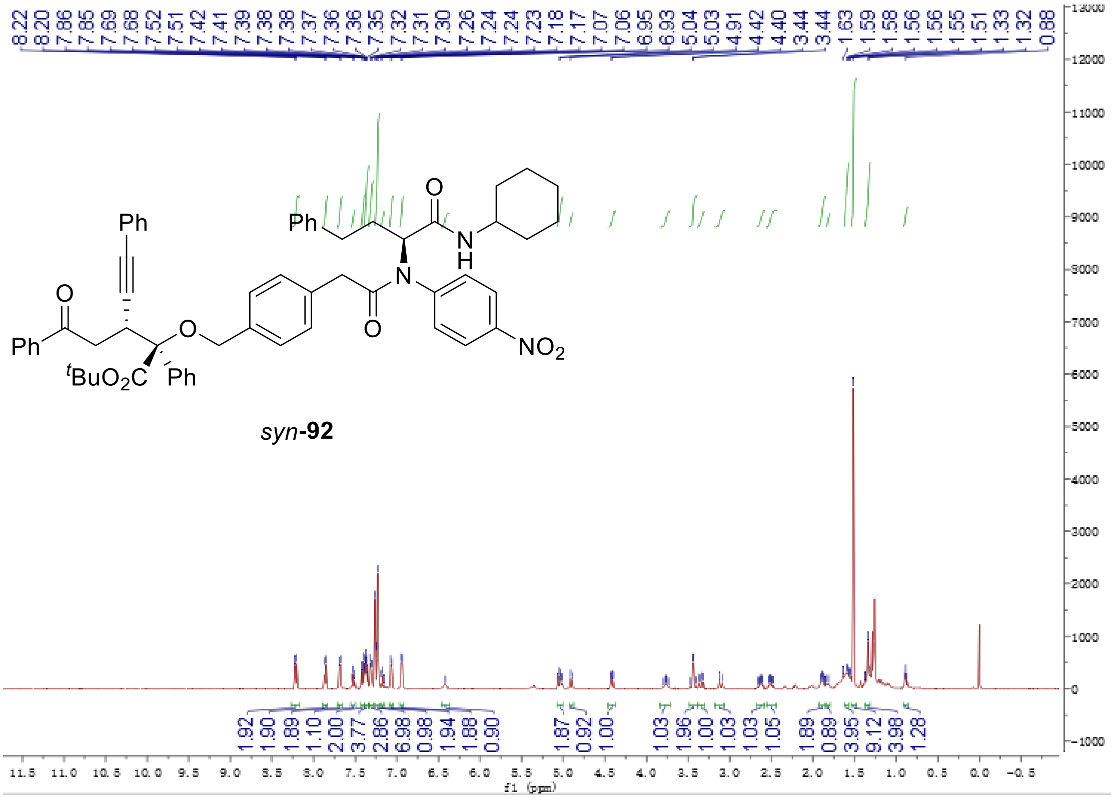

Supplementary Figure 476. ^{1}H NMR (500 MHz, $CDCl_3$) spectrum of **92**.

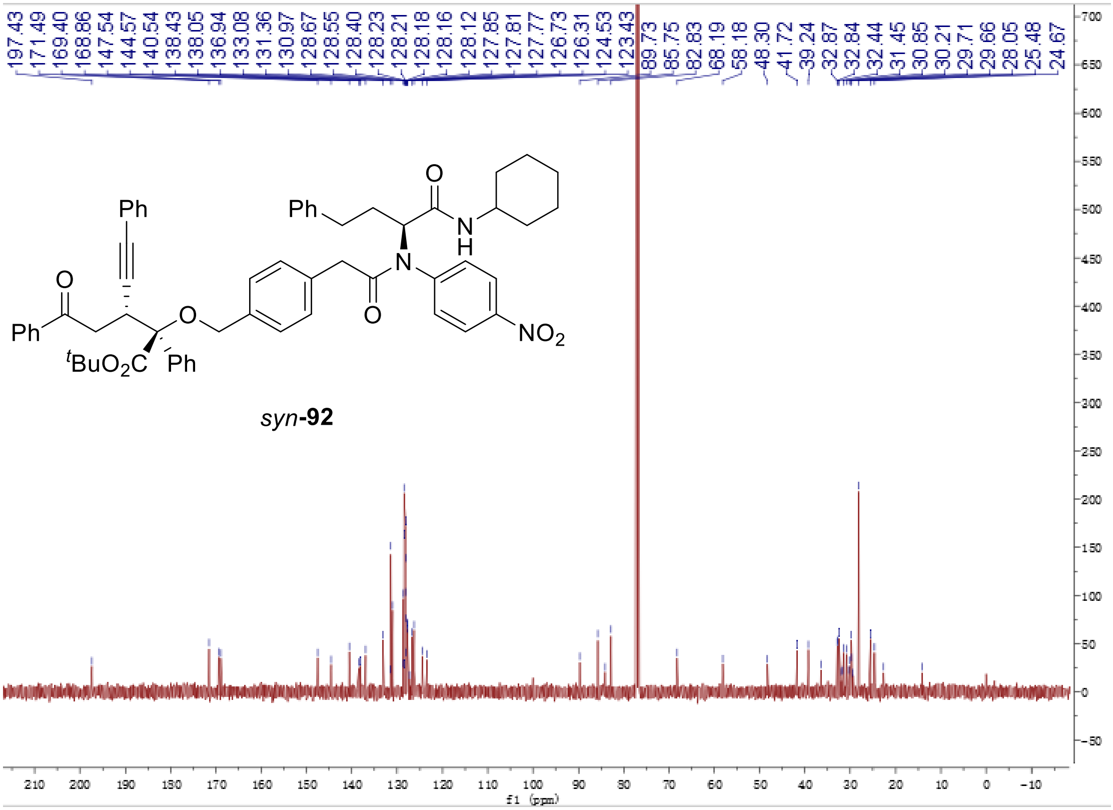

Supplementary Figure 477. ^{13}C NMR (126 MHz, $CDCl_3$) spectrum of **92**.

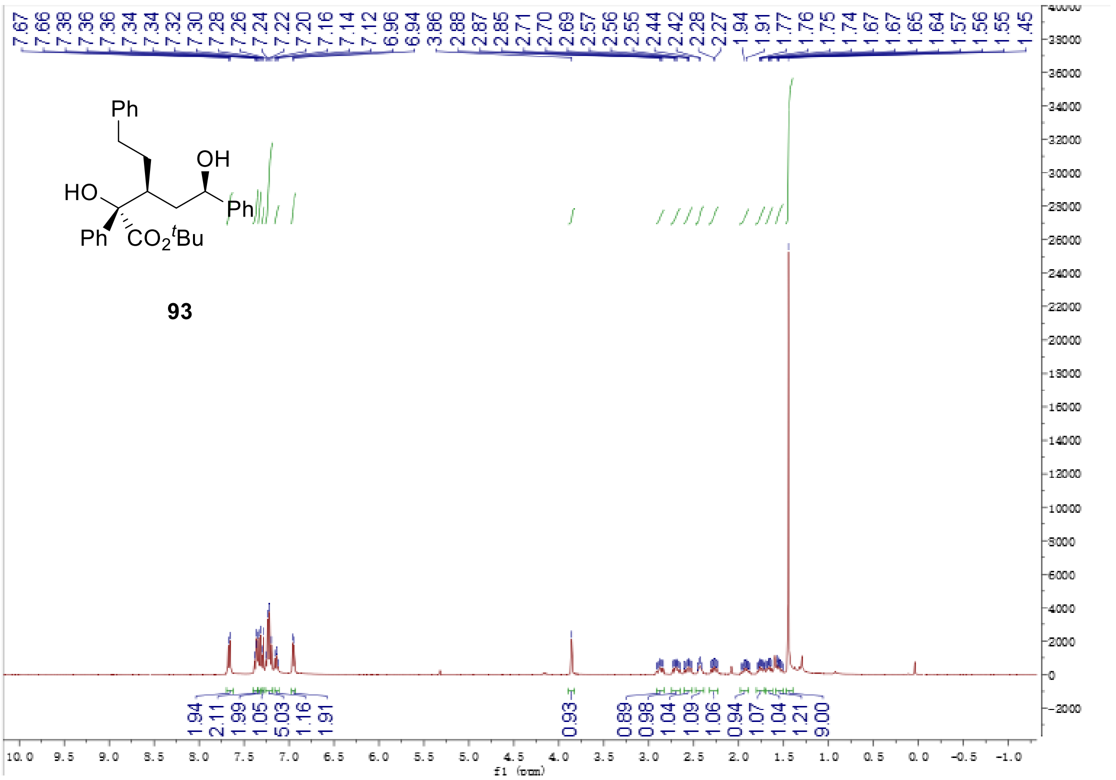

Supplementary Figure 478. ^{1}H NMR (400 MHz, $CDCl_3$) spectrum of **93**.

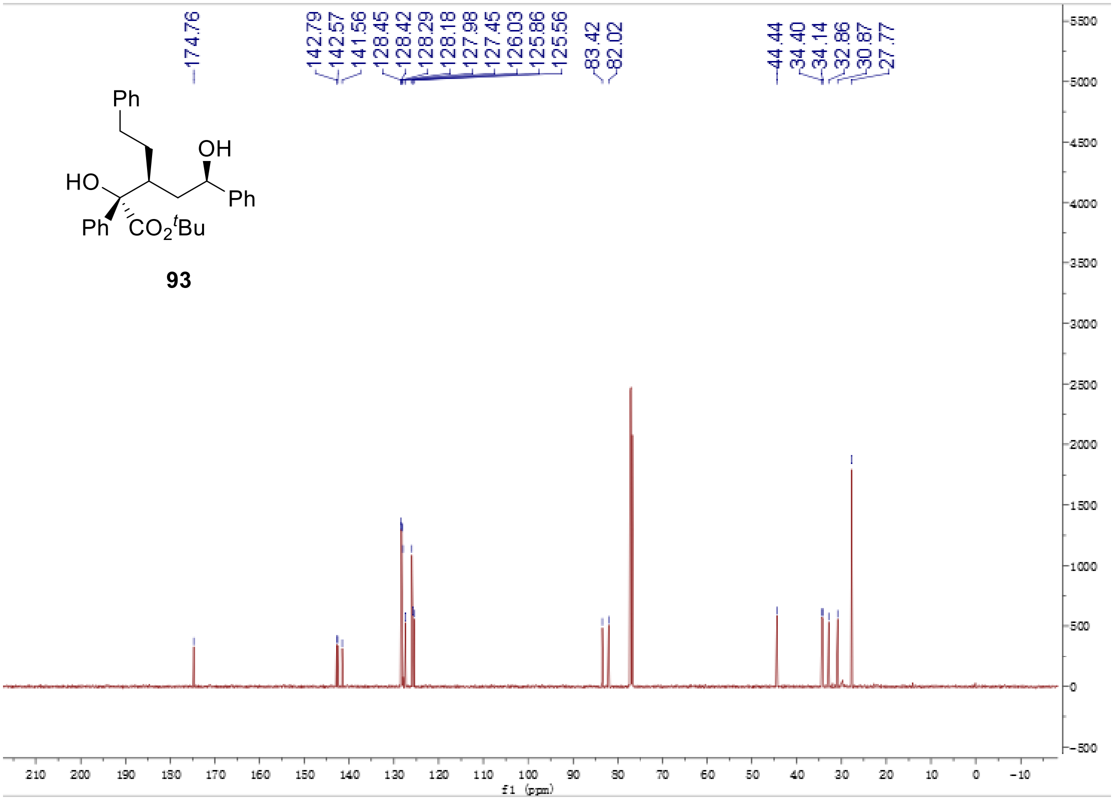

Supplementary Figure 479. ^{13}C NMR (101 MHz, $CDCl_3$) spectrum of **93**.

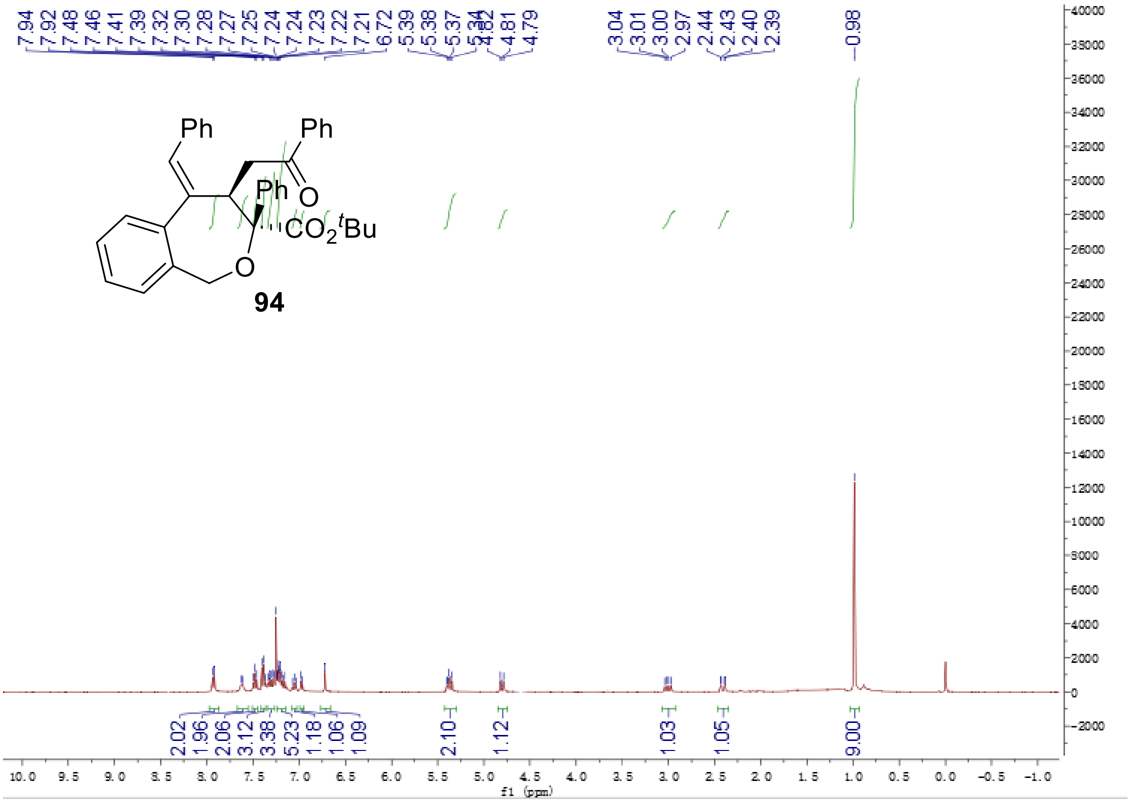

Supplementary Figure 480. ^{1}H NMR (400 MHz, $CDCl_3$) spectrum of **94**.

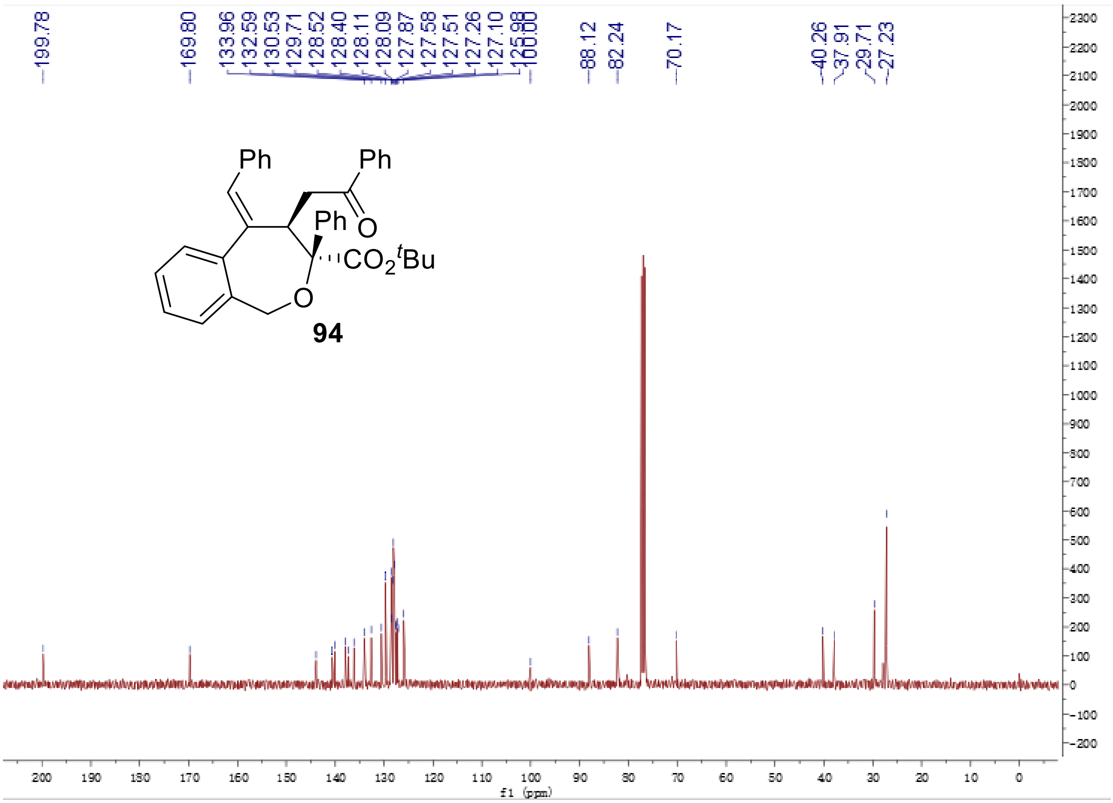

Supplementary Figure 481. ^{13}C NMR (101 MHz, $CDCl_3$) spectrum of **94**.

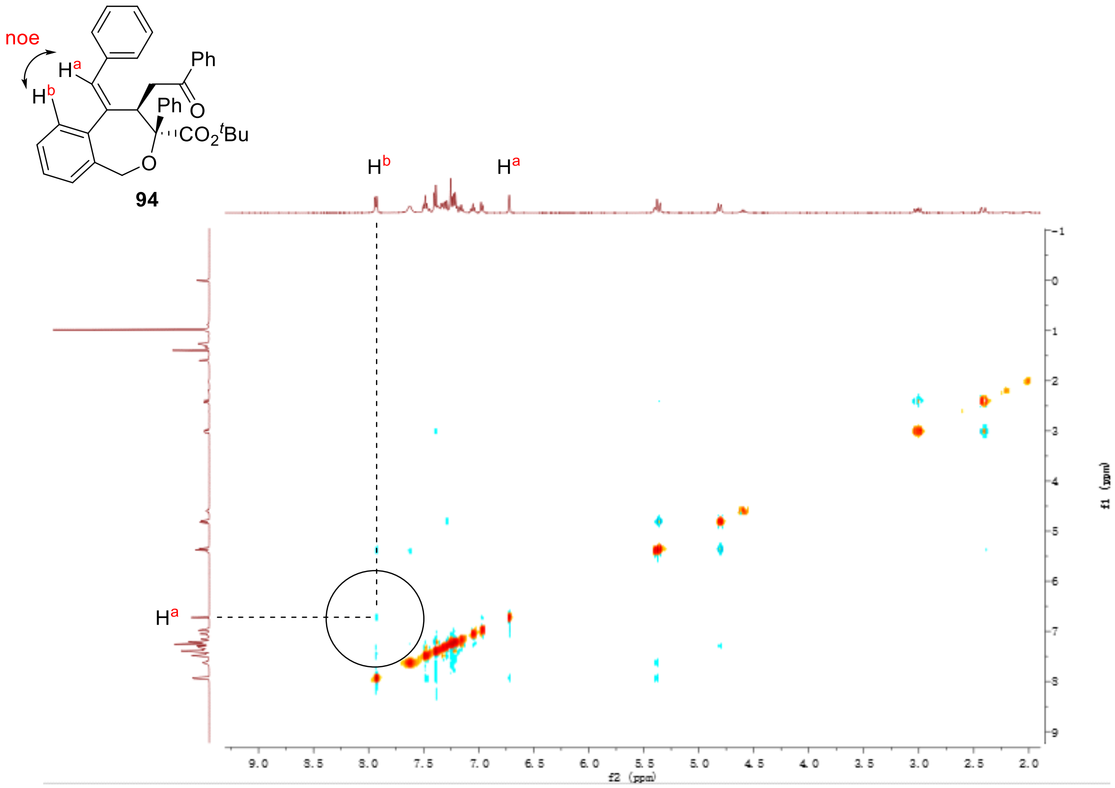

Supplementary Figure 482. NOE spectrum of **94**.

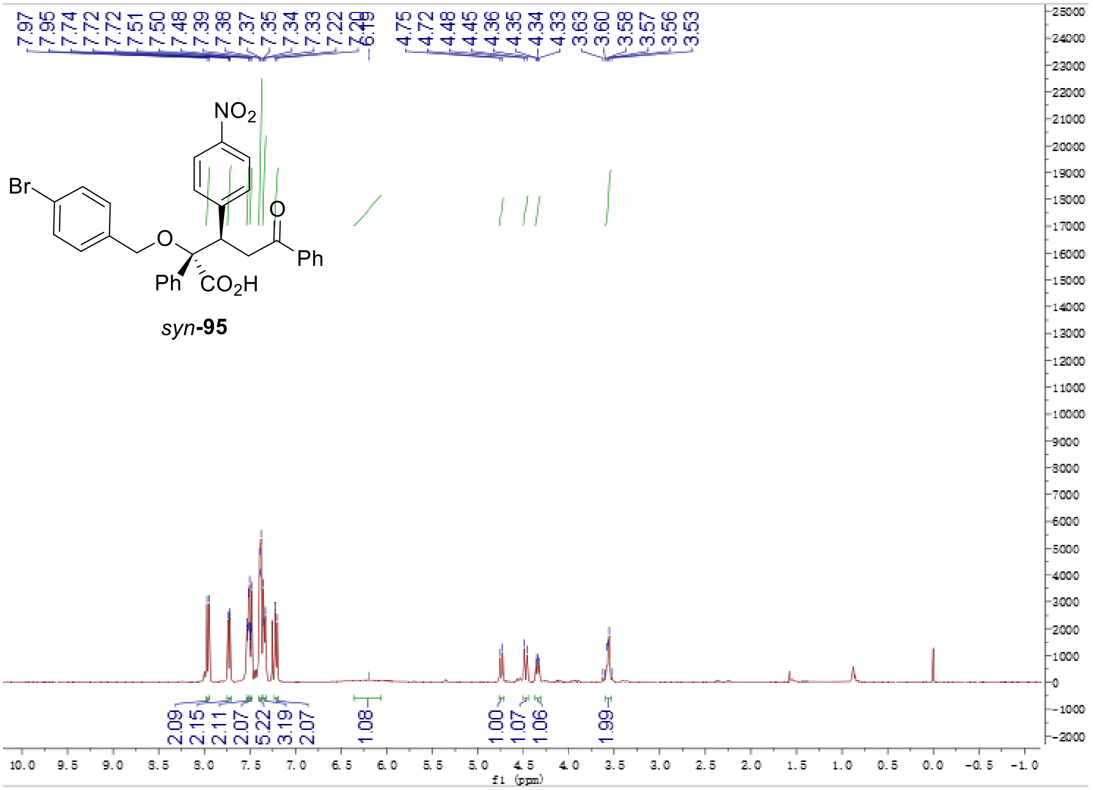

Supplementary Figure 483. ^{1}H NMR (400 MHz, $CDCl_3$) spectrum of **95**.

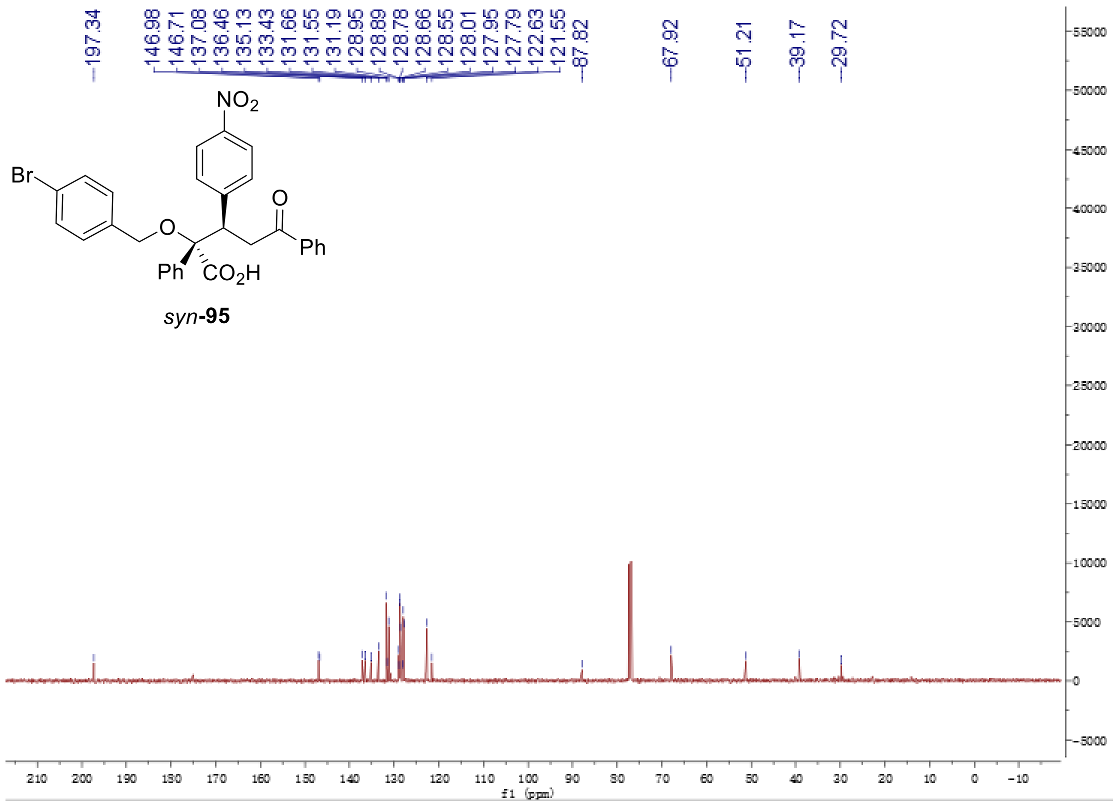

Supplementary Figure 484. ^{13}C NMR (101 MHz, $CDCl_3$) spectrum of **95**.

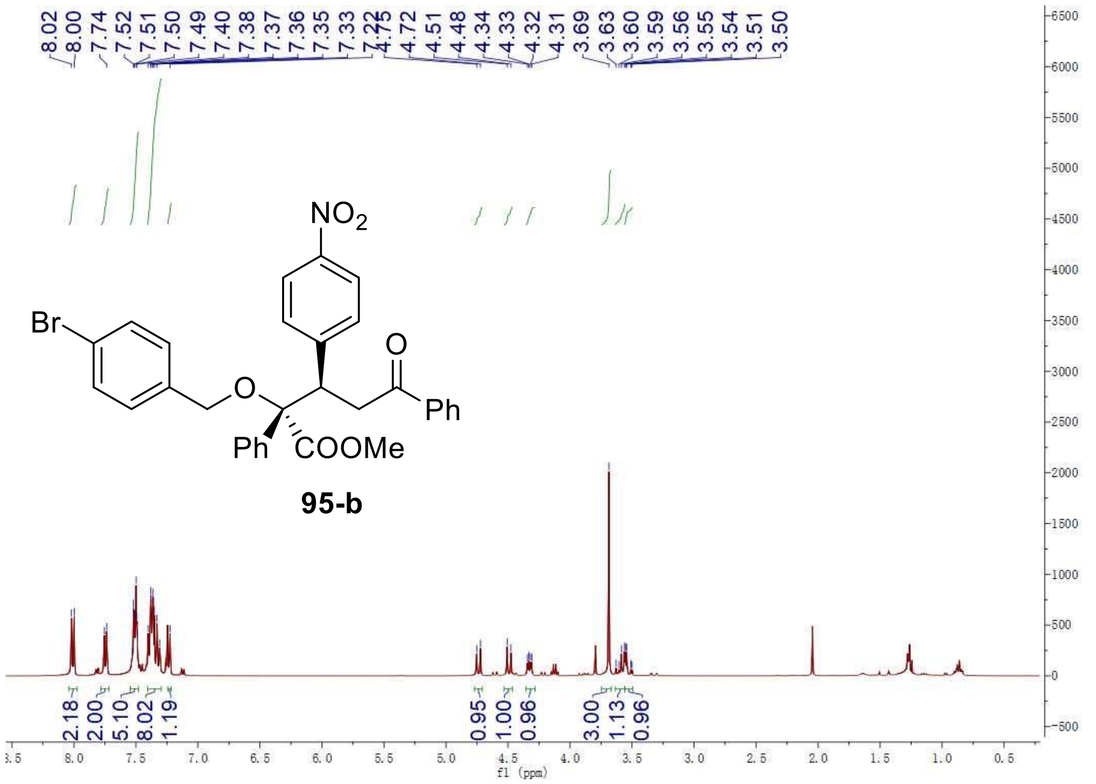

Supplementary Figure 485. ^{1}H NMR (400 MHz, $CDCl_3$) spectrum of **95-b**.

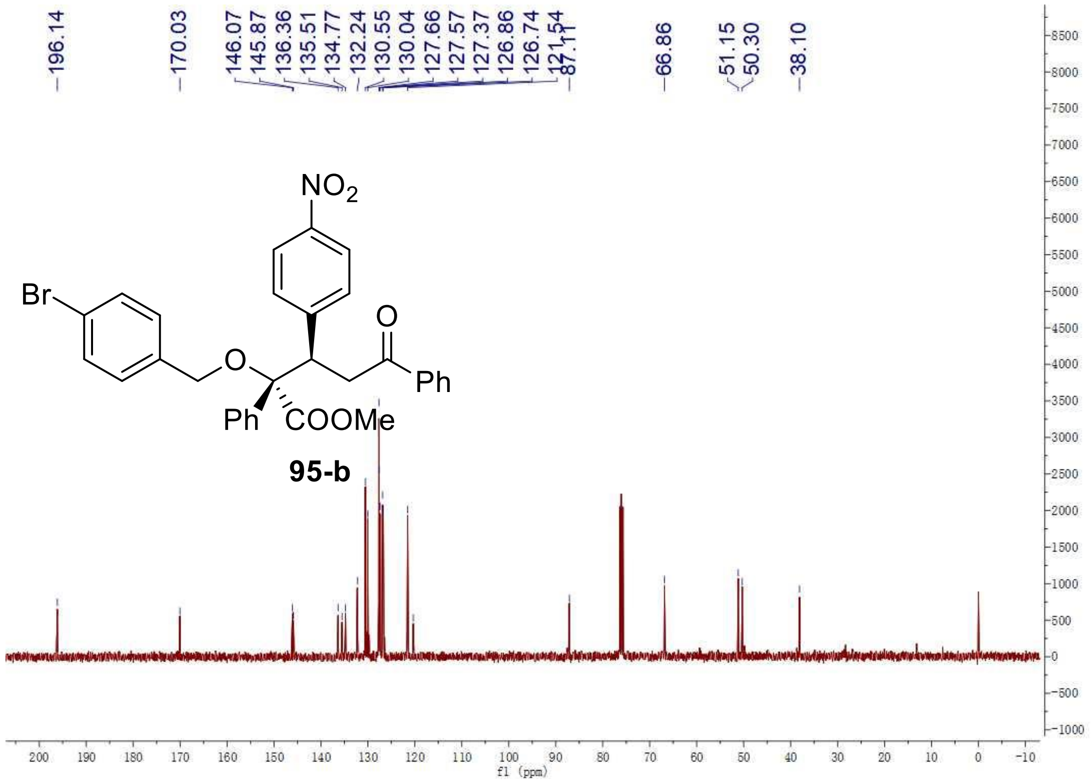

Supplementary Figure 486. ^{13}C NMR (101 MHz, $CDCl_3$) spectrum of **95-b**.

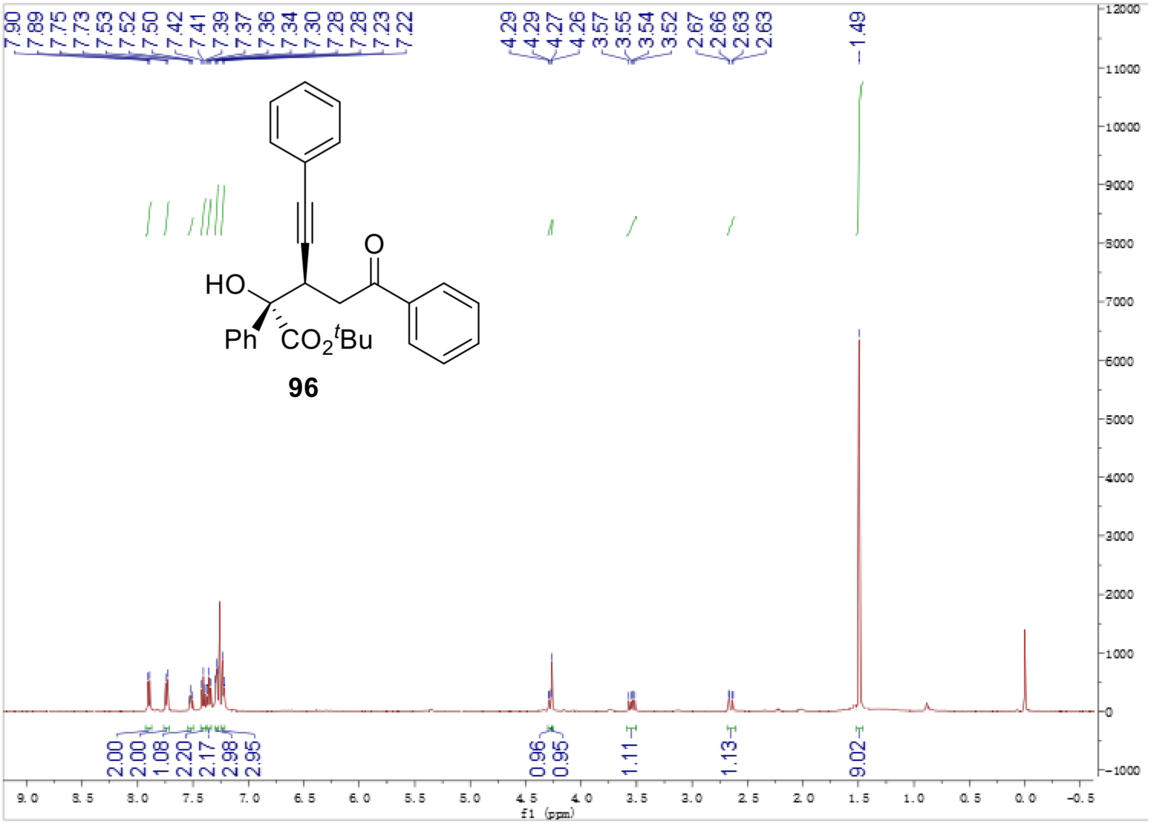

Supplementary Figure 487. ^{1}H NMR (500 MHz, $CDCl_3$) spectrum of **96**.

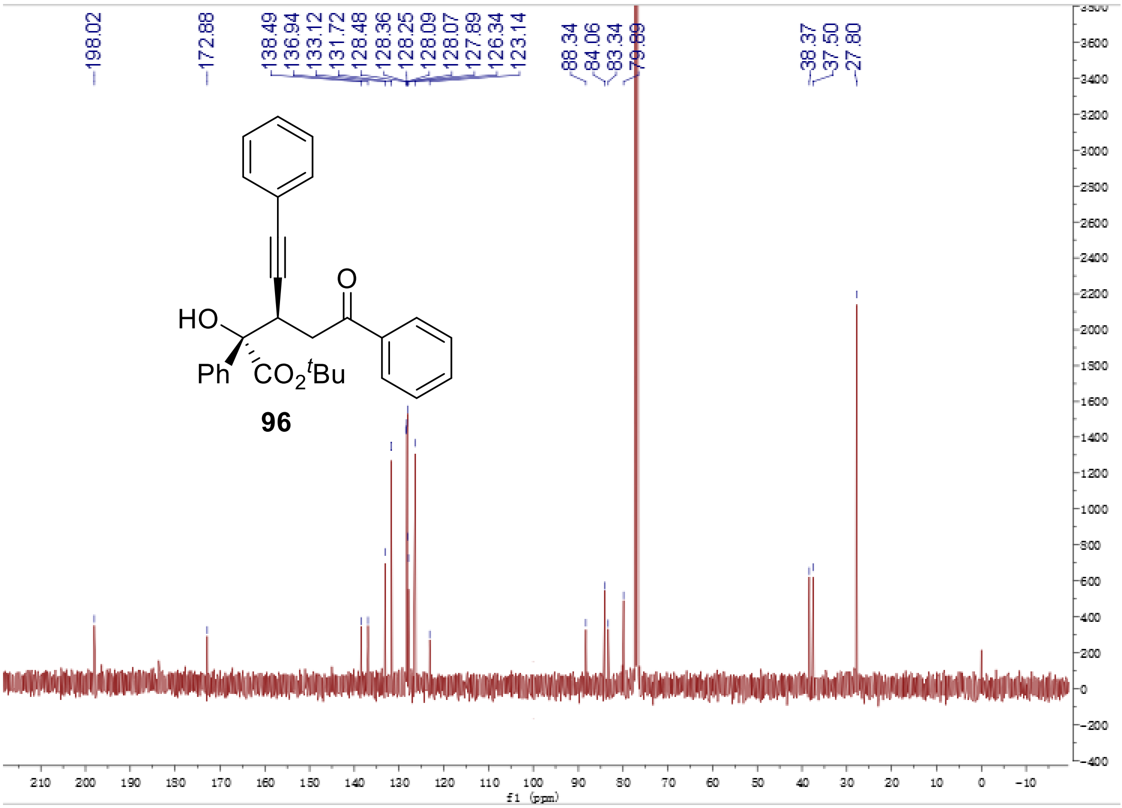

Supplementary Figure 488. ^{13}C NMR (126 MHz, $CDCl_3$) spectrum of **96**.

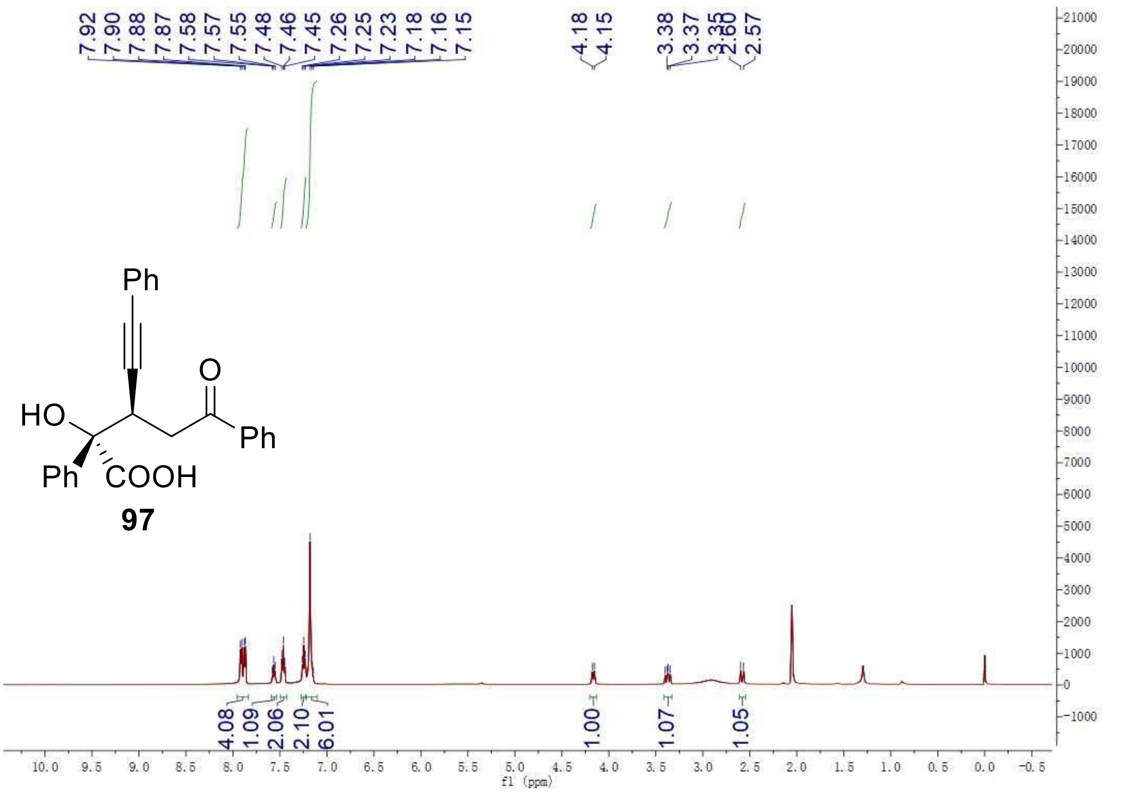

Supplementary Figure 489. ^{1}H NMR (500 MHz, acetone-d6) spectrum of **97**.

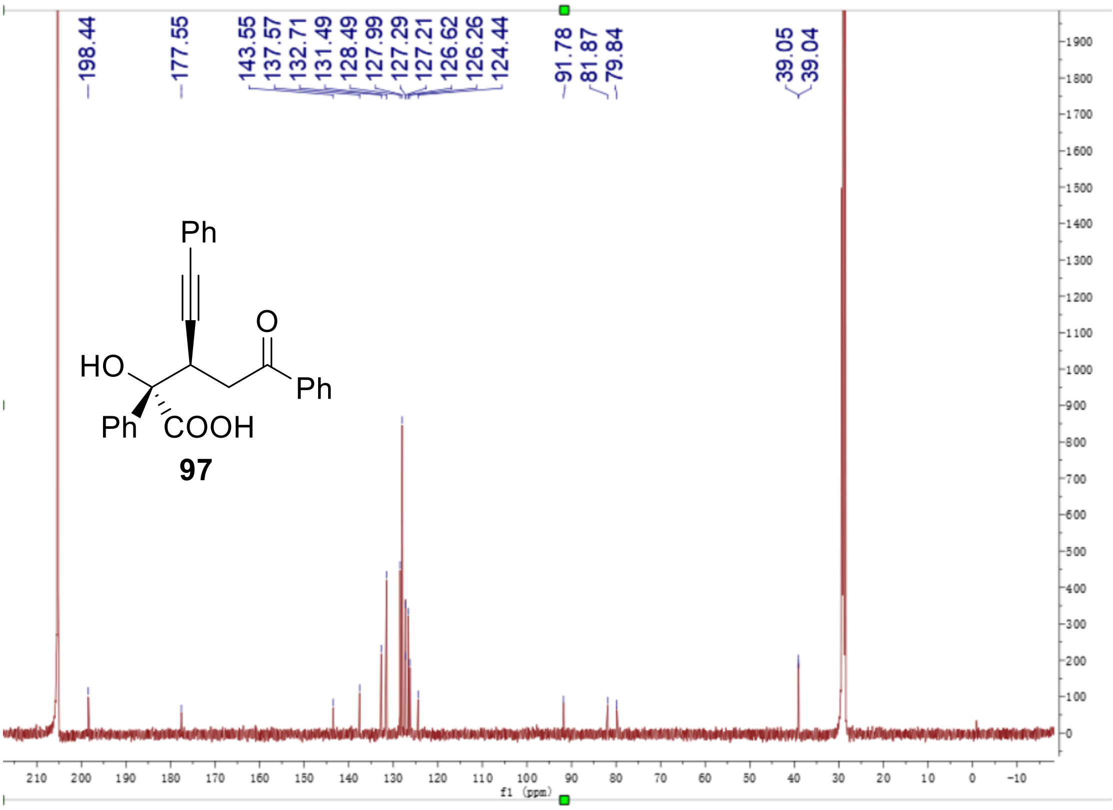

Supplementary Figure 490. ^{13}C NMR (126 MHz, acetone-d6) spectrum of **97**.

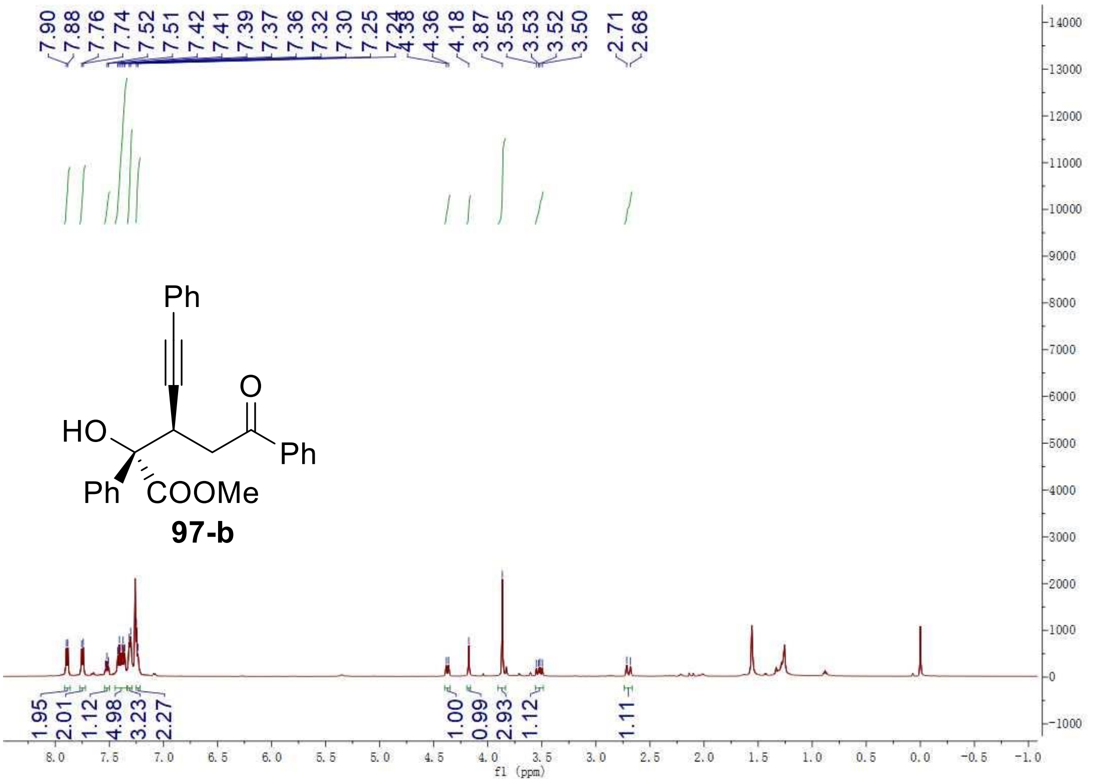

Supplementary Figure 491. ^{1}H NMR (500 MHz, $CDCl_3$) spectrum of **97-b**.

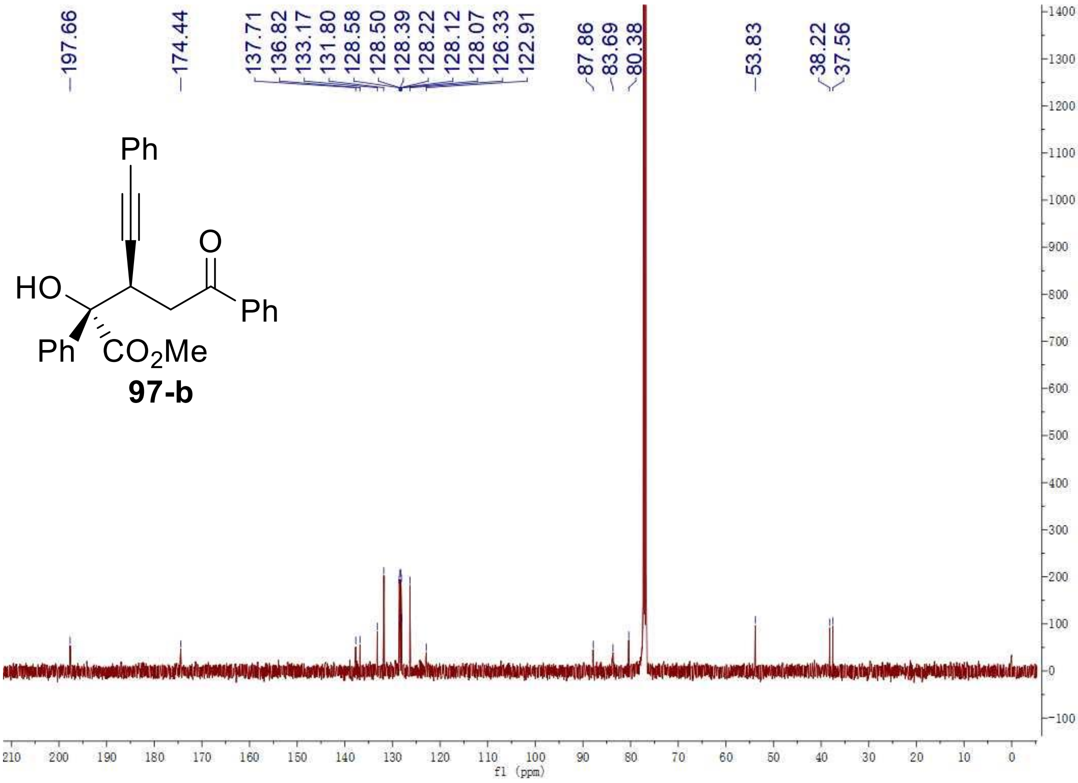

Supplementary Figure 492. ^{13}C NMR (126 MHz, $CDCl_3$) spectrum of **97-b**.

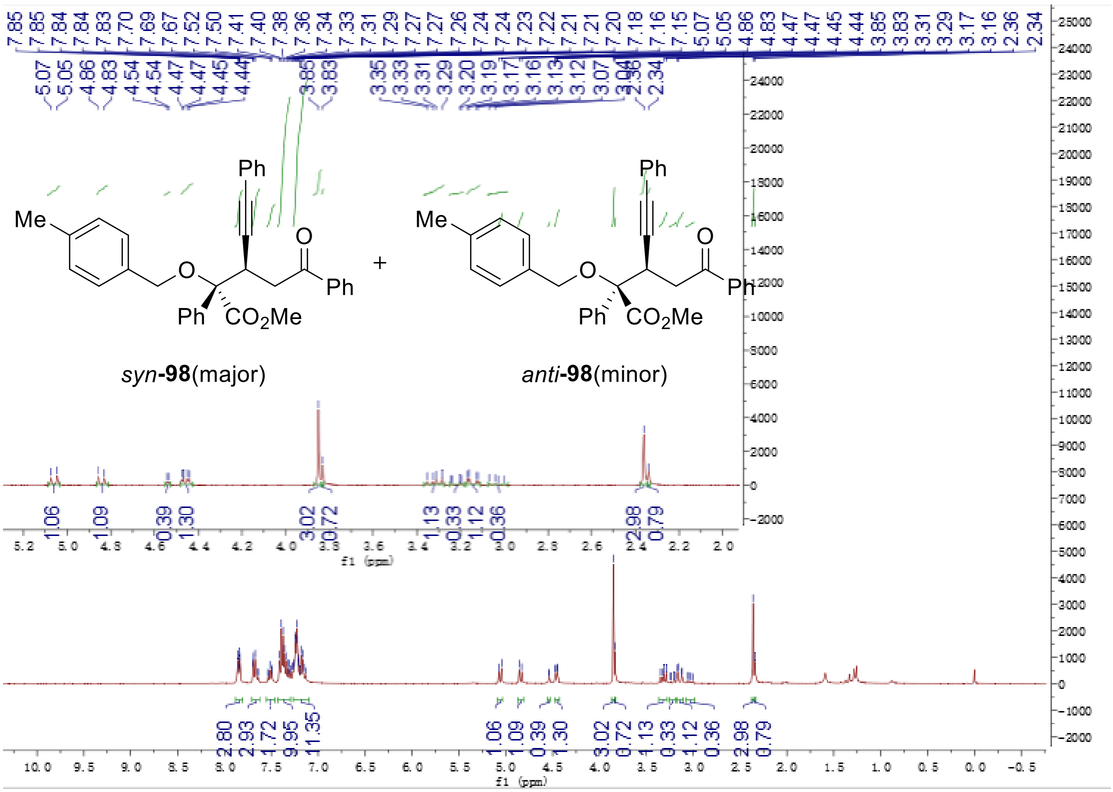

Supplementary Figure 493. ^{1}H NMR (400 MHz, $CDCl_3$) spectrum of **98**.

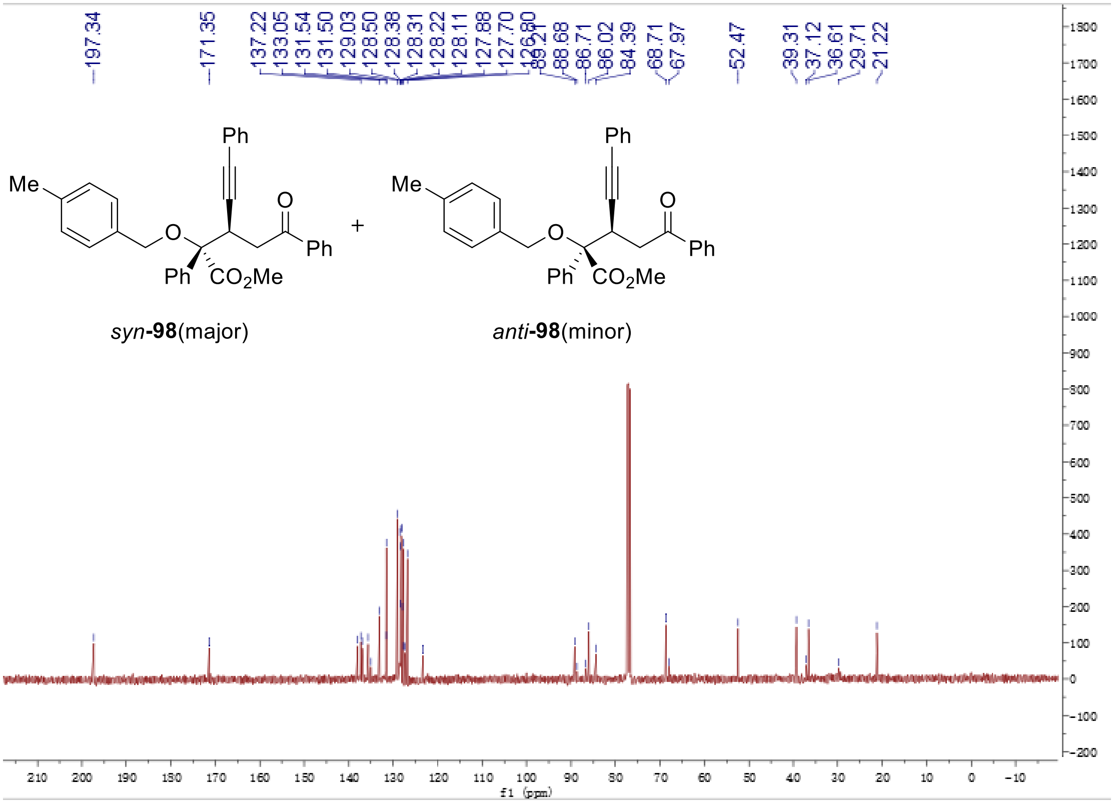

Supplementary Figure 494. ^{13}C NMR (101 MHz, $CDCl_3$) spectrum of **98**.

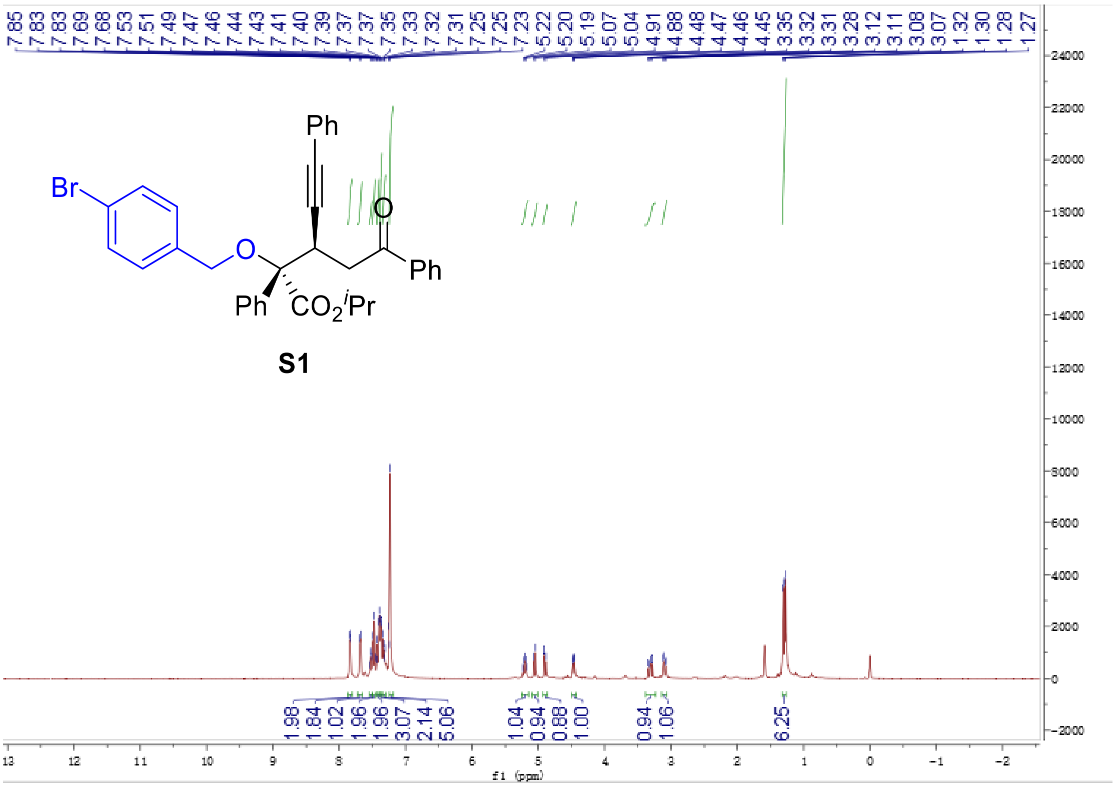

Supplementary Figure 495. ^{1}H NMR (400 MHz, $CDCl_3$) spectrum of **S1**.

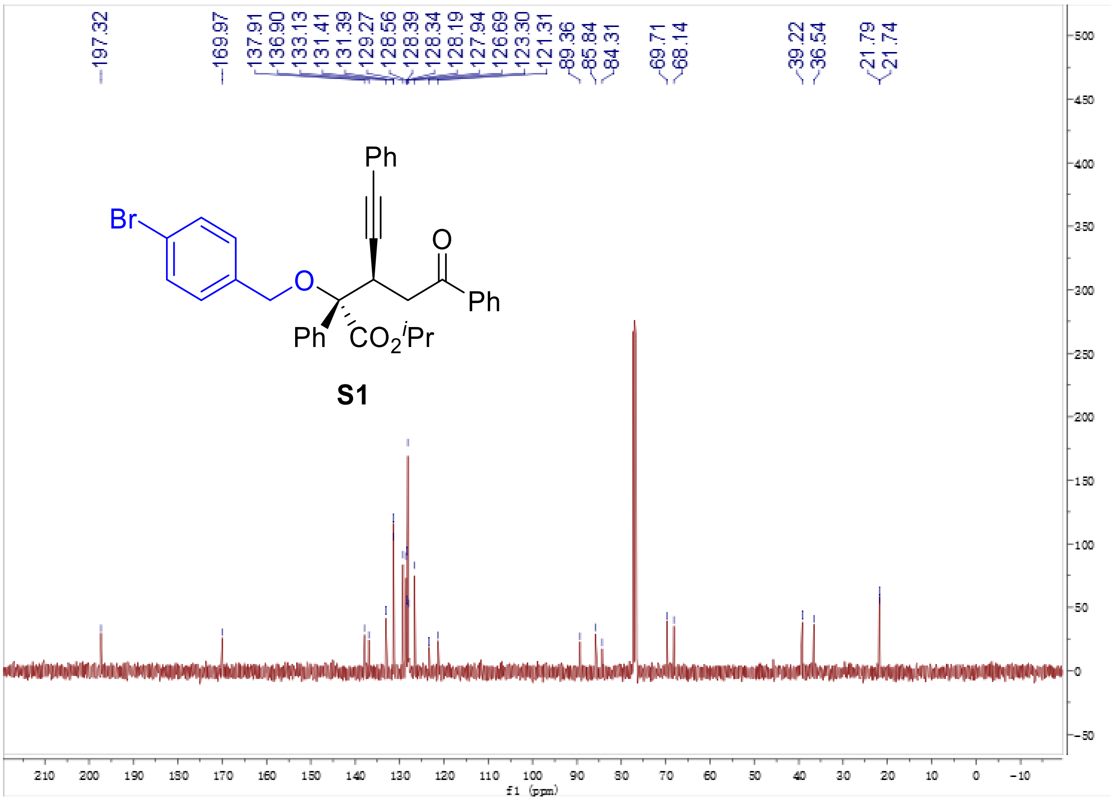

Supplementary Figure 496. ^{13}C NMR (101 MHz, $CDCl_3$) spectrum of **S1**.

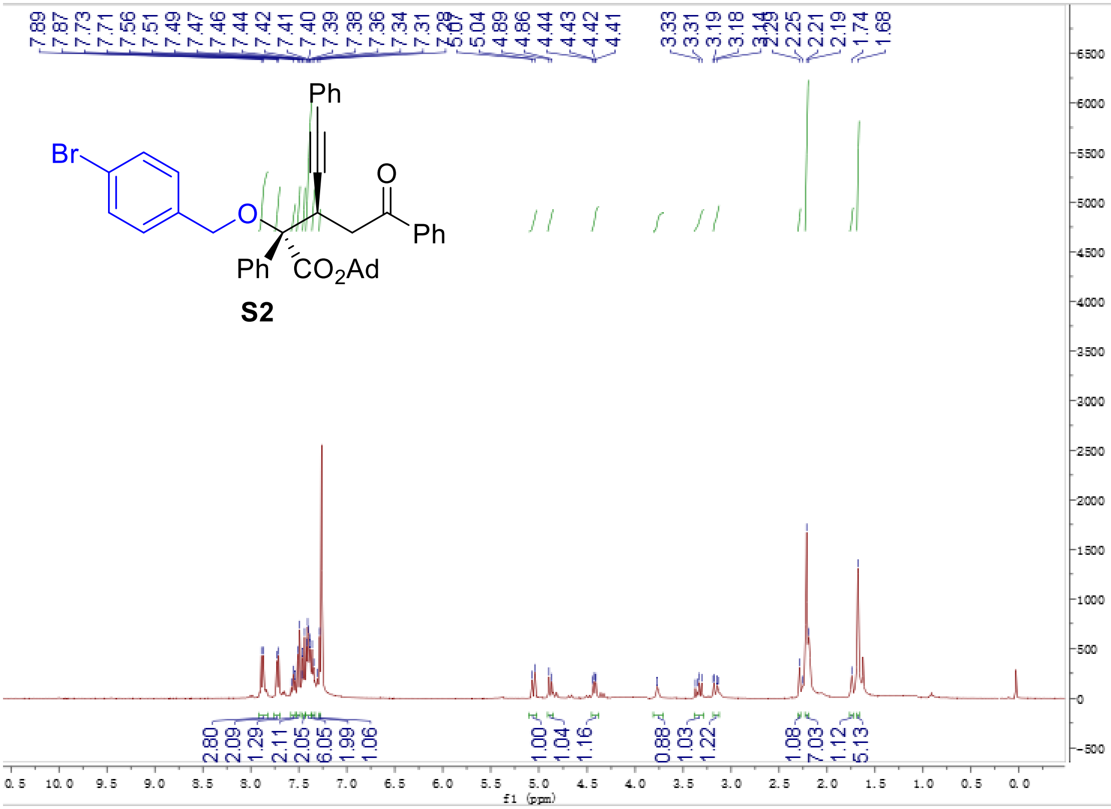

Supplementary Figure 497. ^{1}H NMR (400 MHz, $CDCl_3$) spectrum of **S2**.

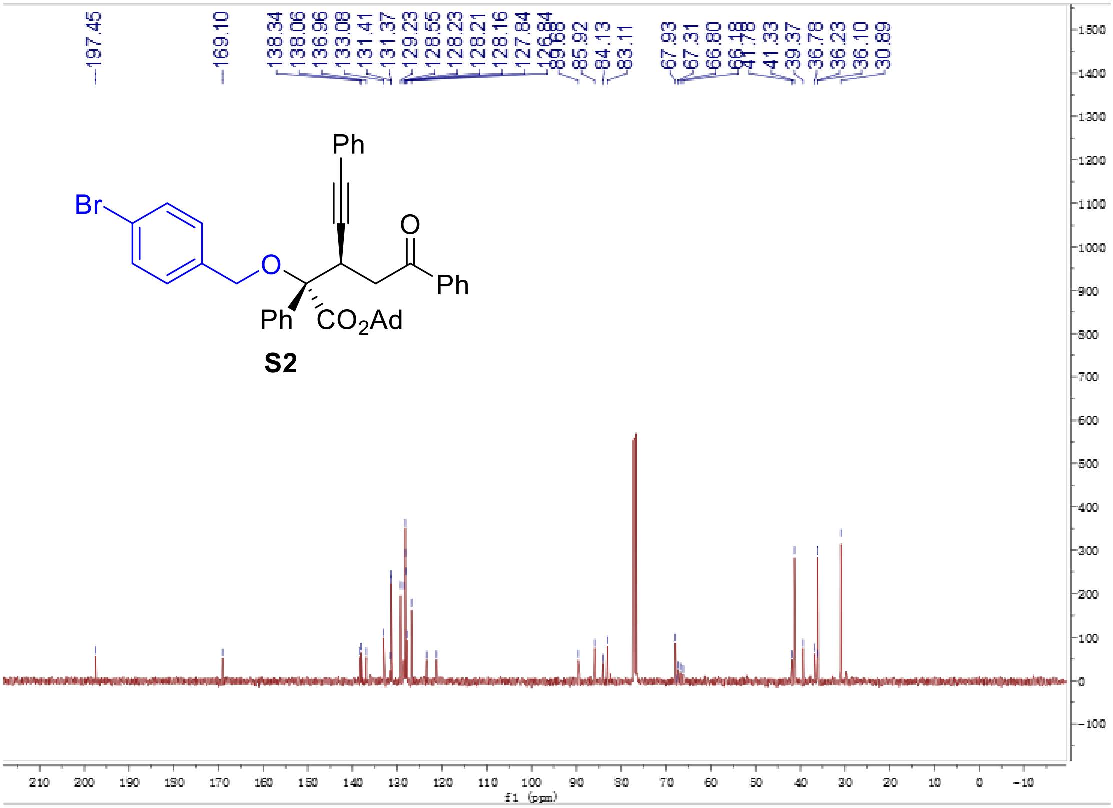

Supplementary Figure 498. ^{13}C NMR (101 MHz, $CDCl_3$) spectrum of **S2**.

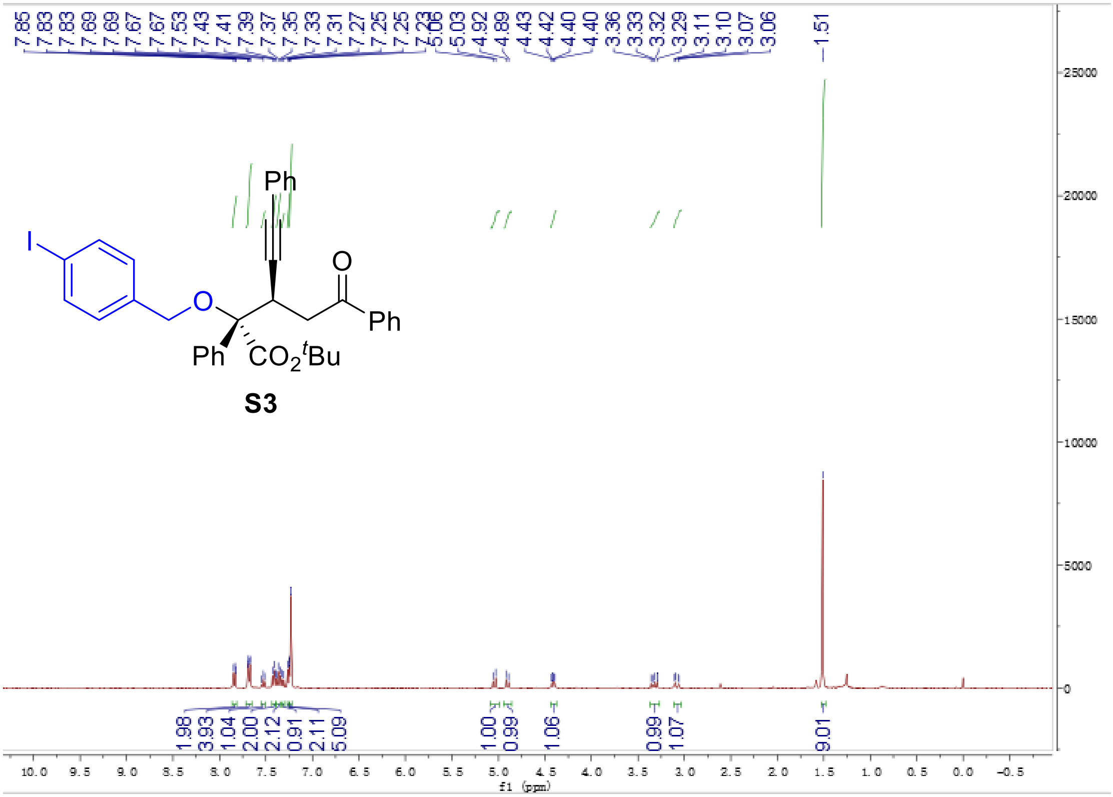

Supplementary Figure 499. ^{1}H NMR (400 MHz, $CDCl_3$) spectrum of **S3**.

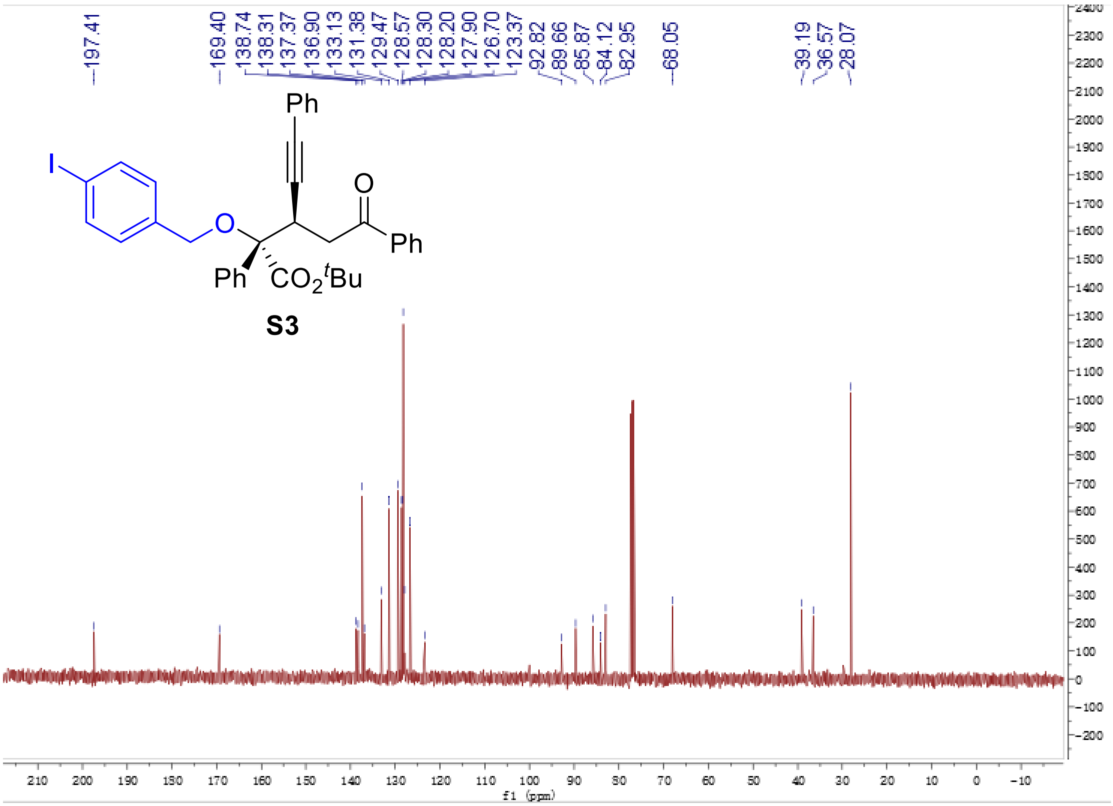

Supplementary Figure 500. ^{13}C NMR (101 MHz, $CDCl_3$) spectrum of **S3**.

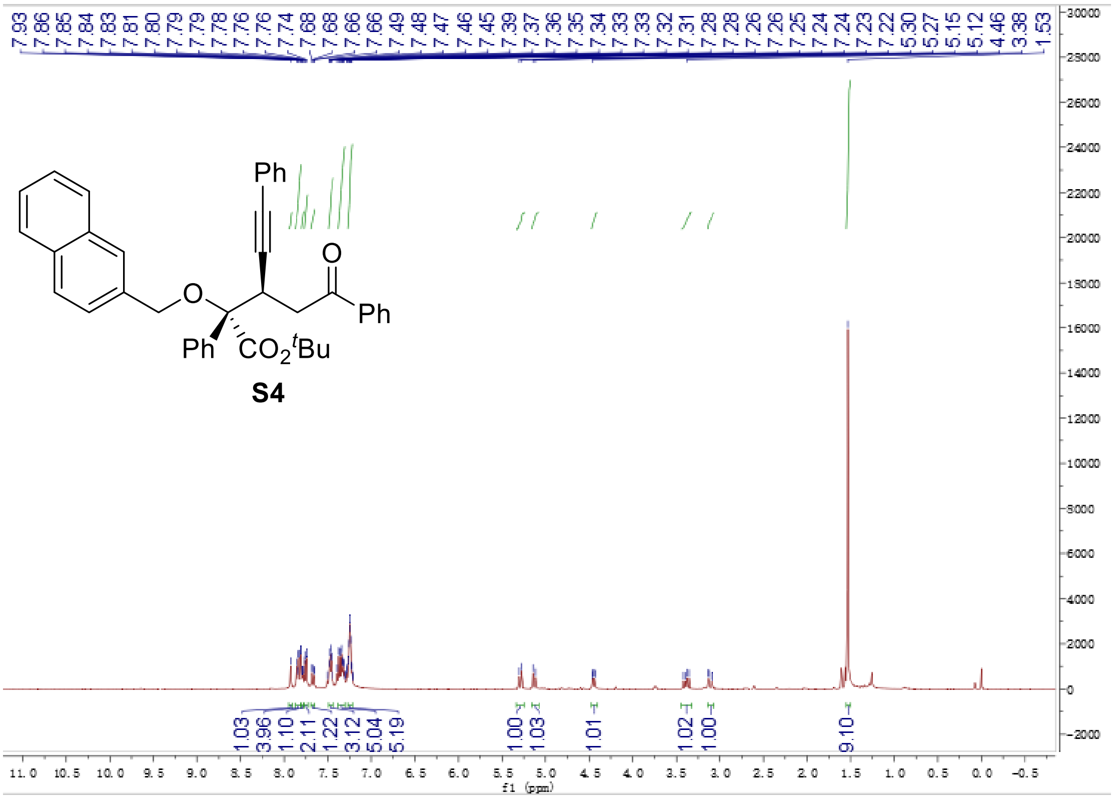

Supplementary Figure 501. ^{1}H NMR (400 MHz, $CDCl_3$) spectrum of **S4**.

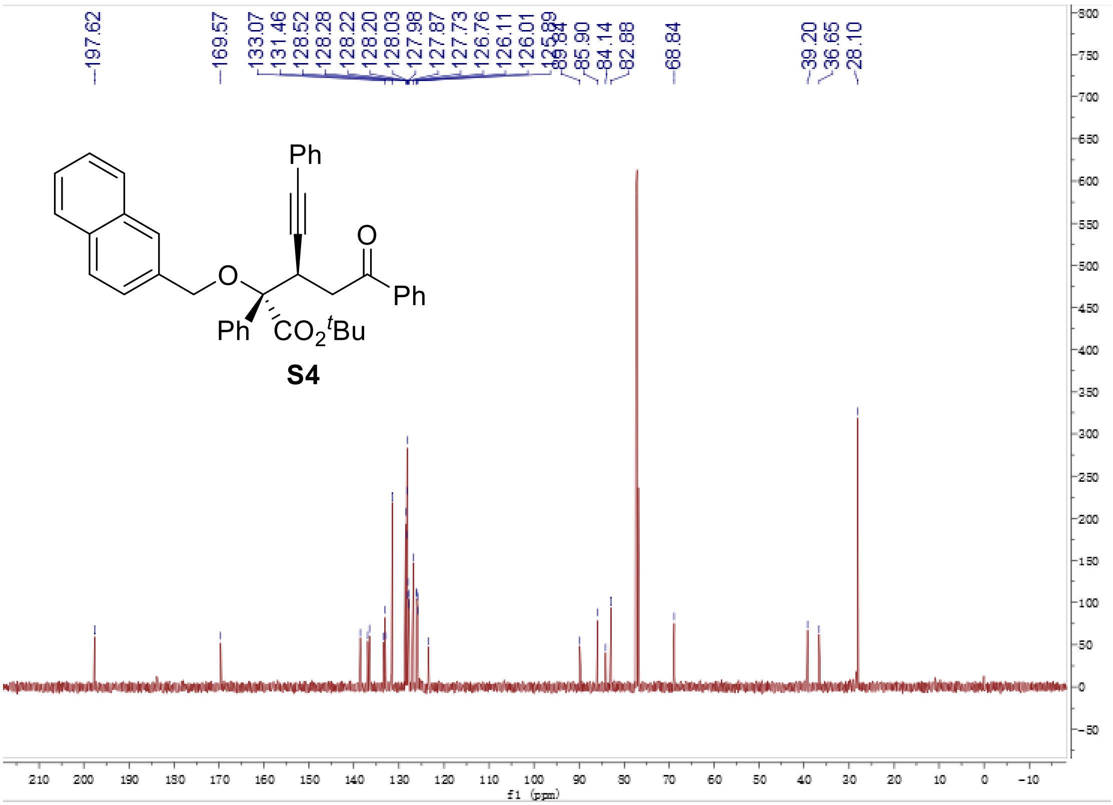

Supplementary Figure 502. ^{13}C NMR (101 MHz, $CDCl_3$) spectrum of **S4**.

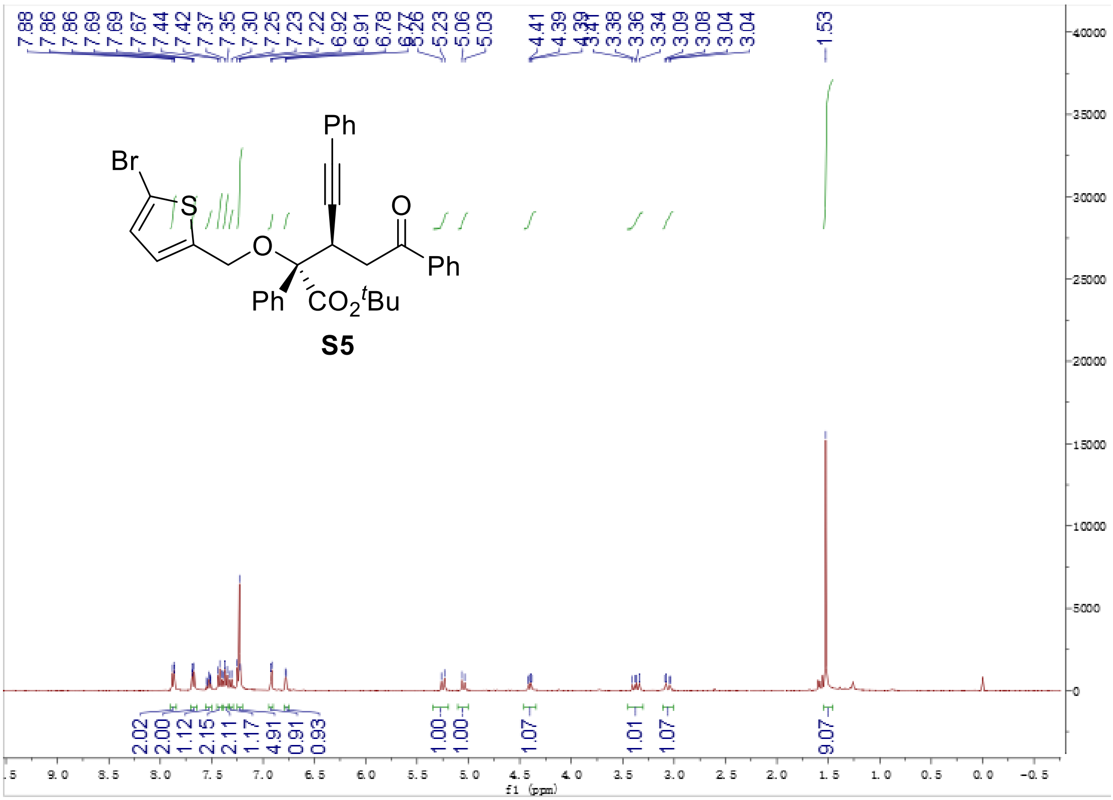

Supplementary Figure 503. ^{1}H NMR (400 MHz, $CDCl_3$) spectrum of **S5**.

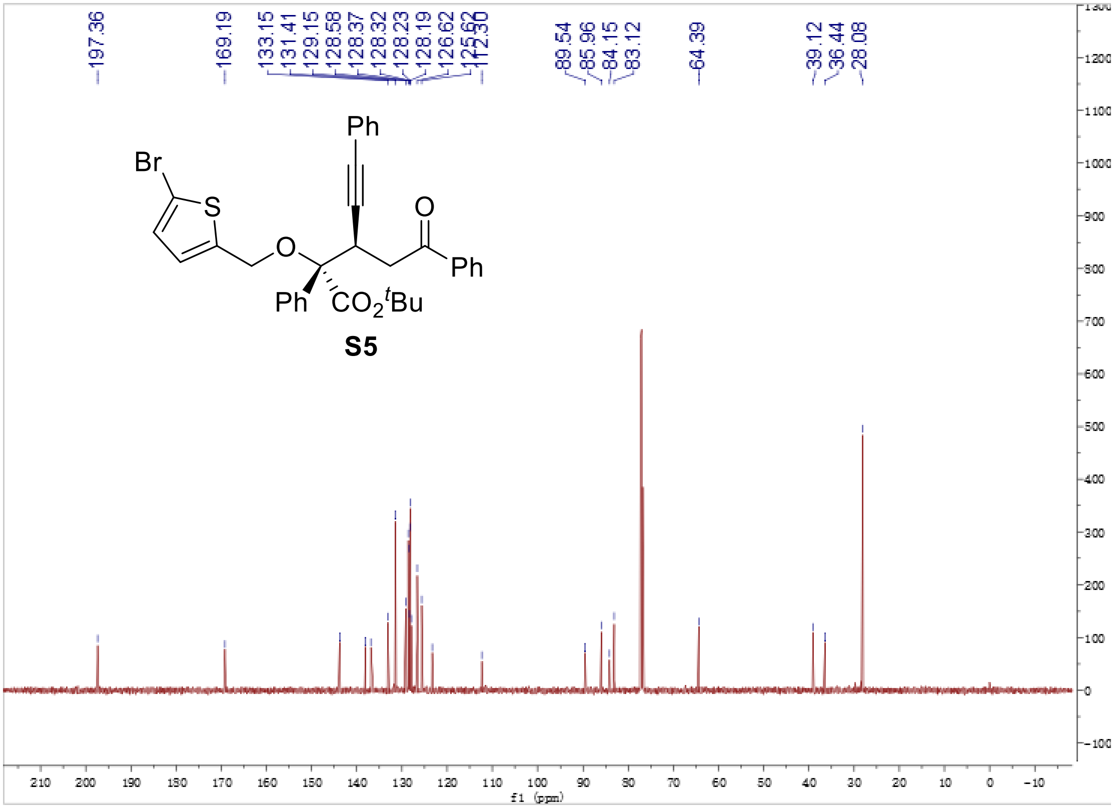

Supplementary Figure 504. ^{13}C NMR (101 MHz, $CDCl_3$) spectrum of **S5**.

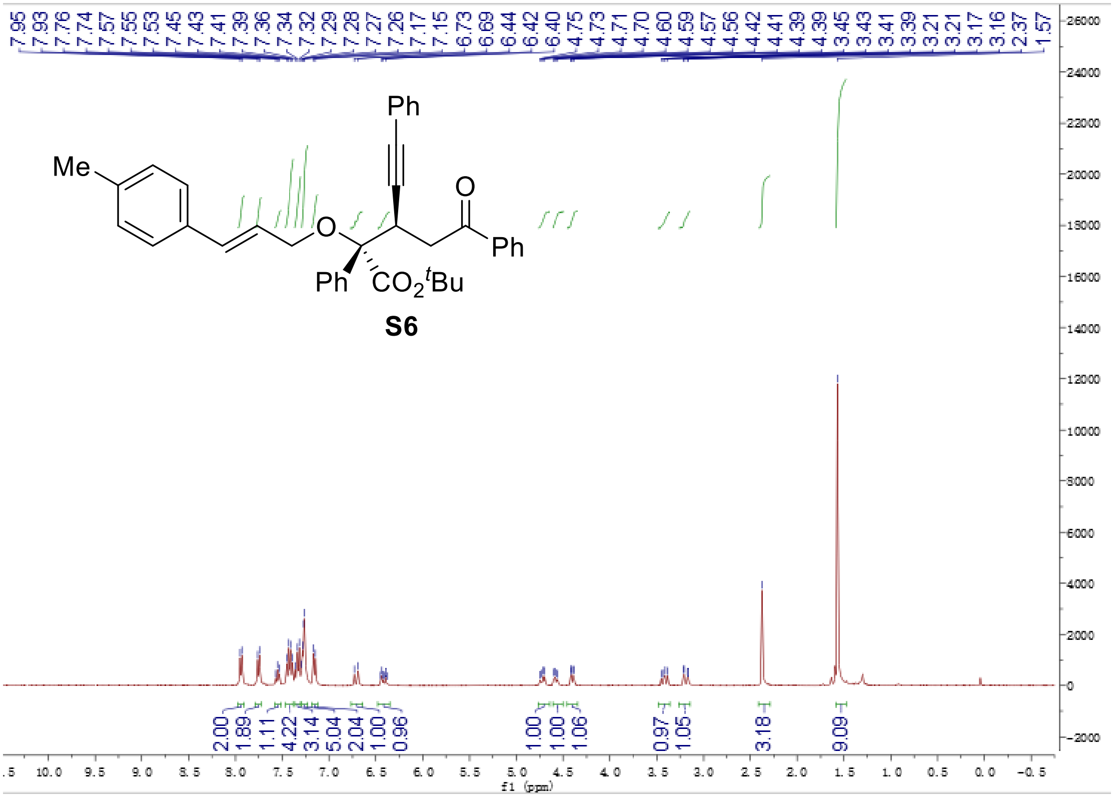

Supplementary Figure 505. ^{1}H NMR (400 MHz, $CDCl_3$) spectrum of **S6**.

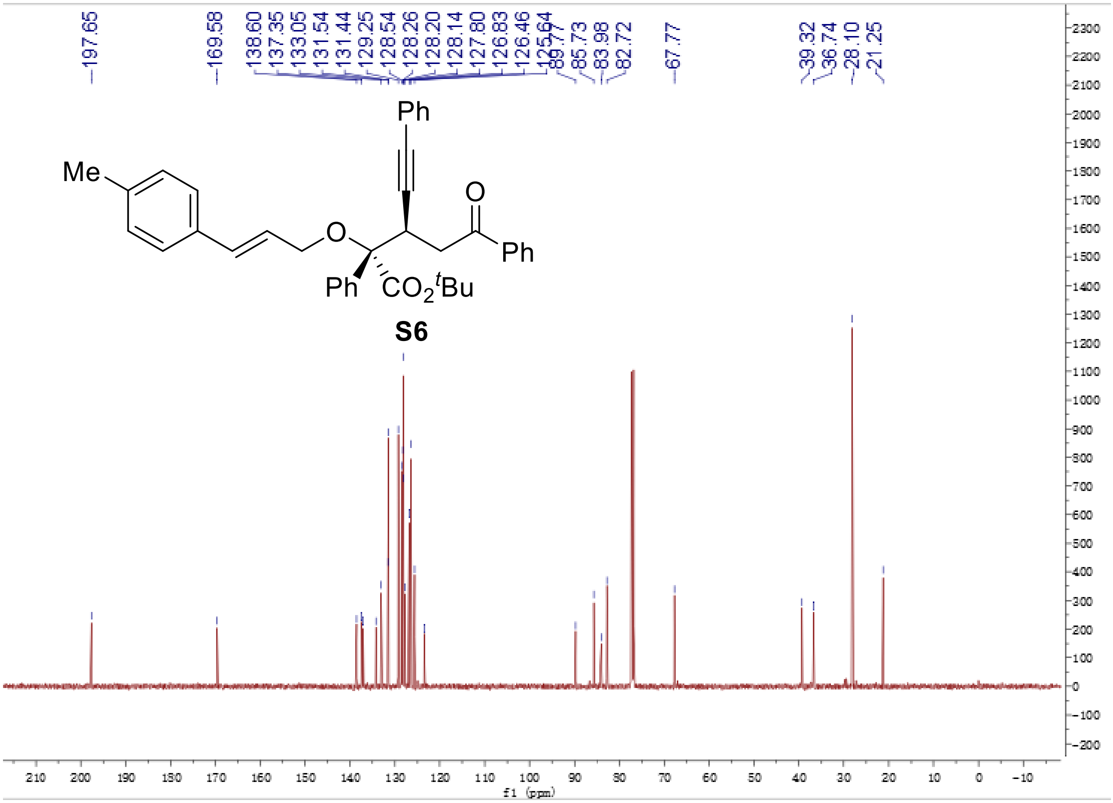

Supplementary Figure 506. ^{13}C NMR (101 MHz, $CDCl_3$) spectrum of **S6**.

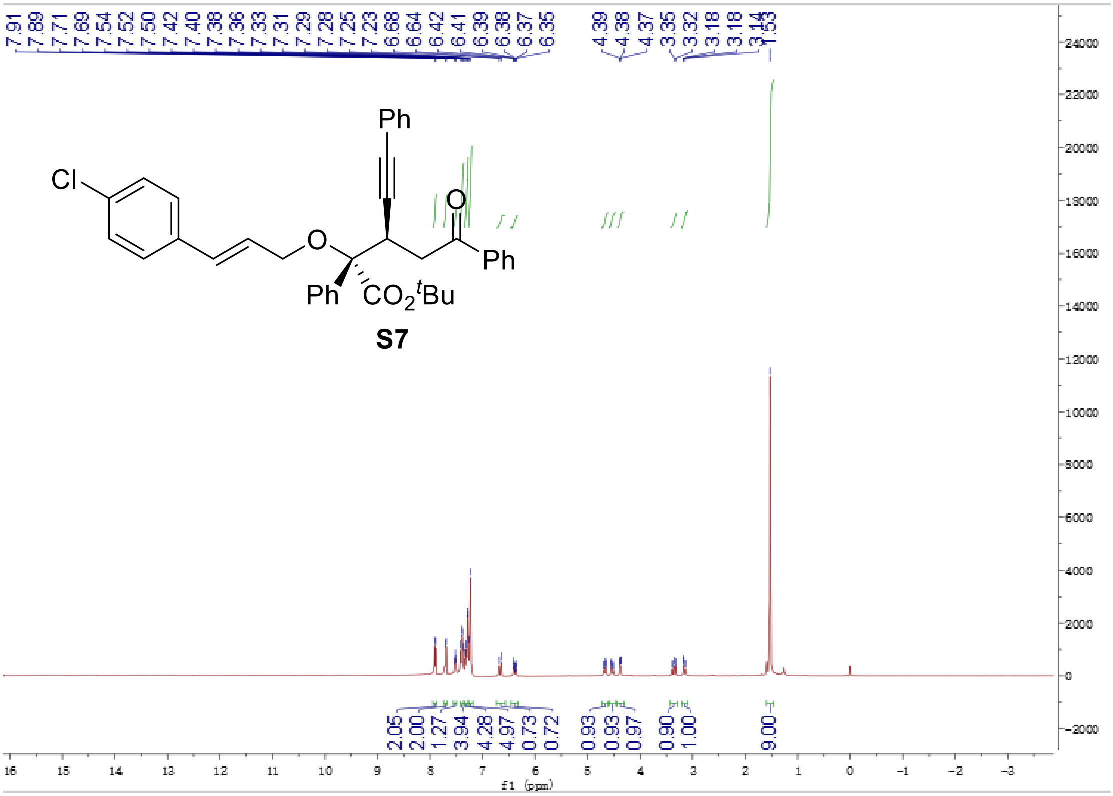

Supplementary Figure 507. ^{1}H NMR (400 MHz, $CDCl_3$) spectrum of **S7**.

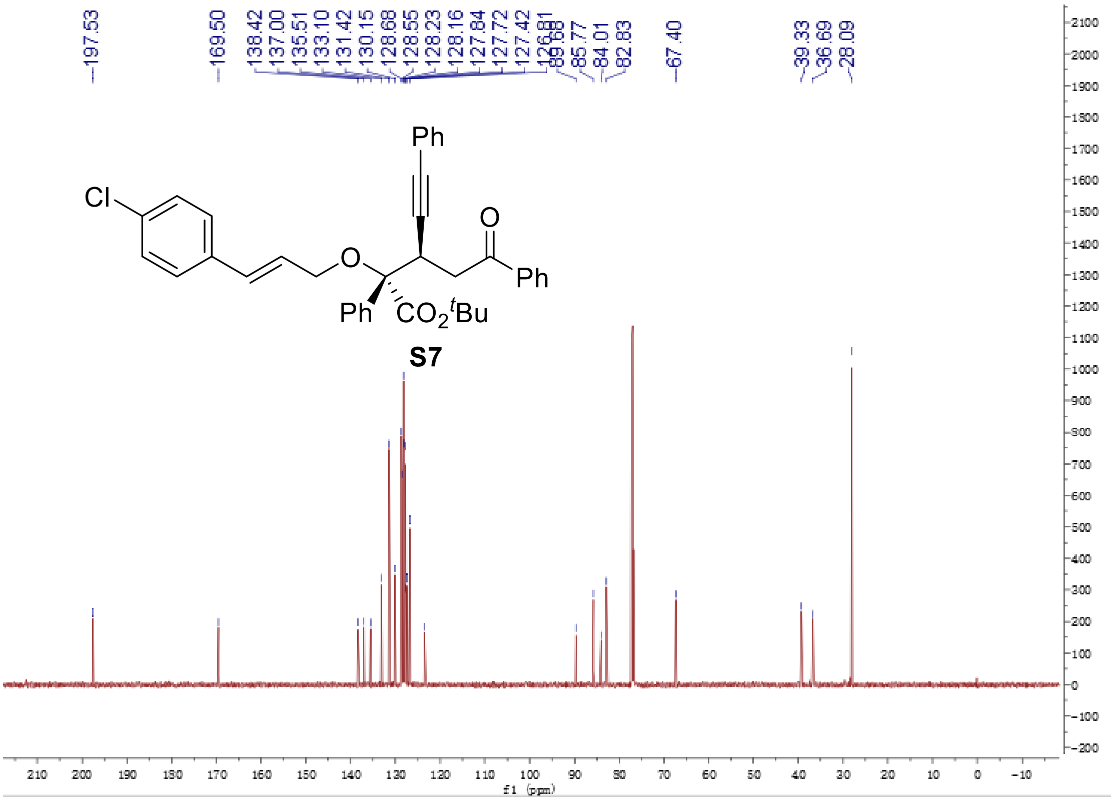

Supplementary Figure 508. ^{13}C NMR (101 MHz, $CDCl_3$) spectrum of **S7**.

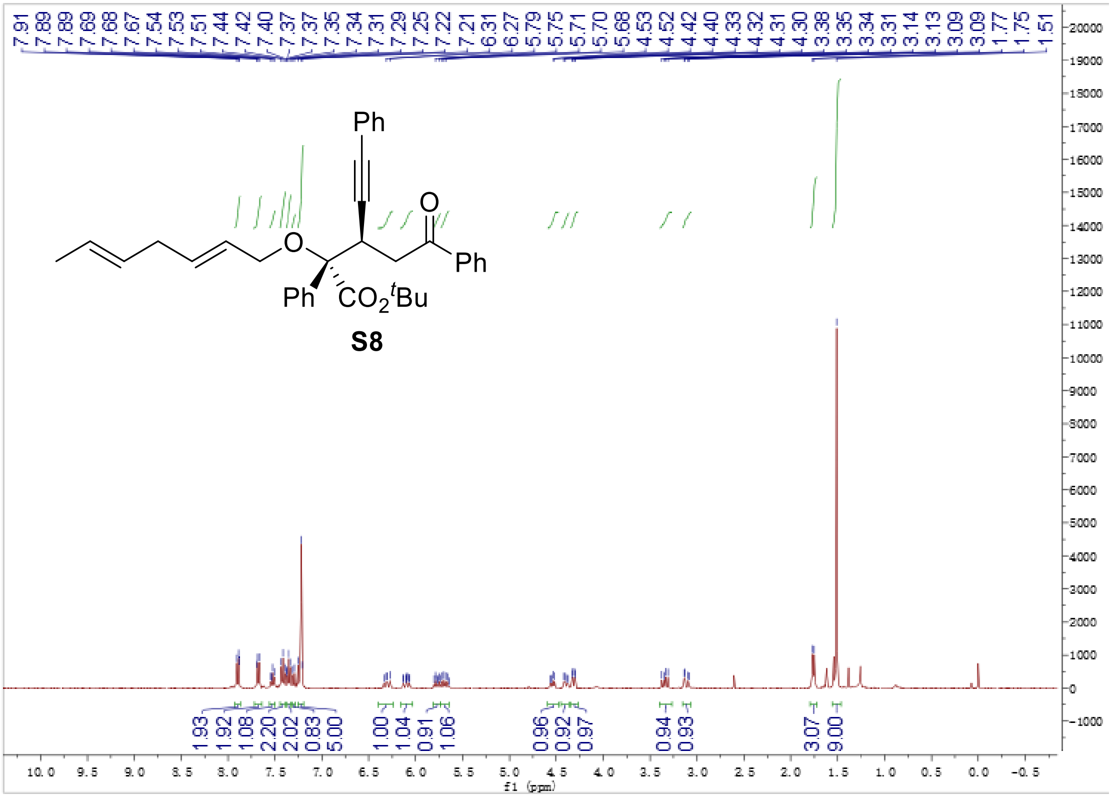

Supplementary Figure 509. ^{1}H NMR (400 MHz, $CDCl_3$) spectrum of **S8**.

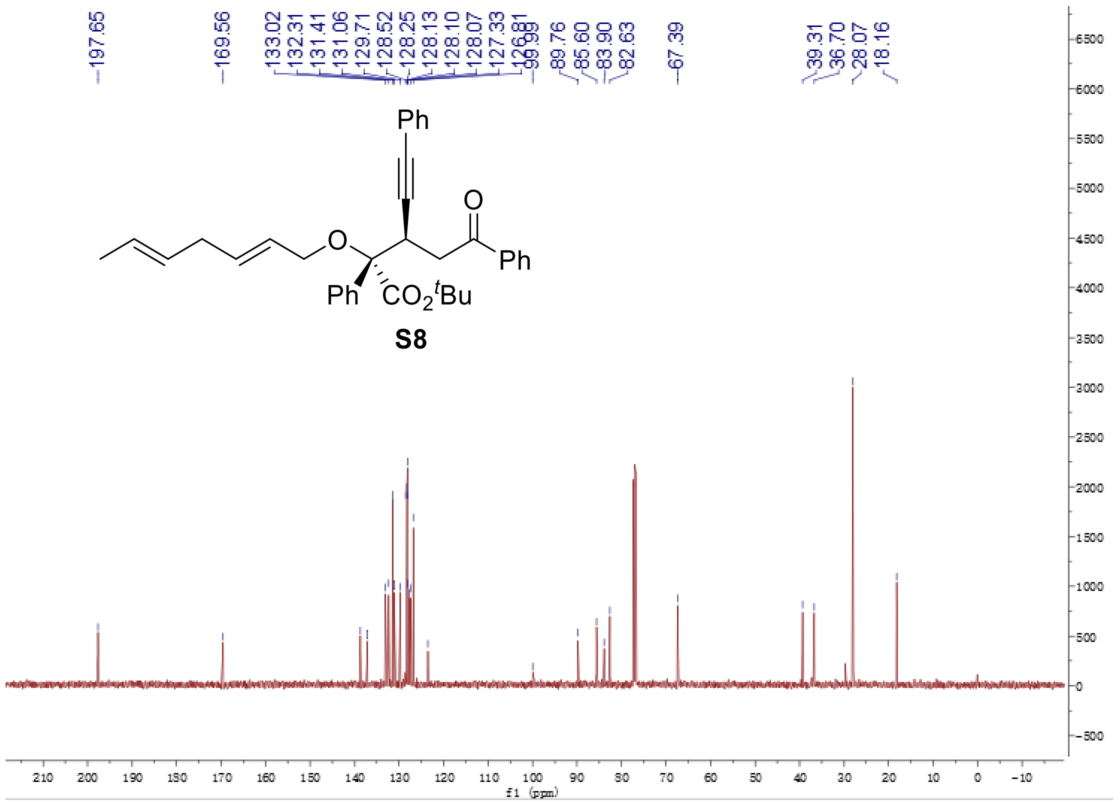

Supplementary Figure 510. ^{13}C NMR (101 MHz, $CDCl_3$) spectrum of **S8**.

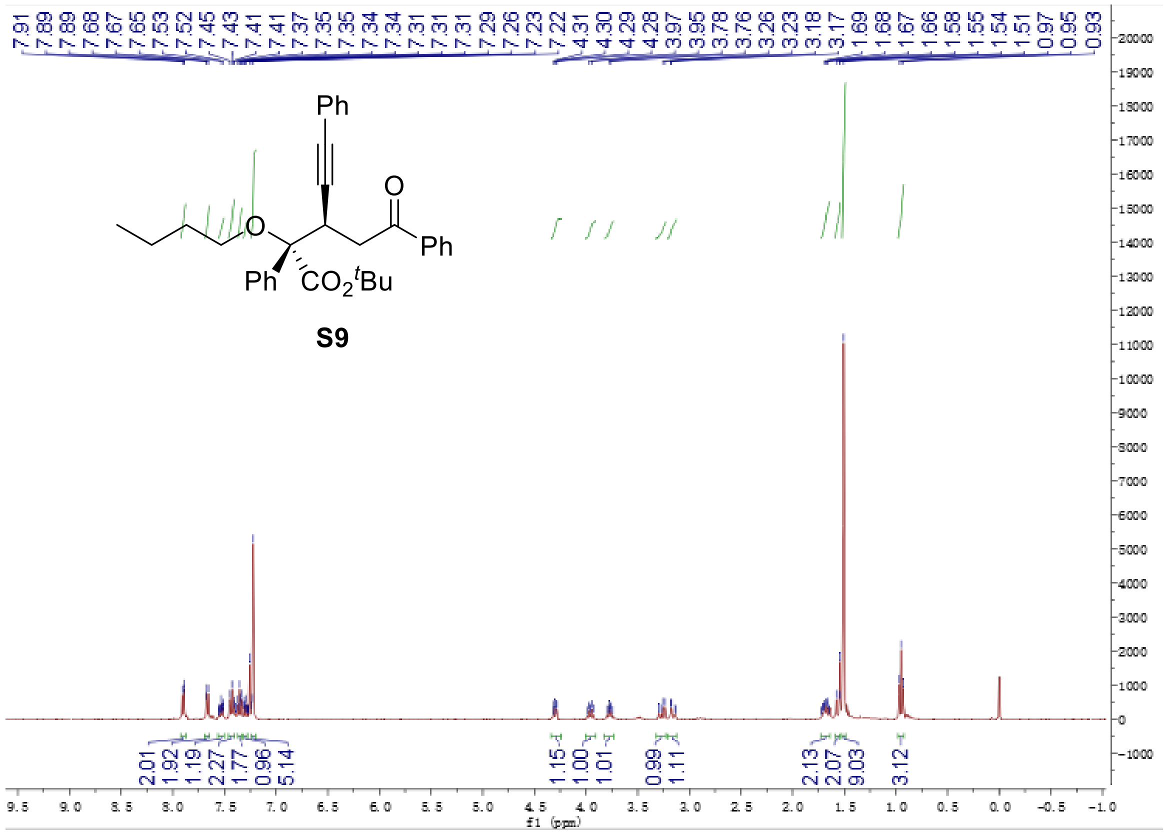

Supplementary Figure 511. ^{1}H NMR (400 MHz, $CDCl_3$) spectrum of **S9**.

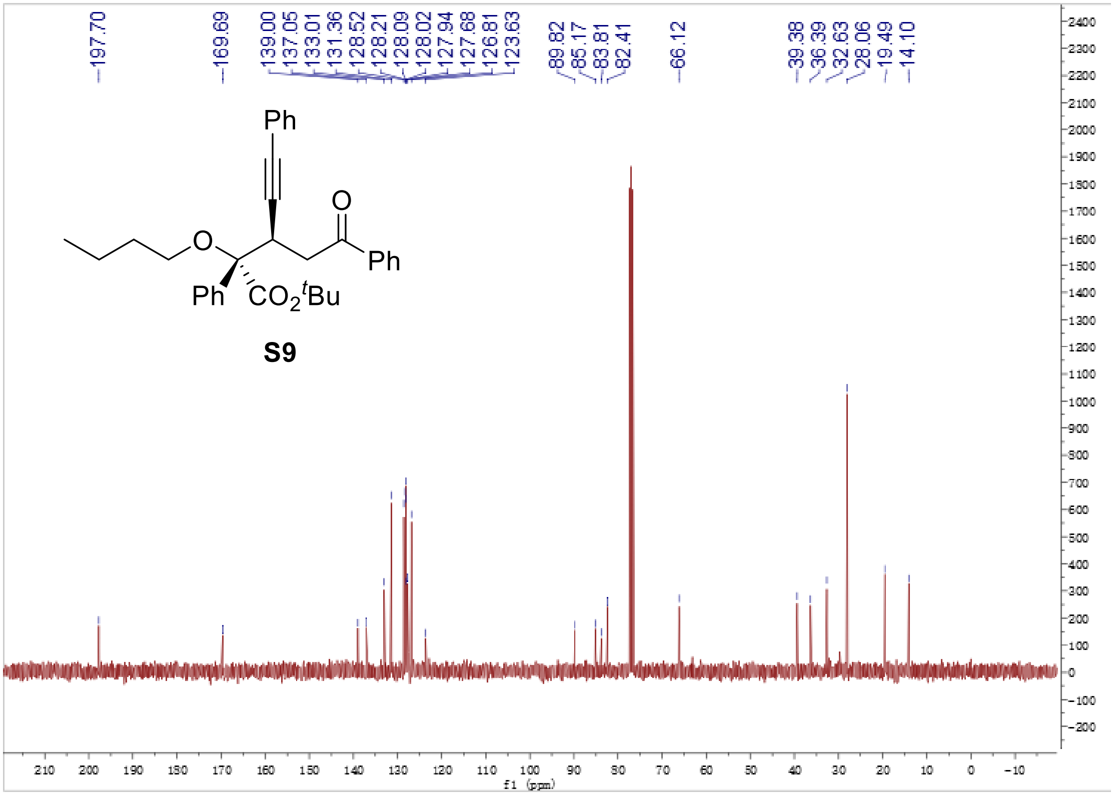

Supplementary Figure 512. ^{13}C NMR (101 MHz, $CDCl_3$) spectrum of **S9**.

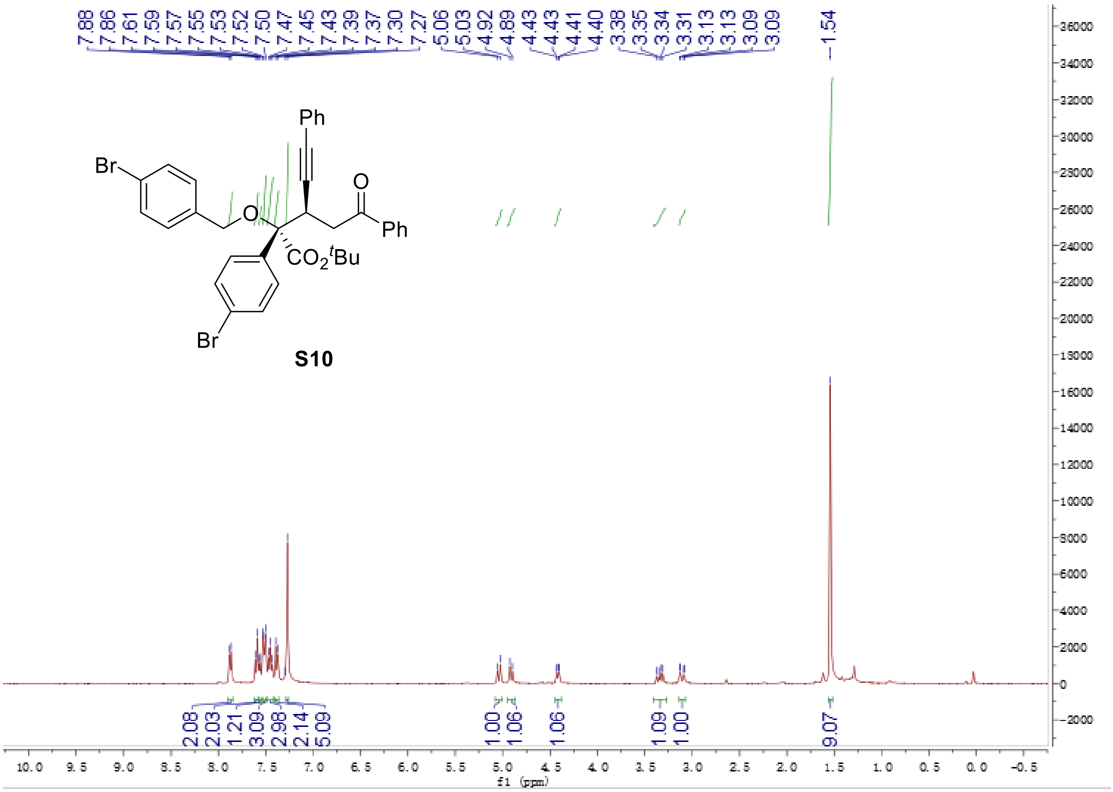

Supplementary Figure 513. ^{1}H NMR (400 MHz, $CDCl_3$) spectrum of **S10**.

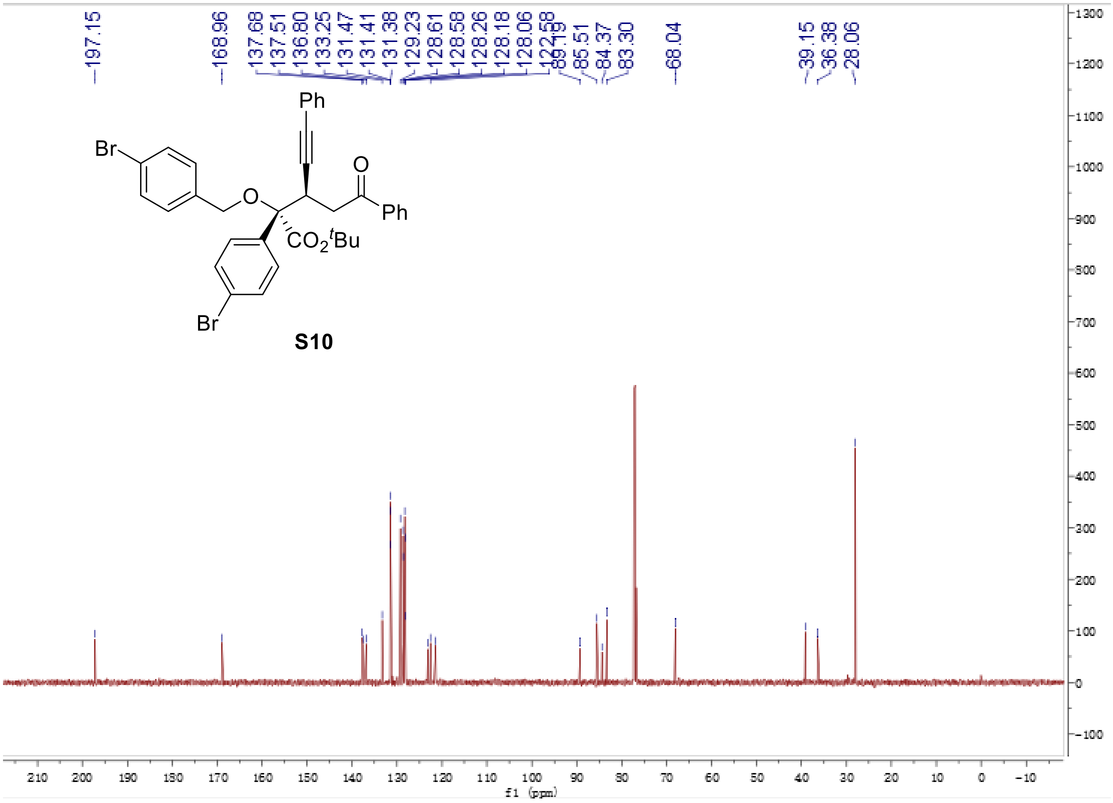

Supplementary Figure 514. ^{13}C NMR (101 MHz, $CDCl_3$) spectrum of **S10**.

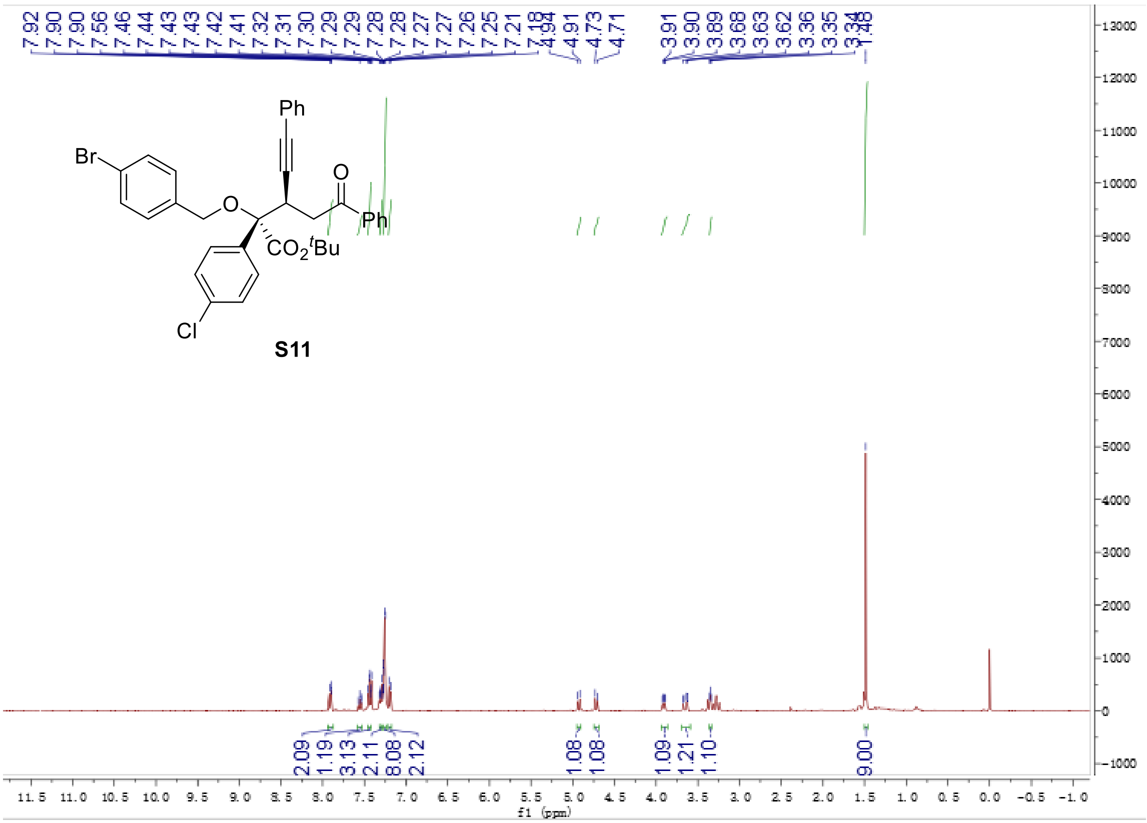

Supplementary 515. ^{1}H NMR (400 MHz, $CDCl_3$) spectrum of **S11**.

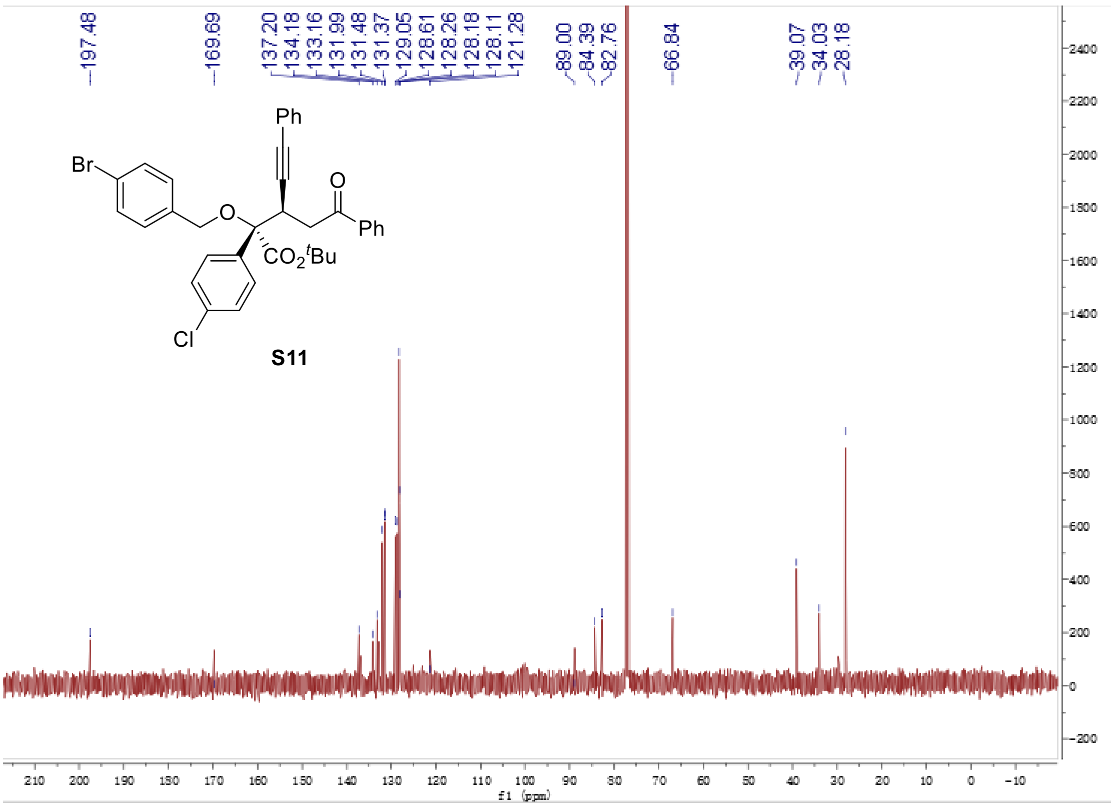

Supplementary 516. ^{13}C NMR (101 MHz, $CDCl_3$) spectrum of **S11**.

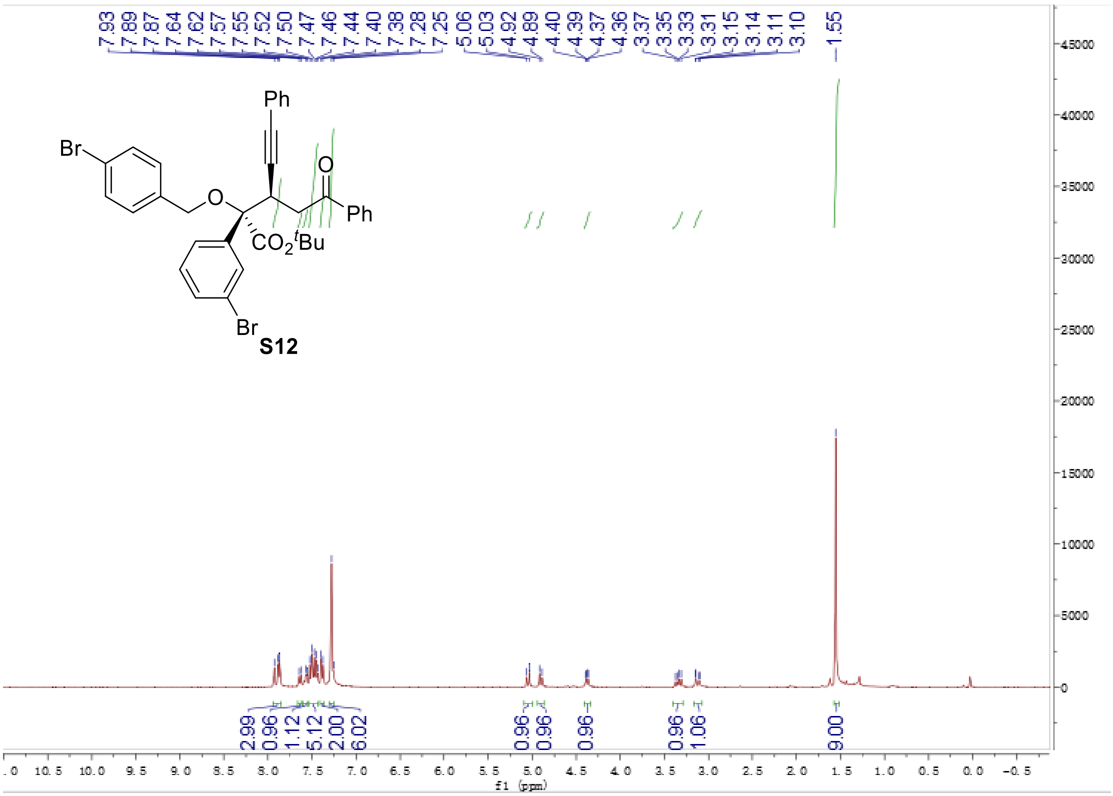

Supplementary Figure 517. ^{1}H NMR (400 MHz, $CDCl_3$) spectrum of **S12**.

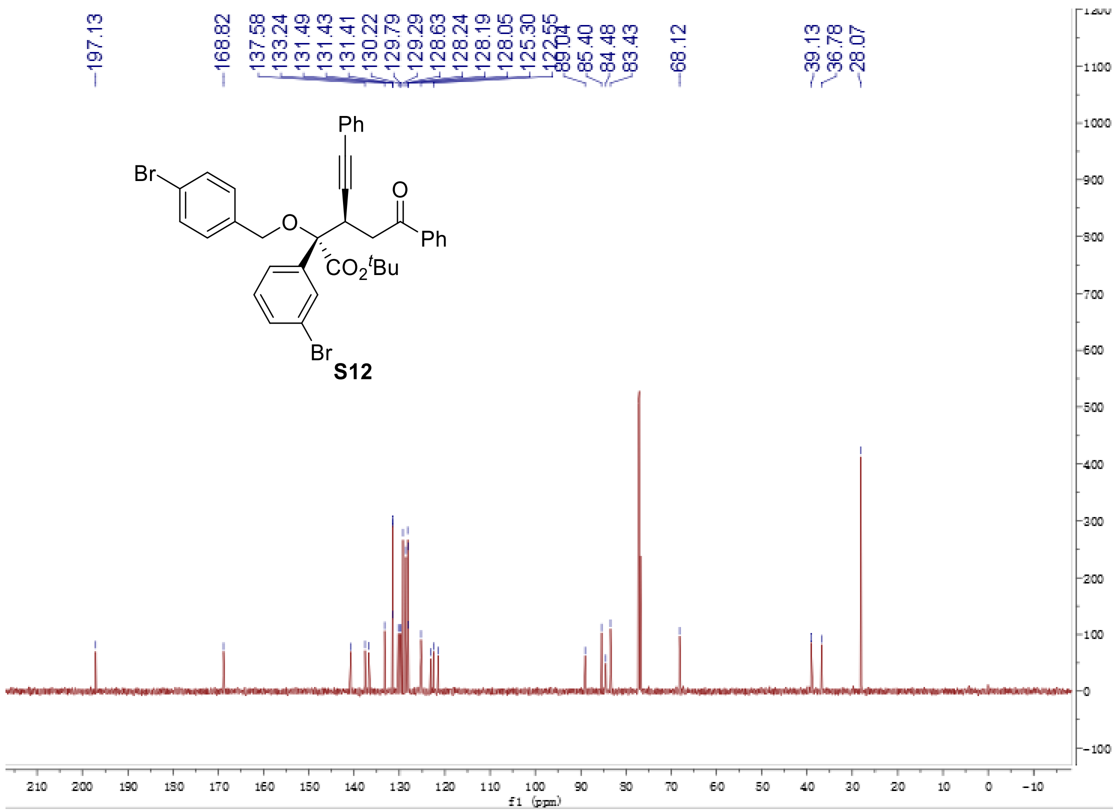

Supplementary Figure 518. ^{13}C NMR (101 MHz, $CDCl_3$) spectrum of **S12**.

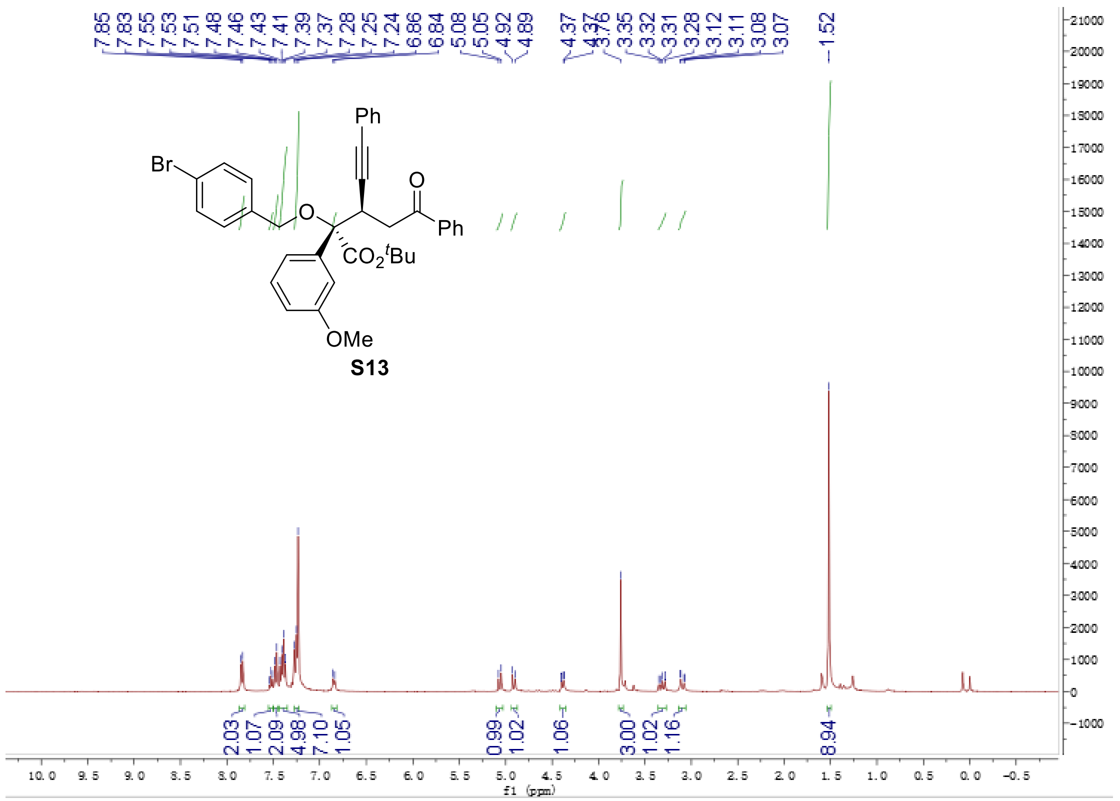

Supplementary Figure 519. ^{1}H NMR (400 MHz, $CDCl_3$) spectrum of **S13**.

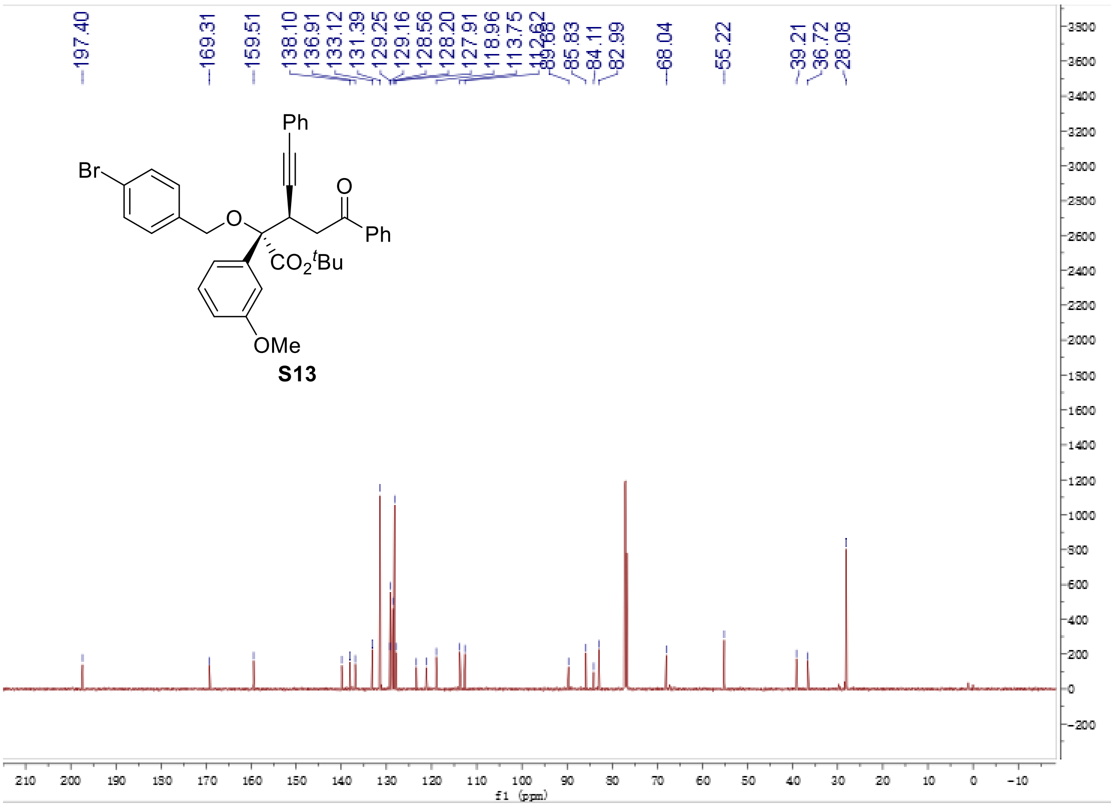

Supplementary Figure 520. ^{13}C NMR (101 MHz, $CDCl_3$) spectrum of **S13**.

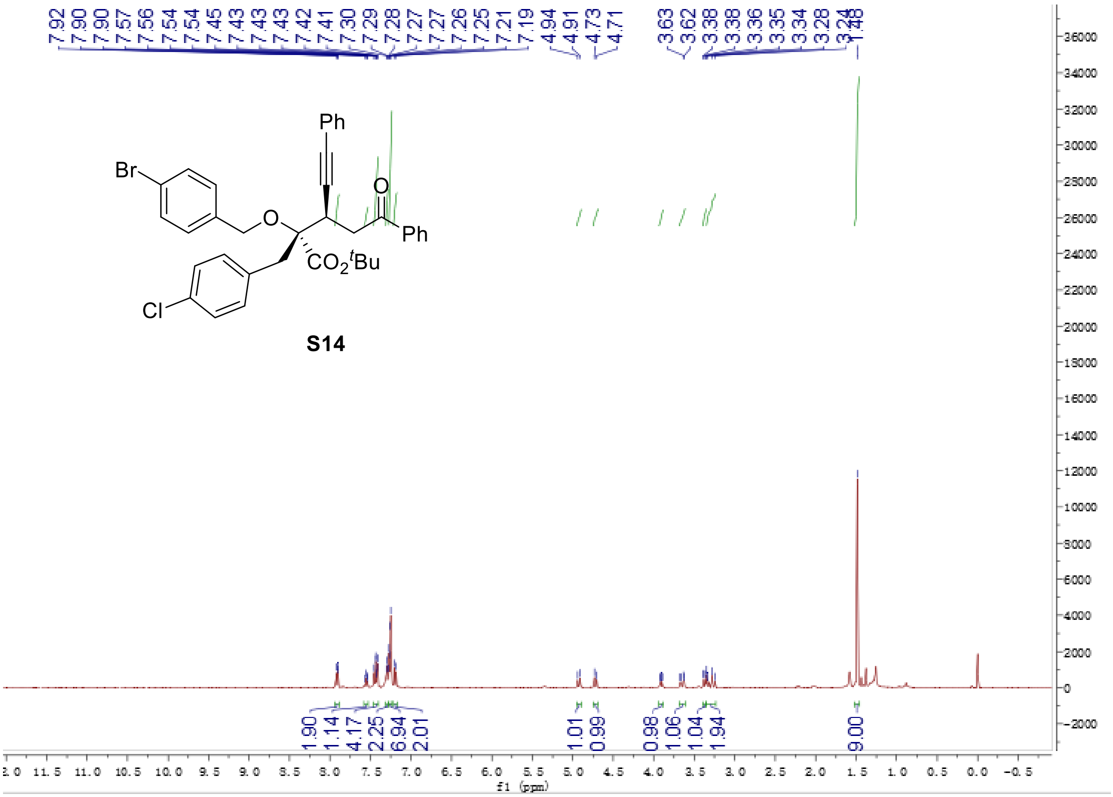

Supplementary Figure 521. ^{1}H NMR (400 MHz, $CDCl_3$) spectrum of **S14**.

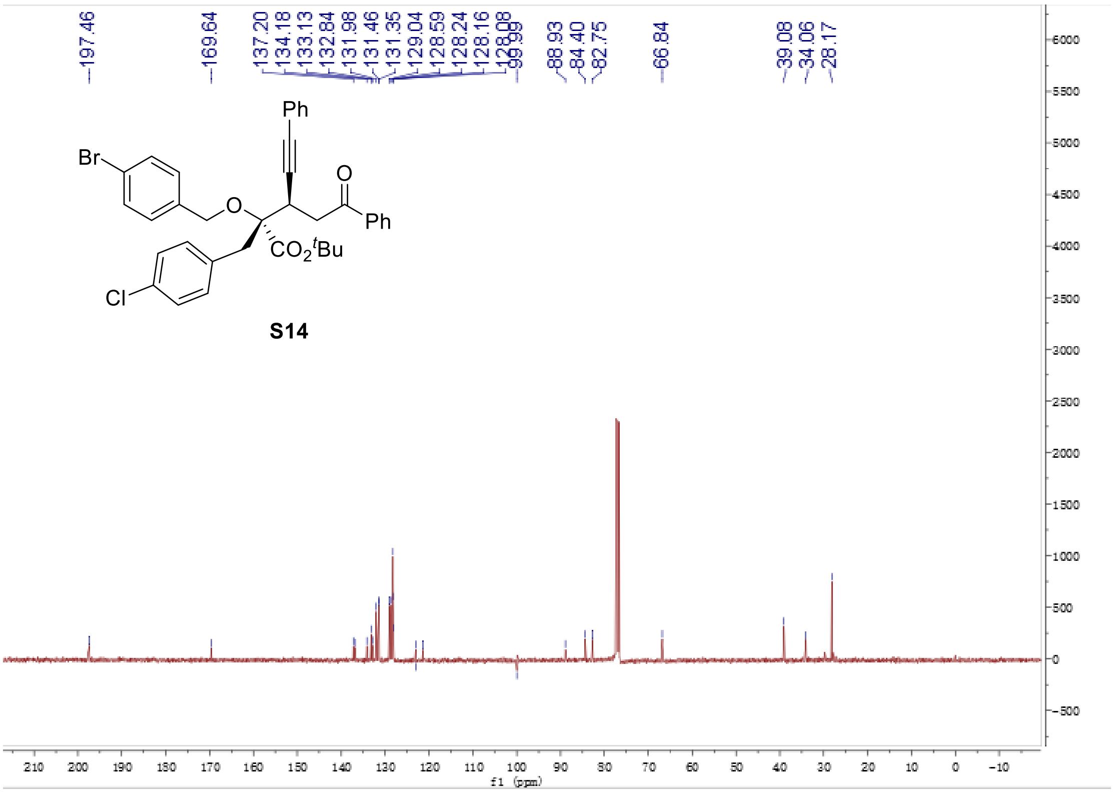

Supplementary Figure 522. ^{13}C NMR (101 MHz, $CDCl_3$) spectrum of **S14**.

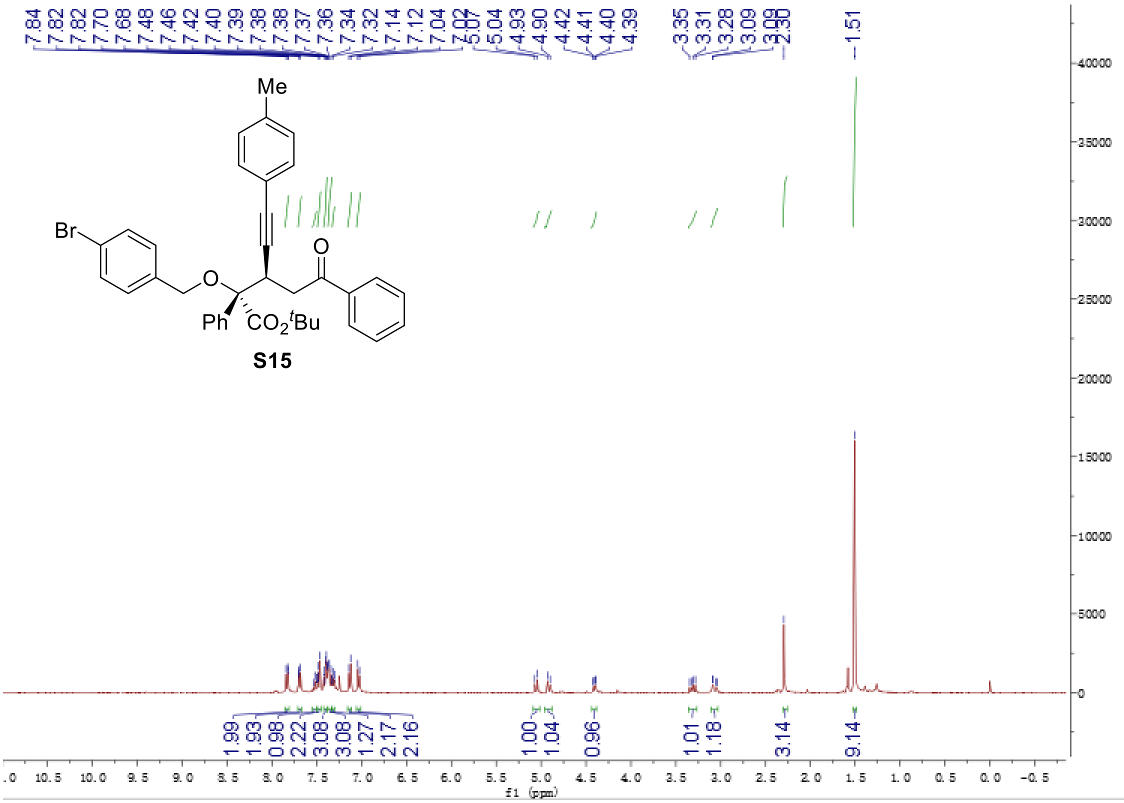

Supplementary Figure 523. ^{1}H NMR (400 MHz, $CDCl_3$) spectrum of **S15**.

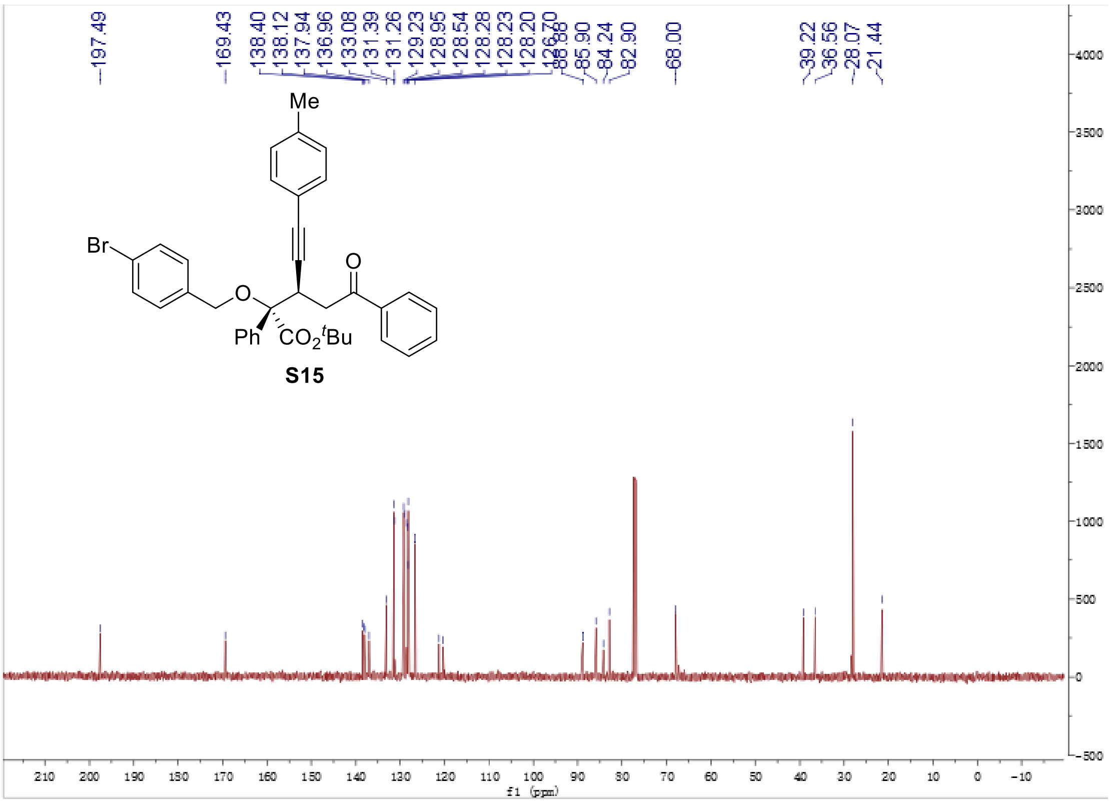

Supplementary Figure 524. ^{13}C NMR (101 MHz, $CDCl_3$) spectrum of **S15**.

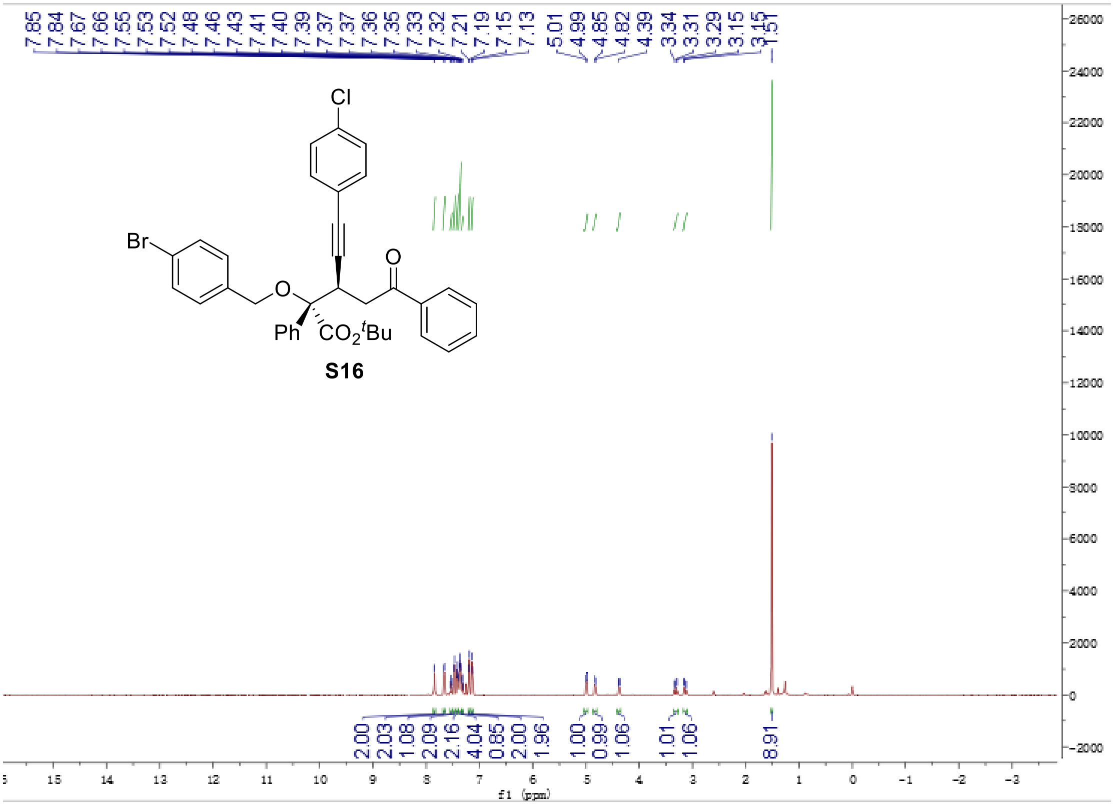

Supplementary Figure 525. ^{1}H NMR (500 MHz, acetone-d6) spectrum of **S16**.

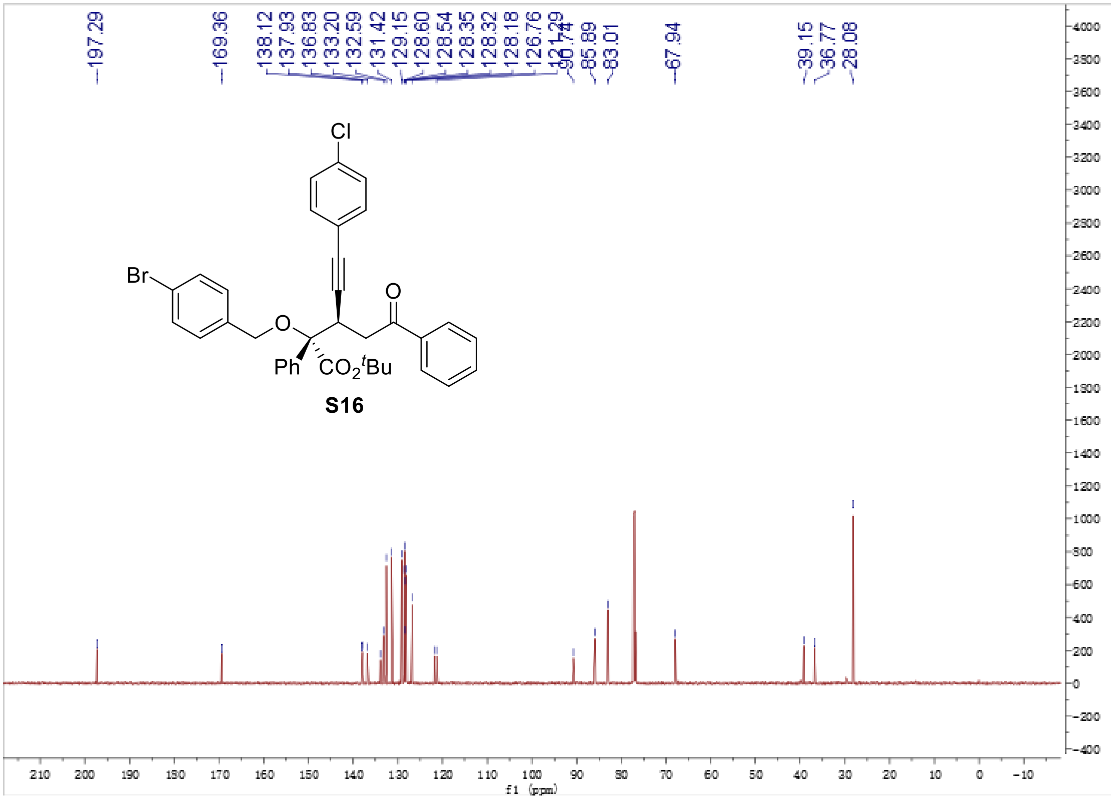

Supplementary Figure 526. ^{13}C NMR (126 MHz, acetone-d6) spectrum of **S16**.

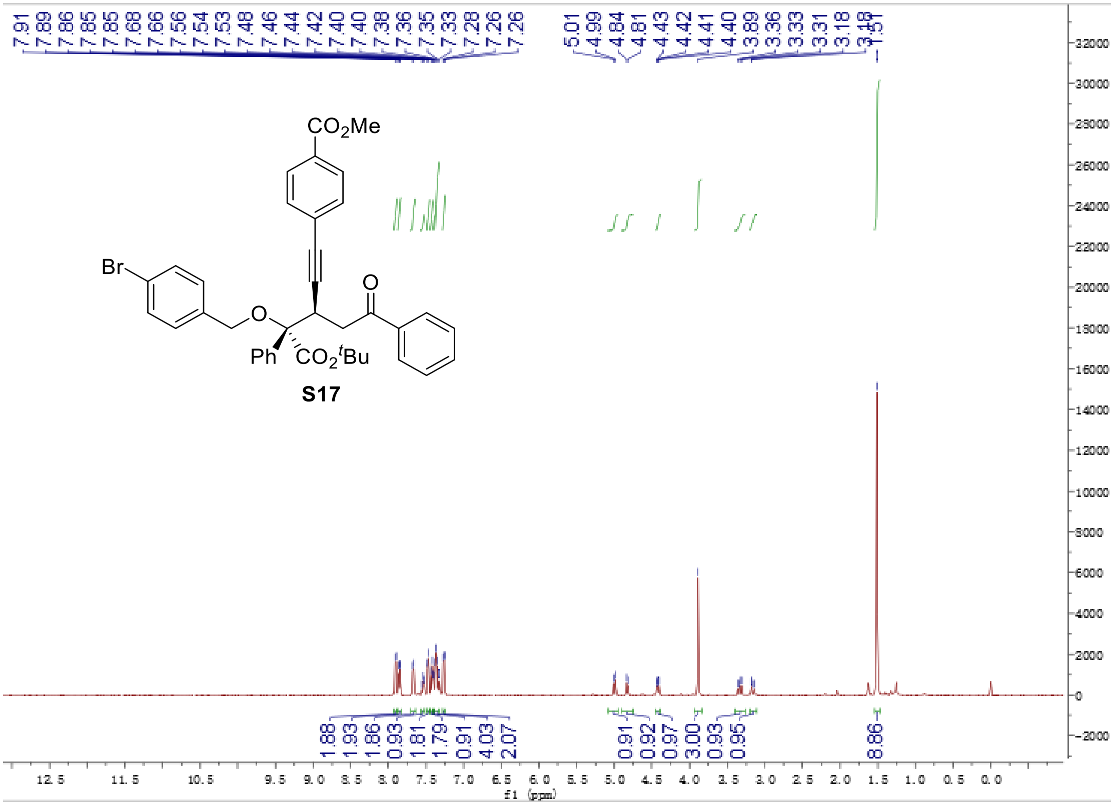

Supplementary Figure 527. ^1H NMR (500 MHz, acetone-d6) spectrum of **S17**.

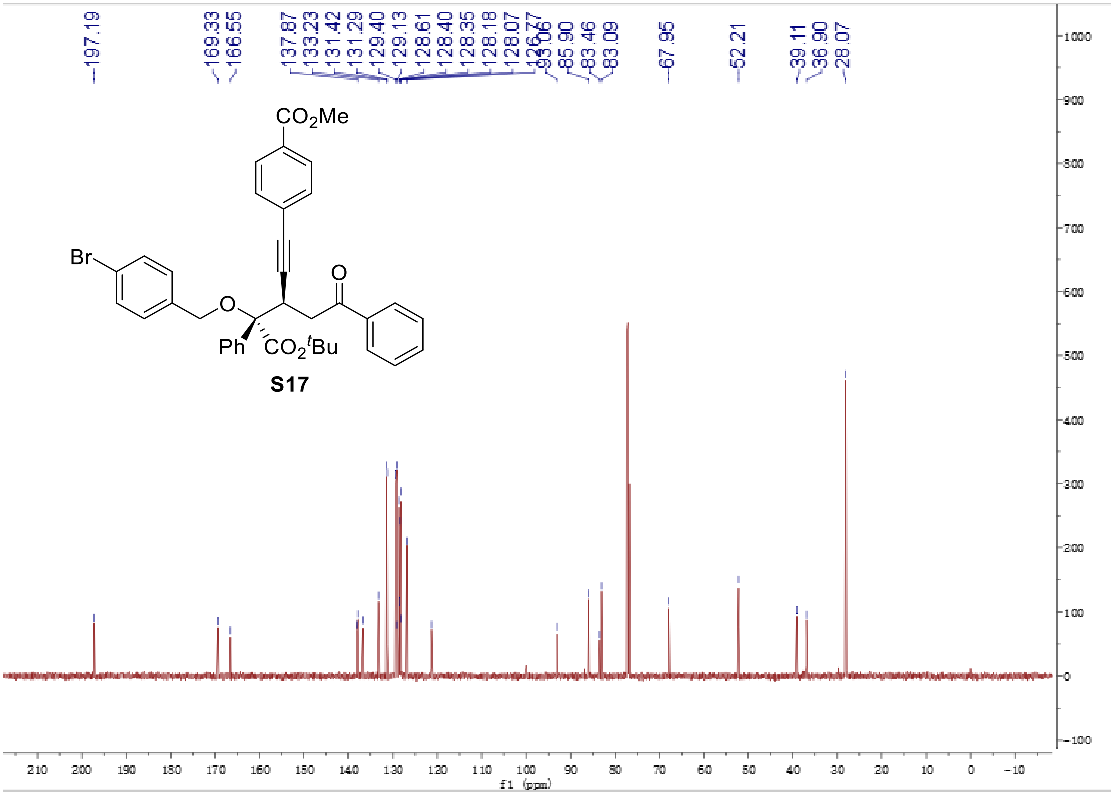

Supplementary Figure 528. ^{13}C NMR (126 MHz, acetone-d6) spectrum of **S17**.

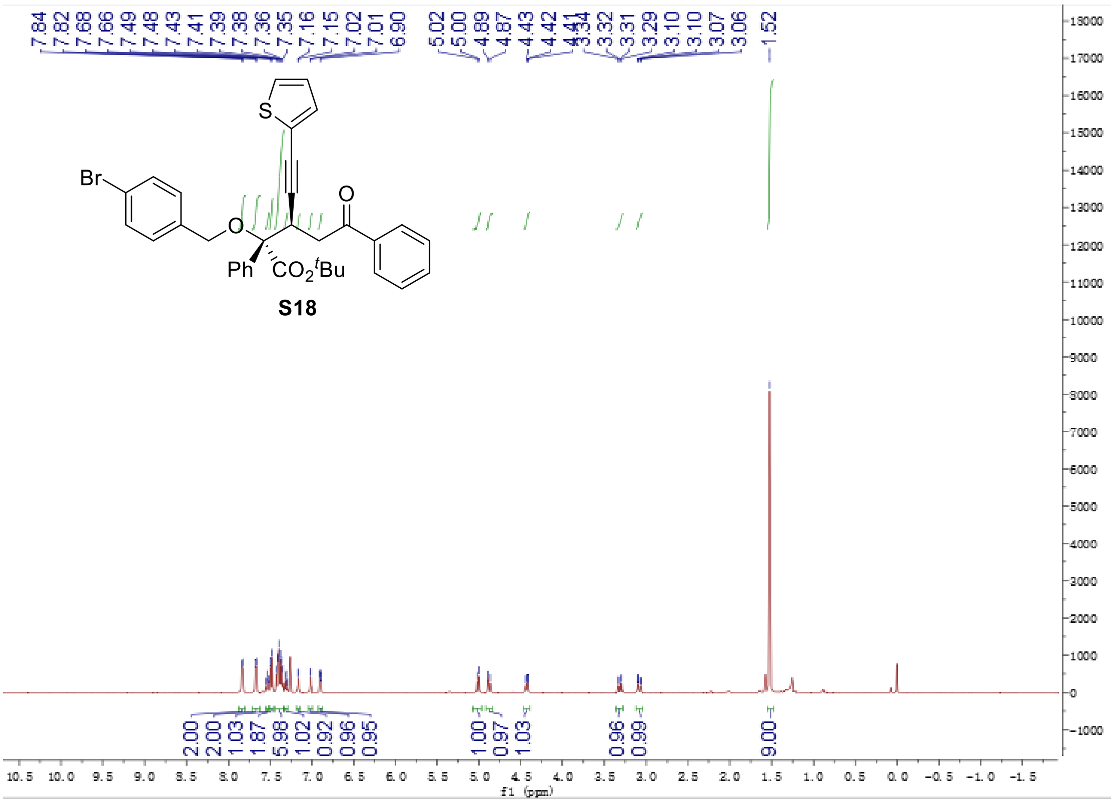

Supplementary Figure 529. ^{1}H NMR (500 MHz, acetone-d6) spectrum of **S18**.

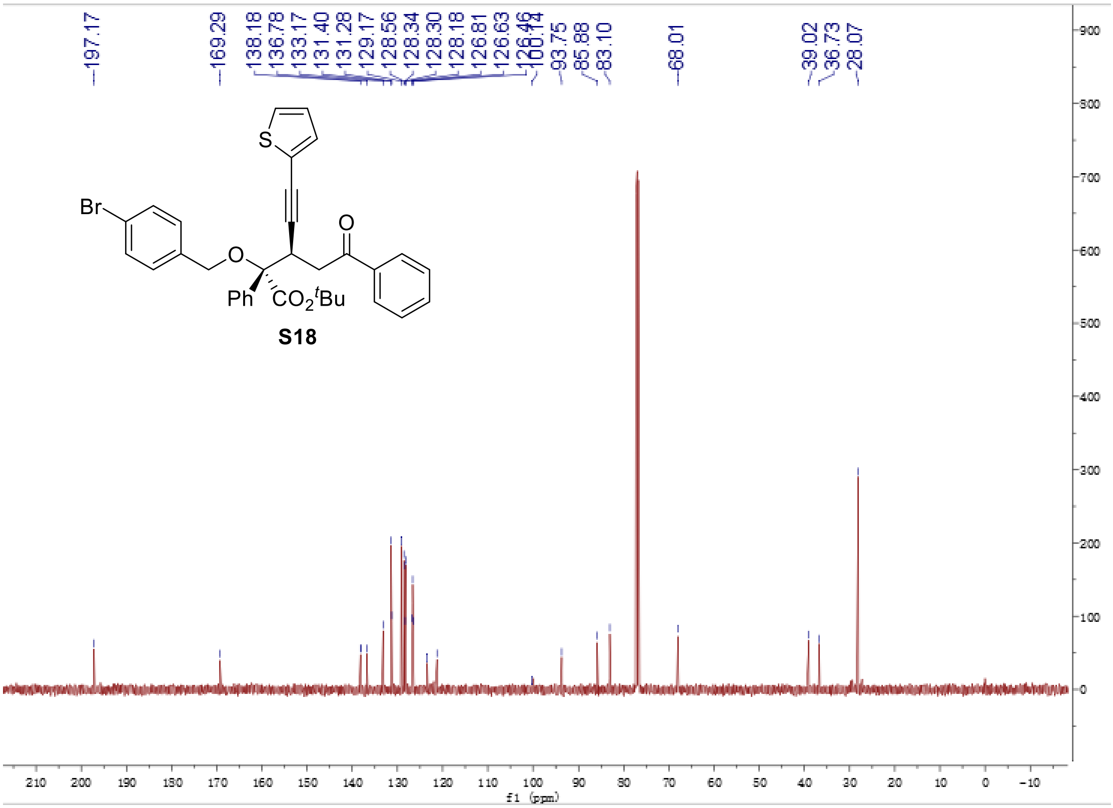

Supplementary Figure 530. ^{13}C NMR (126 MHz, acetone-d6) spectrum of **S18**.

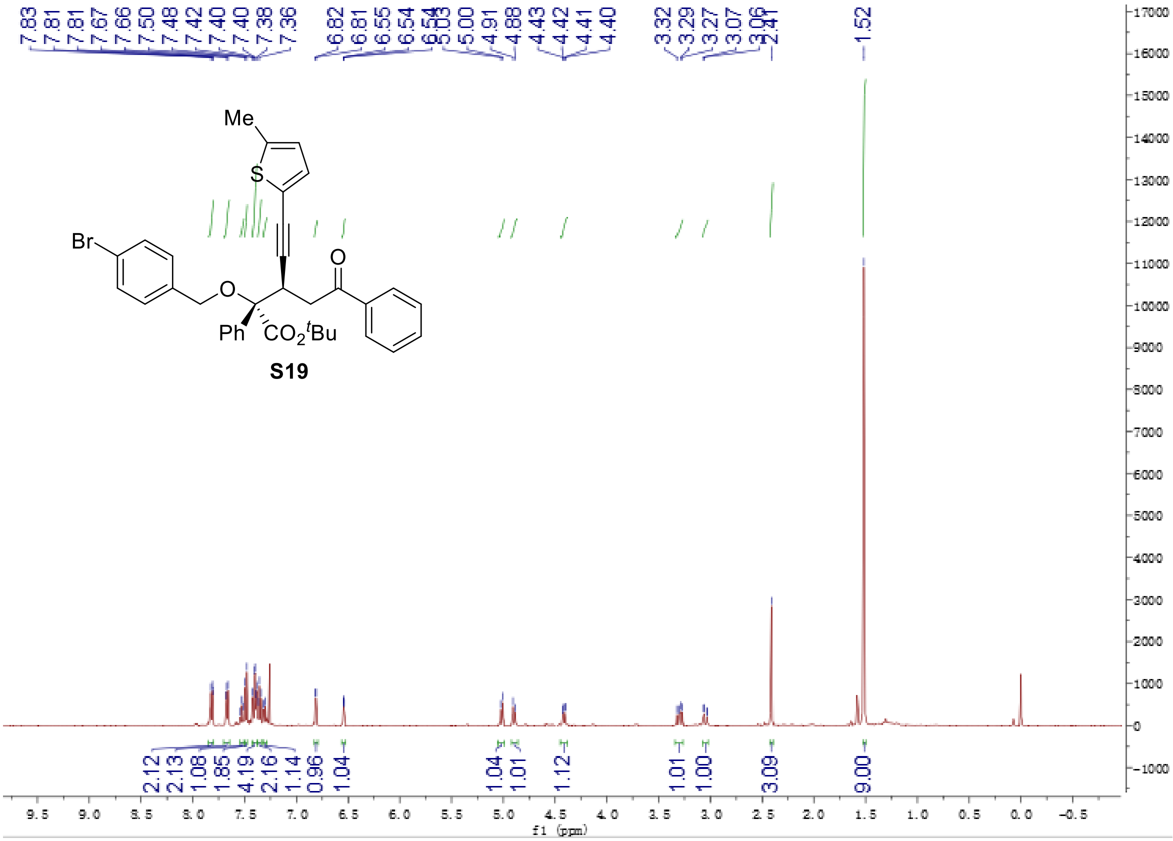

Supplementary Figure 531. ^{1}H NMR (500 MHz, acetone-d6) spectrum of **S19**.

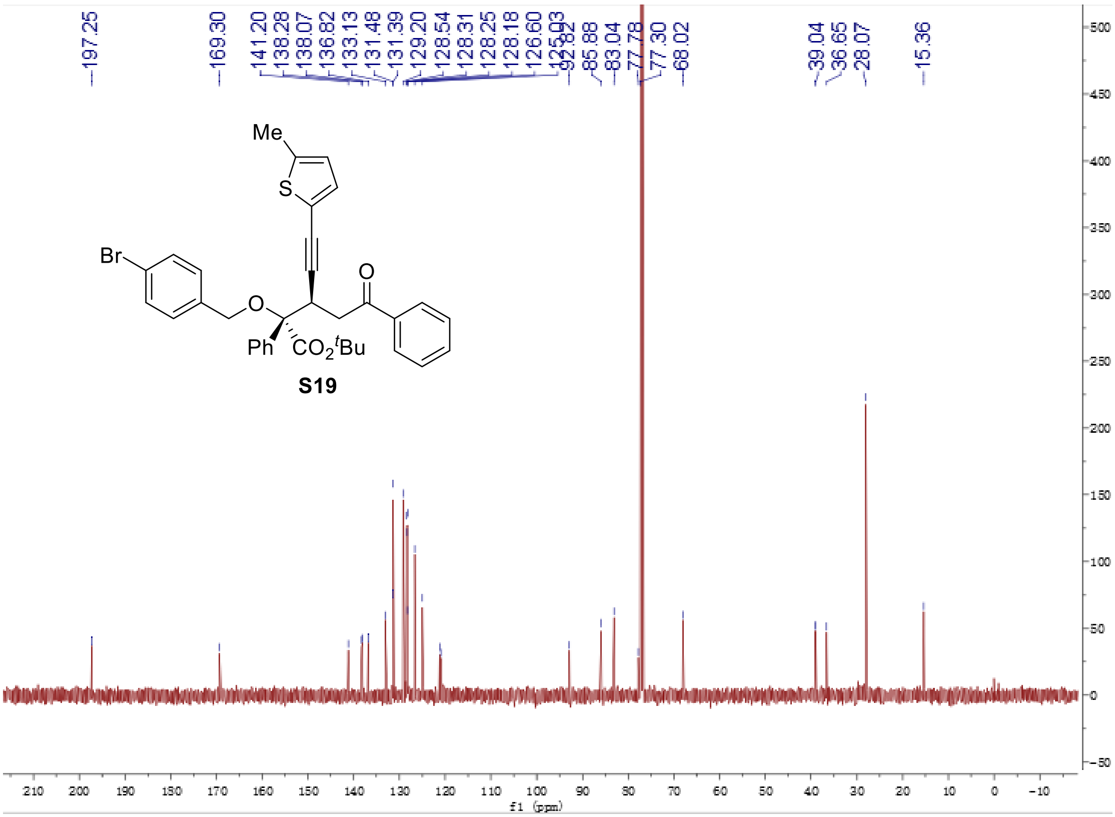

Supplementary Figure 532. ^{13}C NMR (126 MHz, acetone-d6) spectrum of **S19**.

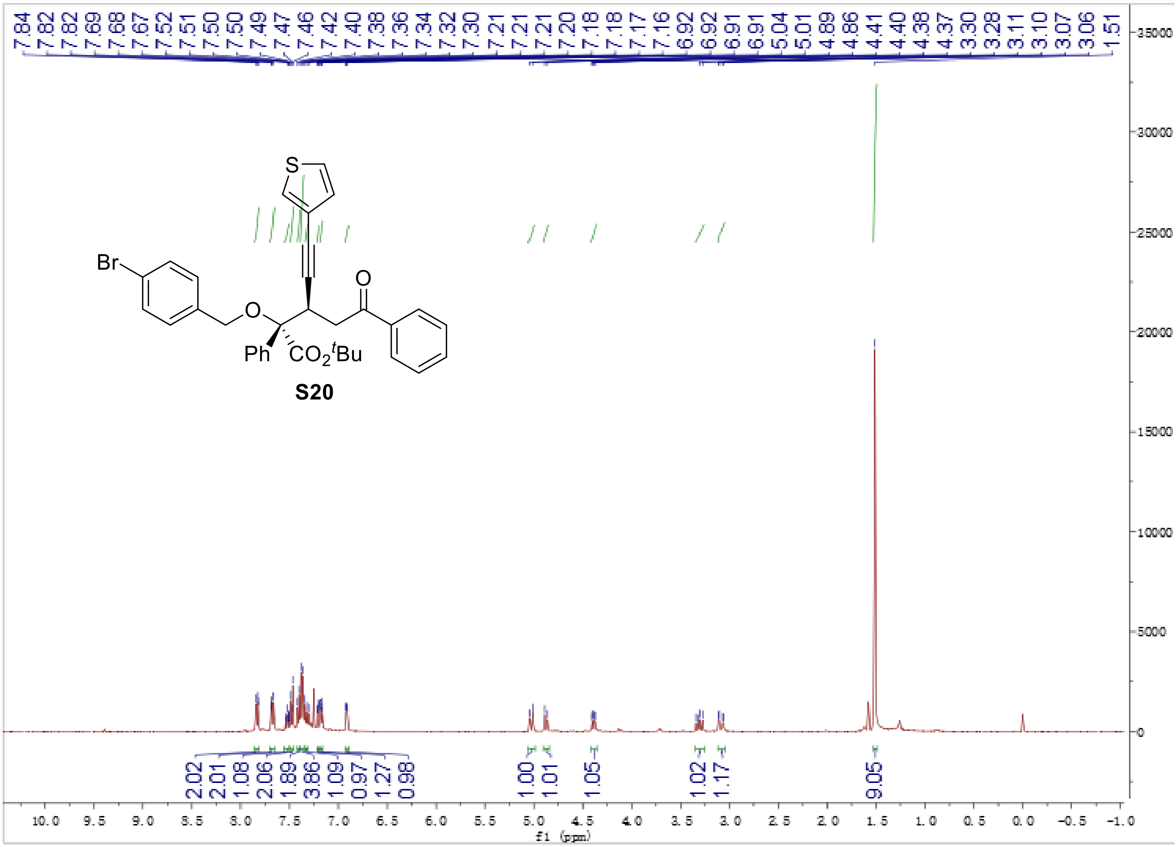

Supplementary Figure 533. ^{1}H NMR (500 MHz, $CDCl_3$) spectrum of **S20**.

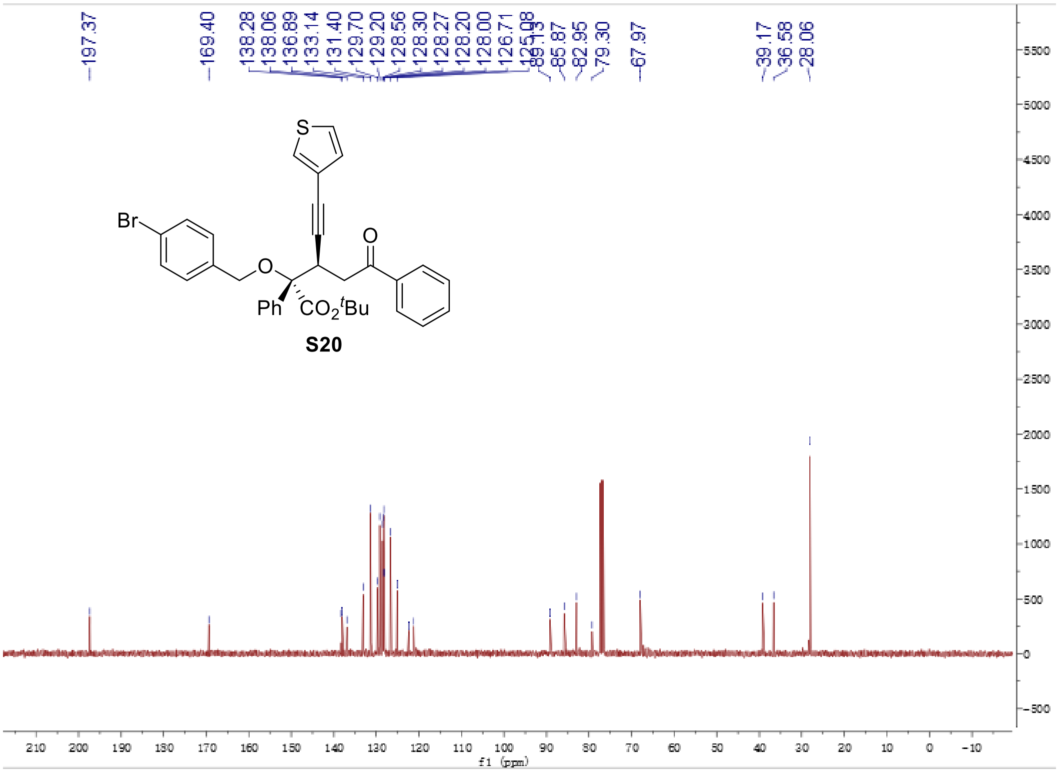

Supplementary Figure 534. ^{13}C NMR (126 MHz, $CDCl_3$) spectrum of **S20**.

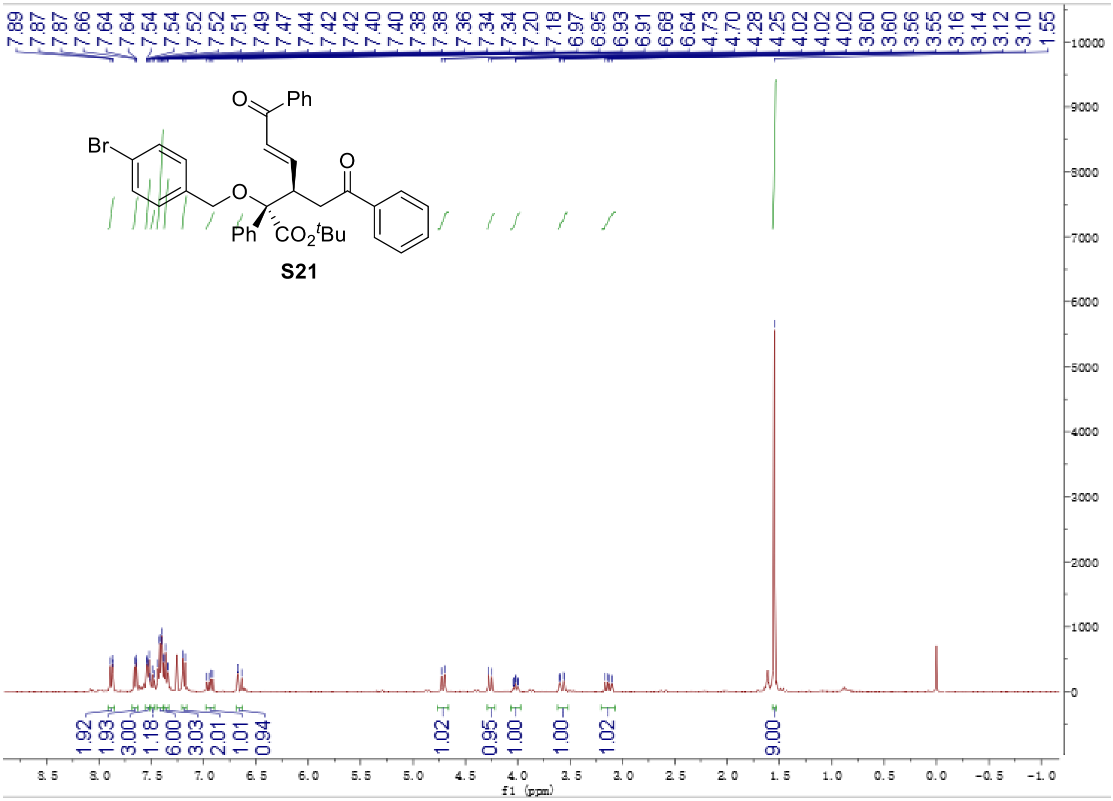

Supplementary Figure 535. ^{1}H NMR (500 MHz, $CDCl_3$) spectrum of **S21**.

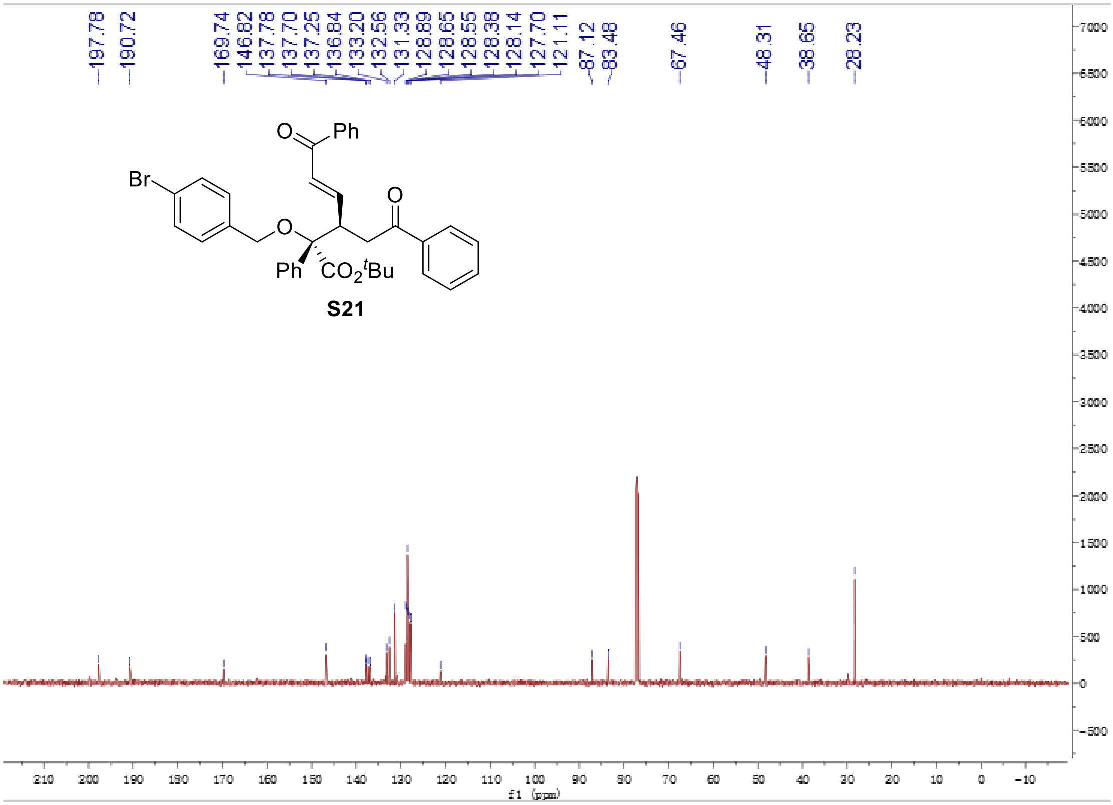

Supplementary Figure 536. ^{13}C NMR (126 MHz, $CDCl_3$) spectrum of **S21**.

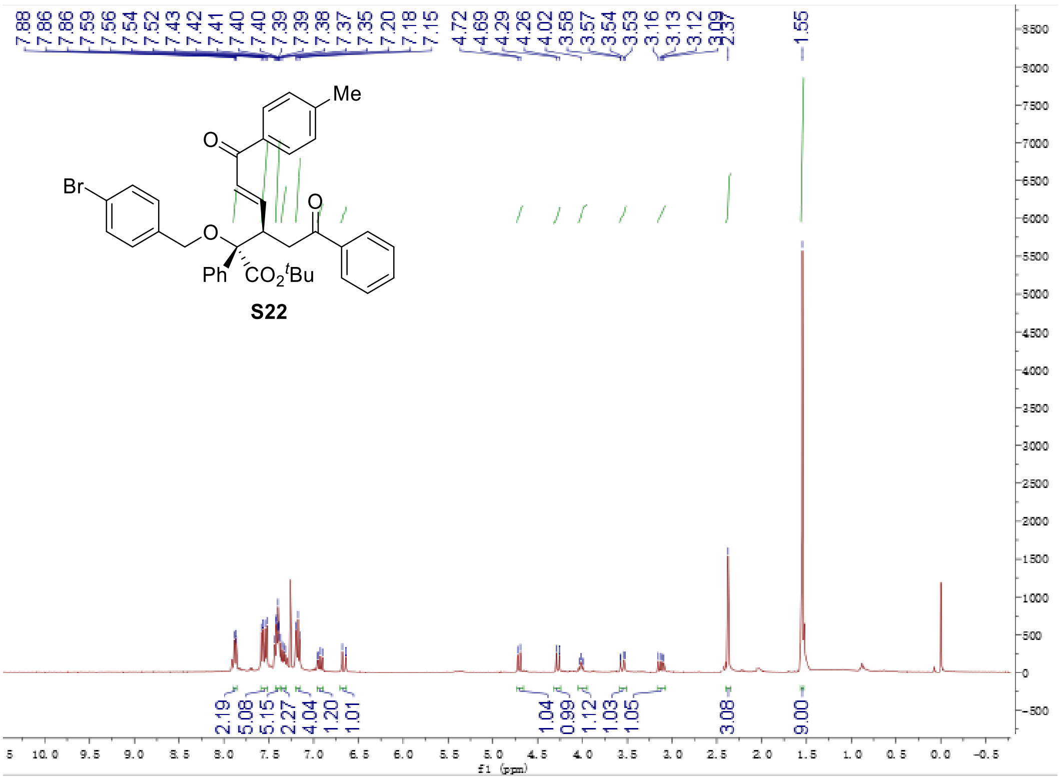

Supplementary Figure 537. ^{1}H NMR (500 MHz, $CDCl_3$) spectrum of **S22**.

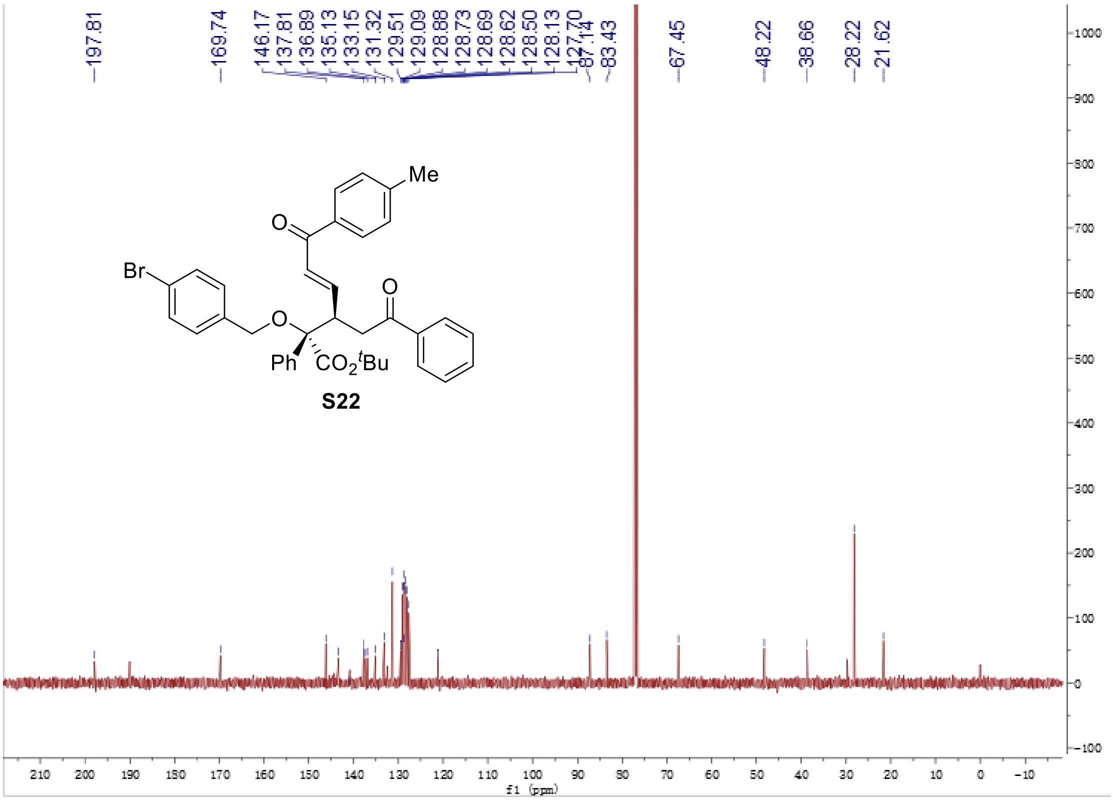

Supplementary Figure 538. ^{13}C NMR (126 MHz, $CDCl_3$) spectrum of **S22**.

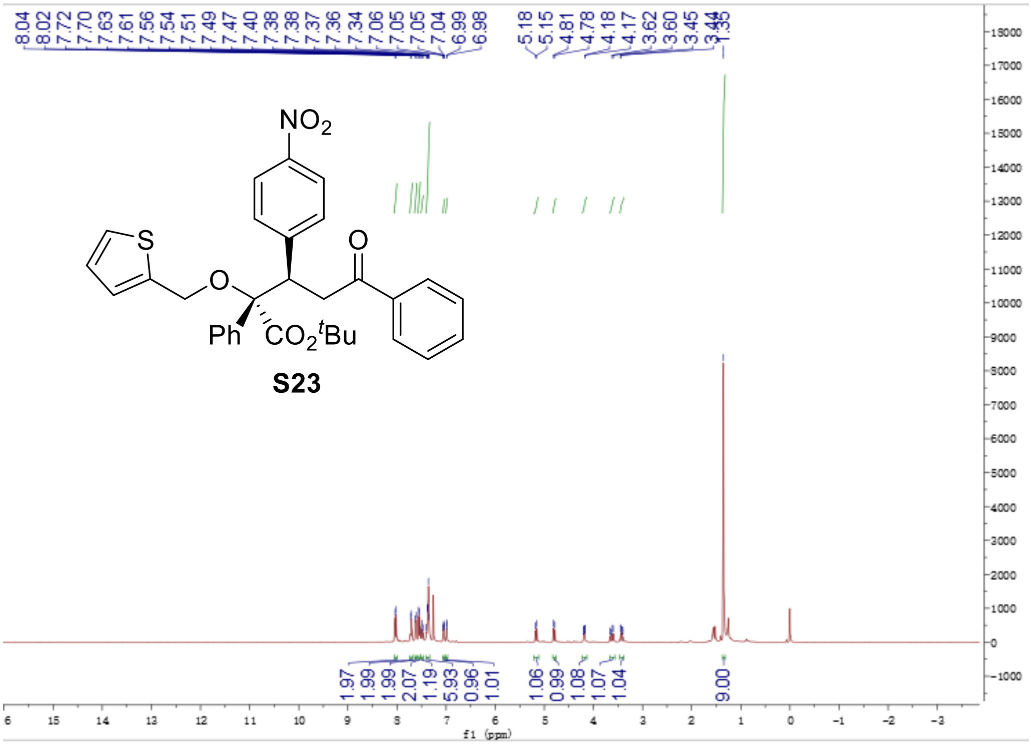

Supplementary Figure 539. ^{1}H NMR (500 MHz, $CDCl_3$) spectrum of **S23**.

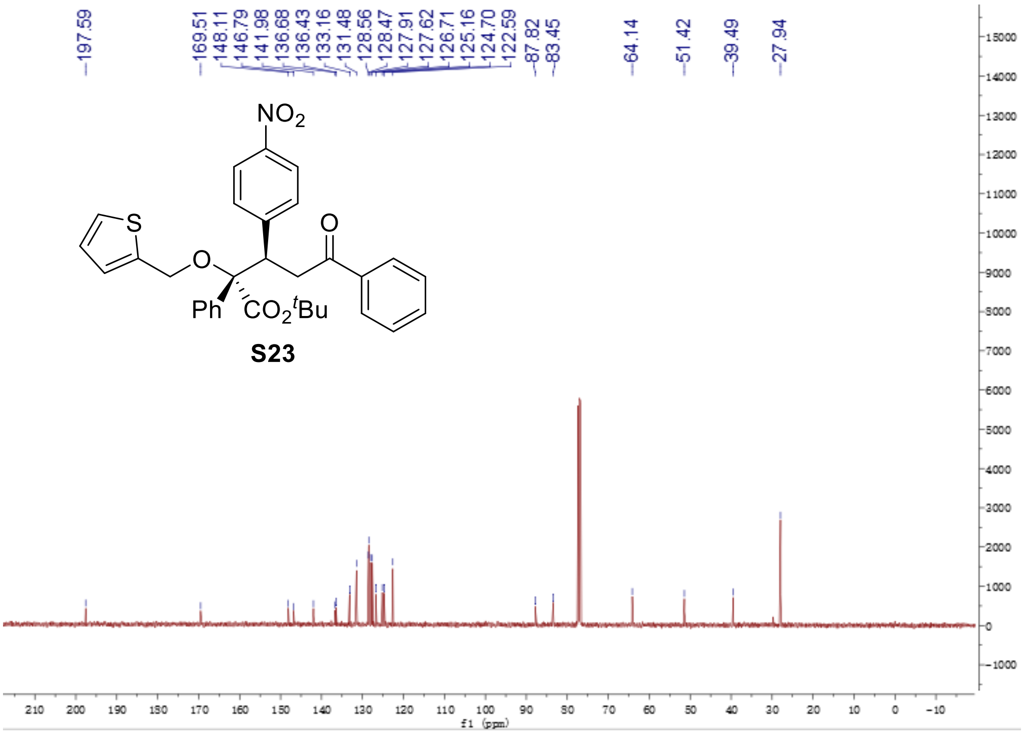

Supplementary Figure 540. ^{13}C NMR (126 MHz, $CDCl_3$) spectrum of **S23**.

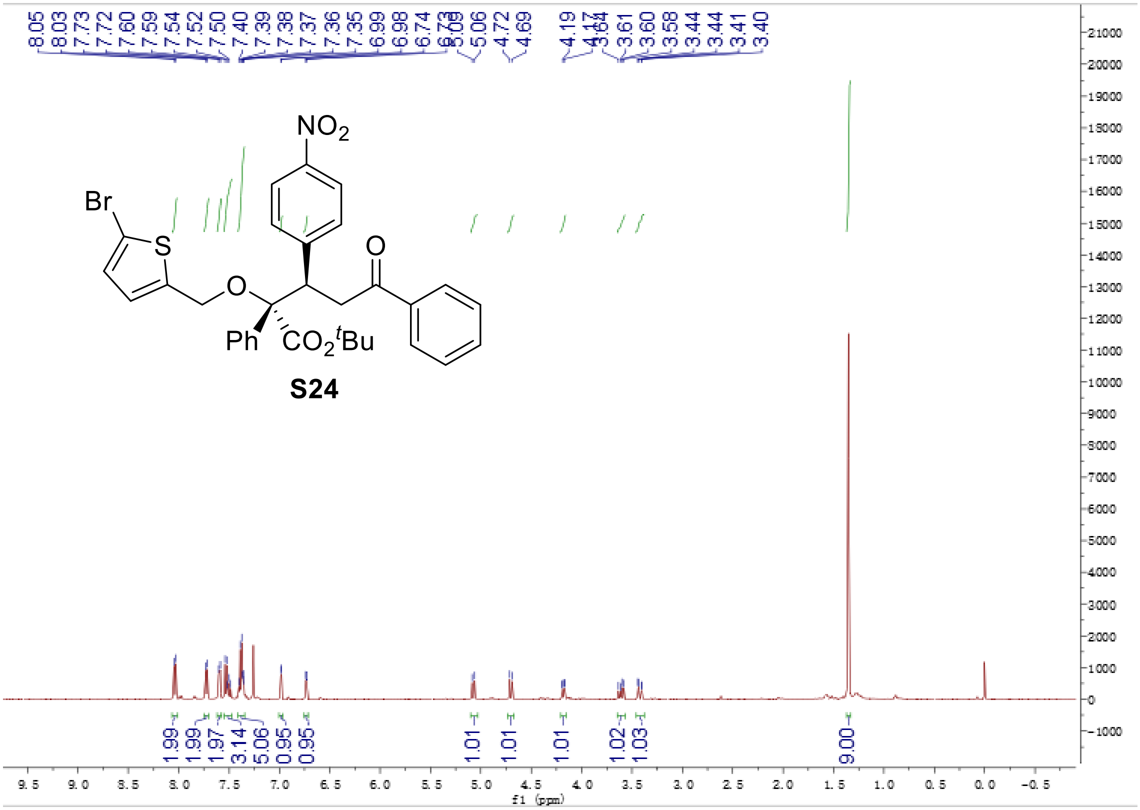

Supplementary Figure 541. ^{1}H NMR (500 MHz, acetone-d6) spectrum of **S24**.

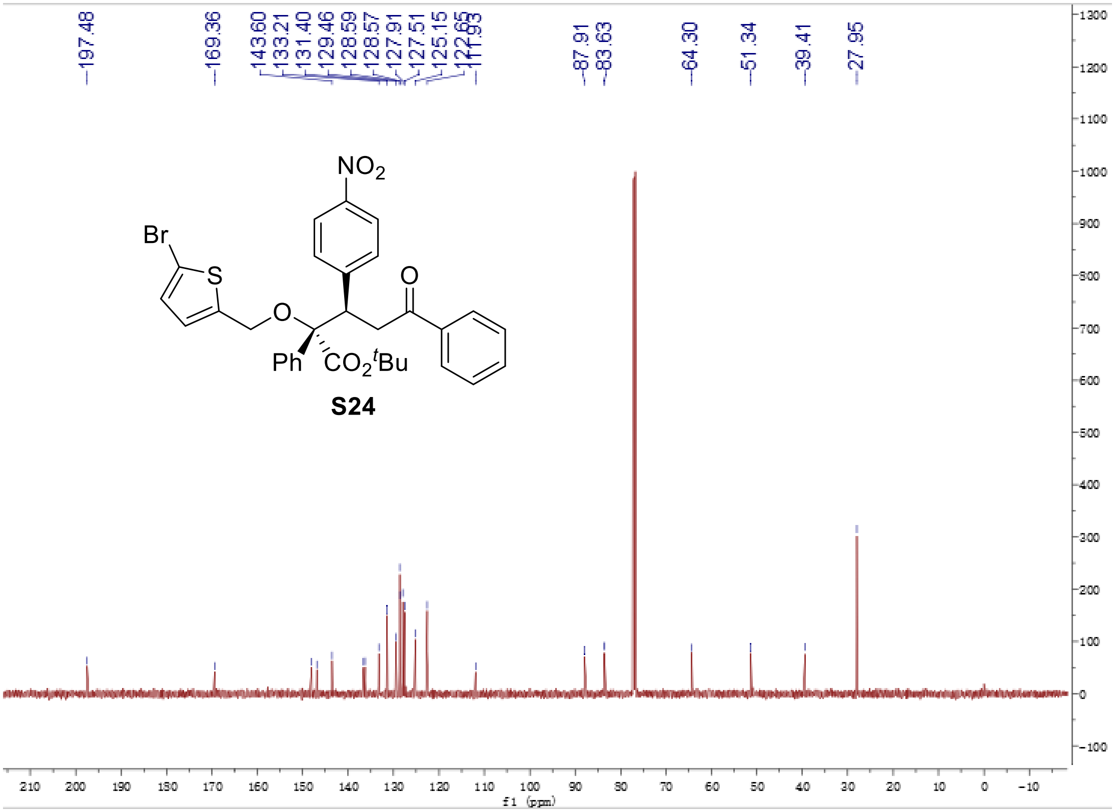

Supplementary Figure 542. ^{13}C NMR (126 MHz, acetone-d6) spectrum of **S24**.

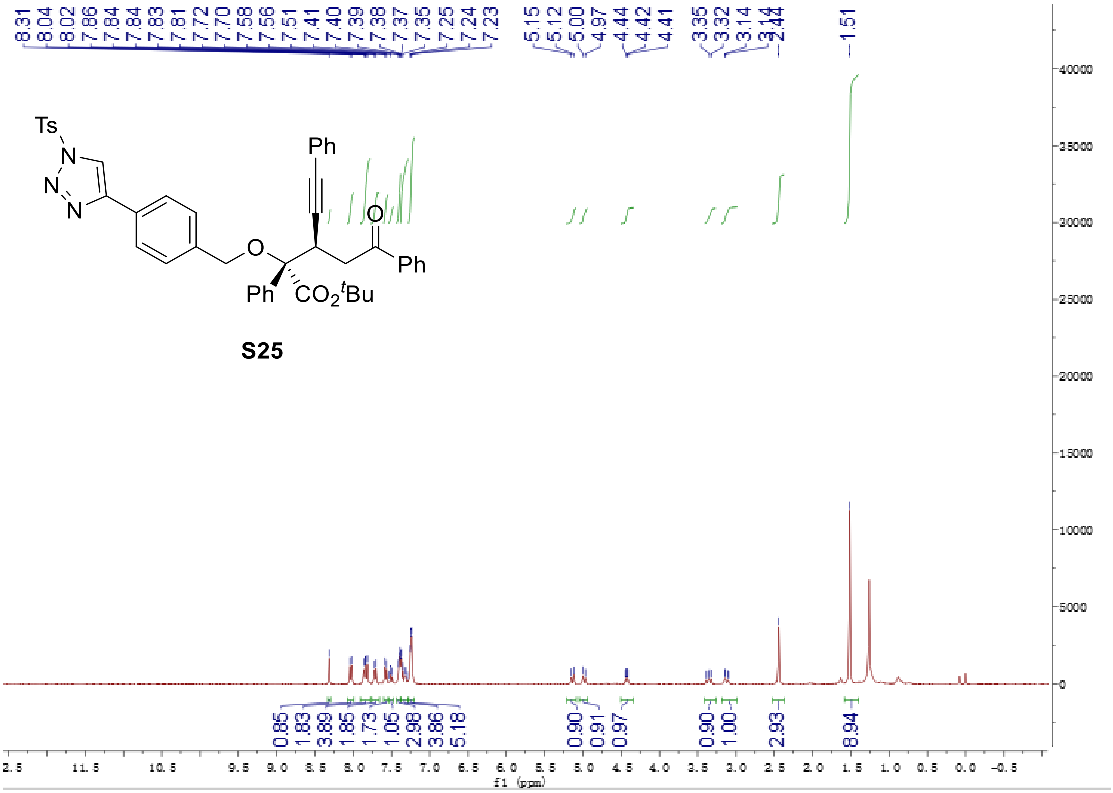

Supplementary Figure 543. ^{1}H NMR (500 MHz, $CDCl_3$) spectrum of **S25**.

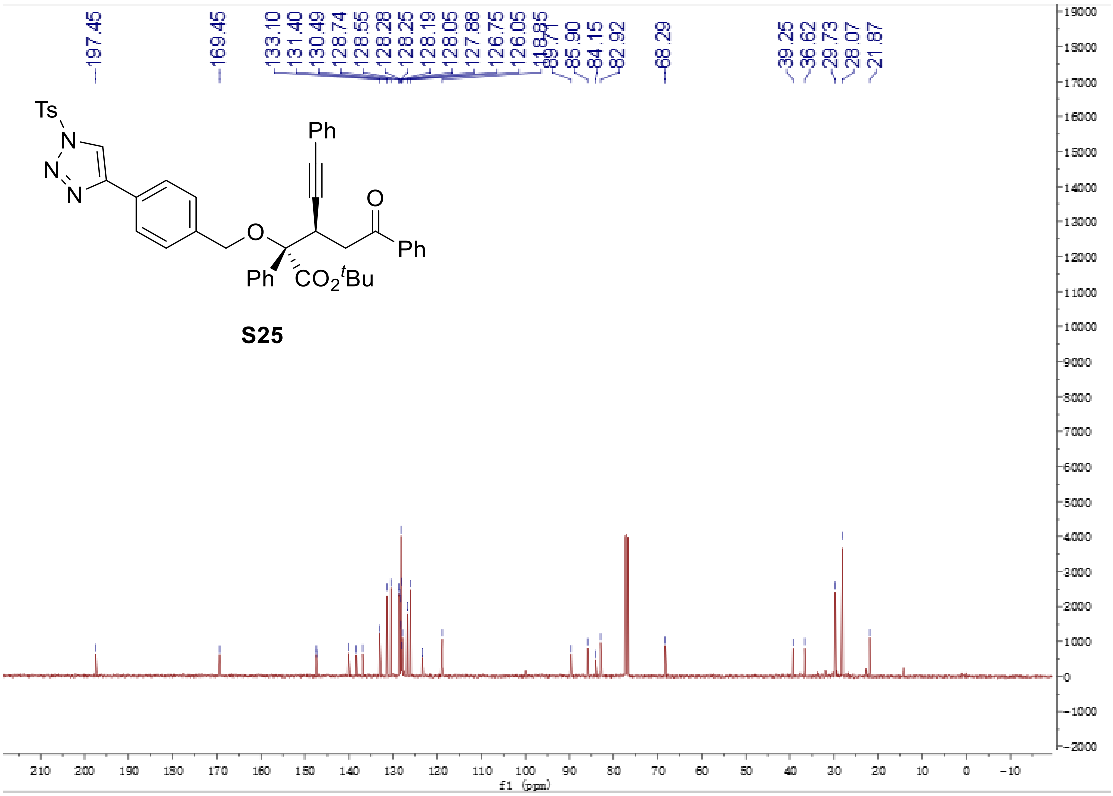

Supplementary Figure 544. ^{13}C NMR (126 MHz, $CDCl_3$) spectrum of **S25**.

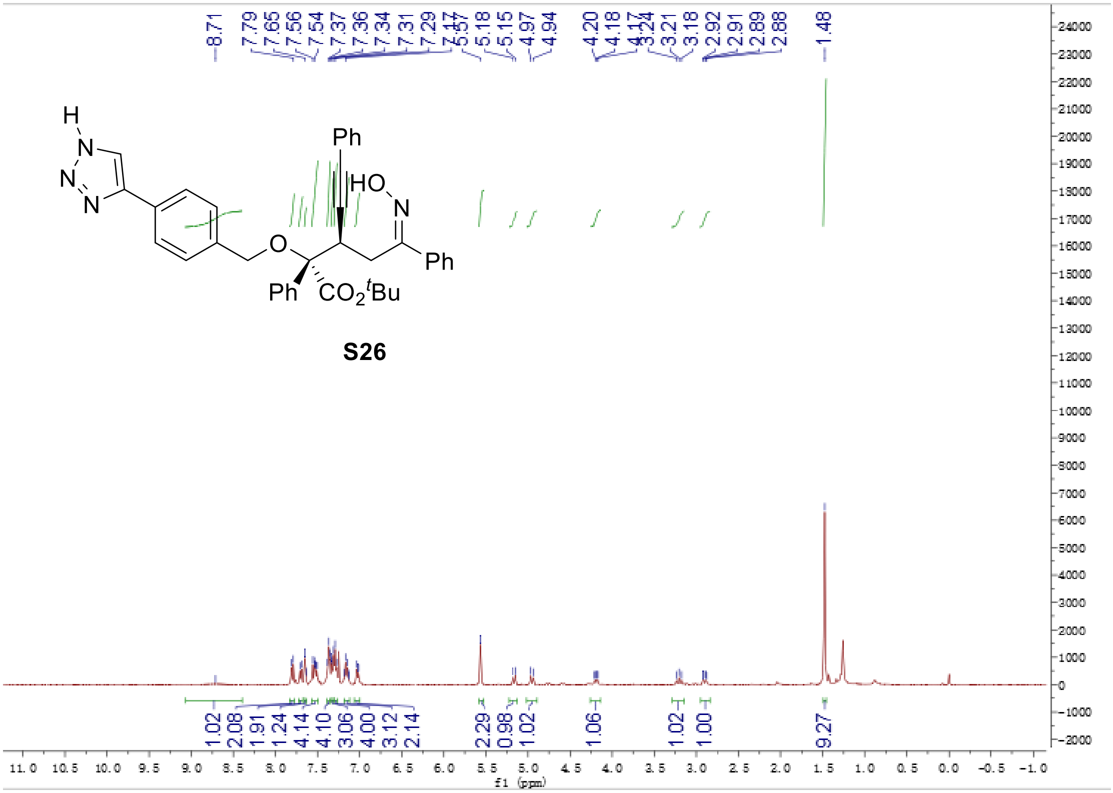

Supplementary Figure 545. ^{1}H NMR (500 MHz, $CDCl_3$) spectrum of **S26**.

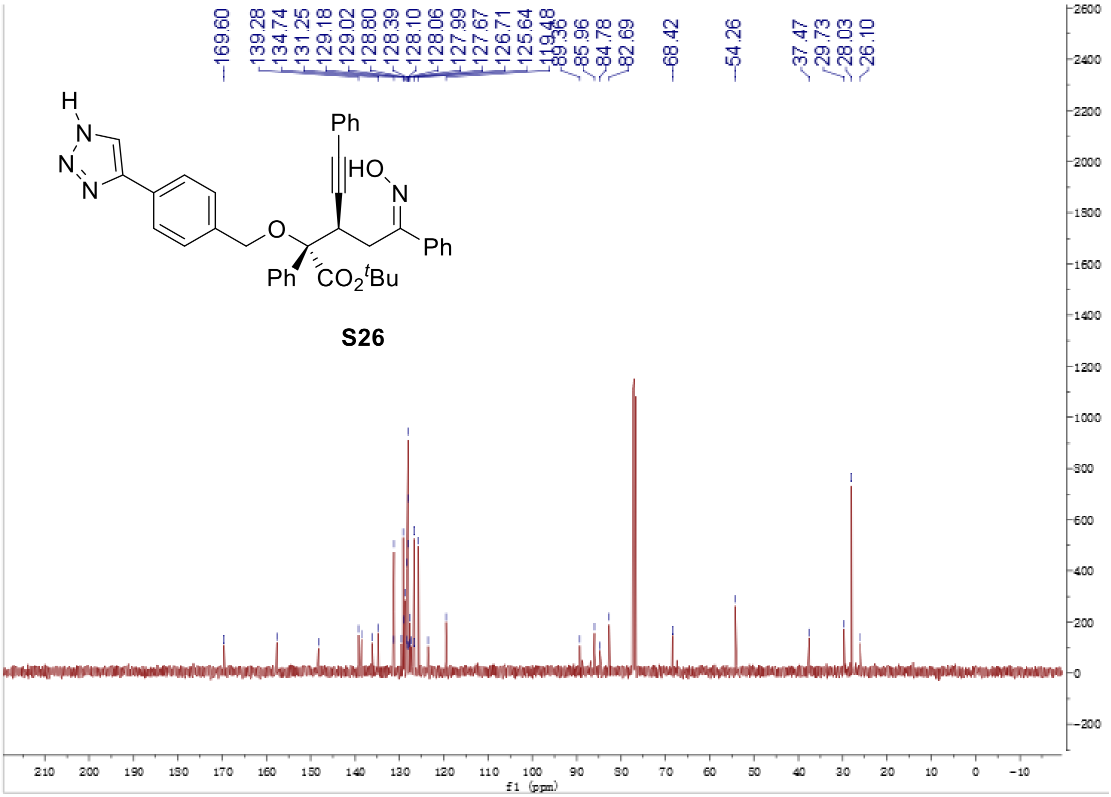

Supplementary Figure 546. ^{13}C NMR (126 MHz, $CDCl_3$) spectrum of **S26**.

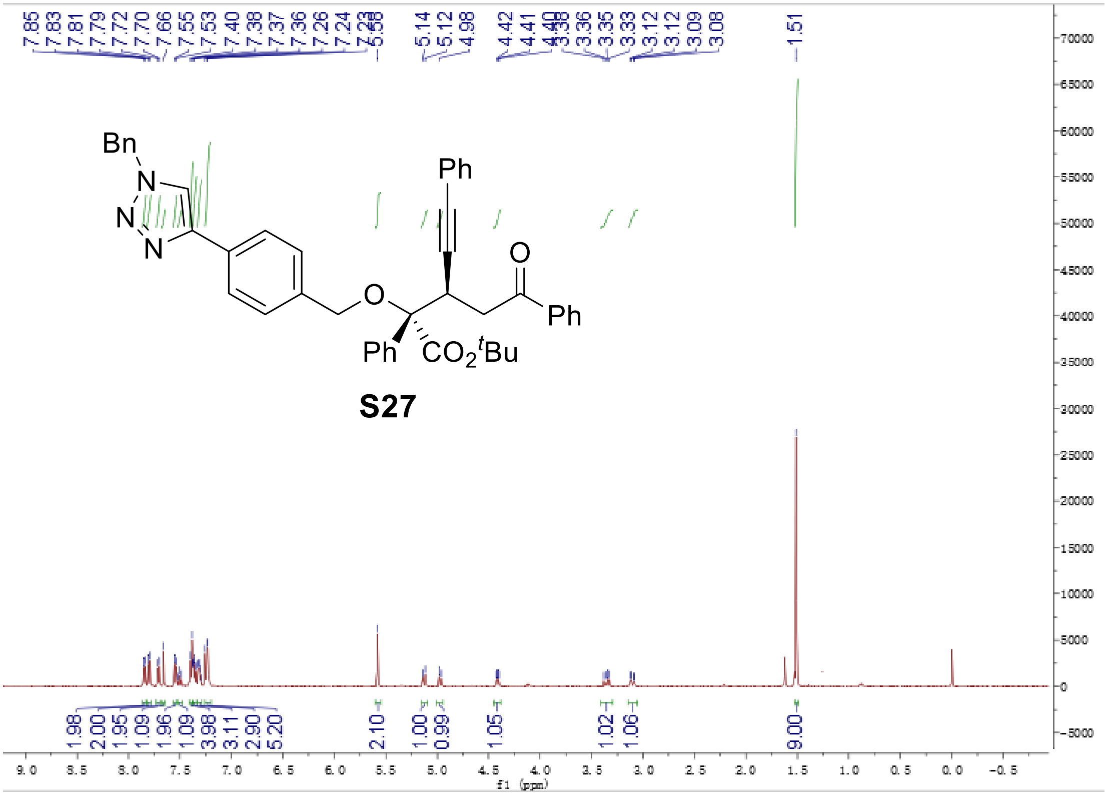

Supplementary Figure 547. ^{1}H NMR (500 MHz, acetone-d6) spectrum of **S27**.

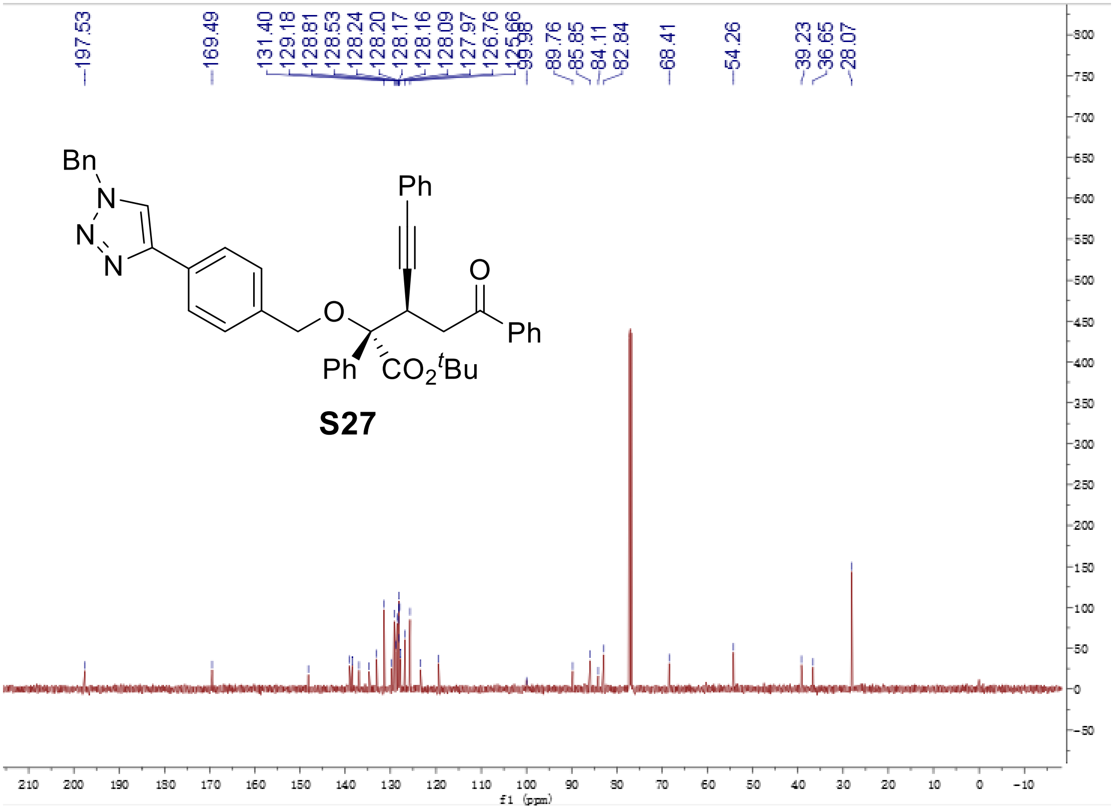

S27

Supplementary Figure 548. ^{13}C NMR (126 MHz, acetone-d6) spectrum of **S27**.

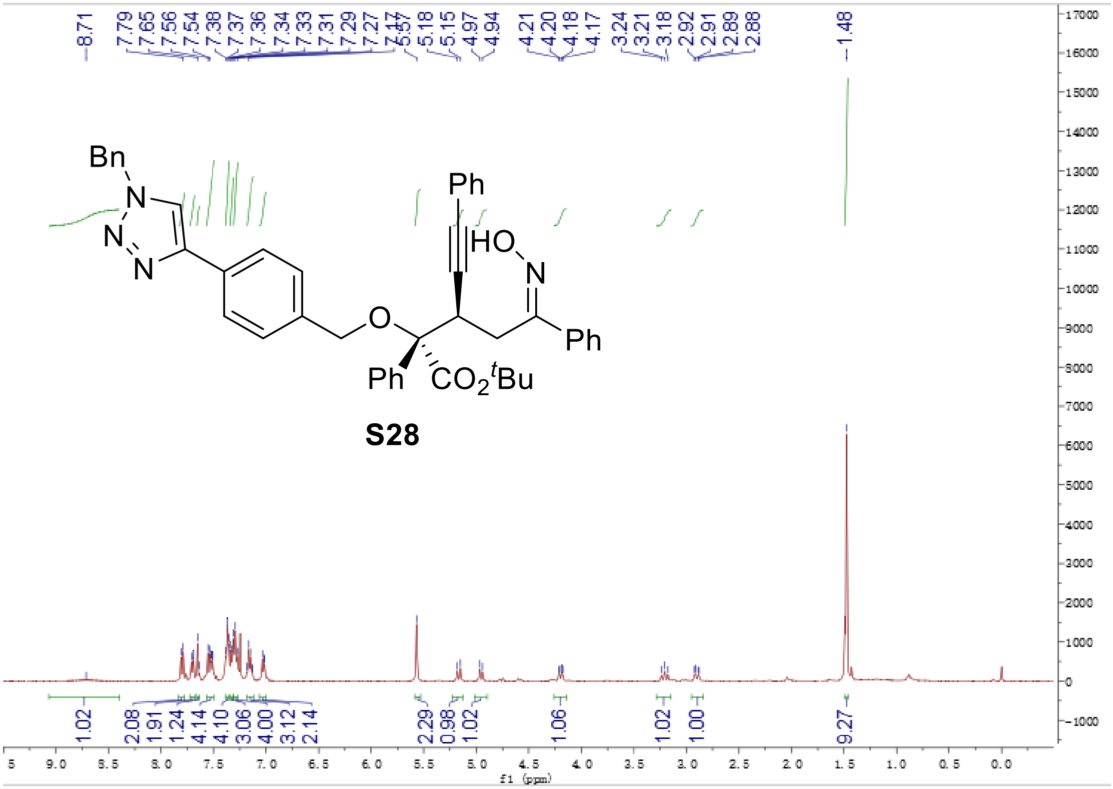

S28

Supplementary Figure 549. ^{1}H NMR (500 MHz, $CDCl_3$) spectrum of **S28**.

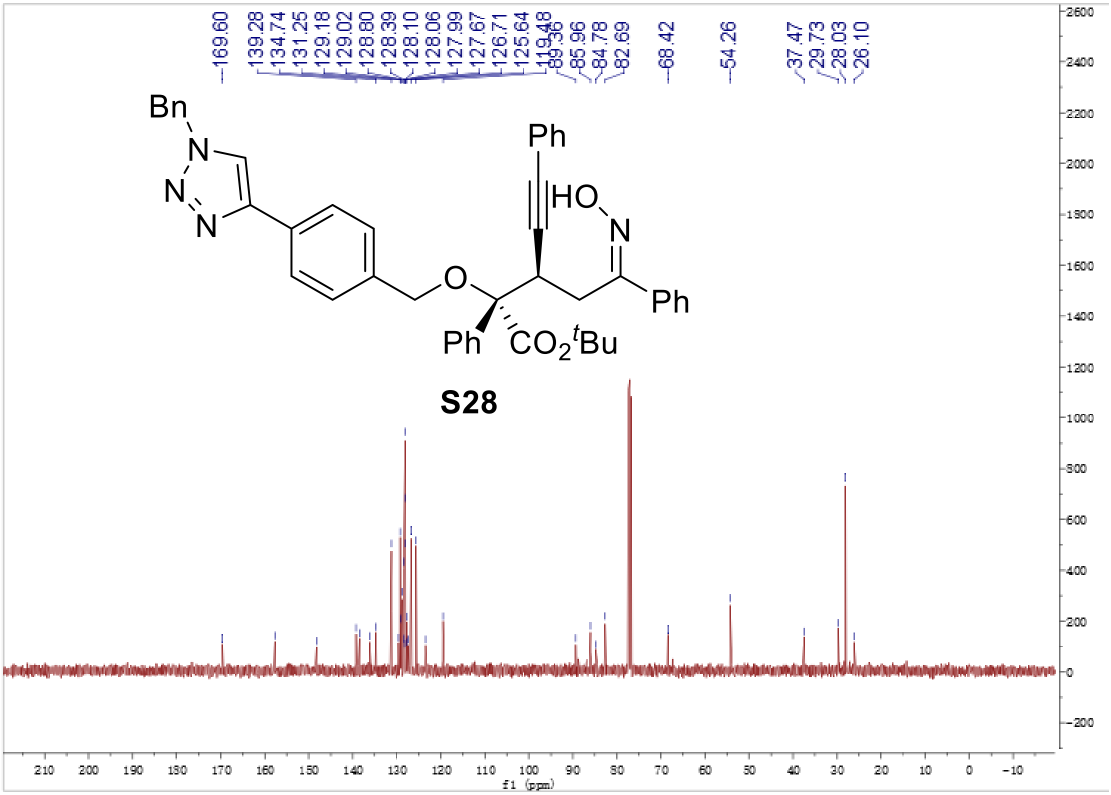

Supplementary Figure 550. ^{13}C NMR (126 MHz, $CDCl_3$) spectrum of **S28**.

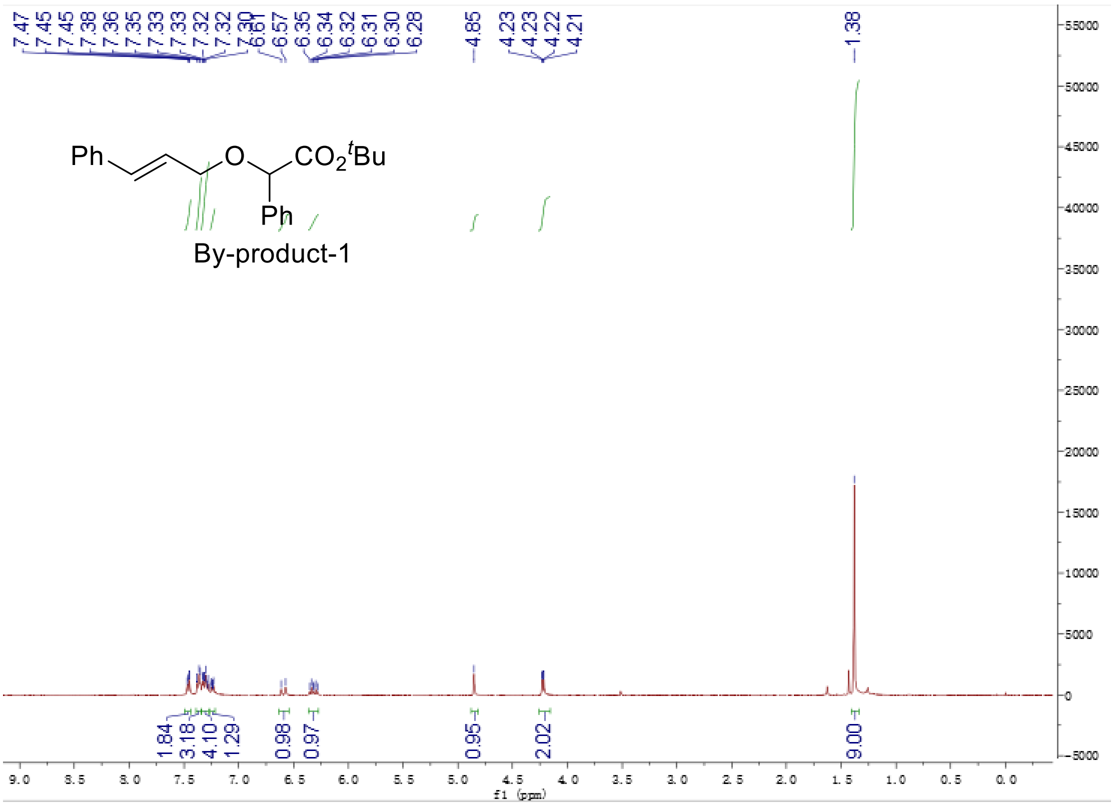

Supplementary Figure 551. ^{1}H NMR (500 MHz, $CDCl_3$) spectrum of **byproduct-1**.

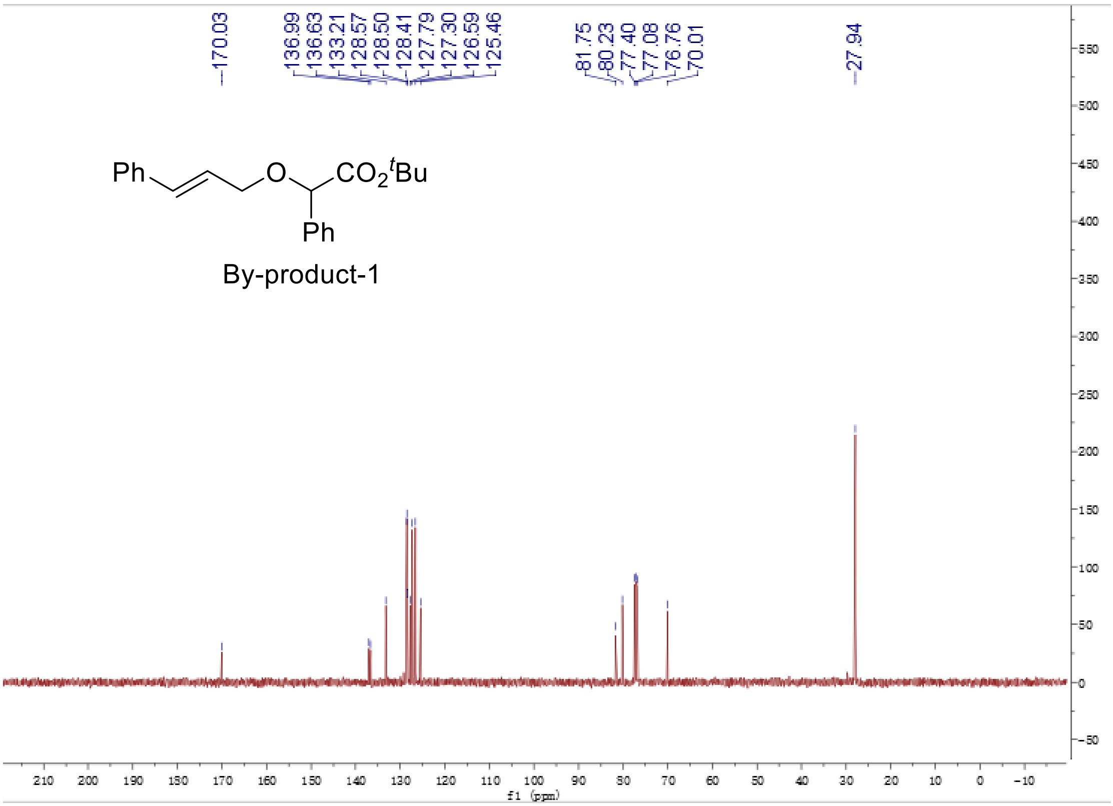

Supplementary Figure 552. ^{13}C NMR (126 MHz, $CDCl_3$) spectrum of **byproduct-1**.

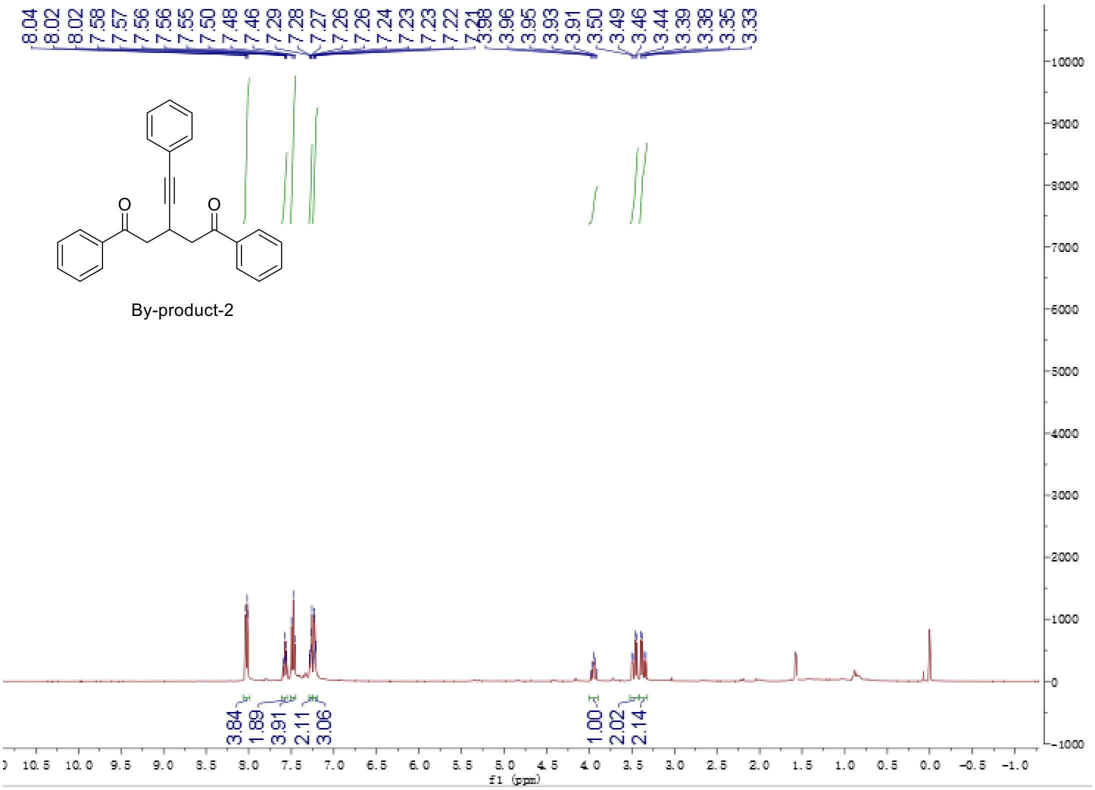

Supplementary Figure 553. ^{1}H NMR (500 MHz, $CDCl_3$) spectrum of **byproduct-2**.

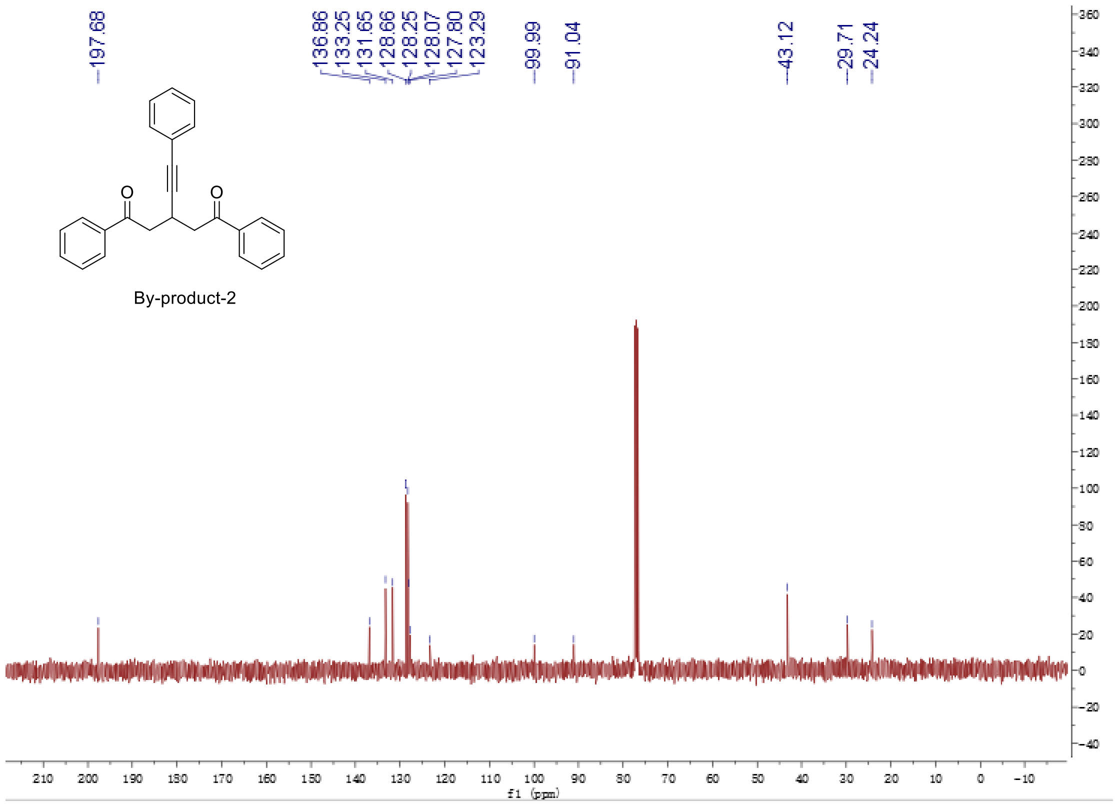

Supplementary Figure 554. ^{13}C NMR (126 MHz, $CDCl_3$) spectrum of **byproduct-2**.

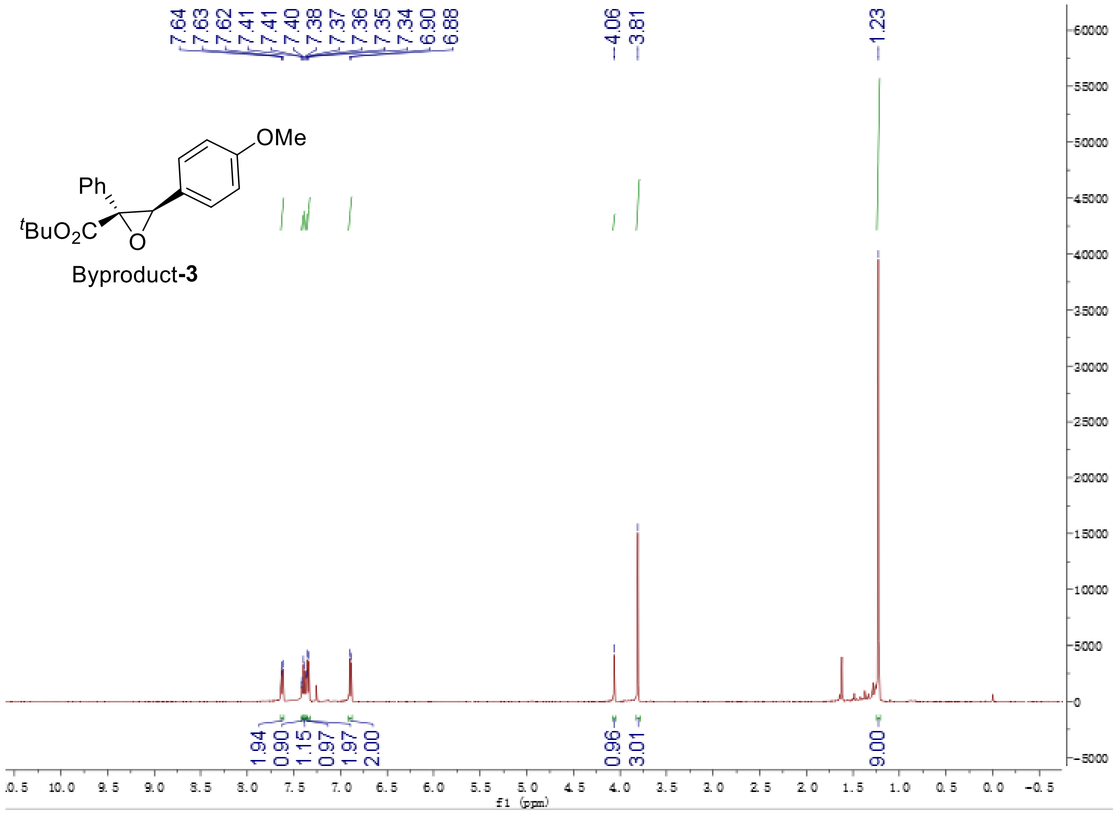

Supplementary Figure 555. ^{1}H NMR (500 MHz, acetone-d6) spectrum of **byproduct-3**.

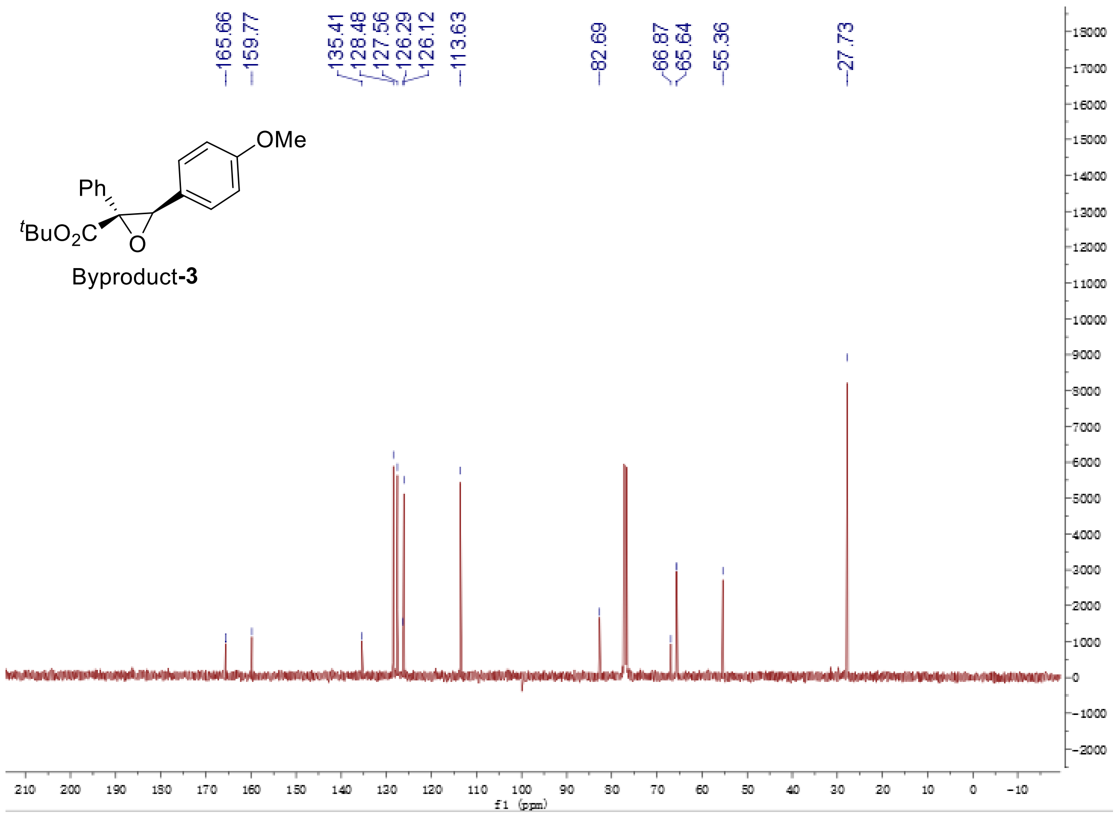

Supplementary Figure 556. ^{13}C NMR (126 MHz, acetone-d6) spectrum of **byproduct-3**.

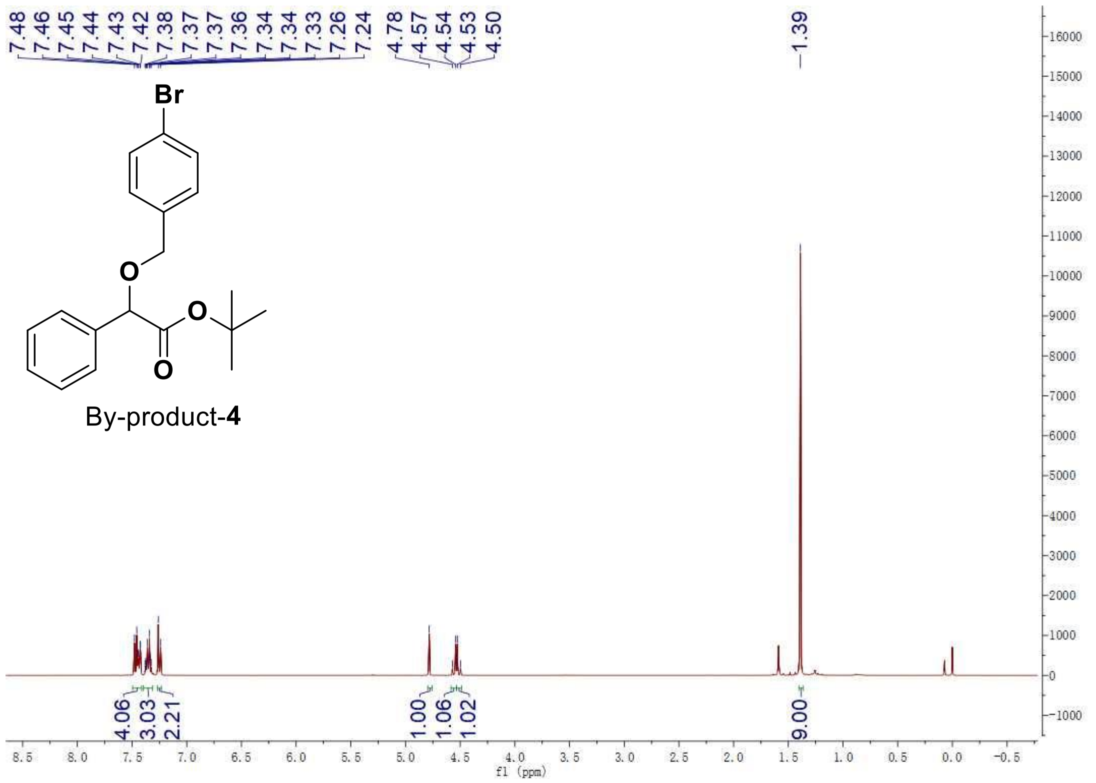

Supplementary Figure 557. ^{1}H NMR (500 MHz, $CDCl_3$) spectrum of **byproduct-4**.

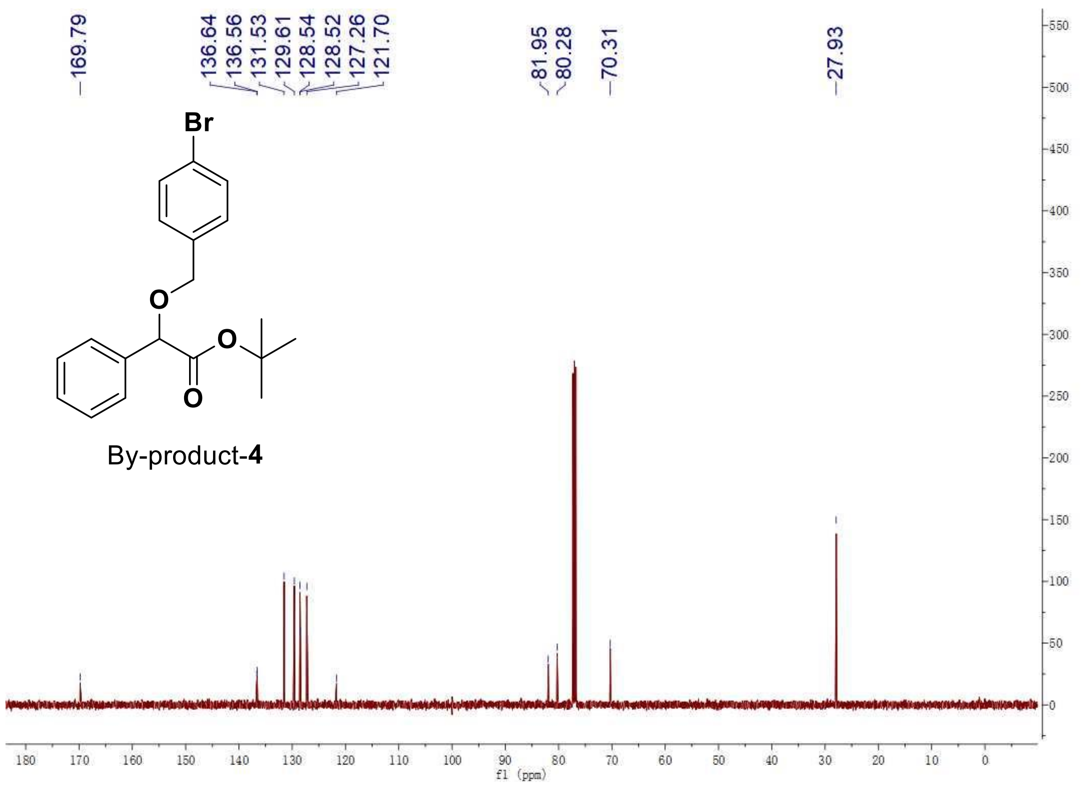

Supplementary Figure 558. ^{13}C NMR (126 MHz, $CDCl_3$) spectrum of **byproduct-4**.

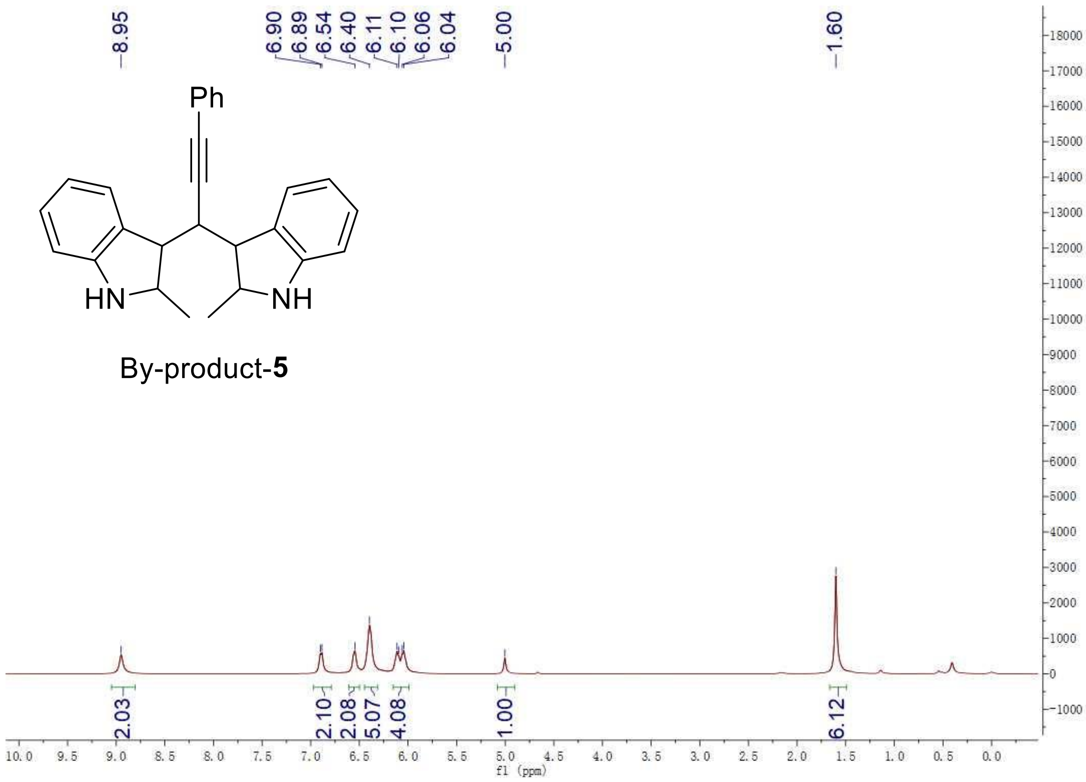

Supplementary Figure 559. ^{1}H NMR (500 MHz, $CDCl_3$) spectrum of **byproduct-5**.

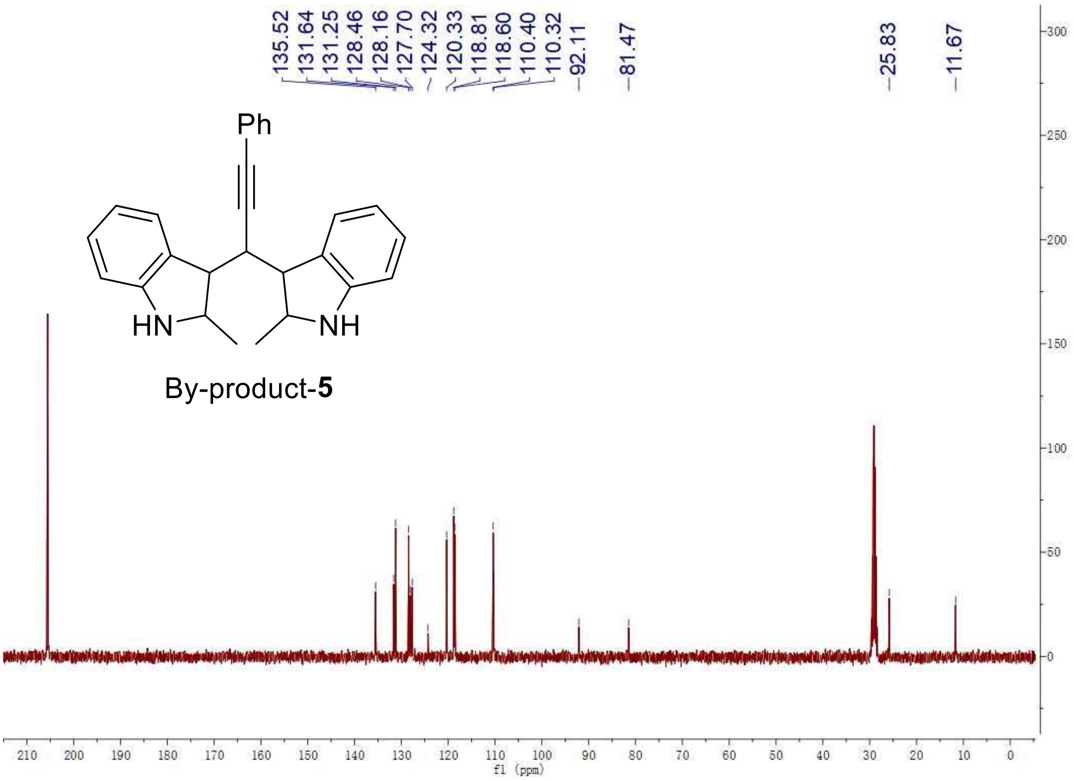

Supplementary Figure 560. ^{13}C NMR (126 MHz, $CDCl_3$) spectrum of **byproduct-5**.

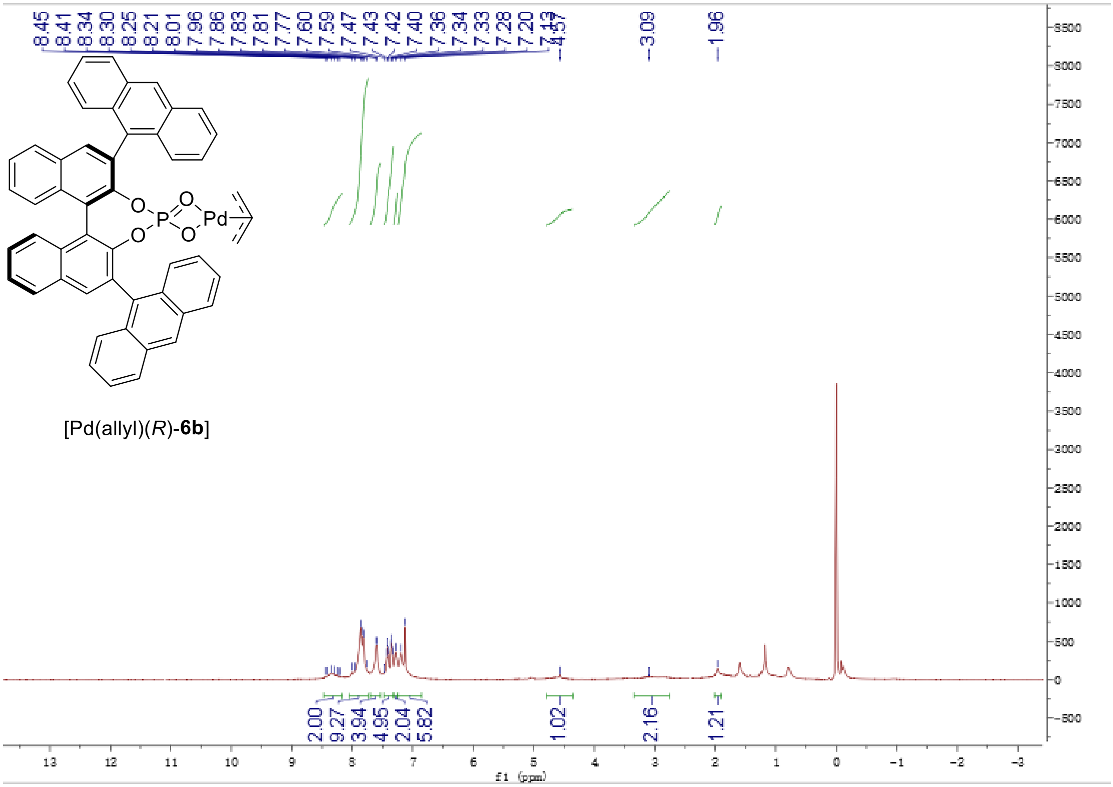

Supplementary Figure 561. ^{1}H NMR (500 MHz, $CDCl_3$) spectrum of **Pd(allyl)(R)-6b**.

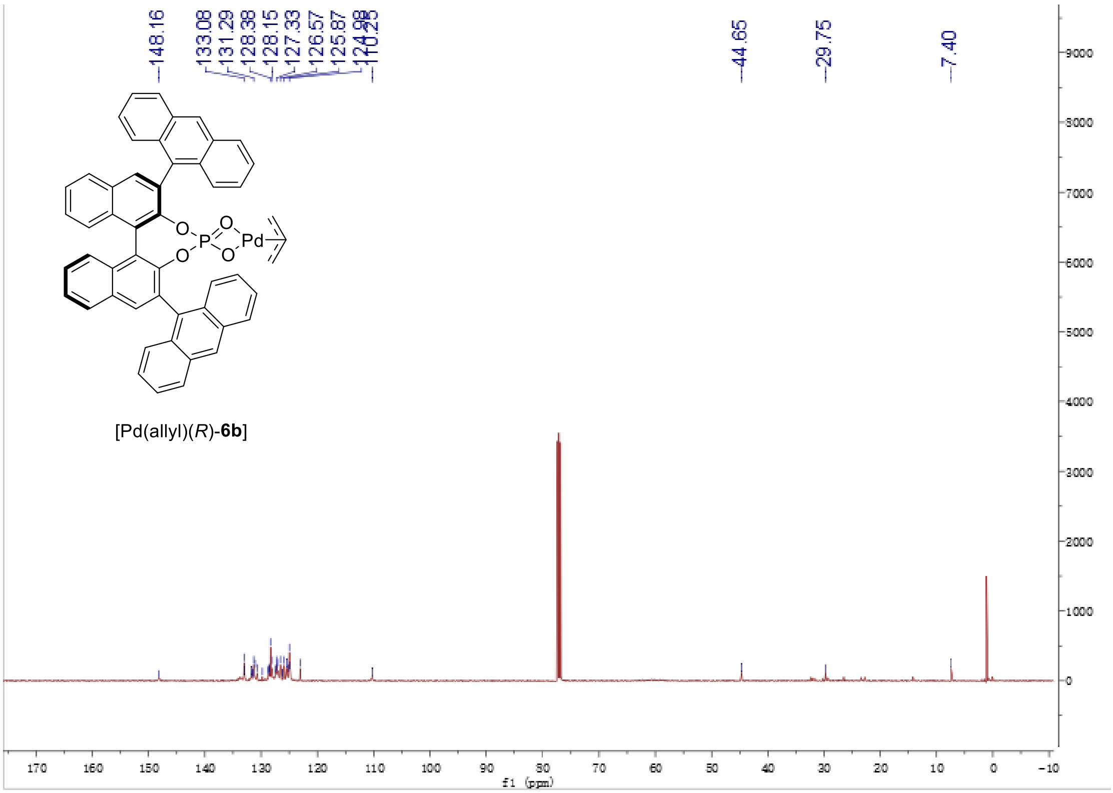

Supplementary Figure 562. ^{13}C NMR (126 MHz, $CDCl_3$) spectrum of **Pd(allyl)(R)-6b**.

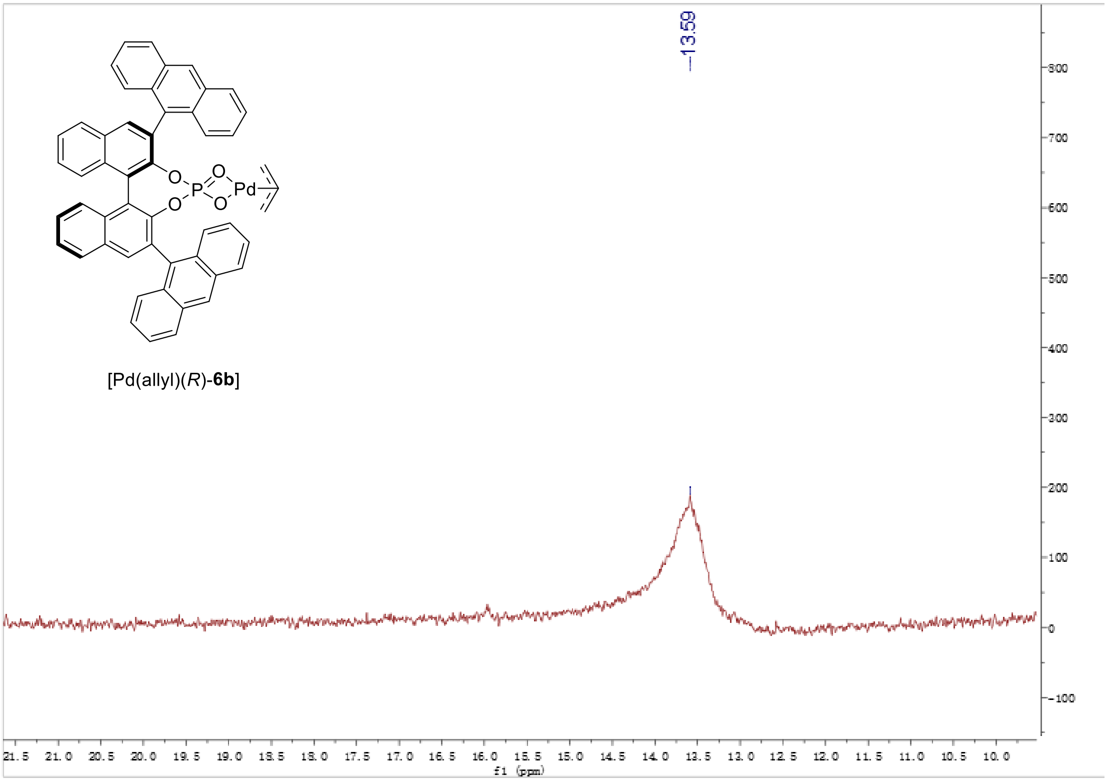

Supplementary Figure 563. ^{31}P NMR (202 MHz, $CDCl_3$) spectrum of **Pd(allyl)(R)-6b**.

IV. Supplementary References

1. Zhang, J.; Yu, P.; Li, S. Y.; Sun, H.; Xiang, S. H.; Wang, J. J.; Houk, K. N.; Tan, B. *Science* **361**, eaas8707 (2018).
2. Hu, B.; Chen, H.; Liu, Y.; Dong, W.; Ren, K.; Xie, X.; Xu, H.; Zhang, Z. *Chem. Commun.* **50**, 13547-13550 (2014).
3. Zhukhovitskiy, A. V.; Kobylianskii, I. J.; Thomas, A. A.; Evans, A. M.; Delaney, C. P.; Flanders, N. C.; Denmark, S. E.; Dichtel, R. D.; Toste, F. D. *J. Am. Chem. Soc.* **141**, 6473-6478 (2019).
4. Lee, S.; Hwang, G. S.; Ryu, D. H. *J. Am. Chem. Soc*. **13519**, 7126-7129 (2013).
5. Osako, T.; Panichakul, D.; Uozumi, Y. *Org. Lett.* **14**, 194-197 (2012).
6. Ma, M.; Li, C.; Peng, L.; Xie, F.; Zhang, X.; Wang, J. *Tetrahedron Lett*. **46**, 3927-3929 (2005).
7. Jiao, K.; Li, Z.; Xu, X.; Zhang, L.; Li, Y.; Zhang, K.; Mei, T. *Org. Chem. Front.* **5**, 2244-2248 (2018).
8. Cabrera-Lobera, N.; Quirós, M. T.; Brennessel, W. W.; Neidig, M. L.; Buñuel, E.; Cárdenas, D. J. *Org. Lett.* **21**, 6552-6556 (2019).
9. Ignatiuk, Z. A.; Janicki, M. J.; Góra, R. W.; Konieczny, K.; Kowalczyk, R. *Adv. Syn. Catal.* **361**, 1108-1116 (2019).
10. Paz, B. M.; Klier, L.; Næsborg, L.; Lauridsen, V. H.; Jensen, F.; Jørgensen, K. A. *Chem. Eur. J.*, **22**, 16810-16818 (2016).
11. García-Fernández, A.; Megens, R. P.; Villarino, L.; Roelfes, G. *J. Am. Chem. Soc.* **138**, 16308-16314 (2016).
12. Takahiro, I.; Toshiaki, M. *Org. Lett.* **6**, 4587-4590 (2004).
13. Su, Y.; Liu, G.; Liu, J.; Tram, L.; Qiu, H.; Doyle, M. P. *J. Am. Chem. Soc.* **142**, 13846-13855 (2020).
14. Boskovic, Z. V.; et al. *ACS Chem. Biol.* **11**, 1844-1851 (2016).

15. Evans, C. G.; Gestwicki, J. E. *Org. Lett.***11**, 2957-2959 (2009).

16. Chen, S.; Liu, Z.; Yang, T.; Hua, Y.; Zhou, Z.; Cheng, H.; Zhou, Q. *Angew. Chem. Int. Ed.* **57**, 7161-7165 (2018).

17. Kroon, E.; Kurpiewska, K.; Kalinowska-Tłuśik, J.; Dömling, A. *Org. Lett.* **18**, 4762-4765 (2016).

18. Zhang, J.; Yu, P.; Li, S.; Sun,H.; Xiang, S.; Wang, J.; Houk, K. N.; Tan, B. *Science* **361**, 1087 (2018).

19. Majumdar, K. C.; Ghosh, T.; Ponra, S. *Tetrahedron Lett.* **54**, 4661-4665 (2013).

20. Peng, X.; Wei, D.; Han, W.; Chen, F.; Yu, W.; Han, B. *ACS Catal.* **7**, 7830-7834 (2017).

21. Zhang, D.; Zhou, J.; Xia, F.; Kang, Z.; Hu, W. *Nat. Commum.* **6**, 5801-5808 (2015).

22. Tian Lu, molclus program, Version 1.8.9, http://www.keinsci.com/research/molclus.html (accessed 10, 9, 2019)

23. Frisch, M. J. *et al. Gaussian 09, Revision A.01*, Gaussian, Inc., Wallingford CT, 2009.

24. (a) Lee, C.; Yang, W.; Parr, R. G. Phys. Rev. B: Condens. Matter Mater. Phys. 1988, 37, 785. (b) Becke, A. D. *J. Chem. Phys.* **98**, 5648 (1993).

25. Marenich, A. V.; Cramer, C. J.; Truhlar, D. G. *J. Phys. Chem. B.* **113**, 6378– 6396 (2009).

26. Bruhn, T.; Schaumlöffel, A.; Hemberger, Y.; Pescitelli, G. SpecDis version 1.71, Berlin, Germany, http:/specdis-software.jimdo.com, 2017.

27. Davies, H. M., Demeese, J. *Tetrahedron Lett.*, **42**, 6803-6805 (2001).

28. Mader, S., Maji, M. S., Atodiresei, I., & Rueping, M. *Org. Chem. Front.*, **9**, 4466-4471 (2022).

29. Esquivel, E. C. C., Rufino, V. C., Nogueira, M. H. T., Souza, A. C. C., Júnior, J. R. P., & Valle, M. S. *J. Mol. Struct.*, **1204**, 127536 (2020).

30. Frisch, M. J. *et al*. Gaussian 09, Revision E.01. Gaussian, Inc., Wallingford CT, 2013.

31. Becke, A. D. *J. Chem. Phys.* **98**, 5648-5652 (1993).

32. Lee, C., Yang, W. & Parr, R. G. *Phys. Rev. B.* **37**, 785-789 (1998).

33. Hay, P. J. & Wadt, W. R. *J. Chem. Phys.* **82**, 270-283 (1985).

34. Wadt, W. R. & Hay, P. J. *J. Chem. Phys.* **82**, 284-298 (1985).

35. Barone, V. & Cossi, M. *J. Phys. Chem. A.* **102**, 1995-2001 (1998).

36. Cossi, M., Rega, N., Scalmani, G. & Barone, V. *J. Comput. Chem.* **24**, 669-681 (2003).

37. Takano, Y. & Houk, K. N. *J. Chem. Theory Comput.* **1**, 70-77 (2005).

38. Fukui, K. *Acc. Chem. Res.* **14**, 363-368 (1981).

39. Zhao, Y. & Truhlar, D. G. *Theor. Chem. Acc.* **120**, 215-241 (2008).

40. Andrae, D., Häußermann, U., Dolg, M., Stoll, H. & Preuß, H. *Theor. Chem. Acc.* **77**, 123-141 (1990).

41. CYLview, 1.0b; Legault, C. Y., Université de Sherbrooke, 2009 (http://www.cylview.org).
